# Identification of circRNA-associated ceRNA network in BMSCs of OVX models for postmenopausal osteoporosis

**DOI:** 10.1038/s41598-020-67750-8

**Published:** 2020-07-02

**Authors:** Huichao Wang, Kaifeng Zhou, Fangzhu Xiao, Zhongyue Huang, Jun Xu, Guangnan Chen, Youwen Liu, Huijie Gu

**Affiliations:** 1 Luoyang Orthopedic-Traumatological Hospital of Henan Province (Henan Provincial Orthopedic Hospital), Orthopedic Institute of Henan Province, Luoyang, 471002 Henan Province People’s Republic of China; 2 Department of Orthopedics, Minhang Hospital, Fudan University, 170 Xin Song Road, Shanghai, 201199 People’s Republic of China; 3 Department of Orthopedics, The Fifth Hospital of Xiamen, 101 Min’an Road, Maxiang Town, Xiang’an District, Xiamen, 361101 Fujian Province People’s Republic of China

**Keywords:** Stem cells, Metabolic disorders, Bone development, Bone remodelling, Disease model

## Abstract

Circular RNAs (circRNAs) serve as competing endogenous RNAs (ceRNAs) and indirectly regulate gene expression through shared microRNAs (miRNAs). However, the potential circRNAs functioning as ceRNAs in osteoporosis remain unclear. The bone marrow mesenchymal stem cells (BMSCs) were isolated from ovariectomy **(**OVX) mice and controls. We systematically analyzed RNA‐seq and miRNA‐microarray data, miRNA‐target interactions, and prominently coexpressed gene pairs to identify aberrantly expressed circRNAs, miRNAs, and messenger RNAs (mRNAs) between the OVX mice and controls. A total of 45 circRNAs, 22 miRNAs, and 548 mRNAs were significantly dysregulated (fold change > 1.5; *p* < 0.05). Gene Ontology and Kyoto Encyclopedia of Genes and Genomes pathway analyses were conducted for differentially expressed mRNAs, and subsequently a circRNA‐associated ceRNA network involved in osteoporosis was constructed. We identified two ceRNA regulatory pathways in this osteoporosis mouse model—novel circRNA 0020/miR-206-3p/Nnmt and circRNA 3832/miR-3473e/Runx3, which were validated by real-time PCR. This is the first study to elucidate the circRNA-associated ceRNA network in OVX and control mice using deep RNA-seq and RNA-microarray analysis. The data further expanded the understanding of circRNA-associated ceRNA networks, and the regulatory functions of circRNAs, miRNAs and mRNAs in the pathogenesis and pathology of osteoporosis.

## Introduction

Osteoporosis (OP) is a systemic skeletal disorder characterized by bone mass reduction and microarchitecture deterioration, which results in bone fragility and increased fracture risk^1^. Postmenopausal osteoporosis (PMOP), which is thought to result from estrogen deficiency, is a type of primary osteoporosis (POP) in clinical practice^2^. Numerous factors, such as hormones, cytokines, mechanical stimulation, etc., regulate the processes of PMOP, but the pathogenesis and regulatory mechanisms underlying PMOP remain unclear^3,4^.

MicroRNAs (miRNAs) can specifically bind to the 3′-untranslated region (3′-UTR) of target messenger RNAs (mRNAs), known as miRNA response elements (MREs), to prevent their translation and/or promote their degradation, and downregulate the expression of specific proteins at the post-transcriptional level^5^. miRNAs are known to play important roles in the development of OP. For example, miRNA-19a-3p was reported to promote the osteogenesis of human mesenchymal stem cells (hMSCs) and alleviate the progression of OP via inhibiting HDAC4 expression^6^. MiR144 can promote proliferation, inhibit apoptosis, induce osteogenic differentiation of bone marrow-derived mesenchymal stem cells (BMSCs), and may help to regulate OP, by targeting SFRP1 and activating the Wnt/β-catenin pathway^7^. Down-regulation of miR-185 promotes osteogenesis and stimulates bone formation in OP partly via the regulation of Bgn expression and BMP signaling^8^. Li et al. found that miRNA-543 depletion could protect osteoblasts against OVX-induced OP by targeting YAF2 and regulating the AKT/p38 MAPK signaling pathway^9^.

Circular RNAs (circRNAs) are a novel type of noncoding RNA, without covalently closed‐loop structures bound together by 3′ heads and 5′ tails or a polyadenylated tail. The unique circular structure of circRNA is more resistant to RNase digestion, and has higher intracellular stability than linear transcripts^10,11^. CircRNAs have been shown to contain multiple conserved MREs and compete with mRNAs, known as miRNA sponges, to regulate gene expression^10,12^. However, the potential role of circRNAs and the regulation of their interactive network on the development of OP remain unclear.

The competitive endogenous RNA (ceRNA) hypothesis was recently proposed by Salmena et al.^13^. According to this hypothesis, circRNAs can act as sponges for miRNAs via shared MREs, inhibit their activity, and subsequently upregulate the expression of target genes. CircRNAs have been shown to serve as ceRNAs and compete with mRNAs for miRNAs in many diseases^10,14,15^. For example, circSLC8A1 was reported to suppress bladder cancer progression via sponging miR-130b/miR-494 and regulate the expression of PTEN^16^. CircSNX29 acts as a sponge of miR-744, and thereby regulates the proliferation and differentiation of myoblasts via activating the Wnt5a/Ca2 + signaling pathway^17^. Zhang et al. reported that hsa_circ_0067301 acts as a sponge of miR-141 in epithelial-mesenchymal transition during endometriosis via the Notch signaling pathway^18^. Yu et al. reported that circRNA_0016624 enhanced BMP2 expression in PMOP via sponging miR-98^19^. However, OP-associated circRNAs and the specific circRNA-associated ceRNA networks involved in OP have not been studied.

In this study, RNA-sequencing and miRNA-microarray were performed in the BMSCs of control and OVX mice to systematically identify differentially expressed circRNAs, miRNAs and mRNAs. We first identified, constructed and functionally analyzed a circRNA‐associated ceRNA network in the BMSCs of OVX mouse model via miRNA‐target interactions, and prominently coexpressed gene pairs. The results of this study may provide new insights for understanding the mechanism of OP.

## Results

### Evaluation of the OVX mouse model

**Figure 1:**
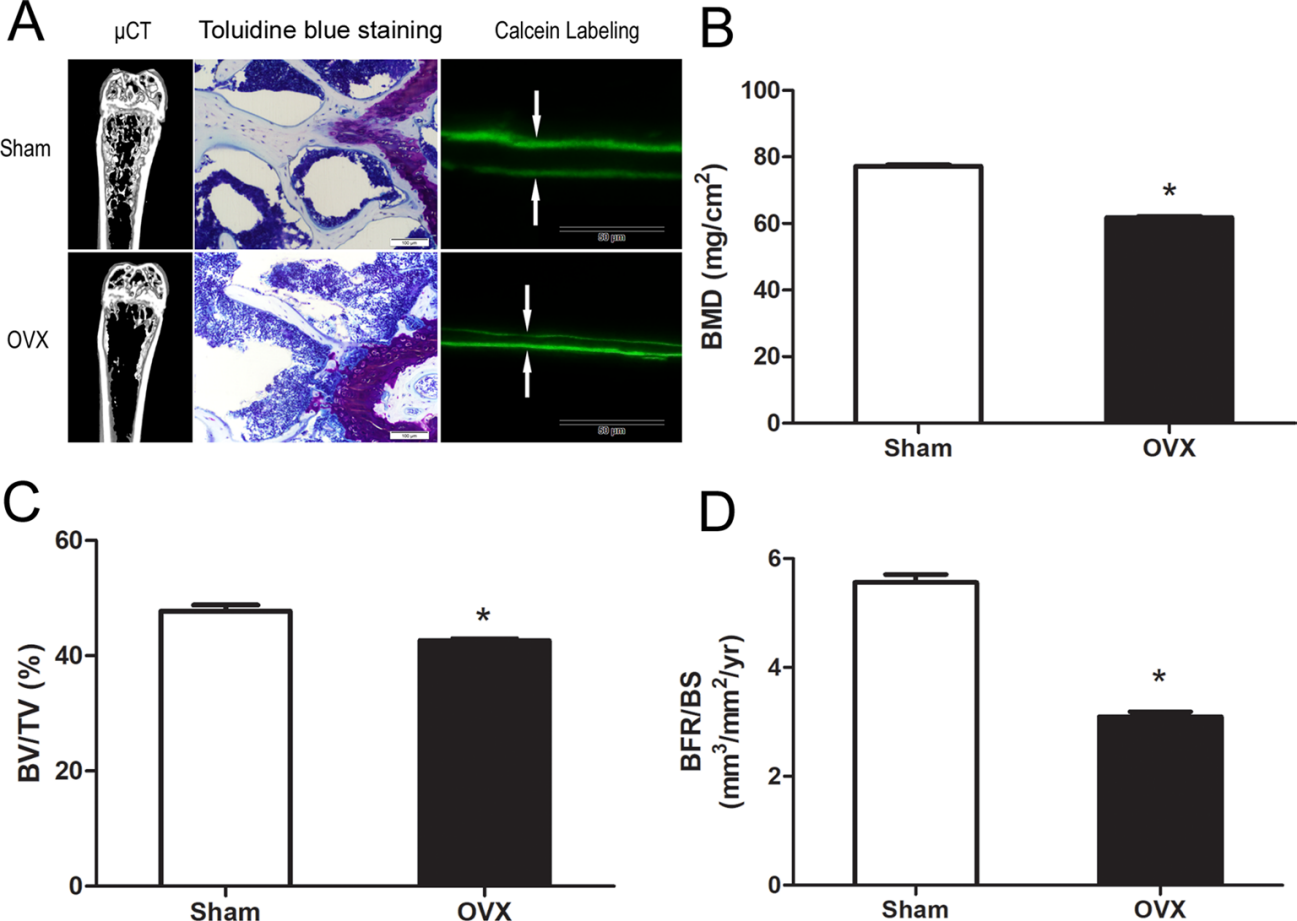
Evaluation of the OVX mouse model. (**A**) Micro-CT section, Toluidine blue staining, and Calcein double-labeling of femur bone. (**B**) OVX mice had lower BMD as compared to the controls. (**C**) BV/TV of femurs were measured by micro-CT. (**D**) Calcein double-labeling–based quantification of bone formation rate per bone surface (BFR/BS) in femurs.

Ovariectomized mice are an accepted in vivo model of human PMOP. Bone mineral density (BMD) measurement, micro-CT analysis and histomorphometric analysis were used to evaluate the OVX mice. Bones were harvested and tested six weeks after the operation. As shown in Fig. 1A, micro-CT indicated reduced bone formation in the OVX group. Toluidine blue staining and double calcein labeling analysis were performed to further confirm the reduced bone formation in the OVX group. Toluidine blue staining showed decreased bone volume in distal femur (Fig. 1A). The calcein labeling analysis showed that the distance between two consecutive labels in the trabecular bone of the femur was less in the OVX group as compared to the control group (Fig. 1A). Further quantification of these parameters demonstrated that OVX mice had reduced BMD, bone volume/total volume ratio (BV/TV) and BRF/BS as compared to the controls (Fig. 1B–D).

### Overview of circRNA-seq, mRNA-seq and miRNA-microarray

In order to obtain high-quality clean reads that could be used for further analysis, the low-quality bases and N-bases or low-quality reads were filtered out. The total number of clean reads in OVX mice were 93847658, 90533856 and 95472272, respectively. The total number of clean reads in controls were 89701356, 91106850 and 89103558, respectively. To assess the efficiency of high-throughput sequencing for RNA detection, total population of clean read was annotated and classified via alignment with the mouse reference genome (GRCm38.p6, NCBI). A total of 92647720 (98.72%), 89309048 (98.65%) and 94098658 (98.56%) reads in OVX library and 88239090 (98.37%), 89919895 (98.70%) and 87814831 (98.55%) reads in controls library were mapped to the genome. Based on the theory of CIRI software (version2.0.3)^20^, 6567 circRNAs were identified. A total of 21878 protein coding transcripts were identified via mapping to the mouse reference genome (GRCm38.p6, NCBI). The Agilent mouse miRNA microarray kit containing 1902 miRNAs was used to identify differentially expressed miRNAs. These circRNAs, mRNAs and miRNAs were used for subsequent analysis.

### Analysis of differentially expressed circRNAs, mRNAs and miRNAs

**Figure 2:**
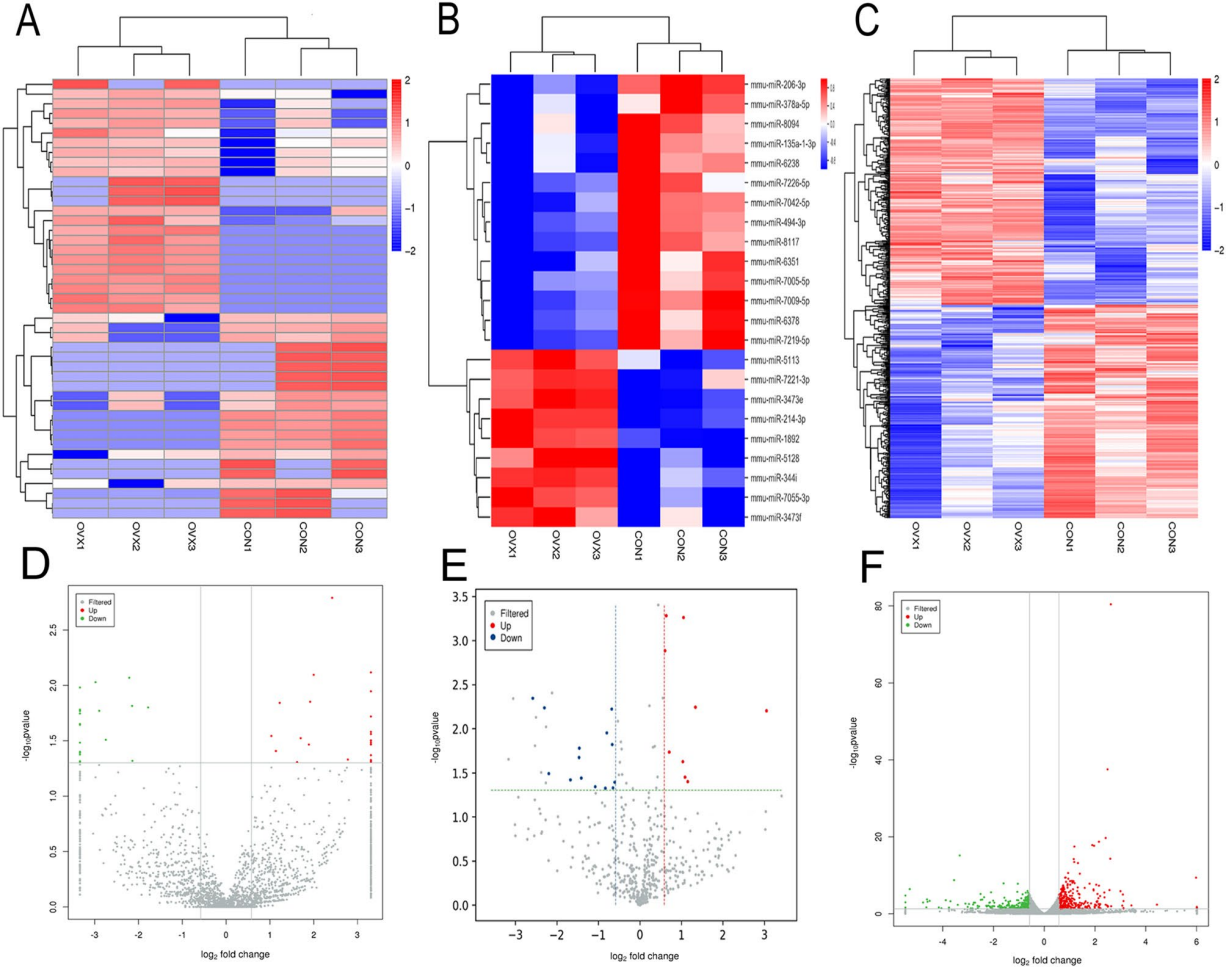
Expression profiles of circRNAs, miRNAs and mRNAs. (**A**) Cluster analysis of differentially expressed circRNAs. (**B**) Cluster analysis of differentially expressed miRNAs. (**C**) Cluster analysis of differentially expressed mRNAs. Red indicates increased expression, and blue denotes decreased expression. (**D**) The volcano plot of differentially expressed circRNAs. (**E**) The volcano plot of differentially expressed miRNAs. (**F**) The volcano plot of differentially expression mRNAs.

In this study, fold change ≥ 1.5 and *p* < 0.05 were used to identify the differentially expressed circRNAs, mRNAs and miRNAs. The heat map analysis and volcano plot were performed to identify the differentially expressed circRNAs, miRNAs and mRNAs between the OVX mice and controls at six weeks (Fig. 2). A total of 45 significantly differentially expressed circRNA transcripts were initially identified, with 24 upregulated and 21 downregulated transcripts in OVX mice vs. controls (Table S1). A total of 548 significantly differentially expressed mRNAs were identified, with 280 upregulated and 268 downregulated mRNAs in OVX mice (Table S2). A total of 22 significantly differentially expressed miRNAs were identified, with eight upregulated and 14 downregulated miRNAs in OVX mice (Table S3).

As shown in Table 1, the most upregulated circRNA was mmu_circ_000865 (*P* = 0.001620073), while the most downregulated circRNA was mmu_circ_0001145 (*P* = 0.00853556). The most upregulated miRNA was mmu-miR-214-3p (*P* = 0.00052319), while the most downregulated miRNA was mmu-miR-206-3p (*P* = 0.004520393). The most upregulated mRNA was Scd1 (*P* = 3.77E−81), while the most downregulated mRNA was A2m (*P* = 6.70E−16).

**Table 1:** Statistical analysis of all differentially expressed ncRNAs and mRNAs.

Expression RNAs	Total No	No. upregulated	No. downregulated	Most upregulated (*P* value)	Most downregulated (*P* value)
circRNA	45	24	21	mmu_circ_0000865 (0.001620073)	mmu_circ_0001145 (0.00853556)
miRNA	22	8	14	mmu-miR-214-3p (0.00052319)	mmu-miR-206-3p (0.004520393)
mRNA	548	280	268	Scd1 (3.77E−81)	A2m (6.70E−16)

### Prediction of miRNA‐target mRNA interactions

In this study, 167 interactions between 22 miRNAs and 36 circRNAs (Table S4), 2438 interactions between 22 miRNAs and 509 mRNAs (Table S5), and 4152 interactions between 45 circRNAs and 530 mRNAs (Table S6) were identified, according to the coexpression values. In addition, 9548 MREs of 22 miRNAs with 544 mRNAs (Table S7), and 549 MREs of 22 miRNAs with 37 circRNAs (Table S8), were identified based on the nucleotide sequence. Furthermore, 21 target pairs were identified among 165 miRNA/circRNA filtered target pairs with 167 miRNA/circRNA interactions, and 1062 interactions among 5043 miRNA/mRNAs filtered target pairs with 2438 miRNA/mRNA interactions.

### Construction of a circRNA-associated ceRNA network

**Figure 3:**
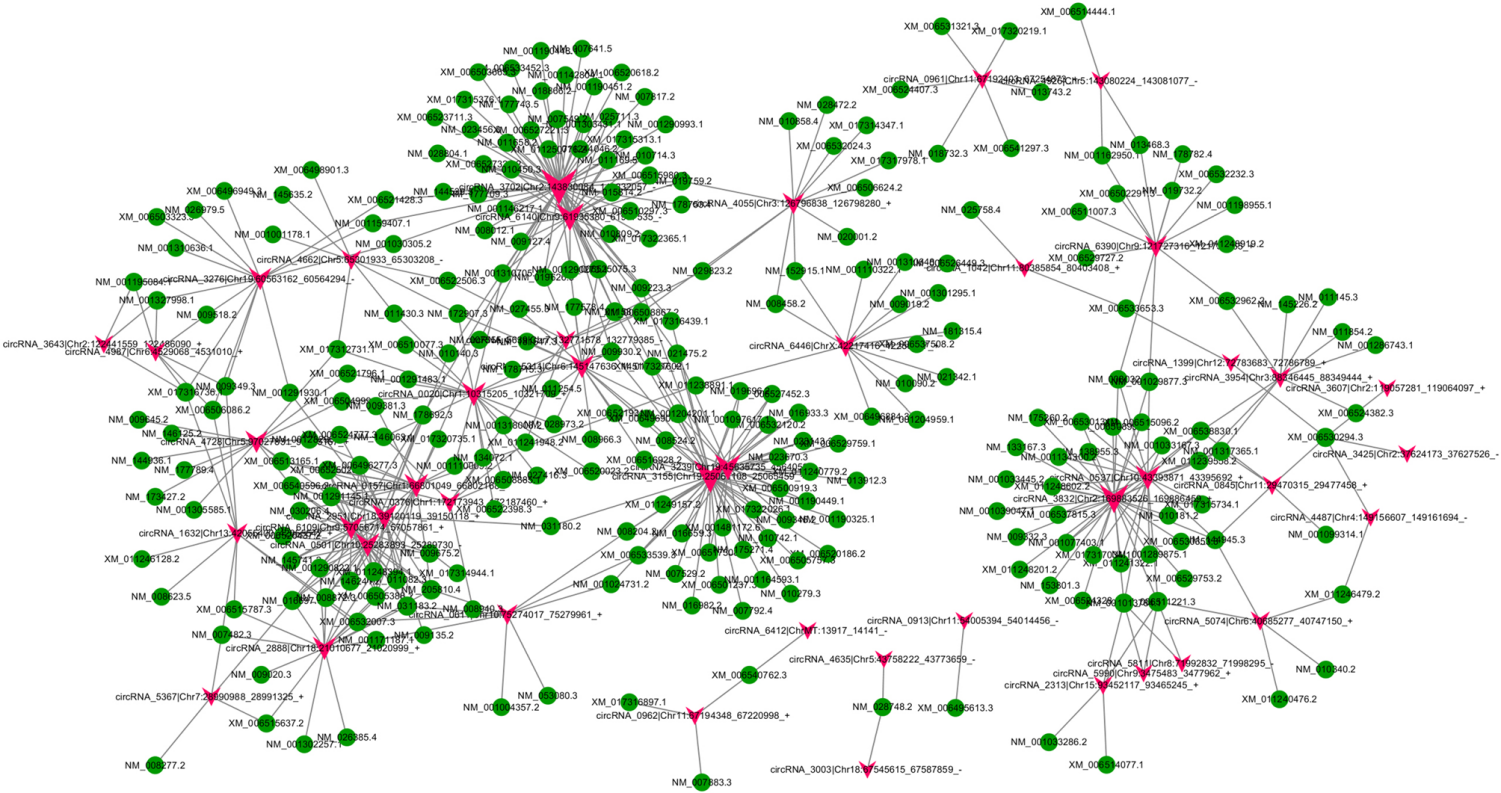
CircRNA‐mRNA coexpression network was generated by Cytoscape 3.5.1. circRNA, circular RNA; mRNA, messenger RNA.

**Figure 4:**
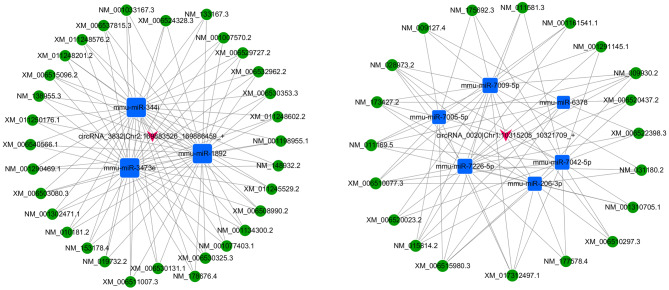
CircRNA-associated ceRNA networks in the OVX mouse model. (**A**) CircRNA_3832-associated ceRNA networks in OVX mice. (**B**) CircRNA_0020-associated ceRNA networks in OVX mice. The blue square nodes represent miRNAs, the pink arrow nodes indicate circRNAs, the green circular frames denote mRNAs, and the edges represent the competing interactions among them.

In the ceRNA hypothesis, circRNAs and mRNAs compete for the same miRNA response elements (MREs) to regulate each other. In this study, 4152 interactions between circRNAs and mRNAs, and 52 ceRNAs based on ceRNA score were identified. We identified 51 ceRNAs (circRNA‐miRNA‐mRNA) between 52 ceRNAs based on score and 4152 circRNA‐mRNAs, by filtering the overlapping results of ceRNAs according to ceRNA scores and circRNA/mRNA pairs. The coexpression network of top 500 circRNA/mRNA was constructed using Cytoscape 3.5.1 (Fig. 3), while the ceRNA network was constructed as shown in Fig. 4. Figure 4 showed ceRNA network included two circRNAs and constructed two ceRNA networks: one was circRNA 0020 (upregulated in OVX mice)-miRNAs (downregulated in OVX mice)-mRNAs (upregulated in OVX mice), and the other was circRNA 3832 (downregulated in OVX mice)-miRNAs (upregulated in OVX mice)-mRNA (downregulated in OVX mice). These ceRNA analyses may facilitate novel exploration of the underlying mechanism of OP. The details of ceRNAs are shown in Table S9.

### GO and KEGG analyses of the ceRNA network

**Figure 5:**
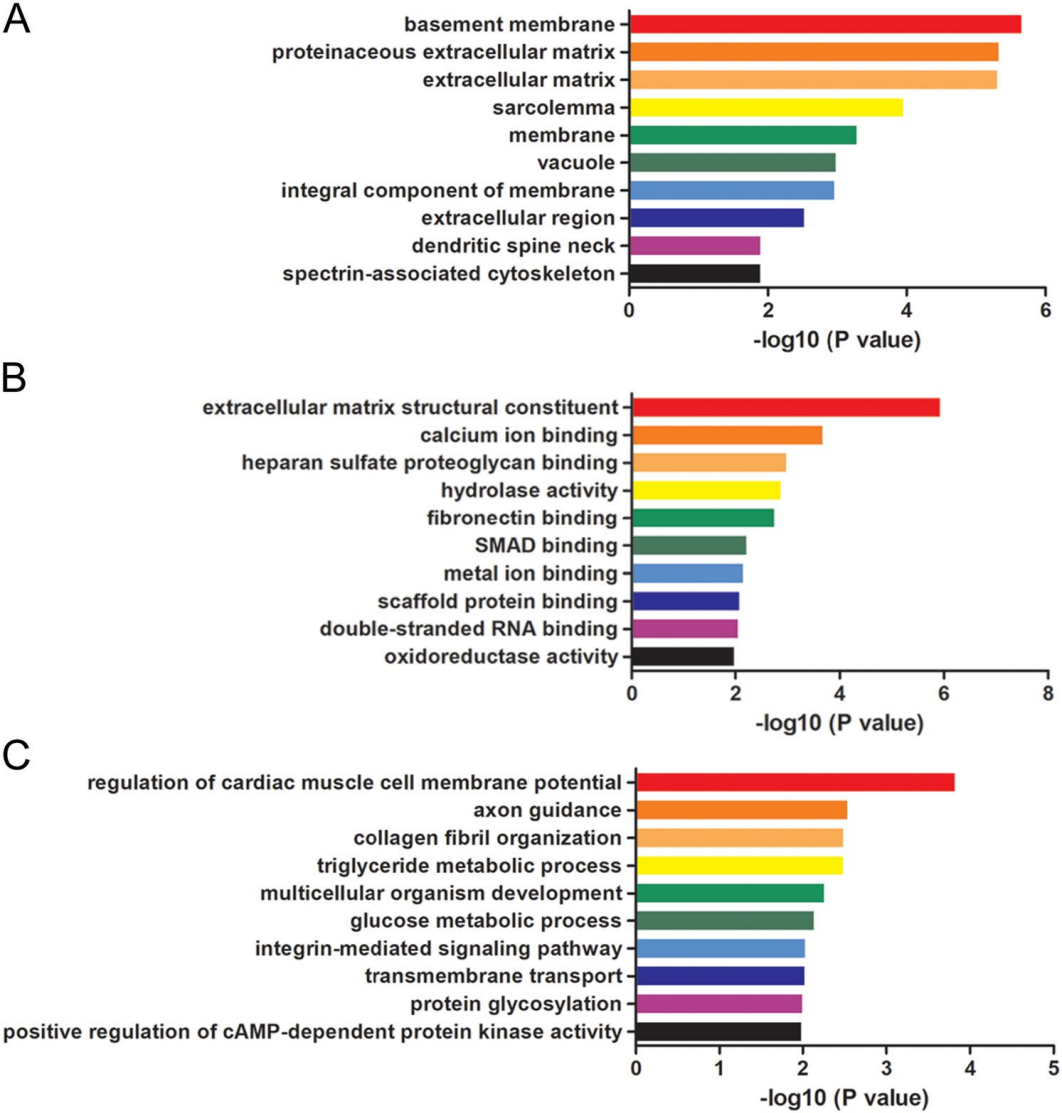
GO enrichment analyses of the ceRNA network. The top 10 GO enrichment analyses of cellular component (**A**), molecular function (**B**) and biological process (**C**).

The mRNAs involved in the ceRNA network were filtered for the Gene Ontology (GO) and Kyoto Encyclopedia of Genes and Genomes (KEGG) pathway analyses to further explore the mechanisms and pathways of ceRNAs in OP. GO analysis showed GO terms, such as basement membrane (GO: 0005604), proteinaceous extracellular matrix (GO: 0005578) and extracellular matrix (GO: 0031012) in cellular component, extracellular matrix structural constituent (GO: ), calcium ion binding (GO: 0005509), heparan sulfate proteoglycan binding (GO: 0043395) and in molecular function, and regulation of cardiac muscle cell membrane potential (GO: 0002026), axon guidance (GO: 0007411) and collagen fibril organization (GO: 0030199) in biological process, were significantly enriched in the OVX mice. The top 10 terms are shown in Fig. 5. A total of 15 enriched KEGG pathways were identified in these differentially expressed mRNAs involved in the ceRNA networks (*p* < 0.05, Table 2), including ECM-receptor interaction, fatty acid metabolism, focal adhesion, and PPAR signaling pathways. The functional annotation indicated that the circRNA-associated ceRNA network may have various regulatory functions in OP.

**Table 2:** KEGG pathway analysis of ceRNA network.

Term_ID	Term_description	Fold enrichment	P_value
path:mmu04512	ECM-receptor interaction	16.3373494	0.000748
path:mmu01212	Fatty acid metabolism	17.38461538	0.005742
path:mmu04510	Focal adhesion	6.814070352	0.008969
path:mmu04970	Salivary secretion	11.58974359	0.012569
path:mmu03320	PPAR signaling pathway	10.63529412	0.014807
path:mmu04974	Protein digestion and absorption	10.04444444	0.016503
path:mmu04925	Aldosterone synthesis and secretion	9.04	0.020133
path:mmu04972	Pancreatic secretion	8.776699029	0.021282
path:mmu00533	Glycosaminoglycan biosynthesis	32.28571429	0.030556
path:mmu04152	AMPK signaling pathway	7.174603175	0.030965
path:mmu00604	Glycosphingolipid biosynthesis	30.13333333	0.032705
path:mmu00603	Glycosphingolipid biosynthesis	28.25	0.034848
path:mmu04261	Adrenergic signaling in cardiomyocytes	6.149659864	0.04106
path:mmu04151	PI3K-Akt signaling pathway	3.81971831	0.041309
path:mmu04964	Proximal tubule bicarbonate reclamation	20.54545455	0.047618

### Validation of the ceRNA network

**Figure 6:**
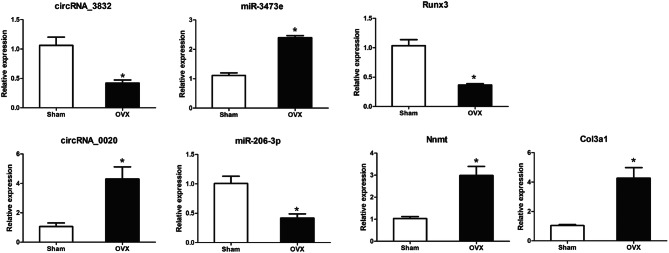
Validation of transcript expression by qRT-PCR between OVX mice and controls. Mouse ACTB and U6 genes were used as housekeeping internal controls. Transcript expression was quantified relative to the expression level of ACTB using the comparative cycle threshold (ΔCt) method. The data are presented as the mean ± SEM (n = 6); ** p < 0.05.

qPCR was used to validate the differentially expressed circRNAs, miRNAs and mRNAs in this study. We selected seven differentially expressed transcripts: two circRNAs, two miRNAs, and three mRNAs in five OVX mice and five controls. As shown in Table 3, miR-206-3p was the most significant in the network of circRNA 0020 and miR-3473e was the most significant in the network of circRNA 3832. NNMT and Col3a1, which were targets gene of miR-206-3p, and Runx3, which was target gene of miR-3473e, were reported to be involved in osteogenic differentiation of stem cells and bone formation. Consistent with sequencing and microarray data, the expression levels of circRNA 0020, Nnmt, Col3a1 and miRNA-3473e were up-regulated, while that of circRNA 3832, Runx3 and miRNA-206-3p were down-regulated in OVX mice (Fig. 6). Thus, qPCR findings supported the results from sequencing and microarray.

**Table 3:** ceRNA network mostly involved in osteoporosis

circRNA	Log2 Fold Change	*P* value	miRNA	Log2 Fold Change	*P* value	Transcript ID	Gene Name	Log2 Fold Change	*P* value
Novel_circRNA 0020	3.31	0.02752	miR-206-3p	− 2.58	0.00452	XM_006510077.3	Nnmt	0.77	0.002043
						NM_009930.2	Col3a1	0.827	4.02E−10
circRNA 3832	− 8.83	0.01655	miR-3473e	1.05	0.00055	NM_019732.2	Runx3	− 0.64	0.002401

## Discussion

Postmenopausal women suffer from multiple independent predisposing factors of osteoporosis (OP), such as estrogen deficiency, continuous calcium loss, and aging, and have a high incidence of OP, which is called postmenopausal osteoporosis (PMOP)^21^. The major clinical manifestations of PMOP are low bone mass, impaired bone microstructure and increased skeletal fragility, with a consequent increased incidence of fractures. The basic pathogenesis of OP is deregulation of bone formation and resorption caused by interactions of several genetic, epigenetic and environmental factors^22^. Numerous studies have examined the detailed physiological and pathological mechanisms, and effective treatment strategies, as well as early biomarkers and potential therapeutic targets of OP. Serum TGF-β3 level may be used as a marker for the diagnosis and follow-up of OP and osteoporotic fractures^23^. MiR-140-3p and miR-23b-3p were reported to be potential biomarkers for PMOP^24^. However, the role of circRNAs in OP remains unclear.

Noncoding RNAs, such as miRNAs, cirRNAs and lncRNAs, regulate various processes at the RNA level and are involved in numerous diseases, including OP. MiRNA-19a-3p was reported to alleviate the progression of OP by promoting the osteogenesis of hMSCs and inhibiting HDAC4 expression^6^. MiR-363-3p, which was highly expressed in OP, promoted osteoclast differentiation and inhibited osteogenesis via targeting PTEN and activating the PI3K/AKT signaling pathway^25^. LncRNA is a research hotspot in the physiological and pathological processes of OP. Low expression of lncRNA MALAT1 was reported in OP rats, which inhibited osteogenesis of BMSCs by enhancing the activation of the MAPK signaling pathway, thereby promoting OP progression^26^. Jiang Y et al. showed that lncRNA SNHG1 inhibited osteogenesis of BMSCs by negatively regulating the p38 MAPK signaling pathway through ubiquitination mediated by Nedd4^27^. However, the biological role of circRNAs in OP remains unclear. Very few studies have examined the function of circRNAs. For example, has-circRNA_0016624 could sponge miR-98 and enhance BMP2 expression to prevent OP^19^. Hsa_circ_0001275 was suggested to be a potential diagnostic biomarker for PMOP^28^. The role of circRNAs in regulating osteogenic differentiation suggests that they are potential regulators in OP^29^.

The competitive endogenous RNA (ceRNA) hypothesis was first proposed by Salmena et al., which stated that circRNA or lncRNA can inhibit miRNA activity via sponging miRNA through shared MREs and subsequently upregulate target gene expression^13^. In this study, we analyzed circRNAs and mRNAs via RNA-seq, and miRNAs via RNA-microarray. Subsequently, we constructed the circRNA-associated ceRNA network in BMSCs of the OVX mice. The OVX-induced OP mouse model can mimic PMOP in clinical practice. BMD, BV/TV, BFR and osteocalcin-positive mature osteoblasts were significantly decreased in the OVX mice as compared to controls. A total of 548 significantly differentially expressed mRNAs were identified via RNA-seq. These differentially expressed mRNAs are reported to be involved in OP pathogenesis. For example, overexpression of LEP stimulated osteogenesis of BMSCs, and upregulated osteogenesis-related genes (Runx2, ALP and Col I) and mineralization^30^. Adipoq inhibits osteoclastic differentiation and promotes osteoblastic commitment via central and peripheral mechanisms through the APPL1/phosphoinositide 3-kinase (PI3K)/Akt-mediated pathways in vitro and *in vivo*^31^. TNNC1 and MYL were identified as hub genes involved in PMOP via Gibbs sampling method^32^. In addition, 45 significantly differentially expressed circRNAs were identified by RNA-seq, while 22 significantly differentially expressed miRNAs were identified by RNA-microarray. These circRNAs, miRNAs and mRNAs were used to create the ceRNA network.

GO and KEGG pathway analyses were performed to define the functions of mRNAs involved in circRNA-associated ceRNA network. GO analyses showed that this network might involve in the pathological process of OP, including extracellular matrix structural constituent (GO: 0005201), calcium ion binding (GO: 0031012), extracellular region (GO: 0005576), etc. KEGG pathway analysis showed that these differentially expressed mRNAs were involved in extracellular matrix-receptor interaction, fatty acid metabolism, PPAR signaling pathway and PI3K-Akt signaling pathway. DKK3 (extracellular region (GO: 0005576) and multicellular organism development (GO: 0007275)) is a secreted protein that belongs to Dickkopf family. It was reported to inhibit Wnt/β-catenin signaling. Zhang et al. found that DKK3 was downregulated during mineral induction in rat dental follicle cells (DFCs), and negatively regulated the osteogenic differentiation of DFCs^33^. The extracellular matrix protein Fibulin-1 (extracellular matrix structural constituent (GO: 0005201) and extracellular matrix (GO: 0031012)) is expressed in adult bone marrow and osteoblasts. Fbln1-deficient mice die perinatally and exhibit reduced bone size and ossification in the skull^34^. Fbln1 is a positive regulator of BMP signaling and promotes the formation of membranous bone and endochondral bone in the skull^35^. Peroxisome proliferator-activated receptor γ (PPARγ) pathway is known as a key regulator of adipocyte and osteoblast differentiation of MSCs. PPARγ, which belongs to the nuclear hormone receptor superfamily of ligand-activated transcription factors, is the most important receptor in the PPARγ pathway. Shen et al. reported that the GDF11-FTO-PPARγ axis controlled the shift in MSCs from osteoporotic to adipocyte, and inhibited bone formation during OP^36^. The PI3K-Akt signaling pathway is reported to promote osteoblast proliferation, differentiation and bone formation, and inhibit the process of OP^37^. MiR-216a was reported to stimulate bone formation and antagonize the DEX-mediated suppression of osteoblast differentiation, via inhibiting c-Cbl expression and activation of the PI3K signaling pathway^38^. Although the regulation of OP by the genes identified in this study have not yet been explored, we speculate that the study of OP in this mouse model may help to understand the mechanisms involved in PMOP. Further research on the function of these genes is necessary.

In this study, we selected the most likely ceRNAs that are involved in the mechanism of OP, under strict constraints. The circRNA-associated ceRNA networks, which regulated OP-related genes and are involved in pathogenesis of OP, were selected. Novel_circRNA_0020 are ceRNAs of mmu-miR-206-3p targeting Nnmt and Col3a1 genes. Nnmt is induced via Enp looping to the NNMT promoter and required for the osteogenic differentiation of hMSCs^39^. Col3a1 plays an essential role in the proliferation of human osteoblasts by ascorbic acid 2-phosphate^40^. CircRNA_3832 (mmu_circ_0009625) are ceRNAs of mmu-miR-344i; mmu-miR-1892; and mmu-miR-3473e targeting Runx3. Runx3 is a positive modulator of the BMP9-induced osteogenic differentiation of MSCs^41^. CircRNA_33287/miR-214-3p/Runx3 axis was shown to regulate the osteogenic differentiation of MSCs. CircRNA_33287 protected Runx3 from miR-214-3p-mediated suppression and increased bone formation *in vivo*^29^. However, the circRNA-associated ceRNA networks in OP are complex. This study provided basic information to understand these circRNA-associated ceRNA networks in OP.

In conclusion, we constructed the circRNA-associated ceRNA network of OVX mice and controls using RNA-seq and miRNA-microarray analyses. The data further expanded the understanding of circRNA-associated ceRNA networks and the regulatory functions of circRNAs, miRNAs and mRNAs in the pathogenesis and pathological process of OP. The novel circRNA 0020 and circRNA 3832 (mmu_circ_0009625, Circbase) ceRNA networks were identified as potentially important mechanisms underlying OP, and biomarkers of OP for further exploration.

## Materials and methods

### OVX mouse model

Animal experiments were approved by the Animal Care and Experiment Committee of Fudan University. All animal protocols were performed in accordance with the NIH (2011) Guide for the Care and Use of Laboratory Animals. Six-week-old C57BL female mice, weighing 19–21 g, were purchased from the Shanghai Model Organisms Center (Shanghai, China). There was no significant difference in the initial body weight of the mice. The mice were housed in standard cages (five mice per cage), and maintained at 22 ± 5 °C, with constant humidity (50 ± 10%) and a 12 h light–dark cycle. The animals had free access to autoclaved water and a pellet diet till one week before the operation. Subsequently, a sham-operation or an ovariectomy-operation (OVX) was performed on the mice (control, n = 12; OVX, n = 12) after inducing anesthesia with pentobarbital sodium (50 mg/kg body weight, i.p.). The ovariectomy operation was performed according to a previously published procedure^42^. Three mice in each group were used to evaluate osteoporosis. Three mice in each group were used to perform RNA extraction, RNA-sequencing, miRNA-microarray and analysis of differentially expressed genes. Six mice in each group were used to Validate the data.

### BMD measurement and micro-CT analysis

The femurs of mice were harvested six weeks after the operation. The left femurs of three mouse in each group were scanned by Dual-energy X-ray absorptiometry (DXA; GE Healthcare, Madison, Wisconsin, USA) to determine the BMD. Then, the left femurs were fixed with 4% paraformaldehyde for 24 h, scanned and analyzed using SkyScan-1176 micro-computed tomography (µCT) (Bruker micro CT, Belgium). The distal femur was used to determine trabecular bone volume/tissue volume ratio (BV/TV).

### Toluidine blue staining and histomorphometric analyses

Toluidine blue staining was performed as previously described^43^. Briefly, the femurs were resected and fixed in 4% neutral buffered formalin for 48 h. Thereafter, the femurs were subjected to decalcification with 10% EDTA for 14 days and subsequently rinsed with running tap water overnight. The bones were embedded in paraffin, and 4-μm-thick sections were made. The bone sections were processed for Toluidine blue staining. Histomorphometric analysis was performed using a standard protocol as previously described^44^. The mice were injected with 25 mg/kg calcein at 8 and 2 d before euthanasia. The non-decalcified femurs were fixed in 70% ethanol and embedded in methyl methacrylate. Sections (5-μm-thick) of the distal femurs were used to obtain BFR/BS parameter to evaluate the bone formation.

### Isolation of mBMSCs

Six weeks after the operation, the bone marrow from the femurs and tibias was used to isolate mBMSCs as previously described^45^. In brief, the mice were euthanized and both the femur and tibia were excised aseptically and the external soft tissues were discarded. The epiphyses of each bone were removed with a razor blade and the marrow was flushed from the diaphysis with growth medium containing low-glucose Dulbecco’s modified Eagle’s medium (LG-DMEM), 10% fetal bovine serum (FBS), 2 mM glutamine, 100 mg/mL streptomycin, and 100 U/mL penicillin. The cell suspension was prepared by repeatedly aspirating the bone marrow cells through a 20-gauge needle. The cells were seeded in 60 mm tissue culture dishes (1 × 10^6^ cells/dish) and grown in the growth medium in a humidified atmosphere of 5% CO^2^ at 37 °C. The medium was changed twice a week and the third to fifth passage cells were used.

### RNA extraction, RNA-sequencing, miRNA-microarray and analysis of differentially expressed genes

The BMSCs of three mice in each group were used to extract total RNA using the mirVana miRNA isolation kit (Ambion) as per the manufacturerʼs protocol. The libraries were constructed using TruSeq stranded total RNA with Ribo‐Zero Gold according to the manufacturerʼs instructions. Then, these libraries were sequenced on the Illumina sequencing platform (HiSeqTM2500; OE biotechnology, Shanghai, China), and 125 bp/150 bp paired‐end reads were generated. Then, the differentially expressed circRNAs and mRNAs between the control and OVX mice were identified using DESeq software packages (https://bioconductor.org/packages/release/bioc/html/ DESeq.html).

The Agilent mouse miRNA microarray kit, Release 21.0, 8 × 60 K (Design ID: 070155) experiment and data analysis of the six samples were conducted by OE Biotechnology. The microarray contains 1902 probes for mature miRNAs.

### Construction of ceRNA networks

We analyzed the differentially expressed circRNAs, miRNAs and mRNAs obtained from RNA-sequencing and miRNA-microarray data of the OVX mice vs controls. The miRanda tools (https://miranda.org.uk) were used to identify miRNA-binding sites, including the position on the miRNA and DNA sequence, and free energy of MREs^46,47^. The ceRNA network was visualized via the software Cytoscape 3.7.0.

### GO and KEGG enrichment analyses

To further understand the underlying biological mechanisms of OP, the circRNA-miRNA-enriched genes were analyzed by the Gene Ontology (GO) and Kyoto Encyclopedia of Genes and Genomes (KEGG) to determine the potential biological functions and pathways. The GO terms and pathways with *p* < 0.05 were considered statistically significant.

### Validation by real-time qPCR

Total RNA was isolated from BMSCs of OVX and control using miRcute miRNA isolation kit (TIANGEN, China, DP501), and 1 μg RNA from each sample was reverse-transcribed into cDNA using the NCodeTM EXPRESS SYBR GreenERTM miRNA qPCR kit (Invitrogen). The qPCR reaction was performed using the GeneAmp PCR system 9600 (Perkin Elmer). All specific primers are listed in Table 4, and were synthesized by Sangon Biotech (Sangon Biotech, Shanghai, China). Relative transcript levels of circRNAs and mRNAs were normalized with β-actin, while those of miRNAs were normalized with U6. The expression level of each mRNA, miRNA, and circRNA was calculated using comparative Ct method.

**Table 4:** The probe sequences and primers for qRT-PCR in the experiment.

Gene	Primer sequence
circRNA_3832	F: CAATGACACTGGGACAGACG
	R: GTTTGGGGTGAGTGTTTGCT
circRNA_0020	F: CCTGAGAGATTTTAGTTCGAGGT
	R: TTTGAACTGCGAGACACTGG
Runx3	F: CAGGTTCAACGACCTTCGATT
	R: GTGGTAGGTAGCCACTTGGG
Nnmt	F: AGCACAAGACGTGAGCGTAA
	R: CGGATATCCAAAGGGGGCTC
Col3a1	F: AAGGCTGCAAGATGGATGCT
	R: GTGCTTACGTGGGACAGTCA

### Statistical analysis

All statistical analysis was conducted by using SPSS 22.0. The data of normal distribution were presented as mean values ± SD. Comparisons between the two groups were performed using Student’s t test. The enumeration data was performed via Chisquare test. The expression level of each mRNA, miRNA, lncRNA, and circRNA was represented as fold change using the 2^−ΔΔCt^ method on real-time qPCR analysis. p values or q values < 0.05 were considered statistically significant.

## Supplementary information

Supplementary file1 (PDF 2369 kb)
