# Supplementary material for: Identification of circRNA-associated ceRNA network in BMSCs of OVX models for postmenopausal osteoporosis
Source: Sci Rep. 2020 Jul 2;10:10896. doi: 10.1038/s41598-020-67750-8 (PMC7331745; doi:10.1038/s41598-020-67750-8)
Supplement: Supplementary file 1 — Supplementary file1 (PDF 2369 kb) [file 41598_2020_67750_MOESM1_ESM.pdf]

Table S1 Dysregulated circRNA transcripts between the OVX and control group

<table>
<tr><th>circRNA_id</th><th>Control-1</th><th>Control-2</th><th>Control-3</th><th>OVX-1</th><th>OVX-2</th><th>OVX-3</th><th>foldChange</th><th>log2FoldChang</th><th>P value</th><th>circbase_id</th><th>up_down</th></tr>
<tr><td>circRNA_2951|Chr18:39120119_39150118_+</td><td>0.033444</td><td>0.032928</td><td>0</td><td>0.127867</td><td>0.209866</td><td>0.136165</td><td>5.34876906</td><td>2.419206916</td><td>0.00162</td><td>mmu_circ_0000865</td><td>Up</td></tr>
<tr><td>circRNA_3702|Chr2:143830084_143832057_-</td><td>0</td><td>0</td><td>0</td><td>0.063933</td><td>0.055228</td><td>0.083794</td><td>558.12036</td><td>9.124432465</td><td>0.007629</td><td>mmu_circ_0009404</td><td>Up</td></tr>
<tr><td>circRNA_0376|Chr1:172173943_172187460_+</td><td>0</td><td>0.087809</td><td>0.03367</td><td>0.223767</td><td>0.220912</td><td>0.094268</td><td>3.99469613</td><td>1.998085763</td><td>0.008024</td><td>mmu_circ_0008436</td><td>Up</td></tr>
<tr><td>circRNA_3954|Chr3:88346445_88349444_+</td><td>0.078037</td><td>0.087809</td><td>0.13467</td><td>0.021311</td><td>0</td><td>0.052371</td><td>0.21598388</td><td>-2.21100444</td><td>0.008536</td><td>mmu_circ_0001145</td><td>Down</td></tr>
<tr><td>circRNA_5811|Chr8:71992832_71998295_-</td><td>0.055741</td><td>0.065857</td><td>0.12345</td><td>0.042622</td><td>0</td><td>0</td><td>0.12680215</td><td>-2.979348834</td><td>0.009349</td><td>-</td><td>Down</td></tr>
<tr><td>circRNA_0537|Chr10:43393871_43395692_+</td><td>0.044592</td><td>0.032928</td><td>0.06734</td><td>0</td><td>0</td><td>0</td><td>0.0020449</td><td>-8.933752802</td><td>0.010451</td><td>mmu_circ_0000163</td><td>Down</td></tr>
<tr><td>circRNA_6140|Chr9:61935380_61937535_-</td><td>0</td><td>0</td><td>0</td><td>0.074589</td><td>0.044182</td><td>0.07332</td><td>521.888721</td><td>9.027598412</td><td>0.011308</td><td>mmu_circ_0015765</td><td>Up</td></tr>
<tr><td>circRNA_6446|ChrX:42217416_42250037_+</td><td>0</td><td>0</td><td>0.11223</td><td>0.159834</td><td>0.165684</td><td>0.146639</td><td>3.77847289</td><td>1.917803271</td><td>0.014059</td><td>-</td><td>Up</td></tr>
<tr><td>circRNA_3155|Chr19:25061108_25065459_+</td><td>0.055741</td><td>0.241475</td><td>0.12345</td><td>0.340978</td><td>0.37555</td><td>0.398021</td><td>2.33423964</td><td>1.222952679</td><td>0.014388</td><td>-</td><td>Up</td></tr>
<tr><td>circRNA_2313|Chr15:93452117_93465245_+</td><td>0.100333</td><td>0.087809</td><td>0.12345</td><td>0.063933</td><td>0.033137</td><td>0</td><td>0.22608707</td><td>-2.145049612</td><td>0.015337</td><td>-</td><td>Down</td></tr>
<tr><td>circRNA_0962|Chr11:67194348_67220998_+</td><td>0.167222</td><td>0.186594</td><td>0.07856</td><td>0.053278</td><td>0.055228</td><td>0.052371</td><td>0.29119293</td><td>-1.779952767</td><td>0.015825</td><td>-</td><td>Down</td></tr>
<tr><td>circRNA_3832|Chr2:169883526_169886459_+</td><td>0.044592</td><td>0.032928</td><td>0.05611</td><td>0</td><td>0</td><td>0</td><td>0.00219596</td><td>-8.830933425</td><td>0.016549</td><td>mmu_circ_0009625</td><td>Down</td></tr>
<tr><td>circRNA_3003|Chr18:67545615_67587859_-</td><td>0.022296</td><td>0.087809</td><td>0.07856</td><td>0</td><td>0.022091</td><td>0</td><td>0.0990297</td><td>-3.335994952</td><td>0.016826</td><td>mmu_circ_0007468</td><td>Down</td></tr>
<tr><td>circRNA_5990|Chr9:3475483_3477962_+</td><td>0.066889</td><td>0.032928</td><td>0.12345</td><td>0.042622</td><td>0</td><td>0</td><td>0.13447435</td><td>-2.89459712</td><td>0.016986</td><td>mmu_circ_0015539</td><td>Down</td></tr>
<tr><td>circRNA_1042|Chr11:80385854_80403408_+</td><td>0</td><td>0.076833</td><td>0.06734</td><td>0</td><td>0</td><td>0</td><td>0.00237532</td><td>-8.717664765</td><td>0.017097</td><td>-</td><td>Down</td></tr>
<tr><td>circRNA_1399|Chr12:72783683_72786789_+</td><td>0</td><td>0.065857</td><td>0.07856</td><td>0</td><td>0</td><td>0</td><td>0.00236224</td><td>-8.725627997</td><td>0.017964</td><td>mmu_circ_0004167</td><td>Down</td></tr>
<tr><td>circRNA_3276|Chr19:60563162_60564294_-</td><td>0</td><td>0</td><td>0</td><td>0.021311</td><td>0.066274</td><td>0.083794</td><td>485.593444</td><td>8.923605134</td><td>0.019097</td><td>mmu_circ_0007987</td><td>Up</td></tr>
<tr><td>circRNA_6412|ChrMT:13917_14141_-</td><td>0.055741</td><td>0.076833</td><td>0</td><td>0</td><td>0</td><td>0</td><td>0.00217044</td><td>-8.847800038</td><td>0.022302</td><td>-</td><td>Down</td></tr>
<tr><td>circRNA_4926|Chr5:143080224_143081077_-</td><td>0.055741</td><td>0</td><td>0.07856</td><td>0</td><td>0</td><td>0</td><td>0.00209622</td><td>-8.897992138</td><td>0.022728</td><td>mmu_circ_0012281</td><td>Down</td></tr>
<tr><td>circRNA_4662|Chr5:65301933_65303208_-</td><td>0</td><td>0</td><td>0</td><td>0.0959</td><td>0.044182</td><td>0.031423</td><td>441.660024</td><td>8.786792446</td><td>0.026284</td><td>-</td><td>Up</td></tr>
<tr><td>circRNA_0020|Chr1:10315205_10321709_+</td><td>0</td><td>0.021952</td><td>0</td><td>0.106556</td><td>0.077319</td><td>0.052371</td><td>9.88536127</td><td>3.305293691</td><td>0.027523</td><td>-</td><td>Up</td></tr>
<tr><td>circRNA_5639|Chr7:132771578_132779385_-</td><td>0.078037</td><td>0.307331</td><td>0.37036</td><td>0.84179</td><td>0.629599</td><td>0.356124</td><td>2.04698487</td><td>1.03350044</td><td>0.028638</td><td>mmu_circ_0013953</td><td>Up</td></tr>
<tr><td>circRNA_3239|Chr19:45635735_45640521_-</td><td>0</td><td>0.098785</td><td>0.04489</td><td>0.138522</td><td>0.154638</td><td>0.199011</td><td>3.25495289</td><td>1.702636661</td><td>0.029942</td><td>mmu_circ_0000956</td><td>Up</td></tr>
<tr><td>circRNA_4635|Chr5:43758222_43773659_-</td><td>0.022296</td><td>0.098785</td><td>0.06734</td><td>0</td><td>0.033137</td><td>0</td><td>0.14914109</td><td>-2.745250352</td><td>0.031002</td><td>mmu_circ_0001345</td><td>Down</td></tr>
<tr><td>circRNA_0501|Chr10:25283893_25289730_-</td><td>0</td><td>0</td><td>0</td><td>0.042622</td><td>0.077319</td><td>0.041897</td><td>434.210838</td><td>8.762251926</td><td>0.031381</td><td>mmu_circ_0002127</td><td>Up</td></tr>
<tr><td>circRNA_6109|Chr9:57056714_57057861_+</td><td>0</td><td>0</td><td>0</td><td>0.042622</td><td>0.077319</td><td>0.041897</td><td>434.210838</td><td>8.762251926</td><td>0.031381</td><td>mmu_circ_0015709</td><td>Up</td></tr>
<tr><td>circRNA_5367|Chr7:28990988_28991325_+</td><td>0</td><td>0</td><td>0</td><td>0.021311</td><td>0.110456</td><td>0.031423</td><td>437.333807</td><td>8.772591065</td><td>0.032737</td><td>-</td><td>Up</td></tr>
<tr><td>circRNA_0961|Chr11:67192403_67254873_+</td><td>0.144925</td><td>0</td><td>0.08978</td><td>0</td><td>0</td><td>0</td><td>0.00111457</td><td>-9.809297928</td><td>0.032886</td><td>-</td><td>Down</td></tr>
<tr><td>circRNA_1632|Chr13:42055400_42055542_+</td><td>0</td><td>0</td><td>0</td><td>0</td><td>0.088365</td><td>0.07332</td><td>459.870322</td><td>8.845083286</td><td>0.033988</td><td>mmu_circ_0000455</td><td>Up</td></tr>
<tr><td>circRNA_0913|Chr11:54005394_54014456_-</td><td>0</td><td>0</td><td>0</td><td>0.0959</td><td>0</td><td>0.062845</td><td>424.462062</td><td>8.729491803</td><td>0.034196</td><td>-</td><td>Up</td></tr>
<tr><td>circRNA_5311|Chr6:145147636_145149357_+</td><td>0</td><td>0.065857</td><td>0.03367</td><td>0.159834</td><td>0.088365</td><td>0.146639</td><td>3.7023197</td><td>1.888429481</td><td>0.0342</td><td>-</td><td>Up</td></tr>
<tr><td>circRNA_0157|Chr1:66801049_66802168_-</td><td>0.066889</td><td>0.076833</td><td>0.10101</td><td>0.213111</td><td>0.27614</td><td>0.178062</td><td>2.19807656</td><td>1.136241635</td><td>0.039112</td><td>mmu_circ_0008912</td><td>Up</td></tr>
<tr><td>circRNA_4487|Chr4:149156607_149161694_-</td><td>0</td><td>0.043904</td><td>0.07856</td><td>0</td><td>0</td><td>0</td><td>0.00277232</td><td>-8.494691761</td><td>0.039783</td><td>mmu_circ_0011414</td><td>Down</td></tr>
<tr><td>circRNA_3425|Chr2:37624173_37627526_-</td><td>0</td><td>0.054881</td><td>0.06734</td><td>0</td><td>0</td><td>0</td><td>0.00279034</td><td>-8.485341653</td><td>0.040538</td><td>-</td><td>Down</td></tr>
<tr><td>circRNA_3607|Chr2:119057281_119064097_+</td><td>0</td><td>0.054881</td><td>0.06734</td><td>0</td><td>0</td><td>0</td><td>0.00279034</td><td>-8.485341653</td><td>0.040538</td><td>-</td><td>Down</td></tr>
<tr><td>circRNA_1980|Chr14:50951404_50963869_+</td><td>0.044592</td><td>0.065857</td><td>0</td><td>0</td><td>0</td><td>0</td><td>0.00262389</td><td>-8.574075958</td><td>0.042157</td><td>-</td><td>Down</td></tr>
<tr><td>circRNA_2888|Chr18:21010677_21020999_+</td><td>0</td><td>0</td><td>0</td><td>0.031967</td><td>0.077319</td><td>0.041897</td><td>408.867587</td><td>8.675489886</td><td>0.042652</td><td>mmu_circ_0007159</td><td>Up</td></tr>
<tr><td>circRNA_0611|Chr10:75274017_75279961_+</td><td>0</td><td>0.032928</td><td>0</td><td>0.053278</td><td>0.110456</td><td>0.07332</td><td>6.85938296</td><td>2.778078805</td><td>0.046728</td><td>mmu_circ_0000180</td><td>Up</td></tr>
<tr><td>circRNA_3643|Chr2:122441559_122486090_+</td><td>0</td><td>0</td><td>0</td><td>0</td><td>0.077319</td><td>0.125691</td><td>594.668231</td><td>9.215941195</td><td>0.047186</td><td>-</td><td>Up</td></tr>
<tr><td>circRNA_6390|Chr9:121727316_121731565_+</td><td>0.066889</td><td>0.032928</td><td>0.10101</td><td>0</td><td>0.022091</td><td>0.031423</td><td>0.22704017</td><td>-2.138980508</td><td>0.047992</td><td>mmu_circ_0001861</td><td>Down</td></tr>
<tr><td>circRNA_5074|Chr6:40685277_40747150_+</td><td>0.022296</td><td>0.021952</td><td>0.06734</td><td>0</td><td>0</td><td>0</td><td>0.00276945</td><td>-8.496183478</td><td>0.048353</td><td>-</td><td>Down</td></tr>
<tr><td>circRNA_4728|Chr5:97027831_97045167_+</td><td>0</td><td>0</td><td>0</td><td>0.063933</td><td>0.066274</td><td>0.020948</td><td>390.593651</td><td>8.609524688</td><td>0.048406</td><td>-</td><td>Up</td></tr>
<tr><td>circRNA_4987|Chr6:4529068_4531010_+</td><td>0</td><td>0</td><td>0</td><td>0</td><td>0.066274</td><td>0.083794</td><td>434.906941</td><td>8.764562925</td><td>0.04887</td><td>-</td><td>Up</td></tr>
<tr><td>circRNA_0845|Chr11:29470315_29477458_+</td><td>0.022296</td><td>0.032928</td><td>0.05611</td><td>0</td><td>0</td><td>0</td><td>0.00278744</td><td>-8.486843064</td><td>0.049192</td><td>mmu_circ_0002941</td><td>Down</td></tr>
<tr><td>circRNA_4055|Chr3:126796838_126798280_+</td><td>0</td><td>0.032928</td><td>0.08978</td><td>0.170489</td><td>0.121502</td><td>0.125691</td><td>3.07279354</td><td>1.619550838</td><td>0.049297</td><td>mmu_circ_0010434</td><td>Up</td></tr>
</table>

Table S2. Dysregulated miRNA transcripts between the OVX and control group

miRNA_id	Control-1	Control-2	Control-3	OVX-1	OVX-2	OVX-3	log2 FoldCl	P value	mirbase accession No	up_down
mmu-miR-206-3p	8.277463	7.6557592	7.375485	4.612796	5.853252	5.09403	-2.582876	0.004520393	MIMAT0000239	down
mmu-miR-7219-5p	3.075996	4.0498627	4.352631	1.162993	1.624653	1.788028	-2.300939	0.005793625	MIMAT0028406	down
mmu-miR-6351	1.50173	2.5798743	3.464429	-0.10631	9.99E-05	1.074244	-2.192666	0.032335586	MIMAT0025094	down
mmu-miR-6238	3.400861	3.5460212	4.599456	1.624653	2.948129	1.95142	-1.674046	0.037992906	MIMAT0024859	down
mmu-miR-7042-5p	6.710346	6.6866595	7.060366	5.983216	6.102368	6.360797	-1.591437	0.015119295	MIMAT0027988	down
mmu-miR-6378	5.233222	5.9681263	6.323487	3.925862	4.47629	4.722292	-1.466798	0.021121958	MIMAT0025124	down
mmu-miR-494-3p	9.029013	8.898522	9.853403	7.375485	7.918452	8.095436	-1.463855	0.01660161	MIMAT0003182	down
mmu-miR-7005-5p	6.994112	7.5467864	8.095436	5.473399	6.44276	6.473857	-1.415439	0.036342016	MIMAT0027914	down
mmu-miR-8094	1.304528	0.9708703	1.712751	0.018503	0.860951	-0.1376	-1.0821	0.045487101	MIMAT0031395	down
mmu-miR-135a-1-3p	1.99719	1.8943377	2.579874	1.014845	1.704753	1.249096	-0.834236	0.04710109	MIMAT0004531	down
mmu-miR-8117	6.863204	6.7103462	7.272118	6.023557	6.186128	6.233462	-0.800841	0.011111978	MIMAT0031423	down
mmu-miR-7009-5p	3.721905	3.9828907	3.925862	3.006757	3.229958	3.349426	-0.681505	0.005972522	MIMAT0027922	down
mmu-miR-378a-5p	4.02159	3.6397235	3.400861	2.896768	3.317958	2.896768	-0.650227	0.046827098	MIMAT0000742	down
mmu-miR-7226-5p	3.464429	3.1687037	3.811846	2.718856	2.916531	2.991545	-0.606016	0.040452096	MIMAT0028420	down
mmu-miR-1892	5.015669	5.0102956	5.116406	5.784253	5.594027	5.575276	0.6037286	0.001302676	MIMAT0007871	up
mmu-miR-214-3p	6.059097	6.1480526	5.983216	6.7747	6.655531	6.655531	0.6317989	0.00052319	MIMAT0000661	up
mmu-miR-344i	3.846642	3.6650502	3.229958	4.282171	4.312461	4.265569	0.7061837	0.018320982	MIMAT0022503	up
mmu-miR-5113	1.990924	2.5950532	2.961162	3.507467	3.679107	3.464429	1.0346212	0.023522891	MIMAT0020621	up
mmu-miR-3473e	5.010296	5.179359	4.9853	5.968126	6.262474	6.102368	1.0526713	0.000549525	MIMAT0025587	up
mmu-miR-3473f	5.418252	4.4762899	4.53086	5.919941	6.167272	5.594027	1.085279	0.035423272	MIMAT0031390	up
mmu-miR-7221-3p	1.878784	2.7188557	1.423697	3.050859	3.217877	3.201332	1.1495768	0.039897635	MIMAT0028411	up
mmu-miR-5128	7.447303	6.8149085	6.944727	8.095436	8.561126	8.561126	1.3369166	0.005690304	MIMAT0020639	up

Table S3. Dysregulated mRNA transcripts between the OVX and control group

gene_id	Control-1	Control-2	Conrol-3	OVX-1	OVX-2	OVX-3	log2FoldChange	P value	up_down
Lep	0	0	0.0215	0.574373	0.489624	0.244954	5.979271989	4.04E-10	up
Inmt	0	0	0.063537	0.091869	0.715465	0.558888	4.442358921	0.004313	up
Itpka	0	0	0.018067	0.223704	0.275709	0.033087	3.435155856	0.006847	up
Krt79	0.01214	0.012391	0	0.071281	0.075687	0.034228	3.124701333	0.047492	up
Vat1l	0.030209	0.005866	0.005827	0.077795	0.118666	0.06514	3.12343578	0.000487	up
Ambn	0.013533	0	0	0.096229	0.051617	0.09491	3.120025713	0.031553	up
Ces1f	0.02163	0.042029	0.020659	0.329099	0.2555	0.193431	3.096837452	9.76E-06	up
Nnat	0.04487	0.00863	0	0.335784	0.175784	0.088617	3.073336843	0.007526	up
Npy	0.350336	0	0.229142	1.65362	1.84938	1.77856	3.00298838	1.34E-06	up
Chrne	0.015207	0.06077	0.015216	0.08662	0.431647	0.279381	2.634191668	0.010994	up
Scd1	19.7982	15.1224	21.0181	112.812	119.594	124.867	2.620762853	3.77E-81	up
Glb1l2	0.079858	0.036246	0.076873	0.45943	0.45889	0.470626	2.601017478	4.92E-15	up
Klhdc7a	0.047998	0.007878	0.023756	0.210554	0.176137	0.127726	2.56979642	6.95E-07	up
Pigr	0.009869	0.009768	0.00948	0.047193	0.064345	0.04039	2.513809162	0.011282	up
Lhx9	0	0.005245	0	0.024243	0.021165	0.031501	2.505424868	0.014425	up
Plin1	0.992752	0.701798	1.29902	5.86859	5.31578	6.26166	2.493634191	2.89E-38	up
Trhde	0.004265	0.004013	0.007892	0.033264	0.056642	0.044299	2.47636124	0.007288	up
Cyp2f2	0.533815	0.307357	0.621747	2.45624	2.95138	2.95931	2.419810081	2.02E-20	up
Apoa2	0.096342	0.031148	0.062471	0.360281	0.570346	0.14393	2.418472481	0.00452	up
Tmem45b	0.246995	0.095554	0.109681	1.00971	0.819536	0.45206	2.372919918	5.49E-05	up
Klb	0.026377	0.095883	0.03186	0.266787	0.299254	0.255555	2.366030782	3.00E-06	up
Aicda	0.020972	0.010088	0.030024	0.122915	0.133048	0.037234	2.216106261	0.038391	up
Mal	0.095963	0.107853	0.024675	0.169834	0.545412	0.389572	2.203320156	0.003065	up
Hoxa11	0.128208	0.076131	0.089574	0.544016	0.415006	0.470779	2.199744206	2.44E-05	up
Wdr93	0.015384	0	0.022024	0.027707	0.052222	0.047674	2.186126794	0.033384	up
Arg1	0.061314	0.0602	0.074678	0.100322	0.519231	0.29051	2.175520005	0.006631	up
Cadm2	0.005121	0.002299	0.01585	0.010438	0.059785	0.046565	2.171592221	0.033587	up
Cfd	25.2213	14.9287	35.7751	131.94	112.699	105.726	2.155321616	1.92E-19	up
Adcyap1r1	0.124853	0.061546	0.153597	0.39954	0.678223	0.487668	2.154527444	4.94E-08	up
Lrrtm4	0.009439	0.011456	0.016935	0.026356	0.066062	0.06347	2.147109037	0.002944	up
Serpind1	0.039778	0.028328	0.009373	0.133224	0.123931	0.061111	2.141725812	0.018311	up
Lctl	0.105162	0.02064	0.051358	0.352414	0.282462	0.209769	2.133029255	9.15E-05	up
H2-M2	0.027768	0.337734	0.080151	0.385156	0.740331	0.700753	2.096945626	7.15E-06	up
Adora1	0.030186	0.025657	0.017089	0.080888	0.134574	0.130093	2.094057312	0.000502	up
Fstl4	0.002741	0.033705	0.010509	0.044335	0.076268	0.07509	2.077412	0.000999	up
Adig	0.381135	0.276199	0.492621	2.65173	1.30028	1.11372	2.065938458	0.000683	up
Acat3	0.082325	0.011543	0	0.119763	0.128375	0.105621	2.054186557	0.00669	up
A530016L1	0.141469	0.074254	0.149554	0.545518	0.454081	0.526703	2.047634182	2.83E-08	up
Tmem196	0.03644	0.016595	0.027007	0.050512	0.143843	0.101005	2.002673803	0.004065	up
Mcpt4	0.123548	0.071475	0	0.226807	0.240438	0.306085	1.989616476	0.030241	up
Sucnr1	0.114793	0.030378	0.015067	0.227975	0.260838	0.169223	1.984920817	0.013965	up
Thrsp	1.3471	1.57464	1.53987	6.41251	6.52182	5.33963	1.962326306	2.08E-18	up
Lgals12	0.141236	0.05804	0.116664	0.421401	0.36798	0.499867	1.943789117	8.42E-07	up
Serpina3n	0.214899	0.152956	0.186378	0.78754	0.693116	0.651074	1.938905176	4.70E-07	up
Cidec	0.930238	0.448985	1.47326	3.75042	3.57445	3.61587	1.890124089	1.37E-18	up
Kcnk3	0.081543	0.064826	0.016545	0.154527	0.207629	0.255872	1.841876102	0.002017	up
Mpz	0.628887	0.251355	0.466087	0.648481	2.79434	1.67486	1.802885783	0.039007	up
Hoxa13	0	0.315926	0	0.487294	0.326719	0.504348	1.79862835	0.03229	up
Acvr1c	0.094339	0.024298	0.053753	0.178328	0.21731	0.208089	1.786271613	1.43E-08	up
2210407C	0.130667	0.061108	0.183883	0.513881	0.215717	0.390771	1.767670938	0.010442	up
Snhg11	0.041729	0.011753	0.011661	0.094587	0.082836	0.0505	1.737616913	0.007426	up
Stfa2	0.183923	1.14873	1.16379	3.51508	1.60995	2.52378	1.710005639	0.001426	up
Ren1	0.080018	0.118722	0.019859	0.150088	0.141022	0.311974	1.697014911	0.037008	up
Trim34b	0.051784	0.063699	0.098646	0.216885	0.089815	0.219693	1.68426062	0.015694	up
Apoc1	0.324217	0.59282	0.59702	2.22058	2.0849	1.51894	1.627452355	0.00057	up
Pllp	0.120228	0.033718	0.117817	0.173044	0.428007	0.294738	1.616767357	0.013245	up
Adh7	0.018616	0.041528	0.011713	0.060739	0.053863	0.070942	1.613053946	0.026742	up
Slc7a10	0.182142	0.014943	0.121548	0.464935	0.413026	0.557651	1.601983855	0.001282	up
Pck1	0.398109	0.110984	0.444665	1.20669	0.962138	0.951088	1.594152781	1.87E-07	up
Cxcl13	0.220165	0.324639	0.118597	0.641264	0.467689	0.715942	1.550220342	0.002747	up
Rgs8	0.0149	0.033067	0.010918	0.03107	0.052127	0.067475	1.514563608	0.04541	up

Dner	0.032787	0.03179	0.071453	0.140542	0.120839	0.102088	1.507983129	0.044638	up
Retn	0.184928	0.101663	0.377402	0.868157	0.818424	0.606958	1.480010614	0.001775	up
Coch	0.132586	0.090707	0.072032	0.270765	0.284876	0.225976	1.451414527	0.005206	up
Als2cr12	0.01263	0.01571	0.011574	0.051608	0.011843	0.036278	1.419522604	0.042797	up
Igf2bp3	0.039729	0.084479	0.067314	0.161518	0.166694	0.16405	1.402485607	0.001298	up
Emilin3	0.059438	0.103819	0.06836	0.214946	0.257931	0.159169	1.401829125	0.009144	up
Aoc3	0.776864	0.561637	0.656952	1.47016	2.06173	1.91734	1.400746754	3.87E-11	up
Stfa3	0.806515	3.1156	3.8835	8.55717	4.57822	8.75441	1.396677042	0.007487	up
Lrrc15	0.140134	0.272703	0.121006	0.56212	0.401949	0.458431	1.391220631	8.98E-06	up
Prlr	0.033797	0.024845	0.041974	0.106384	0.087846	0.094396	1.385873717	7.06E-05	up
Lca5l	0.069688	0.047794	0.006812	0.138288	0.097158	0.151656	1.381726023	0.030938	up
Klhl32	0.040395	0.032554	0.07104	0.108701	0.125097	0.143634	1.358602067	0.018102	up
BC100530	0.896145	3.41508	3.7689	8.38562	4.52978	7.70628	1.355563212	0.004319	up
Epha3	0.22085	0.174054	0.146248	0.407181	0.471137	0.454401	1.353475439	6.23E-06	up
Has1	0.155931	0.06646	0.038116	0.199911	0.298854	0.211646	1.34801115	0.013915	up
Arxes2	0.085428	0.129059	0.223026	0.504369	0.358581	0.251766	1.347676048	0.026109	up
Rarres2	2.92031	3.86626	5.17546	12.1789	10.452	9.89153	1.328054097	6.54E-14	up
Cbln1	0.667806	0.973573	0.896407	2.30705	1.77758	2.30898	1.318610863	1.41E-07	up
Cd209g	0.158203	0.337003	0.294984	0.834738	0.733801	0.558343	1.31721297	0.00507	up
Car5b	0.245675	0.257866	0.506046	0.816524	0.842535	0.900372	1.301954463	1.29E-06	up
Apod	1.48034	0.785555	1.78812	2.04018	4.66775	3.43949	1.250197345	0.010319	up
Cd209f	0.682784	1.24139	1.33255	3.75305	2.81925	2.2187	1.246108236	0.001839	up
Hcar1	0.211917	0.082736	0.241422	0.495416	0.293986	0.541831	1.232268483	0.004776	up
Mmp3	0.246441	0.190702	0.25519	0.559383	0.467219	0.686694	1.210803204	0.002193	up
Rpl39l	0.375159	0.391637	0.568091	1.35712	0.795619	0.623889	1.200121308	0.023835	up
Bfsp2	0.910927	1.44015	1.49147	3.25955	3.00662	3.11481	1.196870509	1.10E-08	up
Cntnap2	0.014975	0.039757	0.028165	0.045253	0.077526	0.068371	1.194907884	0.045152	up
Tmeff1	0.153523	0.073794	0.082456	0.252885	0.159941	0.300359	1.194113797	0.025072	up
Serpina3c	0.260606	0.251313	0.467852	0.77405	0.683903	0.775373	1.188852172	0.000208	up
Fabp4	28.3712	38.1337	39.1467	89.1718	86.3301	76.2267	1.186756218	3.45E-18	up
Myl4	4.47579	6.89762	7.70447	15.5224	14.2333	13.7003	1.182610919	3.47E-14	up
Igfals	0.430575	0.231959	0.239089	0.784187	0.694067	0.829235	1.177470984	0.001524	up
Gabra3	0.075826	0.023702	0.045992	0.083897	0.161372	0.091277	1.16663122	0.034143	up
Cxcl14	4.40636	7.12942	5.70116	12.1929	14.8645	14.5025	1.164867626	6.21E-15	up
LOC10263	0.027378	0.025078	0.027359	0.067439	0.065868	0.080752	1.160323104	0.026877	up
Bcan	0.053204	0.107854	0.048055	0.155118	0.172516	0.143977	1.157507354	0.037658	up
Igll1	5.50344	12.1966	11.133	20.729	23.7605	20.4324	1.151600599	3.37E-09	up
Scn7a	0.266388	0.204351	0.152783	0.370711	0.574562	0.509641	1.142340732	0.000127	up
Vpreb1	5.76148	10.4725	8.30841	19.6237	19.6421	15.5635	1.14083279	4.75E-09	up
Cygb	0.918259	0.754258	1.01868	2.00032	2.55476	2.19459	1.132953078	4.23E-07	up
Rsph9	0.183194	0.274291	0.285595	0.772586	0.618437	0.762028	1.126434708	4.49E-06	up
Vsig4	0.306362	0.195835	0.308974	0.681973	0.601159	0.427953	1.124691977	0.006249	up
Adipoq	20.6738	34.6588	32.7673	73.101	60.6732	60.158	1.105175547	8.66E-09	up
Tusc5	0.379435	0.339564	0.375249	0.941762	0.559886	0.986943	1.098221321	0.000486	up
Sncg	0.938625	0.713681	0.665492	2.49095	1.57649	1.28013	1.096269722	0.004648	up
Aldh1a3	0.205807	0.338816	0.192469	0.354263	0.691542	0.564702	1.089203608	0.003041	up
Dkk3	1.27171	1.30585	1.23866	2.7513	2.65823	2.77571	1.089162198	2.71E-09	up
Egfl6	0.393729	0.307584	0.541903	0.949905	0.867364	0.964242	1.088733899	2.14E-05	up
Bmper	0.264489	0.343079	0.425741	0.789021	0.746572	0.739445	1.086933727	8.85E-05	up
Wnt10a	0.257306	0.345188	0.296072	0.450407	0.756899	0.780236	1.083856889	0.023039	up
Fam26e	0.534683	0.32284	0.682732	1.27291	0.91759	1.12851	1.065250354	0.000926	up
Vnn1	0.09168	0.106507	0.202791	0.271242	0.353829	0.143406	1.053493126	0.041152	up
C1qtnf7	0.124702	0.118403	0.147618	0.247269	0.280189	0.281169	1.043539508	0.002051	up
Dntt	4.18239	9.72273	5.34084	12.3296	14.2835	13.7421	1.038473288	1.09E-06	up
Gm12260	2.23619	4.15437	3.07417	9.71094	6.29558	4.35828	1.038051456	0.04033	up
Clec4n	1.05543	1.35589	1.53118	3.45909	2.25782	2.50304	1.037695984	0.000219	up
Gm15411	0	0.041026	0.081532	0.119201	0.083642	0.299698	1.03268653	0.013344	up
Klra1	0.377369	0.740262	0.596294	1.30423	1.22592	1.26152	1.027843065	0.000273	up
Ccdc3	2.09748	1.57177	2.01703	3.49921	3.92283	4.51759	1.022126356	2.88E-08	up
Enpp2	0.731363	0.673155	0.879291	1.73094	1.61344	1.50825	1.019784799	3.30E-09	up
Calml3	1.29525	1.6804	1.6285	3.81902	2.98698	2.77442	1.014314704	2.15E-05	up
Fam198a	0.261098	0.265815	0.195032	0.520554	0.478581	0.474485	1.005260994	0.000582	up
Islr2	0.161241	0.130598	0.097075	0.291679	0.26171	0.257804	0.999585009	0.010302	up

1500015O	11.1211	8.89346	9.10593	24.7397	19.551	17.0577	0.9841399	0.000101	up
Tnn	1.16699	1.97068	1.14716	3.19425	2.5249	3.01332	0.972833614	2.41E-06	up
Nmnat2	0.120577	0.091178	0.081897	0.165314	0.172387	0.256183	0.963828643	0.005503	up
Arl6	0.798708	1.02137	0.824357	2.13062	1.84833	1.65952	0.963334223	0.000174	up
Mmp12	0.080135	0.126019	0.044931	0.116474	0.18368	0.189141	0.959526776	0.038813	up
Acss3	0.193448	0.133733	0.200173	0.398838	0.432377	0.421031	0.958179464	0.002031	up
Pafah1b3	8.19094	11.9235	11.5831	23.4674	22.5038	20.8446	0.948276013	7.91E-10	up
Olfml1	0.294353	0.486143	0.380453	1.08214	0.661427	0.725995	0.944912029	0.003347	up
Fcrla	10.0153	12.9548	14.7529	24.8793	26.6082	24.3805	0.941503632	2.50E-11	up
Gm4705	2.17006	4.53883	2.65741	6.43264	5.61239	6.05635	0.941014454	0.0085	up
Vpreb2	0.362331	0.996943	0.372956	0.980626	1.09048	0.884479	0.935988835	0.006686	up
C1qtnf2	0.43655	0.309414	0.397978	0.650786	0.888965	0.944773	0.933635308	0.030327	up
Plat	0.696081	0.590147	0.674908	1.14725	1.51795	1.39467	0.928650424	6.94E-05	up
Plekhg4	0.079734	0.087184	0.039978	0.195704	0.116829	0.139392	0.92618416	0.017119	up
Mzb1	3.81766	5.68058	6.80879	11.0645	10.6776	10.9817	0.92111765	2.01E-08	up
Fbln1	1.78034	1.66976	1.33118	3.1895	3.60699	3.03587	0.918283348	1.69E-07	up
Omd	1.14427	1.79148	1.3139	2.6747	2.33768	2.88575	0.914843613	4.35E-08	up
Adamdec1	0.196953	0.27313	0.283531	0.457657	0.499541	0.523081	0.910184697	0.006953	up
Gpc3	0.43264	0.34907	0.324833	0.635977	1.05628	0.598973	0.909970051	0.021253	up
B3galt2	0.555292	0.376474	0.400684	0.87578	0.919077	0.702214	0.904357338	6.44E-05	up
Hist1h2an	61.1476	136.54	128.849	226.08	176.646	155.306	0.900360725	0.000691	up
Pde9a	0.147072	0.221446	0.094976	0.267656	0.244589	0.47437	0.895979285	0.026645	up
Cox16	3.10177	3.38807	2.80008	3.80568	3.90169	3.67702	0.89200084	0.025206	up
Akr1c14	0.538719	0.927008	0.611976	1.35669	1.32301	1.07073	0.887708066	0.000242	up
Serpina12	0.049966	0.084781	0.090486	0.172658	0.11645	0.263494	0.875541328	0.03191	up
Mdk	0.919669	1.49111	1.41481	3.06949	2.70242	2.45266	0.874121377	0.000311	up
Gm31493	0.847909	0.981406	1.59684	2.08103	2.7296	1.99029	0.873335665	0.000831	up
Gdf10	1.55424	1.39329	1.38013	2.4737	3.08255	2.92293	0.86818576	2.61E-05	up
Gulp1	0.506849	0.55312	0.554506	1.02281	1.08873	0.94699	0.868024738	0.000106	up
Dpt	1.68605	2.00621	2.30069	3.98255	3.52673	3.82959	0.867803835	1.09E-05	up
Tmem30b	0.183729	0.368331	0.259656	0.536826	0.39215	0.553215	0.866827594	0.021704	up
Cplx2	2.18495	2.68473	2.84381	4.3694	4.99937	5.34943	0.860466704	5.20E-09	up
Vpreb3	30.3316	44.7121	48.7023	71.9319	82.1438	78.6898	0.859936114	4.83E-09	up
Prr16	0.275478	0.357318	0.185669	0.491153	0.576487	0.433289	0.849626256	0.01779	up
Rag1	8.68945	8.74988	13.0069	15.8045	19.502	19.9806	0.845641167	2.66E-06	up
Cd79b	42.5614	56.4507	64.7358	98.5801	100.699	103.82	0.843623917	3.82E-10	up
Lum	22.1656	35.5636	25.9803	53.6786	44.7681	50.6743	0.842366558	2.30E-06	up
Stfa2l1	15.2373	32.073	29.9034	52.8363	42.2801	48.0331	0.835788127	0.000356	up
Bend5	0.254767	0.255392	0.305448	0.431985	0.588885	0.576472	0.835160259	0.005784	up
Klk8	0.404192	0.782672	0.475549	1.18028	1.18197	0.776357	0.83226684	0.025993	up
Mrap	1.39097	1.76803	1.75953	2.93818	2.92929	2.76667	0.830231413	0.002783	up
4930426D	0.486219	0.235464	0.701811	0.937377	0.683407	1.08156	0.829774901	0.00378	up
Col3a1	26.6871	35.4516	29.2315	61.5968	52.5041	55.1574	0.82128628	4.02E-10	up
Csrp2	5.43629	9.07905	7.18109	13.7001	13.3259	12.2779	0.819524383	6.59E-06	up
Medag	0.687674	0.403915	0.510113	0.783443	0.982766	1.11684	0.817613548	0.000817	up
Spin4	0.256234	0.248015	0.238053	0.386042	0.44341	0.459564	0.809541224	0.015643	up
Fbp1	0.279979	0.489654	0.748317	0.956481	0.713927	1.26851	0.807639216	0.009414	up
Pabpc4l	0.102967	0.100266	0.075308	0.150701	0.131329	0.209126	0.807547697	0.031379	up
Ccdc148	0.146116	0.198517	0.16179	0.382668	0.235158	0.257067	0.804781893	0.016061	up
Twist1	1.65442	1.87047	1.74106	3.36943	2.9537	3.19078	0.795924256	0.001074	up
Edaradd	0.383088	0.382531	0.503528	0.651985	0.765768	0.894772	0.795685296	3.57E-05	up
Sapcd1	0.41682	0.243169	0.174737	0.726435	0.424717	1.02529	0.795125415	0.026076	up
Gfra1	1.7533	2.2949	2.20711	3.38128	3.97845	3.79251	0.791317414	3.99E-07	up
Dusp2	5.34864	6.88995	8.68959	11.622	12.5701	13.0196	0.791120086	1.90E-06	up
Slc16a4	0.175794	0.197453	0.176733	0.381427	0.342378	0.338955	0.788910164	0.047516	up
Cpxm1	1.55129	3.06394	2.19369	3.81324	3.81698	4.1887	0.78012831	2.03E-05	up
Ptn	0.625892	1.17957	0.622716	1.51123	1.48283	1.4806	0.7775608	0.000348	up
Aldh3a1	0.292107	0.3381	0.503358	0.707132	0.500138	0.623336	0.775960393	0.025572	up
Tmem132	0.538454	0.531813	0.609694	0.954418	1.09672	1.16819	0.773833406	8.35E-05	up
Aspn	5.82041	5.7483	5.09293	9.60956	8.81037	9.63185	0.772737885	1.35E-07	up
Nnmt	0.449138	0.632122	0.534097	1.08839	0.992709	0.938087	0.769602679	0.002043	up
Gm5483	16.2622	32.3554	36.1521	58.3168	40.6153	49.9792	0.767438568	0.011751	up
Fam129c	4.87989	3.31039	5.58037	8.59163	8.7475	8.25742	0.762819076	4.80E-05	up

Ly6d	32.578	54.5255	53.132	78.2423	76.8257	83.3578	0.754587935	3.26E-07	up
Tnfrsf13c	5.32759	4.70423	5.59451	7.6063	9.10142	10.1673	0.754522465	7.65E-07	up
Capsl	0.2528	0.485768	0.408035	0.634855	0.689343	0.60943	0.752427815	0.025723	up
Apln	0.172328	0.352123	0.283253	0.384747	0.407348	0.571768	0.749763988	0.042627	up
Pdzrn4	0.243424	0.312742	0.201291	0.423564	0.418999	0.42094	0.746896023	0.010666	up
Ms4a1	14.8856	15.6669	18.4669	26.4659	27.7133	31.0666	0.744817019	4.16E-08	up
Ociad2	0.597432	0.436775	0.500088	0.686123	1.04423	1.06711	0.744154993	0.005235	up
Ddc	0.261793	0.238509	0.257303	0.389586	0.410839	0.448111	0.739453963	0.045451	up
Fmo2	2.04673	2.16008	1.90797	2.8486	3.72651	3.98792	0.739240275	2.00E-06	up
Tifa	14.5553	21.2595	21.8628	31.8037	32.922	32.3304	0.736982895	6.84E-08	up
Slamf9	2.18433	2.19711	2.7361	3.55677	4.04185	4.27969	0.733621951	0.000463	up
Col23a1	0.168257	0.133434	0.11266	0.19733	0.203163	0.33531	0.731069873	0.014828	up
1-Mar	0.42539	0.519113	0.575054	0.948647	0.814425	0.963567	0.726432137	0.005833	up
Cd72	8.77204	7.92717	10.7595	15.3515	16.3171	16.4099	0.724966232	2.25E-07	up
Zkscan16	0.124399	0.122975	0.211064	0.219906	0.278473	0.279922	0.724476036	0.009352	up
B3gnt5	3.16401	3.07692	2.93823	4.48539	5.03059	5.92709	0.722733962	4.46E-07	up
Rhoh	2.52014	3.61863	3.37512	5.06221	5.04311	5.88833	0.720988161	8.98E-07	up
Tnfsf15	0.123127	0.114767	0.076059	0.166703	0.162836	0.208129	0.720465333	0.040599	up
Qpct	0.512926	0.790386	0.700955	1.29795	0.894972	1.15382	0.718239889	0.012884	up
Ptprcap	28.8569	44.8372	40.0119	63.5015	63.4915	68.3076	0.713114841	9.81E-07	up
Snn	12.794	15.1184	14.8333	23.421	22.9528	25.2374	0.710533948	2.27E-07	up
Rbm3	16.5004	26.5972	24.9674	41.5348	36.3919	34.5439	0.707254342	0.000124	up
Kcne4	0.229382	0.351167	0.53758	0.629803	0.671488	0.580209	0.707195927	0.034154	up
Lrrn4cl	0.309768	0.2815	0.329974	0.518833	0.496155	0.577594	0.703983747	0.031223	up
Slamf7	1.70991	1.81329	2.21471	3.06986	2.97628	3.54845	0.701118566	1.98E-06	up
Ddah2	2.97724	4.30771	3.58158	6.00524	6.19017	6.00625	0.700654596	6.46E-05	up
Spib	19.961	19.1039	25.1997	31.1802	38.7764	37.7218	0.695503173	6.47E-07	up
Ric3	0.154554	0.157929	0.221764	0.294378	0.257481	0.331232	0.689529747	0.013078	up
Smoc2	3.38777	3.85587	2.78278	5.37528	6.11228	5.31068	0.686576091	1.61E-05	up
Zfp433	0.198753	0.228505	0.131879	0.332455	0.328028	0.282111	0.684903121	0.029707	up
Cirbp	6.37591	7.88053	8.48865	14.1518	14.5652	10.8514	0.679640059	0.000716	up
Timd4	0.604683	0.985569	1.25525	1.86978	1.19104	1.71835	0.67775782	0.021847	up
Smoc1	1.28938	1.31961	1.42579	2.35422	2.06538	2.20559	0.675725088	0.000194	up
Bmp5	1.39598	1.50149	1.47973	2.29251	2.3894	2.25342	0.673318331	0.000125	up
Hspa12a	0.2271	0.247391	0.208947	0.284809	0.397703	0.457215	0.671024862	0.006906	up
Matn4	0.489604	0.725156	0.299438	0.899774	0.961205	0.89075	0.670020559	0.033073	up
Skint3	0.475628	0.748695	0.63045	1.13865	0.913789	0.993078	0.669916606	0.001761	up
Adamtsl1	0.106416	0.039531	0.102406	0.114645	0.171521	0.168056	0.66974234	0.045816	up
Arl5c	7.65286	12.152	12.9213	17.208	18.6684	19.7109	0.666983449	4.71E-06	up
Il7r	4.33077	4.93618	4.93687	7.14873	8.18607	8.58659	0.664570384	3.23E-06	up
Ptgfr	1.03352	1.37821	1.19498	2.07805	1.73303	1.85095	0.664408385	0.000149	up
Ntrk2	0.04048	0.041543	0.045553	0.067611	0.083152	0.051765	0.663990726	0.012583	up
Zfp286	0.096916	0.084524	0.146054	0.205875	0.156321	0.168709	0.66266257	0.04337	up
Dcn	58.0508	59.312	51.9265	88.5356	90.4713	93.2037	0.659668731	4.20E-07	up
Xlr4a	0.498872	0.843997	0.429932	1.07842	1.19577	0.917627	0.656852087	0.025092	up
Vit	0.717629	0.732419	0.726625	1.1406	1.22061	1.08836	0.65596489	0.000436	up
C1ra	3.48313	5.59052	4.85092	7.00794	7.68458	7.9802	0.65572323	2.18E-05	up
Gm20939	0.241676	0.407846	0.317787	0.475975	0.623959	0.539018	0.653691885	0.023279	up
Tspan12	1.19428	1.4535	1.13646	2.02211	2.09775	2.26676	0.653656076	0.001361	up
Sp6	0.359664	0.331834	0.297301	0.476561	0.659175	0.503876	0.652585476	0.025475	up
Slain1	1.53893	1.80755	2.01266	2.58354	3.24877	3.0434	0.652256526	0.00031	up
Rbp1	0.348273	0.754505	0.559642	0.963132	0.777641	0.971777	0.650862637	0.017728	up
Slc30a4	3.29976	3.41555	3.52318	4.86736	5.42497	5.71618	0.649630941	6.16E-06	up
Thbs2	3.11493	3.72883	3.46599	5.74286	5.23309	5.73034	0.648009301	5.16E-06	up
Akr1b8	0.774151	0.850113	0.987735	1.4264	1.27418	1.46779	0.646334233	0.025068	up
Gm40892	0.542741	0.354029	0.663553	0.814231	0.768762	1.32657	0.644395938	0.010704	up
Ogn	7.47717	7.14641	6.0937	10.5999	10.3087	10.6486	0.642308799	7.42E-06	up
Plscr2	0.275814	0.31768	0.337557	0.403026	0.5446	0.651097	0.642041219	0.027499	up
Ildr2	0.21745	0.139224	0.190647	0.267987	0.305901	0.323364	0.641279083	0.011839	up
Dbp	1.35823	1.1106	1.21557	2.08823	2.4261	1.72879	0.639519032	0.00364	up
2810408A	0.221045	0.164914	0.31685	0.412415	0.335252	0.406068	0.637442764	0.041361	up
Tubb2b	0.839283	1.50305	1.38469	1.84658	1.97072	1.84184	0.63717499	0.010267	up
Mkx	0.432536	0.320303	0.415103	0.577005	0.484783	0.801902	0.636593862	0.032815	up

Rag2	0.983767	1.05787	1.14634	1.38281	1.80178	1.6944	0.634430181	0.00052	up
Nostrin	0.308354	0.55571	0.561572	0.910799	0.616692	0.779603	0.632766411	0.048081	up
Ranbp3l	0.210423	0.360882	0.156491	0.408716	0.289154	0.353333	0.632259267	0.042911	up
Syt15	0.296613	0.436957	0.525137	0.543074	0.871017	0.576334	0.629286972	0.04811	up
Ccdc122	0.478699	0.433641	0.712653	0.930291	0.836354	0.694445	0.62704439	0.023007	up
Itgbl1	1.65808	1.15528	1.41115	1.8616	2.33906	2.70481	0.626458865	0.000966	up
Endou	0.794978	0.6063	0.831151	1.1971	1.20979	1.45209	0.625854023	0.00359	up
Gstt1	1.48498	2.35681	2.27584	3.48796	3.43217	3.08618	0.625346182	0.002584	up
Akap12	6.5166	6.21721	8.45264	9.86993	11.872	12.9505	0.62084703	0.000335	up
Crip1	101.12	191.878	168.778	252.258	238.344	234.978	0.611615469	0.000264	up
Lpar4	0.245809	0.355389	0.265144	0.395503	0.40493	0.484358	0.611357515	0.04881	up
Blk	5.83232	5.66161	6.90763	9.6714	9.34931	11.1972	0.606899083	2.55E-05	up
C130074G	2.15363	2.218	2.2245	3.31603	3.79233	3.59594	0.606547392	0.000404	up
Rasal1	0.231467	0.184981	0.259638	0.333439	0.389728	0.333801	0.606469573	0.025994	up
Cd79a	22.4246	24.5667	28.6051	34.9961	41.0558	43.9964	0.606167402	1.09E-05	up
Matn2	0.932976	1.27484	1.07496	1.75225	1.64591	1.58671	0.605809622	0.001842	up
Cd302	6.35647	12.6945	11.7132	16.8766	16.5844	14.8318	0.605739209	0.005372	up
Il12a	1.40023	1.12278	1.45502	2.0185	2.22906	2.27521	0.605513589	0.002601	up
Stambpl1	2.81051	3.44979	3.44995	4.60462	5.32168	5.43949	0.598468853	5.02E-05	up
C1s1	3.44886	4.08315	3.72538	5.8944	5.84443	5.71892	0.598361071	6.22E-05	up
Pdpn	0.991153	0.868938	1.07352	1.45268	1.84772	1.43212	0.597932492	0.015506	up
Cnp	18.8085	25.8059	25.5526	34.2736	36.409	38.6132	0.597452482	1.02E-05	up
Pmp22	3.85692	4.04353	4.72202	4.81799	8.36648	6.33094	0.59690047	0.019658	up
Rps27rt	16.49	34.6387	27.297	45.0519	44.506	31.4538	0.595063581	0.038377	up
9430038I0	0.417245	0.649777	0.629062	1.04755	1.00655	0.763721	0.590328252	0.017552	up
Hes6	6.73444	9.60526	9.69395	13.6234	14.4106	13.4253	0.590224323	9.61E-05	up
Gpihbp1	3.81206	3.77304	3.68749	4.33794	6.31047	6.8775	0.59008637	0.007071	up
Spp1	99.0277	149.292	111.058	186.569	173.447	192.394	0.586163237	9.20E-06	up
Slamf6	0.780829	0.72261	1.0192	1.17981	1.29385	1.40231	0.585523139	0.000108	up
Trim17	0.344502	0.746	0.484926	0.868487	0.880808	0.910924	0.584977134	0.033973	up
Lag3	1.16634	1.534	1.60667	0.740456	1.1036	0.978249	-0.585384445	0.038103	down
Zfp609	2.51441	2.10576	2.63228	1.41634	1.72439	1.89226	-0.586143801	0.002177	down
Dopey2	4.37179	4.19195	4.54388	2.53283	3.3299	3.49423	-0.587334245	0.000508	down
Epas1	11.99	8.9168	12.3454	5.39854	8.75531	8.79415	-0.58782792	0.030835	down
Ptprz1	1.11995	1.26602	1.0582	0.711557	0.78365	0.833733	-0.588790178	0.000413	down
Crtc1	1.26371	1.10211	1.29031	0.624795	0.911802	0.996817	-0.589821587	0.021893	down
Ttc26	0.342116	0.410283	0.448725	0.25897	0.322855	0.201992	-0.590930203	0.039513	down
Acan	8.03974	9.57519	8.34146	6.20155	6.04321	5.72317	-0.591857622	1.15E-05	down
Srgap1	0.555964	0.465725	0.590915	0.247697	0.388946	0.461311	-0.593784388	0.03787	down
Panx3	1.90013	2.6849	1.96852	1.36843	1.51602	1.46911	-0.594335852	0.002577	down
G6b	2.94337	3.06206	4.21837	1.82709	2.04858	3.03263	-0.597349755	0.015548	down
Grem1	2.09649	3.19606	2.91859	1.61697	1.6427	2.32015	-0.599286059	0.003567	down
Atp2b4	14.6054	11.962	15.5735	8.54053	9.79843	10.6704	-0.599503564	0.002277	down
Gpr84	1.41809	1.96071	2.14058	1.08287	1.15898	1.46938	-0.59961705	0.010424	down
Prr11	4.78049	5.11628	5.79988	2.91409	3.52814	4.09592	-0.600512007	6.19E-05	down
Atf7	4.23495	3.12514	4.32817	1.8704	2.5705	3.40146	-0.600814601	0.04234	down
Dot1l	6.48519	5.41686	6.57175	3.36727	4.85842	4.58955	-0.60264684	0.007066	down
Ssh1	3.77464	2.78121	3.65598	1.88767	2.43336	2.77655	-0.603135203	0.031281	down
Slc30a10	0.866972	0.696549	0.880826	0.650684	0.544531	0.426656	-0.605970804	0.015901	down
Ypel4	11.2417	7.81629	10.9508	7.90472	6.73034	6.37871	-0.606184266	0.033545	down
Fam222b	3.17966	2.90246	3.94408	1.87762	2.06233	2.68269	-0.606865081	0.001708	down
Agrn	2.92402	2.86071	3.18568	1.80288	2.17189	2.25844	-0.607087449	5.11E-05	down
Plagl2	6.21112	4.75146	7.05263	2.84594	3.90807	5.34354	-0.607731607	0.046023	down
Polr2a	16.6886	14.3268	19.7342	8.02883	11.7181	15.1422	-0.608013999	0.024496	down
Sp2	3.13123	3.14971	4.40569	1.80068	2.58775	2.86871	-0.610097209	0.008947	down
Garem	2.52569	2.28972	2.4007	1.64207	1.67901	1.68044	-0.61045924	0.000283	down
Col8a1	5.5034	6.92932	6.93246	4.49179	3.25049	5.24767	-0.612811627	0.000456	down
Slc9a1	4.70907	3.98164	5.4338	2.33139	2.90923	4.42248	-0.613082334	0.033728	down
Srgap3	1.56518	1.43724	1.64971	0.874793	0.948351	1.26306	-0.613231963	0.000213	down
Nol4l	1.0536	0.926118	1.07367	0.637189	0.70063	0.764394	-0.615878026	0.001261	down
Tnfrsf14	26.3927	25.4579	29.9392	18.3381	18.2098	19.4675	-0.616116452	1.61E-05	down
Lifr	15.144	15.0255	13.4866	8.27252	9.21268	11.7846	-0.618191845	5.60E-05	down
Lpar5	0.835834	0.908565	1.04426	0.492893	0.641631	0.738238	-0.619653248	0.00369	down

Abcg4	4.9258	4.15321	5.21208	3.23531	3.1889	3.16602	-0.621485182	0.003861	down
Ank1	24.2936	21.2918	25.2777	16.4721	17.5951	15.8194	-0.623175986	0.000746	down
Gm7694	1.80035	1.53546	2.08193	1.00286	1.20609	1.39236	-0.625507293	0.002258	down
Ntng2	4.79543	3.0143	4.15348	2.12429	2.74759	3.36384	-0.626478671	0.030042	down
St3gal1	8.82706	8.3536	9.90471	5.11902	5.84917	7.25054	-0.627140656	6.81E-05	down
Slc22a23	5.99053	5.26844	6.47641	3.8136	3.98238	4.2124	-0.628565976	0.000326	down
Cpt1a	14.3064	11.7685	15.1763	8.94632	8.77874	10.2617	-0.629556507	0.001664	down
Arid1a	14.3191	11.5342	16.0843	6.57057	9.62921	12.2164	-0.630983348	0.025717	down
Cpeb3	1.81322	1.22088	1.5463	0.884229	1.09523	1.18635	-0.63113822	0.046866	down
Zmiz1	7.27554	5.74469	8.21898	3.12043	4.96652	6.10488	-0.633513971	0.029086	down
Setd1a	3.85074	3.18601	4.22293	1.91482	2.79576	2.9825	-0.634214812	0.010722	down
Pacsin1	0.855069	1.01512	1.01818	0.622844	0.591234	0.7947	-0.635686981	0.003697	down
Zfp341	0.938522	1.06886	1.20568	0.770137	0.842944	0.611252	-0.637335779	0.005103	down
Myh10	6.67105	6.96543	7.36953	4.40402	5.02168	4.80707	-0.638282646	3.36E-06	down
Runx3	2.46881	2.13433	2.8441	1.29166	1.66386	1.92706	-0.639823263	0.002401	down
Xaf1	0.973821	1.20063	1.84071	0.960956	0.779251	1.15178	-0.640689681	0.01765	down
Ppard	2.18275	2.47896	2.65471	1.48413	1.58511	1.88645	-0.643016214	0.000982	down
Ncoa6	4.15799	3.70717	4.98076	1.94164	2.65856	3.84001	-0.644029243	0.019173	down
Zfp703	4.46412	3.10581	3.97858	1.82768	2.99334	2.95851	-0.64491056	0.026208	down
Gpc4	3.10754	3.06345	3.27935	1.72265	2.33028	2.28395	-0.645950019	4.58E-05	down
Ifi44	0.779897	1.36524	1.36586	0.72529	0.597617	1.01021	-0.649420968	0.027335	down
Card10	3.08307	2.91202	2.81417	1.89255	2.11798	1.89643	-0.651996217	0.000269	down
Reep6	1.22098	1.16738	1.4436	0.96438	0.968192	0.655072	-0.652970842	0.011079	down
Bcl9	1.06895	0.870425	1.27272	0.523988	0.784832	0.849639	-0.653877429	0.007789	down
Dlx3	1.36911	2.01711	1.79184	1.02976	1.04152	1.25499	-0.655093114	0.003675	down
Fam78b	1.06502	0.896339	0.740109	0.503665	0.552373	0.687167	-0.655700571	0.007404	down
Ubap2	5.955	4.85029	6.07894	2.73643	4.11753	4.29161	-0.656429715	0.009221	down
Slc35d1	0.960469	0.864996	1.07083	0.490569	0.692339	0.669988	-0.658843598	0.000362	down
Ugt8a	0.534016	0.735269	0.721164	0.478303	0.378036	0.40853	-0.659600648	0.010014	down
Tpcn1	11.5273	10.81	12.8382	6.87652	8.37241	8.46413	-0.659601787	3.31E-05	down
Entpd3	3.26302	4.1537	3.68833	2.42975	2.37536	2.3656	-0.659973089	6.43E-05	down
Phospho1	23.7212	22.4835	19.7503	14.7433	14.989	16.7764	-0.660294346	0.000133	down
Nrp2	11.8995	11.4651	12.85	7.33597	7.75299	8.78329	-0.660370283	1.14E-06	down
2310061I0	2.15484	2.07532	1.45185	0.977517	1.53422	1.43236	-0.663762014	0.002388	down
Mreg	2.5107	2.24342	1.77547	0.97729	1.84823	1.44282	-0.6640402	0.020772	down
R3hdm2	6.39233	4.68371	5.97731	2.65051	3.95735	4.70981	-0.664210203	0.021055	down
Zfp395	1.95473	1.48674	2.03756	0.935224	1.09587	1.48804	-0.667844439	0.013147	down
Zfhx3	1.99943	1.49948	2.02446	0.955071	1.20952	1.43017	-0.669411647	0.006965	down
Nav1	2.52304	1.93392	2.65398	1.20388	1.48261	1.98502	-0.670448132	0.019289	down
Mospd3	2.79792	2.75689	3.18931	1.10295	2.33766	2.62564	-0.673890392	0.019653	down
Inf2	4.73309	3.5496	4.69959	2.38938	2.97385	3.28415	-0.675090205	0.006727	down
Ppargc1b	0.850779	0.708001	0.544197	0.362953	0.568034	0.447929	-0.680041923	0.038428	down
St8sia1	0.157253	0.112825	0.143476	0.047219	0.090072	0.11541	-0.685704004	0.036395	down
Ppp1r3e	1.35465	1.41917	1.67406	0.71308	1.11658	1.1262	-0.689897893	0.026363	down
Mical3	4.48023	3.48454	4.04212	2.41253	2.89705	2.67841	-0.690686529	0.020303	down
Cit	5.43406	4.67546	5.64538	3.08973	3.47217	3.80136	-0.693798988	0.000221	down
Phactr4	2.25745	1.73865	2.30456	0.97433	1.38905	1.51376	-0.702725359	0.008084	down
Dvl3	3.13609	2.58279	3.20804	1.26904	2.08024	2.42195	-0.703263609	0.017868	down
Dqx1	0.611836	0.374743	0.559102	0.263229	0.425894	0.262117	-0.703779448	0.013458	down
Unc5cl	0.699724	0.65171	0.63875	0.422934	0.514692	0.461605	-0.704894083	0.011576	down
Ankrd52	6.78368	5.55447	6.95376	2.84186	4.26912	5.21107	-0.706228236	0.008859	down
Bcl9l	1.80789	1.20306	1.66497	0.700132	1.16128	1.05553	-0.709696391	0.044486	down
Kif21a	0.499498	0.400904	0.376055	0.191975	0.359858	0.27048	-0.711856014	0.037939	down
Sipa1l3	1.80149	1.51837	1.69822	0.825848	1.01271	1.43064	-0.711906065	0.005421	down
Ttc9	0.386998	0.28173	0.306105	0.188208	0.213524	0.200167	-0.715255038	0.03634	down
Brd4	6.63756	5.14873	6.92884	2.90303	4.30925	4.80337	-0.719329895	0.007231	down
Aff2	0.648936	0.535494	0.717662	0.358075	0.388071	0.466689	-0.719967967	0.001749	down
Ago2	25.3868	21.1262	26.0123	12.4392	14.8772	17.9741	-0.720498776	0.000469	down
Igdcc4	1.21082	1.00562	0.811175	0.516989	0.724881	0.665188	-0.720815092	0.011011	down
Aknad1	0.183245	0.135214	0.160196	0.120201	0.098074	0.070053	-0.721818117	0.020258	down
Atp1a3	7.82406	7.41044	9.57026	4.06318	5.01859	6.5367	-0.722712636	0.000369	down
Cbfa2t3	3.29292	2.68562	3.00225	1.49484	2.06315	2.22053	-0.724011713	0.005685	down
Spen	7.84925	5.95315	7.92504	3.35452	4.99407	5.60626	-0.725928631	0.008749	down

Itga10	3.14069	2.87198	3.09532	1.94762	1.69879	2.20044	-0.727808436	4.78E-05	down
Oasl1	2.01912	2.88199	3.41394	1.81956	1.58015	1.92045	-0.72851326	0.000211	down
Vdr	4.1538	4.12148	4.00012	2.26587	2.24526	3.1537	-0.728757153	3.30E-06	down
Pom121	7.20601	5.84379	8.0903	3.36465	4.24588	5.49187	-0.730183568	0.003078	down
Iqsec2	0.687779	0.504559	0.641746	0.323009	0.428179	0.441739	-0.735889787	0.004315	down
Cacna2d3	0.319044	0.329521	0.215637	0.145146	0.200477	0.253124	-0.735894673	0.031964	down
Kmt2b	6.12217	4.61614	5.7966	2.77442	3.53868	4.29537	-0.742634936	0.01448	down
Parvb	17.8878	14.6147	17.9256	8.97712	10.7912	11.1423	-0.744440573	8.62E-05	down
Pprc1	4.23652	3.31997	3.60512	1.97487	2.50162	2.3799	-0.744665187	0.004149	down
Gm15800	22.4502	17.2075	21.0711	11.3624	13.2094	13.818	-0.747294597	0.004343	down
Hipk2	11.3636	8.92808	11.8161	4.94081	6.59407	8.13746	-0.748667314	0.002371	down
Pkd2l2	0.434611	0.306894	0.441764	0.275253	0.241379	0.36085	-0.749212423	0.001959	down
Lars2	2.30124	2.11814	2.16099	1.08461	1.51299	1.4471	-0.755643823	0.000321	down
Ccdc92b	4.05559	3.30896	5.03207	2.89685	2.49041	2.29028	-0.756086586	0.001996	down
Srcap	11.5089	7.69054	10.6372	4.30791	6.54554	7.93506	-0.756118674	0.030528	down
Kcnd1	0.478687	0.30059	0.432713	0.2629	0.247011	0.204785	-0.759376515	0.031938	down
Shank3	2.54798	1.6235	2.0481	1.16918	1.27871	1.53944	-0.760114272	0.037681	down
Fhl3	7.04305	5.37547	4.9649	3.34279	4.7032	3.88749	-0.762965215	0.02376	down
Camta2	3.13242	2.32307	2.77323	1.40084	1.71489	2.19692	-0.77039955	0.015021	down
Brpf3	8.3885	6.21334	8.39161	4.24619	5.17502	4.84695	-0.772354151	0.001746	down
Bcorl1	3.14223	2.68814	3.72879	1.41852	2.04681	2.37604	-0.776118095	0.001765	down
Tcf7l1	0.528225	0.41801	0.482847	0.242799	0.311327	0.285151	-0.77950984	0.013065	down
Atxn2l	10.8292	8.30151	11.3593	4.80079	6.63233	7.46939	-0.78396081	0.005007	down
Ak8	0.249906	0.313189	0.315709	0.20576	0.160116	0.228185	-0.786233705	0.030297	down
C77080	0.747695	0.642551	0.675412	0.357151	0.508416	0.463052	-0.788709964	0.001634	down
Oas3	10.6331	13.2592	17.3706	7.43008	7.12404	10.1933	-0.791114282	0.001168	down
Atp2a2	46.8039	35.5505	32.5214	21.5386	23.7792	24.2112	-0.796663463	0.0089	down
Mbd6	4.32663	2.50021	4.01757	1.74078	2.32849	2.78321	-0.798900926	0.049714	down
Plxnb1	0.67948	0.625927	0.647	0.326815	0.353958	0.528767	-0.79958566	0.00031	down
Hspb8	12.4659	8.2793	9.43577	4.09043	8.09729	5.98793	-0.806299741	0.033712	down
Tram2	2.35288	1.82268	2.61245	0.871436	1.59351	1.27673	-0.806553044	0.016083	down
Cd3e	1.28588	1.22029	1.77829	0.657165	1.00327	0.770915	-0.81378564	0.008216	down
Nav2	0.780439	0.435354	0.709121	0.31494	0.392979	0.424729	-0.824983507	0.026033	down
Ptch2	0.177509	0.218315	0.347884	0.150916	0.183924	0.132499	-0.826471623	0.017802	down
Prr12	3.01425	1.80543	2.41729	1.04241	1.59624	1.76095	-0.835520352	0.043655	down
Ncor2	4.28629	2.95142	3.39849	1.35418	2.45233	2.59547	-0.835779884	0.028181	down
Actc1	8.77039	6.93496	5.63658	4.40776	3.43113	4.5397	-0.836457791	0.012564	down
Tead4	0.271976	0.412297	0.248961	0.109497	0.224248	0.105886	-0.839600959	0.034582	down
Sidt1	0.327371	0.254613	0.353657	0.127445	0.170785	0.170588	-0.842199638	0.024736	down
Dusp18	0.586301	0.406075	0.385682	0.14516	0.341171	0.283316	-0.843544689	0.032384	down
Pkd1l1	0.375879	0.231503	0.274963	0.134609	0.186954	0.167739	-0.845573384	0.004013	down
Trim58	10.0168	7.91378	10.8412	5.13	5.01717	6.6844	-0.845791253	0.001852	down
Col2a1	120.376	139.198	108.014	82.211	76.8356	60.9337	-0.85457691	8.66E-06	down
Bag3	7.30077	5.30505	5.96265	2.54138	4.3891	3.87949	-0.856672818	0.006056	down
Cfap57	0.101533	0.091626	0.154295	0.067437	0.093044	0.084969	-0.861053621	0.04761	down
Gp1ba	5.8641	4.72151	7.05183	2.51157	3.01629	4.2403	-0.872631807	0.001232	down
Slc38a4	1.22421	0.780845	0.674716	0.398131	0.527012	0.560012	-0.874408174	0.035493	down
Acot6	0.580552	0.851004	0.491573	0.278855	0.354746	0.434466	-0.883901854	0.024327	down
Kdm6b	3.73801	2.71613	4.03794	1.37061	2.05561	2.53127	-0.887762377	0.005586	down
Tnfrsf12a	3.83836	2.30986	2.85566	1.26246	1.63964	1.94779	-0.893548107	0.024898	down
Nos1ap	1.30765	1.01876	0.982265	0.642116	0.571601	0.635715	-0.893958963	0.00357	down
Fbn2	1.27417	1.01496	1.17217	0.615018	0.583787	0.746509	-0.904627231	9.73E-05	down
Eda2r	0.458837	0.275578	0.424128	0.132663	0.254887	0.258109	-0.927831854	0.015755	down
Ldhb	23.1208	17.2376	13.0982	8.76533	12.7392	8.20458	-0.937753096	0.036558	down
Kmt2d	11.8807	6.91776	10.3673	3.65854	5.22989	7.16605	-0.952275919	0.031435	down
Chodl	0.216619	0.159271	0.187764	0.059867	0.10437	0.116755	-0.957346376	0.031805	down
Oasl2	4.13475	5.4256	7.14701	2.19975	2.68013	4.13547	-0.961101849	0.001365	down
Atn1	2.58744	1.51933	2.57894	0.746559	1.33787	1.60193	-0.963029601	0.030746	down
Asb17	0.22333	0.63923	0.398486	0.181901	0.150028	0.156522	-0.96671096	0.043044	down
Greb1l	0.093889	0.052032	0.051564	0.03631	0.032785	0.026033	-0.996071409	0.047613	down
Map1a	1.02156	0.744732	0.789985	0.30824	0.514596	0.525041	-1.013080205	0.002051	down
Meis2	0.134619	0.118055	0.107662	0.059857	0.067943	0.076831	-1.01834232	0.009989	down
Oas2	1.78035	1.75474	2.07362	0.690293	0.80243	1.48724	-1.023527434	0.000314	down

Setd1b	5.17743	2.76307	4.63518	1.59043	2.27296	2.70676	-1.030362671	0.025267	down
Creb3l1	3.49628	3.52081	3.88415	1.43089	1.72235	2.202	-1.042416729	1.53E-08	down
Gm22	0.538494	0.459593	0.535846	0.181561	0.304659	0.278577	-1.062961159	2.03E-06	down
Slc25a42	1.67961	1.38903	1.6161	0.565787	0.962117	0.745118	-1.073189782	0.000288	down
Prss30	0.130691	0.204733	0.246903	0.101958	0.065706	0.128955	-1.077230315	0.029014	down
Paqr5	0.205813	0.376123	0.288247	0.093722	0.189699	0.141879	-1.081849466	0.004655	down
Cnnm1	0.208926	0.15259	0.170464	0.074012	0.092253	0.099874	-1.092383837	0.005672	down
Phf24	0.040749	0.040077	0.04417	0.017333	0.014429	0.018487	-1.105261396	0.029415	down
Omp	0.849628	0.44271	0.534272	0.222764	0.407274	0.203967	-1.110193114	0.038046	down
Pdlim3	13.6324	7.11641	10.7995	2.98119	7.44421	4.53562	-1.141090386	0.03329	down
Esyt3	0.084557	0.074676	0.11869	0.054049	0.049018	0.032452	-1.141468657	0.033785	down
Mamstr	0.162613	0.092356	0.115512	0.038513	0.063672	0.078535	-1.156237798	0.046116	down
Scube2	0.259409	0.189553	0.171377	0.053685	0.089984	0.140565	-1.169308805	0.009665	down
Serpina1b	1.24393	0.984652	0.916607	0.250785	0.746317	0.333079	-1.174766949	0.00843	down
Mid1	0.360197	0.332098	0.31268	0.150732	0.23495	0.210577	-1.175712501	0.008621	down
Asb4	0.701115	0.488651	0.339568	0.149663	0.259698	0.280117	-1.177483559	0.023915	down
Dusp27	1.47521	0.991039	1.0045	0.284968	0.811956	0.547103	-1.193107847	0.018269	down
Mapt	0.742703	0.433204	0.404689	0.14116	0.347731	0.245835	-1.206720035	0.048514	down
Hspb1	15.1471	8.16382	14.9707	3.95643	7.22944	6.8071	-1.235538567	0.010656	down
Slc1a1	0.180269	0.203822	0.115459	0.115435	0.02492	0.062365	-1.239096907	0.019022	down
Homer2	1.20824	0.814441	0.622491	0.285664	0.497771	0.379549	-1.242893552	0.008818	down
Sim2	0.321851	0.276047	0.172785	0.101682	0.102819	0.123554	-1.245709627	0.013881	down
Atp1b2	4.5166	2.71499	2.80914	1.08526	1.84882	1.56523	-1.264278237	0.006758	down
Scn3a	0.117973	0.060453	0.087191	0.046438	0.050702	0.035225	-1.271304156	0.028081	down
Corin	0.078603	0.187913	0.092808	0.083429	0.04523	0.041231	-1.2817416	0.008783	down
Car14	0.663081	0.413285	0.318397	0.192033	0.291386	0.117995	-1.298969893	0.027913	down
Cd300ld3	0.181421	0.567864	0.465121	0.188696	0.099456	0.183678	-1.317000466	0.040533	down
Frem2	0.120165	0.08372	0.064022	0.028144	0.060701	0.026316	-1.317502943	0.017855	down
Mybph	3.33726	2.11456	3.06544	1.09229	1.12578	1.3826	-1.344913495	0.000525	down
Mb	128.196	90.9722	56.9871	15.4961	66.5883	32.8137	-1.34622422	0.039515	down
Musk	0.415306	0.232447	0.208626	0.052178	0.183238	0.101462	-1.362299477	0.020968	down
Oas1e	0.163301	0.368434	0.364145	0.161874	0.092813	0.042064	-1.383443029	0.006452	down
Fhl1	46.8545	32.7622	28.5873	8.80595	19.9689	14.8564	-1.398441414	0.00218	down
Cnksr1	0.966493	0.637594	0.684677	0.23663	0.249496	0.336709	-1.41889559	0.000768	down
Fam19a3	0.152308	0.067294	0.067299	0.032024	0.029771	0.042727	-1.430765737	0.046259	down
Klhl40	3.67962	2.10594	3.41794	0.629225	1.98252	1.10838	-1.437105573	0.004876	down
Lmcd1	10.4337	5.4928	8.12272	2.11721	4.55436	2.69314	-1.437782915	0.00741	down
A930009A	0.211566	0.356436	0.588874	0.168854	0.077213	0.377377	-1.454930275	0.006693	down
Hif3a	0.153705	0.070342	0.18734	0.034515	0.054289	0.068259	-1.477847252	0.007516	down
Adprhl1	1.21752	0.588821	0.725018	0.145642	0.506295	0.277935	-1.509094907	0.027639	down
Coro6	2.86521	1.61095	1.28119	0.300825	1.04704	0.71625	-1.512366931	0.025289	down
Nrap	34.4146	18.3871	15.4591	4.64754	12.7962	8.41704	-1.516563327	0.041105	down
Calcr	0.19802	0.281808	0.285445	0.139738	0.063105	0.069228	-1.516681483	0.000368	down
Lrrc2	2.14695	0.875154	1.18061	0.251541	0.759842	0.502229	-1.52700387	0.03995	down
Trim72	7.03557	4.65718	3.20477	1.10855	2.76045	1.62165	-1.559091813	0.017517	down
Serpina1a	0.622151	0.690017	0.894743	0.169181	0.55276	0.154131	-1.57307973	6.66E-05	down
Myo1a	0.042456	0.042744	0.024063	0.005842	0.00622	0.028147	-1.577569602	0.040862	down
Serpina1d	1.50622	2.04372	2.07492	0.674595	0.765081	0.455024	-1.603658293	1.25E-08	down
Nova2	0.179703	0.098046	0.199706	0.02985	0.060606	0.065276	-1.63641504	0.002383	down
Hspa1l	1.57347	0.884656	0.760276	0.177374	0.565293	0.385918	-1.63660368	0.021719	down
Ankrd1	1.52545	0.648576	2.10436	0.343548	0.584018	0.473901	-1.646691304	0.024645	down
Gpr50	0.195932	0.132061	0.325857	0.084675	0.041963	0.106962	-1.697675132	0.009976	down
Ppp1r27	14.1623	8.63478	5.50105	2.15557	4.30292	2.53172	-1.701237849	0.028567	down
Prokr2	0.044698	0.037722	0.055879	0.007032	0.007598	0.01743	-1.720288552	0.048105	down
Hspb6	32.0576	17.4447	14.5894	2.46392	10.1974	6.93911	-1.73759791	0.016199	down
En1	0.278468	0.232538	0.104885	0	0.134983	0.054625	-1.742879776	0.026528	down
2310030G	0.667445	0.48784	0.234798	0.179216	0.171697	0.117161	-1.759444236	0.006037	down
Smtnl1	3.18194	2.04733	1.5049	0.20291	1.22463	0.736042	-1.76664264	0.009995	down
Sbk2	2.16528	1.19436	0.957991	0.288281	0.643028	0.29015	-1.815673481	0.013861	down
Cryab	45.9816	25.077	29.4554	5.34951	13.3565	8.72826	-1.967558889	0.001306	down
Flnc	22.4026	8.75384	15.4002	2.27819	7.31539	3.40743	-1.970253518	0.011209	down
Esrrg	0.609916	0.334204	0.252747	0.055974	0.17345	0.081948	-1.986449699	0.009972	down
Lgi2	0.137524	0.102576	0.214901	0.031901	0.046125	0.031522	-2.010614395	0.000401	down

Ntf5	0.146311	0.084282	0.021164	0.040287	0.032133	0	-2.045645541	0.039535	down
Adamts8	0.722201	0.477249	0.268095	0.088179	0.161647	0.137009	-2.046183498	0.010411	down
Rp1	0.025724	0.013741	0.01526	0.007239	0	0.005405	-2.096300326	0.027196	down
Myh3	0.289302	0.267544	0.181612	0.032531	0.114143	0.044923	-2.096777392	2.71E-06	down
Lmod2	8.49877	3.49026	3.23953	0.331623	2.50822	0.99995	-2.101342053	0.03114	down
Actn2	35.8176	19.4342	13.4463	1.64913	10.0341	5.4494	-2.127346822	0.01886	down
Gck	0.130772	0.039256	0.133711	0.015177	0.024083	0.036327	-2.132806641	0.013997	down
Dsc2	0.068561	0.087471	0.117271	0.021292	0.009242	0.016897	-2.142114894	0.001159	down
Casq2	2.96258	1.87078	1.32995	0.245391	0.857907	0.331573	-2.166391949	0.001366	down
Myh2	78.3626	46.9304	32.1533	4.09977	21.4361	12.2625	-2.196388352	0.007674	down
Fam179a	0.198639	0.067281	0.129882	0.041214	0	0.011353	-2.233039363	0.008229	down
Myom3	2.0422	1.0646	0.773654	0.086187	0.540487	0.265518	-2.262628573	0.01227	down
1700024P:	0.077773	0.030515	0.073196	0.009634	0.017119	0.012475	-2.285709575	0.008135	down
Csrp3	18.6679	9.92735	15.4761	1.00696	5.84543	1.86124	-2.369064688	5.14E-05	down
Hspb7	14.6865	7.58381	9.31524	0.856196	3.74712	1.42858	-2.442494472	0.000211	down
Itih5l-ps	0.11101	0.065804	0.119227	0.004461	0.033258	0.017566	-2.498433429	0.000238	down
Greb1	0.060384	0.07457	0.080386	0.020275	0.008113	0.007428	-2.507656429	7.27E-06	down
Xirp1	7.16889	1.95162	4.54264	0.42842	1.20175	0.851839	-2.56685724	0.01238	down
Sbk3	0.099288	0.059575	0.023795	0.005669	0.030247	0	-2.6090565	0.037372	down
Fabp3	41.0557	22.9143	13.8067	2.53996	6.73194	4.58436	-2.62644761	0.008821	down
Dsg2	0.027019	0.036377	0.01802	0.006816	0.003674	0.00337	-2.626970377	0.0347	down
Klhl34	1.76546	0.966862	0.719793	0.107589	0.336218	0.123772	-2.731086022	0.001294	down
Ankrd2	7.13993	3.99716	3.45175	0.196162	1.25923	0.575018	-2.776005687	0.00123	down
Myoz2	12.8502	6.41104	4.64727	0.490924	1.85021	1.31774	-2.801073818	0.00394	down
Pdk4	32.4612	5.62258	14.65	1.85946	2.92766	2.92262	-2.827278269	0.040807	down
1700113H	0.062899	0.030046	0.044558	0	0.015159	0	-2.897916539	0.038861	down
Tnnt1	23.3854	13.9914	8.22189	1.09495	2.70582	1.7384	-2.966055545	0.002946	down
Kcnt1	0.008731	0.008676	0.008689	0	0.005884	0	-3.016664121	0.016703	down
Plppr5	0.025477	0.089306	0.03942	0.004319	0.007078	0.006489	-3.106793248	0.00235	down
A2m	3.66749	2.64634	3.89072	0.445106	0.200782	0.389277	-3.33247683	6.70E-16	down
Thpo	0.058623	0.027997	0.028463	0	0.00711	0.006525	-3.407567645	0.004762	down
Msmp	0.899026	1.67767	1.74001	0	0.1298	0.078059	-3.552966283	1.79E-09	down
Myl2	67.5396	35.2983	24.2798	0.490486	5.38773	4.59327	-3.698494072	0.000556	down
Ces5a	0.0769	0.010834	0.032477	0	0	0	-3.756558336	0.024141	down
Tecrl	0.156048	0.059798	0.128871	0	0.009976	0.009171	-3.881640739	0.000297	down
Cdh12	0.008541	0.004379	0.003229	0.000997	0	0	-4.053705419	0.037037	down
Vmn1r90	0.18654	0.116418	0.113139	0	0	0	-4.155681826	0.021994	down
Tnnc1	49.5536	24.8087	15.1308	0.195604	2.18567	1.82466	-4.51720803	0.00044	down
Myl3	70.8511	34.2881	20.4897	1.41009	2.52266	1.50212	-4.601376335	0.000754	down
Tnni1	16.2748	8.67654	5.67675	0.068686	0.833592	0.393184	-4.621955902	0.000177	down
Capn11	0.011634	0.097074	0.338913	0	0.00581	0.01059	-4.645273021	0.021337	down
Myh7	47.293	20.9543	13.8488	0.058847	2.21969	1.05628	-4.773961037	0.001172	down
Sln	5.25508	4.83618	2.03941	0	0.144703	0.194969	-5.301414788	3.88E-07	down
Cbln2	0.024763	0.177286	0.095911	0.007532	0	0	-5.471788384	0.00107	down
Kcnj3	0.01334	0.009388	0.012354	0	0	0	-8.842287371	0.023654	down
BC048679	0.407533	0.323504	0.040025	0	0	0	-10.28815427	1.84E-05	down

Table S4 Correlation analysis of miRNA and circRNA

miRNA id	circRNA id	correlation	P value
mmu-miR-206-3p	circRNA_0020|Chr1:10315205_10321709_+	-0.902388823	0.013826896
mmu-miR-206-3p	circRNA_0157|Chr1:66801049_66802168_-	-0.818367091	0.046489689
mmu-miR-206-3p	circRNA_0913|Chr11:54005394_54014456_-	-0.839345213	0.036641694
mmu-miR-206-3p	circRNA_3155|Chr19:25061108_25065459_+	-0.876448359	0.021954504
mmu-miR-206-3p	circRNA_3239|Chr19:45635735_45640521_-	-0.859572081	0.028195381
mmu-miR-206-3p	circRNA_3702|Chr2:143830084_143832057_-	-0.943129588	0.004759399
mmu-miR-206-3p	circRNA_4055|Chr3:126796838_126798280_+	-0.944720168	0.004499326
mmu-miR-206-3p	circRNA_4662|Chr5:65301933_65303208_-	-0.877652755	0.021537574
mmu-miR-206-3p	circRNA_5311|Chr6:145147636_145149357_+	-0.956803725	0.002758577
mmu-miR-206-3p	circRNA_6140|Chr9:61935380_61937535_-	-0.975464845	0.000895576
mmu-miR-206-3p	circRNA_6446|ChrX:42217416_42250037_+	-0.866193271	0.025658507
mmu-miR-214-3p	circRNA_0537|Chr10:43393871_43395692_+	-0.960821676	0.002272343
mmu-miR-214-3p	circRNA_0845|Chr11:29470315_29477458_+	-0.908696603	0.012123899
mmu-miR-214-3p	circRNA_2313|Chr15:93452117_93465245_+	-0.833400493	0.039321076
mmu-miR-214-3p	circRNA_3832|Chr2:169883526_169886459_+	-0.982294129	0.000467471
mmu-miR-214-3p	circRNA_3954|Chr3:88346445_88349444_+	-0.884597129	0.019208276
mmu-miR-214-3p	circRNA_5074|Chr6:40685277_40747150_+	-0.841151263	0.035845273
mmu-miR-214-3p	circRNA_6390|Chr9:121727316_121731565_+	-0.856623097	0.029361709
mmu-miR-378a-5p	circRNA_3155|Chr19:25061108_25065459_+	-0.820486235	0.045445355
mmu-miR-378a-5p	circRNA_3239|Chr19:45635735_45640521_-	-0.841074816	0.035878818
mmu-miR-378a-5p	circRNA_3702|Chr2:143830084_143832057_-	-0.856202242	0.029529983
mmu-miR-378a-5p	circRNA_4055|Chr3:126796838_126798280_+	-0.946238118	0.004257815
mmu-miR-378a-5p	circRNA_5311|Chr6:145147636_145149357_+	-0.931607218	0.006856403
mmu-miR-378a-5p	circRNA_6140|Chr9:61935380_61937535_-	-0.885520358	0.018908221
mmu-miR-378a-5p	circRNA_6446|ChrX:42217416_42250037_+	-0.861043173	0.027621941
mmu-miR-494-3p	circRNA_0020|Chr1:10315205_10321709_+	-0.945690111	0.004344251
mmu-miR-494-3p	circRNA_0376|Chr1:172173943_172187460_+	-0.847076999	0.03329008
mmu-miR-494-3p	circRNA_2951|Chr18:39120119_39150118_+	-0.843931472	0.034635368
mmu-miR-494-3p	circRNA_3702|Chr2:143830084_143832057_-	-0.837794398	0.037332118
mmu-miR-494-3p	circRNA_4662|Chr5:65301933_65303208_-	-0.884840651	0.01912891
mmu-miR-494-3p	circRNA_4728|Chr5:97027831_97045167_+	-0.858271211	0.028707118
mmu-miR-494-3p	circRNA_5311|Chr6:145147636_145149357_+	-0.82773639	0.041956187
mmu-miR-494-3p	circRNA_6140|Chr9:61935380_61937535_-	-0.877583369	0.02156149
mmu-miR-135a-1-3p	circRNA_0020|Chr1:10315205_10321709_+	-0.841972257	0.035485956
mmu-miR-135a-1-3p	circRNA_0913|Chr11:54005394_54014456_-	-0.848894459	0.032524239
mmu-miR-135a-1-3p	circRNA_3702|Chr2:143830084_143832057_-	-0.837611784	0.037413816
mmu-miR-135a-1-3p	circRNA_4662|Chr5:65301933_65303208_-	-0.81363369	0.048862127
mmu-miR-135a-1-3p	circRNA_5311|Chr6:145147636_145149357_+	-0.871652239	0.023652576
mmu-miR-135a-1-3p	circRNA_6140|Chr9:61935380_61937535_-	-0.892635754	0.016671824
mmu-miR-1892	circRNA_0537|Chr10:43393871_43395692_+	-0.854185963	0.03034247
mmu-miR-1892	circRNA_0962|Chr11:67194348_67220998_+	-0.847424703	0.033142915
mmu-miR-1892	circRNA_3832|Chr2:169883526_169886459_+	-0.901039384	0.014205235
mmu-miR-5113	circRNA_0962|Chr11:67194348_67220998_+	-0.890804679	0.017234425
mmu-miR-5128	circRNA_0537|Chr10:43393871_43395692_+	-0.863840328	0.026547021
mmu-miR-5128	circRNA_0845|Chr11:29470315_29477458_+	-0.90241274	0.013820235
mmu-miR-5128	circRNA_1042|Chr11:80385854_80403408_+	-0.852213121	0.031147539
mmu-miR-5128	circRNA_1399|Chr12:72783683_72786789_+	-0.840214639	0.036257274
mmu-miR-5128	circRNA_2313|Chr15:93452117_93465245_+	-0.887515133	0.018267641
mmu-miR-5128	circRNA_3003|Chr18:67545615_67587859_-	-0.872078234	0.023499313
mmu-miR-5128	circRNA_3425|Chr2:37624173_37627526_-	-0.837676595	0.037384811
mmu-miR-5128	circRNA_3607|Chr2:119057281_119064097_+	-0.837676595	0.037384811
mmu-miR-5128	circRNA_3832|Chr2:169883526_169886459_+	-0.882681866	0.01983796
mmu-miR-5128	circRNA_3954|Chr3:88346445_88349444_+	-0.841792898	0.035564309
mmu-miR-5128	circRNA_5811|Chr8:71992832_71998295_-	-0.882909412	0.019762641
mmu-miR-344i	circRNA_0537|Chr10:43393871_43395692_+	-0.961954395	0.002143667

mmu-miR-344i	circRNA_0845|Chr11:29470315_29477458_+	-0.999232043	8.8441E-07
mmu-miR-344i	circRNA_1042|Chr11:80385854_80403408_+	-0.835698039	0.038275025
mmu-miR-344i	circRNA_1399|Chr12:72783683_72786789_+	-0.892713765	0.016648054
mmu-miR-344i	circRNA_2313|Chr15:93452117_93465245_+	-0.849867739	0.032117576
mmu-miR-344i	circRNA_3003|Chr18:67545615_67587859_-	-0.86020542	0.027947817
mmu-miR-344i	circRNA_3425|Chr2:37624173_37627526_-	-0.896319143	0.01556731
mmu-miR-344i	circRNA_3607|Chr2:119057281_119064097_+	-0.896319143	0.01556731
mmu-miR-344i	circRNA_3832|Chr2:169883526_169886459_+	-0.940306112	0.005238685
mmu-miR-344i	circRNA_3954|Chr3:88346445_88349444_+	-0.947748282	0.004024033
mmu-miR-344i	circRNA_4487|Chr4:149156607_149161694_-	-0.919605214	0.009435174
mmu-miR-344i	circRNA_5074|Chr6:40685277_40747150_+	-0.966203007	0.001694053
mmu-miR-344i	circRNA_5811|Chr8:71992832_71998295_-	-0.93743828	0.005748521
mmu-miR-344i	circRNA_5990|Chr9:3475483_3477962_+	-0.854447911	0.030236326
mmu-miR-344i	circRNA_6390|Chr9:121727316_121731565_+	-0.854838156	0.030078519
mmu-miR-6238	circRNA_0020|Chr1:10315205_10321709_+	-0.82481931	0.043344415
mmu-miR-6238	circRNA_0913|Chr11:54005394_54014456_-	-0.861054438	0.027617571
mmu-miR-6238	circRNA_3702|Chr2:143830084_143832057_-	-0.864710095	0.026216908
mmu-miR-6238	circRNA_5311|Chr6:145147636_145149357_+	-0.85719166	0.0291351
mmu-miR-6238	circRNA_6140|Chr9:61935380_61937535_-	-0.914197854	0.010727174
mmu-miR-6351	circRNA_0020|Chr1:10315205_10321709_+	-0.861752248	0.027347536
mmu-miR-6351	circRNA_0501|Chr10:25283893_25289730_-	-0.830876022	0.04048566
mmu-miR-6351	circRNA_2951|Chr18:39120119_39150118_+	-0.875190672	0.022393952
mmu-miR-6351	circRNA_4662|Chr5:65301933_65303208_-	-0.817355773	0.046991964
mmu-miR-6351	circRNA_4728|Chr5:97027831_97045167_+	-0.879430394	0.020929183
mmu-miR-6351	circRNA_6109|Chr9:57056714_57057861_+	-0.830876022	0.04048566
mmu-miR-6378	circRNA_0020|Chr1:10315205_10321709_+	-0.886829993	0.018486466
mmu-miR-6378	circRNA_2951|Chr18:39120119_39150118_+	-0.824720676	0.043391722
mmu-miR-6378	circRNA_3702|Chr2:143830084_143832057_-	-0.81817146	0.046586654
mmu-miR-6378	circRNA_4662|Chr5:65301933_65303208_-	-0.882551817	0.019881069
mmu-miR-6378	circRNA_4728|Chr5:97027831_97045167_+	-0.859234337	0.028327824
mmu-miR-6378	circRNA_6140|Chr9:61935380_61937535_-	-0.860421473	0.027863602
mmu-miR-3473e	circRNA_0537|Chr10:43393871_43395692_+	-0.941232279	0.005078986
mmu-miR-3473e	circRNA_0845|Chr11:29470315_29477458_+	-0.881142831	0.020350991
mmu-miR-3473e	circRNA_2313|Chr15:93452117_93465245_+	-0.898237936	0.015006377
mmu-miR-3473e	circRNA_3832|Chr2:169883526_169886459_+	-0.969389434	0.001391169
mmu-miR-3473e	circRNA_3954|Chr3:88346445_88349444_+	-0.881437845	0.020252164
mmu-miR-3473e	circRNA_5811|Chr8:71992832_71998295_-	-0.855366626	0.02986544
mmu-miR-7005-5p	circRNA_0020|Chr1:10315205_10321709_+	-0.894473971	0.016116059
mmu-miR-7005-5p	circRNA_4662|Chr5:65301933_65303208_-	-0.909984796	0.011789421
mmu-miR-7005-5p	circRNA_4728|Chr5:97027831_97045167_+	-0.820340966	0.045516592
mmu-miR-7005-5p	circRNA_6140|Chr9:61935380_61937535_-	-0.861010201	0.027634733
mmu-miR-7009-5p	circRNA_0020|Chr1:10315205_10321709_+	-0.923870448	0.008472951
mmu-miR-7009-5p	circRNA_0157|Chr1:66801049_66802168_-	-0.86238617	0.027103314
mmu-miR-7009-5p	circRNA_0501|Chr10:25283893_25289730_-	-0.84208325	0.03543751
mmu-miR-7009-5p	circRNA_2951|Chr18:39120119_39150118_+	-0.84908526	0.032444327
mmu-miR-7009-5p	circRNA_3702|Chr2:143830084_143832057_-	-0.872056946	0.023506961
mmu-miR-7009-5p	circRNA_4662|Chr5:65301933_65303208_-	-0.92528025	0.00816598
mmu-miR-7009-5p	circRNA_4728|Chr5:97027831_97045167_+	-0.910777463	0.011585857
mmu-miR-7009-5p	circRNA_6109|Chr9:57056714_57057861_+	-0.84208325	0.03543751
mmu-miR-7009-5p	circRNA_6140|Chr9:61935380_61937535_-	-0.911221445	0.011472588
mmu-miR-7042-5p	circRNA_0020|Chr1:10315205_10321709_+	-0.954899252	0.003005247
mmu-miR-7042-5p	circRNA_0157|Chr1:66801049_66802168_-	-0.844249106	0.034498382
mmu-miR-7042-5p	circRNA_0376|Chr1:172173943_172187460_+	-0.891147102	0.017128534
mmu-miR-7042-5p	circRNA_0501|Chr10:25283893_25289730_-	-0.850625955	0.031802446
mmu-miR-7042-5p	circRNA_2951|Chr18:39120119_39150118_+	-0.889697443	0.017578975
mmu-miR-7042-5p	circRNA_3702|Chr2:143830084_143832057_-	-0.811968713	0.049709652
mmu-miR-7042-5p	circRNA_4662|Chr5:65301933_65303208_-	-0.884014657	0.019398747
mmu-miR-7042-5p	circRNA_4728|Chr5:97027831_97045167_+	-0.916104097	0.010262532

mmu-miR-7042-5p	circRNA_6109|Chr9:57056714_57057861_+	-0.850625955	0.031802446
mmu-miR-7042-5p	circRNA_6140|Chr9:61935380_61937535_-	-0.842322782	0.035333064
mmu-miR-7219-5p	circRNA_0020|Chr1:10315205_10321709_+	-0.885474461	0.018923085
mmu-miR-7219-5p	circRNA_0157|Chr1:66801049_66802168_-	-0.829815175	0.040979791
mmu-miR-7219-5p	circRNA_0501|Chr10:25283893_25289730_-	-0.844871102	0.034230876
mmu-miR-7219-5p	circRNA_2951|Chr18:39120119_39150118_+	-0.878682019	0.021184297
mmu-miR-7219-5p	circRNA_3702|Chr2:143830084_143832057_-	-0.892274676	0.016782056
mmu-miR-7219-5p	circRNA_4662|Chr5:65301933_65303208_-	-0.857872993	0.02886464
mmu-miR-7219-5p	circRNA_4728|Chr5:97027831_97045167_+	-0.860461732	0.027847923
mmu-miR-7219-5p	circRNA_6109|Chr9:57056714_57057861_+	-0.844871102	0.034230876
mmu-miR-7219-5p	circRNA_6140|Chr9:61935380_61937535_-	-0.908713192	0.012119563
mmu-miR-7221-3p	circRNA_0537|Chr10:43393871_43395692_+	-0.966520357	0.001662566
mmu-miR-7221-3p	circRNA_0845|Chr11:29470315_29477458_+	-0.872081566	0.023498116
mmu-miR-7221-3p	circRNA_0961|Chr11:67192403_67254873_+	-0.862984434	0.026873783
mmu-miR-7221-3p	circRNA_2313|Chr15:93452117_93465245_+	-0.873244685	0.023082082
mmu-miR-7221-3p	circRNA_3832|Chr2:169883526_169886459_+	-0.952814153	0.003287227
mmu-miR-7221-3p	circRNA_3954|Chr3:88346445_88349444_+	-0.851038054	0.031631784
mmu-miR-7221-3p	circRNA_4926|Chr5:143080224_143081077_-	-0.971492131	0.001207464
mmu-miR-7221-3p	circRNA_5074|Chr6:40685277_40747150_+	-0.913851049	0.010812779
mmu-miR-7221-3p	circRNA_5811|Chr8:71992832_71998295_-	-0.874809859	0.022527832
mmu-miR-7221-3p	circRNA_5990|Chr9:3475483_3477962_+	-0.939922472	0.005305545
mmu-miR-7221-3p	circRNA_6390|Chr9:121727316_121731565_+	-0.926873818	0.007825639
mmu-miR-7226-5p	circRNA_0020|Chr1:10315205_10321709_+	-0.913220497	0.010969269
mmu-miR-7226-5p	circRNA_0376|Chr1:172173943_172187460_+	-0.844067065	0.034576859
mmu-miR-7226-5p	circRNA_3155|Chr19:25061108_25065459_+	-0.844415272	0.034426822
mmu-miR-7226-5p	circRNA_5311|Chr6:145147636_145149357_+	-0.827158019	0.042229755
mmu-miR-7226-5p	circRNA_6140|Chr9:61935380_61937535_-	-0.812601665	0.049386664
mmu-miR-3473f	circRNA_0845|Chr11:29470315_29477458_+	-0.900174458	0.014450321
mmu-miR-3473f	circRNA_1042|Chr11:80385854_80403408_+	-0.928994213	0.007383733
mmu-miR-3473f	circRNA_1399|Chr12:72783683_72786789_+	-0.922070356	0.00887291
mmu-miR-3473f	circRNA_3003|Chr18:67545615_67587859_-	-0.89265768	0.016665141
mmu-miR-3473f	circRNA_3425|Chr2:37624173_37627526_-	-0.91984905	0.00937881
mmu-miR-3473f	circRNA_3607|Chr2:119057281_119064097_+	-0.91984905	0.00937881
mmu-miR-3473f	circRNA_3954|Chr3:88346445_88349444_+	-0.91698051	0.010052259
mmu-miR-3473f	circRNA_4487|Chr4:149156607_149161694_-	-0.870751538	0.023978189
mmu-miR-3473f	circRNA_4635|Chr5:43758222_43773659_-	-0.816295559	0.047521216
mmu-miR-8094	circRNA_0913|Chr11:54005394_54014456_-	-0.864017529	0.026479607
mmu-miR-8094	circRNA_3155|Chr19:25061108_25065459_+	-0.817667244	0.046837003
mmu-miR-8094	circRNA_3239|Chr19:45635735_45640521_-	-0.827893008	0.04188225
mmu-miR-8094	circRNA_3702|Chr2:143830084_143832057_-	-0.890051861	0.017468331
mmu-miR-8094	circRNA_5311|Chr6:145147636_145149357_+	-0.925367691	0.008147122
mmu-miR-8094	circRNA_6140|Chr9:61935380_61937535_-	-0.920567846	0.009213613
mmu-miR-8117	circRNA_0020|Chr1:10315205_10321709_+	-0.926811949	0.007838721
mmu-miR-8117	circRNA_0376|Chr1:172173943_172187460_+	-0.836580346	0.037876834
mmu-miR-8117	circRNA_0501|Chr10:25283893_25289730_-	-0.82098678	0.045200295
mmu-miR-8117	circRNA_0611|Chr10:75274017_75279961_+	-0.824312637	0.043587687
mmu-miR-8117	circRNA_2951|Chr18:39120119_39150118_+	-0.877691827	0.021524112
mmu-miR-8117	circRNA_3155|Chr19:25061108_25065459_+	-0.866847024	0.02541419
mmu-miR-8117	circRNA_3702|Chr2:143830084_143832057_-	-0.870508744	0.024066324
mmu-miR-8117	circRNA_4662|Chr5:65301933_65303208_-	-0.833525807	0.039263681
mmu-miR-8117	circRNA_4728|Chr5:97027831_97045167_+	-0.834611891	0.038767891
mmu-miR-8117	circRNA_5311|Chr6:145147636_145149357_+	-0.843630339	0.034765472
mmu-miR-8117	circRNA_6109|Chr9:57056714_57057861_+	-0.82098678	0.045200295
mmu-miR-8117	circRNA_6140|Chr9:61935380_61937535_-	-0.886146914	0.018705876

Table S5 correlation analysis of miRNA and mRNA

miRNA id	mRNA id	correlation	P value
mmu-miR-206-3p	NM_024283.3	-0.897803291	0.015132571
mmu-miR-206-3p	NM_144544.2	-0.914158725	0.010736816
mmu-miR-206-3p	XM_006534171.3	-0.883727415	0.019493008
mmu-miR-206-3p	XM_011241948.2	-0.868523667	0.024792687
mmu-miR-206-3p	XM_006515980.3	-0.956243483	0.00283006
mmu-miR-206-3p	NM_001142804.1	-0.901996539	0.013936372
mmu-miR-206-3p	XM_006498086.3	-0.819499688	0.045930161
mmu-miR-206-3p	NM_021475.2	-0.933792647	0.006430013
mmu-miR-206-3p	XM_006500919.3	-0.858897552	0.028460183
mmu-miR-206-3p	NM_145635.2	-0.920324506	0.009269379
mmu-miR-206-3p	NM_009605.4	-0.963220423	0.002004229
mmu-miR-206-3p	NM_031185.3	-0.842613025	0.0352067
mmu-miR-206-3p	NM_008012.1	-0.983580853	0.000402169
mmu-miR-206-3p	NM_134072.1	-0.85672964	0.029319182
mmu-miR-206-3p	XM_006532024.3	-0.933401357	0.006505374
mmu-miR-206-3p	XM_006495613.3	-0.813410425	0.048975384
mmu-miR-206-3p	NM_001303431.1	-0.943554435	0.004689232
mmu-miR-206-3p	NM_009675.2	-0.831808011	0.040053864
mmu-miR-206-3p	NM_001110009.2	-0.94177229	0.00498699
mmu-miR-206-3p	NM_207231.1	-0.889173749	0.017743078
mmu-miR-206-3p	XM_006522398.3	-0.945993959	0.00429622
mmu-miR-206-3p	NM_029823.2	-0.882372326	0.01994064
mmu-miR-206-3p	NM_025711.3	-0.930627116	0.007051964
mmu-miR-206-3p	NM_001159407.1	-0.857415734	0.029046021
mmu-miR-206-3p	NM_001082546.1	-0.910484866	0.011660798
mmu-miR-206-3p	NM_007529.2	-0.830348643	0.040730957
mmu-miR-206-3p	XM_006503323.3	-0.826002204	0.042778938
mmu-miR-206-3p	NM_001002896.2	-0.973544223	0.001040604
mmu-miR-206-3p	NM_007549.2	-0.941932721	0.004959817
mmu-miR-206-3p	NM_007555.4	-0.93620448	0.005974983
mmu-miR-206-3p	NM_028472.2	-0.972102276	0.001156568
mmu-miR-206-3p	NM_178692.3	-0.894599926	0.016078309
mmu-miR-206-3p	XM_006503665.3	-0.910162065	0.011743747
mmu-miR-206-3p	NM_023143.3	-0.878250429	0.021332093
mmu-miR-206-3p	NM_001097617.1	-0.950356622	0.003635525
mmu-miR-206-3p	NM_027416.3	-0.970615886	0.001282454
mmu-miR-206-3p	XM_006520186.2	-0.862151743	0.027193507
mmu-miR-206-3p	NM_181315.4	-0.931051606	0.006966935
mmu-miR-206-3p	NM_019626.3	-0.989030591	0.000179832
mmu-miR-206-3p	NM_001310648.1	-0.82528435	0.043121691
mmu-miR-206-3p	NM_001001178.1	-0.903809929	0.013433794
mmu-miR-206-3p	NM_028804.1	-0.872110681	0.023487659
mmu-miR-206-3p	XM_006508867.2	-0.944556702	0.004525724
mmu-miR-206-3p	XM_006508885.1	-0.929700491	0.007239321
mmu-miR-206-3p	NM_025422.4	-0.823906141	0.043783319
mmu-miR-206-3p	NM_001110322.1	-0.913244375	0.010963323
mmu-miR-206-3p	NM_007655.3	-0.879368232	0.020950317
mmu-miR-206-3p	NM_001313939.1	-0.94654895	0.004209167
mmu-miR-206-3p	XM_017312731.1	-0.935299294	0.006143848
mmu-miR-206-3p	NM_013459.3	-0.922483591	0.008780301
mmu-miR-206-3p	NM_001301295.1	-0.914857384	0.010565287

mmu-miR-206-3p	XM_006513165.1	-0.8938454	0.016305081
mmu-miR-206-3p	NM_020001.2	-0.985029625	0.000334491
mmu-miR-206-3p	XM_006532120.2	-0.912760306	0.011084166
mmu-miR-206-3p	XM_017314944.1	-0.83963152	0.036514892
mmu-miR-206-3p	NM_009930.2	-0.964368505	0.001881786
mmu-miR-206-3p	XM_017315376.1	-0.920010562	0.009341567
mmu-miR-206-3p	NM_019696.2	-0.867884071	0.025028911
mmu-miR-206-3p	NM_007763.3	-0.854156336	0.030354487
mmu-miR-206-3p	NM_007792.4	-0.912174097	0.011231366
mmu-miR-206-3p	NM_018866.2	-0.89123288	0.017102057
mmu-miR-206-3p	XM_006517308.3	-0.904125596	0.013347218
mmu-miR-206-3p	NM_030206.4	-0.875756956	0.022195572
mmu-miR-206-3p	NM_007817.2	-0.912844635	0.011063068
mmu-miR-206-3p	NM_001190451.2	-0.903418174	0.013541614
mmu-miR-206-3p	NM_001190449.1	-0.918074151	0.00979283
mmu-miR-206-3p	NM_001190448.1	-0.903974436	0.013388642
mmu-miR-206-3p	NM_015814.2	-0.949838672	0.003711131
mmu-miR-206-3p	NM_152915.1	-0.932633264	0.006654551
mmu-miR-206-3p	NM_009345.2	-0.827047883	0.042281943
mmu-miR-206-3p	NM_019759.2	-0.986938765	0.00025478
mmu-miR-206-3p	NM_010090.2	-0.917201762	0.009999509
mmu-miR-206-3p	NM_133643.4	-0.864322321	0.026363842
mmu-miR-206-3p	NM_019397.3	-0.944707268	0.004501406
mmu-miR-206-3p	NM_001291145.1	-0.820833565	0.045275241
mmu-miR-206-3p	XM_006520618.2	-0.878423421	0.021272793
mmu-miR-206-3p	NM_015744.4	-0.953756335	0.003158269
mmu-miR-206-3p	NM_010140.3	-0.85576658	0.029704655
mmu-miR-206-3p	NM_024406.2	-0.959417574	0.002436982
mmu-miR-206-3p	XM_006509531.3	-0.854731959	0.030121424
mmu-miR-206-3p	NM_177743.5	-0.914476211	0.010658704
mmu-miR-206-3p	NM_178908.3	-0.922039277	0.008879894
mmu-miR-206-3p	XM_006520437.2	-0.847044057	0.033304039
mmu-miR-206-3p	XM_011244488.2	-0.869996994	0.024252596
mmu-miR-206-3p	NM_145141.2	-0.941634763	0.005010341
mmu-miR-206-3p	NM_145741.2	-0.848755649	0.032582434
mmu-miR-206-3p	NM_010279.3	-0.902504993	0.013794556
mmu-miR-206-3p	XM_006510297.3	-0.93749588	0.005738053
mmu-miR-206-3p	NM_001318003.2	-0.844629519	0.034334659
mmu-miR-206-3p	XM_011238891.1	-0.822090838	0.044661944
mmu-miR-206-3p	XM_001481172.6	-0.878862523	0.021122629
mmu-miR-206-3p	NM_001082547.1	-0.893686769	0.016352952
mmu-miR-206-3p	NM_008185.3	-0.909710632	0.011860228
mmu-miR-206-3p	XM_006496277.3	-0.923510981	0.008552103
mmu-miR-206-3p	NM_175520.4	-0.877145254	0.021712792
mmu-miR-206-3p	XM_006529759.1	-0.909552904	0.011901056
mmu-miR-206-3p	NM_178184.2	-0.835841955	0.038209941
mmu-miR-206-3p	NM_010450.3	-0.954961086	0.002997075
mmu-miR-206-3p	NM_008264.1	-0.838895709	0.036841191
mmu-miR-206-3p	NM_023670.3	-0.943250961	0.004739301
mmu-miR-206-3p	XM_006523711.3	-0.864784061	0.026188925
mmu-miR-206-3p	NM_001190325.1	-0.884045082	0.019388776
mmu-miR-206-3p	NM_001159424.2	-0.865207683	0.026028934
mmu-miR-206-3p	XM_017316439.1	-0.906459577	0.012715486
mmu-miR-206-3p	NM_001161541.1	-0.877093679	0.021730636

mmu-miR-206-3p	NM_021342.1	-0.837086079	0.037649474
mmu-miR-206-3p	NM_031180.2	-0.907828501	0.012351852
mmu-miR-206-3p	NM_173427.2	-0.887948641	0.01812983
mmu-miR-206-3p	XM_017320093.1	-0.888851098	0.017844546
mmu-miR-206-3p	NM_016659.3	-0.950469675	0.003619124
mmu-miR-206-3p	XM_017315313.1	-0.935033169	0.006193931
mmu-miR-206-3p	XM_006523061.3	-0.819363068	0.045997487
mmu-miR-206-3p	XM_006511102.2	-0.867410204	0.025204613
mmu-miR-206-3p	XM_006504999.1	-0.890123388	0.017446042
mmu-miR-206-3p	XM_006527221.3	-0.923775069	0.008493918
mmu-miR-206-3p	NM_010714.3	-0.938704882	0.005520491
mmu-miR-206-3p	NM_175271.4	-0.833008691	0.039500778
mmu-miR-206-3p	NM_028973.2	-0.931525956	0.006872515
mmu-miR-206-3p	XM_006527321.2	-0.943764846	0.00465467
mmu-miR-206-3p	NM_008524.2	-0.930426581	0.007092307
mmu-miR-206-3p	NM_010742.1	-0.908593607	0.012150837
mmu-miR-206-3p	NM_001290273.1	-0.991108993	0.000118224
mmu-miR-206-3p	XM_006520023.2	-0.933863242	0.006416463
mmu-miR-206-3p	NM_001291483.1	-0.959689362	0.00240467
mmu-miR-206-3p	NM_010809.2	-0.931904879	0.006797542
mmu-miR-206-3p	NM_029844.3	-0.95705211	0.002727173
mmu-miR-206-3p	NM_007641.5	-0.93843375	0.005568924
mmu-miR-206-3p	NM_010858.4	-0.962604765	0.002071459
mmu-miR-206-3p	XM_017317978.1	-0.948685212	0.00388225
mmu-miR-206-3p	XM_006510077.3	-0.95620427	0.002835097
mmu-miR-206-3p	NM_181547.3	-0.92617522	0.007973971
mmu-miR-206-3p	NM_023456.3	-0.891182323	0.01711766
mmu-miR-206-3p	NM_008760.4	-0.872010831	0.023523531
mmu-miR-206-3p	NM_172907.3	-0.94681952	0.004167044
mmu-miR-206-3p	XM_006516928.2	-0.935557434	0.006095456
mmu-miR-206-3p	XM_017322026.1	-0.957536761	0.002666407
mmu-miR-206-3p	NM_011044.2	-0.8995399	0.014631414
mmu-miR-206-3p	NM_001164593.1	-0.865267988	0.026006197
mmu-miR-206-3p	NM_011082.3	-0.826615965	0.042486898
mmu-miR-206-3p	NM_008872.3	-0.834817578	0.038674328
mmu-miR-206-3p	XM_017312497.1	-0.860206332	0.027947462
mmu-miR-206-3p	XM_011250776.2	-0.952805204	0.003288463
mmu-miR-206-3p	NM_001030305.2	-0.878715126	0.021172979
mmu-miR-206-3p	NM_011169.5	-0.969658408	0.001366952
mmu-miR-206-3p	NM_008966.3	-0.970262447	0.001313334
mmu-miR-206-3p	XM_006505757.3	-0.836596608	0.037869513
mmu-miR-206-3p	NM_016933.3	-0.924706237	0.0082903
mmu-miR-206-3p	NM_027455.3	-0.95976382	0.002395855
mmu-miR-206-3p	NM_009019.2	-0.858878129	0.028467826
mmu-miR-206-3p	XM_006506624.2	-0.97192431	0.001171301
mmu-miR-206-3p	NM_016809.6	-0.922150993	0.008854801
mmu-miR-206-3p	NM_011254.5	-0.877656555	0.021536264
mmu-miR-206-3p	NM_001204959.1	-0.899516896	0.014638
mmu-miR-206-3p	XM_011240779.2	-0.932528292	0.006675067
mmu-miR-206-3p	XM_006507942.2	-0.938734346	0.005515241
mmu-miR-206-3p	XM_006522506.3	-0.849501979	0.032270117
mmu-miR-206-3p	XM_006525075.3	-0.992049642	9.4561E-05
mmu-miR-206-3p	NM_009127.4	-0.934143249	0.006362853
mmu-miR-206-3p	NM_026535.2	-0.85672934	0.029319302

mmu-miR-206-3p	NM_008458.2	-0.95491394	0.003003305
mmu-miR-206-3p	XM_011244046.2	-0.942727348	0.004826303
mmu-miR-206-3p	NM_177578.4	-0.955935233	0.002869775
mmu-miR-206-3p	XM_006518339.3	-0.860811042	0.027712056
mmu-miR-206-3p	XM_006496884.3	-0.863030571	0.026856121
mmu-miR-206-3p	NM_144539.5	-0.949015469	0.003832868
mmu-miR-206-3p	XM_017313038.1	-0.896419017	0.015537869
mmu-miR-206-3p	NM_001310705.1	-0.963959141	0.001925008
mmu-miR-206-3p	NM_001290993.1	-0.903044813	0.013644758
mmu-miR-206-3p	XM_017322365.1	-0.881068583	0.020375899
mmu-miR-206-3p	NM_001146217.1	-0.987376866	0.00023801
mmu-miR-206-3p	XM_006524777.3	-0.815536509	0.04790182
mmu-miR-206-3p	NM_011430.3	-0.845868569	0.033803936
mmu-miR-206-3p	NM_009223.3	-0.964880338	0.001828428
mmu-miR-206-3p	NM_019866.1	-0.851664299	0.031373269
mmu-miR-206-3p	NM_178753.4	-0.868675074	0.024736924
mmu-miR-206-3p	NM_001204201.1	-0.890050135	0.017468869
mmu-miR-206-3p	XM_006527452.3	-0.893973612	0.016266439
mmu-miR-206-3p	XM_006521931.1	-0.939958298	0.005299284
mmu-miR-206-3p	NM_173869.3	-0.906474096	0.012711602
mmu-miR-206-3p	NM_025288.2	-0.91672874	0.010112448
mmu-miR-206-3p	NM_011581.3	-0.978118838	0.00071294
mmu-miR-206-3p	NM_009381.3	-0.942073627	0.004936012
mmu-miR-206-3p	XM_006501237.3	-0.923497199	0.008555145
mmu-miR-206-3p	XM_006533479.2	-0.91415474	0.010737799
mmu-miR-206-3p	XM_006533452.3	-0.901299824	0.014131832
mmu-miR-206-3p	NM_178715.3	-0.918636528	0.009660708
mmu-miR-206-3p	NM_144936.1	-0.831816364	0.040050004
mmu-miR-206-3p	XM_006521428.3	-0.847080377	0.033288649
mmu-miR-206-3p	XM_006496905.1	-0.91347151	0.010906842
mmu-miR-206-3p	NM_146241.2	-0.818049718	0.046647043
mmu-miR-206-3p	XM_011249157.2	-0.834892286	0.038640372
mmu-miR-206-3p	NM_001243916.1	-0.902108759	0.013905012
mmu-miR-206-3p	XM_017321602.1	-0.900731015	0.014292382
mmu-miR-206-3p	NM_023716.2	-0.840529496	0.036118528
mmu-miR-206-3p	NM_177709.3	-0.938095219	0.005629687
mmu-miR-206-3p	NM_011658.2	-0.97886855	0.000665089
mmu-miR-206-3p	XM_006525028.2	-0.9061359	0.012802211
mmu-miR-206-3p	NM_016982.2	-0.910319981	0.011703133
mmu-miR-206-3p	NM_009514.4	-0.908422854	0.01219556
mmu-miR-206-3p	NM_177789.4	-0.839414926	0.0366108
mmu-miR-206-3p	XM_017314347.1	-0.908783112	0.012101295
mmu-miR-214-3p	XM_017320219.1	-0.902558966	0.013779543
mmu-miR-214-3p	NM_029685.1	-0.912652288	0.011111219
mmu-miR-214-3p	XM_011241322.1	-0.979062342	0.000652989
mmu-miR-214-3p	NM_138955.3	-0.968721117	0.001452252
mmu-miR-214-3p	XM_006540566.1	-0.889907694	0.017513297
mmu-miR-214-3p	NM_033268.4	-0.831303132	0.040287509
mmu-miR-214-3p	XM_017312667.1	-0.838999976	0.03679487
mmu-miR-214-3p	NM_008032.3	-0.947428125	0.004073054
mmu-miR-214-3p	NM_153178.4	-0.945833511	0.00432155
mmu-miR-214-3p	XM_011250176.1	-0.974551367	0.000963209
mmu-miR-214-3p	XM_006498287.3	-0.822898324	0.044270108
mmu-miR-214-3p	XM_011240149.1	-0.817929595	0.046706665

mmu-miR-214-3p	XM_006508990.2	-0.970339356	0.001306584
mmu-miR-214-3p	NM_013468.3	-0.867516941	0.025164986
mmu-miR-214-3p	XM_017318264.1	-0.927718171	0.00764817
mmu-miR-214-3p	NM_172790.2	-0.870938955	0.023910261
mmu-miR-214-3p	NM_001080819.1	-0.817745037	0.046798337
mmu-miR-214-3p	NM_001310070.1	-0.824462065	0.043515875
mmu-miR-214-3p	NM_007881.4	-0.84726265	0.033211465
mmu-miR-214-3p	NM_001290469.1	-0.907125849	0.012537864
mmu-miR-214-3p	NM_013415.5	-0.88976468	0.017557958
mmu-miR-214-3p	NM_009722.3	-0.891843754	0.016914067
mmu-miR-214-3p	XM_006529727.2	-0.947621994	0.004043335
mmu-miR-214-3p	XM_006507725.2	-0.915952643	0.010299084
mmu-miR-214-3p	NM_013863.5	-0.892314499	0.016769881
mmu-miR-214-3p	XM_006502291.3	-0.86222719	0.027164464
mmu-miR-214-3p	XM_006510701.3	-0.870915748	0.023918667
mmu-miR-214-3p	NM_178782.4	-0.911910972	0.011297744
mmu-miR-214-3p	NM_020508.4	-0.887366622	0.018314968
mmu-miR-214-3p	XM_006524328.3	-0.955166417	0.002970016
mmu-miR-214-3p	NM_001285867.1	-0.945216693	0.004419608
mmu-miR-214-3p	NM_007588.2	-0.856558464	0.029387523
mmu-miR-214-3p	NM_001190379.1	-0.885857524	0.018799204
mmu-miR-214-3p	XM_006520253.3	-0.961547882	0.002189421
mmu-miR-214-3p	XM_006500947.3	-0.86322212	0.02678285
mmu-miR-214-3p	NM_009824.2	-0.925757681	0.008063274
mmu-miR-214-3p	NM_001013784.1	-0.879511753	0.020901538
mmu-miR-214-3p	XM_006509966.3	-0.921387507	0.009026977
mmu-miR-214-3p	XM_006523030.3	-0.939101125	0.005450082
mmu-miR-214-3p	XM_006530131.1	-0.978513222	0.000687562
mmu-miR-214-3p	XM_011250217.1	-0.93563029	0.006081833
mmu-miR-214-3p	XM_006527480.3	-0.947733355	0.004026312
mmu-miR-214-3p	NM_001113515.2	-0.829121603	0.041304364
mmu-miR-214-3p	XM_011247208.1	-0.853891706	0.030461917
mmu-miR-214-3p	XM_011248602.2	-0.949423667	0.003772262
mmu-miR-214-3p	NM_011957.2	-0.973854259	0.001016463
mmu-miR-214-3p	XM_011242311.2	-0.891329349	0.017072303
mmu-miR-214-3p	NM_001289782.1	-0.935743194	0.00606075
mmu-miR-214-3p	NM_001198841.1	-0.923698251	0.008510823
mmu-miR-214-3p	NM_010055.3	-0.822153633	0.044631415
mmu-miR-214-3p	NM_027293.1	-0.928782976	0.007427195
mmu-miR-214-3p	XM_011243391.2	-0.909481125	0.011919659
mmu-miR-214-3p	XM_006506808.3	-0.812983716	0.04919218
mmu-miR-214-3p	NM_001317365.1	-0.938111349	0.005626785
mmu-miR-214-3p	NM_007883.3	-0.812668759	0.049352484
mmu-miR-214-3p	NM_001160049.1	-0.849487217	0.032276281
mmu-miR-214-3p	XM_011245824.2	-0.857732971	0.028920126
mmu-miR-214-3p	XM_006528026.2	-0.865815961	0.025800019
mmu-miR-214-3p	NM_178676.4	-0.899378508	0.014677647
mmu-miR-214-3p	NM_010137.3	-0.823746862	0.043860086
mmu-miR-214-3p	XM_006511221.3	-0.879919079	0.020763392
mmu-miR-214-3p	NM_010174.1	-0.88763166	0.018230548
mmu-miR-214-3p	XM_006524407.3	-0.832390291	0.039785191
mmu-miR-214-3p	XM_006532962.2	-0.90339927	0.013546827
mmu-miR-214-3p	NM_001160262.1	-0.816297218	0.047520386
mmu-miR-214-3p	NM_010181.2	-0.959833336	0.00238764

mmu-miR-214-3p	XM_006527803.1	-0.885010921	0.019073511
mmu-miR-214-3p	NM_001081185.1	-0.892998224	0.016561518
mmu-miR-214-3p	NM_001033445.2	-0.984737018	0.00034766
mmu-miR-214-3p	XM_006514444.1	-0.853067816	0.030797536
mmu-miR-214-3p	XM_006530353.3	-0.963596178	0.001963735
mmu-miR-214-3p	XM_011248576.2	-0.978492508	0.000688884
mmu-miR-214-3p	NM_001198955.1	-0.928266574	0.007533968
mmu-miR-214-3p	XM_006532232.3	-0.918059706	0.009796235
mmu-miR-214-3p	XM_011250978.2	-0.955467978	0.002930496
mmu-miR-214-3p	NM_010340.2	-0.83760852	0.037415277
mmu-miR-214-3p	XM_006515096.2	-0.943434415	0.004709003
mmu-miR-214-3p	NM_001162950.1	-0.860550419	0.027813397
mmu-miR-214-3p	XM_006505602.3	-0.911106499	0.011501861
mmu-miR-214-3p	NM_001164086.1	-0.816399936	0.047468989
mmu-miR-214-3p	NM_013558.2	-0.828268494	0.041705237
mmu-miR-214-3p	NM_013560.2	-0.89554479	0.015796487
mmu-miR-214-3p	XM_006539957.3	-0.843570494	0.034791355
mmu-miR-214-3p	NM_013868.4	-0.929782792	0.007222583
mmu-miR-214-3p	NM_030704.3	-0.821371795	0.045012216
mmu-miR-214-3p	XM_006511311.2	-0.816393406	0.047472256
mmu-miR-214-3p	XM_006516224.3	-0.917607807	0.009903051
mmu-miR-214-3p	XM_006528842.1	-0.92015576	0.009308146
mmu-miR-214-3p	NM_001302471.1	-0.943649337	0.004673628
mmu-miR-214-3p	NM_001289875.1	-0.964105565	0.001909492
mmu-miR-214-3p	NM_008423.2	-0.859965409	0.028041513
mmu-miR-214-3p	XM_017315734.1	-0.983868657	0.000388231
mmu-miR-214-3p	XM_006497892.3	-0.878868721	0.021120513
mmu-miR-214-3p	XM_011248919.2	-0.900983927	0.014220888
mmu-miR-214-3p	NM_001081667.2	-0.863620642	0.026630712
mmu-miR-214-3p	XM_006512296.3	-0.8890784	0.017773034
mmu-miR-214-3p	NM_029274.2	-0.904001071	0.013381338
mmu-miR-214-3p	XM_017316676.1	-0.855342915	0.029874985
mmu-miR-214-3p	NM_008479.2	-0.854350655	0.030275714
mmu-miR-214-3p	XM_017313062.1	-0.960571887	0.002301217
mmu-miR-214-3p	NM_144945.3	-0.931503765	0.006876918
mmu-miR-214-3p	XM_006519969.3	-0.884720517	0.019168044
mmu-miR-214-3p	NM_144799.2	-0.906920516	0.012592475
mmu-miR-214-3p	NM_053098.2	-0.819840643	0.045762339
mmu-miR-214-3p	XM_006506340.3	-0.907748931	0.012372849
mmu-miR-214-3p	NM_028838.2	-0.826390465	0.042594087
mmu-miR-214-3p	XM_006541232.3	-0.852000888	0.031234739
mmu-miR-214-3p	XM_006498872.3	-0.913697091	0.010850888
mmu-miR-214-3p	XM_011243266.1	-0.819002651	0.04617532
mmu-miR-214-3p	NM_010825.3	-0.93041435	0.007094772
mmu-miR-214-3p	NM_001270475.1	-0.951312521	0.003498
mmu-miR-214-3p	NM_001290512.1	-0.933051168	0.006573182
mmu-miR-214-3p	NM_001099314.1	-0.948629303	0.00389064
mmu-miR-214-3p	XM_006529753.2	-0.96172162	0.002169808
mmu-miR-214-3p	NM_175260.2	-0.986378307	0.000277062
mmu-miR-214-3p	NM_001039545.2	-0.851199127	0.031565198
mmu-miR-214-3p	XM_006532412.1	-0.882121311	0.020024093
mmu-miR-214-3p	XM_017315841.1	-0.936823513	0.005860826
mmu-miR-214-3p	XM_006530186.3	-0.923194517	0.008622082
mmu-miR-214-3p	NM_010859.2	-0.936469309	0.005926013

mmu-miR-214-3p	XM_017320223.1	-0.82376768	0.043850049
mmu-miR-214-3p	XM_006501829.3	-0.900632702	0.014320221
mmu-miR-214-3p	NM_173437.2	-0.869358047	0.024486126
mmu-miR-214-3p	XM_006541297.3	-0.854705602	0.030132077
mmu-miR-214-3p	NM_001242558.1	-0.822282706	0.044568693
mmu-miR-214-3p	NM_011424.3	-0.84038452	0.036182382
mmu-miR-214-3p	NM_001134300.2	-0.980809316	0.00054889
mmu-miR-214-3p	NM_001109985.1	-0.906158919	0.012796034
mmu-miR-214-3p	NM_001029877.3	-0.930999565	0.006977332
mmu-miR-214-3p	XM_006526747.3	-0.820631594	0.045374125
mmu-miR-214-3p	NM_001077403.1	-0.984447085	0.000360959
mmu-miR-214-3p	XM_006530325.3	-0.880417784	0.020594852
mmu-miR-214-3p	NM_145226.2	-0.848216078	0.032809112
mmu-miR-214-3p	NM_011854.2	-0.844038154	0.034589331
mmu-miR-214-3p	NM_001286743.1	-0.867946372	0.025005855
mmu-miR-214-3p	NM_133167.3	-0.979712173	0.000613219
mmu-miR-214-3p	NM_013743.2	-0.879396637	0.020940658
mmu-miR-214-3p	XM_006509465.2	-0.846732751	0.033436085
mmu-miR-214-3p	XM_006538456.3	-0.926858854	0.007828802
mmu-miR-214-3p	XM_006537815.3	-0.982294953	0.000467428
mmu-miR-214-3p	NM_153104.3	-0.901542706	0.014063544
mmu-miR-214-3p	XM_017314870.1	-0.866803809	0.025430306
mmu-miR-214-3p	XM_006512076.3	-0.901694744	0.014020878
mmu-miR-214-3p	NM_148932.2	-0.90494	0.013125105
mmu-miR-214-3p	NM_011145.3	-0.93006561	0.00716521
mmu-miR-214-3p	NM_026814.3	-0.826401279	0.042588943
mmu-miR-214-3p	NM_001167908.1	-0.890827022	0.017227507
mmu-miR-214-3p	XM_006527008.2	-0.95236073	0.003350191
mmu-miR-214-3p	XM_011239558.2	-0.978246207	0.000704694
mmu-miR-214-3p	NM_175563.5	-0.927509821	0.007691778
mmu-miR-214-3p	NM_175022.2	-0.838758525	0.036902176
mmu-miR-214-3p	XM_006505015.3	-0.902574243	0.013775295
mmu-miR-214-3p	XM_006514153.2	-0.83982415	0.036429694
mmu-miR-214-3p	XM_006514077.1	-0.827469344	0.042082396
mmu-miR-214-3p	NM_001195662.1	-0.822934894	0.0442524
mmu-miR-214-3p	NM_019732.2	-0.921163724	0.009077748
mmu-miR-214-3p	XM_006540138.3	-0.837515318	0.037457007
mmu-miR-214-3p	NM_018732.3	-0.817680274	0.046830526
mmu-miR-214-3p	NM_020052.2	-0.852821102	0.030898375
mmu-miR-214-3p	NM_009243.4	-0.855335215	0.029878085
mmu-miR-214-3p	NM_009244.4	-0.863303029	0.026751929
mmu-miR-214-3p	NM_009246.3	-0.930734986	0.007030309
mmu-miR-214-3p	XM_017322211.1	-0.881132693	0.020354391
mmu-miR-214-3p	XM_006530238.3	-0.874488824	0.022640991
mmu-miR-214-3p	XM_017316715.1	-0.860190459	0.027953654
mmu-miR-214-3p	XM_017317034.1	-0.977886037	0.000728134
mmu-miR-214-3p	XM_011250732.2	-0.871026862	0.023878431
mmu-miR-214-3p	NM_001033167.3	-0.984283851	0.000368555
mmu-miR-214-3p	NM_001007570.2	-0.967868675	0.001532047
mmu-miR-214-3p	NM_001033286.2	-0.823091461	0.044176626
mmu-miR-214-3p	XM_006503080.3	-0.957811179	0.002632299
mmu-miR-214-3p	NM_025540.2	-0.907339532	0.012481154
mmu-miR-214-3p	XM_006500109.3	-0.826728573	0.042433418
mmu-miR-214-3p	XM_006534520.3	-0.841074333	0.035879029

mmu-miR-214-3p	XM_006539074.3	-0.881113256	0.020360911
mmu-miR-214-3p	NM_001304266.1	-0.838234576	0.037135536
mmu-miR-214-3p	NM_001242411.1	-0.85016548	0.031993654
mmu-miR-214-3p	XM_006506145.1	-0.926775258	0.007846484
mmu-miR-214-3p	XM_011248193.2	-0.871863825	0.023576393
mmu-miR-214-3p	XM_011245529.2	-0.932276371	0.006724428
mmu-miR-214-3p	NM_011374.2	-0.832519087	0.039725876
mmu-miR-214-3p	NM_009332.3	-0.968735668	0.001450908
mmu-miR-214-3p	NM_153801.3	-0.928993695	0.00738384
mmu-miR-214-3p	NM_009379.3	-0.858817305	0.028491764
mmu-miR-214-3p	NM_001161746.1	-0.889171071	0.017743918
mmu-miR-214-3p	XM_006538830.1	-0.984052568	0.000379453
mmu-miR-214-3p	NM_009393.2	-0.938466514	0.005563061
mmu-miR-214-3p	XM_006529382.3	-0.937776604	0.00568717
mmu-miR-214-3p	XM_006539726.3	-0.913551992	0.010886863
mmu-miR-214-3p	XM_011248201.2	-0.976668755	0.00081017
mmu-miR-214-3p	NM_177409.3	-0.923856396	0.008476038
mmu-miR-214-3p	NM_001039047.1	-0.943251629	0.004739191
mmu-miR-214-3p	XM_006508002.3	-0.82171225	0.044846209
mmu-miR-214-3p	XM_006506152.3	-0.828866178	0.041424198
mmu-miR-214-3p	NM_001033149.3	-0.906368474	0.012739867
mmu-miR-214-3p	XM_006538224.1	-0.896240629	0.015590473
mmu-miR-214-3p	XM_006501309.3	-0.816520887	0.047408503
mmu-miR-214-3p	XM_006525099.3	-0.953695083	0.003166576
mmu-miR-214-3p	NM_009504.4	-0.915037626	0.010521253
mmu-miR-214-3p	NM_001244031.1	-0.934381688	0.006317376
mmu-miR-214-3p	NM_011724.3	-0.904720176	0.013184881
mmu-miR-214-3p	XM_006530585.3	-0.906069612	0.012820007
mmu-miR-214-3p	NM_199304.1	-0.844521467	0.034381125
mmu-miR-214-3p	NM_199029.2	-0.88579228	0.018820276
mmu-miR-214-3p	XM_006511007.3	-0.939537953	0.005372974
mmu-miR-214-3p	NM_001110508.1	-0.828794143	0.041458023
mmu-miR-378a-5p	NM_144544.2	-0.868258085	0.024890646
mmu-miR-378a-5p	XM_006534171.3	-0.878575934	0.021220579
mmu-miR-378a-5p	NM_001271580.1	-0.813577725	0.048890505
mmu-miR-378a-5p	XM_006515980.3	-0.843394033	0.034867729
mmu-miR-378a-5p	NM_021475.2	-0.881932135	0.020087097
mmu-miR-378a-5p	NM_009605.4	-0.922310251	0.00881909
mmu-miR-378a-5p	NM_031185.3	-0.817334126	0.047002743
mmu-miR-378a-5p	NM_008012.1	-0.953083222	0.00325014
mmu-miR-378a-5p	XM_006532024.3	-0.969169442	0.001411132
mmu-miR-378a-5p	NM_001303431.1	-0.835776897	0.038239356
mmu-miR-378a-5p	NM_013912.3	-0.84030574	0.036217103
mmu-miR-378a-5p	NM_001110009.2	-0.832937653	0.039533401
mmu-miR-378a-5p	NM_207231.1	-0.914136228	0.010742362
mmu-miR-378a-5p	XM_006522398.3	-0.812170795	0.049606426
mmu-miR-378a-5p	NM_029823.2	-0.813973854	0.048689805
mmu-miR-378a-5p	NM_001082546.1	-0.978782743	0.000670482
mmu-miR-378a-5p	NM_001002896.2	-0.91956168	0.009445254
mmu-miR-378a-5p	NM_007549.2	-0.898539903	0.014919004
mmu-miR-378a-5p	NM_007555.4	-0.817641862	0.046849622
mmu-miR-378a-5p	NM_028472.2	-0.910815063	0.011576243
mmu-miR-378a-5p	XM_006503665.3	-0.815227824	0.048057004
mmu-miR-378a-5p	NM_023143.3	-0.865466006	0.025931601

mmu-miR-378a-5p	NM_001097617.1	-0.848973305	0.032491205
mmu-miR-378a-5p	NM_027416.3	-0.873641427	0.022940982
mmu-miR-378a-5p	XM_006520186.2	-0.826483323	0.042549932
mmu-miR-378a-5p	NM_181315.4	-0.899367779	0.014680723
mmu-miR-378a-5p	NM_019626.3	-0.927891259	0.007612035
mmu-miR-378a-5p	NM_001001178.1	-0.816131643	0.047603288
mmu-miR-378a-5p	XM_006508867.2	-0.872300964	0.023419369
mmu-miR-378a-5p	XM_006508885.1	-0.832685868	0.039649131
mmu-miR-378a-5p	NM_025422.4	-0.854856425	0.030071141
mmu-miR-378a-5p	NM_001110322.1	-0.842171567	0.035398983
mmu-miR-378a-5p	NM_007655.3	-0.854266237	0.030309924
mmu-miR-378a-5p	NM_001313939.1	-0.927321298	0.007731339
mmu-miR-378a-5p	NM_013459.3	-0.822111342	0.044651974
mmu-miR-378a-5p	NM_001301295.1	-0.829157802	0.041287395
mmu-miR-378a-5p	NM_020001.2	-0.930574197	0.007062599
mmu-miR-378a-5p	XM_006532120.2	-0.907086934	0.012548205
mmu-miR-378a-5p	NM_009930.2	-0.870125278	0.024205838
mmu-miR-378a-5p	XM_017315376.1	-0.868283205	0.024881373
mmu-miR-378a-5p	NM_019696.2	-0.849384286	0.032319274
mmu-miR-378a-5p	NM_007763.3	-0.881398685	0.020265269
mmu-miR-378a-5p	NM_007792.4	-0.850279003	0.031946464
mmu-miR-378a-5p	XM_006517308.3	-0.828496122	0.041598099
mmu-miR-378a-5p	NM_001190449.1	-0.847368422	0.033166715
mmu-miR-378a-5p	NM_015814.2	-0.82415197	0.043664962
mmu-miR-378a-5p	NM_152915.1	-0.859867286	0.028079861
mmu-miR-378a-5p	NM_019759.2	-0.936783624	0.005868149
mmu-miR-378a-5p	NM_010090.2	-0.918340628	0.009730117
mmu-miR-378a-5p	NM_133643.4	-0.833151193	0.039435374
mmu-miR-378a-5p	NM_019397.3	-0.877857301	0.021467145
mmu-miR-378a-5p	NM_015744.4	-0.845703957	0.03387422
mmu-miR-378a-5p	NM_024406.2	-0.877252714	0.021675634
mmu-miR-378a-5p	NM_178908.3	-0.856236822	0.029516139
mmu-miR-378a-5p	XM_011244488.2	-0.967204381	0.001595692
mmu-miR-378a-5p	NM_145141.2	-0.888759408	0.017873432
mmu-miR-378a-5p	NM_010279.3	-0.833859245	0.039111155
mmu-miR-378a-5p	XM_006504899.3	-0.833204372	0.03941098
mmu-miR-378a-5p	XM_001481172.6	-0.817549544	0.046895533
mmu-miR-378a-5p	NM_001082547.1	-0.96349704	0.00197438
mmu-miR-378a-5p	NM_008185.3	-0.88415434	0.019352988
mmu-miR-378a-5p	NM_175520.4	-0.835917759	0.038175681
mmu-miR-378a-5p	XM_006529759.1	-0.889093682	0.017768232
mmu-miR-378a-5p	NM_178184.2	-0.866726753	0.025459054
mmu-miR-378a-5p	NM_010450.3	-0.817610315	0.046865308
mmu-miR-378a-5p	NM_023670.3	-0.865463242	0.025932642
mmu-miR-378a-5p	NM_001190325.1	-0.868843065	0.024675122
mmu-miR-378a-5p	XM_017316439.1	-0.834863979	0.038653236
mmu-miR-378a-5p	NM_021342.1	-0.866861738	0.025408704
mmu-miR-378a-5p	XM_017320093.1	-0.848386792	0.032737314
mmu-miR-378a-5p	NM_016659.3	-0.894577774	0.016084945
mmu-miR-378a-5p	XM_017315313.1	-0.83964838	0.036507431
mmu-miR-378a-5p	XM_006527221.3	-0.815721389	0.047808987
mmu-miR-378a-5p	NM_010714.3	-0.855825202	0.029681123
mmu-miR-378a-5p	NM_028973.2	-0.816926178	0.047206083
mmu-miR-378a-5p	XM_006527321.2	-0.861335855	0.027508518

mmu-miR-378a-5p	NM_008524.2	-0.871290095	0.02378324
mmu-miR-378a-5p	NM_010742.1	-0.928738728	0.007436315
mmu-miR-378a-5p	NM_001290273.1	-0.955102457	0.002978432
mmu-miR-378a-5p	XM_006520023.2	-0.851823387	0.031307757
mmu-miR-378a-5p	NM_001291483.1	-0.892295518	0.016775683
mmu-miR-378a-5p	NM_010809.2	-0.86036822	0.027884348
mmu-miR-378a-5p	NM_029844.3	-0.8802377	0.020655637
mmu-miR-378a-5p	NM_007641.5	-0.891507161	0.017017526
mmu-miR-378a-5p	NM_010858.4	-0.925093175	0.008206396
mmu-miR-378a-5p	XM_017317978.1	-0.941157736	0.00509175
mmu-miR-378a-5p	XM_006510077.3	-0.856313446	0.029485475
mmu-miR-378a-5p	NM_181547.3	-0.96769825	0.001548253
mmu-miR-378a-5p	NM_172907.3	-0.868254107	0.024892115
mmu-miR-378a-5p	XM_006516928.2	-0.878274382	0.021323877
mmu-miR-378a-5p	XM_017322026.1	-0.892530721	0.016703853
mmu-miR-378a-5p	XM_011250776.2	-0.851522565	0.03143169
mmu-miR-378a-5p	NM_011169.5	-0.866351355	0.025599327
mmu-miR-378a-5p	NM_008966.3	-0.901791316	0.01399381
mmu-miR-378a-5p	NM_016933.3	-0.905885907	0.012869388
mmu-miR-378a-5p	NM_027455.3	-0.948259043	0.003946431
mmu-miR-378a-5p	NM_009019.2	-0.839863585	0.036412265
mmu-miR-378a-5p	XM_006506624.2	-0.916461377	0.010176557
mmu-miR-378a-5p	NM_016809.6	-0.90693342	0.01258904
mmu-miR-378a-5p	NM_011254.5	-0.898284379	0.014992923
mmu-miR-378a-5p	XM_011240779.2	-0.918391328	0.009718207
mmu-miR-378a-5p	XM_006507942.2	-0.948278307	0.003943519
mmu-miR-378a-5p	XM_006525075.3	-0.926376644	0.007931064
mmu-miR-378a-5p	NM_009127.4	-0.813721974	0.048817377
mmu-miR-378a-5p	NM_026535.2	-0.892918153	0.016585855
mmu-miR-378a-5p	NM_008458.2	-0.923499478	0.008554642
mmu-miR-378a-5p	NM_177578.4	-0.915334249	0.01044898
mmu-miR-378a-5p	XM_006496884.3	-0.843686086	0.03474137
mmu-miR-378a-5p	NM_144539.5	-0.925493139	0.008120105
mmu-miR-378a-5p	XM_017313038.1	-0.849235421	0.032381501
mmu-miR-378a-5p	NM_001310705.1	-0.834949362	0.038614439
mmu-miR-378a-5p	NM_001290993.1	-0.81694462	0.047196882
mmu-miR-378a-5p	NM_001146217.1	-0.896621245	0.015478338
mmu-miR-378a-5p	NM_009223.3	-0.900798431	0.014273308
mmu-miR-378a-5p	NM_001204201.1	-0.837905971	0.037282244
mmu-miR-378a-5p	XM_006527452.3	-0.847843686	0.032965989
mmu-miR-378a-5p	XM_006521931.1	-0.969659803	0.001366827
mmu-miR-378a-5p	NM_173869.3	-0.940180941	0.005260454
mmu-miR-378a-5p	NM_025288.2	-0.988688748	0.000191193
mmu-miR-378a-5p	NM_011581.3	-0.901879209	0.013969196
mmu-miR-378a-5p	NM_009381.3	-0.812792078	0.049289689
mmu-miR-378a-5p	XM_006501237.3	-0.91235433	0.011186009
mmu-miR-378a-5p	XM_006533479.2	-0.990355657	0.000139071
mmu-miR-378a-5p	XM_006533452.3	-0.816418245	0.047459831
mmu-miR-378a-5p	NM_178715.3	-0.910020399	0.011780241
mmu-miR-378a-5p	XM_006496905.1	-0.819839792	0.045762758
mmu-miR-378a-5p	NM_001243916.1	-0.927482544	0.007697495
mmu-miR-378a-5p	NM_023716.2	-0.85525273	0.029911302
mmu-miR-378a-5p	NM_177709.3	-0.879580366	0.020878237
mmu-miR-378a-5p	NM_011658.2	-0.875077662	0.022433642

mmu-miR-378a-5p	NM_016982.2	-0.832503714	0.039732954
mmu-miR-378a-5p	NM_009514.4	-0.892749519	0.016637165
mmu-miR-378a-5p	XM_017314347.1	-0.891109614	0.017140111
mmu-miR-494-3p	NM_024283.3	-0.935011371	0.006198042
mmu-miR-494-3p	XM_006515980.3	-0.856611138	0.029366485
mmu-miR-494-3p	NM_153151.3	-0.864615312	0.026252787
mmu-miR-494-3p	NM_001142804.1	-0.811508456	0.049945129
mmu-miR-494-3p	XM_006500919.3	-0.881843819	0.020116542
mmu-miR-494-3p	NM_145635.2	-0.862713902	0.026977461
mmu-miR-494-3p	NM_009605.4	-0.819017876	0.046167802
mmu-miR-494-3p	NM_134072.1	-0.89714964	0.015323309
mmu-miR-494-3p	NM_001303431.1	-0.875201212	0.022390252
mmu-miR-494-3p	NM_001110009.2	-0.87697062	0.021773242
mmu-miR-494-3p	XM_006522398.3	-0.946065795	0.004284903
mmu-miR-494-3p	NM_025711.3	-0.939116393	0.005447378
mmu-miR-494-3p	NM_020025.4	-0.884685112	0.019179584
mmu-miR-494-3p	NM_007529.2	-0.906718693	0.012646264
mmu-miR-494-3p	NM_001002896.2	-0.82424224	0.043621537
mmu-miR-494-3p	NM_007555.4	-0.88002772	0.020726621
mmu-miR-494-3p	NM_178692.3	-0.837221639	0.03758864
mmu-miR-494-3p	NM_001097617.1	-0.895679871	0.015756392
mmu-miR-494-3p	NM_027416.3	-0.896649205	0.015470115
mmu-miR-494-3p	NM_019626.3	-0.861168709	0.027573265
mmu-miR-494-3p	NM_001001178.1	-0.876673617	0.021876235
mmu-miR-494-3p	XM_006508867.2	-0.834664493	0.038743953
mmu-miR-494-3p	XM_006508885.1	-0.871928722	0.02355305
mmu-miR-494-3p	XM_017312731.1	-0.941558609	0.005023294
mmu-miR-494-3p	NM_020001.2	-0.834442533	0.038845007
mmu-miR-494-3p	XM_017314944.1	-0.93201698	0.006775438
mmu-miR-494-3p	NM_009930.2	-0.91668397	0.01012317
mmu-miR-494-3p	NM_001309809.2	-0.95131146	0.003498151
mmu-miR-494-3p	NM_007792.4	-0.840170521	0.036276734
mmu-miR-494-3p	NM_018866.2	-0.910216409	0.011729763
mmu-miR-494-3p	XM_006517308.3	-0.823915444	0.043778838
mmu-miR-494-3p	NM_007817.2	-0.817270759	0.047034301
mmu-miR-494-3p	XM_006540596.2	-0.836750623	0.037800211
mmu-miR-494-3p	NM_001190451.2	-0.9248578	0.008257386
mmu-miR-494-3p	NM_001190449.1	-0.84751991	0.033102672
mmu-miR-494-3p	NM_001190448.1	-0.811575598	0.049910746
mmu-miR-494-3p	NM_015814.2	-0.904136315	0.013344283
mmu-miR-494-3p	NM_001291145.1	-0.873502764	0.02299025
mmu-miR-494-3p	NM_015744.4	-0.842947469	0.035061357
mmu-miR-494-3p	NM_010140.3	-0.886770619	0.018505488
mmu-miR-494-3p	NM_024406.2	-0.850276775	0.03194739
mmu-miR-494-3p	NM_177743.5	-0.969205626	0.001407839
mmu-miR-494-3p	XM_006520437.2	-0.933320841	0.006520934
mmu-miR-494-3p	NM_145741.2	-0.834874219	0.038648582
mmu-miR-494-3p	XM_006510297.3	-0.869898054	0.024288688
mmu-miR-494-3p	NM_001318003.2	-0.848563886	0.03266291
mmu-miR-494-3p	XM_001481172.6	-0.853917604	0.030451396
mmu-miR-494-3p	XM_006496277.3	-0.880611229	0.020529653
mmu-miR-494-3p	XM_011246309.2	-0.814142223	0.048604617
mmu-miR-494-3p	NM_010450.3	-0.915028066	0.010523586
mmu-miR-494-3p	NM_008264.1	-0.851371418	0.031494046

mmu-miR-494-3p	NM_023670.3	-0.854736243	0.030119693
mmu-miR-494-3p	XM_006523711.3	-0.880517232	0.020561322
mmu-miR-494-3p	NM_001161541.1	-0.966926288	0.001622717
mmu-miR-494-3p	NM_010608.2	-0.81361951	0.048869317
mmu-miR-494-3p	NM_031180.2	-0.908937837	0.012060918
mmu-miR-494-3p	NM_173427.2	-0.912101326	0.011249705
mmu-miR-494-3p	NM_008940.3	-0.864292734	0.026375069
mmu-miR-494-3p	NM_016659.3	-0.843628082	0.034766448
mmu-miR-494-3p	NM_146063.1	-0.915505858	0.010407277
mmu-miR-494-3p	XM_017315313.1	-0.833585088	0.039236544
mmu-miR-494-3p	XM_006523061.3	-0.904696582	0.013191304
mmu-miR-494-3p	XM_006511102.2	-0.904138557	0.013343669
mmu-miR-494-3p	XM_006504999.1	-0.896250933	0.015587433
mmu-miR-494-3p	XM_006527221.3	-0.822197105	0.044610286
mmu-miR-494-3p	NM_010714.3	-0.859206268	0.028338844
mmu-miR-494-3p	NM_028973.2	-0.951338089	0.003494357
mmu-miR-494-3p	NM_008524.2	-0.87039944	0.024106053
mmu-miR-494-3p	XM_006520023.2	-0.886249016	0.018673001
mmu-miR-494-3p	NM_001252563.1	-0.923012663	0.008662421
mmu-miR-494-3p	NM_010779.2	-0.879561825	0.020884532
mmu-miR-494-3p	NM_001291483.1	-0.849318564	0.03234674
mmu-miR-494-3p	NM_029844.3	-0.845203487	0.034088327
mmu-miR-494-3p	XM_006498901.3	-0.892227374	0.016796522
mmu-miR-494-3p	XM_006510077.3	-0.908364366	0.012210898
mmu-miR-494-3p	NM_023456.3	-0.837458746	0.037482347
mmu-miR-494-3p	NM_008760.4	-0.953713539	0.003164072
mmu-miR-494-3p	NM_172907.3	-0.890706395	0.017264877
mmu-miR-494-3p	XM_006516928.2	-0.859025381	0.028409911
mmu-miR-494-3p	XM_017322026.1	-0.836618385	0.037859711
mmu-miR-494-3p	NM_001164593.1	-0.94865579	0.003886664
mmu-miR-494-3p	NM_011082.3	-0.846607362	0.033489341
mmu-miR-494-3p	XM_017312497.1	-0.961329392	0.00221421
mmu-miR-494-3p	XM_011250776.2	-0.833935812	0.03907617
mmu-miR-494-3p	NM_011169.5	-0.852591218	0.030992475
mmu-miR-494-3p	NM_001081224.2	-0.92349368	0.008555922
mmu-miR-494-3p	NM_008966.3	-0.890502214	0.017328221
mmu-miR-494-3p	XM_006505757.3	-0.886289453	0.018659989
mmu-miR-494-3p	NM_198024.2	-0.817012731	0.047162907
mmu-miR-494-3p	XM_006525075.3	-0.855084522	0.029979095
mmu-miR-494-3p	NM_009127.4	-0.851245999	0.031545833
mmu-miR-494-3p	XM_011244046.2	-0.910681999	0.011610281
mmu-miR-494-3p	XM_006521796.1	-0.925734418	0.008068264
mmu-miR-494-3p	NM_177578.4	-0.847343022	0.033177459
mmu-miR-494-3p	NM_001310705.1	-0.93618345	0.00597888
mmu-miR-494-3p	NM_001146217.1	-0.869070294	0.024591645
mmu-miR-494-3p	XM_006524777.3	-0.934966375	0.006206533
mmu-miR-494-3p	NM_011430.3	-0.92576603	0.008061484
mmu-miR-494-3p	NM_175692.3	-0.900379304	0.014392093
mmu-miR-494-3p	NM_009223.3	-0.834177151	0.038965993
mmu-miR-494-3p	NM_178753.4	-0.82737597	0.042126567
mmu-miR-494-3p	NM_001204201.1	-0.840521967	0.036121843
mmu-miR-494-3p	NM_032400.2	-0.894876968	0.015995428
mmu-miR-494-3p	NM_011581.3	-0.879414088	0.020934726
mmu-miR-494-3p	NM_009381.3	-0.898893573	0.014816984

mmu-miR-494-3p	NM_144936.1	-0.912943507	0.011038356
mmu-miR-494-3p	NM_177371.3	-0.85115896	0.031581796
mmu-miR-494-3p	XM_006496905.1	-0.917842009	0.009847623
mmu-miR-494-3p	XM_017321602.1	-0.892032135	0.016856296
mmu-miR-494-3p	NM_011658.2	-0.911877396	0.011306228
mmu-miR-494-3p	XM_011248394.1	-0.828038687	0.041813532
mmu-miR-494-3p	XM_006525028.2	-0.886410182	0.018621166
mmu-miR-494-3p	NM_016982.2	-0.847515915	0.03310436
mmu-miR-494-3p	NM_177789.4	-0.822404546	0.044509525
mmu-miR-494-3p	XM_017318541.1	-0.899192421	0.01473104
mmu-miR-494-3p	XM_011243586.2	-0.980705249	0.00055484
mmu-miR-135a-1-3p	NM_024283.3	-0.844680246	0.034312855
mmu-miR-135a-1-3p	XM_006500919.3	-0.904750748	0.013176559
mmu-miR-135a-1-3p	XM_006495613.3	-0.858070795	0.028786345
mmu-miR-135a-1-3p	NM_001303431.1	-0.909491894	0.011916867
mmu-miR-135a-1-3p	XM_006522398.3	-0.85857108	0.028588769
mmu-miR-135a-1-3p	NM_025711.3	-0.906988628	0.012574347
mmu-miR-135a-1-3p	NM_001097617.1	-0.816916305	0.047211009
mmu-miR-135a-1-3p	NM_027416.3	-0.816699602	0.047319196
mmu-miR-135a-1-3p	NM_019626.3	-0.851407524	0.031479145
mmu-miR-135a-1-3p	NM_001001178.1	-0.872657689	0.023291601
mmu-miR-135a-1-3p	XM_017312731.1	-0.839190497	0.036710302
mmu-miR-135a-1-3p	NM_009930.2	-0.870466138	0.024081806
mmu-miR-135a-1-3p	NM_001309809.2	-0.861584927	0.02741217
mmu-miR-135a-1-3p	NM_018866.2	-0.956008183	0.002860352
mmu-miR-135a-1-3p	NM_001190451.2	-0.868571669	0.024775002
mmu-miR-135a-1-3p	NM_015814.2	-0.84261516	0.035205771
mmu-miR-135a-1-3p	NM_177743.5	-0.907743633	0.012374248
mmu-miR-135a-1-3p	XM_006520437.2	-0.821228845	0.045082004
mmu-miR-135a-1-3p	XM_001481172.6	-0.824490536	0.043502199
mmu-miR-135a-1-3p	NM_010450.3	-0.871348229	0.023762242
mmu-miR-135a-1-3p	NM_008264.1	-0.883865201	0.019447765
mmu-miR-135a-1-3p	XM_006523711.3	-0.857606233	0.028970391
mmu-miR-135a-1-3p	NM_001161541.1	-0.906874645	0.012604691
mmu-miR-135a-1-3p	XM_017315313.1	-0.81326109	0.049051205
mmu-miR-135a-1-3p	XM_006523061.3	-0.956452193	0.002803325
mmu-miR-135a-1-3p	NM_010714.3	-0.865062161	0.026083842
mmu-miR-135a-1-3p	NM_028973.2	-0.935941487	0.006023808
mmu-miR-135a-1-3p	NM_008524.2	-0.851884108	0.031282769
mmu-miR-135a-1-3p	XM_006520023.2	-0.812009721	0.049688697
mmu-miR-135a-1-3p	NM_001252563.1	-0.863927383	0.026513892
mmu-miR-135a-1-3p	NM_010779.2	-0.872338786	0.023405806
mmu-miR-135a-1-3p	XM_006510077.3	-0.827363903	0.042132277
mmu-miR-135a-1-3p	NM_008760.4	-0.908904355	0.01206965
mmu-miR-135a-1-3p	NM_172907.3	-0.871373384	0.023753158
mmu-miR-135a-1-3p	XM_006516928.2	-0.861928247	0.027279627
mmu-miR-135a-1-3p	XM_011239969.2	-0.846989482	0.03332717
mmu-miR-135a-1-3p	NM_001164593.1	-0.899363053	0.014682078
mmu-miR-135a-1-3p	XM_017312497.1	-0.976731573	0.000805831
mmu-miR-135a-1-3p	NM_008966.3	-0.861080013	0.027607652
mmu-miR-135a-1-3p	XM_006505757.3	-0.840579766	0.036096399
mmu-miR-135a-1-3p	NM_027455.3	-0.837012254	0.037682623
mmu-miR-135a-1-3p	NM_198024.2	-0.881438456	0.020251959
mmu-miR-135a-1-3p	XM_006525075.3	-0.830420952	0.040697283

mmu-miR-135a-1-3p	NM_023893.4	-0.868168805	0.024923618
mmu-miR-135a-1-3p	XM_011244046.2	-0.827475303	0.042079578
mmu-miR-135a-1-3p	NM_177578.4	-0.8241113	0.043684532
mmu-miR-135a-1-3p	NM_001310705.1	-0.867736803	0.025083453
mmu-miR-135a-1-3p	NM_001146217.1	-0.821726182	0.044839422
mmu-miR-135a-1-3p	XM_006524777.3	-0.829228703	0.041254166
mmu-miR-135a-1-3p	NM_011430.3	-0.843107765	0.034991795
mmu-miR-135a-1-3p	NM_001204201.1	-0.825300347	0.043114039
mmu-miR-135a-1-3p	NM_011581.3	-0.845197137	0.034091047
mmu-miR-135a-1-3p	XM_006537836.1	-0.853882266	0.030465753
mmu-miR-135a-1-3p	NM_178715.3	-0.846362019	0.03359366
mmu-miR-135a-1-3p	NM_177371.3	-0.890675341	0.017274504
mmu-miR-135a-1-3p	XM_006496905.1	-0.915089768	0.010508531
mmu-miR-135a-1-3p	XM_017321602.1	-0.854547534	0.030196003
mmu-miR-135a-1-3p	NM_177709.3	-0.876533868	0.021924777
mmu-miR-135a-1-3p	NM_011658.2	-0.869820165	0.024317119
mmu-miR-135a-1-3p	XM_011243586.2	-0.885660794	0.018862777
mmu-miR-1892	NM_029685.1	-0.881459499	0.020244919
mmu-miR-1892	NM_001033630.1	-0.84547067	0.033973944
mmu-miR-1892	XM_011241322.1	-0.931600105	0.006857812
mmu-miR-1892	NM_138955.3	-0.880311504	0.020630715
mmu-miR-1892	XM_006540566.1	-0.914020729	0.010770854
mmu-miR-1892	NM_172580.1	-0.866565217	0.025519369
mmu-miR-1892	NM_009608.4	-0.856505275	0.029408773
mmu-miR-1892	NM_033268.4	-0.90375384	0.013449205
mmu-miR-1892	NM_013906.3	-0.87996091	0.02074923
mmu-miR-1892	XM_017312667.1	-0.84646671	0.033549127
mmu-miR-1892	NM_008032.3	-0.867815069	0.02505446
mmu-miR-1892	NM_153178.4	-0.918771339	0.009629166
mmu-miR-1892	XM_011250176.1	-0.951450443	0.003478372
mmu-miR-1892	XM_006508990.2	-0.896508117	0.015511626
mmu-miR-1892	XM_017318264.1	-0.959318875	0.002448768
mmu-miR-1892	NM_172790.2	-0.863149659	0.026810556
mmu-miR-1892	NM_023048.5	-0.876902268	0.021796924
mmu-miR-1892	NM_001290469.1	-0.867447642	0.025190711
mmu-miR-1892	NM_013415.5	-0.915899029	0.010312038
mmu-miR-1892	NM_009722.3	-0.935129662	0.006175749
mmu-miR-1892	XM_006529727.2	-0.88047849	0.020574381
mmu-miR-1892	XM_006507725.2	-0.874420534	0.022665097
mmu-miR-1892	XM_006540762.3	-0.813107379	0.049129306
mmu-miR-1892	NM_013863.5	-0.908722993	0.012117001
mmu-miR-1892	XM_006510701.3	-0.831623341	0.040139251
mmu-miR-1892	NM_178782.4	-0.862288805	0.027140756
mmu-miR-1892	NM_020508.4	-0.854759927	0.030110122
mmu-miR-1892	XM_006524328.3	-0.887742684	0.018195241
mmu-miR-1892	NM_001285867.1	-0.957607077	0.002657646
mmu-miR-1892	NM_009785.1	-0.814017912	0.048667507
mmu-miR-1892	NM_001190379.1	-0.878213555	0.021344743
mmu-miR-1892	XM_006520253.3	-0.974448494	0.000970978
mmu-miR-1892	XM_006500947.3	-0.918415825	0.009712455
mmu-miR-1892	NM_009824.2	-0.932758692	0.006630078
mmu-miR-1892	XM_006509966.3	-0.830784305	0.04052827
mmu-miR-1892	XM_011245344.2	-0.827210921	0.042204698
mmu-miR-1892	XM_006523030.3	-0.935284417	0.006146642

mmu-miR-1892	XM_006530131.1	-0.933227557	0.006538984
mmu-miR-1892	XM_011250217.1	-0.937931359	0.005659214
mmu-miR-1892	XM_006527480.3	-0.941087346	0.005103817
mmu-miR-1892	NM_001113515.2	-0.858498013	0.028617585
mmu-miR-1892	XM_006532938.2	-0.879544426	0.02089044
mmu-miR-1892	XM_011247208.1	-0.821028212	0.045180038
mmu-miR-1892	XM_011248602.2	-0.870301004	0.024141857
mmu-miR-1892	NM_011957.2	-0.965474169	0.001767472
mmu-miR-1892	XM_011242311.2	-0.883785083	0.019474067
mmu-miR-1892	NM_001289782.1	-0.945202879	0.004421816
mmu-miR-1892	NM_001198841.1	-0.90933786	0.011956831
mmu-miR-1892	NM_010055.3	-0.844386366	0.034439265
mmu-miR-1892	NM_027293.1	-0.933742922	0.006439566
mmu-miR-1892	XM_011243391.2	-0.885599202	0.018882701
mmu-miR-1892	NM_001317365.1	-0.860051898	0.028007732
mmu-miR-1892	NM_007883.3	-0.898105944	0.015044646
mmu-miR-1892	XM_006514873.2	-0.862438914	0.027083041
mmu-miR-1892	NM_001160049.1	-0.888184914	0.01805493
mmu-miR-1892	XM_011245824.2	-0.853381132	0.030669699
mmu-miR-1892	XM_006528026.2	-0.815938661	0.047699997
mmu-miR-1892	NM_010133.2	-0.842124288	0.035419605
mmu-miR-1892	NM_178676.4	-0.912938216	0.011039678
mmu-miR-1892	XM_011238916.2	-0.861853671	0.027308392
mmu-miR-1892	NM_010174.1	-0.951862772	0.003420017
mmu-miR-1892	XM_006532962.2	-0.82892375	0.041397174
mmu-miR-1892	NM_001160262.1	-0.904479345	0.013250519
mmu-miR-1892	NM_010181.2	-0.930224239	0.007133128
mmu-miR-1892	XM_006527803.1	-0.938789287	0.005505456
mmu-miR-1892	XM_006502769.3	-0.865994375	0.025733058
mmu-miR-1892	NM_001081185.1	-0.865570251	0.025892372
mmu-miR-1892	NM_172862.3	-0.838026903	0.037228221
mmu-miR-1892	NM_001033445.2	-0.964702798	0.00184685
mmu-miR-1892	XM_006530353.3	-0.926627709	0.00787774
mmu-miR-1892	XM_011248576.2	-0.961916254	0.00214794
mmu-miR-1892	NM_001198955.1	-0.854095612	0.030379123
mmu-miR-1892	XM_006532232.3	-0.857532355	0.02899971
mmu-miR-1892	XM_011250978.2	-0.957367711	0.002687526
mmu-miR-1892	XM_006515096.2	-0.899159914	0.014740377
mmu-miR-1892	XM_006505602.3	-0.880861673	0.02044539
mmu-miR-1892	NM_001164086.1	-0.888763848	0.017872033
mmu-miR-1892	NM_013558.2	-0.884647598	0.019191815
mmu-miR-1892	NM_013560.2	-0.836247065	0.038027016
mmu-miR-1892	XM_006539957.3	-0.905912069	0.01286235
mmu-miR-1892	NM_013868.4	-0.932078815	0.006763261
mmu-miR-1892	NM_030704.3	-0.840811183	0.035994611
mmu-miR-1892	XM_006511311.2	-0.909766565	0.011845766
mmu-miR-1892	XM_006516224.3	-0.866210782	0.025651949
mmu-miR-1892	XM_006528842.1	-0.879129075	0.021031722
mmu-miR-1892	NM_001302471.1	-0.917489305	0.009931155
mmu-miR-1892	NM_001289875.1	-0.889789424	0.017550227
mmu-miR-1892	XM_017315734.1	-0.944104562	0.004599133
mmu-miR-1892	XM_006497892.3	-0.884619434	0.019201
mmu-miR-1892	XM_011248919.2	-0.854035377	0.03040357
mmu-miR-1892	XM_017316478.1	-0.867213028	0.025277895

mmu-miR-1892	NM_001081667.2	-0.908413728	0.012197953
mmu-miR-1892	XM_006512296.3	-0.852658234	0.030965029
mmu-miR-1892	NM_029274.2	-0.882206134	0.019995874
mmu-miR-1892	XM_017316676.1	-0.8265786	0.04250465
mmu-miR-1892	NM_008479.2	-0.845074158	0.034143758
mmu-miR-1892	XM_017313062.1	-0.979678099	0.000615273
mmu-miR-1892	NM_001302765.1	-0.846691809	0.03345347
mmu-miR-1892	XM_006519969.3	-0.94742729	0.004073182
mmu-miR-1892	NM_144799.2	-0.877780909	0.021493435
mmu-miR-1892	NM_053098.2	-0.864866067	0.026157917
mmu-miR-1892	XM_006506340.3	-0.87946503	0.020917412
mmu-miR-1892	NM_028838.2	-0.827909058	0.041874677
mmu-miR-1892	XM_006541232.3	-0.850932036	0.031675648
mmu-miR-1892	XM_006498872.3	-0.937855941	0.00567283
mmu-miR-1892	XM_006532410.3	-0.843009164	0.035034576
mmu-miR-1892	NM_013593.3	-0.838572989	0.036984732
mmu-miR-1892	NM_010825.3	-0.973694105	0.001028898
mmu-miR-1892	NM_001270475.1	-0.921427933	0.009017819
mmu-miR-1892	NM_001290512.1	-0.977286199	0.000768016
mmu-miR-1892	NM_001005423.2	-0.860545355	0.027815368
mmu-miR-1892	NM_001099314.1	-0.928460817	0.007493718
mmu-miR-1892	XM_006537659.2	-0.833688024	0.039189443
mmu-miR-1892	XM_006529753.2	-0.90674211	0.012640017
mmu-miR-1892	NM_175260.2	-0.964654937	0.001851832
mmu-miR-1892	NM_001039545.2	-0.921285343	0.009050138
mmu-miR-1892	XM_006532412.1	-0.950267609	0.003648464
mmu-miR-1892	XM_017315841.1	-0.975397307	0.000900493
mmu-miR-1892	XM_006530186.3	-0.974089497	0.000998334
mmu-miR-1892	NM_010859.2	-0.965714209	0.001743121
mmu-miR-1892	XM_006513859.3	-0.851349411	0.03150313
mmu-miR-1892	XM_017320223.1	-0.88701701	0.018426611
mmu-miR-1892	XM_006501829.3	-0.951792047	0.003429992
mmu-miR-1892	NM_173437.2	-0.8204166	0.045479496
mmu-miR-1892	NM_011424.3	-0.8652126	0.02602708
mmu-miR-1892	NM_001134300.2	-0.938128853	0.005623636
mmu-miR-1892	NM_001109985.1	-0.916668684	0.010126831
mmu-miR-1892	NM_001029877.3	-0.834503981	0.038817019
mmu-miR-1892	XM_006526747.3	-0.883500668	0.019567572
mmu-miR-1892	NM_001077403.1	-0.953544346	0.003187063
mmu-miR-1892	XM_006530325.3	-0.871123425	0.023843491
mmu-miR-1892	NM_001286743.1	-0.863676817	0.0266093
mmu-miR-1892	NM_172454.2	-0.877502915	0.021589237
mmu-miR-1892	NM_028748.2	-0.83758402	0.037426244
mmu-miR-1892	NM_133167.3	-0.942281183	0.004901049
mmu-miR-1892	NM_013743.2	-0.831287575	0.040294718
mmu-miR-1892	XM_006509465.2	-0.823509056	0.043974817
mmu-miR-1892	XM_006538456.3	-0.887470318	0.018281916
mmu-miR-1892	XM_006537815.3	-0.946594122	0.00420212
mmu-miR-1892	NM_153104.3	-0.965151425	0.001800474
mmu-miR-1892	XM_017314870.1	-0.856939012	0.029235695
mmu-miR-1892	XM_006512076.3	-0.919022427	0.009570551
mmu-miR-1892	NM_148932.2	-0.856869692	0.029263323
mmu-miR-1892	NM_011145.3	-0.907902944	0.012332224
mmu-miR-1892	XM_006525698.2	-0.848716822	0.032598721

mmu-miR-1892	NM_026814.3	-0.899607435	0.014612089
mmu-miR-1892	NM_001167908.1	-0.869141516	0.024565508
mmu-miR-1892	XM_006527008.2	-0.952692964	0.003303998
mmu-miR-1892	XM_011239558.2	-0.91042399	0.01167642
mmu-miR-1892	NM_175563.5	-0.905213551	0.013050903
mmu-miR-1892	NM_175022.2	-0.82243183	0.04449628
mmu-miR-1892	XM_006505015.3	-0.975202482	0.000914751
mmu-miR-1892	XM_006514153.2	-0.831089508	0.040386559
mmu-miR-1892	NM_019732.2	-0.864234434	0.026397198
mmu-miR-1892	XM_006540138.3	-0.88173283	0.020153576
mmu-miR-1892	NM_020052.2	-0.912177323	0.011230553
mmu-miR-1892	NM_009244.4	-0.897478244	0.015227278
mmu-miR-1892	NM_009246.3	-0.900192787	0.014445106
mmu-miR-1892	XM_017322211.1	-0.839202043	0.03670518
mmu-miR-1892	XM_006530238.3	-0.816752285	0.047292884
mmu-miR-1892	XM_017316715.1	-0.829672369	0.041046522
mmu-miR-1892	XM_017317034.1	-0.909159212	0.012003262
mmu-miR-1892	XM_017316897.1	-0.901745698	0.014006593
mmu-miR-1892	XM_011250732.2	-0.880856211	0.020447226
mmu-miR-1892	NM_001033167.3	-0.913641768	0.010864598
mmu-miR-1892	NM_001007570.2	-0.952917497	0.003272958
mmu-miR-1892	XM_006503080.3	-0.912513504	0.011146024
mmu-miR-1892	NM_027052.3	-0.845367107	0.034018258
mmu-miR-1892	NM_025540.2	-0.971250323	0.001227934
mmu-miR-1892	XM_006500109.3	-0.902667254	0.013749446
mmu-miR-1892	XM_006539074.3	-0.854382569	0.030262787
mmu-miR-1892	NM_001304266.1	-0.817704565	0.046818451
mmu-miR-1892	NM_001242411.1	-0.825769826	0.042889751
mmu-miR-1892	XM_006506145.1	-0.904785209	0.013167183
mmu-miR-1892	XM_011248193.2	-0.830062445	0.040864365
mmu-miR-1892	XM_011245529.2	-0.90219129	0.01388197
mmu-miR-1892	NM_011374.2	-0.828037098	0.041814281
mmu-miR-1892	NM_009332.3	-0.942942278	0.004790497
mmu-miR-1892	NM_001080979.1	-0.83994054	0.036378262
mmu-miR-1892	NM_153801.3	-0.860084996	0.02799481
mmu-miR-1892	NM_009379.3	-0.88062081	0.020526427
mmu-miR-1892	NM_001161746.1	-0.882032495	0.020053661
mmu-miR-1892	XM_006538830.1	-0.916554894	0.010154111
mmu-miR-1892	NM_009393.2	-0.983341829	0.000413931
mmu-miR-1892	XM_006529382.3	-0.973485421	0.001045214
mmu-miR-1892	XM_006539726.3	-0.965772623	0.001737221
mmu-miR-1892	XM_011248201.2	-0.935133218	0.006175079
mmu-miR-1892	NM_177409.3	-0.871664491	0.023648161
mmu-miR-1892	NM_001039047.1	-0.877059033	0.021742628
mmu-miR-1892	XM_006508002.3	-0.8991374	0.014746845
mmu-miR-1892	NM_001033149.3	-0.898485742	0.014934657
mmu-miR-1892	XM_006538224.1	-0.877006968	0.021760654
mmu-miR-1892	XM_006525099.3	-0.976554515	0.000818092
mmu-miR-1892	NM_009504.4	-0.938762793	0.005510174
mmu-miR-1892	NM_001244031.1	-0.940655453	0.005178164
mmu-miR-1892	NM_011724.3	-0.854975197	0.030023196
mmu-miR-1892	XM_006530585.3	-0.854367011	0.030269088
mmu-miR-1892	NM_199029.2	-0.832515013	0.039727752
mmu-miR-1892	XM_006511007.3	-0.890245579	0.017407997

mmu-miR-1892	NM_001110508.1	-0.817599386	0.046870743
mmu-miR-5113	NM_029685.1	-0.877071827	0.021738199
mmu-miR-5113	NM_025865.2	-0.956814862	0.002757165
mmu-miR-5113	XM_011241322.1	-0.867016397	0.025351074
mmu-miR-5113	NM_009608.4	-0.984863683	0.000341928
mmu-miR-5113	NM_033268.4	-0.82137845	0.045008968
mmu-miR-5113	NM_013906.3	-0.973168211	0.001070259
mmu-miR-5113	XM_017312667.1	-0.844163273	0.034535374
mmu-miR-5113	XM_011240149.1	-0.826356827	0.042610087
mmu-miR-5113	XM_017318264.1	-0.908092103	0.012282417
mmu-miR-5113	NM_023048.5	-0.947302992	0.004092293
mmu-miR-5113	NM_013415.5	-0.91049217	0.011658925
mmu-miR-5113	NM_009722.3	-0.977281709	0.000768318
mmu-miR-5113	XM_006540762.3	-0.941384553	0.005052961
mmu-miR-5113	NM_001285867.1	-0.845724795	0.033865319
mmu-miR-5113	NM_001190379.1	-0.820320905	0.045526434
mmu-miR-5113	XM_006501443.3	-0.896712835	0.015451412
mmu-miR-5113	XM_006520253.3	-0.897693508	0.015164526
mmu-miR-5113	XM_006500947.3	-0.911903291	0.011299685
mmu-miR-5113	NM_009824.2	-0.817977662	0.046682803
mmu-miR-5113	XM_011245344.2	-0.985657863	0.00030707
mmu-miR-5113	XM_006531321.3	-0.878584442	0.021217668
mmu-miR-5113	XM_006523030.3	-0.859382659	0.028269625
mmu-miR-5113	XM_011250217.1	-0.958551468	0.002541367
mmu-miR-5113	XM_006527480.3	-0.929347717	0.007311279
mmu-miR-5113	NM_001113515.2	-0.814326132	0.048511645
mmu-miR-5113	XM_006532938.2	-0.884520642	0.019233237
mmu-miR-5113	XM_011247208.1	-0.814864792	0.048239809
mmu-miR-5113	NM_001289782.1	-0.858457281	0.028633654
mmu-miR-5113	NM_007883.3	-0.863042283	0.026851638
mmu-miR-5113	XM_006514873.2	-0.833992085	0.039050467
mmu-miR-5113	NM_001160049.1	-0.817122026	0.047108412
mmu-miR-5113	NM_010133.2	-0.829538194	0.041109267
mmu-miR-5113	XM_011238916.2	-0.9405786	0.005191449
mmu-miR-5113	NM_010174.1	-0.929662672	0.007247018
mmu-miR-5113	XM_006524407.3	-0.891610902	0.016985607
mmu-miR-5113	XM_017319626.1	-0.94708464	0.00412597
mmu-miR-5113	NM_001160262.1	-0.95633505	0.002818315
mmu-miR-5113	NM_010181.2	-0.902124945	0.013900491
mmu-miR-5113	XM_006527803.1	-0.851026932	0.031636385
mmu-miR-5113	XM_006502769.3	-0.870163187	0.024192029
mmu-miR-5113	NM_172862.3	-0.888693306	0.017894271
mmu-miR-5113	NM_001033445.2	-0.907644531	0.012400424
mmu-miR-5113	XM_006530353.3	-0.85243867	0.031054994
mmu-miR-5113	XM_011248576.2	-0.813987967	0.048682662
mmu-miR-5113	XM_006526069.2	-0.942433489	0.00487547
mmu-miR-5113	NM_001164086.1	-0.941269774	0.005072572
mmu-miR-5113	NM_013558.2	-0.905468718	0.012981871
mmu-miR-5113	XM_006539957.3	-0.824727214	0.043388586
mmu-miR-5113	NM_013868.4	-0.837099089	0.037643633
mmu-miR-5113	XM_006511311.2	-0.922368004	0.008806157
mmu-miR-5113	NM_001302471.1	-0.87430517	0.022705847
mmu-miR-5113	XM_017315734.1	-0.889974044	0.017492595
mmu-miR-5113	NM_001081667.2	-0.954951211	0.002998379

mmu-miR-5113	XM_017313062.1	-0.834726965	0.038715533
mmu-miR-5113	NM_001302765.1	-0.879203759	0.021006285
mmu-miR-5113	XM_006519969.3	-0.851935436	0.031261654
mmu-miR-5113	NM_053098.2	-0.837269311	0.037567257
mmu-miR-5113	NM_028838.2	-0.849053975	0.032457423
mmu-miR-5113	XM_006541232.3	-0.884734732	0.019163411
mmu-miR-5113	XM_006498872.3	-0.884673352	0.019183418
mmu-miR-5113	XM_006532410.3	-0.87951009	0.020902102
mmu-miR-5113	NM_010825.3	-0.959333458	0.002447025
mmu-miR-5113	NM_001270475.1	-0.865283468	0.026000362
mmu-miR-5113	NM_001290512.1	-0.860882704	0.027684221
mmu-miR-5113	XM_006537659.2	-0.877589851	0.021559255
mmu-miR-5113	XM_006529753.2	-0.871170405	0.0238265
mmu-miR-5113	NM_001039545.2	-0.821492137	0.044953504
mmu-miR-5113	XM_006532412.1	-0.896743435	0.015442421
mmu-miR-5113	XM_017315841.1	-0.92105213	0.009103117
mmu-miR-5113	XM_006530186.3	-0.881602614	0.020197068
mmu-miR-5113	NM_010859.2	-0.952329535	0.003354545
mmu-miR-5113	XM_006513859.3	-0.855808208	0.029687944
mmu-miR-5113	XM_017320223.1	-0.919376855	0.009488108
mmu-miR-5113	XM_006501829.3	-0.936041782	0.006005165
mmu-miR-5113	NM_001134300.2	-0.82470424	0.043399608
mmu-miR-5113	NM_001109985.1	-0.982620847	0.000450428
mmu-miR-5113	XM_006526747.3	-0.848734101	0.032591473
mmu-miR-5113	NM_198190.1	-0.871843316	0.023583772
mmu-miR-5113	NM_011010.2	-0.846483623	0.033541935
mmu-miR-5113	NM_133167.3	-0.823716103	0.043874918
mmu-miR-5113	NM_013743.2	-0.874174654	0.022751992
mmu-miR-5113	XM_006537815.3	-0.841816747	0.035553886
mmu-miR-5113	NM_153104.3	-0.955742782	0.002894709
mmu-miR-5113	XM_017314870.1	-0.899434519	0.014661594
mmu-miR-5113	XM_006512076.3	-0.826382627	0.042597815
mmu-miR-5113	XM_006525698.2	-0.863845632	0.026545002
mmu-miR-5113	NM_026814.3	-0.921125509	0.009086432
mmu-miR-5113	XM_006527008.2	-0.894811736	0.016014925
mmu-miR-5113	XM_006505015.3	-0.842247461	0.035365891
mmu-miR-5113	NM_001195662.1	-0.958215617	0.002582426
mmu-miR-5113	XM_006540138.3	-0.930116539	0.007154902
mmu-miR-5113	XM_006540134.3	-0.89755924	0.015203652
mmu-miR-5113	NM_018732.3	-0.855382886	0.029858895
mmu-miR-5113	NM_020052.2	-0.919613603	0.009433232
mmu-miR-5113	XM_017316715.1	-0.870539057	0.024055312
mmu-miR-5113	XM_017316897.1	-0.979503734	0.00062584
mmu-miR-5113	NM_009199.2	-0.848874617	0.032532555
mmu-miR-5113	NM_001007570.2	-0.812627724	0.049373387
mmu-miR-5113	NM_027052.3	-0.949900076	0.003702128
mmu-miR-5113	NM_025540.2	-0.948569037	0.003899695
mmu-miR-5113	XM_006500109.3	-0.851812869	0.031312086
mmu-miR-5113	NM_009332.3	-0.872869912	0.023215747
mmu-miR-5113	NM_153801.3	-0.859177113	0.028350293
mmu-miR-5113	NM_009379.3	-0.962711154	0.002059763
mmu-miR-5113	NM_001161746.1	-0.885243621	0.018997925
mmu-miR-5113	NM_009393.2	-0.927591698	0.007674626
mmu-miR-5113	XM_006529382.3	-0.944254912	0.004574658

mmu-miR-5113	XM_006539726.3	-0.944455077	0.004542173
mmu-miR-5113	XM_006508002.3	-0.884192578	0.019340471
mmu-miR-5113	NM_001033149.3	-0.939315041	0.005412255
mmu-miR-5113	XM_006525099.3	-0.880518291	0.020560965
mmu-miR-5113	NM_009504.4	-0.845865493	0.033805248
mmu-miR-5113	NM_001244031.1	-0.96766207	0.001551704
mmu-miR-5113	NM_011724.3	-0.842922416	0.035072235
mmu-miR-5128	XM_011241322.1	-0.914098254	0.010751726
mmu-miR-5128	NM_138955.3	-0.847222697	0.033228376
mmu-miR-5128	XM_006540566.1	-0.972766075	0.00110243
mmu-miR-5128	XM_006498287.3	-0.910711153	0.01160282
mmu-miR-5128	XM_006508990.2	-0.844608207	0.034343821
mmu-miR-5128	NM_025758.4	-0.882942161	0.019751812
mmu-miR-5128	NM_007588.2	-0.994353529	4.77339E-05
mmu-miR-5128	XM_006520253.3	-0.864435145	0.026321051
mmu-miR-5128	XM_006526449.3	-0.879240683	0.020993714
mmu-miR-5128	NM_001013784.1	-0.828936408	0.041391233
mmu-miR-5128	XM_006533653.3	-0.881486695	0.020235822
mmu-miR-5128	NM_001113515.2	-0.920892389	0.009139493
mmu-miR-5128	NM_007739.2	-0.848166981	0.032829775
mmu-miR-5128	XM_011240755.1	-0.820616253	0.04538164
mmu-miR-5128	NM_011957.2	-0.849156639	0.032414455
mmu-miR-5128	NM_010055.3	-0.896511554	0.015510615
mmu-miR-5128	NM_001317365.1	-0.954425616	0.003068207
mmu-miR-5128	NM_007883.3	-0.889076119	0.017773751
mmu-miR-5128	NM_178676.4	-0.977250337	0.000770434
mmu-miR-5128	XM_006511221.3	-0.84740973	0.033149246
mmu-miR-5128	NM_001033445.2	-0.87467452	0.022575503
mmu-miR-5128	XM_006515096.2	-0.987139079	0.000247041
mmu-miR-5128	NM_001302471.1	-0.863737528	0.026586168
mmu-miR-5128	XM_017315734.1	-0.8752639	0.022368252
mmu-miR-5128	NM_144945.3	-0.813133069	0.049116248
mmu-miR-5128	NM_001099314.1	-0.953335038	0.003215619
mmu-miR-5128	NM_175260.2	-0.900531984	0.014348767
mmu-miR-5128	NM_010859.2	-0.834289438	0.03891478
mmu-miR-5128	NM_001077403.1	-0.860386944	0.027877053
mmu-miR-5128	NM_145210.2	-0.946908168	0.004153288
mmu-miR-5128	NM_145226.2	-0.817490223	0.046925044
mmu-miR-5128	XM_006530294.3	-0.882641809	0.019851234
mmu-miR-5128	NM_001286743.1	-0.861001894	0.027637956
mmu-miR-5128	NM_172454.2	-0.874636111	0.022589041
mmu-miR-5128	NM_028748.2	-0.819851849	0.045756828
mmu-miR-5128	XM_006537815.3	-0.938432043	0.00556923
mmu-miR-5128	XM_006502235.3	-0.852708347	0.030944513
mmu-miR-5128	NM_011145.3	-0.867303087	0.025244411
mmu-miR-5128	XM_011239558.2	-0.858251582	0.028714873
mmu-miR-5128	XM_006524382.3	-0.86218676	0.027180026
mmu-miR-5128	XM_006505015.3	-0.871429361	0.023732951
mmu-miR-5128	XM_006514077.1	-0.839768803	0.036454164
mmu-miR-5128	NM_009246.3	-0.965173136	0.001798245
mmu-miR-5128	NM_009199.2	-0.826890002	0.042356807
mmu-miR-5128	NM_001033167.3	-0.8340512	0.039023474
mmu-miR-5128	NM_025540.2	-0.845205841	0.034087318
mmu-miR-5128	XM_006538830.1	-0.89980197	0.014556491

mmu-miR-5128	XM_006506152.3	-0.855621469	0.029762943
mmu-miR-5128	XM_006501309.3	-0.979146062	0.000647796
mmu-miR-5128	NM_001244031.1	-0.812228573	0.04957693
mmu-miR-5128	NM_199304.1	-0.887307772	0.018333738
mmu-miR-344i	XM_011241322.1	-0.901838901	0.013980482
mmu-miR-344i	NM_138955.3	-0.906273631	0.012765273
mmu-miR-344i	XM_006540566.1	-0.837769209	0.037343382
mmu-miR-344i	NM_008032.3	-0.889196358	0.017735978
mmu-miR-344i	NM_153178.4	-0.830115131	0.040839791
mmu-miR-344i	XM_011250176.1	-0.89468643	0.016052408
mmu-miR-344i	XM_006498287.3	-0.894840627	0.016006288
mmu-miR-344i	XM_006508990.2	-0.889855058	0.017529728
mmu-miR-344i	NM_013468.3	-0.833821573	0.039128373
mmu-miR-344i	NM_001290469.1	-0.863494861	0.026678685
mmu-miR-344i	XM_006529727.2	-0.857931524	0.028841462
mmu-miR-344i	NM_178782.4	-0.842976734	0.035048652
mmu-miR-344i	XM_006524328.3	-0.833050633	0.039481523
mmu-miR-344i	NM_007588.2	-0.912573558	0.011130957
mmu-miR-344i	XM_011246479.2	-0.902917069	0.013680135
mmu-miR-344i	NM_001013784.1	-0.907241869	0.012507057
mmu-miR-344i	XM_006533653.3	-0.816623398	0.047357267
mmu-miR-344i	XM_006509966.3	-0.920706276	0.009181962
mmu-miR-344i	XM_006503371.2	-0.856603132	0.029369682
mmu-miR-344i	XM_006530131.1	-0.878358209	0.021295138
mmu-miR-344i	NM_007739.2	-0.826079209	0.042742246
mmu-miR-344i	XM_011248602.2	-0.881892628	0.020100266
mmu-miR-344i	NM_011957.2	-0.891935588	0.016885892
mmu-miR-344i	NM_010055.3	-0.862605788	0.027018947
mmu-miR-344i	NM_001317365.1	-0.988321043	0.000203801
mmu-miR-344i	NM_178676.4	-0.88251109	0.019894579
mmu-miR-344i	XM_006511221.3	-0.937461808	0.005744244
mmu-miR-344i	XM_006532962.2	-0.892605559	0.016681028
mmu-miR-344i	NM_010181.2	-0.827538687	0.042049607
mmu-miR-344i	NM_001033221.3	-0.850904055	0.03168723
mmu-miR-344i	NM_001033445.2	-0.85180903	0.031313667
mmu-miR-344i	XM_006530353.3	-0.813517255	0.048921176
mmu-miR-344i	XM_011248576.2	-0.843762536	0.034708328
mmu-miR-344i	NM_001198955.1	-0.877702716	0.021520361
mmu-miR-344i	XM_006532232.3	-0.860986366	0.027643981
mmu-miR-344i	XM_011250978.2	-0.843392014	0.034868604
mmu-miR-344i	NM_010340.2	-0.911584523	0.01138036
mmu-miR-344i	XM_006521554.3	-0.90566251	0.012929562
mmu-miR-344i	XM_006515096.2	-0.940264542	0.00524591
mmu-miR-344i	NM_001162950.1	-0.817223552	0.047057818
mmu-miR-344i	NM_001302471.1	-0.855149913	0.029952732
mmu-miR-344i	NM_001289875.1	-0.863991248	0.0264896
mmu-miR-344i	XM_017315734.1	-0.868984067	0.024623306
mmu-miR-344i	NM_008479.2	-0.873829781	0.022874139
mmu-miR-344i	NM_144945.3	-0.953818671	0.003149827
mmu-miR-344i	XM_006506340.3	-0.895626378	0.015772264
mmu-miR-344i	NM_001099314.1	-0.945433476	0.004385022
mmu-miR-344i	XM_006529753.2	-0.835125023	0.038534676
mmu-miR-344i	NM_175260.2	-0.925941326	0.008023936
mmu-miR-344i	NM_001134300.2	-0.875389503	0.022324202

mmu-miR-344i	NM_001029877.3	-0.847956836	0.032918285
mmu-miR-344i	NM_001077403.1	-0.913636862	0.010865814
mmu-miR-344i	NM_145210.2	-0.87651758	0.021930438
mmu-miR-344i	XM_006530325.3	-0.819458247	0.045950578
mmu-miR-344i	NM_145226.2	-0.940010942	0.00529009
mmu-miR-344i	XM_006530294.3	-0.950049227	0.003680304
mmu-miR-344i	NM_011854.2	-0.898708777	0.014870248
mmu-miR-344i	NM_001286743.1	-0.882707262	0.019829547
mmu-miR-344i	NM_133167.3	-0.855575986	0.029781224
mmu-miR-344i	XM_006537815.3	-0.923363493	0.008584682
mmu-miR-344i	NM_148932.2	-0.82294831	0.044245905
mmu-miR-344i	NM_011145.3	-0.930635922	0.007050195
mmu-miR-344i	NM_001167908.1	-0.846426295	0.033566316
mmu-miR-344i	XM_011239558.2	-0.940448343	0.005214003
mmu-miR-344i	NM_175563.5	-0.898519941	0.014924772
mmu-miR-344i	XM_006524382.3	-0.931463874	0.006884837
mmu-miR-344i	XM_011240476.2	-0.90729822	0.012492108
mmu-miR-344i	XM_006514077.1	-0.84602819	0.033735848
mmu-miR-344i	NM_019732.2	-0.856629	0.029359353
mmu-miR-344i	NM_009243.4	-0.821647824	0.044877601
mmu-miR-344i	NM_009246.3	-0.919287608	0.009508835
mmu-miR-344i	XM_017317034.1	-0.890635432	0.01728688
mmu-miR-344i	NM_001033167.3	-0.920033251	0.00933634
mmu-miR-344i	NM_001007570.2	-0.822281883	0.044569093
mmu-miR-344i	XM_006503080.3	-0.874683503	0.022572337
mmu-miR-344i	XM_006534520.3	-0.857462057	0.029027621
mmu-miR-344i	XM_006506145.1	-0.840849869	0.035977608
mmu-miR-344i	XM_011245529.2	-0.867430819	0.025196957
mmu-miR-344i	XM_006538830.1	-0.958151473	0.002590304
mmu-miR-344i	XM_011248201.2	-0.902390196	0.013826514
mmu-miR-344i	NM_177409.3	-0.817194249	0.047072418
mmu-miR-344i	NM_001039047.1	-0.873332063	0.023050971
mmu-miR-344i	XM_006506152.3	-0.868796751	0.024692153
mmu-miR-344i	XM_006501309.3	-0.912089806	0.011252609
mmu-miR-344i	XM_006533569.3	-0.852009331	0.031231268
mmu-miR-344i	NM_199304.1	-0.901435604	0.014093637
mmu-miR-344i	XM_006511007.3	-0.83413368	0.038985827
mmu-miR-6238	NM_024283.3	-0.865790766	0.025809482
mmu-miR-6238	XM_006515980.3	-0.844236497	0.034503815
mmu-miR-6238	NM_153151.3	-0.814016681	0.04866813
mmu-miR-6238	XM_006500919.3	-0.886692578	0.018530505
mmu-miR-6238	XM_006495613.3	-0.860159425	0.027965761
mmu-miR-6238	NM_001303431.1	-0.938951483	0.005476621
mmu-miR-6238	XM_006522398.3	-0.851239031	0.031548712
mmu-miR-6238	NM_025711.3	-0.922634909	0.008746507
mmu-miR-6238	NM_001097617.1	-0.812811145	0.049279984
mmu-miR-6238	NM_027416.3	-0.811632853	0.049881434
mmu-miR-6238	NM_019626.3	-0.856874046	0.029261588
mmu-miR-6238	NM_001001178.1	-0.852675395	0.030958003
mmu-miR-6238	XM_017312731.1	-0.839664933	0.036500107
mmu-miR-6238	XM_017314944.1	-0.82886043	0.041426897
mmu-miR-6238	NM_009930.2	-0.859285296	0.028307822
mmu-miR-6238	NM_001309809.2	-0.836286606	0.038009184
mmu-miR-6238	NM_018866.2	-0.953715945	0.003163745

mmu-miR-6238	NM_001190451.2	-0.880760389	0.020479448
mmu-miR-6238	NM_015814.2	-0.859636704	0.028170074
mmu-miR-6238	NM_010140.3	-0.829624125	0.041069078
mmu-miR-6238	NM_177743.5	-0.914591183	0.010630485
mmu-miR-6238	XM_006520437.2	-0.833078593	0.039468689
mmu-miR-6238	XM_006510297.3	-0.824535917	0.043480404
mmu-miR-6238	NM_010450.3	-0.896424523	0.015536246
mmu-miR-6238	NM_008264.1	-0.845306956	0.034044009
mmu-miR-6238	XM_006523711.3	-0.899873945	0.014535947
mmu-miR-6238	NM_001161541.1	-0.924453372	0.008345356
mmu-miR-6238	NM_010608.2	-0.819154745	0.046100237
mmu-miR-6238	NM_173427.2	-0.816844006	0.047247091
mmu-miR-6238	XM_017315313.1	-0.843395988	0.034866883
mmu-miR-6238	XM_006523061.3	-0.978448038	0.000691725
mmu-miR-6238	XM_006511102.2	-0.819585645	0.045887825
mmu-miR-6238	XM_006527221.3	-0.850909574	0.031684945
mmu-miR-6238	NM_010714.3	-0.876933225	0.021786197
mmu-miR-6238	NM_028973.2	-0.916421388	0.010186162
mmu-miR-6238	XM_006527321.2	-0.817004855	0.047166835
mmu-miR-6238	NM_008524.2	-0.825145865	0.04318796
mmu-miR-6238	NM_001252563.1	-0.831930133	0.039997445
mmu-miR-6238	NM_010779.2	-0.899894916	0.014529964
mmu-miR-6238	NM_010809.2	-0.845873436	0.033801859
mmu-miR-6238	XM_006529456.3	-0.814946258	0.048198759
mmu-miR-6238	XM_006510077.3	-0.815175549	0.048083308
mmu-miR-6238	NM_008760.4	-0.923528814	0.008548168
mmu-miR-6238	NM_172907.3	-0.852366294	0.031084676
mmu-miR-6238	XM_006516928.2	-0.847407635	0.033150132
mmu-miR-6238	XM_011239969.2	-0.875291348	0.022358622
mmu-miR-6238	NM_001164593.1	-0.882432309	0.019920723
mmu-miR-6238	XM_017312497.1	-0.968162741	0.001504281
mmu-miR-6238	NM_011169.5	-0.825855873	0.042848703
mmu-miR-6238	NM_008966.3	-0.84442481	0.034422717
mmu-miR-6238	NM_027455.3	-0.821240684	0.045076222
mmu-miR-6238	NM_198024.2	-0.822539455	0.044444052
mmu-miR-6238	XM_006525075.3	-0.84146362	0.035708366
mmu-miR-6238	NM_023893.4	-0.910361367	0.0116925
mmu-miR-6238	XM_011244046.2	-0.850356447	0.031914291
mmu-miR-6238	NM_001310705.1	-0.873105357	0.023131731
mmu-miR-6238	XM_017322365.1	-0.823440051	0.044008135
mmu-miR-6238	NM_001146217.1	-0.840954827	0.035931498
mmu-miR-6238	XM_006524777.3	-0.820178309	0.045596418
mmu-miR-6238	NM_011430.3	-0.851876099	0.031286065
mmu-miR-6238	NM_011581.3	-0.846274652	0.033630845
mmu-miR-6238	XM_006537836.1	-0.905336678	0.013017571
mmu-miR-6238	NM_178715.3	-0.823756544	0.043855418
mmu-miR-6238	NM_177371.3	-0.912241631	0.01121436
mmu-miR-6238	XM_006496905.1	-0.891098757	0.017143465
mmu-miR-6238	XM_017321602.1	-0.85305045	0.030804629
mmu-miR-6238	NM_177709.3	-0.906884196	0.012602147
mmu-miR-6238	NM_011658.2	-0.876897017	0.021798744
mmu-miR-6238	XM_011243586.2	-0.866406523	0.02557869
mmu-miR-6351	NM_024283.3	-0.934741026	0.00624914
mmu-miR-6351	XM_006515980.3	-0.822795148	0.044320085

mmu-miR-6351	NM_153151.3	-0.966825668	0.00163255
mmu-miR-6351	NM_001142804.1	-0.823259661	0.044095289
mmu-miR-6351	XM_006498086.3	-0.865926911	0.025758368
mmu-miR-6351	NM_145635.2	-0.812045354	0.049670491
mmu-miR-6351	NM_009645.2	-0.819458348	0.045950528
mmu-miR-6351	NM_009675.2	-0.825377181	0.043077295
mmu-miR-6351	NM_001305585.1	-0.8472079	0.03323464
mmu-miR-6351	XM_006522398.3	-0.859071023	0.028391971
mmu-miR-6351	NM_025711.3	-0.878430731	0.021270289
mmu-miR-6351	NM_020025.4	-0.961858357	0.002154433
mmu-miR-6351	NM_007555.4	-0.828574985	0.04156101
mmu-miR-6351	XM_017312731.1	-0.885888713	0.018789135
mmu-miR-6351	XM_017314944.1	-0.962944695	0.002034203
mmu-miR-6351	NM_001309809.2	-0.879255824	0.02098856
mmu-miR-6351	XM_006540596.2	-0.913946441	0.0107892
mmu-miR-6351	NM_001190451.2	-0.873939325	0.022835307
mmu-miR-6351	NM_015814.2	-0.848182724	0.032823149
mmu-miR-6351	NM_001291145.1	-0.843916637	0.034641772
mmu-miR-6351	NM_015744.4	-0.814191322	0.048579788
mmu-miR-6351	NM_010140.3	-0.893417854	0.016434257
mmu-miR-6351	NM_177743.5	-0.916773632	0.010101704
mmu-miR-6351	XM_006520437.2	-0.934954738	0.00620873
mmu-miR-6351	NM_145741.2	-0.843849045	0.034670957
mmu-miR-6351	XM_006510297.3	-0.845814734	0.033826914
mmu-miR-6351	NM_016697.3	-0.849589691	0.032233506
mmu-miR-6351	XM_006496277.3	-0.844782479	0.034268931
mmu-miR-6351	XM_011246309.2	-0.93029041	0.007119766
mmu-miR-6351	NM_010450.3	-0.871664394	0.023648196
mmu-miR-6351	XM_006523711.3	-0.889274074	0.017711583
mmu-miR-6351	NM_001161541.1	-0.953057819	0.003253632
mmu-miR-6351	NM_031180.2	-0.832573838	0.039700675
mmu-miR-6351	NM_173427.2	-0.926795358	0.007842231
mmu-miR-6351	NM_146063.1	-0.931579071	0.006861982
mmu-miR-6351	XM_006523061.3	-0.844158015	0.034537641
mmu-miR-6351	XM_006511102.2	-0.933115647	0.006560671
mmu-miR-6351	XM_006504999.1	-0.881299095	0.020298615
mmu-miR-6351	NM_001252563.1	-0.82337522	0.044039448
mmu-miR-6351	NM_010779.2	-0.868083305	0.024955213
mmu-miR-6351	XM_006498901.3	-0.876484436	0.021941959
mmu-miR-6351	NM_023456.3	-0.859251006	0.028321281
mmu-miR-6351	NM_008760.4	-0.920856931	0.009147577
mmu-miR-6351	NM_001164593.1	-0.842399846	0.035299491
mmu-miR-6351	NM_011082.3	-0.868593757	0.024766866
mmu-miR-6351	XM_017312497.1	-0.850914493	0.031682909
mmu-miR-6351	NM_001081224.2	-0.88775841	0.018190242
mmu-miR-6351	NM_009127.4	-0.830877122	0.040485149
mmu-miR-6351	NM_009135.2	-0.839307017	0.036658626
mmu-miR-6351	XM_011244046.2	-0.887077356	0.018407317
mmu-miR-6351	XM_006521796.1	-0.95251528	0.003328664
mmu-miR-6351	NM_001310705.1	-0.860128076	0.027977995
mmu-miR-6351	XM_006524777.3	-0.892759255	0.016634201
mmu-miR-6351	NM_011430.3	-0.910775168	0.011586443
mmu-miR-6351	NM_175692.3	-0.969720624	0.00136138
mmu-miR-6351	NM_031183.2	-0.861625559	0.027396467

mmu-miR-6351	NM_032400.2	-0.980012213	0.000595275
mmu-miR-6351	NM_009381.3	-0.853602136	0.030579678
mmu-miR-6351	NM_144936.1	-0.942546592	0.004856517
mmu-miR-6351	NM_011658.2	-0.813897159	0.048728633
mmu-miR-6351	XM_011248394.1	-0.915864336	0.010320425
mmu-miR-6351	XM_006525028.2	-0.86703467	0.02534427
mmu-miR-6351	NM_177789.4	-0.866838535	0.025417356
mmu-miR-6351	XM_011243586.2	-0.929869359	0.007204998
mmu-miR-6378	NM_024283.3	-0.970228192	0.001316347
mmu-miR-6378	XM_006515980.3	-0.882595114	0.019866712
mmu-miR-6378	NM_153151.3	-0.955091191	0.002979916
mmu-miR-6378	NM_001142804.1	-0.852008772	0.031231497
mmu-miR-6378	XM_006498086.3	-0.876103252	0.022074674
mmu-miR-6378	NM_145635.2	-0.884321028	0.019298451
mmu-miR-6378	NM_001303431.1	-0.883117738	0.019693804
mmu-miR-6378	NM_009675.2	-0.823063152	0.044190323
mmu-miR-6378	NM_001110009.2	-0.829737119	0.041016259
mmu-miR-6378	XM_006522398.3	-0.886298963	0.018656929
mmu-miR-6378	NM_025711.3	-0.916500311	0.010167209
mmu-miR-6378	NM_020025.4	-0.952144144	0.003380475
mmu-miR-6378	NM_007555.4	-0.844863529	0.034234127
mmu-miR-6378	NM_178692.3	-0.814995748	0.048173829
mmu-miR-6378	NM_001097617.1	-0.819910011	0.04572823
mmu-miR-6378	NM_027416.3	-0.842805407	0.03512306
mmu-miR-6378	XM_017312731.1	-0.915849576	0.010323994
mmu-miR-6378	NM_013459.3	-0.83408506	0.039008017
mmu-miR-6378	XM_017314944.1	-0.956704261	0.002771202
mmu-miR-6378	NM_009930.2	-0.825070996	0.043223807
mmu-miR-6378	NM_001309809.2	-0.848582438	0.032655121
mmu-miR-6378	NM_018866.2	-0.825126866	0.043197056
mmu-miR-6378	NM_030206.4	-0.816956118	0.047191146
mmu-miR-6378	NM_007817.2	-0.838364893	0.03707743
mmu-miR-6378	XM_006540596.2	-0.886291614	0.018659294
mmu-miR-6378	NM_001190451.2	-0.889752127	0.017561881
mmu-miR-6378	NM_001190448.1	-0.821270167	0.045061825
mmu-miR-6378	NM_015814.2	-0.882085082	0.020036152
mmu-miR-6378	NM_015744.4	-0.858379024	0.028664541
mmu-miR-6378	NM_010140.3	-0.894806995	0.016016342
mmu-miR-6378	NM_177743.5	-0.939245481	0.005424542
mmu-miR-6378	XM_006520437.2	-0.921613374	0.008975873
mmu-miR-6378	NM_145741.2	-0.840393794	0.036178296
mmu-miR-6378	XM_006510297.3	-0.880608674	0.020530514
mmu-miR-6378	XM_006496277.3	-0.854060642	0.030393314
mmu-miR-6378	XM_011246309.2	-0.8756515	0.022232452
mmu-miR-6378	NM_010450.3	-0.926796142	0.007842065
mmu-miR-6378	XM_006523711.3	-0.933321425	0.006520821
mmu-miR-6378	NM_001161541.1	-0.97598075	0.000858458
mmu-miR-6378	NM_031180.2	-0.833135087	0.039442764
mmu-miR-6378	NM_173427.2	-0.952773471	0.003292852
mmu-miR-6378	NM_146063.1	-0.903214094	0.013597946
mmu-miR-6378	XM_017315313.1	-0.824817354	0.043345353
mmu-miR-6378	XM_006523061.3	-0.89901109	0.014783159
mmu-miR-6378	XM_006511102.2	-0.96270756	0.002060157
mmu-miR-6378	XM_006504999.1	-0.895393604	0.01584142

mmu-miR-6378	XM_006527221.3	-0.856074522	0.02958114
mmu-miR-6378	NM_028973.2	-0.847970635	0.03291247
mmu-miR-6378	XM_006527321.2	-0.814196767	0.048577034
mmu-miR-6378	NM_010779.2	-0.881676068	0.020172529
mmu-miR-6378	XM_006498901.3	-0.916675828	0.01012512
mmu-miR-6378	XM_006510077.3	-0.82361116	0.043925539
mmu-miR-6378	NM_023456.3	-0.886902127	0.018463369
mmu-miR-6378	NM_008760.4	-0.937448962	0.005746579
mmu-miR-6378	NM_011044.2	-0.86828019	0.024882486
mmu-miR-6378	NM_001164593.1	-0.845274807	0.034057776
mmu-miR-6378	NM_011082.3	-0.83957232	0.036541094
mmu-miR-6378	XM_017312497.1	-0.915996045	0.010288603
mmu-miR-6378	XM_011250776.2	-0.84930011	0.032354454
mmu-miR-6378	NM_011169.5	-0.874303494	0.022706439
mmu-miR-6378	NM_001081224.2	-0.834589677	0.038778001
mmu-miR-6378	NM_009127.4	-0.86569722	0.02584463
mmu-miR-6378	XM_011244046.2	-0.923947633	0.008456002
mmu-miR-6378	XM_006521796.1	-0.934339464	0.006325418
mmu-miR-6378	NM_001310705.1	-0.898337741	0.014977472
mmu-miR-6378	XM_017322365.1	-0.860654074	0.027773071
mmu-miR-6378	NM_001146217.1	-0.855931502	0.029638475
mmu-miR-6378	XM_006524777.3	-0.864328131	0.026361638
mmu-miR-6378	NM_011430.3	-0.955865464	0.002878802
mmu-miR-6378	NM_175692.3	-0.970348989	0.001305739
mmu-miR-6378	NM_178753.4	-0.814223623	0.048563456
mmu-miR-6378	NM_032400.2	-0.956115484	0.002846519
mmu-miR-6378	NM_009381.3	-0.873952783	0.022830539
mmu-miR-6378	XM_006537836.1	-0.815438535	0.047951049
mmu-miR-6378	NM_144936.1	-0.953086863	0.003249639
mmu-miR-6378	NM_177371.3	-0.822289677	0.044565307
mmu-miR-6378	NM_011658.2	-0.864612544	0.026253835
mmu-miR-6378	XM_011248394.1	-0.868515518	0.024795691
mmu-miR-6378	XM_006525028.2	-0.869528514	0.024423717
mmu-miR-6378	NM_177789.4	-0.893413103	0.016435695
mmu-miR-6378	XM_011243586.2	-0.908078808	0.012285914
mmu-miR-3473e	XM_017320219.1	-0.874444201	0.022656741
mmu-miR-3473e	NM_029685.1	-0.872795805	0.023242221
mmu-miR-3473e	XM_011241322.1	-0.996628098	1.70354E-05
mmu-miR-3473e	NM_138955.3	-0.963986755	0.001922077
mmu-miR-3473e	XM_006540566.1	-0.907768333	0.012367728
mmu-miR-3473e	NM_009608.4	-0.878719445	0.021171503
mmu-miR-3473e	NM_008032.3	-0.899415926	0.014666922
mmu-miR-3473e	NM_153178.4	-0.871130015	0.023841107
mmu-miR-3473e	XM_011250176.1	-0.886191121	0.018691639
mmu-miR-3473e	XM_006498287.3	-0.858528102	0.028605717
mmu-miR-3473e	XM_011240149.1	-0.885989378	0.018756654
mmu-miR-3473e	XM_006508990.2	-0.941286527	0.005069707
mmu-miR-3473e	XM_017318264.1	-0.868884511	0.024659886
mmu-miR-3473e	NM_001290469.1	-0.814865839	0.048239281
mmu-miR-3473e	NM_013415.5	-0.824904141	0.043303747
mmu-miR-3473e	NM_009722.3	-0.878549612	0.021229587
mmu-miR-3473e	XM_006529727.2	-0.874152264	0.022759913
mmu-miR-3473e	XM_006524328.3	-0.889947406	0.017500905
mmu-miR-3473e	NM_001285867.1	-0.851848976	0.031297226

mmu-miR-3473e	NM_007588.2	-0.920147309	0.00931009
mmu-miR-3473e	NM_001190379.1	-0.814504323	0.048421642
mmu-miR-3473e	XM_006520253.3	-0.932900339	0.006602493
mmu-miR-3473e	XM_006500947.3	-0.813442417	0.048959148
mmu-miR-3473e	NM_009824.2	-0.825677274	0.042933923
mmu-miR-3473e	NM_001013784.1	-0.908396488	0.012202473
mmu-miR-3473e	XM_006509966.3	-0.816581538	0.047378186
mmu-miR-3473e	XM_011245344.2	-0.848653292	0.032625378
mmu-miR-3473e	XM_006523030.3	-0.861963472	0.027266045
mmu-miR-3473e	XM_006530131.1	-0.921512872	0.008998595
mmu-miR-3473e	XM_011250217.1	-0.933397915	0.006506038
mmu-miR-3473e	XM_006527480.3	-0.917598876	0.009905168
mmu-miR-3473e	NM_001113515.2	-0.874104165	0.022776933
mmu-miR-3473e	NM_007739.2	-0.823575541	0.043942727
mmu-miR-3473e	XM_011248602.2	-0.932443356	0.006691689
mmu-miR-3473e	NM_011957.2	-0.921309859	0.009044577
mmu-miR-3473e	NM_001289782.1	-0.85077474	0.03174078
mmu-miR-3473e	NM_001198841.1	-0.818111894	0.046616197
mmu-miR-3473e	NM_001317365.1	-0.941547023	0.005025266
mmu-miR-3473e	NM_007883.3	-0.852607903	0.030985641
mmu-miR-3473e	NM_178676.4	-0.903622249	0.013485396
mmu-miR-3473e	XM_006511221.3	-0.881538665	0.020218443
mmu-miR-3473e	NM_010174.1	-0.834605877	0.038770628
mmu-miR-3473e	XM_006524407.3	-0.88925148	0.017718674
mmu-miR-3473e	XM_006532962.2	-0.83622582	0.038036599
mmu-miR-3473e	NM_010181.2	-0.95963884	0.00241066
mmu-miR-3473e	NM_001033445.2	-0.976631486	0.000812751
mmu-miR-3473e	XM_006514444.1	-0.819313295	0.046022026
mmu-miR-3473e	XM_006530353.3	-0.907282073	0.012496391
mmu-miR-3473e	XM_011248576.2	-0.893603874	0.016377994
mmu-miR-3473e	NM_001198955.1	-0.843141912	0.034976985
mmu-miR-3473e	XM_006532232.3	-0.846611309	0.033487664
mmu-miR-3473e	XM_011250978.2	-0.843993966	0.034608396
mmu-miR-3473e	NM_010340.2	-0.83810088	0.037195192
mmu-miR-3473e	XM_006515096.2	-0.968958206	0.001430434
mmu-miR-3473e	XM_006505602.3	-0.812371033	0.04950424
mmu-miR-3473e	NM_013868.4	-0.846116569	0.033698177
mmu-miR-3473e	XM_006516224.3	-0.83078788	0.040526609
mmu-miR-3473e	XM_006528842.1	-0.837099885	0.037643276
mmu-miR-3473e	NM_001302471.1	-0.968898289	0.001435932
mmu-miR-3473e	NM_001289875.1	-0.902630365	0.013759695
mmu-miR-3473e	NM_008423.2	-0.879677149	0.02084539
mmu-miR-3473e	XM_017315734.1	-0.982418668	0.000460938
mmu-miR-3473e	NM_001081667.2	-0.844509352	0.034386337
mmu-miR-3473e	NM_029274.2	-0.81665493	0.047341512
mmu-miR-3473e	XM_017313062.1	-0.86994553	0.024271366
mmu-miR-3473e	NM_144945.3	-0.89170452	0.016956827
mmu-miR-3473e	XM_006519969.3	-0.83625762	0.038022256
mmu-miR-3473e	NM_144799.2	-0.816554477	0.047391711
mmu-miR-3473e	XM_006498872.3	-0.83291916	0.039541896
mmu-miR-3473e	NM_010825.3	-0.912642911	0.011113569
mmu-miR-3473e	NM_001270475.1	-0.892071035	0.016844378
mmu-miR-3473e	NM_001290512.1	-0.845259199	0.034064461
mmu-miR-3473e	NM_001099314.1	-0.914317629	0.010697686

mmu-miR-3473e	XM_006529753.2	-0.946951814	0.004146523
mmu-miR-3473e	NM_175260.2	-0.927302307	0.00773533
mmu-miR-3473e	XM_006532412.1	-0.837654098	0.037394878
mmu-miR-3473e	XM_017315841.1	-0.885528264	0.018905661
mmu-miR-3473e	XM_006530186.3	-0.840346432	0.036199167
mmu-miR-3473e	NM_010859.2	-0.930153516	0.007147423
mmu-miR-3473e	XM_006501829.3	-0.848928251	0.03251008
mmu-miR-3473e	NM_001134300.2	-0.93109435	0.006958401
mmu-miR-3473e	NM_001109985.1	-0.942049308	0.004940117
mmu-miR-3473e	NM_001029877.3	-0.871034831	0.023875547
mmu-miR-3473e	NM_001077403.1	-0.945000856	0.004454175
mmu-miR-3473e	XM_006530325.3	-0.821686639	0.044858687
mmu-miR-3473e	NM_145226.2	-0.81287673	0.049246606
mmu-miR-3473e	NM_001286743.1	-0.851014446	0.031641549
mmu-miR-3473e	NM_133167.3	-0.913520806	0.010894603
mmu-miR-3473e	NM_013743.2	-0.85280879	0.030903411
mmu-miR-3473e	XM_006538456.3	-0.822862345	0.044287533
mmu-miR-3473e	XM_006537815.3	-0.990594679	0.000132274
mmu-miR-3473e	NM_153104.3	-0.899779172	0.014563002
mmu-miR-3473e	XM_017314870.1	-0.822155712	0.044630404
mmu-miR-3473e	XM_006512076.3	-0.861466233	0.027458064
mmu-miR-3473e	NM_148932.2	-0.814068308	0.048642007
mmu-miR-3473e	NM_011145.3	-0.877821676	0.021479403
mmu-miR-3473e	XM_006527008.2	-0.890257208	0.017404378
mmu-miR-3473e	XM_011239558.2	-0.952366754	0.003349351
mmu-miR-3473e	NM_175563.5	-0.834322086	0.038899896
mmu-miR-3473e	XM_006505015.3	-0.871915474	0.023557814
mmu-miR-3473e	XM_006514077.1	-0.835102139	0.038545063
mmu-miR-3473e	NM_001195662.1	-0.901195748	0.014161143
mmu-miR-3473e	NM_019732.2	-0.819552599	0.045904099
mmu-miR-3473e	NM_018732.3	-0.821776318	0.044815001
mmu-miR-3473e	NM_009246.3	-0.917807341	0.009855818
mmu-miR-3473e	XM_017316715.1	-0.829115354	0.041307294
mmu-miR-3473e	XM_017317034.1	-0.919579963	0.00944102
mmu-miR-3473e	XM_017316897.1	-0.827861211	0.041897256
mmu-miR-3473e	NM_009199.2	-0.815428493	0.047956096
mmu-miR-3473e	NM_001033167.3	-0.950356101	0.003635601
mmu-miR-3473e	NM_001007570.2	-0.881754005	0.020146509
mmu-miR-3473e	NM_001033286.2	-0.887569181	0.018250432
mmu-miR-3473e	XM_006503080.3	-0.848608842	0.032644036
mmu-miR-3473e	NM_025540.2	-0.908012461	0.012303375
mmu-miR-3473e	XM_006506145.1	-0.873937128	0.022836086
mmu-miR-3473e	XM_011245529.2	-0.856711942	0.029326244
mmu-miR-3473e	NM_009332.3	-0.91417837	0.010731975
mmu-miR-3473e	NM_153801.3	-0.912403882	0.011173554
mmu-miR-3473e	NM_009379.3	-0.846372136	0.033589355
mmu-miR-3473e	NM_001161746.1	-0.835551734	0.038341242
mmu-miR-3473e	XM_006538830.1	-0.967189434	0.001597139
mmu-miR-3473e	NM_009393.2	-0.890135462	0.017442281
mmu-miR-3473e	XM_006529382.3	-0.911736488	0.011341865
mmu-miR-3473e	XM_006539726.3	-0.882139722	0.020017967
mmu-miR-3473e	XM_011248201.2	-0.887343202	0.018322436
mmu-miR-3473e	NM_001039047.1	-0.922007936	0.00888694
mmu-miR-3473e	NM_001033149.3	-0.888421141	0.017980193

mmu-miR-3473e	XM_006501309.3	-0.862869377	0.026917854
mmu-miR-3473e	XM_006525099.3	-0.890286004	0.017395419
mmu-miR-3473e	NM_009504.4	-0.897938909	0.015093142
mmu-miR-3473e	NM_001244031.1	-0.946094318	0.004280414
mmu-miR-3473e	NM_011724.3	-0.850705406	0.031769509
mmu-miR-3473e	XM_006499466.3	-0.868084629	0.024954724
mmu-miR-3473e	XM_006530585.3	-0.819405559	0.045976543
mmu-miR-3473e	NM_199304.1	-0.814120009	0.048615853
mmu-miR-3473e	NM_199029.2	-0.815453422	0.047943567
mmu-miR-3473e	XM_006511007.3	-0.849225422	0.032385683
mmu-miR-7005-5p	NM_024283.3	-0.950283186	0.003646198
mmu-miR-7005-5p	XM_006515980.3	-0.850505457	0.031852429
mmu-miR-7005-5p	NM_153151.3	-0.900572376	0.014337315
mmu-miR-7005-5p	NM_145635.2	-0.893022205	0.016554233
mmu-miR-7005-5p	NM_001303431.1	-0.886655632	0.018542354
mmu-miR-7005-5p	XM_006522398.3	-0.878136934	0.02137104
mmu-miR-7005-5p	NM_025711.3	-0.897304428	0.015278038
mmu-miR-7005-5p	NM_020025.4	-0.895006635	0.015956707
mmu-miR-7005-5p	NM_027416.3	-0.842843302	0.035106595
mmu-miR-7005-5p	NM_001001178.1	-0.862461779	0.027074254
mmu-miR-7005-5p	XM_017312731.1	-0.904570926	0.01322554
mmu-miR-7005-5p	XM_017314944.1	-0.909087905	0.012021819
mmu-miR-7005-5p	NM_009930.2	-0.826262928	0.042654766
mmu-miR-7005-5p	NM_001309809.2	-0.834248004	0.038933674
mmu-miR-7005-5p	NM_018866.2	-0.841448245	0.035715099
mmu-miR-7005-5p	XM_006540596.2	-0.814204409	0.048573171
mmu-miR-7005-5p	NM_001190451.2	-0.856085631	0.029576688
mmu-miR-7005-5p	NM_015814.2	-0.849792941	0.032148743
mmu-miR-7005-5p	NM_015744.4	-0.81954299	0.045908832
mmu-miR-7005-5p	NM_010140.3	-0.837688765	0.037379366
mmu-miR-7005-5p	NM_177743.5	-0.92139863	0.009024457
mmu-miR-7005-5p	XM_006520437.2	-0.87324373	0.023082422
mmu-miR-7005-5p	XM_006510297.3	-0.835833871	0.038213596
mmu-miR-7005-5p	NM_010450.3	-0.907380527	0.012470288
mmu-miR-7005-5p	XM_006523711.3	-0.898212013	0.015013889
mmu-miR-7005-5p	NM_001161541.1	-0.95490242	0.003004828
mmu-miR-7005-5p	NM_173427.2	-0.922197118	0.008844451
mmu-miR-7005-5p	NM_146063.1	-0.866601906	0.025505664
mmu-miR-7005-5p	XM_006523061.3	-0.903748054	0.013450796
mmu-miR-7005-5p	XM_006511102.2	-0.933592141	0.006468576
mmu-miR-7005-5p	XM_006504999.1	-0.868472448	0.024811565
mmu-miR-7005-5p	XM_006527221.3	-0.818954367	0.046199168
mmu-miR-7005-5p	NM_028973.2	-0.872285904	0.02342477
mmu-miR-7005-5p	NM_010779.2	-0.845417623	0.033996639
mmu-miR-7005-5p	XM_006498901.3	-0.932111353	0.006756858
mmu-miR-7005-5p	XM_006510077.3	-0.815958279	0.047690161
mmu-miR-7005-5p	NM_023456.3	-0.827610621	0.042015605
mmu-miR-7005-5p	NM_008760.4	-0.911818413	0.011321139
mmu-miR-7005-5p	NM_172907.3	-0.847094796	0.03328254
mmu-miR-7005-5p	NM_011044.2	-0.826388952	0.042594806
mmu-miR-7005-5p	NM_001164593.1	-0.837305731	0.037550926
mmu-miR-7005-5p	XM_017312497.1	-0.961089825	0.002241548
mmu-miR-7005-5p	NM_011169.5	-0.848774571	0.032574499
mmu-miR-7005-5p	NM_009127.4	-0.816864523	0.04723685

mmu-miR-7005-5p	XM_011244046.2	-0.891680206	0.016964299
mmu-miR-7005-5p	XM_006521796.1	-0.908967734	0.012053124
mmu-miR-7005-5p	NM_001310705.1	-0.883391163	0.01960363
mmu-miR-7005-5p	XM_017322365.1	-0.814046733	0.048652923
mmu-miR-7005-5p	NM_001146217.1	-0.838417213	0.037054113
mmu-miR-7005-5p	XM_006524777.3	-0.827380818	0.042124273
mmu-miR-7005-5p	NM_011430.3	-0.9651868	0.001796842
mmu-miR-7005-5p	NM_175692.3	-0.93815217	0.005619442
mmu-miR-7005-5p	NM_032400.2	-0.897441373	0.015238038
mmu-miR-7005-5p	NM_009381.3	-0.836325432	0.037991677
mmu-miR-7005-5p	XM_006537836.1	-0.816066968	0.047635688
mmu-miR-7005-5p	NM_144936.1	-0.926555988	0.007892955
mmu-miR-7005-5p	NM_177709.3	-0.81440581	0.04847139
mmu-miR-7005-5p	NM_011658.2	-0.853243332	0.030725892
mmu-miR-7005-5p	XM_006525028.2	-0.81850617	0.046420811
mmu-miR-7005-5p	NM_177789.4	-0.855801348	0.029690698
mmu-miR-7005-5p	XM_011243586.2	-0.898167064	0.015026919
mmu-miR-7009-5p	NM_024283.3	-0.998841223	2.01337E-06
mmu-miR-7009-5p	NM_144544.2	-0.852272339	0.031123228
mmu-miR-7009-5p	XM_006515980.3	-0.950030027	0.00368311
mmu-miR-7009-5p	NM_153151.3	-0.915686945	0.010363359
mmu-miR-7009-5p	NM_001142804.1	-0.92591643	0.008029264
mmu-miR-7009-5p	XM_006498086.3	-0.913622389	0.010869402
mmu-miR-7009-5p	NM_021475.2	-0.811789511	0.049801274
mmu-miR-7009-5p	XM_006505388.2	-0.845770033	0.033845999
mmu-miR-7009-5p	NM_145635.2	-0.951419223	0.00348281
mmu-miR-7009-5p	NM_009605.4	-0.81922155	0.046067275
mmu-miR-7009-5p	NM_009645.2	-0.859276247	0.028311374
mmu-miR-7009-5p	NM_008012.1	-0.850129755	0.032008511
mmu-miR-7009-5p	NM_001303431.1	-0.914555581	0.010639219
mmu-miR-7009-5p	NM_009675.2	-0.873080431	0.023140619
mmu-miR-7009-5p	NM_001305585.1	-0.825712627	0.042917048
mmu-miR-7009-5p	NM_001110009.2	-0.912393678	0.011176118
mmu-miR-7009-5p	XM_006522398.3	-0.932868549	0.006608679
mmu-miR-7009-5p	NM_029823.2	-0.852776348	0.030916684
mmu-miR-7009-5p	NM_025711.3	-0.936636219	0.005895251
mmu-miR-7009-5p	NM_020025.4	-0.968691535	0.001454985
mmu-miR-7009-5p	NM_001159407.1	-0.814204224	0.048573264
mmu-miR-7009-5p	NM_001002896.2	-0.852995358	0.030827135
mmu-miR-7009-5p	NM_007549.2	-0.840590129	0.036091838
mmu-miR-7009-5p	NM_007555.4	-0.91297983	0.011029284
mmu-miR-7009-5p	NM_028472.2	-0.881579724	0.020204718
mmu-miR-7009-5p	NM_178692.3	-0.880142266	0.020687884
mmu-miR-7009-5p	XM_006503665.3	-0.875628226	0.022240595
mmu-miR-7009-5p	NM_001097617.1	-0.883220347	0.019659941
mmu-miR-7009-5p	NM_027416.3	-0.918854651	0.009609698
mmu-miR-7009-5p	NM_181315.4	-0.828569841	0.041563428
mmu-miR-7009-5p	NM_019626.3	-0.868157738	0.024927707
mmu-miR-7009-5p	NM_001001178.1	-0.843368847	0.034878636
mmu-miR-7009-5p	NM_028804.1	-0.873462823	0.023004451
mmu-miR-7009-5p	XM_006508867.2	-0.870524027	0.024060772
mmu-miR-7009-5p	XM_006508885.1	-0.878342576	0.021300496
mmu-miR-7009-5p	NM_001110322.1	-0.873296209	0.023063734
mmu-miR-7009-5p	XM_017312731.1	-0.96431526	0.001887381

mmu-miR-7009-5p	NM_013459.3	-0.932939581	0.006594861
mmu-miR-7009-5p	NM_001301295.1	-0.907612633	0.012408856
mmu-miR-7009-5p	XM_006513165.1	-0.859137277	0.028365939
mmu-miR-7009-5p	NM_020001.2	-0.886215319	0.018683848
mmu-miR-7009-5p	XM_017314944.1	-0.968623431	0.001461289
mmu-miR-7009-5p	NM_009930.2	-0.878357207	0.021295481
mmu-miR-7009-5p	NM_001309809.2	-0.820640439	0.045369793
mmu-miR-7009-5p	NM_018866.2	-0.818809186	0.046270909
mmu-miR-7009-5p	NM_030206.4	-0.896353674	0.015557128
mmu-miR-7009-5p	NM_007817.2	-0.912336836	0.011190407
mmu-miR-7009-5p	XM_006540596.2	-0.930400419	0.007097579
mmu-miR-7009-5p	NM_001190451.2	-0.910413496	0.011679113
mmu-miR-7009-5p	NM_001190448.1	-0.880351446	0.020617234
mmu-miR-7009-5p	NM_015814.2	-0.933119386	0.006559946
mmu-miR-7009-5p	NM_152915.1	-0.902570112	0.013776444
mmu-miR-7009-5p	NM_019759.2	-0.875179353	0.022397926
mmu-miR-7009-5p	NM_019397.3	-0.896628735	0.015476135
mmu-miR-7009-5p	NM_001291145.1	-0.859552233	0.028203156
mmu-miR-7009-5p	XM_006520618.2	-0.849523409	0.03226117
mmu-miR-7009-5p	NM_015744.4	-0.947742507	0.004024915
mmu-miR-7009-5p	NM_010140.3	-0.916114035	0.010260136
mmu-miR-7009-5p	NM_024406.2	-0.885186469	0.019016476
mmu-miR-7009-5p	XM_006509531.3	-0.898054317	0.015059627
mmu-miR-7009-5p	NM_177743.5	-0.95125258	0.003506547
mmu-miR-7009-5p	NM_178908.3	-0.898752511	0.014857634
mmu-miR-7009-5p	XM_006520437.2	-0.932343918	0.006711176
mmu-miR-7009-5p	NM_145141.2	-0.836801241	0.037777448
mmu-miR-7009-5p	NM_145741.2	-0.882596435	0.019866274
mmu-miR-7009-5p	XM_006510297.3	-0.942417598	0.004878136
mmu-miR-7009-5p	XM_006496277.3	-0.923540562	0.008545576
mmu-miR-7009-5p	XM_011246309.2	-0.84953317	0.032257096
mmu-miR-7009-5p	NM_175520.4	-0.815879381	0.047729722
mmu-miR-7009-5p	NM_010450.3	-0.971116696	0.00123932
mmu-miR-7009-5p	NM_023670.3	-0.841879998	0.03552625
mmu-miR-7009-5p	XM_006523711.3	-0.942427929	0.004876403
mmu-miR-7009-5p	NM_001159424.2	-0.880412564	0.020596613
mmu-miR-7009-5p	XM_017316439.1	-0.812976074	0.049196067
mmu-miR-7009-5p	XM_006496593.3	-0.844293213	0.034479381
mmu-miR-7009-5p	NM_001161541.1	-0.97153686	0.001203696
mmu-miR-7009-5p	NM_146125.2	-0.82354714	0.043956434
mmu-miR-7009-5p	NM_031180.2	-0.878664378	0.021190328
mmu-miR-7009-5p	NM_173427.2	-0.994412195	4.67481E-05
mmu-miR-7009-5p	NM_016659.3	-0.812257971	0.049561926
mmu-miR-7009-5p	NM_146063.1	-0.924558471	0.008322451
mmu-miR-7009-5p	XM_017315313.1	-0.882336937	0.019952396
mmu-miR-7009-5p	XM_006523061.3	-0.859295697	0.02830374
mmu-miR-7009-5p	XM_006511102.2	-0.996803483	1.53102E-05
mmu-miR-7009-5p	XM_006504999.1	-0.956946863	0.002740458
mmu-miR-7009-5p	XM_006527221.3	-0.910852357	0.011566712
mmu-miR-7009-5p	NM_010714.3	-0.840970913	0.035924433
mmu-miR-7009-5p	NM_028973.2	-0.866119711	0.025686067
mmu-miR-7009-5p	XM_006527321.2	-0.888752065	0.017875747
mmu-miR-7009-5p	NM_001290273.1	-0.852274729	0.031122247
mmu-miR-7009-5p	XM_006520023.2	-0.829456854	0.041147326

mmu-miR-7009-5p	NM_010779.2	-0.852127064	0.031182883
mmu-miR-7009-5p	NM_001291483.1	-0.858035937	0.028800135
mmu-miR-7009-5p	NM_010809.2	-0.861647089	0.027388149
mmu-miR-7009-5p	NM_029844.3	-0.870173043	0.024188439
mmu-miR-7009-5p	NM_007641.5	-0.832224981	0.039861382
mmu-miR-7009-5p	NM_010858.4	-0.82901096	0.041356253
mmu-miR-7009-5p	XM_006498901.3	-0.935197671	0.006162949
mmu-miR-7009-5p	XM_006510077.3	-0.887910334	0.018141987
mmu-miR-7009-5p	NM_023456.3	-0.943690573	0.004666856
mmu-miR-7009-5p	NM_008760.4	-0.930006808	0.00717712
mmu-miR-7009-5p	NM_172907.3	-0.859590903	0.028188009
mmu-miR-7009-5p	XM_017322026.1	-0.850961387	0.031663501
mmu-miR-7009-5p	NM_011044.2	-0.952113379	0.003384788
mmu-miR-7009-5p	NM_001290822.1	-0.855748863	0.029711769
mmu-miR-7009-5p	NM_001164593.1	-0.842695492	0.035170835
mmu-miR-7009-5p	NM_011082.3	-0.896400195	0.015543415
mmu-miR-7009-5p	NM_008872.3	-0.861824622	0.027319601
mmu-miR-7009-5p	XM_017312497.1	-0.891711829	0.016954581
mmu-miR-7009-5p	XM_011250776.2	-0.928076567	0.007573441
mmu-miR-7009-5p	NM_001030305.2	-0.822168942	0.044623973
mmu-miR-7009-5p	NM_011169.5	-0.954019259	0.003122736
mmu-miR-7009-5p	NM_001081224.2	-0.815650584	0.04784453
mmu-miR-7009-5p	NM_008966.3	-0.849451664	0.032291128
mmu-miR-7009-5p	XM_006506624.2	-0.879330158	0.020963267
mmu-miR-7009-5p	XM_017320735.1	-0.864462979	0.026310499
mmu-miR-7009-5p	NM_001204959.1	-0.914140852	0.010741222
mmu-miR-7009-5p	XM_006522506.3	-0.857957448	0.028831199
mmu-miR-7009-5p	XM_006525075.3	-0.887043368	0.018418183
mmu-miR-7009-5p	NM_009127.4	-0.933939701	0.006401802
mmu-miR-7009-5p	NM_008458.2	-0.854715822	0.030127946
mmu-miR-7009-5p	XM_011244046.2	-0.976955285	0.000790469
mmu-miR-7009-5p	XM_006521796.1	-0.940193989	0.005258183
mmu-miR-7009-5p	NM_144539.5	-0.812931144	0.049218921
mmu-miR-7009-5p	NM_001310705.1	-0.948169349	0.003960005
mmu-miR-7009-5p	NM_001290993.1	-0.839006513	0.036791967
mmu-miR-7009-5p	XM_017322365.1	-0.910904975	0.011553271
mmu-miR-7009-5p	NM_001146217.1	-0.934834479	0.006231454
mmu-miR-7009-5p	XM_006524777.3	-0.861226732	0.027550781
mmu-miR-7009-5p	NM_011430.3	-0.968812174	0.001443853
mmu-miR-7009-5p	NM_175692.3	-0.970313125	0.001308884
mmu-miR-7009-5p	NM_009223.3	-0.851193502	0.031567522
mmu-miR-7009-5p	NM_031183.2	-0.819306684	0.046025286
mmu-miR-7009-5p	NM_178753.4	-0.85853964	0.028601166
mmu-miR-7009-5p	NM_032400.2	-0.947488822	0.004063738
mmu-miR-7009-5p	NM_011581.3	-0.873969269	0.022824698
mmu-miR-7009-5p	NM_009381.3	-0.938347635	0.00558435
mmu-miR-7009-5p	XM_006537836.1	-0.813579887	0.048889409
mmu-miR-7009-5p	XM_006533452.3	-0.846467989	0.033548583
mmu-miR-7009-5p	NM_144936.1	-0.980110028	0.000589482
mmu-miR-7009-5p	NM_146241.2	-0.827695344	0.041975574
mmu-miR-7009-5p	XM_017321602.1	-0.834803511	0.038680724
mmu-miR-7009-5p	NM_177709.3	-0.844016805	0.034598541
mmu-miR-7009-5p	NM_011658.2	-0.921812767	0.008930876
mmu-miR-7009-5p	XM_011248394.1	-0.893427749	0.016431262

mmu-miR-7009-5p	XM_006525028.2	-0.929513402	0.007277439
mmu-miR-7009-5p	NM_016982.2	-0.812524478	0.049425999
mmu-miR-7009-5p	NM_177789.4	-0.962022365	0.002136064
mmu-miR-7009-5p	XM_017314347.1	-0.831569928	0.040163964
mmu-miR-7009-5p	XM_011243586.2	-0.876572562	0.021911331
mmu-miR-7042-5p	NM_024283.3	-0.94611912	0.004276512
mmu-miR-7042-5p	XM_011241948.2	-0.820519743	0.045428931
mmu-miR-7042-5p	XM_006515980.3	-0.852256125	0.031129883
mmu-miR-7042-5p	NM_153151.3	-0.884198527	0.019338524
mmu-miR-7042-5p	NM_001142804.1	-0.830595185	0.040616198
mmu-miR-7042-5p	XM_006498086.3	-0.828146105	0.041762896
mmu-miR-7042-5p	XM_006500919.3	-0.836610967	0.037863049
mmu-miR-7042-5p	NM_145635.2	-0.866009006	0.02572757
mmu-miR-7042-5p	NM_009605.4	-0.815863825	0.047737524
mmu-miR-7042-5p	NM_009645.2	-0.836466907	0.03792792
mmu-miR-7042-5p	NM_134072.1	-0.908071149	0.012287929
mmu-miR-7042-5p	NM_001303431.1	-0.833261848	0.039384622
mmu-miR-7042-5p	NM_009675.2	-0.821093868	0.045147946
mmu-miR-7042-5p	NM_001305585.1	-0.832084205	0.039926319
mmu-miR-7042-5p	NM_001110009.2	-0.899300321	0.014700069
mmu-miR-7042-5p	XM_006522398.3	-0.949594551	0.003747031
mmu-miR-7042-5p	NM_025711.3	-0.922831709	0.008702651
mmu-miR-7042-5p	NM_020025.4	-0.925598279	0.008097495
mmu-miR-7042-5p	NM_007529.2	-0.916141134	0.010253603
mmu-miR-7042-5p	NM_001002896.2	-0.823503335	0.043977579
mmu-miR-7042-5p	NM_007555.4	-0.895286691	0.015873231
mmu-miR-7042-5p	NM_028472.2	-0.816210631	0.047563731
mmu-miR-7042-5p	NM_178692.3	-0.856719096	0.029323389
mmu-miR-7042-5p	NM_001097617.1	-0.897298215	0.015279853
mmu-miR-7042-5p	NM_027416.3	-0.898157789	0.015029609
mmu-miR-7042-5p	NM_019626.3	-0.8341711	0.038968753
mmu-miR-7042-5p	NM_001001178.1	-0.842254679	0.035362745
mmu-miR-7042-5p	XM_006508867.2	-0.848605425	0.03264547
mmu-miR-7042-5p	XM_006508885.1	-0.890650258	0.017282282
mmu-miR-7042-5p	XM_017312731.1	-0.953445743	0.0032005
mmu-miR-7042-5p	XM_006513165.1	-0.843916639	0.034641771
mmu-miR-7042-5p	NM_020001.2	-0.81836895	0.046488768
mmu-miR-7042-5p	XM_017314944.1	-0.958307678	0.002571139
mmu-miR-7042-5p	NM_009930.2	-0.901576898	0.014053943
mmu-miR-7042-5p	NM_001309809.2	-0.954170696	0.003102359
mmu-miR-7042-5p	NM_007792.4	-0.84106591	0.035882726
mmu-miR-7042-5p	NM_018866.2	-0.85668409	0.02933736
mmu-miR-7042-5p	XM_006517308.3	-0.828214196	0.041730813
mmu-miR-7042-5p	NM_030206.4	-0.825425213	0.043054333
mmu-miR-7042-5p	NM_007817.2	-0.831687074	0.040109773
mmu-miR-7042-5p	XM_006540596.2	-0.896373554	0.015551267
mmu-miR-7042-5p	NM_001190451.2	-0.919449395	0.009471278
mmu-miR-7042-5p	NM_001190449.1	-0.847164799	0.03325289
mmu-miR-7042-5p	NM_015814.2	-0.90198524	0.013939531
mmu-miR-7042-5p	NM_001291145.1	-0.920026363	0.009337927
mmu-miR-7042-5p	NM_015744.4	-0.859784084	0.028112397
mmu-miR-7042-5p	NM_010140.3	-0.897985951	0.015079476
mmu-miR-7042-5p	NM_024406.2	-0.86338818	0.026719405
mmu-miR-7042-5p	NM_177743.5	-0.963697752	0.001952859

mmu-miR-7042-5p	XM_006520437.2	-0.953201774	0.003233865
mmu-miR-7042-5p	NM_145741.2	-0.853917349	0.030451499
mmu-miR-7042-5p	XM_006510297.3	-0.87663426	0.021889901
mmu-miR-7042-5p	NM_001318003.2	-0.858215746	0.028729033
mmu-miR-7042-5p	XM_001481172.6	-0.828475046	0.041608013
mmu-miR-7042-5p	NM_016697.3	-0.822446067	0.044489369
mmu-miR-7042-5p	XM_006496277.3	-0.905245985	0.013042119
mmu-miR-7042-5p	XM_011246309.2	-0.85823712	0.028720587
mmu-miR-7042-5p	NM_010450.3	-0.906022705	0.012832607
mmu-miR-7042-5p	NM_023670.3	-0.856513388	0.029405531
mmu-miR-7042-5p	XM_006523711.3	-0.867532566	0.025159187
mmu-miR-7042-5p	NM_001161541.1	-0.963322481	0.001993191
mmu-miR-7042-5p	NM_146125.2	-0.834864246	0.038653115
mmu-miR-7042-5p	NM_031180.2	-0.919619356	0.0094319
mmu-miR-7042-5p	NM_173427.2	-0.936071708	0.005999608
mmu-miR-7042-5p	NM_008940.3	-0.89283027	0.016612585
mmu-miR-7042-5p	NM_016659.3	-0.836068244	0.038107711
mmu-miR-7042-5p	NM_146063.1	-0.962893109	0.002039835
mmu-miR-7042-5p	XM_017315313.1	-0.817587015	0.046876896
mmu-miR-7042-5p	XM_006523061.3	-0.854152548	0.030356023
mmu-miR-7042-5p	XM_006511102.2	-0.926796777	0.00784193
mmu-miR-7042-5p	XM_006504999.1	-0.931057338	0.006965791
mmu-miR-7042-5p	NM_010714.3	-0.827204635	0.042207675
mmu-miR-7042-5p	NM_028973.2	-0.920199519	0.009298086
mmu-miR-7042-5p	NM_008524.2	-0.841394779	0.035738518
mmu-miR-7042-5p	XM_006520023.2	-0.882230916	0.019987633
mmu-miR-7042-5p	NM_001252563.1	-0.912000539	0.011275128
mmu-miR-7042-5p	NM_010779.2	-0.857682323	0.028940208
mmu-miR-7042-5p	NM_001291483.1	-0.85569694	0.029732621
mmu-miR-7042-5p	NM_029844.3	-0.855728533	0.029719933
mmu-miR-7042-5p	XM_006498901.3	-0.906712446	0.012647931
mmu-miR-7042-5p	XM_006510077.3	-0.908917529	0.012066214
mmu-miR-7042-5p	NM_023456.3	-0.853425063	0.030651795
mmu-miR-7042-5p	NM_008760.4	-0.943198753	0.004747941
mmu-miR-7042-5p	NM_172907.3	-0.862620469	0.027013312
mmu-miR-7042-5p	XM_006516928.2	-0.823139625	0.044153328
mmu-miR-7042-5p	XM_017322026.1	-0.844424828	0.034422709
mmu-miR-7042-5p	NM_001164593.1	-0.934169851	0.006357772
mmu-miR-7042-5p	NM_011082.3	-0.896953329	0.015380818
mmu-miR-7042-5p	XM_017312497.1	-0.918896821	0.009599851
mmu-miR-7042-5p	XM_011250776.2	-0.836312292	0.037997602
mmu-miR-7042-5p	NM_011169.5	-0.851700713	0.031358269
mmu-miR-7042-5p	NM_001081224.2	-0.949150454	0.003812774
mmu-miR-7042-5p	NM_008966.3	-0.867332073	0.025233639
mmu-miR-7042-5p	XM_006505757.3	-0.867888684	0.025027204
mmu-miR-7042-5p	XM_006525075.3	-0.835975283	0.038149692
mmu-miR-7042-5p	NM_009127.4	-0.860167839	0.027962479
mmu-miR-7042-5p	NM_009135.2	-0.81766256	0.046839332
mmu-miR-7042-5p	XM_011244046.2	-0.919202244	0.009528681
mmu-miR-7042-5p	XM_006521796.1	-0.967309302	0.001585555
mmu-miR-7042-5p	NM_177578.4	-0.821101995	0.045143974
mmu-miR-7042-5p	NM_001310705.1	-0.933773968	0.006433601
mmu-miR-7042-5p	NM_001146217.1	-0.86137534	0.027493234
mmu-miR-7042-5p	XM_006524777.3	-0.948237764	0.00394965

mmu-miR-7042-5p	NM_011430.3	-0.931633713	0.006851153
mmu-miR-7042-5p	NM_175692.3	-0.935876646	0.006035875
mmu-miR-7042-5p	NM_009223.3	-0.824019202	0.043728866
mmu-miR-7042-5p	NM_031183.2	-0.851549302	0.031420665
mmu-miR-7042-5p	NM_178753.4	-0.832265642	0.039842635
mmu-miR-7042-5p	NM_032400.2	-0.933414931	0.006502752
mmu-miR-7042-5p	NM_011581.3	-0.862192307	0.02717789
mmu-miR-7042-5p	NM_009381.3	-0.914562676	0.010637478
mmu-miR-7042-5p	NM_144936.1	-0.946696957	0.004186099
mmu-miR-7042-5p	XM_006496905.1	-0.881297489	0.020299153
mmu-miR-7042-5p	XM_017321602.1	-0.876427744	0.021961673
mmu-miR-7042-5p	NM_011658.2	-0.898474021	0.014938046
mmu-miR-7042-5p	XM_011248394.1	-0.887689696	0.018212088
mmu-miR-7042-5p	XM_006525028.2	-0.913362919	0.010933827
mmu-miR-7042-5p	NM_016982.2	-0.861576323	0.027415495
mmu-miR-7042-5p	NM_177789.4	-0.865250703	0.026012713
mmu-miR-7042-5p	XM_017318541.1	-0.913644792	0.010863848
mmu-miR-7042-5p	XM_011243586.2	-0.986145766	0.00028658
mmu-miR-7219-5p	NM_024283.3	-0.978795721	0.000669665
mmu-miR-7219-5p	XM_006515980.3	-0.937615887	0.005716274
mmu-miR-7219-5p	NM_153151.3	-0.962993561	0.002028875
mmu-miR-7219-5p	NM_001142804.1	-0.920546905	0.009218406
mmu-miR-7219-5p	XM_006498086.3	-0.938242152	0.005603275
mmu-miR-7219-5p	XM_006505388.2	-0.848261831	0.032789862
mmu-miR-7219-5p	NM_145635.2	-0.882458473	0.019912038
mmu-miR-7219-5p	NM_001291930.1	-0.850523729	0.031844848
mmu-miR-7219-5p	NM_008012.1	-0.817251659	0.047043815
mmu-miR-7219-5p	NM_001303431.1	-0.922489171	0.008779053
mmu-miR-7219-5p	NM_009675.2	-0.901164194	0.014170035
mmu-miR-7219-5p	NM_001110009.2	-0.871698302	0.023635981
mmu-miR-7219-5p	XM_006522398.3	-0.913500668	0.010899602
mmu-miR-7219-5p	NM_025711.3	-0.960026719	0.002364859
mmu-miR-7219-5p	NM_020025.4	-0.966398337	0.001674638
mmu-miR-7219-5p	NM_001159407.1	-0.864506628	0.026293956
mmu-miR-7219-5p	NM_007529.2	-0.819485051	0.045937372
mmu-miR-7219-5p	NM_001002896.2	-0.82216267	0.044627022
mmu-miR-7219-5p	NM_007549.2	-0.828395377	0.041645501
mmu-miR-7219-5p	NM_007555.4	-0.905602952	0.012945627
mmu-miR-7219-5p	NM_028472.2	-0.840722345	0.03603367
mmu-miR-7219-5p	NM_178692.3	-0.890586431	0.017302081
mmu-miR-7219-5p	NM_026979.5	-0.846987985	0.033327805
mmu-miR-7219-5p	XM_006503665.3	-0.874965717	0.022472992
mmu-miR-7219-5p	NM_001097617.1	-0.876343386	0.021991023
mmu-miR-7219-5p	NM_027416.3	-0.87156814	0.023682889
mmu-miR-7219-5p	NM_019626.3	-0.855890735	0.029654827
mmu-miR-7219-5p	NM_028804.1	-0.892860724	0.01660332
mmu-miR-7219-5p	XM_006508885.1	-0.82628464	0.042644433
mmu-miR-7219-5p	NM_001110322.1	-0.843623715	0.034768337
mmu-miR-7219-5p	XM_017312731.1	-0.932369702	0.00670612
mmu-miR-7219-5p	NM_013459.3	-0.892577531	0.016689575
mmu-miR-7219-5p	NM_001301295.1	-0.869408827	0.024467527
mmu-miR-7219-5p	NM_020001.2	-0.82284909	0.044293953
mmu-miR-7219-5p	XM_017314944.1	-0.980441254	0.000570076
mmu-miR-7219-5p	NM_009930.2	-0.868360194	0.024852962

mmu-miR-7219-5p	NM_001309809.2	-0.866409534	0.025577564
mmu-miR-7219-5p	NM_018866.2	-0.8656093	0.025877684
mmu-miR-7219-5p	NM_030206.4	-0.891713142	0.016954177
mmu-miR-7219-5p	NM_007817.2	-0.912059586	0.01126023
mmu-miR-7219-5p	XM_006540596.2	-0.914124039	0.010745367
mmu-miR-7219-5p	NM_001190451.2	-0.943097492	0.004764721
mmu-miR-7219-5p	NM_001190448.1	-0.90217078	0.013887694
mmu-miR-7219-5p	NM_015814.2	-0.938758281	0.005510977
mmu-miR-7219-5p	NM_152915.1	-0.823993437	0.043741273
mmu-miR-7219-5p	NM_019759.2	-0.837661327	0.037391643
mmu-miR-7219-5p	NM_019397.3	-0.861140474	0.027584209
mmu-miR-7219-5p	NM_001291145.1	-0.841352133	0.035757203
mmu-miR-7219-5p	XM_006520618.2	-0.856213312	0.02952555
mmu-miR-7219-5p	NM_015744.4	-0.910684473	0.011609648
mmu-miR-7219-5p	NM_010140.3	-0.95221268	0.003370878
mmu-miR-7219-5p	NM_024406.2	-0.850995435	0.031649414
mmu-miR-7219-5p	XM_006509531.3	-0.858083565	0.028781294
mmu-miR-7219-5p	NM_177743.5	-0.968814523	0.001443637
mmu-miR-7219-5p	NM_178908.3	-0.846758735	0.033425054
mmu-miR-7219-5p	XM_006520437.2	-0.959599817	0.002415292
mmu-miR-7219-5p	XM_006496949.3	-0.817524088	0.046908196
mmu-miR-7219-5p	NM_145741.2	-0.913546189	0.010888303
mmu-miR-7219-5p	XM_006510297.3	-0.941209981	0.005082803
mmu-miR-7219-5p	XM_006496277.3	-0.908291761	0.01222995
mmu-miR-7219-5p	XM_011246309.2	-0.906917825	0.012593191
mmu-miR-7219-5p	NM_010450.3	-0.965123739	0.001803319
mmu-miR-7219-5p	NM_023670.3	-0.833570528	0.039243208
mmu-miR-7219-5p	XM_006523711.3	-0.974343	0.000978978
mmu-miR-7219-5p	NM_001159424.2	-0.879291373	0.020976462
mmu-miR-7219-5p	XM_017316439.1	-0.82522418	0.043150479
mmu-miR-7219-5p	XM_006496593.3	-0.872637878	0.023298688
mmu-miR-7219-5p	NM_001161541.1	-0.993897916	5.57395E-05
mmu-miR-7219-5p	NM_010608.2	-0.870162512	0.024192275
mmu-miR-7219-5p	NM_031180.2	-0.886460126	0.018605116
mmu-miR-7219-5p	NM_173427.2	-0.96531757	0.001783447
mmu-miR-7219-5p	NM_146063.1	-0.903548146	0.013505796
mmu-miR-7219-5p	XM_017315313.1	-0.901138224	0.014177356
mmu-miR-7219-5p	XM_006523061.3	-0.922836105	0.008701673
mmu-miR-7219-5p	XM_006511102.2	-0.968521327	0.001470764
mmu-miR-7219-5p	XM_006504999.1	-0.90833565	0.012218432
mmu-miR-7219-5p	XM_006527221.3	-0.922869582	0.008694224
mmu-miR-7219-5p	NM_010714.3	-0.86912375	0.024572026
mmu-miR-7219-5p	NM_028973.2	-0.868479328	0.024809029
mmu-miR-7219-5p	XM_006527321.2	-0.891429984	0.017041291
mmu-miR-7219-5p	NM_001290273.1	-0.823971841	0.043751672
mmu-miR-7219-5p	NM_001252563.1	-0.814484269	0.048431767
mmu-miR-7219-5p	NM_010779.2	-0.929594328	0.007260939
mmu-miR-7219-5p	NM_001291483.1	-0.812450243	0.049463844
mmu-miR-7219-5p	XM_006504940.3	-0.847273744	0.03320677
mmu-miR-7219-5p	NM_010809.2	-0.873566046	0.022967759
mmu-miR-7219-5p	NM_029844.3	-0.841452513	0.03571323
mmu-miR-7219-5p	NM_205810.4	-0.814709188	0.048318262
mmu-miR-7219-5p	NM_007641.5	-0.820123471	0.045623345
mmu-miR-7219-5p	XM_006529456.3	-0.812374248	0.0495026

mmu-miR-7219-5p	XM_006498901.3	-0.87749386	0.02159236
mmu-miR-7219-5p	XM_006510077.3	-0.864796047	0.026184392
mmu-miR-7219-5p	NM_023456.3	-0.946779736	0.004173224
mmu-miR-7219-5p	NM_008760.4	-0.971999696	0.001165049
mmu-miR-7219-5p	XM_017322026.1	-0.816310246	0.047513866
mmu-miR-7219-5p	NM_011044.2	-0.909760316	0.011847381
mmu-miR-7219-5p	NM_001290822.1	-0.835270592	0.038468637
mmu-miR-7219-5p	NM_001164593.1	-0.881587331	0.020202175
mmu-miR-7219-5p	NM_011082.3	-0.889110699	0.017762884
mmu-miR-7219-5p	NM_008872.3	-0.882555054	0.019879996
mmu-miR-7219-5p	XM_017312497.1	-0.895334928	0.015858875
mmu-miR-7219-5p	XM_011250776.2	-0.915996692	0.010288447
mmu-miR-7219-5p	NM_001030305.2	-0.858172814	0.028746003
mmu-miR-7219-5p	NM_011169.5	-0.923084982	0.008646368
mmu-miR-7219-5p	NM_001081224.2	-0.852757044	0.030924582
mmu-miR-7219-5p	NM_008966.3	-0.824204424	0.043639726
mmu-miR-7219-5p	XM_006506624.2	-0.825417083	0.043058219
mmu-miR-7219-5p	XM_017320735.1	-0.83396247	0.039063993
mmu-miR-7219-5p	NM_001204959.1	-0.845568172	0.033932247
mmu-miR-7219-5p	XM_006525075.3	-0.86887934	0.024661787
mmu-miR-7219-5p	NM_023893.4	-0.811409177	0.04999599
mmu-miR-7219-5p	NM_009127.4	-0.930776239	0.007022036
mmu-miR-7219-5p	NM_009135.2	-0.871035079	0.023875457
mmu-miR-7219-5p	XM_011244046.2	-0.963474033	0.001976854
mmu-miR-7219-5p	XM_006521796.1	-0.916839118	0.010086039
mmu-miR-7219-5p	NM_001310705.1	-0.936515853	0.005917427
mmu-miR-7219-5p	NM_001290993.1	-0.855727619	0.0297203
mmu-miR-7219-5p	XM_017322365.1	-0.923853912	0.008476584
mmu-miR-7219-5p	NM_001146217.1	-0.908292027	0.01222988
mmu-miR-7219-5p	XM_006524777.3	-0.901949334	0.013949573
mmu-miR-7219-5p	NM_011430.3	-0.929319481	0.007317053
mmu-miR-7219-5p	NM_175692.3	-0.952883961	0.003277585
mmu-miR-7219-5p	NM_009223.3	-0.846272124	0.033631921
mmu-miR-7219-5p	NM_031183.2	-0.853809775	0.030495215
mmu-miR-7219-5p	NM_178753.4	-0.895099257	0.015929076
mmu-miR-7219-5p	NM_032400.2	-0.966899845	0.001625298
mmu-miR-7219-5p	NM_011581.3	-0.866332681	0.025606314
mmu-miR-7219-5p	NM_009381.3	-0.924864941	0.008255837
mmu-miR-7219-5p	XM_006537836.1	-0.856216529	0.029524263
mmu-miR-7219-5p	XM_006533452.3	-0.857576417	0.028982222
mmu-miR-7219-5p	NM_144936.1	-0.945918727	0.004308088
mmu-miR-7219-5p	XM_006521428.3	-0.833214161	0.03940649
mmu-miR-7219-5p	NM_177371.3	-0.87692077	0.021790513
mmu-miR-7219-5p	XM_006496905.1	-0.823340247	0.044056345
mmu-miR-7219-5p	NM_146241.2	-0.840511229	0.036126571
mmu-miR-7219-5p	XM_017321602.1	-0.871685484	0.023640598
mmu-miR-7219-5p	NM_177709.3	-0.844302064	0.034475568
mmu-miR-7219-5p	NM_011658.2	-0.91401597	0.010772029
mmu-miR-7219-5p	XM_011248394.1	-0.90506043	0.013092413
mmu-miR-7219-5p	XM_006525028.2	-0.922597846	0.008754778
mmu-miR-7219-5p	NM_177789.4	-0.899985754	0.01450406
mmu-miR-7219-5p	XM_011243586.2	-0.910294462	0.011709691
mmu-miR-7221-3p	XM_017320219.1	-0.960748881	0.002280739
mmu-miR-7221-3p	NM_029685.1	-0.85895068	0.028439284

mmu-miR-7221-3p	XM_011241322.1	-0.905684127	0.012923733
mmu-miR-7221-3p	NM_138955.3	-0.968928893	0.001433122
mmu-miR-7221-3p	NM_008032.3	-0.932573216	0.006666283
mmu-miR-7221-3p	NM_153178.4	-0.845463334	0.033977082
mmu-miR-7221-3p	XM_011250176.1	-0.83192934	0.039997811
mmu-miR-7221-3p	XM_011240149.1	-0.85668898	0.029335408
mmu-miR-7221-3p	XM_006508990.2	-0.937111036	0.005808169
mmu-miR-7221-3p	NM_013468.3	-0.969794793	0.001354753
mmu-miR-7221-3p	NM_007881.4	-0.825331755	0.043099017
mmu-miR-7221-3p	XM_006529727.2	-0.914547179	0.010641281
mmu-miR-7221-3p	XM_006507725.2	-0.844424106	0.03442302
mmu-miR-7221-3p	XM_006502291.3	-0.835574254	0.038331046
mmu-miR-7221-3p	XM_006510701.3	-0.823006055	0.044217953
mmu-miR-7221-3p	NM_178782.4	-0.832529281	0.039721184
mmu-miR-7221-3p	XM_006524328.3	-0.930718392	0.007033638
mmu-miR-7221-3p	NM_001013784.1	-0.976591448	0.000815527
mmu-miR-7221-3p	XM_006509966.3	-0.87792085	0.021445286
mmu-miR-7221-3p	XM_006503371.2	-0.843610823	0.034773912
mmu-miR-7221-3p	XM_006523030.3	-0.821493258	0.044952957
mmu-miR-7221-3p	XM_006530131.1	-0.900600632	0.014329307
mmu-miR-7221-3p	XM_011250217.1	-0.82696504	0.042321218
mmu-miR-7221-3p	XM_006527480.3	-0.846389572	0.033581937
mmu-miR-7221-3p	XM_011247208.1	-0.828986325	0.04136781
mmu-miR-7221-3p	XM_011248602.2	-0.944536986	0.004528913
mmu-miR-7221-3p	NM_011957.2	-0.813240245	0.049061793
mmu-miR-7221-3p	XM_006506808.3	-0.838518407	0.037009036
mmu-miR-7221-3p	NM_001317365.1	-0.870263502	0.024155504
mmu-miR-7221-3p	XM_006528026.2	-0.843390581	0.034869224
mmu-miR-7221-3p	XM_006511221.3	-0.947545356	0.00405507
mmu-miR-7221-3p	XM_006524407.3	-0.886653045	0.018543184
mmu-miR-7221-3p	XM_006532962.2	-0.864552093	0.02627673
mmu-miR-7221-3p	NM_010181.2	-0.883790215	0.019472381
mmu-miR-7221-3p	NM_001081185.1	-0.818570187	0.046389123
mmu-miR-7221-3p	NM_001033445.2	-0.872766352	0.023252747
mmu-miR-7221-3p	XM_006514444.1	-0.958183791	0.002586333
mmu-miR-7221-3p	XM_006530353.3	-0.894338825	0.01615661
mmu-miR-7221-3p	XM_011248576.2	-0.844727072	0.034292733
mmu-miR-7221-3p	NM_001198955.1	-0.894441339	0.016125846
mmu-miR-7221-3p	XM_006532232.3	-0.86825635	0.024891287
mmu-miR-7221-3p	NM_010340.2	-0.953855449	0.003144851
mmu-miR-7221-3p	XM_006515096.2	-0.831453869	0.040217685
mmu-miR-7221-3p	NM_001162950.1	-0.961543085	0.002189964
mmu-miR-7221-3p	XM_006505602.3	-0.822193802	0.044611891
mmu-miR-7221-3p	NM_013560.2	-0.870070757	0.024225705
mmu-miR-7221-3p	XM_006516224.3	-0.877863047	0.021465168
mmu-miR-7221-3p	XM_006528842.1	-0.872085834	0.023496583
mmu-miR-7221-3p	NM_001302471.1	-0.850559396	0.03183005
mmu-miR-7221-3p	NM_001289875.1	-0.940020051	0.0052885
mmu-miR-7221-3p	NM_008423.2	-0.939615884	0.005359275
mmu-miR-7221-3p	XM_017315734.1	-0.905725446	0.012912596
mmu-miR-7221-3p	XM_011248919.2	-0.841477197	0.035702421
mmu-miR-7221-3p	XM_006512296.3	-0.813561042	0.048898967
mmu-miR-7221-3p	NM_029274.2	-0.819153671	0.046100767
mmu-miR-7221-3p	NM_144945.3	-0.969248849	0.00140391

mmu-miR-7221-3p	NM_144799.2	-0.832094387	0.03992162
mmu-miR-7221-3p	XM_011243266.1	-0.845106246	0.034130001
mmu-miR-7221-3p	NM_001270475.1	-0.876219666	0.022034102
mmu-miR-7221-3p	XM_006529753.2	-0.92927746	0.007325651
mmu-miR-7221-3p	NM_175260.2	-0.828371823	0.041656587
mmu-miR-7221-3p	NM_173437.2	-0.827281244	0.0421714
mmu-miR-7221-3p	XM_006541297.3	-0.910222116	0.011728295
mmu-miR-7221-3p	NM_001134300.2	-0.901088361	0.014191417
mmu-miR-7221-3p	NM_001029877.3	-0.958576429	0.002538329
mmu-miR-7221-3p	NM_001077403.1	-0.865293928	0.025996419
mmu-miR-7221-3p	NM_133167.3	-0.891979733	0.016872357
mmu-miR-7221-3p	NM_013743.2	-0.881938764	0.020084888
mmu-miR-7221-3p	XM_006538456.3	-0.852493018	0.031032713
mmu-miR-7221-3p	XM_006537815.3	-0.873383573	0.02303264
mmu-miR-7221-3p	XM_017317946.1	-0.847205053	0.033235846
mmu-miR-7221-3p	NM_148932.2	-0.83995867	0.036370254
mmu-miR-7221-3p	XM_006527008.2	-0.825432086	0.043051048
mmu-miR-7221-3p	XM_011239558.2	-0.923719773	0.008506085
mmu-miR-7221-3p	XM_006514077.1	-0.850171708	0.031991064
mmu-miR-7221-3p	NM_019732.2	-0.856497509	0.029411876
mmu-miR-7221-3p	NM_018732.3	-0.859461156	0.028238847
mmu-miR-7221-3p	XM_006530238.3	-0.872264977	0.023432277
mmu-miR-7221-3p	XM_017316715.1	-0.839986412	0.036358001
mmu-miR-7221-3p	XM_017317034.1	-0.937682422	0.005704216
mmu-miR-7221-3p	NM_001033167.3	-0.942913732	0.004795245
mmu-miR-7221-3p	NM_001007570.2	-0.833510173	0.039270839
mmu-miR-7221-3p	NM_001033286.2	-0.900867532	0.01425377
mmu-miR-7221-3p	XM_006503080.3	-0.859602907	0.028183308
mmu-miR-7221-3p	XM_006506145.1	-0.814505699	0.048420947
mmu-miR-7221-3p	XM_011248193.2	-0.829716639	0.04102583
mmu-miR-7221-3p	XM_011245529.2	-0.818059256	0.046642311
mmu-miR-7221-3p	NM_009332.3	-0.876890822	0.021800891
mmu-miR-7221-3p	NM_153801.3	-0.938397746	0.005575371
mmu-miR-7221-3p	NM_001161746.1	-0.813218993	0.049072589
mmu-miR-7221-3p	XM_006538830.1	-0.920581772	0.009210427
mmu-miR-7221-3p	XM_011248201.2	-0.867646019	0.025117104
mmu-miR-7221-3p	NM_177409.3	-0.850701769	0.031771017
mmu-miR-7221-3p	NM_001039047.1	-0.915621851	0.010379136
mmu-miR-7221-3p	NM_001033149.3	-0.829203874	0.041265801
mmu-miR-7221-3p	NM_001244031.1	-0.813849985	0.048752523
mmu-miR-7221-3p	NM_011724.3	-0.889174444	0.017742859
mmu-miR-7221-3p	XM_006499466.3	-0.94999077	0.00368885
mmu-miR-7221-3p	XM_006530585.3	-0.866783887	0.025437737
mmu-miR-7221-3p	NM_199029.2	-0.851574257	0.031410377
mmu-miR-7221-3p	XM_006511007.3	-0.875820957	0.022173205
mmu-miR-7226-5p	NM_024283.3	-0.841751106	0.035582578
mmu-miR-7226-5p	XM_006500919.3	-0.913677919	0.010855638
mmu-miR-7226-5p	NM_134072.1	-0.92274066	0.008722928
mmu-miR-7226-5p	NM_001110009.2	-0.831812296	0.040051884
mmu-miR-7226-5p	XM_006522398.3	-0.912885981	0.011052731
mmu-miR-7226-5p	NM_025711.3	-0.887867779	0.018155498
mmu-miR-7226-5p	NM_007529.2	-0.934934313	0.006212586
mmu-miR-7226-5p	NM_007555.4	-0.832133603	0.039903527
mmu-miR-7226-5p	NM_001097617.1	-0.873911785	0.022845067

mmu-miR-7226-5p	NM_027416.3	-0.85184903	0.031297204
mmu-miR-7226-5p	NM_019626.3	-0.826256599	0.042657778
mmu-miR-7226-5p	NM_001001178.1	-0.847646321	0.033049276
mmu-miR-7226-5p	XM_006508885.1	-0.84528848	0.034051921
mmu-miR-7226-5p	XM_017312731.1	-0.88451512	0.019235039
mmu-miR-7226-5p	XM_017314944.1	-0.856785571	0.029296868
mmu-miR-7226-5p	NM_009930.2	-0.90364719	0.013478533
mmu-miR-7226-5p	NM_001309809.2	-0.97330423	0.001059484
mmu-miR-7226-5p	NM_019696.2	-0.813876479	0.048739106
mmu-miR-7226-5p	NM_007792.4	-0.858997846	0.028420736
mmu-miR-7226-5p	NM_018866.2	-0.906792388	0.01262661
mmu-miR-7226-5p	XM_006517308.3	-0.822626033	0.044402058
mmu-miR-7226-5p	NM_001190451.2	-0.886221921	0.018681723
mmu-miR-7226-5p	NM_001190449.1	-0.85859437	0.028579587
mmu-miR-7226-5p	NM_015814.2	-0.847858709	0.032959653
mmu-miR-7226-5p	NM_009345.2	-0.863443908	0.02669813
mmu-miR-7226-5p	NM_001291145.1	-0.855375561	0.029861844
mmu-miR-7226-5p	NM_010140.3	-0.823717029	0.043874471
mmu-miR-7226-5p	NM_177743.5	-0.924506132	0.008333854
mmu-miR-7226-5p	XM_006520437.2	-0.884305047	0.019303676
mmu-miR-7226-5p	NM_001318003.2	-0.845548281	0.033940751
mmu-miR-7226-5p	XM_001481172.6	-0.902297454	0.013852357
mmu-miR-7226-5p	XM_006496277.3	-0.82719158	0.042213858
mmu-miR-7226-5p	NM_010450.3	-0.833428877	0.039308072
mmu-miR-7226-5p	NM_008264.1	-0.911166216	0.011486648
mmu-miR-7226-5p	NM_023670.3	-0.841592821	0.035651809
mmu-miR-7226-5p	NM_001161541.1	-0.899970144	0.01450851
mmu-miR-7226-5p	NM_031180.2	-0.894011386	0.016255063
mmu-miR-7226-5p	NM_173427.2	-0.814717734	0.048313952
mmu-miR-7226-5p	NM_008940.3	-0.893310869	0.016466658
mmu-miR-7226-5p	NM_016659.3	-0.843487106	0.034827437
mmu-miR-7226-5p	NM_146063.1	-0.865288031	0.025998642
mmu-miR-7226-5p	XM_006523061.3	-0.856612253	0.02936604
mmu-miR-7226-5p	XM_006504999.1	-0.823985931	0.043744887
mmu-miR-7226-5p	NM_010714.3	-0.832392486	0.03978418
mmu-miR-7226-5p	NM_028973.2	-0.951293055	0.003500774
mmu-miR-7226-5p	NM_008524.2	-0.894165216	0.016208774
mmu-miR-7226-5p	XM_006520023.2	-0.893569798	0.016388294
mmu-miR-7226-5p	NM_001252563.1	-0.968517876	0.001471085
mmu-miR-7226-5p	NM_010779.2	-0.832036699	0.039948243
mmu-miR-7226-5p	NM_001291483.1	-0.826019307	0.042770787
mmu-miR-7226-5p	NM_029844.3	-0.812628948	0.049372764
mmu-miR-7226-5p	XM_006510077.3	-0.889833904	0.017536334
mmu-miR-7226-5p	NM_008760.4	-0.906976282	0.012577632
mmu-miR-7226-5p	NM_172907.3	-0.868049164	0.024967835
mmu-miR-7226-5p	XM_006516928.2	-0.872875362	0.0232138
mmu-miR-7226-5p	XM_017322026.1	-0.812617417	0.049378638
mmu-miR-7226-5p	NM_001164593.1	-0.961030482	0.002248345
mmu-miR-7226-5p	XM_017312497.1	-0.928445262	0.007496938
mmu-miR-7226-5p	NM_001081224.2	-0.938708867	0.005519781
mmu-miR-7226-5p	NM_008966.3	-0.88252631	0.019889529
mmu-miR-7226-5p	XM_006505757.3	-0.938650797	0.005530136
mmu-miR-7226-5p	NM_198024.2	-0.904083084	0.013358862
mmu-miR-7226-5p	XM_011244046.2	-0.828626861	0.041536621

mmu-miR-7226-5p	XM_006521796.1	-0.862675012	0.026992381
mmu-miR-7226-5p	NM_177578.4	-0.852998701	0.030825769
mmu-miR-7226-5p	NM_001310705.1	-0.887221403	0.018361302
mmu-miR-7226-5p	XM_006524777.3	-0.930803793	0.007016513
mmu-miR-7226-5p	NM_011430.3	-0.837448695	0.037486849
mmu-miR-7226-5p	NM_001204201.1	-0.883827471	0.019460149
mmu-miR-7226-5p	NM_011581.3	-0.852268837	0.031124666
mmu-miR-7226-5p	NM_009381.3	-0.842918736	0.035073832
mmu-miR-7226-5p	NM_178715.3	-0.818986851	0.046183124
mmu-miR-7226-5p	NM_144936.1	-0.820746951	0.045317635
mmu-miR-7226-5p	NM_177371.3	-0.824577409	0.043460481
mmu-miR-7226-5p	XM_006496905.1	-0.939128546	0.005445226
mmu-miR-7226-5p	XM_011249157.2	-0.838907899	0.036835774
mmu-miR-7226-5p	XM_017321602.1	-0.887536764	0.018260753
mmu-miR-7226-5p	NM_011658.2	-0.867666478	0.025109519
mmu-miR-7226-5p	XM_006525028.2	-0.829522368	0.041116671
mmu-miR-7226-5p	NM_016982.2	-0.851876608	0.031285856
mmu-miR-7226-5p	XM_017316944.1	-0.880057495	0.020716548
mmu-miR-7226-5p	XM_017318541.1	-0.945359947	0.004396738
mmu-miR-7226-5p	XM_011243586.2	-0.979706586	0.000613555
mmu-miR-3473f	XM_011241322.1	-0.815570732	0.04788463
mmu-miR-3473f	NM_029982.1	-0.82945418	0.041148578
mmu-miR-3473f	XM_006540566.1	-0.872899944	0.023205022
mmu-miR-3473f	XM_011250176.1	-0.825050056	0.043233836
mmu-miR-3473f	XM_006498287.3	-0.983814677	0.000390827
mmu-miR-3473f	NM_025758.4	-0.904122412	0.01334809
mmu-miR-3473f	NM_001290469.1	-0.815780749	0.047779199
mmu-miR-3473f	NM_007588.2	-0.906485138	0.012708649
mmu-miR-3473f	XM_006526449.3	-0.906021915	0.012832819
mmu-miR-3473f	XM_006533653.3	-0.95850184	0.002547414
mmu-miR-3473f	NM_007739.2	-0.944435704	0.004545312
mmu-miR-3473f	NM_011957.2	-0.873229037	0.023087655
mmu-miR-3473f	NM_010055.3	-0.984437638	0.000361396
mmu-miR-3473f	NM_001317365.1	-0.92496044	0.008235132
mmu-miR-3473f	NM_178676.4	-0.930508075	0.007075899
mmu-miR-3473f	XM_006532962.2	-0.812845642	0.049262426
mmu-miR-3473f	NM_001033221.3	-0.830251905	0.040776028
mmu-miR-3473f	XM_006521554.3	-0.966341971	0.001680229
mmu-miR-3473f	XM_006515096.2	-0.898286361	0.014992349
mmu-miR-3473f	XM_006499444.3	-0.962561778	0.002076194
mmu-miR-3473f	NM_133871.2	-0.95042898	0.003625024
mmu-miR-3473f	NM_001302471.1	-0.834058503	0.03902014
mmu-miR-3473f	NM_008479.2	-0.855305132	0.029890198
mmu-miR-3473f	XM_006506340.3	-0.870956221	0.023904008
mmu-miR-3473f	NM_001099314.1	-0.93045619	0.007086343
mmu-miR-3473f	NM_175260.2	-0.861383105	0.027490228
mmu-miR-3473f	NM_001077403.1	-0.863087906	0.026834179
mmu-miR-3473f	NM_145210.2	-0.878622045	0.021204805
mmu-miR-3473f	XM_006530325.3	-0.843654182	0.034755163
mmu-miR-3473f	NM_145226.2	-0.945944717	0.004303986
mmu-miR-3473f	XM_006530294.3	-0.966013884	0.001712956
mmu-miR-3473f	NM_011854.2	-0.915883282	0.010315844
mmu-miR-3473f	NM_001286743.1	-0.969599243	0.001372261
mmu-miR-3473f	NM_172454.2	-0.859674017	0.028155466

mmu-miR-3473f	NM_028748.2	-0.853407616	0.030658905
mmu-miR-3473f	XM_006537815.3	-0.872407243	0.023381268
mmu-miR-3473f	XM_006502235.3	-0.860854544	0.027695158
mmu-miR-3473f	NM_011145.3	-0.947705857	0.004030512
mmu-miR-3473f	XM_011239558.2	-0.846253977	0.033639647
mmu-miR-3473f	NM_175563.5	-0.880580482	0.02054001
mmu-miR-3473f	XM_006524382.3	-0.972118927	0.001155195
mmu-miR-3473f	XM_006505015.3	-0.850254565	0.03195662
mmu-miR-3473f	NM_009246.3	-0.860631378	0.027781898
mmu-miR-3473f	XM_006506145.1	-0.816032075	0.047653173
mmu-miR-3473f	XM_011245529.2	-0.827664754	0.041990026
mmu-miR-3473f	XM_006538830.1	-0.866378182	0.025589291
mmu-miR-3473f	XM_006501309.3	-0.952996412	0.003262082
mmu-miR-3473f	NM_009504.4	-0.85057331	0.031824279
mmu-miR-3473f	XM_006533569.3	-0.813388518	0.048986503
mmu-miR-8094	XM_006515980.3	-0.819506867	0.045926625
mmu-miR-8094	XM_006500919.3	-0.942595481	0.004848336
mmu-miR-8094	NM_008012.1	-0.811719095	0.049837298
mmu-miR-8094	XM_006495613.3	-0.864853784	0.026162561
mmu-miR-8094	NM_001303431.1	-0.9267245	0.007857229
mmu-miR-8094	XM_006522398.3	-0.825828571	0.042861725
mmu-miR-8094	NM_025711.3	-0.900942415	0.014232611
mmu-miR-8094	NM_001159407.1	-0.829865559	0.04095626
mmu-miR-8094	NM_001097617.1	-0.827952766	0.041854056
mmu-miR-8094	NM_019626.3	-0.896156984	0.015615169
mmu-miR-8094	NM_001001178.1	-0.83068653	0.040573718
mmu-miR-8094	XM_011248983.2	-0.823186041	0.044130881
mmu-miR-8094	NM_009930.2	-0.879155422	0.021022746
mmu-miR-8094	NM_018866.2	-0.981582117	0.000505704
mmu-miR-8094	NM_001190451.2	-0.866192692	0.025658724
mmu-miR-8094	NM_015814.2	-0.84872722	0.032594359
mmu-miR-8094	NM_177743.5	-0.87286229	0.023218469
mmu-miR-8094	XM_001481172.6	-0.852567844	0.03100205
mmu-miR-8094	NM_010450.3	-0.85754883	0.02899317
mmu-miR-8094	NM_008264.1	-0.919898322	0.009367441
mmu-miR-8094	XM_006523711.3	-0.840993274	0.035914614
mmu-miR-8094	NM_001161541.1	-0.850000411	0.032062329
mmu-miR-8094	NM_010608.2	-0.825058029	0.043230017
mmu-miR-8094	NM_016659.3	-0.821505018	0.044947222
mmu-miR-8094	XM_017315313.1	-0.859613677	0.02817909
mmu-miR-8094	XM_006523061.3	-0.946005342	0.004294426
mmu-miR-8094	XM_006527221.3	-0.839910018	0.036391746
mmu-miR-8094	NM_010714.3	-0.91977968	0.009394829
mmu-miR-8094	NM_175271.4	-0.876475351	0.021945118
mmu-miR-8094	NM_028973.2	-0.923487052	0.008557385
mmu-miR-8094	XM_006527321.2	-0.82898211	0.041369788
mmu-miR-8094	NM_008524.2	-0.884968563	0.019087286
mmu-miR-8094	NM_001290273.1	-0.843638231	0.03476206
mmu-miR-8094	XM_006520023.2	-0.817747677	0.046797026
mmu-miR-8094	NM_001252563.1	-0.824930879	0.043290932
mmu-miR-8094	NM_010779.2	-0.866897992	0.025395189
mmu-miR-8094	NM_010809.2	-0.867137439	0.025306015
mmu-miR-8094	XM_006529456.3	-0.846584464	0.033499071
mmu-miR-8094	XM_006510077.3	-0.822407656	0.044508015

mmu-miR-8094	NM_008760.4	-0.879401731	0.020938926
mmu-miR-8094	NM_172907.3	-0.851590238	0.03140379
mmu-miR-8094	XM_006516928.2	-0.915674822	0.010366296
mmu-miR-8094	XM_011239969.2	-0.93093885	0.006989472
mmu-miR-8094	NM_008804.4	-0.907708488	0.012383528
mmu-miR-8094	NM_001164593.1	-0.884955459	0.019091548
mmu-miR-8094	XM_017312497.1	-0.912260556	0.011209597
mmu-miR-8094	NM_008966.3	-0.876998629	0.021763541
mmu-miR-8094	XM_006505757.3	-0.847419765	0.033145003
mmu-miR-8094	NM_027455.3	-0.88783377	0.018166298
mmu-miR-8094	NM_198024.2	-0.861079174	0.027607978
mmu-miR-8094	NM_011254.5	-0.83190409	0.040009473
mmu-miR-8094	NM_031192.3	-0.893156223	0.016513546
mmu-miR-8094	XM_011240779.2	-0.821346337	0.045024641
mmu-miR-8094	XM_006525075.3	-0.870662138	0.024010624
mmu-miR-8094	NM_023893.4	-0.919934588	0.009359077
mmu-miR-8094	NM_026535.2	-0.875185258	0.022395853
mmu-miR-8094	NM_177578.4	-0.85208019	0.031202143
mmu-miR-8094	NM_001310705.1	-0.852063834	0.031208864
mmu-miR-8094	NM_001146217.1	-0.83661252	0.03786235
mmu-miR-8094	NM_009223.3	-0.842121199	0.035420952
mmu-miR-8094	NM_001204201.1	-0.871794212	0.023601445
mmu-miR-8094	NM_011581.3	-0.879437498	0.020926768
mmu-miR-8094	XM_006537836.1	-0.886569859	0.018569877
mmu-miR-8094	NM_178715.3	-0.920933599	0.009130102
mmu-miR-8094	NM_177371.3	-0.916505922	0.010165862
mmu-miR-8094	XM_006496905.1	-0.929846534	0.007209633
mmu-miR-8094	NM_001243916.1	-0.855801435	0.029690662
mmu-miR-8094	XM_017321602.1	-0.883457273	0.019581857
mmu-miR-8094	NM_177709.3	-0.928850161	0.007413359
mmu-miR-8094	NM_011658.2	-0.88103049	0.020388685
mmu-miR-8117	NM_024283.3	-0.911708038	0.011349067
mmu-miR-8117	XM_006515980.3	-0.862709141	0.026979287
mmu-miR-8117	NM_153151.3	-0.855475875	0.02982148
mmu-miR-8117	NM_001142804.1	-0.828826743	0.041442714
mmu-miR-8117	XM_006498086.3	-0.825854889	0.042849172
mmu-miR-8117	NM_021475.2	-0.824512602	0.043491601
mmu-miR-8117	XM_006500919.3	-0.927960304	0.007597644
mmu-miR-8117	NM_145635.2	-0.820442671	0.045466713
mmu-miR-8117	NM_009605.4	-0.837445614	0.03748823
mmu-miR-8117	NM_001291930.1	-0.832218534	0.039864354
mmu-miR-8117	NM_134072.1	-0.913871397	0.010807747
mmu-miR-8117	NM_001303431.1	-0.876956555	0.021778114
mmu-miR-8117	NM_009675.2	-0.834443366	0.038844628
mmu-miR-8117	NM_001110009.2	-0.882514615	0.019893409
mmu-miR-8117	XM_006522398.3	-0.948683734	0.003882472
mmu-miR-8117	NM_025711.3	-0.955710932	0.002898845
mmu-miR-8117	NM_020025.4	-0.869539463	0.024419712
mmu-miR-8117	NM_001159407.1	-0.845577279	0.033928354
mmu-miR-8117	NM_007529.2	-0.941562808	0.005022579
mmu-miR-8117	NM_001002896.2	-0.849395	0.032314798
mmu-miR-8117	NM_007555.4	-0.903729749	0.013455827
mmu-miR-8117	NM_028472.2	-0.82455459	0.043471437
mmu-miR-8117	NM_178692.3	-0.874062375	0.022791725

mmu-miR-8117	XM_006503665.3	-0.822893107	0.044272634
mmu-miR-8117	NM_001097617.1	-0.922102126	0.008865773
mmu-miR-8117	NM_027416.3	-0.890732331	0.017256839
mmu-miR-8117	NM_019626.3	-0.883012876	0.019728438
mmu-miR-8117	NM_001001178.1	-0.842236676	0.035370593
mmu-miR-8117	XM_006508867.2	-0.826403414	0.042587928
mmu-miR-8117	XM_006508885.1	-0.871748258	0.023617989
mmu-miR-8117	XM_017312731.1	-0.929421943	0.007296109
mmu-miR-8117	NM_020001.2	-0.821140704	0.045125059
mmu-miR-8117	XM_017314944.1	-0.931359159	0.006905645
mmu-miR-8117	NM_009930.2	-0.934915844	0.006216074
mmu-miR-8117	NM_001309809.2	-0.976986228	0.000788356
mmu-miR-8117	NM_019696.2	-0.817511963	0.046914228
mmu-miR-8117	NM_007792.4	-0.870696531	0.023998143
mmu-miR-8117	NM_018866.2	-0.939116545	0.005447351
mmu-miR-8117	XM_006517308.3	-0.873219646	0.023091001
mmu-miR-8117	NM_030206.4	-0.819295803	0.046030652
mmu-miR-8117	NM_007817.2	-0.841761514	0.035578028
mmu-miR-8117	XM_006540596.2	-0.831132267	0.040366724
mmu-miR-8117	NM_001190451.2	-0.953801485	0.003152153
mmu-miR-8117	NM_001190449.1	-0.886819309	0.018489888
mmu-miR-8117	NM_001190448.1	-0.847027052	0.033311246
mmu-miR-8117	NM_015814.2	-0.924752099	0.008280334
mmu-miR-8117	NM_009345.2	-0.861619537	0.027398794
mmu-miR-8117	NM_019759.2	-0.826423599	0.042578329
mmu-miR-8117	NM_001291145.1	-0.886859944	0.018476874
mmu-miR-8117	NM_015744.4	-0.844455769	0.034409393
mmu-miR-8117	NM_010140.3	-0.91448084	0.010657567
mmu-miR-8117	NM_024406.2	-0.868153755	0.024929179
mmu-miR-8117	NM_177743.5	-0.978273472	0.000702935
mmu-miR-8117	XM_006520437.2	-0.951055427	0.003534732
mmu-miR-8117	XM_006496949.3	-0.829677698	0.041044032
mmu-miR-8117	NM_145741.2	-0.872134259	0.023479192
mmu-miR-8117	NM_010279.3	-0.837434794	0.037493078
mmu-miR-8117	XM_006510297.3	-0.886606068	0.018558256
mmu-miR-8117	NM_001318003.2	-0.822429173	0.04449757
mmu-miR-8117	XM_001481172.6	-0.890455908	0.017342603
mmu-miR-8117	XM_006496277.3	-0.897868932	0.015113481
mmu-miR-8117	XM_011246309.2	-0.830492666	0.0406639
mmu-miR-8117	NM_010450.3	-0.914664763	0.010612444
mmu-miR-8117	NM_008264.1	-0.889879929	0.017521963
mmu-miR-8117	NM_023670.3	-0.890056269	0.017466957
mmu-miR-8117	XM_006523711.3	-0.886866717	0.018474705
mmu-miR-8117	XM_017316439.1	-0.835400865	0.038409581
mmu-miR-8117	NM_001161541.1	-0.964550864	0.001862688
mmu-miR-8117	NM_010608.2	-0.868411863	0.024833903
mmu-miR-8117	NM_031180.2	-0.939320135	0.005411356
mmu-miR-8117	NM_173427.2	-0.8892675	0.017713646
mmu-miR-8117	NM_008940.3	-0.869469357	0.024445366
mmu-miR-8117	NM_016659.3	-0.876025351	0.022101843
mmu-miR-8117	NM_146063.1	-0.900686476	0.014304991
mmu-miR-8117	XM_017315313.1	-0.866571083	0.025517178
mmu-miR-8117	XM_006523061.3	-0.916398722	0.010191609
mmu-miR-8117	XM_006511102.2	-0.874670091	0.022577064

mmu-miR-8117	XM_006504999.1	-0.876272048	0.022015858
mmu-miR-8117	XM_006527221.3	-0.840492435	0.036134846
mmu-miR-8117	NM_010714.3	-0.899127549	0.014749676
mmu-miR-8117	NM_175271.4	-0.831715379	0.040096684
mmu-miR-8117	NM_028973.2	-0.960123408	0.002353509
mmu-miR-8117	XM_006527321.2	-0.827072568	0.042270243
mmu-miR-8117	NM_008524.2	-0.900376719	0.014392827
mmu-miR-8117	NM_001290273.1	-0.822009265	0.044701617
mmu-miR-8117	XM_006520023.2	-0.907859782	0.012343603
mmu-miR-8117	NM_001252563.1	-0.959541237	0.002422254
mmu-miR-8117	NM_010779.2	-0.912107723	0.011248092
mmu-miR-8117	NM_001291483.1	-0.861388595	0.027488103
mmu-miR-8117	NM_029844.3	-0.868680465	0.02473494
mmu-miR-8117	XM_006498901.3	-0.833185964	0.039419423
mmu-miR-8117	XM_006510077.3	-0.922216251	0.008840159
mmu-miR-8117	NM_023456.3	-0.850231449	0.031966228
mmu-miR-8117	NM_008760.4	-0.970689995	0.001276025
mmu-miR-8117	NM_172907.3	-0.873293801	0.023064591
mmu-miR-8117	XM_006516928.2	-0.898868671	0.014824156
mmu-miR-8117	XM_017322026.1	-0.859283748	0.02830843
mmu-miR-8117	NM_001164593.1	-0.980310089	0.000577722
mmu-miR-8117	NM_011082.3	-0.864436189	0.026320655
mmu-miR-8117	NM_008872.3	-0.820773999	0.045304394
mmu-miR-8117	XM_017312497.1	-0.935769387	0.006055863
mmu-miR-8117	XM_011250776.2	-0.849810099	0.032141592
mmu-miR-8117	NM_001030305.2	-0.837128375	0.037630488
mmu-miR-8117	NM_011169.5	-0.85049939	0.031854947
mmu-miR-8117	NM_001081224.2	-0.947679027	0.004034612
mmu-miR-8117	NM_008966.3	-0.902612664	0.013764615
mmu-miR-8117	XM_006505757.3	-0.926767735	0.007848076
mmu-miR-8117	NM_016933.3	-0.814595351	0.048375694
mmu-miR-8117	NM_198024.2	-0.829883131	0.040948054
mmu-miR-8117	XM_006525075.3	-0.874078255	0.022786103
mmu-miR-8117	NM_009127.4	-0.869802522	0.024323561
mmu-miR-8117	NM_009135.2	-0.839355608	0.036637087
mmu-miR-8117	XM_011244046.2	-0.91070597	0.011604146
mmu-miR-8117	XM_006521796.1	-0.89561193	0.015776552
mmu-miR-8117	NM_177578.4	-0.860947298	0.027659144
mmu-miR-8117	NM_001310705.1	-0.94301218	0.00477888
mmu-miR-8117	NM_001290993.1	-0.836845336	0.037757623
mmu-miR-8117	NM_001146217.1	-0.875248913	0.02237351
mmu-miR-8117	XM_006524777.3	-0.96617213	0.001697132
mmu-miR-8117	NM_011430.3	-0.876932752	0.021786361
mmu-miR-8117	NM_175692.3	-0.861190855	0.027564682
mmu-miR-8117	NM_009223.3	-0.869905597	0.024285936
mmu-miR-8117	NM_031183.2	-0.830159773	0.040818974
mmu-miR-8117	NM_178753.4	-0.87227021	0.023430399
mmu-miR-8117	NM_001204201.1	-0.884354884	0.019287382
mmu-miR-8117	NM_032400.2	-0.885377517	0.018954497
mmu-miR-8117	NM_011581.3	-0.905098224	0.013082162
mmu-miR-8117	NM_009381.3	-0.91307108	0.01100651
mmu-miR-8117	XM_006533452.3	-0.815880944	0.047728938
mmu-miR-8117	NM_178715.3	-0.824900124	0.043305672
mmu-miR-8117	NM_144936.1	-0.87882002	0.021137142

mmu-miR-8117	NM_177371.3	-0.893252106	0.016484467
mmu-miR-8117	XM_006496905.1	-0.944280486	0.004570501
mmu-miR-8117	XM_011249157.2	-0.827391169	0.042119376
mmu-miR-8117	XM_017321602.1	-0.936899819	0.005846828
mmu-miR-8117	NM_011658.2	-0.926847151	0.007831276
mmu-miR-8117	XM_011248394.1	-0.838029734	0.037226957
mmu-miR-8117	XM_006525028.2	-0.904229733	0.013318716
mmu-miR-8117	NM_016982.2	-0.870447734	0.024088496
mmu-miR-8117	XM_017316944.1	-0.820476692	0.045450033
mmu-miR-8117	XM_017318541.1	-0.927717635	0.007648282
mmu-miR-8117	XM_011243586.2	-0.988350382	0.00020278

Table S6 Correlation analysis of mRNA and circRNA

right_name	left_name	correlation	pvalue
XM_017316736.1	circRNA_3276|Chr19:60563162_60564294_-	0.997768343	7.46E-06
XM_017315313.1	circRNA_3702|Chr2:143830084_143832057_-	0.99753106	9.14E-06
NM_205810.4	circRNA_2888|Chr18:21010677_21020999_+	0.997452763	9.72E-06
XM_006495613.3	circRNA_0913|Chr11:54005394_54014456_-	0.995208948	3.44E-05
NM_027455.3	circRNA_5311|Chr6:145147636_145149357_+	0.99480954	4.03E-05
NM_138955.3	circRNA_3832|Chr2:169883526_169886459_+	0.994640588	4.3E-05
NM_031183.2	circRNA_2951|Chr18:39120119_39150118_+	0.994007356	5.38E-05
NM_008277.2	circRNA_5367|Chr7:28990988_28991325_+	0.992673711	8.03E-05
NM_011082.3	circRNA_0501|Chr10:25283893_25289730_-	0.992327216	8.81E-05
NM_011082.3	circRNA_6109|Chr9:57056714_57057861_+	0.992327216	8.81E-05
XM_006515637.2	circRNA_5367|Chr7:28990988_28991325_+	0.992299692	8.87E-05
NM_001303431.1	circRNA_6140|Chr9:61935380_61937535_-	0.991884536	9.85E-05
NM_205810.4	circRNA_0501|Chr10:25283893_25289730_-	0.991792362	0.000101
NM_205810.4	circRNA_6109|Chr9:57056714_57057861_+	0.991792362	0.000101
NM_144945.3	circRNA_0537|Chr10:43393871_43395692_+	0.991557113	0.000107
NM_001327998.1	circRNA_3276|Chr19:60563162_60564294_-	0.990949628	0.000122
NM_009345.2	circRNA_3155|Chr19:25061108_25065459_+	0.990257923	0.000142
XM_006538830.1	circRNA_3832|Chr2:169883526_169886459_+	0.990062534	0.000148
XM_006527321.2	circRNA_3702|Chr2:143830084_143832057_-	0.990024805	0.000149
NM_001033167.3	circRNA_3832|Chr2:169883526_169886459_+	0.989869079	0.000153
NM_010714.3	circRNA_3702|Chr2:143830084_143832057_-	0.989856153	0.000154
NM_009349.3	circRNA_1632|Chr13:42055400_42055542_+	0.989461887	0.000166
NM_175271.4	circRNA_3239|Chr19:45635735_45640521_-	0.989370487	0.000169
NM_178715.3	circRNA_5311|Chr6:145147636_145149357_+	0.989196209	0.000174
NM_011082.3	circRNA_0157|Chr1:66801049_66802168_-	0.988449632	0.000199
NM_010809.2	circRNA_3702|Chr2:143830084_143832057_-	0.988037921	0.000214
NM_019696.2	circRNA_3155|Chr19:25061108_25065459_+	0.987936699	0.000217
NM_001190449.1	circRNA_3155|Chr19:25061108_25065459_+	0.987578613	0.00023
NM_146063.1	circRNA_4728|Chr5:97027831_97045167_+	0.987059436	0.00025
XM_017312731.1	circRNA_0020|Chr1:10315205_10321709_+	0.986180578	0.000285
NM_145635.2	circRNA_4662|Chr5:65301933_65303208_-	0.985618251	0.000309
NM_031183.2	circRNA_2888|Chr18:21010677_21020999_+	0.985297723	0.000323
NM_023143.3	circRNA_3155|Chr19:25061108_25065459_+	0.985169717	0.000328
NM_001195084.1	circRNA_3276|Chr19:60563162_60564294_-	0.985040831	0.000334
NM_001317365.1	circRNA_0845|Chr11:29470315_29477458_+	0.984762741	0.000346
NM_008940.3	circRNA_0376|Chr1:172173943_172187460_+	0.984677926	0.00035
NM_138955.3	circRNA_0537|Chr10:43393871_43395692_+	0.984538249	0.000357
XM_006527221.3	circRNA_3702|Chr2:143830084_143832057_-	0.984462559	0.00036
NM_001190448.1	circRNA_3702|Chr2:143830084_143832057_-	0.984386304	0.000364
XM_006498901.3	circRNA_4662|Chr5:65301933_65303208_-	0.984336672	0.000366
XM_006517308.3	circRNA_3155|Chr19:25061108_25065459_+	0.984252178	0.00037
NM_001204201.1	circRNA_3155|Chr19:25061108_25065459_+	0.984159771	0.000374
XM_006538830.1	circRNA_0537|Chr10:43393871_43395692_+	0.983851665	0.000389
NM_031183.2	circRNA_0501|Chr10:25283893_25289730_-	0.983826745	0.00039
NM_031183.2	circRNA_6109|Chr9:57056714_57057861_+	0.983826745	0.00039
NM_009645.2	circRNA_4728|Chr5:97027831_97045167_+	0.983654404	0.000399
NM_205810.4	circRNA_2951|Chr18:39120119_39150118_+	0.983263403	0.000418
XM_011241322.1	circRNA_3832|Chr2:169883526_169886459_+	0.983023361	0.00043
NM_001162950.1	circRNA_6390|Chr9:121727316_121731565_+	0.982666501	0.000448
XM_006508990.2	circRNA_3832|Chr2:169883526_169886459_+	0.982535089	0.000455
NM_145226.2	circRNA_3954|Chr3:88346445_88349444_+	0.982481714	0.000458
XM_011239558.2	circRNA_3832|Chr2:169883526_169886459_+	0.982420136	0.000461
XM_011249157.2	circRNA_3155|Chr19:25061108_25065459_+	0.982178244	0.000474
NM_053080.3	circRNA_0611|Chr10:75274017_75279961_+	0.98216978	0.000474
XM_006504999.1	circRNA_4728|Chr5:97027831_97045167_+	0.981848376	0.000491
XM_006522398.3	circRNA_0020|Chr1:10315205_10321709_+	0.981359922	0.000518

NM_001033167.3	circRNA_0537|Chr10:43393871_43395692_+	0.981137247	0.00053
NM_009518.2	circRNA_3276|Chr19:60563162_60564294_-	0.981058748	0.000535
NM_011082.3	circRNA_2951|Chr18:39120119_39150118_+	0.980829145	0.000548
NM_016933.3	circRNA_3155|Chr19:25061108_25065459_+	0.98070234	0.000555
NM_146125.2	circRNA_4728|Chr5:97027831_97045167_+	0.980555614	0.000563
XM_006525075.3	circRNA_6140|Chr9:61935380_61937535_-	0.980143944	0.000587
XM_011240779.2	circRNA_3239|Chr19:45635735_45640521_-	0.97997175	0.000598
XM_017315734.1	circRNA_3832|Chr2:169883526_169886459_+	0.979968013	0.000598
XM_017316736.1	circRNA_4987|Chr6:4529068_4531010_+	0.979952233	0.000599
NM_010450.3	circRNA_6140|Chr9:61935380_61937535_-	0.979578962	0.000621
NM_001033286.2	circRNA_2313|Chr15:93452117_93465245_+	0.979493529	0.000626
NM_019626.3	circRNA_6140|Chr9:61935380_61937535_-	0.979317828	0.000637
NM_011430.3	circRNA_4662|Chr5:65301933_65303208_-	0.979130074	0.000649
XM_006506086.2	circRNA_3276|Chr19:60563162_60564294_-	0.979095884	0.000651
NM_001290822.1	circRNA_0157|Chr1:66801049_66802168_-	0.979081516	0.000652
NM_001146217.1	circRNA_6140|Chr9:61935380_61937535_-	0.979063045	0.000653
NM_177709.3	circRNA_6140|Chr9:61935380_61937535_-	0.979013686	0.000656
NM_023143.3	circRNA_3239|Chr19:45635735_45640521_-	0.978960108	0.000659
NM_019696.2	circRNA_3239|Chr19:45635735_45640521_-	0.978900272	0.000663
XM_011246128.2	circRNA_1632|Chr13:42055400_42055542_+	0.978340108	0.000699
NM_146241.2	circRNA_0501|Chr10:25283893_25289730_-	0.978324464	0.0007
NM_146241.2	circRNA_6109|Chr9:57056714_57057861_+	0.978324464	0.0007
NM_011658.2	circRNA_6140|Chr9:61935380_61937535_-	0.978091343	0.000715
XM_006505388.2	circRNA_0501|Chr10:25283893_25289730_-	0.97798736	0.000722
XM_006505388.2	circRNA_6109|Chr9:57056714_57057861_+	0.97798736	0.000722
NM_013468.3	circRNA_4926|Chr5:143080224_143081077_-	0.977976349	0.000722
XM_017317034.1	circRNA_3832|Chr2:169883526_169886459_+	0.97793471	0.000725
NM_001030305.2	circRNA_3702|Chr2:143830084_143832057_-	0.977849272	0.000731
NM_177578.4	circRNA_5311|Chr6:145147636_145149357_+	0.977265796	0.000769
NM_001290822.1	circRNA_0501|Chr10:25283893_25289730_-	0.977084843	0.000782
NM_001290822.1	circRNA_6109|Chr9:57056714_57057861_+	0.977084843	0.000782
XM_011239558.2	circRNA_0537|Chr10:43393871_43395692_+	0.976962188	0.00079
NM_015814.2	circRNA_3702|Chr2:143830084_143832057_-	0.976890477	0.000795
XM_006521796.1	circRNA_4728|Chr5:97027831_97045167_+	0.976756951	0.000804
NM_146241.2	circRNA_2888|Chr18:21010677_21020999_+	0.976726189	0.000806
XM_006533653.3	circRNA_1042|Chr11:80385854_80403408_+	0.976113954	0.000849
NM_016659.3	circRNA_3155|Chr19:25061108_25065459_+	0.975936014	0.000862
NM_001159407.1	circRNA_3702|Chr2:143830084_143832057_-	0.975789613	0.000872
XM_011248394.1	circRNA_2951|Chr18:39120119_39150118_+	0.975557675	0.000889
XM_006516928.2	circRNA_3155|Chr19:25061108_25065459_+	0.975158408	0.000918
NM_008966.3	circRNA_5311|Chr6:145147636_145149357_+	0.975064361	0.000925
XM_006537815.3	circRNA_3832|Chr2:169883526_169886459_+	0.975043027	0.000927
NM_027416.3	circRNA_0020|Chr1:10315205_10321709_+	0.974969135	0.000932
XM_011248394.1	circRNA_0501|Chr10:25283893_25289730_-	0.974918239	0.000936
XM_011248394.1	circRNA_6109|Chr9:57056714_57057861_+	0.974918239	0.000936
NM_001162950.1	circRNA_4926|Chr5:143080224_143081077_-	0.974602094	0.000959
NM_028748.2	circRNA_4635|Chr5:43758222_43773659_-	0.974561551	0.000962
XM_006496949.3	circRNA_3276|Chr19:60563162_60564294_-	0.974302366	0.000982
NM_029823.2	circRNA_5639|Chr7:132771578_132779385_-	0.974163152	0.000993
XM_006515980.3	circRNA_6140|Chr9:61935380_61937535_-	0.97411396	0.000996
NM_023670.3	circRNA_3155|Chr19:25061108_25065459_+	0.973860483	0.001016
NM_016697.3	circRNA_2888|Chr18:21010677_21020999_+	0.973640805	0.001033
XM_006505388.2	circRNA_2888|Chr18:21010677_21020999_+	0.973640598	0.001033
NM_010809.2	circRNA_6140|Chr9:61935380_61937535_-	0.973582104	0.001038
XM_011250776.2	circRNA_3702|Chr2:143830084_143832057_-	0.973511166	0.001043
XM_011240779.2	circRNA_3155|Chr19:25061108_25065459_+	0.973360129	0.001055
XM_006532024.3	circRNA_4055|Chr3:126796838_126798280_+	0.973323416	0.001058
NM_016697.3	circRNA_0501|Chr10:25283893_25289730_-	0.973239912	0.001065
NM_016697.3	circRNA_6109|Chr9:57056714_57057861_+	0.973239912	0.001065

XM_006540762.3	circRNA_0962|Chr11:67194348_67220998_+	0.973146309	0.001072
NM_013912.3	circRNA_3239|Chr19:45635735_45640521_-	0.973110388	0.001075
XM_001481172.6	circRNA_3155|Chr19:25061108_25065459_+	0.972860438	0.001095
NM_025711.3	circRNA_3702|Chr2:143830084_143832057_-	0.972851965	0.001096
XM_006520437.2	circRNA_2951|Chr18:39120119_39150118_+	0.972777509	0.001102
NM_007549.2	circRNA_3702|Chr2:143830084_143832057_-	0.972490896	0.001125
NM_144945.3	circRNA_3832|Chr2:169883526_169886459_+	0.972456222	0.001128
XM_006508885.1	circRNA_0020|Chr1:10315205_10321709_+	0.972426182	0.00113
NM_013468.3	circRNA_6390|Chr9:121727316_121731565_+	0.972378016	0.001134
XM_006532007.3	circRNA_1632|Chr13:42055400_42055542_+	0.972242865	0.001145
NM_009223.3	circRNA_3702|Chr2:143830084_143832057_-	0.972003164	0.001165
XM_006525075.3	circRNA_3702|Chr2:143830084_143832057_-	0.971910459	0.001172
NM_001290993.1	circRNA_3702|Chr2:143830084_143832057_-	0.971603127	0.001198
NM_007792.4	circRNA_3155|Chr19:25061108_25065459_+	0.971591546	0.001199
NM_001024731.2	circRNA_0611|Chr10:75274017_75279961_+	0.971570239	0.001201
XM_006531321.3	circRNA_0961|Chr11:67192403_67254873_+	0.971474974	0.001209
NM_016933.3	circRNA_3239|Chr19:45635735_45640521_-	0.971191231	0.001233
NM_025711.3	circRNA_6140|Chr9:61935380_61937535_-	0.970997527	0.00125
NM_175271.4	circRNA_3155|Chr19:25061108_25065459_+	0.970735409	0.001272
XM_006515980.3	circRNA_3702|Chr2:143830084_143832057_-	0.970512531	0.001291
XM_006510297.3	circRNA_3702|Chr2:143830084_143832057_-	0.970492053	0.001293
XM_006514077.1	circRNA_2313|Chr15:93452117_93465245_+	0.970308046	0.001309
XM_006504999.1	circRNA_0020|Chr1:10315205_10321709_+	0.969732173	0.00136
XM_006506086.2	circRNA_1632|Chr13:42055400_42055542_+	0.969620637	0.00137
NM_001198955.1	circRNA_6390|Chr9:121727316_121731565_+	0.969583432	0.001374
XM_006522506.3	circRNA_4662|Chr5:65301933_65303208_-	0.969494645	0.001382
NM_008524.2	circRNA_3155|Chr19:25061108_25065459_+	0.96948995	0.001382
XM_006505757.3	circRNA_3155|Chr19:25061108_25065459_+	0.969352142	0.001395
XM_006502291.3	circRNA_6390|Chr9:121727316_121731565_+	0.969189167	0.001409
NM_181315.4	circRNA_6446|ChrX:42217416_42250037_+	0.969089662	0.001418
NM_009127.4	circRNA_3702|Chr2:143830084_143832057_-	0.969066247	0.001421
XM_006533452.3	circRNA_3702|Chr2:143830084_143832057_-	0.969032198	0.001424
NM_001289875.1	circRNA_3832|Chr2:169883526_169886459_+	0.96900365	0.001426
XM_006516928.2	circRNA_3239|Chr19:45635735_45640521_-	0.96897804	0.001429
NM_019626.3	circRNA_3702|Chr2:143830084_143832057_-	0.968892548	0.001436
NM_001282961.1	circRNA_0157|Chr1:66801049_66802168_-	0.968773611	0.001447
XM_006527221.3	circRNA_6140|Chr9:61935380_61937535_-	0.968738241	0.001451
XM_011246479.2	circRNA_4487|Chr4:149156607_149161694_-	0.968628766	0.001461
NM_011169.5	circRNA_6140|Chr9:61935380_61937535_-	0.968536885	0.001469
XM_017317034.1	circRNA_0537|Chr10:43393871_43395692_+	0.968497005	0.001473
XM_006514444.1	circRNA_4926|Chr5:143080224_143081077_-	0.968396407	0.001482
NM_016697.3	circRNA_2951|Chr18:39120119_39150118_+	0.968360531	0.001486
NM_001204201.1	circRNA_3239|Chr19:45635735_45640521_-	0.968267056	0.001494
XM_006527321.2	circRNA_6140|Chr9:61935380_61937535_-	0.968192802	0.001501
NM_011658.2	circRNA_3702|Chr2:143830084_143832057_-	0.968100496	0.00151
XM_006508990.2	circRNA_0537|Chr10:43393871_43395692_+	0.968052029	0.001515
NM_008524.2	circRNA_5311|Chr6:145147636_145149357_+	0.967990325	0.001521
XM_017315313.1	circRNA_6140|Chr9:61935380_61937535_-	0.967826348	0.001536
NM_146241.2	circRNA_2951|Chr18:39120119_39150118_+	0.96769038	0.001549
NM_001290273.1	circRNA_6140|Chr9:61935380_61937535_-	0.967636891	0.001554
XM_011241948.2	circRNA_0376|Chr1:172173943_172187460_+	0.967378391	0.001579
NM_010714.3	circRNA_6140|Chr9:61935380_61937535_-	0.96722618	0.001594
NM_001004357.2	circRNA_0611|Chr10:75274017_75279961_+	0.967174635	0.001599
NM_144936.1	circRNA_4728|Chr5:97027831_97045167_+	0.967105405	0.001605
NM_011082.3	circRNA_2888|Chr18:21010677_21020999_+	0.967030909	0.001613
XM_006525028.2	circRNA_0157|Chr1:66801049_66802168_-	0.966953674	0.00162
XM_006506624.2	circRNA_4055|Chr3:126796838_126798280_+	0.966866323	0.001629
XM_017321602.1	circRNA_3155|Chr19:25061108_25065459_+	0.96682305	0.001633
NM_001033445.2	circRNA_3832|Chr2:169883526_169886459_+	0.966806651	0.001634

NM_001291145.1	circRNA_0157|Chr1:66801049_66802168_-	0.966794466	0.001636
NM_001310636.1	circRNA_3276|Chr19:60563162_60564294_-	0.966558503	0.001659
XM_006496277.3	circRNA_0157|Chr1:66801049_66802168_-	0.966525477	0.001662
NM_152915.1	circRNA_4055|Chr3:126796838_126798280_+	0.966521319	0.001662
XM_006516928.2	circRNA_5311|Chr6:145147636_145149357_+	0.966233502	0.001691
NM_178753.4	circRNA_3702|Chr2:143830084_143832057_-	0.966179739	0.001696
NM_144945.3	circRNA_5074|Chr6:40685277_40747150_+	0.966079977	0.001706
NM_011581.3	circRNA_6140|Chr9:61935380_61937535_-	0.965828692	0.001732
NM_007641.5	circRNA_3702|Chr2:143830084_143832057_-	0.965678843	0.001747
NM_007482.3	circRNA_1632|Chr13:42055400_42055542_+	0.965668586	0.001748
NM_019626.3	circRNA_5311|Chr6:145147636_145149357_+	0.965666288	0.001748
XM_011238891.1	circRNA_6446|ChrX:42217416_42250037_+	0.965653816	0.001749
NM_001134300.2	circRNA_3832|Chr2:169883526_169886459_+	0.965400056	0.001775
NM_001190451.2	circRNA_3702|Chr2:143830084_143832057_-	0.965349546	0.00178
XM_006515637.2	circRNA_2888|Chr18:21010677_21020999_+	0.965252362	0.00179
NM_001013784.1	circRNA_5990|Chr9:3475483_3477962_+	0.965190918	0.001796
NM_021342.1	circRNA_6446|ChrX:42217416_42250037_+	0.965179938	0.001798
XM_011248602.2	circRNA_3832|Chr2:169883526_169886459_+	0.965109539	0.001805
NM_015814.2	circRNA_6140|Chr9:61935380_61937535_-	0.965024434	0.001814
NM_001029877.3	circRNA_6390|Chr9:121727316_121731565_+	0.964945717	0.001822
NM_011581.3	circRNA_3702|Chr2:143830084_143832057_-	0.964914404	0.001825
XM_006529753.2	circRNA_3832|Chr2:169883526_169886459_+	0.96485317	0.001831
XM_017316736.1	circRNA_3643|Chr2:122441559_122486090_+	0.96483074	0.001834
NM_001013784.1	circRNA_0537|Chr10:43393871_43395692_+	0.964779516	0.001839
NM_001024731.2	circRNA_3155|Chr19:25061108_25065459_+	0.964500847	0.001868
NM_028804.1	circRNA_3702|Chr2:143830084_143832057_-	0.964494764	0.001869
XM_006532120.2	circRNA_3155|Chr19:25061108_25065459_+	0.964435764	0.001875
XM_006530294.3	circRNA_3425|Chr2:37624173_37627526_-	0.964433486	0.001875
XM_006530294.3	circRNA_3607|Chr2:119057281_119064097_+	0.964433486	0.001875
XM_006525028.2	circRNA_0501|Chr10:25283893_25289730_-	0.964335428	0.001885
XM_006525028.2	circRNA_6109|Chr9:57056714_57057861_+	0.964335428	0.001885
NM_009135.2	circRNA_2951|Chr18:39120119_39150118_+	0.964274593	0.001892
NM_001290822.1	circRNA_2888|Chr18:21010677_21020999_+	0.964172958	0.001902
XM_006511221.3	circRNA_0537|Chr10:43393871_43395692_+	0.963998352	0.001921
NM_001318003.2	circRNA_0020|Chr1:10315205_10321709_+	0.963945473	0.001926
XM_006532120.2	circRNA_3239|Chr19:45635735_45640521_-	0.963717434	0.001951
NM_009345.2	circRNA_3239|Chr19:45635735_45640521_-	0.963629411	0.00196
XM_006533539.3	circRNA_3155|Chr19:25061108_25065459_+	0.963601767	0.001963
NM_008458.2	circRNA_6446|ChrX:42217416_42250037_+	0.963535751	0.00197
XM_011249157.2	circRNA_3239|Chr19:45635735_45640521_-	0.963436682	0.001981
NM_020001.2	circRNA_4055|Chr3:126796838_126798280_+	0.963425809	0.001982
NM_001303431.1	circRNA_3702|Chr2:143830084_143832057_-	0.963397753	0.001985
XM_006513165.1	circRNA_0157|Chr1:66801049_66802168_-	0.963354533	0.00199
NM_144539.5	circRNA_3702|Chr2:143830084_143832057_-	0.963255197	0.002
XM_006524777.3	circRNA_2951|Chr18:39120119_39150118_+	0.963190515	0.002007
NM_001327998.1	circRNA_4987|Chr6:4529068_4531010_+	0.963092179	0.002018
XM_006540596.2	circRNA_0157|Chr1:66801049_66802168_-	0.963039358	0.002024
XM_006530294.3	circRNA_1399|Chr12:72783683_72786789_+	0.96299651	0.002029
NM_010279.3	circRNA_3155|Chr19:25061108_25065459_+	0.962968309	0.002032
XM_006530131.1	circRNA_3832|Chr2:169883526_169886459_+	0.962893241	0.00204
XM_006515787.3	circRNA_2888|Chr18:21010677_21020999_+	0.962881436	0.002041
NM_001146217.1	circRNA_3702|Chr2:143830084_143832057_-	0.962874725	0.002042
NM_008204.2	circRNA_3155|Chr19:25061108_25065459_+	0.962735519	0.002057
XM_006517308.3	circRNA_3239|Chr19:45635735_45640521_-	0.962674546	0.002064
NM_145741.2	circRNA_2951|Chr18:39120119_39150118_+	0.962504849	0.002082
XM_017314347.1	circRNA_4055|Chr3:126796838_126798280_+	0.962488121	0.002084
XM_006510077.3	circRNA_0020|Chr1:10315205_10321709_+	0.962273822	0.002108
NM_007817.2	circRNA_3702|Chr2:143830084_143832057_-	0.962097146	0.002128
NM_001077403.1	circRNA_3832|Chr2:169883526_169886459_+	0.961840047	0.002156

NM_018732.3	circRNA_0961|Chr11:67192403_67254873_+	0.961771067	0.002164
NM_001291145.1	circRNA_0376|Chr1:172173943_172187460_+	0.961716776	0.00217
XM_006520023.2	circRNA_3155|Chr19:25061108_25065459_+	0.961636294	0.002179
NM_010450.3	circRNA_3702|Chr2:143830084_143832057_-	0.961620251	0.002181
XM_006520186.2	circRNA_3155|Chr19:25061108_25065459_+	0.961541594	0.00219
NM_001318003.2	circRNA_5639|Chr7:132771578_132779385_-	0.961448065	0.002201
NM_001310705.1	circRNA_6140|Chr9:61935380_61937535_-	0.961441707	0.002201
NM_001290273.1	circRNA_3702|Chr2:143830084_143832057_-	0.961339253	0.002213
NM_008204.2	circRNA_0611|Chr10:75274017_75279961_+	0.961327297	0.002214
XM_006533539.3	circRNA_0611|Chr10:75274017_75279961_+	0.961213331	0.002227
XM_011250776.2	circRNA_6140|Chr9:61935380_61937535_-	0.961154728	0.002234
XM_006521931.1	circRNA_5311|Chr6:145147636_145149357_+	0.961004444	0.002251
XM_006540596.2	circRNA_0501|Chr10:25283893_25289730_-	0.960732853	0.002283
XM_006540596.2	circRNA_6109|Chr9:57056714_57057861_+	0.960732853	0.002283
XM_006505388.2	circRNA_0157|Chr1:66801049_66802168_-	0.960710008	0.002285
NM_001317365.1	circRNA_0537|Chr10:43393871_43395692_+	0.960280089	0.002335
NM_008012.1	circRNA_6140|Chr9:61935380_61937535_-	0.960149474	0.00235
XM_006525028.2	circRNA_2951|Chr18:39120119_39150118_+	0.960122497	0.002354
NM_007883.3	circRNA_0962|Chr11:67194348_67220998_+	0.960055439	0.002361
NM_205810.4	circRNA_0611|Chr10:75274017_75279961_+	0.959999168	0.002368
NM_001291930.1	circRNA_3276|Chr19:60563162_60564294_-	0.95994504	0.002374
XM_017316439.1	circRNA_3702|Chr2:143830084_143832057_-	0.959920319	0.002377
XM_006503665.3	circRNA_3702|Chr2:143830084_143832057_-	0.959597016	0.002416
XM_006515787.3	circRNA_1632|Chr13:42055400_42055542_+	0.959543374	0.002422
NM_001097617.1	circRNA_3155|Chr19:25061108_25065459_+	0.959481912	0.002429
XM_006530294.3	circRNA_4487|Chr4:149156607_149161694_-	0.959380763	0.002441
NM_008623.5	circRNA_1632|Chr13:42055400_42055542_+	0.959251556	0.002457
NM_001001178.1	circRNA_4662|Chr5:65301933_65303208_-	0.959236542	0.002459
NM_013743.2	circRNA_0961|Chr11:67192403_67254873_+	0.959209127	0.002462
NM_028748.2	circRNA_3003|Chr18:67545615_67587859_-	0.95900676	0.002486
NM_001282961.1	circRNA_4728|Chr5:97027831_97045167_+	0.958936328	0.002495
XM_006508867.2	circRNA_0020|Chr1:10315205_10321709_+	0.958908336	0.002498
NM_001110009.2	circRNA_0020|Chr1:10315205_10321709_+	0.958829843	0.002508
NM_008012.1	circRNA_3702|Chr2:143830084_143832057_-	0.958792366	0.002512
NM_001327998.1	circRNA_3643|Chr2:122441559_122486090_+	0.958785663	0.002513
XM_006496905.1	circRNA_5311|Chr6:145147636_145149357_+	0.958759813	0.002516
NM_001289875.1	circRNA_0537|Chr10:43393871_43395692_+	0.958310399	0.002571
NM_001171187.1	circRNA_1632|Chr13:42055400_42055542_+	0.958275077	0.002575
NM_133167.3	circRNA_3832|Chr2:169883526_169886459_+	0.958217776	0.002582
XM_006506086.2	circRNA_4987|Chr6:4529068_4531010_+	0.958200615	0.002584
NM_030206.4	circRNA_0501|Chr10:25283893_25289730_-	0.958180062	0.002587
NM_030206.4	circRNA_6109|Chr9:57056714_57057861_+	0.958180062	0.002587
XM_017316897.1	circRNA_0962|Chr11:67194348_67220998_+	0.958131212	0.002593
NM_205810.4	circRNA_0157|Chr1:66801049_66802168_-	0.957855275	0.002627
XM_006529727.2	circRNA_6390|Chr9:121727316_121731565_+	0.957800431	0.002634
XM_011248602.2	circRNA_0537|Chr10:43393871_43395692_+	0.957655756	0.002652
NM_001305585.1	circRNA_4728|Chr5:97027831_97045167_+	0.9576381	0.002654
XM_017316736.1	circRNA_1632|Chr13:42055400_42055542_+	0.95755729	0.002664
XM_006505388.2	circRNA_2951|Chr18:39120119_39150118_+	0.957524992	0.002668
XM_011241322.1	circRNA_0537|Chr10:43393871_43395692_+	0.957361945	0.002688
XM_006533539.3	circRNA_3239|Chr19:45635735_45640521_-	0.957304586	0.002695
NM_011254.5	circRNA_5311|Chr6:145147636_145149357_+	0.957222157	0.002706
NM_134072.1	circRNA_0376|Chr1:172173943_172187460_+	0.957119883	0.002719
XM_017321602.1	circRNA_3702|Chr2:143830084_143832057_-	0.957036225	0.002729
NM_001318003.2	circRNA_0376|Chr1:172173943_172187460_+	0.956961953	0.002739
XM_006508885.1	circRNA_0376|Chr1:172173943_172187460_+	0.956830172	0.002755
NM_007482.3	circRNA_5367|Chr7:28990988_28991325_+	0.956820293	0.002756
NM_178692.3	circRNA_0501|Chr10:25283893_25289730_-	0.95678552	0.002761
NM_178692.3	circRNA_6109|Chr9:57056714_57057861_+	0.95678552	0.002761

XM_006540596.2	circRNA_4728|Chr5:97027831_97045167_+	0.956720516	0.002769
XM_006527452.3	circRNA_3155|Chr19:25061108_25065459_+	0.956656192	0.002777
NM_178692.3	circRNA_2951|Chr18:39120119_39150118_+	0.956653812	0.002778
NM_001291145.1	circRNA_4728|Chr5:97027831_97045167_+	0.956639839	0.002779
XM_006520618.2	circRNA_3702|Chr2:143830084_143832057_-	0.956580445	0.002787
NM_010140.3	circRNA_2951|Chr18:39120119_39150118_+	0.956578199	0.002787
NM_026979.5	circRNA_3276|Chr19:60563162_60564294_-	0.956326514	0.002819
NM_008032.3	circRNA_6390|Chr9:121727316_121731565_+	0.956229667	0.002832
NM_031180.2	circRNA_3155|Chr19:25061108_25065459_+	0.95616104	0.002841
NM_001310705.1	circRNA_0020|Chr1:10315205_10321709_+	0.956124487	0.002845
XM_017322365.1	circRNA_3702|Chr2:143830084_143832057_-	0.956051987	0.002855
XM_011248394.1	circRNA_2888|Chr18:21010677_21020999_+	0.956038877	0.002856
NM_001190449.1	circRNA_3239|Chr19:45635735_45640521_-	0.955966256	0.002866
NM_172907.3	circRNA_0020|Chr1:10315205_10321709_+	0.955852916	0.00288
NM_008872.3	circRNA_0501|Chr10:25283893_25289730_-	0.955836424	0.002883
NM_008872.3	circRNA_6109|Chr9:57056714_57057861_+	0.955836424	0.002883
NM_028973.2	circRNA_5311|Chr6:145147636_145149357_+	0.955764462	0.002892
NM_007529.2	circRNA_3155|Chr19:25061108_25065459_+	0.95555771	0.002919
NM_008032.3	circRNA_3832|Chr2:169883526_169886459_+	0.955508324	0.002925
NM_001291145.1	circRNA_0501|Chr10:25283893_25289730_-	0.955485495	0.002928
NM_001291145.1	circRNA_6109|Chr9:57056714_57057861_+	0.955485495	0.002928
XM_006524328.3	circRNA_3832|Chr2:169883526_169886459_+	0.955478907	0.002929
NM_016697.3	circRNA_5367|Chr7:28990988_28991325_+	0.955298101	0.002953
NM_009019.2	circRNA_6446|ChrX:42217416_42250037_+	0.955196205	0.002966
XM_011241948.2	circRNA_5639|Chr7:132771578_132779385_-	0.955165205	0.00297
NM_181547.3	circRNA_5311|Chr6:145147636_145149357_+	0.955072272	0.002982
NM_001317365.1	circRNA_3832|Chr2:169883526_169886459_+	0.955003788	0.002991
NM_008032.3	circRNA_0537|Chr10:43393871_43395692_+	0.954896754	0.003006
NM_001204959.1	circRNA_6446|ChrX:42217416_42250037_+	0.954809139	0.003017
XM_006496277.3	circRNA_0501|Chr10:25283893_25289730_-	0.954736274	0.003027
XM_006496277.3	circRNA_6109|Chr9:57056714_57057861_+	0.954736274	0.003027
XM_006524382.3	circRNA_3954|Chr3:88346445_88349444_+	0.954656777	0.003037
NM_019732.2	circRNA_6390|Chr9:121727316_121731565_+	0.954613074	0.003043
XM_006526449.3	circRNA_1042|Chr11:80385854_80403408_+	0.954569823	0.003049
NM_010858.4	circRNA_4055|Chr3:126796838_126798280_+	0.95431105	0.003084
NM_152915.1	circRNA_6446|ChrX:42217416_42250037_+	0.954294574	0.003086
NM_175260.2	circRNA_3832|Chr2:169883526_169886459_+	0.95419716	0.003099
NM_008872.3	circRNA_2951|Chr18:39120119_39150118_+	0.954156267	0.003104
NM_146241.2	circRNA_0157|Chr1:66801049_66802168_-	0.954145734	0.003106
XM_006503323.3	circRNA_3276|Chr19:60563162_60564294_-	0.954145457	0.003106
XM_006510297.3	circRNA_6140|Chr9:61935380_61937535_-	0.954097719	0.003112
XM_017317978.1	circRNA_4055|Chr3:126796838_126798280_+	0.954021448	0.003122
NM_001171187.1	circRNA_0611|Chr10:75274017_75279961_+	0.953754947	0.003158
XM_017315376.1	circRNA_3702|Chr2:143830084_143832057_-	0.953692234	0.003167
NM_009349.3	circRNA_4987|Chr6:4529068_4531010_+	0.953642723	0.003174
NM_030206.4	circRNA_0157|Chr1:66801049_66802168_-	0.953616884	0.003177
XM_006541297.3	circRNA_0961|Chr11:67192403_67254873_+	0.953536191	0.003188
XM_006532962.2	circRNA_3954|Chr3:88346445_88349444_+	0.953522092	0.00319
NM_009930.2	circRNA_5311|Chr6:145147636_145149357_+	0.953463287	0.003198
NM_001171187.1	circRNA_2888|Chr18:21010677_21020999_+	0.953394831	0.003207
NM_009223.3	circRNA_3155|Chr19:25061108_25065459_+	0.953378557	0.00321
XM_011244046.2	circRNA_6140|Chr9:61935380_61937535_-	0.95335255	0.003213
NM_172907.3	circRNA_5311|Chr6:145147636_145149357_+	0.953305171	0.00322
NM_001159407.1	circRNA_3276|Chr19:60563162_60564294_-	0.953304008	0.00322
XM_006532007.3	circRNA_0611|Chr10:75274017_75279961_+	0.953234461	0.003229
NM_011145.3	circRNA_3954|Chr3:88346445_88349444_+	0.953187888	0.003236
XM_011239558.2	circRNA_3954|Chr3:88346445_88349444_+	0.95311784	0.003245
NM_144945.3	circRNA_0845|Chr11:29470315_29477458_+	0.953058009	0.003254
NM_001013784.1	circRNA_2313|Chr15:93452117_93465245_+	0.95289259	0.003276

NM_008204.2	circRNA_3239|Chr19:45635735_45640521_-	0.952885491	0.003277
XM_006511221.3	circRNA_5811|Chr8:71992832_71998295_-	0.952738493	0.003298
NM_001291930.1	circRNA_2951|Chr18:39120119_39150118_+	0.952668382	0.003307
NM_026385.4	circRNA_2888|Chr18:21010677_21020999_+	0.952608441	0.003316
NM_001190325.1	circRNA_3155|Chr19:25061108_25065459_+	0.952580358	0.00332
NM_019759.2	circRNA_6140|Chr9:61935380_61937535_-	0.95243442	0.00334
NM_001030305.2	circRNA_3276|Chr19:60563162_60564294_-	0.952400742	0.003345
XM_006532007.3	circRNA_2888|Chr18:21010677_21020999_+	0.952327498	0.003355
NM_008458.2	circRNA_4055|Chr3:126796838_126798280_+	0.95231002	0.003357
XM_006527452.3	circRNA_3239|Chr19:45635735_45640521_-	0.952261149	0.003364
XM_006508867.2	circRNA_4055|Chr3:126796838_126798280_+	0.952093643	0.003388
NM_177789.4	circRNA_4728|Chr5:97027831_97045167_+	0.951951474	0.003408
NM_021475.2	circRNA_3155|Chr19:25061108_25065459_+	0.951822619	0.003426
NM_001110009.2	circRNA_0157|Chr1:66801049_66802168_-	0.951804144	0.003428
NM_010340.2	circRNA_5074|Chr6:40685277_40747150_+	0.951641898	0.003451
NM_001110322.1	circRNA_6446|ChrX:42217416_42250037_+	0.951535731	0.003466
NM_009223.3	circRNA_6140|Chr9:61935380_61937535_-	0.95152534	0.003468
NM_001013784.1	circRNA_3832|Chr2:169883526_169886459_+	0.951506578	0.00347
XM_006521428.3	circRNA_3702|Chr2:143830084_143832057_-	0.951498179	0.003472
NM_009349.3	circRNA_3276|Chr19:60563162_60564294_-	0.951362431	0.003491
NM_011581.3	circRNA_5311|Chr6:145147636_145149357_+	0.951340081	0.003494
XM_006537815.3	circRNA_0537|Chr10:43393871_43395692_+	0.951260471	0.003505
NM_009930.2	circRNA_3155|Chr19:25061108_25065459_+	0.951161656	0.00352
NM_019759.2	circRNA_4055|Chr3:126796838_126798280_+	0.951138943	0.003523
NM_009675.2	circRNA_2951|Chr18:39120119_39150118_+	0.951102296	0.003528
XM_006496905.1	circRNA_3155|Chr19:25061108_25065459_+	0.951074098	0.003532
NM_011581.3	circRNA_3155|Chr19:25061108_25065459_+	0.951062679	0.003534
NM_016982.2	circRNA_3155|Chr19:25061108_25065459_+	0.950861271	0.003563
XM_006511221.3	circRNA_2313|Chr15:93452117_93465245_+	0.950794841	0.003572
NM_021475.2	circRNA_3702|Chr2:143830084_143832057_-	0.950784632	0.003574
NM_001195084.1	circRNA_3643|Chr2:122441559_122486090_+	0.950646301	0.003594
XM_006538830.1	circRNA_0845|Chr11:29470315_29477458_+	0.950433954	0.003624
NM_001029877.3	circRNA_3832|Chr2:169883526_169886459_+	0.950303095	0.003643
XM_011248201.2	circRNA_3832|Chr2:169883526_169886459_+	0.950269797	0.003648
NM_001029877.3	circRNA_0537|Chr10:43393871_43395692_+	0.950152376	0.003665
NM_011169.5	circRNA_3702|Chr2:143830084_143832057_-	0.950067432	0.003678
NM_134072.1	circRNA_0020|Chr1:10315205_10321709_+	0.95003693	0.003682
NM_001310648.1	circRNA_6446|ChrX:42217416_42250037_+	0.950035739	0.003682
XM_011238891.1	circRNA_0157|Chr1:66801049_66802168_-	0.949855068	0.003709
XM_011246479.2	circRNA_5074|Chr6:40685277_40747150_+	0.949822949	0.003713
XM_006500919.3	circRNA_3155|Chr19:25061108_25065459_+	0.949804846	0.003716
NM_001302257.1	circRNA_2888|Chr18:21010677_21020999_+	0.949736718	0.003726
XM_006521428.3	circRNA_3276|Chr19:60563162_60564294_-	0.949679175	0.003735
NM_016659.3	circRNA_3239|Chr19:45635735_45640521_-	0.949632069	0.003742
XM_011240476.2	circRNA_5074|Chr6:40685277_40747150_+	0.949572336	0.00375
NM_008872.3	circRNA_2888|Chr18:21010677_21020999_+	0.949519511	0.003758
NM_001195084.1	circRNA_4987|Chr6:4529068_4531010_+	0.949425607	0.003772
NM_010181.2	circRNA_3832|Chr2:169883526_169886459_+	0.949359326	0.003782
NM_145741.2	circRNA_0501|Chr10:25283893_25289730_-	0.949220381	0.003802
NM_145741.2	circRNA_6109|Chr9:57056714_57057861_+	0.949220381	0.003802
XM_017321602.1	circRNA_3239|Chr19:45635735_45640521_-	0.949209497	0.003804
NM_001039047.1	circRNA_3832|Chr2:169883526_169886459_+	0.94908698	0.003822
NM_029823.2	circRNA_4055|Chr3:126796838_126798280_+	0.948882174	0.003853
XM_017314944.1	circRNA_2951|Chr18:39120119_39150118_+	0.948876221	0.003854
NM_028472.2	circRNA_4055|Chr3:126796838_126798280_+	0.948843657	0.003859
XM_011248394.1	circRNA_0157|Chr1:66801049_66802168_-	0.948820282	0.003862
XM_017315734.1	circRNA_0537|Chr10:43393871_43395692_+	0.948741548	0.003874
XM_006508867.2	circRNA_5639|Chr7:132771578_132779385_-	0.948682222	0.003883
XM_017320735.1	circRNA_0157|Chr1:66801049_66802168_-	0.948550403	0.003902

XM_006511221.3	circRNA_5074|Chr6:40685277_40747150_+	0.948439139	0.003919
NM_146063.1	circRNA_0020|Chr1:10315205_10321709_+	0.948386872	0.003927
NM_007555.4	circRNA_0157|Chr1:66801049_66802168_-	0.948365891	0.00393
NM_010742.1	circRNA_3155|Chr19:25061108_25065459_+	0.948290439	0.003942
NM_007482.3	circRNA_2888|Chr18:21010677_21020999_+	0.948281983	0.003943
NM_009127.4	circRNA_6140|Chr9:61935380_61937535_-	0.948203007	0.003955
XM_006530294.3	circRNA_0845|Chr11:29470315_29477458_+	0.948044216	0.003979
NM_009381.3	circRNA_0157|Chr1:66801049_66802168_-	0.948016062	0.003983
XM_006525075.3	circRNA_5311|Chr6:145147636_145149357_+	0.94794419	0.003994
NM_178692.3	circRNA_3702|Chr2:143830084_143832057_-	0.947938548	0.003995
NM_001142804.1	circRNA_3702|Chr2:143830084_143832057_-	0.947932805	0.003996
NM_010090.2	circRNA_6446|ChrX:42217416_42250037_+	0.947913623	0.003999
XM_006522506.3	circRNA_5639|Chr7:132771578_132779385_-	0.947863085	0.004007
NM_001291145.1	circRNA_2951|Chr18:39120119_39150118_+	0.947558762	0.004053
XM_006538830.1	circRNA_3954|Chr3:88346445_88349444_+	0.947550116	0.004054
NM_019759.2	circRNA_3702|Chr2:143830084_143832057_-	0.947498004	0.004062
NM_001286743.1	circRNA_3954|Chr3:88346445_88349444_+	0.947369354	0.004082
NM_031183.2	circRNA_0611|Chr10:75274017_75279961_+	0.947231552	0.004103
NM_025758.4	circRNA_1042|Chr11:80385854_80403408_+	0.947162103	0.004114
NM_009020.3	circRNA_2888|Chr18:21010677_21020999_+	0.947062443	0.004129
XM_006530131.1	circRNA_0537|Chr10:43393871_43395692_+	0.946983665	0.004142
NM_023456.3	circRNA_3702|Chr2:143830084_143832057_-	0.946851388	0.004162
XM_006523711.3	circRNA_3702|Chr2:143830084_143832057_-	0.946806835	0.004169
NM_001134300.2	circRNA_0537|Chr10:43393871_43395692_+	0.94672187	0.004182
NM_178782.4	circRNA_6390|Chr9:121727316_121731565_+	0.946530885	0.004212
NM_028973.2	circRNA_0020|Chr1:10315205_10321709_+	0.946235552	0.004258
NM_008524.2	circRNA_3239|Chr19:45635735_45640521_-	0.946106446	0.004279
XM_006515096.2	circRNA_3832|Chr2:169883526_169886459_+	0.946104745	0.004279
NM_173427.2	circRNA_4728|Chr5:97027831_97045167_+	0.94596848	0.0043
XM_006540596.2	circRNA_2951|Chr18:39120119_39150118_+	0.945945886	0.004304
NM_009930.2	circRNA_0020|Chr1:10315205_10321709_+	0.945876915	0.004315
XM_006530353.3	circRNA_3832|Chr2:169883526_169886459_+	0.945787512	0.004329
XM_006540762.3	circRNA_6412|ChrMT:13917_14141_-	0.94576331	0.004333
NM_009930.2	circRNA_6140|Chr9:61935380_61937535_-	0.94575866	0.004333
NM_011854.2	circRNA_3954|Chr3:88346445_88349444_+	0.945741323	0.004336
XM_006521796.1	circRNA_0020|Chr1:10315205_10321709_+	0.945734525	0.004337
NM_001310705.1	circRNA_3702|Chr2:143830084_143832057_-	0.94567557	0.004347
NM_010140.3	circRNA_3702|Chr2:143830084_143832057_-	0.945636871	0.004353
XM_006501237.3	circRNA_3155|Chr19:25061108_25065459_+	0.945614203	0.004356
NM_146063.1	circRNA_0376|Chr1:172173943_172187460_+	0.945590951	0.00436
NM_007555.4	circRNA_3702|Chr2:143830084_143832057_-	0.945583239	0.004361
NM_007549.2	circRNA_6140|Chr9:61935380_61937535_-	0.945553872	0.004366
NM_009332.3	circRNA_3832|Chr2:169883526_169886459_+	0.945526494	0.00437
XM_006520437.2	circRNA_0501|Chr10:25283893_25289730_-	0.945483938	0.004377
XM_006520437.2	circRNA_6109|Chr9:57056714_57057861_+	0.945483938	0.004377
XM_006496277.3	circRNA_2951|Chr18:39120119_39150118_+	0.945453471	0.004382
XM_017312731.1	circRNA_4728|Chr5:97027831_97045167_+	0.94542562	0.004386
XM_006537508.2	circRNA_6446|ChrX:42217416_42250037_+	0.945386892	0.004392
NM_001099314.1	circRNA_0845|Chr11:29470315_29477458_+	0.945362819	0.004396
NM_011430.3	circRNA_0020|Chr1:10315205_10321709_+	0.94535267	0.004398
NM_177743.5	circRNA_6140|Chr9:61935380_61937535_-	0.945346166	0.004399
NM_010742.1	circRNA_3239|Chr19:45635735_45640521_-	0.945331556	0.004401
XM_006513165.1	circRNA_0376|Chr1:172173943_172187460_+	0.945318206	0.004403
XM_006532962.2	circRNA_6390|Chr9:121727316_121731565_+	0.945315874	0.004404
XM_006533653.3	circRNA_1399|Chr12:72783683_72786789_+	0.945286874	0.004408
XM_006524407.3	circRNA_0961|Chr11:67192403_67254873_+	0.94527818	0.00441
NM_001291483.1	circRNA_0020|Chr1:10315205_10321709_+	0.945268925	0.004411
NM_001317365.1	circRNA_3954|Chr3:88346445_88349444_+	0.945232075	0.004417
XM_017320219.1	circRNA_0961|Chr11:67192403_67254873_+	0.945057399	0.004445

NM_023670.3	circRNA_3239|Chr19:45635735_45640521_-	0.944998161	0.004455
XM_017322026.1	circRNA_3155|Chr19:25061108_25065459_+	0.944953294	0.004462
NM_001164593.1	circRNA_3155|Chr19:25061108_25065459_+	0.944940634	0.004464
NM_031180.2	circRNA_2951|Chr18:39120119_39150118_+	0.94491112	0.004469
XM_006496884.3	circRNA_6446|ChrX:42217416_42250037_+	0.944834309	0.004481
NM_009675.2	circRNA_0501|Chr10:25283893_25289730_-	0.944817337	0.004484
NM_009675.2	circRNA_6109|Chr9:57056714_57057861_+	0.944817337	0.004484
NM_001301295.1	circRNA_6446|ChrX:42217416_42250037_+	0.944750886	0.004494
XM_006532232.3	circRNA_6390|Chr9:121727316_121731565_+	0.944721832	0.004499
XM_006520023.2	circRNA_0020|Chr1:10315205_10321709_+	0.944602452	0.004518
NM_172907.3	circRNA_4662|Chr5:65301933_65303208_-	0.94445232	0.004543
NM_001077403.1	circRNA_0537|Chr10:43393871_43395692_+	0.944423889	0.004547
XM_017312731.1	circRNA_4662|Chr5:65301933_65303208_-	0.944360594	0.004557
XM_006511007.3	circRNA_6390|Chr9:121727316_121731565_+	0.944344992	0.00456
XM_001481172.6	circRNA_3239|Chr19:45635735_45640521_-	0.944244474	0.004576
NM_001204201.1	circRNA_5311|Chr6:145147636_145149357_+	0.944171827	0.004588
XM_017316439.1	circRNA_3155|Chr19:25061108_25065459_+	0.944163311	0.00459
NM_177709.3	circRNA_3702|Chr2:143830084_143832057_-	0.943935879	0.004627
NM_153801.3	circRNA_3832|Chr2:169883526_169886459_+	0.943928831	0.004628
XM_006529759.1	circRNA_3155|Chr19:25061108_25065459_+	0.943885585	0.004635
XM_006511221.3	circRNA_5990|Chr9:3475483_3477962_+	0.943874888	0.004637
NM_009518.2	circRNA_4987|Chr6:4529068_4531010_+	0.943823929	0.004645
NM_001290273.1	circRNA_5311|Chr6:145147636_145149357_+	0.943808874	0.004647
XM_006500919.3	circRNA_3239|Chr19:45635735_45640521_-	0.943799284	0.004649
XM_011244046.2	circRNA_3702|Chr2:143830084_143832057_-	0.943494592	0.004699
NM_021475.2	circRNA_3239|Chr19:45635735_45640521_-	0.943438696	0.004708
XM_006513165.1	circRNA_4728|Chr5:97027831_97045167_+	0.943312804	0.004729
NM_018866.2	circRNA_6140|Chr9:61935380_61937535_-	0.943284663	0.004734
XM_011248919.2	circRNA_6390|Chr9:121727316_121731565_+	0.943170846	0.004753
XM_006511221.3	circRNA_3832|Chr2:169883526_169886459_+	0.943117806	0.004761
NM_001013784.1	circRNA_5811|Chr8:71992832_71998295_-	0.943069409	0.004769
XM_006503371.2	circRNA_6390|Chr9:121727316_121731565_+	0.94300064	0.004781
XM_006529727.2	circRNA_3832|Chr2:169883526_169886459_+	0.942972752	0.004785
XM_006498287.3	circRNA_3954|Chr3:88346445_88349444_+	0.942900618	0.004797
NM_144936.1	circRNA_0020|Chr1:10315205_10321709_+	0.942854411	0.004805
XM_006514444.1	circRNA_0961|Chr11:67192403_67254873_+	0.942791866	0.004816
NM_008423.2	circRNA_0961|Chr11:67192403_67254873_+	0.942784596	0.004817
NM_009223.3	circRNA_3239|Chr19:45635735_45640521_-	0.942757453	0.004821
NM_145741.2	circRNA_3702|Chr2:143830084_143832057_-	0.942520286	0.004861
NM_178692.3	circRNA_0157|Chr1:66801049_66802168_-	0.942275404	0.004902
NM_145635.2	circRNA_0020|Chr1:10315205_10321709_+	0.942205956	0.004914
NM_133643.4	circRNA_3702|Chr2:143830084_143832057_-	0.942068875	0.004937
NM_007555.4	circRNA_0501|Chr10:25283893_25289730_-	0.9420593	0.004938
NM_007555.4	circRNA_6109|Chr9:57056714_57057861_+	0.9420593	0.004938
NM_001282961.1	circRNA_0501|Chr10:25283893_25289730_-	0.942047864	0.00494
NM_001282961.1	circRNA_6109|Chr9:57056714_57057861_+	0.942047864	0.00494
NM_001081224.2	circRNA_2951|Chr18:39120119_39150118_+	0.941964051	0.004955
NM_019397.3	circRNA_3702|Chr2:143830084_143832057_-	0.9419593	0.004955
XM_011248201.2	circRNA_0537|Chr10:43393871_43395692_+	0.941897801	0.004966
XM_011240476.2	circRNA_4487|Chr4:149156607_149161694_-	0.941837486	0.004976
NM_008966.3	circRNA_0020|Chr1:10315205_10321709_+	0.941809085	0.004981
NM_010279.3	circRNA_3239|Chr19:45635735_45640521_-	0.941661924	0.005006
NM_001002896.2	circRNA_3155|Chr19:25061108_25065459_+	0.941507407	0.005032
NM_030206.4	circRNA_2951|Chr18:39120119_39150118_+	0.941448743	0.005042
XM_006510077.3	circRNA_3155|Chr19:25061108_25065459_+	0.941438404	0.005044
NM_029844.3	circRNA_3155|Chr19:25061108_25065459_+	0.941411017	0.005048
NM_009020.3	circRNA_3276|Chr19:60563162_60564294_-	0.941366928	0.005056
NM_008760.4	circRNA_3702|Chr2:143830084_143832057_-	0.941329689	0.005062
XM_017315376.1	circRNA_3155|Chr19:25061108_25065459_+	0.941329324	0.005062

NM_008012.1	circRNA_4055|Chr3:126796838_126798280_+	0.941139383	0.005095
XM_011246309.2	circRNA_2951|Chr18:39120119_39150118_+	0.94107087	0.005107
NM_019397.3	circRNA_6446|ChrX:42217416_42250037_+	0.941067818	0.005107
XM_006526449.3	circRNA_4635|Chr5:43758222_43773659_-	0.941034102	0.005113
XM_006524328.3	circRNA_0537|Chr10:43393871_43395692_+	0.941011588	0.005117
NM_010340.2	circRNA_0537|Chr10:43393871_43395692_+	0.941000502	0.005119
XM_017313038.1	circRNA_3702|Chr2:143830084_143832057_-	0.940919673	0.005133
XM_006533653.3	circRNA_3425|Chr2:37624173_37627526_-	0.940866129	0.005142
XM_006533653.3	circRNA_3607|Chr2:119057281_119064097_+	0.940866129	0.005142
XM_006530585.3	circRNA_6390|Chr9:121727316_121731565_+	0.940851167	0.005144
NM_024283.3	circRNA_0020|Chr1:10315205_10321709_+	0.940808895	0.005152
XM_006498901.3	circRNA_0020|Chr1:10315205_10321709_+	0.940683658	0.005173
NM_001290822.1	circRNA_2951|Chr18:39120119_39150118_+	0.940673144	0.005175
XM_006509966.3	circRNA_0537|Chr10:43393871_43395692_+	0.940664664	0.005177
XM_017320219.1	circRNA_4926|Chr5:143080224_143081077_-	0.940663881	0.005177
XM_006504999.1	circRNA_0157|Chr1:66801049_66802168_-	0.940660825	0.005177
NM_001171187.1	circRNA_2951|Chr18:39120119_39150118_+	0.940652439	0.005179
NM_133643.4	circRNA_3276|Chr19:60563162_60564294_-	0.940569973	0.005193
XM_006506624.2	circRNA_6446|ChrX:42217416_42250037_+	0.940550694	0.005196
XM_017320093.1	circRNA_3702|Chr2:143830084_143832057_-	0.940455177	0.005213
NM_001190451.2	circRNA_6140|Chr9:61935380_61937535_-	0.940426634	0.005218
NM_025865.2	circRNA_0962|Chr11:67194348_67220998_+	0.94038108	0.005226
XM_006524777.3	circRNA_0611|Chr10:75274017_75279961_+	0.940344524	0.005232
NM_009518.2	circRNA_1632|Chr13:42055400_42055542_+	0.940320345	0.005236
XM_006514444.1	circRNA_6390|Chr9:121727316_121731565_+	0.940319803	0.005236
XM_006523711.3	circRNA_6140|Chr9:61935380_61937535_-	0.940299422	0.00524
NM_144945.3	circRNA_6390|Chr9:121727316_121731565_+	0.940127501	0.00527
XM_006511221.3	circRNA_0845|Chr11:29470315_29477458_+	0.940105924	0.005274
XM_006529753.2	circRNA_0537|Chr10:43393871_43395692_+	0.940049949	0.005283
NM_021342.1	circRNA_4055|Chr3:126796838_126798280_+	0.939967668	0.005298
NM_009135.2	circRNA_2888|Chr18:21010677_21020999_+	0.93993465	0.005303
NM_001291930.1	circRNA_2888|Chr18:21010677_21020999_+	0.939901499	0.005309
NM_001039047.1	circRNA_0537|Chr10:43393871_43395692_+	0.939826279	0.005322
NM_001190448.1	circRNA_6140|Chr9:61935380_61937535_-	0.939812483	0.005325
XM_011248576.2	circRNA_3832|Chr2:169883526_169886459_+	0.939779384	0.005331
NM_001097617.1	circRNA_3702|Chr2:143830084_143832057_-	0.939747127	0.005336
NM_008940.3	circRNA_0020|Chr1:10315205_10321709_+	0.939666883	0.00535
NM_146241.2	circRNA_0611|Chr10:75274017_75279961_+	0.939609251	0.00536
NM_011082.3	circRNA_4728|Chr5:97027831_97045167_+	0.939533488	0.005374
XM_006503371.2	circRNA_5074|Chr6:40685277_40747150_+	0.93953188	0.005374
NM_009381.3	circRNA_3702|Chr2:143830084_143832057_-	0.938875808	0.00549
NM_020001.2	circRNA_5311|Chr6:145147636_145149357_+	0.938836158	0.005497
NM_016659.3	circRNA_5311|Chr6:145147636_145149357_+	0.938834776	0.005497
NM_148932.2	circRNA_6390|Chr9:121727316_121731565_+	0.938826018	0.005499
XM_017322365.1	circRNA_6140|Chr9:61935380_61937535_-	0.938785392	0.005506
NM_010714.3	circRNA_3239|Chr19:45635735_45640521_-	0.938697237	0.005522
NM_007555.4	circRNA_2951|Chr18:39120119_39150118_+	0.938636648	0.005533
NM_173427.2	circRNA_0020|Chr1:10315205_10321709_+	0.938559527	0.005546
NM_001305585.1	circRNA_0157|Chr1:66801049_66802168_-	0.938342616	0.005585
NM_177743.5	circRNA_3702|Chr2:143830084_143832057_-	0.938293998	0.005594
XM_017316439.1	circRNA_3239|Chr19:45635735_45640521_-	0.938286949	0.005595
NM_010608.2	circRNA_3276|Chr19:60563162_60564294_-	0.938280287	0.005596
NM_009514.4	circRNA_3155|Chr19:25061108_25065459_+	0.938242287	0.005603
NM_030206.4	circRNA_2888|Chr18:21010677_21020999_+	0.938221877	0.005607
XM_006516224.3	circRNA_6390|Chr9:121727316_121731565_+	0.938197495	0.005611
NM_001243916.1	circRNA_0913|Chr11:54005394_54014456_-	0.938179496	0.005615
XM_017315376.1	circRNA_3239|Chr19:45635735_45640521_-	0.938175654	0.005615
NM_009605.4	circRNA_4055|Chr3:126796838_126798280_+	0.938168215	0.005617
NM_001029877.3	circRNA_4926|Chr5:143080224_143081077_-	0.938140815	0.005621

NM_007529.2	circRNA_0611|Chr10:75274017_75279961_+	0.938116495	0.005626
NM_020001.2	circRNA_6140|Chr9:61935380_61937535_-	0.93802719	0.005642
NM_001033221.3	circRNA_3954|Chr3:88346445_88349444_+	0.937945109	0.005657
NM_025540.2	circRNA_0962|Chr11:67194348_67220998_+	0.937924107	0.005661
NM_178692.3	circRNA_2888|Chr18:21010677_21020999_+	0.937836828	0.005676
XM_006529727.2	circRNA_0537|Chr10:43393871_43395692_+	0.937827255	0.005678
NM_001302257.1	circRNA_1632|Chr13:42055400_42055542_+	0.937678377	0.005705
NM_178753.4	circRNA_2951|Chr18:39120119_39150118_+	0.937556884	0.005727
XM_006533479.2	circRNA_4055|Chr3:126796838_126798280_+	0.937493353	0.005739
NM_009605.4	circRNA_5311|Chr6:145147636_145149357_+	0.937486218	0.00574
NM_144544.2	circRNA_0913|Chr11:54005394_54014456_-	0.937269302	0.005779
NM_024406.2	circRNA_0157|Chr1:66801049_66802168_-	0.937239395	0.005785
NM_008479.2	circRNA_3003|Chr18:67545615_67587859_-	0.937188011	0.005794
NM_008264.1	circRNA_5311|Chr6:145147636_145149357_+	0.937172991	0.005797
NM_018866.2	circRNA_3702|Chr2:143830084_143832057_-	0.937162366	0.005799
NM_178753.4	circRNA_3276|Chr19:60563162_60564294_-	0.93715738	0.0058
NM_145741.2	circRNA_2888|Chr18:21010677_21020999_+	0.937117741	0.005807
NM_133167.3	circRNA_0537|Chr10:43393871_43395692_+	0.93708238	0.005813
XM_006507725.2	circRNA_6390|Chr9:121727316_121731565_+	0.937043181	0.005821
XM_006526449.3	circRNA_3003|Chr18:67545615_67587859_-	0.936885356	0.005849
NM_010340.2	circRNA_3954|Chr3:88346445_88349444_+	0.936869033	0.005852
NM_016982.2	circRNA_0376|Chr1:172173943_172187460_+	0.93681632	0.005862
NM_009675.2	circRNA_2888|Chr18:21010677_21020999_+	0.93680152	0.005865
XM_006529456.3	circRNA_3702|Chr2:143830084_143832057_-	0.936699247	0.005884
NM_009605.4	circRNA_3155|Chr19:25061108_25065459_+	0.936694424	0.005885
NM_019397.3	circRNA_6140|Chr9:61935380_61937535_-	0.936634201	0.005896
NM_009020.3	circRNA_0501|Chr10:25283893_25289730_-	0.936445681	0.00593
NM_009020.3	circRNA_6109|Chr9:57056714_57057861_+	0.936445681	0.00593
NM_145141.2	circRNA_0157|Chr1:66801049_66802168_-	0.936420887	0.005935
NM_207231.1	circRNA_3239|Chr19:45635735_45640521_-	0.936398809	0.005939
NM_175260.2	circRNA_0537|Chr10:43393871_43395692_+	0.936387215	0.005941
XM_006515096.2	circRNA_0845|Chr11:29470315_29477458_+	0.936369043	0.005945
XM_001481172.6	circRNA_5311|Chr6:145147636_145149357_+	0.93634868	0.005948
XM_011240149.1	circRNA_2313|Chr15:93452117_93465245_+	0.936259928	0.005965
NM_008966.3	circRNA_6140|Chr9:61935380_61937535_-	0.936216213	0.005973
XM_011250176.1	circRNA_3832|Chr2:169883526_169886459_+	0.936136272	0.005988
NM_144539.5	circRNA_6140|Chr9:61935380_61937535_-	0.936122108	0.00599
NM_015744.4	circRNA_6140|Chr9:61935380_61937535_-	0.936113474	0.005992
NM_001001178.1	circRNA_0020|Chr1:10315205_10321709_+	0.936041229	0.006005
NM_023716.2	circRNA_3155|Chr19:25061108_25065459_+	0.936004988	0.006012
NM_001082546.1	circRNA_5311|Chr6:145147636_145149357_+	0.935953017	0.006022
NM_011254.5	circRNA_3239|Chr19:45635735_45640521_-	0.935853962	0.00604
NM_001313939.1	circRNA_4055|Chr3:126796838_126798280_+	0.935798037	0.006051
NM_001110009.2	circRNA_4728|Chr5:97027831_97045167_+	0.935795223	0.006051
NM_010714.3	circRNA_3155|Chr19:25061108_25065459_+	0.935686294	0.006071
NM_016697.3	circRNA_0157|Chr1:66801049_66802168_-	0.935661885	0.006076
NM_008623.5	circRNA_2888|Chr18:21010677_21020999_+	0.935627193	0.006082
NM_001290273.1	circRNA_4055|Chr3:126796838_126798280_+	0.935614141	0.006085
XM_017320735.1	circRNA_0501|Chr10:25283893_25289730_-	0.935480214	0.00611
XM_017320735.1	circRNA_6109|Chr9:57056714_57057861_+	0.935480214	0.00611
NM_001310648.1	circRNA_4055|Chr3:126796838_126798280_+	0.935463722	0.006113
NM_177743.5	circRNA_0020|Chr1:10315205_10321709_+	0.935362106	0.006132
NM_031183.2	circRNA_0157|Chr1:66801049_66802168_-	0.935233652	0.006156
NM_001317365.1	circRNA_5811|Chr8:71992832_71998295_-	0.935225666	0.006158
NM_001302471.1	circRNA_3832|Chr2:169883526_169886459_+	0.935192274	0.006164
NM_013560.2	circRNA_6390|Chr9:121727316_121731565_+	0.935172521	0.006168
NM_009381.3	circRNA_0501|Chr10:25283893_25289730_-	0.935172338	0.006168
NM_009381.3	circRNA_6109|Chr9:57056714_57057861_+	0.935172338	0.006168
NM_029823.2	circRNA_4662|Chr5:65301933_65303208_-	0.935011521	0.006198

NM_008264.1	circRNA_3155|Chr19:25061108_25065459_+	0.935003252	0.0062
XM_006504999.1	circRNA_0376|Chr1:172173943_172187460_+	0.934981788	0.006204
NM_019866.1	circRNA_6446|ChrX:42217416_42250037_+	0.934952377	0.006209
NM_010340.2	circRNA_6390|Chr9:121727316_121731565_+	0.934940158	0.006211
XM_017322211.1	circRNA_6390|Chr9:121727316_121731565_+	0.934938103	0.006212
NM_001159424.2	circRNA_3702|Chr2:143830084_143832057_-	0.93490122	0.006219
NM_001002896.2	circRNA_3702|Chr2:143830084_143832057_-	0.93484479	0.00623
NM_153801.3	circRNA_0961|Chr11:67192403_67254873_+	0.934829196	0.006232
NM_011658.2	circRNA_5311|Chr6:145147636_145149357_+	0.934826339	0.006233
XM_017317034.1	circRNA_6390|Chr9:121727316_121731565_+	0.934763817	0.006245
XM_006521931.1	circRNA_4055|Chr3:126796838_126798280_+	0.934677364	0.006261
NM_001039047.1	circRNA_3954|Chr3:88346445_88349444_+	0.934638403	0.006269
NM_144936.1	circRNA_4662|Chr5:65301933_65303208_-	0.934530452	0.006289
NM_010608.2	circRNA_3702|Chr2:143830084_143832057_-	0.934433925	0.006307
NM_024283.3	circRNA_4662|Chr5:65301933_65303208_-	0.934331559	0.006327
NM_013468.3	circRNA_0537|Chr10:43393871_43395692_+	0.934315623	0.00633
XM_006504999.1	circRNA_4662|Chr5:65301933_65303208_-	0.934306559	0.006332
NM_013459.3	circRNA_6446|ChrX:42217416_42250037_+	0.934302741	0.006332
NM_001291483.1	circRNA_4055|Chr3:126796838_126798280_+	0.934291347	0.006335
NM_007881.4	circRNA_6390|Chr9:121727316_121731565_+	0.934261519	0.00634
NM_027416.3	circRNA_4662|Chr5:65301933_65303208_-	0.934228956	0.006346
NM_016982.2	circRNA_0020|Chr1:10315205_10321709_+	0.934192926	0.006353
XM_006518921.3	circRNA_3276|Chr19:60563162_60564294_-	0.934136053	0.006364
XM_006503323.3	circRNA_2888|Chr18:21010677_21020999_+	0.934116482	0.006368
NM_207231.1	circRNA_3155|Chr19:25061108_25065459_+	0.934013388	0.006388
XM_017320219.1	circRNA_3832|Chr2:169883526_169886459_+	0.933940445	0.006402
NM_175692.3	circRNA_4728|Chr5:97027831_97045167_+	0.9339253	0.006405
XM_006517308.3	circRNA_0611|Chr10:75274017_75279961_+	0.933879273	0.006413
NM_011957.2	circRNA_3832|Chr2:169883526_169886459_+	0.933778894	0.006433
NM_023670.3	circRNA_3702|Chr2:143830084_143832057_-	0.933659897	0.006456
NM_008966.3	circRNA_3155|Chr19:25061108_25065459_+	0.933640386	0.006459
XM_006516928.2	circRNA_3702|Chr2:143830084_143832057_-	0.93344368	0.006497
NM_001077403.1	circRNA_3954|Chr3:88346445_88349444_+	0.933413594	0.006503
NM_001204959.1	circRNA_0157|Chr1:66801049_66802168_-	0.93340077	0.006505
XM_006529829.3	circRNA_3276|Chr19:60563162_60564294_-	0.933274532	0.00653
NM_001082547.1	circRNA_4055|Chr3:126796838_126798280_+	0.933210344	0.006542
XM_011241948.2	circRNA_0020|Chr1:10315205_10321709_+	0.933194885	0.006545
NM_001291930.1	circRNA_0501|Chr10:25283893_25289730_-	0.933183198	0.006548
NM_001291930.1	circRNA_6109|Chr9:57056714_57057861_+	0.933183198	0.006548
NM_001190451.2	circRNA_2951|Chr18:39120119_39150118_+	0.933041438	0.006575
NM_011724.3	circRNA_0961|Chr11:67192403_67254873_+	0.932991639	0.006585
XM_006540762.3	circRNA_1980|Chr14:50951404_50963869_+	0.932907822	0.006601
NM_009675.2	circRNA_3702|Chr2:143830084_143832057_-	0.932889328	0.006605
NM_009381.3	circRNA_0020|Chr1:10315205_10321709_+	0.932842312	0.006614
NM_146125.2	circRNA_0376|Chr1:172173943_172187460_+	0.932814572	0.006619
NM_011254.5	circRNA_3155|Chr19:25061108_25065459_+	0.93278244	0.006625
NM_001290993.1	circRNA_3276|Chr19:60563162_60564294_-	0.932732461	0.006635
NM_178715.3	circRNA_3239|Chr19:45635735_45640521_-	0.932727102	0.006636
NM_028472.2	circRNA_6446|ChrX:42217416_42250037_+	0.93265789	0.00665
NM_146063.1	circRNA_0157|Chr1:66801049_66802168_-	0.932458065	0.006689
NM_028472.2	circRNA_3702|Chr2:143830084_143832057_-	0.932421612	0.006696
XM_006507942.2	circRNA_3702|Chr2:143830084_143832057_-	0.932389488	0.006702
NM_031180.2	circRNA_0611|Chr10:75274017_75279961_+	0.932377653	0.006705
NM_001024731.2	circRNA_3239|Chr19:45635735_45640521_-	0.932372452	0.006706
NM_001302257.1	circRNA_5367|Chr7:28990988_28991325_+	0.932186851	0.006742
NM_009381.3	circRNA_6140|Chr9:61935380_61937535_-	0.932028643	0.006773
NM_025288.2	circRNA_5311|Chr6:145147636_145149357_+	0.931737349	0.006831
NM_015744.4	circRNA_0157|Chr1:66801049_66802168_-	0.931705754	0.006837
XM_006511102.2	circRNA_4728|Chr5:97027831_97045167_+	0.931693467	0.006839

NM_011581.3	circRNA_3239|Chr19:45635735_45640521_-	0.931663889	0.006845
NM_001002896.2	circRNA_4055|Chr3:126796838_126798280_+	0.931634635	0.006851
XM_017317978.1	circRNA_6446|ChrX:42217416_42250037_+	0.931626153	0.006853
NM_031180.2	circRNA_0501|Chr10:25283893_25289730_-	0.931622184	0.006853
NM_031180.2	circRNA_6109|Chr9:57056714_57057861_+	0.931622184	0.006853
XM_006538456.3	circRNA_6390|Chr9:121727316_121731565_+	0.931613693	0.006855
NM_001002896.2	circRNA_6140|Chr9:61935380_61937535_-	0.931517902	0.006874
XM_006505757.3	circRNA_3239|Chr19:45635735_45640521_-	0.931501797	0.006877
NM_145226.2	circRNA_0845|Chr11:29470315_29477458_+	0.931499997	0.006878
NM_001004357.2	circRNA_3155|Chr19:25061108_25065459_+	0.931475453	0.006883
NM_025758.4	circRNA_4635|Chr5:43758222_43773659_-	0.931424058	0.006893
NM_020001.2	circRNA_4662|Chr5:65301933_65303208_-	0.931417282	0.006894
NM_027416.3	circRNA_4055|Chr3:126796838_126798280_+	0.931376532	0.006902
NM_009381.3	circRNA_2951|Chr18:39120119_39150118_+	0.931351031	0.006907
XM_011246128.2	circRNA_4987|Chr6:4529068_4531010_+	0.931286408	0.00692
NM_008760.4	circRNA_6140|Chr9:61935380_61937535_-	0.931268796	0.006924
NM_028973.2	circRNA_6140|Chr9:61935380_61937535_-	0.931190485	0.006939
NM_029844.3	circRNA_0157|Chr1:66801049_66802168_-	0.931165174	0.006944
XM_006520023.2	circRNA_5311|Chr6:145147636_145149357_+	0.931092687	0.006959
NM_001291145.1	circRNA_0020|Chr1:10315205_10321709_+	0.931014019	0.006974
NM_009135.2	circRNA_0501|Chr10:25283893_25289730_-	0.930968713	0.006983
NM_009135.2	circRNA_6109|Chr9:57056714_57057861_+	0.930968713	0.006983
NM_001270475.1	circRNA_3832|Chr2:169883526_169886459_+	0.93093552	0.00699
NM_028472.2	circRNA_6140|Chr9:61935380_61937535_-	0.930879306	0.007001
NM_001097617.1	circRNA_6140|Chr9:61935380_61937535_-	0.930878315	0.007002
XM_006516510.1	circRNA_4728|Chr5:97027831_97045167_+	0.930761658	0.007025
NM_009518.2	circRNA_0611|Chr10:75274017_75279961_+	0.930725014	0.007032
XM_006496949.3	circRNA_3702|Chr2:143830084_143832057_-	0.930716927	0.007034
NM_199029.2	circRNA_6390|Chr9:121727316_121731565_+	0.930659208	0.007046
NM_007792.4	circRNA_3239|Chr19:45635735_45640521_-	0.930644567	0.007048
XM_011239558.2	circRNA_0845|Chr11:29470315_29477458_+	0.930614788	0.007054
NM_010279.3	circRNA_0611|Chr10:75274017_75279961_+	0.93055231	0.007067
NM_134072.1	circRNA_3155|Chr19:25061108_25065459_+	0.930534829	0.007071
XM_011240779.2	circRNA_3702|Chr2:143830084_143832057_-	0.930489944	0.00708
XM_006503080.3	circRNA_3832|Chr2:169883526_169886459_+	0.930372097	0.007103
NM_010140.3	circRNA_0501|Chr10:25283893_25289730_-	0.930338105	0.00711
NM_010140.3	circRNA_6109|Chr9:57056714_57057861_+	0.930338105	0.00711
NM_009930.2	circRNA_3702|Chr2:143830084_143832057_-	0.930102577	0.007158
XM_017314944.1	circRNA_0501|Chr10:25283893_25289730_-	0.929980044	0.007183
XM_017314944.1	circRNA_6109|Chr9:57056714_57057861_+	0.929980044	0.007183
XM_006508867.2	circRNA_0376|Chr1:172173943_172187460_+	0.929969557	0.007185
NM_008185.3	circRNA_3155|Chr19:25061108_25065459_+	0.929944612	0.00719
NM_001198955.1	circRNA_0537|Chr10:43393871_43395692_+	0.929942794	0.00719
NM_020025.4	circRNA_4728|Chr5:97027831_97045167_+	0.929814621	0.007216
NM_001310636.1	circRNA_4987|Chr6:4529068_4531010_+	0.929812359	0.007217
NM_010340.2	circRNA_5990|Chr9:3475483_3477962_+	0.929748533	0.00723
XM_006530294.3	circRNA_3954|Chr3:88346445_88349444_+	0.929687601	0.007242
NM_001110009.2	circRNA_0376|Chr1:172173943_172187460_+	0.929686902	0.007242
XM_006513165.1	circRNA_0020|Chr1:10315205_10321709_+	0.929679533	0.007244
XM_006515096.2	circRNA_0537|Chr10:43393871_43395692_+	0.929629524	0.007254
NM_010055.3	circRNA_1042|Chr11:80385854_80403408_+	0.92956101	0.007268
XM_006534171.3	circRNA_6446|ChrX:42217416_42250037_+	0.929544017	0.007271
NM_001007570.2	circRNA_3832|Chr2:169883526_169886459_+	0.929536155	0.007273
NM_001097617.1	circRNA_0020|Chr1:10315205_10321709_+	0.929533591	0.007273
NM_145141.2	circRNA_6446|ChrX:42217416_42250037_+	0.929521495	0.007276
NM_020001.2	circRNA_0020|Chr1:10315205_10321709_+	0.929418418	0.007297
NM_001110322.1	circRNA_3702|Chr2:143830084_143832057_-	0.92940094	0.0073
NM_173437.2	circRNA_6390|Chr9:121727316_121731565_+	0.929374442	0.007306
NM_015744.4	circRNA_3702|Chr2:143830084_143832057_-	0.929332488	0.007314

XM_006515787.3	circRNA_5367	Chr7:28990988_28991325_+	0.929323875	0.007316
XM_006508885.1	circRNA_4728	Chr5:97027831_97045167_+	0.92927565	0.007326
NM_145210.2	circRNA_1042	Chr11:80385854_80403408_+	0.929254623	0.00733
NM_011658.2	circRNA_0020	Chr1:10315205_10321709_+	0.92925109	0.007331
NM_181315.4	circRNA_4055	Chr3:126796838_126798280_+	0.929122062	0.007357
XM_006527452.3	circRNA_3702	Chr2:143830084_143832057_-	0.929057653	0.007371
NM_001161541.1	circRNA_6140	Chr9:61935380_61937535_-	0.929045288	0.007373
NM_001033445.2	circRNA_0537	Chr10:43393871_43395692_+	0.929029978	0.007376
XM_017314347.1	circRNA_6446	ChrX:42217416_42250037_+	0.929023586	0.007378
NM_024406.2	circRNA_0020	Chr1:10315205_10321709_+	0.928884855	0.007406
NM_001081224.2	circRNA_0611	Chr10:75274017_75279961_+	0.928875098	0.007408
NM_205810.4	circRNA_5367	Chr7:28990988_28991325_+	0.928848637	0.007414
NM_001291930.1	circRNA_0611	Chr10:75274017_75279961_+	0.928790924	0.007426
XM_006516928.2	circRNA_6140	Chr9:61935380_61937535_-	0.928769941	0.00743
NM_145141.2	circRNA_4055	Chr3:126796838_126798280_+	0.928647643	0.007455
XM_006530294.3	circRNA_1042	Chr11:80385854_80403408_+	0.928638656	0.007457
XM_006525028.2	circRNA_2888	Chr18:21010677_21020999_+	0.928532486	0.007479
NM_001313939.1	circRNA_6446	ChrX:42217416_42250037_+	0.928495972	0.007486
NM_018866.2	circRNA_5311	Chr6:145147636_145149357_+	0.928477398	0.00749
XM_006533452.3	circRNA_3276	Chr19:60563162_60564294_-	0.928454201	0.007495
NM_019626.3	circRNA_3155	Chr19:25061108_25065459_+	0.928374129	0.007512
XM_006501237.3	circRNA_3239	Chr19:45635735_45640521_-	0.928282089	0.007531
NM_177578.4	circRNA_3155	Chr19:25061108_25065459_+	0.928213455	0.007545
NM_027416.3	circRNA_6140	Chr9:61935380_61937535_-	0.928196279	0.007549
XM_006503323.3	circRNA_0501	Chr10:25283893_25289730_-	0.928155425	0.007557
XM_006503323.3	circRNA_6109	Chr9:57056714_57057861_+	0.928155425	0.007557
NM_001204959.1	circRNA_4055	Chr3:126796838_126798280_+	0.928078579	0.007573
XM_006508885.1	circRNA_5639	Chr7:132771578_132779385_-	0.927932859	0.007603
XM_006534520.3	circRNA_6390	Chr9:121727316_121731565_+	0.92792127	0.007606
NM_026979.5	circRNA_3702	Chr2:143830084_143832057_-	0.927577168	0.007678
NM_053080.3	circRNA_2888	Chr18:21010677_21020999_+	0.927536927	0.007686
NM_007641.5	circRNA_6140	Chr9:61935380_61937535_-	0.927507126	0.007692
NM_032400.2	circRNA_2951	Chr18:39120119_39150118_+	0.927501921	0.007693
NM_001310636.1	circRNA_1632	Chr13:42055400_42055542_+	0.927430419	0.007708
XM_006518339.3	circRNA_0157	Chr1:66801049_66802168_-	0.927367521	0.007722
XM_006522398.3	circRNA_6140	Chr9:61935380_61937535_-	0.92736726	0.007722
NM_177371.3	circRNA_3702	Chr2:143830084_143832057_-	0.927320843	0.007731
NM_007817.2	circRNA_6140	Chr9:61935380_61937535_-	0.92731228	0.007733
XM_006509966.3	circRNA_0845	Chr11:29470315_29477458_+	0.927019197	0.007795
XM_006515637.2	circRNA_0501	Chr10:25283893_25289730_-	0.926985659	0.007802
XM_006515637.2	circRNA_6109	Chr9:57056714_57057861_+	0.926985659	0.007802
NM_010340.2	circRNA_4926	Chr5:143080224_143081077_-	0.926879164	0.007825
NM_001318003.2	circRNA_4662	Chr5:65301933_65303208_-	0.926854306	0.00783
XM_011240779.2	circRNA_5311	Chr6:145147636_145149357_+	0.926848144	0.007831
XM_006509531.3	circRNA_6446	ChrX:42217416_42250037_+	0.926809632	0.007839
NM_025758.4	circRNA_3003	Chr18:67545615_67587859_-	0.926793662	0.007843
XM_011248602.2	circRNA_6390	Chr9:121727316_121731565_+	0.926693942	0.007864
XM_011245529.2	circRNA_3954	Chr3:88346445_88349444_+	0.926652343	0.007873
XM_006503665.3	circRNA_0501	Chr10:25283893_25289730_-	0.92656083	0.007892
XM_006503665.3	circRNA_6109	Chr9:57056714_57057861_+	0.92656083	0.007892
XM_006518339.3	circRNA_0501	Chr10:25283893_25289730_-	0.926473191	0.007911
XM_006518339.3	circRNA_6109	Chr9:57056714_57057861_+	0.926473191	0.007911
NM_010090.2	circRNA_4055	Chr3:126796838_126798280_+	0.926421985	0.007921
NM_024406.2	circRNA_3155	Chr19:25061108_25065459_+	0.926409336	0.007924
NM_008872.3	circRNA_0157	Chr1:66801049_66802168_-	0.926405349	0.007925
XM_006503080.3	circRNA_6390	Chr9:121727316_121731565_+	0.926398103	0.007926
XM_017316715.1	circRNA_0961	Chr11:67192403_67254873_+	0.926387996	0.007929
NM_011082.3	circRNA_0611	Chr10:75274017_75279961_+	0.926358721	0.007935
NM_173427.2	circRNA_4662	Chr5:65301933_65303208_-	0.926297375	0.007948

XM_006524382.3	circRNA_3425|Chr2:37624173_37627526_-	0.926284877	0.007951
XM_006524382.3	circRNA_3607|Chr2:119057281_119064097_+	0.926284877	0.007951
NM_173869.3	circRNA_5311|Chr6:145147636_145149357_+	0.92628163	0.007951
NM_007739.2	circRNA_3954|Chr3:88346445_88349444_+	0.926262515	0.007955
XM_011239969.2	circRNA_3702|Chr2:143830084_143832057_-	0.926224625	0.007963
NM_007655.3	circRNA_3702|Chr2:143830084_143832057_-	0.926061669	0.007998
NM_001099314.1	circRNA_3003|Chr18:67545615_67587859_-	0.926011526	0.008009
NM_031180.2	circRNA_0157|Chr1:66801049_66802168_-	0.925961532	0.00802
NM_008872.3	circRNA_3702|Chr2:143830084_143832057_-	0.925937794	0.008025
NM_001190325.1	circRNA_3239|Chr19:45635735_45640521_-	0.925791752	0.008056
NM_001291145.1	circRNA_0611|Chr10:75274017_75279961_+	0.925769932	0.008061
XM_011244046.2	circRNA_0020|Chr1:10315205_10321709_+	0.925685031	0.008079
NM_016809.6	circRNA_4055|Chr3:126796838_126798280_+	0.925668679	0.008082
NM_031183.2	circRNA_5367|Chr7:28990988_28991325_+	0.925663439	0.008083
XM_006511102.2	circRNA_4662|Chr5:65301933_65303208_-	0.925510151	0.008116
NM_007555.4	circRNA_6140|Chr9:61935380_61937535_-	0.925498889	0.008119
NM_053080.3	circRNA_1632|Chr13:42055400_42055542_+	0.925441622	0.008131
NM_001305585.1	circRNA_0501|Chr10:25283893_25289730_-	0.925434676	0.008133
NM_001305585.1	circRNA_6109|Chr9:57056714_57057861_+	0.925434676	0.008133
NM_001291483.1	circRNA_3155|Chr19:25061108_25065459_+	0.925432552	0.008133
XM_011243586.2	circRNA_0020|Chr1:10315205_10321709_+	0.925391343	0.008142
NM_009020.3	circRNA_0611|Chr10:75274017_75279961_+	0.925307359	0.00816
XM_006517308.3	circRNA_3702|Chr2:143830084_143832057_-	0.92529822	0.008162
XM_006515787.3	circRNA_0501|Chr10:25283893_25289730_-	0.925284171	0.008165
XM_006515787.3	circRNA_6109|Chr9:57056714_57057861_+	0.925284171	0.008165
NM_145210.2	circRNA_1399|Chr12:72783683_72786789_+	0.925267373	0.008169
XM_006540596.2	circRNA_2888|Chr18:21010677_21020999_+	0.925057962	0.008214
XM_006532007.3	circRNA_5367|Chr7:28990988_28991325_+	0.925053647	0.008215
NM_181547.3	circRNA_4055|Chr3:126796838_126798280_+	0.924916123	0.008245
XM_006524382.3	circRNA_1399|Chr12:72783683_72786789_+	0.924916002	0.008245
NM_008032.3	circRNA_3954|Chr3:88346445_88349444_+	0.924883605	0.008252
XM_006503080.3	circRNA_0537|Chr10:43393871_43395692_+	0.924821081	0.008265
XM_006496905.1	circRNA_3239|Chr19:45635735_45640521_-	0.924799373	0.00827
NM_175260.2	circRNA_0845|Chr11:29470315_29477458_+	0.924769013	0.008277
XM_006506086.2	circRNA_3643|Chr2:122441559_122486090_+	0.924769002	0.008277
NM_010779.2	circRNA_3702|Chr2:143830084_143832057_-	0.924676358	0.008297
XM_006510077.3	circRNA_5311|Chr6:145147636_145149357_+	0.92463351	0.008306
NM_030206.4	circRNA_3702|Chr2:143830084_143832057_-	0.924618771	0.008309
XM_006532232.3	circRNA_3954|Chr3:88346445_88349444_+	0.924498413	0.008336
XM_006524382.3	circRNA_0845|Chr11:29470315_29477458_+	0.924479274	0.00834
XM_017313038.1	circRNA_6446|ChrX:42217416_42250037_+	0.92440686	0.008356
XM_006509966.3	circRNA_3832|Chr2:169883526_169886459_+	0.924402488	0.008356
XM_006501309.3	circRNA_1042|Chr11:80385854_80403408_+	0.924177908	0.008406
XM_006507942.2	circRNA_6140|Chr9:61935380_61937535_-	0.924071485	0.008429
NM_024406.2	circRNA_4055|Chr3:126796838_126798280_+	0.924021212	0.00844
NM_009514.4	circRNA_3239|Chr19:45635735_45640521_-	0.923998259	0.008445
NM_178715.3	circRNA_3155|Chr19:25061108_25065459_+	0.923989742	0.008447
NM_001198955.1	circRNA_3832|Chr2:169883526_169886459_+	0.923961899	0.008453
XM_006520186.2	circRNA_3239|Chr19:45635735_45640521_-	0.923920835	0.008462
NM_016933.3	circRNA_5311|Chr6:145147636_145149357_+	0.923798495	0.008489
XM_011248602.2	circRNA_3954|Chr3:88346445_88349444_+	0.923781585	0.008492
XM_006502235.3	circRNA_4635|Chr5:43758222_43773659_-	0.923761892	0.008497
NM_001317365.1	circRNA_5074|Chr6:40685277_40747150_+	0.923757286	0.008498
NM_020508.4	circRNA_6390|Chr9:121727316_121731565_+	0.9236957	0.008511
XM_006496277.3	circRNA_0020|Chr1:10315205_10321709_+	0.923658824	0.00852
NM_010279.3	circRNA_0157|Chr1:66801049_66802168_-	0.923635348	0.008525
XM_017316439.1	circRNA_3276|Chr19:60563162_60564294_-	0.923513931	0.008551
XM_006530325.3	circRNA_3954|Chr3:88346445_88349444_+	0.923493892	0.008556
XM_006506145.1	circRNA_3954|Chr3:88346445_88349444_+	0.923376845	0.008582

XM_017322026.1	circRNA_4055|Chr3:126796838_126798280_+	0.923357563	0.008586
NM_026385.4	circRNA_0501|Chr10:25283893_25289730_-	0.923341328	0.00859
NM_026385.4	circRNA_6109|Chr9:57056714_57057861_+	0.923341328	0.00859
NM_145210.2	circRNA_3425|Chr2:37624173_37627526_-	0.923305807	0.008597
NM_145210.2	circRNA_3607|Chr2:119057281_119064097_+	0.923305807	0.008597
NM_018807.5	circRNA_6390|Chr9:121727316_121731565_+	0.923239182	0.008612
NM_145210.2	circRNA_3003|Chr18:67545615_67587859_-	0.923237055	0.008613
NM_008264.1	circRNA_3239|Chr19:45635735_45640521_-	0.923217667	0.008617
NM_010714.3	circRNA_5311|Chr6:145147636_145149357_+	0.923120747	0.008638
NM_026385.4	circRNA_1632|Chr13:42055400_42055542_+	0.92311763	0.008639
NM_001313939.1	circRNA_3155|Chr19:25061108_25065459_+	0.923072718	0.008649
NM_010279.3	circRNA_0501|Chr10:25283893_25289730_-	0.923030229	0.008659
NM_010279.3	circRNA_6109|Chr9:57056714_57057861_+	0.923030229	0.008659
NM_011145.3	circRNA_0845|Chr11:29470315_29477458_+	0.923001065	0.008665
XM_006518339.3	circRNA_2888|Chr18:21010677_21020999_+	0.922922959	0.008682
NM_009246.3	circRNA_0845|Chr11:29470315_29477458_+	0.922877125	0.008693
NM_001002896.2	circRNA_5311|Chr6:145147636_145149357_+	0.922847108	0.008699
NM_008458.2	circRNA_3702|Chr2:143830084_143832057_-	0.922834986	0.008702
XM_011250176.1	circRNA_0537|Chr10:43393871_43395692_+	0.92277368	0.008716
NM_013912.3	circRNA_3155|Chr19:25061108_25065459_+	0.922727442	0.008726
XM_017321602.1	circRNA_6140|Chr9:61935380_61937535_-	0.922694309	0.008733
NM_007817.2	circRNA_0501|Chr10:25283893_25289730_-	0.922684869	0.008735
NM_007817.2	circRNA_6109|Chr9:57056714_57057861_+	0.922684869	0.008735
XM_006503665.3	circRNA_0157|Chr1:66801049_66802168_-	0.922646969	0.008744
XM_006521554.3	circRNA_3954|Chr3:88346445_88349444_+	0.92254234	0.008767
NM_008458.2	circRNA_6140|Chr9:61935380_61937535_-	0.922474365	0.008782
XM_006524777.3	circRNA_0501|Chr10:25283893_25289730_-	0.922401708	0.008799
XM_006524777.3	circRNA_6109|Chr9:57056714_57057861_+	0.922401708	0.008799
NM_023456.3	circRNA_6140|Chr9:61935380_61937535_-	0.922374365	0.008805
NM_178908.3	circRNA_6140|Chr9:61935380_61937535_-	0.922362209	0.008807
XM_017320219.1	circRNA_0537|Chr10:43393871_43395692_+	0.922342457	0.008812
NM_007555.4	circRNA_3155|Chr19:25061108_25065459_+	0.922271952	0.008828
NM_029844.3	circRNA_3702|Chr2:143830084_143832057_-	0.922257652	0.008831
NM_001327998.1	circRNA_1632|Chr13:42055400_42055542_+	0.922222289	0.008839
XM_017320093.1	circRNA_6446|ChrX:42217416_42250037_+	0.92219418	0.008845
NM_007763.3	circRNA_3155|Chr19:25061108_25065459_+	0.922181647	0.008848
NM_001033167.3	circRNA_6390|Chr9:121727316_121731565_+	0.922176257	0.008849
NM_031180.2	circRNA_0020|Chr1:10315205_10321709_+	0.922153355	0.008854
NM_178692.3	circRNA_0611|Chr10:75274017_75279961_+	0.922058964	0.008875
XM_006510077.3	circRNA_6140|Chr9:61935380_61937535_-	0.921900112	0.008911
NM_010279.3	circRNA_3702|Chr2:143830084_143832057_-	0.921887253	0.008914
XM_006524328.3	circRNA_6390|Chr9:121727316_121731565_+	0.921849967	0.008922
NM_001097617.1	circRNA_3239|Chr19:45635735_45640521_-	0.921830618	0.008927
NM_001290469.1	circRNA_3954|Chr3:88346445_88349444_+	0.921820045	0.008929
NM_001146217.1	circRNA_4055|Chr3:126796838_126798280_+	0.921788433	0.008936
XM_011248193.2	circRNA_6390|Chr9:121727316_121731565_+	0.921731098	0.008949
XM_006496277.3	circRNA_3702|Chr2:143830084_143832057_-	0.921676895	0.008962
NM_175563.5	circRNA_3954|Chr3:88346445_88349444_+	0.921511628	0.008999
XM_017322026.1	circRNA_0020|Chr1:10315205_10321709_+	0.921394421	0.009025
NM_008872.3	circRNA_3276|Chr19:60563162_60564294_-	0.921388743	0.009027
XM_017314944.1	circRNA_4728|Chr5:97027831_97045167_+	0.921349016	0.009036
XM_006530238.3	circRNA_6390|Chr9:121727316_121731565_+	0.921323649	0.009041
XM_006524382.3	circRNA_4487|Chr4:149156607_149161694_-	0.921293191	0.009048
NM_053080.3	circRNA_2951|Chr18:39120119_39150118_+	0.921283585	0.009051
NM_001162950.1	circRNA_0537|Chr10:43393871_43395692_+	0.921259551	0.009056
NM_009223.3	circRNA_5311|Chr6:145147636_145149357_+	0.921202649	0.009069
NM_181315.4	circRNA_3702|Chr2:143830084_143832057_-	0.921191865	0.009071
NM_001013784.1	circRNA_5074|Chr6:40685277_40747150_+	0.921145848	0.009082
XM_006511102.2	circRNA_0020|Chr1:10315205_10321709_+	0.921074152	0.009098

XM_006529829.3	circRNA_3239|Chr19:45635735_45640521_-	0.92099455	0.009116
NM_010858.4	circRNA_3155|Chr19:25061108_25065459_+	0.920941522	0.009128
NM_024283.3	circRNA_4728|Chr5:97027831_97045167_+	0.920870582	0.009144
NM_025288.2	circRNA_4055|Chr3:126796838_126798280_+	0.920779518	0.009165
XM_006525028.2	circRNA_3702|Chr2:143830084_143832057_-	0.92063166	0.009199
NM_009135.2	circRNA_3276|Chr19:60563162_60564294_-	0.920600501	0.009206
NM_031180.2	circRNA_3702|Chr2:143830084_143832057_-	0.920568934	0.009213
NM_009675.2	circRNA_3276|Chr19:60563162_60564294_-	0.920490684	0.009231
NM_177578.4	circRNA_0020|Chr1:10315205_10321709_+	0.920487759	0.009232
NM_001289875.1	circRNA_6390|Chr9:121727316_121731565_+	0.920362624	0.009261
XM_011243266.1	circRNA_6390|Chr9:121727316_121731565_+	0.920358241	0.009262
NM_001004357.2	circRNA_3239|Chr19:45635735_45640521_-	0.920321633	0.00927
NM_001099314.1	circRNA_3832|Chr2:169883526_169886459_+	0.920320272	0.00927
NM_001171187.1	circRNA_3276|Chr19:60563162_60564294_-	0.92030124	0.009275
XM_011240476.2	circRNA_0845|Chr11:29470315_29477458_+	0.920300463	0.009275
NM_031185.3	circRNA_6446|ChrX:42217416_42250037_+	0.920221207	0.009293
XM_006515787.3	circRNA_2951|Chr18:39120119_39150118_+	0.920123303	0.009316
XM_006513165.1	circRNA_5639|Chr7:132771578_132779385_-	0.920110386	0.009319
XM_006521554.3	circRNA_3425|Chr2:37624173_37627526_-	0.920093411	0.009322
XM_006521554.3	circRNA_3607|Chr2:119057281_119064097_+	0.920093411	0.009322
XM_017314870.1	circRNA_0961|Chr11:67192403_67254873_+	0.920055356	0.009331
NM_001142804.1	circRNA_0501|Chr10:25283893_25289730_-	0.920032982	0.009336
NM_001142804.1	circRNA_6109|Chr9:57056714_57057861_+	0.920032982	0.009336
NM_001291930.1	circRNA_3702|Chr2:143830084_143832057_-	0.920020589	0.009339
NM_011658.2	circRNA_3155|Chr19:25061108_25065459_+	0.920006086	0.009343
XM_017320735.1	circRNA_6446|ChrX:42217416_42250037_+	0.919983037	0.009348
XM_006521554.3	circRNA_1399|Chr12:72783683_72786789_+	0.919982157	0.009348
NM_001142804.1	circRNA_0157|Chr1:66801049_66802168_-	0.919912224	0.009364
XM_006520253.3	circRNA_3832|Chr2:169883526_169886459_+	0.91987879	0.009372
NM_007883.3	circRNA_1980|Chr14:50951404_50963869_+	0.919815616	0.009387
NM_027455.3	circRNA_6140|Chr9:61935380_61937535_-	0.919770476	0.009397
XM_006523061.3	circRNA_6140|Chr9:61935380_61937535_-	0.919769287	0.009397
NM_009349.3	circRNA_2888|Chr18:21010677_21020999_+	0.919769283	0.009397
XM_006541297.3	circRNA_4926|Chr5:143080224_143081077_-	0.919764828	0.009398
XM_006511007.3	circRNA_3832|Chr2:169883526_169886459_+	0.919708038	0.009411
NM_146125.2	circRNA_0157|Chr1:66801049_66802168_-	0.919704994	0.009412
NM_008067.4	circRNA_2888|Chr18:21010677_21020999_+	0.919519607	0.009455
NM_007655.3	circRNA_3276|Chr19:60563162_60564294_-	0.919492912	0.009461
NM_015744.4	circRNA_6446|ChrX:42217416_42250037_+	0.919470458	0.009466
NM_145141.2	circRNA_3155|Chr19:25061108_25065459_+	0.9194564	0.00947
XM_006505602.3	circRNA_6390|Chr9:121727316_121731565_+	0.919375252	0.009488
XM_006530353.3	circRNA_0537|Chr10:43393871_43395692_+	0.919321003	0.009501
XM_006522398.3	circRNA_0376|Chr1:172173943_172187460_+	0.919218455	0.009525
XM_006498086.3	circRNA_3702|Chr2:143830084_143832057_-	0.919211989	0.009526
NM_009518.2	circRNA_3643|Chr2:122441559_122486090_+	0.919149764	0.009541
XM_006496949.3	circRNA_2951|Chr18:39120119_39150118_+	0.919128789	0.009546
XM_006496949.3	circRNA_0611|Chr10:75274017_75279961_+	0.919124301	0.009547
XM_006525028.2	circRNA_4728|Chr5:97027831_97045167_+	0.919088946	0.009555
NM_007588.2	circRNA_5811|Chr8:71992832_71998295_-	0.919087148	0.009555
XM_006506624.2	circRNA_6140|Chr9:61935380_61937535_-	0.919082975	0.009556
XM_006496277.3	circRNA_4728|Chr5:97027831_97045167_+	0.919068811	0.00956
XM_006508885.1	circRNA_0157|Chr1:66801049_66802168_-	0.919037529	0.009567
NM_007817.2	circRNA_0157|Chr1:66801049_66802168_-	0.91902384	0.00957
NM_007883.3	circRNA_6412|ChrMT:13917_14141_-	0.919015007	0.009572
XM_006509966.3	circRNA_5074|Chr6:40685277_40747150_+	0.918995337	0.009577
XM_006527480.3	circRNA_3832|Chr2:169883526_169886459_+	0.918966409	0.009584
XM_017312731.1	circRNA_0376|Chr1:172173943_172187460_+	0.918953651	0.009587
NM_008067.4	circRNA_5367|Chr7:28990988_28991325_+	0.918940169	0.00959
XM_006521796.1	circRNA_0376|Chr1:172173943_172187460_+	0.9189041	0.009598

NM_144544.2	circRNA_6140|Chr9:61935380_61937535_-	0.918836695	0.009614
NM_177789.4	circRNA_4662|Chr5:65301933_65303208_-	0.918833723	0.009615
NM_015744.4	circRNA_4055|Chr3:126796838_126798280_+	0.91881228	0.00962
NM_178753.4	circRNA_0501|Chr10:25283893_25289730_-	0.918780996	0.009627
NM_178753.4	circRNA_6109|Chr9:57056714_57057861_+	0.918780996	0.009627
NM_029844.3	circRNA_0020|Chr1:10315205_10321709_+	0.918761851	0.009631
NM_153801.3	circRNA_0537|Chr10:43393871_43395692_+	0.918750169	0.009634
NM_009645.2	circRNA_0157|Chr1:66801049_66802168_-	0.918747716	0.009635
NM_009605.4	circRNA_0020|Chr1:10315205_10321709_+	0.918643883	0.009659
NM_175520.4	circRNA_6140|Chr9:61935380_61937535_-	0.918617109	0.009665
NM_028472.2	circRNA_0157|Chr1:66801049_66802168_-	0.918528146	0.009686
NM_019759.2	circRNA_5311|Chr6:145147636_145149357_+	0.918411993	0.009713
NM_001142804.1	circRNA_6140|Chr9:61935380_61937535_-	0.91834592	0.009729
NM_146063.1	circRNA_2951|Chr18:39120119_39150118_+	0.918303913	0.009739
NM_019626.3	circRNA_3239|Chr19:45635735_45640521_-	0.918258214	0.009749
NM_001302471.1	circRNA_3954|Chr3:88346445_88349444_+	0.918241198	0.009753
NM_001291145.1	circRNA_2888|Chr18:21010677_21020999_+	0.918166319	0.009771
NM_010055.3	circRNA_3003|Chr18:67545615_67587859_-	0.918085087	0.00979
NM_016809.6	circRNA_3155|Chr19:25061108_25065459_+	0.918065153	0.009795
NM_020025.4	circRNA_2951|Chr18:39120119_39150118_+	0.918049758	0.009799
NM_027416.3	circRNA_5311|Chr6:145147636_145149357_+	0.917940225	0.009824
NM_019759.2	circRNA_6446|ChrX:42217416_42250037_+	0.917898555	0.009834
NM_144945.3	circRNA_4926|Chr5:143080224_143081077_-	0.917842281	0.009848
NM_013906.3	circRNA_0962|Chr11:67194348_67220998_+	0.917764266	0.009866
XM_006520618.2	circRNA_6140|Chr9:61935380_61937535_-	0.917743033	0.009871
XM_006506086.2	circRNA_2888|Chr18:21010677_21020999_+	0.917716154	0.009877
XM_006529759.1	circRNA_3239|Chr19:45635735_45640521_-	0.917611786	0.009902
NM_146063.1	circRNA_0501|Chr10:25283893_25289730_-	0.917581649	0.009909
NM_146063.1	circRNA_6109|Chr9:57056714_57057861_+	0.917581649	0.009909
NM_001002896.2	circRNA_3239|Chr19:45635735_45640521_-	0.917555635	0.009915
NM_001001178.1	circRNA_5311|Chr6:145147636_145149357_+	0.917538339	0.00992
NM_009020.3	circRNA_2951|Chr18:39120119_39150118_+	0.917493289	0.00993
XM_006500919.3	circRNA_5311|Chr6:145147636_145149357_+	0.917474067	0.009935
XM_006530131.1	circRNA_6390|Chr9:121727316_121731565_+	0.917467761	0.009936
XM_006533539.3	circRNA_3276|Chr19:60563162_60564294_-	0.917388895	0.009955
NM_001082546.1	circRNA_4055|Chr3:126796838_126798280_+	0.917384297	0.009956
XM_006508867.2	circRNA_4662|Chr5:65301933_65303208_-	0.917378797	0.009957
NM_001171187.1	circRNA_0501|Chr10:25283893_25289730_-	0.917352578	0.009964
NM_001171187.1	circRNA_6109|Chr9:57056714_57057861_+	0.917352578	0.009964
XM_017320735.1	circRNA_2888|Chr18:21010677_21020999_+	0.917345025	0.009965
NM_008185.3	circRNA_4055|Chr3:126796838_126798280_+	0.917265424	0.009984
XM_006498086.3	circRNA_2951|Chr18:39120119_39150118_+	0.917231809	0.009992
XM_006503323.3	circRNA_3702|Chr2:143830084_143832057_-	0.917193767	0.010001
XM_006501237.3	circRNA_4055|Chr3:126796838_126798280_+	0.917186873	0.010003
XM_006520437.2	circRNA_0157|Chr1:66801049_66802168_-	0.917072544	0.01003
XM_017312731.1	circRNA_6140|Chr9:61935380_61937535_-	0.917065066	0.010032
NM_009246.3	circRNA_3832|Chr2:169883526_169886459_+	0.917014526	0.010044
XM_006501309.3	circRNA_1399|Chr12:72783683_72786789_+	0.91694108	0.010062
NM_001291483.1	circRNA_5311|Chr6:145147636_145149357_+	0.916851738	0.010083
NM_024406.2	circRNA_3702|Chr2:143830084_143832057_-	0.916805376	0.010094
NM_008012.1	circRNA_5311|Chr6:145147636_145149357_+	0.916773761	0.010102
NM_011957.2	circRNA_3954|Chr3:88346445_88349444_+	0.916711991	0.010116
XM_017322026.1	circRNA_0157|Chr1:66801049_66802168_-	0.916619888	0.010139
NM_001039047.1	circRNA_6390|Chr9:121727316_121731565_+	0.916587545	0.010146
NM_001301295.1	circRNA_3702|Chr2:143830084_143832057_-	0.916447402	0.01018
XM_006520023.2	circRNA_3239|Chr19:45635735_45640521_-	0.916326871	0.010209
XM_011248394.1	circRNA_4728|Chr5:97027831_97045167_+	0.91623045	0.010232
NM_024283.3	circRNA_6140|Chr9:61935380_61937535_-	0.916230398	0.010232
XM_006522398.3	circRNA_4728|Chr5:97027831_97045167_+	0.91619318	0.010241

NM_008623.5	circRNA_5367|Chr7:28990988_28991325_+	0.916181061	0.010244
XM_017312497.1	circRNA_4662|Chr5:65301933_65303208_-	0.916106435	0.010262
XM_006503323.3	circRNA_2951|Chr18:39120119_39150118_+	0.916082025	0.010268
NM_010181.2	circRNA_0537|Chr10:43393871_43395692_+	0.916077636	0.010269
NM_024406.2	circRNA_6140|Chr9:61935380_61937535_-	0.915875282	0.010318
NM_001033167.3	circRNA_3954|Chr3:88346445_88349444_+	0.915841455	0.010326
NM_009135.2	circRNA_0611|Chr10:75274017_75279961_+	0.915775384	0.010342
NM_177709.3	circRNA_5311|Chr6:145147636_145149357_+	0.915738164	0.010351
NM_144539.5	circRNA_6446|ChrX:42217416_42250037_+	0.915733088	0.010352
NM_007641.5	circRNA_6446|ChrX:42217416_42250037_+	0.915715464	0.010356
XM_006496277.3	circRNA_2888|Chr18:21010677_21020999_+	0.915595961	0.010385
XM_006525075.3	circRNA_3155|Chr19:25061108_25065459_+	0.915540597	0.010399
NM_009645.2	circRNA_0376|Chr1:172173943_172187460_+	0.915535045	0.0104
NM_019397.3	circRNA_4055|Chr3:126796838_126798280_+	0.915453198	0.01042
XM_006515637.2	circRNA_0611|Chr10:75274017_75279961_+	0.91537077	0.01044
XM_011248201.2	circRNA_6390|Chr9:121727316_121731565_+	0.915366536	0.010441
NM_007529.2	circRNA_2951|Chr18:39120119_39150118_+	0.915353313	0.010444
NM_028804.1	circRNA_6140|Chr9:61935380_61937535_-	0.915318724	0.010453
XM_011247208.1	circRNA_0961|Chr11:67192403_67254873_+	0.915308796	0.010455
NM_001190448.1	circRNA_3276|Chr19:60563162_60564294_-	0.915268653	0.010465
NM_029844.3	circRNA_4055|Chr3:126796838_126798280_+	0.915248262	0.01047
XM_017318541.1	circRNA_0376|Chr1:172173943_172187460_+	0.915230107	0.010474
NM_009930.2	circRNA_3239|Chr19:45635735_45640521_-	0.915214144	0.010478
NM_145741.2	circRNA_3276|Chr19:60563162_60564294_-	0.915183485	0.010486
NM_013468.3	circRNA_3832|Chr2:169883526_169886459_+	0.915182883	0.010486
NM_007817.2	circRNA_2951|Chr18:39120119_39150118_+	0.915168365	0.010489
XM_017314944.1	circRNA_0157|Chr1:66801049_66802168_-	0.915164218	0.01049
NM_177409.3	circRNA_6390|Chr9:121727316_121731565_+	0.91513692	0.010497
NM_001161541.1	circRNA_3702|Chr2:143830084_143832057_-	0.91509166	0.010508
NM_001290993.1	circRNA_6140|Chr9:61935380_61937535_-	0.915063444	0.010515
NM_029844.3	circRNA_6140|Chr9:61935380_61937535_-	0.915031637	0.010523
XM_006509966.3	circRNA_6390|Chr9:121727316_121731565_+	0.914986583	0.010534
NM_013459.3	circRNA_6140|Chr9:61935380_61937535_-	0.914985354	0.010534
NM_177709.3	circRNA_0913|Chr11:54005394_54014456_-	0.914976557	0.010536
XM_006513165.1	circRNA_4055|Chr3:126796838_126798280_+	0.91492029	0.01055
XM_011246128.2	circRNA_3276|Chr19:60563162_60564294_-	0.914868168	0.010563
XM_006525075.3	circRNA_4055|Chr3:126796838_126798280_+	0.914857304	0.010565
NM_023716.2	circRNA_3239|Chr19:45635735_45640521_-	0.914803989	0.010578
XM_006529456.3	circRNA_3276|Chr19:60563162_60564294_-	0.914764085	0.010588
NM_009127.4	circRNA_0157|Chr1:66801049_66802168_-	0.914735969	0.010595
XM_017312497.1	circRNA_0020|Chr1:10315205_10321709_+	0.914721854	0.010598
NM_153178.4	circRNA_3832|Chr2:169883526_169886459_+	0.914720812	0.010599
NM_019696.2	circRNA_5311|Chr6:145147636_145149357_+	0.914713226	0.010601
XM_006501309.3	circRNA_3425|Chr2:37624173_37627526_-	0.91470038	0.010604
XM_006501309.3	circRNA_3607|Chr2:119057281_119064097_+	0.91470038	0.010604
NM_010858.4	circRNA_6446|ChrX:42217416_42250037_+	0.914592205	0.01063
XM_006503665.3	circRNA_2951|Chr18:39120119_39150118_+	0.914543919	0.010642
NM_001030305.2	circRNA_6140|Chr9:61935380_61937535_-	0.914481979	0.010657
NM_016809.6	circRNA_5311|Chr6:145147636_145149357_+	0.914402385	0.010677
NM_001290993.1	circRNA_3155|Chr19:25061108_25065459_+	0.914366136	0.010686
NM_001290469.1	circRNA_6390|Chr9:121727316_121731565_+	0.914321762	0.010697
NM_178676.4	circRNA_3003|Chr18:67545615_67587859_-	0.914273526	0.010709
NM_007792.4	circRNA_0020|Chr1:10315205_10321709_+	0.914208248	0.010725
NM_028973.2	circRNA_3155|Chr19:25061108_25065459_+	0.914191053	0.010729
XM_006525028.2	circRNA_0020|Chr1:10315205_10321709_+	0.914187786	0.01073
XM_006537815.3	circRNA_0845|Chr11:29470315_29477458_+	0.914167679	0.010735
NM_008012.1	circRNA_6446|ChrX:42217416_42250037_+	0.914086315	0.010755
XM_011241948.2	circRNA_4728|Chr5:97027831_97045167_+	0.914069014	0.010759
XM_006528026.2	circRNA_6390|Chr9:121727316_121731565_+	0.9140512	0.010763

NM_023670.3	circRNA_6140|Chr9:61935380_61937535_-	0.913957617	0.010786
NM_199304.1	circRNA_0845|Chr11:29470315_29477458_+	0.913917819	0.010796
XM_006496905.1	circRNA_6140|Chr9:61935380_61937535_-	0.913881031	0.010805
NM_001282961.1	circRNA_2888|Chr18:21010677_21020999_+	0.913858592	0.010811
XM_006506624.2	circRNA_0157|Chr1:66801049_66802168_-	0.913848392	0.010813
XM_011248576.2	circRNA_0537|Chr10:43393871_43395692_+	0.91382813	0.010818
NM_001013784.1	circRNA_4926|Chr5:143080224_143081077_-	0.913632271	0.010867
NM_145741.2	circRNA_0157|Chr1:66801049_66802168_-	0.913592189	0.010877
XM_006503665.3	circRNA_6140|Chr9:61935380_61937535_-	0.913562064	0.010884
NM_009020.3	circRNA_0157|Chr1:66801049_66802168_-	0.913452477	0.010912
XM_006533452.3	circRNA_6140|Chr9:61935380_61937535_-	0.913452297	0.010912
XM_006532962.2	circRNA_0537|Chr10:43393871_43395692_+	0.913440178	0.010915
NM_013459.3	circRNA_3702|Chr2:143830084_143832057_-	0.913431852	0.010917
NM_172454.2	circRNA_4635|Chr5:43758222_43773659_-	0.913416883	0.01092
NM_001301354.1	circRNA_2888|Chr18:21010677_21020999_+	0.91340867	0.010922
XM_006510077.3	circRNA_0376|Chr1:172173943_172187460_+	0.913309982	0.010947
XM_006498901.3	circRNA_4728|Chr5:97027831_97045167_+	0.913271193	0.010957
XM_006539074.3	circRNA_6390|Chr9:121727316_121731565_+	0.913170065	0.010982
XM_006533479.2	circRNA_5311|Chr6:145147636_145149357_+	0.913067971	0.011007
NM_015814.2	circRNA_2951|Chr18:39120119_39150118_+	0.913010789	0.011022
XM_006499466.3	circRNA_0961|Chr11:67192403_67254873_+	0.913004163	0.011023
XM_006499466.3	circRNA_4926|Chr5:143080224_143081077_-	0.912940316	0.011039
XM_006537815.3	circRNA_3954|Chr3:88346445_88349444_+	0.912914467	0.011046
NM_011169.5	circRNA_4055|Chr3:126796838_126798280_+	0.912905576	0.011048
NM_009127.4	circRNA_0501|Chr10:25283893_25289730_-	0.912904961	0.011048
NM_009127.4	circRNA_6109|Chr9:57056714_57057861_+	0.912904961	0.011048
XM_006532007.3	circRNA_2951|Chr18:39120119_39150118_+	0.912867726	0.011057
XM_006520437.2	circRNA_2888|Chr18:21010677_21020999_+	0.912827308	0.011067
NM_010279.3	circRNA_2951|Chr18:39120119_39150118_+	0.912750628	0.011087
XM_006529829.3	circRNA_3643|Chr2:122441559_122486090_+	0.912736612	0.01109
XM_006508885.1	circRNA_4055|Chr3:126796838_126798280_+	0.912589782	0.011127
NM_007792.4	circRNA_5311|Chr6:145147636_145149357_+	0.912515815	0.011145
NM_001320077.1	circRNA_0611|Chr10:75274017_75279961_+	0.912471828	0.011156
XM_006506152.3	circRNA_3003|Chr18:67545615_67587859_-	0.912466383	0.011158
NM_020025.4	circRNA_0501|Chr10:25283893_25289730_-	0.912456132	0.01116
NM_020025.4	circRNA_6109|Chr9:57056714_57057861_+	0.912456132	0.01116
XM_006520023.2	circRNA_0376|Chr1:172173943_172187460_+	0.912447084	0.011163
NM_001110009.2	circRNA_4055|Chr3:126796838_126798280_+	0.912426301	0.011168
NM_001291483.1	circRNA_0376|Chr1:172173943_172187460_+	0.912417974	0.01117
NM_173869.3	circRNA_3155|Chr19:25061108_25065459_+	0.91239221	0.011176
NM_001110009.2	circRNA_0501|Chr10:25283893_25289730_-	0.912380921	0.011179
NM_001110009.2	circRNA_6109|Chr9:57056714_57057861_+	0.912380921	0.011179
NM_029823.2	circRNA_0020|Chr1:10315205_10321709_+	0.912361521	0.011184
XM_006541297.3	circRNA_6390|Chr9:121727316_121731565_+	0.912331942	0.011192
NM_001190451.2	circRNA_3155|Chr19:25061108_25065459_+	0.912320945	0.011194
NM_001097617.1	circRNA_0157|Chr1:66801049_66802168_-	0.91229274	0.011201
NM_001190258.1	circRNA_0376|Chr1:172173943_172187460_+	0.912284306	0.011204
XM_011244046.2	circRNA_0157|Chr1:66801049_66802168_-	0.912197253	0.011226
XM_017318541.1	circRNA_0611|Chr10:75274017_75279961_+	0.912062228	0.01126
XM_006537836.1	circRNA_6140|Chr9:61935380_61937535_-	0.912051692	0.011262
NM_016659.3	circRNA_3702|Chr2:143830084_143832057_-	0.911989764	0.011278
NM_007555.4	circRNA_0020|Chr1:10315205_10321709_+	0.911986597	0.011279
NM_001313939.1	circRNA_3239|Chr19:45635735_45640521_-	0.911793486	0.011327
NM_011957.2	circRNA_0537|Chr10:43393871_43395692_+	0.911763539	0.011335
XM_006510297.3	circRNA_0157|Chr1:66801049_66802168_-	0.911716723	0.011347
XM_006527008.2	circRNA_3832|Chr2:169883526_169886459_+	0.911685351	0.011355
NM_009332.3	circRNA_0537|Chr10:43393871_43395692_+	0.911666217	0.01136
NM_026385.4	circRNA_5367|Chr7:28990988_28991325_+	0.911665166	0.01136
NM_001033167.3	circRNA_0845|Chr11:29470315_29477458_+	0.911615206	0.011373

XM_006510297.3	circRNA_2951|Chr18:39120119_39150118_+	0.911610079	0.011374
NM_023456.3	circRNA_0501|Chr10:25283893_25289730_-	0.911518654	0.011397
NM_023456.3	circRNA_6109|Chr9:57056714_57057861_+	0.911518654	0.011397
NM_001146217.1	circRNA_5311|Chr6:145147636_145149357_+	0.911505662	0.0114
NM_001146217.1	circRNA_0020|Chr1:10315205_10321709_+	0.911371021	0.011435
NM_001302257.1	circRNA_0501|Chr10:25283893_25289730_-	0.911355842	0.011438
NM_001302257.1	circRNA_6109|Chr9:57056714_57057861_+	0.911355842	0.011438
XM_006515096.2	circRNA_2313|Chr15:93452117_93465245_+	0.911305144	0.011451
NM_178692.3	circRNA_3155|Chr19:25061108_25065459_+	0.911154692	0.01149
NM_001313939.1	circRNA_3702|Chr2:143830084_143832057_-	0.911138853	0.011494
XM_017318541.1	circRNA_3155|Chr19:25061108_25065459_+	0.911076783	0.011509
XM_006533653.3	circRNA_3003|Chr18:67545615_67587859_-	0.911031002	0.011521
NM_001159407.1	circRNA_6140|Chr9:61935380_61937535_-	0.911016352	0.011525
XM_006511007.3	circRNA_0537|Chr10:43393871_43395692_+	0.910956279	0.01154
NM_008760.4	circRNA_2951|Chr18:39120119_39150118_+	0.910865334	0.011563
XM_006524777.3	circRNA_3155|Chr19:25061108_25065459_+	0.910676432	0.011612
XM_006510297.3	circRNA_0501|Chr10:25283893_25289730_-	0.910667947	0.011614
XM_006510297.3	circRNA_6109|Chr9:57056714_57057861_+	0.910667947	0.011614
NM_001161541.1	circRNA_0020|Chr1:10315205_10321709_+	0.91065944	0.011616
XM_017317978.1	circRNA_3155|Chr19:25061108_25065459_+	0.910645745	0.01162
NM_177743.5	circRNA_2951|Chr18:39120119_39150118_+	0.910628402	0.011624
XM_006506340.3	circRNA_3954|Chr3:88346445_88349444_+	0.910592154	0.011633
XM_006518339.3	circRNA_0611|Chr10:75274017_75279961_+	0.910532799	0.011649
XM_006532007.3	circRNA_3276|Chr19:60563162_60564294_-	0.910515637	0.011653
NM_010450.3	circRNA_0020|Chr1:10315205_10321709_+	0.910429122	0.011675
XM_006496949.3	circRNA_4987|Chr6:4529068_4531010_+	0.910366689	0.011691
XM_006522398.3	circRNA_4662|Chr5:65301933_65303208_-	0.910348602	0.011696
NM_023456.3	circRNA_2951|Chr18:39120119_39150118_+	0.910321454	0.011703
NM_146241.2	circRNA_3276|Chr19:60563162_60564294_-	0.910187346	0.011737
XM_006501309.3	circRNA_0845|Chr11:29470315_29477458_+	0.910166755	0.011743
XM_011239558.2	circRNA_6390|Chr9:121727316_121731565_+	0.910159699	0.011744
XM_006527452.3	circRNA_0611|Chr10:75274017_75279961_+	0.910078188	0.011765
NM_031185.3	circRNA_3276|Chr19:60563162_60564294_-	0.909998021	0.011786
NM_001290993.1	circRNA_2951|Chr18:39120119_39150118_+	0.909930531	0.011803
NM_152915.1	circRNA_0157|Chr1:66801049_66802168_-	0.909796618	0.011838
NM_009381.3	circRNA_4728|Chr5:97027831_97045167_+	0.909678361	0.011869
NM_009675.2	circRNA_0157|Chr1:66801049_66802168_-	0.90957351	0.011896
XM_011246479.2	circRNA_0845|Chr11:29470315_29477458_+	0.909563359	0.011898
NM_009127.4	circRNA_2951|Chr18:39120119_39150118_+	0.909542702	0.011904
XM_006523061.3	circRNA_3702|Chr2:143830084_143832057_-	0.909542147	0.011904
NM_021475.2	circRNA_6140|Chr9:61935380_61937535_-	0.90950241	0.011914
NM_001097617.1	circRNA_5311|Chr6:145147636_145149357_+	0.909495853	0.011916
NM_001309809.2	circRNA_2951|Chr18:39120119_39150118_+	0.909482991	0.011919
NM_027416.3	circRNA_0376|Chr1:172173943_172187460_+	0.90943402	0.011932
NM_028804.1	circRNA_3276|Chr19:60563162_60564294_-	0.909352027	0.011953
XM_006504940.3	circRNA_3276|Chr19:60563162_60564294_-	0.909253012	0.011979
NM_001290993.1	circRNA_3239|Chr19:45635735_45640521_-	0.909249903	0.01198
NM_031180.2	circRNA_3239|Chr19:45635735_45640521_-	0.909237799	0.011983
NM_007588.2	circRNA_0845|Chr11:29470315_29477458_+	0.90923048	0.011985
XM_006532412.1	circRNA_0962|Chr11:67194348_67220998_+	0.909123362	0.012013
NM_001004357.2	circRNA_2888|Chr18:21010677_21020999_+	0.909111773	0.012016
XM_006529759.1	circRNA_4055|Chr3:126796838_126798280_+	0.909071046	0.012026
XM_006518339.3	circRNA_3155|Chr19:25061108_25065459_+	0.909024671	0.012038
NM_009345.2	circRNA_0611|Chr10:75274017_75279961_+	0.908988598	0.012048
XM_006529829.3	circRNA_4987|Chr6:4529068_4531010_+	0.908921418	0.012065
XM_006515637.2	circRNA_1632|Chr13:42055400_42055542_+	0.908880963	0.012076
NM_009246.3	circRNA_3003|Chr18:67545615_67587859_-	0.908821425	0.012091
NM_173869.3	circRNA_4055|Chr3:126796838_126798280_+	0.908810445	0.012094
NM_008277.2	circRNA_2888|Chr18:21010677_21020999_+	0.908809832	0.012094

XM_006532232.3	circRNA_0537|Chr10:43393871_43395692_+	0.908750688	0.01211
NM_001142804.1	circRNA_2951|Chr18:39120119_39150118_+	0.908708809	0.012121
NM_001110322.1	circRNA_0157|Chr1:66801049_66802168_-	0.908680803	0.012128
XM_006506624.2	circRNA_3702|Chr2:143830084_143832057_-	0.90867217	0.01213
NM_001282961.1	circRNA_0376|Chr1:172173943_172187460_+	0.908622818	0.012143
XM_006534171.3	circRNA_4055|Chr3:126796838_126798280_+	0.908537443	0.012166
XM_017312731.1	circRNA_0157|Chr1:66801049_66802168_-	0.90849039	0.012178
NM_001244031.1	circRNA_3832|Chr2:169883526_169886459_+	0.908430199	0.012194
NM_001190449.1	circRNA_5311|Chr6:145147636_145149357_+	0.908394525	0.012203
XM_006515637.2	circRNA_2951|Chr18:39120119_39150118_+	0.908380944	0.012207
NM_001290993.1	circRNA_0501|Chr10:25283893_25289730_-	0.90832457	0.012221
NM_001290993.1	circRNA_6109|Chr9:57056714_57057861_+	0.90832457	0.012221
XM_017322026.1	circRNA_5311|Chr6:145147636_145149357_+	0.908321911	0.012222
XM_006532007.3	circRNA_0501|Chr10:25283893_25289730_-	0.908319702	0.012223
XM_006532007.3	circRNA_6109|Chr9:57056714_57057861_+	0.908319702	0.012223
NM_145141.2	circRNA_3702|Chr2:143830084_143832057_-	0.908295677	0.012229
XM_006519134.3	circRNA_6390|Chr9:121727316_121731565_+	0.90828676	0.012231
NM_001080819.1	circRNA_6390|Chr9:121727316_121731565_+	0.908263273	0.012237
NM_001198955.1	circRNA_3954|Chr3:88346445_88349444_+	0.908198365	0.012254
XM_006498086.3	circRNA_0501|Chr10:25283893_25289730_-	0.908185202	0.012258
XM_006498086.3	circRNA_6109|Chr9:57056714_57057861_+	0.908185202	0.012258
NM_001099314.1	circRNA_0537|Chr10:43393871_43395692_+	0.908033681	0.012298
XM_006507942.2	circRNA_4055|Chr3:126796838_126798280_+	0.908014359	0.012303
NM_016982.2	circRNA_0157|Chr1:66801049_66802168_-	0.9080018	0.012306
XM_017322026.1	circRNA_3239|Chr19:45635735_45640521_-	0.907904027	0.012332
XM_006532232.3	circRNA_3832|Chr2:169883526_169886459_+	0.907879657	0.012338
XM_011250217.1	circRNA_3832|Chr2:169883526_169886459_+	0.90787578	0.012339
XM_006513165.1	circRNA_0501|Chr10:25283893_25289730_-	0.907856444	0.012344
XM_006513165.1	circRNA_6109|Chr9:57056714_57057861_+	0.907856444	0.012344
NM_178753.4	circRNA_2888|Chr18:21010677_21020999_+	0.907722818	0.01238
NM_016659.3	circRNA_6140|Chr9:61935380_61937535_-	0.907707036	0.012384
XM_006517308.3	circRNA_2951|Chr18:39120119_39150118_+	0.907684372	0.01239
NM_011581.3	circRNA_0020|Chr1:10315205_10321709_+	0.907675395	0.012392
NM_010140.3	circRNA_2888|Chr18:21010677_21020999_+	0.907625421	0.012405
NM_001134300.2	circRNA_6390|Chr9:121727316_121731565_+	0.907621353	0.012407
NM_016697.3	circRNA_0611|Chr10:75274017_75279961_+	0.907573081	0.012419
XM_006507942.2	circRNA_6446|ChrX:42217416_42250037_+	0.907560801	0.012423
NM_023670.3	circRNA_5311|Chr6:145147636_145149357_+	0.907389561	0.012468
XM_006508867.2	circRNA_4728|Chr5:97027831_97045167_+	0.907384166	0.012469
NM_172907.3	circRNA_6140|Chr9:61935380_61937535_-	0.907356909	0.012477
XM_006528842.1	circRNA_6390|Chr9:121727316_121731565_+	0.907341268	0.012481
NM_001301295.1	circRNA_6140|Chr9:61935380_61937535_-	0.90725417	0.012504
NM_008423.2	circRNA_3832|Chr2:169883526_169886459_+	0.907192241	0.01252
NM_010340.2	circRNA_3832|Chr2:169883526_169886459_+	0.907127016	0.012538
NM_019866.1	circRNA_0157|Chr1:66801049_66802168_-	0.907097806	0.012545
NM_001310705.1	circRNA_5311|Chr6:145147636_145149357_+	0.906950894	0.012584
XM_006533452.3	circRNA_0501|Chr10:25283893_25289730_-	0.906858104	0.012609
XM_006533452.3	circRNA_6109|Chr9:57056714_57057861_+	0.906858104	0.012609
NM_007529.2	circRNA_0020|Chr1:10315205_10321709_+	0.906745015	0.012639
NM_020025.4	circRNA_0157|Chr1:66801049_66802168_-	0.9067423	0.01264
NM_009349.3	circRNA_3643|Chr2:122441559_122486090_+	0.906685178	0.012655
NM_011044.2	circRNA_6140|Chr9:61935380_61937535_-	0.906607629	0.012676
NM_001310070.1	circRNA_6390|Chr9:121727316_121731565_+	0.906599457	0.012678
NM_145741.2	circRNA_0611|Chr10:75274017_75279961_+	0.906540859	0.012694
NM_199304.1	circRNA_2313|Chr15:93452117_93465245_+	0.906539369	0.012694
NM_153178.4	circRNA_6390|Chr9:121727316_121731565_+	0.906495884	0.012706
XM_006510077.3	circRNA_3702|Chr2:143830084_143832057_-	0.906441589	0.01272
XM_006503665.3	circRNA_2888|Chr18:21010677_21020999_+	0.906438447	0.012721
XM_017316439.1	circRNA_0501|Chr10:25283893_25289730_-	0.906348734	0.012745

XM_017316439.1	circRNA_6109|Chr9:57056714_57057861_+	0.906348734	0.012745
NM_023670.3	circRNA_0157|Chr1:66801049_66802168_-	0.906340963	0.012747
XM_017322026.1	circRNA_3702|Chr2:143830084_143832057_-	0.906318629	0.012753
XM_006527452.3	circRNA_3276|Chr19:60563162_60564294_-	0.906290057	0.012761
XM_006496277.3	circRNA_6140|Chr9:61935380_61937535_-	0.906267327	0.012767
NM_010279.3	circRNA_2888|Chr18:21010677_21020999_+	0.906145926	0.0128
NM_015814.2	circRNA_3155|Chr19:25061108_25065459_+	0.906062499	0.012822
NM_001242411.1	circRNA_6390|Chr9:121727316_121731565_+	0.906037214	0.012829
NM_001243916.1	circRNA_6140|Chr9:61935380_61937535_-	0.90597923	0.012844
NM_011430.3	circRNA_4728|Chr5:97027831_97045167_+	0.905905772	0.012864
NM_178753.4	circRNA_6140|Chr9:61935380_61937535_-	0.905881711	0.012871
XM_006515787.3	circRNA_3276|Chr19:60563162_60564294_-	0.905881237	0.012871
XM_011241948.2	circRNA_0157|Chr1:66801049_66802168_-	0.90586306	0.012876
NM_173427.2	circRNA_0157|Chr1:66801049_66802168_-	0.905843245	0.012881
XM_017313038.1	circRNA_3276|Chr19:60563162_60564294_-	0.905791349	0.012895
NM_009518.2	circRNA_3239|Chr19:45635735_45640521_-	0.905784258	0.012897
NM_001171187.1	circRNA_5367|Chr7:28990988_28991325_+	0.905769155	0.012901
XM_006500919.3	circRNA_3702|Chr2:143830084_143832057_-	0.905766123	0.012902
XM_017322026.1	circRNA_6140|Chr9:61935380_61937535_-	0.905644137	0.012935
NM_001097617.1	circRNA_2951|Chr18:39120119_39150118_+	0.905620847	0.012941
NM_007655.3	circRNA_6446|ChrX:42217416_42250037_+	0.905573153	0.012954
NM_007549.2	circRNA_6446|ChrX:42217416_42250037_+	0.905540095	0.012963
NM_007792.4	circRNA_0376|Chr1:172173943_172187460_+	0.905538065	0.012963
NM_001190449.1	circRNA_0611|Chr10:75274017_75279961_+	0.905529773	0.012965
NM_007555.4	circRNA_2888|Chr18:21010677_21020999_+	0.90547515	0.01298
NM_009514.4	circRNA_6446|ChrX:42217416_42250037_+	0.905466287	0.012983
NM_015744.4	circRNA_0020|Chr1:10315205_10321709_+	0.905306352	0.013026
XM_017316439.1	circRNA_2951|Chr18:39120119_39150118_+	0.905232283	0.013046
XM_017316439.1	circRNA_0611|Chr10:75274017_75279961_+	0.905222157	0.013049
NM_007482.3	circRNA_0611|Chr10:75274017_75279961_+	0.905219253	0.013049
NM_001302471.1	circRNA_0537|Chr10:43393871_43395692_+	0.905161461	0.013065
XM_017320219.1	circRNA_6390|Chr9:121727316_121731565_+	0.905151871	0.013068
XM_006520437.2	circRNA_3702|Chr2:143830084_143832057_-	0.905100818	0.013081
XM_006508990.2	circRNA_2313|Chr15:93452117_93465245_+	0.904988265	0.013112
NM_032400.2	circRNA_0501|Chr10:25283893_25289730_-	0.904958963	0.01312
NM_032400.2	circRNA_6109|Chr9:57056714_57057861_+	0.904958963	0.01312
NM_001204959.1	circRNA_4728|Chr5:97027831_97045167_+	0.904946012	0.013123
NM_009518.2	circRNA_2888|Chr18:21010677_21020999_+	0.904939869	0.013125
XM_011246309.2	circRNA_0501|Chr10:25283893_25289730_-	0.904934007	0.013127
XM_011246309.2	circRNA_6109|Chr9:57056714_57057861_+	0.904934007	0.013127
NM_001310636.1	circRNA_3643|Chr2:122441559_122486090_+	0.904905894	0.013134
XM_017315376.1	circRNA_3276|Chr19:60563162_60564294_-	0.904799872	0.013163
NM_016659.3	circRNA_0020|Chr1:10315205_10321709_+	0.904742599	0.013179
NM_010742.1	circRNA_5311|Chr6:145147636_145149357_+	0.904728253	0.013183
NM_019732.2	circRNA_0537|Chr10:43393871_43395692_+	0.904722791	0.013184
XM_017314944.1	circRNA_0020|Chr1:10315205_10321709_+	0.904593728	0.013219
XM_006532007.3	circRNA_4987|Chr6:4529068_4531010_+	0.904581087	0.013223
NM_001082547.1	circRNA_5311|Chr6:145147636_145149357_+	0.90454661	0.013232
NM_010858.4	circRNA_5311|Chr6:145147636_145149357_+	0.904507913	0.013243
XM_006525028.2	circRNA_0611|Chr10:75274017_75279961_+	0.904506644	0.013243
NM_009020.3	circRNA_1632|Chr13:42055400_42055542_+	0.904420852	0.013266
NM_029844.3	circRNA_3239|Chr19:45635735_45640521_-	0.904419251	0.013267
NM_133167.3	circRNA_6390|Chr9:121727316_121731565_+	0.904416222	0.013268
NM_001162950.1	circRNA_3832|Chr2:169883526_169886459_+	0.904366181	0.013281
XM_006522398.3	circRNA_3155|Chr19:25061108_25065459_+	0.90435089	0.013286
NM_031185.3	circRNA_3702|Chr2:143830084_143832057_-	0.904347126	0.013287
NM_008067.4	circRNA_0501|Chr10:25283893_25289730_-	0.904323949	0.013293
NM_008067.4	circRNA_6109|Chr9:57056714_57057861_+	0.904323949	0.013293
NM_023456.3	circRNA_0157|Chr1:66801049_66802168_-	0.904323674	0.013293

NM_053080.3	circRNA_3276|Chr19:60563162_60564294_-	0.904295135	0.013301
XM_006521796.1	circRNA_4662|Chr5:65301933_65303208_-	0.904265768	0.013309
NM_010140.3	circRNA_6140|Chr9:61935380_61937535_-	0.904184503	0.013331
NM_001013784.1	circRNA_0845|Chr11:29470315_29477458_+	0.904137604	0.013344
NM_144945.3	circRNA_5990|Chr9:3475483_3477962_+	0.904136327	0.013344
NM_153104.3	circRNA_0962|Chr11:67194348_67220998_+	0.90406268	0.013364
NM_009605.4	circRNA_3239|Chr19:45635735_45640521_-	0.903933287	0.0134
NM_177578.4	circRNA_6140|Chr9:61935380_61937535_-	0.903899079	0.013409
NM_007529.2	circRNA_0376|Chr1:172173943_172187460_+	0.903856711	0.013421
NM_010137.3	circRNA_6390|Chr9:121727316_121731565_+	0.903676544	0.01347
NM_001243916.1	circRNA_5311|Chr6:145147636_145149357_+	0.903671758	0.013472
XM_006517308.3	circRNA_0501|Chr10:25283893_25289730_-	0.903632883	0.013482
XM_006517308.3	circRNA_6109|Chr9:57056714_57057861_+	0.903632883	0.013482
NM_027416.3	circRNA_4728|Chr5:97027831_97045167_+	0.90358999	0.013494
XM_017321602.1	circRNA_2951|Chr18:39120119_39150118_+	0.903560565	0.013502
NM_001077403.1	circRNA_0845|Chr11:29470315_29477458_+	0.903522732	0.013513
XM_006499466.3	circRNA_5990|Chr9:3475483_3477962_+	0.903435339	0.013537
XM_006496905.1	circRNA_0020|Chr1:10315205_10321709_+	0.903288889	0.013577
NM_178184.2	circRNA_5639|Chr7:132771578_132779385_-	0.903286269	0.013578
XM_017316439.1	circRNA_6140|Chr9:61935380_61937535_-	0.903281909	0.013579
XM_011238891.1	circRNA_0501|Chr10:25283893_25289730_-	0.903268353	0.013583
XM_011238891.1	circRNA_6109|Chr9:57056714_57057861_+	0.903268353	0.013583
NM_173869.3	circRNA_3239|Chr19:45635735_45640521_-	0.903255172	0.013587
NM_007763.3	circRNA_3239|Chr19:45635735_45640521_-	0.902957303	0.013669
XM_006505388.2	circRNA_0611|Chr10:75274017_75279961_+	0.902943745	0.013673
NM_001164593.1	circRNA_3239|Chr19:45635735_45640521_-	0.902938572	0.013674
XM_017315376.1	circRNA_6140|Chr9:61935380_61937535_-	0.902935788	0.013675
XM_006538830.1	circRNA_5074|Chr6:40685277_40747150_+	0.902932328	0.013676
XM_006496949.3	circRNA_3239|Chr19:45635735_45640521_-	0.902897853	0.013685
XM_017321602.1	circRNA_0611|Chr10:75274017_75279961_+	0.902857547	0.013697
NM_024406.2	circRNA_0501|Chr10:25283893_25289730_-	0.902812675	0.013709
NM_024406.2	circRNA_6109|Chr9:57056714_57057861_+	0.902812675	0.013709
NM_001030305.2	circRNA_3239|Chr19:45635735_45640521_-	0.902717194	0.013736
XM_006501309.3	circRNA_3003|Chr18:67545615_67587859_-	0.902638941	0.013757
NM_009514.4	circRNA_4055|Chr3:126796838_126798280_+	0.902627585	0.01376
NM_001291483.1	circRNA_0157|Chr1:66801049_66802168_-	0.902576938	0.013775
NM_032400.2	circRNA_4728|Chr5:97027831_97045167_+	0.902569923	0.013776
NM_027416.3	circRNA_5639|Chr7:132771578_132779385_-	0.902518573	0.013791
NM_026535.2	circRNA_3702|Chr2:143830084_143832057_-	0.902501259	0.013796
NM_001290273.1	circRNA_3155|Chr19:25061108_25065459_+	0.902407435	0.013822
NM_178908.3	circRNA_4055|Chr3:126796838_126798280_+	0.902362453	0.013834
NM_008966.3	circRNA_3702|Chr2:143830084_143832057_-	0.902276394	0.013858
XM_006520437.2	circRNA_0611|Chr10:75274017_75279961_+	0.902244703	0.013867
NM_007588.2	circRNA_2313|Chr15:93452117_93465245_+	0.902185034	0.013884
XM_006533452.3	circRNA_2951|Chr18:39120119_39150118_+	0.902168086	0.013888
NM_015814.2	circRNA_0020|Chr1:10315205_10321709_+	0.902152848	0.013893
NM_019732.2	circRNA_3832|Chr2:169883526_169886459_+	0.90214896	0.013894
XM_006522398.3	circRNA_3702|Chr2:143830084_143832057_-	0.902130633	0.013899
XM_006538224.1	circRNA_6390|Chr9:121727316_121731565_+	0.90208843	0.013911
XM_006522398.3	circRNA_0157|Chr1:66801049_66802168_-	0.90206047	0.013919
XM_006516224.3	circRNA_3832|Chr2:169883526_169886459_+	0.902053427	0.01392
NM_001004357.2	circRNA_3276|Chr19:60563162_60564294_-	0.902037422	0.013925
XM_006521554.3	circRNA_0845|Chr11:29470315_29477458_+	0.902036254	0.013925
XM_017316897.1	circRNA_6412|ChrMT:13917_14141_-	0.902034543	0.013926
XM_006525075.3	circRNA_3239|Chr19:45635735_45640521_-	0.902002991	0.013935
NM_019866.1	circRNA_0501|Chr10:25283893_25289730_-	0.901960012	0.013947
NM_019866.1	circRNA_6109|Chr9:57056714_57057861_+	0.901960012	0.013947
XM_006506624.2	circRNA_0020|Chr1:10315205_10321709_+	0.901951295	0.013949
NM_145635.2	circRNA_4055|Chr3:126796838_126798280_+	0.901949538	0.01395

NM_008940.3	circRNA_4728|Chr5:97027831_97045167_+	0.901933559	0.013954
XM_006532962.2	circRNA_3832|Chr2:169883526_169886459_+	0.90190725	0.013961
XM_006532024.3	circRNA_6446|ChrX:42217416_42250037_+	0.901591476	0.01405
NM_029844.3	circRNA_0501|Chr10:25283893_25289730_-	0.901588887	0.014051
NM_029844.3	circRNA_6109|Chr9:57056714_57057861_+	0.901588887	0.014051
XM_006503323.3	circRNA_0157|Chr1:66801049_66802168_-	0.901580851	0.014053
XM_006496884.3	circRNA_3702|Chr2:143830084_143832057_-	0.901546777	0.014062
NM_001310705.1	circRNA_3155|Chr19:25061108_25065459_+	0.901494247	0.014077
NM_010340.2	circRNA_0845|Chr11:29470315_29477458_+	0.901476837	0.014082
NM_145141.2	circRNA_0501|Chr10:25283893_25289730_-	0.901453042	0.014089
NM_145141.2	circRNA_6109|Chr9:57056714_57057861_+	0.901453042	0.014089
NM_001080979.1	circRNA_4635|Chr5:43758222_43773659_-	0.901405429	0.014102
XM_006496949.3	circRNA_3155|Chr19:25061108_25065459_+	0.901388091	0.014107
XM_011243586.2	circRNA_2951|Chr18:39120119_39150118_+	0.901376657	0.01411
NM_144539.5	circRNA_4055|Chr3:126796838_126798280_+	0.90134629	0.014119
NM_001024731.2	circRNA_0501|Chr10:25283893_25289730_-	0.901302456	0.014131
NM_001024731.2	circRNA_6109|Chr9:57056714_57057861_+	0.901302456	0.014131
NM_009381.3	circRNA_3155|Chr19:25061108_25065459_+	0.901256471	0.014144
XM_006541047.3	circRNA_3276|Chr19:60563162_60564294_-	0.901190254	0.014163
XM_006499466.3	circRNA_2313|Chr15:93452117_93465245_+	0.901172667	0.014168
XM_006503323.3	circRNA_0611|Chr10:75274017_75279961_+	0.901165134	0.01417
NM_001164593.1	circRNA_0020|Chr1:10315205_10321709_+	0.901127269	0.01418
XM_006521554.3	circRNA_1042|Chr11:80385854_80403408_+	0.901124326	0.014181
NM_015814.2	circRNA_0501|Chr10:25283893_25289730_-	0.901104595	0.014187
NM_015814.2	circRNA_6109|Chr9:57056714_57057861_+	0.901104595	0.014187
NM_029685.1	circRNA_3832|Chr2:169883526_169886459_+	0.901093416	0.01419
NM_008423.2	circRNA_4926|Chr5:143080224_143081077_-	0.901074631	0.014195
NM_008524.2	circRNA_6140|Chr9:61935380_61937535_-	0.901031728	0.014207
NM_001097617.1	circRNA_0501|Chr10:25283893_25289730_-	0.901028263	0.014208
NM_001097617.1	circRNA_6109|Chr9:57056714_57057861_+	0.901028263	0.014208
XM_006522398.3	circRNA_5311|Chr6:145147636_145149357_+	0.901023372	0.01421
XM_006508885.1	circRNA_4662|Chr5:65301933_65303208_-	0.900995093	0.014218
XM_006496277.3	circRNA_3155|Chr19:25061108_25065459_+	0.900992498	0.014218
NM_008966.3	circRNA_3239|Chr19:45635735_45640521_-	0.900917257	0.01424
NM_010858.4	circRNA_0157|Chr1:66801049_66802168_-	0.900826473	0.014265
NM_001171187.1	circRNA_4987|Chr6:4529068_4531010_+	0.900769421	0.014282
XM_006533569.3	circRNA_4487|Chr4:149156607_149161694_-	0.90076048	0.014284
NM_011724.3	circRNA_3832|Chr2:169883526_169886459_+	0.900757819	0.014285
XM_011245529.2	circRNA_3832|Chr2:169883526_169886459_+	0.900740862	0.01429
XM_006528842.1	circRNA_3832|Chr2:169883526_169886459_+	0.900718185	0.014296
NM_008524.2	circRNA_0020|Chr1:10315205_10321709_+	0.900653148	0.014314
NM_023670.3	circRNA_0020|Chr1:10315205_10321709_+	0.900634498	0.01432
NM_013459.3	circRNA_4055|Chr3:126796838_126798280_+	0.900601079	0.014329
NM_013459.3	circRNA_0157|Chr1:66801049_66802168_-	0.900596483	0.01433
NM_001291930.1	circRNA_1632|Chr13:42055400_42055542_+	0.9005702	0.014338
XM_006541232.3	circRNA_0961|Chr11:67192403_67254873_+	0.900566645	0.014339
NM_001024731.2	circRNA_2888|Chr18:21010677_21020999_+	0.90053849	0.014347
NM_001303431.1	circRNA_5311|Chr6:145147636_145149357_+	0.900492543	0.01436
NM_001007570.2	circRNA_0537|Chr10:43393871_43395692_+	0.900405787	0.014385
NM_009246.3	circRNA_0537|Chr10:43393871_43395692_+	0.90038047	0.014392
XM_006518339.3	circRNA_3276|Chr19:60563162_60564294_-	0.900346657	0.014401
NM_023670.3	circRNA_0611|Chr10:75274017_75279961_+	0.900252555	0.014428
NM_026385.4	circRNA_2951|Chr18:39120119_39150118_+	0.900199851	0.014443
XM_011240779.2	circRNA_6140|Chr9:61935380_61937535_-	0.900118491	0.014466
NM_144945.3	circRNA_5811|Chr8:71992832_71998295_-	0.900085838	0.014476
XM_011241948.2	circRNA_4055|Chr3:126796838_126798280_+	0.900082901	0.014476
NM_177409.3	circRNA_3832|Chr2:169883526_169886459_+	0.900081626	0.014477
NM_001024731.2	circRNA_2951|Chr18:39120119_39150118_+	0.900021546	0.014494
NM_019759.2	circRNA_3155|Chr19:25061108_25065459_+	0.900010729	0.014497

XM_017320093.1	circRNA_3276|Chr19:60563162_60564294_-	0.899997732	0.014501
NM_001002896.2	circRNA_0020|Chr1:10315205_10321709_+	0.899958945	0.014512
NM_177789.4	circRNA_0020|Chr1:10315205_10321709_+	0.899917719	0.014523
NM_029823.2	circRNA_4728|Chr5:97027831_97045167_+	0.899834464	0.014547
NM_001190451.2	circRNA_0501|Chr10:25283893_25289730_-	0.899830778	0.014548
NM_001190451.2	circRNA_6109|Chr9:57056714_57057861_+	0.899830778	0.014548
NM_019866.1	circRNA_3702|Chr2:143830084_143832057_-	0.899822529	0.014551
NM_001110322.1	circRNA_6140|Chr9:61935380_61937535_-	0.899773437	0.014565
XM_006503665.3	circRNA_3276|Chr19:60563162_60564294_-	0.89977028	0.014566
XM_006521554.3	circRNA_4487|Chr4:149156607_149161694_-	0.899744633	0.014573
NM_001291483.1	circRNA_6140|Chr9:61935380_61937535_-	0.899710645	0.014583
XM_006525028.2	circRNA_6140|Chr9:61935380_61937535_-	0.899688111	0.014589
NM_177789.4	circRNA_0157|Chr1:66801049_66802168_-	0.899678451	0.014592
XM_011245529.2	circRNA_6390|Chr9:121727316_121731565_+	0.899634	0.014604
XM_006496593.3	circRNA_3702|Chr2:143830084_143832057_-	0.899613726	0.01461
XM_006496949.3	circRNA_2888|Chr18:21010677_21020999_+	0.899580714	0.01462
NM_001081224.2	circRNA_0501|Chr10:25283893_25289730_-	0.899534847	0.014633
NM_001081224.2	circRNA_6109|Chr9:57056714_57057861_+	0.899534847	0.014633
NM_178692.3	circRNA_6140|Chr9:61935380_61937535_-	0.899526631	0.014635
XM_006522506.3	circRNA_0020|Chr1:10315205_10321709_+	0.899453895	0.014656
NM_177578.4	circRNA_3239|Chr19:45635735_45640521_-	0.899449258	0.014657
XM_006515096.2	circRNA_5811|Chr8:71992832_71998295_-	0.899377226	0.014678
NM_008479.2	circRNA_4635|Chr5:43758222_43773659_-	0.899375956	0.014678
NM_001195084.1	circRNA_1632|Chr13:42055400_42055542_+	0.899368375	0.014681
XM_006522506.3	circRNA_4055|Chr3:126796838_126798280_+	0.899362762	0.014682
NM_008872.3	circRNA_0611|Chr10:75274017_75279961_+	0.899352422	0.014685
NM_001164593.1	circRNA_3702|Chr2:143830084_143832057_-	0.899335955	0.01469
NM_015814.2	circRNA_0157|Chr1:66801049_66802168_-	0.89929127	0.014703
NM_007555.4	circRNA_0611|Chr10:75274017_75279961_+	0.89922129	0.014723
NM_178908.3	circRNA_6446|ChrX:42217416_42250037_+	0.89917997	0.014735
NM_138955.3	circRNA_0845|Chr11:29470315_29477458_+	0.899163456	0.014739
NM_001290273.1	circRNA_3239|Chr19:45635735_45640521_-	0.899162155	0.01474
NM_009605.4	circRNA_6140|Chr9:61935380_61937535_-	0.899159701	0.01474
NM_001309809.2	circRNA_0611|Chr10:75274017_75279961_+	0.899133114	0.014748
NM_019626.3	circRNA_4055|Chr3:126796838_126798280_+	0.899101087	0.014757
NM_010055.3	circRNA_1399|Chr12:72783683_72786789_+	0.899077612	0.014764
NM_001195084.1	circRNA_3239|Chr19:45635735_45640521_-	0.899070523	0.014766
NM_172580.1	circRNA_0962|Chr11:67194348_67220998_+	0.899065705	0.014767
XM_006530238.3	circRNA_0961|Chr11:67192403_67254873_+	0.898865746	0.014825
XM_006510077.3	circRNA_0157|Chr1:66801049_66802168_-	0.89882732	0.014836
NM_010858.4	circRNA_6140|Chr9:61935380_61937535_-	0.898817738	0.014839
XM_011244046.2	circRNA_2951|Chr18:39120119_39150118_+	0.898810319	0.014841
XM_011244046.2	circRNA_4728|Chr5:97027831_97045167_+	0.898804039	0.014843
XM_006538456.3	circRNA_3832|Chr2:169883526_169886459_+	0.898786864	0.014848
NM_001291068.1	circRNA_6390|Chr9:121727316_121731565_+	0.898735008	0.014863
NM_031180.2	circRNA_6140|Chr9:61935380_61937535_-	0.89867451	0.01488
NM_026979.5	circRNA_2951|Chr18:39120119_39150118_+	0.898635605	0.014891
XM_017313062.1	circRNA_3832|Chr2:169883526_169886459_+	0.898530483	0.014922
XM_011248201.2	circRNA_0845|Chr11:29470315_29477458_+	0.898527805	0.014922
NM_007655.3	circRNA_3239|Chr19:45635735_45640521_-	0.898496244	0.014932
XM_006514444.1	circRNA_0537|Chr10:43393871_43395692_+	0.898417709	0.014954
NM_138955.3	circRNA_2313|Chr15:93452117_93465245_+	0.89833007	0.01498
NM_178753.4	circRNA_0611|Chr10:75274017_75279961_+	0.898306072	0.014987
XM_011246309.2	circRNA_2888|Chr18:21010677_21020999_+	0.898122026	0.01504
XM_006515787.3	circRNA_0611|Chr10:75274017_75279961_+	0.898089321	0.015049
XM_006525099.3	circRNA_3832|Chr2:169883526_169886459_+	0.898054947	0.015059
XM_006518339.3	circRNA_6446|ChrX:42217416_42250037_+	0.897982582	0.01508
NM_028472.2	circRNA_3155|Chr19:25061108_25065459_+	0.897910034	0.015102
XM_006517308.3	circRNA_0157|Chr1:66801049_66802168_-	0.897875029	0.015112

XM_006518339.3	circRNA_2951|Chr18:39120119_39150118_+	0.89787332	0.015112
NM_001190449.1	circRNA_3702|Chr2:143830084_143832057_-	0.897752845	0.015147
XM_006504999.1	circRNA_5639|Chr7:132771578_132779385_-	0.897714122	0.015159
NM_178715.3	circRNA_6140|Chr9:61935380_61937535_-	0.897712067	0.015159
XM_006506086.2	circRNA_0611|Chr10:75274017_75279961_+	0.897681546	0.015168
XM_011244046.2	circRNA_0501|Chr10:25283893_25289730_-	0.897616509	0.015187
XM_011244046.2	circRNA_6109|Chr9:57056714_57057861_+	0.897616509	0.015187
NM_172580.1	circRNA_1980|Chr14:50951404_50963869_+	0.89760749	0.01519
XM_006537836.1	circRNA_3702|Chr2:143830084_143832057_-	0.89756535	0.015202
NM_016982.2	circRNA_3239|Chr19:45635735_45640521_-	0.897557983	0.015204
NM_001310705.1	circRNA_0157|Chr1:66801049_66802168_-	0.897436244	0.01524
NM_001030305.2	circRNA_3155|Chr19:25061108_25065459_+	0.897405653	0.015248
XM_011250978.2	circRNA_3832|Chr2:169883526_169886459_+	0.897401058	0.01525
XM_006496277.3	circRNA_0611|Chr10:75274017_75279961_+	0.897380259	0.015256
NM_177578.4	circRNA_4055|Chr3:126796838_126798280_+	0.897365923	0.01526
NM_026979.5	circRNA_2888|Chr18:21010677_21020999_+	0.897360872	0.015262
NM_001282961.1	circRNA_2951|Chr18:39120119_39150118_+	0.897331446	0.01527
NM_029274.2	circRNA_6390|Chr9:121727316_121731565_+	0.897310158	0.015276
NM_015744.4	circRNA_0501|Chr10:25283893_25289730_-	0.897277086	0.015286
NM_015744.4	circRNA_6109|Chr9:57056714_57057861_+	0.897277086	0.015286
NM_007482.3	circRNA_0501|Chr10:25283893_25289730_-	0.897269777	0.015288
NM_007482.3	circRNA_6109|Chr9:57056714_57057861_+	0.897269777	0.015288
NM_009514.4	circRNA_0157|Chr1:66801049_66802168_-	0.897259049	0.015291
XM_006532120.2	circRNA_3702|Chr2:143830084_143832057_-	0.897234838	0.015298
NM_001002896.2	circRNA_0157|Chr1:66801049_66802168_-	0.897228207	0.0153
NM_031180.2	circRNA_2888|Chr18:21010677_21020999_+	0.897168404	0.015318
NM_001270475.1	circRNA_0537|Chr10:43393871_43395692_+	0.89716452	0.015319
NM_021475.2	circRNA_0157|Chr1:66801049_66802168_-	0.897161321	0.01532
NM_008185.3	circRNA_0376|Chr1:172173943_172187460_+	0.897159967	0.01532
NM_053080.3	circRNA_0501|Chr10:25283893_25289730_-	0.897094672	0.015339
NM_053080.3	circRNA_6109|Chr9:57056714_57057861_+	0.897094672	0.015339
XM_006496905.1	circRNA_3702|Chr2:143830084_143832057_-	0.897077932	0.015344
XM_017317978.1	circRNA_3239|Chr19:45635735_45640521_-	0.89707024	0.015347
NM_153178.4	circRNA_0537|Chr10:43393871_43395692_+	0.897038303	0.015356
NM_007529.2	circRNA_3239|Chr19:45635735_45640521_-	0.897023371	0.01536
NM_001033149.3	circRNA_0961|Chr11:67192403_67254873_+	0.897000981	0.015367
NM_011145.3	circRNA_3832|Chr2:169883526_169886459_+	0.896857106	0.015409
NM_001282961.1	circRNA_5367|Chr7:28990988_28991325_+	0.896811046	0.015423
XM_006514444.1	circRNA_3832|Chr2:169883526_169886459_+	0.896638902	0.015473
NM_019866.1	circRNA_2888|Chr18:21010677_21020999_+	0.89663333	0.015475
NM_001301295.1	circRNA_4055|Chr3:126796838_126798280_+	0.896628851	0.015476
NM_178692.3	circRNA_3276|Chr19:60563162_60564294_-	0.896571913	0.015493
NM_025711.3	circRNA_0020|Chr1:10315205_10321709_+	0.896568389	0.015494
NM_001252563.1	circRNA_3155|Chr19:25061108_25065459_+	0.896476942	0.015521
XM_017312497.1	circRNA_6140|Chr9:61935380_61937535_-	0.896441928	0.015531
XM_011246128.2	circRNA_2888|Chr18:21010677_21020999_+	0.896394473	0.015545
XM_006523030.3	circRNA_3832|Chr2:169883526_169886459_+	0.896381434	0.015549
NM_009518.2	circRNA_3155|Chr19:25061108_25065459_+	0.89637158	0.015552
XM_011246479.2	circRNA_3425|Chr2:37624173_37627526_-	0.896328193	0.015565
XM_011246479.2	circRNA_3607|Chr2:119057281_119064097_+	0.896328193	0.015565
NM_001190449.1	circRNA_0020|Chr1:10315205_10321709_+	0.896239429	0.015591
XM_006533569.3	circRNA_3954|Chr3:88346445_88349444_+	0.896230307	0.015594
NM_001110009.2	circRNA_6140|Chr9:61935380_61937535_-	0.896207189	0.0156
NM_010858.4	circRNA_3702|Chr2:143830084_143832057_-	0.896177283	0.015609
NM_010742.1	circRNA_4055|Chr3:126796838_126798280_+	0.896136084	0.015621
XM_006506145.1	circRNA_3832|Chr2:169883526_169886459_+	0.896117011	0.015627
NM_181315.4	circRNA_6140|Chr9:61935380_61937535_-	0.896066202	0.015642
XM_006518339.3	circRNA_3239|Chr19:45635735_45640521_-	0.896065454	0.015642
XM_006504999.1	circRNA_0501|Chr10:25283893_25289730_-	0.895978496	0.015668

XM_006504999.1	circRNA_6109|Chr9:57056714_57057861_+	0.895978496	0.015668
NM_001302257.1	circRNA_0611|Chr10:75274017_75279961_+	0.895953312	0.015675
XM_011243391.2	circRNA_6390|Chr9:121727316_121731565_+	0.895902455	0.01569
NM_029844.3	circRNA_5311|Chr6:145147636_145149357_+	0.895885768	0.015695
NM_001301354.1	circRNA_1632|Chr13:42055400_42055542_+	0.895880607	0.015697
XM_006505015.3	circRNA_0962|Chr11:67194348_67220998_+	0.895876578	0.015698
XM_006524777.3	circRNA_2888|Chr18:21010677_21020999_+	0.895783679	0.015726
NM_001001178.1	circRNA_0913|Chr11:54005394_54014456_-	0.895718351	0.015745
XM_006518339.3	circRNA_3702|Chr2:143830084_143832057_-	0.895419004	0.015834
NM_019866.1	circRNA_3276|Chr19:60563162_60564294_-	0.895415644	0.015835
NM_007817.2	circRNA_2888|Chr18:21010677_21020999_+	0.895412191	0.015836
NM_001164593.1	circRNA_2951|Chr18:39120119_39150118_+	0.895343496	0.015856
NM_009246.3	circRNA_2313|Chr15:93452117_93465245_+	0.895310963	0.015866
XM_006520437.2	circRNA_0020|Chr1:10315205_10321709_+	0.895294834	0.015871
XM_006529759.1	circRNA_0157|Chr1:66801049_66802168_-	0.895262712	0.01588
NM_001004357.2	circRNA_1632|Chr13:42055400_42055542_+	0.895080617	0.015935
XM_011250776.2	circRNA_0157|Chr1:66801049_66802168_-	0.895072775	0.015937
XM_006496949.3	circRNA_1632|Chr13:42055400_42055542_+	0.895055739	0.015942
NM_018866.2	circRNA_3155|Chr19:25061108_25065459_+	0.895040594	0.015947
NM_015744.4	circRNA_4728|Chr5:97027831_97045167_+	0.895027978	0.01595
NM_152915.1	circRNA_4728|Chr5:97027831_97045167_+	0.894996262	0.01596
XM_006539726.3	circRNA_0962|Chr11:67194348_67220998_+	0.894990042	0.015962
NM_001290993.1	circRNA_2888|Chr18:21010677_21020999_+	0.894949494	0.015974
XM_006530131.1	circRNA_3954|Chr3:88346445_88349444_+	0.894907832	0.015986
NM_001305585.1	circRNA_2888|Chr18:21010677_21020999_+	0.894887057	0.015992
NM_010055.3	circRNA_3425|Chr2:37624173_37627526_-	0.894767888	0.016028
NM_010055.3	circRNA_3607|Chr2:119057281_119064097_+	0.894767888	0.016028
XM_006513165.1	circRNA_6446|ChrX:42217416_42250037_+	0.894714427	0.016044
XM_017318541.1	circRNA_0020|Chr1:10315205_10321709_+	0.894713806	0.016044
XM_011249157.2	circRNA_5311|Chr6:145147636_145149357_+	0.894695178	0.01605
NM_001110009.2	circRNA_3155|Chr19:25061108_25065459_+	0.89450423	0.016107
XM_006527452.3	circRNA_0501|Chr10:25283893_25289730_-	0.894443735	0.016125
XM_006527452.3	circRNA_6109|Chr9:57056714_57057861_+	0.894443735	0.016125
NM_177409.3	circRNA_0537|Chr10:43393871_43395692_+	0.894440245	0.016126
NM_023670.3	circRNA_0501|Chr10:25283893_25289730_-	0.89439196	0.016141
NM_023670.3	circRNA_6109|Chr9:57056714_57057861_+	0.89439196	0.016141
XM_011239558.2	circRNA_5074|Chr6:40685277_40747150_+	0.894355137	0.016152
NM_027455.3	circRNA_4055|Chr3:126796838_126798280_+	0.894286541	0.016172
NM_010858.4	circRNA_0020|Chr1:10315205_10321709_+	0.894270745	0.016177
XM_006503665.3	circRNA_6446|ChrX:42217416_42250037_+	0.894246125	0.016184
NM_001305585.1	circRNA_2951|Chr18:39120119_39150118_+	0.894186986	0.016202
NM_011169.5	circRNA_0020|Chr1:10315205_10321709_+	0.894088147	0.016232
NM_138955.3	circRNA_4926|Chr5:143080224_143081077_-	0.894077134	0.016235
XM_006533539.3	circRNA_2951|Chr18:39120119_39150118_+	0.894073348	0.016236
NM_153801.3	circRNA_4926|Chr5:143080224_143081077_-	0.894067581	0.016238
XM_006530353.3	circRNA_6390|Chr9:121727316_121731565_+	0.893968758	0.016268
NM_175692.3	circRNA_4662|Chr5:65301933_65303208_-	0.893845281	0.016305
NM_199304.1	circRNA_3003|Chr18:67545615_67587859_-	0.893805073	0.016317
NM_016809.6	circRNA_0020|Chr1:10315205_10321709_+	0.893764668	0.016329
NM_178753.4	circRNA_3155|Chr19:25061108_25065459_+	0.893752072	0.016333
NM_029685.1	circRNA_0961|Chr11:67192403_67254873_+	0.893747215	0.016335
NM_001030305.2	circRNA_2951|Chr18:39120119_39150118_+	0.893742701	0.016336
NM_001310705.1	circRNA_4662|Chr5:65301933_65303208_-	0.893716975	0.016344
NM_001310636.1	circRNA_2888|Chr18:21010677_21020999_+	0.893698158	0.01635
XM_017316439.1	circRNA_0157|Chr1:66801049_66802168_-	0.893682073	0.016354
NM_028472.2	circRNA_0020|Chr1:10315205_10321709_+	0.893557462	0.016392
NM_001190448.1	circRNA_2951|Chr18:39120119_39150118_+	0.893527764	0.016401
NM_173427.2	circRNA_6140|Chr9:61935380_61937535_-	0.893503107	0.016408
NM_024406.2	circRNA_6446|ChrX:42217416_42250037_+	0.893459705	0.016422

XM_011250776.2	circRNA_6446|ChrX:42217416_42250037_+	0.893454311	0.016423
XM_006525075.3	circRNA_0020|Chr1:10315205_10321709_+	0.893444554	0.016426
XM_017316056.1	circRNA_5367|Chr7:28990988_28991325_+	0.893440687	0.016427
XM_017316439.1	circRNA_2888|Chr18:21010677_21020999_+	0.893427429	0.016431
XM_006503323.3	circRNA_1632|Chr13:42055400_42055542_+	0.893364038	0.016451
NM_001134300.2	circRNA_3954|Chr3:88346445_88349444_+	0.893349682	0.016455
NM_016933.3	circRNA_3702|Chr2:143830084_143832057_-	0.893316946	0.016465
XM_006496949.3	circRNA_3643|Chr2:122441559_122486090_+	0.89324448	0.016487
NM_028973.2	circRNA_3702|Chr2:143830084_143832057_-	0.89322271	0.016493
XM_011245529.2	circRNA_0537|Chr10:43393871_43395692_+	0.893196156	0.016501
NM_001195084.1	circRNA_3702|Chr2:143830084_143832057_-	0.893170286	0.016509
NM_013468.3	circRNA_5074|Chr6:40685277_40747150_+	0.893170059	0.016509
NM_028748.2	circRNA_1042|Chr11:80385854_80403408_+	0.893167841	0.01651
NM_010140.3	circRNA_0157|Chr1:66801049_66802168_-	0.893158408	0.016513
NM_008966.3	circRNA_4055|Chr3:126796838_126798280_+	0.893139978	0.016518
NM_023670.3	circRNA_2951|Chr18:39120119_39150118_+	0.893117527	0.016525
NM_001309809.2	circRNA_3155|Chr19:25061108_25065459_+	0.893116256	0.016526
XM_006533452.3	circRNA_0157|Chr1:66801049_66802168_-	0.893064207	0.016541
NM_145635.2	circRNA_4728|Chr5:97027831_97045167_+	0.893007924	0.016559
NM_011044.2	circRNA_6446|ChrX:42217416_42250037_+	0.892985879	0.016565
XM_006533452.3	circRNA_2888|Chr18:21010677_21020999_+	0.892981248	0.016567
NM_011169.5	circRNA_6446|ChrX:42217416_42250037_+	0.892950478	0.016576
NM_011145.3	circRNA_0537|Chr10:43393871_43395692_+	0.892919188	0.016586
NM_133871.2	circRNA_1042|Chr11:80385854_80403408_+	0.892886514	0.016595
NM_001161541.1	circRNA_2951|Chr18:39120119_39150118_+	0.892874802	0.016599
XM_006510077.3	circRNA_3239|Chr19:45635735_45640521_-	0.892715892	0.016647
NM_178184.2	circRNA_4055|Chr3:126796838_126798280_+	0.892691563	0.016655
XM_006510701.3	circRNA_6390|Chr9:121727316_121731565_+	0.892578991	0.016689
NM_016809.6	circRNA_5639|Chr7:132771578_132779385_-	0.892561377	0.016695
NM_175692.3	circRNA_0020|Chr1:10315205_10321709_+	0.892526449	0.016705
NM_146241.2	circRNA_3702|Chr2:143830084_143832057_-	0.892518847	0.016707
NM_175271.4	circRNA_3702|Chr2:143830084_143832057_-	0.89246983	0.016722
NM_026979.5	circRNA_0501|Chr10:25283893_25289730_-	0.892366546	0.016754
NM_026979.5	circRNA_6109|Chr9:57056714_57057861_+	0.892366546	0.016754
XM_017316056.1	circRNA_0157|Chr1:66801049_66802168_-	0.892336337	0.016763
NM_010858.4	circRNA_3239|Chr19:45635735_45640521_-	0.892280544	0.01678
NM_010340.2	circRNA_5811|Chr8:71992832_71998295_-	0.892253002	0.016789
NM_175520.4	circRNA_3702|Chr2:143830084_143832057_-	0.892209953	0.016802
NM_175563.5	circRNA_0845|Chr11:29470315_29477458_+	0.892206022	0.016803
NM_025865.2	circRNA_6412|ChrMT:13917_14141_-	0.892164847	0.016816
NM_001110009.2	circRNA_2951|Chr18:39120119_39150118_+	0.892144028	0.016822
NM_001301295.1	circRNA_0157|Chr1:66801049_66802168_-	0.892082445	0.016841
NM_001309809.2	circRNA_0020|Chr1:10315205_10321709_+	0.892075145	0.016843
XM_006524382.3	circRNA_1042|Chr11:80385854_80403408_+	0.89204164	0.016853
NM_009518.2	circRNA_2951|Chr18:39120119_39150118_+	0.892034803	0.016855
XM_006520437.2	circRNA_4728|Chr5:97027831_97045167_+	0.892013643	0.016862
NM_010608.2	circRNA_2951|Chr18:39120119_39150118_+	0.89201148	0.016863
XM_006502235.3	circRNA_3003|Chr18:67545615_67587859_-	0.892010289	0.016863
NM_145635.2	circRNA_6140|Chr9:61935380_61937535_-	0.891973489	0.016874
NM_001110322.1	circRNA_4055|Chr3:126796838_126798280_+	0.891965515	0.016877
NM_001097617.1	circRNA_0611|Chr10:75274017_75279961_+	0.891897996	0.016897
NM_001002896.2	circRNA_6446|ChrX:42217416_42250037_+	0.891877717	0.016904
NM_145635.2	circRNA_5639|Chr7:132771578_132779385_-	0.8918612	0.016909
XM_011241322.1	circRNA_0845|Chr11:29470315_29477458_+	0.891803604	0.016926
XM_017315376.1	circRNA_0157|Chr1:66801049_66802168_-	0.891784216	0.016932
XM_006508867.2	circRNA_0157|Chr1:66801049_66802168_-	0.891665629	0.016969
NM_007641.5	circRNA_3239|Chr19:45635735_45640521_-	0.891603175	0.016988
NM_011854.2	circRNA_0845|Chr11:29470315_29477458_+	0.891558607	0.017002
XM_017315376.1	circRNA_0501|Chr10:25283893_25289730_-	0.891497903	0.01702

XM_017315376.1	circRNA_6109|Chr9:57056714_57057861_+	0.891497903	0.01702
NM_001291483.1	circRNA_5639|Chr7:132771578_132779385_-	0.891497759	0.01702
NM_001159424.2	circRNA_0501|Chr10:25283893_25289730_-	0.891442014	0.017038
NM_001159424.2	circRNA_6109|Chr9:57056714_57057861_+	0.891442014	0.017038
NM_175271.4	circRNA_5311|Chr6:145147636_145149357_+	0.891429035	0.017042
NM_010859.2	circRNA_3832|Chr2:169883526_169886459_+	0.891385615	0.017055
XM_017315313.1	circRNA_3276|Chr19:60563162_60564294_-	0.891287675	0.017085
XM_006501309.3	circRNA_5811|Chr8:71992832_71998295_-	0.891276732	0.017089
XM_006506340.3	circRNA_0845|Chr11:29470315_29477458_+	0.891246639	0.017098
XM_017316676.1	circRNA_6390|Chr9:121727316_121731565_+	0.891150697	0.017127
NM_001159407.1	circRNA_3239|Chr19:45635735_45640521_-	0.891145237	0.017129
NM_144945.3	circRNA_3954|Chr3:88346445_88349444_+	0.891081649	0.017149
NM_001289875.1	circRNA_4926|Chr5:143080224_143081077_-	0.891027251	0.017166
NM_145226.2	circRNA_5074|Chr6:40685277_40747150_+	0.890995177	0.017175
XM_011239969.2	circRNA_6140|Chr9:61935380_61937535_-	0.89098128	0.01718
XM_017320735.1	circRNA_2951|Chr18:39120119_39150118_+	0.890895281	0.017206
NM_001291930.1	circRNA_4987|Chr6:4529068_4531010_+	0.890858836	0.017218
NM_008185.3	circRNA_3239|Chr19:45635735_45640521_-	0.8908061	0.017234
NM_152915.1	circRNA_5639|Chr7:132771578_132779385_-	0.890777975	0.017243
NM_145141.2	circRNA_3239|Chr19:45635735_45640521_-	0.890743388	0.017253
NM_133871.2	circRNA_1399|Chr12:72783683_72786789_+	0.890726325	0.017259
NM_019626.3	circRNA_0020|Chr1:10315205_10321709_+	0.890695951	0.017268
NM_011658.2	circRNA_3239|Chr19:45635735_45640521_-	0.890657294	0.01728
NM_010090.2	circRNA_3155|Chr19:25061108_25065459_+	0.890621318	0.017291
XM_006500919.3	circRNA_6140|Chr9:61935380_61937535_-	0.890575071	0.017306
NM_001290993.1	circRNA_0157|Chr1:66801049_66802168_-	0.890538425	0.017317
NM_009381.3	circRNA_2888|Chr18:21010677_21020999_+	0.890513545	0.017325
NM_001313939.1	circRNA_0157|Chr1:66801049_66802168_-	0.890493448	0.017331
XM_006527452.3	circRNA_0157|Chr1:66801049_66802168_-	0.89047344	0.017337
NM_152915.1	circRNA_0020|Chr1:10315205_10321709_+	0.890453273	0.017343
NM_024406.2	circRNA_5311|Chr6:145147636_145149357_+	0.890413005	0.017356
XM_006505757.3	circRNA_5311|Chr6:145147636_145149357_+	0.890396011	0.017361
NM_018866.2	circRNA_3239|Chr19:45635735_45640521_-	0.890376331	0.017367
XM_006524328.3	circRNA_4926|Chr5:143080224_143081077_-	0.890350333	0.017375
NM_008067.4	circRNA_2951|Chr18:39120119_39150118_+	0.89034921	0.017376
XM_006496949.3	circRNA_0501|Chr10:25283893_25289730_-	0.89027123	0.0174
XM_006496949.3	circRNA_6109|Chr9:57056714_57057861_+	0.89027123	0.0174
XM_006530585.3	circRNA_3832|Chr2:169883526_169886459_+	0.890177072	0.017429
NM_001290822.1	circRNA_4728|Chr5:97027831_97045167_+	0.890164786	0.017433
NM_008623.5	circRNA_3276|Chr19:60563162_60564294_-	0.890117639	0.017448
NM_024406.2	circRNA_0376|Chr1:172173943_172187460_+	0.890114087	0.017449
NM_023893.4	circRNA_6140|Chr9:61935380_61937535_-	0.890105519	0.017452
NM_001242558.1	circRNA_6390|Chr9:121727316_121731565_+	0.890042699	0.017471
NM_028838.2	circRNA_0961|Chr11:67192403_67254873_+	0.890037454	0.017473
NM_010090.2	circRNA_3702|Chr2:143830084_143832057_-	0.890020555	0.017478
XM_011243266.1	circRNA_0961|Chr11:67192403_67254873_+	0.889999701	0.017485
NM_138955.3	circRNA_6390|Chr9:121727316_121731565_+	0.889989666	0.017488
XM_017321602.1	circRNA_5311|Chr6:145147636_145149357_+	0.88998569	0.017489
NM_144936.1	circRNA_0157|Chr1:66801049_66802168_-	0.889937485	0.017504
NM_001142804.1	circRNA_2888|Chr18:21010677_21020999_+	0.889929947	0.017506
NM_021475.2	circRNA_0501|Chr10:25283893_25289730_-	0.889921971	0.017509
NM_021475.2	circRNA_6109|Chr9:57056714_57057861_+	0.889921971	0.017509
NM_001313939.1	circRNA_6140|Chr9:61935380_61937535_-	0.889886563	0.01752
NM_029982.1	circRNA_3954|Chr3:88346445_88349444_+	0.889841551	0.017534
NM_001081224.2	circRNA_0376|Chr1:172173943_172187460_+	0.889826802	0.017539
NM_008623.5	circRNA_0501|Chr10:25283893_25289730_-	0.889823191	0.01754
NM_008623.5	circRNA_6109|Chr9:57056714_57057861_+	0.889823191	0.01754
XM_011241322.1	circRNA_3954|Chr3:88346445_88349444_+	0.889804914	0.017545
NM_030206.4	circRNA_6446|ChrX:42217416_42250037_+	0.889764727	0.017558

NM_172580.1	circRNA_6412|ChrMT:13917_14141_-	0.889748708	0.017563
XM_011250176.1	circRNA_0845|Chr11:29470315_29477458_+	0.889748675	0.017563
XM_006530294.3	circRNA_5811|Chr8:71992832_71998295_-	0.889719233	0.017572
NM_019732.2	circRNA_3954|Chr3:88346445_88349444_+	0.889654436	0.017592
NM_025422.4	circRNA_3155|Chr19:25061108_25065459_+	0.889594709	0.017611
XM_017316056.1	circRNA_2888|Chr18:21010677_21020999_+	0.889424145	0.017665
NM_001033286.2	circRNA_3832|Chr2:169883526_169886459_+	0.889391458	0.017675
NM_177595.4	circRNA_3702|Chr2:143830084_143832057_-	0.889369064	0.017682
NM_001310705.1	circRNA_4728|Chr5:97027831_97045167_+	0.889304552	0.017702
XM_006530294.3	circRNA_5074|Chr6:40685277_40747150_+	0.889299619	0.017704
NM_001164593.1	circRNA_0611|Chr10:75274017_75279961_+	0.889298213	0.017704
NM_172454.2	circRNA_3003|Chr18:67545615_67587859_-	0.889277293	0.017711
NM_007641.5	circRNA_3155|Chr19:25061108_25065459_+	0.889257192	0.017717
XM_006516224.3	circRNA_0537|Chr10:43393871_43395692_+	0.889230676	0.017725
NM_178908.3	circRNA_3702|Chr2:143830084_143832057_-	0.889228749	0.017726
NM_207231.1	circRNA_4055|Chr3:126796838_126798280_+	0.889213145	0.017731
XM_006515787.3	circRNA_4987|Chr6:4529068_4531010_+	0.889206641	0.017733
XM_006529829.3	circRNA_3155|Chr19:25061108_25065459_+	0.889205915	0.017733
NM_025711.3	circRNA_2951|Chr18:39120119_39150118_+	0.889129007	0.017757
NM_001159424.2	circRNA_6140|Chr9:61935380_61937535_-	0.88912778	0.017758
XM_017322026.1	circRNA_0376|Chr1:172173943_172187460_+	0.889120244	0.01776
XM_006525028.2	circRNA_3155|Chr19:25061108_25065459_+	0.889079331	0.017773
NM_001285867.1	circRNA_3832|Chr2:169883526_169886459_+	0.889075	0.017774
XM_006520023.2	circRNA_6140|Chr9:61935380_61937535_-	0.889025384	0.01779
NM_133871.2	circRNA_3425|Chr2:37624173_37627526_-	0.888989585	0.017801
NM_133871.2	circRNA_3607|Chr2:119057281_119064097_+	0.888989585	0.017801
XM_006508885.1	circRNA_3155|Chr19:25061108_25065459_+	0.888973663	0.017806
XM_017317978.1	circRNA_3702|Chr2:143830084_143832057_-	0.888957492	0.017811
NM_010090.2	circRNA_3239|Chr19:45635735_45640521_-	0.88895192	0.017813
NM_008204.2	circRNA_3276|Chr19:60563162_60564294_-	0.8889324	0.017819
XM_006501237.3	circRNA_6446|ChrX:42217416_42250037_+	0.888921312	0.017822
XM_006498086.3	circRNA_2888|Chr18:21010677_21020999_+	0.888828932	0.017852
XM_006524777.3	circRNA_0020|Chr1:10315205_10321709_+	0.88882532	0.017853
XM_017316897.1	circRNA_1980|Chr14:50951404_50963869_+	0.888802947	0.01786
NM_001310705.1	circRNA_2951|Chr18:39120119_39150118_+	0.888768167	0.017871
XM_011246479.2	circRNA_1399|Chr12:72783683_72786789_+	0.888746751	0.017877
XM_017316944.1	circRNA_3155|Chr19:25061108_25065459_+	0.888731573	0.017882
NM_008623.5	circRNA_4987|Chr6:4529068_4531010_+	0.888716087	0.017887
NM_009504.4	circRNA_3954|Chr3:88346445_88349444_+	0.888687222	0.017896
XM_006520186.2	circRNA_0376|Chr1:172173943_172187460_+	0.888685848	0.017897
XM_006533452.3	circRNA_3155|Chr19:25061108_25065459_+	0.888667248	0.017902
NM_177371.3	circRNA_6140|Chr9:61935380_61937535_-	0.888654496	0.017907
NM_145141.2	circRNA_6140|Chr9:61935380_61937535_-	0.888464016	0.017967
XM_006520186.2	circRNA_0611|Chr10:75274017_75279961_+	0.888458546	0.017968
XM_006510077.3	circRNA_4055|Chr3:126796838_126798280_+	0.888381728	0.017993
XM_006508990.2	circRNA_0845|Chr11:29470315_29477458_+	0.888336657	0.018007
NM_152915.1	circRNA_4662|Chr5:65301933_65303208_-	0.888210844	0.018047
NM_001159424.2	circRNA_6446|ChrX:42217416_42250037_+	0.888183497	0.018055
XM_006526449.3	circRNA_1399|Chr12:72783683_72786789_+	0.888163714	0.018062
XM_006507725.2	circRNA_3832|Chr2:169883526_169886459_+	0.888102079	0.018081
NM_001304266.1	circRNA_6390|Chr9:121727316_121731565_+	0.888092609	0.018084
NM_001164593.1	circRNA_6140|Chr9:61935380_61937535_-	0.888062921	0.018094
NM_024406.2	circRNA_4728|Chr5:97027831_97045167_+	0.888046593	0.018099
XM_017317978.1	circRNA_5311|Chr6:145147636_145149357_+	0.888033986	0.018103
XM_017319626.1	circRNA_0961|Chr11:67192403_67254873_+	0.887985646	0.018118
NM_023893.4	circRNA_3702|Chr2:143830084_143832057_-	0.887970906	0.018123
NM_007555.4	circRNA_4728|Chr5:97027831_97045167_+	0.887970115	0.018123
XM_011248602.2	circRNA_4926|Chr5:143080224_143081077_-	0.887939783	0.018133
NM_028472.2	circRNA_5311|Chr6:145147636_145149357_+	0.887798556	0.018177

NM_178782.4	circRNA_0537|Chr10:43393871_43395692_+	0.88770792	0.018206
NM_008032.3	circRNA_4926|Chr5:143080224_143081077_-	0.8876619	0.018221
NM_001290273.1	circRNA_6446|ChrX:42217416_42250037_+	0.887632133	0.01823
XM_006532120.2	circRNA_5311|Chr6:145147636_145149357_+	0.887625649	0.018232
NM_007655.3	circRNA_3155|Chr19:25061108_25065459_+	0.887562867	0.018252
XM_006499466.3	circRNA_3832|Chr2:169883526_169886459_+	0.887451423	0.018288
NM_148932.2	circRNA_3954|Chr3:88346445_88349444_+	0.887446788	0.018289
NM_008524.2	circRNA_3702|Chr2:143830084_143832057_-	0.887426858	0.018296
NM_009135.2	circRNA_1632|Chr13:42055400_42055542_+	0.887391708	0.018307
NM_001004357.2	circRNA_0501|Chr10:25283893_25289730_-	0.887371407	0.018313
NM_001004357.2	circRNA_6109|Chr9:57056714_57057861_+	0.887371407	0.018313
NM_001301354.1	circRNA_0501|Chr10:25283893_25289730_-	0.887346933	0.018321
NM_001301354.1	circRNA_6109|Chr9:57056714_57057861_+	0.887346933	0.018321
NM_001004357.2	circRNA_2951|Chr18:39120119_39150118_+	0.887215605	0.018363
NM_172907.3	circRNA_4055|Chr3:126796838_126798280_+	0.887183291	0.018373
NM_001161746.1	circRNA_0961|Chr11:67192403_67254873_+	0.887137479	0.018388
XM_006541047.3	circRNA_2888|Chr18:21010677_21020999_+	0.887133024	0.01839
NM_001290993.1	circRNA_0611|Chr10:75274017_75279961_+	0.887063431	0.018412
NM_181315.4	circRNA_0157|Chr1:66801049_66802168_-	0.887045274	0.018418
NM_001033286.2	circRNA_5990|Chr9:3475483_3477962_+	0.887034635	0.018421
NM_009349.3	circRNA_0611|Chr10:75274017_75279961_+	0.886936934	0.018452
NM_020001.2	circRNA_5639|Chr7:132771578_132779385_-	0.88685125	0.01848
NM_016809.6	circRNA_0376|Chr1:172173943_172187460_+	0.886688887	0.018532
XM_011250176.1	circRNA_6390|Chr9:121727316_121731565_+	0.886675735	0.018536
XM_017313038.1	circRNA_0157|Chr1:66801049_66802168_-	0.886619769	0.018554
XM_017320093.1	circRNA_6140|Chr9:61935380_61937535_-	0.886614876	0.018555
XM_011250176.1	circRNA_3954|Chr3:88346445_88349444_+	0.886610476	0.018557
NM_008423.2	circRNA_0537|Chr10:43393871_43395692_+	0.886596699	0.018561
NM_011581.3	circRNA_4055|Chr3:126796838_126798280_+	0.886497282	0.018593
NM_001142804.1	circRNA_6446|ChrX:42217416_42250037_+	0.886443759	0.01861
XM_011241322.1	circRNA_2313|Chr15:93452117_93465245_+	0.886424535	0.018617
NM_009675.2	circRNA_0611|Chr10:75274017_75279961_+	0.886355917	0.018639
NM_016659.3	circRNA_4055|Chr3:126796838_126798280_+	0.886355027	0.018639
NM_001110009.2	circRNA_5639|Chr7:132771578_132779385_-	0.886225634	0.018681
NM_027416.3	circRNA_0157|Chr1:66801049_66802168_-	0.886217888	0.018683
NM_026979.5	circRNA_4987|Chr6:4529068_4531010_+	0.886201327	0.018688
NM_031180.2	circRNA_0376|Chr1:172173943_172187460_+	0.886162534	0.018701
NM_001110322.1	circRNA_0501|Chr10:25283893_25289730_-	0.886153053	0.018704
NM_001110322.1	circRNA_6109|Chr9:57056714_57057861_+	0.886153053	0.018704
XM_006538456.3	circRNA_0537|Chr10:43393871_43395692_+	0.886122793	0.018714
XM_011242311.2	circRNA_6390|Chr9:121727316_121731565_+	0.886103162	0.01872
NM_001190258.1	circRNA_5639|Chr7:132771578_132779385_-	0.886072435	0.01873
XM_011238891.1	circRNA_4055|Chr3:126796838_126798280_+	0.886063297	0.018733
XM_017314944.1	circRNA_2888|Chr18:21010677_21020999_+	0.886040508	0.01874
NM_001318003.2	circRNA_4728|Chr5:97027831_97045167_+	0.886019201	0.018747
NM_145210.2	circRNA_5811|Chr8:71992832_71998295_-	0.88594566	0.018771
NM_007529.2	circRNA_0501|Chr10:25283893_25289730_-	0.885881779	0.018791
NM_007529.2	circRNA_6109|Chr9:57056714_57057861_+	0.885881779	0.018791
NM_027416.3	circRNA_3702|Chr2:143830084_143832057_-	0.885797936	0.018818
NM_175563.5	circRNA_3832|Chr2:169883526_169886459_+	0.885761251	0.01883
NM_029844.3	circRNA_6446|ChrX:42217416_42250037_+	0.885586595	0.018887
XM_006516510.1	circRNA_0157|Chr1:66801049_66802168_-	0.885571442	0.018892
NM_008185.3	circRNA_0020|Chr1:10315205_10321709_+	0.885560122	0.018895
NM_019759.2	circRNA_0020|Chr1:10315205_10321709_+	0.885535039	0.018903
NM_009019.2	circRNA_3702|Chr2:143830084_143832057_-	0.885506389	0.018913
NM_007641.5	circRNA_4055|Chr3:126796838_126798280_+	0.88547753	0.018922
NM_001024731.2	circRNA_0157|Chr1:66801049_66802168_-	0.885447448	0.018932
NM_178782.4	circRNA_3832|Chr2:169883526_169886459_+	0.885290576	0.018983
NM_011044.2	circRNA_3702|Chr2:143830084_143832057_-	0.885284154	0.018985

NM_010779.2	circRNA_6140|Chr9:61935380_61937535_-	0.885278386	0.018987
NM_205810.4	circRNA_1632|Chr13:42055400_42055542_+	0.885271444	0.018989
NM_001290822.1	circRNA_5367|Chr7:28990988_28991325_+	0.885228649	0.019003
NM_007641.5	circRNA_3276|Chr19:60563162_60564294_-	0.88522753	0.019003
XM_006510077.3	circRNA_4728|Chr5:97027831_97045167_+	0.885177007	0.01902
XM_011248394.1	circRNA_5367|Chr7:28990988_28991325_+	0.885162043	0.019024
XM_006506624.2	circRNA_5311|Chr6:145147636_145149357_+	0.885145434	0.01903
NM_001305585.1	circRNA_5367|Chr7:28990988_28991325_+	0.885098131	0.019045
NM_001033149.3	circRNA_3832|Chr2:169883526_169886459_+	0.885084677	0.01905
XM_017313038.1	circRNA_6140|Chr9:61935380_61937535_-	0.885076619	0.019052
NM_001289782.1	circRNA_3832|Chr2:169883526_169886459_+	0.885027057	0.019068
XM_011244046.2	circRNA_4662|Chr5:65301933_65303208_-	0.884969501	0.019087
XM_006518921.3	circRNA_3702|Chr2:143830084_143832057_-	0.884926651	0.019101
XM_006527452.3	circRNA_2888|Chr18:21010677_21020999_+	0.884926576	0.019101
NM_001110009.2	circRNA_4662|Chr5:65301933_65303208_-	0.884915445	0.019105
NM_001159424.2	circRNA_0157|Chr1:66801049_66802168_-	0.88489947	0.01911
NM_001081224.2	circRNA_0020|Chr1:10315205_10321709_+	0.884888717	0.019113
XM_006521796.1	circRNA_0157|Chr1:66801049_66802168_-	0.884886051	0.019114
NM_029844.3	circRNA_2951|Chr18:39120119_39150118_+	0.884818334	0.019136
XM_011238891.1	circRNA_2888|Chr18:21010677_21020999_+	0.884782757	0.019148
NM_024406.2	circRNA_3239|Chr19:45635735_45640521_-	0.884734464	0.019163
NM_016982.2	circRNA_5311|Chr6:145147636_145149357_+	0.884675096	0.019183
NM_053080.3	circRNA_3155|Chr19:25061108_25065459_+	0.884636524	0.019195
NM_013868.4	circRNA_3832|Chr2:169883526_169886459_+	0.884624874	0.019199
XM_017315376.1	circRNA_0611|Chr10:75274017_75279961_+	0.884606168	0.019205
NM_029823.2	circRNA_0376|Chr1:172173943_172187460_+	0.884599378	0.019208
NM_145226.2	circRNA_4487|Chr4:149156607_149161694_-	0.884599338	0.019208
XM_017317034.1	circRNA_0845|Chr11:29470315_29477458_+	0.884595143	0.019209
XM_006508867.2	circRNA_5311|Chr6:145147636_145149357_+	0.884525723	0.019232
NM_026535.2	circRNA_6140|Chr9:61935380_61937535_-	0.88445784	0.019254
NM_001033221.3	circRNA_6390|Chr9:121727316_121731565_+	0.884446514	0.019257
XM_017315313.1	circRNA_3155|Chr19:25061108_25065459_+	0.884427799	0.019264
XM_006538687.2	circRNA_6390|Chr9:121727316_121731565_+	0.884402468	0.019272
NM_021475.2	circRNA_0611|Chr10:75274017_75279961_+	0.884365332	0.019284
XM_006501237.3	circRNA_5311|Chr6:145147636_145149357_+	0.884307565	0.019303
XM_006503323.3	circRNA_4987|Chr6:4529068_4531010_+	0.884301514	0.019305
XM_006538830.1	circRNA_6390|Chr9:121727316_121731565_+	0.884257777	0.019319
XM_006522398.3	circRNA_2951|Chr18:39120119_39150118_+	0.884240883	0.019325
XM_006533452.3	circRNA_3239|Chr19:45635735_45640521_-	0.884227112	0.019329
NM_025711.3	circRNA_3155|Chr19:25061108_25065459_+	0.884204642	0.019337
XM_006496277.3	circRNA_0376|Chr1:172173943_172187460_+	0.884192062	0.019341
XM_006499444.3	circRNA_1042|Chr11:80385854_80403408_+	0.884174615	0.019346
XM_006511311.2	circRNA_0962|Chr11:67194348_67220998_+	0.884173802	0.019347
XM_006533539.3	circRNA_2888|Chr18:21010677_21020999_+	0.884154336	0.019353
XM_006524777.3	circRNA_0157|Chr1:66801049_66802168_-	0.884152883	0.019353
XM_006506152.3	circRNA_0845|Chr11:29470315_29477458_+	0.884077981	0.019378
NM_001033167.3	circRNA_5074|Chr6:40685277_40747150_+	0.884066455	0.019382
NM_175563.5	circRNA_0537|Chr10:43393871_43395692_+	0.884003231	0.019402
NM_011704.3	circRNA_0157|Chr1:66801049_66802168_-	0.883970174	0.019413
NM_009020.3	circRNA_3702|Chr2:143830084_143832057_-	0.883953573	0.019419
NM_001110009.2	circRNA_3702|Chr2:143830084_143832057_-	0.88394784	0.019421
NM_001109985.1	circRNA_3832|Chr2:169883526_169886459_+	0.883938088	0.019424
XM_017315313.1	circRNA_3239|Chr19:45635735_45640521_-	0.883911787	0.019432
XM_006529382.3	circRNA_3832|Chr2:169883526_169886459_+	0.883895191	0.019438
NM_145741.2	circRNA_6140|Chr9:61935380_61937535_-	0.883879192	0.019443
NM_001190451.2	circRNA_0611|Chr10:75274017_75279961_+	0.883861421	0.019449
NM_010450.3	circRNA_4662|Chr5:65301933_65303208_-	0.883829819	0.019459
XM_006516510.1	circRNA_0376|Chr1:172173943_172187460_+	0.883809644	0.019466
NM_001190448.1	circRNA_0501|Chr10:25283893_25289730_-	0.883772801	0.019478

NM_001190448.1	circRNA_6109|Chr9:57056714_57057861_+	0.883772801	0.019478
NM_172790.2	circRNA_6390|Chr9:121727316_121731565_+	0.883768014	0.01948
NM_001310636.1	circRNA_2951|Chr18:39120119_39150118_+	0.883736152	0.01949
NM_011082.3	circRNA_0376|Chr1:172173943_172187460_+	0.883558267	0.019549
XM_006527452.3	circRNA_2951|Chr18:39120119_39150118_+	0.883538313	0.019555
NM_009223.3	circRNA_4055|Chr3:126796838_126798280_+	0.883393981	0.019603
NM_001198841.1	circRNA_3832|Chr2:169883526_169886459_+	0.883382766	0.019606
XM_006517308.3	circRNA_2888|Chr18:21010677_21020999_+	0.883365427	0.019612
NM_010140.3	circRNA_0611|Chr10:75274017_75279961_+	0.883363197	0.019613
NM_148932.2	circRNA_3832|Chr2:169883526_169886459_+	0.883315942	0.019628
NM_010859.2	circRNA_0962|Chr11:67194348_67220998_+	0.883244221	0.019652
NM_023143.3	circRNA_5311|Chr6:145147636_145149357_+	0.883197086	0.019668
NM_024406.2	circRNA_2951|Chr18:39120119_39150118_+	0.883111978	0.019696
XM_006530294.3	circRNA_3003|Chr18:67545615_67587859_-	0.883038914	0.01972
NM_025711.3	circRNA_5311|Chr6:145147636_145149357_+	0.883010561	0.019729
NM_145210.2	circRNA_0845|Chr11:29470315_29477458_+	0.883006207	0.019731
XM_006529759.1	circRNA_6446|ChrX:42217416_42250037_+	0.882869918	0.019776
NM_007482.3	circRNA_2951|Chr18:39120119_39150118_+	0.882857874	0.01978
XM_017318541.1	circRNA_2951|Chr18:39120119_39150118_+	0.88285062	0.019782
XM_006529753.2	circRNA_6390|Chr9:121727316_121731565_+	0.882833451	0.019788
XM_006502235.3	circRNA_1042|Chr11:80385854_80403408_+	0.882803429	0.019798
NM_001159407.1	circRNA_2951|Chr18:39120119_39150118_+	0.882784212	0.019804
XM_011250776.2	circRNA_0501|Chr10:25283893_25289730_-	0.882742091	0.019818
XM_011250776.2	circRNA_6109|Chr9:57056714_57057861_+	0.882742091	0.019818
XM_006515096.2	circRNA_3954|Chr3:88346445_88349444_+	0.882740841	0.019818
XM_011248394.1	circRNA_0611|Chr10:75274017_75279961_+	0.88272315	0.019824
XM_006498287.3	circRNA_0845|Chr11:29470315_29477458_+	0.882699116	0.019832
NM_026814.3	circRNA_0962|Chr11:67194348_67220998_+	0.882633527	0.019854
NM_007817.2	circRNA_6446|ChrX:42217416_42250037_+	0.882627029	0.019856
XM_006496593.3	circRNA_3276|Chr19:60563162_60564294_-	0.882588955	0.019869
NM_144539.5	circRNA_3239|Chr19:45635735_45640521_-	0.882569195	0.019875
XM_011248201.2	circRNA_3954|Chr3:88346445_88349444_+	0.88240646	0.019929
NM_009605.4	circRNA_3702|Chr2:143830084_143832057_-	0.882395047	0.019933
XM_006511102.2	circRNA_6140|Chr9:61935380_61937535_-	0.882337722	0.019952
NM_008623.5	circRNA_2951|Chr18:39120119_39150118_+	0.882213447	0.019993
NM_019759.2	circRNA_3239|Chr19:45635735_45640521_-	0.882201472	0.019997
XM_006504940.3	circRNA_3702|Chr2:143830084_143832057_-	0.882178298	0.020005
NM_001327998.1	circRNA_3239|Chr19:45635735_45640521_-	0.882149265	0.020015
NM_011957.2	circRNA_0845|Chr11:29470315_29477458_+	0.882133312	0.02002
NM_001159407.1	circRNA_3155|Chr19:25061108_25065459_+	0.882112445	0.020027
NM_001146217.1	circRNA_6446|ChrX:42217416_42250037_+	0.882062348	0.020044
XM_006514077.1	circRNA_5811|Chr8:71992832_71998295_-	0.882053287	0.020047
NM_008479.2	circRNA_0845|Chr11:29470315_29477458_+	0.882043052	0.02005
NM_013743.2	circRNA_3832|Chr2:169883526_169886459_+	0.88202319	0.020057
NM_026535.2	circRNA_5311|Chr6:145147636_145149357_+	0.882017593	0.020059
NM_011082.3	circRNA_0020|Chr1:10315205_10321709_+	0.882006264	0.020062
NM_001099314.1	circRNA_3954|Chr3:88346445_88349444_+	0.881945556	0.020083
XM_006517308.3	circRNA_6140|Chr9:61935380_61937535_-	0.881903099	0.020097
NM_001190449.1	circRNA_0157|Chr1:66801049_66802168_-	0.881776478	0.020139
NM_016809.6	circRNA_3239|Chr19:45635735_45640521_-	0.881723644	0.020157
NM_013560.2	circRNA_3832|Chr2:169883526_169886459_+	0.881696753	0.020166
XM_006506086.2	circRNA_0501|Chr10:25283893_25289730_-	0.881692815	0.020167
XM_006506086.2	circRNA_6109|Chr9:57056714_57057861_+	0.881692815	0.020167
NM_172907.3	circRNA_5639|Chr7:132771578_132779385_-	0.881669228	0.020175
NM_023143.3	circRNA_0611|Chr10:75274017_75279961_+	0.881569285	0.020208
NM_144799.2	circRNA_3832|Chr2:169883526_169886459_+	0.881557109	0.020212
XM_017313038.1	circRNA_0501|Chr10:25283893_25289730_-	0.881511761	0.020227
XM_017313038.1	circRNA_6109|Chr9:57056714_57057861_+	0.881511761	0.020227
NM_016982.2	circRNA_0611|Chr10:75274017_75279961_+	0.881453578	0.020247

NM_178753.4	circRNA_0157|Chr1:66801049_66802168_-	0.881439969	0.020251
NM_153178.4	circRNA_3954|Chr3:88346445_88349444_+	0.881347724	0.020282
NM_009199.2	circRNA_0962|Chr11:67194348_67220998_+	0.88131342	0.020294
NM_007555.4	circRNA_3239|Chr19:45635735_45640521_-	0.881255684	0.020313
NM_001313939.1	circRNA_5311|Chr6:145147636_145149357_+	0.881194502	0.020334
NM_011658.2	circRNA_4055|Chr3:126796838_126798280_+	0.881161058	0.020345
NM_029844.3	circRNA_0376|Chr1:172173943_172187460_+	0.88115782	0.020346
XM_006526069.2	circRNA_0961|Chr11:67192403_67254873_+	0.881150827	0.020348
NM_021475.2	circRNA_6446|ChrX:42217416_42250037_+	0.881111302	0.020362
NM_178692.3	circRNA_3239|Chr19:45635735_45640521_-	0.881100281	0.020365
NM_019759.2	circRNA_0157|Chr1:66801049_66802168_-	0.881092917	0.020368
NM_007549.2	circRNA_4055|Chr3:126796838_126798280_+	0.881077415	0.020373
NM_001190451.2	circRNA_3239|Chr19:45635735_45640521_-	0.881055089	0.02038
XM_006532962.2	circRNA_0845|Chr11:29470315_29477458_+	0.881040016	0.020385
XM_017317034.1	circRNA_4926|Chr5:143080224_143081077_-	0.880994826	0.020401
NM_009020.3	circRNA_3155|Chr19:25061108_25065459_+	0.880981561	0.020405
NM_011169.5	circRNA_4662|Chr5:65301933_65303208_-	0.880973362	0.020408
NM_026385.4	circRNA_3276|Chr19:60563162_60564294_-	0.880971591	0.020408
NM_021475.2	circRNA_3276|Chr19:60563162_60564294_-	0.880966846	0.02041
NM_001291483.1	circRNA_3702|Chr2:143830084_143832057_-	0.880962132	0.020412
NM_001317365.1	circRNA_2313|Chr15:93452117_93465245_+	0.880916803	0.020427
XM_006533569.3	circRNA_5074|Chr6:40685277_40747150_+	0.880879564	0.020439
NM_178753.4	circRNA_3239|Chr19:45635735_45640521_-	0.880836046	0.020454
XM_006518921.3	circRNA_4987|Chr6:4529068_4531010_+	0.880793465	0.020468
NM_001190451.2	circRNA_0020|Chr1:10315205_10321709_+	0.880788434	0.02047
XM_011248983.2	circRNA_3276|Chr19:60563162_60564294_-	0.880738844	0.020487
NM_008185.3	circRNA_5311|Chr6:145147636_145149357_+	0.880732662	0.020489
XM_011250776.2	circRNA_4055|Chr3:126796838_126798280_+	0.880705874	0.020498
NM_148932.2	circRNA_0537|Chr10:43393871_43395692_+	0.880641134	0.02052
XM_006526449.3	circRNA_3425|Chr2:37624173_37627526_-	0.880627861	0.020524
XM_006526449.3	circRNA_3607|Chr2:119057281_119064097_+	0.880627861	0.020524
XM_006520437.2	circRNA_6140|Chr9:61935380_61937535_-	0.880610262	0.02053
NM_009645.2	circRNA_5639|Chr7:132771578_132779385_-	0.880583936	0.020539
NM_178676.4	circRNA_0845|Chr11:29470315_29477458_+	0.880576471	0.020541
XM_006505388.2	circRNA_3276|Chr19:60563162_60564294_-	0.880515243	0.020562
NM_001252563.1	circRNA_0611|Chr10:75274017_75279961_+	0.880416981	0.020595
NM_008185.3	circRNA_0157|Chr1:66801049_66802168_-	0.880394557	0.020603
XM_006514077.1	circRNA_0537|Chr10:43393871_43395692_+	0.880327199	0.020625
NM_009605.4	circRNA_0376|Chr1:172173943_172187460_+	0.880315275	0.020629
NM_007763.3	circRNA_5311|Chr6:145147636_145149357_+	0.880313236	0.02063
NM_001290822.1	circRNA_6446|ChrX:42217416_42250037_+	0.880269456	0.020645
XM_006530585.3	circRNA_0537|Chr10:43393871_43395692_+	0.880258892	0.020648
XM_006506145.1	circRNA_0537|Chr10:43393871_43395692_+	0.880238588	0.020655
XM_017315376.1	circRNA_2951|Chr18:39120119_39150118_+	0.880229544	0.020658
NM_001190325.1	circRNA_4055|Chr3:126796838_126798280_+	0.880159082	0.020682
NM_177743.5	circRNA_3155|Chr19:25061108_25065459_+	0.880043957	0.020721
NM_144936.1	circRNA_0376|Chr1:172173943_172187460_+	0.879999355	0.020736
XM_006521428.3	circRNA_6140|Chr9:61935380_61937535_-	0.879937849	0.020757
XM_001481172.6	circRNA_0020|Chr1:10315205_10321709_+	0.879918178	0.020764
NM_010133.2	circRNA_0962|Chr11:67194348_67220998_+	0.879896957	0.020771
NM_001029877.3	circRNA_0961|Chr11:67192403_67254873_+	0.879836089	0.020792
XM_017322026.1	circRNA_0501|Chr10:25283893_25289730_-	0.879811387	0.0208
XM_017322026.1	circRNA_6109|Chr9:57056714_57057861_+	0.879811387	0.0208
NM_001310705.1	circRNA_0501|Chr10:25283893_25289730_-	0.879807218	0.020801
NM_001310705.1	circRNA_6109|Chr9:57056714_57057861_+	0.879807218	0.020801
XM_006529727.2	circRNA_3954|Chr3:88346445_88349444_+	0.879806375	0.020802
NM_001291483.1	circRNA_3239|Chr19:45635735_45640521_-	0.87966677	0.020849
NM_015814.2	circRNA_5311|Chr6:145147636_145149357_+	0.87963792	0.020859
NM_010174.1	circRNA_0962|Chr11:67194348_67220998_+	0.879635916	0.020859

XM_017315376.1	circRNA_6446|ChrX:42217416_42250037_+	0.87955916	0.020885
NM_016697.3	circRNA_4728|Chr5:97027831_97045167_+	0.879558327	0.020886
NM_008185.3	circRNA_5639|Chr7:132771578_132779385_-	0.879551098	0.020888
XM_017317978.1	circRNA_6140|Chr9:61935380_61937535_-	0.879516527	0.0209
NM_138955.3	circRNA_5074|Chr6:40685277_40747150_+	0.879511839	0.020902
NM_001290469.1	circRNA_0537|Chr10:43393871_43395692_+	0.879485658	0.02091
NM_008204.2	circRNA_2951|Chr18:39120119_39150118_+	0.879470701	0.020915
NM_009020.3	circRNA_4987|Chr6:4529068_4531010_+	0.87927415	0.020982
NM_001190449.1	circRNA_6140|Chr9:61935380_61937535_-	0.879271809	0.020983
XM_006499466.3	circRNA_0537|Chr10:43393871_43395692_+	0.879213317	0.021003
XM_006541047.3	circRNA_1632|Chr13:42055400_42055542_+	0.879136548	0.021029
NM_009518.2	circRNA_0501|Chr10:25283893_25289730_-	0.879089708	0.021045
NM_009518.2	circRNA_6109|Chr9:57056714_57057861_+	0.879089708	0.021045
NM_011010.2	circRNA_0961|Chr11:67192403_67254873_+	0.879003603	0.021074
XM_006529727.2	circRNA_4926|Chr5:143080224_143081077_-	0.879002988	0.021075
NM_134072.1	circRNA_4728|Chr5:97027831_97045167_+	0.878968786	0.021086
XM_006507725.2	circRNA_0537|Chr10:43393871_43395692_+	0.878891808	0.021113
XM_006511102.2	circRNA_0157|Chr1:66801049_66802168_-	0.878833161	0.021133
NM_030206.4	circRNA_6140|Chr9:61935380_61937535_-	0.878827293	0.021135
NM_173427.2	circRNA_0501|Chr10:25283893_25289730_-	0.878816982	0.021138
NM_173427.2	circRNA_6109|Chr9:57056714_57057861_+	0.878816982	0.021138
XM_017312731.1	circRNA_3702|Chr2:143830084_143832057_-	0.878779895	0.021151
NM_001302257.1	circRNA_0157|Chr1:66801049_66802168_-	0.878686188	0.021183
XM_011246128.2	circRNA_3643|Chr2:122441559_122486090_+	0.878672551	0.021188
NM_032400.2	circRNA_0157|Chr1:66801049_66802168_-	0.878654162	0.021194
XM_006501237.3	circRNA_0157|Chr1:66801049_66802168_-	0.878595233	0.021214
NM_009608.4	circRNA_0962|Chr11:67194348_67220998_+	0.878588281	0.021216
NM_008760.4	circRNA_0020|Chr1:10315205_10321709_+	0.8785442	0.021231
XM_017314944.1	circRNA_3702|Chr2:143830084_143832057_-	0.87846238	0.021259
XM_017322026.1	circRNA_6446|ChrX:42217416_42250037_+	0.878431006	0.02127
NM_009514.4	circRNA_3702|Chr2:143830084_143832057_-	0.878428369	0.021271
NM_001159424.2	circRNA_3276|Chr19:60563162_60564294_-	0.878410253	0.021277
NM_023456.3	circRNA_2888|Chr18:21010677_21020999_+	0.878386732	0.021285
NM_001291483.1	circRNA_4728|Chr5:97027831_97045167_+	0.878328785	0.021305
NM_010140.3	circRNA_3276|Chr19:60563162_60564294_-	0.878270577	0.021325
NM_133643.4	circRNA_6446|ChrX:42217416_42250037_+	0.878191132	0.021352
XM_006528842.1	circRNA_0537|Chr10:43393871_43395692_+	0.878114376	0.021379
NM_001291930.1	circRNA_0157|Chr1:66801049_66802168_-	0.878100253	0.021384
XM_006529753.2	circRNA_0961|Chr11:67192403_67254873_+	0.878030649	0.021408
NM_021475.2	circRNA_2951|Chr18:39120119_39150118_+	0.878024614	0.02141
XM_006528842.1	circRNA_0961|Chr11:67192403_67254873_+	0.878012013	0.021414
NM_025758.4	circRNA_1399|Chr12:72783683_72786789_+	0.877972283	0.021428
XM_006540566.1	circRNA_3003|Chr18:67545615_67587859_-	0.877912389	0.021448
NM_001303431.1	circRNA_0913|Chr11:54005394_54014456_-	0.877895073	0.021454
XM_006520618.2	circRNA_3276|Chr19:60563162_60564294_-	0.877892153	0.021455
NM_028472.2	circRNA_0501|Chr10:25283893_25289730_-	0.877840469	0.021473
NM_028472.2	circRNA_6109|Chr9:57056714_57057861_+	0.877840469	0.021473
XM_006503665.3	circRNA_3155|Chr19:25061108_25065459_+	0.877828191	0.021477
XM_006506624.2	circRNA_3155|Chr19:25061108_25065459_+	0.877809895	0.021483
NM_001327998.1	circRNA_3702|Chr2:143830084_143832057_-	0.877804761	0.021485
NM_001162950.1	circRNA_0961|Chr11:67192403_67254873_+	0.877771677	0.021497
NM_008032.3	circRNA_0845|Chr11:29470315_29477458_+	0.877758655	0.021501
XM_006511221.3	circRNA_4926|Chr5:143080224_143081077_-	0.877755868	0.021502
NM_001290469.1	circRNA_3832|Chr2:169883526_169886459_+	0.877745088	0.021506
NM_001301354.1	circRNA_3276|Chr19:60563162_60564294_-	0.877636052	0.021543
NM_008940.3	circRNA_5639|Chr7:132771578_132779385_-	0.877523571	0.021582
NM_177743.5	circRNA_0501|Chr10:25283893_25289730_-	0.877519888	0.021583
NM_177743.5	circRNA_6109|Chr9:57056714_57057861_+	0.877519888	0.021583
XM_011248983.2	circRNA_3643|Chr2:122441559_122486090_+	0.877456985	0.021605

NM_001146217.1	circRNA_4662|Chr5:65301933_65303208_-	0.877422814	0.021617
XM_006505602.3	circRNA_3832|Chr2:169883526_169886459_+	0.877420007	0.021618
XM_006518921.3	circRNA_3643|Chr2:122441559_122486090_+	0.877410635	0.021621
NM_011169.5	circRNA_0157|Chr1:66801049_66802168_-	0.877367728	0.021636
NM_145210.2	circRNA_4487|Chr4:149156607_149161694_-	0.877328627	0.021649
NM_053080.3	circRNA_4987|Chr6:4529068_4531010_+	0.877275756	0.021668
NM_010090.2	circRNA_0157|Chr1:66801049_66802168_-	0.877216607	0.021688
XM_017317978.1	circRNA_0157|Chr1:66801049_66802168_-	0.877206303	0.021692
NM_001030305.2	circRNA_0501|Chr10:25283893_25289730_-	0.877186803	0.021698
NM_001030305.2	circRNA_6109|Chr9:57056714_57057861_+	0.877186803	0.021698
NM_001164593.1	circRNA_5311|Chr6:145147636_145149357_+	0.877142415	0.021714
XM_006532962.2	circRNA_5074|Chr6:40685277_40747150_+	0.877125074	0.02172
NM_031180.2	circRNA_4728|Chr5:97027831_97045167_+	0.877111286	0.021725
XM_017312497.1	circRNA_5311|Chr6:145147636_145149357_+	0.877003617	0.021762
XM_006498086.3	circRNA_0157|Chr1:66801049_66802168_-	0.876989683	0.021767
NM_205810.4	circRNA_3276|Chr19:60563162_60564294_-	0.876985993	0.021768
NM_001033286.2	circRNA_0537|Chr10:43393871_43395692_+	0.876984603	0.021768
NM_178676.4	circRNA_4635|Chr5:43758222_43773659_-	0.876963726	0.021776
NM_001320077.1	circRNA_3276|Chr19:60563162_60564294_-	0.876884201	0.021803
XM_006522398.3	circRNA_0501|Chr10:25283893_25289730_-	0.876836772	0.02182
XM_006522398.3	circRNA_6109|Chr9:57056714_57057861_+	0.876836772	0.02182
XM_006521554.3	circRNA_3003|Chr18:67545615_67587859_-	0.876796942	0.021833
NM_009127.4	circRNA_2888|Chr18:21010677_21020999_+	0.87678222	0.021839
XM_011248919.2	circRNA_3832|Chr2:169883526_169886459_+	0.876782049	0.021839
NM_001097617.1	circRNA_0376|Chr1:172173943_172187460_+	0.876758194	0.021847
XM_006506086.2	circRNA_2951|Chr18:39120119_39150118_+	0.876702222	0.021866
NM_008804.4	circRNA_3239|Chr19:45635735_45640521_-	0.876695127	0.021869
NM_009019.2	circRNA_0157|Chr1:66801049_66802168_-	0.876689065	0.021871
NM_001160262.1	circRNA_0962|Chr11:67194348_67220998_+	0.876661461	0.02188
XM_006514077.1	circRNA_3832|Chr2:169883526_169886459_+	0.876625695	0.021893
NM_010279.3	circRNA_3276|Chr19:60563162_60564294_-	0.876625037	0.021893
NM_008423.2	circRNA_2313|Chr15:93452117_93465245_+	0.876571588	0.021912
NM_001310648.1	circRNA_5639|Chr7:132771578_132779385_-	0.876474398	0.021945
NM_001113515.2	circRNA_0962|Chr11:67194348_67220998_+	0.876471656	0.021946
XM_006520023.2	circRNA_3702|Chr2:143830084_143832057_-	0.876419858	0.021964
XM_006509531.3	circRNA_0157|Chr1:66801049_66802168_-	0.876408417	0.021968
NM_016982.2	circRNA_4728|Chr5:97027831_97045167_+	0.876405111	0.02197
NM_027416.3	circRNA_3155|Chr19:25061108_25065459_+	0.876360498	0.021985
XM_006505757.3	circRNA_0611|Chr10:75274017_75279961_+	0.876339359	0.021992
NM_009379.3	circRNA_0961|Chr11:67192403_67254873_+	0.876307258	0.022004
XM_011245824.2	circRNA_6390|Chr9:121727316_121731565_+	0.876285935	0.022011
XM_006532120.2	circRNA_4055|Chr3:126796838_126798280_+	0.876274573	0.022015
XM_006510297.3	circRNA_0020|Chr1:10315205_10321709_+	0.876272959	0.022016
XM_006504999.1	circRNA_2951|Chr18:39120119_39150118_+	0.87626025	0.02202
XM_011243586.2	circRNA_0376|Chr1:172173943_172187460_+	0.876251586	0.022023
NM_020001.2	circRNA_3702|Chr2:143830084_143832057_-	0.876221625	0.022033
NM_007817.2	circRNA_3276|Chr19:60563162_60564294_-	0.876202296	0.02204
XM_011250978.2	circRNA_0537|Chr10:43393871_43395692_+	0.876186828	0.022046
XM_017312497.1	circRNA_0913|Chr11:54005394_54014456_-	0.876151621	0.022058
NM_001146217.1	circRNA_0157|Chr1:66801049_66802168_-	0.876149281	0.022059
XM_011240476.2	circRNA_3425|Chr2:37624173_37627526_-	0.876135198	0.022064
XM_011240476.2	circRNA_3607|Chr2:119057281_119064097_+	0.876135198	0.022064
NM_024283.3	circRNA_3702|Chr2:143830084_143832057_-	0.87611481	0.022071
NM_008012.1	circRNA_3239|Chr19:45635735_45640521_-	0.876063162	0.022089
XM_006529382.3	circRNA_0962|Chr11:67194348_67220998_+	0.876037695	0.022098
NM_009381.3	circRNA_0611|Chr10:75274017_75279961_+	0.875990451	0.022114
NM_015814.2	circRNA_3239|Chr19:45635735_45640521_-	0.875796775	0.022182
NM_007641.5	circRNA_0157|Chr1:66801049_66802168_-	0.875791214	0.022184
XM_006508885.1	circRNA_5311|Chr6:145147636_145149357_+	0.875733365	0.022204

NM_178676.4	circRNA_1042|Chr11:80385854_80403408_+	0.875655129	0.022231
NM_025865.2	circRNA_1980|Chr14:50951404_50963869_+	0.875638258	0.022237
NM_008012.1	circRNA_3155|Chr19:25061108_25065459_+	0.875628615	0.02224
NM_030206.4	circRNA_0611|Chr10:75274017_75279961_+	0.875586769	0.022255
NM_008032.3	circRNA_5074|Chr6:40685277_40747150_+	0.87552041	0.022278
XM_017315376.1	circRNA_2888|Chr18:21010677_21020999_+	0.875352181	0.022337
XM_006505757.3	circRNA_0020|Chr1:10315205_10321709_+	0.875254797	0.022371
NM_026385.4	circRNA_0157|Chr1:66801049_66802168_-	0.875252495	0.022372
NM_207231.1	circRNA_6446|ChrX:42217416_42250037_+	0.875217137	0.022385
NM_026979.5	circRNA_1632|Chr13:42055400_42055542_+	0.875214787	0.022385
NM_023048.5	circRNA_0962|Chr11:67194348_67220998_+	0.875205167	0.022389
NM_001190325.1	circRNA_0157|Chr1:66801049_66802168_-	0.875195907	0.022392
XM_017315841.1	circRNA_3832|Chr2:169883526_169886459_+	0.875153466	0.022407
NM_001320077.1	circRNA_3155|Chr19:25061108_25065459_+	0.875133858	0.022414
NM_001110009.2	circRNA_6446|ChrX:42217416_42250037_+	0.87509629	0.022427
NM_009645.2	circRNA_0020|Chr1:10315205_10321709_+	0.875020343	0.022454
NM_009223.3	circRNA_0157|Chr1:66801049_66802168_-	0.874911158	0.022492
XM_006506808.3	circRNA_0961|Chr11:67192403_67254873_+	0.874864396	0.022509
NM_009518.2	circRNA_3702|Chr2:143830084_143832057_-	0.874762552	0.022544
NM_001159424.2	circRNA_2951|Chr18:39120119_39150118_+	0.87474545	0.022551
NM_001204959.1	circRNA_0501|Chr10:25283893_25289730_-	0.874733244	0.022555
NM_001204959.1	circRNA_6109|Chr9:57056714_57057861_+	0.874733244	0.022555
NM_009127.4	circRNA_6446|ChrX:42217416_42250037_+	0.874645436	0.022586
XM_006510077.3	circRNA_4662|Chr5:65301933_65303208_-	0.874604782	0.0226
XM_006505388.2	circRNA_5367|Chr7:28990988_28991325_+	0.874579885	0.022609
NM_021475.2	circRNA_5311|Chr6:145147636_145149357_+	0.874576266	0.02261
NM_010055.3	circRNA_4635|Chr5:43758222_43773659_-	0.874568864	0.022613
XM_006498086.3	circRNA_6140|Chr9:61935380_61937535_-	0.874507035	0.022635
NM_134072.1	circRNA_0611|Chr10:75274017_75279961_+	0.874447648	0.022656
NM_016982.2	circRNA_4055|Chr3:126796838_126798280_+	0.874359419	0.022687
NM_146063.1	circRNA_4662|Chr5:65301933_65303208_-	0.874265396	0.02272
NM_001291145.1	circRNA_3155|Chr19:25061108_25065459_+	0.874220691	0.022736
XM_011250776.2	circRNA_2951|Chr18:39120119_39150118_+	0.874173958	0.022752
NM_011704.3	circRNA_4728|Chr5:97027831_97045167_+	0.874164391	0.022756
XM_006506145.1	circRNA_6390|Chr9:121727316_121731565_+	0.874073714	0.022788
NM_010181.2	circRNA_3954|Chr3:88346445_88349444_+	0.874069901	0.022789
NM_173427.2	circRNA_2951|Chr18:39120119_39150118_+	0.874061941	0.022792
NM_146241.2	circRNA_1632|Chr13:42055400_42055542_+	0.874053426	0.022795
XM_006512076.3	circRNA_3954|Chr3:88346445_88349444_+	0.874009784	0.02281
NM_138955.3	circRNA_3954|Chr3:88346445_88349444_+	0.873955543	0.02283
NM_001190449.1	circRNA_0376|Chr1:172173943_172187460_+	0.873955096	0.02283
NM_144945.3	circRNA_2313|Chr15:93452117_93465245_+	0.873909056	0.022846
NM_178715.3	circRNA_3702|Chr2:143830084_143832057_-	0.873887095	0.022854
NM_007588.2	circRNA_3832|Chr2:169883526_169886459_+	0.873884377	0.022855
NM_010450.3	circRNA_5311|Chr6:145147636_145149357_+	0.873875301	0.022858
NM_001317365.1	circRNA_4487|Chr4:149156607_149161694_-	0.87386473	0.022862
XM_006524328.3	circRNA_0961|Chr11:67192403_67254873_+	0.873762604	0.022898
XM_006528026.2	circRNA_0961|Chr11:67192403_67254873_+	0.87375108	0.022902
NM_199029.2	circRNA_3832|Chr2:169883526_169886459_+	0.873727964	0.02291
XM_006515980.3	circRNA_4055|Chr3:126796838_126798280_+	0.873721574	0.022913
XM_006514153.2	circRNA_6390|Chr9:121727316_121731565_+	0.873676423	0.022929
NM_028973.2	circRNA_4662|Chr5:65301933_65303208_-	0.873614471	0.022951
NM_008966.3	circRNA_4662|Chr5:65301933_65303208_-	0.873589417	0.022959
XM_006521428.3	circRNA_0501|Chr10:25283893_25289730_-	0.873574123	0.022965
XM_006521428.3	circRNA_6109|Chr9:57056714_57057861_+	0.873574123	0.022965
NM_015744.4	circRNA_2951|Chr18:39120119_39150118_+	0.873552577	0.022973
NM_009381.3	circRNA_0376|Chr1:172173943_172187460_+	0.873536079	0.022978
NM_001190449.1	circRNA_2951|Chr18:39120119_39150118_+	0.873535951	0.022978
NM_016982.2	circRNA_0501|Chr10:25283893_25289730_-	0.873522275	0.022983

NM_016982.2	circRNA_6109|Chr9:57056714_57057861_+	0.873522275	0.022983
NM_199304.1	circRNA_5811|Chr8:71992832_71998295_-	0.873487738	0.022996
XM_017314944.1	circRNA_6140|Chr9:61935380_61937535_-	0.873481098	0.022998
NM_199029.2	circRNA_3954|Chr3:88346445_88349444_+	0.873479212	0.022999
NM_152915.1	circRNA_6140|Chr9:61935380_61937535_-	0.873443803	0.023011
XM_011248983.2	circRNA_3702|Chr2:143830084_143832057_-	0.873403082	0.023026
NM_028973.2	circRNA_3239|Chr19:45635735_45640521_-	0.873385766	0.023032
XM_006527480.3	circRNA_0537|Chr10:43393871_43395692_+	0.873306359	0.02306
XM_006529456.3	circRNA_6140|Chr9:61935380_61937535_-	0.873293243	0.023065
XM_017312731.1	circRNA_0501|Chr10:25283893_25289730_-	0.873290632	0.023066
XM_017312731.1	circRNA_6109|Chr9:57056714_57057861_+	0.873290632	0.023066
NM_001162950.1	circRNA_5074|Chr6:40685277_40747150_+	0.873215489	0.023092
NM_027455.3	circRNA_3155|Chr19:25061108_25065459_+	0.87318723	0.023103
NM_021475.2	circRNA_4055|Chr3:126796838_126798280_+	0.873161167	0.023112
NM_028804.1	circRNA_2951|Chr18:39120119_39150118_+	0.873083673	0.023139
NM_001167908.1	circRNA_6390|Chr9:121727316_121731565_+	0.87308223	0.02314
XM_006506340.3	circRNA_6390|Chr9:121727316_121731565_+	0.873063066	0.023147
XM_006530238.3	circRNA_4926|Chr5:143080224_143081077_-	0.872995007	0.023171
XM_006517308.3	circRNA_5311|Chr6:145147636_145149357_+	0.872883537	0.023211
NM_007482.3	circRNA_4987|Chr6:4529068_4531010_+	0.872829743	0.02323
XM_017312731.1	circRNA_5639|Chr7:132771578_132779385_-	0.872691513	0.02328
NM_001317365.1	circRNA_3425|Chr2:37624173_37627526_-	0.872682074	0.023283
NM_001317365.1	circRNA_3607|Chr2:119057281_119064097_+	0.872682074	0.023283
NM_001159424.2	circRNA_2888|Chr18:21010677_21020999_+	0.87268103	0.023283
XM_006503080.3	circRNA_0845|Chr11:29470315_29477458_+	0.872661593	0.02329
XM_006515637.2	circRNA_0157|Chr1:66801049_66802168_-	0.872660951	0.02329
NM_001291930.1	circRNA_3155|Chr19:25061108_25065459_+	0.872649738	0.023294
NM_010279.3	circRNA_6140|Chr9:61935380_61937535_-	0.872647423	0.023295
NM_007792.4	circRNA_0611|Chr10:75274017_75279961_+	0.87263773	0.023299
NM_009393.2	circRNA_3832|Chr2:169883526_169886459_+	0.872628171	0.023302
XM_006533539.3	circRNA_0501|Chr10:25283893_25289730_-	0.87262338	0.023304
XM_006533539.3	circRNA_6109|Chr9:57056714_57057861_+	0.87262338	0.023304
XM_017316056.1	circRNA_0501|Chr10:25283893_25289730_-	0.872618435	0.023306
XM_017316056.1	circRNA_6109|Chr9:57056714_57057861_+	0.872618435	0.023306
NM_001190451.2	circRNA_0157|Chr1:66801049_66802168_-	0.872590785	0.023316
XM_017315734.1	circRNA_2313|Chr15:93452117_93465245_+	0.872569242	0.023323
XM_006525028.2	circRNA_0376|Chr1:172173943_172187460_+	0.872538303	0.023334
NM_010825.3	circRNA_3832|Chr2:169883526_169886459_+	0.872481673	0.023355
NM_011082.3	circRNA_5367|Chr7:28990988_28991325_+	0.872466613	0.02336
NM_029844.3	circRNA_0611|Chr10:75274017_75279961_+	0.872422434	0.023376
NM_145226.2	circRNA_0537|Chr10:43393871_43395692_+	0.872410206	0.02338
NM_016933.3	circRNA_6140|Chr9:61935380_61937535_-	0.87239631	0.023385
NM_145226.2	circRNA_3425|Chr2:37624173_37627526_-	0.872332139	0.023408
NM_145226.2	circRNA_3607|Chr2:119057281_119064097_+	0.872332139	0.023408
XM_017312731.1	circRNA_2951|Chr18:39120119_39150118_+	0.87232093	0.023412
XM_006541297.3	circRNA_3832|Chr2:169883526_169886459_+	0.872290425	0.023423
XM_006524777.3	circRNA_3702|Chr2:143830084_143832057_-	0.872230655	0.023445
NM_029823.2	circRNA_6446|ChrX:42217416_42250037_+	0.872230294	0.023445
NM_013560.2	circRNA_0537|Chr10:43393871_43395692_+	0.872208631	0.023452
XM_006520186.2	circRNA_0157|Chr1:66801049_66802168_-	0.872203989	0.023454
XM_006521796.1	circRNA_2951|Chr18:39120119_39150118_+	0.872175586	0.023464
NM_144544.2	circRNA_4055|Chr3:126796838_126798280_+	0.872040245	0.023513
NM_016933.3	circRNA_4055|Chr3:126796838_126798280_+	0.871938821	0.023549
NM_030206.4	circRNA_3276|Chr19:60563162_60564294_-	0.871930168	0.023553
NM_145141.2	circRNA_0020|Chr1:10315205_10321709_+	0.871870409	0.023574
NM_175022.2	circRNA_0961|Chr11:67192403_67254873_+	0.871870191	0.023574
NM_001305585.1	circRNA_0376|Chr1:172173943_172187460_+	0.87184264	0.023584
NM_199304.1	circRNA_0537|Chr10:43393871_43395692_+	0.871769192	0.02361
XM_006520253.3	circRNA_0537|Chr10:43393871_43395692_+	0.871748902	0.023618

NM_001198955.1	circRNA_5074|Chr6:40685277_40747150_+	0.871709102	0.023632
XM_006510297.3	circRNA_2888|Chr18:21010677_21020999_+	0.871707868	0.023633
NM_029844.3	circRNA_4728|Chr5:97027831_97045167_+	0.871704636	0.023634
NM_027455.3	circRNA_0913|Chr11:54005394_54014456_-	0.871689527	0.023639
XM_006506340.3	circRNA_0537|Chr10:43393871_43395692_+	0.871649861	0.023653
NM_001310705.1	circRNA_4055|Chr3:126796838_126798280_+	0.871639305	0.023657
XM_017312731.1	circRNA_4055|Chr3:126796838_126798280_+	0.871613854	0.023666
NM_024283.3	circRNA_0157|Chr1:66801049_66802168_-	0.871598797	0.023672
XM_006506152.3	circRNA_4635|Chr5:43758222_43773659_-	0.871508474	0.023704
NM_009020.3	circRNA_3239|Chr19:45635735_45640521_-	0.871456504	0.023723
NM_001302257.1	circRNA_2951|Chr18:39120119_39150118_+	0.871448395	0.023726
XM_006538830.1	circRNA_5811|Chr8:71992832_71998295_-	0.871425348	0.023734
XM_006529456.3	circRNA_3643|Chr2:122441559_122486090_+	0.871405929	0.023741
NM_153801.3	circRNA_6390|Chr9:121727316_121731565_+	0.871295134	0.023781
XM_011248919.2	circRNA_0537|Chr10:43393871_43395692_+	0.871280802	0.023787
NM_010825.3	circRNA_0962|Chr11:67194348_67220998_+	0.871204773	0.023814
XM_006521428.3	circRNA_2888|Chr18:21010677_21020999_+	0.871147854	0.023835
NM_146125.2	circRNA_0501|Chr10:25283893_25289730_-	0.871046928	0.023871
NM_146125.2	circRNA_6109|Chr9:57056714_57057861_+	0.871046928	0.023871
NM_020025.4	circRNA_0020|Chr1:10315205_10321709_+	0.871031864	0.023877
NM_009605.4	circRNA_5639|Chr7:132771578_132779385_-	0.871000874	0.023888
NM_001081224.2	circRNA_2888|Chr18:21010677_21020999_+	0.870950615	0.023906
NM_001317365.1	circRNA_1399|Chr12:72783683_72786789_+	0.870914984	0.023919
XM_011240476.2	circRNA_5811|Chr8:71992832_71998295_-	0.870913893	0.023919
NM_146125.2	circRNA_5639|Chr7:132771578_132779385_-	0.870838694	0.023947
NM_133643.4	circRNA_3239|Chr19:45635735_45640521_-	0.870777774	0.023969
NM_133643.4	circRNA_6140|Chr9:61935380_61937535_-	0.870726298	0.023987
NM_011044.2	circRNA_4055|Chr3:126796838_126798280_+	0.870706198	0.023995
NM_009381.3	circRNA_4055|Chr3:126796838_126798280_+	0.870683692	0.024003
XM_006505388.2	circRNA_3702|Chr2:143830084_143832057_-	0.870671401	0.024007
XM_006501237.3	circRNA_3702|Chr2:143830084_143832057_-	0.870661914	0.024011
NM_011724.3	circRNA_0537|Chr10:43393871_43395692_+	0.870654319	0.024013
NM_001270475.1	circRNA_0961|Chr11:67192403_67254873_+	0.870651654	0.024014
NM_023670.3	circRNA_4055|Chr3:126796838_126798280_+	0.87063356	0.024021
NM_009930.2	circRNA_0376|Chr1:172173943_172187460_+	0.870629324	0.024023
NM_001077403.1	circRNA_6390|Chr9:121727316_121731565_+	0.870512972	0.024065
XM_006533452.3	circRNA_6446|ChrX:42217416_42250037_+	0.870509926	0.024066
XM_011243266.1	circRNA_4926|Chr5:143080224_143081077_-	0.870505146	0.024068
NM_001146217.1	circRNA_3155|Chr19:25061108_25065459_+	0.870495378	0.024071
NM_178782.4	circRNA_3954|Chr3:88346445_88349444_+	0.870472208	0.02408
NM_007529.2	circRNA_0157|Chr1:66801049_66802168_-	0.870461351	0.024084
NM_015744.4	circRNA_4662|Chr5:65301933_65303208_-	0.870427475	0.024096
XM_006521428.3	circRNA_2951|Chr18:39120119_39150118_+	0.870408994	0.024103
NM_009349.3	circRNA_0501|Chr10:25283893_25289730_-	0.870387596	0.02411
NM_009349.3	circRNA_6109|Chr9:57056714_57057861_+	0.870387596	0.02411
NM_028804.1	circRNA_0501|Chr10:25283893_25289730_-	0.870378708	0.024114
NM_028804.1	circRNA_6109|Chr9:57056714_57057861_+	0.870378708	0.024114
XM_017317034.1	circRNA_3954|Chr3:88346445_88349444_+	0.870376161	0.024115
XM_011246128.2	circRNA_5367|Chr7:28990988_28991325_+	0.870364658	0.024119
XM_011244235.2	circRNA_3702|Chr2:143830084_143832057_-	0.870349447	0.024124
NM_001320077.1	circRNA_3239|Chr19:45635735_45640521_-	0.870338463	0.024128
XM_017317946.1	circRNA_6390|Chr9:121727316_121731565_+	0.870302579	0.024141
NM_001190449.1	circRNA_0501|Chr10:25283893_25289730_-	0.870226872	0.024169
NM_001190449.1	circRNA_6109|Chr9:57056714_57057861_+	0.870226872	0.024169
NM_001290822.1	circRNA_0611|Chr10:75274017_75279961_+	0.870226527	0.024169
NM_009824.2	circRNA_3832|Chr2:169883526_169886459_+	0.870216017	0.024173
NM_001286743.1	circRNA_0845|Chr11:29470315_29477458_+	0.87020662	0.024176
NM_025758.4	circRNA_3425|Chr2:37624173_37627526_-	0.870202606	0.024178
NM_025758.4	circRNA_3607|Chr2:119057281_119064097_+	0.870202606	0.024178

NM_001204201.1	circRNA_3702|Chr2:143830084_143832057_-	0.870194302	0.024181
NM_001099314.1	circRNA_1399|Chr12:72783683_72786789_+	0.870172719	0.024189
NM_028472.2	circRNA_3239|Chr19:45635735_45640521_-	0.870146713	0.024198
NM_011658.2	circRNA_0157|Chr1:66801049_66802168_-	0.870126106	0.024206
XM_006510077.3	circRNA_2951|Chr18:39120119_39150118_+	0.870038065	0.024238
XM_006508867.2	circRNA_6140|Chr9:61935380_61937535_-	0.870005517	0.024249
NM_001033167.3	circRNA_4926|Chr5:143080224_143081077_-	0.869963522	0.024265
NM_009019.2	circRNA_3276|Chr19:60563162_60564294_-	0.869944542	0.024272
XM_017318264.1	circRNA_3832|Chr2:169883526_169886459_+	0.869863398	0.024301
NM_001001178.1	circRNA_6140|Chr9:61935380_61937535_-	0.869797881	0.024325
NM_009349.3	circRNA_5367|Chr7:28990988_28991325_+	0.869783053	0.024331
XM_017321602.1	circRNA_3276|Chr19:60563162_60564294_-	0.869698958	0.024361
NM_145226.2	circRNA_1399|Chr12:72783683_72786789_+	0.869666544	0.024373
NM_175260.2	circRNA_3954|Chr3:88346445_88349444_+	0.869655715	0.024377
XM_006510077.3	circRNA_0501|Chr10:25283893_25289730_-	0.869532096	0.024422
XM_006510077.3	circRNA_6109|Chr9:57056714_57057861_+	0.869532096	0.024422
NM_001081224.2	circRNA_4728|Chr5:97027831_97045167_+	0.869528041	0.024424
NM_001099314.1	circRNA_1042|Chr11:80385854_80403408_+	0.869436137	0.024458
NM_177743.5	circRNA_4728|Chr5:97027831_97045167_+	0.869416866	0.024465
NM_009930.2	circRNA_4055|Chr3:126796838_126798280_+	0.869356885	0.024487
NM_144539.5	circRNA_5311|Chr6:145147636_145149357_+	0.869346295	0.02449
NM_029274.2	circRNA_3832|Chr2:169883526_169886459_+	0.869288756	0.024512
XM_006515980.3	circRNA_0020|Chr1:10315205_10321709_+	0.869279063	0.024515
XM_006508867.2	circRNA_6446|ChrX:42217416_42250037_+	0.869232952	0.024532
XM_017313038.1	circRNA_2888|Chr18:21010677_21020999_+	0.86921004	0.02454
NM_016982.2	circRNA_5639|Chr7:132771578_132779385_-	0.8691617	0.024558
NM_133500.2	circRNA_6390|Chr9:121727316_121731565_+	0.869156811	0.02456
NM_144539.5	circRNA_3155|Chr19:25061108_25065459_+	0.869150724	0.024562
XM_011240476.2	circRNA_1399|Chr12:72783683_72786789_+	0.869127676	0.024571
XM_006540134.3	circRNA_0962|Chr11:67194348_67220998_+	0.869034246	0.024605
NM_001198955.1	circRNA_0845|Chr11:29470315_29477458_+	0.868997602	0.024618
NM_001327998.1	circRNA_0611|Chr10:75274017_75279961_+	0.868934069	0.024642
XM_006527452.3	circRNA_6140|Chr9:61935380_61937535_-	0.868933245	0.024642
XM_011243586.2	circRNA_4728|Chr5:97027831_97045167_+	0.868914755	0.024649
NM_146241.2	circRNA_5367|Chr7:28990988_28991325_+	0.868901423	0.024654
XM_006524407.3	circRNA_3832|Chr2:169883526_169886459_+	0.868825683	0.024682
NM_001099314.1	circRNA_3425|Chr2:37624173_37627526_-	0.868733226	0.024716
NM_001099314.1	circRNA_3607|Chr2:119057281_119064097_+	0.868733226	0.024716
NM_008479.2	circRNA_1399|Chr12:72783683_72786789_+	0.868727331	0.024718
NM_175563.5	circRNA_6390|Chr9:121727316_121731565_+	0.868724712	0.024719
NM_001030305.2	circRNA_2888|Chr18:21010677_21020999_+	0.868680602	0.024735
XM_006521428.3	circRNA_4987|Chr6:4529068_4531010_+	0.868654474	0.024745
XM_006530131.1	circRNA_0845|Chr11:29470315_29477458_+	0.868616481	0.024758
NM_145141.2	circRNA_2951|Chr18:39120119_39150118_+	0.868437864	0.024824
NM_009675.2	circRNA_6140|Chr9:61935380_61937535_-	0.868435361	0.024825
XM_006515980.3	circRNA_6446|ChrX:42217416_42250037_+	0.868425086	0.024829
NM_001302257.1	circRNA_3276|Chr19:60563162_60564294_-	0.868370802	0.024849
NM_008277.2	circRNA_0501|Chr10:25283893_25289730_-	0.86837064	0.024849
NM_008277.2	circRNA_6109|Chr9:57056714_57057861_+	0.86837064	0.024849
NM_009605.4	circRNA_0157|Chr1:66801049_66802168_-	0.868359215	0.024853
NM_001001178.1	circRNA_5639|Chr7:132771578_132779385_-	0.86834923	0.024857
XM_006515980.3	circRNA_0157|Chr1:66801049_66802168_-	0.868346602	0.024858
NM_007655.3	circRNA_0157|Chr1:66801049_66802168_-	0.868323069	0.024867
XM_006527008.2	circRNA_0537|Chr10:43393871_43395692_+	0.868269982	0.024886
XM_006496884.3	circRNA_3276|Chr19:60563162_60564294_-	0.868249546	0.024894
NM_001310636.1	circRNA_0501|Chr10:25283893_25289730_-	0.868217816	0.024906
NM_001310636.1	circRNA_6109|Chr9:57056714_57057861_+	0.868217816	0.024906
NM_009135.2	circRNA_3702|Chr2:143830084_143832057_-	0.868212009	0.024908
NM_001310705.1	circRNA_0376|Chr1:172173943_172187460_+	0.868112772	0.024944

NM_019397.3	circRNA_0157|Chr1:66801049_66802168_-	0.868110331	0.024945
XM_011248602.2	circRNA_0845|Chr11:29470315_29477458_+	0.868109088	0.024946
NM_016659.3	circRNA_0157|Chr1:66801049_66802168_-	0.868056276	0.024965
NM_008479.2	circRNA_3425|Chr2:37624173_37627526_-	0.868021741	0.024978
NM_008479.2	circRNA_3607|Chr2:119057281_119064097_+	0.868021741	0.024978
NM_010714.3	circRNA_3276|Chr19:60563162_60564294_-	0.868009726	0.024982
NM_009345.2	circRNA_5311|Chr6:145147636_145149357_+	0.86797138	0.024997
NM_010779.2	circRNA_2951|Chr18:39120119_39150118_+	0.867926008	0.025013
XM_017321602.1	circRNA_0501|Chr10:25283893_25289730_-	0.867870576	0.025034
XM_017321602.1	circRNA_6109|Chr9:57056714_57057861_+	0.867870576	0.025034
NM_021475.2	circRNA_2888|Chr18:21010677_21020999_+	0.867864882	0.025036
XM_006532024.3	circRNA_6140|Chr9:61935380_61937535_-	0.86780031	0.02506
XM_006503323.3	circRNA_3155|Chr19:25061108_25065459_+	0.867784706	0.025066
XM_006530238.3	circRNA_3832|Chr2:169883526_169886459_+	0.867743715	0.025081
XM_006509531.3	circRNA_3702|Chr2:143830084_143832057_-	0.867740547	0.025082
NM_025288.2	circRNA_3239|Chr19:45635735_45640521_-	0.867707188	0.025094
XM_006541047.3	circRNA_0501|Chr10:25283893_25289730_-	0.867672399	0.025107
XM_006541047.3	circRNA_6109|Chr9:57056714_57057861_+	0.867672399	0.025107
NM_001291483.1	circRNA_4662|Chr5:65301933_65303208_-	0.867583521	0.02514
NM_001081224.2	circRNA_3155|Chr19:25061108_25065459_+	0.867580416	0.025141
XM_006520023.2	circRNA_0157|Chr1:66801049_66802168_-	0.867554658	0.025151
XM_006503665.3	circRNA_0611|Chr10:75274017_75279961_+	0.867469097	0.025183
NM_053080.3	circRNA_5367|Chr7:28990988_28991325_+	0.867467897	0.025183
NM_007792.4	circRNA_0157|Chr1:66801049_66802168_-	0.867408695	0.025205
NM_146241.2	circRNA_3155|Chr19:25061108_25065459_+	0.867377509	0.025217
NM_026979.5	circRNA_3643|Chr2:122441559_122486090_+	0.867340988	0.02523
NM_177743.5	circRNA_0157|Chr1:66801049_66802168_-	0.867334403	0.025233
NM_009223.3	circRNA_0020|Chr1:10315205_10321709_+	0.867297221	0.025247
XM_011246479.2	circRNA_5811|Chr8:71992832_71998295_-	0.867285831	0.025251
XM_006533452.3	circRNA_0611|Chr10:75274017_75279961_+	0.867118389	0.025313
NM_199029.2	circRNA_0537|Chr10:43393871_43395692_+	0.867104813	0.025318
NM_001164086.1	circRNA_0962|Chr11:67194348_67220998_+	0.867063199	0.025334
XM_006527321.2	circRNA_3276|Chr19:60563162_60564294_-	0.867036115	0.025344
NM_001190325.1	circRNA_0611|Chr10:75274017_75279961_+	0.867031043	0.025346
NM_013560.2	circRNA_4926|Chr5:143080224_143081077_-	0.866995627	0.025359
XM_006529759.1	circRNA_5311|Chr6:145147636_145149357_+	0.866945061	0.025378
NM_007655.3	circRNA_0501|Chr10:25283893_25289730_-	0.866913121	0.02539
NM_007655.3	circRNA_6109|Chr9:57056714_57057861_+	0.866913121	0.02539
NM_001030305.2	circRNA_0611|Chr10:75274017_75279961_+	0.866877898	0.025403
NM_138955.3	circRNA_5990|Chr9:3475483_3477962_+	0.86682363	0.025423
NM_001097617.1	circRNA_4055|Chr3:126796838_126798280_+	0.866743531	0.025453
NM_145141.2	circRNA_2888|Chr18:21010677_21020999_+	0.866733366	0.025457
NM_013468.3	circRNA_0961|Chr11:67192403_67254873_+	0.866727261	0.025459
XM_006498901.3	circRNA_5639|Chr7:132771578_132779385_-	0.866718878	0.025462
NM_001190451.2	circRNA_2888|Chr18:21010677_21020999_+	0.866699824	0.025469
XM_017315313.1	circRNA_5311|Chr6:145147636_145149357_+	0.866639414	0.025492
NM_027455.3	circRNA_0020|Chr1:10315205_10321709_+	0.866639341	0.025492
NM_027293.1	circRNA_3832|Chr2:169883526_169886459_+	0.866621992	0.025498
XM_006506340.3	circRNA_3832|Chr2:169883526_169886459_+	0.866600094	0.025506
XM_017316736.1	circRNA_0611|Chr10:75274017_75279961_+	0.866565888	0.025519
NM_146125.2	circRNA_0020|Chr1:10315205_10321709_+	0.866498774	0.025544
XM_006532024.3	circRNA_5311|Chr6:145147636_145149357_+	0.866466126	0.025556
XM_006533539.3	circRNA_1632|Chr13:42055400_42055542_+	0.866426319	0.025571
XM_017317034.1	circRNA_5074|Chr6:40685277_40747150_+	0.866339381	0.025604
NM_011169.5	circRNA_5311|Chr6:145147636_145149357_+	0.86626686	0.025631
XM_006496277.3	circRNA_6446|ChrX:42217416_42250037_+	0.866213208	0.025651
XM_006524382.3	circRNA_5074|Chr6:40685277_40747150_+	0.866212833	0.025651
NM_027455.3	circRNA_3239|Chr19:45635735_45640521_-	0.866202067	0.025655
NM_134072.1	circRNA_0157|Chr1:66801049_66802168_-	0.866090468	0.025697

XM_017316736.1	circRNA_2888|Chr18:21010677_21020999_+	0.866008891	0.025728
NM_020025.4	circRNA_2888|Chr18:21010677_21020999_+	0.865966603	0.025743
XM_006504999.1	circRNA_4055|Chr3:126796838_126798280_+	0.865942923	0.025752
XM_011248576.2	circRNA_6390|Chr9:121727316_121731565_+	0.865898765	0.025769
XM_006508885.1	circRNA_6140|Chr9:61935380_61937535_-	0.865854614	0.025786
NM_013459.3	circRNA_0501|Chr10:25283893_25289730_-	0.86577918	0.025814
NM_013459.3	circRNA_6109|Chr9:57056714_57057861_+	0.86577918	0.025814
NM_133500.2	circRNA_0961|Chr11:67192403_67254873_+	0.86571974	0.025836
XM_011243391.2	circRNA_3832|Chr2:169883526_169886459_+	0.865697412	0.025845
NM_001159407.1	circRNA_3643|Chr2:122441559_122486090_+	0.865689958	0.025847
NM_177743.5	circRNA_5311|Chr6:145147636_145149357_+	0.865668747	0.025855
NM_172907.3	circRNA_0913|Chr11:54005394_54014456_-	0.865668317	0.025855
XM_006517308.3	circRNA_3276|Chr19:60563162_60564294_-	0.865624267	0.025872
NM_008204.2	circRNA_2888|Chr18:21010677_21020999_+	0.865583714	0.025887
XM_006508885.1	circRNA_0501|Chr10:25283893_25289730_-	0.865542137	0.025903
XM_006508885.1	circRNA_6109|Chr9:57056714_57057861_+	0.865542137	0.025903
NM_001159407.1	circRNA_4987|Chr6:4529068_4531010_+	0.865529293	0.025908
NM_009514.4	circRNA_0501|Chr10:25283893_25289730_-	0.865521634	0.025911
NM_009514.4	circRNA_6109|Chr9:57056714_57057861_+	0.865521634	0.025911
NM_032400.2	circRNA_2888|Chr18:21010677_21020999_+	0.865493471	0.025921
XM_006501309.3	circRNA_4487|Chr4:149156607_149161694_-	0.865485328	0.025924
NM_001252563.1	circRNA_2951|Chr18:39120119_39150118_+	0.865409771	0.025953
NM_001081185.1	circRNA_3832|Chr2:169883526_169886459_+	0.865352434	0.025974
XM_006540596.2	circRNA_0020|Chr1:10315205_10321709_+	0.865342072	0.025978
NM_145210.2	circRNA_4635|Chr5:43758222_43773659_-	0.865337827	0.02598
XM_006521796.1	circRNA_0501|Chr10:25283893_25289730_-	0.865321749	0.025986
XM_006521796.1	circRNA_6109|Chr9:57056714_57057861_+	0.865321749	0.025986
XM_006513165.1	circRNA_2951|Chr18:39120119_39150118_+	0.865183592	0.026038
XM_006505602.3	circRNA_0537|Chr10:43393871_43395692_+	0.865153744	0.026049
NM_026535.2	circRNA_3239|Chr19:45635735_45640521_-	0.865099355	0.02607
NM_144544.2	circRNA_4662|Chr5:65301933_65303208_-	0.865089146	0.026074
XM_006520023.2	circRNA_4055|Chr3:126796838_126798280_+	0.865076215	0.026079
NM_001134300.2	circRNA_0845|Chr11:29470315_29477458_+	0.865056067	0.026086
XM_006522398.3	circRNA_4055|Chr3:126796838_126798280_+	0.865006995	0.026105
NM_007482.3	circRNA_3276|Chr19:60563162_60564294_-	0.864992742	0.02611
NM_001033630.1	circRNA_0962|Chr11:67194348_67220998_+	0.864990492	0.026111
XM_017315313.1	circRNA_2951|Chr18:39120119_39150118_+	0.864957966	0.026123
NM_001081667.2	circRNA_0962|Chr11:67194348_67220998_+	0.864918632	0.026138
NM_009514.4	circRNA_0611|Chr10:75274017_75279961_+	0.864876454	0.026154
NM_177578.4	circRNA_3702|Chr2:143830084_143832057_-	0.864840278	0.026168
NM_001099314.1	circRNA_4635|Chr5:43758222_43773659_-	0.864778983	0.026191
XM_006533653.3	circRNA_4635|Chr5:43758222_43773659_-	0.864718136	0.026214
NM_009019.2	circRNA_4055|Chr3:126796838_126798280_+	0.864673724	0.026231
NM_001291483.1	circRNA_6446|ChrX:42217416_42250037_+	0.864667007	0.026233
NM_011658.2	circRNA_4662|Chr5:65301933_65303208_-	0.864641232	0.026243
NM_023143.3	circRNA_3702|Chr2:143830084_143832057_-	0.864603874	0.026257
NM_001204201.1	circRNA_6140|Chr9:61935380_61937535_-	0.864600497	0.026258
NM_009223.3	circRNA_0501|Chr10:25283893_25289730_-	0.864535052	0.026283
NM_009223.3	circRNA_6109|Chr9:57056714_57057861_+	0.864535052	0.026283
NM_009504.4	circRNA_3832|Chr2:169883526_169886459_+	0.864476866	0.026305
NM_007641.5	circRNA_0501|Chr10:25283893_25289730_-	0.864389201	0.026338
NM_007641.5	circRNA_6109|Chr9:57056714_57057861_+	0.864389201	0.026338
NM_145741.2	circRNA_3155|Chr19:25061108_25065459_+	0.864377189	0.026343
NM_009785.1	circRNA_0962|Chr11:67194348_67220998_+	0.864252768	0.02639
NM_027455.3	circRNA_3702|Chr2:143830084_143832057_-	0.864236462	0.026396
NM_007588.2	circRNA_0537|Chr10:43393871_43395692_+	0.864175653	0.02642
NM_011658.2	circRNA_2951|Chr18:39120119_39150118_+	0.864175126	0.02642
XM_006533653.3	circRNA_4487|Chr4:149156607_149161694_-	0.86415901	0.026426
XM_006502291.3	circRNA_0537|Chr10:43393871_43395692_+	0.864149544	0.026429

NM_009223.3	circRNA_2951|Chr18:39120119_39150118_+	0.864104878	0.026446
NM_007549.2	circRNA_3276|Chr19:60563162_60564294_-	0.863929304	0.026513
NM_001204959.1	circRNA_0020|Chr1:10315205_10321709_+	0.863871532	0.026535
NM_031180.2	circRNA_5311|Chr6:145147636_145149357_+	0.86384137	0.026547
XM_006498086.3	circRNA_3276|Chr19:60563162_60564294_-	0.863817372	0.026556
XM_006529753.2	circRNA_4926|Chr5:143080224_143081077_-	0.863767144	0.026575
XM_006527321.2	circRNA_6446|ChrX:42217416_42250037_+	0.863473201	0.026687
XM_006537815.3	circRNA_2313|Chr15:93452117_93465245_+	0.863446824	0.026697
NM_001097617.1	circRNA_4728|Chr5:97027831_97045167_+	0.86342698	0.026705
NM_001190448.1	circRNA_3155|Chr19:25061108_25065459_+	0.863426447	0.026705
XM_006496277.3	circRNA_4055|Chr3:126796838_126798280_+	0.863384796	0.026721
XM_011244488.2	circRNA_4055|Chr3:126796838_126798280_+	0.863351989	0.026733
NM_019866.1	circRNA_2951|Chr18:39120119_39150118_+	0.863323014	0.026744
NM_001081224.2	circRNA_0157|Chr1:66801049_66802168_-	0.863307816	0.02675
NM_025422.4	circRNA_4055|Chr3:126796838_126798280_+	0.863303292	0.026752
XM_006503371.2	circRNA_4926|Chr5:143080224_143081077_-	0.863298863	0.026754
NM_008940.3	circRNA_3155|Chr19:25061108_25065459_+	0.863270033	0.026765
NM_001033286.2	circRNA_5811|Chr8:71992832_71998295_-	0.863228912	0.02678
XM_011240149.1	circRNA_3832|Chr2:169883526_169886459_+	0.863212438	0.026787
XM_006495613.3	circRNA_5311|Chr6:145147636_145149357_+	0.863201785	0.026791
NM_133643.4	circRNA_4987|Chr6:4529068_4531010_+	0.863157733	0.026807
NM_009127.4	circRNA_0020|Chr1:10315205_10321709_+	0.863125847	0.02682
NM_001082546.1	circRNA_3239|Chr19:45635735_45640521_-	0.863115559	0.026824
NM_010809.2	circRNA_5311|Chr6:145147636_145149357_+	0.863022905	0.026859
XM_006503323.3	circRNA_3239|Chr19:45635735_45640521_-	0.863020095	0.02686
NM_008760.4	circRNA_0501|Chr10:25283893_25289730_-	0.862977767	0.026876
NM_008760.4	circRNA_6109|Chr9:57056714_57057861_+	0.862977767	0.026876
NM_016659.3	circRNA_0376|Chr1:172173943_172187460_+	0.862973529	0.026878
NM_001190448.1	circRNA_2888|Chr18:21010677_21020999_+	0.862955818	0.026885
XM_017322026.1	circRNA_4728|Chr5:97027831_97045167_+	0.86294271	0.02689
NM_153151.3	circRNA_2951|Chr18:39120119_39150118_+	0.862846545	0.026927
NM_001002896.2	circRNA_0501|Chr10:25283893_25289730_-	0.862802089	0.026944
NM_001002896.2	circRNA_6109|Chr9:57056714_57057861_+	0.862802089	0.026944
NM_001195662.1	circRNA_0961|Chr11:67192403_67254873_+	0.86279623	0.026946
NM_010608.2	circRNA_4987|Chr6:4529068_4531010_+	0.862753016	0.026962
XM_006503371.2	circRNA_0845|Chr11:29470315_29477458_+	0.862743217	0.026966
NM_009645.2	circRNA_0501|Chr10:25283893_25289730_-	0.862714247	0.026977
NM_009645.2	circRNA_6109|Chr9:57056714_57057861_+	0.862714247	0.026977
NM_007655.3	circRNA_2888|Chr18:21010677_21020999_+	0.86270121	0.026982
XM_006510297.3	circRNA_3155|Chr19:25061108_25065459_+	0.862696692	0.026984
XM_006534520.3	circRNA_3954|Chr3:88346445_88349444_+	0.862681201	0.02699
NM_001198955.1	circRNA_4926|Chr5:143080224_143081077_-	0.862652891	0.027001
NM_008966.3	circRNA_0376|Chr1:172173943_172187460_+	0.862625137	0.027012
NM_001029877.3	circRNA_5074|Chr6:40685277_40747150_+	0.862589805	0.027025
NM_001190448.1	circRNA_0157|Chr1:66801049_66802168_-	0.862561277	0.027036
XM_006500947.3	circRNA_0962|Chr11:67194348_67220998_+	0.862554403	0.027039
NM_001161541.1	circRNA_4662|Chr5:65301933_65303208_-	0.862543195	0.027043
XM_006506624.2	circRNA_4728|Chr5:97027831_97045167_+	0.86254115	0.027044
NM_009722.3	circRNA_0962|Chr11:67194348_67220998_+	0.862491656	0.027063
NM_001110009.2	circRNA_5311|Chr6:145147636_145149357_+	0.862486409	0.027065
XM_017312731.1	circRNA_5311|Chr6:145147636_145149357_+	0.862481223	0.027067
NM_199304.1	circRNA_3832|Chr2:169883526_169886459_+	0.862418527	0.027091
XM_017313038.1	circRNA_3239|Chr19:45635735_45640521_-	0.862380876	0.027105
XM_006521931.1	circRNA_6140|Chr9:61935380_61937535_-	0.862335423	0.027123
XM_006522506.3	circRNA_4728|Chr5:97027831_97045167_+	0.862330269	0.027125
XM_006511007.3	circRNA_3954|Chr3:88346445_88349444_+	0.862245891	0.027157
NM_007555.4	circRNA_4055|Chr3:126796838_126798280_+	0.86214549	0.027196
NM_025540.2	circRNA_6412|ChrMT:13917_14141_-	0.862137445	0.027199
NM_007763.3	circRNA_4055|Chr3:126796838_126798280_+	0.862105285	0.027211

NM_146063.1	circRNA_2888|Chr18:21010677_21020999_+	0.862042601	0.027236
NM_009393.2	circRNA_0962|Chr11:67194348_67220998_+	0.861996593	0.027253
XM_006524382.3	circRNA_5811|Chr8:71992832_71998295_-	0.861948902	0.027272
NM_011724.3	circRNA_4926|Chr5:143080224_143081077_-	0.861900298	0.02729
NM_053080.3	circRNA_3239|Chr19:45635735_45640521_-	0.861858717	0.027306
XM_006534171.3	circRNA_6140|Chr9:61935380_61937535_-	0.861790432	0.027333
NM_001161541.1	circRNA_4728|Chr5:97027831_97045167_+	0.861608089	0.027403
NM_016659.3	circRNA_0611|Chr10:75274017_75279961_+	0.861590566	0.02741
NM_001310636.1	circRNA_3702|Chr2:143830084_143832057_-	0.86153635	0.027431
XM_006508990.2	circRNA_6390|Chr9:121727316_121731565_+	0.861530119	0.027433
NM_001004357.2	circRNA_4987|Chr6:4529068_4531010_+	0.861509925	0.027441
XM_011247208.1	circRNA_6390|Chr9:121727316_121731565_+	0.861505199	0.027443
XM_006533539.3	circRNA_3702|Chr2:143830084_143832057_-	0.861498226	0.027446
XM_006503665.3	circRNA_3239|Chr19:45635735_45640521_-	0.861494112	0.027447
XM_006516928.2	circRNA_0020|Chr1:10315205_10321709_+	0.861474466	0.027455
NM_010608.2	circRNA_6140|Chr9:61935380_61937535_-	0.861435422	0.02747
NM_133643.4	circRNA_3643|Chr2:122441559_122486090_+	0.8614191	0.027476
XM_006507942.2	circRNA_5311|Chr6:145147636_145149357_+	0.861352521	0.027502
XM_006527480.3	circRNA_0961|Chr11:67192403_67254873_+	0.861348494	0.027504
NM_001030305.2	circRNA_4987|Chr6:4529068_4531010_+	0.861341472	0.027506
XM_006499444.3	circRNA_1399|Chr12:72783683_72786789_+	0.861304956	0.02752
NM_177743.5	circRNA_4662|Chr5:65301933_65303208_-	0.861280046	0.02753
XM_006516510.1	circRNA_5367|Chr7:28990988_28991325_+	0.861217153	0.027554
NM_011374.2	circRNA_6390|Chr9:121727316_121731565_+	0.861206376	0.027559
XM_011240779.2	circRNA_4055|Chr3:126796838_126798280_+	0.861191603	0.027564
XM_006510297.3	circRNA_6446|ChrX:42217416_42250037_+	0.861181302	0.027568
NM_145141.2	circRNA_0611|Chr10:75274017_75279961_+	0.861122654	0.027591
NM_016982.2	circRNA_2951|Chr18:39120119_39150118_+	0.861104253	0.027598
XM_017322026.1	circRNA_2951|Chr18:39120119_39150118_+	0.861015781	0.027633
NM_025422.4	circRNA_3239|Chr19:45635735_45640521_-	0.860980317	0.027646
NM_001289875.1	circRNA_0845|Chr11:29470315_29477458_+	0.860754651	0.027734
XM_017313062.1	circRNA_0537|Chr10:43393871_43395692_+	0.860731572	0.027743
XM_006498287.3	circRNA_1399|Chr12:72783683_72786789_+	0.860703167	0.027754
XM_006525698.2	circRNA_0962|Chr11:67194348_67220998_+	0.860668045	0.027768
NM_010055.3	circRNA_3954|Chr3:88346445_88349444_+	0.860658638	0.027771
NM_024406.2	circRNA_0611|Chr10:75274017_75279961_+	0.860631364	0.027782
NM_007555.4	circRNA_6446|ChrX:42217416_42250037_+	0.860594031	0.027796
XM_006498287.3	circRNA_1042|Chr11:80385854_80403408_+	0.860567054	0.027807
NM_013468.3	circRNA_5990|Chr9:3475483_3477962_+	0.860558165	0.02781
XM_006505388.2	circRNA_6446|ChrX:42217416_42250037_+	0.86050784	0.02783
NM_178676.4	circRNA_3832|Chr2:169883526_169886459_+	0.860494848	0.027835
NM_029685.1	circRNA_0537|Chr10:43393871_43395692_+	0.860436946	0.027858
XM_006525075.3	circRNA_6446|ChrX:42217416_42250037_+	0.860381356	0.027879
NM_009349.3	circRNA_2951|Chr18:39120119_39150118_+	0.860381213	0.027879
NM_134072.1	circRNA_5311|Chr6:145147636_145149357_+	0.860351109	0.027891
XM_006506624.2	circRNA_0501|Chr10:25283893_25289730_-	0.860336399	0.027897
XM_006506624.2	circRNA_6109|Chr9:57056714_57057861_+	0.860336399	0.027897
XM_017315734.1	circRNA_0845|Chr11:29470315_29477458_+	0.860316591	0.027904
XM_006533539.3	circRNA_4987|Chr6:4529068_4531010_+	0.860314405	0.027905
XM_006523030.3	circRNA_0537|Chr10:43393871_43395692_+	0.860310401	0.027907
XM_006506624.2	circRNA_5639|Chr7:132771578_132779385_-	0.860303865	0.027909
NM_001291930.1	circRNA_3643|Chr2:122441559_122486090_+	0.860283841	0.027917
NM_001301295.1	circRNA_0501|Chr10:25283893_25289730_-	0.860260926	0.027926
NM_001301295.1	circRNA_6109|Chr9:57056714_57057861_+	0.860260926	0.027926
NM_134072.1	circRNA_3239|Chr19:45635735_45640521_-	0.860105902	0.027987
NM_173427.2	circRNA_3702|Chr2:143830084_143832057_-	0.860097193	0.02799
NM_001302765.1	circRNA_0962|Chr11:67194348_67220998_+	0.860073657	0.027999
XM_006529759.1	circRNA_0611|Chr10:75274017_75279961_+	0.860008622	0.028025
XM_017316056.1	circRNA_0611|Chr10:75274017_75279961_+	0.859989701	0.028032

NM_023670.3	circRNA_0376|Chr1:172173943_172187460_+	0.859969217	0.02804
NM_207231.1	circRNA_5311|Chr6:145147636_145149357_+	0.859906951	0.028064
NM_008479.2	circRNA_1042|Chr11:80385854_80403408_+	0.859894059	0.028069
NM_001190325.1	circRNA_5311|Chr6:145147636_145149357_+	0.859860873	0.028082
NM_177595.4	circRNA_6140|Chr9:61935380_61937535_-	0.859843246	0.028089
NM_138955.3	circRNA_5811|Chr8:71992832_71998295_-	0.859837998	0.028091
NM_009930.2	circRNA_4662|Chr5:65301933_65303208_-	0.859828741	0.028095
XM_006529829.3	circRNA_0611|Chr10:75274017_75279961_+	0.859806985	0.028103
NM_001317365.1	circRNA_3003|Chr18:67545615_67587859_-	0.859782053	0.028113
XM_011244046.2	circRNA_4055|Chr3:126796838_126798280_+	0.859760702	0.028122
NM_007555.4	circRNA_0376|Chr1:172173943_172187460_+	0.859748916	0.028126
XM_006534520.3	circRNA_5074|Chr6:40685277_40747150_+	0.859727756	0.028134
NM_009605.4	circRNA_6446|ChrX:42217416_42250037_+	0.859693558	0.028148
NM_001309809.2	circRNA_0376|Chr1:172173943_172187460_+	0.859633376	0.028171
NM_145141.2	circRNA_5311|Chr6:145147636_145149357_+	0.859611378	0.02818
NM_001290273.1	circRNA_0020|Chr1:10315205_10321709_+	0.859450235	0.028243
XM_017313038.1	circRNA_3155|Chr19:25061108_25065459_+	0.859362722	0.028277
XM_006512296.3	circRNA_3832|Chr2:169883526_169886459_+	0.859331656	0.02829
NM_015814.2	circRNA_2888|Chr18:21010677_21020999_+	0.859288175	0.028307
XM_006532120.2	circRNA_6140|Chr9:61935380_61937535_-	0.859286528	0.028307
NM_010858.4	circRNA_0376|Chr1:172173943_172187460_+	0.859278025	0.028311
XM_017317946.1	circRNA_4926|Chr5:143080224_143081077_-	0.859260807	0.028317
XM_006498287.3	circRNA_3425|Chr2:37624173_37627526_-	0.859225822	0.028331
XM_006498287.3	circRNA_3607|Chr2:119057281_119064097_+	0.859225822	0.028331
NM_021342.1	circRNA_0157|Chr1:66801049_66802168_-	0.859171366	0.028353
XM_006520437.2	circRNA_3155|Chr19:25061108_25065459_+	0.859163912	0.028355
XM_006541297.3	circRNA_0537|Chr10:43393871_43395692_+	0.859149809	0.028361
NM_172454.2	circRNA_0962|Chr11:67194348_67220998_+	0.859114688	0.028375
NM_144936.1	circRNA_0501|Chr10:25283893_25289730_-	0.85910602	0.028378
NM_144936.1	circRNA_6109|Chr9:57056714_57057861_+	0.85910602	0.028378
NM_007792.4	circRNA_4055|Chr3:126796838_126798280_+	0.859038659	0.028405
NM_023670.3	circRNA_2888|Chr18:21010677_21020999_+	0.858998599	0.02842
XM_011248602.2	circRNA_5074|Chr6:40685277_40747150_+	0.858967671	0.028433
NM_009381.3	circRNA_6446|ChrX:42217416_42250037_+	0.858938442	0.028444
XM_017322026.1	circRNA_0611|Chr10:75274017_75279961_+	0.858930052	0.028447
XM_006516510.1	circRNA_0501|Chr10:25283893_25289730_-	0.858921304	0.028451
XM_006516510.1	circRNA_6109|Chr9:57056714_57057861_+	0.858921304	0.028451
XM_006521428.3	circRNA_3643|Chr2:122441559_122486090_+	0.858902682	0.028458
NM_001190448.1	circRNA_3239|Chr19:45635735_45640521_-	0.858876541	0.028468
NM_011724.3	circRNA_6390|Chr9:121727316_121731565_+	0.858816974	0.028492
NM_008872.3	circRNA_6140|Chr9:61935380_61937535_-	0.858746997	0.028519
NM_007655.3	circRNA_6140|Chr9:61935380_61937535_-	0.858725908	0.028528
NM_001291930.1	circRNA_3239|Chr19:45635735_45640521_-	0.858702933	0.028537
XM_006530353.3	circRNA_0961|Chr11:67192403_67254873_+	0.85866699	0.028551
NM_009127.4	circRNA_3155|Chr19:25061108_25065459_+	0.858627314	0.028567
NM_001195084.1	circRNA_3155|Chr19:25061108_25065459_+	0.858610169	0.028573
NM_001242558.1	circRNA_3954|Chr3:88346445_88349444_+	0.85859896	0.028578
NM_010055.3	circRNA_0845|Chr11:29470315_29477458_+	0.858581316	0.028585
NM_010608.2	circRNA_3643|Chr2:122441559_122486090_+	0.85853804	0.028602
NM_029844.3	circRNA_2888|Chr18:21010677_21020999_+	0.858425167	0.028646
NM_001097617.1	circRNA_2888|Chr18:21010677_21020999_+	0.858405278	0.028654
NM_008804.4	circRNA_3702|Chr2:143830084_143832057_-	0.858375575	0.028666
XM_017312667.1	circRNA_0961|Chr11:67192403_67254873_+	0.858330916	0.028684
NM_001190325.1	circRNA_0376|Chr1:172173943_172187460_+	0.858258207	0.028712
XM_006520618.2	circRNA_6446|ChrX:42217416_42250037_+	0.858137452	0.02876
XM_006532120.2	circRNA_6446|ChrX:42217416_42250037_+	0.858135697	0.028761
NM_001290512.1	circRNA_3832|Chr2:169883526_169886459_+	0.858020113	0.028806
NM_009135.2	circRNA_0157|Chr1:66801049_66802168_-	0.858016707	0.028808
XM_006516224.3	circRNA_4926|Chr5:143080224_143081077_-	0.858003591	0.028813

NM_001039047.1	circRNA_0845|Chr11:29470315_29477458_+	0.857995126	0.028816
NM_008940.3	circRNA_0157|Chr1:66801049_66802168_-	0.85798899	0.028819
NM_027293.1	circRNA_6390|Chr9:121727316_121731565_+	0.857959222	0.02883
NM_010450.3	circRNA_0157|Chr1:66801049_66802168_-	0.857929996	0.028842
NM_001190379.1	circRNA_6390|Chr9:121727316_121731565_+	0.857831737	0.028881
XM_006541047.3	circRNA_4987|Chr6:4529068_4531010_+	0.857793408	0.028896
XM_011250776.2	circRNA_0020|Chr1:10315205_10321709_+	0.85778146	0.028901
XM_006499444.3	circRNA_3425|Chr2:37624173_37627526_-	0.857748381	0.028914
XM_006499444.3	circRNA_3607|Chr2:119057281_119064097_+	0.857748381	0.028914
NM_175260.2	circRNA_3003|Chr18:67545615_67587859_-	0.857742939	0.028916
XM_006527452.3	circRNA_6446|ChrX:42217416_42250037_+	0.857733378	0.02892
NM_001110009.2	circRNA_2888|Chr18:21010677_21020999_+	0.857699015	0.028934
NM_010450.3	circRNA_2951|Chr18:39120119_39150118_+	0.85763707	0.028958
NM_172907.3	circRNA_0376|Chr1:172173943_172187460_+	0.857608526	0.028969
XM_006515787.3	circRNA_0157|Chr1:66801049_66802168_-	0.857561605	0.028988
NM_009223.3	circRNA_6446|ChrX:42217416_42250037_+	0.857513799	0.029007
XM_006508990.2	circRNA_4926|Chr5:143080224_143081077_-	0.85748729	0.029018
NM_020001.2	circRNA_6446|ChrX:42217416_42250037_+	0.857454544	0.029031
NM_011581.3	circRNA_0157|Chr1:66801049_66802168_-	0.857447902	0.029033
XM_006503371.2	circRNA_0537|Chr10:43393871_43395692_+	0.857431126	0.02904
NM_001110322.1	circRNA_2888|Chr18:21010677_21020999_+	0.85739312	0.029055
XM_006532120.2	circRNA_0611|Chr10:75274017_75279961_+	0.857340336	0.029076
NM_001161746.1	circRNA_3832|Chr2:169883526_169886459_+	0.857164625	0.029146
XM_006501309.3	circRNA_3954|Chr3:88346445_88349444_+	0.857117003	0.029165
NM_030206.4	circRNA_4728|Chr5:97027831_97045167_+	0.85703221	0.029199
NM_009135.2	circRNA_4987|Chr6:4529068_4531010_+	0.857018629	0.029204
NM_009019.2	circRNA_0501|Chr10:25283893_25289730_-	0.856993106	0.029214
NM_009019.2	circRNA_6109|Chr9:57056714_57057861_+	0.856993106	0.029214
XM_017313038.1	circRNA_4055|Chr3:126796838_126798280_+	0.856956842	0.029229
XM_006513165.1	circRNA_2888|Chr18:21010677_21020999_+	0.856935932	0.029237
NM_010608.2	circRNA_0611|Chr10:75274017_75279961_+	0.856862872	0.029266
NM_173437.2	circRNA_3954|Chr3:88346445_88349444_+	0.856856518	0.029269
NM_144539.5	circRNA_3276|Chr19:60563162_60564294_-	0.85680658	0.029288
XM_006529753.2	circRNA_3954|Chr3:88346445_88349444_+	0.856804405	0.029289
XM_017321602.1	circRNA_0020|Chr1:10315205_10321709_+	0.856783101	0.029298
NM_024283.3	circRNA_2951|Chr18:39120119_39150118_+	0.856780454	0.029299
XM_006520023.2	circRNA_0611|Chr10:75274017_75279961_+	0.85673666	0.029316
NM_010279.3	circRNA_6446|ChrX:42217416_42250037_+	0.856730901	0.029319
XM_006499444.3	circRNA_3954|Chr3:88346445_88349444_+	0.85663155	0.029358
NM_010858.4	circRNA_5639|Chr7:132771578_132779385_-	0.856628477	0.02936
XM_006498901.3	circRNA_0376|Chr1:172173943_172187460_+	0.85661187	0.029366
XM_006529759.1	circRNA_0376|Chr1:172173943_172187460_+	0.85659024	0.029375
NM_001110508.1	circRNA_6390|Chr9:121727316_121731565_+	0.856570254	0.029383
NM_001113515.2	circRNA_2313|Chr15:93452117_93465245_+	0.856535097	0.029397
NM_009246.3	circRNA_4635|Chr5:43758222_43773659_-	0.856439394	0.029435
NM_008185.3	circRNA_6446|ChrX:42217416_42250037_+	0.856414247	0.029445
NM_001204201.1	circRNA_0020|Chr1:10315205_10321709_+	0.856281673	0.029498
XM_006534520.3	circRNA_0845|Chr11:29470315_29477458_+	0.856279637	0.029499
XM_006529829.3	circRNA_1632|Chr13:42055400_42055542_+	0.856270583	0.029503
XM_006504999.1	circRNA_6140|Chr9:61935380_61937535_-	0.856213037	0.029526
XM_006520186.2	circRNA_0020|Chr1:10315205_10321709_+	0.856131822	0.029558
NM_031183.2	circRNA_4728|Chr5:97027831_97045167_+	0.856109698	0.029567
XM_006524777.3	circRNA_4728|Chr5:97027831_97045167_+	0.856051334	0.02959
XM_011250217.1	circRNA_0537|Chr10:43393871_43395692_+	0.856003782	0.029609
NM_133871.2	circRNA_3954|Chr3:88346445_88349444_+	0.855986082	0.029617
NM_001190451.2	circRNA_3276|Chr19:60563162_60564294_-	0.855886533	0.029657
NM_024406.2	circRNA_2888|Chr18:21010677_21020999_+	0.855773449	0.029702
NM_001030305.2	circRNA_3643|Chr2:122441559_122486090_+	0.855767039	0.029704
XM_011249157.2	circRNA_0611|Chr10:75274017_75279961_+	0.855731942	0.029719

NM_010090.2	circRNA_6140|Chr9:61935380_61937535_-	0.855714229	0.029726
XM_006508990.2	circRNA_5074|Chr6:40685277_40747150_+	0.855711182	0.029727
NM_009381.3	circRNA_5311|Chr6:145147636_145149357_+	0.855706637	0.029729
NM_008760.4	circRNA_3155|Chr19:25061108_25065459_+	0.855691538	0.029735
XM_011245529.2	circRNA_0845|Chr11:29470315_29477458_+	0.855681752	0.029739
NM_011044.2	circRNA_0157|Chr1:66801049_66802168_-	0.855655524	0.029749
XM_006509531.3	circRNA_6140|Chr9:61935380_61937535_-	0.855619856	0.029764
XM_006527321.2	circRNA_5311|Chr6:145147636_145149357_+	0.855591309	0.029775
XM_011248201.2	circRNA_5074|Chr6:40685277_40747150_+	0.855591158	0.029775
NM_008872.3	circRNA_1632|Chr13:42055400_42055542_+	0.855558327	0.029788
NM_001204959.1	circRNA_4662|Chr5:65301933_65303208_-	0.855504577	0.02981
NM_001310705.1	circRNA_3239|Chr19:45635735_45640521_-	0.855490756	0.029815
NM_144936.1	circRNA_2951|Chr18:39120119_39150118_+	0.8554826	0.029819
NM_001301354.1	circRNA_0157|Chr1:66801049_66802168_-	0.855466162	0.029825
NM_008458.2	circRNA_0157|Chr1:66801049_66802168_-	0.855439988	0.029836
NM_025711.3	circRNA_0501|Chr10:25283893_25289730_-	0.855432614	0.029839
NM_025711.3	circRNA_6109|Chr9:57056714_57057861_+	0.855432614	0.029839
XM_017315376.1	circRNA_4055|Chr3:126796838_126798280_+	0.855318102	0.029885
NM_009930.2	circRNA_0157|Chr1:66801049_66802168_-	0.855291512	0.029896
XM_006514077.1	circRNA_0845|Chr11:29470315_29477458_+	0.855280681	0.0299
NM_001039047.1	circRNA_4926|Chr5:143080224_143081077_-	0.855256064	0.02991
NM_001302257.1	circRNA_4987|Chr6:4529068_4531010_+	0.855212714	0.029927
XM_006512296.3	circRNA_6390|Chr9:121727316_121731565_+	0.855104631	0.029971
XM_006496884.3	circRNA_4055|Chr3:126796838_126798280_+	0.855044068	0.029995
NM_013743.2	circRNA_4926|Chr5:143080224_143081077_-	0.855005268	0.030011
XM_006515980.3	circRNA_5311|Chr6:145147636_145149357_+	0.854942608	0.030036
NM_134072.1	circRNA_2951|Chr18:39120119_39150118_+	0.854919946	0.030045
NM_007555.4	circRNA_5311|Chr6:145147636_145149357_+	0.854896171	0.030055
NM_015814.2	circRNA_0611|Chr10:75274017_75279961_+	0.854858028	0.03007
NM_008067.4	circRNA_0157|Chr1:66801049_66802168_-	0.854845054	0.030076
NM_009785.1	circRNA_6412|ChrMT:13917_14141_-	0.854799928	0.030094
NM_199304.1	circRNA_5074|Chr6:40685277_40747150_+	0.854797341	0.030095
NM_025711.3	circRNA_3239|Chr19:45635735_45640521_-	0.854770538	0.030106
XM_006501829.3	circRNA_0962|Chr11:67194348_67220998_+	0.854762795	0.030109
NM_009223.3	circRNA_0611|Chr10:75274017_75279961_+	0.854717907	0.030127
NM_013560.2	circRNA_0961|Chr11:67192403_67254873_+	0.854678246	0.030143
XM_011243586.2	circRNA_0611|Chr10:75274017_75279961_+	0.854658197	0.030151
NM_001204959.1	circRNA_6140|Chr9:61935380_61937535_-	0.854636193	0.03016
XM_017316736.1	circRNA_3702|Chr2:143830084_143832057_-	0.854611256	0.03017
XM_006498872.3	circRNA_3832|Chr2:169883526_169886459_+	0.854596252	0.030176
NM_001161541.1	circRNA_0501|Chr10:25283893_25289730_-	0.854527579	0.030204
NM_001161541.1	circRNA_6109|Chr9:57056714_57057861_+	0.854527579	0.030204
NM_173427.2	circRNA_0376|Chr1:172173943_172187460_+	0.854466788	0.030229
NM_001198841.1	circRNA_0537|Chr10:43393871_43395692_+	0.854439534	0.03024
XM_006524777.3	circRNA_0376|Chr1:172173943_172187460_+	0.854430062	0.030244
XM_006513165.1	circRNA_3155|Chr19:25061108_25065459_+	0.854369307	0.030268
NM_007549.2	circRNA_3239|Chr19:45635735_45640521_-	0.854344315	0.030278
XM_006503323.3	circRNA_6446|ChrX:42217416_42250037_+	0.854319327	0.030288
NM_011044.2	circRNA_4662|Chr5:65301933_65303208_-	0.854319092	0.030289
XM_017313038.1	circRNA_2951|Chr18:39120119_39150118_+	0.854300121	0.030296
XM_006503323.3	circRNA_3643|Chr2:122441559_122486090_+	0.85427468	0.030307
XM_006538687.2	circRNA_3954|Chr3:88346445_88349444_+	0.854269683	0.030309
XM_011244488.2	circRNA_5311|Chr6:145147636_145149357_+	0.854237449	0.030322
XM_006540566.1	circRNA_4635|Chr5:43758222_43773659_-	0.854236555	0.030322
XM_006537508.2	circRNA_3276|Chr19:60563162_60564294_-	0.854229584	0.030325
NM_016933.3	circRNA_0611|Chr10:75274017_75279961_+	0.854152586	0.030356
NM_177578.4	circRNA_0376|Chr1:172173943_172187460_+	0.854089784	0.030381
XM_006517308.3	circRNA_0020|Chr1:10315205_10321709_+	0.854015927	0.030411
NM_011658.2	circRNA_0501|Chr10:25283893_25289730_-	0.853986656	0.030423

NM_011658.2	circRNA_6109|Chr9:57056714_57057861_+	0.853986656	0.030423
XM_006529759.1	circRNA_0501|Chr10:25283893_25289730_-	0.853967101	0.030431
XM_006529759.1	circRNA_6109|Chr9:57056714_57057861_+	0.853967101	0.030431
XM_017314347.1	circRNA_6140|Chr9:61935380_61937535_-	0.853963545	0.030433
NM_025540.2	circRNA_1980|Chr14:50951404_50963869_+	0.853871657	0.03047
NM_007529.2	circRNA_2888|Chr18:21010677_21020999_+	0.853840564	0.030483
NM_007792.4	circRNA_3702|Chr2:143830084_143832057_-	0.853830263	0.030487
NM_007792.4	circRNA_6140|Chr9:61935380_61937535_-	0.853812773	0.030494
XM_017315376.1	circRNA_5311|Chr6:145147636_145149357_+	0.853765533	0.030513
NM_001327998.1	circRNA_3155|Chr19:25061108_25065459_+	0.853706325	0.030537
XM_017320735.1	circRNA_4728|Chr5:97027831_97045167_+	0.853691581	0.030543
XM_006508867.2	circRNA_3155|Chr19:25061108_25065459_+	0.85367245	0.030551
XM_006520186.2	circRNA_5311|Chr6:145147636_145149357_+	0.853607274	0.030578
XM_006523030.3	circRNA_6390|Chr9:121727316_121731565_+	0.853541068	0.030605
XM_006510077.3	circRNA_5639|Chr7:132771578_132779385_-	0.853487398	0.030626
NM_010779.2	circRNA_3276|Chr19:60563162_60564294_-	0.853395456	0.030664
NM_028804.1	circRNA_2888|Chr18:21010677_21020999_+	0.853385437	0.030668
NM_008623.5	circRNA_0611|Chr10:75274017_75279961_+	0.853350633	0.030682
XM_006506152.3	circRNA_2313|Chr15:93452117_93465245_+	0.85332633	0.030692
NM_001291483.1	circRNA_0501|Chr10:25283893_25289730_-	0.853277781	0.030712
NM_001291483.1	circRNA_6109|Chr9:57056714_57057861_+	0.853277781	0.030712
NM_001159407.1	circRNA_0501|Chr10:25283893_25289730_-	0.853265917	0.030717
NM_001159407.1	circRNA_6109|Chr9:57056714_57057861_+	0.853265917	0.030717
XM_017320093.1	circRNA_0157|Chr1:66801049_66802168_-	0.853208931	0.03074
NM_001309809.2	circRNA_0501|Chr10:25283893_25289730_-	0.853203752	0.030742
NM_001309809.2	circRNA_6109|Chr9:57056714_57057861_+	0.853203752	0.030742
NM_001301354.1	circRNA_5367|Chr7:28990988_28991325_+	0.853196357	0.030745
NM_001290469.1	circRNA_0845|Chr11:29470315_29477458_+	0.853176227	0.030753
XM_006515980.3	circRNA_0501|Chr10:25283893_25289730_-	0.853124129	0.030775
XM_006515980.3	circRNA_6109|Chr9:57056714_57057861_+	0.853124129	0.030775
NM_009381.3	circRNA_3239|Chr19:45635735_45640521_-	0.852982931	0.030832
XM_017315841.1	circRNA_0962|Chr11:67194348_67220998_+	0.852976909	0.030835
NM_001142804.1	circRNA_3276|Chr19:60563162_60564294_-	0.852976872	0.030835
NM_133167.3	circRNA_3954|Chr3:88346445_88349444_+	0.852941961	0.030849
XM_006508002.3	circRNA_0962|Chr11:67194348_67220998_+	0.852906148	0.030864
NM_001190451.2	circRNA_5311|Chr6:145147636_145149357_+	0.852901339	0.030866
NM_009020.3	circRNA_6446|ChrX:42217416_42250037_+	0.852857216	0.030884
NM_008204.2	circRNA_0501|Chr10:25283893_25289730_-	0.85284086	0.03089
NM_008204.2	circRNA_6109|Chr9:57056714_57057861_+	0.85284086	0.03089
NM_010140.3	circRNA_3155|Chr19:25061108_25065459_+	0.852796147	0.030909
NM_007588.2	circRNA_3003|Chr18:67545615_67587859_-	0.852772589	0.030918
XM_006506624.2	circRNA_4662|Chr5:65301933_65303208_-	0.852675036	0.030958
NM_011430.3	circRNA_6140|Chr9:61935380_61937535_-	0.852629288	0.030977
NM_028472.2	circRNA_4728|Chr5:97027831_97045167_+	0.852621767	0.03098
XM_006510077.3	circRNA_0611|Chr10:75274017_75279961_+	0.852578192	0.030998
NM_009381.3	circRNA_4662|Chr5:65301933_65303208_-	0.852481218	0.031038
XM_006511102.2	circRNA_0501|Chr10:25283893_25289730_-	0.85237513	0.031081
XM_006511102.2	circRNA_6109|Chr9:57056714_57057861_+	0.85237513	0.031081
XM_006513165.1	circRNA_4662|Chr5:65301933_65303208_-	0.852329577	0.0311
NM_020001.2	circRNA_0913|Chr11:54005394_54014456_-	0.852280868	0.03112
XM_011241948.2	circRNA_4662|Chr5:65301933_65303208_-	0.852209628	0.031149
XM_006530238.3	circRNA_0537|Chr10:43393871_43395692_+	0.852170416	0.031165
NM_026385.4	circRNA_4987|Chr6:4529068_4531010_+	0.852128389	0.031182
XM_006505388.2	circRNA_1632|Chr13:42055400_42055542_+	0.852045022	0.031217
NM_177371.3	circRNA_3276|Chr19:60563162_60564294_-	0.851953998	0.031254
NM_009246.3	circRNA_5811|Chr8:71992832_71998295_-	0.851953121	0.031254
NM_001313939.1	circRNA_0501|Chr10:25283893_25289730_-	0.851926615	0.031265
NM_001313939.1	circRNA_6109|Chr9:57056714_57057861_+	0.851926615	0.031265
NM_009127.4	circRNA_4055|Chr3:126796838_126798280_+	0.851882278	0.031284

NM_001244031.1	circRNA_0537|Chr10:43393871_43395692_+	0.85187382	0.031287
XM_006524777.3	circRNA_3239|Chr19:45635735_45640521_-	0.851865433	0.03129
XM_006510297.3	circRNA_4728|Chr5:97027831_97045167_+	0.851840741	0.031301
NM_001110009.2	circRNA_0611|Chr10:75274017_75279961_+	0.851829188	0.031305
XM_006515096.2	circRNA_3003|Chr18:67545615_67587859_-	0.85177657	0.031327
XM_011241948.2	circRNA_3155|Chr19:25061108_25065459_+	0.851775168	0.031328
XM_006529759.1	circRNA_3702|Chr2:143830084_143832057_-	0.851668961	0.031371
XM_006522398.3	circRNA_5639|Chr7:132771578_132779385_-	0.851454618	0.03146
XM_006513859.3	circRNA_0962|Chr11:67194348_67220998_+	0.851431196	0.031469
XM_011250776.2	circRNA_3155|Chr19:25061108_25065459_+	0.851325361	0.031513
XM_017316736.1	circRNA_2951|Chr18:39120119_39150118_+	0.851154726	0.031584
NM_173437.2	circRNA_3832|Chr2:169883526_169886459_+	0.851150003	0.031585
NM_013906.3	circRNA_6412|ChrMT:13917_14141_-	0.851118859	0.031598
NM_001159407.1	circRNA_0611|Chr10:75274017_75279961_+	0.851059898	0.031623
NM_177578.4	circRNA_4662|Chr5:65301933_65303208_-	0.850948549	0.031669
NM_013912.3	circRNA_5311|Chr6:145147636_145149357_+	0.850944744	0.03167
XM_011243391.2	circRNA_0537|Chr10:43393871_43395692_+	0.850833836	0.031716
XM_006510701.3	circRNA_0961|Chr11:67192403_67254873_+	0.850827272	0.031719
XM_006515980.3	circRNA_2951|Chr18:39120119_39150118_+	0.850822666	0.031721
XM_006501237.3	circRNA_6140|Chr9:61935380_61937535_-	0.85077444	0.031741
NM_023716.2	circRNA_4055|Chr3:126796838_126798280_+	0.850749031	0.031751
NM_001110322.1	circRNA_2951|Chr18:39120119_39150118_+	0.850724664	0.031762
XM_006540596.2	circRNA_0376|Chr1:172173943_172187460_+	0.850712376	0.031767
NM_009824.2	circRNA_6390|Chr9:121727316_121731565_+	0.850671017	0.031784
NM_026385.4	circRNA_0611|Chr10:75274017_75279961_+	0.850635691	0.031798
XM_006512076.3	circRNA_3832|Chr2:169883526_169886459_+	0.850563736	0.031828
NM_010608.2	circRNA_3239|Chr19:45635735_45640521_-	0.850548601	0.031835
NM_009514.4	circRNA_5311|Chr6:145147636_145149357_+	0.850526368	0.031844
XM_017322026.1	circRNA_5639|Chr7:132771578_132779385_-	0.85050218	0.031854
XM_006538830.1	circRNA_2313|Chr15:93452117_93465245_+	0.850482848	0.031862
NM_010858.4	circRNA_0501|Chr10:25283893_25289730_-	0.850481104	0.031863
NM_010858.4	circRNA_6109|Chr9:57056714_57057861_+	0.850481104	0.031863
NM_144799.2	circRNA_0537|Chr10:43393871_43395692_+	0.850464041	0.03187
NM_007588.2	circRNA_1042|Chr11:80385854_80403408_+	0.850415041	0.03189
NM_011169.5	circRNA_4728|Chr5:97027831_97045167_+	0.850400034	0.031896
XM_006528026.2	circRNA_4926|Chr5:143080224_143081077_-	0.850371674	0.031908
NM_181315.4	circRNA_0501|Chr10:25283893_25289730_-	0.85036176	0.031912
NM_181315.4	circRNA_6109|Chr9:57056714_57057861_+	0.85036176	0.031912
XM_017314347.1	circRNA_4662|Chr5:65301933_65303208_-	0.85033845	0.031922
NM_010450.3	circRNA_4055|Chr3:126796838_126798280_+	0.850323299	0.031928
XM_017315313.1	circRNA_0501|Chr10:25283893_25289730_-	0.850293673	0.03194
XM_017315313.1	circRNA_6109|Chr9:57056714_57057861_+	0.850293673	0.03194
NM_001082546.1	circRNA_3155|Chr19:25061108_25065459_+	0.85029167	0.031941
NM_001289875.1	circRNA_0961|Chr11:67192403_67254873_+	0.850263419	0.031953
NM_007588.2	circRNA_1399|Chr12:72783683_72786789_+	0.850254417	0.031957
NM_009223.3	circRNA_3276|Chr19:60563162_60564294_-	0.850246216	0.03196
NM_023456.3	circRNA_6446|ChrX:42217416_42250037_+	0.850215408	0.031973
NM_175022.2	circRNA_6390|Chr9:121727316_121731565_+	0.850167354	0.031993
NM_020508.4	circRNA_3832|Chr2:169883526_169886459_+	0.85015011	0.032
XM_006511102.2	circRNA_2951|Chr18:39120119_39150118_+	0.850028798	0.032051
NM_007817.2	circRNA_3155|Chr19:25061108_25065459_+	0.850023894	0.032053
NM_009127.4	circRNA_3276|Chr19:60563162_60564294_-	0.850022322	0.032053
NM_001290993.1	circRNA_6446|ChrX:42217416_42250037_+	0.85001544	0.032056
NM_007588.2	circRNA_3954|Chr3:88346445_88349444_+	0.850005858	0.03206
NM_178184.2	circRNA_0376|Chr1:172173943_172187460_+	0.84996108	0.032079
XM_006540566.1	circRNA_3832|Chr2:169883526_169886459_+	0.849919716	0.032096
NM_001289875.1	circRNA_5074|Chr6:40685277_40747150_+	0.849890652	0.032108
XM_006525099.3	circRNA_0537|Chr10:43393871_43395692_+	0.84987632	0.032114
XM_017316439.1	circRNA_6446|ChrX:42217416_42250037_+	0.849801639	0.032145

NM_001013784.1	circRNA_6390|Chr9:121727316_121731565_+	0.849792164	0.032149
NM_031183.2	circRNA_1632|Chr13:42055400_42055542_+	0.849766519	0.03216
XM_006529759.1	circRNA_0020|Chr1:10315205_10321709_+	0.849707755	0.032184
XM_006502291.3	circRNA_3832|Chr2:169883526_169886459_+	0.849642333	0.032212
XM_006527321.2	circRNA_3239|Chr19:45635735_45640521_-	0.849596642	0.032231
NM_010608.2	circRNA_3155|Chr19:25061108_25065459_+	0.849595781	0.032231
NM_024406.2	circRNA_5639|Chr7:132771578_132779385_-	0.849570919	0.032241
NM_145226.2	circRNA_3832|Chr2:169883526_169886459_+	0.849550947	0.03225
NM_009199.2	circRNA_6412|ChrMT:13917_14141_-	0.84951779	0.032264
NM_009019.2	circRNA_2888|Chr18:21010677_21020999_+	0.849513821	0.032265
NM_207231.1	circRNA_3702|Chr2:143830084_143832057_-	0.849512716	0.032266
XM_006527321.2	circRNA_4055|Chr3:126796838_126798280_+	0.849468395	0.032284
NM_024283.3	circRNA_0501|Chr10:25283893_25289730_-	0.849438392	0.032297
NM_024283.3	circRNA_6109|Chr9:57056714_57057861_+	0.849438392	0.032297
XM_006505602.3	circRNA_3954|Chr3:88346445_88349444_+	0.849436694	0.032297
NM_009785.1	circRNA_1980|Chr14:50951404_50963869_+	0.849364401	0.032328
NM_028472.2	circRNA_2951|Chr18:39120119_39150118_+	0.849331211	0.032341
XM_017316715.1	circRNA_3832|Chr2:169883526_169886459_+	0.849293374	0.032357
NM_001301354.1	circRNA_2951|Chr18:39120119_39150118_+	0.849287627	0.03236
NM_007549.2	circRNA_5311|Chr6:145147636_145149357_+	0.849282884	0.032362
XM_006539726.3	circRNA_3832|Chr2:169883526_169886459_+	0.849260771	0.032371
NM_007641.5	circRNA_5311|Chr6:145147636_145149357_+	0.849225968	0.032385
NM_134072.1	circRNA_5639|Chr7:132771578_132779385_-	0.849211625	0.032391
NM_001270475.1	circRNA_6390|Chr9:121727316_121731565_+	0.84908781	0.032443
XM_006530585.3	circRNA_4926|Chr5:143080224_143081077_-	0.849064785	0.032453
NM_001004357.2	circRNA_0157|Chr1:66801049_66802168_-	0.849044111	0.032462
NM_019696.2	circRNA_0611|Chr10:75274017_75279961_+	0.84904026	0.032463
XM_006496277.3	circRNA_3239|Chr19:45635735_45640521_-	0.849008883	0.032476
NM_013459.3	circRNA_4728|Chr5:97027831_97045167_+	0.848971731	0.032492
XM_006538224.1	circRNA_3832|Chr2:169883526_169886459_+	0.848971346	0.032492
XM_006538374.1	circRNA_3276|Chr19:60563162_60564294_-	0.848923935	0.032512
NM_010450.3	circRNA_4728|Chr5:97027831_97045167_+	0.848826284	0.032553
NM_001285867.1	circRNA_0537|Chr10:43393871_43395692_+	0.848813899	0.032558
NM_007588.2	circRNA_3425|Chr2:37624173_37627526_-	0.84876835	0.032577
NM_007588.2	circRNA_3607|Chr2:119057281_119064097_+	0.84876835	0.032577
XM_006520023.2	circRNA_4728|Chr5:97027831_97045167_+	0.848752681	0.032584
XM_006523711.3	circRNA_2951|Chr18:39120119_39150118_+	0.84870459	0.032604
XM_006529727.2	circRNA_0845|Chr11:29470315_29477458_+	0.848614736	0.032642
NM_178184.2	circRNA_5311|Chr6:145147636_145149357_+	0.848509941	0.032686
NM_010742.1	circRNA_3702|Chr2:143830084_143832057_-	0.848480465	0.032698
NM_019732.2	circRNA_0845|Chr11:29470315_29477458_+	0.848479529	0.032698
NM_015814.2	circRNA_4728|Chr5:97027831_97045167_+	0.848455079	0.032709
XM_006514077.1	circRNA_5990|Chr9:3475483_3477962_+	0.848454264	0.032709
XM_017320093.1	circRNA_4055|Chr3:126796838_126798280_+	0.848443191	0.032714
XM_006532232.3	circRNA_0845|Chr11:29470315_29477458_+	0.848442162	0.032714
XM_011243586.2	circRNA_0501|Chr10:25283893_25289730_-	0.848442124	0.032714
XM_011243586.2	circRNA_6109|Chr9:57056714_57057861_+	0.848442124	0.032714
NM_175520.4	circRNA_0913|Chr11:54005394_54014456_-	0.848413258	0.032726
NM_028804.1	circRNA_0157|Chr1:66801049_66802168_-	0.848379334	0.03274
XM_006527321.2	circRNA_3155|Chr19:25061108_25065459_+	0.848356192	0.03275
XM_006527221.3	circRNA_3276|Chr19:60563162_60564294_-	0.848328587	0.032762
NM_011082.3	circRNA_3702|Chr2:143830084_143832057_-	0.848243432	0.032798
XM_006525028.2	circRNA_6446|ChrX:42217416_42250037_+	0.848206069	0.032813
XM_011248193.2	circRNA_3832|Chr2:169883526_169886459_+	0.848150334	0.032837
NM_009930.2	circRNA_2951|Chr18:39120119_39150118_+	0.848135313	0.032843
XM_006509531.3	circRNA_4055|Chr3:126796838_126798280_+	0.848133409	0.032844
NM_011581.3	circRNA_2951|Chr18:39120119_39150118_+	0.84811765	0.032851
XM_011250776.2	circRNA_5311|Chr6:145147636_145149357_+	0.848088083	0.032863
XM_006528842.1	circRNA_4926|Chr5:143080224_143081077_-	0.84805319	0.032878

XM_006510297.3	circRNA_4055|Chr3:126796838_126798280_+	0.848020594	0.032891
XM_006528026.2	circRNA_3832|Chr2:169883526_169886459_+	0.847987329	0.032905
XM_006532120.2	circRNA_0157|Chr1:66801049_66802168_-	0.847963357	0.032916
NM_177789.4	circRNA_5639|Chr7:132771578_132779385_-	0.847926315	0.032931
NM_133167.3	circRNA_0845|Chr11:29470315_29477458_+	0.847790044	0.032989
XM_017320093.1	circRNA_0501|Chr10:25283893_25289730_-	0.847646268	0.033049
XM_017320093.1	circRNA_6109|Chr9:57056714_57057861_+	0.847646268	0.033049
NM_001320077.1	circRNA_4987|Chr6:4529068_4531010_+	0.847624789	0.033058
NM_009332.3	circRNA_6390|Chr9:121727316_121731565_+	0.847620374	0.03306
XM_006496884.3	circRNA_6140|Chr9:61935380_61937535_-	0.847590349	0.033073
NM_178692.3	circRNA_0020|Chr1:10315205_10321709_+	0.847589392	0.033073
XM_006506624.2	circRNA_0376|Chr1:172173943_172187460_+	0.847579535	0.033077
NM_145141.2	circRNA_4728|Chr5:97027831_97045167_+	0.847569152	0.033082
NM_001271580.1	circRNA_6446|ChrX:42217416_42250037_+	0.847565602	0.033083
NM_029274.2	circRNA_0537|Chr10:43393871_43395692_+	0.847539946	0.033094
NM_178692.3	circRNA_6446|ChrX:42217416_42250037_+	0.847404708	0.033151
NM_008204.2	circRNA_1632|Chr13:42055400_42055542_+	0.847372558	0.033165
XM_006509531.3	circRNA_0501|Chr10:25283893_25289730_-	0.847298381	0.033196
XM_006509531.3	circRNA_6109|Chr9:57056714_57057861_+	0.847298381	0.033196
NM_028973.2	circRNA_0376|Chr1:172173943_172187460_+	0.847277027	0.033205
NM_001167908.1	circRNA_0845|Chr11:29470315_29477458_+	0.847153137	0.033258
NM_133643.4	circRNA_3155|Chr19:25061108_25065459_+	0.847041553	0.033305
NM_027293.1	circRNA_0537|Chr10:43393871_43395692_+	0.847021757	0.033313
NM_001190379.1	circRNA_3832|Chr2:169883526_169886459_+	0.846991523	0.033326
NM_016982.2	circRNA_6140|Chr9:61935380_61937535_-	0.84698185	0.03333
XM_011243586.2	circRNA_3155|Chr19:25061108_25065459_+	0.84691109	0.03336
NM_025288.2	circRNA_3155|Chr19:25061108_25065459_+	0.846889645	0.03337
XM_006525075.3	circRNA_0157|Chr1:66801049_66802168_-	0.846867938	0.033379
XM_006540566.1	circRNA_0962|Chr11:67194348_67220998_+	0.846863466	0.033381
NM_001320077.1	circRNA_2951|Chr18:39120119_39150118_+	0.846854334	0.033384
XM_017322211.1	circRNA_3832|Chr2:169883526_169886459_+	0.846815517	0.033401
NM_001243916.1	circRNA_3702|Chr2:143830084_143832057_-	0.846789063	0.033412
NM_010809.2	circRNA_3276|Chr19:60563162_60564294_-	0.846749457	0.033429
NM_026979.5	circRNA_0157|Chr1:66801049_66802168_-	0.846739078	0.033433
NM_001080979.1	circRNA_0962|Chr11:67194348_67220998_+	0.846718124	0.033442
NM_133871.2	circRNA_4487|Chr4:149156607_149161694_-	0.846594019	0.033495
NM_008277.2	circRNA_0611|Chr10:75274017_75279961_+	0.846515126	0.033529
XM_006516224.3	circRNA_0961|Chr11:67192403_67254873_+	0.846495388	0.033537
NM_001033445.2	circRNA_2313|Chr15:93452117_93465245_+	0.846488949	0.03354
NM_009199.2	circRNA_1980|Chr14:50951404_50963869_+	0.846481759	0.033543
NM_145141.2	circRNA_0376|Chr1:172173943_172187460_+	0.846450636	0.033556
NM_173437.2	circRNA_0537|Chr10:43393871_43395692_+	0.846364589	0.033593
NM_177578.4	circRNA_5639|Chr7:132771578_132779385_-	0.846339814	0.033603
XM_006537815.3	circRNA_5811|Chr8:71992832_71998295_-	0.846283859	0.033627
XM_017318541.1	circRNA_0501|Chr10:25283893_25289730_-	0.846263568	0.033636
XM_017318541.1	circRNA_6109|Chr9:57056714_57057861_+	0.846263568	0.033636
NM_001159407.1	circRNA_2888|Chr18:21010677_21020999_+	0.846258473	0.033638
XM_006504940.3	circRNA_4987|Chr6:4529068_4531010_+	0.846249511	0.033642
NM_032400.2	circRNA_0020|Chr1:10315205_10321709_+	0.84620853	0.033659
NM_013743.2	circRNA_0537|Chr10:43393871_43395692_+	0.84618161	0.03367
NM_181547.3	circRNA_6140|Chr9:61935380_61937535_-	0.846055896	0.033724
NM_177789.4	circRNA_0501|Chr10:25283893_25289730_-	0.846038908	0.033731
NM_177789.4	circRNA_6109|Chr9:57056714_57057861_+	0.846038908	0.033731
NM_175271.4	circRNA_3276|Chr19:60563162_60564294_-	0.84599537	0.03375
XM_006534520.3	circRNA_0537|Chr10:43393871_43395692_+	0.845986442	0.033754
XM_017322211.1	circRNA_0537|Chr10:43393871_43395692_+	0.845909124	0.033787
NM_009645.2	circRNA_4662|Chr5:65301933_65303208_-	0.845887219	0.033796
XM_006521428.3	circRNA_0157|Chr1:66801049_66802168_-	0.84580603	0.033831
XM_006505757.3	circRNA_0376|Chr1:172173943_172187460_+	0.845801606	0.033833

NM_010450.3	circRNA_0501|Chr10:25283893_25289730_-	0.845801031	0.033833
NM_010450.3	circRNA_6109|Chr9:57056714_57057861_+	0.845801031	0.033833
XM_006533569.3	circRNA_0845|Chr11:29470315_29477458_+	0.845712457	0.033871
XM_006529727.2	circRNA_5074|Chr6:40685277_40747150_+	0.845619397	0.03391
NM_001164593.1	circRNA_0501|Chr10:25283893_25289730_-	0.845530767	0.033948
NM_001164593.1	circRNA_6109|Chr9:57056714_57057861_+	0.845530767	0.033948
NM_001002896.2	circRNA_2951|Chr18:39120119_39150118_+	0.845475621	0.033972
XM_006530186.3	circRNA_3832|Chr2:169883526_169886459_+	0.845441278	0.033987
NM_009332.3	circRNA_0961|Chr11:67192403_67254873_+	0.845418896	0.033996
XM_011241322.1	circRNA_5811|Chr8:71992832_71998295_-	0.845275141	0.034058
NM_009127.4	circRNA_4728|Chr5:97027831_97045167_+	0.845258662	0.034065
NM_009675.2	circRNA_1632|Chr13:42055400_42055542_+	0.845230253	0.034077
NM_007641.5	circRNA_2951|Chr18:39120119_39150118_+	0.845202244	0.034089
NM_001301354.1	circRNA_4987|Chr6:4529068_4531010_+	0.845175178	0.0341
XM_006541047.3	circRNA_6446|ChrX:42217416_42250037_+	0.84509106	0.034137
XM_006511221.3	circRNA_6390|Chr9:121727316_121731565_+	0.845089266	0.034137
XM_017315734.1	circRNA_3954|Chr3:88346445_88349444_+	0.845085511	0.034139
NM_015744.4	circRNA_2888|Chr18:21010677_21020999_+	0.845081812	0.03414
XM_006503080.3	circRNA_3954|Chr3:88346445_88349444_+	0.845060282	0.03415
NM_011854.2	circRNA_5074|Chr6:40685277_40747150_+	0.845020169	0.034167
NM_001204959.1	circRNA_5639|Chr7:132771578_132779385_-	0.845020147	0.034167
NM_027416.3	circRNA_6446|ChrX:42217416_42250037_+	0.845010788	0.034171
NM_007655.3	circRNA_0611|Chr10:75274017_75279961_+	0.844984759	0.034182
XM_006506624.2	circRNA_3239|Chr19:45635735_45640521_-	0.844860953	0.034235
NM_024406.2	circRNA_4662|Chr5:65301933_65303208_-	0.844814267	0.034255
NM_144544.2	circRNA_5311|Chr6:145147636_145149357_+	0.84480864	0.034258
NM_026979.5	circRNA_6140|Chr9:61935380_61937535_-	0.844807659	0.034258
NM_007529.2	circRNA_4728|Chr5:97027831_97045167_+	0.844797212	0.034263
XM_006505388.2	circRNA_4728|Chr5:97027831_97045167_+	0.844695336	0.034306
XM_011250217.1	circRNA_0961|Chr11:67192403_67254873_+	0.844689446	0.034309
NM_001002896.2	circRNA_0376|Chr1:172173943_172187460_+	0.844655132	0.034324
NM_016982.2	circRNA_3702|Chr2:143830084_143832057_-	0.844491222	0.034394
NM_001190325.1	circRNA_6446|ChrX:42217416_42250037_+	0.844455134	0.03441
NM_134072.1	circRNA_0501|Chr10:25283893_25289730_-	0.844417715	0.034426
NM_134072.1	circRNA_6109|Chr9:57056714_57057861_+	0.844417715	0.034426
NM_001146217.1	circRNA_0501|Chr10:25283893_25289730_-	0.844362581	0.03445
NM_001146217.1	circRNA_6109|Chr9:57056714_57057861_+	0.844362581	0.03445
XM_006521428.3	circRNA_6446|ChrX:42217416_42250037_+	0.844356706	0.034452
NM_178692.3	circRNA_4728|Chr5:97027831_97045167_+	0.844277258	0.034486
XM_006508885.1	circRNA_2951|Chr18:39120119_39150118_+	0.844212176	0.034514
NM_144799.2	circRNA_0961|Chr11:67192403_67254873_+	0.844193642	0.034522
XM_011250978.2	circRNA_6390|Chr9:121727316_121731565_+	0.844133806	0.034548
XM_001481172.6	circRNA_6140|Chr9:61935380_61937535_-	0.844105194	0.03456
XM_006522398.3	circRNA_3239|Chr19:45635735_45640521_-	0.844055579	0.034582
NM_010279.3	circRNA_5311|Chr6:145147636_145149357_+	0.84404954	0.034584
NM_008940.3	circRNA_0611|Chr10:75274017_75279961_+	0.84404688	0.034586
NM_011169.5	circRNA_0501|Chr10:25283893_25289730_-	0.843997098	0.034607
NM_011169.5	circRNA_6109|Chr9:57056714_57057861_+	0.843997098	0.034607
NM_175271.4	circRNA_6140|Chr9:61935380_61937535_-	0.843991316	0.03461
XM_006496593.3	circRNA_2951|Chr18:39120119_39150118_+	0.843909365	0.034645
XM_006510701.3	circRNA_3832|Chr2:169883526_169886459_+	0.843897982	0.03465
NM_001001178.1	circRNA_4055|Chr3:126796838_126798280_+	0.843880437	0.034657
XM_006520023.2	circRNA_5639|Chr7:132771578_132779385_-	0.843849495	0.034671
NM_031185.3	circRNA_0157|Chr1:66801049_66802168_-	0.843840735	0.034675
NM_007817.2	circRNA_0611|Chr10:75274017_75279961_+	0.843799931	0.034692
NM_028748.2	circRNA_1399|Chr12:72783683_72786789_+	0.843732936	0.034721
XM_006501237.3	circRNA_0611|Chr10:75274017_75279961_+	0.843726548	0.034724
NM_001146217.1	circRNA_3239|Chr19:45635735_45640521_-	0.843717807	0.034728
XM_006515980.3	circRNA_4662|Chr5:65301933_65303208_-	0.843674193	0.034747

XM_011244046.2	circRNA_6446|ChrX:42217416_42250037_+	0.84362473	0.034768
NM_031192.3	circRNA_3702|Chr2:143830084_143832057_-	0.843623729	0.034768
XM_011245344.2	circRNA_0962|Chr11:67194348_67220998_+	0.843572313	0.034791
NM_178676.4	circRNA_1399|Chr12:72783683_72786789_+	0.843542764	0.034803
XM_001481172.6	circRNA_0376|Chr1:172173943_172187460_+	0.843464503	0.034837
NM_001320077.1	circRNA_1632|Chr13:42055400_42055542_+	0.843391444	0.034869
NM_001190449.1	circRNA_4055|Chr3:126796838_126798280_+	0.843386104	0.034871
NM_013868.4	circRNA_0537|Chr10:43393871_43395692_+	0.843328667	0.034896
XM_006503080.3	circRNA_5074|Chr6:40685277_40747150_+	0.843320262	0.0349
XM_017312731.1	circRNA_3155|Chr19:25061108_25065459_+	0.843200803	0.034951
NM_001171187.1	circRNA_3643|Chr2:122441559_122486090_+	0.843167503	0.034966
NM_198190.1	circRNA_0962|Chr11:67194348_67220998_+	0.843122669	0.034985
NM_001252563.1	circRNA_3239|Chr19:45635735_45640521_-	0.843119084	0.034987
NM_001033445.2	circRNA_0845|Chr11:29470315_29477458_+	0.843065466	0.03501
NM_175692.3	circRNA_0157|Chr1:66801049_66802168_-	0.843065192	0.03501
XM_011244046.2	circRNA_2888|Chr18:21010677_21020999_+	0.843034558	0.035024
NM_001282961.1	circRNA_0611|Chr10:75274017_75279961_+	0.842982085	0.035046
NM_001146217.1	circRNA_4728|Chr5:97027831_97045167_+	0.842959403	0.035056
XM_006520023.2	circRNA_2951|Chr18:39120119_39150118_+	0.842935645	0.035066
NM_009514.4	circRNA_2888|Chr18:21010677_21020999_+	0.84288586	0.035088
NM_016659.3	circRNA_0501|Chr10:25283893_25289730_-	0.842867785	0.035096
NM_016659.3	circRNA_6109|Chr9:57056714_57057861_+	0.842867785	0.035096
NM_011082.3	circRNA_3155|Chr19:25061108_25065459_+	0.842843364	0.035107
NM_031192.3	circRNA_3239|Chr19:45635735_45640521_-	0.842819061	0.035117
XM_006520253.3	circRNA_0962|Chr11:67194348_67220998_+	0.842788068	0.035131
NM_001030305.2	circRNA_0157|Chr1:66801049_66802168_-	0.842678573	0.035178
NM_015814.2	circRNA_4055|Chr3:126796838_126798280_+	0.842650827	0.03519
XM_006521931.1	circRNA_3155|Chr19:25061108_25065459_+	0.842634736	0.035197
NM_007549.2	circRNA_3155|Chr19:25061108_25065459_+	0.842593382	0.035215
XM_017314870.1	circRNA_3832|Chr2:169883526_169886459_+	0.84251937	0.035247
XM_011244046.2	circRNA_5311|Chr6:145147636_145149357_+	0.842515875	0.035249
XM_006511102.2	circRNA_3702|Chr2:143830084_143832057_-	0.842476461	0.035266
NM_010279.3	circRNA_4055|Chr3:126796838_126798280_+	0.842473445	0.035267
XM_006518921.3	circRNA_1632|Chr13:42055400_42055542_+	0.842423071	0.035289
XM_006503665.3	circRNA_4055|Chr3:126796838_126798280_+	0.842385694	0.035306
NM_008872.3	circRNA_3155|Chr19:25061108_25065459_+	0.84237065	0.035312
NM_025422.4	circRNA_5639|Chr7:132771578_132779385_-	0.842354326	0.035319
NM_009514.4	circRNA_6140|Chr9:61935380_61937535_-	0.842289784	0.035347
XM_006521428.3	circRNA_1632|Chr13:42055400_42055542_+	0.842276816	0.035353
NM_001310648.1	circRNA_0157|Chr1:66801049_66802168_-	0.842263536	0.035359
NM_001167908.1	circRNA_0537|Chr10:43393871_43395692_+	0.842256071	0.035362
NM_029823.2	circRNA_0157|Chr1:66801049_66802168_-	0.842228646	0.035374
NM_001109985.1	circRNA_0962|Chr11:67194348_67220998_+	0.842128182	0.035418
NM_001029877.3	circRNA_3954|Chr3:88346445_88349444_+	0.842074449	0.035441
NM_010742.1	circRNA_6446|ChrX:42217416_42250037_+	0.842033898	0.035459
XM_006520186.2	circRNA_4055|Chr3:126796838_126798280_+	0.842030163	0.035461
NM_031185.3	circRNA_0501|Chr10:25283893_25289730_-	0.841835696	0.035546
NM_031185.3	circRNA_6109|Chr9:57056714_57057861_+	0.841835696	0.035546
XM_017316439.1	circRNA_5311|Chr6:145147636_145149357_+	0.841769763	0.035574
XM_011250776.2	circRNA_2888|Chr18:21010677_21020999_+	0.841754442	0.035581
NM_152915.1	circRNA_3702|Chr2:143830084_143832057_-	0.841738537	0.035588
XM_006506808.3	circRNA_4926|Chr5:143080224_143081077_-	0.841729238	0.035592
XM_017321602.1	circRNA_2888|Chr18:21010677_21020999_+	0.841708682	0.035601
XM_006496593.3	circRNA_0501|Chr10:25283893_25289730_-	0.841687544	0.03561
XM_006496593.3	circRNA_6109|Chr9:57056714_57057861_+	0.841687544	0.03561
XM_017314944.1	circRNA_0611|Chr10:75274017_75279961_+	0.841663458	0.035621
XM_006520186.2	circRNA_0501|Chr10:25283893_25289730_-	0.841630283	0.035635
XM_006520186.2	circRNA_6109|Chr9:57056714_57057861_+	0.841630283	0.035635
NM_145210.2	circRNA_2313|Chr15:93452117_93465245_+	0.841625268	0.035638

NM_019732.2	circRNA_5074|Chr6:40685277_40747150_+	0.841607217	0.035646
NM_177789.4	circRNA_0376|Chr1:172173943_172187460_+	0.841597591	0.03565
NM_008458.2	circRNA_5311|Chr6:145147636_145149357_+	0.841572832	0.035661
NM_020508.4	circRNA_0537|Chr10:43393871_43395692_+	0.841549443	0.035671
NM_001289782.1	circRNA_0537|Chr10:43393871_43395692_+	0.841457498	0.035711
NM_205810.4	circRNA_4728|Chr5:97027831_97045167_+	0.841395314	0.035738
NM_023716.2	circRNA_5311|Chr6:145147636_145149357_+	0.841373558	0.035748
NM_001081185.1	circRNA_0961|Chr11:67192403_67254873_+	0.84130351	0.035779
NM_025540.2	circRNA_3832|Chr2:169883526_169886459_+	0.841292957	0.035783
NM_031185.3	circRNA_2888|Chr18:21010677_21020999_+	0.841258098	0.035798
NM_019696.2	circRNA_3702|Chr2:143830084_143832057_-	0.841256002	0.035799
NM_011145.3	circRNA_3003|Chr18:67545615_67587859_-	0.841212209	0.035819
XM_006532007.3	circRNA_3643|Chr2:122441559_122486090_+	0.841187906	0.035829
XM_011250978.2	circRNA_0845|Chr11:29470315_29477458_+	0.841180756	0.035832
NM_178753.4	circRNA_4987|Chr6:4529068_4531010_+	0.84110183	0.035867
XM_006496949.3	circRNA_6140|Chr9:61935380_61937535_-	0.841099708	0.035868
NM_011145.3	circRNA_5074|Chr6:40685277_40747150_+	0.841056317	0.035887
XM_006510297.3	circRNA_3276|Chr19:60563162_60564294_-	0.840989893	0.035916
NM_025711.3	circRNA_0157|Chr1:66801049_66802168_-	0.840975608	0.035922
XM_006539074.3	circRNA_3832|Chr2:169883526_169886459_+	0.8409298	0.035942
NM_023456.3	circRNA_4728|Chr5:97027831_97045167_+	0.840910013	0.035951
NM_001318003.2	circRNA_4055|Chr3:126796838_126798280_+	0.84089581	0.035957
XM_006540596.2	circRNA_5367|Chr7:28990988_28991325_+	0.840864578	0.035971
NM_011854.2	circRNA_4487|Chr4:149156607_149161694_-	0.840849002	0.035978
XM_006534171.3	circRNA_3702|Chr2:143830084_143832057_-	0.840839964	0.035982
NM_007655.3	circRNA_4055|Chr3:126796838_126798280_+	0.840784126	0.036007
NM_177789.4	circRNA_4055|Chr3:126796838_126798280_+	0.840740587	0.036026
XM_006529456.3	circRNA_4987|Chr6:4529068_4531010_+	0.840737383	0.036027
NM_146241.2	circRNA_6446|ChrX:42217416_42250037_+	0.840686547	0.036049
NM_001252563.1	circRNA_0020|Chr1:10315205_10321709_+	0.840635635	0.036072
XM_006512296.3	circRNA_0537|Chr10:43393871_43395692_+	0.84062635	0.036076
NM_011581.3	circRNA_0501|Chr10:25283893_25289730_-	0.840623993	0.036077
NM_011581.3	circRNA_6109|Chr9:57056714_57057861_+	0.840623993	0.036077
NM_001029877.3	circRNA_0845|Chr11:29470315_29477458_+	0.840617862	0.03608
XM_006524777.3	circRNA_6140|Chr9:61935380_61937535_-	0.840608504	0.036084
XM_006541047.3	circRNA_0157|Chr1:66801049_66802168_-	0.840578392	0.036097
XM_006511007.3	circRNA_4926|Chr5:143080224_143081077_-	0.84053293	0.036117
NM_026979.5	circRNA_0611|Chr10:75274017_75279961_+	0.840516606	0.036124
NM_007641.5	circRNA_2888|Chr18:21010677_21020999_+	0.840489051	0.036136
NM_001080979.1	circRNA_3003|Chr18:67545615_67587859_-	0.84040404	0.036174
NM_172454.2	circRNA_1042|Chr11:80385854_80403408_+	0.840399076	0.036176
NM_009020.3	circRNA_3643|Chr2:122441559_122486090_+	0.840387958	0.036181
XM_006515096.2	circRNA_5074|Chr6:40685277_40747150_+	0.84036813	0.03619
NM_007655.3	circRNA_2951|Chr18:39120119_39150118_+	0.840364437	0.036191
NM_016659.3	circRNA_2951|Chr18:39120119_39150118_+	0.84035883	0.036194
XM_006532232.3	circRNA_5074|Chr6:40685277_40747150_+	0.840348637	0.036198
NM_016933.3	circRNA_0020|Chr1:10315205_10321709_+	0.840348566	0.036198
NM_001039047.1	circRNA_5074|Chr6:40685277_40747150_+	0.84033956	0.036202
NM_023456.3	circRNA_3276|Chr19:60563162_60564294_-	0.840313673	0.036214
XM_011240476.2	circRNA_0537|Chr10:43393871_43395692_+	0.840310504	0.036215
NM_172454.2	circRNA_1980|Chr14:50951404_50963869_+	0.840252676	0.03624
XM_011239969.2	circRNA_3276|Chr19:60563162_60564294_-	0.840161954	0.036281
NM_001327998.1	circRNA_2951|Chr18:39120119_39150118_+	0.840144722	0.036288
NM_011658.2	circRNA_4728|Chr5:97027831_97045167_+	0.840126706	0.036296
NM_011704.3	circRNA_5639|Chr7:132771578_132779385_-	0.840081965	0.036316
NM_001204959.1	circRNA_3702|Chr2:143830084_143832057_-	0.840077735	0.036318
XM_006518339.3	circRNA_1632|Chr13:42055400_42055542_+	0.840046781	0.036331
NM_001142804.1	circRNA_4728|Chr5:97027831_97045167_+	0.840028609	0.036339
NM_011957.2	circRNA_6390|Chr9:121727316_121731565_+	0.840020588	0.036343

XM_011246128.2	circRNA_0501|Chr10:25283893_25289730_-	0.839984184	0.036359
XM_011246128.2	circRNA_6109|Chr9:57056714_57057861_+	0.839984184	0.036359
NM_019759.2	circRNA_0501|Chr10:25283893_25289730_-	0.839918872	0.036388
NM_019759.2	circRNA_6109|Chr9:57056714_57057861_+	0.839918872	0.036388
XM_011248576.2	circRNA_0845|Chr11:29470315_29477458_+	0.839892502	0.036399
NM_013912.3	circRNA_3702|Chr2:143830084_143832057_-	0.839826219	0.036429
NM_018732.3	circRNA_3832|Chr2:169883526_169886459_+	0.839825215	0.036429
XM_006530325.3	circRNA_3832|Chr2:169883526_169886459_+	0.839738397	0.036468
XM_017321602.1	circRNA_0157|Chr1:66801049_66802168_-	0.839713049	0.036479
NM_001033221.3	circRNA_0845|Chr11:29470315_29477458_+	0.83961084	0.036524
XM_006502291.3	circRNA_4926|Chr5:143080224_143081077_-	0.839537852	0.036556
XM_006504940.3	circRNA_3643|Chr2:122441559_122486090_+	0.839479499	0.036582
XM_011239558.2	circRNA_4926|Chr5:143080224_143081077_-	0.839461942	0.03659
NM_178676.4	circRNA_3425|Chr2:37624173_37627526_-	0.839182006	0.036714
NM_178676.4	circRNA_3607|Chr2:119057281_119064097_+	0.839182006	0.036714
XM_011239558.2	circRNA_5811|Chr8:71992832_71998295_-	0.839169912	0.036719
XM_011248919.2	circRNA_3954|Chr3:88346445_88349444_+	0.839130844	0.036737
XM_006501237.3	circRNA_0020|Chr1:10315205_10321709_+	0.839065094	0.036766
XM_006520023.2	circRNA_0501|Chr10:25283893_25289730_-	0.83904942	0.036773
XM_006520023.2	circRNA_6109|Chr9:57056714_57057861_+	0.83904942	0.036773
NM_007881.4	circRNA_4926|Chr5:143080224_143081077_-	0.839046251	0.036774
NM_011044.2	circRNA_4728|Chr5:97027831_97045167_+	0.839018709	0.036787
XM_006537508.2	circRNA_0157|Chr1:66801049_66802168_-	0.838998246	0.036796
NM_175692.3	circRNA_2951|Chr18:39120119_39150118_+	0.838967846	0.036809
NM_008264.1	circRNA_6140|Chr9:61935380_61937535_-	0.83893245	0.036825
NM_001162950.1	circRNA_5990|Chr9:3475483_3477962_+	0.838924081	0.036829
NM_008277.2	circRNA_2951|Chr18:39120119_39150118_+	0.838900316	0.036839
XM_017317978.1	circRNA_0020|Chr1:10315205_10321709_+	0.83889959	0.036839
XM_006496593.3	circRNA_6140|Chr9:61935380_61937535_-	0.838895783	0.036841
NM_010714.3	circRNA_2951|Chr18:39120119_39150118_+	0.83889319	0.036842
NM_008479.2	circRNA_4487|Chr4:149156607_149161694_-	0.838832602	0.036869
NM_009722.3	circRNA_3832|Chr2:169883526_169886459_+	0.838771793	0.036896
NM_009243.4	circRNA_3003|Chr18:67545615_67587859_-	0.8387479	0.036907
NM_138955.3	circRNA_0961|Chr11:67192403_67254873_+	0.83873443	0.036913
NM_177743.5	circRNA_0611|Chr10:75274017_75279961_+	0.838728956	0.036915
NM_025288.2	circRNA_6140|Chr9:61935380_61937535_-	0.838728855	0.036915
XM_006527452.3	circRNA_5311|Chr6:145147636_145149357_+	0.838693718	0.036931
NM_001167908.1	circRNA_3832|Chr2:169883526_169886459_+	0.838693334	0.036931
NM_001161541.1	circRNA_0157|Chr1:66801049_66802168_-	0.838631419	0.036959
NM_010279.3	circRNA_0020|Chr1:10315205_10321709_+	0.838624448	0.036962
XM_006540138.3	circRNA_0962|Chr11:67194348_67220998_+	0.838576335	0.036983
NM_001099314.1	circRNA_5074|Chr6:40685277_40747150_+	0.838495331	0.037019
NM_007792.4	circRNA_0501|Chr10:25283893_25289730_-	0.838482238	0.037025
NM_007792.4	circRNA_6109|Chr9:57056714_57057861_+	0.838482238	0.037025
XM_006530353.3	circRNA_4926|Chr5:143080224_143081077_-	0.838481969	0.037025
XM_011246128.2	circRNA_0611|Chr10:75274017_75279961_+	0.838468987	0.037031
XM_006508885.1	circRNA_6446|ChrX:42217416_42250037_+	0.838452582	0.037038
XM_011238916.2	circRNA_0962|Chr11:67194348_67220998_+	0.838449742	0.03704
NM_177789.4	circRNA_6446|ChrX:42217416_42250037_+	0.83842647	0.03705
NM_175260.2	circRNA_5074|Chr6:40685277_40747150_+	0.838321706	0.037097
NM_144544.2	circRNA_3702|Chr2:143830084_143832057_-	0.838317902	0.037098
XM_006506340.3	circRNA_5074|Chr6:40685277_40747150_+	0.838317792	0.037098
NM_028804.1	circRNA_6446|ChrX:42217416_42250037_+	0.838286404	0.037112
NM_025422.4	circRNA_0376|Chr1:172173943_172187460_+	0.838251866	0.037128
NM_009605.4	circRNA_4662|Chr5:65301933_65303208_-	0.838224374	0.03714
NM_001077403.1	circRNA_5074|Chr6:40685277_40747150_+	0.838192781	0.037154
XM_006525075.3	circRNA_4662|Chr5:65301933_65303208_-	0.83816217	0.037168
XM_011240779.2	circRNA_0611|Chr10:75274017_75279961_+	0.83815254	0.037172
XM_001481172.6	circRNA_3702|Chr2:143830084_143832057_-	0.838107952	0.037192

NM_199304.1	circRNA_3425|Chr2:37624173_37627526_-	0.838012452	0.037235
NM_199304.1	circRNA_3607|Chr2:119057281_119064097_+	0.838012452	0.037235
NM_001310648.1	circRNA_4728|Chr5:97027831_97045167_+	0.837822125	0.03732
XM_006513165.1	circRNA_0611|Chr10:75274017_75279961_+	0.837812134	0.037324
XM_017320735.1	circRNA_3702|Chr2:143830084_143832057_-	0.83781122	0.037325
NM_028748.2	circRNA_3425|Chr2:37624173_37627526_-	0.837807046	0.037326
NM_028748.2	circRNA_3607|Chr2:119057281_119064097_+	0.837807046	0.037326
NM_010090.2	circRNA_0501|Chr10:25283893_25289730_-	0.837781327	0.037338
NM_010090.2	circRNA_6109|Chr9:57056714_57057861_+	0.837781327	0.037338
NM_030206.4	circRNA_3155|Chr19:25061108_25065459_+	0.837722277	0.037364
XM_006501237.3	circRNA_0501|Chr10:25283893_25289730_-	0.837698132	0.037375
XM_006501237.3	circRNA_6109|Chr9:57056714_57057861_+	0.837698132	0.037375
NM_152915.1	circRNA_0376|Chr1:172173943_172187460_+	0.837657834	0.037393
NM_010608.2	circRNA_0501|Chr10:25283893_25289730_-	0.837613709	0.037413
NM_010608.2	circRNA_6109|Chr9:57056714_57057861_+	0.837613709	0.037413
XM_017314944.1	circRNA_4662|Chr5:65301933_65303208_-	0.837603087	0.037418
XM_011240779.2	circRNA_3276|Chr19:60563162_60564294_-	0.837577248	0.037429
NM_001302471.1	circRNA_0845|Chr11:29470315_29477458_+	0.837552784	0.03744
NM_009930.2	circRNA_4728|Chr5:97027831_97045167_+	0.837547689	0.037443
NM_001002896.2	circRNA_0611|Chr10:75274017_75279961_+	0.837491362	0.037468
NM_010608.2	circRNA_2888|Chr18:21010677_21020999_+	0.837424211	0.037498
XM_006499444.3	circRNA_3003|Chr18:67545615_67587859_-	0.837310288	0.037549
NM_001110322.1	circRNA_3276|Chr19:60563162_60564294_-	0.837287889	0.037559
NM_001013784.1	circRNA_3954|Chr3:88346445_88349444_+	0.837284199	0.037561
NM_007655.3	circRNA_4987|Chr6:4529068_4531010_+	0.837240552	0.03758
NM_001190325.1	circRNA_0501|Chr10:25283893_25289730_-	0.837199485	0.037599
NM_001190325.1	circRNA_6109|Chr9:57056714_57057861_+	0.837199485	0.037599
XM_011244046.2	circRNA_3155|Chr19:25061108_25065459_+	0.837188234	0.037604
XM_006540596.2	circRNA_0611|Chr10:75274017_75279961_+	0.837147681	0.037622
XM_017318541.1	circRNA_4728|Chr5:97027831_97045167_+	0.837066946	0.037658
NM_001303431.1	circRNA_0020|Chr1:10315205_10321709_+	0.837026932	0.037676
NM_009824.2	circRNA_0537|Chr10:43393871_43395692_+	0.837008412	0.037684
NM_145635.2	circRNA_0376|Chr1:172173943_172187460_+	0.836968551	0.037702
XM_011246309.2	circRNA_0157|Chr1:66801049_66802168_-	0.836940509	0.037715
NM_027455.3	circRNA_4662|Chr5:65301933_65303208_-	0.836911107	0.037728
NM_013459.3	circRNA_0020|Chr1:10315205_10321709_+	0.83689169	0.037737
NM_001317365.1	circRNA_5990|Chr9:3475483_3477962_+	0.836874555	0.037744
NM_001190325.1	circRNA_0020|Chr1:10315205_10321709_+	0.836869119	0.037747
XM_006496884.3	circRNA_0157|Chr1:66801049_66802168_-	0.836843791	0.037758
NM_001244031.1	circRNA_0962|Chr11:67194348_67220998_+	0.836842583	0.037759
XM_006533479.2	circRNA_6140|Chr9:61935380_61937535_-	0.836839247	0.03776
XM_017315313.1	circRNA_0157|Chr1:66801049_66802168_-	0.836831183	0.037764
XM_006520023.2	circRNA_4662|Chr5:65301933_65303208_-	0.836819281	0.037769
NM_001204201.1	circRNA_0611|Chr10:75274017_75279961_+	0.836811434	0.037773
NM_010133.2	circRNA_6412|ChrMT:13917_14141_-	0.836790683	0.037782
XM_006530294.3	circRNA_0537|Chr10:43393871_43395692_+	0.836742767	0.037804
NM_008524.2	circRNA_0376|Chr1:172173943_172187460_+	0.836728705	0.03781
NM_009243.4	circRNA_0845|Chr11:29470315_29477458_+	0.83665406	0.037844
NM_001146217.1	circRNA_2951|Chr18:39120119_39150118_+	0.836636671	0.037851
NM_010858.4	circRNA_4728|Chr5:97027831_97045167_+	0.836633339	0.037853
NM_152915.1	circRNA_0501|Chr10:25283893_25289730_-	0.836593264	0.037871
NM_152915.1	circRNA_6109|Chr9:57056714_57057861_+	0.836593264	0.037871
NM_199304.1	circRNA_1399|Chr12:72783683_72786789_+	0.836591202	0.037872
XM_006530585.3	circRNA_3954|Chr3:88346445_88349444_+	0.836588355	0.037873
NM_145741.2	circRNA_3239|Chr19:45635735_45640521_-	0.836471104	0.037926
NM_001033221.3	circRNA_5074|Chr6:40685277_40747150_+	0.836424199	0.037947
XM_006522398.3	circRNA_0611|Chr10:75274017_75279961_+	0.836405705	0.037955
XM_006540566.1	circRNA_0845|Chr11:29470315_29477458_+	0.836371051	0.037971
NM_011430.3	circRNA_0376|Chr1:172173943_172187460_+	0.836354681	0.037978

XM_006527321.2	circRNA_0157|Chr1:66801049_66802168_-	0.836319307	0.037994
NM_007817.2	circRNA_4055|Chr3:126796838_126798280_+	0.836316754	0.037996
XM_006527321.2	circRNA_2951|Chr18:39120119_39150118_+	0.836283765	0.03801
NM_013415.5	circRNA_3832|Chr2:169883526_169886459_+	0.836279856	0.038012
XM_011242311.2	circRNA_3832|Chr2:169883526_169886459_+	0.836258752	0.038022
XM_017318541.1	circRNA_3239|Chr19:45635735_45640521_-	0.836231571	0.038034
NM_178782.4	circRNA_0845|Chr11:29470315_29477458_+	0.836143426	0.038074
XM_011238891.1	circRNA_4728|Chr5:97027831_97045167_+	0.836136515	0.038077
NM_011854.2	circRNA_0537|Chr10:43393871_43395692_+	0.83609514	0.038096
NM_009020.3	circRNA_5367|Chr7:28990988_28991325_+	0.83608398	0.038101
NM_175271.4	circRNA_0611|Chr10:75274017_75279961_+	0.835990798	0.038143
XM_006521931.1	circRNA_3239|Chr19:45635735_45640521_-	0.83590636	0.038181
NM_001303431.1	circRNA_4662|Chr5:65301933_65303208_-	0.835892085	0.038187
XM_006510297.3	circRNA_5311|Chr6:145147636_145149357_+	0.835883369	0.038191
NM_172862.3	circRNA_0962|Chr11:67194348_67220998_+	0.835880626	0.038192
NM_001318003.2	circRNA_5311|Chr6:145147636_145149357_+	0.835880048	0.038193
NM_008204.2	circRNA_4987|Chr6:4529068_4531010_+	0.835871351	0.038197
XM_011241948.2	circRNA_0501|Chr10:25283893_25289730_-	0.835822262	0.038219
XM_011241948.2	circRNA_6109|Chr9:57056714_57057861_+	0.835822262	0.038219
NM_010140.3	circRNA_0020|Chr1:10315205_10321709_+	0.835805598	0.038226
NM_015744.4	circRNA_5311|Chr6:145147636_145149357_+	0.835754575	0.038249
NM_199304.1	circRNA_4487|Chr4:149156607_149161694_-	0.835750217	0.038251
NM_023670.3	circRNA_6446|ChrX:42217416_42250037_+	0.835719554	0.038265
NM_008966.3	circRNA_5639|Chr7:132771578_132779385_-	0.83568405	0.038281
XM_006521931.1	circRNA_5639|Chr7:132771578_132779385_-	0.835682212	0.038282
XM_017316944.1	circRNA_0611|Chr10:75274017_75279961_+	0.83568131	0.038283
NM_001190449.1	circRNA_2888|Chr18:21010677_21020999_+	0.835667499	0.038289
NM_015814.2	circRNA_3276|Chr19:60563162_60564294_-	0.835628457	0.038307
XM_011240149.1	circRNA_0961|Chr11:67192403_67254873_+	0.835619471	0.038311
NM_007529.2	circRNA_3702|Chr2:143830084_143832057_-	0.835615486	0.038312
NM_001310636.1	circRNA_0611|Chr10:75274017_75279961_+	0.835605159	0.038317
NM_010090.2	circRNA_5311|Chr6:145147636_145149357_+	0.835600559	0.038319
XM_006532007.3	circRNA_0157|Chr1:66801049_66802168_-	0.835591511	0.038323
XM_006533569.3	circRNA_3425|Chr2:37624173_37627526_-	0.835410829	0.038405
XM_006533569.3	circRNA_3607|Chr2:119057281_119064097_+	0.835410829	0.038405
XM_006527321.2	circRNA_0501|Chr10:25283893_25289730_-	0.835365891	0.038425
XM_006527321.2	circRNA_6109|Chr9:57056714_57057861_+	0.835365891	0.038425
XM_006508885.1	circRNA_3702|Chr2:143830084_143832057_-	0.835330247	0.038442
XM_006525028.2	circRNA_3239|Chr19:45635735_45640521_-	0.835309692	0.038451
XM_006538224.1	circRNA_0537|Chr10:43393871_43395692_+	0.835305245	0.038453
NM_011854.2	circRNA_3425|Chr2:37624173_37627526_-	0.835210507	0.038496
NM_011854.2	circRNA_3607|Chr2:119057281_119064097_+	0.835210507	0.038496
XM_011247208.1	circRNA_3832|Chr2:169883526_169886459_+	0.83519759	0.038502
NM_001007570.2	circRNA_6390|Chr9:121727316_121731565_+	0.835175572	0.038512
NM_019866.1	circRNA_4055|Chr3:126796838_126798280_+	0.835034257	0.038576
NM_007792.4	circRNA_2951|Chr18:39120119_39150118_+	0.83499676	0.038593
NM_031185.3	circRNA_4987|Chr6:4529068_4531010_+	0.834984232	0.038599
NM_001033630.1	circRNA_6412|ChrMT:13917_14141_-	0.83496223	0.038609
XM_017320223.1	circRNA_0962|Chr11:67194348_67220998_+	0.834910219	0.038632
NM_031183.2	circRNA_3276|Chr19:60563162_60564294_-	0.834851386	0.038659
XM_017320093.1	circRNA_2888|Chr18:21010677_21020999_+	0.834850852	0.038659
NM_205810.4	circRNA_3155|Chr19:25061108_25065459_+	0.834822191	0.038672
XM_006525028.2	circRNA_4055|Chr3:126796838_126798280_+	0.834795971	0.038684
XM_011248193.2	circRNA_0537|Chr10:43393871_43395692_+	0.834724	0.038717
NM_019397.3	circRNA_0501|Chr10:25283893_25289730_-	0.834701179	0.038727
NM_019397.3	circRNA_6109|Chr9:57056714_57057861_+	0.834701179	0.038727
NM_001317365.1	circRNA_1042|Chr11:80385854_80403408_+	0.83466697	0.038743
NM_009930.2	circRNA_0501|Chr10:25283893_25289730_-	0.834657714	0.038747
NM_009930.2	circRNA_6109|Chr9:57056714_57057861_+	0.834657714	0.038747

NM_016933.3	circRNA_0157|Chr1:66801049_66802168_-	0.834624496	0.038762
XM_006496593.3	circRNA_2888|Chr18:21010677_21020999_+	0.834616876	0.038766
NM_008423.2	circRNA_5990|Chr9:3475483_3477962_+	0.834610756	0.038768
NM_011854.2	circRNA_6390|Chr9:121727316_121731565_+	0.834529809	0.038805
NM_001081185.1	circRNA_0537|Chr10:43393871_43395692_+	0.834521872	0.038809
NM_177743.5	circRNA_0376|Chr1:172173943_172187460_+	0.834489523	0.038824
NM_010181.2	circRNA_6390|Chr9:121727316_121731565_+	0.834399874	0.038864
XM_006520437.2	circRNA_0376|Chr1:172173943_172187460_+	0.834347128	0.038888
XM_017316736.1	circRNA_3239|Chr19:45635735_45640521_-	0.834271793	0.038923
NM_028472.2	circRNA_0376|Chr1:172173943_172187460_+	0.834252434	0.038932
NM_146241.2	circRNA_3239|Chr19:45635735_45640521_-	0.834251846	0.038932
NM_153104.3	circRNA_3832|Chr2:169883526_169886459_+	0.834245922	0.038935
NM_019866.1	circRNA_6140|Chr9:61935380_61937535_-	0.834210055	0.038951
NM_007792.4	circRNA_5639|Chr7:132771578_132779385_-	0.834144648	0.038981
NM_008012.1	circRNA_0020|Chr1:10315205_10321709_+	0.834109383	0.038997
NM_001110009.2	circRNA_3239|Chr19:45635735_45640521_-	0.834103104	0.039
XM_006510297.3	circRNA_0611|Chr10:75274017_75279961_+	0.834097469	0.039002
NM_001291145.1	circRNA_5639|Chr7:132771578_132779385_-	0.834035658	0.039031
NM_025422.4	circRNA_5311|Chr6:145147636_145149357_+	0.83399025	0.039051
NM_011145.3	circRNA_3425|Chr2:37624173_37627526_-	0.833891908	0.039096
NM_011145.3	circRNA_3607|Chr2:119057281_119064097_+	0.833891908	0.039096
NM_133643.4	circRNA_0501|Chr10:25283893_25289730_-	0.833891646	0.039096
NM_133643.4	circRNA_6109|Chr9:57056714_57057861_+	0.833891646	0.039096
NM_024283.3	circRNA_0376|Chr1:172173943_172187460_+	0.833862877	0.039109
NM_011145.3	circRNA_1399|Chr12:72783683_72786789_+	0.833843584	0.039118
NM_013415.5	circRNA_0961|Chr11:67192403_67254873_+	0.833838341	0.039121
XM_006530131.1	circRNA_4926|Chr5:143080224_143081077_-	0.833817767	0.03913
NM_146063.1	circRNA_0611|Chr10:75274017_75279961_+	0.833787501	0.039144
NM_009135.2	circRNA_5367|Chr7:28990988_28991325_+	0.833766938	0.039153
NM_001164593.1	circRNA_0376|Chr1:172173943_172187460_+	0.833686222	0.03919
XM_017316676.1	circRNA_0961|Chr11:67192403_67254873_+	0.83367903	0.039194
NM_009127.4	circRNA_0611|Chr10:75274017_75279961_+	0.833663539	0.039201
NM_009930.2	circRNA_0611|Chr10:75274017_75279961_+	0.833619175	0.039221
NM_001290993.1	circRNA_4987|Chr6:4529068_4531010_+	0.83355772	0.039249
XM_011250776.2	circRNA_3276|Chr19:60563162_60564294_-	0.833542898	0.039256
NM_001190448.1	circRNA_0611|Chr10:75274017_75279961_+	0.833542551	0.039256
NM_001313939.1	circRNA_0020|Chr1:10315205_10321709_+	0.833533937	0.03926
NM_008760.4	circRNA_0611|Chr10:75274017_75279961_+	0.833454634	0.039296
NM_001291930.1	circRNA_6140|Chr9:61935380_61937535_-	0.833442005	0.039302
XM_017322026.1	circRNA_2888|Chr18:21010677_21020999_+	0.833441618	0.039302
NM_023670.3	circRNA_4728|Chr5:97027831_97045167_+	0.833356336	0.039341
XM_011244488.2	circRNA_3702|Chr2:143830084_143832057_-	0.833343335	0.039347
NM_020025.4	circRNA_4662|Chr5:65301933_65303208_-	0.833311356	0.039362
NM_001171187.1	circRNA_0157|Chr1:66801049_66802168_-	0.833302392	0.039366
XM_017314944.1	circRNA_0376|Chr1:172173943_172187460_+	0.833259896	0.039386
XM_006521931.1	circRNA_0020|Chr1:10315205_10321709_+	0.833247777	0.039391
NM_031185.3	circRNA_6140|Chr9:61935380_61937535_-	0.833207393	0.03941
XM_006506152.3	circRNA_0537|Chr10:43393871_43395692_+	0.833198608	0.039414
NM_001142804.1	circRNA_4055|Chr3:126796838_126798280_+	0.833181503	0.039421
XM_006501829.3	circRNA_3832|Chr2:169883526_169886459_+	0.83317573	0.039424
XM_017320093.1	circRNA_3239|Chr19:45635735_45640521_-	0.83315533	0.039433
NM_011854.2	circRNA_1399|Chr12:72783683_72786789_+	0.833153718	0.039434
NM_013912.3	circRNA_3276|Chr19:60563162_60564294_-	0.833129815	0.039445
NM_008872.3	circRNA_6446|ChrX:42217416_42250037_+	0.833123442	0.039448
NM_013906.3	circRNA_1980|Chr14:50951404_50963869_+	0.833107095	0.039456
NM_027416.3	circRNA_0501|Chr10:25283893_25289730_-	0.833085861	0.039465
NM_027416.3	circRNA_6109|Chr9:57056714_57057861_+	0.833085861	0.039465
NM_145741.2	circRNA_1632|Chr13:42055400_42055542_+	0.833081359	0.039467
NM_016809.6	circRNA_0157|Chr1:66801049_66802168_-	0.833064696	0.039475

XM_006530131.1	circRNA_5074|Chr6:40685277_40747150_+	0.833025942	0.039493
NM_013459.3	circRNA_2951|Chr18:39120119_39150118_+	0.833007953	0.039501
XM_006541047.3	circRNA_2951|Chr18:39120119_39150118_+	0.832997369	0.039506
XM_006524407.3	circRNA_4926|Chr5:143080224_143081077_-	0.832989791	0.039509
NM_001291483.1	circRNA_2951|Chr18:39120119_39150118_+	0.832966035	0.03952
XM_006510297.3	circRNA_3239|Chr19:45635735_45640521_-	0.832930894	0.039537
XM_006528026.2	circRNA_0537|Chr10:43393871_43395692_+	0.832906081	0.039548
NM_001327998.1	circRNA_2888|Chr18:21010677_21020999_+	0.832890349	0.039555
XM_017316944.1	circRNA_3239|Chr19:45635735_45640521_-	0.832885036	0.039558
NM_019759.2	circRNA_4662|Chr5:65301933_65303208_-	0.8328688	0.039565
NM_001243916.1	circRNA_4055|Chr3:126796838_126798280_+	0.83285863	0.03957
XM_006522506.3	circRNA_0376|Chr1:172173943_172187460_+	0.832813708	0.03959
XM_011248602.2	circRNA_0961|Chr11:67192403_67254873_+	0.832743659	0.039623
XM_006530325.3	circRNA_6390|Chr9:121727316_121731565_+	0.832717281	0.039635
XM_006537836.1	circRNA_0913|Chr11:54005394_54014456_-	0.832674428	0.039654
XM_006505015.3	circRNA_1980|Chr14:50951404_50963869_+	0.832645293	0.039668
XM_006533539.3	circRNA_3643|Chr2:122441559_122486090_+	0.832615748	0.039681
XM_011244235.2	circRNA_3276|Chr19:60563162_60564294_-	0.832569854	0.039703
NM_133643.4	circRNA_2888|Chr18:21010677_21020999_+	0.83250586	0.039732
XM_006506086.2	circRNA_3702|Chr2:143830084_143832057_-	0.832505215	0.039732
XM_011246479.2	circRNA_3954|Chr3:88346445_88349444_+	0.832486858	0.039741
NM_178753.4	circRNA_1632|Chr13:42055400_42055542_+	0.832471513	0.039748
NM_001033149.3	circRNA_0537|Chr10:43393871_43395692_+	0.832453083	0.039756
NM_178676.4	circRNA_0537|Chr10:43393871_43395692_+	0.832375515	0.039792
NM_029844.3	circRNA_5639|Chr7:132771578_132779385_-	0.832367516	0.039796
XM_006505757.3	circRNA_3702|Chr2:143830084_143832057_-	0.832364594	0.039797
NM_008012.1	circRNA_0157|Chr1:66801049_66802168_-	0.832310889	0.039822
NM_001082547.1	circRNA_3239|Chr19:45635735_45640521_-	0.832302055	0.039826
XM_011238891.1	circRNA_2951|Chr18:39120119_39150118_+	0.832259034	0.039846
NM_008872.3	circRNA_4987|Chr6:4529068_4531010_+	0.832256014	0.039847
NM_009243.4	circRNA_0537|Chr10:43393871_43395692_+	0.832247583	0.039851
XM_011239969.2	circRNA_3239|Chr19:45635735_45640521_-	0.832177954	0.039883
NM_001195084.1	circRNA_0611|Chr10:75274017_75279961_+	0.832155072	0.039894
XM_006503323.3	circRNA_6140|Chr9:61935380_61937535_-	0.832114844	0.039912
NM_001082547.1	circRNA_3155|Chr19:25061108_25065459_+	0.832069136	0.039933
NM_001286743.1	circRNA_3832|Chr2:169883526_169886459_+	0.832059528	0.039938
NM_007529.2	circRNA_5311|Chr6:145147636_145149357_+	0.832033884	0.03995
XM_006505015.3	circRNA_6412|ChrMT:13917_14141_-	0.832033277	0.03995
NM_009243.4	circRNA_3832|Chr2:169883526_169886459_+	0.832016771	0.039957
NM_010809.2	circRNA_3239|Chr19:45635735_45640521_-	0.832008735	0.039961
NM_001291145.1	circRNA_5367|Chr7:28990988_28991325_+	0.831961123	0.039983
XM_006532412.1	circRNA_6412|ChrMT:13917_14141_-	0.831904862	0.040009
XM_006518339.3	circRNA_4055|Chr3:126796838_126798280_+	0.831819747	0.040048
NM_144936.1	circRNA_6140|Chr9:61935380_61937535_-	0.831776059	0.040069
XM_011248193.2	circRNA_0961|Chr11:67192403_67254873_+	0.831775112	0.040069
NM_010742.1	circRNA_6140|Chr9:61935380_61937535_-	0.831751581	0.04008
NM_181315.4	circRNA_3155|Chr19:25061108_25065459_+	0.831727468	0.040091
NM_199304.1	circRNA_4635|Chr5:43758222_43773659_-	0.831727083	0.040091
XM_006521428.3	circRNA_3239|Chr19:45635735_45640521_-	0.831708408	0.0401
XM_006496277.3	circRNA_4662|Chr5:65301933_65303208_-	0.831681287	0.040112
XM_006527452.3	circRNA_4055|Chr3:126796838_126798280_+	0.831655977	0.040124
NM_015744.4	circRNA_3155|Chr19:25061108_25065459_+	0.831622221	0.04014
NM_178908.3	circRNA_4662|Chr5:65301933_65303208_-	0.831621655	0.04014
NM_009345.2	circRNA_0376|Chr1:172173943_172187460_+	0.831584019	0.040157
XM_006524382.3	circRNA_3003|Chr18:67545615_67587859_-	0.831572236	0.040163
NM_009127.4	circRNA_3239|Chr19:45635735_45640521_-	0.831559862	0.040169
NM_001024731.2	circRNA_3276|Chr19:60563162_60564294_-	0.831558567	0.040169
NM_178184.2	circRNA_3155|Chr19:25061108_25065459_+	0.831548271	0.040174
XM_006495613.3	circRNA_6140|Chr9:61935380_61937535_-	0.831510994	0.040191

XM_006529759.1	circRNA_6140|Chr9:61935380_61937535_-	0.831466423	0.040212
NM_009675.2	circRNA_3155|Chr19:25061108_25065459_+	0.831427296	0.04023
NM_016809.6	circRNA_6140|Chr9:61935380_61937535_-	0.83136959	0.040257
NM_028472.2	circRNA_4662|Chr5:65301933_65303208_-	0.831353336	0.040264
NM_146063.1	circRNA_5639|Chr7:132771578_132779385_-	0.831350813	0.040265
XM_006527221.3	circRNA_2951|Chr18:39120119_39150118_+	0.831345779	0.040268
XM_017317946.1	circRNA_0961|Chr11:67192403_67254873_+	0.831342974	0.040269
XM_006537815.3	circRNA_5074|Chr6:40685277_40747150_+	0.831318361	0.04028
NM_028472.2	circRNA_2888|Chr18:21010677_21020999_+	0.831294729	0.040291
XM_011250776.2	circRNA_3239|Chr19:45635735_45640521_-	0.831269269	0.040303
NM_023456.3	circRNA_0020|Chr1:10315205_10321709_+	0.831209204	0.040331
NM_018866.2	circRNA_0020|Chr1:10315205_10321709_+	0.831183877	0.040343
NM_175563.5	circRNA_5074|Chr6:40685277_40747150_+	0.831178981	0.040345
XM_011246309.2	circRNA_0611|Chr10:75274017_75279961_+	0.83113892	0.040364
NM_010859.2	circRNA_0537|Chr10:43393871_43395692_+	0.831023395	0.040417
NM_010450.3	circRNA_3155|Chr19:25061108_25065459_+	0.830909749	0.04047
NM_011581.3	circRNA_0611|Chr10:75274017_75279961_+	0.830867299	0.04049
NM_013468.3	circRNA_0845|Chr11:29470315_29477458_+	0.830863581	0.040491
NM_001190258.1	circRNA_3155|Chr19:25061108_25065459_+	0.830812471	0.040515
XM_011250732.2	circRNA_3954|Chr3:88346445_88349444_+	0.830811263	0.040516
NM_001033445.2	circRNA_3954|Chr3:88346445_88349444_+	0.830796577	0.040523
NM_001190448.1	circRNA_6446|ChrX:42217416_42250037_+	0.830769971	0.040535
NM_011169.5	circRNA_2951|Chr18:39120119_39150118_+	0.830760307	0.040539
XM_006527221.3	circRNA_5311|Chr6:145147636_145149357_+	0.830691146	0.040572
XM_017316715.1	circRNA_6390|Chr9:121727316_121731565_+	0.830617588	0.040606
NM_177743.5	circRNA_3239|Chr19:45635735_45640521_-	0.830531316	0.040646
XM_006524407.3	circRNA_0537|Chr10:43393871_43395692_+	0.830525318	0.040649
NM_001195662.1	circRNA_3832|Chr2:169883526_169886459_+	0.830520329	0.040651
NM_001099314.1	circRNA_4487|Chr4:149156607_149161694_-	0.830480147	0.04067
XM_011241948.2	circRNA_6446|ChrX:42217416_42250037_+	0.83036526	0.040723
NM_009246.3	circRNA_1042|Chr11:80385854_80403408_+	0.830353358	0.040729
XM_006514077.1	circRNA_5074|Chr6:40685277_40747150_+	0.830336155	0.040737
XM_006511221.3	circRNA_3954|Chr3:88346445_88349444_+	0.830279267	0.040763
XM_001481172.6	circRNA_0611|Chr10:75274017_75279961_+	0.830266511	0.040769
NM_009514.4	circRNA_2951|Chr18:39120119_39150118_+	0.830194763	0.040803
NM_007555.4	circRNA_3276|Chr19:60563162_60564294_-	0.830145032	0.040826
NM_199029.2	circRNA_4926|Chr5:143080224_143081077_-	0.830128661	0.040833
NM_009246.3	circRNA_1399|Chr12:72783683_72786789_+	0.83012007	0.040837
NM_009345.2	circRNA_2951|Chr18:39120119_39150118_+	0.830108476	0.040843
NM_007482.3	circRNA_0157|Chr1:66801049_66802168_-	0.830091164	0.040851
XM_006496949.3	circRNA_0157|Chr1:66801049_66802168_-	0.830078512	0.040857
NM_007817.2	circRNA_0020|Chr1:10315205_10321709_+	0.830044628	0.040873
NM_001134300.2	circRNA_4926|Chr5:143080224_143081077_-	0.830021407	0.040884
NM_001024731.2	circRNA_0376|Chr1:172173943_172187460_+	0.829989281	0.040899
NM_018732.3	circRNA_4926|Chr5:143080224_143081077_-	0.829965715	0.04091
XM_006501237.3	circRNA_0376|Chr1:172173943_172187460_+	0.82992679	0.040928
NM_013863.5	circRNA_3832|Chr2:169883526_169886459_+	0.829878135	0.04095
NM_021342.1	circRNA_5639|Chr7:132771578_132779385_-	0.829859392	0.040959
NM_001002896.2	circRNA_4728|Chr5:97027831_97045167_+	0.829849297	0.040964
NM_011704.3	circRNA_0376|Chr1:172173943_172187460_+	0.829847416	0.040965
NM_023716.2	circRNA_0376|Chr1:172173943_172187460_+	0.82981167	0.040981
NM_175692.3	circRNA_0501|Chr10:25283893_25289730_-	0.829789627	0.040992
NM_175692.3	circRNA_6109|Chr9:57056714_57057861_+	0.829789627	0.040992
NM_016809.6	circRNA_6446|ChrX:42217416_42250037_+	0.829726285	0.041021
NM_001099314.1	circRNA_5811|Chr8:71992832_71998295_-	0.829632347	0.041065
XM_006507725.2	circRNA_3954|Chr3:88346445_88349444_+	0.829551432	0.041103
NM_009605.4	circRNA_4728|Chr5:97027831_97045167_+	0.829511897	0.041122
XM_006501309.3	circRNA_4635|Chr5:43758222_43773659_-	0.829448313	0.041151
XM_017318541.1	circRNA_0157|Chr1:66801049_66802168_-	0.829411887	0.041168

XM_011248983.2	circRNA_4987|Chr6:4529068_4531010_+	0.829318398	0.041212
NM_019626.3	circRNA_4662|Chr5:65301933_65303208_-	0.829313049	0.041215
NM_172907.3	circRNA_3155|Chr19:25061108_25065459_+	0.829310277	0.041216
XM_011250176.1	circRNA_5074|Chr6:40685277_40747150_+	0.829297848	0.041222
NM_144539.5	circRNA_0157|Chr1:66801049_66802168_-	0.829212782	0.041262
XM_006496277.3	circRNA_5311|Chr6:145147636_145149357_+	0.829181144	0.041276
NM_009223.3	circRNA_2888|Chr18:21010677_21020999_+	0.82917667	0.041279
NM_023716.2	circRNA_0611|Chr10:75274017_75279961_+	0.829168363	0.041282
NM_009675.2	circRNA_4987|Chr6:4529068_4531010_+	0.829091512	0.041318
NM_009345.2	circRNA_0020|Chr1:10315205_10321709_+	0.82904688	0.041339
NM_008524.2	circRNA_4055|Chr3:126796838_126798280_+	0.82903477	0.041345
XM_017318264.1	circRNA_0962|Chr11:67194348_67220998_+	0.829020588	0.041352
XM_006504999.1	circRNA_6446|ChrX:42217416_42250037_+	0.82894587	0.041387
NM_008185.3	circRNA_0611|Chr10:75274017_75279961_+	0.828928119	0.041395
XM_011240149.1	circRNA_0537|Chr10:43393871_43395692_+	0.828905758	0.041406
NM_172454.2	circRNA_6412|ChrMT:13917_14141_-	0.828850219	0.041432
XM_006539074.3	circRNA_0537|Chr10:43393871_43395692_+	0.828823865	0.041444
NM_008760.4	circRNA_0157|Chr1:66801049_66802168_-	0.828809621	0.041451
NM_001290273.1	circRNA_0157|Chr1:66801049_66802168_-	0.828801485	0.041455
XM_017316736.1	circRNA_0501|Chr10:25283893_25289730_-	0.828798153	0.041456
XM_017316736.1	circRNA_6109|Chr9:57056714_57057861_+	0.828798153	0.041456
NM_007817.2	circRNA_4728|Chr5:97027831_97045167_+	0.828771204	0.041469
NM_001310705.1	circRNA_0611|Chr10:75274017_75279961_+	0.828745389	0.041481
XM_006533452.3	circRNA_4987|Chr6:4529068_4531010_+	0.828725813	0.04149
XM_006532120.2	circRNA_3276|Chr19:60563162_60564294_-	0.828722077	0.041492
XM_006502291.3	circRNA_5074|Chr6:40685277_40747150_+	0.828711743	0.041497
NM_009246.3	circRNA_3425|Chr2:37624173_37627526_-	0.828662286	0.04152
NM_009246.3	circRNA_3607|Chr2:119057281_119064097_+	0.828662286	0.04152
NM_019866.1	circRNA_0611|Chr10:75274017_75279961_+	0.828611621	0.041544
NM_020001.2	circRNA_0376|Chr1:172173943_172187460_+	0.828581135	0.041558
XM_017322365.1	circRNA_3276|Chr19:60563162_60564294_-	0.828579998	0.041559
XM_006504940.3	circRNA_2951|Chr18:39120119_39150118_+	0.828573177	0.041562
NM_001282961.1	circRNA_0020|Chr1:10315205_10321709_+	0.828558075	0.041569
XM_011250732.2	circRNA_6390|Chr9:121727316_121731565_+	0.82852051	0.041587
NM_146241.2	circRNA_4728|Chr5:97027831_97045167_+	0.8285174	0.041588
XM_006533569.3	circRNA_1399|Chr12:72783683_72786789_+	0.828509898	0.041592
XM_006504999.1	circRNA_2888|Chr18:21010677_21020999_+	0.828413692	0.041637
XM_006529829.3	circRNA_3702|Chr2:143830084_143832057_-	0.828380609	0.041652
NM_007881.4	circRNA_3832|Chr2:169883526_169886459_+	0.828360698	0.041662
NM_146241.2	circRNA_4987|Chr6:4529068_4531010_+	0.828289966	0.041695
NM_013459.3	circRNA_4662|Chr5:65301933_65303208_-	0.828282541	0.041699
NM_021475.2	circRNA_0020|Chr1:10315205_10321709_+	0.828274355	0.041702
XM_006506152.3	circRNA_3425|Chr2:37624173_37627526_-	0.828236236	0.04172
XM_006506152.3	circRNA_3607|Chr2:119057281_119064097_+	0.828236236	0.04172
NM_013743.2	circRNA_6390|Chr9:121727316_121731565_+	0.828176419	0.041749
NM_146125.2	circRNA_2951|Chr18:39120119_39150118_+	0.82813687	0.041767
XM_006532232.3	circRNA_4926|Chr5:143080224_143081077_-	0.828121565	0.041774
NM_031192.3	circRNA_3276|Chr19:60563162_60564294_-	0.828115247	0.041777
XM_006527008.2	circRNA_0961|Chr11:67192403_67254873_+	0.82809293	0.041788
NM_207231.1	circRNA_0157|Chr1:66801049_66802168_-	0.828057882	0.041804
XM_006530325.3	circRNA_0537|Chr10:43393871_43395692_+	0.827961695	0.04185
NM_001162950.1	circRNA_3954|Chr3:88346445_88349444_+	0.827907681	0.041875
XM_006524328.3	circRNA_0845|Chr11:29470315_29477458_+	0.82787928	0.041889
NM_001030305.2	circRNA_1632|Chr13:42055400_42055542_+	0.827868116	0.041894
NM_011044.2	circRNA_0020|Chr1:10315205_10321709_+	0.827860999	0.041897
XM_017322026.1	circRNA_4662|Chr5:65301933_65303208_-	0.827831577	0.041911
NM_145635.2	circRNA_5311|Chr6:145147636_145149357_+	0.827830225	0.041912
NM_011658.2	circRNA_0376|Chr1:172173943_172187460_+	0.827825394	0.041914
NM_025711.3	circRNA_4662|Chr5:65301933_65303208_-	0.827805661	0.041923

NM_008204.2	circRNA_3702|Chr2:143830084_143832057_-	0.827780393	0.041935
XM_006506152.3	circRNA_1399|Chr12:72783683_72786789_+	0.827780099	0.041936
XM_006506086.2	circRNA_3239|Chr19:45635735_45640521_-	0.827764968	0.041943
NM_010714.3	circRNA_0020|Chr1:10315205_10321709_+	0.827666094	0.041989
NM_019626.3	circRNA_6446|ChrX:42217416_42250037_+	0.827624353	0.042009
NM_144936.1	circRNA_5639|Chr7:132771578_132779385_-	0.82759654	0.042022
NM_010140.3	circRNA_4728|Chr5:97027831_97045167_+	0.827537606	0.04205
XM_006497892.3	circRNA_3832|Chr2:169883526_169886459_+	0.827531929	0.042053
XM_011244046.2	circRNA_0376|Chr1:172173943_172187460_+	0.827464918	0.042084
NM_007549.2	circRNA_0157|Chr1:66801049_66802168_-	0.827451756	0.042091
XM_006516510.1	circRNA_2888|Chr18:21010677_21020999_+	0.827450452	0.042091
XM_006509966.3	circRNA_4926|Chr5:143080224_143081077_-	0.827444115	0.042094
XM_011244488.2	circRNA_3239|Chr19:45635735_45640521_-	0.827396947	0.042117
XM_006506152.3	circRNA_3832|Chr2:169883526_169886459_+	0.827378737	0.042125
NM_008940.3	circRNA_2951|Chr18:39120119_39150118_+	0.827362675	0.042133
XM_006505757.3	circRNA_2951|Chr18:39120119_39150118_+	0.827344518	0.042141
XM_006508990.2	circRNA_5811|Chr8:71992832_71998295_-	0.827327149	0.04215
NM_007817.2	circRNA_3239|Chr19:45635735_45640521_-	0.827253375	0.042185
NM_145226.2	circRNA_6390|Chr9:121727316_121731565_+	0.82713757	0.042239
NM_016982.2	circRNA_2888|Chr18:21010677_21020999_+	0.827111162	0.042252
NM_009504.4	circRNA_0537|Chr10:43393871_43395692_+	0.826987992	0.04231
XM_017316676.1	circRNA_3832|Chr2:169883526_169886459_+	0.826965349	0.042321
XM_011248193.2	circRNA_4926|Chr5:143080224_143081077_-	0.826940672	0.042333
NM_011430.3	circRNA_5639|Chr7:132771578_132779385_-	0.826886628	0.042358
NM_172907.3	circRNA_3702|Chr2:143830084_143832057_-	0.826876646	0.042363
NM_015744.4	circRNA_0376|Chr1:172173943_172187460_+	0.826830856	0.042385
NM_001142804.1	circRNA_0020|Chr1:10315205_10321709_+	0.826827797	0.042386
NM_133167.3	circRNA_4926|Chr5:143080224_143081077_-	0.826781786	0.042408
NM_010608.2	circRNA_1632|Chr13:42055400_42055542_+	0.826779285	0.042409
XM_011244488.2	circRNA_6446|ChrX:42217416_42250037_+	0.826777923	0.04241
XM_006517308.3	circRNA_0376|Chr1:172173943_172187460_+	0.826751596	0.042422
XM_006532120.2	circRNA_0501|Chr10:25283893_25289730_-	0.826685478	0.042454
XM_006532120.2	circRNA_6109|Chr9:57056714_57057861_+	0.826685478	0.042454
NM_178782.4	circRNA_5074|Chr6:40685277_40747150_+	0.826596835	0.042496
NM_181315.4	circRNA_3239|Chr19:45635735_45640521_-	0.826584192	0.042502
XM_017316944.1	circRNA_0376|Chr1:172173943_172187460_+	0.826568444	0.042509
NM_001313939.1	circRNA_0611|Chr10:75274017_75279961_+	0.826541321	0.042522
XM_017313038.1	circRNA_0611|Chr10:75274017_75279961_+	0.826505904	0.042539
NM_008264.1	circRNA_3702|Chr2:143830084_143832057_-	0.826492356	0.042546
XM_006521554.3	circRNA_5074|Chr6:40685277_40747150_+	0.826464858	0.042559
NM_009127.4	circRNA_5311|Chr6:145147636_145149357_+	0.826446663	0.042567
NM_015814.2	circRNA_6446|ChrX:42217416_42250037_+	0.826386909	0.042596
XM_006518339.3	circRNA_6140|Chr9:61935380_61937535_-	0.826348898	0.042614
NM_001309809.2	circRNA_4728|Chr5:97027831_97045167_+	0.82634008	0.042618
NM_001309809.2	circRNA_3239|Chr19:45635735_45640521_-	0.82633569	0.04262
XM_006506086.2	circRNA_5367|Chr7:28990988_28991325_+	0.826308961	0.042633
NM_173427.2	circRNA_4055|Chr3:126796838_126798280_+	0.826256716	0.042658
XM_006540566.1	circRNA_2313|Chr15:93452117_93465245_+	0.826223527	0.042674
XM_006533539.3	circRNA_0157|Chr1:66801049_66802168_-	0.826187076	0.042691
NM_001004357.2	circRNA_5367|Chr7:28990988_28991325_+	0.826165648	0.042701
XM_006524382.3	circRNA_0537|Chr10:43393871_43395692_+	0.826114394	0.042725
XM_006530585.3	circRNA_0961|Chr11:67192403_67254873_+	0.826106851	0.042729
NM_001033630.1	circRNA_1980|Chr14:50951404_50963869_+	0.826092822	0.042736
NM_008940.3	circRNA_0501|Chr10:25283893_25289730_-	0.82607568	0.042744
NM_008940.3	circRNA_6109|Chr9:57056714_57057861_+	0.82607568	0.042744
NM_001320077.1	circRNA_3643|Chr2:122441559_122486090_+	0.82607378	0.042745
NM_007792.4	circRNA_4728|Chr5:97027831_97045167_+	0.826050094	0.042756
NM_205810.4	circRNA_3702|Chr2:143830084_143832057_-	0.826024967	0.042768
NM_053080.3	circRNA_0157|Chr1:66801049_66802168_-	0.826009979	0.042775

NM_028472.2	circRNA_5639|Chr7:132771578_132779385_-	0.825939888	0.042809
XM_017317978.1	circRNA_0501|Chr10:25283893_25289730_-	0.825875782	0.042839
XM_017317978.1	circRNA_6109|Chr9:57056714_57057861_+	0.825875782	0.042839
NM_023143.3	circRNA_0157|Chr1:66801049_66802168_-	0.825769907	0.04289
XM_011250776.2	circRNA_4728|Chr5:97027831_97045167_+	0.825759889	0.042894
NM_008804.4	circRNA_3276|Chr19:60563162_60564294_-	0.82567493	0.042935
NM_145635.2	circRNA_0913|Chr11:54005394_54014456_-	0.825594628	0.042973
XM_017316439.1	circRNA_4987|Chr6:4529068_4531010_+	0.82556766	0.042986
NM_053080.3	circRNA_3643|Chr2:122441559_122486090_+	0.825563684	0.042988
NM_020001.2	circRNA_4728|Chr5:97027831_97045167_+	0.825555765	0.042992
NM_153104.3	circRNA_6412|ChrMT:13917_14141_-	0.825552977	0.042993
NM_009019.2	circRNA_6140|Chr9:61935380_61937535_-	0.8255414	0.042999
NM_001134300.2	circRNA_5074|Chr6:40685277_40747150_+	0.825535184	0.043002
XM_006510701.3	circRNA_0537|Chr10:43393871_43395692_+	0.825530339	0.043004
XM_011247208.1	circRNA_4926|Chr5:143080224_143081077_-	0.825442309	0.043046
XM_011244488.2	circRNA_6140|Chr9:61935380_61937535_-	0.825416364	0.043059
XM_006506145.1	circRNA_0845|Chr11:29470315_29477458_+	0.825357312	0.043087
NM_177743.5	circRNA_2888|Chr18:21010677_21020999_+	0.825334442	0.043098
NM_010181.2	circRNA_0961|Chr11:67192403_67254873_+	0.825274469	0.043126
NM_008185.3	circRNA_4728|Chr5:97027831_97045167_+	0.825209713	0.043157
NM_015814.2	circRNA_4662|Chr5:65301933_65303208_-	0.825187967	0.043168
XM_017317034.1	circRNA_0961|Chr11:67192403_67254873_+	0.825176932	0.043173
NM_008185.3	circRNA_0501|Chr10:25283893_25289730_-	0.825169082	0.043177
NM_008185.3	circRNA_6109|Chr9:57056714_57057861_+	0.825169082	0.043177
NM_011581.3	circRNA_6446|ChrX:42217416_42250037_+	0.825157928	0.043182
NM_011581.3	circRNA_4662|Chr5:65301933_65303208_-	0.825132571	0.043194
XM_006532024.3	circRNA_0913|Chr11:54005394_54014456_-	0.825067748	0.043225
XM_006511007.3	circRNA_0845|Chr11:29470315_29477458_+	0.824996394	0.04326
XM_006501309.3	circRNA_2313|Chr15:93452117_93465245_+	0.824980753	0.043267
XM_006537508.2	circRNA_3702|Chr2:143830084_143832057_-	0.824954654	0.04328
NM_001167908.1	circRNA_3954|Chr3:88346445_88349444_+	0.824918796	0.043297
NM_007881.4	circRNA_0961|Chr11:67192403_67254873_+	0.824911082	0.0433
NM_178676.4	circRNA_3954|Chr3:88346445_88349444_+	0.824888895	0.043311
NM_029844.3	circRNA_4662|Chr5:65301933_65303208_-	0.824816126	0.043346
NM_001082546.1	circRNA_6140|Chr9:61935380_61937535_-	0.82481345	0.043347
NM_010133.2	circRNA_1980|Chr14:50951404_50963869_+	0.824802631	0.043352
XM_006515787.3	circRNA_3643|Chr2:122441559_122486090_+	0.824750338	0.043377
XM_006515980.3	circRNA_4728|Chr5:97027831_97045167_+	0.824727779	0.043388
NM_001290469.1	circRNA_5074|Chr6:40685277_40747150_+	0.824727532	0.043388
XM_006529759.1	circRNA_5639|Chr7:132771578_132779385_-	0.824621399	0.043439
NM_009345.2	circRNA_3702|Chr2:143830084_143832057_-	0.824616241	0.043442
XM_006508867.2	circRNA_3702|Chr2:143830084_143832057_-	0.824593102	0.043453
XM_006506086.2	circRNA_0157|Chr1:66801049_66802168_-	0.824478612	0.043508
XM_011241322.1	circRNA_5074|Chr6:40685277_40747150_+	0.824454342	0.04352
XM_006506624.2	circRNA_2951|Chr18:39120119_39150118_+	0.824433815	0.043529
XM_006511102.2	circRNA_0376|Chr1:172173943_172187460_+	0.824345214	0.043572
NM_001291483.1	circRNA_0611|Chr10:75274017_75279961_+	0.824314247	0.043587
NM_023143.3	circRNA_4055|Chr3:126796838_126798280_+	0.824308453	0.04359
NM_001301295.1	circRNA_2951|Chr18:39120119_39150118_+	0.82427118	0.043608
NM_001190325.1	circRNA_3702|Chr2:143830084_143832057_-	0.824231499	0.043627
NM_008623.5	circRNA_3643|Chr2:122441559_122486090_+	0.824162408	0.04366
NM_001097617.1	circRNA_4662|Chr5:65301933_65303208_-	0.824154734	0.043664
NM_008760.4	circRNA_5311|Chr6:145147636_145149357_+	0.824042738	0.043718
XM_006538456.3	circRNA_4926|Chr5:143080224_143081077_-	0.824042425	0.043718
NM_023143.3	circRNA_6140|Chr9:61935380_61937535_-	0.823926332	0.043774
NM_001033167.3	circRNA_2313|Chr15:93452117_93465245_+	0.823874818	0.043798
NM_020001.2	circRNA_3155|Chr19:25061108_25065459_+	0.823842113	0.043814
NM_019732.2	circRNA_4926|Chr5:143080224_143081077_-	0.823787128	0.043841
XM_006505757.3	circRNA_6140|Chr9:61935380_61937535_-	0.823734345	0.043866

XM_006530353.3	circRNA_3954|Chr3:88346445_88349444_+	0.823691028	0.043887
NM_001310648.1	circRNA_4662|Chr5:65301933_65303208_-	0.823625396	0.043919
XM_006529382.3	circRNA_0537|Chr10:43393871_43395692_+	0.823621535	0.043921
XM_011248919.2	circRNA_4926|Chr5:143080224_143081077_-	0.823617969	0.043922
XM_006537508.2	circRNA_2888|Chr18:21010677_21020999_+	0.823598757	0.043932
XM_006537508.2	circRNA_4055|Chr3:126796838_126798280_+	0.823581272	0.04394
NM_177371.3	circRNA_3239|Chr19:45635735_45640521_-	0.823544067	0.043958
NM_001190258.1	circRNA_0020|Chr1:10315205_10321709_+	0.823531194	0.043964
NM_145226.2	circRNA_1042|Chr11:80385854_80403408_+	0.823495889	0.043981
NM_001080979.1	circRNA_1980|Chr14:50951404_50963869_+	0.823487038	0.043985
NM_001082547.1	circRNA_5639|Chr7:132771578_132779385_-	0.823416912	0.044019
NM_001097617.1	circRNA_6446|ChrX:42217416_42250037_+	0.823380159	0.044037
XM_006504899.3	circRNA_3702|Chr2:143830084_143832057_-	0.823363136	0.044045
NM_007655.3	circRNA_3643|Chr2:122441559_122486090_+	0.823333469	0.04406
NM_198024.2	circRNA_5311|Chr6:145147636_145149357_+	0.82327591	0.044087
XM_006523030.3	circRNA_0961|Chr11:67192403_67254873_+	0.823246842	0.044101
NM_001286743.1	circRNA_0537|Chr10:43393871_43395692_+	0.823231727	0.044109
NM_001109985.1	circRNA_0537|Chr10:43393871_43395692_+	0.823195197	0.044126
XM_006515980.3	circRNA_3155|Chr19:25061108_25065459_+	0.823086994	0.044179
XM_006507942.2	circRNA_3239|Chr19:45635735_45640521_-	0.823058069	0.044193
NM_181547.3	circRNA_0913|Chr11:54005394_54014456_-	0.823027292	0.044208
NM_031185.3	circRNA_3643|Chr2:122441559_122486090_+	0.823021557	0.04421
NM_031192.3	circRNA_3643|Chr2:122441559_122486090_+	0.823018491	0.044212
NM_001271580.1	circRNA_6140|Chr9:61935380_61937535_-	0.823009206	0.044216
XM_006533479.2	circRNA_6446|ChrX:42217416_42250037_+	0.822976193	0.044232
NM_181547.3	circRNA_5639|Chr7:132771578_132779385_-	0.822808403	0.044314
NM_007763.3	circRNA_0376|Chr1:172173943_172187460_+	0.822803682	0.044316
XM_006510701.3	circRNA_4926|Chr5:143080224_143081077_-	0.822791774	0.044322
XM_017317946.1	circRNA_3954|Chr3:88346445_88349444_+	0.822777477	0.044329
NM_133643.4	circRNA_1632|Chr13:42055400_42055542_+	0.822738649	0.044347
NM_133643.4	circRNA_0157|Chr1:66801049_66802168_-	0.82271889	0.044357
XM_011245344.2	circRNA_0961|Chr11:67192403_67254873_+	0.822690933	0.044371
NM_011658.2	circRNA_6446|ChrX:42217416_42250037_+	0.822681143	0.044375
XM_006532412.1	circRNA_1980|Chr14:50951404_50963869_+	0.822669676	0.044381
NM_001290993.1	circRNA_5311|Chr6:145147636_145149357_+	0.822569421	0.04443
NM_001161746.1	circRNA_6390|Chr9:121727316_121731565_+	0.822530597	0.044448
XM_006498287.3	circRNA_5811|Chr8:71992832_71998295_-	0.82252781	0.04445
NM_007881.4	circRNA_0537|Chr10:43393871_43395692_+	0.822431056	0.044497
XM_006521428.3	circRNA_3155|Chr19:25061108_25065459_+	0.822430823	0.044497
NM_134072.1	circRNA_4662|Chr5:65301933_65303208_-	0.822391912	0.044516
XM_011243586.2	circRNA_0157|Chr1:66801049_66802168_-	0.822317877	0.044552
NM_001289875.1	circRNA_2313|Chr15:93452117_93465245_+	0.82228709	0.044567
XM_006520186.2	circRNA_2951|Chr18:39120119_39150118_+	0.822239939	0.044589
XM_006529759.1	circRNA_2888|Chr18:21010677_21020999_+	0.822237902	0.04459
XM_006529753.2	circRNA_0845|Chr11:29470315_29477458_+	0.822227912	0.044595
NM_009379.3	circRNA_3832|Chr2:169883526_169886459_+	0.822202817	0.044608
NM_178753.4	circRNA_3643|Chr2:122441559_122486090_+	0.822195588	0.044611
XM_006501443.3	circRNA_0962|Chr11:67194348_67220998_+	0.822174886	0.044621
XM_006501309.3	circRNA_3832|Chr2:169883526_169886459_+	0.822062214	0.044676
XM_011246309.2	circRNA_5367|Chr7:28990988_28991325_+	0.822037229	0.044688
NM_008760.4	circRNA_2888|Chr18:21010677_21020999_+	0.821929828	0.04474
XM_006502291.3	circRNA_3954|Chr3:88346445_88349444_+	0.821920434	0.044745
NM_001310705.1	circRNA_2888|Chr18:21010677_21020999_+	0.821802126	0.044802
XM_006538374.1	circRNA_1632|Chr13:42055400_42055542_+	0.821790302	0.044808
NM_173869.3	circRNA_5639|Chr7:132771578_132779385_-	0.821786214	0.04481
XM_006527221.3	circRNA_6446|ChrX:42217416_42250037_+	0.821720978	0.044842
NM_008277.2	circRNA_0157|Chr1:66801049_66802168_-	0.821691713	0.044856
XM_006541047.3	circRNA_3643|Chr2:122441559_122486090_+	0.821642003	0.04488
NM_001159407.1	circRNA_1632|Chr13:42055400_42055542_+	0.821596789	0.044902

XM_006520253.3	circRNA_2313|Chr15:93452117_93465245_+	0.821551848	0.044924
NM_001007570.2	circRNA_0845|Chr11:29470315_29477458_+	0.821535543	0.044932
NM_181547.3	circRNA_3155|Chr19:25061108_25065459_+	0.821511643	0.044944
NM_001082547.1	circRNA_6446|ChrX:42217416_42250037_+	0.821495671	0.044952
NM_027416.3	circRNA_3239|Chr19:45635735_45640521_-	0.82149273	0.044953
XM_006508885.1	circRNA_3239|Chr19:45635735_45640521_-	0.821476639	0.044961
XM_006538830.1	circRNA_4926|Chr5:143080224_143081077_-	0.821470682	0.044964
XM_006521428.3	circRNA_0611|Chr10:75274017_75279961_+	0.821454294	0.044972
XM_006538830.1	circRNA_5990|Chr9:3475483_3477962_+	0.821434012	0.044982
NM_001204959.1	circRNA_2951|Chr18:39120119_39150118_+	0.82141425	0.044991
NM_019866.1	circRNA_1632|Chr13:42055400_42055542_+	0.821402039	0.044997
NM_008804.4	circRNA_3155|Chr19:25061108_25065459_+	0.821399594	0.044999
NM_001190451.2	circRNA_4728|Chr5:97027831_97045167_+	0.821396152	0.045
NM_008264.1	circRNA_0020|Chr1:10315205_10321709_+	0.82139453	0.045001
XM_006506808.3	circRNA_3832|Chr2:169883526_169886459_+	0.821314964	0.04504
XM_017320093.1	circRNA_3155|Chr19:25061108_25065459_+	0.821301288	0.045047
XM_006514444.1	circRNA_5990|Chr9:3475483_3477962_+	0.821264865	0.045064
XM_011240779.2	circRNA_6446|ChrX:42217416_42250037_+	0.821251532	0.045071
NM_144799.2	circRNA_6390|Chr9:121727316_121731565_+	0.821160681	0.045115
NM_001033167.3	circRNA_5811|Chr8:71992832_71998295_-	0.821150143	0.04512
XM_017315313.1	circRNA_6446|ChrX:42217416_42250037_+	0.82113178	0.045129
XM_006521796.1	circRNA_5639|Chr7:132771578_132779385_-	0.821124503	0.045133
NM_030206.4	circRNA_0020|Chr1:10315205_10321709_+	0.821044803	0.045172
NM_020025.4	circRNA_6140|Chr9:61935380_61937535_-	0.821008658	0.04519
NM_178184.2	circRNA_0020|Chr1:10315205_10321709_+	0.820956873	0.045215
NM_001001178.1	circRNA_0376|Chr1:172173943_172187460_+	0.820938002	0.045224
XM_011242311.2	circRNA_0537|Chr10:43393871_43395692_+	0.820920548	0.045233
XM_006532962.2	circRNA_4926|Chr5:143080224_143081077_-	0.820912609	0.045237
NM_008277.2	circRNA_1632|Chr13:42055400_42055542_+	0.820882145	0.045251
XM_017316439.1	circRNA_4055|Chr3:126796838_126798280_+	0.820880251	0.045252
NM_001286743.1	circRNA_1042|Chr11:80385854_80403408_+	0.820861604	0.045262
XM_006501309.3	circRNA_0537|Chr10:43393871_43395692_+	0.820752303	0.045315
NM_001204959.1	circRNA_2888|Chr18:21010677_21020999_+	0.820740558	0.045321
XM_006498287.3	circRNA_4487|Chr4:149156607_149161694_-	0.820730547	0.045326
NM_011082.3	circRNA_6446|ChrX:42217416_42250037_+	0.820713186	0.045334
NM_009518.2	circRNA_0157|Chr1:66801049_66802168_-	0.82061623	0.045382
XM_006538456.3	circRNA_3954|Chr3:88346445_88349444_+	0.82059967	0.04539
NM_010055.3	circRNA_4487|Chr4:149156607_149161694_-	0.820517603	0.04543
XM_006524328.3	circRNA_5074|Chr6:40685277_40747150_+	0.820468542	0.045454
NM_177409.3	circRNA_4926|Chr5:143080224_143081077_-	0.820450861	0.045463
XM_006529727.2	circRNA_0961|Chr11:67192403_67254873_+	0.8204232	0.045476
XM_017315313.1	circRNA_2888|Chr18:21010677_21020999_+	0.820377697	0.045499
XM_006508990.2	circRNA_5990|Chr9:3475483_3477962_+	0.820339113	0.045518
NM_001033221.3	circRNA_0537|Chr10:43393871_43395692_+	0.820282505	0.045545
NM_001142804.1	circRNA_0611|Chr10:75274017_75279961_+	0.820267532	0.045553
NM_008185.3	circRNA_6140|Chr9:61935380_61937535_-	0.820238198	0.045567
XM_006509966.3	circRNA_3954|Chr3:88346445_88349444_+	0.820219797	0.045576
XM_006507725.2	circRNA_4926|Chr5:143080224_143081077_-	0.820210137	0.045581
NM_027416.3	circRNA_2951|Chr18:39120119_39150118_+	0.820197187	0.045587
XM_006504999.1	circRNA_3702|Chr2:143830084_143832057_-	0.820172245	0.045599
NM_019626.3	circRNA_0157|Chr1:66801049_66802168_-	0.820133179	0.045619
NM_175260.2	circRNA_6390|Chr9:121727316_121731565_+	0.820056916	0.045656
NM_011254.5	circRNA_6140|Chr9:61935380_61937535_-	0.820036357	0.045666
NM_010714.3	circRNA_0611|Chr10:75274017_75279961_+	0.820036322	0.045666
XM_006525075.3	circRNA_0501|Chr10:25283893_25289730_-	0.820017286	0.045676
XM_006525075.3	circRNA_6109|Chr9:57056714_57057861_+	0.820017286	0.045676
NM_007655.3	circRNA_1632|Chr13:42055400_42055542_+	0.819872017	0.045747
XM_006505015.3	circRNA_3832|Chr2:169883526_169886459_+	0.819857599	0.045754
XM_006540566.1	circRNA_1042|Chr11:80385854_80403408_+	0.819854931	0.045755

XM_006527221.3	circRNA_0501|Chr10:25283893_25289730_-	0.819845059	0.04576
XM_006527221.3	circRNA_6109|Chr9:57056714_57057861_+	0.819845059	0.04576
XM_006516928.2	circRNA_0611|Chr10:75274017_75279961_+	0.819843665	0.045761
NM_181315.4	circRNA_2888|Chr18:21010677_21020999_+	0.819789797	0.045787
XM_011248394.1	circRNA_0020|Chr1:10315205_10321709_+	0.819767334	0.045798
XM_017320093.1	circRNA_2951|Chr18:39120119_39150118_+	0.819756651	0.045804
XM_006508885.1	circRNA_0611|Chr10:75274017_75279961_+	0.819732936	0.045815
XM_006530186.3	circRNA_0962|Chr11:67194348_67220998_+	0.8196981	0.045832
XM_006529759.1	circRNA_2951|Chr18:39120119_39150118_+	0.819678966	0.045842
NM_001286743.1	circRNA_1399|Chr12:72783683_72786789_+	0.819666622	0.045848
XM_006534520.3	circRNA_3832|Chr2:169883526_169886459_+	0.819623418	0.045869
NM_181315.4	circRNA_3276|Chr19:60563162_60564294_-	0.819559684	0.045901
NM_010279.3	circRNA_0376|Chr1:172173943_172187460_+	0.819509673	0.045925
XM_006516224.3	circRNA_3954|Chr3:88346445_88349444_+	0.819488019	0.045936
NM_001301295.1	circRNA_2888|Chr18:21010677_21020999_+	0.819459189	0.04595
XM_006496884.3	circRNA_0501|Chr10:25283893_25289730_-	0.819436496	0.045961
XM_006496884.3	circRNA_6109|Chr9:57056714_57057861_+	0.819436496	0.045961
NM_019696.2	circRNA_6140|Chr9:61935380_61937535_-	0.819298224	0.046029
NM_030206.4	circRNA_4055|Chr3:126796838_126798280_+	0.819284779	0.046036
XM_006508867.2	circRNA_0501|Chr10:25283893_25289730_-	0.81926295	0.046047
XM_006508867.2	circRNA_6109|Chr9:57056714_57057861_+	0.81926295	0.046047
NM_025288.2	circRNA_6446|ChrX:42217416_42250037_+	0.819243934	0.046056
XM_006525028.2	circRNA_4662|Chr5:65301933_65303208_-	0.819238055	0.046059
NM_025711.3	circRNA_0611|Chr10:75274017_75279961_+	0.81921702	0.04607
XM_006517308.3	circRNA_4055|Chr3:126796838_126798280_+	0.819191214	0.046082
XM_017315734.1	circRNA_6390|Chr9:121727316_121731565_+	0.819186615	0.046085
NM_001313939.1	circRNA_2888|Chr18:21010677_21020999_+	0.819150399	0.046102
XM_006525099.3	circRNA_0962|Chr11:67194348_67220998_+	0.819118692	0.046118
XM_006495613.3	circRNA_4662|Chr5:65301933_65303208_-	0.819068867	0.046143
XM_011243586.2	circRNA_6140|Chr9:61935380_61937535_-	0.819009993	0.046172
NM_001290993.1	circRNA_1632|Chr13:42055400_42055542_+	0.818972687	0.04619
NM_181547.3	circRNA_3239|Chr19:45635735_45640521_-	0.818896251	0.046228
NM_031185.3	circRNA_4055|Chr3:126796838_126798280_+	0.81889086	0.046231
NM_177409.3	circRNA_0845|Chr11:29470315_29477458_+	0.818875107	0.046238
XM_006537508.2	circRNA_0501|Chr10:25283893_25289730_-	0.818872324	0.04624
XM_006537508.2	circRNA_6109|Chr9:57056714_57057861_+	0.818872324	0.04624
NM_018807.5	circRNA_3954|Chr3:88346445_88349444_+	0.818692789	0.046328
NM_146241.2	circRNA_6140|Chr9:61935380_61937535_-	0.818672118	0.046339
NM_145226.2	circRNA_5811|Chr8:71992832_71998295_-	0.81866684	0.046341
XM_006525075.3	circRNA_2951|Chr18:39120119_39150118_+	0.818665906	0.046342
NM_016659.3	circRNA_6446|ChrX:42217416_42250037_+	0.818658655	0.046345
XM_006516510.1	circRNA_2951|Chr18:39120119_39150118_+	0.818617931	0.046365
NM_020025.4	circRNA_3702|Chr2:143830084_143832057_-	0.818575628	0.046386
XM_006541232.3	circRNA_3832|Chr2:169883526_169886459_+	0.818537815	0.046405
NM_001244031.1	circRNA_0961|Chr11:67192403_67254873_+	0.818460251	0.046444
NM_023670.3	circRNA_3276|Chr19:60563162_60564294_-	0.818450403	0.046448
NM_011581.3	circRNA_0376|Chr1:172173943_172187460_+	0.818436878	0.046455
NM_207231.1	circRNA_0611|Chr10:75274017_75279961_+	0.818421154	0.046463
NM_001004357.2	circRNA_3643|Chr2:122441559_122486090_+	0.818410703	0.046468
NM_146125.2	circRNA_2888|Chr18:21010677_21020999_+	0.818354503	0.046496
NM_001301295.1	circRNA_4728|Chr5:97027831_97045167_+	0.818346307	0.0465
XM_011244235.2	circRNA_6140|Chr9:61935380_61937535_-	0.818340109	0.046503
XM_006500919.3	circRNA_0611|Chr10:75274017_75279961_+	0.818277873	0.046534
NM_001270475.1	circRNA_4926|Chr5:143080224_143081077_-	0.818239959	0.046553
NM_019397.3	circRNA_5311|Chr6:145147636_145149357_+	0.818220896	0.046562
NM_001290993.1	circRNA_3643|Chr2:122441559_122486090_+	0.818196827	0.046574
NM_010858.4	circRNA_2951|Chr18:39120119_39150118_+	0.818153939	0.046595
NM_001286743.1	circRNA_3425|Chr2:37624173_37627526_-	0.818140058	0.046602
NM_001286743.1	circRNA_3607|Chr2:119057281_119064097_+	0.818140058	0.046602

NM_013459.3	circRNA_2888|Chr18:21010677_21020999_+	0.81809807	0.046623
NM_001164593.1	circRNA_0157|Chr1:66801049_66802168_-	0.818053854	0.046645
NM_001290822.1	circRNA_3702|Chr2:143830084_143832057_-	0.818002416	0.046671
NM_011854.2	circRNA_3832|Chr2:169883526_169886459_+	0.817956402	0.046693
NM_153178.4	circRNA_0845|Chr11:29470315_29477458_+	0.817947501	0.046698
XM_011250732.2	circRNA_3832|Chr2:169883526_169886459_+	0.817946211	0.046698
NM_027293.1	circRNA_3954|Chr3:88346445_88349444_+	0.817838361	0.046752
NM_001033286.2	circRNA_4926|Chr5:143080224_143081077_-	0.817793298	0.046774
XM_006518339.3	circRNA_4987|Chr6:4529068_4531010_+	0.817752909	0.046794
NM_001002896.2	circRNA_5639|Chr7:132771578_132779385_-	0.817738811	0.046801
NM_010140.3	circRNA_3239|Chr19:45635735_45640521_-	0.817720603	0.04681
NM_001309809.2	circRNA_2888|Chr18:21010677_21020999_+	0.817702645	0.046819
NM_007641.5	circRNA_0611|Chr10:75274017_75279961_+	0.817671772	0.046835
NM_001002896.2	circRNA_2888|Chr18:21010677_21020999_+	0.817661994	0.04684
NM_019759.2	circRNA_2951|Chr18:39120119_39150118_+	0.81759158	0.046875
XM_011250978.2	circRNA_3954|Chr3:88346445_88349444_+	0.817586814	0.046877
NM_011658.2	circRNA_0611|Chr10:75274017_75279961_+	0.817585061	0.046878
XM_017317978.1	circRNA_5639|Chr7:132771578_132779385_-	0.817578168	0.046881
NM_177789.4	circRNA_6140|Chr9:61935380_61937535_-	0.817547988	0.046896
NM_001313939.1	circRNA_2951|Chr18:39120119_39150118_+	0.81750629	0.046917
NM_001301354.1	circRNA_6446|ChrX:42217416_42250037_+	0.817470078	0.046935
XM_017320219.1	circRNA_5990|Chr9:3475483_3477962_+	0.817463797	0.046938
XM_017315841.1	circRNA_0537|Chr10:43393871_43395692_+	0.817437727	0.046951
NM_153151.3	circRNA_0501|Chr10:25283893_25289730_-	0.817417348	0.046961
NM_153151.3	circRNA_6109|Chr9:57056714_57057861_+	0.817417348	0.046961
NM_172790.2	circRNA_3832|Chr2:169883526_169886459_+	0.817401119	0.046969
XM_006533452.3	circRNA_4055|Chr3:126796838_126798280_+	0.817384804	0.046978
XM_006521931.1	circRNA_0913|Chr11:54005394_54014456_-	0.817377246	0.046981
XM_006513165.1	circRNA_6140|Chr9:61935380_61937535_-	0.817368012	0.046986
NM_011044.2	circRNA_0501|Chr10:25283893_25289730_-	0.817349104	0.046995
NM_011044.2	circRNA_6109|Chr9:57056714_57057861_+	0.817349104	0.046995
NM_011145.3	circRNA_1042|Chr11:80385854_80403408_+	0.817332758	0.047003
NM_008872.3	circRNA_3239|Chr19:45635735_45640521_-	0.817322778	0.047008
XM_006506086.2	circRNA_3155|Chr19:25061108_25065459_+	0.817321556	0.047009
NM_145741.2	circRNA_4987|Chr6:4529068_4531010_+	0.817291333	0.047024
XM_006533479.2	circRNA_0913|Chr11:54005394_54014456_-	0.817208791	0.047065
NM_001081185.1	circRNA_6390|Chr9:121727316_121731565_+	0.817141522	0.047099
NM_133643.4	circRNA_2951|Chr18:39120119_39150118_+	0.817129239	0.047105
XM_017316715.1	circRNA_4926|Chr5:143080224_143081077_-	0.817106426	0.047116
NM_023716.2	circRNA_0157|Chr1:66801049_66802168_-	0.817082	0.047128
NM_009246.3	circRNA_5074|Chr6:40685277_40747150_+	0.817078147	0.04713
NM_009605.4	circRNA_0501|Chr10:25283893_25289730_-	0.817072101	0.047133
NM_009605.4	circRNA_6109|Chr9:57056714_57057861_+	0.817072101	0.047133
XM_011243266.1	circRNA_3832|Chr2:169883526_169886459_+	0.817065396	0.047137
NM_008185.3	circRNA_3702|Chr2:143830084_143832057_-	0.817057086	0.047141
NM_010858.4	circRNA_4662|Chr5:65301933_65303208_-	0.817004023	0.047167
XM_006500919.3	circRNA_0020|Chr1:10315205_10321709_+	0.816814012	0.047262
NM_019759.2	circRNA_4728|Chr5:97027831_97045167_+	0.816813024	0.047263
XM_006513859.3	circRNA_6412|ChrMT:13917_14141_-	0.816779881	0.047279
NM_016933.3	circRNA_6446|ChrX:42217416_42250037_+	0.816767261	0.047285
XM_011240476.2	circRNA_5990|Chr9:3475483_3477962_+	0.816756963	0.047291
XM_006503371.2	circRNA_4487|Chr4:149156607_149161694_-	0.816753667	0.047292
XM_006512076.3	circRNA_0537|Chr10:43393871_43395692_+	0.816749015	0.047295
NM_011145.3	circRNA_6390|Chr9:121727316_121731565_+	0.816736765	0.047301
NM_177371.3	circRNA_3155|Chr19:25061108_25065459_+	0.816692326	0.047323
NM_001195084.1	circRNA_2888|Chr18:21010677_21020999_+	0.816612571	0.047363
NM_001002896.2	circRNA_4662|Chr5:65301933_65303208_-	0.816519486	0.047409
NM_175692.3	circRNA_0376|Chr1:172173943_172187460_+	0.816451268	0.047443
NM_008760.4	circRNA_3239|Chr19:45635735_45640521_-	0.816447363	0.047445

NM_008872.3	circRNA_5367|Chr7:28990988_28991325_+	0.816421979	0.047458
NM_001099314.1	circRNA_2313|Chr15:93452117_93465245_+	0.81637985	0.047479
NM_019866.1	circRNA_3155|Chr19:25061108_25065459_+	0.816368037	0.047485
XM_006533653.3	circRNA_0845|Chr11:29470315_29477458_+	0.816296417	0.047521
NM_145141.2	circRNA_5639|Chr7:132771578_132779385_-	0.8162941	0.047522
NM_031185.3	circRNA_3239|Chr19:45635735_45640521_-	0.816244466	0.047547
NM_009645.2	circRNA_2951|Chr18:39120119_39150118_+	0.816238688	0.04755
NM_001190379.1	circRNA_0537|Chr10:43393871_43395692_+	0.81622775	0.047555
NM_010858.4	circRNA_0611|Chr10:75274017_75279961_+	0.816215837	0.047561
XM_011249157.2	circRNA_0020|Chr1:10315205_10321709_+	0.816202314	0.047568
NM_016982.2	circRNA_4662|Chr5:65301933_65303208_-	0.81613015	0.047604
NM_001204959.1	circRNA_0376|Chr1:172173943_172187460_+	0.816070936	0.047634
XM_006515096.2	circRNA_1399|Chr12:72783683_72786789_+	0.81601942	0.04766
NM_001080979.1	circRNA_6412|ChrMT:13917_14141_-	0.815994994	0.047672
XM_006498287.3	circRNA_3832|Chr2:169883526_169886459_+	0.815994103	0.047672
NM_007763.3	circRNA_5639|Chr7:132771578_132779385_-	0.815836131	0.047751
NM_009135.2	circRNA_3643|Chr2:122441559_122486090_+	0.815835641	0.047752
XM_017314347.1	circRNA_5639|Chr7:132771578_132779385_-	0.815788428	0.047775
NM_173869.3	circRNA_0020|Chr1:10315205_10321709_+	0.815772952	0.047783
NM_029274.2	circRNA_3954|Chr3:88346445_88349444_+	0.815743212	0.047798
XM_011239969.2	circRNA_5311|Chr6:145147636_145149357_+	0.81573822	0.047801
XM_006504940.3	circRNA_1632|Chr13:42055400_42055542_+	0.815673549	0.047833
NM_010809.2	circRNA_3155|Chr19:25061108_25065459_+	0.815636959	0.047851
NM_008623.5	circRNA_0157|Chr1:66801049_66802168_-	0.81562902	0.047855
NM_011169.5	circRNA_3155|Chr19:25061108_25065459_+	0.815538584	0.047901
NM_007529.2	circRNA_6140|Chr9:61935380_61937535_-	0.815513326	0.047913
NM_010809.2	circRNA_4055|Chr3:126796838_126798280_+	0.81550088	0.04792
NM_001310705.1	circRNA_6446|ChrX:42217416_42250037_+	0.81545093	0.047945
XM_006520186.2	circRNA_5639|Chr7:132771578_132779385_-	0.815413604	0.047964
XM_006515096.2	circRNA_3425|Chr2:37624173_37627526_-	0.815315379	0.048013
XM_006515096.2	circRNA_3607|Chr2:119057281_119064097_+	0.815315379	0.048013
NM_001309809.2	circRNA_0157|Chr1:66801049_66802168_-	0.815303089	0.048019
NM_153104.3	circRNA_1980|Chr14:50951404_50963869_+	0.815295728	0.048023
XM_006533569.3	circRNA_5811|Chr8:71992832_71998295_-	0.815285122	0.048028
NM_008940.3	circRNA_4662|Chr5:65301933_65303208_-	0.815263514	0.048039
NM_025711.3	circRNA_4728|Chr5:97027831_97045167_+	0.815231699	0.048055
NM_028472.2	circRNA_0611|Chr10:75274017_75279961_+	0.815164444	0.048089
NM_001109985.1	circRNA_0961|Chr11:67192403_67254873_+	0.815154614	0.048094
NM_007555.4	circRNA_4662|Chr5:65301933_65303208_-	0.815107818	0.048117
NM_001271580.1	circRNA_3702|Chr2:143830084_143832057_-	0.815103497	0.04812
NM_029982.1	circRNA_4487|Chr4:149156607_149161694_-	0.815076758	0.048133
XM_006509465.2	circRNA_6390|Chr9:121727316_121731565_+	0.815070556	0.048136
XM_006527452.3	circRNA_4987|Chr6:4529068_4531010_+	0.815062296	0.04814
XM_006523061.3	circRNA_5311|Chr6:145147636_145149357_+	0.815046495	0.048148
XM_017316439.1	circRNA_1632|Chr13:42055400_42055542_+	0.815008542	0.048167
NM_010090.2	circRNA_3276|Chr19:60563162_60564294_-	0.814965875	0.048189
XM_017315313.1	circRNA_4055|Chr3:126796838_126798280_+	0.814868958	0.048238
XM_006524407.3	circRNA_2313|Chr15:93452117_93465245_+	0.814849003	0.048248
NM_001289875.1	circRNA_3954|Chr3:88346445_88349444_+	0.814848984	0.048248
XM_006533452.3	circRNA_1632|Chr13:42055400_42055542_+	0.814847838	0.048248
NM_011145.3	circRNA_4487|Chr4:149156607_149161694_-	0.81480228	0.048271
NM_001190449.1	circRNA_4728|Chr5:97027831_97045167_+	0.814799233	0.048273
XM_011240755.1	circRNA_4635|Chr5:43758222_43773659_-	0.814746164	0.0483
NM_001161746.1	circRNA_0537|Chr10:43393871_43395692_+	0.814686342	0.04833
NM_001290512.1	circRNA_0962|Chr11:67194348_67220998_+	0.814509284	0.048419
XM_006510297.3	circRNA_4662|Chr5:65301933_65303208_-	0.814476438	0.048436
NM_025288.2	circRNA_3702|Chr2:143830084_143832057_-	0.814422745	0.048463
NM_001142804.1	circRNA_3155|Chr19:25061108_25065459_+	0.814347521	0.048501
XM_017312497.1	circRNA_3702|Chr2:143830084_143832057_-	0.814338732	0.048505

XM_006523711.3	circRNA_0501|Chr10:25283893_25289730_-	0.814313388	0.048518
XM_006523711.3	circRNA_6109|Chr9:57056714_57057861_+	0.814313388	0.048518
XM_006506152.3	circRNA_4487|Chr4:149156607_149161694_-	0.814307427	0.048521
NM_010090.2	circRNA_2888|Chr18:21010677_21020999_+	0.814264364	0.048543
XM_011248602.2	circRNA_5990|Chr9:3475483_3477962_+	0.814259124	0.048546
NM_031185.3	circRNA_1632|Chr13:42055400_42055542_+	0.814255039	0.048548
XM_006518921.3	circRNA_2951|Chr18:39120119_39150118_+	0.814236754	0.048557
NM_145741.2	circRNA_4728|Chr5:97027831_97045167_+	0.814221715	0.048564
XM_006522398.3	circRNA_2888|Chr18:21010677_21020999_+	0.814158177	0.048597
NM_023143.3	circRNA_0501|Chr10:25283893_25289730_-	0.814103389	0.048624
NM_023143.3	circRNA_6109|Chr9:57056714_57057861_+	0.814103389	0.048624
NM_009393.2	circRNA_0537|Chr10:43393871_43395692_+	0.814079294	0.048636
XM_006540134.3	circRNA_6412|ChrMT:13917_14141_-	0.814050893	0.048651
XM_006496905.1	circRNA_0376|Chr1:172173943_172187460_+	0.814039968	0.048656
NM_008760.4	circRNA_4728|Chr5:97027831_97045167_+	0.81403163	0.048661
XM_006498287.3	circRNA_0537|Chr10:43393871_43395692_+	0.813990181	0.048682
NM_173869.3	circRNA_6140|Chr9:61935380_61937535_-	0.813963033	0.048695
NM_001309809.2	circRNA_3702|Chr2:143830084_143832057_-	0.813903569	0.048725
NM_010450.3	circRNA_6446|ChrX:42217416_42250037_+	0.813881882	0.048736
NM_009019.2	circRNA_3239|Chr19:45635735_45640521_-	0.813878546	0.048738
NM_031180.2	circRNA_3276|Chr19:60563162_60564294_-	0.813865151	0.048745
XM_006509465.2	circRNA_0961|Chr11:67192403_67254873_+	0.81385023	0.048752
XM_006511311.2	circRNA_6412|ChrMT:13917_14141_-	0.813718221	0.048819
NM_011704.3	circRNA_6446|ChrX:42217416_42250037_+	0.813717395	0.04882
XM_017318264.1	circRNA_0537|Chr10:43393871_43395692_+	0.813634335	0.048862
NM_007549.2	circRNA_0501|Chr10:25283893_25289730_-	0.81340634	0.048977
NM_007549.2	circRNA_6109|Chr9:57056714_57057861_+	0.81340634	0.048977
XM_017322365.1	circRNA_2951|Chr18:39120119_39150118_+	0.813396069	0.048983
XM_006538374.1	circRNA_4987|Chr6:4529068_4531010_+	0.813372241	0.048995
NM_008966.3	circRNA_0157|Chr1:66801049_66802168_-	0.813340067	0.049011
XM_011245529.2	circRNA_5074|Chr6:40685277_40747150_+	0.813306351	0.049028
NM_173437.2	circRNA_4926|Chr5:143080224_143081077_-	0.813303405	0.04903
XM_011246128.2	circRNA_2951|Chr18:39120119_39150118_+	0.81326999	0.049047
XM_011241948.2	circRNA_5311|Chr6:145147636_145149357_+	0.813253775	0.049055
XM_006541047.3	circRNA_3702|Chr2:143830084_143832057_-	0.813244931	0.049059
NM_207231.1	circRNA_6140|Chr9:61935380_61937535_-	0.813208935	0.049078
NM_011082.3	circRNA_6140|Chr9:61935380_61937535_-	0.813171738	0.049097
XM_017320735.1	circRNA_5367|Chr7:28990988_28991325_+	0.813163818	0.049101
XM_006503665.3	circRNA_0020|Chr1:10315205_10321709_+	0.813134112	0.049116
XM_011249157.2	circRNA_0376|Chr1:172173943_172187460_+	0.813127115	0.049119
XM_006510077.3	circRNA_2888|Chr18:21010677_21020999_+	0.813118177	0.049124
XM_006533452.3	circRNA_3643|Chr2:122441559_122486090_+	0.813105585	0.04913
XM_006511007.3	circRNA_5074|Chr6:40685277_40747150_+	0.813085415	0.04914
NM_009514.4	circRNA_0020|Chr1:10315205_10321709_+	0.813062428	0.049152
XM_006506808.3	circRNA_6390|Chr9:121727316_121731565_+	0.813047768	0.04916
XM_006506152.3	circRNA_5074|Chr6:40685277_40747150_+	0.813020487	0.049173
NM_178715.3	circRNA_0020|Chr1:10315205_10321709_+	0.812949131	0.04921
NM_001190325.1	circRNA_5639|Chr7:132771578_132779385_-	0.81290497	0.049232
NM_008804.4	circRNA_3643|Chr2:122441559_122486090_+	0.812845588	0.049262
XM_011243586.2	circRNA_4662|Chr5:65301933_65303208_-	0.81284114	0.049265
XM_006510077.3	circRNA_6446|ChrX:42217416_42250037_+	0.812784286	0.049294
XM_006525028.2	circRNA_3276|Chr19:60563162_60564294_-	0.812617112	0.049379
NM_024283.3	circRNA_4055|Chr3:126796838_126798280_+	0.812530063	0.049423
XM_006527221.3	circRNA_0157|Chr1:66801049_66802168_-	0.81245097	0.049463
XM_011238891.1	circRNA_0611|Chr10:75274017_75279961_+	0.812361663	0.049509
NM_181315.4	circRNA_5311|Chr6:145147636_145149357_+	0.812351509	0.049514
XM_017316715.1	circRNA_0537|Chr10:43393871_43395692_+	0.812240338	0.049571
NM_177789.4	circRNA_2951|Chr18:39120119_39150118_+	0.812187302	0.049598
XM_006532024.3	circRNA_3702|Chr2:143830084_143832057_-	0.812179455	0.049602

NM_010809.2	circRNA_6446|ChrX:42217416_42250037_+	0.812177927	0.049603
NM_019696.2	circRNA_0020|Chr1:10315205_10321709_+	0.812150781	0.049617
XM_017315313.1	circRNA_0020|Chr1:10315205_10321709_+	0.812146384	0.049619
NM_173427.2	circRNA_2888|Chr18:21010677_21020999_+	0.812136463	0.049624
NM_172907.3	circRNA_4728|Chr5:97027831_97045167_+	0.812120947	0.049632
XM_011240476.2	circRNA_3003|Chr18:67545615_67587859_-	0.812075525	0.049655
XM_011248576.2	circRNA_3954|Chr3:88346445_88349444_+	0.812062213	0.049662
NM_010174.1	circRNA_3832|Chr2:169883526_169886459_+	0.812047308	0.049669
NM_027052.3	circRNA_0962|Chr11:67194348_67220998_+	0.812001019	0.049693
XM_006520186.2	circRNA_2888|Chr18:21010677_21020999_+	0.811987935	0.0497
XM_011248394.1	circRNA_0376|Chr1:172173943_172187460_+	0.811975834	0.049706
XM_011240779.2	circRNA_0157|Chr1:66801049_66802168_-	0.811959864	0.049714
XM_006508990.2	circRNA_0961|Chr11:67192403_67254873_+	0.811868423	0.049761
NM_001024731.2	circRNA_3702|Chr2:143830084_143832057_-	0.811813098	0.049789
XM_006508990.2	circRNA_3954|Chr3:88346445_88349444_+	0.811733221	0.04983
NM_010181.2	circRNA_0845|Chr11:29470315_29477458_+	0.811701909	0.049846
NM_001190379.1	circRNA_0961|Chr11:67192403_67254873_+	0.811684312	0.049855
NM_177709.3	circRNA_4055|Chr3:126796838_126798280_+	0.811609485	0.049893
NM_015744.4	circRNA_5639|Chr7:132771578_132779385_-	0.811598408	0.049899
NM_001033167.3	circRNA_5990|Chr9:3475483_3477962_+	0.811585867	0.049905
NM_001190448.1	circRNA_5311|Chr6:145147636_145149357_+	0.811523614	0.049937
XM_017318541.1	circRNA_2888|Chr18:21010677_21020999_+	0.811512743	0.049943
NM_008524.2	circRNA_0611|Chr10:75274017_75279961_+	0.811437513	0.049981
NM_146125.2	circRNA_4662|Chr5:65301933_65303208_-	0.811425072	0.049988
NM_145635.2	circRNA_0157|Chr1:66801049_66802168_-	0.811413564	0.049994

Table S7 MRE analysis of mRNA and miRNA

miRNA id	mRNA id	score	energy	miRNA_location	mRNA_location	MRE_length
mmu-miR-206-3p	NM_029685.1	141.00	-12.98	2 18	208 229	16
mmu-miR-206-3p	XM_006534171.3	142.00	-20.31	2 19	440 461	17
mmu-miR-206-3p	XM_006515980.3	154.00	-18.26	2 18	2756 2775	16
mmu-miR-206-3p	NM_001142804.1	141.00	-11.01	2 15	1092 1114	14
mmu-miR-206-3p	XM_006498086.3	140.00	-12.85	2 9	2874 2895	7
mmu-miR-206-3p	NM_013906.3	145.00	-15.54	2 10	3888 3909	8
mmu-miR-206-3p	NM_009605.4	142.00	-14.77	2 21	564 588	22
mmu-miR-206-3p	NM_001291930.1	144.00	-15.63	2 13	4306 4327	11
mmu-miR-206-3p	NM_008032.3	155.00	-17.75	2 18	1253 1273	16
mmu-miR-206-3p	NM_153178.4	140.00	-8.48	2 9	2340 2361	7
mmu-miR-206-3p	XM_006498287.3	143.00	-16.79	2 21	366 388	20
mmu-miR-206-3p	NM_031185.3	142.00	-9.70	2 11	5725 5746	9
mmu-miR-206-3p	NM_031185.3	140.00	-12.28	2 19	4536 4556	17
mmu-miR-206-3p	XM_006508990.2	140.00	-15.66	2 21	5778 5799	19
mmu-miR-206-3p	XM_017318264.1	140.00	-10.08	2 9	474 495	7
mmu-miR-206-3p	NM_009675.2	140.00	-15.29	2 18	4232 4254	17
mmu-miR-206-3p	NM_001080819.1	148.00	-13.87	2 21	4731 4752	19
mmu-miR-206-3p	NM_001080819.1	144.00	-14.78	2 13	3581 3602	11
mmu-miR-206-3p	NM_207231.1	140.00	-11.26	2 9	1256 1277	7
mmu-miR-206-3p	NM_025711.3	146.00	-20.45	2 21	555 580	23
mmu-miR-206-3p	NM_001310070.1	155.00	-16.02	2 20	1122 1143	18
mmu-miR-206-3p	NM_013863.5	145.00	-14.14	2 10	546 567	8
mmu-miR-206-3p	XM_006502291.3	150.00	-15.60	2 17	4546 4566	15
mmu-miR-206-3p	XM_006510701.3	144.00	-15.72	2 19	1153 1173	17
mmu-miR-206-3p	XM_006510701.3	143.00	-16.01	2 14	4783 4803	12
mmu-miR-206-3p	NM_007555.4	140.00	-19.06	2 21	2920 2941	19
mmu-miR-206-3p	NM_020508.4	152.00	-16.24	2 18	3463 3485	17
mmu-miR-206-3p	XM_006524328.3	140.00	-13.45	2 9	1555 1576	7
mmu-miR-206-3p	NM_023143.3	152.00	-17.58	2 18	26 49	18
mmu-miR-206-3p	NM_001285867.1	148.00	-19.39	2 21	3479 3500	19
mmu-miR-206-3p	XM_011246128.2	142.00	-18.47	2 20	8684 8707	20
mmu-miR-206-3p	XM_011246128.2	141.00	-15.94	2 20	2170 2193	20
mmu-miR-206-3p	NM_007588.2	146.00	-19.37	2 21	20 40	19
mmu-miR-206-3p	NM_181315.4	140.00	-10.81	2 21	1165 1186	19
mmu-miR-206-3p	XM_006503371.2	142.00	-18.86	2 20	1674 1696	19
mmu-miR-206-3p	XM_006530131.1	146.00	-13.16	2 19	6716 6737	17
mmu-miR-206-3p	XM_006532120.2	144.00	-20.06	2 13	174 195	11
mmu-miR-206-3p	NM_001004357.2	144.00	-12.15	2 21	2680 2701	19
mmu-miR-206-3p	NM_001004357.2	144.00	-16.70	2 21	4386 4407	19
mmu-miR-206-3p	NM_001004357.2	143.00	-22.95	2 20	2655 2676	18
mmu-miR-206-3p	NM_001004357.2	141.00	-19.85	2 21	306 327	20
mmu-miR-206-3p	NM_009930.2	154.00	-17.18	2 19	4874 4895	17
mmu-miR-206-3p	NM_009930.2	143.00	-20.01	2 16	543 564	14
mmu-miR-206-3p	XM_011240755.1	148.00	-11.16	2 21	4156 4177	19
mmu-miR-206-3p	XM_011248602.2	146.00	-12.50	2 21	4066 4086	19
mmu-miR-206-3p	XM_011248602.2	142.00	-19.04	2 20	1710 1734	21
mmu-miR-206-3p	XM_011248602.2	142.00	-18.74	2 20	3013 3036	20
mmu-miR-206-3p	XM_011242311.2	147.00	-12.87	2 15	5596 5615	13
mmu-miR-206-3p	NM_030206.4	148.00	-15.59	2 21	1609 1630	19
mmu-miR-206-3p	NM_015814.2	144.00	-13.93	2 21	1289 1310	19
mmu-miR-206-3p	NM_009345.2	140.00	-10.59	2 9	27 48	7
mmu-miR-206-3p	NM_027293.1	141.00	-13.31	2 18	5443 5464	16
mmu-miR-206-3p	XM_011243391.2	140.00	-17.25	2 21	2897 2918	19
mmu-miR-206-3p	NM_019759.2	150.00	-16.28	2 20	805 827	19
mmu-miR-206-3p	NM_007883.3	140.00	-16.02	2 9	15 36	7
mmu-miR-206-3p	XM_006514873.2	141.00	-13.63	2 20	3281 3301	18
mmu-miR-206-3p	NM_001160049.1	146.00	-15.23	2 19	716 737	17
mmu-miR-206-3p	NM_019397.3	147.00	-19.74	2 20	20 41	18
mmu-miR-206-3p	NM_178676.4	152.00	-17.33	2 21	2745 2766	19
mmu-miR-206-3p	NM_010137.3	144.00	-15.63	2 18	3325 3349	19
mmu-miR-206-3p	NM_010137.3	141.00	-17.11	2 21	2339 2358	19
mmu-miR-206-3p	XM_011238916.2	153.00	-21.73	2 18	1871 1892	16
mmu-miR-214-3p	NM_024283.3	158.00	-23.39	2 21	144 164	19

mmu-miR-214-3p	NM_024283.3	143.00	-19.63	3 16	745 766	13
mmu-miR-214-3p	XM_017320219.1	159.00	-21.96	2 17	4722 4745	17
mmu-miR-214-3p	XM_017320219.1	140.00	-13.62	2 9	1523 1544	7
mmu-miR-214-3p	NM_144544.2	142.00	-21.59	3 21	467 487	18
mmu-miR-214-3p	XM_006534171.3	140.00	-17.05	2 9	780 801	7
mmu-miR-214-3p	XM_011241948.2	156.00	-25.22	2 14	1396 1418	13
mmu-miR-214-3p	XM_011241322.1	151.00	-21.71	2 16	139 160	14
mmu-miR-214-3p	XM_011241322.1	147.00	-14.91	2 12	2815 2836	10
mmu-miR-214-3p	XM_006515980.3	142.00	-17.47	2 19	1366 1384	17
mmu-miR-214-3p	NM_138955.3	151.00	-21.12	2 19	3330 3351	18
mmu-miR-214-3p	NM_138955.3	144.00	-16.72	2 21	450 471	19
mmu-miR-214-3p	XM_006540566.1	143.00	-22.23	2 20	5651 5672	18
mmu-miR-214-3p	XM_006540566.1	142.00	-13.79	2 11	286 307	9
mmu-miR-214-3p	XM_006498086.3	157.00	-22.83	2 20	244 264	18
mmu-miR-214-3p	NM_021475.2	147.00	-21.22	2 20	2132 2150	18
mmu-miR-214-3p	NM_021475.2	141.00	-18.32	3 20	61 84	19
mmu-miR-214-3p	NM_013906.3	149.00	-19.68	2 14	119 140	12
mmu-miR-214-3p	NM_013906.3	147.00	-19.48	2 21	3546 3568	20
mmu-miR-214-3p	NM_013906.3	146.00	-17.59	2 21	301 321	19
mmu-miR-214-3p	XM_006538374.1	165.00	-24.34	2 20	1321 1341	18
mmu-miR-214-3p	XM_006538374.1	144.00	-19.79	2 19	499 522	19
mmu-miR-214-3p	XM_006538374.1	140.00	-15.01	2 9	7126 7147	7
mmu-miR-214-3p	XM_006505388.2	159.00	-23.14	2 21	1230 1252	20
mmu-miR-214-3p	XM_006505388.2	154.00	-24.86	2 21	6796 6816	19
mmu-miR-214-3p	XM_006505388.2	151.00	-22.63	2 19	349 368	17
mmu-miR-214-3p	XM_006505388.2	143.00	-18.32	2 20	2422 2443	18
mmu-miR-214-3p	XM_006505388.2	140.00	-13.38	2 9	5082 5103	7
mmu-miR-214-3p	NM_001291930.1	148.00	-14.66	2 21	2475 2496	19
mmu-miR-214-3p	XM_017312667.1	145.00	-17.49	2 18	6361 6382	16
mmu-miR-214-3p	NM_008032.3	154.00	-20.78	2 21	1046 1066	19
mmu-miR-214-3p	NM_153178.4	150.00	-16.26	2 19	4357 4378	17
mmu-miR-214-3p	NM_153178.4	143.00	-16.61	2 16	4605 4626	14
mmu-miR-214-3p	NM_153178.4	142.00	-15.82	3 21	6307 6327	18
mmu-miR-214-3p	NM_153178.4	141.00	-17.43	2 21	4914 4935	20
mmu-miR-214-3p	NM_153178.4	140.00	-14.54	2 9	1885 1906	7
mmu-miR-214-3p	NM_153178.4	140.00	-20.20	3 20	3649 3670	18
mmu-miR-214-3p	XM_011250176.1	147.00	-19.63	2 12	342 363	10
mmu-miR-214-3p	XM_011250176.1	141.00	-21.61	2 20	593 613	18
mmu-miR-214-3p	XM_011250176.1	141.00	-19.90	2 20	6193 6213	18
mmu-miR-214-3p	NM_009645.2	143.00	-21.05	2 17	858 881	17
mmu-miR-214-3p	NM_008012.1	150.00	-21.84	2 11	950 971	9
mmu-miR-214-3p	NM_053080.3	140.00	-15.05	2 9	600 621	7
mmu-miR-214-3p	XM_006532024.3	150.00	-19.49	2 20	1641 1663	19
mmu-miR-214-3p	XM_006495613.3	159.00	-22.23	2 21	154 176	20
mmu-miR-214-3p	XM_006508990.2	149.00	-20.14	2 14	1948 1969	12
mmu-miR-214-3p	XM_006508990.2	143.00	-14.70	2 17	1253 1276	17
mmu-miR-214-3p	XM_006508990.2	140.00	-14.22	2 9	1156 1177	7
mmu-miR-214-3p	XM_006508990.2	140.00	-15.48	2 9	1849 1870	7
mmu-miR-214-3p	NM_172790.2	175.00	-31.56	2 21	5594 5616	20
mmu-miR-214-3p	NM_172790.2	170.00	-32.29	2 21	2303 2323	19
mmu-miR-214-3p	NM_172790.2	148.00	-23.68	2 21	4334 4355	19
mmu-miR-214-3p	NM_172790.2	146.00	-22.97	2 21	5122 5145	21
mmu-miR-214-3p	NM_172790.2	142.00	-20.68	2 16	3021 3043	15
mmu-miR-214-3p	NM_172790.2	140.00	-13.87	2 9	5338 5359	7
mmu-miR-214-3p	NM_009675.2	146.00	-17.03	2 19	4227 4248	17
mmu-miR-214-3p	NM_009675.2	144.00	-17.45	2 19	2810 2830	17
mmu-miR-214-3p	NM_013912.3	141.00	-24.79	2 21	2159 2181	21
mmu-miR-214-3p	NM_007482.3	151.00	-17.09	2 19	742 763	18
mmu-miR-214-3p	NM_001080819.1	154.00	-19.54	2 20	3110 3134	21
mmu-miR-214-3p	NM_001080819.1	152.00	-19.59	2 19	2136 2156	17
mmu-miR-214-3p	NM_001080819.1	149.00	-19.55	2 14	6025 6046	12
mmu-miR-214-3p	NM_001080819.1	142.00	-17.39	3 20	5499 5521	18
mmu-miR-214-3p	NM_029823.2	142.00	-16.48	2 11	134 155	9
mmu-miR-214-3p	NM_023048.5	149.00	-19.37	2 19	913 935	18
mmu-miR-214-3p	NM_023048.5	146.00	-21.11	2 19	727 748	17

mmu-miR-214-3p	NM_001310070.1	140.00	-16.45	3 19	798 818	16
mmu-miR-214-3p	NM_001290469.1	161.00	-21.29	2 20	440 460	18
mmu-miR-214-3p	NM_009722.3	155.00	-26.31	2 21	1458 1481	21
mmu-miR-214-3p	NM_009722.3	147.00	-14.94	2 12	1004 1025	10
mmu-miR-214-3p	XM_006529727.2	156.00	-22.80	2 19	7972 7992	17
mmu-miR-214-3p	XM_006529727.2	152.00	-24.41	2 20	8080 8103	21
mmu-miR-214-3p	XM_006529727.2	148.00	-17.07	2 14	1508 1530	13
mmu-miR-214-3p	XM_006529727.2	143.00	-13.95	2 16	7231 7252	14
mmu-miR-214-3p	XM_006529727.2	141.00	-19.92	2 18	7521 7542	16
mmu-miR-214-3p	XM_006507725.2	145.00	-17.46	2 15	2958 2980	14
mmu-miR-214-3p	NM_001159407.1	150.00	-17.94	2 21	4682 4702	19
mmu-miR-214-3p	NM_001159407.1	149.00	-21.43	2 21	4622 4643	20
mmu-miR-214-3p	NM_013863.5	147.00	-18.43	2 17	492 514	16
mmu-miR-214-3p	NM_013863.5	141.00	-16.09	2 14	1349 1370	12
mmu-miR-214-3p	NM_007529.2	165.00	-27.38	2 21	283 302	19
mmu-miR-214-3p	XM_006502291.3	153.00	-28.33	2 19	3414 3437	19
mmu-miR-214-3p	XM_006502291.3	147.00	-26.04	2 20	45 66	18
mmu-miR-214-3p	XM_006502291.3	147.00	-18.12	2 12	4736 4757	10
mmu-miR-214-3p	XM_006510701.3	155.00	-24.23	2 17	5871 5896	19
mmu-miR-214-3p	XM_006510701.3	144.00	-17.54	2 20	1018 1039	19
mmu-miR-214-3p	XM_006510701.3	140.00	-16.35	2 9	4676 4697	7
mmu-miR-214-3p	NM_178782.4	156.00	-24.12	2 21	2401 2420	19
mmu-miR-214-3p	NM_178782.4	154.00	-20.92	2 18	5448 5469	17
mmu-miR-214-3p	NM_178782.4	152.00	-19.13	2 21	4724 4745	19
mmu-miR-214-3p	NM_178782.4	143.00	-19.61	3 21	1114 1137	20
mmu-miR-214-3p	NM_178782.4	140.00	-14.16	2 9	4094 4115	7
mmu-miR-214-3p	NM_001002896.2	147.00	-18.80	2 20	616 634	18
mmu-miR-214-3p	NM_001002896.2	142.00	-15.93	2 19	1312 1333	17
mmu-miR-214-3p	NM_001002896.2	140.00	-14.04	2 9	1261 1282	7
mmu-miR-214-3p	NM_007549.2	145.00	-22.57	2 21	2352 2371	19
mmu-miR-214-3p	NM_007549.2	140.00	-20.65	3 13	2028 2049	10
mmu-miR-214-3p	NM_007555.4	171.00	-25.99	2 20	29 50	18
mmu-miR-214-3p	NM_028472.2	155.00	-22.88	2 20	616 637	18
mmu-miR-214-3p	NM_028472.2	147.00	-20.17	2 12	850 871	10
mmu-miR-214-3p	NM_028472.2	147.00	-17.95	2 12	1024 1045	10
mmu-miR-214-3p	NM_020508.4	143.00	-16.76	2 14	931 951	12
mmu-miR-214-3p	NM_020508.4	140.00	-23.90	2 21	222 243	19
mmu-miR-214-3p	XM_006524328.3	158.00	-29.92	2 21	5784 5804	19
mmu-miR-214-3p	XM_006524328.3	154.00	-19.16	2 15	2417 2438	13
mmu-miR-214-3p	XM_006524328.3	150.00	-20.99	2 11	1011 1032	9
mmu-miR-214-3p	NM_178692.3	150.00	-20.77	2 15	2654 2675	13
mmu-miR-214-3p	NM_178692.3	143.00	-16.09	2 21	2138 2160	20
mmu-miR-214-3p	NM_178692.3	140.00	-13.24	2 9	2036 2057	7
mmu-miR-214-3p	XM_006503665.3	164.00	-23.09	2 18	3023 3045	17
mmu-miR-214-3p	NM_001285867.1	163.00	-27.21	2 21	1889 1911	20
mmu-miR-214-3p	NM_001285867.1	149.00	-17.56	2 14	3067 3088	12
mmu-miR-214-3p	XM_011246128.2	147.00	-25.28	2 21	8548 8570	20
mmu-miR-214-3p	NM_007588.2	152.00	-20.15	2 19	1753 1773	17
mmu-miR-214-3p	NM_007588.2	145.00	-18.75	2 20	217 241	21
mmu-miR-214-3p	NM_007588.2	141.00	-16.42	2 18	621 642	16
mmu-miR-214-3p	NM_007588.2	140.00	-19.74	2 15	3095 3115	13
mmu-miR-214-3p	NM_001190379.1	151.00	-15.25	2 17	3308 3330	16
mmu-miR-214-3p	XM_011246479.2	154.00	-16.25	2 19	1100 1121	17
mmu-miR-214-3p	XM_011246479.2	141.00	-17.62	2 21	1483 1504	20
mmu-miR-214-3p	XM_006501443.3	152.00	-21.83	2 13	937 958	11
mmu-miR-214-3p	XM_006501443.3	144.00	-25.02	2 21	1662 1683	19
mmu-miR-214-3p	XM_006520253.3	165.00	-26.63	2 19	4185 4207	18
mmu-miR-214-3p	XM_006520253.3	153.00	-19.52	2 20	2772 2795	20
mmu-miR-214-3p	XM_006520253.3	144.00	-15.55	2 19	2241 2261	17
mmu-miR-214-3p	XM_006500947.3	148.00	-20.48	2 21	2152 2173	19
mmu-miR-214-3p	NM_009824.2	157.00	-22.78	2 19	974 997	19
mmu-miR-214-3p	NM_009824.2	142.00	-23.08	3 16	6705 6728	15
mmu-miR-214-3p	NM_009824.2	140.00	-17.77	3 19	2017 2037	16
mmu-miR-214-3p	NM_019626.3	146.00	-23.85	3 21	1921 1946	22
mmu-miR-214-3p	XM_006526449.3	154.00	-21.80	2 20	434 456	19

mmu-miR-214-3p	XM_006526449.3	152.00	-27.20	2 21	455 479	23
mmu-miR-214-3p	NM_001001178.1	140.00	-12.72	2 9	3159 3180	7
mmu-miR-214-3p	NM_028804.1	151.00	-31.36	2 21	1001 1023	20
mmu-miR-214-3p	NM_001013784.1	157.00	-24.61	2 20	2012 2032	18
mmu-miR-214-3p	NM_001013784.1	146.00	-20.73	3 19	2639 2660	16
mmu-miR-214-3p	XM_006509966.3	148.00	-18.64	2 21	510 531	19
mmu-miR-214-3p	XM_006509966.3	140.00	-15.80	2 9	963 984	7
mmu-miR-214-3p	NM_001110322.1	141.00	-16.74	2 11	1105 1127	10
mmu-miR-214-3p	NM_001110322.1	140.00	-15.14	2 9	612 633	7
mmu-miR-214-3p	NM_007655.3	142.00	-14.71	2 19	1128 1149	17
mmu-miR-214-3p	NM_001313939.1	170.00	-28.04	2 21	113 133	19
mmu-miR-214-3p	XM_011245344.2	148.00	-22.37	2 20	411 437	23
mmu-miR-214-3p	XM_011245344.2	145.00	-17.29	2 15	2543 2565	14
mmu-miR-214-3p	XM_011245344.2	142.00	-16.13	2 21	9310 9330	19
mmu-miR-214-3p	XM_011245344.2	140.00	-15.36	2 9	7193 7214	7
mmu-miR-214-3p	XM_011245344.2	140.00	-13.71	2 9	10165 10186	7
mmu-miR-214-3p	XM_006503371.2	152.00	-15.78	2 21	1822 1843	19
mmu-miR-214-3p	XM_006503371.2	147.00	-13.71	2 20	445 466	18
mmu-miR-214-3p	XM_006503371.2	147.00	-18.45	2 12	1225 1246	10
mmu-miR-214-3p	NM_013459.3	171.00	-33.30	2 21	47 69	20
mmu-miR-214-3p	NM_013459.3	151.00	-21.57	2 19	35 54	17
mmu-miR-214-3p	NM_013459.3	144.00	-18.92	2 19	216 242	22
mmu-miR-214-3p	XM_006523030.3	154.00	-26.58	2 21	1199 1223	22
mmu-miR-214-3p	XM_006523030.3	146.00	-23.99	2 21	3296 3320	22
mmu-miR-214-3p	XM_006523030.3	143.00	-20.31	2 21	468 491	21
mmu-miR-214-3p	NM_001301295.1	150.00	-19.83	2 21	382 406	22
mmu-miR-214-3p	XM_006530131.1	159.00	-23.53	2 21	7806 7828	20
mmu-miR-214-3p	XM_006530131.1	148.00	-20.01	2 14	8373 8395	13
mmu-miR-214-3p	XM_006530131.1	142.00	-21.62	2 21	1102 1126	22
mmu-miR-214-3p	XM_006530131.1	142.00	-21.92	2 19	1291 1312	17
mmu-miR-214-3p	XM_006530131.1	142.00	-18.53	2 15	5690 5711	13
mmu-miR-214-3p	XM_006530131.1	142.00	-15.14	2 11	5770 5791	9
mmu-miR-214-3p	XM_006530131.1	140.00	-14.06	2 9	4926 4947	7
mmu-miR-214-3p	XM_006527480.3	152.00	-19.88	2 19	4192 4212	17
mmu-miR-214-3p	XM_006527480.3	150.00	-21.46	2 20	730 753	20
mmu-miR-214-3p	XM_006527480.3	148.00	-19.74	2 20	690 711	19
mmu-miR-214-3p	XM_006527480.3	146.00	-25.51	3 20	814 837	19
mmu-miR-214-3p	XM_006527480.3	140.00	-17.85	2 9	771 792	7
mmu-miR-214-3p	NM_001004357.2	151.00	-21.52	2 21	1021 1043	20
mmu-miR-214-3p	NM_001004357.2	141.00	-18.25	2 19	31 57	22
mmu-miR-214-3p	NM_001004357.2	140.00	-15.80	2 9	3612 3633	7
mmu-miR-214-3p	XM_017314944.1	151.00	-18.95	2 17	1803 1826	17
mmu-miR-214-3p	XM_017314944.1	141.00	-17.26	2 18	15 36	16
mmu-miR-214-3p	XM_011248983.2	165.00	-29.05	2 14	524 545	12
mmu-miR-214-3p	XM_011248983.2	147.00	-25.47	2 20	563 581	18
mmu-miR-214-3p	NM_001113515.2	157.00	-21.24	2 21	3481 3500	19
mmu-miR-214-3p	NM_001113515.2	155.00	-17.47	2 20	1022 1043	18
mmu-miR-214-3p	NM_001113515.2	154.00	-21.22	2 20	3244 3266	19
mmu-miR-214-3p	NM_001113515.2	152.00	-20.37	2 19	753 773	17
mmu-miR-214-3p	NM_001113515.2	151.00	-18.46	2 16	1436 1457	14
mmu-miR-214-3p	NM_001113515.2	151.00	-19.75	2 20	2777 2798	18
mmu-miR-214-3p	NM_001113515.2	151.00	-17.17	2 20	3317 3338	18
mmu-miR-214-3p	NM_001113515.2	149.00	-22.41	2 14	2507 2528	12
mmu-miR-214-3p	NM_001113515.2	147.00	-19.27	2 20	1958 1979	18
mmu-miR-214-3p	NM_009930.2	159.00	-24.68	2 21	2740 2762	20
mmu-miR-214-3p	NM_009930.2	155.00	-21.41	2 20	2012 2033	18
mmu-miR-214-3p	NM_009930.2	154.00	-24.06	2 21	3778 3798	19
mmu-miR-214-3p	NM_009930.2	152.00	-20.92	2 19	870 890	17
mmu-miR-214-3p	NM_009930.2	149.00	-17.47	2 14	3398 3419	12
mmu-miR-214-3p	NM_009930.2	147.00	-17.55	2 20	2615 2636	18
mmu-miR-214-3p	NM_009930.2	141.00	-19.29	2 12	2481 2501	10
mmu-miR-214-3p	NM_009930.2	141.00	-17.94	2 14	3425 3446	12
mmu-miR-214-3p	NM_009930.2	140.00	-12.63	2 9	2318 2339	7
mmu-miR-214-3p	NM_009930.2	140.00	-19.79	2 21	5209 5227	19
mmu-miR-214-3p	XM_011240755.1	149.00	-22.39	2 21	587 609	21

mmu-miR-214-3p	XM_011240755.1	143.00	-20.98	2 21	536 558	20
mmu-miR-214-3p	XM_011240755.1	140.00	-18.77	2 9	2575 2596	7
mmu-miR-214-3p	XM_006532938.2	144.00	-25.25	2 21	2632 2653	19
mmu-miR-214-3p	XM_011247208.1	166.00	-26.81	2 21	6506 6530	22
mmu-miR-214-3p	XM_011247208.1	148.00	-18.22	2 20	1660 1681	19
mmu-miR-214-3p	XM_011247208.1	141.00	-15.88	2 14	5156 5177	12
mmu-miR-214-3p	XM_011248602.2	147.00	-25.96	2 20	680 701	18
mmu-miR-214-3p	XM_011248602.2	140.00	-15.54	2 17	4096 4117	15
mmu-miR-214-3p	NM_019696.2	145.00	-15.75	2 10	835 856	8
mmu-miR-214-3p	XM_011242311.2	161.00	-25.96	2 21	2831 2852	20
mmu-miR-214-3p	XM_011242311.2	147.00	-17.08	2 21	5291 5315	22
mmu-miR-214-3p	NM_001289782.1	145.00	-18.31	2 10	933 954	8
mmu-miR-214-3p	XM_006517308.3	155.00	-23.89	2 20	378 399	18
mmu-miR-214-3p	NM_007817.2	142.00	-20.21	2 21	189 214	23
mmu-miR-214-3p	NM_007817.2	142.00	-15.15	2 11	523 544	9
mmu-miR-214-3p	NM_007817.2	140.00	-13.04	2 9	928 949	7
mmu-miR-214-3p	XM_006540596.2	140.00	-14.61	2 9	553 574	7
mmu-miR-214-3p	NM_001190451.2	158.00	-27.11	2 19	1138 1159	17
mmu-miR-214-3p	NM_001190448.1	140.00	-17.37	2 9	867 888	7
mmu-miR-214-3p	NM_015814.2	146.00	-24.05	2 21	773 797	22
mmu-miR-214-3p	NM_010055.3	148.00	-17.47	2 17	2398 2419	15
mmu-miR-214-3p	NM_010055.3	145.00	-20.46	2 20	2038 2058	18
mmu-miR-214-3p	NM_152915.1	167.00	-28.89	2 21	532 554	20
mmu-miR-214-3p	NM_152915.1	140.00	-19.03	2 21	1190 1211	19
mmu-miR-214-3p	NM_009345.2	143.00	-16.80	2 17	581 604	17
mmu-miR-214-3p	NM_027293.1	165.00	-25.64	2 21	1699 1718	19
mmu-miR-214-3p	NM_027293.1	153.00	-27.30	2 21	2432 2453	20
mmu-miR-214-3p	NM_027293.1	150.00	-20.91	2 11	6945 6966	9
mmu-miR-214-3p	NM_027293.1	146.00	-20.14	2 21	624 644	19
mmu-miR-214-3p	NM_027293.1	145.00	-15.93	2 10	4295 4316	8
mmu-miR-214-3p	NM_027293.1	144.00	-17.65	2 13	3167 3188	11
mmu-miR-214-3p	NM_027293.1	141.00	-20.68	2 21	1282 1301	19
mmu-miR-214-3p	XM_011243391.2	160.00	-24.02	2 21	4147 4168	19
mmu-miR-214-3p	XM_011243391.2	158.00	-20.16	2 19	5233 5254	17
mmu-miR-214-3p	XM_011243391.2	153.00	-24.86	2 21	7186 7208	21
mmu-miR-214-3p	XM_011243391.2	152.00	-21.64	2 14	6611 6633	13
mmu-miR-214-3p	XM_011243391.2	151.00	-18.22	2 16	6969 6990	14
mmu-miR-214-3p	XM_011243391.2	150.00	-24.03	3 20	5691 5713	18
mmu-miR-214-3p	XM_011243391.2	142.00	-28.16	2 21	5277 5302	23
mmu-miR-214-3p	NM_019759.2	151.00	-16.08	2 17	409 432	17
mmu-miR-214-3p	NM_019759.2	141.00	-26.47	2 19	910 932	18
mmu-miR-214-3p	XM_006506808.3	142.00	-16.84	2 20	1522 1544	19
mmu-miR-214-3p	NM_001317365.1	140.00	-19.96	3 21	3048 3069	18
mmu-miR-214-3p	NM_007883.3	145.00	-17.69	2 21	227 248	20
mmu-miR-214-3p	XM_006514873.2	140.00	-18.91	2 19	3534 3558	20
mmu-miR-214-3p	NM_010090.2	150.00	-21.12	2 19	537 555	17
mmu-miR-214-3p	NM_010090.2	141.00	-19.11	2 20	402 422	18
mmu-miR-214-3p	NM_010090.2	140.00	-20.64	2 17	697 718	15
mmu-miR-214-3p	NM_001160049.1	143.00	-20.90	2 20	372 393	18
mmu-miR-214-3p	XM_011245824.2	140.00	-14.77	2 9	1686 1707	7
mmu-miR-214-3p	XM_006528026.2	146.00	-21.84	2 20	4254 4276	19
mmu-miR-214-3p	NM_133643.4	150.00	-19.54	2 20	7427 7449	19
mmu-miR-214-3p	NM_133643.4	148.00	-20.29	2 21	420 439	19
mmu-miR-214-3p	NM_133643.4	141.00	-19.41	2 17	5342 5363	16
mmu-miR-214-3p	NM_133643.4	140.00	-16.68	3 19	5778 5798	16
mmu-miR-214-3p	NM_019397.3	148.00	-18.56	2 21	1439 1458	19
mmu-miR-214-3p	NM_019397.3	146.00	-18.28	2 13	317 337	11
mmu-miR-214-3p	NM_001291145.1	154.00	-21.49	2 19	934 955	17
mmu-miR-214-3p	NM_001291145.1	147.00	-21.18	2 21	1137 1161	22
mmu-miR-214-3p	NM_001291145.1	140.00	-17.92	2 21	2104 2125	19
mmu-miR-214-3p	NM_178676.4	154.00	-24.02	2 18	2567 2586	16
mmu-miR-214-3p	NM_178676.4	143.00	-20.64	2 20	493 514	18
mmu-miR-214-3p	NM_010137.3	140.00	-21.81	2 9	1056 1077	7
mmu-miR-214-3p	NM_010137.3	140.00	-15.81	2 9	4694 4715	7
mmu-miR-214-3p	XM_006511221.3	151.00	-17.61	2 20	3479 3500	18

mmu-miR-214-3p	XM_006511221.3	151.00	-22.71	2 21	4563 4585	20
mmu-miR-214-3p	XM_006511221.3	150.00	-21.00	2 21	1766 1790	22
mmu-miR-214-3p	XM_006511221.3	145.00	-19.04	2 20	1860 1880	18
mmu-miR-214-3p	XM_006511221.3	145.00	-15.85	2 10	1898 1919	8
mmu-miR-214-3p	XM_006511221.3	141.00	-15.64	2 12	4412 4432	10
mmu-miR-378a-5p	NM_001271580.1	146.00	-32.89	2 20	266 288	19
mmu-miR-378a-5p	XM_006515980.3	143.00	-27.21	2 21	1861 1883	20
mmu-miR-378a-5p	XM_006540566.1	171.00	-27.28	2 21	2088 2111	21
mmu-miR-378a-5p	XM_006540566.1	140.00	-24.21	2 21	5900 5921	19
mmu-miR-378a-5p	NM_153151.3	148.00	-21.42	2 17	485 506	15
mmu-miR-378a-5p	NM_172580.1	159.00	-34.27	2 21	210 232	20
mmu-miR-378a-5p	NM_009608.4	142.00	-21.23	2 20	678 701	20
mmu-miR-378a-5p	NM_021475.2	141.00	-13.39	2 19	588 610	18
mmu-miR-378a-5p	NM_013906.3	158.00	-22.16	2 15	2485 2506	13
mmu-miR-378a-5p	NM_013906.3	142.00	-18.21	2 20	3208 3230	19
mmu-miR-378a-5p	XM_006538374.1	144.00	-14.41	2 21	7603 7624	19
mmu-miR-378a-5p	XM_006505388.2	143.00	-24.78	2 16	5491 5512	14
mmu-miR-378a-5p	NM_001291930.1	140.00	-24.35	3 21	2074 2095	18
mmu-miR-378a-5p	XM_017312667.1	154.00	-18.02	2 21	4203 4223	19
mmu-miR-378a-5p	XM_017312667.1	146.00	-21.58	2 21	5078 5098	19
mmu-miR-378a-5p	XM_017312667.1	144.00	-16.01	2 21	8055 8073	19
mmu-miR-378a-5p	NM_008032.3	153.00	-16.88	2 21	3131 3152	20
mmu-miR-378a-5p	NM_153178.4	146.00	-27.01	2 21	7646 7671	23
mmu-miR-378a-5p	NM_153178.4	143.00	-23.14	2 20	5926 5947	18
mmu-miR-378a-5p	XM_011250176.1	148.00	-24.78	2 17	5971 5992	15
mmu-miR-378a-5p	NM_031185.3	153.00	-19.43	2 19	4014 4036	18
mmu-miR-378a-5p	NM_008012.1	140.00	-23.01	2 21	404 425	19
mmu-miR-378a-5p	NM_053080.3	146.00	-14.19	2 20	3020 3043	20
mmu-miR-378a-5p	XM_006495613.3	155.00	-24.20	2 21	327 351	22
mmu-miR-378a-5p	XM_006508990.2	149.00	-24.14	2 21	5321 5343	21
mmu-miR-378a-5p	XM_006508990.2	147.00	-18.98	2 21	650 672	20
mmu-miR-378a-5p	NM_013468.3	140.00	-20.05	2 9	1589 1610	7
mmu-miR-378a-5p	NM_013912.3	156.00	-26.94	2 21	2067 2088	19
mmu-miR-378a-5p	NM_001301354.1	141.00	-29.22	2 21	1437 1458	20
mmu-miR-378a-5p	NM_207231.1	156.00	-26.80	2 18	334 358	19
mmu-miR-378a-5p	NM_025758.4	145.00	-14.94	2 10	300 321	8
mmu-miR-378a-5p	NM_025758.4	141.00	-17.85	2 20	740 760	18
mmu-miR-378a-5p	NM_023048.5	147.00	-32.38	2 21	834 856	20
mmu-miR-378a-5p	NM_001290469.1	140.00	-25.32	2 21	1354 1372	19
mmu-miR-378a-5p	NM_013415.5	143.00	-28.15	2 21	1666 1685	20
mmu-miR-378a-5p	XM_006529727.2	146.00	-15.62	2 15	6745 6766	13
mmu-miR-378a-5p	NM_013863.5	158.00	-25.06	2 20	1725 1748	20
mmu-miR-378a-5p	NM_013863.5	140.00	-27.36	4 21	2054 2075	17
mmu-miR-378a-5p	XM_006502291.3	149.00	-24.36	2 21	2792 2811	19
mmu-miR-378a-5p	XM_006502291.3	142.00	-25.61	3 20	3575 3598	19
mmu-miR-378a-5p	XM_006510701.3	145.00	-15.98	2 10	2223 2244	8
mmu-miR-378a-5p	NM_178782.4	151.00	-29.00	2 21	3761 3783	20
mmu-miR-378a-5p	NM_178782.4	141.00	-13.70	2 20	4479 4499	18
mmu-miR-378a-5p	NM_028472.2	160.00	-20.13	2 19	3001 3021	17
mmu-miR-378a-5p	NM_178692.3	151.00	-19.59	2 17	1274 1297	17
mmu-miR-378a-5p	NM_178692.3	148.00	-24.79	2 20	697 716	18
mmu-miR-378a-5p	NM_023143.3	154.00	-27.25	2 21	118 145	25
mmu-miR-378a-5p	NM_001285867.1	158.00	-21.19	2 16	450 473	16
mmu-miR-378a-5p	NM_007588.2	148.00	-21.32	2 20	266 285	18
mmu-miR-378a-5p	NM_007588.2	146.00	-16.35	2 16	67 90	16
mmu-miR-378a-5p	NM_001190379.1	140.00	-25.47	2 21	2193 2213	20
mmu-miR-378a-5p	XM_011246479.2	148.00	-15.65	2 17	3377 3398	15
mmu-miR-378a-5p	XM_006501443.3	155.00	-23.12	2 21	838 860	20
mmu-miR-378a-5p	NM_009824.2	162.00	-25.37	2 15	4680 4701	13
mmu-miR-378a-5p	NM_009824.2	153.00	-23.92	2 20	1402 1422	18
mmu-miR-378a-5p	NM_001310648.1	147.00	-21.06	2 21	235 258	21
mmu-miR-378a-5p	NM_028804.1	164.00	-27.21	2 21	2557 2578	19
mmu-miR-378a-5p	XM_006533653.3	154.00	-27.50	2 21	197 220	21
mmu-miR-378a-5p	NM_001110322.1	156.00	-15.39	2 17	869 890	15
mmu-miR-378a-5p	NM_001110322.1	140.00	-20.11	2 21	837 858	19

mmu-miR-378a-5p	NM_001313939.1	140.00	-24.87	2 17	897 918	15
mmu-miR-378a-5p	XM_011245344.2	140.00	-21.57	2 9	3809 3830	7
mmu-miR-378a-5p	XM_006531321.3	154.00	-19.07	2 15	112 133	13
mmu-miR-378a-5p	XM_006523030.3	145.00	-15.44	2 15	3526 3548	14
mmu-miR-378a-5p	XM_006513165.1	144.00	-25.10	2 21	516 537	19
mmu-miR-378a-5p	XM_006527480.3	144.00	-22.15	3 21	118 139	18
mmu-miR-378a-5p	NM_001113515.2	149.00	-22.88	2 15	2444 2467	15
mmu-miR-378a-5p	NM_001113515.2	140.00	-24.85	2 18	283 306	18
mmu-miR-378a-5p	NM_009930.2	164.00	-23.03	2 21	889 910	19
mmu-miR-378a-5p	NM_007739.2	142.00	-29.38	2 20	1158 1186	25
mmu-miR-378a-5p	XM_011240755.1	154.00	-16.90	2 21	4712 4732	19
mmu-miR-378a-5p	XM_006532938.2	145.00	-15.56	2 18	1624 1645	16
mmu-miR-378a-5p	XM_011247208.1	151.00	-22.41	2 21	3597 3619	20
mmu-miR-378a-5p	XM_011247208.1	146.00	-17.76	2 11	7366 7387	9
mmu-miR-378a-5p	XM_011248602.2	141.00	-23.21	2 21	250 271	20
mmu-miR-378a-5p	NM_011957.2	140.00	-25.11	2 17	8 29	15
mmu-miR-378a-5p	XM_011242311.2	151.00	-27.46	3 16	2269 2290	13
mmu-miR-378a-5p	XM_011242311.2	151.00	-19.81	2 20	4761 4782	18
mmu-miR-378a-5p	XM_006517308.3	140.00	-19.89	2 21	1577 1598	19
mmu-miR-378a-5p	NM_001190448.1	143.00	-24.88	2 21	1143 1165	20
mmu-miR-378a-5p	NM_015814.2	141.00	-24.29	3 21	2116 2137	19
mmu-miR-378a-5p	NM_027293.1	147.00	-20.97	2 21	6772 6795	21
mmu-miR-378a-5p	XM_011243391.2	159.00	-31.86	2 16	4470 4491	14
mmu-miR-378a-5p	XM_011243391.2	144.00	-26.94	2 21	8363 8385	20
mmu-miR-378a-5p	XM_011243391.2	143.00	-31.15	2 20	2105 2126	18
mmu-miR-378a-5p	XM_006506808.3	154.00	-16.89	2 21	1881 1901	19
mmu-miR-378a-5p	XM_006506808.3	140.00	-19.47	3 21	1729 1750	18
mmu-miR-378a-5p	NM_007883.3	143.00	-16.33	2 21	4139 4165	24
mmu-miR-378a-5p	XM_011245824.2	140.00	-20.60	2 15	1514 1534	13
mmu-miR-378a-5p	NM_133643.4	147.00	-18.75	2 21	3808 3826	19
mmu-miR-378a-5p	NM_015744.4	141.00	-11.85	2 21	1565 1584	19
mmu-miR-378a-5p	NM_178676.4	143.00	-29.04	2 21	1902 1924	20
mmu-miR-378a-5p	XM_011238916.2	147.00	-14.33	2 16	4726 4747	14
mmu-miR-494-3p	XM_017320219.1	140.00	-8.73	2 9	317 338	7
mmu-miR-494-3p	NM_001271580.1	141.00	-12.65	2 14	1346 1367	12
mmu-miR-494-3p	XM_011241948.2	142.00	-18.18	2 15	1719 1740	13
mmu-miR-494-3p	XM_011241322.1	146.00	-12.70	2 13	429 449	11
mmu-miR-494-3p	XM_011241322.1	141.00	-8.81	2 12	2911 2931	10
mmu-miR-494-3p	XM_006498086.3	140.00	-10.44	2 9	8546 8567	7
mmu-miR-494-3p	NM_021475.2	141.00	-9.41	2 19	1865 1888	19
mmu-miR-494-3p	NM_013906.3	141.00	-11.77	2 20	4030 4050	18
mmu-miR-494-3p	XM_006505388.2	151.00	-15.85	2 21	228 251	21
mmu-miR-494-3p	XM_006500919.3	156.00	-17.60	2 19	2429 2452	19
mmu-miR-494-3p	XM_017312667.1	155.00	-18.19	2 20	4405 4426	18
mmu-miR-494-3p	XM_017312667.1	140.00	-11.94	2 9	1317 1338	7
mmu-miR-494-3p	NM_008032.3	144.00	-16.64	2 13	4444 4465	11
mmu-miR-494-3p	NM_031185.3	146.00	-12.09	2 20	5565 5587	19
mmu-miR-494-3p	NM_001303431.1	142.00	-14.67	2 19	748 769	17
mmu-miR-494-3p	NM_172790.2	156.00	-11.59	2 21	6155 6176	19
mmu-miR-494-3p	NM_023048.5	145.00	-12.89	2 20	3143 3171	25
mmu-miR-494-3p	NM_025711.3	146.00	-10.55	2 17	1838 1858	15
mmu-miR-494-3p	XM_006529727.2	149.00	-16.55	2 20	7049 7073	21
mmu-miR-494-3p	NM_001159407.1	151.00	-13.09	2 16	2338 2359	14
mmu-miR-494-3p	NM_007529.2	151.00	-18.41	3 21	2199 2222	20
mmu-miR-494-3p	XM_006502291.3	142.00	-15.32	2 21	2568 2591	21
mmu-miR-494-3p	XM_006503323.3	144.00	-11.41	2 13	858 879	11
mmu-miR-494-3p	NM_007555.4	149.00	-14.05	2 20	1940 1960	18
mmu-miR-494-3p	NM_028472.2	144.00	-15.22	3 20	1279 1300	18
mmu-miR-494-3p	NM_020508.4	145.00	-14.91	2 18	5182 5203	16
mmu-miR-494-3p	XM_006503665.3	145.00	-8.18	2 10	2377 2398	8
mmu-miR-494-3p	NM_001285867.1	152.00	-15.22	2 21	4626 4647	19
mmu-miR-494-3p	NM_001190379.1	140.00	-13.09	2 9	760 781	7
mmu-miR-494-3p	XM_006520253.3	144.00	-12.20	2 18	4228 4245	16
mmu-miR-494-3p	NM_019626.3	144.00	-11.46	2 14	1247 1269	13
mmu-miR-494-3p	NM_019626.3	140.00	-10.63	2 21	1351 1372	19

mmu-miR-494-3p	NM_028804.1	145.00	-7.75	2 19	1684 1706	18
mmu-miR-494-3p	NM_001013784.1	146.00	-19.79	2 16	1163 1186	16
mmu-miR-494-3p	NM_001313939.1	140.00	-8.71	2 9	18 39	7
mmu-miR-494-3p	XM_011245344.2	148.00	-15.65	2 15	12507 12527	13
mmu-miR-494-3p	XM_011245344.2	142.00	-11.39	2 11	9058 9079	9
mmu-miR-494-3p	XM_011245344.2	141.00	-9.95	2 18	4311 4332	16
mmu-miR-494-3p	XM_011245344.2	140.00	-20.19	2 18	8795 8818	18
mmu-miR-494-3p	XM_006523030.3	144.00	-8.82	2 19	2561 2581	17
mmu-miR-494-3p	XM_006527480.3	143.00	-14.95	2 19	4486 4507	18
mmu-miR-494-3p	XM_017314944.1	140.00	-13.55	2 19	2394 2419	21
mmu-miR-494-3p	XM_011247208.1	142.00	-13.35	2 19	4443 4464	17
mmu-miR-494-3p	XM_011247208.1	140.00	-13.40	3 18	3153 3176	17
mmu-miR-494-3p	XM_011248602.2	144.00	-11.98	2 13	1492 1513	11
mmu-miR-494-3p	NM_018866.2	144.00	-7.49	2 20	1097 1116	18
mmu-miR-494-3p	NM_015814.2	140.00	-12.20	2 9	249 270	7
mmu-miR-494-3p	NM_027293.1	150.00	-12.28	2 20	6380 6402	19
mmu-miR-494-3p	NM_027293.1	140.00	-11.21	2 9	1766 1787	7
mmu-miR-494-3p	NM_019759.2	140.00	-7.26	2 9	788 809	7
mmu-miR-494-3p	NM_007883.3	140.00	-8.89	2 9	5145 5166	7
mmu-miR-494-3p	XM_006528026.2	165.00	-19.85	2 18	2787 2808	16
mmu-miR-494-3p	XM_006528026.2	154.00	-15.55	2 17	3213 3237	18
mmu-miR-494-3p	NM_133643.4	145.00	-9.57	2 10	4940 4961	8
mmu-miR-494-3p	NM_010133.2	154.00	-12.78	2 19	2241 2262	17
mmu-miR-494-3p	NM_178676.4	156.00	-12.22	2 18	3107 3130	18
mmu-miR-494-3p	NM_010137.3	142.00	-14.39	2 20	3248 3270	19
mmu-miR-494-3p	NM_010140.3	151.00	-12.27	2 16	727 748	14
mmu-miR-494-3p	XM_011238916.2	144.00	-18.10	2 13	4747 4768	11
mmu-miR-494-3p	XM_011238916.2	143.00	-10.42	2 20	2673 2694	18
mmu-miR-494-3p	XM_006511221.3	146.00	-17.65	3 21	680 700	18
mmu-miR-135a-1-3p	XM_017320219.1	140.00	-20.26	3 21	2832 2853	18
mmu-miR-135a-1-3p	NM_001271580.1	142.00	-20.75	2 19	898 919	17
mmu-miR-135a-1-3p	XM_006515980.3	149.00	-22.76	2 21	780 799	19
mmu-miR-135a-1-3p	XM_006515980.3	146.00	-19.82	2 15	355 376	13
mmu-miR-135a-1-3p	NM_029982.1	149.00	-24.47	2 21	470 495	23
mmu-miR-135a-1-3p	NM_138955.3	147.00	-20.91	2 21	1856 1878	20
mmu-miR-135a-1-3p	NM_138955.3	145.00	-23.85	2 20	1089 1117	25
mmu-miR-135a-1-3p	NM_138955.3	142.00	-21.92	2 15	3506 3527	13
mmu-miR-135a-1-3p	NM_153151.3	148.00	-23.89	2 21	867 888	19
mmu-miR-135a-1-3p	NM_172580.1	144.00	-19.36	2 21	1062 1083	19
mmu-miR-135a-1-3p	XM_006498086.3	152.00	-21.91	2 21	4100 4121	19
mmu-miR-135a-1-3p	XM_006498086.3	148.00	-18.57	2 16	7481 7500	14
mmu-miR-135a-1-3p	XM_006498086.3	140.00	-17.70	3 21	2002 2023	18
mmu-miR-135a-1-3p	XM_006505388.2	149.00	-19.50	2 14	5274 5295	12
mmu-miR-135a-1-3p	XM_017312667.1	150.00	-21.70	2 20	7434 7456	19
mmu-miR-135a-1-3p	XM_017312667.1	142.00	-23.70	2 21	8254 8274	19
mmu-miR-135a-1-3p	XM_017312667.1	140.00	-27.13	4 21	5334 5355	17
mmu-miR-135a-1-3p	XM_011250176.1	152.00	-21.55	2 21	7275 7296	19
mmu-miR-135a-1-3p	NM_031185.3	142.00	-21.08	2 21	83 103	19
mmu-miR-135a-1-3p	XM_011240149.1	148.00	-22.99	2 21	2317 2338	19
mmu-miR-135a-1-3p	NM_008012.1	150.00	-22.46	2 20	982 1004	19
mmu-miR-135a-1-3p	XM_006516510.1	152.00	-21.63	2 15	114 134	13
mmu-miR-135a-1-3p	NM_001303431.1	141.00	-19.88	3 18	142 163	15
mmu-miR-135a-1-3p	NM_172790.2	152.00	-21.12	2 19	5284 5304	17
mmu-miR-135a-1-3p	NM_172790.2	143.00	-24.31	3 21	3256 3280	21
mmu-miR-135a-1-3p	NM_172790.2	141.00	-19.24	2 20	5701 5721	18
mmu-miR-135a-1-3p	NM_009675.2	142.00	-27.84	2 19	3550 3571	17
mmu-miR-135a-1-3p	NM_013912.3	158.00	-23.49	2 21	858 878	19
mmu-miR-135a-1-3p	NM_001080819.1	171.00	-30.71	2 21	3824 3847	21
mmu-miR-135a-1-3p	NM_001080819.1	143.00	-17.90	2 19	2775 2794	17
mmu-miR-135a-1-3p	NM_029823.2	151.00	-24.98	2 21	640 662	20
mmu-miR-135a-1-3p	NM_023048.5	155.00	-27.23	2 12	1227 1248	10
mmu-miR-135a-1-3p	NM_007881.4	150.00	-25.06	2 21	1659 1682	21
mmu-miR-135a-1-3p	NM_007881.4	141.00	-25.64	2 20	4234 4254	18
mmu-miR-135a-1-3p	NM_001290469.1	154.00	-31.48	2 20	2081 2103	19
mmu-miR-135a-1-3p	NM_009722.3	144.00	-25.01	2 21	2886 2907	19

mmu-miR-135a-1-3p	XM_006529727.2	140.00	-23.13	2 21	4685 4706	19
mmu-miR-135a-1-3p	XM_006529727.2	140.00	-14.37	2 9	5339 5360	7
mmu-miR-135a-1-3p	XM_006507725.2	148.00	-25.13	2 21	1862 1883	19
mmu-miR-135a-1-3p	NM_020025.4	144.00	-17.56	2 20	1486 1510	21
mmu-miR-135a-1-3p	NM_001159407.1	146.00	-15.21	2 15	1848 1869	13
mmu-miR-135a-1-3p	NM_013863.5	143.00	-24.45	2 21	1389 1411	20
mmu-miR-135a-1-3p	NM_007529.2	140.00	-25.17	3 21	279 300	18
mmu-miR-135a-1-3p	NM_178782.4	150.00	-26.75	2 21	3887 3907	19
mmu-miR-135a-1-3p	XM_006503323.3	143.00	-22.40	3 21	629 651	19
mmu-miR-135a-1-3p	NM_020508.4	142.00	-20.64	2 21	5730 5750	19
mmu-miR-135a-1-3p	XM_006524328.3	152.00	-28.74	2 21	1352 1370	19
mmu-miR-135a-1-3p	XM_006524328.3	148.00	-20.67	3 21	5046 5067	18
mmu-miR-135a-1-3p	XM_006524328.3	146.00	-23.06	2 21	3840 3863	21
mmu-miR-135a-1-3p	XM_006503665.3	149.00	-17.86	2 21	2603 2622	19
mmu-miR-135a-1-3p	XM_006503665.3	142.00	-17.38	2 19	1818 1839	17
mmu-miR-135a-1-3p	NM_001285867.1	149.00	-18.86	2 14	4837 4858	12
mmu-miR-135a-1-3p	NM_007588.2	141.00	-21.37	2 10	3589 3610	8
mmu-miR-135a-1-3p	NM_181315.4	147.00	-32.93	2 21	212 238	24
mmu-miR-135a-1-3p	NM_009824.2	147.00	-16.91	2 20	6385 6406	18
mmu-miR-135a-1-3p	NM_009824.2	142.00	-29.94	2 20	3638 3662	21
mmu-miR-135a-1-3p	NM_019626.3	144.00	-24.80	3 21	1221 1241	19
mmu-miR-135a-1-3p	NM_001013784.1	152.00	-20.82	2 13	3675 3696	11
mmu-miR-135a-1-3p	NM_007655.3	142.00	-29.80	3 21	360 386	23
mmu-miR-135a-1-3p	XM_011245344.2	143.00	-17.69	2 21	10467 10489	20
mmu-miR-135a-1-3p	XM_011245344.2	142.00	-16.73	2 15	3769 3790	13
mmu-miR-135a-1-3p	XM_006531321.3	140.00	-17.21	2 21	1683 1704	19
mmu-miR-135a-1-3p	XM_006530131.1	147.00	-15.94	2 17	8420 8442	16
mmu-miR-135a-1-3p	NM_009930.2	151.00	-22.47	2 20	4983 5003	19
mmu-miR-135a-1-3p	XM_011240755.1	143.00	-20.06	2 20	4636 4657	18
mmu-miR-135a-1-3p	XM_006532938.2	149.00	-15.26	2 21	461 484	22
mmu-miR-135a-1-3p	XM_011247208.1	144.00	-23.26	2 21	7557 7578	19
mmu-miR-135a-1-3p	NM_001289782.1	146.00	-21.86	2 21	841 861	19
mmu-miR-135a-1-3p	NM_001190451.2	140.00	-23.41	2 19	1383 1403	17
mmu-miR-135a-1-3p	NM_001190448.1	160.00	-26.59	2 20	346 367	19
mmu-miR-135a-1-3p	NM_015814.2	142.00	-22.18	2 21	1605 1625	19
mmu-miR-135a-1-3p	NM_027293.1	146.00	-27.73	2 21	862 882	19
mmu-miR-135a-1-3p	NM_027293.1	140.00	-28.44	2 21	2553 2575	20
mmu-miR-135a-1-3p	NM_007883.3	144.00	-12.52	2 21	4539 4560	19
mmu-miR-135a-1-3p	NM_015744.4	143.00	-15.62	2 15	2219 2238	13
mmu-miR-135a-1-3p	NM_178676.4	146.00	-14.37	2 11	2480 2501	9
mmu-miR-135a-1-3p	NM_010137.3	158.00	-25.22	2 21	1997 2021	22
mmu-miR-135a-1-3p	NM_010137.3	140.00	-19.34	2 21	1488 1507	19
mmu-miR-135a-1-3p	XM_011238916.2	150.00	-21.38	2 20	2941 2965	21
mmu-miR-1892	XM_017320219.1	147.00	-26.05	2 18	2946 2966	16
mmu-miR-1892	XM_017320219.1	141.00	-15.30	2 11	544 566	10
mmu-miR-1892	XM_017320219.1	140.00	-18.99	2 18	3143 3166	18
mmu-miR-1892	NM_025865.2	151.00	-15.33	2 21	960 982	20
mmu-miR-1892	XM_006534171.3	162.00	-23.40	2 19	1060 1081	17
mmu-miR-1892	NM_001271580.1	151.00	-21.59	2 20	642 662	19
mmu-miR-1892	NM_001271580.1	147.00	-30.58	3 21	784 806	19
mmu-miR-1892	XM_011241322.1	151.00	-17.16	2 19	3997 4016	17
mmu-miR-1892	XM_011241322.1	144.00	-28.57	2 21	711 732	19
mmu-miR-1892	NM_138955.3	151.00	-29.24	2 21	3289 3311	20
mmu-miR-1892	NM_138955.3	148.00	-29.63	3 21	2412 2433	18
mmu-miR-1892	NM_138955.3	141.00	-27.66	2 20	2973 2993	18
mmu-miR-1892	XM_006540566.1	168.00	-28.87	2 21	7111 7132	19
mmu-miR-1892	XM_006540566.1	146.00	-16.79	2 15	6033 6054	13
mmu-miR-1892	XM_006540566.1	144.00	-25.64	2 21	609 630	19
mmu-miR-1892	XM_006540566.1	140.00	-19.42	2 21	951 972	19
mmu-miR-1892	NM_172580.1	143.00	-18.04	2 20	997 1018	18
mmu-miR-1892	NM_001142804.1	143.00	-22.56	2 21	267 289	20
mmu-miR-1892	NM_021475.2	143.00	-16.93	2 20	1248 1269	18
mmu-miR-1892	NM_021475.2	140.00	-11.65	2 9	1579 1600	7
mmu-miR-1892	NM_013906.3	145.00	-26.30	3 21	2735 2754	18
mmu-miR-1892	NM_013906.3	140.00	-25.38	2 21	2142 2163	19

mmu-miR-1892	XM_006538374.1	152.00	-23.77	2 21	3781 3802	19
mmu-miR-1892	XM_006538374.1	147.00	-24.79	2 21	6918 6940	20
mmu-miR-1892	XM_006538374.1	144.00	-15.51	2 18	3599 3622	18
mmu-miR-1892	XM_006538374.1	144.00	-26.87	2 20	6890 6909	18
mmu-miR-1892	XM_006538374.1	140.00	-12.41	2 9	2727 2748	7
mmu-miR-1892	XM_006505388.2	145.00	-26.65	3 21	3012 3031	18
mmu-miR-1892	XM_006505388.2	143.00	-19.05	2 16	2202 2223	14
mmu-miR-1892	NM_001291930.1	161.00	-28.23	2 18	3499 3520	16
mmu-miR-1892	NM_001291930.1	141.00	-25.97	2 21	537 560	22
mmu-miR-1892	XM_017312667.1	145.00	-24.26	2 18	5887 5908	16
mmu-miR-1892	XM_017312667.1	140.00	-27.65	3 17	4667 4688	14
mmu-miR-1892	XM_017312667.1	140.00	-13.14	2 9	7000 7021	7
mmu-miR-1892	NM_008032.3	148.00	-24.73	2 21	1057 1078	19
mmu-miR-1892	NM_008032.3	145.00	-15.89	2 15	753 775	14
mmu-miR-1892	NM_153178.4	160.00	-32.89	2 19	5760 5780	17
mmu-miR-1892	NM_153178.4	147.00	-12.52	2 12	301 322	10
mmu-miR-1892	NM_153178.4	140.00	-19.02	2 21	911 932	19
mmu-miR-1892	NM_153178.4	140.00	-15.85	2 9	7392 7413	7
mmu-miR-1892	XM_011250176.1	148.00	-22.07	2 18	4035 4058	18
mmu-miR-1892	NM_009645.2	143.00	-15.88	2 19	1910 1929	17
mmu-miR-1892	XM_006498287.3	140.00	-24.97	2 21	1691 1712	19
mmu-miR-1892	XM_011240149.1	160.00	-29.35	2 18	984 1006	17
mmu-miR-1892	XM_011240149.1	140.00	-11.64	2 9	945 966	7
mmu-miR-1892	NM_008012.1	145.00	-11.71	2 10	422 443	8
mmu-miR-1892	NM_008012.1	143.00	-14.89	2 15	126 145	13
mmu-miR-1892	NM_008012.1	143.00	-21.47	2 21	742 767	23
mmu-miR-1892	NM_001303431.1	147.00	-22.34	3 20	1092 1113	17
mmu-miR-1892	NM_001303431.1	141.00	-22.25	2 19	1270 1292	18
mmu-miR-1892	XM_006508990.2	148.00	-19.90	2 21	541 562	19
mmu-miR-1892	XM_006508990.2	142.00	-18.07	2 17	5699 5719	15
mmu-miR-1892	XM_006508990.2	140.00	-12.64	2 9	6387 6408	7
mmu-miR-1892	NM_172790.2	151.00	-31.82	3 21	4291 4315	21
mmu-miR-1892	NM_172790.2	142.00	-22.91	2 21	3557 3580	21
mmu-miR-1892	NM_009675.2	150.00	-21.01	2 20	3080 3102	19
mmu-miR-1892	NM_009675.2	146.00	-27.81	3 21	63 83	18
mmu-miR-1892	NM_009675.2	146.00	-24.90	2 20	2565 2588	20
mmu-miR-1892	NM_009675.2	146.00	-29.02	2 19	2781 2802	17
mmu-miR-1892	NM_013912.3	147.00	-21.47	2 21	2113 2135	20
mmu-miR-1892	NM_001301354.1	151.00	-28.12	2 21	1679 1701	20
mmu-miR-1892	NM_001301354.1	140.00	-23.92	2 17	265 286	15
mmu-miR-1892	NM_001080819.1	149.00	-18.46	2 17	7536 7555	15
mmu-miR-1892	NM_001080819.1	146.00	-26.97	2 21	6321 6341	19
mmu-miR-1892	NM_001080819.1	142.00	-24.84	2 21	3928 3948	19
mmu-miR-1892	NM_001080819.1	141.00	-27.26	3 21	1574 1599	22
mmu-miR-1892	NM_001080819.1	140.00	-31.35	3 21	423 444	18
mmu-miR-1892	NM_023048.5	153.00	-21.16	2 17	1791 1810	15
mmu-miR-1892	NM_023048.5	140.00	-11.43	2 9	1 19	7
mmu-miR-1892	NM_001310070.1	149.00	-25.28	3 18	945 966	15
mmu-miR-1892	NM_007881.4	153.00	-23.36	2 18	519 538	16
mmu-miR-1892	NM_007881.4	143.00	-25.96	2 21	2058 2082	22
mmu-miR-1892	NM_007881.4	140.00	-28.33	3 21	3676 3697	18
mmu-miR-1892	NM_001290469.1	145.00	-26.03	2 18	3241 3262	16
mmu-miR-1892	NM_013415.5	175.00	-32.99	2 21	1529 1551	20
mmu-miR-1892	NM_013415.5	142.00	-23.12	2 21	2139 2164	23
mmu-miR-1892	NM_009722.3	152.00	-23.26	2 19	2019 2043	20
mmu-miR-1892	XM_006529727.2	154.00	-25.58	2 21	4958 4978	19
mmu-miR-1892	XM_006529727.2	142.00	-14.39	2 19	6943 6964	17
mmu-miR-1892	XM_006529727.2	141.00	-18.82	2 18	7664 7685	16
mmu-miR-1892	XM_006529727.2	140.00	-19.39	2 18	6587 6609	17
mmu-miR-1892	XM_006529727.2	140.00	-28.93	2 21	7468 7489	19
mmu-miR-1892	XM_006507725.2	151.00	-27.70	2 21	1658 1680	20
mmu-miR-1892	XM_006507725.2	150.00	-31.57	3 21	3667 3687	18
mmu-miR-1892	XM_006507725.2	146.00	-29.62	2 21	3232 3256	22
mmu-miR-1892	XM_006507725.2	144.00	-19.87	2 21	1916 1937	19
mmu-miR-1892	NM_020025.4	140.00	-21.19	3 21	170 191	18

mmu-miR-1892	NM_013863.5	156.00	-19.53	2	17	1498	1519	15
mmu-miR-1892	NM_013863.5	150.00	-26.68	2	19	1435	1456	17
mmu-miR-1892	XM_006502291.3	150.00	-21.97	2	21	4194	4214	19
mmu-miR-1892	XM_006502291.3	149.00	-30.03	3	20	301	321	17
mmu-miR-1892	XM_006502291.3	145.00	-29.15	2	20	1575	1599	21
mmu-miR-1892	XM_006502291.3	142.00	-26.41	2	21	1171	1191	19
mmu-miR-1892	XM_006502291.3	140.00	-20.20	3	21	2335	2356	18
mmu-miR-1892	XM_006510701.3	148.00	-26.19	2	21	5251	5272	19
mmu-miR-1892	XM_006510701.3	141.00	-20.74	2	20	4453	4473	18
mmu-miR-1892	NM_178782.4	151.00	-20.43	2	20	4776	4797	18
mmu-miR-1892	NM_178782.4	142.00	-24.67	2	21	2454	2474	19
mmu-miR-1892	XM_006503323.3	154.00	-18.69	2	19	364	385	17
mmu-miR-1892	XM_006503323.3	145.00	-12.03	2	10	1643	1664	8
mmu-miR-1892	NM_007555.4	147.00	-26.80	2	20	2782	2803	18
mmu-miR-1892	NM_028472.2	146.00	-15.77	2	20	3176	3198	19
mmu-miR-1892	NM_028472.2	144.00	-18.19	2	15	1215	1235	13
mmu-miR-1892	NM_028472.2	143.00	-31.39	3	20	1176	1197	17
mmu-miR-1892	NM_028472.2	140.00	-27.45	2	17	252	273	15
mmu-miR-1892	NM_020508.4	155.00	-30.63	2	21	1182	1204	20
mmu-miR-1892	NM_020508.4	154.00	-20.52	2	20	2898	2920	19
mmu-miR-1892	NM_020508.4	151.00	-27.14	2	21	938	961	21
mmu-miR-1892	XM_006524328.3	153.00	-30.52	2	18	2827	2848	16
mmu-miR-1892	XM_006524328.3	144.00	-24.40	3	21	5646	5667	18
mmu-miR-1892	XM_006524328.3	143.00	-25.22	2	21	5052	5074	20
mmu-miR-1892	XM_006524328.3	142.00	-23.58	3	20	1426	1448	18
mmu-miR-1892	NM_178692.3	151.00	-32.53	2	21	2417	2440	21
mmu-miR-1892	NM_026979.5	145.00	-26.97	2	21	274	293	19
mmu-miR-1892	NM_023143.3	146.00	-16.48	2	15	1712	1733	13
mmu-miR-1892	NM_023143.3	143.00	-31.64	2	21	262	284	20
mmu-miR-1892	NM_023143.3	140.00	-14.88	2	9	1296	1317	7
mmu-miR-1892	NM_001097617.1	155.00	-24.39	2	21	2160	2182	20
mmu-miR-1892	NM_001097617.1	154.00	-30.95	2	21	703	726	21
mmu-miR-1892	NM_001097617.1	143.00	-24.30	3	21	2144	2167	20
mmu-miR-1892	NM_001285867.1	151.00	-29.49	2	21	1539	1561	20
mmu-miR-1892	NM_001285867.1	148.00	-29.47	2	18	1949	1971	17
mmu-miR-1892	NM_001285867.1	144.00	-29.46	2	21	3342	3363	19
mmu-miR-1892	NM_001285867.1	143.00	-16.73	2	16	1652	1673	14
mmu-miR-1892	NM_001285867.1	142.00	-20.96	2	21	1992	2012	19
mmu-miR-1892	NM_009785.1	152.00	-21.35	2	13	1622	1643	11
mmu-miR-1892	XM_011246128.2	150.00	-26.09	2	21	927	947	19
mmu-miR-1892	XM_011246128.2	140.00	-13.14	2	9	4778	4799	7
mmu-miR-1892	NM_007588.2	140.00	-25.87	2	21	3012	3033	19
mmu-miR-1892	NM_001190379.1	156.00	-22.08	2	18	460	482	17
mmu-miR-1892	NM_001190379.1	156.00	-19.60	2	21	1351	1372	19
mmu-miR-1892	NM_001190379.1	150.00	-30.24	2	20	1044	1068	21
mmu-miR-1892	NM_001190379.1	144.00	-24.49	3	21	1616	1637	18
mmu-miR-1892	NM_001190379.1	142.00	-23.28	2	17	331	351	15
mmu-miR-1892	XM_011246479.2	162.00	-25.59	2	21	1298	1318	19
mmu-miR-1892	XM_011246479.2	152.00	-24.81	2	21	2262	2283	19
mmu-miR-1892	XM_011246479.2	146.00	-28.62	2	20	721	743	19
mmu-miR-1892	XM_011246479.2	143.00	-17.63	2	21	651	673	20
mmu-miR-1892	XM_006520186.2	143.00	-17.84	2	20	620	641	18
mmu-miR-1892	XM_006501443.3	163.00	-28.88	2	21	688	712	22
mmu-miR-1892	XM_006501443.3	141.00	-23.30	3	20	1026	1046	17
mmu-miR-1892	XM_006520253.3	158.00	-34.29	2	21	2866	2891	23
mmu-miR-1892	NM_009824.2	163.00	-25.88	2	20	7204	7225	18
mmu-miR-1892	NM_009824.2	144.00	-28.96	2	21	3268	3289	19
mmu-miR-1892	NM_009824.2	143.00	-16.41	2	20	2167	2188	18
mmu-miR-1892	NM_009824.2	141.00	-33.61	2	20	165	185	18
mmu-miR-1892	NM_009824.2	141.00	-23.95	2	18	5607	5628	16
mmu-miR-1892	NM_009824.2	140.00	-29.62	4	21	3494	3515	17
mmu-miR-1892	NM_019626.3	147.00	-25.92	2	20	1130	1151	18
mmu-miR-1892	NM_019626.3	144.00	-22.24	2	19	1152	1172	17
mmu-miR-1892	NM_001310648.1	143.00	-25.85	2	20	1575	1596	18
mmu-miR-1892	NM_028804.1	140.00	-18.87	2	21	1689	1710	19

mmu-miR-1892	NM_001013784.1	155.00	-28.72	2 21	2987 3009	20
mmu-miR-1892	NM_001013784.1	140.00	-26.12	2 18	3007 3031	19
mmu-miR-1892	NM_001110322.1	143.00	-23.71	2 21	1196 1220	22
mmu-miR-1892	NM_007655.3	156.00	-27.10	2 21	1157 1178	19
mmu-miR-1892	NM_007655.3	146.00	-22.52	2 19	828 849	17
mmu-miR-1892	NM_007655.3	146.00	-26.53	2 21	1244 1267	21
mmu-miR-1892	NM_007655.3	143.00	-21.48	3 20	1143 1164	17
mmu-miR-1892	NM_007655.3	140.00	-21.03	2 21	261 282	19
mmu-miR-1892	NM_001313939.1	154.00	-17.91	2 15	27 48	13
mmu-miR-1892	XM_011245344.2	148.00	-21.32	2 21	1625 1646	19
mmu-miR-1892	XM_011245344.2	146.00	-24.79	2 21	1796 1816	19
mmu-miR-1892	XM_011245344.2	140.00	-24.05	3 21	151 172	18
mmu-miR-1892	XM_017312731.1	151.00	-21.17	2 17	1376 1399	17
mmu-miR-1892	XM_017312731.1	147.00	-14.93	2 20	1002 1023	18
mmu-miR-1892	XM_006503371.2	141.00	-21.78	2 14	3332 3353	12
mmu-miR-1892	NM_013459.3	142.00	-27.25	2 20	322 344	19
mmu-miR-1892	XM_006532007.3	152.00	-15.75	2 13	69 90	11
mmu-miR-1892	XM_006532007.3	147.00	-29.21	2 21	1581 1603	20
mmu-miR-1892	XM_006530131.1	155.00	-21.54	2 20	1066 1087	18
mmu-miR-1892	XM_006530131.1	140.00	-13.81	2 9	4525 4546	7
mmu-miR-1892	XM_011250217.1	146.00	-24.76	2 20	1244 1267	20
mmu-miR-1892	XM_011250217.1	140.00	-25.26	2 21	1214 1235	19
mmu-miR-1892	XM_006527480.3	149.00	-23.22	2 19	2365 2387	18
mmu-miR-1892	XM_006527480.3	143.00	-23.31	2 21	3105 3127	20
mmu-miR-1892	XM_006527480.3	140.00	-20.94	3 21	4225 4246	18
mmu-miR-1892	NM_001004357.2	166.00	-23.14	2 19	6374 6395	17
mmu-miR-1892	NM_001004357.2	148.00	-23.40	2 20	2625 2644	18
mmu-miR-1892	NM_001004357.2	140.00	-30.84	2 20	309 330	19
mmu-miR-1892	XM_017314944.1	140.00	-13.05	2 9	988 1009	7
mmu-miR-1892	XM_011248983.2	160.00	-24.00	2 17	884 905	15
mmu-miR-1892	XM_011248983.2	143.00	-26.85	2 21	1395 1418	21
mmu-miR-1892	XM_011248983.2	142.00	-13.44	2 11	1082 1103	9
mmu-miR-1892	NM_001113515.2	160.00	-24.13	2 19	1364 1384	17
mmu-miR-1892	NM_001113515.2	156.00	-21.59	2 19	1832 1852	17
mmu-miR-1892	NM_001113515.2	144.00	-32.58	2 18	1336 1358	17
mmu-miR-1892	NM_009930.2	144.00	-28.22	2 18	3181 3203	17
mmu-miR-1892	NM_009930.2	142.00	-23.36	2 16	3451 3473	15
mmu-miR-1892	NM_009930.2	140.00	-22.22	2 17	517 538	15
mmu-miR-1892	NM_007739.2	155.00	-23.55	2 16	1757 1778	14
mmu-miR-1892	NM_007739.2	151.00	-17.39	2 12	3038 3059	10
mmu-miR-1892	NM_007739.2	141.00	-22.02	2 18	923 944	16
mmu-miR-1892	NM_007739.2	141.00	-20.27	2 18	1487 1508	16
mmu-miR-1892	XM_011240755.1	146.00	-25.26	2 16	1046 1068	15
mmu-miR-1892	XM_011240755.1	143.00	-29.76	2 21	2549 2571	20
mmu-miR-1892	XM_006532938.2	162.00	-23.98	2 20	2106 2129	20
mmu-miR-1892	XM_006532938.2	159.00	-28.53	2 21	608 632	22
mmu-miR-1892	XM_006532938.2	152.00	-17.54	2 20	1675 1694	18
mmu-miR-1892	XM_006532938.2	147.00	-25.58	2 20	2667 2688	18
mmu-miR-1892	XM_011247208.1	149.00	-31.71	2 21	6256 6278	21
mmu-miR-1892	XM_011247208.1	143.00	-28.56	2 21	352 376	22
mmu-miR-1892	XM_011247208.1	143.00	-21.38	2 21	1832 1855	21
mmu-miR-1892	XM_011248602.2	153.00	-19.66	2 18	1687 1708	16
mmu-miR-1892	NM_011957.2	144.00	-24.00	2 18	1744 1766	17
mmu-miR-1892	XM_011242311.2	146.00	-28.25	2 21	759 783	22
mmu-miR-1892	XM_011242311.2	145.00	-22.50	2 21	2076 2100	22
mmu-miR-1892	XM_011242311.2	140.00	-25.92	2 17	2925 2946	15
mmu-miR-1892	NM_001289782.1	152.00	-29.13	2 21	490 511	19
mmu-miR-1892	NM_001198841.1	154.00	-20.39	2 19	350 371	17
mmu-miR-1892	NM_018866.2	142.00	-14.53	2 13	196 216	11
mmu-miR-1892	NM_018866.2	140.00	-24.31	2 21	313 334	19
mmu-miR-1892	XM_006517308.3	158.00	-27.56	2 20	1498 1516	18
mmu-miR-1892	NM_030206.4	167.00	-36.09	2 21	1828 1850	20
mmu-miR-1892	NM_007817.2	140.00	-14.48	2 9	1018 1039	7
mmu-miR-1892	NM_015814.2	140.00	-26.43	2 21	1730 1751	19
mmu-miR-1892	NM_010055.3	153.00	-25.58	2 21	2468 2490	21

mmu-miR-1892	NM_010055.3	148.00	-28.76	2 21	2150 2178	26
mmu-miR-1892	NM_010055.3	143.00	-30.00	3 20	2506 2527	17
mmu-miR-1892	NM_010055.3	141.00	-23.81	2 21	80 99	19
mmu-miR-1892	NM_010055.3	140.00	-14.52	2 9	1628 1649	7
mmu-miR-1892	NM_152915.1	145.00	-16.56	2 14	561 582	12
mmu-miR-1892	XM_011243391.2	150.00	-29.38	2 21	7059 7086	25
mmu-miR-1892	XM_011243391.2	143.00	-24.66	2 21	8564 8586	20
mmu-miR-1892	XM_011243391.2	142.00	-22.80	2 20	7895 7917	19
mmu-miR-1892	XM_011243391.2	140.00	-22.38	3 18	255 277	16
mmu-miR-1892	NM_019759.2	147.00	-21.57	2 21	225 248	21
mmu-miR-1892	NM_019759.2	141.00	-22.93	2 20	1104 1124	18
mmu-miR-1892	XM_006506808.3	159.00	-25.71	2 21	1297 1319	20
mmu-miR-1892	NM_007883.3	168.00	-26.77	2 21	5647 5668	19
mmu-miR-1892	NM_007883.3	163.00	-27.41	2 21	2878 2900	20
mmu-miR-1892	NM_001160049.1	148.00	-21.94	2 21	3719 3740	19
mmu-miR-1892	NM_001160049.1	142.00	-16.99	2 15	2904 2925	13
mmu-miR-1892	NM_001160049.1	141.00	-28.07	2 21	212 235	22
mmu-miR-1892	XM_006528026.2	144.00	-22.40	2 17	339 360	15
mmu-miR-1892	NM_133643.4	140.00	-20.73	2 13	1225 1246	11
mmu-miR-1892	NM_133643.4	140.00	-27.22	2 21	4005 4026	19
mmu-miR-1892	NM_019397.3	150.00	-24.88	2 21	1539 1563	22
mmu-miR-1892	XM_006520618.2	147.00	-19.96	2 21	2101 2123	20
mmu-miR-1892	NM_015744.4	143.00	-22.40	2 21	2231 2255	22
mmu-miR-1892	NM_178676.4	148.00	-22.42	2 21	391 412	19
mmu-miR-1892	NM_178676.4	142.00	-30.17	2 16	1 23	15
mmu-miR-1892	NM_010137.3	145.00	-16.23	2 10	3333 3354	8
mmu-miR-1892	NM_010137.3	140.00	-26.88	2 21	3495 3516	19
mmu-miR-1892	NM_010140.3	142.00	-22.58	2 19	3884 3905	17
mmu-miR-1892	XM_011238916.2	146.00	-19.47	2 20	123 146	20
mmu-miR-1892	XM_006511221.3	149.00	-19.95	2 14	4301 4322	12
mmu-miR-1892	XM_006511221.3	143.00	-19.30	2 20	724 745	18
mmu-miR-5113	XM_011241948.2	154.00	-19.27	2 22	2140 2163	22
mmu-miR-5113	XM_011241948.2	146.00	-21.68	2 20	1895 1918	19
mmu-miR-5113	XM_006515980.3	149.00	-15.42	2 19	44 67	18
mmu-miR-5113	XM_006515980.3	146.00	-17.91	2 22	1280 1303	22
mmu-miR-5113	XM_006515980.3	143.00	-17.49	2 20	1459 1481	18
mmu-miR-5113	XM_006515980.3	140.00	-25.16	2 22	345 368	21
mmu-miR-5113	NM_029982.1	152.00	-23.79	3 22	429 452	20
mmu-miR-5113	NM_138955.3	163.00	-25.46	2 20	2453 2475	18
mmu-miR-5113	NM_138955.3	155.00	-25.70	2 22	3516 3540	22
mmu-miR-5113	NM_138955.3	154.00	-24.48	2 21	2473 2494	19
mmu-miR-5113	NM_138955.3	144.00	-23.35	2 17	2185 2207	15
mmu-miR-5113	NM_138955.3	140.00	-27.29	3 22	2656 2684	25
mmu-miR-5113	XM_006540566.1	152.00	-18.20	2 21	4183 4205	19
mmu-miR-5113	XM_006540566.1	145.00	-24.99	2 22	3103 3125	20
mmu-miR-5113	XM_006540566.1	140.00	-13.76	2 9	4588 4610	7
mmu-miR-5113	XM_006540566.1	140.00	-14.05	2 9	4648 4670	7
mmu-miR-5113	NM_153151.3	143.00	-17.84	2 17	1139 1162	16
mmu-miR-5113	NM_172580.1	140.00	-16.36	2 9	103 125	7
mmu-miR-5113	NM_009608.4	141.00	-21.11	3 16	835 856	13
mmu-miR-5113	NM_009608.4	140.00	-16.64	2 9	1320 1342	7
mmu-miR-5113	NM_033268.4	160.00	-23.17	2 17	2870 2892	15
mmu-miR-5113	XM_006498086.3	148.00	-22.26	2 18	7648 7672	18
mmu-miR-5113	XM_006498086.3	141.00	-24.70	2 22	1964 1983	20
mmu-miR-5113	XM_006498086.3	140.00	-17.02	2 9	6997 7019	7
mmu-miR-5113	NM_013906.3	143.00	-21.91	2 21	569 592	20
mmu-miR-5113	XM_006538374.1	165.00	-26.46	2 22	1256 1278	20
mmu-miR-5113	XM_006538374.1	152.00	-22.77	2 22	9050 9073	21
mmu-miR-5113	XM_006538374.1	143.00	-22.34	2 16	5515 5537	14
mmu-miR-5113	XM_006505388.2	156.00	-15.43	2 21	5337 5359	19
mmu-miR-5113	XM_006505388.2	151.00	-18.33	2 22	2316 2340	22
mmu-miR-5113	XM_006505388.2	149.00	-21.55	2 19	2779 2802	18
mmu-miR-5113	XM_006505388.2	148.00	-24.24	2 22	34 57	21
mmu-miR-5113	XM_006505388.2	140.00	-17.73	2 19	6803 6827	19
mmu-miR-5113	NM_009605.4	143.00	-20.77	3 22	126 147	19

mmu-miR-5113	NM_001291930.1	159.00	-21.32	2 22	3918 3939	20
mmu-miR-5113	NM_001291930.1	155.00	-19.79	2 21	115 139	21
mmu-miR-5113	NM_001291930.1	155.00	-24.34	2 22	527 548	20
mmu-miR-5113	NM_001291930.1	142.00	-22.88	3 21	594 618	20
mmu-miR-5113	NM_001291930.1	142.00	-21.73	2 19	4322 4344	17
mmu-miR-5113	XM_017312667.1	155.00	-20.12	2 17	5868 5891	16
mmu-miR-5113	XM_017312667.1	146.00	-17.83	2 11	1021 1043	9
mmu-miR-5113	XM_017312667.1	142.00	-22.18	2 19	8261 8283	17
mmu-miR-5113	XM_017312667.1	141.00	-24.26	3 22	5629 5651	19
mmu-miR-5113	NM_008032.3	140.00	-15.97	2 9	3677 3699	7
mmu-miR-5113	NM_153178.4	153.00	-29.09	2 18	6307 6329	16
mmu-miR-5113	NM_153178.4	146.00	-16.33	3 19	6295 6317	16
mmu-miR-5113	NM_153178.4	145.00	-18.14	2 10	8007 8029	8
mmu-miR-5113	NM_153178.4	140.00	-16.53	2 17	2297 2319	15
mmu-miR-5113	NM_153178.4	140.00	-19.37	2 22	5400 5423	21
mmu-miR-5113	XM_011250176.1	148.00	-24.36	2 20	3900 3922	19
mmu-miR-5113	XM_011250176.1	147.00	-21.29	2 22	4766 4787	20
mmu-miR-5113	XM_011250176.1	146.00	-19.92	2 11	3095 3117	9
mmu-miR-5113	XM_011250176.1	145.00	-19.78	2 10	6474 6496	8
mmu-miR-5113	XM_011250176.1	140.00	-22.49	2 17	1 17	15
mmu-miR-5113	XM_006498287.3	150.00	-25.51	2 22	1845 1865	20
mmu-miR-5113	NM_031185.3	145.00	-18.63	2 20	5791 5812	18
mmu-miR-5113	NM_134072.1	141.00	-16.41	2 19	1485 1508	18
mmu-miR-5113	XM_006532024.3	143.00	-17.23	2 13	937 960	12
mmu-miR-5113	XM_006495613.3	143.00	-17.02	2 20	1595 1617	18
mmu-miR-5113	XM_006508990.2	153.00	-20.74	2 22	2097 2119	20
mmu-miR-5113	XM_006508990.2	147.00	-18.76	2 22	4470 4494	22
mmu-miR-5113	XM_006508990.2	144.00	-20.90	3 21	1404 1426	18
mmu-miR-5113	XM_006508990.2	144.00	-23.74	2 21	8222 8244	19
mmu-miR-5113	NM_172790.2	160.00	-22.41	2 17	3676 3698	15
mmu-miR-5113	NM_172790.2	147.00	-19.51	2 18	3654 3675	16
mmu-miR-5113	NM_172790.2	142.00	-18.86	2 20	1233 1256	19
mmu-miR-5113	NM_172790.2	140.00	-19.35	2 22	2558 2581	21
mmu-miR-5113	NM_172790.2	140.00	-19.92	2 9	5643 5665	7
mmu-miR-5113	NM_009675.2	145.00	-21.46	2 20	2803 2827	20
mmu-miR-5113	NM_013912.3	148.00	-23.19	2 22	1965 1988	21
mmu-miR-5113	NM_013912.3	147.00	-19.89	2 17	301 324	16
mmu-miR-5113	NM_013912.3	147.00	-20.47	2 22	1288 1309	20
mmu-miR-5113	NM_013912.3	141.00	-16.05	3 22	1510 1532	19
mmu-miR-5113	NM_207231.1	152.00	-19.25	2 22	1433 1456	21
mmu-miR-5113	XM_006522398.3	141.00	-17.08	2 10	100 122	8
mmu-miR-5113	NM_029823.2	172.00	-26.39	2 21	892 914	19
mmu-miR-5113	NM_029823.2	140.00	-15.63	2 9	435 457	7
mmu-miR-5113	NM_023048.5	151.00	-22.00	2 13	472 495	12
mmu-miR-5113	NM_023048.5	142.00	-23.95	2 20	2689 2712	19
mmu-miR-5113	NM_025711.3	157.00	-20.23	2 22	249 271	20
mmu-miR-5113	NM_001310070.1	151.00	-31.92	2 22	139 163	22
mmu-miR-5113	NM_001310070.1	140.00	-17.62	2 22	648 671	21
mmu-miR-5113	NM_007881.4	167.00	-29.16	2 20	1502 1524	18
mmu-miR-5113	NM_007881.4	156.00	-20.70	2 22	766 789	21
mmu-miR-5113	NM_007881.4	152.00	-18.65	2 13	1421 1443	11
mmu-miR-5113	NM_007881.4	148.00	-21.16	3 17	4119 4141	14
mmu-miR-5113	NM_001290469.1	160.00	-22.70	2 22	3379 3404	23
mmu-miR-5113	NM_001290469.1	152.00	-23.63	2 18	3433 3456	17
mmu-miR-5113	NM_001290469.1	151.00	-18.08	2 21	2551 2574	20
mmu-miR-5113	NM_001290469.1	143.00	-19.94	3 21	1054 1077	19
mmu-miR-5113	NM_001290469.1	140.00	-21.48	2 22	3502 3525	21
mmu-miR-5113	NM_013415.5	148.00	-25.39	3 21	695 717	18
mmu-miR-5113	NM_013415.5	142.00	-24.09	3 19	2717 2739	16
mmu-miR-5113	NM_013415.5	140.00	-21.32	3 22	2027 2051	21
mmu-miR-5113	NM_009722.3	140.00	-27.78	2 22	2345 2369	22
mmu-miR-5113	XM_006529727.2	148.00	-23.65	2 22	817 840	21
mmu-miR-5113	XM_006529727.2	147.00	-19.56	3 20	4691 4713	17
mmu-miR-5113	XM_006529727.2	141.00	-20.57	2 18	4317 4339	16
mmu-miR-5113	XM_006529727.2	140.00	-15.63	2 9	4129 4151	7

mmu-miR-5113	XM_006507725.2	156.00	-25.44	2 21	89 111	19
mmu-miR-5113	XM_006507725.2	145.00	-21.93	3 22	2217 2239	19
mmu-miR-5113	XM_006507725.2	143.00	-21.62	2 22	2685 2706	20
mmu-miR-5113	XM_006507725.2	142.00	-16.18	2 11	1848 1870	9
mmu-miR-5113	NM_001159407.1	148.00	-20.77	2 22	2036 2059	21
mmu-miR-5113	NM_001159407.1	140.00	-24.80	3 22	1394 1418	21
mmu-miR-5113	XM_006540762.3	154.00	-20.04	2 17	78 99	15
mmu-miR-5113	XM_006540762.3	146.00	-22.33	2 15	176 198	13
mmu-miR-5113	NM_013863.5	146.00	-18.32	2 11	1375 1397	9
mmu-miR-5113	NM_007529.2	169.00	-23.88	2 19	1339 1362	18
mmu-miR-5113	XM_006502291.3	140.00	-18.87	2 22	998 1022	22
mmu-miR-5113	XM_006502291.3	140.00	-15.97	2 9	2730 2752	7
mmu-miR-5113	XM_006510701.3	165.00	-26.84	2 22	6078 6098	20
mmu-miR-5113	XM_006510701.3	161.00	-27.13	2 22	5968 5988	20
mmu-miR-5113	NM_178782.4	148.00	-18.51	2 13	1304 1326	11
mmu-miR-5113	NM_178782.4	145.00	-20.11	2 18	5998 6020	16
mmu-miR-5113	XM_006503323.3	159.00	-29.82	3 22	2612 2638	23
mmu-miR-5113	NM_007555.4	149.00	-20.76	2 22	754 776	20
mmu-miR-5113	NM_007555.4	148.00	-23.65	2 22	3199 3224	23
mmu-miR-5113	NM_007555.4	145.00	-17.70	2 10	1112 1134	8
mmu-miR-5113	NM_028472.2	144.00	-17.07	2 21	2589 2611	19
mmu-miR-5113	NM_020508.4	161.00	-25.36	2 22	2354 2373	20
mmu-miR-5113	NM_020508.4	147.00	-20.38	2 22	2385 2406	20
mmu-miR-5113	NM_020508.4	146.00	-25.46	2 19	5271 5293	17
mmu-miR-5113	NM_020508.4	145.00	-20.23	3 22	115 137	19
mmu-miR-5113	XM_006524328.3	161.00	-25.82	3 22	3916 3938	19
mmu-miR-5113	XM_006524328.3	160.00	-22.58	2 18	4163 4186	17
mmu-miR-5113	XM_006524328.3	152.00	-20.04	2 19	5659 5680	17
mmu-miR-5113	NM_178692.3	148.00	-18.67	2 13	2775 2797	11
mmu-miR-5113	NM_026979.5	147.00	-18.57	2 20	1183 1205	18
mmu-miR-5113	XM_006503665.3	142.00	-21.54	3 20	1080 1105	20
mmu-miR-5113	NM_001097617.1	140.00	-21.82	2 18	2843 2867	18
mmu-miR-5113	NM_001285867.1	162.00	-29.66	2 22	2546 2568	21
mmu-miR-5113	NM_001285867.1	161.00	-24.09	2 18	4849 4871	16
mmu-miR-5113	NM_001285867.1	158.00	-21.60	2 22	4689 4711	21
mmu-miR-5113	NM_001285867.1	150.00	-20.68	3 19	1724 1746	16
mmu-miR-5113	NM_001285867.1	140.00	-17.37	3 22	1117 1140	20
mmu-miR-5113	XM_011246128.2	146.00	-27.96	4 19	8387 8409	15
mmu-miR-5113	XM_011246128.2	145.00	-22.72	2 14	3768 3790	12
mmu-miR-5113	XM_011246128.2	141.00	-17.96	3 22	6598 6620	19
mmu-miR-5113	NM_007588.2	148.00	-25.13	2 22	1435 1458	21
mmu-miR-5113	NM_001190379.1	142.00	-21.34	3 19	1077 1099	16
mmu-miR-5113	NM_001190379.1	141.00	-28.91	2 22	2025 2047	20
mmu-miR-5113	NM_001190379.1	140.00	-21.45	3 21	4235 4257	18
mmu-miR-5113	XM_011246479.2	143.00	-16.25	2 12	151 173	10
mmu-miR-5113	XM_006501443.3	147.00	-20.50	3 20	2100 2122	17
mmu-miR-5113	NM_009824.2	158.00	-16.46	2 20	6215 6238	19
mmu-miR-5113	NM_009824.2	140.00	-21.74	3 19	3449 3470	16
mmu-miR-5113	NM_009824.2	140.00	-16.48	2 19	5394 5419	20
mmu-miR-5113	NM_009824.2	140.00	-17.77	2 17	6878 6900	15
mmu-miR-5113	NM_019626.3	164.00	-32.23	2 22	1678 1701	21
mmu-miR-5113	NM_019626.3	142.00	-22.53	2 21	888 912	21
mmu-miR-5113	XM_006526449.3	164.00	-22.59	2 18	2051 2074	17
mmu-miR-5113	NM_001310648.1	143.00	-14.17	2 22	1150 1174	22
mmu-miR-5113	NM_001001178.1	144.00	-24.94	2 22	3836 3859	21
mmu-miR-5113	NM_001013784.1	149.00	-20.39	2 18	2907 2929	16
mmu-miR-5113	NM_001013784.1	147.00	-18.17	2 12	2029 2051	10
mmu-miR-5113	NM_001013784.1	142.00	-24.21	2 20	3842 3867	21
mmu-miR-5113	XM_006508867.2	169.00	-28.73	2 22	936 958	20
mmu-miR-5113	XM_006508885.1	169.00	-28.72	2 22	1020 1042	20
mmu-miR-5113	XM_006533653.3	140.00	-24.10	2 9	71 93	7
mmu-miR-5113	NM_025422.4	140.00	-18.20	3 22	884 907	20
mmu-miR-5113	XM_006509966.3	167.00	-28.02	2 20	852 874	18
mmu-miR-5113	NM_001110322.1	142.00	-20.56	2 15	592 614	13
mmu-miR-5113	NM_007655.3	150.00	-24.94	3 15	37 59	12

mmu-miR-5113	XM_011245344.2	151.00	-22.21	2 22	5764 5789	23
mmu-miR-5113	XM_011245344.2	150.00	-22.24	2 20	14843 14867	20
mmu-miR-5113	XM_011245344.2	149.00	-19.81	3 18	11730 11752	15
mmu-miR-5113	XM_011245344.2	147.00	-18.47	2 22	964 985	20
mmu-miR-5113	XM_011245344.2	144.00	-25.11	2 22	1011 1039	26
mmu-miR-5113	XM_011245344.2	143.00	-20.85	2 16	10453 10475	14
mmu-miR-5113	XM_006531321.3	148.00	-20.24	2 22	609 632	21
mmu-miR-5113	XM_006531321.3	142.00	-16.98	2 19	28 50	17
mmu-miR-5113	XM_006530131.1	157.00	-24.59	3 18	6936 6958	15
mmu-miR-5113	XM_006530131.1	153.00	-22.87	2 14	1279 1301	12
mmu-miR-5113	XM_006530131.1	141.00	-18.33	2 10	4086 4108	8
mmu-miR-5113	XM_011250217.1	152.00	-22.75	2 13	662 684	11
mmu-miR-5113	XM_011250217.1	145.00	-26.09	2 15	120 143	14
mmu-miR-5113	XM_006527480.3	147.00	-20.87	2 21	2241 2266	22
mmu-miR-5113	NM_001004357.2	160.00	-21.64	2 20	4041 4063	19
mmu-miR-5113	NM_001004357.2	157.00	-17.80	2 19	3637 3660	18
mmu-miR-5113	NM_001004357.2	145.00	-21.50	3 22	5740 5760	19
mmu-miR-5113	NM_001004357.2	143.00	-21.10	3 22	110 131	19
mmu-miR-5113	NM_001004357.2	143.00	-20.53	3 22	3406 3430	21
mmu-miR-5113	NM_001004357.2	142.00	-15.69	2 11	5244 5266	9
mmu-miR-5113	XM_017314944.1	146.00	-25.73	2 22	1840 1862	21
mmu-miR-5113	NM_001113515.2	140.00	-30.19	2 21	99 122	20
mmu-miR-5113	NM_009930.2	148.00	-27.45	2 17	159 181	15
mmu-miR-5113	NM_009930.2	148.00	-16.99	2 22	5345 5368	21
mmu-miR-5113	NM_009930.2	143.00	-17.54	3 20	149 171	17
mmu-miR-5113	NM_009930.2	141.00	-19.19	2 22	751 773	20
mmu-miR-5113	NM_009930.2	140.00	-21.14	2 13	5202 5224	11
mmu-miR-5113	XM_011247208.1	140.00	-18.50	2 19	3980 4001	17
mmu-miR-5113	XM_011248602.2	141.00	-16.44	2 20	2121 2142	18
mmu-miR-5113	XM_011248602.2	140.00	-27.17	2 22	1054 1077	21
mmu-miR-5113	NM_011957.2	156.00	-23.98	2 20	476 502	22
mmu-miR-5113	XM_011242311.2	143.00	-21.64	2 20	3368 3390	18
mmu-miR-5113	XM_011242311.2	140.00	-14.96	3 21	5279 5301	18
mmu-miR-5113	NM_030206.4	163.00	-28.43	2 18	2074 2099	19
mmu-miR-5113	NM_030206.4	143.00	-22.80	3 22	1800 1826	23
mmu-miR-5113	NM_030206.4	140.00	-17.68	2 9	686 708	7
mmu-miR-5113	NM_001190449.1	156.00	-22.12	2 15	165 186	13
mmu-miR-5113	NM_001190448.1	151.00	-18.04	2 21	1955 1978	20
mmu-miR-5113	NM_001190448.1	150.00	-20.07	2 20	702 725	19
mmu-miR-5113	NM_015814.2	152.00	-19.64	2 13	1259 1281	11
mmu-miR-5113	NM_015814.2	144.00	-16.40	2 21	2874 2896	19
mmu-miR-5113	NM_015814.2	143.00	-19.53	2 22	341 366	23
mmu-miR-5113	NM_015814.2	140.00	-19.48	2 17	1310 1332	15
mmu-miR-5113	NM_152915.1	151.00	-26.07	3 22	119 143	21
mmu-miR-5113	NM_152915.1	140.00	-16.82	2 19	165 186	17
mmu-miR-5113	NM_009345.2	142.00	-16.62	2 11	732 754	9
mmu-miR-5113	NM_027293.1	147.00	-20.63	2 12	5141 5163	10
mmu-miR-5113	NM_027293.1	140.00	-25.71	2 22	1915 1940	23
mmu-miR-5113	XM_011243391.2	157.00	-24.29	2 18	5670 5692	16
mmu-miR-5113	XM_011243391.2	153.00	-18.05	2 21	5219 5241	20
mmu-miR-5113	XM_011243391.2	152.00	-23.26	2 22	4773 4798	23
mmu-miR-5113	XM_011243391.2	144.00	-20.10	2 21	7373 7395	19
mmu-miR-5113	XM_011243391.2	140.00	-14.50	2 9	3207 3229	7
mmu-miR-5113	XM_011243391.2	140.00	-16.58	2 21	4650 4672	19
mmu-miR-5113	XM_011243391.2	140.00	-21.94	2 13	4884 4906	11
mmu-miR-5113	XM_006506808.3	151.00	-22.30	2 22	2413 2434	20
mmu-miR-5113	XM_006506808.3	143.00	-21.85	2 21	2631 2654	20
mmu-miR-5113	NM_001317365.1	146.00	-16.60	2 22	2057 2077	20
mmu-miR-5113	NM_007883.3	140.00	-15.22	2 9	3457 3479	7
mmu-miR-5113	NM_001160049.1	151.00	-24.65	2 20	2794 2816	18
mmu-miR-5113	XM_006528026.2	162.00	-25.36	2 21	2196 2220	21
mmu-miR-5113	NM_133643.4	164.00	-24.97	2 17	6586 6608	15
mmu-miR-5113	NM_133643.4	161.00	-21.85	2 22	1126 1146	20
mmu-miR-5113	NM_133643.4	159.00	-23.46	2 21	2560 2583	20
mmu-miR-5113	NM_133643.4	154.00	-24.99	2 20	7651 7675	20

mmu-miR-5113	NM_133643.4	147.00	-28.86	3 21	5550 5577	23
mmu-miR-5113	NM_133643.4	143.00	-15.00	2 14	6452 6473	12
mmu-miR-5113	NM_133643.4	142.00	-21.67	2 15	5666 5688	13
mmu-miR-5113	NM_019397.3	161.00	-24.38	2 22	205 227	20
mmu-miR-5113	NM_019397.3	156.00	-23.24	2 22	649 672	21
mmu-miR-5113	NM_019397.3	154.00	-24.61	2 19	1844 1866	17
mmu-miR-5113	NM_001291145.1	171.00	-29.29	2 22	727 751	22
mmu-miR-5113	NM_001291145.1	146.00	-26.18	2 21	2688 2709	19
mmu-miR-5113	NM_010133.2	152.00	-18.45	2 21	2250 2272	19
mmu-miR-5113	NM_010133.2	150.00	-21.05	2 19	2232 2254	17
mmu-miR-5113	NM_010133.2	148.00	-20.01	2 21	1416 1436	19
mmu-miR-5113	XM_006520618.2	140.00	-20.14	2 9	1095 1117	7
mmu-miR-5113	NM_015744.4	140.00	-13.85	2 9	1148 1170	7
mmu-miR-5113	NM_010137.3	142.00	-26.30	3 20	4213 4237	19
mmu-miR-5113	NM_010137.3	141.00	-24.64	2 20	121 142	18
mmu-miR-5113	XM_011238916.2	168.00	-26.57	2 18	1963 1986	17
mmu-miR-5113	XM_011238916.2	144.00	-17.72	2 19	1853 1874	17
mmu-miR-5113	XM_011238916.2	142.00	-24.55	2 19	530 552	17
mmu-miR-5113	XM_011238916.2	141.00	-19.97	3 14	2011 2033	11
mmu-miR-5113	XM_006511221.3	140.00	-17.06	2 9	2893 2915	7
mmu-miR-5113	NM_010174.1	149.00	-15.72	2 20	417 438	18
mmu-miR-5128	NM_144544.2	141.00	-18.83	2 22	706 728	20
mmu-miR-5128	XM_011241948.2	144.00	-16.94	2 22	95 118	21
mmu-miR-5128	XM_011241322.1	144.00	-18.73	2 22	3187 3206	20
mmu-miR-5128	XM_006515980.3	142.00	-19.93	3 15	1354 1376	12
mmu-miR-5128	XM_006498086.3	143.00	-19.09	2 20	1639 1661	18
mmu-miR-5128	XM_006538374.1	147.00	-20.66	3 20	5758 5780	17
mmu-miR-5128	XM_006538374.1	142.00	-25.49	2 22	535 555	20
mmu-miR-5128	XM_006505388.2	151.00	-24.35	2 22	3791 3816	23
mmu-miR-5128	XM_006505388.2	145.00	-14.40	2 20	2956 2977	18
mmu-miR-5128	NM_001291930.1	145.00	-18.75	2 18	2925 2947	16
mmu-miR-5128	NM_001291930.1	140.00	-21.27	2 22	3498 3521	21
mmu-miR-5128	XM_017312667.1	143.00	-27.94	3 20	4342 4364	17
mmu-miR-5128	XM_017312667.1	141.00	-14.96	2 20	2254 2275	18
mmu-miR-5128	NM_153178.4	145.00	-12.65	2 10	5320 5342	8
mmu-miR-5128	NM_153178.4	145.00	-22.33	3 20	6339 6360	17
mmu-miR-5128	NM_153178.4	143.00	-10.98	2 15	5639 5659	13
mmu-miR-5128	NM_153178.4	142.00	-15.11	2 22	3078 3098	20
mmu-miR-5128	NM_009645.2	140.00	-10.69	2 9	1422 1444	7
mmu-miR-5128	XM_006498287.3	143.00	-10.05	2 12	767 789	10
mmu-miR-5128	NM_031185.3	140.00	-20.48	2 15	3173 3194	13
mmu-miR-5128	XM_006495613.3	143.00	-28.85	4 22	1339 1360	18
mmu-miR-5128	NM_001303431.1	148.00	-24.40	3 21	1092 1114	18
mmu-miR-5128	XM_006508990.2	155.00	-28.99	3 20	1870 1892	17
mmu-miR-5128	NM_001301354.1	151.00	-25.48	3 22	1038 1059	19
mmu-miR-5128	NM_207231.1	141.00	-13.53	2 10	711 733	8
mmu-miR-5128	NM_025758.4	141.00	-14.32	2 14	776 798	12
mmu-miR-5128	NM_007881.4	157.00	-18.42	2 22	1107 1127	20
mmu-miR-5128	NM_001290469.1	142.00	-25.58	2 22	3544 3564	20
mmu-miR-5128	XM_006507725.2	143.00	-15.18	2 22	2629 2650	20
mmu-miR-5128	NM_001159407.1	148.00	-12.54	2 13	2649 2671	11
mmu-miR-5128	NM_013863.5	155.00	-20.79	2 20	1383 1405	18
mmu-miR-5128	XM_006502291.3	143.00	-24.60	3 18	301 322	15
mmu-miR-5128	XM_006502291.3	141.00	-19.14	2 22	3733 3755	20
mmu-miR-5128	XM_006510701.3	141.00	-27.80	2 22	1767 1790	21
mmu-miR-5128	NM_178782.4	149.00	-26.68	3 18	1378 1400	15
mmu-miR-5128	NM_007555.4	147.00	-15.59	2 12	3172 3194	10
mmu-miR-5128	NM_028472.2	147.00	-25.44	2 21	634 657	20
mmu-miR-5128	XM_006524328.3	160.00	-23.28	2 13	861 883	11
mmu-miR-5128	XM_006524328.3	144.00	-23.34	2 22	5705 5728	21
mmu-miR-5128	NM_001097617.1	160.00	-22.07	2 17	796 818	15
mmu-miR-5128	NM_001097617.1	140.00	-16.31	2 20	306 326	18
mmu-miR-5128	NM_001097617.1	140.00	-20.02	3 18	2144 2168	17
mmu-miR-5128	XM_011246128.2	142.00	-13.04	2 12	2083 2106	11
mmu-miR-5128	XM_011246479.2	143.00	-15.04	2 22	1200 1221	20

mmu-miR-5128	XM_006520253.3	149.00	-20.04	2	22	2486	2508	20
mmu-miR-5128	NM_019626.3	153.00	-22.11	2	19	2004	2027	18
mmu-miR-5128	NM_019626.3	147.00	-21.21	2	21	1312	1335	20
mmu-miR-5128	NM_001001178.1	145.00	-19.78	2	22	1785	1807	20
mmu-miR-5128	NM_001013784.1	153.00	-21.23	2	18	2076	2098	16
mmu-miR-5128	NM_007655.3	158.00	-24.98	2	17	1195	1216	15
mmu-miR-5128	XM_011245344.2	152.00	-19.43	2	22	5199	5223	22
mmu-miR-5128	XM_011245344.2	140.00	-17.31	2	22	11253	11277	22
mmu-miR-5128	XM_011245344.2	140.00	-20.70	3	22	13986	14009	20
mmu-miR-5128	XM_017312731.1	146.00	-21.14	2	22	1439	1461	21
mmu-miR-5128	XM_006531321.3	157.00	-25.09	2	22	442	462	20
mmu-miR-5128	NM_001301295.1	145.00	-20.02	2	18	975	997	16
mmu-miR-5128	XM_006527480.3	140.00	-29.93	4	22	3104	3128	20
mmu-miR-5128	NM_001004357.2	152.00	-23.57	2	22	6861	6884	21
mmu-miR-5128	NM_001113515.2	140.00	-22.37	3	17	975	997	14
mmu-miR-5128	NM_009930.2	145.00	-16.45	2	10	656	678	8
mmu-miR-5128	XM_011240755.1	152.00	-13.99	2	21	4165	4187	19
mmu-miR-5128	XM_011247208.1	141.00	-16.16	2	19	4494	4517	18
mmu-miR-5128	NM_027293.1	151.00	-25.90	2	22	2236	2257	20
mmu-miR-5128	NM_027293.1	150.00	-21.20	2	21	7240	7261	19
mmu-miR-5128	XM_011243391.2	144.00	-24.32	2	22	4718	4742	22
mmu-miR-5128	NM_001317365.1	142.00	-20.79	2	22	2958	2978	20
mmu-miR-5128	NM_001317365.1	142.00	-18.92	2	22	3347	3369	21
mmu-miR-5128	NM_001317365.1	140.00	-21.32	2	22	2494	2517	21
mmu-miR-5128	NM_007883.3	157.00	-19.61	2	20	3495	3516	18
mmu-miR-5128	NM_007883.3	146.00	-13.37	2	17	3465	3486	15
mmu-miR-5128	NM_007883.3	144.00	-14.77	2	13	5037	5059	11
mmu-miR-5128	XM_006514873.2	152.00	-16.42	2	22	1310	1334	22
mmu-miR-5128	XM_006514873.2	144.00	-25.37	2	22	379	402	21
mmu-miR-5128	NM_001160049.1	144.00	-29.83	2	22	3718	3741	21
mmu-miR-5128	XM_006528026.2	148.00	-19.80	2	14	951	974	13
mmu-miR-5128	NM_001291145.1	153.00	-16.65	2	16	2968	2989	14
mmu-miR-5128	NM_010133.2	143.00	-19.05	2	22	2208	2229	20
mmu-miR-5128	NM_015744.4	156.00	-16.94	2	17	1818	1840	15
mmu-miR-5128	NM_015744.4	141.00	-24.08	3	15	1733	1756	13
mmu-miR-5128	XM_006511221.3	144.00	-15.80	2	19	5373	5397	19
mmu-miR-5128	NM_010174.1	144.00	-21.28	2	21	538	560	19
mmu-miR-344i	XM_017320219.1	146.00	-22.56	2	17	4085	4103	15
mmu-miR-344i	XM_006515980.3	150.00	-17.79	2	19	398	417	17
mmu-miR-344i	NM_138955.3	143.00	-19.77	2	18	2176	2194	16
mmu-miR-344i	NM_138955.3	140.00	-20.90	3	18	1997	2017	16
mmu-miR-344i	XM_006540566.1	145.00	-25.04	2	10	4198	4217	8
mmu-miR-344i	XM_006540566.1	140.00	-14.37	2	9	7277	7296	7
mmu-miR-344i	NM_009608.4	160.00	-22.21	2	18	328	348	17
mmu-miR-344i	XM_006498086.3	143.00	-23.46	2	16	5427	5446	14
mmu-miR-344i	XM_006498086.3	143.00	-18.43	2	16	7349	7368	14
mmu-miR-344i	NM_021475.2	142.00	-13.44	2	11	1380	1399	9
mmu-miR-344i	NM_013906.3	147.00	-20.02	2	19	2681	2700	18
mmu-miR-344i	NM_013906.3	144.00	-22.81	3	17	477	496	14
mmu-miR-344i	XM_006538374.1	140.00	-20.58	2	18	5891	5911	17
mmu-miR-344i	XM_006505388.2	150.00	-28.56	2	19	513	532	17
mmu-miR-344i	XM_006505388.2	147.00	-18.51	2	16	120	139	14
mmu-miR-344i	XM_006505388.2	145.00	-16.31	2	19	3492	3512	18
mmu-miR-344i	XM_006505388.2	140.00	-17.39	2	9	1218	1237	7
mmu-miR-344i	NM_001291930.1	140.00	-29.18	3	19	3951	3972	18
mmu-miR-344i	XM_017312667.1	156.00	-21.72	2	14	5142	5162	13
mmu-miR-344i	XM_017312667.1	143.00	-18.00	2	17	6605	6625	16
mmu-miR-344i	NM_153178.4	152.00	-26.30	2	19	2080	2098	17
mmu-miR-344i	NM_153178.4	140.00	-15.64	2	9	6842	6861	7
mmu-miR-344i	XM_011250176.1	148.00	-25.83	2	17	2574	2593	15
mmu-miR-344i	XM_011250176.1	148.00	-19.63	2	14	5356	5377	14
mmu-miR-344i	XM_011250176.1	145.00	-14.25	2	19	6878	6899	19
mmu-miR-344i	NM_053080.3	142.00	-14.37	2	11	2238	2257	9
mmu-miR-344i	XM_006495613.3	140.00	-20.30	2	19	2435	2453	17
mmu-miR-344i	NM_001303431.1	142.00	-12.80	2	11	1784	1803	9

mmu-miR-344i	XM_006508990.2	154.00	-26.92	2 19	7364 7383	17
mmu-miR-344i	XM_006508990.2	149.00	-19.34	2 19	1399 1420	19
mmu-miR-344i	NM_172790.2	149.00	-17.18	2 18	2840 2857	16
mmu-miR-344i	NM_172790.2	141.00	-21.53	2 19	2293 2314	19
mmu-miR-344i	NM_001301354.1	145.00	-16.96	2 10	134 153	8
mmu-miR-344i	NM_025758.4	144.00	-22.87	2 14	394 415	14
mmu-miR-344i	NM_001310070.1	142.00	-22.61	3 19	123 142	16
mmu-miR-344i	NM_007881.4	146.00	-28.99	2 15	3211 3230	13
mmu-miR-344i	NM_007881.4	144.00	-30.17	2 19	870 888	17
mmu-miR-344i	NM_001290469.1	140.00	-13.11	2 9	786 805	7
mmu-miR-344i	NM_009722.3	144.00	-23.70	2 19	2206 2227	19
mmu-miR-344i	NM_009722.3	142.00	-18.25	2 11	2295 2314	9
mmu-miR-344i	NM_009722.3	140.00	-19.35	2 17	1431 1450	15
mmu-miR-344i	XM_006529727.2	141.00	-20.96	3 19	3927 3948	18
mmu-miR-344i	NM_020025.4	142.00	-15.01	2 19	2829 2846	17
mmu-miR-344i	NM_020025.4	141.00	-13.70	2 12	2476 2494	10
mmu-miR-344i	NM_001159407.1	153.00	-24.29	2 19	1879 1900	19
mmu-miR-344i	XM_006502291.3	158.00	-24.53	2 16	4244 4265	16
mmu-miR-344i	XM_006502291.3	142.00	-25.00	2 16	3974 3995	16
mmu-miR-344i	XM_006502291.3	141.00	-25.37	2 19	3612 3632	18
mmu-miR-344i	XM_006510701.3	145.00	-24.50	3 19	1050 1070	17
mmu-miR-344i	XM_006510701.3	144.00	-22.27	2 18	1248 1270	19
mmu-miR-344i	NM_007549.2	142.00	-17.52	2 16	138 158	15
mmu-miR-344i	NM_020508.4	143.00	-24.87	3 19	3495 3515	18
mmu-miR-344i	NM_020508.4	141.00	-22.18	2 19	4470 4492	20
mmu-miR-344i	NM_020508.4	140.00	-18.82	2 17	4782 4801	15
mmu-miR-344i	XM_006524328.3	157.00	-26.82	2 19	704 725	19
mmu-miR-344i	XM_006524328.3	150.00	-18.99	2 17	4245 4263	15
mmu-miR-344i	XM_006524328.3	142.00	-21.90	3 19	3697 3716	16
mmu-miR-344i	XM_006524328.3	142.00	-22.15	2 19	4862 4881	17
mmu-miR-344i	XM_006524328.3	141.00	-23.65	2 19	3637 3657	18
mmu-miR-344i	XM_006503665.3	144.00	-22.74	2 18	1787 1807	17
mmu-miR-344i	NM_001097617.1	141.00	-27.69	2 19	155 175	18
mmu-miR-344i	NM_001285867.1	141.00	-20.93	2 18	2590 2609	16
mmu-miR-344i	NM_007588.2	148.00	-18.69	2 19	880 898	17
mmu-miR-344i	NM_001190379.1	153.00	-28.71	2 18	2238 2257	16
mmu-miR-344i	NM_001190379.1	152.00	-20.93	2 19	4083 4101	17
mmu-miR-344i	NM_001190379.1	151.00	-15.94	2 16	4267 4286	14
mmu-miR-344i	NM_001190379.1	148.00	-27.80	3 17	1214 1233	14
mmu-miR-344i	NM_001190379.1	142.00	-28.47	2 19	4421 4440	17
mmu-miR-344i	NM_001190379.1	140.00	-17.70	2 9	4097 4116	7
mmu-miR-344i	XM_011246479.2	144.00	-19.69	2 19	642 660	17
mmu-miR-344i	XM_006501443.3	148.00	-24.84	2 18	1222 1242	17
mmu-miR-344i	XM_006520253.3	159.00	-23.56	2 16	619 638	14
mmu-miR-344i	XM_006520253.3	148.00	-22.21	2 17	862 881	15
mmu-miR-344i	XM_006500947.3	146.00	-14.37	2 19	1111 1130	17
mmu-miR-344i	XM_006500947.3	144.00	-26.38	2 19	20 38	17
mmu-miR-344i	NM_009824.2	147.00	-19.74	2 14	1759 1780	14
mmu-miR-344i	NM_009824.2	146.00	-22.18	2 19	2431 2450	17
mmu-miR-344i	NM_009824.2	142.00	-27.97	3 19	4181 4200	16
mmu-miR-344i	NM_001013784.1	142.00	-13.12	2 11	2382 2401	9
mmu-miR-344i	NM_001110322.1	153.00	-22.63	2 18	847 866	16
mmu-miR-344i	NM_007655.3	142.00	-23.38	2 19	697 716	17
mmu-miR-344i	XM_011245344.2	152.00	-19.18	2 18	583 603	17
mmu-miR-344i	XM_011245344.2	146.00	-20.89	2 19	13338 13357	17
mmu-miR-344i	XM_011245344.2	141.00	-13.98	2 14	10619 10638	12
mmu-miR-344i	XM_011245344.2	140.00	-25.13	2 18	1413 1434	18
mmu-miR-344i	XM_011245344.2	140.00	-17.51	2 19	13719 13737	17
mmu-miR-344i	XM_006531321.3	146.00	-18.03	2 19	548 567	17
mmu-miR-344i	XM_006523030.3	145.00	-16.00	2 10	1474 1493	8
mmu-miR-344i	XM_006530131.1	145.00	-20.89	2 10	7557 7576	8
mmu-miR-344i	XM_006530131.1	141.00	-25.44	3 18	4645 4664	15
mmu-miR-344i	XM_006527480.3	140.00	-14.73	2 9	2442 2461	7
mmu-miR-344i	NM_001113515.2	147.00	-19.82	2 16	1626 1645	14
mmu-miR-344i	NM_001113515.2	144.00	-24.67	2 19	2374 2392	17

mmu-miR-344i	NM_007739.2	142.00	-24.57	2 16	952 972	15
mmu-miR-344i	XM_011240755.1	142.00	-21.35	2 19	1544 1563	17
mmu-miR-344i	XM_006532938.2	155.00	-16.87	2 16	1310 1329	14
mmu-miR-344i	XM_011247208.1	144.00	-20.93	2 18	3147 3167	17
mmu-miR-344i	XM_017315376.1	146.00	-20.45	2 19	471 490	17
mmu-miR-344i	XM_017315376.1	141.00	-25.27	3 19	874 895	18
mmu-miR-344i	XM_011248602.2	142.00	-12.42	2 11	2260 2279	9
mmu-miR-344i	XM_011242311.2	153.00	-23.54	2 16	1338 1360	17
mmu-miR-344i	XM_011242311.2	149.00	-24.27	2 18	3850 3869	16
mmu-miR-344i	XM_011242311.2	149.00	-23.70	2 19	4410 4430	18
mmu-miR-344i	NM_030206.4	147.00	-21.06	2 18	1059 1082	20
mmu-miR-344i	NM_030206.4	146.00	-22.20	2 16	1841 1863	17
mmu-miR-344i	XM_006540596.2	142.00	-17.28	2 19	1138 1157	17
mmu-miR-344i	NM_001190448.1	144.00	-24.00	2 13	232 251	11
mmu-miR-344i	NM_001190448.1	140.00	-19.66	2 18	827 847	17
mmu-miR-344i	NM_152915.1	162.00	-34.31	3 19	965 984	16
mmu-miR-344i	NM_009345.2	140.00	-15.32	2 9	2056 2075	7
mmu-miR-344i	NM_027293.1	141.00	-24.84	2 19	3068 3089	19
mmu-miR-344i	NM_027293.1	140.00	-12.89	2 9	4898 4917	7
mmu-miR-344i	NM_027293.1	140.00	-15.03	2 9	6163 6182	7
mmu-miR-344i	XM_011243391.2	151.00	-21.89	2 19	6995 7014	18
mmu-miR-344i	XM_011243391.2	146.00	-16.01	2 19	7808 7827	17
mmu-miR-344i	XM_006506808.3	166.00	-32.47	2 19	415 434	17
mmu-miR-344i	NM_001317365.1	152.00	-18.28	2 19	493 514	19
mmu-miR-344i	NM_007883.3	143.00	-14.37	2 17	1570 1590	16
mmu-miR-344i	NM_001160049.1	144.00	-19.41	2 19	3867 3888	19
mmu-miR-344i	XM_006528026.2	156.00	-24.18	2 19	3209 3227	17
mmu-miR-344i	NM_001291145.1	147.00	-25.17	2 19	1118 1138	19
mmu-miR-344i	NM_001291145.1	143.00	-23.32	2 19	2388 2405	17
mmu-miR-344i	NM_015744.4	144.00	-16.19	2 18	382 403	18
mmu-miR-344i	NM_015744.4	143.00	-20.28	2 16	1101 1120	14
mmu-miR-344i	NM_178676.4	146.00	-14.22	2 19	2171 2190	17
mmu-miR-344i	NM_010137.3	155.00	-20.26	2 16	4188 4207	14
mmu-miR-344i	NM_010137.3	152.00	-19.47	2 18	843 864	18
mmu-miR-344i	XM_011238916.2	143.00	-19.64	2 18	1651 1669	16
mmu-miR-344i	XM_011238916.2	140.00	-28.55	4 17	66 85	13
mmu-miR-344i	XM_011238916.2	140.00	-20.89	3 19	3153 3171	16
mmu-miR-344i	XM_011238916.2	140.00	-13.72	2 9	4227 4246	7
mmu-miR-344i	XM_006511221.3	145.00	-20.94	2 19	3157 3179	20
mmu-miR-344i	NM_010174.1	142.00	-15.22	2 11	333 352	9
mmu-miR-6238	NM_024283.3	143.00	-16.29	2 20	414 435	18
mmu-miR-6238	XM_006534171.3	141.00	-17.35	2 21	1220 1239	19
mmu-miR-6238	NM_138955.3	150.00	-17.37	2 21	3589 3609	19
mmu-miR-6238	XM_006498086.3	150.00	-9.81	2 19	8158 8179	17
mmu-miR-6238	XM_006498086.3	144.00	-8.09	2 17	4991 5012	15
mmu-miR-6238	XM_006538374.1	152.00	-9.54	2 19	10343 10363	17
mmu-miR-6238	XM_006495613.3	144.00	-17.22	2 19	500 520	17
mmu-miR-6238	XM_006495613.3	144.00	-21.10	2 21	2486 2507	19
mmu-miR-6238	NM_207231.1	142.00	-15.28	2 21	73 96	21
mmu-miR-6238	NM_007881.4	152.00	-16.93	2 21	1569 1590	19
mmu-miR-6238	XM_006529727.2	159.00	-19.56	2 20	643 664	18
mmu-miR-6238	XM_006529727.2	155.00	-17.17	2 21	8279 8301	20
mmu-miR-6238	NM_020025.4	142.00	-14.03	2 21	1220 1240	19
mmu-miR-6238	NM_020025.4	140.00	-8.51	2 21	2606 2627	19
mmu-miR-6238	NM_001159407.1	146.00	-11.53	3 21	4453 4473	18
mmu-miR-6238	NM_178782.4	147.00	-13.37	2 18	182 202	16
mmu-miR-6238	NM_007555.4	147.00	-18.59	2 20	2366 2387	18
mmu-miR-6238	NM_009785.1	141.00	-13.34	2 14	3365 3386	12
mmu-miR-6238	XM_011246128.2	140.00	-9.07	2 21	9663 9684	19
mmu-miR-6238	NM_181315.4	154.00	-21.37	2 21	2378 2398	19
mmu-miR-6238	NM_009824.2	153.00	-21.19	2 20	3105 3125	18
mmu-miR-6238	NM_001001178.1	141.00	-19.80	2 19	1882 1905	19
mmu-miR-6238	XM_011245344.2	143.00	-6.72	2 21	10589 10611	20
mmu-miR-6238	XM_011245344.2	140.00	-12.75	2 9	11871 11892	7
mmu-miR-6238	XM_011245344.2	140.00	-13.86	2 21	11947 11968	19

mmu-miR-6238	XM_006523030.3	147.00	-16.40	2 20	1367 1388	18
mmu-miR-6238	XM_006523030.3	143.00	-16.60	2 21	1178 1200	20
mmu-miR-6238	NM_020001.2	142.00	-10.80	2 20	805 827	19
mmu-miR-6238	NM_009930.2	157.00	-20.27	2 20	1412 1438	23
mmu-miR-6238	NM_007739.2	146.00	-13.19	2 20	4643 4665	19
mmu-miR-6238	XM_011247208.1	144.00	-13.08	3 21	7583 7604	18
mmu-miR-6238	NM_027293.1	140.00	-9.17	2 9	1415 1436	7
mmu-miR-6238	XM_011243391.2	140.00	-18.77	2 18	3823 3847	19
mmu-miR-6238	NM_007883.3	141.00	-14.84	2 14	1197 1218	12
mmu-miR-6238	NM_133643.4	144.00	-15.52	3 19	7407 7427	16
mmu-miR-6238	NM_178676.4	143.00	-10.13	2 20	2128 2149	18
mmu-miR-6238	XM_011238916.2	160.00	-12.58	2 21	1918 1939	19
mmu-miR-6238	XM_011238916.2	156.00	-15.34	2 21	2737 2758	19
mmu-miR-6238	NM_024406.2	141.00	-12.04	2 19	544 561	17
mmu-miR-6351	XM_011241948.2	140.00	-21.01	3 18	791 814	16
mmu-miR-6351	XM_011241322.1	153.00	-18.31	2 22	1472 1492	20
mmu-miR-6351	NM_138955.3	152.00	-24.60	2 18	131 155	18
mmu-miR-6351	NM_172580.1	141.00	-21.40	3 22	1497 1517	19
mmu-miR-6351	XM_006538374.1	152.00	-22.44	2 19	6635 6656	17
mmu-miR-6351	XM_006538374.1	148.00	-30.59	2 21	961 983	19
mmu-miR-6351	XM_006505388.2	146.00	-20.74	2 20	6961 6984	19
mmu-miR-6351	XM_006500919.3	140.00	-21.35	2 20	1386 1412	22
mmu-miR-6351	NM_153178.4	143.00	-23.03	3 16	141 163	13
mmu-miR-6351	NM_153178.4	143.00	-25.99	2 22	7946 7971	23
mmu-miR-6351	XM_011250176.1	140.00	-24.44	3 21	1251 1273	18
mmu-miR-6351	XM_011250176.1	140.00	-25.26	2 22	3324 3348	22
mmu-miR-6351	NM_031185.3	149.00	-30.20	4 22	1308 1330	18
mmu-miR-6351	XM_006508990.2	151.00	-26.02	2 22	6747 6768	20
mmu-miR-6351	XM_006508990.2	140.00	-26.92	2 19	3700 3721	17
mmu-miR-6351	NM_172790.2	161.00	-24.03	2 22	5504 5526	20
mmu-miR-6351	NM_172790.2	152.00	-19.76	2 22	5617 5641	22
mmu-miR-6351	NM_172790.2	149.00	-21.93	2 21	287 307	19
mmu-miR-6351	NM_001305585.1	142.00	-26.41	2 20	780 803	19
mmu-miR-6351	NM_001301354.1	140.00	-20.52	2 17	1664 1686	15
mmu-miR-6351	NM_001080819.1	149.00	-25.07	3 22	5603 5625	19
mmu-miR-6351	NM_001080819.1	147.00	-19.81	2 21	792 815	20
mmu-miR-6351	NM_001080819.1	144.00	-22.61	2 20	1773 1793	18
mmu-miR-6351	NM_001080819.1	140.00	-25.62	2 15	534 555	13
mmu-miR-6351	XM_006522398.3	141.00	-15.53	2 20	615 636	18
mmu-miR-6351	NM_029823.2	155.00	-20.91	2 20	415 437	18
mmu-miR-6351	NM_023048.5	149.00	-16.25	2 18	750 772	16
mmu-miR-6351	NM_007881.4	140.00	-23.47	2 15	2879 2900	13
mmu-miR-6351	NM_013415.5	148.00	-15.82	2 17	1362 1384	15
mmu-miR-6351	XM_006529727.2	142.00	-14.27	2 11	3785 3807	9
mmu-miR-6351	XM_006507725.2	141.00	-19.84	2 18	2688 2710	16
mmu-miR-6351	NM_007529.2	147.00	-18.30	2 18	1215 1236	16
mmu-miR-6351	XM_006510701.3	147.00	-22.29	3 22	5691 5712	19
mmu-miR-6351	NM_178782.4	149.00	-24.50	2 19	2238 2261	18
mmu-miR-6351	XM_006503323.3	143.00	-18.72	2 16	1014 1036	14
mmu-miR-6351	NM_028472.2	149.00	-22.69	2 22	596 618	20
mmu-miR-6351	NM_026979.5	150.00	-25.24	2 22	200 222	21
mmu-miR-6351	NM_023143.3	142.00	-19.21	2 22	2435 2455	20
mmu-miR-6351	NM_001285867.1	148.00	-20.97	2 19	1040 1064	19
mmu-miR-6351	NM_007588.2	163.00	-23.40	2 16	992 1014	14
mmu-miR-6351	NM_007588.2	148.00	-20.85	2 19	1941 1966	20
mmu-miR-6351	NM_007588.2	147.00	-27.03	3 22	1467 1492	22
mmu-miR-6351	NM_027416.3	156.00	-23.72	2 21	1024 1050	23
mmu-miR-6351	NM_027416.3	145.00	-27.36	2 19	1187 1212	20
mmu-miR-6351	XM_006520253.3	154.00	-21.03	2 15	575 597	13
mmu-miR-6351	NM_009824.2	149.00	-13.66	2 14	6139 6161	12
mmu-miR-6351	NM_001013784.1	144.00	-16.75	2 22	3870 3893	21
mmu-miR-6351	XM_006508867.2	142.00	-16.11	2 11	568 590	9
mmu-miR-6351	NM_025422.4	144.00	-23.18	2 21	28 47	19
mmu-miR-6351	XM_011245344.2	145.00	-20.80	2 22	9328 9350	20
mmu-miR-6351	XM_006523030.3	140.00	-24.39	2 22	659 683	22

mmu-miR-6351	XM_006532007.3	141.00	-22.25	2 19	685 710	20
mmu-miR-6351	XM_006530131.1	146.00	-29.98	2 19	2990 3012	17
mmu-miR-6351	XM_006530131.1	141.00	-21.85	3 19	3172 3196	18
mmu-miR-6351	XM_006530131.1	140.00	-24.75	2 18	4975 5000	19
mmu-miR-6351	XM_006527480.3	141.00	-18.02	2 18	2195 2217	16
mmu-miR-6351	NM_001004357.2	140.00	-21.96	2 19	247 268	17
mmu-miR-6351	XM_017314944.1	140.00	-26.61	2 21	1862 1884	19
mmu-miR-6351	XM_011247208.1	142.00	-22.00	3 19	781 803	16
mmu-miR-6351	XM_011242311.2	142.00	-23.94	2 22	2141 2166	24
mmu-miR-6351	NM_018866.2	157.00	-24.48	2 19	33 56	18
mmu-miR-6351	NM_007817.2	159.00	-24.36	2 21	1132 1157	22
mmu-miR-6351	NM_007817.2	142.00	-21.95	2 21	1077 1101	21
mmu-miR-6351	XM_011243391.2	156.00	-20.38	2 18	5649 5673	18
mmu-miR-6351	NM_007883.3	144.00	-15.99	2 21	3727 3749	19
mmu-miR-6351	NM_010090.2	143.00	-21.70	2 22	1470 1496	24
mmu-miR-6351	NM_001160049.1	147.00	-24.36	2 18	3955 3976	16
mmu-miR-6351	XM_006528026.2	143.00	-22.82	2 22	5207 5225	20
mmu-miR-6351	NM_010133.2	147.00	-19.44	2 20	2277 2296	18
mmu-miR-6378	XM_017320219.1	154.00	-19.67	2 15	3218 3239	13
mmu-miR-6378	XM_011241948.2	146.00	-17.14	2 18	955 974	16
mmu-miR-6378	XM_011241322.1	141.00	-24.55	2 20	1921 1946	22
mmu-miR-6378	XM_006515980.3	140.00	-14.92	2 9	620 641	7
mmu-miR-6378	XM_006540566.1	142.00	-17.14	2 15	4284 4305	13
mmu-miR-6378	NM_033268.4	144.00	-22.41	2 19	1042 1062	17
mmu-miR-6378	NM_021475.2	163.00	-22.39	2 18	1543 1563	16
mmu-miR-6378	NM_013906.3	142.00	-19.57	2 20	2316 2338	19
mmu-miR-6378	XM_006538374.1	143.00	-27.66	2 21	2610 2632	20
mmu-miR-6378	XM_006505388.2	143.00	-23.73	2 16	6715 6736	14
mmu-miR-6378	XM_006505388.2	140.00	-13.14	2 9	2615 2636	7
mmu-miR-6378	XM_006500919.3	144.00	-26.34	2 18	525 550	20
mmu-miR-6378	XM_006500919.3	140.00	-15.79	2 9	416 437	7
mmu-miR-6378	XM_017312667.1	151.00	-29.48	2 21	2571 2593	20
mmu-miR-6378	NM_008032.3	156.00	-19.48	2 13	1735 1756	11
mmu-miR-6378	NM_153178.4	146.00	-25.27	2 21	567 587	19
mmu-miR-6378	XM_011250176.1	154.00	-18.39	2 16	438 460	15
mmu-miR-6378	XM_011250176.1	148.00	-18.47	2 19	639 659	17
mmu-miR-6378	XM_011250176.1	146.00	-24.98	2 21	2568 2588	19
mmu-miR-6378	XM_011250176.1	144.00	-14.20	2 18	1086 1108	17
mmu-miR-6378	XM_011250176.1	142.00	-21.93	2 16	258 280	15
mmu-miR-6378	XM_006532024.3	146.00	-20.10	2 11	212 233	9
mmu-miR-6378	NM_172790.2	145.00	-16.04	2 14	1910 1931	12
mmu-miR-6378	NM_172790.2	142.00	-12.97	2 11	551 572	9
mmu-miR-6378	NM_172790.2	141.00	-20.77	2 10	878 899	8
mmu-miR-6378	NM_009675.2	146.00	-26.81	2 20	1092 1115	20
mmu-miR-6378	NM_007482.3	140.00	-10.87	2 9	920 941	7
mmu-miR-6378	NM_001080819.1	156.00	-20.16	2 21	4565 4586	19
mmu-miR-6378	NM_001080819.1	156.00	-19.42	2 19	7551 7571	17
mmu-miR-6378	XM_006522398.3	145.00	-19.07	2 16	286 306	14
mmu-miR-6378	NM_029823.2	146.00	-18.25	2 11	423 444	9
mmu-miR-6378	NM_025711.3	147.00	-15.30	2 12	450 471	10
mmu-miR-6378	NM_001290469.1	152.00	-20.51	2 18	2095 2117	17
mmu-miR-6378	XM_006507725.2	140.00	-28.50	2 17	2338 2359	15
mmu-miR-6378	NM_013863.5	140.00	-25.72	2 20	385 407	20
mmu-miR-6378	XM_006502291.3	147.00	-28.92	2 21	5719 5741	20
mmu-miR-6378	XM_006502291.3	146.00	-24.10	2 19	1698 1719	17
mmu-miR-6378	XM_006510701.3	141.00	-17.72	2 10	1424 1445	8
mmu-miR-6378	NM_178782.4	149.00	-29.84	2 19	5293 5317	20
mmu-miR-6378	NM_007549.2	153.00	-16.82	2 20	1050 1070	18
mmu-miR-6378	NM_007549.2	144.00	-20.98	2 17	6 27	15
mmu-miR-6378	NM_009785.1	146.00	-15.53	2 11	3410 3431	9
mmu-miR-6378	XM_011246128.2	140.00	-19.72	2 17	1762 1783	15
mmu-miR-6378	NM_007588.2	146.00	-22.85	3 19	607 628	16
mmu-miR-6378	NM_181315.4	145.00	-17.17	2 15	3103 3126	15
mmu-miR-6378	XM_006520253.3	145.00	-27.29	2 18	4091 4112	16
mmu-miR-6378	NM_009824.2	143.00	-21.82	2 16	4332 4353	14

mmu-miR-6378	NM_028804.1	143.00	-18.15	2 12	865 886	10
mmu-miR-6378	XM_006533653.3	146.00	-22.55	3 21	713 736	20
mmu-miR-6378	XM_006509966.3	146.00	-23.85	2 15	730 751	13
mmu-miR-6378	XM_006503371.2	158.00	-30.42	2 19	3626 3647	17
mmu-miR-6378	NM_013459.3	145.00	-22.74	2 18	325 346	16
mmu-miR-6378	XM_006513165.1	148.00	-17.01	2 21	632 651	19
mmu-miR-6378	NM_001113515.2	147.00	-16.63	2 13	695 717	12
mmu-miR-6378	NM_001113515.2	147.00	-18.95	2 17	3107 3129	16
mmu-miR-6378	NM_001113515.2	140.00	-22.69	2 20	641 660	18
mmu-miR-6378	NM_001113515.2	140.00	-15.42	2 9	1380 1401	7
mmu-miR-6378	NM_009930.2	159.00	-23.28	2 17	803 825	16
mmu-miR-6378	NM_009930.2	159.00	-27.02	2 17	1487 1509	16
mmu-miR-6378	NM_009930.2	144.00	-18.35	2 20	3216 3237	19
mmu-miR-6378	NM_009930.2	140.00	-16.44	2 9	2163 2184	7
mmu-miR-6378	NM_009930.2	140.00	-15.70	2 9	2424 2445	7
mmu-miR-6378	NM_009930.2	140.00	-15.18	2 9	2649 2670	7
mmu-miR-6378	XM_011240755.1	148.00	-20.65	2 19	2653 2678	21
mmu-miR-6378	XM_006532938.2	141.00	-13.24	2 10	1513 1534	8
mmu-miR-6378	NM_019696.2	146.00	-25.95	2 21	1630 1650	19
mmu-miR-6378	XM_011242311.2	157.00	-28.16	2 21	255 281	24
mmu-miR-6378	XM_011242311.2	145.00	-23.62	2 20	3800 3820	18
mmu-miR-6378	NM_007792.4	144.00	-14.20	2 17	653 674	15
mmu-miR-6378	NM_030206.4	141.00	-21.01	2 21	531 552	20
mmu-miR-6378	XM_006540596.2	140.00	-20.45	2 13	686 707	11
mmu-miR-6378	NM_001190448.1	146.00	-18.49	2 19	1046 1067	17
mmu-miR-6378	NM_015814.2	155.00	-21.74	2 17	352 375	17
mmu-miR-6378	NM_010055.3	142.00	-17.87	2 11	369 390	9
mmu-miR-6378	NM_027293.1	142.00	-20.04	2 15	783 804	13
mmu-miR-6378	XM_006506808.3	155.00	-18.41	2 21	149 171	20
mmu-miR-6378	XM_006506808.3	143.00	-26.76	2 20	2246 2267	18
mmu-miR-6378	NM_001317365.1	142.00	-14.61	2 20	441 463	19
mmu-miR-6378	XM_006514873.2	149.00	-19.15	2 15	4022 4045	15
mmu-miR-6378	NM_133643.4	140.00	-24.47	2 19	4125 4149	20
mmu-miR-6378	NM_015744.4	148.00	-18.50	2 21	8 29	19
mmu-miR-3473e	NM_024283.3	159.00	-24.90	2 20	243 263	18
mmu-miR-3473e	XM_017320219.1	152.00	-20.90	2 19	657 676	17
mmu-miR-3473e	XM_017320219.1	142.00	-20.61	2 16	792 814	16
mmu-miR-3473e	XM_017320219.1	142.00	-27.55	2 19	2236 2256	17
mmu-miR-3473e	NM_144544.2	144.00	-25.05	2 17	28 48	15
mmu-miR-3473e	NM_001271580.1	151.00	-28.30	2 18	47 66	16
mmu-miR-3473e	XM_011241948.2	142.00	-21.42	2 20	2459 2483	22
mmu-miR-3473e	XM_011241322.1	153.00	-30.14	2 18	93 113	16
mmu-miR-3473e	XM_011241322.1	149.00	-23.09	2 19	3038 3060	19
mmu-miR-3473e	XM_011241322.1	147.00	-23.49	2 16	2476 2496	14
mmu-miR-3473e	XM_011241322.1	145.00	-26.40	2 18	1701 1721	16
mmu-miR-3473e	XM_011241322.1	144.00	-21.17	2 18	1052 1074	18
mmu-miR-3473e	XM_006515980.3	152.00	-22.21	2 18	755 776	17
mmu-miR-3473e	NM_138955.3	154.00	-27.73	2 19	2346 2366	17
mmu-miR-3473e	NM_138955.3	145.00	-24.27	2 18	2211 2231	16
mmu-miR-3473e	XM_006540566.1	159.00	-20.12	2 16	728 748	14
mmu-miR-3473e	NM_153151.3	143.00	-20.32	2 19	979 997	17
mmu-miR-3473e	NM_009608.4	159.00	-27.10	2 19	921 941	18
mmu-miR-3473e	NM_009608.4	149.00	-22.41	2 20	80 103	21
mmu-miR-3473e	XM_006498086.3	141.00	-20.41	3 18	7395 7415	15
mmu-miR-3473e	NM_013906.3	150.00	-20.43	2 19	672 692	17
mmu-miR-3473e	XM_006538374.1	147.00	-18.33	2 19	2539 2557	17
mmu-miR-3473e	XM_006538374.1	144.00	-27.07	2 20	6323 6341	18
mmu-miR-3473e	XM_006538374.1	143.00	-25.38	2 18	10415 10434	16
mmu-miR-3473e	XM_006505388.2	163.00	-23.67	2 18	3571 3590	16
mmu-miR-3473e	XM_006505388.2	151.00	-25.00	2 19	756 776	18
mmu-miR-3473e	XM_006505388.2	147.00	-20.21	2 16	2733 2753	14
mmu-miR-3473e	XM_006505388.2	144.00	-23.30	2 19	940 959	17
mmu-miR-3473e	XM_006505388.2	141.00	-24.03	2 17	3787 3805	15
mmu-miR-3473e	XM_006505388.2	140.00	-25.56	2 19	1027 1049	19
mmu-miR-3473e	NM_001291930.1	145.00	-19.00	2 10	3680 3700	8

mmu-miR-3473e	NM_001291930.1	141.00	-17.42	2 18	3348 3368	16
mmu-miR-3473e	XM_017312667.1	159.00	-38.02	2 20	6214 6234	18
mmu-miR-3473e	XM_017312667.1	151.00	-25.76	2 20	1873 1893	18
mmu-miR-3473e	XM_017312667.1	145.00	-23.66	2 10	7563 7583	8
mmu-miR-3473e	NM_153178.4	150.00	-24.40	2 19	4143 4163	17
mmu-miR-3473e	NM_153178.4	145.00	-22.81	2 18	4854 4874	16
mmu-miR-3473e	XM_011250176.1	148.00	-24.45	2 20	492 517	23
mmu-miR-3473e	XM_011250176.1	143.00	-19.22	2 16	3442 3462	14
mmu-miR-3473e	XM_011250176.1	140.00	-18.61	2 9	7192 7212	7
mmu-miR-3473e	XM_006498287.3	152.00	-24.80	2 20	1379 1399	19
mmu-miR-3473e	XM_006498287.3	149.00	-26.70	2 14	552 572	12
mmu-miR-3473e	XM_006498287.3	140.00	-16.73	2 9	1939 1959	7
mmu-miR-3473e	NM_031185.3	159.00	-27.61	2 20	3762 3782	18
mmu-miR-3473e	XM_011240149.1	153.00	-20.76	2 19	2107 2129	19
mmu-miR-3473e	NM_134072.1	141.00	-22.39	3 18	1748 1768	15
mmu-miR-3473e	NM_053080.3	148.00	-21.49	2 20	1643 1661	18
mmu-miR-3473e	NM_001303431.1	157.00	-25.01	2 18	483 503	16
mmu-miR-3473e	NM_001303431.1	141.00	-25.76	2 18	450 470	16
mmu-miR-3473e	XM_006508990.2	150.00	-21.43	2 11	2978 2998	9
mmu-miR-3473e	XM_006508990.2	145.00	-20.34	2 10	6010 6030	8
mmu-miR-3473e	XM_006508990.2	145.00	-24.38	2 10	7782 7802	8
mmu-miR-3473e	XM_006508990.2	141.00	-21.32	2 19	4353 4374	18
mmu-miR-3473e	XM_006508990.2	140.00	-27.70	2 20	60 78	18
mmu-miR-3473e	NM_172790.2	141.00	-18.12	2 10	1617 1637	8
mmu-miR-3473e	NM_009675.2	153.00	-20.95	2 20	4076 4095	18
mmu-miR-3473e	NM_009675.2	143.00	-20.08	2 16	464 484	14
mmu-miR-3473e	NM_009675.2	140.00	-25.26	2 20	2434 2455	20
mmu-miR-3473e	NM_013912.3	141.00	-17.83	2 16	868 887	14
mmu-miR-3473e	NM_001080819.1	147.00	-19.73	2 16	4746 4766	14
mmu-miR-3473e	NM_001080819.1	146.00	-18.63	2 20	5254 5275	19
mmu-miR-3473e	NM_001080819.1	141.00	-20.61	2 20	3380 3399	18
mmu-miR-3473e	NM_001080819.1	140.00	-21.31	3 18	3481 3502	16
mmu-miR-3473e	XM_006522398.3	157.00	-26.00	2 14	145 165	12
mmu-miR-3473e	NM_023048.5	142.00	-17.96	2 19	2908 2928	17
mmu-miR-3473e	NM_001310070.1	150.00	-29.52	2 20	1363 1387	22
mmu-miR-3473e	NM_007881.4	143.00	-21.28	2 20	2097 2117	18
mmu-miR-3473e	NM_001290469.1	148.00	-26.65	2 20	1009 1029	19
mmu-miR-3473e	XM_006529727.2	140.00	-20.07	2 9	1827 1847	7
mmu-miR-3473e	NM_020025.4	148.00	-16.70	2 15	278 297	13
mmu-miR-3473e	NM_013863.5	149.00	-24.86	2 20	1805 1827	20
mmu-miR-3473e	NM_013863.5	146.00	-25.13	2 20	796 820	22
mmu-miR-3473e	NM_013863.5	146.00	-17.28	2 19	1103 1121	17
mmu-miR-3473e	NM_007529.2	145.00	-31.49	2 19	1464 1485	18
mmu-miR-3473e	XM_006502291.3	149.00	-25.69	3 20	4610 4629	17
mmu-miR-3473e	XM_006502291.3	143.00	-28.40	2 16	194 214	14
mmu-miR-3473e	NM_178782.4	155.00	-24.87	2 20	1392 1412	18
mmu-miR-3473e	NM_178782.4	147.00	-22.83	2 19	1454 1472	17
mmu-miR-3473e	NM_178782.4	143.00	-20.54	2 16	3032 3052	14
mmu-miR-3473e	XM_006503323.3	154.00	-23.75	2 16	667 688	15
mmu-miR-3473e	NM_007555.4	161.00	-28.04	2 18	3401 3421	16
mmu-miR-3473e	NM_020508.4	149.00	-21.95	2 18	3170 3190	16
mmu-miR-3473e	NM_020508.4	147.00	-16.57	2 20	3527 3547	18
mmu-miR-3473e	NM_020508.4	145.00	-23.50	2 16	3662 3685	17
mmu-miR-3473e	XM_006524328.3	162.00	-28.99	2 20	3494 3515	19
mmu-miR-3473e	XM_006524328.3	141.00	-24.50	3 19	5416 5439	19
mmu-miR-3473e	NM_178692.3	157.00	-27.51	2 19	2633 2655	19
mmu-miR-3473e	XM_006503665.3	151.00	-25.27	3 16	1829 1849	13
mmu-miR-3473e	NM_023143.3	171.00	-28.90	2 20	778 798	18
mmu-miR-3473e	NM_001097617.1	149.00	-16.86	2 20	1497 1516	18
mmu-miR-3473e	NM_001285867.1	153.00	-23.25	2 18	1968 1988	16
mmu-miR-3473e	NM_001285867.1	145.00	-23.79	2 10	4336 4356	8
mmu-miR-3473e	NM_001285867.1	144.00	-21.11	3 17	4183 4203	14
mmu-miR-3473e	NM_001285867.1	141.00	-19.14	2 20	3273 3292	18
mmu-miR-3473e	NM_001190379.1	148.00	-21.50	2 20	1936 1958	21
mmu-miR-3473e	NM_001190379.1	145.00	-27.67	2 16	2694 2713	14

mmu-miR-3473e	NM_181315.4	175.00	-41.02	2 20	1688 1708	18
mmu-miR-3473e	NM_181315.4	141.00	-22.57	2 17	966 984	15
mmu-miR-3473e	XM_006520253.3	151.00	-23.28	2 20	3315 3333	18
mmu-miR-3473e	XM_006520253.3	143.00	-25.42	2 20	3909 3927	18
mmu-miR-3473e	XM_006520253.3	143.00	-25.42	2 20	3975 3993	18
mmu-miR-3473e	XM_006500947.3	155.00	-27.72	2 20	1465 1485	18
mmu-miR-3473e	XM_006500947.3	147.00	-17.98	2 18	770 792	18
mmu-miR-3473e	NM_009824.2	162.00	-29.95	2 20	158 180	20
mmu-miR-3473e	NM_009824.2	152.00	-21.72	2 17	6232 6252	15
mmu-miR-3473e	NM_009824.2	151.00	-29.41	2 19	76 100	21
mmu-miR-3473e	NM_009824.2	148.00	-20.34	2 15	6362 6381	13
mmu-miR-3473e	NM_009824.2	146.00	-24.89	2 19	5731 5751	17
mmu-miR-3473e	NM_009824.2	145.00	-23.81	2 10	3623 3643	8
mmu-miR-3473e	NM_009824.2	142.00	-25.87	2 16	3707 3730	17
mmu-miR-3473e	NM_009824.2	142.00	-21.86	2 16	4912 4935	17
mmu-miR-3473e	NM_019626.3	141.00	-20.96	2 14	2102 2122	12
mmu-miR-3473e	XM_006526449.3	154.00	-25.98	2 20	409 433	22
mmu-miR-3473e	XM_006526449.3	143.00	-21.54	2 20	536 553	18
mmu-miR-3473e	NM_028804.1	149.00	-18.87	2 19	607 628	18
mmu-miR-3473e	NM_028804.1	145.00	-27.54	2 19	576 597	18
mmu-miR-3473e	NM_001013784.1	143.00	-23.48	2 18	2116 2135	16
mmu-miR-3473e	NM_001013784.1	140.00	-22.26	2 9	1111 1131	7
mmu-miR-3473e	XM_006509966.3	149.00	-24.59	2 18	808 828	16
mmu-miR-3473e	XM_006509966.3	143.00	-23.16	2 19	744 762	17
mmu-miR-3473e	XM_006509966.3	141.00	-19.05	2 19	954 975	18
mmu-miR-3473e	NM_001110322.1	144.00	-21.02	3 17	1064 1084	14
mmu-miR-3473e	NM_001110322.1	142.00	-25.58	2 20	220 241	19
mmu-miR-3473e	NM_007655.3	155.00	-21.49	2 20	758 778	18
mmu-miR-3473e	XM_006531321.3	144.00	-26.34	2 19	591 615	21
mmu-miR-3473e	XM_006531321.3	140.00	-27.25	2 18	669 690	17
mmu-miR-3473e	XM_006503371.2	148.00	-26.90	2 20	3639 3657	18
mmu-miR-3473e	XM_006523030.3	153.00	-27.37	2 19	285 306	18
mmu-miR-3473e	XM_006513165.1	150.00	-26.10	2 20	1240 1262	20
mmu-miR-3473e	XM_006513165.1	142.00	-29.67	2 20	1299 1320	19
mmu-miR-3473e	XM_006530131.1	146.00	-18.41	2 15	6810 6830	13
mmu-miR-3473e	XM_006530131.1	146.00	-23.70	2 20	7355 7376	19
mmu-miR-3473e	XM_006530131.1	145.00	-17.63	2 16	4336 4355	14
mmu-miR-3473e	XM_006530131.1	145.00	-25.25	2 10	6186 6206	8
mmu-miR-3473e	XM_006530131.1	142.00	-24.70	2 20	8238 8259	19
mmu-miR-3473e	NM_020001.2	148.00	-21.49	2 18	423 445	18
mmu-miR-3473e	XM_006527480.3	157.00	-26.80	2 20	310 329	18
mmu-miR-3473e	XM_006527480.3	143.00	-21.78	2 16	29 49	14
mmu-miR-3473e	XM_006527480.3	142.00	-18.73	2 16	2180 2202	16
mmu-miR-3473e	NM_001004357.2	151.00	-24.13	2 16	3894 3914	14
mmu-miR-3473e	NM_001004357.2	141.00	-21.86	2 20	1750 1774	22
mmu-miR-3473e	NM_009930.2	165.00	-21.28	2 18	4272 4292	16
mmu-miR-3473e	XM_011247208.1	153.00	-24.96	2 18	301 321	16
mmu-miR-3473e	XM_011247208.1	146.00	-20.12	2 15	1743 1763	13
mmu-miR-3473e	XM_011247208.1	143.00	-22.01	3 18	1105 1124	15
mmu-miR-3473e	XM_011247208.1	143.00	-20.95	2 16	4165 4185	14
mmu-miR-3473e	XM_017315376.1	143.00	-26.61	2 18	1010 1032	18
mmu-miR-3473e	XM_011248602.2	140.00	-20.56	2 20	1861 1881	19
mmu-miR-3473e	NM_011957.2	147.00	-23.96	2 12	734 754	10
mmu-miR-3473e	XM_011242311.2	156.00	-27.77	2 18	4393 4417	20
mmu-miR-3473e	XM_011242311.2	148.00	-21.00	2 16	2810 2830	15
mmu-miR-3473e	XM_011242311.2	143.00	-24.76	3 17	1676 1697	15
mmu-miR-3473e	XM_011242311.2	140.00	-21.18	2 20	3705 3725	19
mmu-miR-3473e	NM_001190449.1	159.00	-29.30	2 20	126 146	18
mmu-miR-3473e	NM_015814.2	159.00	-24.17	2 18	1296 1318	18
mmu-miR-3473e	NM_015814.2	152.00	-26.63	2 17	586 606	15
mmu-miR-3473e	NM_010055.3	154.00	-24.96	2 20	600 621	19
mmu-miR-3473e	NM_010055.3	153.00	-27.06	2 16	1583 1605	16
mmu-miR-3473e	NM_010055.3	142.00	-23.74	2 20	1365 1387	20
mmu-miR-3473e	NM_152915.1	153.00	-24.50	2 19	1701 1725	21
mmu-miR-3473e	NM_152915.1	147.00	-23.58	2 14	272 291	12

mmu-miR-3473e	NM_152915.1	145.00	-22.61	2 20	454 473	18
mmu-miR-3473e	NM_027293.1	160.00	-24.77	2 17	3179 3199	15
mmu-miR-3473e	NM_027293.1	152.00	-20.69	2 17	3662 3682	15
mmu-miR-3473e	NM_027293.1	143.00	-26.67	2 17	534 556	17
mmu-miR-3473e	NM_027293.1	142.00	-24.99	2 20	5221 5245	22
mmu-miR-3473e	XM_011243391.2	149.00	-26.37	2 19	6514 6537	20
mmu-miR-3473e	XM_011243391.2	144.00	-20.19	2 17	4128 4148	15
mmu-miR-3473e	NM_019759.2	141.00	-17.69	2 18	1568 1588	16
mmu-miR-3473e	XM_006506808.3	146.00	-23.70	2 15	1998 2018	13
mmu-miR-3473e	XM_006506808.3	140.00	-18.53	2 9	2078 2098	7
mmu-miR-3473e	NM_007883.3	151.00	-22.17	2 20	3182 3202	18
mmu-miR-3473e	NM_007883.3	141.00	-17.24	2 14	1423 1443	12
mmu-miR-3473e	XM_006514873.2	162.00	-26.71	2 20	1807 1828	19
mmu-miR-3473e	XM_006514873.2	142.00	-17.82	2 18	841 862	18
mmu-miR-3473e	XM_006514873.2	140.00	-26.19	2 19	567 586	17
mmu-miR-3473e	NM_133643.4	155.00	-37.83	2 20	4828 4848	18
mmu-miR-3473e	NM_133643.4	144.00	-22.74	2 17	3787 3807	15
mmu-miR-3473e	NM_133643.4	143.00	-21.77	3 16	7646 7666	13
mmu-miR-3473e	NM_001291145.1	152.00	-21.99	2 19	2376 2395	17
mmu-miR-3473e	NM_178676.4	143.00	-22.65	2 20	3099 3119	18
mmu-miR-3473e	NM_178676.4	143.00	-26.71	2 20	3139 3159	18
mmu-miR-3473e	NM_178676.4	140.00	-21.66	3 20	3331 3349	17
mmu-miR-3473e	NM_010137.3	159.00	-21.63	2 20	2117 2135	18
mmu-miR-3473e	NM_010137.3	142.00	-26.55	2 19	2910 2930	17
mmu-miR-3473e	NM_010137.3	140.00	-25.51	2 17	2766 2786	15
mmu-miR-3473e	XM_011238916.2	150.00	-25.06	2 19	406 426	17
mmu-miR-3473e	XM_011238916.2	141.00	-21.78	2 16	2728 2747	14
mmu-miR-7005-5p	XM_017320219.1	154.00	-23.58	2 21	2945 2965	19
mmu-miR-7005-5p	XM_017320219.1	152.00	-31.01	2 19	3300 3320	17
mmu-miR-7005-5p	XM_017320219.1	142.00	-18.92	2 11	2819 2840	9
mmu-miR-7005-5p	NM_029685.1	140.00	-20.18	3 17	958 979	14
mmu-miR-7005-5p	NM_144544.2	144.00	-26.75	2 21	267 288	19
mmu-miR-7005-5p	NM_001033630.1	146.00	-28.37	2 16	295 318	16
mmu-miR-7005-5p	XM_011241948.2	147.00	-30.75	2 21	1578 1601	21
mmu-miR-7005-5p	XM_011241322.1	154.00	-22.69	2 20	2097 2120	20
mmu-miR-7005-5p	XM_011241322.1	144.00	-23.29	2 17	710 731	15
mmu-miR-7005-5p	XM_011241322.1	142.00	-21.84	3 20	3866 3888	18
mmu-miR-7005-5p	XM_006515980.3	155.00	-22.06	2 21	2140 2162	20
mmu-miR-7005-5p	XM_006515980.3	148.00	-18.51	2 17	2758 2779	15
mmu-miR-7005-5p	XM_006515980.3	142.00	-19.83	2 16	717 740	16
mmu-miR-7005-5p	NM_138955.3	151.00	-24.61	2 21	2970 2992	20
mmu-miR-7005-5p	NM_138955.3	148.00	-21.93	2 15	3500 3520	13
mmu-miR-7005-5p	NM_138955.3	147.00	-27.64	2 17	1382 1404	16
mmu-miR-7005-5p	XM_006540566.1	164.00	-26.84	2 17	735 756	15
mmu-miR-7005-5p	XM_006540566.1	150.00	-20.35	2 11	1178 1199	9
mmu-miR-7005-5p	XM_006540566.1	142.00	-23.83	3 16	507 530	15
mmu-miR-7005-5p	XM_006540566.1	140.00	-28.42	2 19	7599 7619	17
mmu-miR-7005-5p	NM_172580.1	146.00	-26.64	2 21	956 979	21
mmu-miR-7005-5p	NM_033268.4	156.00	-26.19	2 21	193 212	19
mmu-miR-7005-5p	XM_006498086.3	148.00	-23.76	2 21	1638 1659	19
mmu-miR-7005-5p	XM_006498086.3	141.00	-24.73	2 18	3131 3152	16
mmu-miR-7005-5p	NM_013906.3	150.00	-27.38	3 21	1608 1631	20
mmu-miR-7005-5p	NM_013906.3	144.00	-28.31	3 21	4124 4145	18
mmu-miR-7005-5p	XM_006538374.1	152.00	-25.53	3 21	924 945	18
mmu-miR-7005-5p	XM_006538374.1	149.00	-27.22	2 14	6438 6459	12
mmu-miR-7005-5p	XM_006538374.1	146.00	-23.74	2 21	3547 3570	21
mmu-miR-7005-5p	XM_006538374.1	142.00	-22.39	2 15	750 771	13
mmu-miR-7005-5p	XM_006538374.1	141.00	-23.90	2 16	7144 7164	14
mmu-miR-7005-5p	XM_006505388.2	150.00	-18.95	2 15	2201 2222	13
mmu-miR-7005-5p	XM_006505388.2	149.00	-29.15	3 20	389 414	21
mmu-miR-7005-5p	XM_006505388.2	143.00	-21.94	2 12	1343 1364	10
mmu-miR-7005-5p	NM_009605.4	150.00	-29.29	3 19	5 26	16
mmu-miR-7005-5p	NM_001291930.1	155.00	-35.16	2 21	3766 3788	20
mmu-miR-7005-5p	NM_001291930.1	150.00	-28.46	2 21	948 973	23
mmu-miR-7005-5p	NM_001291930.1	146.00	-26.17	2 16	3252 3274	15

mmu-miR-7005-5p	NM_001291930.1	144.00	-29.33	2 21	2801 2822	19
mmu-miR-7005-5p	NM_001291930.1	144.00	-22.83	2 17	3135 3156	15
mmu-miR-7005-5p	NM_001291930.1	142.00	-20.15	2 11	1721 1742	9
mmu-miR-7005-5p	NM_001291930.1	140.00	-24.04	2 21	2918 2939	19
mmu-miR-7005-5p	XM_017312667.1	168.00	-28.42	2 21	5886 5907	19
mmu-miR-7005-5p	XM_017312667.1	150.00	-19.78	2 11	4473 4494	9
mmu-miR-7005-5p	XM_017312667.1	147.00	-24.50	2 16	2478 2499	14
mmu-miR-7005-5p	XM_017312667.1	144.00	-24.21	2 18	1581 1603	17
mmu-miR-7005-5p	XM_017312667.1	142.00	-23.12	2 16	3796 3818	15
mmu-miR-7005-5p	XM_017312667.1	140.00	-23.88	2 21	6278 6297	19
mmu-miR-7005-5p	NM_008032.3	158.00	-23.05	2 17	3965 3985	15
mmu-miR-7005-5p	NM_008032.3	154.00	-25.54	2 20	4031 4056	22
mmu-miR-7005-5p	NM_153178.4	152.00	-22.07	2 17	1525 1546	15
mmu-miR-7005-5p	NM_153178.4	152.00	-33.95	3 21	5758 5779	18
mmu-miR-7005-5p	NM_153178.4	147.00	-21.87	3 21	5886 5908	19
mmu-miR-7005-5p	NM_153178.4	146.00	-25.77	3 17	3769 3789	14
mmu-miR-7005-5p	NM_153178.4	146.00	-16.69	2 11	5213 5234	9
mmu-miR-7005-5p	NM_153178.4	146.00	-22.12	2 21	6015 6035	19
mmu-miR-7005-5p	XM_011250176.1	149.00	-24.13	2 18	4053 4074	16
mmu-miR-7005-5p	XM_011250176.1	146.00	-24.72	2 20	6834 6856	19
mmu-miR-7005-5p	NM_031185.3	143.00	-18.25	2 13	1793 1815	12
mmu-miR-7005-5p	NM_031185.3	141.00	-17.56	2 10	5109 5130	8
mmu-miR-7005-5p	XM_011240149.1	147.00	-28.57	3 21	983 1005	19
mmu-miR-7005-5p	NM_008012.1	148.00	-22.78	2 18	694 717	18
mmu-miR-7005-5p	NM_053080.3	148.00	-28.85	2 17	792 813	15
mmu-miR-7005-5p	NM_053080.3	144.00	-17.72	2 17	2490 2511	15
mmu-miR-7005-5p	XM_006532024.3	151.00	-28.75	2 20	1172 1193	18
mmu-miR-7005-5p	NM_001303431.1	153.00	-22.45	2 20	1267 1291	21
mmu-miR-7005-5p	NM_001303431.1	152.00	-21.80	2 13	1106 1127	11
mmu-miR-7005-5p	NM_001303431.1	151.00	-25.71	2 21	139 163	22
mmu-miR-7005-5p	NM_001303431.1	140.00	-16.46	2 9	949 970	7
mmu-miR-7005-5p	XM_006508990.2	150.00	-25.25	2 19	3078 3099	17
mmu-miR-7005-5p	XM_006508990.2	149.00	-31.92	2 20	3227 3247	18
mmu-miR-7005-5p	NM_172790.2	147.00	-29.63	2 21	11 35	22
mmu-miR-7005-5p	NM_172790.2	143.00	-25.11	2 12	5465 5486	10
mmu-miR-7005-5p	NM_172790.2	142.00	-22.43	2 19	1048 1069	17
mmu-miR-7005-5p	NM_172790.2	141.00	-29.82	2 21	4299 4320	20
mmu-miR-7005-5p	NM_009675.2	161.00	-31.34	2 14	1271 1292	12
mmu-miR-7005-5p	NM_009675.2	159.00	-31.58	3 21	2779 2801	19
mmu-miR-7005-5p	NM_009675.2	147.00	-26.30	2 21	1229 1251	20
mmu-miR-7005-5p	NM_009675.2	142.00	-24.43	2 21	3111 3134	21
mmu-miR-7005-5p	NM_009675.2	141.00	-19.96	3 18	2669 2690	15
mmu-miR-7005-5p	NM_009675.2	141.00	-25.08	2 14	3861 3882	12
mmu-miR-7005-5p	NM_009675.2	140.00	-16.78	2 9	1446 1467	7
mmu-miR-7005-5p	NM_013912.3	156.00	-27.12	2 21	1241 1262	19
mmu-miR-7005-5p	NM_013912.3	146.00	-22.30	2 15	936 957	13
mmu-miR-7005-5p	NM_013912.3	140.00	-25.10	2 13	1700 1721	11
mmu-miR-7005-5p	NM_001301354.1	140.00	-20.04	2 21	10 31	19
mmu-miR-7005-5p	NM_001080819.1	162.00	-25.35	2 18	1425 1444	16
mmu-miR-7005-5p	NM_001080819.1	152.00	-28.74	2 21	664 685	19
mmu-miR-7005-5p	NM_001080819.1	152.00	-21.77	2 13	4050 4071	11
mmu-miR-7005-5p	NM_001080819.1	151.00	-23.98	3 21	6994 7016	19
mmu-miR-7005-5p	NM_001080819.1	150.00	-18.91	2 11	1621 1642	9
mmu-miR-7005-5p	NM_001080819.1	147.00	-25.57	2 21	1542 1564	20
mmu-miR-7005-5p	NM_001080819.1	147.00	-25.74	2 21	1824 1847	21
mmu-miR-7005-5p	NM_001080819.1	143.00	-21.69	2 16	1765 1786	14
mmu-miR-7005-5p	NM_001080819.1	143.00	-25.85	2 21	7725 7743	19
mmu-miR-7005-5p	NM_001080819.1	142.00	-27.19	3 21	2301 2324	20
mmu-miR-7005-5p	NM_001080819.1	142.00	-24.41	2 20	3726 3749	20
mmu-miR-7005-5p	NM_001080819.1	140.00	-19.07	2 9	847 868	7
mmu-miR-7005-5p	NM_029823.2	142.00	-29.67	2 20	252 274	19
mmu-miR-7005-5p	NM_007881.4	160.00	-30.03	2 13	2214 2235	11
mmu-miR-7005-5p	NM_007881.4	159.00	-27.48	2 17	4371 4394	17
mmu-miR-7005-5p	NM_007881.4	144.00	-27.51	2 17	4104 4125	15
mmu-miR-7005-5p	NM_007881.4	142.00	-32.66	2 20	114 136	19

mmu-miR-7005-5p	NM_007881.4	142.00	-24.33	3 21	1398 1418	18
mmu-miR-7005-5p	NM_001290469.1	146.00	-27.95	3 19	3305 3326	16
mmu-miR-7005-5p	NM_013415.5	157.00	-28.08	2 21	2390 2413	22
mmu-miR-7005-5p	NM_013415.5	143.00	-22.20	2 21	2851 2873	20
mmu-miR-7005-5p	NM_013415.5	140.00	-26.87	2 21	59 80	19
mmu-miR-7005-5p	XM_006507725.2	158.00	-24.85	2 19	2550 2571	17
mmu-miR-7005-5p	XM_006507725.2	155.00	-28.51	2 12	2922 2943	10
mmu-miR-7005-5p	XM_006507725.2	155.00	-26.87	2 20	2949 2970	18
mmu-miR-7005-5p	XM_006507725.2	151.00	-19.85	2 21	2207 2230	21
mmu-miR-7005-5p	XM_006507725.2	149.00	-20.98	2 14	1350 1371	12
mmu-miR-7005-5p	XM_006507725.2	145.00	-29.19	2 18	474 495	16
mmu-miR-7005-5p	XM_006507725.2	144.00	-28.70	3 18	3662 3686	18
mmu-miR-7005-5p	XM_006507725.2	143.00	-23.40	3 16	3279 3300	13
mmu-miR-7005-5p	XM_006507725.2	142.00	-23.40	2 16	173 195	15
mmu-miR-7005-5p	NM_013863.5	147.00	-25.26	2 20	882 903	18
mmu-miR-7005-5p	NM_007529.2	152.00	-21.37	2 21	2140 2161	19
mmu-miR-7005-5p	NM_007529.2	146.00	-18.88	2 15	1279 1300	13
mmu-miR-7005-5p	XM_006502291.3	145.00	-20.17	2 18	3682 3703	16
mmu-miR-7005-5p	XM_006502291.3	144.00	-22.94	2 21	3551 3572	19
mmu-miR-7005-5p	XM_006502291.3	141.00	-15.81	2 10	1843 1864	8
mmu-miR-7005-5p	XM_006502291.3	141.00	-23.24	2 14	3248 3269	12
mmu-miR-7005-5p	XM_006510701.3	152.00	-33.88	2 21	3024 3045	19
mmu-miR-7005-5p	XM_006510701.3	152.00	-20.33	2 18	4450 4472	17
mmu-miR-7005-5p	XM_006510701.3	146.00	-33.62	2 21	3039 3059	19
mmu-miR-7005-5p	XM_006510701.3	145.00	-22.55	2 19	198 220	18
mmu-miR-7005-5p	XM_006510701.3	143.00	-20.07	2 16	4468 4489	14
mmu-miR-7005-5p	XM_006510701.3	141.00	-27.91	2 18	4918 4939	16
mmu-miR-7005-5p	XM_006510701.3	140.00	-22.20	2 18	4162 4185	18
mmu-miR-7005-5p	NM_178782.4	141.00	-15.81	2 10	3298 3319	8
mmu-miR-7005-5p	NM_178782.4	141.00	-26.67	2 20	3439 3464	22
mmu-miR-7005-5p	NM_007549.2	145.00	-22.89	2 10	1095 1116	8
mmu-miR-7005-5p	NM_028472.2	159.00	-24.11	2 16	1175 1196	14
mmu-miR-7005-5p	NM_028472.2	153.00	-21.81	2 18	1737 1758	16
mmu-miR-7005-5p	NM_028472.2	151.00	-21.57	2 16	251 272	14
mmu-miR-7005-5p	NM_028472.2	147.00	-25.14	2 18	2598 2618	16
mmu-miR-7005-5p	NM_020508.4	153.00	-20.69	2 14	3512 3533	12
mmu-miR-7005-5p	NM_020508.4	140.00	-24.53	3 17	124 145	14
mmu-miR-7005-5p	XM_006524328.3	173.00	-34.01	2 18	5558 5579	16
mmu-miR-7005-5p	XM_006524328.3	164.00	-25.02	2 21	2826 2847	19
mmu-miR-7005-5p	XM_006524328.3	140.00	-25.18	3 13	666 687	10
mmu-miR-7005-5p	XM_006524328.3	140.00	-20.04	2 19	3493 3513	17
mmu-miR-7005-5p	NM_178692.3	142.00	-25.98	3 18	2411 2432	16
mmu-miR-7005-5p	NM_026979.5	150.00	-30.14	2 19	1114 1135	17
mmu-miR-7005-5p	NM_026979.5	144.00	-24.85	2 13	104 125	11
mmu-miR-7005-5p	XM_006503665.3	150.00	-22.60	2 21	1827 1847	19
mmu-miR-7005-5p	NM_001097617.1	153.00	-30.24	2 21	311 334	22
mmu-miR-7005-5p	NM_001285867.1	155.00	-27.71	2 18	1537 1560	18
mmu-miR-7005-5p	NM_001285867.1	154.00	-30.07	2 21	1633 1653	19
mmu-miR-7005-5p	NM_001285867.1	153.00	-30.42	2 19	1507 1530	19
mmu-miR-7005-5p	NM_001285867.1	151.00	-24.07	2 12	4201 4222	10
mmu-miR-7005-5p	NM_001285867.1	150.00	-24.65	2 11	41 62	9
mmu-miR-7005-5p	NM_001285867.1	150.00	-18.67	2 15	154 175	13
mmu-miR-7005-5p	NM_001285867.1	148.00	-26.56	2 20	2806 2825	18
mmu-miR-7005-5p	NM_001285867.1	143.00	-26.59	2 21	3619 3643	22
mmu-miR-7005-5p	XM_011246128.2	152.00	-21.55	2 17	925 946	15
mmu-miR-7005-5p	NM_007588.2	150.00	-21.31	2 17	3235 3255	15
mmu-miR-7005-5p	NM_001190379.1	152.00	-25.90	3 21	4255 4276	18
mmu-miR-7005-5p	NM_001190379.1	148.00	-23.16	2 18	1718 1741	18
mmu-miR-7005-5p	NM_001190379.1	147.00	-28.00	2 17	1092 1114	16
mmu-miR-7005-5p	NM_001190379.1	145.00	-19.58	2 14	3185 3206	12
mmu-miR-7005-5p	NM_001190379.1	144.00	-17.02	2 13	1819 1840	11
mmu-miR-7005-5p	XM_011246479.2	150.00	-25.54	2 21	3150 3173	21
mmu-miR-7005-5p	XM_011246479.2	143.00	-35.36	2 16	1462 1483	14
mmu-miR-7005-5p	XM_006520186.2	142.00	-24.77	2 15	75 96	13
mmu-miR-7005-5p	XM_006501443.3	143.00	-21.50	3 21	36 58	19

mmu-miR-7005-5p	NM_181315.4	149.00	-19.97	2 21	3131 3150	19
mmu-miR-7005-5p	XM_006520253.3	160.00	-25.63	2 18	4329 4352	18
mmu-miR-7005-5p	XM_006520253.3	148.00	-26.88	2 21	2035 2054	19
mmu-miR-7005-5p	XM_006500947.3	144.00	-21.54	2 17	1573 1594	15
mmu-miR-7005-5p	NM_009824.2	153.00	-26.54	3 20	5381 5401	17
mmu-miR-7005-5p	NM_009824.2	143.00	-22.68	3 21	456 478	19
mmu-miR-7005-5p	NM_009824.2	140.00	-19.89	2 17	95 116	15
mmu-miR-7005-5p	NM_009824.2	140.00	-31.17	2 21	163 184	19
mmu-miR-7005-5p	XM_006526449.3	140.00	-25.40	2 21	1653 1674	19
mmu-miR-7005-5p	NM_001310648.1	152.00	-25.64	2 17	1574 1595	15
mmu-miR-7005-5p	NM_001001178.1	158.00	-26.39	2 15	3025 3046	13
mmu-miR-7005-5p	NM_001001178.1	150.00	-26.33	2 20	124 146	19
mmu-miR-7005-5p	NM_001001178.1	144.00	-27.23	2 21	935 956	19
mmu-miR-7005-5p	NM_001013784.1	167.00	-31.44	2 21	3437 3459	20
mmu-miR-7005-5p	NM_001013784.1	160.00	-32.98	2 13	2952 2973	11
mmu-miR-7005-5p	XM_006508867.2	142.00	-23.45	2 21	445 465	19
mmu-miR-7005-5p	XM_006508867.2	141.00	-28.10	2 21	230 249	19
mmu-miR-7005-5p	XM_006508885.1	165.00	-29.10	2 14	1141 1162	12
mmu-miR-7005-5p	XM_006508885.1	142.00	-23.45	2 21	529 549	19
mmu-miR-7005-5p	XM_006508885.1	141.00	-28.10	2 21	314 333	19
mmu-miR-7005-5p	XM_006533653.3	141.00	-22.76	2 18	537 558	16
mmu-miR-7005-5p	XM_006509966.3	142.00	-28.30	3 20	1431 1453	18
mmu-miR-7005-5p	NM_007655.3	149.00	-27.74	2 21	742 764	21
mmu-miR-7005-5p	NM_001313939.1	154.00	-21.86	2 21	842 862	19
mmu-miR-7005-5p	XM_006531321.3	156.00	-25.73	2 20	669 688	18
mmu-miR-7005-5p	XM_006523030.3	140.00	-15.49	2 9	3517 3538	7
mmu-miR-7005-5p	NM_001301295.1	150.00	-32.81	2 21	1099 1124	23
mmu-miR-7005-5p	XM_006513165.1	166.00	-29.10	2 20	1246 1268	19
mmu-miR-7005-5p	XM_006513165.1	143.00	-22.86	2 21	790 813	21
mmu-miR-7005-5p	XM_006530131.1	143.00	-22.21	2 12	6353 6374	10
mmu-miR-7005-5p	XM_006530131.1	141.00	-24.57	2 10	6317 6338	8
mmu-miR-7005-5p	XM_011250217.1	148.00	-24.13	2 17	1426 1447	15
mmu-miR-7005-5p	XM_011250217.1	144.00	-34.56	2 21	452 473	19
mmu-miR-7005-5p	XM_011250217.1	141.00	-22.01	2 19	1301 1323	18
mmu-miR-7005-5p	XM_006527480.3	143.00	-24.92	2 21	2544 2566	20
mmu-miR-7005-5p	XM_006527480.3	140.00	-19.28	2 9	2314 2335	7
mmu-miR-7005-5p	NM_001004357.2	148.00	-22.62	3 21	6373 6394	18
mmu-miR-7005-5p	NM_001004357.2	146.00	-20.91	2 15	2707 2728	13
mmu-miR-7005-5p	NM_001113515.2	174.00	-40.53	2 20	2928 2950	19
mmu-miR-7005-5p	NM_001113515.2	159.00	-33.00	2 21	1785 1807	20
mmu-miR-7005-5p	NM_001113515.2	145.00	-21.98	2 20	230 256	23
mmu-miR-7005-5p	NM_001113515.2	143.00	-40.67	2 21	2028 2051	21
mmu-miR-7005-5p	NM_001113515.2	141.00	-26.86	2 19	840 863	19
mmu-miR-7005-5p	NM_001113515.2	140.00	-27.42	2 21	1947 1968	19
mmu-miR-7005-5p	NM_001113515.2	140.00	-35.95	2 21	3495 3516	19
mmu-miR-7005-5p	NM_009930.2	165.00	-36.61	2 19	3018 3041	19
mmu-miR-7005-5p	NM_009930.2	159.00	-37.27	2 21	2757 2779	20
mmu-miR-7005-5p	NM_009930.2	141.00	-27.10	3 19	3450 3472	17
mmu-miR-7005-5p	NM_009930.2	140.00	-27.09	2 9	1553 1574	7
mmu-miR-7005-5p	XM_011240755.1	161.00	-30.83	2 20	138 161	20
mmu-miR-7005-5p	XM_011240755.1	143.00	-21.40	2 20	1376 1397	18
mmu-miR-7005-5p	XM_006532938.2	146.00	-17.75	2 11	2297 2318	9
mmu-miR-7005-5p	XM_006532938.2	142.00	-29.71	3 20	2665 2687	18
mmu-miR-7005-5p	XM_011247208.1	142.00	-25.70	2 11	6348 6369	9
mmu-miR-7005-5p	XM_011247208.1	140.00	-15.90	2 17	4134 4155	15
mmu-miR-7005-5p	NM_011957.2	152.00	-24.48	2 19	1742 1765	19
mmu-miR-7005-5p	NM_011957.2	146.00	-27.11	3 19	881 902	16
mmu-miR-7005-5p	XM_011242311.2	163.00	-25.78	2 21	760 782	20
mmu-miR-7005-5p	XM_011242311.2	160.00	-21.40	2 17	1393 1414	15
mmu-miR-7005-5p	XM_011242311.2	155.00	-25.12	2 12	2515 2536	10
mmu-miR-7005-5p	XM_011242311.2	145.00	-22.09	3 18	784 805	15
mmu-miR-7005-5p	XM_011242311.2	143.00	-22.54	3 16	905 926	13
mmu-miR-7005-5p	NM_001289782.1	143.00	-26.15	3 21	488 510	19
mmu-miR-7005-5p	NM_001198841.1	140.00	-18.22	3 21	349 370	18
mmu-miR-7005-5p	NM_018866.2	142.00	-27.56	2 21	64 88	22

mmu-miR-7005-5p	XM_006517308.3	154.00	-25.03	2 21	1375 1399	22
mmu-miR-7005-5p	NM_030206.4	147.00	-14.93	2 16	159 180	14
mmu-miR-7005-5p	NM_030206.4	147.00	-27.12	3 16	1828 1849	13
mmu-miR-7005-5p	NM_030206.4	141.00	-17.91	2 10	605 626	8
mmu-miR-7005-5p	NM_007817.2	143.00	-30.07	2 21	845 868	21
mmu-miR-7005-5p	NM_001190448.1	142.00	-22.87	2 11	638 659	9
mmu-miR-7005-5p	NM_015814.2	147.00	-21.81	2 21	581 604	21
mmu-miR-7005-5p	NM_010055.3	159.00	-25.53	2 16	2505 2526	14
mmu-miR-7005-5p	NM_010055.3	142.00	-21.65	2 21	1580 1603	21
mmu-miR-7005-5p	NM_027293.1	151.00	-28.81	3 21	3608 3631	20
mmu-miR-7005-5p	NM_027293.1	147.00	-22.38	2 14	6420 6440	12
mmu-miR-7005-5p	NM_027293.1	142.00	-34.07	2 21	600 623	21
mmu-miR-7005-5p	XM_011243391.2	148.00	-24.03	2 21	187 208	19
mmu-miR-7005-5p	XM_006506808.3	159.00	-31.75	2 16	2213 2234	14
mmu-miR-7005-5p	NM_007883.3	150.00	-24.38	2 15	4823 4844	13
mmu-miR-7005-5p	NM_007883.3	143.00	-19.77	2 16	4768 4789	14
mmu-miR-7005-5p	XM_006514873.2	152.00	-20.81	2 13	2588 2609	11
mmu-miR-7005-5p	NM_001160049.1	141.00	-19.32	2 10	3213 3234	8
mmu-miR-7005-5p	NM_133643.4	145.00	-17.14	2 14	4144 4165	12
mmu-miR-7005-5p	NM_133643.4	141.00	-19.85	2 10	1839 1860	8
mmu-miR-7005-5p	NM_019397.3	147.00	-20.06	2 21	23 45	20
mmu-miR-7005-5p	NM_001291145.1	142.00	-24.46	2 19	3025 3043	17
mmu-miR-7005-5p	XM_006520618.2	146.00	-19.33	2 20	84 106	19
mmu-miR-7005-5p	NM_015744.4	148.00	-20.24	2 13	2233 2254	11
mmu-miR-7005-5p	NM_178676.4	141.00	-21.88	2 20	3322 3347	22
mmu-miR-7005-5p	NM_010137.3	154.00	-23.98	2 16	2761 2784	16
mmu-miR-7005-5p	NM_010137.3	146.00	-22.31	2 15	616 637	13
mmu-miR-7005-5p	NM_010137.3	144.00	-33.69	2 21	3397 3416	19
mmu-miR-7005-5p	NM_010137.3	142.00	-27.57	2 15	155 176	13
mmu-miR-7005-5p	XM_011238916.2	154.00	-21.16	2 20	2306 2328	19
mmu-miR-7005-5p	XM_011238916.2	145.00	-17.13	2 10	608 629	8
mmu-miR-7005-5p	XM_011238916.2	144.00	-22.54	3 18	2137 2159	16
mmu-miR-7005-5p	XM_006511221.3	155.00	-23.09	2 18	4021 4041	16
mmu-miR-7005-5p	XM_006511221.3	146.00	-28.31	2 21	3181 3201	19
mmu-miR-7009-5p	NM_025865.2	141.00	-24.23	3 18	659 680	15
mmu-miR-7009-5p	NM_001033630.1	140.00	-21.33	2 9	167 188	7
mmu-miR-7009-5p	NM_001033630.1	140.00	-21.92	2 13	215 236	11
mmu-miR-7009-5p	XM_006515980.3	140.00	-18.43	2 9	1920 1941	7
mmu-miR-7009-5p	XM_006540566.1	155.00	-29.75	2 21	2956 2978	20
mmu-miR-7009-5p	XM_006540566.1	151.00	-19.64	2 21	562 584	20
mmu-miR-7009-5p	XM_006540566.1	151.00	-25.42	2 21	2899 2921	20
mmu-miR-7009-5p	XM_006540566.1	147.00	-18.52	2 20	6833 6854	18
mmu-miR-7009-5p	XM_006540566.1	146.00	-26.20	2 20	2851 2873	19
mmu-miR-7009-5p	XM_006540566.1	144.00	-28.88	2 21	607 628	19
mmu-miR-7009-5p	XM_006540566.1	143.00	-30.15	2 16	3039 3060	14
mmu-miR-7009-5p	NM_153151.3	141.00	-28.88	2 18	930 951	16
mmu-miR-7009-5p	NM_172580.1	142.00	-17.76	2 11	1344 1365	9
mmu-miR-7009-5p	NM_001142804.1	144.00	-20.94	2 21	583 604	19
mmu-miR-7009-5p	NM_001142804.1	141.00	-27.82	2 21	268 287	19
mmu-miR-7009-5p	NM_001142804.1	140.00	-22.52	2 9	2066 2087	7
mmu-miR-7009-5p	NM_009608.4	149.00	-28.04	2 19	1 21	18
mmu-miR-7009-5p	NM_033268.4	155.00	-26.71	2 21	2626 2643	19
mmu-miR-7009-5p	NM_033268.4	145.00	-19.52	2 10	837 858	8
mmu-miR-7009-5p	XM_006498086.3	157.00	-26.31	2 19	206 228	18
mmu-miR-7009-5p	XM_006498086.3	146.00	-22.58	2 19	5881 5902	17
mmu-miR-7009-5p	NM_021475.2	153.00	-31.87	2 21	1246 1267	20
mmu-miR-7009-5p	NM_013906.3	155.00	-20.88	2 18	272 292	16
mmu-miR-7009-5p	NM_013906.3	140.00	-25.29	3 19	4364 4388	19
mmu-miR-7009-5p	XM_006505388.2	156.00	-21.37	2 21	4969 4990	19
mmu-miR-7009-5p	XM_006505388.2	152.00	-32.61	2 21	2953 2974	19
mmu-miR-7009-5p	XM_006505388.2	147.00	-27.88	2 21	3086 3109	21
mmu-miR-7009-5p	XM_006505388.2	141.00	-27.33	3 19	928 950	17
mmu-miR-7009-5p	NM_145635.2	146.00	-22.86	2 21	149 169	19
mmu-miR-7009-5p	NM_001291930.1	157.00	-30.92	2 18	1410 1431	16
mmu-miR-7009-5p	NM_001291930.1	144.00	-30.71	2 21	3954 3975	19

mmu-miR-7009-5p	NM_001291930.1	140.00	-17.64	2 9	3735 3756	7
mmu-miR-7009-5p	XM_017312667.1	145.00	-20.18	2 10	7060 7081	8
mmu-miR-7009-5p	XM_017312667.1	144.00	-25.60	2 21	1883 1904	19
mmu-miR-7009-5p	XM_017312667.1	143.00	-19.50	2 16	3219 3240	14
mmu-miR-7009-5p	XM_017312667.1	142.00	-22.40	2 16	3420 3442	15
mmu-miR-7009-5p	XM_017312667.1	141.00	-20.53	2 18	2317 2338	16
mmu-miR-7009-5p	XM_017312667.1	140.00	-13.86	2 9	4243 4264	7
mmu-miR-7009-5p	NM_008032.3	143.00	-22.65	2 20	1548 1569	18
mmu-miR-7009-5p	NM_008032.3	140.00	-23.74	2 9	128 149	7
mmu-miR-7009-5p	NM_153178.4	140.00	-20.01	2 9	2933 2954	7
mmu-miR-7009-5p	XM_011250176.1	160.00	-22.46	2 18	3681 3703	17
mmu-miR-7009-5p	XM_011250176.1	152.00	-21.34	2 21	1479 1500	19
mmu-miR-7009-5p	XM_011250176.1	150.00	-23.64	2 11	3523 3544	9
mmu-miR-7009-5p	XM_011250176.1	148.00	-29.17	3 18	1041 1063	16
mmu-miR-7009-5p	XM_011250176.1	144.00	-28.50	3 17	826 847	14
mmu-miR-7009-5p	XM_006498287.3	150.00	-17.39	2 15	1931 1952	13
mmu-miR-7009-5p	XM_006498287.3	140.00	-17.91	2 9	462 483	7
mmu-miR-7009-5p	NM_008012.1	140.00	-16.01	2 9	744 765	7
mmu-miR-7009-5p	NM_053080.3	151.00	-20.93	2 16	1945 1966	14
mmu-miR-7009-5p	XM_006495613.3	157.00	-23.63	2 19	129 151	18
mmu-miR-7009-5p	XM_006508990.2	147.00	-19.47	2 12	2293 2314	10
mmu-miR-7009-5p	XM_006508990.2	145.00	-22.48	2 10	971 992	8
mmu-miR-7009-5p	XM_017318264.1	149.00	-23.06	2 19	70 94	20
mmu-miR-7009-5p	NM_172790.2	144.00	-30.52	2 19	3591 3617	22
mmu-miR-7009-5p	NM_172790.2	140.00	-17.87	2 9	1456 1477	7
mmu-miR-7009-5p	NM_009675.2	147.00	-21.38	2 21	2480 2502	20
mmu-miR-7009-5p	NM_009675.2	146.00	-29.37	2 20	2777 2800	20
mmu-miR-7009-5p	NM_009675.2	140.00	-23.61	2 9	268 289	7
mmu-miR-7009-5p	NM_001301354.1	155.00	-22.42	2 12	1014 1035	10
mmu-miR-7009-5p	NM_001301354.1	151.00	-35.27	2 21	894 916	20
mmu-miR-7009-5p	NM_001080819.1	161.00	-26.29	2 19	7049 7073	20
mmu-miR-7009-5p	NM_001080819.1	151.00	-24.69	2 20	1267 1288	18
mmu-miR-7009-5p	NM_001080819.1	145.00	-18.13	2 10	3575 3596	8
mmu-miR-7009-5p	NM_001080819.1	144.00	-25.65	2 21	6299 6320	19
mmu-miR-7009-5p	NM_001080819.1	142.00	-22.37	2 15	3604 3625	13
mmu-miR-7009-5p	NM_001080819.1	140.00	-13.81	2 9	5872 5893	7
mmu-miR-7009-5p	NM_023048.5	140.00	-17.11	2 9	643 664	7
mmu-miR-7009-5p	NM_007881.4	153.00	-21.46	2 14	3920 3941	12
mmu-miR-7009-5p	NM_007881.4	140.00	-15.16	2 9	2285 2306	7
mmu-miR-7009-5p	NM_007881.4	140.00	-29.28	2 21	3530 3551	19
mmu-miR-7009-5p	NM_009722.3	145.00	-27.77	3 21	1114 1133	18
mmu-miR-7009-5p	XM_006529727.2	154.00	-25.95	2 21	1613 1633	19
mmu-miR-7009-5p	XM_006529727.2	147.00	-29.65	2 21	5245 5268	21
mmu-miR-7009-5p	XM_006529727.2	143.00	-23.96	2 21	7471 7498	25
mmu-miR-7009-5p	XM_006529727.2	141.00	-22.70	2 14	4955 4976	12
mmu-miR-7009-5p	XM_006507725.2	141.00	-27.96	3 14	1932 1953	11
mmu-miR-7009-5p	NM_013863.5	151.00	-30.03	3 21	2208 2231	20
mmu-miR-7009-5p	NM_013863.5	142.00	-33.55	2 21	245 270	23
mmu-miR-7009-5p	NM_007529.2	145.00	-31.70	2 21	304 323	19
mmu-miR-7009-5p	XM_006502291.3	155.00	-28.71	2 21	3338 3361	21
mmu-miR-7009-5p	XM_006502291.3	142.00	-31.99	2 21	377 400	21
mmu-miR-7009-5p	XM_006502291.3	142.00	-23.35	2 15	5722 5743	13
mmu-miR-7009-5p	XM_006502291.3	140.00	-17.70	2 9	4809 4830	7
mmu-miR-7009-5p	XM_006510701.3	162.00	-23.78	2 15	4457 4478	13
mmu-miR-7009-5p	XM_006510701.3	153.00	-27.40	2 20	814 834	18
mmu-miR-7009-5p	XM_006510701.3	145.00	-20.13	2 10	1801 1822	8
mmu-miR-7009-5p	XM_006510701.3	143.00	-31.36	2 21	5612 5634	20
mmu-miR-7009-5p	XM_006503323.3	142.00	-27.24	2 21	597 621	22
mmu-miR-7009-5p	XM_006503323.3	140.00	-28.87	3 21	874 895	18
mmu-miR-7009-5p	NM_020508.4	145.00	-20.30	2 14	1155 1176	12
mmu-miR-7009-5p	NM_020508.4	142.00	-24.29	2 21	943 966	21
mmu-miR-7009-5p	XM_006524328.3	142.00	-22.60	2 21	3797 3817	19
mmu-miR-7009-5p	NM_026979.5	140.00	-20.97	2 9	992 1013	7
mmu-miR-7009-5p	XM_006503665.3	167.00	-31.89	2 18	425 448	18
mmu-miR-7009-5p	NM_001285867.1	149.00	-24.08	2 14	421 442	12

mmu-miR-7009-5p	NM_001285867.1	145.00	-19.87	2 21	1739 1758	19
mmu-miR-7009-5p	NM_001285867.1	140.00	-21.82	2 13	1964 1985	11
mmu-miR-7009-5p	NM_009785.1	140.00	-22.50	3 18	2846 2868	16
mmu-miR-7009-5p	NM_001190379.1	156.00	-26.16	2 21	56 77	19
mmu-miR-7009-5p	NM_001190379.1	147.00	-22.07	2 21	1723 1747	22
mmu-miR-7009-5p	NM_001190379.1	142.00	-31.54	2 21	3986 4006	19
mmu-miR-7009-5p	XM_011246479.2	144.00	-41.46	4 21	3191 3212	17
mmu-miR-7009-5p	XM_011246479.2	143.00	-23.27	2 21	823 845	20
mmu-miR-7009-5p	XM_011246479.2	140.00	-20.27	2 9	2223 2244	7
mmu-miR-7009-5p	XM_006520186.2	143.00	-19.59	2 16	597 618	14
mmu-miR-7009-5p	XM_006501443.3	150.00	-35.53	2 19	711 733	18
mmu-miR-7009-5p	XM_006501443.3	147.00	-24.55	2 21	1936 1959	21
mmu-miR-7009-5p	NM_009824.2	164.00	-23.79	2 21	101 122	19
mmu-miR-7009-5p	NM_009824.2	161.00	-25.93	2 18	5194 5215	16
mmu-miR-7009-5p	NM_009824.2	145.00	-26.05	2 14	2116 2137	12
mmu-miR-7009-5p	NM_009824.2	140.00	-24.86	2 21	252 273	19
mmu-miR-7009-5p	NM_001001178.1	154.00	-27.68	2 21	2903 2928	23
mmu-miR-7009-5p	NM_028804.1	140.00	-17.29	2 21	1158 1179	19
mmu-miR-7009-5p	XM_011245344.2	145.00	-20.53	2 20	9154 9178	21
mmu-miR-7009-5p	XM_011245344.2	140.00	-16.56	2 17	10841 10862	15
mmu-miR-7009-5p	XM_017312731.1	140.00	-22.40	2 9	1000 1021	7
mmu-miR-7009-5p	XM_006531321.3	144.00	-17.38	2 13	1210 1231	11
mmu-miR-7009-5p	XM_006530131.1	158.00	-28.10	2 21	1059 1085	24
mmu-miR-7009-5p	XM_006530131.1	142.00	-37.90	2 21	6545 6575	28
mmu-miR-7009-5p	XM_006530131.1	140.00	-18.94	2 9	985 1006	7
mmu-miR-7009-5p	XM_006530131.1	140.00	-16.14	2 9	6412 6433	7
mmu-miR-7009-5p	XM_011250217.1	140.00	-21.69	2 13	1176 1197	11
mmu-miR-7009-5p	XM_011250217.1	140.00	-26.87	2 21	1279 1300	19
mmu-miR-7009-5p	XM_006527480.3	141.00	-23.51	2 14	2613 2634	12
mmu-miR-7009-5p	NM_001004357.2	140.00	-19.01	2 9	4161 4182	7
mmu-miR-7009-5p	NM_001113515.2	165.00	-39.98	2 19	1360 1382	18
mmu-miR-7009-5p	NM_001113515.2	158.00	-27.87	2 17	3714 3734	15
mmu-miR-7009-5p	NM_001113515.2	153.00	-34.14	2 20	1826 1850	21
mmu-miR-7009-5p	NM_001113515.2	148.00	-30.28	2 18	3556 3578	17
mmu-miR-7009-5p	NM_001113515.2	146.00	-34.58	2 21	3492 3515	21
mmu-miR-7009-5p	NM_001113515.2	144.00	-27.97	2 19	1441 1464	19
mmu-miR-7009-5p	NM_001113515.2	143.00	-26.83	2 21	1945 1967	20
mmu-miR-7009-5p	NM_001113515.2	141.00	-33.04	2 19	721 743	18
mmu-miR-7009-5p	NM_001113515.2	141.00	-26.59	3 19	2386 2409	18
mmu-miR-7009-5p	NM_009930.2	167.00	-33.31	2 18	3178 3201	18
mmu-miR-7009-5p	NM_009930.2	146.00	-28.86	2 19	1082 1103	17
mmu-miR-7009-5p	NM_009930.2	140.00	-26.88	2 18	514 536	17
mmu-miR-7009-5p	NM_007739.2	147.00	-29.02	2 17	1484 1506	16
mmu-miR-7009-5p	NM_007739.2	143.00	-26.85	2 21	1754 1776	20
mmu-miR-7009-5p	NM_007739.2	141.00	-30.21	3 19	1886 1909	18
mmu-miR-7009-5p	XM_011240755.1	141.00	-25.54	2 20	2415 2439	21
mmu-miR-7009-5p	XM_006532938.2	159.00	-26.87	2 17	2823 2845	16
mmu-miR-7009-5p	XM_006532938.2	144.00	-26.38	2 21	1311 1332	19
mmu-miR-7009-5p	XM_006532938.2	142.00	-17.19	2 11	2457 2478	9
mmu-miR-7009-5p	XM_011247208.1	159.00	-30.34	2 21	1831 1853	20
mmu-miR-7009-5p	XM_011248602.2	145.00	-23.08	2 19	2010 2033	19
mmu-miR-7009-5p	XM_011248602.2	140.00	-17.49	2 21	2647 2668	19
mmu-miR-7009-5p	NM_019696.2	140.00	-16.71	2 9	784 805	7
mmu-miR-7009-5p	XM_011242311.2	161.00	-26.29	2 19	300 322	18
mmu-miR-7009-5p	XM_011242311.2	151.00	-26.74	2 21	5514 5536	20
mmu-miR-7009-5p	XM_011242311.2	142.00	-26.21	2 19	881 902	17
mmu-miR-7009-5p	XM_011242311.2	141.00	-25.23	3 20	2916 2936	17
mmu-miR-7009-5p	XM_011242311.2	141.00	-28.31	2 19	4555 4577	18
mmu-miR-7009-5p	NM_001289782.1	149.00	-25.99	2 14	328 349	12
mmu-miR-7009-5p	NM_007817.2	160.00	-28.86	2 19	834 857	19
mmu-miR-7009-5p	NM_007817.2	143.00	-24.98	2 16	563 584	14
mmu-miR-7009-5p	NM_015814.2	160.00	-28.39	2 21	831 849	19
mmu-miR-7009-5p	NM_015814.2	143.00	-25.52	2 21	2675 2700	23
mmu-miR-7009-5p	NM_010055.3	147.00	-32.42	2 21	1872 1895	21
mmu-miR-7009-5p	NM_010055.3	142.00	-19.12	2 15	967 988	13

mmu-miR-7009-5p	NM_010055.3	141.00	-20.15	2 19	931 948	17
mmu-miR-7009-5p	NM_152915.1	143.00	-27.70	2 21	558 580	20
mmu-miR-7009-5p	NM_009345.2	154.00	-29.97	2 20	1830 1853	20
mmu-miR-7009-5p	NM_027293.1	140.00	-23.11	2 19	6100 6120	17
mmu-miR-7009-5p	XM_011243391.2	150.00	-28.40	2 15	3214 3235	13
mmu-miR-7009-5p	XM_011243391.2	150.00	-29.66	2 21	4719 4739	19
mmu-miR-7009-5p	XM_011243391.2	145.00	-25.21	2 21	3852 3873	20
mmu-miR-7009-5p	XM_011243391.2	140.00	-23.67	3 18	45 68	17
mmu-miR-7009-5p	XM_006514873.2	152.00	-24.18	2 21	2795 2816	19
mmu-miR-7009-5p	XM_006514873.2	147.00	-25.74	2 21	2036 2059	21
mmu-miR-7009-5p	XM_006528026.2	149.00	-17.14	2 14	4087 4108	12
mmu-miR-7009-5p	NM_133643.4	162.00	-27.43	2 19	6081 6102	17
mmu-miR-7009-5p	NM_133643.4	146.00	-27.56	2 20	1844 1866	19
mmu-miR-7009-5p	NM_133643.4	144.00	-19.54	2 13	552 573	11
mmu-miR-7009-5p	NM_001291145.1	148.00	-28.32	2 17	694 715	15
mmu-miR-7009-5p	NM_001291145.1	143.00	-13.62	2 16	2960 2981	14
mmu-miR-7009-5p	NM_001291145.1	142.00	-19.24	2 11	3102 3123	9
mmu-miR-7009-5p	XM_011238916.2	152.00	-25.00	2 21	5313 5334	19
mmu-miR-7009-5p	XM_006511221.3	140.00	-19.40	2 21	2751 2772	19
mmu-miR-7042-5p	NM_025865.2	154.00	-19.82	2 19	717 737	17
mmu-miR-7042-5p	XM_011241948.2	141.00	-19.48	2 17	64 82	15
mmu-miR-7042-5p	XM_006515980.3	147.00	-22.49	2 18	359 382	19
mmu-miR-7042-5p	NM_029982.1	147.00	-18.09	2 19	425 446	19
mmu-miR-7042-5p	XM_006540566.1	155.00	-20.97	2 18	5117 5136	16
mmu-miR-7042-5p	XM_006540566.1	143.00	-20.71	3 20	4232 4252	17
mmu-miR-7042-5p	NM_172580.1	148.00	-23.87	2 20	1257 1275	18
mmu-miR-7042-5p	NM_172580.1	143.00	-18.94	3 20	995 1015	17
mmu-miR-7042-5p	NM_009608.4	157.00	-22.70	2 19	1265 1287	19
mmu-miR-7042-5p	NM_009608.4	155.00	-23.42	2 12	555 575	10
mmu-miR-7042-5p	NM_021475.2	164.00	-25.03	2 20	2181 2199	18
mmu-miR-7042-5p	XM_006538374.1	140.00	-16.29	2 9	4874 4894	7
mmu-miR-7042-5p	XM_006500919.3	149.00	-22.50	2 18	1066 1086	16
mmu-miR-7042-5p	NM_001291930.1	167.00	-26.23	2 19	1312 1332	18
mmu-miR-7042-5p	NM_001291930.1	147.00	-20.11	2 20	1468 1488	18
mmu-miR-7042-5p	XM_017312667.1	145.00	-16.96	2 10	5545 5565	8
mmu-miR-7042-5p	XM_017312667.1	141.00	-9.27	2 16	5621 5640	14
mmu-miR-7042-5p	NM_008032.3	145.00	-17.74	2 19	3983 4005	19
mmu-miR-7042-5p	NM_153178.4	148.00	-16.45	2 14	5618 5639	13
mmu-miR-7042-5p	NM_153178.4	145.00	-15.70	2 20	3279 3298	18
mmu-miR-7042-5p	NM_153178.4	145.00	-22.68	2 20	4610 4633	21
mmu-miR-7042-5p	XM_011250176.1	157.00	-23.82	2 18	5424 5444	16
mmu-miR-7042-5p	XM_011250176.1	144.00	-20.56	3 17	3102 3122	14
mmu-miR-7042-5p	XM_011240149.1	143.00	-17.85	3 20	595 615	17
mmu-miR-7042-5p	NM_134072.1	140.00	-16.56	2 9	1890 1910	7
mmu-miR-7042-5p	XM_006495613.3	149.00	-22.94	2 19	977 999	19
mmu-miR-7042-5p	XM_006495613.3	147.00	-15.53	2 16	1829 1849	14
mmu-miR-7042-5p	XM_006508990.2	161.00	-16.62	2 18	6790 6810	16
mmu-miR-7042-5p	XM_006508990.2	140.00	-15.39	2 9	5398 5418	7
mmu-miR-7042-5p	NM_172790.2	162.00	-25.97	2 19	4327 4347	17
mmu-miR-7042-5p	NM_172790.2	152.00	-24.33	2 13	926 946	11
mmu-miR-7042-5p	NM_009675.2	159.00	-19.52	2 19	2826 2844	17
mmu-miR-7042-5p	NM_009675.2	144.00	-20.91	2 18	3688 3709	17
mmu-miR-7042-5p	NM_001301354.1	140.00	-20.00	2 17	687 707	15
mmu-miR-7042-5p	NM_001080819.1	147.00	-24.16	3 19	7862 7880	16
mmu-miR-7042-5p	NM_001080819.1	142.00	-13.85	2 19	3285 3305	17
mmu-miR-7042-5p	XM_006522398.3	141.00	-15.85	2 19	683 704	18
mmu-miR-7042-5p	NM_001310070.1	157.00	-26.09	2 20	719 741	20
mmu-miR-7042-5p	NM_007881.4	148.00	-17.36	2 14	1580 1601	13
mmu-miR-7042-5p	NM_001290469.1	167.00	-23.69	2 18	3444 3463	16
mmu-miR-7042-5p	NM_001290469.1	157.00	-23.34	2 14	2354 2374	12
mmu-miR-7042-5p	NM_013415.5	151.00	-18.51	2 14	2811 2830	12
mmu-miR-7042-5p	NM_013415.5	146.00	-19.17	2 20	31 52	19
mmu-miR-7042-5p	XM_006529727.2	156.00	-17.25	2 17	5734 5754	15
mmu-miR-7042-5p	XM_006529727.2	149.00	-20.07	2 14	3953 3973	12
mmu-miR-7042-5p	XM_006507725.2	143.00	-14.36	2 14	3627 3646	12

mmu-miR-7042-5p	NM_020025.4	141.00	-15.04	2 19	855 876	18
mmu-miR-7042-5p	NM_001159407.1	148.00	-16.04	2 15	1318 1337	13
mmu-miR-7042-5p	NM_013863.5	143.00	-21.49	2 20	1424 1444	18
mmu-miR-7042-5p	NM_007529.2	141.00	-16.56	2 12	618 637	10
mmu-miR-7042-5p	XM_006502291.3	141.00	-14.29	2 10	1283 1303	8
mmu-miR-7042-5p	XM_006502291.3	141.00	-12.84	3 18	3709 3729	15
mmu-miR-7042-5p	XM_006510701.3	141.00	-14.84	2 12	5663 5682	10
mmu-miR-7042-5p	NM_178782.4	142.00	-20.57	2 20	2815 2837	20
mmu-miR-7042-5p	NM_028472.2	148.00	-18.76	2 19	1412 1437	22
mmu-miR-7042-5p	NM_028472.2	140.00	-20.58	2 9	659 679	7
mmu-miR-7042-5p	NM_020508.4	142.00	-16.56	2 11	4550 4570	9
mmu-miR-7042-5p	XM_006524328.3	160.00	-21.30	2 20	4474 4494	19
mmu-miR-7042-5p	XM_006524328.3	154.00	-19.28	2 17	5666 5685	15
mmu-miR-7042-5p	NM_178692.3	144.00	-18.90	2 13	2463 2483	11
mmu-miR-7042-5p	XM_006503665.3	159.00	-16.18	2 16	1709 1729	14
mmu-miR-7042-5p	XM_006503665.3	146.00	-19.95	2 19	2675 2695	17
mmu-miR-7042-5p	NM_001285867.1	150.00	-18.70	2 19	4818 4838	17
mmu-miR-7042-5p	NM_001285867.1	140.00	-17.07	2 9	785 805	7
mmu-miR-7042-5p	NM_001285867.1	140.00	-14.34	2 15	4488 4507	13
mmu-miR-7042-5p	NM_009785.1	150.00	-24.76	2 11	3133 3153	9
mmu-miR-7042-5p	XM_011246128.2	156.00	-21.06	2 19	5157 5176	17
mmu-miR-7042-5p	XM_011246128.2	141.00	-14.54	2 12	1302 1321	10
mmu-miR-7042-5p	NM_001190379.1	152.00	-21.85	2 17	2064 2084	15
mmu-miR-7042-5p	NM_001190379.1	150.00	-18.85	2 16	877 898	15
mmu-miR-7042-5p	NM_181315.4	146.00	-16.45	2 15	1475 1495	13
mmu-miR-7042-5p	NM_181315.4	140.00	-17.50	2 14	2304 2325	13
mmu-miR-7042-5p	XM_006500947.3	152.00	-17.46	2 19	364 383	17
mmu-miR-7042-5p	XM_006500947.3	143.00	-24.97	2 20	247 267	18
mmu-miR-7042-5p	NM_009824.2	161.00	-21.94	2 20	2396 2418	20
mmu-miR-7042-5p	NM_009824.2	160.00	-20.53	2 20	4462 4480	18
mmu-miR-7042-5p	NM_009824.2	159.00	-25.35	2 20	501 521	18
mmu-miR-7042-5p	NM_009824.2	146.00	-16.96	2 20	6309 6331	20
mmu-miR-7042-5p	NM_019626.3	144.00	-22.47	3 19	1884 1907	19
mmu-miR-7042-5p	NM_001013784.1	140.00	-16.59	2 9	2330 2350	7
mmu-miR-7042-5p	XM_006509966.3	148.00	-20.26	2 18	1212 1234	18
mmu-miR-7042-5p	XM_011245344.2	151.00	-22.86	3 16	9315 9335	13
mmu-miR-7042-5p	XM_011245344.2	149.00	-19.19	2 15	13165 13187	15
mmu-miR-7042-5p	XM_011245344.2	148.00	-19.40	2 18	12287 12310	19
mmu-miR-7042-5p	XM_011245344.2	141.00	-15.97	2 19	5714 5735	18
mmu-miR-7042-5p	XM_011245344.2	140.00	-11.91	2 9	3847 3867	7
mmu-miR-7042-5p	XM_017312731.1	156.00	-23.03	2 18	77 98	17
mmu-miR-7042-5p	XM_006503371.2	140.00	-10.67	2 9	3278 3298	7
mmu-miR-7042-5p	XM_006523030.3	140.00	-18.92	3 18	2495 2516	16
mmu-miR-7042-5p	NM_001301295.1	142.00	-19.14	3 20	992 1014	19
mmu-miR-7042-5p	XM_006530131.1	160.00	-23.64	2 18	8315 8337	18
mmu-miR-7042-5p	XM_006530131.1	149.00	-13.60	2 14	8498 8518	12
mmu-miR-7042-5p	XM_006527480.3	143.00	-19.77	2 13	245 266	12
mmu-miR-7042-5p	XM_006527480.3	141.00	-21.16	3 19	2270 2291	17
mmu-miR-7042-5p	NM_001004357.2	147.00	-16.12	2 12	224 244	10
mmu-miR-7042-5p	NM_001004357.2	140.00	-16.53	2 19	5116 5139	20
mmu-miR-7042-5p	XM_017314944.1	141.00	-14.88	2 10	1952 1972	8
mmu-miR-7042-5p	NM_001113515.2	155.00	-23.12	2 20	716 736	18
mmu-miR-7042-5p	XM_006532938.2	160.00	-19.92	2 18	48 69	17
mmu-miR-7042-5p	NM_001309809.2	161.00	-22.82	2 18	726 746	16
mmu-miR-7042-5p	XM_011242311.2	147.00	-22.02	2 20	5507 5527	18
mmu-miR-7042-5p	XM_011242311.2	146.00	-13.72	2 17	3430 3449	15
mmu-miR-7042-5p	NM_007817.2	141.00	-21.43	2 18	1766 1786	16
mmu-miR-7042-5p	NM_001190449.1	150.00	-17.18	2 16	944 965	15
mmu-miR-7042-5p	NM_001190449.1	145.00	-23.19	3 20	768 787	17
mmu-miR-7042-5p	NM_152915.1	154.00	-24.03	2 20	3491 3513	20
mmu-miR-7042-5p	NM_027293.1	140.00	-10.84	2 9	1043 1063	7
mmu-miR-7042-5p	NM_019759.2	151.00	-17.53	2 14	1565 1584	12
mmu-miR-7042-5p	XM_006506808.3	158.00	-20.56	2 19	2067 2087	17
mmu-miR-7042-5p	NM_001317365.1	143.00	-28.72	2 20	2890 2910	18
mmu-miR-7042-5p	NM_007883.3	148.00	-14.12	2 18	811 833	18

mmu-miR-7042-5p	XM_006514873.2	156.00	-19.37	2 19	1228 1247	17
mmu-miR-7042-5p	XM_006514873.2	146.00	-15.97	2 11	1615 1635	9
mmu-miR-7042-5p	XM_006514873.2	145.00	-18.15	2 10	1754 1774	8
mmu-miR-7042-5p	XM_006514873.2	145.00	-20.19	2 19	4157 4178	18
mmu-miR-7042-5p	NM_010090.2	149.00	-19.31	2 19	689 710	18
mmu-miR-7042-5p	XM_011245824.2	145.00	-13.90	2 20	974 993	18
mmu-miR-7042-5p	NM_133643.4	145.00	-16.15	2 10	7530 7550	8
mmu-miR-7042-5p	NM_133643.4	141.00	-12.60	2 14	4674 4694	12
mmu-miR-7042-5p	NM_019397.3	141.00	-20.48	2 19	2044 2065	18
mmu-miR-7042-5p	NM_001291145.1	161.00	-19.81	2 19	3316 3337	18
mmu-miR-7042-5p	XM_006520618.2	146.00	-15.96	2 19	1410 1430	17
mmu-miR-7042-5p	NM_178676.4	146.00	-15.75	2 15	799 819	13
mmu-miR-7042-5p	NM_178676.4	143.00	-21.25	2 20	1582 1602	18
mmu-miR-7042-5p	NM_178676.4	142.00	-14.46	2 13	3027 3046	11
mmu-miR-7042-5p	NM_010140.3	152.00	-18.41	2 18	3921 3943	18
mmu-miR-7042-5p	XM_011238916.2	143.00	-21.08	3 18	1972 1991	15
mmu-miR-7042-5p	XM_006511221.3	146.00	-20.98	2 16	4516 4538	16
mmu-miR-7219-5p	NM_025865.2	152.00	-15.32	2 22	662 685	21
mmu-miR-7219-5p	XM_011241948.2	157.00	-19.18	2 22	307 329	20
mmu-miR-7219-5p	XM_011241948.2	149.00	-17.07	2 22	1887 1909	20
mmu-miR-7219-5p	XM_006498086.3	145.00	-22.03	2 20	2133 2157	20
mmu-miR-7219-5p	XM_006498086.3	144.00	-17.28	2 22	5823 5847	22
mmu-miR-7219-5p	XM_006498086.3	144.00	-14.44	2 22	8156 8180	22
mmu-miR-7219-5p	XM_006498086.3	141.00	-23.54	2 22	5693 5715	20
mmu-miR-7219-5p	NM_013906.3	153.00	-24.66	2 22	2908 2930	20
mmu-miR-7219-5p	XM_006538374.1	164.00	-22.32	2 21	6953 6975	19
mmu-miR-7219-5p	XM_006538374.1	142.00	-16.77	2 20	10341 10364	19
mmu-miR-7219-5p	NM_153178.4	142.00	-18.31	2 20	4745 4770	21
mmu-miR-7219-5p	NM_134072.1	149.00	-20.82	2 18	1497 1519	16
mmu-miR-7219-5p	NM_172790.2	143.00	-18.91	3 17	3091 3114	15
mmu-miR-7219-5p	NM_001080819.1	141.00	-21.26	2 22	5738 5757	20
mmu-miR-7219-5p	NM_029823.2	155.00	-29.20	2 18	833 854	16
mmu-miR-7219-5p	NM_025711.3	149.00	-19.59	2 22	36 58	20
mmu-miR-7219-5p	NM_001310070.1	146.00	-23.81	2 22	1384 1404	20
mmu-miR-7219-5p	NM_007881.4	140.00	-21.72	2 21	3318 3340	19
mmu-miR-7219-5p	XM_006507725.2	144.00	-20.61	3 21	3186 3208	18
mmu-miR-7219-5p	XM_006507725.2	142.00	-21.04	2 22	299 319	20
mmu-miR-7219-5p	NM_020025.4	144.00	-26.78	2 22	1030 1053	21
mmu-miR-7219-5p	XM_006502291.3	143.00	-19.30	2 20	3644 3666	18
mmu-miR-7219-5p	XM_006510701.3	146.00	-28.02	2 21	1139 1166	24
mmu-miR-7219-5p	XM_006510701.3	145.00	-15.24	2 19	2291 2310	17
mmu-miR-7219-5p	NM_178782.4	143.00	-14.96	3 21	1820 1843	19
mmu-miR-7219-5p	XM_006503323.3	141.00	-18.16	2 22	203 225	20
mmu-miR-7219-5p	XM_006503323.3	141.00	-21.92	2 18	1032 1054	16
mmu-miR-7219-5p	XM_006503665.3	152.00	-16.43	2 21	2365 2387	19
mmu-miR-7219-5p	XM_006503665.3	143.00	-20.86	2 21	3313 3336	20
mmu-miR-7219-5p	NM_001097617.1	154.00	-20.50	3 20	2701 2724	18
mmu-miR-7219-5p	XM_011246128.2	149.00	-23.70	2 18	472 494	16
mmu-miR-7219-5p	XM_011246128.2	147.00	-15.92	2 21	1913 1936	20
mmu-miR-7219-5p	XM_011246128.2	140.00	-13.27	2 9	5852 5874	7
mmu-miR-7219-5p	NM_009824.2	148.00	-19.06	2 21	2074 2096	19
mmu-miR-7219-5p	NM_001001178.1	149.00	-18.20	2 20	3067 3088	18
mmu-miR-7219-5p	XM_006533653.3	150.00	-20.15	2 19	575 597	17
mmu-miR-7219-5p	NM_025422.4	148.00	-26.65	2 22	840 865	23
mmu-miR-7219-5p	NM_007655.3	159.00	-23.36	2 22	197 218	20
mmu-miR-7219-5p	XM_011245344.2	148.00	-12.29	2 22	1045 1068	21
mmu-miR-7219-5p	XM_011245344.2	140.00	-15.96	2 9	10080 10102	7
mmu-miR-7219-5p	XM_006503371.2	146.00	-14.07	2 15	902 924	13
mmu-miR-7219-5p	XM_006503371.2	143.00	-16.67	2 22	726 747	20
mmu-miR-7219-5p	XM_006532007.3	153.00	-19.65	2 21	89 109	19
mmu-miR-7219-5p	NM_001301295.1	160.00	-18.16	2 21	1013 1035	19
mmu-miR-7219-5p	XM_006530131.1	142.00	-17.09	2 20	4952 4976	20
mmu-miR-7219-5p	NM_001004357.2	158.00	-27.21	2 21	3185 3211	23
mmu-miR-7219-5p	XM_011240755.1	142.00	-18.28	3 21	941 962	18
mmu-miR-7219-5p	XM_011240755.1	141.00	-14.34	2 18	4293 4315	16

mmu-miR-7219-5p	XM_011240755.1	140.00	-16.28	2 9	3922 3944	7
mmu-miR-7219-5p	XM_011247208.1	159.00	-24.89	2 22	7671 7698	25
mmu-miR-7219-5p	XM_011247208.1	140.00	-19.66	2 22	3578 3601	21
mmu-miR-7219-5p	XM_011248602.2	155.00	-21.29	2 21	2916 2935	19
mmu-miR-7219-5p	NM_152915.1	142.00	-14.37	2 21	1371 1392	19
mmu-miR-7219-5p	NM_152915.1	140.00	-17.14	2 20	2123 2143	18
mmu-miR-7219-5p	XM_011243391.2	149.00	-21.93	2 21	7119 7141	20
mmu-miR-7219-5p	NM_007883.3	150.00	-16.79	2 11	2859 2881	9
mmu-miR-7219-5p	NM_007883.3	147.00	-22.37	2 22	1186 1207	20
mmu-miR-7219-5p	XM_006514873.2	151.00	-25.50	2 21	1513 1536	20
mmu-miR-7219-5p	XM_011245824.2	140.00	-25.47	2 21	1331 1353	19
mmu-miR-7219-5p	NM_010137.3	152.00	-15.58	2 13	3118 3140	11
mmu-miR-7221-3p	XM_006534171.3	144.00	-26.04	2 17	463 483	15
mmu-miR-7221-3p	XM_011241322.1	161.00	-25.05	2 18	406 426	16
mmu-miR-7221-3p	NM_138955.3	164.00	-23.97	2 17	698 718	15
mmu-miR-7221-3p	NM_138955.3	140.00	-27.51	2 18	1393 1414	17
mmu-miR-7221-3p	XM_006540566.1	149.00	-27.99	3 19	2977 2998	17
mmu-miR-7221-3p	XM_006540566.1	145.00	-28.37	2 20	3051 3073	20
mmu-miR-7221-3p	NM_001142804.1	153.00	-20.65	2 14	636 656	12
mmu-miR-7221-3p	XM_006498086.3	141.00	-19.67	2 20	432 451	18
mmu-miR-7221-3p	XM_006538374.1	147.00	-23.95	2 20	5620 5637	18
mmu-miR-7221-3p	XM_006538374.1	146.00	-21.12	2 18	4035 4055	17
mmu-miR-7221-3p	XM_006538374.1	144.00	-25.84	2 13	5318 5338	11
mmu-miR-7221-3p	XM_006538374.1	144.00	-21.60	2 14	8925 8946	13
mmu-miR-7221-3p	XM_006538374.1	141.00	-23.91	3 20	2805 2824	17
mmu-miR-7221-3p	XM_006505388.2	149.00	-21.51	2 20	1665 1684	18
mmu-miR-7221-3p	XM_017312667.1	166.00	-26.50	2 15	4648 4668	13
mmu-miR-7221-3p	NM_008032.3	154.00	-19.39	2 15	1758 1778	13
mmu-miR-7221-3p	NM_009645.2	149.00	-20.74	2 19	1384 1408	21
mmu-miR-7221-3p	XM_006498287.3	148.00	-15.93	2 13	516 536	11
mmu-miR-7221-3p	NM_031185.3	141.00	-22.85	2 15	506 527	14
mmu-miR-7221-3p	NM_008012.1	148.00	-23.75	2 19	683 702	17
mmu-miR-7221-3p	NM_134072.1	148.00	-18.71	2 18	1121 1142	17
mmu-miR-7221-3p	NM_053080.3	141.00	-23.82	2 19	1235 1257	19
mmu-miR-7221-3p	XM_006508990.2	141.00	-22.11	2 14	2241 2261	12
mmu-miR-7221-3p	NM_172790.2	148.00	-26.17	3 17	4792 4812	14
mmu-miR-7221-3p	NM_172790.2	143.00	-23.01	3 20	3041 3061	17
mmu-miR-7221-3p	NM_001305585.1	158.00	-29.73	2 19	417 437	17
mmu-miR-7221-3p	NM_007482.3	152.00	-19.86	2 13	390 410	11
mmu-miR-7221-3p	NM_001080819.1	150.00	-18.69	2 17	1658 1677	15
mmu-miR-7221-3p	NM_001080819.1	145.00	-21.58	2 18	3498 3518	16
mmu-miR-7221-3p	NM_023048.5	141.00	-21.07	2 18	982 1002	16
mmu-miR-7221-3p	NM_007881.4	158.00	-30.60	2 20	622 643	19
mmu-miR-7221-3p	XM_006529727.2	141.00	-31.05	2 20	3404 3423	18
mmu-miR-7221-3p	XM_006507725.2	150.00	-23.47	2 11	759 779	9
mmu-miR-7221-3p	NM_007529.2	150.00	-24.29	2 15	454 474	13
mmu-miR-7221-3p	XM_006502291.3	142.00	-20.86	2 18	5634 5652	16
mmu-miR-7221-3p	XM_006510701.3	145.00	-19.58	2 10	2131 2151	8
mmu-miR-7221-3p	NM_178782.4	156.00	-27.86	2 19	1135 1154	17
mmu-miR-7221-3p	NM_178782.4	156.00	-23.84	2 17	2196 2216	15
mmu-miR-7221-3p	NM_178782.4	140.00	-28.22	2 17	3005 3025	15
mmu-miR-7221-3p	NM_028472.2	140.00	-17.60	2 9	1836 1856	7
mmu-miR-7221-3p	XM_006524328.3	145.00	-26.58	2 20	669 693	22
mmu-miR-7221-3p	XM_006524328.3	142.00	-21.99	2 17	2190 2209	15
mmu-miR-7221-3p	XM_006503665.3	152.00	-24.59	2 13	972 992	11
mmu-miR-7221-3p	XM_006503665.3	149.00	-23.77	2 20	3485 3510	23
mmu-miR-7221-3p	NM_001097617.1	150.00	-27.24	2 20	2155 2176	19
mmu-miR-7221-3p	NM_001285867.1	152.00	-21.45	2 20	2656 2677	20
mmu-miR-7221-3p	NM_001285867.1	141.00	-24.78	2 18	3089 3109	16
mmu-miR-7221-3p	NM_001285867.1	141.00	-27.16	2 20	3614 3636	20
mmu-miR-7221-3p	NM_001190379.1	147.00	-22.55	2 20	1491 1511	18
mmu-miR-7221-3p	XM_011246479.2	144.00	-29.13	3 20	3169 3189	18
mmu-miR-7221-3p	NM_181315.4	144.00	-12.37	2 13	983 1003	11
mmu-miR-7221-3p	XM_006520253.3	151.00	-21.20	2 20	1513 1532	19
mmu-miR-7221-3p	XM_006520253.3	146.00	-22.57	2 11	4426 4446	9

mmu-miR-7221-3p	NM_009824.2	146.00	-21.20	2 19	5329 5349	17
mmu-miR-7221-3p	NM_001013784.1	147.00	-26.09	2 20	92 112	18
mmu-miR-7221-3p	XM_006533653.3	144.00	-26.02	2 20	539 560	20
mmu-miR-7221-3p	XM_006533653.3	142.00	-24.13	2 20	554 575	19
mmu-miR-7221-3p	XM_006533653.3	142.00	-24.13	2 20	569 590	19
mmu-miR-7221-3p	XM_017312731.1	149.00	-17.46	2 20	1651 1670	18
mmu-miR-7221-3p	XM_017312731.1	140.00	-18.91	2 9	602 622	7
mmu-miR-7221-3p	XM_006530131.1	156.00	-24.66	2 17	8258 8278	15
mmu-miR-7221-3p	XM_006530131.1	148.00	-31.85	2 20	5373 5391	18
mmu-miR-7221-3p	XM_006530131.1	142.00	-27.64	2 19	8246 8267	18
mmu-miR-7221-3p	XM_006527480.3	160.00	-30.47	2 17	1570 1590	15
mmu-miR-7221-3p	NM_001113515.2	141.00	-25.20	2 20	4543 4562	18
mmu-miR-7221-3p	NM_007739.2	144.00	-24.87	3 19	4744 4766	18
mmu-miR-7221-3p	XM_006532938.2	152.00	-35.23	2 20	789 815	24
mmu-miR-7221-3p	XM_011247208.1	140.00	-21.81	2 19	2579 2601	19
mmu-miR-7221-3p	XM_011248602.2	164.00	-29.78	2 17	188 208	15
mmu-miR-7221-3p	XM_011248602.2	140.00	-13.57	2 9	2692 2712	7
mmu-miR-7221-3p	NM_001289782.1	146.00	-20.98	2 11	212 232	9
mmu-miR-7221-3p	NM_030206.4	162.00	-35.64	2 19	191 211	17
mmu-miR-7221-3p	XM_006540596.2	141.00	-24.31	2 19	232 256	21
mmu-miR-7221-3p	NM_001190448.1	147.00	-19.58	2 20	31 51	18
mmu-miR-7221-3p	NM_001190448.1	145.00	-16.07	2 18	1131 1151	16
mmu-miR-7221-3p	NM_001190448.1	141.00	-13.34	2 17	344 362	15
mmu-miR-7221-3p	NM_015814.2	156.00	-27.85	2 20	934 954	19
mmu-miR-7221-3p	NM_015814.2	140.00	-12.04	2 9	423 443	7
mmu-miR-7221-3p	NM_010055.3	144.00	-26.20	2 17	297 317	15
mmu-miR-7221-3p	NM_027293.1	153.00	-25.09	2 16	7160 7179	14
mmu-miR-7221-3p	XM_011243391.2	150.00	-21.88	2 19	3657 3677	17
mmu-miR-7221-3p	XM_011243391.2	142.00	-23.47	3 17	2556 2575	14
mmu-miR-7221-3p	NM_007883.3	141.00	-24.13	2 20	3581 3600	18
mmu-miR-7221-3p	XM_006514873.2	145.00	-23.21	2 10	285 305	8
mmu-miR-7221-3p	XM_011245824.2	143.00	-25.07	3 18	1471 1490	15
mmu-miR-7221-3p	NM_001291145.1	142.00	-22.11	2 16	3012 3033	15
mmu-miR-7221-3p	NM_015744.4	143.00	-30.07	2 20	3083 3103	18
mmu-miR-7221-3p	NM_010137.3	145.00	-25.30	3 20	536 559	20
mmu-miR-7221-3p	NM_010137.3	140.00	-24.88	3 20	2822 2840	17
mmu-miR-7221-3p	NM_010140.3	169.00	-32.60	2 18	2254 2274	16
mmu-miR-7221-3p	NM_010140.3	149.00	-22.06	2 19	3893 3917	21
mmu-miR-7221-3p	NM_010140.3	140.00	-22.75	3 19	740 759	16
mmu-miR-7221-3p	XM_011238916.2	144.00	-26.50	2 18	3376 3397	17
mmu-miR-7221-3p	XM_006511221.3	144.00	-25.25	2 18	905 927	18
mmu-miR-7221-3p	XM_006511221.3	143.00	-23.48	3 16	3372 3392	13
mmu-miR-7226-5p	XM_017320219.1	148.00	-27.90	2 25	291 314	23
mmu-miR-7226-5p	XM_017320219.1	144.00	-22.82	3 25	4720 4743	22
mmu-miR-7226-5p	NM_029685.1	149.00	-22.24	2 23	661 687	22
mmu-miR-7226-5p	NM_029685.1	143.00	-22.34	4 25	874 900	22
mmu-miR-7226-5p	NM_025865.2	153.00	-22.60	2 23	6 35	25
mmu-miR-7226-5p	NM_001271580.1	142.00	-16.54	3 24	961 988	23
mmu-miR-7226-5p	XM_011241322.1	174.00	-37.97	2 25	2943 2972	27
mmu-miR-7226-5p	XM_011241322.1	158.00	-25.75	3 25	2310 2334	22
mmu-miR-7226-5p	XM_011241322.1	157.00	-20.93	2 22	1969 1994	20
mmu-miR-7226-5p	XM_011241322.1	147.00	-23.42	3 21	2807 2834	20
mmu-miR-7226-5p	XM_011241322.1	147.00	-16.12	2 24	3535 3560	22
mmu-miR-7226-5p	XM_011241322.1	144.00	-22.44	3 25	1510 1535	22
mmu-miR-7226-5p	XM_011241322.1	142.00	-23.05	2 22	3284 3307	20
mmu-miR-7226-5p	NM_029982.1	141.00	-23.55	3 22	199 224	19
mmu-miR-7226-5p	NM_138955.3	150.00	-24.86	2 23	960 983	21
mmu-miR-7226-5p	NM_138955.3	147.00	-25.89	2 25	3380 3407	25
mmu-miR-7226-5p	NM_138955.3	145.00	-32.39	2 25	820 852	30
mmu-miR-7226-5p	NM_138955.3	141.00	-24.92	2 22	3031 3056	20
mmu-miR-7226-5p	XM_006540566.1	167.00	-30.64	2 22	6725 6749	20
mmu-miR-7226-5p	XM_006540566.1	166.00	-23.27	2 23	1076 1101	21
mmu-miR-7226-5p	XM_006540566.1	155.00	-23.87	2 25	1824 1851	25
mmu-miR-7226-5p	XM_006540566.1	142.00	-16.50	2 23	5035 5058	21
mmu-miR-7226-5p	XM_006540566.1	140.00	-23.90	3 21	2408 2433	18

mmu-miR-7226-5p	NM_153151.3	141.00	-19.84	2 25	1005 1034	27
mmu-miR-7226-5p	NM_172580.1	156.00	-26.21	2 21	770 795	19
mmu-miR-7226-5p	NM_001142804.1	146.00	-21.73	2 24	1638 1665	24
mmu-miR-7226-5p	XM_006498086.3	158.00	-21.62	2 21	5410 5437	21
mmu-miR-7226-5p	XM_006498086.3	148.00	-25.67	2 25	6405 6430	23
mmu-miR-7226-5p	NM_013906.3	151.00	-23.50	2 24	161 186	22
mmu-miR-7226-5p	NM_013906.3	150.00	-24.12	2 25	4555 4584	27
mmu-miR-7226-5p	NM_013906.3	148.00	-21.40	2 24	4530 4551	22
mmu-miR-7226-5p	NM_013906.3	146.00	-20.47	2 21	691 715	19
mmu-miR-7226-5p	NM_013906.3	141.00	-21.17	2 15	3639 3666	15
mmu-miR-7226-5p	NM_013906.3	140.00	-15.97	2 9	1895 1920	7
mmu-miR-7226-5p	XM_006538374.1	149.00	-22.21	2 22	6726 6750	21
mmu-miR-7226-5p	XM_006538374.1	147.00	-28.67	2 22	4872 4896	20
mmu-miR-7226-5p	XM_006538374.1	143.00	-21.53	2 25	6600 6628	26
mmu-miR-7226-5p	XM_006538374.1	140.00	-19.63	2 25	7402 7427	23
mmu-miR-7226-5p	XM_006505388.2	162.00	-26.82	2 25	2650 2678	26
mmu-miR-7226-5p	XM_006505388.2	151.00	-27.01	2 25	5948 5970	23
mmu-miR-7226-5p	XM_006505388.2	147.00	-19.26	2 25	1501 1526	25
mmu-miR-7226-5p	NM_145635.2	164.00	-24.56	2 23	424 448	21
mmu-miR-7226-5p	NM_145635.2	151.00	-29.08	2 22	160 184	20
mmu-miR-7226-5p	NM_009605.4	146.00	-18.18	2 21	1029 1053	19
mmu-miR-7226-5p	NM_001291930.1	155.00	-23.40	2 25	4467 4495	26
mmu-miR-7226-5p	NM_001291930.1	150.00	-28.51	2 25	1599 1623	23
mmu-miR-7226-5p	NM_001291930.1	150.00	-26.30	2 25	2146 2175	27
mmu-miR-7226-5p	XM_017312667.1	161.00	-27.91	2 20	5265 5289	18
mmu-miR-7226-5p	XM_017312667.1	144.00	-20.42	3 21	1158 1183	18
mmu-miR-7226-5p	XM_017312667.1	143.00	-25.10	2 21	5286 5312	20
mmu-miR-7226-5p	XM_017312667.1	141.00	-23.52	2 22	927 952	20
mmu-miR-7226-5p	XM_017312667.1	140.00	-14.87	2 21	6699 6724	19
mmu-miR-7226-5p	NM_008032.3	144.00	-17.24	2 23	3927 3954	23
mmu-miR-7226-5p	NM_153178.4	146.00	-26.32	2 25	6892 6919	25
mmu-miR-7226-5p	NM_153178.4	141.00	-19.60	2 21	4100 4123	19
mmu-miR-7226-5p	XM_011250176.1	144.00	-21.39	2 17	5121 5146	15
mmu-miR-7226-5p	XM_011250176.1	142.00	-25.18	2 24	4304 4330	23
mmu-miR-7226-5p	XM_011250176.1	140.00	-22.68	2 25	5587 5612	23
mmu-miR-7226-5p	NM_009645.2	142.00	-21.54	2 25	1336 1360	23
mmu-miR-7226-5p	NM_031185.3	153.00	-21.79	2 22	5724 5749	20
mmu-miR-7226-5p	NM_031185.3	141.00	-19.14	2 24	2541 2569	25
mmu-miR-7226-5p	NM_031185.3	141.00	-23.50	3 24	4789 4813	21
mmu-miR-7226-5p	NM_031185.3	140.00	-14.46	2 22	1535 1561	21
mmu-miR-7226-5p	XM_011240149.1	140.00	-17.34	3 23	67 91	20
mmu-miR-7226-5p	NM_053080.3	141.00	-22.25	2 23	1678 1704	22
mmu-miR-7226-5p	XM_006508990.2	153.00	-25.27	3 23	3688 3714	21
mmu-miR-7226-5p	XM_006508990.2	152.00	-29.93	2 21	7852 7877	19
mmu-miR-7226-5p	XM_006508990.2	149.00	-24.68	2 14	5131 5156	12
mmu-miR-7226-5p	XM_006508990.2	143.00	-23.14	4 25	3580 3606	22
mmu-miR-7226-5p	XM_017318264.1	158.00	-23.71	2 22	316 339	20
mmu-miR-7226-5p	XM_017318264.1	156.00	-24.27	2 22	583 609	21
mmu-miR-7226-5p	NM_172790.2	157.00	-30.44	2 25	2695 2718	23
mmu-miR-7226-5p	NM_172790.2	146.00	-23.76	3 25	3720 3748	25
mmu-miR-7226-5p	NM_172790.2	143.00	-25.39	2 25	4662 4691	27
mmu-miR-7226-5p	NM_172790.2	143.00	-26.04	2 22	6458 6485	22
mmu-miR-7226-5p	NM_172790.2	141.00	-24.30	2 25	4103 4126	23
mmu-miR-7226-5p	NM_172790.2	141.00	-30.47	2 24	4874 4904	27
mmu-miR-7226-5p	NM_013912.3	151.00	-24.02	2 25	629 657	26
mmu-miR-7226-5p	NM_001301354.1	150.00	-23.13	2 24	885 911	23
mmu-miR-7226-5p	NM_207231.1	157.00	-21.46	3 22	1536 1561	19
mmu-miR-7226-5p	XM_006522398.3	147.00	-18.94	2 21	1072 1099	21
mmu-miR-7226-5p	XM_006522398.3	142.00	-19.20	2 25	1276 1304	26
mmu-miR-7226-5p	NM_029823.2	150.00	-25.75	3 25	817 845	25
mmu-miR-7226-5p	NM_029823.2	143.00	-24.99	3 22	129 153	19
mmu-miR-7226-5p	NM_029823.2	143.00	-23.47	3 25	329 355	23
mmu-miR-7226-5p	NM_023048.5	145.00	-15.35	2 24	1370 1398	25
mmu-miR-7226-5p	NM_023048.5	141.00	-19.11	2 23	230 256	22
mmu-miR-7226-5p	NM_001310070.1	151.00	-19.56	3 24	1819 1844	21

mmu-miR-7226-5p	NM_001310070.1	143.00	-20.01	2 23	1119 1146	24
mmu-miR-7226-5p	NM_001310070.1	143.00	-16.49	2 20	1504 1529	18
mmu-miR-7226-5p	NM_007881.4	170.00	-31.15	2 25	1881 1908	25
mmu-miR-7226-5p	NM_007881.4	153.00	-18.90	2 21	1304 1327	19
mmu-miR-7226-5p	NM_001290469.1	153.00	-27.30	2 14	2590 2615	12
mmu-miR-7226-5p	NM_001290469.1	150.00	-22.77	3 23	2480 2505	20
mmu-miR-7226-5p	NM_001290469.1	149.00	-21.42	2 25	3480 3506	25
mmu-miR-7226-5p	NM_001290469.1	146.00	-21.52	3 25	2079 2103	22
mmu-miR-7226-5p	NM_001290469.1	145.00	-29.48	2 25	835 860	24
mmu-miR-7226-5p	NM_013415.5	157.00	-26.61	2 22	311 336	20
mmu-miR-7226-5p	NM_013415.5	149.00	-22.66	2 23	2211 2238	23
mmu-miR-7226-5p	NM_013415.5	145.00	-18.73	3 22	1220 1245	19
mmu-miR-7226-5p	XM_006529727.2	167.00	-24.94	2 23	1197 1222	22
mmu-miR-7226-5p	XM_006529727.2	166.00	-29.12	2 21	3514 3538	19
mmu-miR-7226-5p	XM_006529727.2	153.00	-22.66	2 19	5834 5860	18
mmu-miR-7226-5p	XM_006529727.2	146.00	-20.43	2 21	817 841	19
mmu-miR-7226-5p	XM_006529727.2	146.00	-20.92	2 24	4471 4493	22
mmu-miR-7226-5p	XM_006507725.2	148.00	-27.09	2 22	2013 2039	21
mmu-miR-7226-5p	NM_001159407.1	146.00	-18.33	3 25	1311 1333	22
mmu-miR-7226-5p	NM_013863.5	142.00	-21.96	2 19	9 34	17
mmu-miR-7226-5p	XM_006502291.3	149.00	-25.50	2 24	906 930	22
mmu-miR-7226-5p	XM_006502291.3	142.00	-22.51	2 25	5691 5715	23
mmu-miR-7226-5p	XM_006502291.3	142.00	-18.86	2 25	5783 5807	23
mmu-miR-7226-5p	XM_006502291.3	141.00	-19.08	3 19	275 301	17
mmu-miR-7226-5p	XM_006510701.3	150.00	-26.71	2 11	3020 3045	9
mmu-miR-7226-5p	XM_006510701.3	147.00	-23.35	2 22	5115 5139	20
mmu-miR-7226-5p	XM_006510701.3	140.00	-27.58	3 22	1158 1186	22
mmu-miR-7226-5p	XM_006510701.3	140.00	-24.56	2 25	1901 1923	23
mmu-miR-7226-5p	XM_006510701.3	140.00	-21.07	2 25	5660 5684	24
mmu-miR-7226-5p	NM_178782.4	154.00	-25.06	2 25	1846 1870	23
mmu-miR-7226-5p	NM_178782.4	144.00	-19.70	2 22	3657 3684	22
mmu-miR-7226-5p	NM_178782.4	142.00	-22.63	2 23	2352 2377	21
mmu-miR-7226-5p	NM_178782.4	140.00	-18.14	2 23	3843 3867	21
mmu-miR-7226-5p	XM_006503323.3	150.00	-19.80	3 24	435 461	22
mmu-miR-7226-5p	NM_001002896.2	164.00	-24.77	2 22	189 215	21
mmu-miR-7226-5p	NM_001002896.2	153.00	-23.38	2 23	1457 1483	22
mmu-miR-7226-5p	NM_001002896.2	140.00	-15.61	3 17	709 734	14
mmu-miR-7226-5p	NM_007549.2	148.00	-29.64	2 25	1225 1250	23
mmu-miR-7226-5p	NM_007555.4	140.00	-19.49	2 9	1020 1045	7
mmu-miR-7226-5p	NM_028472.2	146.00	-26.38	3 23	1888 1913	20
mmu-miR-7226-5p	NM_020508.4	148.00	-21.12	2 25	2124 2149	23
mmu-miR-7226-5p	NM_020508.4	141.00	-17.51	3 25	530 553	22
mmu-miR-7226-5p	XM_006524328.3	155.00	-19.56	2 22	5271 5295	20
mmu-miR-7226-5p	XM_006524328.3	153.00	-21.75	2 22	4335 4360	20
mmu-miR-7226-5p	XM_006524328.3	149.00	-23.11	2 25	1523 1548	24
mmu-miR-7226-5p	NM_178692.3	156.00	-31.11	2 25	264 287	23
mmu-miR-7226-5p	NM_178692.3	144.00	-25.16	2 25	2677 2702	23
mmu-miR-7226-5p	NM_026979.5	162.00	-21.27	2 19	332 357	17
mmu-miR-7226-5p	XM_006503665.3	164.00	-21.53	2 25	3582 3607	23
mmu-miR-7226-5p	XM_006503665.3	161.00	-25.03	2 22	372 397	20
mmu-miR-7226-5p	XM_006503665.3	142.00	-21.06	3 25	32 56	22
mmu-miR-7226-5p	XM_006503665.3	141.00	-20.96	2 20	445 469	18
mmu-miR-7226-5p	NM_023143.3	144.00	-22.20	2 25	865 888	23
mmu-miR-7226-5p	NM_023143.3	140.00	-17.24	3 24	13 41	24
mmu-miR-7226-5p	NM_023143.3	140.00	-19.28	2 22	1722 1749	22
mmu-miR-7226-5p	NM_001097617.1	159.00	-25.01	2 25	1569 1597	26
mmu-miR-7226-5p	NM_001097617.1	152.00	-19.93	2 19	990 1014	17
mmu-miR-7226-5p	NM_001097617.1	142.00	-20.45	2 24	1815 1841	23
mmu-miR-7226-5p	NM_001285867.1	148.00	-19.75	2 20	4490 4513	18
mmu-miR-7226-5p	NM_001285867.1	147.00	-26.27	2 18	993 1017	16
mmu-miR-7226-5p	NM_009785.1	165.00	-28.81	2 22	2597 2622	20
mmu-miR-7226-5p	NM_009785.1	142.00	-20.27	2 25	2376 2405	27
mmu-miR-7226-5p	XM_011246128.2	162.00	-30.79	2 25	7258 7290	30
mmu-miR-7226-5p	XM_011246128.2	148.00	-22.61	2 23	224 252	24
mmu-miR-7226-5p	XM_011246128.2	142.00	-20.23	2 19	3766 3791	17

mmu-miR-7226-5p	XM_011246128.2	140.00	-20.70	3 21	26 51	18
mmu-miR-7226-5p	XM_011246128.2	140.00	-17.60	3 21	5828 5853	18
mmu-miR-7226-5p	NM_007588.2	151.00	-20.91	3 25	3024 3051	24
mmu-miR-7226-5p	NM_007588.2	150.00	-18.94	2 24	1774 1800	23
mmu-miR-7226-5p	NM_007588.2	148.00	-27.74	2 22	40 69	24
mmu-miR-7226-5p	NM_027416.3	154.00	-24.44	2 21	210 237	21
mmu-miR-7226-5p	NM_001190379.1	168.00	-27.52	2 25	1926 1951	23
mmu-miR-7226-5p	NM_001190379.1	151.00	-24.78	2 16	4421 4446	14
mmu-miR-7226-5p	XM_011246479.2	143.00	-25.89	2 25	3299 3321	23
mmu-miR-7226-5p	XM_006520186.2	148.00	-19.49	2 18	559 585	17
mmu-miR-7226-5p	XM_006501443.3	166.00	-26.70	2 25	594 618	23
mmu-miR-7226-5p	XM_006501443.3	155.00	-28.24	3 22	1012 1039	21
mmu-miR-7226-5p	XM_006501443.3	140.00	-15.12	3 25	1993 2018	22
mmu-miR-7226-5p	NM_181315.4	147.00	-21.84	2 20	2593 2618	18
mmu-miR-7226-5p	NM_181315.4	143.00	-21.84	2 24	3263 3287	23
mmu-miR-7226-5p	XM_006520253.3	146.00	-23.36	3 25	2330 2358	25
mmu-miR-7226-5p	XM_006520253.3	143.00	-23.70	2 25	1985 2007	23
mmu-miR-7226-5p	XM_006520253.3	142.00	-21.64	2 24	2825 2851	23
mmu-miR-7226-5p	XM_006520253.3	141.00	-22.31	3 23	2641 2665	21
mmu-miR-7226-5p	XM_006500947.3	140.00	-19.40	2 21	701 726	19
mmu-miR-7226-5p	NM_009824.2	162.00	-20.03	2 24	4667 4693	23
mmu-miR-7226-5p	NM_009824.2	152.00	-21.80	2 17	4217 4242	15
mmu-miR-7226-5p	NM_009824.2	146.00	-18.11	3 23	6744 6769	20
mmu-miR-7226-5p	NM_009824.2	143.00	-23.37	4 24	6381 6406	20
mmu-miR-7226-5p	NM_009824.2	141.00	-14.70	3 22	5382 5407	19
mmu-miR-7226-5p	NM_019626.3	155.00	-22.06	2 25	887 913	24
mmu-miR-7226-5p	NM_019626.3	153.00	-28.28	2 25	2087 2110	23
mmu-miR-7226-5p	XM_006526449.3	163.00	-26.37	2 25	1883 1909	24
mmu-miR-7226-5p	XM_006526449.3	161.00	-26.02	2 23	559 586	23
mmu-miR-7226-5p	XM_006526449.3	146.00	-16.96	2 25	1957 1981	23
mmu-miR-7226-5p	NM_028804.1	163.00	-27.21	2 25	2482 2505	24
mmu-miR-7226-5p	NM_028804.1	150.00	-24.40	3 25	1815 1839	22
mmu-miR-7226-5p	NM_001013784.1	149.00	-20.75	2 22	260 285	20
mmu-miR-7226-5p	NM_001013784.1	147.00	-27.50	2 25	3627 3655	26
mmu-miR-7226-5p	NM_001013784.1	146.00	-18.70	2 24	1932 1959	24
mmu-miR-7226-5p	NM_001013784.1	145.00	-19.68	2 25	1980 2003	23
mmu-miR-7226-5p	NM_001013784.1	141.00	-22.80	3 22	1425 1450	19
mmu-miR-7226-5p	XM_006508867.2	148.00	-24.12	2 22	605 631	21
mmu-miR-7226-5p	XM_006508867.2	142.00	-23.89	2 25	401 424	24
mmu-miR-7226-5p	XM_006508885.1	153.00	-24.68	2 23	689 715	22
mmu-miR-7226-5p	XM_006508885.1	142.00	-23.89	2 25	485 508	24
mmu-miR-7226-5p	XM_006509966.3	141.00	-23.55	2 25	1313 1333	23
mmu-miR-7226-5p	NM_001313939.1	145.00	-22.95	3 22	764 789	19
mmu-miR-7226-5p	NM_001313939.1	142.00	-20.66	2 25	33 57	23
mmu-miR-7226-5p	NM_001313939.1	140.00	-17.55	2 22	900 927	22
mmu-miR-7226-5p	XM_011245344.2	152.00	-27.43	2 25	1018 1040	23
mmu-miR-7226-5p	XM_011245344.2	148.00	-24.98	2 25	3190 3213	23
mmu-miR-7226-5p	XM_011245344.2	143.00	-17.24	2 24	542 567	22
mmu-miR-7226-5p	XM_011245344.2	143.00	-19.64	3 23	7970 7999	24
mmu-miR-7226-5p	XM_011245344.2	142.00	-19.85	2 25	10627 10651	23
mmu-miR-7226-5p	XM_011245344.2	142.00	-21.90	3 25	11829 11853	22
mmu-miR-7226-5p	XM_011245344.2	140.00	-21.56	2 22	10499 10525	21
mmu-miR-7226-5p	XM_006531321.3	150.00	-22.19	2 24	1700 1727	24
mmu-miR-7226-5p	XM_006531321.3	142.00	-18.76	3 23	1679 1704	20
mmu-miR-7226-5p	XM_006531321.3	141.00	-29.71	2 25	458 487	27
mmu-miR-7226-5p	XM_006503371.2	158.00	-23.39	2 21	2660 2684	19
mmu-miR-7226-5p	XM_006503371.2	147.00	-19.04	3 25	1175 1202	24
mmu-miR-7226-5p	XM_006503371.2	142.00	-23.06	2 25	863 887	23
mmu-miR-7226-5p	XM_006532007.3	157.00	-22.86	2 24	1487 1511	22
mmu-miR-7226-5p	NM_001301295.1	158.00	-23.37	2 25	296 323	25
mmu-miR-7226-5p	NM_001301295.1	143.00	-20.67	2 25	694 716	23
mmu-miR-7226-5p	NM_001301295.1	140.00	-21.45	2 23	798 822	21
mmu-miR-7226-5p	XM_006530131.1	163.00	-26.35	2 23	5902 5927	22
mmu-miR-7226-5p	XM_006530131.1	158.00	-29.05	3 25	5718 5742	22
mmu-miR-7226-5p	XM_006530131.1	155.00	-19.77	2 24	2622 2645	22

mmu-miR-7226-5p	XM_006530131.1	151.00	-27.30	2 25	5675 5702	25
mmu-miR-7226-5p	XM_006530131.1	149.00	-27.28	2 25	7472 7497	24
mmu-miR-7226-5p	XM_006530131.1	148.00	-20.33	2 21	2265 2288	19
mmu-miR-7226-5p	XM_006530131.1	147.00	-21.84	2 21	7558 7585	21
mmu-miR-7226-5p	XM_006530131.1	145.00	-18.48	2 17	5361 5384	15
mmu-miR-7226-5p	XM_006530131.1	144.00	-23.93	2 22	5654 5681	22
mmu-miR-7226-5p	XM_006530131.1	144.00	-23.57	3 25	7801 7826	22
mmu-miR-7226-5p	XM_006530131.1	143.00	-26.26	3 23	2935 2963	23
mmu-miR-7226-5p	NM_020001.2	148.00	-22.97	3 25	94 119	22
mmu-miR-7226-5p	XM_011250217.1	148.00	-19.21	2 13	1 17	11
mmu-miR-7226-5p	XM_011250217.1	140.00	-25.35	2 25	224 249	23
mmu-miR-7226-5p	XM_006527480.3	154.00	-18.48	2 25	3331 3355	23
mmu-miR-7226-5p	XM_006527480.3	153.00	-26.31	2 22	348 373	20
mmu-miR-7226-5p	XM_006527480.3	149.00	-31.06	2 25	2298 2323	24
mmu-miR-7226-5p	XM_006532120.2	162.00	-25.02	2 23	408 430	21
mmu-miR-7226-5p	NM_001004357.2	153.00	-24.75	2 24	3670 3697	24
mmu-miR-7226-5p	NM_001004357.2	145.00	-25.01	3 25	368 393	23
mmu-miR-7226-5p	XM_011248983.2	150.00	-24.11	2 22	1160 1186	22
mmu-miR-7226-5p	NM_001113515.2	164.00	-30.79	2 25	1820 1845	23
mmu-miR-7226-5p	NM_001113515.2	160.00	-31.72	2 22	1107 1134	22
mmu-miR-7226-5p	NM_001113515.2	154.00	-24.06	2 23	1307 1332	21
mmu-miR-7226-5p	NM_001113515.2	154.00	-25.54	2 19	2702 2727	17
mmu-miR-7226-5p	NM_001113515.2	148.00	-21.94	2 17	812 837	15
mmu-miR-7226-5p	NM_001113515.2	147.00	-20.57	2 18	3549 3573	16
mmu-miR-7226-5p	NM_001113515.2	146.00	-30.48	2 25	2168 2196	26
mmu-miR-7226-5p	NM_009930.2	166.00	-28.24	2 25	584 612	26
mmu-miR-7226-5p	NM_009930.2	160.00	-25.64	2 25	1928 1953	23
mmu-miR-7226-5p	NM_009930.2	154.00	-20.39	2 23	965 990	21
mmu-miR-7226-5p	NM_009930.2	154.00	-25.08	2 25	3517 3546	27
mmu-miR-7226-5p	NM_009930.2	149.00	-28.13	2 25	1214 1242	26
mmu-miR-7226-5p	NM_009930.2	148.00	-25.22	2 25	2261 2286	23
mmu-miR-7226-5p	NM_009930.2	141.00	-18.58	2 19	1126 1152	18
mmu-miR-7226-5p	NM_007739.2	167.00	-28.21	2 25	1429 1456	25
mmu-miR-7226-5p	NM_007739.2	154.00	-25.30	2 18	1514 1537	16
mmu-miR-7226-5p	NM_007739.2	145.00	-29.33	2 10	1791 1816	8
mmu-miR-7226-5p	NM_007739.2	143.00	-21.87	2 25	4267 4289	23
mmu-miR-7226-5p	XM_011240755.1	142.00	-26.94	3 25	774 803	26
mmu-miR-7226-5p	XM_011247208.1	158.00	-22.19	2 23	3830 3853	21
mmu-miR-7226-5p	XM_011247208.1	142.00	-28.47	2 25	1246 1277	29
mmu-miR-7226-5p	XM_017315376.1	159.00	-22.66	2 25	354 376	23
mmu-miR-7226-5p	XM_011248602.2	163.00	-25.26	2 25	1096 1122	24
mmu-miR-7226-5p	XM_011248602.2	151.00	-22.20	2 25	383 410	25
mmu-miR-7226-5p	XM_011248602.2	147.00	-24.20	2 22	3219 3243	20
mmu-miR-7226-5p	XM_011248602.2	146.00	-16.10	2 24	2749 2775	23
mmu-miR-7226-5p	XM_011248602.2	145.00	-19.85	2 24	3011 3040	26
mmu-miR-7226-5p	XM_011248602.2	141.00	-13.84	2 21	1368 1391	19
mmu-miR-7226-5p	XM_011248602.2	141.00	-20.69	2 21	1568 1591	19
mmu-miR-7226-5p	NM_011957.2	152.00	-27.05	2 21	1876 1901	19
mmu-miR-7226-5p	NM_011957.2	151.00	-24.47	2 24	102 127	22
mmu-miR-7226-5p	NM_011957.2	140.00	-23.65	3 25	314 337	22
mmu-miR-7226-5p	XM_011242311.2	157.00	-30.88	2 25	5383 5409	25
mmu-miR-7226-5p	XM_011242311.2	155.00	-28.42	2 24	1908 1934	23
mmu-miR-7226-5p	XM_011242311.2	145.00	-21.54	3 18	4403 4428	15
mmu-miR-7226-5p	XM_011242311.2	144.00	-26.89	2 25	1711 1736	25
mmu-miR-7226-5p	NM_001289782.1	160.00	-29.80	3 25	557 582	22
mmu-miR-7226-5p	NM_001289782.1	151.00	-24.53	3 25	834 861	24
mmu-miR-7226-5p	NM_001289782.1	140.00	-16.76	3 23	271 295	20
mmu-miR-7226-5p	NM_018866.2	145.00	-24.31	2 25	877 900	23
mmu-miR-7226-5p	NM_030206.4	155.00	-22.47	3 24	806 829	21
mmu-miR-7226-5p	NM_030206.4	153.00	-21.02	3 25	1285 1310	23
mmu-miR-7226-5p	NM_030206.4	148.00	-25.51	2 25	970 995	23
mmu-miR-7226-5p	NM_030206.4	140.00	-15.34	2 9	1131 1156	7
mmu-miR-7226-5p	NM_007817.2	153.00	-26.22	2 22	826 851	20
mmu-miR-7226-5p	NM_015814.2	141.00	-23.08	3 22	1603 1625	19
mmu-miR-7226-5p	NM_010055.3	154.00	-26.72	2 25	1696 1720	23

mmu-miR-7226-5p	NM_010055.3	153.00	-25.99	2 23	2135 2161	22
mmu-miR-7226-5p	NM_010055.3	147.00	-17.61	2 21	1839 1866	21
mmu-miR-7226-5p	NM_027293.1	144.00	-26.25	2 25	4178 4201	23
mmu-miR-7226-5p	NM_027293.1	140.00	-20.27	3 25	2451 2476	22
mmu-miR-7226-5p	XM_011243391.2	143.00	-20.00	2 25	2131 2158	26
mmu-miR-7226-5p	XM_011243391.2	143.00	-24.44	2 24	4131 4156	22
mmu-miR-7226-5p	XM_011243391.2	140.00	-20.95	3 22	1371 1397	20
mmu-miR-7226-5p	NM_019759.2	158.00	-24.87	2 25	703 727	23
mmu-miR-7226-5p	NM_019759.2	141.00	-18.22	2 22	1475 1500	20
mmu-miR-7226-5p	XM_006506808.3	155.00	-27.22	2 25	2477 2503	24
mmu-miR-7226-5p	NM_007883.3	147.00	-18.37	2 21	2677 2703	20
mmu-miR-7226-5p	NM_007883.3	144.00	-18.80	2 24	5681 5704	22
mmu-miR-7226-5p	NM_007883.3	141.00	-27.04	3 18	5051 5076	15
mmu-miR-7226-5p	XM_006514873.2	146.00	-19.21	2 11	4335 4360	9
mmu-miR-7226-5p	XM_006514873.2	144.00	-18.40	2 24	2519 2545	24
mmu-miR-7226-5p	XM_006514873.2	141.00	-23.61	2 24	3512 3536	22
mmu-miR-7226-5p	NM_001160049.1	146.00	-24.28	2 25	51 72	23
mmu-miR-7226-5p	NM_001160049.1	140.00	-20.02	2 22	1071 1098	22
mmu-miR-7226-5p	NM_001160049.1	140.00	-22.62	2 22	2791 2817	21
mmu-miR-7226-5p	XM_011245824.2	143.00	-22.34	3 25	1423 1449	23
mmu-miR-7226-5p	XM_011245824.2	142.00	-24.53	2 22	649 674	21
mmu-miR-7226-5p	XM_006528026.2	147.00	-18.18	2 22	4678 4702	20
mmu-miR-7226-5p	XM_006528026.2	145.00	-18.07	2 19	2434 2460	18
mmu-miR-7226-5p	XM_006528026.2	144.00	-20.72	2 22	3086 3112	21
mmu-miR-7226-5p	XM_006528026.2	140.00	-18.07	2 24	662 685	22
mmu-miR-7226-5p	NM_133643.4	147.00	-21.99	2 18	2230 2254	16
mmu-miR-7226-5p	NM_133643.4	147.00	-16.37	3 24	3028 3053	21
mmu-miR-7226-5p	NM_133643.4	146.00	-19.05	2 21	2549 2573	19
mmu-miR-7226-5p	NM_133643.4	141.00	-22.56	2 25	7694 7719	24
mmu-miR-7226-5p	NM_133643.4	140.00	-22.23	2 22	922 951	24
mmu-miR-7226-5p	NM_133643.4	140.00	-17.27	2 9	6319 6344	7
mmu-miR-7226-5p	NM_001291145.1	158.00	-27.81	2 25	1837 1865	26
mmu-miR-7226-5p	NM_001291145.1	151.00	-20.26	2 21	1533 1559	20
mmu-miR-7226-5p	NM_001291145.1	146.00	-24.22	2 25	2353 2380	25
mmu-miR-7226-5p	NM_001291145.1	145.00	-19.12	2 22	2755 2780	20
mmu-miR-7226-5p	NM_010133.2	147.00	-22.55	2 25	2247 2273	24
mmu-miR-7226-5p	XM_006520618.2	149.00	-24.72	2 25	814 837	23
mmu-miR-7226-5p	XM_006520618.2	141.00	-18.86	2 20	1513 1537	18
mmu-miR-7226-5p	NM_015744.4	181.00	-31.90	2 23	3213 3240	23
mmu-miR-7226-5p	NM_015744.4	145.00	-26.03	2 24	60 84	22
mmu-miR-7226-5p	NM_015744.4	143.00	-23.19	3 25	1043 1069	23
mmu-miR-7226-5p	NM_015744.4	142.00	-26.98	3 25	671 695	22
mmu-miR-7226-5p	NM_015744.4	140.00	-19.30	2 19	2759 2783	17
mmu-miR-7226-5p	NM_178676.4	148.00	-23.49	2 22	647 674	22
mmu-miR-7226-5p	NM_178676.4	145.00	-19.94	3 23	2162 2188	21
mmu-miR-7226-5p	NM_010137.3	146.00	-18.44	2 20	2378 2404	19
mmu-miR-7226-5p	NM_010137.3	141.00	-20.08	2 24	4562 4593	28
mmu-miR-7226-5p	NM_010140.3	143.00	-18.54	2 23	2536 2559	21
mmu-miR-7226-5p	XM_011238916.2	155.00	-25.81	2 25	1151 1173	23
mmu-miR-7226-5p	XM_011238916.2	146.00	-21.59	3 24	2970 2996	22
mmu-miR-7226-5p	XM_011238916.2	143.00	-16.10	3 25	4217 4244	24
mmu-miR-7226-5p	XM_006511221.3	166.00	-31.12	2 23	3277 3300	21
mmu-miR-7226-5p	XM_006511221.3	150.00	-21.13	3 24	4550 4572	21
mmu-miR-7226-5p	XM_006511221.3	140.00	-19.40	2 23	2244 2268	21
mmu-miR-3473f	XM_006515980.3	149.00	-15.42	2 18	2768 2787	16
mmu-miR-3473f	NM_138955.3	141.00	-15.99	3 18	1628 1647	15
mmu-miR-3473f	XM_006540566.1	143.00	-14.08	2 17	7669 7691	18
mmu-miR-3473f	NM_009608.4	141.00	-17.82	2 19	927 947	18
mmu-miR-3473f	XM_006498086.3	155.00	-19.18	2 19	1973 1990	17
mmu-miR-3473f	NM_009645.2	140.00	-6.81	2 9	2039 2058	7
mmu-miR-3473f	NM_008012.1	149.00	-18.52	2 19	1064 1086	20
mmu-miR-3473f	NM_053080.3	142.00	-15.58	2 16	2506 2526	15
mmu-miR-3473f	NM_013468.3	150.00	-12.62	2 19	1204 1223	17
mmu-miR-3473f	NM_172790.2	147.00	-7.64	2 12	6014 6033	10
mmu-miR-3473f	NM_009675.2	140.00	-13.61	2 9	3012 3031	7

mmu-miR-3473f	NM_001080819.1	145.00	-14.35	2 16	7063 7081	14
mmu-miR-3473f	NM_007881.4	144.00	-15.56	3 18	2026 2047	17
mmu-miR-3473f	NM_013415.5	140.00	-14.42	2 18	1795 1815	17
mmu-miR-3473f	XM_006529727.2	158.00	-15.32	2 19	5085 5104	17
mmu-miR-3473f	XM_006507725.2	146.00	-10.94	2 19	414 433	17
mmu-miR-3473f	XM_006503323.3	145.00	-15.67	2 19	2620 2641	19
mmu-miR-3473f	NM_007555.4	150.00	-19.48	2 19	828 847	17
mmu-miR-3473f	XM_006524328.3	145.00	-18.06	2 19	5669 5689	18
mmu-miR-3473f	XM_006503665.3	140.00	-7.07	2 9	2605 2624	7
mmu-miR-3473f	NM_001097617.1	144.00	-13.53	2 17	246 265	15
mmu-miR-3473f	NM_027416.3	152.00	-18.82	2 14	870 891	14
mmu-miR-3473f	XM_011246479.2	141.00	-17.85	2 14	3013 3032	12
mmu-miR-3473f	NM_181315.4	148.00	-17.78	2 19	1933 1951	17
mmu-miR-3473f	NM_181315.4	142.00	-7.80	2 19	1172 1191	17
mmu-miR-3473f	XM_011245344.2	158.00	-20.81	2 16	5813 5833	15
mmu-miR-3473f	XM_011245344.2	154.00	-10.72	2 15	10243 10262	13
mmu-miR-3473f	XM_011245344.2	152.00	-18.68	2 18	11787 11807	17
mmu-miR-3473f	XM_011245344.2	148.00	-23.48	2 18	1029 1049	17
mmu-miR-3473f	XM_011245344.2	143.00	-10.62	2 18	11285 11303	16
mmu-miR-3473f	XM_011245344.2	142.00	-9.97	2 19	7825 7844	17
mmu-miR-3473f	XM_011245344.2	142.00	-14.20	2 19	9833 9852	17
mmu-miR-3473f	XM_011245344.2	141.00	-12.70	2 19	5707 5727	18
mmu-miR-3473f	XM_006532007.3	140.00	-7.22	2 9	636 655	7
mmu-miR-3473f	NM_020001.2	148.00	-19.00	2 18	255 275	17
mmu-miR-3473f	NM_001004357.2	143.00	-12.52	2 18	4495 4513	16
mmu-miR-3473f	NM_001004357.2	141.00	-12.01	2 12	4746 4764	10
mmu-miR-3473f	NM_001113515.2	143.00	-15.14	2 17	4678 4698	16
mmu-miR-3473f	NM_009930.2	146.00	-12.85	2 19	256 275	17
mmu-miR-3473f	NM_009930.2	145.00	-10.54	2 10	50 69	8
mmu-miR-3473f	NM_009930.2	141.00	-17.54	3 15	3669 3689	13
mmu-miR-3473f	XM_011240755.1	165.00	-20.93	2 14	3067 3086	12
mmu-miR-3473f	XM_011247208.1	146.00	-14.52	2 19	4619 4638	17
mmu-miR-3473f	XM_011247208.1	142.00	-9.04	2 19	5318 5337	17
mmu-miR-3473f	XM_017315376.1	156.00	-20.43	2 17	1208 1227	15
mmu-miR-3473f	XM_011248602.2	158.00	-13.77	2 19	1343 1362	17
mmu-miR-3473f	NM_001190448.1	151.00	-15.37	2 13	349 369	12
mmu-miR-3473f	NM_010055.3	148.00	-11.27	2 19	2327 2349	20
mmu-miR-3473f	NM_010055.3	140.00	-14.55	2 18	1738 1759	18
mmu-miR-3473f	XM_011243391.2	141.00	-13.03	2 18	5306 5325	16
mmu-miR-3473f	NM_001317365.1	141.00	-18.81	2 19	3747 3767	18
mmu-miR-3473f	NM_001160049.1	144.00	-13.06	2 14	3169 3190	14
mmu-miR-3473f	XM_006528026.2	148.00	-18.28	2 16	94 111	14
mmu-miR-3473f	XM_006528026.2	140.00	-11.84	2 19	4219 4237	17
mmu-miR-3473f	NM_133643.4	144.00	-11.76	2 19	7498 7516	17
mmu-miR-3473f	NM_178676.4	144.00	-9.41	2 17	2490 2509	15
mmu-miR-8094	NM_024283.3	148.00	-16.42	2 18	749 770	17
mmu-miR-8094	XM_017320219.1	148.00	-14.06	2 20	834 852	18
mmu-miR-8094	XM_006540566.1	141.00	-10.95	2 16	3954 3973	14
mmu-miR-8094	XM_006540566.1	140.00	-10.58	2 9	5593 5613	7
mmu-miR-8094	NM_001142804.1	146.00	-12.08	2 15	142 162	13
mmu-miR-8094	XM_006498086.3	151.00	-14.76	2 17	4956 4978	17
mmu-miR-8094	XM_006498086.3	145.00	-17.90	2 18	2727 2747	16
mmu-miR-8094	NM_013906.3	145.00	-16.09	2 10	2404 2424	8
mmu-miR-8094	XM_006538374.1	151.00	-14.73	2 13	2697 2718	12
mmu-miR-8094	XM_006505388.2	153.00	-14.66	2 18	6690 6710	16
mmu-miR-8094	XM_006505388.2	142.00	-14.69	2 19	3343 3363	17
mmu-miR-8094	NM_001291930.1	156.00	-18.01	2 20	1192 1210	18
mmu-miR-8094	NM_001291930.1	150.00	-17.87	2 11	2135 2155	9
mmu-miR-8094	NM_001291930.1	149.00	-12.98	2 16	1895 1914	14
mmu-miR-8094	NM_001291930.1	148.00	-18.05	2 18	3394 3415	17
mmu-miR-8094	XM_017312667.1	143.00	-21.99	3 20	5469 5489	17
mmu-miR-8094	NM_008032.3	145.00	-13.82	2 10	864 884	8
mmu-miR-8094	XM_011250176.1	146.00	-13.28	2 11	6911 6931	9
mmu-miR-8094	NM_031185.3	145.00	-12.03	2 15	2158 2179	14
mmu-miR-8094	NM_031185.3	145.00	-10.34	2 15	5010 5031	14

mmu-miR-8094	XM_006508990.2	141.00	-11.23	2 10	6297 6317	8
mmu-miR-8094	XM_006508990.2	140.00	-12.97	2 15	6246 6265	13
mmu-miR-8094	NM_013468.3	154.00	-19.80	2 19	869 889	17
mmu-miR-8094	NM_001301354.1	150.00	-16.20	2 20	221 243	20
mmu-miR-8094	NM_001080819.1	141.00	-12.12	2 10	1884 1904	8
mmu-miR-8094	NM_001080819.1	140.00	-9.55	2 9	1521 1541	7
mmu-miR-8094	NM_207231.1	142.00	-12.53	2 11	650 670	9
mmu-miR-8094	NM_207231.1	140.00	-11.61	2 9	16 36	7
mmu-miR-8094	NM_023048.5	145.00	-15.45	2 10	2438 2458	8
mmu-miR-8094	NM_025711.3	150.00	-17.53	2 19	33 50	17
mmu-miR-8094	NM_009722.3	159.00	-19.54	2 20	2595 2615	18
mmu-miR-8094	NM_009722.3	146.00	-14.94	2 13	3894 3913	11
mmu-miR-8094	NM_009722.3	141.00	-15.33	2 19	4032 4053	18
mmu-miR-8094	XM_006529727.2	141.00	-14.12	2 14	5046 5066	12
mmu-miR-8094	NM_020025.4	142.00	-10.84	2 11	759 779	9
mmu-miR-8094	NM_020025.4	140.00	-9.38	2 9	4410 4430	7
mmu-miR-8094	NM_001159407.1	149.00	-18.77	2 20	1650 1672	20
mmu-miR-8094	XM_006502291.3	160.00	-19.97	2 19	5689 5708	17
mmu-miR-8094	XM_006502291.3	148.00	-12.40	2 15	2514 2533	13
mmu-miR-8094	XM_006502291.3	146.00	-21.19	2 16	5609 5630	15
mmu-miR-8094	NM_178782.4	150.00	-16.58	2 11	1461 1481	9
mmu-miR-8094	NM_178782.4	141.00	-11.97	2 19	817 842	22
mmu-miR-8094	XM_006503323.3	140.00	-14.11	2 9	1437 1457	7
mmu-miR-8094	NM_028472.2	141.00	-17.49	3 20	565 584	17
mmu-miR-8094	NM_020508.4	145.00	-16.87	2 10	3656 3676	8
mmu-miR-8094	XM_006503665.3	140.00	-17.05	3 19	2558 2577	16
mmu-miR-8094	NM_007588.2	146.00	-14.45	2 19	230 250	17
mmu-miR-8094	NM_001190379.1	155.00	-19.46	2 18	1706 1725	16
mmu-miR-8094	XM_006526449.3	140.00	-9.69	2 9	1376 1396	7
mmu-miR-8094	NM_001310648.1	154.00	-14.18	2 20	1656 1677	19
mmu-miR-8094	NM_001310648.1	140.00	-12.37	2 9	542 562	7
mmu-miR-8094	NM_001001178.1	158.00	-17.17	2 20	2383 2404	19
mmu-miR-8094	NM_001001178.1	140.00	-9.33	2 9	2247 2267	7
mmu-miR-8094	NM_028804.1	147.00	-12.87	2 12	1469 1489	10
mmu-miR-8094	NM_001013784.1	149.00	-12.97	2 19	2346 2368	19
mmu-miR-8094	NM_025422.4	143.00	-12.30	2 19	153 173	18
mmu-miR-8094	NM_007655.3	147.00	-14.50	2 17	190 211	16
mmu-miR-8094	XM_011245344.2	148.00	-12.67	2 13	9302 9322	11
mmu-miR-8094	XM_011245344.2	146.00	-12.25	2 20	6403 6424	19
mmu-miR-8094	XM_011245344.2	140.00	-11.58	2 20	5173 5194	20
mmu-miR-8094	XM_006530131.1	155.00	-15.24	2 20	5168 5188	18
mmu-miR-8094	XM_006530131.1	149.00	-13.80	2 19	7114 7135	18
mmu-miR-8094	XM_006530131.1	148.00	-13.74	2 13	8543 8563	11
mmu-miR-8094	XM_006530131.1	141.00	-14.55	2 10	6451 6471	8
mmu-miR-8094	NM_020001.2	144.00	-13.08	2 19	356 375	17
mmu-miR-8094	NM_001004357.2	164.00	-19.15	2 19	1450 1469	17
mmu-miR-8094	NM_001004357.2	142.00	-20.59	3 20	356 377	18
mmu-miR-8094	NM_009930.2	141.00	-20.60	3 18	1483 1503	15
mmu-miR-8094	XM_011240755.1	140.00	-18.47	2 17	1844 1864	15
mmu-miR-8094	NM_001309809.2	155.00	-13.48	2 20	755 775	18
mmu-miR-8094	XM_011247208.1	152.00	-14.63	2 20	6523 6543	19
mmu-miR-8094	XM_011247208.1	142.00	-11.34	2 20	5668 5689	19
mmu-miR-8094	NM_018866.2	147.00	-15.14	2 20	759 779	18
mmu-miR-8094	XM_006517308.3	153.00	-13.74	2 20	1631 1650	18
mmu-miR-8094	NM_001190448.1	141.00	-12.95	2 10	53 73	8
mmu-miR-8094	NM_027293.1	152.00	-14.46	2 19	6280 6299	17
mmu-miR-8094	XM_011243391.2	152.00	-16.77	2 13	4255 4275	11
mmu-miR-8094	XM_011243391.2	150.00	-21.47	3 15	812 832	12
mmu-miR-8094	XM_011243391.2	141.00	-20.14	3 20	1766 1785	17
mmu-miR-8094	NM_007883.3	142.00	-10.81	2 11	4629 4649	9
mmu-miR-8094	NM_010090.2	142.00	-15.47	2 19	969 989	17
mmu-miR-8094	XM_006528026.2	146.00	-11.22	2 11	3790 3810	9
mmu-miR-8094	XM_006528026.2	141.00	-12.31	2 10	3318 3338	8
mmu-miR-8094	XM_006520618.2	148.00	-13.46	2 15	1627 1646	13
mmu-miR-8094	NM_015744.4	155.00	-14.40	2 20	1037 1057	18

mmu-miR-8094	NM_015744.4	151.00	-14.93	2 13	1843 1864	12
mmu-miR-8094	NM_015744.4	147.00	-14.45	2 12	1071 1091	10
mmu-miR-8094	NM_010137.3	142.00	-9.32	2 11	2092 2112	9
mmu-miR-8094	NM_010137.3	140.00	-12.15	2 9	1963 1983	7
mmu-miR-8094	NM_010140.3	146.00	-10.89	2 11	3294 3314	9
mmu-miR-8117	XM_011241948.2	150.00	-15.71	2 15	209 229	13
mmu-miR-8117	XM_006515980.3	151.00	-21.98	2 20	67 87	18
mmu-miR-8117	NM_138955.3	160.00	-22.65	2 18	813 834	17
mmu-miR-8117	NM_172580.1	144.00	-18.89	2 18	637 658	17
mmu-miR-8117	XM_006498086.3	140.00	-15.52	2 9	700 720	7
mmu-miR-8117	XM_006505388.2	144.00	-18.73	2 13	5697 5717	11
mmu-miR-8117	XM_017312667.1	140.00	-16.99	2 9	3855 3875	7
mmu-miR-8117	NM_153178.4	156.00	-22.35	2 17	2776 2796	15
mmu-miR-8117	NM_153178.4	151.00	-26.19	2 16	1483 1503	14
mmu-miR-8117	XM_011250176.1	144.00	-24.87	2 18	4355 4376	17
mmu-miR-8117	XM_011250176.1	142.00	-30.60	2 20	3913 3934	19
mmu-miR-8117	XM_006495613.3	145.00	-18.48	2 20	1656 1675	18
mmu-miR-8117	XM_006508990.2	143.00	-22.42	2 17	6159 6181	17
mmu-miR-8117	NM_172790.2	140.00	-19.34	2 9	1136 1156	7
mmu-miR-8117	NM_001305585.1	145.00	-21.21	2 10	634 654	8
mmu-miR-8117	NM_029823.2	149.00	-16.69	2 14	1267 1287	12
mmu-miR-8117	NM_029823.2	142.00	-16.17	2 15	1464 1484	13
mmu-miR-8117	NM_007529.2	147.00	-20.38	3 16	2302 2322	13
mmu-miR-8117	NM_007555.4	147.00	-21.02	2 20	1422 1442	18
mmu-miR-8117	NM_026979.5	144.00	-24.60	2 19	827 849	19
mmu-miR-8117	XM_011246128.2	142.00	-12.28	2 11	4109 4129	9
mmu-miR-8117	NM_007588.2	150.00	-27.94	2 16	1314 1335	15
mmu-miR-8117	XM_006520253.3	150.00	-23.84	2 20	4357 4379	20
mmu-miR-8117	NM_001001178.1	151.00	-17.19	2 20	1184 1204	18
mmu-miR-8117	NM_028804.1	148.00	-26.52	2 20	2320 2338	18
mmu-miR-8117	XM_011245344.2	140.00	-22.54	2 17	2466 2486	15
mmu-miR-8117	XM_006530131.1	141.00	-21.43	2 19	652 673	18
mmu-miR-8117	XM_006530131.1	140.00	-11.84	2 9	2543 2563	7
mmu-miR-8117	XM_011250217.1	160.00	-16.75	2 17	1507 1527	15
mmu-miR-8117	XM_006527480.3	156.00	-20.16	2 13	1257 1277	11
mmu-miR-8117	NM_009930.2	140.00	-16.83	2 9	1473 1493	7
mmu-miR-8117	XM_006532938.2	140.00	-13.20	2 9	103 123	7
mmu-miR-8117	XM_011247208.1	148.00	-22.08	2 19	3583 3606	20
mmu-miR-8117	XM_011248602.2	143.00	-16.36	2 16	1350 1370	14
mmu-miR-8117	NM_019696.2	154.00	-18.54	2 16	2215 2237	16
mmu-miR-8117	XM_011242311.2	151.00	-25.56	2 20	1471 1491	18
mmu-miR-8117	XM_011242311.2	142.00	-20.43	2 20	326 348	20
mmu-miR-8117	NM_009345.2	148.00	-19.48	2 17	1546 1566	15
mmu-miR-8117	NM_027293.1	142.00	-19.07	2 17	2989 3012	18
mmu-miR-8117	NM_027293.1	142.00	-18.75	2 17	3510 3529	15
mmu-miR-8117	XM_011243391.2	142.00	-16.02	2 11	1002 1022	9
mmu-miR-8117	NM_010133.2	153.00	-27.65	2 20	1036 1055	18
mmu-miR-8117	XM_011238916.2	141.00	-18.47	2 19	2844 2866	19
mmu-miR-206-3p	XM_006509531.3	150.00	-19.76	2 21	860 883	21
mmu-miR-206-3p	XM_006524407.3	144.00	-12.05	2 21	2398 2419	19
mmu-miR-206-3p	XM_006532962.2	152.00	-17.43	2 20	3597 3616	18
mmu-miR-206-3p	NM_178908.3	156.00	-16.98	2 18	1435 1457	17
mmu-miR-206-3p	NM_178908.3	144.00	-13.66	3 19	1611 1631	16
mmu-miR-206-3p	NM_001160262.1	141.00	-15.67	2 21	3729 3748	19
mmu-miR-206-3p	NM_010181.2	144.00	-13.94	2 18	3987 4009	17
mmu-miR-206-3p	NM_010181.2	141.00	-16.33	2 21	10140 10159	19
mmu-miR-206-3p	XM_011244488.2	149.00	-17.86	2 20	1546 1566	18
mmu-miR-206-3p	NM_145141.2	149.00	-15.97	2 18	1411 1432	16
mmu-miR-206-3p	NM_172862.3	150.00	-15.21	2 18	6791 6810	16
mmu-miR-206-3p	NM_172862.3	146.00	-19.21	2 19	3714 3735	17
mmu-miR-206-3p	NM_172862.3	146.00	-17.88	2 21	3877 3901	22
mmu-miR-206-3p	NM_172862.3	143.00	-15.88	3 21	10370 10392	19
mmu-miR-206-3p	NM_172862.3	141.00	-16.12	3 20	8749 8773	20
mmu-miR-206-3p	XM_006514444.1	140.00	-17.37	2 9	2516 2537	7
mmu-miR-206-3p	XM_006510297.3	155.00	-15.59	2 21	2483 2505	20

mmu-miR-206-3p	XM_006530353.3	154.00	-19.95	2 19	7052 7073	17
mmu-miR-206-3p	XM_006530353.3	146.00	-17.02	2 21	10992 11012	19
mmu-miR-206-3p	XM_011248576.2	147.00	-13.55	2 12	2308 2329	10
mmu-miR-206-3p	XM_006532232.3	149.00	-20.10	2 20	200 220	18
mmu-miR-206-3p	XM_011250978.2	140.00	-13.72	2 18	3298 3320	17
mmu-miR-206-3p	XM_006515096.2	144.00	-18.85	2 21	2699 2720	19
mmu-miR-206-3p	XM_006496277.3	153.00	-13.72	2 18	477 498	16
mmu-miR-206-3p	XM_006496277.3	146.00	-19.76	2 21	2700 2720	19
mmu-miR-206-3p	NM_008204.2	144.00	-16.48	2 20	1003 1022	18
mmu-miR-206-3p	NM_001162950.1	142.00	-17.91	2 16	3676 3698	15
mmu-miR-206-3p	NM_001162950.1	141.00	-11.08	2 18	3225 3246	16
mmu-miR-206-3p	XM_006505602.3	154.00	-23.24	2 21	5747 5767	19
mmu-miR-206-3p	NM_001164086.1	154.00	-13.01	2 21	6461 6481	19
mmu-miR-206-3p	NM_001327998.1	146.00	-16.91	2 21	5277 5297	19
mmu-miR-206-3p	NM_001327998.1	143.00	-16.26	2 20	317 338	18
mmu-miR-206-3p	XM_017316439.1	140.00	-15.37	2 9	2057 2078	7
mmu-miR-206-3p	XM_006516224.3	150.00	-16.22	2 21	2365 2389	22
mmu-miR-206-3p	NM_001302471.1	148.00	-21.96	2 21	3965 3986	19
mmu-miR-206-3p	NM_001289875.1	150.00	-18.40	2 20	3216 3241	22
mmu-miR-206-3p	NM_001289875.1	143.00	-19.43	2 21	165 188	21
mmu-miR-206-3p	NM_001289875.1	141.00	-15.65	2 21	2504 2523	19
mmu-miR-206-3p	NM_031180.2	140.00	-21.80	2 18	2206 2228	17
mmu-miR-206-3p	NM_173427.2	142.00	-14.74	2 15	356 377	13
mmu-miR-206-3p	XM_017320093.1	147.00	-14.98	2 21	2796 2818	20
mmu-miR-206-3p	NM_008479.2	140.00	-15.93	2 18	152 175	18
mmu-miR-206-3p	XM_017313062.1	140.00	-13.66	2 9	2201 2222	7
mmu-miR-206-3p	XM_006511102.2	140.00	-13.45	3 21	872 893	18
mmu-miR-206-3p	NM_010714.3	145.00	-14.48	2 21	4023 4045	21
mmu-miR-206-3p	NM_175271.4	140.00	-15.69	2 21	2680 2701	19
mmu-miR-206-3p	XM_006506340.3	154.00	-23.55	2 21	1958 1982	22
mmu-miR-206-3p	XM_006506340.3	152.00	-15.23	2 17	3162 3183	15
mmu-miR-206-3p	XM_006506340.3	148.00	-15.72	2 21	3127 3148	19
mmu-miR-206-3p	NM_028973.2	145.00	-17.03	2 21	300 321	20
mmu-miR-206-3p	NM_008524.2	140.00	-11.73	2 9	1048 1069	7
mmu-miR-206-3p	XM_006541232.3	148.00	-12.18	2 21	6106 6124	19
mmu-miR-206-3p	XM_006541232.3	143.00	-18.44	2 20	5874 5895	18
mmu-miR-206-3p	XM_006498872.3	164.00	-19.38	2 17	9945 9966	15
mmu-miR-206-3p	XM_006498872.3	143.00	-15.04	2 21	9427 9449	20
mmu-miR-206-3p	XM_006498872.3	142.00	-15.63	3 21	2933 2953	18
mmu-miR-206-3p	XM_006498872.3	142.00	-28.50	2 20	10347 10372	22
mmu-miR-206-3p	NM_013593.3	140.00	-14.93	2 9	495 516	7
mmu-miR-206-3p	NM_008623.5	148.00	-13.50	2 17	1912 1933	15
mmu-miR-206-3p	NM_205810.4	148.00	-19.66	2 21	2177 2198	19
mmu-miR-206-3p	NM_205810.4	141.00	-20.71	2 19	3303 3326	19
mmu-miR-206-3p	NM_001099314.1	158.00	-17.94	2 20	67 89	19
mmu-miR-206-3p	XM_006537659.2	152.00	-15.10	2 21	5835 5856	19
mmu-miR-206-3p	XM_006537659.2	148.00	-14.28	2 17	390 411	15
mmu-miR-206-3p	XM_006537659.2	142.00	-11.86	2 19	1817 1838	17
mmu-miR-206-3p	XM_006537659.2	140.00	-20.10	2 9	1854 1875	7
mmu-miR-206-3p	NM_175260.2	144.00	-18.10	2 21	7630 7651	19
mmu-miR-206-3p	NM_173437.2	140.00	-16.76	2 21	8174 8195	19
mmu-miR-206-3p	NM_011424.3	148.00	-17.85	2 21	5907 5927	20
mmu-miR-206-3p	XM_006510077.3	155.00	-19.92	2 21	612 638	24
mmu-miR-206-3p	XM_006510077.3	150.00	-15.11	2 19	307 328	17
mmu-miR-206-3p	XM_006510077.3	145.00	-11.70	2 18	583 604	16
mmu-miR-206-3p	NM_001134300.2	155.00	-18.34	2 20	2737 2758	18
mmu-miR-206-3p	NM_181547.3	140.00	-17.44	2 21	1418 1439	19
mmu-miR-206-3p	XM_006526747.3	144.00	-16.93	2 17	2487 2508	15
mmu-miR-206-3p	NM_001282961.1	149.00	-19.04	3 21	4028 4049	19
mmu-miR-206-3p	NM_001282961.1	143.00	-16.40	2 20	774 795	18
mmu-miR-206-3p	NM_001282961.1	140.00	-13.97	2 9	1985 2006	7
mmu-miR-206-3p	NM_011854.2	140.00	-9.03	2 9	2333 2354	7
mmu-miR-206-3p	NM_008760.4	148.00	-17.17	3 19	898 918	16
mmu-miR-206-3p	NM_008760.4	140.00	-19.14	2 21	1542 1563	19
mmu-miR-206-3p	XM_006516928.2	158.00	-16.96	2 21	3951 3971	19

mmu-miR-206-3p	XM_006516928.2	152.00	-17.52	2 21	3872 3893	19
mmu-miR-214-3p	XM_006509531.3	143.00	-17.08	3 21	3097 3120	20
mmu-miR-214-3p	XM_006524407.3	170.00	-27.04	2 20	1563 1585	19
mmu-miR-214-3p	XM_006524407.3	143.00	-19.66	3 20	157 178	17
mmu-miR-214-3p	NM_177743.5	150.00	-19.08	2 21	1671 1691	19
mmu-miR-214-3p	NM_177743.5	143.00	-15.34	2 16	1775 1796	14
mmu-miR-214-3p	NM_177743.5	140.00	-17.96	2 21	795 816	19
mmu-miR-214-3p	NM_177743.5	140.00	-18.26	2 9	2005 2026	7
mmu-miR-214-3p	XM_017319626.1	150.00	-19.43	2 11	1532 1553	9
mmu-miR-214-3p	XM_006532962.2	151.00	-21.89	2 20	1054 1073	18
mmu-miR-214-3p	NM_178908.3	158.00	-18.13	2 19	650 671	17
mmu-miR-214-3p	NM_001160262.1	162.00	-28.40	2 21	1910 1930	19
mmu-miR-214-3p	XM_006520437.2	164.00	-27.91	2 21	603 624	19
mmu-miR-214-3p	NM_010181.2	151.00	-20.20	2 21	4898 4921	21
mmu-miR-214-3p	NM_010181.2	146.00	-22.11	2 20	2775 2797	19
mmu-miR-214-3p	NM_010181.2	145.00	-19.68	2 10	3157 3178	8
mmu-miR-214-3p	NM_010181.2	144.00	-16.45	2 21	1016 1038	21
mmu-miR-214-3p	XM_011244488.2	159.00	-27.76	2 21	475 498	21
mmu-miR-214-3p	NM_145141.2	158.00	-24.66	2 17	76 96	15
mmu-miR-214-3p	NM_145141.2	140.00	-19.38	2 19	919 939	17
mmu-miR-214-3p	XM_006527803.1	142.00	-21.71	3 21	800 825	22
mmu-miR-214-3p	NM_001081185.1	150.00	-19.46	2 11	6268 6289	9
mmu-miR-214-3p	NM_001081185.1	149.00	-17.40	2 14	6950 6971	12
mmu-miR-214-3p	XM_006496949.3	145.00	-19.64	2 10	1046 1067	8
mmu-miR-214-3p	NM_172862.3	158.00	-23.25	2 15	1403 1424	13
mmu-miR-214-3p	NM_172862.3	158.00	-19.88	2 21	3061 3081	19
mmu-miR-214-3p	NM_172862.3	148.00	-19.16	2 15	9930 9950	13
mmu-miR-214-3p	NM_172862.3	148.00	-24.87	3 21	11611 11629	18
mmu-miR-214-3p	NM_172862.3	146.00	-21.60	2 20	2032 2054	19
mmu-miR-214-3p	NM_172862.3	140.00	-13.92	2 9	7970 7991	7
mmu-miR-214-3p	XM_006533539.3	151.00	-21.99	2 20	1183 1206	21
mmu-miR-214-3p	XM_006533539.3	151.00	-18.41	2 16	2676 2697	14
mmu-miR-214-3p	XM_006533539.3	146.00	-21.75	2 16	385 407	15
mmu-miR-214-3p	XM_006533539.3	140.00	-15.68	2 9	3698 3719	7
mmu-miR-214-3p	XM_006533539.3	140.00	-13.18	2 9	6170 6191	7
mmu-miR-214-3p	NM_008067.4	154.00	-17.24	2 21	1589 1609	19
mmu-miR-214-3p	NM_001033445.2	148.00	-20.78	2 21	429 448	19
mmu-miR-214-3p	NM_001033445.2	142.00	-13.56	2 11	511 532	9
mmu-miR-214-3p	NM_001033445.2	142.00	-22.87	3 21	2134 2154	18
mmu-miR-214-3p	NM_145741.2	156.00	-24.35	2 21	360 379	19
mmu-miR-214-3p	NM_145741.2	140.00	-14.82	2 9	1842 1863	7
mmu-miR-214-3p	NM_010279.3	160.00	-21.43	2 21	55 76	19
mmu-miR-214-3p	NM_010279.3	143.00	-16.79	2 20	4319 4340	18
mmu-miR-214-3p	NM_010279.3	140.00	-22.05	2 19	1808 1828	17
mmu-miR-214-3p	XM_006510297.3	146.00	-16.30	2 21	2127 2147	19
mmu-miR-214-3p	XM_006510297.3	143.00	-26.85	2 21	201 224	21
mmu-miR-214-3p	XM_006510297.3	140.00	-14.00	2 9	124 145	7
mmu-miR-214-3p	XM_006530353.3	162.00	-26.44	2 19	3268 3289	17
mmu-miR-214-3p	XM_006530353.3	160.00	-23.37	2 21	915 936	19
mmu-miR-214-3p	XM_006530353.3	156.00	-27.72	2 18	12964 12987	18
mmu-miR-214-3p	XM_006530353.3	155.00	-23.57	2 21	13112 13137	23
mmu-miR-214-3p	XM_006530353.3	151.00	-23.01	2 20	4368 4389	18
mmu-miR-214-3p	XM_006530353.3	150.00	-22.66	2 20	1210 1234	21
mmu-miR-214-3p	XM_006530353.3	141.00	-15.64	2 20	12604 12624	18
mmu-miR-214-3p	XM_006530353.3	140.00	-13.72	2 9	1380 1401	7
mmu-miR-214-3p	XM_006530353.3	140.00	-15.01	2 9	8712 8733	7
mmu-miR-214-3p	XM_006530353.3	140.00	-15.85	2 9	13310 13331	7
mmu-miR-214-3p	NM_001024731.2	165.00	-27.65	2 21	28 49	20
mmu-miR-214-3p	XM_011248576.2	154.00	-26.42	3 19	7984 8005	16
mmu-miR-214-3p	XM_011248576.2	146.00	-19.31	2 19	1575 1594	17
mmu-miR-214-3p	XM_011248576.2	146.00	-21.13	2 20	6271 6293	19
mmu-miR-214-3p	XM_011248576.2	142.00	-15.88	2 11	7962 7983	9
mmu-miR-214-3p	XM_011238891.1	142.00	-21.96	3 21	798 818	18
mmu-miR-214-3p	NM_001198955.1	164.00	-24.13	2 19	882 902	17
mmu-miR-214-3p	NM_001198955.1	140.00	-19.62	2 9	848 869	7

mmu-miR-214-3p	XM_006532232.3	156.00	-19.16	2 19	182 202	17
mmu-miR-214-3p	XM_006532232.3	145.00	-16.14	2 10	238 259	8
mmu-miR-214-3p	XM_011250978.2	152.00	-18.99	3 21	3356 3377	18
mmu-miR-214-3p	XM_011250978.2	148.00	-18.71	3 21	3710 3731	18
mmu-miR-214-3p	XM_006515096.2	156.00	-21.92	2 20	2341 2362	19
mmu-miR-214-3p	XM_006515096.2	148.00	-28.22	2 21	7696 7715	19
mmu-miR-214-3p	XM_006515096.2	147.00	-20.66	2 21	1817 1839	20
mmu-miR-214-3p	XM_006515096.2	145.00	-16.76	2 15	3452 3474	14
mmu-miR-214-3p	XM_006515096.2	144.00	-17.10	2 21	6045 6066	19
mmu-miR-214-3p	XM_006515096.2	141.00	-26.81	2 20	1483 1503	18
mmu-miR-214-3p	XM_006515096.2	141.00	-24.01	2 18	2258 2279	16
mmu-miR-214-3p	XM_006515096.2	140.00	-16.41	2 9	6231 6252	7
mmu-miR-214-3p	XM_006526069.2	151.00	-20.43	2 21	851 873	20
mmu-miR-214-3p	XM_006526069.2	147.00	-19.91	2 21	1715 1740	23
mmu-miR-214-3p	XM_006526069.2	147.00	-19.33	2 12	3811 3832	10
mmu-miR-214-3p	XM_006526069.2	145.00	-20.47	2 10	4239 4260	8
mmu-miR-214-3p	NM_008185.3	148.00	-20.50	2 20	740 759	18
mmu-miR-214-3p	NM_008185.3	140.00	-22.32	2 18	96 119	18
mmu-miR-214-3p	NM_008204.2	154.00	-17.10	2 15	28 49	13
mmu-miR-214-3p	XM_006529759.1	158.00	-20.64	2 16	87 109	15
mmu-miR-214-3p	XM_006529759.1	155.00	-24.47	2 12	1405 1426	10
mmu-miR-214-3p	NM_001162950.1	149.00	-21.16	2 19	1825 1847	18
mmu-miR-214-3p	NM_001164086.1	140.00	-17.02	3 19	5734 5754	16
mmu-miR-214-3p	NM_001164086.1	140.00	-21.70	2 19	10544 10567	19
mmu-miR-214-3p	NM_008277.2	159.00	-23.56	2 21	1317 1339	20
mmu-miR-214-3p	NM_001327998.1	145.00	-19.09	2 10	136 157	8
mmu-miR-214-3p	NM_001327998.1	142.00	-17.93	2 21	4071 4091	19
mmu-miR-214-3p	NM_013558.2	140.00	-14.15	2 9	1527 1548	7
mmu-miR-214-3p	NM_013560.2	145.00	-19.24	2 10	767 788	8
mmu-miR-214-3p	NM_013560.2	144.00	-15.14	2 13	844 865	11
mmu-miR-214-3p	XM_006539957.3	151.00	-25.00	2 19	413 436	20
mmu-miR-214-3p	XM_006539957.3	146.00	-21.89	2 19	505 526	17
mmu-miR-214-3p	NM_133871.2	148.00	-26.14	2 21	1496 1517	19
mmu-miR-214-3p	NM_133871.2	146.00	-23.06	2 20	1526 1550	21
mmu-miR-214-3p	XM_006511311.2	157.00	-26.78	2 21	107 129	21
mmu-miR-214-3p	XM_006511311.2	152.00	-19.54	2 13	3109 3130	11
mmu-miR-214-3p	XM_006511311.2	148.00	-20.04	2 17	225 246	15
mmu-miR-214-3p	XM_006511311.2	142.00	-20.31	3 21	3702 3722	18
mmu-miR-214-3p	XM_006511311.2	141.00	-21.65	2 20	2980 3000	18
mmu-miR-214-3p	XM_006511311.2	141.00	-21.31	2 15	5246 5269	15
mmu-miR-214-3p	NM_023670.3	162.00	-29.19	2 21	1063 1087	22
mmu-miR-214-3p	XM_006523711.3	156.00	-24.42	2 19	1106 1126	17
mmu-miR-214-3p	XM_006523711.3	142.00	-23.48	2 21	840 863	21
mmu-miR-214-3p	XM_006523711.3	141.00	-20.11	3 21	2055 2076	19
mmu-miR-214-3p	XM_006523711.3	140.00	-14.42	2 9	2282 2303	7
mmu-miR-214-3p	NM_001190325.1	150.00	-19.33	2 16	694 716	15
mmu-miR-214-3p	NM_001190325.1	146.00	-15.52	2 15	142 163	13
mmu-miR-214-3p	NM_001159424.2	145.00	-20.24	2 20	259 282	20
mmu-miR-214-3p	XM_017316439.1	148.00	-16.31	2 19	2456 2476	17
mmu-miR-214-3p	XM_017316439.1	145.00	-15.79	2 15	2756 2779	15
mmu-miR-214-3p	XM_017316439.1	144.00	-20.50	2 19	1733 1753	17
mmu-miR-214-3p	XM_006496593.3	149.00	-20.67	2 20	263 283	18
mmu-miR-214-3p	XM_006496593.3	147.00	-21.14	2 21	972 994	20
mmu-miR-214-3p	XM_006496593.3	145.00	-16.95	2 16	5723 5743	14
mmu-miR-214-3p	XM_006496593.3	140.00	-20.36	2 21	907 928	19
mmu-miR-214-3p	XM_006516224.3	153.00	-21.99	2 18	371 392	16
mmu-miR-214-3p	XM_006516224.3	153.00	-17.49	2 20	429 452	20
mmu-miR-214-3p	XM_006516224.3	150.00	-22.56	2 19	1056 1074	17
mmu-miR-214-3p	XM_006516224.3	150.00	-17.84	2 21	3162 3185	21
mmu-miR-214-3p	XM_006516224.3	148.00	-26.09	3 21	2480 2501	18
mmu-miR-214-3p	NM_009349.3	157.00	-21.56	2 20	552 572	18
mmu-miR-214-3p	XM_006528842.1	171.00	-28.00	2 21	5376 5400	22
mmu-miR-214-3p	XM_006528842.1	147.00	-16.39	2 12	342 363	10
mmu-miR-214-3p	XM_006528842.1	143.00	-27.65	2 21	2722 2746	22
mmu-miR-214-3p	NM_001161541.1	155.00	-26.27	2 18	1956 1976	16

mmu-miR-214-3p	NM_001161541.1	153.00	-25.09	2 21	2023 2044	20
mmu-miR-214-3p	NM_001161541.1	147.00	-19.42	2 20	2009 2030	18
mmu-miR-214-3p	NM_001302471.1	154.00	-21.59	2 15	341 362	13
mmu-miR-214-3p	NM_001302471.1	142.00	-23.61	2 21	4628 4652	22
mmu-miR-214-3p	XM_006518921.3	142.00	-16.56	2 21	289 309	19
mmu-miR-214-3p	NM_001289875.1	141.00	-16.46	2 14	2975 2996	12
mmu-miR-214-3p	NM_008423.2	162.00	-25.52	2 20	1661 1683	19
mmu-miR-214-3p	NM_008423.2	159.00	-25.47	2 16	2877 2898	14
mmu-miR-214-3p	NM_008423.2	150.00	-18.64	2 16	3449 3471	15
mmu-miR-214-3p	NM_008423.2	147.00	-17.23	2 12	808 829	10
mmu-miR-214-3p	NM_008423.2	142.00	-13.26	2 11	1148 1169	9
mmu-miR-214-3p	NM_008423.2	142.00	-24.02	2 19	2713 2731	17
mmu-miR-214-3p	NM_008423.2	141.00	-19.15	3 20	2587 2610	19
mmu-miR-214-3p	NM_021342.1	147.00	-28.44	3 20	2325 2346	17
mmu-miR-214-3p	NM_010608.2	147.00	-20.16	2 12	547 568	10
mmu-miR-214-3p	NM_010608.2	143.00	-16.86	2 19	187 208	18
mmu-miR-214-3p	XM_006497892.3	168.00	-32.17	2 21	5285 5306	19
mmu-miR-214-3p	XM_006497892.3	156.00	-27.11	2 19	833 856	19
mmu-miR-214-3p	XM_006497892.3	152.00	-23.19	2 19	386 406	17
mmu-miR-214-3p	XM_006497892.3	152.00	-23.81	2 19	2177 2203	22
mmu-miR-214-3p	XM_006497892.3	148.00	-16.98	2 21	904 925	19
mmu-miR-214-3p	XM_006497892.3	142.00	-20.74	2 21	2421 2441	19
mmu-miR-214-3p	XM_006497892.3	140.00	-15.05	2 9	1597 1618	7
mmu-miR-214-3p	XM_006497892.3	140.00	-13.19	2 9	1822 1843	7
mmu-miR-214-3p	XM_011248919.2	152.00	-15.37	2 21	1839 1860	19
mmu-miR-214-3p	NM_031180.2	149.00	-26.66	2 21	121 143	21
mmu-miR-214-3p	NM_173427.2	154.00	-23.61	2 20	5227 5249	19
mmu-miR-214-3p	NM_173427.2	143.00	-18.25	2 21	88 111	21
mmu-miR-214-3p	NM_173427.2	141.00	-20.96	2 20	2302 2322	18
mmu-miR-214-3p	NM_001081667.2	163.00	-28.70	2 20	674 692	18
mmu-miR-214-3p	NM_001081667.2	157.00	-21.99	2 20	233 253	18
mmu-miR-214-3p	XM_006512296.3	149.00	-22.64	2 20	1308 1331	20
mmu-miR-214-3p	XM_006512296.3	142.00	-17.95	2 20	1053 1073	19
mmu-miR-214-3p	NM_029274.2	145.00	-22.22	2 20	2061 2084	20
mmu-miR-214-3p	NM_029274.2	145.00	-18.09	2 10	5971 5992	8
mmu-miR-214-3p	NM_029274.2	144.00	-24.07	2 13	3625 3646	11
mmu-miR-214-3p	NM_029274.2	142.00	-21.45	2 16	4526 4548	15
mmu-miR-214-3p	XM_017316676.1	154.00	-21.36	2 19	4446 4465	17
mmu-miR-214-3p	XM_017316676.1	152.00	-17.43	2 13	10621 10642	11
mmu-miR-214-3p	XM_017316676.1	151.00	-19.38	2 13	5323 5345	12
mmu-miR-214-3p	XM_017316676.1	150.00	-28.09	2 19	18563 18584	17
mmu-miR-214-3p	XM_017316676.1	149.00	-20.96	2 20	15237 15257	18
mmu-miR-214-3p	XM_017316676.1	149.00	-18.57	2 20	15392 15412	18
mmu-miR-214-3p	XM_017316676.1	149.00	-18.52	2 20	17430 17450	18
mmu-miR-214-3p	XM_017316676.1	147.00	-19.52	2 20	272 293	18
mmu-miR-214-3p	XM_017316676.1	147.00	-18.90	2 12	5261 5282	10
mmu-miR-214-3p	XM_017316676.1	147.00	-15.79	2 20	8831 8852	18
mmu-miR-214-3p	XM_017316676.1	147.00	-15.45	2 16	14440 14461	14
mmu-miR-214-3p	XM_017316676.1	146.00	-17.88	2 20	6484 6506	19
mmu-miR-214-3p	XM_017316676.1	145.00	-18.61	2 16	13494 13514	14
mmu-miR-214-3p	XM_017316676.1	145.00	-20.29	2 10	18932 18953	8
mmu-miR-214-3p	XM_017316676.1	144.00	-18.39	2 18	12825 12847	17
mmu-miR-214-3p	XM_017316676.1	142.00	-14.57	2 11	3928 3949	9
mmu-miR-214-3p	XM_017316676.1	141.00	-21.98	2 14	19204 19225	12
mmu-miR-214-3p	XM_017316676.1	140.00	-21.77	2 21	984 1005	19
mmu-miR-214-3p	XM_017316676.1	140.00	-15.08	2 9	5470 5491	7
mmu-miR-214-3p	XM_017316676.1	140.00	-16.72	2 9	6527 6548	7
mmu-miR-214-3p	XM_017316676.1	140.00	-16.65	2 9	9490 9511	7
mmu-miR-214-3p	NM_146063.1	156.00	-22.28	2 21	453 474	19
mmu-miR-214-3p	XM_017315313.1	142.00	-19.70	2 20	499 522	20
mmu-miR-214-3p	NM_008479.2	143.00	-14.62	2 18	111 131	16
mmu-miR-214-3p	XM_017313062.1	161.00	-25.22	2 21	2743 2764	20
mmu-miR-214-3p	XM_017313062.1	153.00	-21.27	2 21	592 614	21
mmu-miR-214-3p	XM_006511102.2	147.00	-19.90	2 21	318 340	20
mmu-miR-214-3p	XM_006511102.2	142.00	-14.55	2 16	573 595	15

mmu-miR-214-3p	NM_001302765.1	145.00	-15.94	2 10	33 54	8
mmu-miR-214-3p	XM_006527221.3	152.00	-15.88	2 21	727 748	19
mmu-miR-214-3p	NM_010714.3	141.00	-28.65	2 20	288 312	21
mmu-miR-214-3p	XM_006519969.3	150.00	-23.19	2 19	560 581	17
mmu-miR-214-3p	XM_006519969.3	143.00	-26.39	2 21	3987 4010	21
mmu-miR-214-3p	NM_144799.2	147.00	-19.13	2 12	372 393	10
mmu-miR-214-3p	NM_053098.2	140.00	-25.88	3 21	39 60	18
mmu-miR-214-3p	XM_006506340.3	154.00	-16.83	2 21	1992 2012	19
mmu-miR-214-3p	XM_006506340.3	148.00	-23.40	2 21	1015 1034	19
mmu-miR-214-3p	XM_006506340.3	147.00	-19.53	2 21	886 908	20
mmu-miR-214-3p	XM_006506340.3	145.00	-15.41	2 15	1168 1190	14
mmu-miR-214-3p	XM_006506340.3	140.00	-17.05	2 9	1145 1166	7
mmu-miR-214-3p	NM_028973.2	153.00	-26.73	2 18	1746 1767	16
mmu-miR-214-3p	NM_028973.2	153.00	-27.28	2 21	3952 3973	20
mmu-miR-214-3p	NM_028973.2	145.00	-20.71	2 15	5200 5222	14
mmu-miR-214-3p	NM_028973.2	142.00	-16.00	2 19	2653 2674	17
mmu-miR-214-3p	NM_028973.2	141.00	-18.41	2 18	2475 2496	16
mmu-miR-214-3p	NM_028973.2	141.00	-25.01	3 21	3365 3390	22
mmu-miR-214-3p	NM_028973.2	140.00	-18.65	2 9	466 487	7
mmu-miR-214-3p	NM_028838.2	143.00	-23.25	2 21	838 860	20
mmu-miR-214-3p	NM_028838.2	142.00	-17.53	2 11	935 956	9
mmu-miR-214-3p	XM_006506086.2	147.00	-15.75	2 21	456 478	20
mmu-miR-214-3p	NM_008524.2	150.00	-18.05	2 11	990 1011	9
mmu-miR-214-3p	XM_006541232.3	153.00	-22.36	2 21	2730 2755	23
mmu-miR-214-3p	XM_006541232.3	146.00	-19.19	2 19	2694 2713	17
mmu-miR-214-3p	XM_006541232.3	145.00	-18.81	3 20	2364 2384	17
mmu-miR-214-3p	XM_006541232.3	144.00	-20.99	2 18	5511 5533	17
mmu-miR-214-3p	XM_006541232.3	143.00	-16.78	3 21	521 544	20
mmu-miR-214-3p	XM_006498872.3	156.00	-24.52	2 20	8863 8888	22
mmu-miR-214-3p	XM_006498872.3	150.00	-17.19	2 19	10202 10223	17
mmu-miR-214-3p	XM_006498872.3	143.00	-19.05	2 21	2279 2298	20
mmu-miR-214-3p	XM_006498872.3	140.00	-12.63	2 9	4536 4557	7
mmu-miR-214-3p	XM_006532410.3	156.00	-25.24	2 21	4406 4425	19
mmu-miR-214-3p	XM_006532410.3	155.00	-17.05	2 18	6010 6030	16
mmu-miR-214-3p	XM_006532410.3	149.00	-20.00	2 20	4683 4709	23
mmu-miR-214-3p	XM_006532410.3	144.00	-18.16	2 21	1115 1136	19
mmu-miR-214-3p	NM_001290273.1	140.00	-14.72	2 9	565 586	7
mmu-miR-214-3p	XM_006520023.2	143.00	-21.06	2 21	287 309	20
mmu-miR-214-3p	XM_006520023.2	140.00	-25.33	2 21	2349 2370	19
mmu-miR-214-3p	NM_001252563.1	144.00	-16.16	2 19	1766 1786	17
mmu-miR-214-3p	NM_001252563.1	140.00	-16.09	2 9	102 123	7
mmu-miR-214-3p	XM_011243266.1	158.00	-21.15	2 19	159 180	17
mmu-miR-214-3p	XM_011243266.1	149.00	-23.49	2 21	2657 2679	21
mmu-miR-214-3p	XM_011243266.1	146.00	-19.98	2 19	2313 2334	17
mmu-miR-214-3p	NM_001270475.1	149.00	-20.33	2 20	6200 6224	21
mmu-miR-214-3p	NM_001270475.1	146.00	-21.08	2 19	7630 7651	17
mmu-miR-214-3p	NM_001270475.1	143.00	-18.18	2 20	6574 6595	18
mmu-miR-214-3p	NM_001270475.1	142.00	-14.74	2 11	4957 4978	9
mmu-miR-214-3p	NM_001270475.1	141.00	-18.88	2 14	7434 7455	12
mmu-miR-214-3p	NM_001270475.1	140.00	-15.20	2 9	1316 1337	7
mmu-miR-214-3p	NM_001270475.1	140.00	-19.32	3 19	8336 8356	16
mmu-miR-214-3p	NM_001290512.1	155.00	-20.87	2 17	2942 2964	16
mmu-miR-214-3p	NM_001290512.1	154.00	-19.92	2 20	1370 1392	19
mmu-miR-214-3p	NM_177595.4	140.00	-13.38	2 9	154 175	7
mmu-miR-214-3p	NM_001320077.1	147.00	-18.55	2 21	1740 1766	24
mmu-miR-214-3p	NM_010809.2	153.00	-21.62	2 20	87 107	18
mmu-miR-214-3p	XM_006504613.3	146.00	-19.89	2 21	791 811	19
mmu-miR-214-3p	NM_008623.5	140.00	-20.48	3 19	201 221	16
mmu-miR-214-3p	NM_001005423.2	147.00	-20.21	2 12	211 232	10
mmu-miR-214-3p	NM_001099314.1	151.00	-27.72	2 21	425 447	20
mmu-miR-214-3p	NM_001099314.1	151.00	-16.73	2 18	475 495	16
mmu-miR-214-3p	XM_006537659.2	157.00	-21.77	2 21	2058 2080	21
mmu-miR-214-3p	XM_006537659.2	150.00	-18.14	2 11	2957 2978	9
mmu-miR-214-3p	XM_006529753.2	145.00	-19.54	2 21	1537 1558	20
mmu-miR-214-3p	NM_175260.2	142.00	-17.01	2 17	4168 4188	15

mmu-miR-214-3p	NM_175260.2	140.00	-15.65	3	17	7696	7717	14
mmu-miR-214-3p	NM_001039545.2	169.00	-27.47	2	20	4153	4176	20
mmu-miR-214-3p	NM_001039545.2	140.00	-16.73	2	9	795	816	7
mmu-miR-214-3p	NM_001039545.2	140.00	-12.72	2	9	2646	2667	7
mmu-miR-214-3p	NM_001039545.2	140.00	-14.45	2	9	5169	5190	7
mmu-miR-214-3p	XM_006532412.1	169.00	-27.47	2	20	4125	4148	20
mmu-miR-214-3p	XM_006532412.1	140.00	-14.20	2	9	779	800	7
mmu-miR-214-3p	XM_006532412.1	140.00	-14.87	2	9	2618	2639	7
mmu-miR-214-3p	XM_017315841.1	166.00	-23.87	2	20	4171	4193	19
mmu-miR-214-3p	XM_017315841.1	152.00	-17.47	2	13	2786	2807	11
mmu-miR-214-3p	XM_017315841.1	147.00	-18.11	2	12	2453	2474	10
mmu-miR-214-3p	XM_017315841.1	142.00	-14.09	2	11	2579	2600	9
mmu-miR-214-3p	XM_017315841.1	141.00	-18.83	2	20	5094	5114	18
mmu-miR-214-3p	XM_017315841.1	140.00	-13.70	2	9	5186	5207	7
mmu-miR-214-3p	NM_010859.2	149.00	-21.14	2	20	801	824	20
mmu-miR-214-3p	NM_010859.2	142.00	-18.09	2	11	251	272	9
mmu-miR-214-3p	XM_017320223.1	159.00	-24.28	2	19	4618	4637	17
mmu-miR-214-3p	XM_006501829.3	140.00	-14.42	2	9	142	163	7
mmu-miR-214-3p	XM_017317978.1	140.00	-18.23	2	9	1034	1055	7
mmu-miR-214-3p	NM_173437.2	146.00	-20.49	2	21	4061	4086	23
mmu-miR-214-3p	XM_006541297.3	154.00	-18.36	2	19	4936	4957	17
mmu-miR-214-3p	XM_006541297.3	146.00	-15.06	2	20	6818	6840	19
mmu-miR-214-3p	NM_011424.3	141.00	-17.83	2	20	677	697	18
mmu-miR-214-3p	NM_011424.3	141.00	-16.07	2	20	2670	2690	18
mmu-miR-214-3p	NM_011424.3	140.00	-15.59	2	9	658	679	7
mmu-miR-214-3p	XM_006529456.3	159.00	-25.65	2	21	761	783	20
mmu-miR-214-3p	XM_006529456.3	154.00	-19.23	2	18	1280	1301	17
mmu-miR-214-3p	XM_006529456.3	147.00	-21.12	2	16	2603	2624	14
mmu-miR-214-3p	XM_006529456.3	143.00	-30.61	2	21	998	1020	20
mmu-miR-214-3p	XM_006529456.3	140.00	-14.55	2	9	186	207	7
mmu-miR-214-3p	XM_006510077.3	150.00	-20.54	2	11	2425	2446	9
mmu-miR-214-3p	XM_006510077.3	141.00	-19.27	3	15	2364	2386	13
mmu-miR-214-3p	NM_001134300.2	149.00	-18.38	2	14	2237	2258	12
mmu-miR-214-3p	NM_001134300.2	148.00	-19.36	2	20	2183	2205	20
mmu-miR-214-3p	NM_001134300.2	140.00	-17.97	2	9	3227	3248	7
mmu-miR-214-3p	NM_001109985.1	141.00	-16.03	2	14	1473	1494	12
mmu-miR-214-3p	NM_001109985.1	140.00	-13.63	2	9	2666	2687	7
mmu-miR-214-3p	NM_181547.3	144.00	-16.06	2	13	1538	1559	11
mmu-miR-214-3p	NM_001029877.3	163.00	-23.22	2	20	7308	7329	18
mmu-miR-214-3p	NM_001029877.3	149.00	-16.33	2	14	2391	2412	12
mmu-miR-214-3p	NM_001029877.3	140.00	-16.56	2	9	1003	1024	7
mmu-miR-214-3p	XM_006526747.3	156.00	-25.29	2	21	3115	3134	19
mmu-miR-214-3p	XM_006526747.3	141.00	-15.60	2	16	216	236	14
mmu-miR-214-3p	NM_001077403.1	147.00	-20.79	2	18	3506	3526	16
mmu-miR-214-3p	NM_198190.1	157.00	-16.92	2	19	391	413	18
mmu-miR-214-3p	NM_133500.2	173.00	-30.72	2	20	1632	1652	18
mmu-miR-214-3p	NM_133500.2	164.00	-27.54	2	20	54	75	19
mmu-miR-214-3p	NM_133500.2	164.00	-25.51	2	19	1597	1620	19
mmu-miR-214-3p	NM_133500.2	157.00	-29.98	2	21	807	831	22
mmu-miR-214-3p	NM_001282961.1	155.00	-22.86	2	16	671	692	14
mmu-miR-214-3p	NM_001282961.1	155.00	-19.63	2	16	1949	1970	14
mmu-miR-214-3p	NM_001282961.1	146.00	-26.95	2	21	2667	2693	24
mmu-miR-214-3p	NM_145210.2	143.00	-17.31	2	13	986	1008	12
mmu-miR-214-3p	XM_006530325.3	161.00	-24.66	2	21	363	384	20
mmu-miR-214-3p	XM_006530325.3	144.00	-23.76	3	21	3510	3531	18
mmu-miR-214-3p	NM_145226.2	141.00	-21.19	3	19	1812	1834	17
mmu-miR-214-3p	NM_145226.2	140.00	-13.19	2	9	627	648	7
mmu-miR-214-3p	XM_006530294.3	152.00	-19.12	2	13	1484	1505	11
mmu-miR-214-3p	NM_011854.2	164.00	-26.14	2	18	1712	1734	17
mmu-miR-214-3p	NM_001310636.1	142.00	-17.85	3	21	736	756	18
mmu-miR-214-3p	NM_008760.4	151.00	-29.87	3	21	404	426	19
mmu-miR-214-3p	NM_008760.4	147.00	-23.68	2	20	104	125	18
mmu-miR-214-3p	NM_011010.2	161.00	-26.78	2	20	1416	1439	20
mmu-miR-214-3p	NM_011010.2	149.00	-19.62	2	16	914	934	14
mmu-miR-214-3p	NM_001286743.1	145.00	-15.94	2	10	614	635	8

mmu-miR-214-3p	NM_001286743.1	142.00	-20.67	2 21	1044 1067	21
mmu-miR-214-3p	XM_017322026.1	151.00	-23.59	3 21	920 942	19
mmu-miR-378a-5p	XM_006509531.3	147.00	-22.01	2 16	1598 1619	14
mmu-miR-378a-5p	NM_177743.5	151.00	-24.70	2 21	470 492	20
mmu-miR-378a-5p	NM_177743.5	143.00	-24.70	3 21	867 889	19
mmu-miR-378a-5p	XM_017319626.1	146.00	-24.99	2 21	2247 2267	19
mmu-miR-378a-5p	XM_006520437.2	140.00	-22.89	2 21	1536 1557	19
mmu-miR-378a-5p	NM_010181.2	145.00	-19.06	2 21	5042 5068	24
mmu-miR-378a-5p	XM_006502769.3	143.00	-14.26	2 21	355 379	22
mmu-miR-378a-5p	NM_001081185.1	151.00	-31.69	2 21	3278 3300	20
mmu-miR-378a-5p	NM_001081185.1	148.00	-26.89	2 19	4665 4685	17
mmu-miR-378a-5p	NM_001081185.1	140.00	-20.58	2 18	7948 7971	18
mmu-miR-378a-5p	NM_172862.3	147.00	-19.28	2 12	9393 9414	10
mmu-miR-378a-5p	XM_006510297.3	150.00	-21.03	2 20	3477 3500	20
mmu-miR-378a-5p	XM_006530353.3	155.00	-18.27	2 21	2885 2907	20
mmu-miR-378a-5p	XM_006530353.3	144.00	-17.66	2 21	4362 4382	20
mmu-miR-378a-5p	XM_011238891.1	140.00	-19.07	2 20	22 41	18
mmu-miR-378a-5p	XM_006532232.3	161.00	-24.33	2 20	2311 2331	18
mmu-miR-378a-5p	XM_006515096.2	140.00	-14.34	2 9	3198 3219	7
mmu-miR-378a-5p	XM_006526069.2	153.00	-18.54	2 21	1083 1102	19
mmu-miR-378a-5p	XM_006496277.3	148.00	-15.20	2 17	554 575	15
mmu-miR-378a-5p	NM_008204.2	156.00	-28.96	2 17	982 1003	15
mmu-miR-378a-5p	NM_175520.4	141.00	-20.71	2 18	1683 1704	16
mmu-miR-378a-5p	NM_001162950.1	141.00	-27.83	2 20	998 1022	21
mmu-miR-378a-5p	XM_006505602.3	146.00	-20.14	2 19	4306 4327	17
mmu-miR-378a-5p	XM_006505602.3	141.00	-17.69	2 10	8236 8257	8
mmu-miR-378a-5p	NM_001164086.1	158.00	-18.59	2 15	6505 6526	13
mmu-miR-378a-5p	NM_001164086.1	144.00	-30.28	4 21	3930 3951	17
mmu-miR-378a-5p	NM_001164086.1	143.00	-25.26	2 20	8002 8023	18
mmu-miR-378a-5p	NM_001164086.1	142.00	-31.81	4 19	886 907	15
mmu-miR-378a-5p	NM_010450.3	144.00	-22.22	2 21	682 703	19
mmu-miR-378a-5p	NM_001327998.1	141.00	-25.28	2 20	3638 3665	24
mmu-miR-378a-5p	NM_013868.4	144.00	-24.76	2 21	897 918	19
mmu-miR-378a-5p	XM_006496593.3	149.00	-26.30	2 18	6520 6541	16
mmu-miR-378a-5p	NM_001302471.1	146.00	-19.88	2 20	3300 3322	19
mmu-miR-378a-5p	NM_008423.2	140.00	-23.87	2 17	2870 2891	15
mmu-miR-378a-5p	NM_021342.1	144.00	-21.42	2 17	342 363	15
mmu-miR-378a-5p	XM_006497892.3	155.00	-21.11	2 20	5653 5671	18
mmu-miR-378a-5p	XM_006497892.3	143.00	-30.82	2 21	5420 5443	21
mmu-miR-378a-5p	XM_006497892.3	142.00	-20.30	2 19	1078 1099	17
mmu-miR-378a-5p	NM_173427.2	154.00	-23.79	2 19	3995 4016	17
mmu-miR-378a-5p	NM_173427.2	150.00	-22.73	2 11	3836 3857	9
mmu-miR-378a-5p	NM_173427.2	142.00	-23.61	2 20	152 174	19
mmu-miR-378a-5p	NM_001081667.2	151.00	-13.81	2 21	2090 2112	20
mmu-miR-378a-5p	NM_001081667.2	142.00	-26.85	2 20	1786 1809	20
mmu-miR-378a-5p	NM_029274.2	167.00	-25.51	2 21	5931 5953	20
mmu-miR-378a-5p	NM_029274.2	147.00	-20.04	2 21	1481 1504	21
mmu-miR-378a-5p	NM_029274.2	144.00	-23.37	3 17	2649 2670	14
mmu-miR-378a-5p	XM_017316676.1	148.00	-22.05	2 21	14048 14069	19
mmu-miR-378a-5p	XM_017316676.1	147.00	-28.91	2 20	18004 18025	18
mmu-miR-378a-5p	XM_017316676.1	141.00	-24.85	3 15	19270 19292	13
mmu-miR-378a-5p	XM_006523061.3	143.00	-20.10	2 20	539 560	18
mmu-miR-378a-5p	NM_175271.4	150.00	-24.89	2 21	2550 2576	24
mmu-miR-378a-5p	NM_175271.4	147.00	-23.99	2 18	3563 3583	16
mmu-miR-378a-5p	XM_006506340.3	140.00	-19.62	2 9	4243 4264	7
mmu-miR-378a-5p	NM_028973.2	153.00	-19.54	2 18	2906 2927	16
mmu-miR-378a-5p	NM_028973.2	153.00	-16.10	2 16	4845 4865	14
mmu-miR-378a-5p	NM_028973.2	148.00	-27.29	2 21	2879 2900	19
mmu-miR-378a-5p	NM_001171187.1	148.00	-23.95	2 20	271 290	18
mmu-miR-378a-5p	XM_006541232.3	151.00	-16.37	2 16	3256 3277	14
mmu-miR-378a-5p	XM_006541232.3	145.00	-26.30	2 19	1852 1874	18
mmu-miR-378a-5p	XM_006541232.3	144.00	-15.50	2 14	428 451	14
mmu-miR-378a-5p	XM_006532410.3	140.00	-24.80	2 21	945 966	19
mmu-miR-378a-5p	NM_001290273.1	148.00	-24.24	2 19	51 71	17
mmu-miR-378a-5p	NM_001252563.1	141.00	-20.37	2 19	1107 1130	19

mmu-miR-378a-5p	NM_001291483.1	157.00	-25.83	2 21	619 638	19
mmu-miR-378a-5p	NM_001270475.1	151.00	-19.27	2 19	3215 3234	17
mmu-miR-378a-5p	NM_001270475.1	146.00	-24.81	2 21	6798 6824	24
mmu-miR-378a-5p	NM_001290512.1	147.00	-31.10	2 20	3040 3061	18
mmu-miR-378a-5p	NM_175260.2	146.00	-31.83	2 21	3720 3740	19
mmu-miR-378a-5p	XM_017317978.1	148.00	-26.29	3 21	373 394	18
mmu-miR-378a-5p	NM_173437.2	143.00	-18.23	2 21	3148 3171	21
mmu-miR-378a-5p	NM_001242558.1	162.00	-28.45	2 21	2432 2452	19
mmu-miR-378a-5p	NM_011424.3	163.00	-41.63	2 21	8565 8587	20
mmu-miR-378a-5p	NM_011424.3	143.00	-16.22	2 21	312 334	20
mmu-miR-378a-5p	NM_001109985.1	155.00	-21.21	2 21	1545 1569	22
mmu-miR-378a-5p	NM_181547.3	142.00	-20.65	3 20	235 257	18
mmu-miR-378a-5p	NM_001029877.3	140.00	-22.75	2 20	3359 3381	20
mmu-miR-378a-5p	NM_001077403.1	142.00	-20.96	2 19	5874 5895	17
mmu-miR-378a-5p	NM_001282961.1	140.00	-27.35	2 21	3837 3855	19
mmu-miR-378a-5p	NM_145210.2	162.00	-21.57	2 19	1136 1157	17
mmu-miR-378a-5p	NM_145210.2	142.00	-25.61	3 21	1316 1341	22
mmu-miR-378a-5p	XM_006530325.3	160.00	-22.39	2 21	946 967	19
mmu-miR-378a-5p	XM_006530294.3	157.00	-29.07	2 20	1084 1104	18
mmu-miR-378a-5p	NM_011854.2	146.00	-23.04	2 20	891 913	19
mmu-miR-378a-5p	NM_011854.2	146.00	-15.60	2 20	1587 1609	19
mmu-miR-378a-5p	NM_011854.2	140.00	-18.72	2 17	212 233	15
mmu-miR-378a-5p	XM_006516928.2	158.00	-19.25	2 21	4239 4262	21
mmu-miR-378a-5p	NM_011010.2	151.00	-34.91	2 21	1787 1809	20
mmu-miR-378a-5p	XM_011239969.2	152.00	-24.57	2 17	661 682	15
mmu-miR-378a-5p	NM_001286743.1	150.00	-18.33	2 20	1493 1516	20
mmu-miR-494-3p	NM_177743.5	145.00	-10.83	2 19	3517 3539	18
mmu-miR-494-3p	NM_010181.2	160.00	-20.28	2 19	4621 4641	17
mmu-miR-494-3p	NM_010181.2	142.00	-10.62	3 21	10400 10420	18
mmu-miR-494-3p	XM_006527803.1	156.00	-10.09	2 17	1546 1567	15
mmu-miR-494-3p	XM_006496949.3	157.00	-12.09	2 18	3961 3982	16
mmu-miR-494-3p	XM_006496949.3	149.00	-22.52	2 21	1232 1260	26
mmu-miR-494-3p	NM_172862.3	141.00	-15.29	2 18	8254 8275	16
mmu-miR-494-3p	NM_172862.3	140.00	-10.81	2 9	6775 6796	7
mmu-miR-494-3p	XM_006533539.3	140.00	-11.82	2 9	4977 4998	7
mmu-miR-494-3p	NM_008067.4	154.00	-20.00	2 21	1819 1839	19
mmu-miR-494-3p	NM_010279.3	140.00	-8.53	2 9	3363 3384	7
mmu-miR-494-3p	NM_016697.3	146.00	-10.08	2 12	2184 2206	11
mmu-miR-494-3p	NM_016697.3	142.00	-17.14	2 16	2043 2065	15
mmu-miR-494-3p	XM_011250978.2	144.00	-12.32	2 13	688 709	11
mmu-miR-494-3p	XM_006526069.2	141.00	-15.01	3 20	5234 5254	17
mmu-miR-494-3p	XM_006499444.3	156.00	-21.98	2 21	1007 1028	19
mmu-miR-494-3p	XM_006505602.3	147.00	-7.72	2 20	11293 11314	18
mmu-miR-494-3p	NM_001164086.1	143.00	-13.75	2 20	4516 4537	18
mmu-miR-494-3p	NM_001164086.1	143.00	-11.03	3 20	10390 10411	17
mmu-miR-494-3p	NM_001164086.1	140.00	-9.06	2 9	5870 5891	7
mmu-miR-494-3p	NM_008264.1	148.00	-15.68	2 15	142 162	13
mmu-miR-494-3p	NM_001327998.1	140.00	-22.24	2 21	534 553	19
mmu-miR-494-3p	NM_030704.3	140.00	-14.49	2 21	1393 1414	19
mmu-miR-494-3p	NM_133871.2	147.00	-15.99	2 21	382 407	23
mmu-miR-494-3p	XM_006511311.2	151.00	-14.41	2 16	5588 5609	14
mmu-miR-494-3p	XM_006511311.2	145.00	-13.74	2 20	4590 4613	20
mmu-miR-494-3p	XM_006496593.3	140.00	-15.44	2 18	2595 2617	17
mmu-miR-494-3p	XM_006518921.3	143.00	-14.60	2 16	1796 1817	14
mmu-miR-494-3p	XM_017315734.1	154.00	-17.20	3 19	2535 2556	16
mmu-miR-494-3p	XM_017316478.1	140.00	-10.96	2 9	1335 1356	7
mmu-miR-494-3p	XM_017316676.1	148.00	-12.74	2 19	17600 17620	17
mmu-miR-494-3p	XM_017316676.1	140.00	-13.82	2 9	16817 16838	7
mmu-miR-494-3p	XM_017315313.1	142.00	-10.71	2 19	2478 2499	17
mmu-miR-494-3p	NM_010714.3	141.00	-16.15	2 18	4380 4401	16
mmu-miR-494-3p	NM_010714.3	140.00	-9.66	2 9	4169 4190	7
mmu-miR-494-3p	XM_006519969.3	149.00	-13.45	2 18	1842 1863	16
mmu-miR-494-3p	NM_053098.2	145.00	-8.15	2 14	1908 1929	12
mmu-miR-494-3p	NM_175271.4	147.00	-9.71	2 12	1194 1215	10
mmu-miR-494-3p	XM_006506340.3	143.00	-14.19	2 14	3421 3441	12

mmu-miR-494-3p	XM_006506340.3	140.00	-9.50	2 9	3096 3117	7
mmu-miR-494-3p	NM_028838.2	143.00	-12.51	2 16	1624 1645	14
mmu-miR-494-3p	NM_001171187.1	156.00	-21.49	2 21	2559 2577	19
mmu-miR-494-3p	XM_006541232.3	145.00	-15.94	2 10	970 991	8
mmu-miR-494-3p	XM_006541232.3	142.00	-12.07	2 11	3651 3672	9
mmu-miR-494-3p	XM_006498872.3	143.00	-18.46	2 20	763 784	18
mmu-miR-494-3p	NM_001290273.1	161.00	-20.22	2 20	1388 1408	18
mmu-miR-494-3p	NM_001290273.1	150.00	-16.21	2 21	1317 1343	24
mmu-miR-494-3p	NM_001290512.1	140.00	-15.55	2 9	2424 2445	7
mmu-miR-494-3p	NM_001320077.1	140.00	-8.70	2 9	3391 3412	7
mmu-miR-494-3p	NM_008623.5	140.00	-14.74	2 21	8 29	19
mmu-miR-494-3p	XM_006537659.2	145.00	-8.79	2 20	5190 5210	18
mmu-miR-494-3p	NM_173437.2	141.00	-11.18	3 20	12405 12425	17
mmu-miR-494-3p	NM_001077403.1	145.00	-9.50	2 10	881 902	8
mmu-miR-494-3p	XM_006516928.2	155.00	-14.58	2 12	2489 2510	10
mmu-miR-494-3p	XM_006516928.2	140.00	-22.44	2 17	1255 1276	15
mmu-miR-494-3p	XM_011239969.2	149.00	-15.67	3 18	4382 4403	15
mmu-miR-494-3p	XM_011239969.2	145.00	-9.45	2 18	4854 4875	16
mmu-miR-494-3p	XM_011239969.2	142.00	-15.78	2 20	4439 4461	19
mmu-miR-135a-1-3p	XM_006532962.2	140.00	-28.07	3 21	2228 2249	18
mmu-miR-135a-1-3p	NM_001160262.1	152.00	-15.31	2 19	1768 1788	17
mmu-miR-135a-1-3p	NM_010181.2	146.00	-31.44	3 21	6572 6592	18
mmu-miR-135a-1-3p	NM_145141.2	140.00	-16.41	2 9	1251 1272	7
mmu-miR-135a-1-3p	XM_006527803.1	142.00	-19.10	2 20	1762 1784	19
mmu-miR-135a-1-3p	NM_001081185.1	153.00	-24.33	2 21	4830 4849	19
mmu-miR-135a-1-3p	NM_172862.3	152.00	-21.03	2 18	1881 1903	17
mmu-miR-135a-1-3p	NM_172862.3	146.00	-21.67	2 20	1579 1601	19
mmu-miR-135a-1-3p	XM_006533539.3	141.00	-15.97	2 15	8125 8147	14
mmu-miR-135a-1-3p	NM_001033445.2	156.00	-22.16	2 21	1618 1639	19
mmu-miR-135a-1-3p	NM_001033445.2	154.00	-20.33	2 21	1130 1150	19
mmu-miR-135a-1-3p	NM_001033445.2	140.00	-15.45	2 9	1708 1729	7
mmu-miR-135a-1-3p	XM_006514444.1	140.00	-16.82	2 21	2764 2785	19
mmu-miR-135a-1-3p	XM_006530353.3	142.00	-29.63	2 21	13377 13400	21
mmu-miR-135a-1-3p	XM_011248576.2	140.00	-20.96	2 14	5013 5035	13
mmu-miR-135a-1-3p	NM_001198955.1	150.00	-18.29	2 11	2175 2196	9
mmu-miR-135a-1-3p	NM_010340.2	144.00	-13.22	2 14	2368 2391	14
mmu-miR-135a-1-3p	XM_006526069.2	145.00	-15.36	2 10	624 645	8
mmu-miR-135a-1-3p	XM_006496277.3	150.00	-15.19	2 15	481 502	13
mmu-miR-135a-1-3p	XM_006496277.3	141.00	-21.41	3 20	1151 1176	21
mmu-miR-135a-1-3p	XM_006505602.3	144.00	-24.35	2 21	9766 9787	19
mmu-miR-135a-1-3p	XM_006505602.3	142.00	-22.48	2 19	10339 10360	17
mmu-miR-135a-1-3p	XM_006505602.3	140.00	-21.55	3 21	3052 3070	18
mmu-miR-135a-1-3p	NM_001164086.1	157.00	-24.79	2 21	6260 6287	25
mmu-miR-135a-1-3p	NM_010450.3	144.00	-22.03	2 21	1218 1239	19
mmu-miR-135a-1-3p	NM_001327998.1	141.00	-22.05	2 20	3762 3786	21
mmu-miR-135a-1-3p	NM_013560.2	148.00	-15.94	2 21	604 625	19
mmu-miR-135a-1-3p	NM_013868.4	142.00	-22.91	2 15	407 428	13
mmu-miR-135a-1-3p	NM_013868.4	140.00	-14.12	2 9	1723 1744	7
mmu-miR-135a-1-3p	XM_006511311.2	148.00	-22.78	2 21	4074 4095	19
mmu-miR-135a-1-3p	XM_006511311.2	140.00	-32.58	3 21	4991 5012	18
mmu-miR-135a-1-3p	XM_006496593.3	150.00	-27.14	3 21	6281 6301	18
mmu-miR-135a-1-3p	XM_006496593.3	142.00	-19.06	2 12	1371 1393	11
mmu-miR-135a-1-3p	XM_006528842.1	143.00	-16.93	2 21	3988 4012	22
mmu-miR-135a-1-3p	NM_001161541.1	150.00	-23.94	2 21	2961 2981	19
mmu-miR-135a-1-3p	NM_001161541.1	143.00	-33.19	2 21	2091 2113	20
mmu-miR-135a-1-3p	NM_001289875.1	144.00	-19.51	2 21	4091 4112	19
mmu-miR-135a-1-3p	XM_017315734.1	158.00	-16.82	2 21	4531 4551	19
mmu-miR-135a-1-3p	XM_017315734.1	142.00	-14.21	2 14	2064 2083	12
mmu-miR-135a-1-3p	XM_011248919.2	143.00	-23.50	2 16	5911 5932	14
mmu-miR-135a-1-3p	XM_011248919.2	141.00	-26.51	3 21	1858 1881	21
mmu-miR-135a-1-3p	NM_173427.2	158.00	-23.16	2 21	325 345	19
mmu-miR-135a-1-3p	XM_017320093.1	145.00	-14.63	2 21	2184 2203	19
mmu-miR-135a-1-3p	NM_029274.2	147.00	-16.02	2 21	8062 8085	21
mmu-miR-135a-1-3p	NM_029274.2	141.00	-25.61	3 19	735 759	19
mmu-miR-135a-1-3p	XM_017316676.1	151.00	-17.75	2 21	14635 14658	21

mmu-miR-135a-1-3p	XM_017316676.1	142.00	-19.73	2 21	4654 4678	22
mmu-miR-135a-1-3p	NM_146063.1	154.00	-25.26	2 21	2033 2053	19
mmu-miR-135a-1-3p	XM_017315313.1	144.00	-17.29	2 21	1886 1907	19
mmu-miR-135a-1-3p	XM_006504999.1	144.00	-26.60	2 17	2101 2122	15
mmu-miR-135a-1-3p	NM_144945.3	152.00	-22.72	2 21	3756 3774	19
mmu-miR-135a-1-3p	NM_144945.3	145.00	-15.51	2 14	6005 6026	12
mmu-miR-135a-1-3p	NM_144945.3	143.00	-20.29	2 21	2262 2286	22
mmu-miR-135a-1-3p	NM_144799.2	156.00	-19.07	2 21	641 662	19
mmu-miR-135a-1-3p	XM_006506340.3	154.00	-17.51	2 19	3536 3557	17
mmu-miR-135a-1-3p	XM_006506340.3	140.00	-29.38	2 21	4496 4517	19
mmu-miR-135a-1-3p	NM_028973.2	142.00	-24.83	2 15	4646 4667	13
mmu-miR-135a-1-3p	XM_006541232.3	153.00	-15.94	2 19	3371 3394	19
mmu-miR-135a-1-3p	XM_006541232.3	148.00	-22.40	3 21	1032 1053	18
mmu-miR-135a-1-3p	XM_006541232.3	146.00	-14.99	2 20	5923 5945	19
mmu-miR-135a-1-3p	XM_006498872.3	142.00	-24.60	3 21	9080 9100	18
mmu-miR-135a-1-3p	XM_006532410.3	140.00	-19.99	3 21	1952 1973	18
mmu-miR-135a-1-3p	XM_006532410.3	140.00	-15.09	2 21	4052 4073	19
mmu-miR-135a-1-3p	XM_011243266.1	142.00	-17.43	2 15	3958 3979	13
mmu-miR-135a-1-3p	XM_006504940.3	144.00	-18.76	3 21	1162 1183	18
mmu-miR-135a-1-3p	NM_001270475.1	142.00	-27.04	2 21	5500 5520	19
mmu-miR-135a-1-3p	NM_001320077.1	150.00	-16.28	2 19	2381 2402	17
mmu-miR-135a-1-3p	NM_001005423.2	144.00	-19.08	2 17	1600 1621	15
mmu-miR-135a-1-3p	XM_006537659.2	162.00	-21.98	2 20	4195 4217	19
mmu-miR-135a-1-3p	XM_006537659.2	156.00	-18.09	2 19	2722 2742	17
mmu-miR-135a-1-3p	XM_006537659.2	141.00	-16.70	2 21	1974 1993	19
mmu-miR-135a-1-3p	NM_175260.2	146.00	-22.85	2 21	1230 1250	19
mmu-miR-135a-1-3p	XM_017315841.1	140.00	-19.82	2 21	110 131	19
mmu-miR-135a-1-3p	XM_017320223.1	142.00	-13.88	2 11	2455 2476	9
mmu-miR-135a-1-3p	XM_017320223.1	142.00	-13.61	2 11	4378 4399	9
mmu-miR-135a-1-3p	XM_017317978.1	151.00	-17.52	2 17	564 586	16
mmu-miR-135a-1-3p	NM_173437.2	147.00	-18.23	2 21	12256 12278	20
mmu-miR-135a-1-3p	NM_173437.2	143.00	-18.75	3 20	2735 2756	17
mmu-miR-135a-1-3p	XM_006541297.3	145.00	-22.27	2 21	5397 5416	19
mmu-miR-135a-1-3p	XM_006541297.3	142.00	-15.27	2 11	3511 3532	9
mmu-miR-135a-1-3p	NM_011424.3	147.00	-26.23	3 21	6109 6131	19
mmu-miR-135a-1-3p	NM_001134300.2	150.00	-18.24	2 21	5066 5086	19
mmu-miR-135a-1-3p	NM_001029877.3	158.00	-28.21	2 21	6580 6600	19
mmu-miR-135a-1-3p	NM_001029877.3	155.00	-22.45	2 21	7487 7510	21
mmu-miR-135a-1-3p	NM_001029877.3	149.00	-27.75	2 18	4312 4333	16
mmu-miR-135a-1-3p	NM_001029877.3	145.00	-26.84	2 21	2552 2579	25
mmu-miR-135a-1-3p	NM_001029877.3	140.00	-17.68	3 21	3610 3631	18
mmu-miR-135a-1-3p	NM_001029877.3	140.00	-15.36	2 9	4476 4497	7
mmu-miR-135a-1-3p	NM_001077403.1	142.00	-17.74	2 17	4860 4880	15
mmu-miR-135a-1-3p	NM_001282961.1	140.00	-20.98	2 18	3409 3432	18
mmu-miR-135a-1-3p	XM_006530325.3	141.00	-20.26	2 20	381 401	18
mmu-miR-135a-1-3p	XM_011239969.2	157.00	-19.50	2 21	4613 4632	19
mmu-miR-135a-1-3p	XM_011239969.2	143.00	-17.58	2 20	5679 5700	18
mmu-miR-1892	XM_017319626.1	157.00	-25.76	2 18	3884 3905	16
mmu-miR-1892	XM_017319626.1	153.00	-30.70	2 20	2592 2615	20
mmu-miR-1892	XM_006532962.2	154.00	-21.30	2 19	3624 3645	17
mmu-miR-1892	XM_006532962.2	153.00	-20.60	2 19	2136 2158	18
mmu-miR-1892	XM_006532962.2	145.00	-27.41	2 21	541 566	23
mmu-miR-1892	XM_006532962.2	143.00	-19.92	2 21	3348 3370	20
mmu-miR-1892	XM_006532962.2	140.00	-29.94	2 21	3222 3243	19
mmu-miR-1892	NM_001160262.1	162.00	-22.03	2 15	3350 3371	13
mmu-miR-1892	NM_001160262.1	155.00	-21.33	2 20	3593 3614	18
mmu-miR-1892	NM_001160262.1	150.00	-24.73	3 19	1839 1860	16
mmu-miR-1892	NM_001160262.1	147.00	-25.40	2 21	4362 4385	21
mmu-miR-1892	NM_001160262.1	144.00	-25.07	2 21	2507 2528	19
mmu-miR-1892	NM_001160262.1	144.00	-11.64	2 13	3060 3081	11
mmu-miR-1892	NM_010181.2	155.00	-22.66	2 20	2035 2056	18
mmu-miR-1892	NM_010181.2	147.00	-18.71	2 13	6514 6536	12
mmu-miR-1892	NM_010181.2	143.00	-23.35	2 18	8363 8383	16
mmu-miR-1892	NM_010181.2	140.00	-25.23	3 20	485 506	18
mmu-miR-1892	XM_011244488.2	146.00	-27.26	2 21	1501 1521	19

mmu-miR-1892	NM_145141.2	168.00	-28.84	2 17	1585 1606	15
mmu-miR-1892	NM_145141.2	150.00	-20.48	2 16	739 761	15
mmu-miR-1892	NM_145141.2	143.00	-22.03	2 16	943 964	14
mmu-miR-1892	NM_001081185.1	161.00	-23.56	2 14	3148 3169	12
mmu-miR-1892	NM_001081185.1	142.00	-21.89	2 15	8179 8200	13
mmu-miR-1892	NM_001081185.1	141.00	-25.87	3 19	561 583	17
mmu-miR-1892	NM_001081185.1	141.00	-22.94	2 21	6315 6334	19
mmu-miR-1892	XM_006496949.3	151.00	-18.29	2 21	1960 1982	20
mmu-miR-1892	NM_172862.3	156.00	-18.80	2 21	2837 2858	19
mmu-miR-1892	NM_172862.3	142.00	-16.07	2 12	5189 5211	11
mmu-miR-1892	NM_172862.3	142.00	-19.88	2 17	7449 7469	15
mmu-miR-1892	XM_006533539.3	166.00	-27.80	2 21	6008 6028	19
mmu-miR-1892	XM_006533539.3	155.00	-24.17	2 21	4657 4679	20
mmu-miR-1892	XM_006533539.3	153.00	-21.44	2 16	6408 6428	14
mmu-miR-1892	XM_006533539.3	142.00	-24.02	2 19	6151 6172	17
mmu-miR-1892	XM_006533539.3	141.00	-23.77	2 18	2684 2705	16
mmu-miR-1892	XM_006533539.3	140.00	-27.42	2 21	8457 8478	19
mmu-miR-1892	NM_001033221.3	156.00	-22.69	2 17	1224 1245	15
mmu-miR-1892	NM_001033221.3	148.00	-25.46	2 21	838 859	19
mmu-miR-1892	NM_008067.4	140.00	-30.66	2 20	414 440	23
mmu-miR-1892	NM_001033445.2	164.00	-27.31	2 18	4495 4517	17
mmu-miR-1892	NM_001033445.2	155.00	-25.25	2 21	2538 2562	22
mmu-miR-1892	NM_001033445.2	148.00	-29.48	2 21	2731 2752	19
mmu-miR-1892	NM_001033445.2	146.00	-27.34	2 21	1901 1927	24
mmu-miR-1892	NM_001033445.2	146.00	-28.55	2 21	2007 2027	19
mmu-miR-1892	NM_001033445.2	143.00	-25.28	2 20	1264 1285	18
mmu-miR-1892	NM_001033445.2	142.00	-25.64	2 21	453 473	19
mmu-miR-1892	NM_001033445.2	141.00	-24.76	2 21	1964 1985	20
mmu-miR-1892	NM_001033445.2	140.00	-14.55	2 9	1423 1444	7
mmu-miR-1892	NM_010279.3	140.00	-20.01	2 21	4599 4620	19
mmu-miR-1892	XM_006504899.3	145.00	-24.13	2 21	680 699	19
mmu-miR-1892	XM_006530353.3	155.00	-20.87	2 16	6218 6239	14
mmu-miR-1892	XM_006530353.3	147.00	-26.50	2 20	6329 6350	18
mmu-miR-1892	XM_006530353.3	146.00	-19.46	2 16	15054 15076	15
mmu-miR-1892	XM_006530353.3	145.00	-23.21	2 21	10547 10568	20
mmu-miR-1892	XM_006530353.3	144.00	-30.61	2 21	10586 10607	19
mmu-miR-1892	XM_006530353.3	143.00	-28.94	3 21	3602 3624	19
mmu-miR-1892	XM_006530353.3	141.00	-24.95	2 14	386 407	12
mmu-miR-1892	XM_006530353.3	140.00	-11.64	2 9	1943 1964	7
mmu-miR-1892	XM_006530353.3	140.00	-31.82	2 18	10690 10712	17
mmu-miR-1892	XM_006530353.3	140.00	-19.40	2 21	11870 11891	19
mmu-miR-1892	NM_001024731.2	154.00	-17.51	2 19	346 367	17
mmu-miR-1892	NM_001024731.2	148.00	-17.54	2 21	430 451	19
mmu-miR-1892	XM_011248576.2	160.00	-35.62	2 21	10361 10382	19
mmu-miR-1892	XM_011248576.2	159.00	-32.35	2 20	2750 2771	18
mmu-miR-1892	XM_011248576.2	154.00	-30.77	2 21	7766 7789	21
mmu-miR-1892	XM_011248576.2	153.00	-28.02	2 18	1385 1406	16
mmu-miR-1892	XM_011248576.2	152.00	-28.42	2 21	7901 7920	19
mmu-miR-1892	XM_011248576.2	149.00	-19.49	2 14	2500 2521	12
mmu-miR-1892	XM_011248576.2	148.00	-27.95	3 18	9862 9884	16
mmu-miR-1892	XM_011248576.2	147.00	-21.38	2 21	4303 4325	20
mmu-miR-1892	XM_011248576.2	144.00	-29.58	2 21	5459 5480	19
mmu-miR-1892	XM_011248576.2	144.00	-18.25	2 13	7335 7356	11
mmu-miR-1892	XM_011248576.2	142.00	-28.43	2 21	2004 2029	23
mmu-miR-1892	XM_011248576.2	140.00	-21.07	2 17	221 242	15
mmu-miR-1892	XM_011244235.2	154.00	-20.99	2 21	1390 1410	19
mmu-miR-1892	NM_001198955.1	144.00	-20.78	2 17	2899 2920	15
mmu-miR-1892	XM_006532232.3	156.00	-21.52	2 21	1482 1503	19
mmu-miR-1892	XM_006532232.3	145.00	-24.24	3 18	1302 1323	15
mmu-miR-1892	XM_006532232.3	144.00	-24.33	2 21	1386 1407	19
mmu-miR-1892	XM_006532232.3	144.00	-23.22	2 21	1626 1647	19
mmu-miR-1892	XM_006532232.3	144.00	-23.22	2 21	1722 1743	19
mmu-miR-1892	XM_006532232.3	140.00	-20.39	3 19	37 57	16
mmu-miR-1892	XM_006532232.3	140.00	-22.10	2 21	1434 1455	19
mmu-miR-1892	XM_006532232.3	140.00	-20.69	2 21	1578 1599	19

mmu-miR-1892	XM_006532232.3	140.00	-20.15	2 21	1674 1695	19
mmu-miR-1892	NM_016697.3	146.00	-25.89	2 20	142 164	19
mmu-miR-1892	XM_011250978.2	142.00	-16.85	2 15	4211 4232	13
mmu-miR-1892	NM_010340.2	157.00	-25.89	2 19	1296 1318	18
mmu-miR-1892	XM_006515096.2	152.00	-20.10	2 18	817 839	17
mmu-miR-1892	XM_006515096.2	147.00	-29.39	3 21	258 280	19
mmu-miR-1892	XM_006515096.2	147.00	-25.59	2 21	5134 5157	21
mmu-miR-1892	XM_006515096.2	146.00	-25.71	2 21	3417 3437	19
mmu-miR-1892	XM_006515096.2	140.00	-23.01	2 21	8000 8021	19
mmu-miR-1892	XM_006526069.2	143.00	-21.42	2 21	4964 4986	20
mmu-miR-1892	XM_006526069.2	141.00	-21.83	2 21	1645 1666	20
mmu-miR-1892	XM_006496277.3	144.00	-18.03	2 21	1690 1711	19
mmu-miR-1892	NM_008204.2	145.00	-12.64	2 10	1281 1302	8
mmu-miR-1892	NM_008204.2	142.00	-14.36	2 11	621 642	9
mmu-miR-1892	XM_011246309.2	156.00	-25.00	2 18	347 370	18
mmu-miR-1892	XM_011246309.2	141.00	-18.33	2 19	313 335	18
mmu-miR-1892	NM_175520.4	163.00	-28.45	2 18	1260 1280	16
mmu-miR-1892	NM_175520.4	141.00	-32.84	2 21	2530 2561	29
mmu-miR-1892	NM_001162950.1	147.00	-21.05	2 20	2680 2701	18
mmu-miR-1892	NM_001162950.1	146.00	-24.86	2 21	5853 5877	22
mmu-miR-1892	NM_001162950.1	143.00	-27.37	2 21	1800 1822	20
mmu-miR-1892	NM_001162950.1	142.00	-15.64	2 11	1344 1365	9
mmu-miR-1892	NM_001162950.1	140.00	-23.16	3 21	2164 2185	18
mmu-miR-1892	NM_001162950.1	140.00	-20.46	2 21	5896 5917	19
mmu-miR-1892	XM_006505602.3	151.00	-29.46	3 21	498 520	19
mmu-miR-1892	XM_006505602.3	149.00	-28.23	3 18	9464 9485	15
mmu-miR-1892	XM_006505602.3	147.00	-13.53	2 12	7572 7593	10
mmu-miR-1892	XM_006505602.3	146.00	-15.81	2 19	1252 1273	17
mmu-miR-1892	XM_006505602.3	145.00	-14.53	2 19	13102 13124	18
mmu-miR-1892	XM_006505602.3	145.00	-12.78	2 10	13663 13684	8
mmu-miR-1892	XM_006505602.3	143.00	-19.32	2 21	795 817	20
mmu-miR-1892	XM_006505602.3	141.00	-20.43	2 14	13706 13727	12
mmu-miR-1892	NM_001164086.1	146.00	-13.82	2 19	3508 3529	17
mmu-miR-1892	NM_001164086.1	143.00	-25.73	2 21	4181 4204	21
mmu-miR-1892	NM_001164086.1	142.00	-18.60	2 21	6595 6615	19
mmu-miR-1892	NM_001164086.1	142.00	-14.56	2 15	8234 8255	13
mmu-miR-1892	NM_010450.3	148.00	-23.73	2 21	183 204	19
mmu-miR-1892	NM_010450.3	145.00	-27.92	2 18	1946 1967	16
mmu-miR-1892	NM_008264.1	144.00	-16.74	2 15	960 980	13
mmu-miR-1892	NM_001327998.1	142.00	-26.63	2 21	4485 4508	21
mmu-miR-1892	NM_001327998.1	141.00	-13.48	2 11	2988 3010	10
mmu-miR-1892	NM_001327998.1	140.00	-12.06	2 9	4382 4403	7
mmu-miR-1892	NM_013558.2	140.00	-26.48	2 18	1192 1214	17
mmu-miR-1892	XM_006539957.3	152.00	-24.65	3 21	1298 1319	18
mmu-miR-1892	XM_006539957.3	140.00	-14.84	2 9	1101 1122	7
mmu-miR-1892	NM_013868.4	161.00	-26.73	2 19	2540 2562	18
mmu-miR-1892	NM_013868.4	156.00	-23.01	2 19	1654 1674	17
mmu-miR-1892	NM_013868.4	140.00	-13.15	2 9	2518 2539	7
mmu-miR-1892	NM_030704.3	144.00	-30.58	3 21	994 1015	18
mmu-miR-1892	NM_133871.2	143.00	-25.55	2 21	1608 1631	21
mmu-miR-1892	NM_023670.3	151.00	-19.48	2 21	4269 4291	20
mmu-miR-1892	NM_023670.3	149.00	-27.94	2 19	222 244	18
mmu-miR-1892	NM_001159424.2	156.00	-21.32	2 17	928 949	15
mmu-miR-1892	XM_006496593.3	152.00	-22.02	2 21	4800 4821	19
mmu-miR-1892	XM_006516224.3	142.00	-21.47	2 21	2519 2543	22
mmu-miR-1892	XM_006528842.1	166.00	-28.34	2 19	3331 3352	17
mmu-miR-1892	XM_006528842.1	148.00	-22.93	3 21	2367 2388	18
mmu-miR-1892	XM_006528842.1	147.00	-27.16	3 21	4632 4654	19
mmu-miR-1892	XM_006528842.1	144.00	-25.06	2 21	3390 3411	19
mmu-miR-1892	XM_006528842.1	141.00	-18.28	2 16	1765 1785	14
mmu-miR-1892	NM_001161541.1	145.00	-24.76	2 21	2524 2543	19
mmu-miR-1892	NM_001302471.1	145.00	-19.52	2 10	3499 3520	8
mmu-miR-1892	NM_001302471.1	143.00	-24.19	2 21	1575 1598	21
mmu-miR-1892	NM_001289875.1	154.00	-25.21	2 20	2562 2584	19
mmu-miR-1892	NM_001289875.1	140.00	-16.04	2 9	2139 2160	7

mmu-miR-1892	NM_021342.1	145.00	-12.70	2 10	1953 1974	8
mmu-miR-1892	NM_010608.2	146.00	-23.44	2 21	1522 1542	19
mmu-miR-1892	NM_010608.2	146.00	-25.69	3 21	3610 3630	18
mmu-miR-1892	NM_010608.2	145.00	-23.37	2 21	1869 1891	21
mmu-miR-1892	NM_010608.2	140.00	-15.78	2 9	1446 1467	7
mmu-miR-1892	NM_010608.2	140.00	-23.15	2 21	2053 2074	19
mmu-miR-1892	XM_011248919.2	149.00	-26.35	2 21	581 600	19
mmu-miR-1892	XM_011248919.2	145.00	-23.91	2 18	6060 6081	16
mmu-miR-1892	XM_011248919.2	144.00	-26.09	3 18	2117 2140	17
mmu-miR-1892	XM_011248919.2	144.00	-28.28	2 21	2319 2340	19
mmu-miR-1892	XM_011248919.2	142.00	-19.29	2 15	2377 2398	13
mmu-miR-1892	XM_011248919.2	140.00	-22.04	3 21	1466 1487	18
mmu-miR-1892	XM_017316478.1	151.00	-21.33	2 12	795 816	10
mmu-miR-1892	NM_173427.2	148.00	-23.96	2 18	4473 4496	18
mmu-miR-1892	NM_173427.2	145.00	-28.20	2 18	3502 3523	16
mmu-miR-1892	XM_006512296.3	145.00	-27.40	2 21	1667 1691	22
mmu-miR-1892	NM_008940.3	155.00	-31.62	2 21	400 422	20
mmu-miR-1892	NM_016659.3	150.00	-21.73	2 21	1581 1601	19
mmu-miR-1892	NM_029274.2	163.00	-26.66	2 21	6866 6888	20
mmu-miR-1892	NM_029274.2	158.00	-24.16	2 16	508 531	16
mmu-miR-1892	NM_029274.2	158.00	-26.07	2 21	1568 1588	19
mmu-miR-1892	NM_029274.2	154.00	-27.42	2 21	1513 1533	19
mmu-miR-1892	NM_029274.2	150.00	-18.89	2 11	7207 7228	9
mmu-miR-1892	NM_029274.2	145.00	-21.67	2 21	6530 6553	22
mmu-miR-1892	NM_029274.2	140.00	-17.61	2 9	832 853	7
mmu-miR-1892	NM_029274.2	140.00	-14.12	2 9	1013 1034	7
mmu-miR-1892	XM_017316676.1	164.00	-31.88	2 21	13373 13394	19
mmu-miR-1892	XM_017316676.1	164.00	-26.09	2 17	19212 19233	15
mmu-miR-1892	XM_017316676.1	156.00	-28.76	2 21	18711 18732	19
mmu-miR-1892	XM_017316676.1	155.00	-19.43	2 21	7034 7056	20
mmu-miR-1892	XM_017316676.1	154.00	-26.76	2 21	16965 16990	23
mmu-miR-1892	XM_017316676.1	146.00	-23.63	2 19	2796 2817	17
mmu-miR-1892	XM_017316676.1	146.00	-22.50	2 21	6866 6886	19
mmu-miR-1892	XM_017316676.1	144.00	-33.47	4 21	1653 1674	17
mmu-miR-1892	XM_017316676.1	144.00	-31.59	2 21	9548 9567	19
mmu-miR-1892	XM_017316676.1	140.00	-33.97	4 21	2241 2262	17
mmu-miR-1892	XM_017316676.1	140.00	-32.15	2 21	4010 4031	19
mmu-miR-1892	XM_017315313.1	146.00	-25.33	2 19	1971 1992	17
mmu-miR-1892	NM_008479.2	150.00	-27.59	2 21	476 496	19
mmu-miR-1892	XM_006511102.2	141.00	-13.81	2 12	1524 1544	10
mmu-miR-1892	XM_006504999.1	146.00	-23.20	2 21	340 360	19
mmu-miR-1892	NM_144945.3	140.00	-23.43	2 17	764 785	15
mmu-miR-1892	NM_010714.3	140.00	-13.65	2 9	4231 4252	7
mmu-miR-1892	XM_006519969.3	168.00	-27.49	2 21	1 21	19
mmu-miR-1892	NM_053098.2	142.00	-23.59	2 21	1317 1340	21
mmu-miR-1892	NM_053098.2	141.00	-31.39	2 21	1410 1431	20
mmu-miR-1892	NM_053098.2	140.00	-17.39	2 13	1816 1837	11
mmu-miR-1892	NM_175271.4	152.00	-18.91	2 17	1558 1579	15
mmu-miR-1892	XM_006506340.3	151.00	-18.88	2 19	4925 4944	17
mmu-miR-1892	NM_028973.2	141.00	-23.36	2 20	3915 3935	18
mmu-miR-1892	NM_028973.2	141.00	-23.00	2 14	4903 4924	12
mmu-miR-1892	NM_028838.2	158.00	-21.61	2 20	134 156	19
mmu-miR-1892	XM_006506086.2	163.00	-25.72	2 20	1546 1567	18
mmu-miR-1892	XM_006506086.2	147.00	-24.06	3 21	789 811	19
mmu-miR-1892	XM_006506086.2	140.00	-11.29	2 9	2306 2327	7
mmu-miR-1892	NM_008524.2	151.00	-26.66	3 21	81 103	19
mmu-miR-1892	NM_001171187.1	152.00	-23.26	2 19	2174 2197	19
mmu-miR-1892	XM_006541232.3	155.00	-26.61	2 21	1473 1495	20
mmu-miR-1892	XM_006541232.3	151.00	-29.37	2 21	1602 1625	21
mmu-miR-1892	XM_006541232.3	143.00	-17.84	2 17	1420 1442	16
mmu-miR-1892	XM_006498872.3	160.00	-24.04	2 21	6715 6736	19
mmu-miR-1892	XM_006498872.3	157.00	-19.58	2 14	6931 6952	12
mmu-miR-1892	XM_006498872.3	146.00	-22.64	2 21	3298 3321	21
mmu-miR-1892	XM_006498872.3	144.00	-30.60	2 21	6247 6268	19
mmu-miR-1892	XM_006498872.3	143.00	-28.91	2 21	3918 3942	22

mmu-miR-1892	XM_006498872.3	143.00	-25.21	2 19	4219 4240	18
mmu-miR-1892	XM_006498872.3	143.00	-29.54	2 21	9079 9101	20
mmu-miR-1892	XM_006498872.3	142.00	-22.78	3 19	7347 7368	16
mmu-miR-1892	XM_006498872.3	141.00	-24.89	2 14	7027 7048	12
mmu-miR-1892	XM_006498872.3	140.00	-23.62	2 21	1712 1733	19
mmu-miR-1892	XM_006532410.3	143.00	-18.94	2 21	1127 1150	21
mmu-miR-1892	XM_006532410.3	143.00	-26.82	2 21	1636 1661	23
mmu-miR-1892	XM_006520023.2	145.00	-18.72	2 20	2324 2349	22
mmu-miR-1892	NM_013593.3	154.00	-18.54	2 19	879 900	17
mmu-miR-1892	NM_013593.3	146.00	-20.35	2 19	1037 1058	17
mmu-miR-1892	XM_011243266.1	162.00	-28.77	2 18	3641 3660	16
mmu-miR-1892	XM_011243266.1	152.00	-28.11	2 21	928 949	19
mmu-miR-1892	XM_011243266.1	151.00	-29.44	2 21	1255 1277	20
mmu-miR-1892	XM_011243266.1	142.00	-33.32	2 21	3963 3986	21
mmu-miR-1892	XM_011243266.1	140.00	-33.66	2 21	949 970	19
mmu-miR-1892	XM_011243266.1	140.00	-19.26	2 21	1878 1899	19
mmu-miR-1892	NM_010779.2	148.00	-29.05	2 21	896 917	19
mmu-miR-1892	XM_006504940.3	142.00	-23.71	2 19	205 226	17
mmu-miR-1892	NM_010825.3	155.00	-23.83	2 20	93 114	18
mmu-miR-1892	NM_010825.3	145.00	-20.61	2 18	644 665	16
mmu-miR-1892	NM_010825.3	145.00	-11.86	2 10	1080 1101	8
mmu-miR-1892	NM_010825.3	145.00	-27.24	2 21	2900 2919	19
mmu-miR-1892	NM_010825.3	142.00	-20.74	2 19	1438 1459	17
mmu-miR-1892	NM_010825.3	142.00	-20.09	2 20	1952 1975	20
mmu-miR-1892	NM_001270475.1	164.00	-25.08	2 21	4516 4537	19
mmu-miR-1892	NM_001270475.1	144.00	-29.26	2 21	6493 6512	19
mmu-miR-1892	NM_001270475.1	142.00	-14.07	2 11	4217 4238	9
mmu-miR-1892	NM_001270475.1	142.00	-31.30	2 21	4376 4399	21
mmu-miR-1892	NM_001270475.1	140.00	-13.41	2 9	929 950	7
mmu-miR-1892	NM_177595.4	147.00	-28.05	3 21	215 237	19
mmu-miR-1892	NM_001320077.1	141.00	-22.17	2 18	1133 1154	16
mmu-miR-1892	NM_008623.5	151.00	-24.75	2 21	1936 1958	20
mmu-miR-1892	NM_008623.5	144.00	-27.86	2 20	1306 1327	19
mmu-miR-1892	NM_007641.5	144.00	-26.22	2 21	934 955	19
mmu-miR-1892	XM_006537659.2	140.00	-12.87	2 9	814 835	7
mmu-miR-1892	XM_006529753.2	166.00	-32.19	2 20	1730 1755	22
mmu-miR-1892	NM_175260.2	145.00	-15.10	2 10	1908 1929	8
mmu-miR-1892	NM_010859.2	148.00	-17.35	2 13	101 122	11
mmu-miR-1892	NM_010859.2	145.00	-20.14	2 18	56 77	16
mmu-miR-1892	XM_006513859.3	143.00	-20.10	2 21	2314 2336	20
mmu-miR-1892	XM_006513859.3	140.00	-22.64	2 13	2779 2800	11
mmu-miR-1892	XM_006513859.3	140.00	-20.81	2 17	2853 2874	15
mmu-miR-1892	XM_017320223.1	154.00	-25.30	2 21	5129 5149	19
mmu-miR-1892	XM_017320223.1	146.00	-26.96	2 20	2687 2709	19
mmu-miR-1892	XM_017320223.1	142.00	-25.47	3 20	5041 5066	21
mmu-miR-1892	XM_006501829.3	140.00	-17.48	2 9	705 726	7
mmu-miR-1892	NM_173437.2	156.00	-21.75	2 18	2648 2670	17
mmu-miR-1892	NM_173437.2	151.00	-29.01	2 18	11322 11342	16
mmu-miR-1892	NM_173437.2	149.00	-20.17	2 18	3774 3795	16
mmu-miR-1892	NM_173437.2	149.00	-18.94	2 14	10957 10978	12
mmu-miR-1892	NM_173437.2	143.00	-27.76	2 21	9533 9555	20
mmu-miR-1892	XM_006541297.3	149.00	-29.94	2 21	477 496	19
mmu-miR-1892	XM_006541297.3	147.00	-20.09	2 21	2198 2220	20
mmu-miR-1892	XM_006541297.3	145.00	-12.69	2 10	2000 2021	8
mmu-miR-1892	XM_006541297.3	145.00	-25.86	2 16	2366 2386	14
mmu-miR-1892	XM_006541297.3	143.00	-21.22	2 16	7771 7792	14
mmu-miR-1892	XM_006541297.3	141.00	-22.57	3 18	7176 7197	15
mmu-miR-1892	XM_006541297.3	140.00	-13.35	2 9	4082 4103	7
mmu-miR-1892	NM_001242558.1	145.00	-19.46	2 17	2167 2186	15
mmu-miR-1892	NM_001242558.1	145.00	-15.28	2 18	3025 3046	16
mmu-miR-1892	NM_001242558.1	143.00	-25.06	3 21	1472 1494	19
mmu-miR-1892	NM_001242558.1	140.00	-12.64	2 9	3078 3099	7
mmu-miR-1892	NM_011424.3	143.00	-18.82	2 16	4132 4153	14
mmu-miR-1892	NM_011424.3	143.00	-18.90	2 21	8245 8267	20
mmu-miR-1892	NM_011424.3	142.00	-22.44	2 16	2791 2813	15

mmu-miR-1892	NM_011424.3	140.00	-23.16	2 17	2712 2733	15
mmu-miR-1892	NM_011424.3	140.00	-10.89	2 9	3276 3297	7
mmu-miR-1892	XM_006529456.3	168.00	-27.86	2 21	3466 3487	19
mmu-miR-1892	XM_006498901.3	155.00	-29.30	2 20	296 317	18
mmu-miR-1892	XM_006498901.3	155.00	-26.58	2 21	2723 2749	24
mmu-miR-1892	XM_006498901.3	143.00	-13.57	2 17	577 599	16
mmu-miR-1892	XM_006498901.3	142.00	-28.36	2 21	1665 1690	23
mmu-miR-1892	NM_001134300.2	148.00	-30.12	2 21	695 717	21
mmu-miR-1892	NM_001134300.2	142.00	-31.63	2 21	4330 4350	19
mmu-miR-1892	NM_001134300.2	141.00	-16.70	2 14	3725 3746	12
mmu-miR-1892	NM_001134300.2	140.00	-25.57	2 18	2245 2267	17
mmu-miR-1892	NM_001029877.3	159.00	-24.36	2 19	5165 5184	17
mmu-miR-1892	NM_001029877.3	156.00	-27.08	3 21	1716 1737	18
mmu-miR-1892	NM_001029877.3	149.00	-20.75	2 20	540 560	18
mmu-miR-1892	NM_001029877.3	143.00	-25.00	3 21	588 610	19
mmu-miR-1892	NM_001029877.3	142.00	-27.97	2 21	3411 3431	19
mmu-miR-1892	NM_001029877.3	142.00	-28.25	3 21	6570 6594	21
mmu-miR-1892	NM_001029877.3	141.00	-26.17	2 20	3461 3481	18
mmu-miR-1892	NM_001029877.3	140.00	-14.61	2 9	3707 3728	7
mmu-miR-1892	NM_001029877.3	140.00	-27.26	2 21	7453 7474	19
mmu-miR-1892	NM_001077403.1	141.00	-24.49	2 18	6055 6076	16
mmu-miR-1892	XM_006530325.3	149.00	-26.99	2 18	3476 3497	16
mmu-miR-1892	XM_006530325.3	145.00	-14.73	2 10	778 799	8
mmu-miR-1892	XM_006530325.3	144.00	-15.56	2 20	1894 1913	18
mmu-miR-1892	NM_145226.2	149.00	-24.27	3 18	4699 4718	15
mmu-miR-1892	NM_145226.2	142.00	-27.60	2 21	575 598	21
mmu-miR-1892	XM_006530294.3	145.00	-29.12	3 21	2153 2175	20
mmu-miR-1892	NM_011854.2	151.00	-28.04	2 21	1132 1154	20
mmu-miR-1892	NM_011854.2	146.00	-19.17	2 19	2690 2711	17
mmu-miR-1892	NM_011854.2	143.00	-29.90	2 21	2291 2314	21
mmu-miR-1892	NM_001310636.1	149.00	-22.11	2 21	544 565	20
mmu-miR-1892	NM_172907.3	149.00	-23.25	2 18	1673 1694	16
mmu-miR-1892	XM_011239969.2	142.00	-15.74	2 14	88 107	12
mmu-miR-1892	XM_011239969.2	140.00	-18.20	2 21	1446 1467	19
mmu-miR-1892	NM_001286743.1	159.00	-38.71	2 21	3 26	21
mmu-miR-1892	NM_001286743.1	157.00	-30.30	3 18	2553 2574	15
mmu-miR-1892	NM_001286743.1	154.00	-30.96	2 21	3449 3469	19
mmu-miR-1892	NM_001286743.1	152.00	-32.75	2 21	3761 3782	19
mmu-miR-1892	NM_001286743.1	145.00	-21.86	3 18	2765 2786	15
mmu-miR-1892	NM_001286743.1	141.00	-21.10	2 19	2195 2217	18
mmu-miR-1892	XM_017322026.1	142.00	-30.12	2 20	483 505	19
mmu-miR-5113	XM_006509531.3	155.00	-21.00	2 22	1815 1841	24
mmu-miR-5113	XM_006524407.3	149.00	-18.80	2 19	568 591	18
mmu-miR-5113	XM_006524407.3	142.00	-23.34	2 22	1532 1554	21
mmu-miR-5113	XM_006524407.3	141.00	-20.72	2 10	794 816	8
mmu-miR-5113	NM_177743.5	140.00	-13.76	2 9	2434 2456	7
mmu-miR-5113	XM_017319626.1	153.00	-19.67	2 19	4024 4047	18
mmu-miR-5113	XM_017319626.1	148.00	-22.12	2 20	3701 3723	19
mmu-miR-5113	XM_006532962.2	155.00	-24.54	2 22	3318 3342	22
mmu-miR-5113	XM_006532962.2	144.00	-20.17	2 21	2288 2310	19
mmu-miR-5113	NM_001160262.1	151.00	-18.72	2 20	2981 3003	18
mmu-miR-5113	NM_001160262.1	149.00	-22.73	3 19	2102 2125	17
mmu-miR-5113	NM_001160262.1	142.00	-20.44	2 21	4621 4647	23
mmu-miR-5113	NM_010181.2	149.00	-31.51	2 22	3456 3478	20
mmu-miR-5113	NM_010181.2	146.00	-16.81	2 19	7719 7741	17
mmu-miR-5113	XM_011244488.2	141.00	-22.86	2 14	465 487	12
mmu-miR-5113	XM_011244488.2	140.00	-15.45	2 9	1424 1446	7
mmu-miR-5113	NM_145141.2	159.00	-25.76	2 22	893 914	20
mmu-miR-5113	NM_145141.2	140.00	-20.37	3 17	672 694	14
mmu-miR-5113	XM_006527803.1	152.00	-23.29	2 17	1719 1741	15
mmu-miR-5113	XM_006527803.1	148.00	-21.73	2 22	2407 2430	21
mmu-miR-5113	XM_006496949.3	162.00	-24.75	2 21	1689 1710	19
mmu-miR-5113	XM_006496949.3	151.00	-25.82	2 22	3838 3864	24
mmu-miR-5113	XM_006496949.3	140.00	-16.01	2 21	535 555	19
mmu-miR-5113	NM_172862.3	153.00	-24.59	3 19	12192 12216	18

mmu-miR-5113	NM_172862.3	146.00	-21.30	2 20	8244 8267	19
mmu-miR-5113	NM_172862.3	144.00	-26.26	3 17	1379 1401	14
mmu-miR-5113	NM_172862.3	140.00	-20.42	2 17	10346 10368	15
mmu-miR-5113	XM_006533539.3	155.00	-21.30	2 17	8248 8271	16
mmu-miR-5113	XM_006533539.3	154.00	-19.70	2 19	5511 5533	17
mmu-miR-5113	XM_006533539.3	153.00	-22.48	2 18	6347 6369	16
mmu-miR-5113	XM_006533539.3	145.00	-24.19	3 18	5468 5490	15
mmu-miR-5113	NM_001033221.3	156.00	-21.84	2 17	1355 1377	15
mmu-miR-5113	NM_001033221.3	151.00	-21.90	2 22	1610 1631	20
mmu-miR-5113	NM_001033221.3	146.00	-24.27	3 21	1941 1962	18
mmu-miR-5113	NM_001033221.3	142.00	-18.07	2 19	754 776	17
mmu-miR-5113	NM_001033221.3	141.00	-21.40	2 19	775 798	18
mmu-miR-5113	NM_008067.4	142.00	-18.04	3 19	191 213	16
mmu-miR-5113	NM_001033445.2	149.00	-20.92	3 20	4169 4190	17
mmu-miR-5113	NM_001033445.2	141.00	-23.98	2 22	4579 4607	26
mmu-miR-5113	NM_145741.2	154.00	-29.26	2 21	1478 1505	24
mmu-miR-5113	NM_010279.3	145.00	-18.80	2 10	3546 3568	8
mmu-miR-5113	XM_006510297.3	169.00	-26.92	2 22	2325 2347	20
mmu-miR-5113	XM_006510297.3	152.00	-27.70	2 22	2293 2316	21
mmu-miR-5113	XM_006510297.3	149.00	-21.09	2 19	1077 1100	18
mmu-miR-5113	XM_006510297.3	142.00	-22.29	2 21	2427 2448	19
mmu-miR-5113	XM_006530353.3	149.00	-18.75	2 19	2197 2220	18
mmu-miR-5113	XM_006530353.3	144.00	-22.57	2 19	4150 4171	17
mmu-miR-5113	XM_006530353.3	140.00	-17.16	3 21	6187 6209	18
mmu-miR-5113	XM_006530353.3	140.00	-19.91	2 9	14948 14970	7
mmu-miR-5113	XM_011248576.2	169.00	-29.12	2 22	135 157	20
mmu-miR-5113	XM_011248576.2	151.00	-21.15	2 17	6283 6306	16
mmu-miR-5113	XM_011248576.2	146.00	-19.19	2 11	1614 1636	9
mmu-miR-5113	XM_011248576.2	145.00	-20.48	2 18	5138 5160	16
mmu-miR-5113	XM_011248576.2	145.00	-16.90	2 10	7694 7716	8
mmu-miR-5113	XM_011248576.2	141.00	-19.81	2 22	8097 8119	20
mmu-miR-5113	XM_011248576.2	140.00	-14.74	2 21	165 187	19
mmu-miR-5113	NM_001198955.1	161.00	-22.46	2 19	2849 2873	19
mmu-miR-5113	NM_001198955.1	160.00	-28.82	2 22	1981 2005	22
mmu-miR-5113	NM_001198955.1	157.00	-20.96	2 18	1081 1103	16
mmu-miR-5113	NM_001198955.1	150.00	-25.27	2 21	1140 1161	19
mmu-miR-5113	NM_001198955.1	147.00	-16.55	2 14	1422 1443	12
mmu-miR-5113	NM_001198955.1	144.00	-22.00	3 21	2083 2105	18
mmu-miR-5113	NM_001198955.1	144.00	-20.86	3 18	2561 2584	16
mmu-miR-5113	XM_006532232.3	154.00	-21.06	2 19	2015 2037	17
mmu-miR-5113	XM_006532232.3	140.00	-26.62	3 22	783 810	24
mmu-miR-5113	NM_016697.3	152.00	-22.35	2 17	2203 2225	15
mmu-miR-5113	NM_016697.3	142.00	-27.12	2 21	60 81	19
mmu-miR-5113	XM_011250978.2	150.00	-20.02	2 19	2084 2106	17
mmu-miR-5113	NM_010340.2	150.00	-20.81	2 11	1398 1420	9
mmu-miR-5113	XM_006521554.3	149.00	-14.87	2 18	1850 1872	16
mmu-miR-5113	XM_006521554.3	148.00	-21.10	3 22	1169 1194	22
mmu-miR-5113	XM_006515096.2	163.00	-25.83	2 22	3213 3234	20
mmu-miR-5113	XM_006515096.2	149.00	-22.75	2 20	7411 7436	21
mmu-miR-5113	XM_006515096.2	145.00	-27.19	2 22	1780 1802	20
mmu-miR-5113	XM_006515096.2	142.00	-16.48	2 11	6837 6859	9
mmu-miR-5113	XM_006526069.2	141.00	-15.13	2 19	1810 1833	18
mmu-miR-5113	XM_006526069.2	140.00	-14.39	2 9	1149 1171	7
mmu-miR-5113	XM_006526069.2	140.00	-18.88	3 20	5281 5303	18
mmu-miR-5113	NM_008204.2	144.00	-24.28	3 22	10 33	20
mmu-miR-5113	XM_011246309.2	143.00	-15.68	2 16	1582 1604	14
mmu-miR-5113	XM_011246309.2	142.00	-20.60	2 22	194 214	20
mmu-miR-5113	XM_011246309.2	141.00	-20.35	2 10	775 797	8
mmu-miR-5113	NM_175520.4	157.00	-25.08	2 15	1130 1153	14
mmu-miR-5113	NM_175520.4	142.00	-14.93	2 19	816 838	17
mmu-miR-5113	NM_175520.4	140.00	-18.74	2 22	2771 2797	24
mmu-miR-5113	NM_001162950.1	160.00	-26.64	2 21	2119 2139	19
mmu-miR-5113	NM_001162950.1	153.00	-17.93	2 18	3977 3999	16
mmu-miR-5113	NM_001162950.1	149.00	-19.57	2 22	3669 3691	20
mmu-miR-5113	NM_001162950.1	145.00	-18.32	3 18	2458 2480	15

mmu-miR-5113	NM_001162950.1	142.00	-21.98	2 22	2949 2971	21
mmu-miR-5113	NM_001162950.1	141.00	-18.81	2 18	4006 4028	16
mmu-miR-5113	NM_001162950.1	140.00	-20.47	2 18	3328 3352	18
mmu-miR-5113	NM_001162950.1	140.00	-18.59	2 9	4618 4640	7
mmu-miR-5113	XM_006505602.3	173.00	-29.20	2 22	7087 7109	20
mmu-miR-5113	XM_006505602.3	166.00	-24.60	2 21	13915 13936	19
mmu-miR-5113	XM_006505602.3	147.00	-15.67	2 21	7828 7852	21
mmu-miR-5113	XM_006505602.3	144.00	-22.16	2 19	9052 9077	20
mmu-miR-5113	NM_001164086.1	164.00	-31.99	2 22	1320 1343	21
mmu-miR-5113	NM_001164086.1	153.00	-28.86	2 18	8904 8926	16
mmu-miR-5113	NM_001164086.1	149.00	-15.73	2 22	4392 4414	20
mmu-miR-5113	NM_001164086.1	148.00	-19.93	2 22	10361 10384	21
mmu-miR-5113	NM_010450.3	140.00	-22.54	3 22	106 129	20
mmu-miR-5113	NM_001327998.1	146.00	-19.51	2 20	5152 5177	21
mmu-miR-5113	NM_001327998.1	140.00	-29.87	2 22	3954 3978	22
mmu-miR-5113	XM_006539957.3	149.00	-17.60	2 22	1172 1194	20
mmu-miR-5113	XM_006539957.3	148.00	-15.72	2 21	995 1017	19
mmu-miR-5113	NM_013868.4	174.00	-29.33	2 19	355 377	17
mmu-miR-5113	NM_013868.4	146.00	-23.96	3 17	1062 1083	14
mmu-miR-5113	NM_030704.3	152.00	-20.91	2 17	539 561	15
mmu-miR-5113	NM_133871.2	151.00	-17.68	2 16	1580 1602	14
mmu-miR-5113	XM_006511311.2	163.00	-27.65	2 22	3814 3835	20
mmu-miR-5113	XM_006511311.2	146.00	-20.87	2 19	4369 4391	17
mmu-miR-5113	XM_006511311.2	145.00	-18.28	2 10	1539 1561	8
mmu-miR-5113	XM_006511311.2	142.00	-23.67	2 21	2966 2993	24
mmu-miR-5113	XM_006523711.3	158.00	-21.14	2 20	39 62	19
mmu-miR-5113	NM_001159424.2	145.00	-18.25	3 19	162 185	17
mmu-miR-5113	XM_006496593.3	141.00	-22.06	2 22	1097 1116	20
mmu-miR-5113	XM_006496593.3	140.00	-16.18	2 9	4021 4043	7
mmu-miR-5113	XM_006528842.1	160.00	-24.65	2 18	4967 4990	17
mmu-miR-5113	XM_006528842.1	152.00	-21.00	2 22	5255 5280	23
mmu-miR-5113	XM_006528842.1	151.00	-28.23	3 20	2517 2539	17
mmu-miR-5113	XM_006528842.1	142.00	-20.75	2 21	1549 1570	19
mmu-miR-5113	XM_006528842.1	142.00	-19.16	3 19	5128 5150	16
mmu-miR-5113	NM_001161541.1	142.00	-26.21	3 22	2668 2688	19
mmu-miR-5113	NM_001302471.1	151.00	-21.71	2 20	4949 4971	18
mmu-miR-5113	NM_001302471.1	149.00	-22.27	2 19	4274 4297	18
mmu-miR-5113	NM_001302471.1	146.00	-33.52	3 20	99 122	18
mmu-miR-5113	NM_001302471.1	145.00	-20.56	2 20	3456 3477	18
mmu-miR-5113	NM_001289875.1	168.00	-24.40	2 19	3186 3207	17
mmu-miR-5113	NM_001289875.1	153.00	-19.88	2 22	616 638	20
mmu-miR-5113	NM_008423.2	156.00	-23.83	2 22	3028 3051	21
mmu-miR-5113	NM_008423.2	148.00	-19.93	2 18	3637 3660	17
mmu-miR-5113	NM_008423.2	143.00	-20.32	2 19	2830 2856	21
mmu-miR-5113	NM_021342.1	144.00	-24.93	2 21	654 676	19
mmu-miR-5113	NM_021342.1	142.00	-17.60	2 11	1819 1841	9
mmu-miR-5113	XM_017315734.1	140.00	-21.57	3 22	4727 4749	21
mmu-miR-5113	NM_010608.2	160.00	-21.11	2 17	1858 1880	15
mmu-miR-5113	NM_010608.2	140.00	-23.25	3 18	1842 1865	16
mmu-miR-5113	NM_010608.2	140.00	-19.94	3 19	2984 3008	18
mmu-miR-5113	XM_006497892.3	152.00	-17.10	2 13	478 500	11
mmu-miR-5113	XM_006497892.3	140.00	-15.28	2 9	4861 4883	7
mmu-miR-5113	XM_017316478.1	168.00	-28.66	2 22	5887 5910	21
mmu-miR-5113	NM_031180.2	149.00	-20.61	2 22	958 978	20
mmu-miR-5113	NM_031180.2	140.00	-16.93	2 9	2837 2859	7
mmu-miR-5113	NM_031180.2	140.00	-20.83	2 18	3371 3394	17
mmu-miR-5113	NM_173427.2	155.00	-23.95	2 21	358 381	20
mmu-miR-5113	NM_173427.2	145.00	-25.82	2 22	5549 5568	20
mmu-miR-5113	XM_006512296.3	143.00	-19.43	2 22	453 478	23
mmu-miR-5113	XM_006512296.3	143.00	-19.81	2 16	2397 2419	14
mmu-miR-5113	NM_008940.3	143.00	-20.96	2 22	109 130	20
mmu-miR-5113	NM_016659.3	148.00	-22.13	2 18	728 751	17
mmu-miR-5113	NM_016659.3	142.00	-18.96	2 19	308 330	17
mmu-miR-5113	NM_029274.2	167.00	-26.31	2 22	5629 5653	22
mmu-miR-5113	NM_029274.2	155.00	-24.61	2 22	6839 6860	20

mmu-miR-5113	NM_029274.2	153.00	-30.82	3 22	1398 1420	19
mmu-miR-5113	NM_029274.2	149.00	-23.97	3 22	5777 5797	19
mmu-miR-5113	NM_029274.2	142.00	-21.57	3 15	2022 2044	12
mmu-miR-5113	NM_029274.2	140.00	-19.96	3 13	1275 1297	10
mmu-miR-5113	XM_017316676.1	169.00	-26.03	2 18	17592 17614	16
mmu-miR-5113	XM_017316676.1	161.00	-26.81	2 22	12799 12821	20
mmu-miR-5113	XM_017316676.1	146.00	-22.55	2 11	1800 1822	9
mmu-miR-5113	XM_017316676.1	146.00	-21.02	2 11	1989 2011	9
mmu-miR-5113	XM_017316676.1	143.00	-28.96	2 22	17988 18010	21
mmu-miR-5113	NM_146063.1	150.00	-20.69	2 11	330 352	9
mmu-miR-5113	NM_008479.2	140.00	-28.02	2 18	231 255	18
mmu-miR-5113	XM_017313062.1	164.00	-21.85	2 22	233 256	21
mmu-miR-5113	XM_017313062.1	149.00	-27.30	2 22	3130 3149	20
mmu-miR-5113	NM_144945.3	149.00	-16.84	2 14	2540 2562	12
mmu-miR-5113	NM_144945.3	147.00	-20.36	2 21	3790 3813	20
mmu-miR-5113	NM_010714.3	150.00	-24.24	2 22	4431 4453	21
mmu-miR-5113	NM_053098.2	149.00	-21.83	3 18	1399 1421	15
mmu-miR-5113	NM_175271.4	154.00	-20.43	2 21	1180 1201	19
mmu-miR-5113	NM_175271.4	149.00	-25.73	2 22	374 396	20
mmu-miR-5113	NM_175271.4	147.00	-18.31	2 21	1203 1226	20
mmu-miR-5113	XM_006506340.3	177.00	-28.85	2 18	2947 2969	16
mmu-miR-5113	XM_006506340.3	144.00	-20.26	2 17	2838 2860	15
mmu-miR-5113	NM_028973.2	143.00	-24.18	3 20	20 42	17
mmu-miR-5113	NM_028973.2	142.00	-19.64	2 20	2666 2689	19
mmu-miR-5113	NM_028838.2	141.00	-20.62	2 19	1737 1760	18
mmu-miR-5113	XM_006527321.2	148.00	-20.10	2 14	560 583	13
mmu-miR-5113	XM_006527321.2	145.00	-18.97	2 22	1277 1299	20
mmu-miR-5113	XM_006527321.2	142.00	-20.88	2 21	1364 1385	19
mmu-miR-5113	XM_006506086.2	145.00	-20.71	3 22	1691 1713	19
mmu-miR-5113	NM_008524.2	159.00	-27.44	2 22	922 947	23
mmu-miR-5113	NM_008524.2	148.00	-18.54	2 14	1977 2000	13
mmu-miR-5113	NM_001171187.1	168.00	-28.03	2 21	410 432	19
mmu-miR-5113	XM_006541232.3	175.00	-26.20	2 20	402 424	18
mmu-miR-5113	XM_006541232.3	161.00	-27.60	2 22	1543 1565	20
mmu-miR-5113	XM_006541232.3	146.00	-23.96	3 17	5898 5919	14
mmu-miR-5113	XM_006498872.3	164.00	-26.99	2 21	7241 7263	19
mmu-miR-5113	XM_006498872.3	154.00	-26.48	2 15	3248 3270	13
mmu-miR-5113	XM_006498872.3	153.00	-24.11	2 22	7570 7589	20
mmu-miR-5113	XM_006498872.3	143.00	-15.84	2 22	9123 9150	25
mmu-miR-5113	XM_006532410.3	152.00	-25.73	2 18	6269 6293	18
mmu-miR-5113	XM_006532410.3	148.00	-23.16	2 17	290 312	15
mmu-miR-5113	XM_006520023.2	165.00	-24.67	2 22	149 171	20
mmu-miR-5113	NM_001252563.1	147.00	-28.44	2 22	1176 1197	20
mmu-miR-5113	XM_011243266.1	168.00	-27.53	2 22	4018 4042	22
mmu-miR-5113	XM_011243266.1	145.00	-22.00	3 22	1188 1210	19
mmu-miR-5113	XM_011243266.1	144.00	-28.73	2 22	1218 1241	21
mmu-miR-5113	NM_010779.2	141.00	-15.58	2 10	405 427	8
mmu-miR-5113	XM_006504940.3	142.00	-23.38	2 22	972 999	25
mmu-miR-5113	NM_010825.3	156.00	-23.69	2 21	573 595	19
mmu-miR-5113	NM_010825.3	149.00	-18.86	2 22	488 510	20
mmu-miR-5113	NM_010825.3	140.00	-13.76	2 9	1856 1878	7
mmu-miR-5113	NM_001270475.1	165.00	-25.39	2 20	8285 8306	18
mmu-miR-5113	NM_001270475.1	148.00	-24.68	2 22	3175 3198	21
mmu-miR-5113	NM_001270475.1	146.00	-21.35	2 21	5517 5538	19
mmu-miR-5113	NM_001270475.1	145.00	-20.60	2 10	2815 2837	8
mmu-miR-5113	NM_001290512.1	161.00	-19.76	2 19	2433 2456	18
mmu-miR-5113	NM_177595.4	144.00	-18.27	3 18	1789 1812	16
mmu-miR-5113	NM_001005423.2	145.00	-21.27	2 19	295 318	18
mmu-miR-5113	NM_205810.4	145.00	-22.28	2 22	1044 1066	20
mmu-miR-5113	NM_205810.4	142.00	-18.78	3 19	2413 2435	16
mmu-miR-5113	NM_205810.4	141.00	-21.63	3 22	884 906	19
mmu-miR-5113	XM_006537659.2	147.00	-22.33	2 22	4461 4482	20
mmu-miR-5113	XM_006537659.2	141.00	-20.23	2 18	1882 1904	16
mmu-miR-5113	XM_006537659.2	141.00	-15.26	2 14	2801 2823	12
mmu-miR-5113	NM_175260.2	148.00	-19.48	2 17	7233 7255	15

mmu-miR-5113	NM_175260.2	140.00	-20.71	2 17	643 665	15
mmu-miR-5113	NM_175260.2	140.00	-21.49	2 21	6716 6738	19
mmu-miR-5113	NM_001039545.2	141.00	-18.25	2 20	1981 2002	18
mmu-miR-5113	NM_001039545.2	140.00	-22.22	2 17	472 494	15
mmu-miR-5113	XM_017315841.1	140.00	-21.87	2 17	507 529	15
mmu-miR-5113	XM_006530186.3	161.00	-28.78	2 22	822 848	24
mmu-miR-5113	NM_010859.2	144.00	-19.39	3 17	27 49	14
mmu-miR-5113	NM_010859.2	140.00	-24.51	3 17	11 33	14
mmu-miR-5113	XM_006513859.3	165.00	-23.49	2 22	3433 3455	20
mmu-miR-5113	XM_006513859.3	161.00	-29.13	2 19	30 54	19
mmu-miR-5113	XM_006513859.3	142.00	-20.94	2 16	3463 3486	15
mmu-miR-5113	XM_017320223.1	140.00	-22.26	2 13	1735 1757	11
mmu-miR-5113	XM_017317978.1	167.00	-21.25	2 20	314 336	18
mmu-miR-5113	XM_017317978.1	149.00	-16.40	2 18	63 85	16
mmu-miR-5113	NM_173437.2	160.00	-23.55	2 22	5707 5730	21
mmu-miR-5113	NM_173437.2	153.00	-22.62	2 22	425 447	20
mmu-miR-5113	NM_173437.2	153.00	-20.46	2 18	3834 3856	16
mmu-miR-5113	NM_173437.2	153.00	-18.51	2 19	12740 12765	20
mmu-miR-5113	NM_173437.2	147.00	-20.10	2 22	12652 12679	25
mmu-miR-5113	NM_173437.2	146.00	-23.20	2 15	1761 1783	13
mmu-miR-5113	NM_173437.2	142.00	-24.15	3 19	9025 9047	16
mmu-miR-5113	NM_173437.2	141.00	-18.24	3 19	5883 5906	17
mmu-miR-5113	XM_006541297.3	164.00	-23.47	2 18	1096 1119	17
mmu-miR-5113	XM_006541297.3	151.00	-27.22	2 22	2904 2925	20
mmu-miR-5113	XM_006541297.3	145.00	-25.05	2 22	4932 4954	20
mmu-miR-5113	XM_006541297.3	144.00	-27.92	2 22	8928 8951	21
mmu-miR-5113	NM_011424.3	162.00	-21.02	2 19	3652 3674	17
mmu-miR-5113	XM_006529456.3	142.00	-16.89	3 19	1514 1536	16
mmu-miR-5113	XM_006510077.3	143.00	-26.18	2 21	2365 2388	20
mmu-miR-5113	XM_006510077.3	141.00	-15.57	2 19	2157 2180	18
mmu-miR-5113	NM_001134300.2	172.00	-29.89	2 22	1043 1066	21
mmu-miR-5113	NM_001134300.2	147.00	-16.65	3 22	4952 4973	19
mmu-miR-5113	NM_001134300.2	141.00	-27.26	2 22	3476 3498	20
mmu-miR-5113	NM_001109985.1	142.00	-18.64	3 21	1273 1297	20
mmu-miR-5113	NM_181547.3	143.00	-26.80	2 22	1082 1103	20
mmu-miR-5113	NM_001029877.3	163.00	-27.30	2 18	7117 7141	18
mmu-miR-5113	NM_001029877.3	153.00	-27.74	3 22	6808 6830	19
mmu-miR-5113	NM_001029877.3	151.00	-18.22	2 12	4773 4795	10
mmu-miR-5113	NM_001029877.3	150.00	-22.50	3 20	7410 7433	18
mmu-miR-5113	NM_001029877.3	148.00	-21.66	3 17	1699 1721	14
mmu-miR-5113	NM_001029877.3	145.00	-16.46	3 18	5598 5620	15
mmu-miR-5113	NM_001029877.3	141.00	-18.37	3 19	2324 2347	17
mmu-miR-5113	NM_001029877.3	140.00	-16.46	2 17	5200 5222	15
mmu-miR-5113	NM_023456.3	141.00	-19.61	2 19	428 451	18
mmu-miR-5113	NM_001077403.1	151.00	-20.89	3 22	3398 3419	19
mmu-miR-5113	NM_001077403.1	144.00	-17.67	2 13	106 128	11
mmu-miR-5113	NM_001077403.1	143.00	-23.52	2 22	1766 1790	22
mmu-miR-5113	NM_198190.1	141.00	-24.31	3 20	245 271	21
mmu-miR-5113	NM_001282961.1	149.00	-20.01	3 22	5219 5241	19
mmu-miR-5113	NM_145210.2	147.00	-32.89	2 22	1151 1175	22
mmu-miR-5113	XM_006530325.3	140.00	-22.61	2 17	2880 2902	15
mmu-miR-5113	XM_006530294.3	160.00	-21.90	2 18	1826 1850	18
mmu-miR-5113	XM_006530294.3	159.00	-27.92	2 22	1024 1045	20
mmu-miR-5113	XM_006530294.3	150.00	-20.97	2 15	1759 1781	13
mmu-miR-5113	XM_006530294.3	141.00	-27.80	2 22	1970 1993	21
mmu-miR-5113	NM_001310636.1	146.00	-22.03	2 20	1721 1744	19
mmu-miR-5113	NM_008760.4	157.00	-20.00	2 20	2179 2200	18
mmu-miR-5113	NM_008760.4	142.00	-18.25	2 19	2331 2353	17
mmu-miR-5113	XM_006516928.2	144.00	-19.31	2 19	3092 3113	17
mmu-miR-5113	XM_006516928.2	141.00	-17.25	3 22	1375 1397	19
mmu-miR-5113	XM_006516928.2	140.00	-15.45	2 22	4110 4133	21
mmu-miR-5113	XM_011239969.2	140.00	-18.44	2 13	3224 3246	11
mmu-miR-5113	XM_017322026.1	146.00	-19.45	3 19	1241 1261	16
mmu-miR-5113	XM_017322026.1	145.00	-30.43	2 22	698 720	20
mmu-miR-5128	XM_006532962.2	142.00	-17.65	2 21	3173 3194	19

mmu-miR-5128	NM_178908.3	145.00	-15.57	2 10	1523 1545	8
mmu-miR-5128	NM_001160262.1	145.00	-21.95	2 22	2507 2529	20
mmu-miR-5128	NM_145141.2	147.00	-24.34	2 18	572 593	16
mmu-miR-5128	NM_001081185.1	140.00	-21.51	2 22	1056 1080	22
mmu-miR-5128	XM_006496949.3	141.00	-14.97	2 12	2515 2536	10
mmu-miR-5128	NM_172862.3	157.00	-27.03	2 22	10915 10937	20
mmu-miR-5128	NM_001033445.2	140.00	-17.29	2 9	1459 1481	7
mmu-miR-5128	NM_145741.2	140.00	-25.19	2 22	990 1013	21
mmu-miR-5128	XM_006530353.3	151.00	-22.08	2 13	2722 2745	12
mmu-miR-5128	XM_006530353.3	141.00	-26.06	2 22	15118 15140	20
mmu-miR-5128	NM_001024731.2	158.00	-16.20	2 20	2450 2475	21
mmu-miR-5128	XM_011248576.2	146.00	-22.41	2 22	10976 10999	22
mmu-miR-5128	XM_011248576.2	142.00	-28.38	2 22	13045 13069	23
mmu-miR-5128	XM_011248576.2	141.00	-31.61	2 18	9863 9885	16
mmu-miR-5128	XM_006532232.3	150.00	-25.43	2 15	1806 1828	13
mmu-miR-5128	XM_006521554.3	172.00	-37.49	2 22	32 55	21
mmu-miR-5128	XM_006521554.3	145.00	-17.14	2 22	1183 1205	20
mmu-miR-5128	XM_006526069.2	145.00	-18.56	2 22	1637 1659	20
mmu-miR-5128	XM_006526069.2	145.00	-22.12	2 22	4965 4987	20
mmu-miR-5128	XM_006499444.3	140.00	-9.48	2 9	1632 1654	7
mmu-miR-5128	NM_001162950.1	144.00	-21.55	3 20	3866 3888	18
mmu-miR-5128	XM_006505602.3	166.00	-28.09	2 20	7347 7370	19
mmu-miR-5128	XM_006505602.3	143.00	-24.53	3 18	9465 9486	15
mmu-miR-5128	NM_001164086.1	143.00	-24.33	3 22	3060 3084	21
mmu-miR-5128	NM_001164086.1	141.00	-19.43	2 22	2546 2568	20
mmu-miR-5128	NM_013868.4	154.00	-19.66	2 22	2290 2312	21
mmu-miR-5128	NM_030704.3	143.00	-20.28	2 22	192 213	20
mmu-miR-5128	XM_006511311.2	141.00	-17.19	2 21	4390 4410	19
mmu-miR-5128	NM_001190325.1	149.00	-27.52	2 22	347 366	20
mmu-miR-5128	XM_017316439.1	143.00	-15.32	2 17	1764 1787	16
mmu-miR-5128	XM_006528842.1	152.00	-24.62	3 22	4632 4655	20
mmu-miR-5128	XM_006528842.1	144.00	-22.68	3 22	2366 2389	20
mmu-miR-5128	XM_006528842.1	142.00	-19.73	2 20	558 581	19
mmu-miR-5128	XM_006528842.1	140.00	-23.32	3 22	2293 2316	20
mmu-miR-5128	NM_001081667.2	146.00	-20.43	2 22	750 773	22
mmu-miR-5128	NM_008940.3	142.00	-19.42	2 21	394 415	19
mmu-miR-5128	NM_029274.2	151.00	-22.93	2 22	1330 1351	20
mmu-miR-5128	NM_029274.2	140.00	-10.54	2 9	4026 4048	7
mmu-miR-5128	XM_017316676.1	167.00	-27.45	2 22	17455 17476	20
mmu-miR-5128	XM_017316676.1	141.00	-24.21	2 22	1534 1554	20
mmu-miR-5128	XM_017316676.1	141.00	-22.33	2 15	4863 4887	15
mmu-miR-5128	XM_017316676.1	141.00	-26.90	2 22	7274 7296	20
mmu-miR-5128	XM_017316676.1	141.00	-22.87	3 22	12673 12693	19
mmu-miR-5128	XM_017316676.1	140.00	-26.42	3 18	6758 6781	16
mmu-miR-5128	XM_017315313.1	147.00	-15.53	2 12	2256 2278	10
mmu-miR-5128	NM_144945.3	149.00	-20.86	3 22	1444 1466	19
mmu-miR-5128	NM_010714.3	144.00	-17.81	2 17	1503 1525	15
mmu-miR-5128	NM_144799.2	144.00	-24.71	2 13	908 930	11
mmu-miR-5128	NM_144799.2	140.00	-16.45	2 20	4 24	18
mmu-miR-5128	NM_175271.4	142.00	-12.87	2 20	1416 1435	18
mmu-miR-5128	NM_175271.4	140.00	-18.96	2 22	352 375	21
mmu-miR-5128	NM_028973.2	145.00	-22.18	2 20	504 525	18
mmu-miR-5128	NM_028973.2	143.00	-29.59	2 22	5134 5160	24
mmu-miR-5128	XM_006506086.2	153.00	-22.34	2 21	1220 1244	22
mmu-miR-5128	XM_006506086.2	148.00	-23.07	2 21	2074 2096	19
mmu-miR-5128	NM_008524.2	149.00	-18.76	2 22	88 110	20
mmu-miR-5128	XM_006498872.3	143.00	-24.75	2 16	9759 9781	14
mmu-miR-5128	XM_006532410.3	147.00	-22.74	2 22	4331 4352	20
mmu-miR-5128	XM_006520023.2	147.00	-19.11	2 12	2219 2241	10
mmu-miR-5128	NM_013593.3	147.00	-20.34	2 12	97 119	10
mmu-miR-5128	XM_011243266.1	143.00	-21.45	2 21	3892 3915	20
mmu-miR-5128	XM_011243266.1	140.00	-32.64	2 18	1540 1563	17
mmu-miR-5128	NM_001290512.1	151.00	-21.93	2 21	595 618	20
mmu-miR-5128	NM_177595.4	154.00	-16.92	2 22	2219 2239	20
mmu-miR-5128	NM_177595.4	150.00	-28.82	3 21	214 238	20

mmu-miR-5128	NM_001320077.1	177.00	-37.30	2 22	1890 1912	20
mmu-miR-5128	XM_006530186.3	143.00	-17.48	2 16	333 355	14
mmu-miR-5128	XM_006513859.3	142.00	-24.90	2 22	1962 1984	21
mmu-miR-5128	XM_017320223.1	141.00	-25.93	4 22	2526 2548	18
mmu-miR-5128	XM_006541297.3	147.00	-29.33	2 22	498 524	24
mmu-miR-5128	XM_006541297.3	144.00	-26.22	2 18	9013 9036	17
mmu-miR-5128	NM_001242558.1	142.00	-19.89	3 20	720 743	18
mmu-miR-5128	NM_011424.3	152.00	-29.16	2 18	5382 5405	17
mmu-miR-5128	XM_006529456.3	143.00	-15.20	2 22	1903 1924	20
mmu-miR-5128	NM_001134300.2	143.00	-19.21	2 16	1932 1954	14
mmu-miR-5128	NM_001029877.3	144.00	-25.25	3 18	6572 6595	16
mmu-miR-5128	NM_001077403.1	146.00	-16.77	2 17	1829 1853	17
mmu-miR-5128	NM_001077403.1	144.00	-23.93	3 22	2895 2918	20
mmu-miR-5128	NM_001077403.1	143.00	-18.30	3 20	1339 1361	17
mmu-miR-5128	NM_001282961.1	144.00	-21.47	2 22	1186 1209	21
mmu-miR-5128	NM_145210.2	142.00	-20.13	3 20	39 63	19
mmu-miR-5128	NM_145226.2	146.00	-23.96	2 22	1455 1481	24
mmu-miR-5128	NM_145226.2	140.00	-22.84	2 22	3130 3155	23
mmu-miR-5128	NM_001310636.1	140.00	-19.54	2 22	1101 1124	21
mmu-miR-5128	NM_172907.3	141.00	-12.41	2 16	1389 1410	14
mmu-miR-5128	XM_006516928.2	146.00	-13.96	2 19	3317 3339	17
mmu-miR-5128	XM_006516928.2	144.00	-18.69	2 21	557 579	19
mmu-miR-5128	XM_011239969.2	144.00	-14.70	2 21	6300 6322	19
mmu-miR-5128	NM_001286743.1	144.00	-25.20	2 22	149 172	21
mmu-miR-5128	NM_001286743.1	143.00	-25.67	3 18	2554 2575	15
mmu-miR-344i	XM_006524407.3	158.00	-30.00	2 19	1668 1687	17
mmu-miR-344i	XM_017319626.1	140.00	-15.02	2 9	570 589	7
mmu-miR-344i	XM_006532962.2	149.00	-22.57	2 16	682 700	14
mmu-miR-344i	XM_006532962.2	142.00	-26.35	3 19	1203 1222	16
mmu-miR-344i	NM_001160262.1	160.00	-26.76	2 18	414 436	19
mmu-miR-344i	NM_001160262.1	150.00	-17.12	2 11	2709 2728	9
mmu-miR-344i	XM_006520437.2	141.00	-21.96	3 19	681 701	17
mmu-miR-344i	NM_010181.2	156.00	-20.12	2 19	22 40	17
mmu-miR-344i	NM_010181.2	146.00	-19.77	2 19	4766 4785	17
mmu-miR-344i	NM_010181.2	144.00	-18.46	2 19	9459 9481	20
mmu-miR-344i	NM_010181.2	140.00	-23.77	2 18	2028 2051	20
mmu-miR-344i	XM_006527803.1	140.00	-14.53	2 9	1257 1276	7
mmu-miR-344i	NM_001081185.1	162.00	-27.15	2 16	6814 6835	16
mmu-miR-344i	NM_172862.3	155.00	-17.56	2 17	9845 9866	17
mmu-miR-344i	NM_172862.3	152.00	-19.15	2 16	11975 11992	14
mmu-miR-344i	NM_172862.3	140.00	-16.34	2 9	3 22	7
mmu-miR-344i	NM_172862.3	140.00	-21.54	2 18	8445 8465	17
mmu-miR-344i	XM_006533539.3	144.00	-18.70	2 19	3378 3396	17
mmu-miR-344i	XM_006533539.3	142.00	-27.13	2 19	3848 3867	17
mmu-miR-344i	NM_001033221.3	145.00	-15.88	2 17	1323 1340	15
mmu-miR-344i	NM_145741.2	155.00	-27.40	2 18	1799 1817	16
mmu-miR-344i	NM_145741.2	141.00	-21.27	3 18	861 880	15
mmu-miR-344i	XM_006510297.3	140.00	-14.89	2 9	2617 2636	7
mmu-miR-344i	XM_006530353.3	160.00	-23.40	2 19	3441 3459	17
mmu-miR-344i	XM_006530353.3	153.00	-20.33	2 19	15475 15497	20
mmu-miR-344i	XM_006530353.3	147.00	-16.24	2 16	2135 2154	14
mmu-miR-344i	XM_006530353.3	141.00	-22.21	3 19	11761 11781	17
mmu-miR-344i	XM_011248576.2	147.00	-22.75	2 16	5742 5761	14
mmu-miR-344i	XM_011248576.2	144.00	-19.60	2 14	8840 8860	13
mmu-miR-344i	NM_001198955.1	145.00	-25.94	2 19	352 373	19
mmu-miR-344i	NM_001198955.1	143.00	-21.08	2 16	769 788	14
mmu-miR-344i	XM_006532232.3	144.00	-18.43	2 19	1381 1399	17
mmu-miR-344i	XM_006532232.3	144.00	-24.32	2 17	2183 2202	15
mmu-miR-344i	XM_006532232.3	143.00	-17.44	2 16	1476 1495	14
mmu-miR-344i	XM_006532232.3	143.00	-17.46	2 16	1524 1543	14
mmu-miR-344i	XM_006532232.3	143.00	-17.46	2 16	1620 1639	14
mmu-miR-344i	XM_006532232.3	143.00	-17.46	2 16	1716 1735	14
mmu-miR-344i	NM_016697.3	150.00	-19.93	2 11	563 582	9
mmu-miR-344i	XM_006521554.3	169.00	-24.51	2 18	840 859	16
mmu-miR-344i	XM_006521554.3	158.00	-30.87	2 19	813 832	17

mmu-miR-344i	XM_006515096.2	140.00	-22.24	2 18	1305 1326	18
mmu-miR-344i	XM_006526069.2	142.00	-23.31	3 18	1313 1332	16
mmu-miR-344i	XM_006499444.3	141.00	-23.19	3 19	51 71	17
mmu-miR-344i	XM_011246309.2	145.00	-25.34	2 19	2011 2032	19
mmu-miR-344i	NM_175520.4	146.00	-23.50	3 19	170 189	16
mmu-miR-344i	NM_175520.4	144.00	-22.77	2 17	1741 1760	15
mmu-miR-344i	XM_006529759.1	160.00	-25.10	2 19	1445 1467	20
mmu-miR-344i	XM_006529759.1	141.00	-20.12	2 18	179 198	16
mmu-miR-344i	XM_006529759.1	140.00	-23.23	3 18	515 535	16
mmu-miR-344i	NM_001162950.1	149.00	-24.88	3 19	1468 1488	17
mmu-miR-344i	NM_001162950.1	146.00	-22.80	2 19	3267 3286	17
mmu-miR-344i	NM_001162950.1	140.00	-20.77	2 9	2666 2685	7
mmu-miR-344i	XM_006505602.3	157.00	-22.50	2 19	8121 8141	18
mmu-miR-344i	XM_006505602.3	150.00	-23.32	2 16	9436 9456	15
mmu-miR-344i	XM_006505602.3	145.00	-24.32	2 19	6486 6506	18
mmu-miR-344i	XM_006505602.3	143.00	-18.11	2 18	13549 13567	16
mmu-miR-344i	XM_006505602.3	141.00	-18.33	2 19	2588 2608	18
mmu-miR-344i	XM_006505602.3	141.00	-24.81	2 14	9849 9868	12
mmu-miR-344i	NM_001164086.1	148.00	-15.69	2 14	5827 5847	13
mmu-miR-344i	NM_001164086.1	147.00	-24.15	2 18	7256 7274	16
mmu-miR-344i	NM_001164086.1	145.00	-22.23	2 19	1359 1380	19
mmu-miR-344i	NM_001327998.1	150.00	-20.57	2 11	2344 2363	9
mmu-miR-344i	NM_001327998.1	142.00	-15.53	2 11	5229 5248	9
mmu-miR-344i	NM_001327998.1	141.00	-14.44	2 18	4028 4047	16
mmu-miR-344i	XM_006539957.3	155.00	-18.20	2 18	16 34	16
mmu-miR-344i	NM_133871.2	142.00	-16.94	2 19	2490 2509	17
mmu-miR-344i	XM_006511311.2	158.00	-22.10	2 18	5154 5171	16
mmu-miR-344i	XM_006511311.2	145.00	-22.32	2 18	5015 5034	16
mmu-miR-344i	NM_023670.3	142.00	-15.46	2 11	1647 1666	9
mmu-miR-344i	XM_017316439.1	146.00	-22.03	2 16	1325 1345	15
mmu-miR-344i	XM_006496593.3	147.00	-19.38	2 14	7519 7540	14
mmu-miR-344i	XM_006496593.3	142.00	-15.58	2 11	1403 1422	9
mmu-miR-344i	XM_006528842.1	142.00	-20.37	2 19	1065 1084	17
mmu-miR-344i	NM_001302471.1	165.00	-27.31	2 18	3665 3684	16
mmu-miR-344i	NM_001302471.1	151.00	-19.43	2 19	3090 3109	18
mmu-miR-344i	NM_001302471.1	149.00	-30.16	2 19	2300 2320	18
mmu-miR-344i	NM_146125.2	141.00	-19.41	2 19	1393 1414	19
mmu-miR-344i	XM_011248919.2	155.00	-20.86	2 18	5052 5073	18
mmu-miR-344i	XM_011248919.2	152.00	-29.28	2 19	4118 4136	17
mmu-miR-344i	NM_031180.2	146.00	-23.95	2 19	2566 2585	17
mmu-miR-344i	XM_017320093.1	150.00	-20.29	2 11	358 377	9
mmu-miR-344i	NM_001081667.2	142.00	-22.13	2 19	593 612	17
mmu-miR-344i	XM_006512296.3	142.00	-20.77	2 19	1030 1049	17
mmu-miR-344i	NM_029274.2	144.00	-22.95	2 17	34 53	15
mmu-miR-344i	NM_029274.2	140.00	-18.19	2 9	1446 1465	7
mmu-miR-344i	XM_017316676.1	157.00	-22.86	2 18	11389 11408	16
mmu-miR-344i	XM_017316676.1	149.00	-19.42	2 19	6591 6611	18
mmu-miR-344i	XM_017316676.1	144.00	-18.26	2 19	14225 14246	19
mmu-miR-344i	XM_017316676.1	143.00	-16.13	2 16	17801 17820	14
mmu-miR-344i	XM_017316676.1	142.00	-26.66	2 19	14976 14996	19
mmu-miR-344i	XM_017316676.1	141.00	-15.48	2 19	17479 17499	18
mmu-miR-344i	XM_017316676.1	140.00	-23.52	2 13	9012 9031	11
mmu-miR-344i	XM_017315313.1	140.00	-15.44	2 9	407 426	7
mmu-miR-344i	XM_017313062.1	147.00	-15.71	2 19	1488 1505	17
mmu-miR-344i	XM_017313062.1	143.00	-16.39	2 13	1670 1690	12
mmu-miR-344i	XM_006523061.3	154.00	-23.69	2 15	483 502	13
mmu-miR-344i	XM_006511102.2	145.00	-16.64	2 19	38 58	18
mmu-miR-344i	NM_001302765.1	142.00	-16.81	2 19	487 506	17
mmu-miR-344i	NM_144945.3	149.00	-20.78	2 16	583 601	14
mmu-miR-344i	NM_144945.3	146.00	-17.16	2 19	6021 6040	17
mmu-miR-344i	NM_144945.3	140.00	-13.66	2 9	3478 3497	7
mmu-miR-344i	XM_006519969.3	142.00	-12.94	2 11	1356 1375	9
mmu-miR-344i	NM_053098.2	141.00	-26.44	3 19	10 31	18
mmu-miR-344i	XM_006506340.3	155.00	-18.31	2 16	488 507	14
mmu-miR-344i	NM_028973.2	142.00	-17.75	2 17	2299 2317	15

mmu-miR-344i	XM_006527321.2	141.00	-22.43	2 18	1040 1059	16
mmu-miR-344i	XM_006506086.2	146.00	-21.15	2 19	943 962	17
mmu-miR-344i	NM_001171187.1	157.00	-21.46	2 18	1775 1794	16
mmu-miR-344i	NM_001171187.1	140.00	-19.15	2 9	617 636	7
mmu-miR-344i	XM_006541232.3	158.00	-25.83	2 16	2459 2479	15
mmu-miR-344i	XM_006541232.3	157.00	-22.93	2 18	342 361	16
mmu-miR-344i	XM_006541232.3	141.00	-22.51	3 19	5637 5657	17
mmu-miR-344i	XM_006541232.3	140.00	-14.92	2 9	3056 3075	7
mmu-miR-344i	XM_006532410.3	148.00	-22.63	2 16	4146 4163	14
mmu-miR-344i	NM_001252563.1	142.00	-23.11	3 19	1896 1915	16
mmu-miR-344i	XM_011243266.1	147.00	-14.60	2 12	3477 3496	10
mmu-miR-344i	XM_011243266.1	141.00	-22.36	2 18	2017 2034	16
mmu-miR-344i	NM_001290512.1	146.00	-15.99	2 16	1062 1083	16
mmu-miR-344i	NM_029844.3	140.00	-25.50	2 19	594 616	20
mmu-miR-344i	NM_205810.4	141.00	-13.24	2 11	3079 3099	10
mmu-miR-344i	XM_006537659.2	140.00	-22.61	2 18	2214 2234	17
mmu-miR-344i	NM_175260.2	152.00	-23.38	2 19	7610 7628	17
mmu-miR-344i	XM_017315841.1	143.00	-14.82	2 18	4685 4703	16
mmu-miR-344i	XM_017315841.1	140.00	-13.64	2 17	4507 4526	15
mmu-miR-344i	XM_017320223.1	146.00	-18.55	2 16	3794 3815	16
mmu-miR-344i	XM_017320223.1	144.00	-21.72	3 18	534 554	16
mmu-miR-344i	XM_017320223.1	144.00	-26.19	2 19	3611 3632	19
mmu-miR-344i	XM_017317978.1	160.00	-22.56	2 18	1236 1257	18
mmu-miR-344i	NM_173437.2	153.00	-23.48	2 19	6930 6950	18
mmu-miR-344i	NM_173437.2	144.00	-16.11	2 17	3755 3772	15
mmu-miR-344i	NM_173437.2	142.00	-14.91	2 15	7653 7672	13
mmu-miR-344i	NM_001242558.1	149.00	-25.10	2 19	458 478	18
mmu-miR-344i	NM_011424.3	158.00	-25.38	2 19	4386 4405	17
mmu-miR-344i	XM_006529456.3	145.00	-21.90	2 19	1234 1254	18
mmu-miR-344i	XM_006510077.3	144.00	-18.22	2 18	92 112	17
mmu-miR-344i	NM_001134300.2	140.00	-17.15	2 9	4698 4717	7
mmu-miR-344i	NM_001109985.1	145.00	-25.30	2 18	1890 1909	16
mmu-miR-344i	XM_006526747.3	150.00	-15.43	2 19	5053 5072	17
mmu-miR-344i	XM_006526747.3	149.00	-20.90	2 18	1384 1403	16
mmu-miR-344i	XM_006526747.3	147.00	-21.62	2 17	2020 2040	16
mmu-miR-344i	XM_006526747.3	144.00	-23.31	2 18	2515 2537	19
mmu-miR-344i	NM_001077403.1	149.00	-24.15	2 18	353 372	16
mmu-miR-344i	NM_198190.1	143.00	-21.40	2 19	1163 1180	17
mmu-miR-344i	NM_001282961.1	141.00	-29.40	2 19	562 583	19
mmu-miR-344i	XM_006530325.3	143.00	-19.46	2 18	1157 1175	16
mmu-miR-344i	NM_145226.2	153.00	-20.03	2 19	4142 4163	19
mmu-miR-344i	NM_145226.2	150.00	-26.51	2 16	362 384	17
mmu-miR-344i	NM_145226.2	144.00	-21.11	2 19	2484 2502	17
mmu-miR-344i	NM_011010.2	153.00	-28.76	3 19	1505 1525	17
mmu-miR-344i	NM_011010.2	147.00	-15.19	2 12	1269 1288	10
mmu-miR-344i	NM_011010.2	145.00	-19.57	2 19	1683 1703	18
mmu-miR-344i	NM_011010.2	141.00	-25.02	3 16	78 96	13
mmu-miR-344i	XM_011239969.2	142.00	-21.60	2 15	506 525	13
mmu-miR-344i	NM_001286743.1	140.00	-27.14	2 19	2018 2040	20
mmu-miR-6238	XM_006509531.3	144.00	-18.82	2 20	984 1003	18
mmu-miR-6238	NM_177743.5	147.00	-16.87	3 20	2486 2507	17
mmu-miR-6238	NM_145141.2	145.00	-10.14	2 10	1623 1644	8
mmu-miR-6238	NM_172862.3	147.00	-20.58	2 20	12202 12223	18
mmu-miR-6238	XM_006533539.3	141.00	-9.68	2 18	8585 8606	16
mmu-miR-6238	NM_008067.4	147.00	-19.04	2 20	319 340	18
mmu-miR-6238	NM_010279.3	154.00	-22.95	3 20	3819 3841	18
mmu-miR-6238	XM_011250978.2	159.00	-20.93	2 19	4319 4338	17
mmu-miR-6238	XM_006505602.3	157.00	-13.33	2 21	12489 12508	19
mmu-miR-6238	XM_006505602.3	141.00	-16.86	2 19	9036 9058	18
mmu-miR-6238	XM_006505602.3	140.00	-11.16	2 9	14211 14232	7
mmu-miR-6238	NM_001164086.1	153.00	-13.59	2 18	6977 6998	16
mmu-miR-6238	NM_001164086.1	142.00	-10.53	2 21	9774 9794	19
mmu-miR-6238	NM_001327998.1	152.00	-20.68	2 21	1486 1507	19
mmu-miR-6238	NM_001327998.1	142.00	-11.36	2 19	2278 2299	17
mmu-miR-6238	NM_001327998.1	140.00	-13.42	2 21	3162 3183	19

mmu-miR-6238	XM_017316439.1	140.00	-15.54	2 21	1936 1957	19
mmu-miR-6238	XM_006496593.3	143.00	-13.82	2 21	5850 5872	20
mmu-miR-6238	NM_001302471.1	144.00	-6.39	2 13	1538 1559	11
mmu-miR-6238	XM_017315734.1	155.00	-12.05	2 20	2873 2894	18
mmu-miR-6238	XM_017315734.1	142.00	-11.63	2 19	3503 3524	17
mmu-miR-6238	NM_016659.3	144.00	-18.66	2 21	1414 1435	19
mmu-miR-6238	NM_029274.2	141.00	-24.75	2 18	6410 6431	16
mmu-miR-6238	XM_017313062.1	158.00	-14.26	2 19	3505 3526	17
mmu-miR-6238	XM_017313062.1	141.00	-15.52	2 15	4070 4092	14
mmu-miR-6238	NM_144945.3	144.00	-6.89	2 17	826 847	15
mmu-miR-6238	NM_175271.4	155.00	-14.81	2 21	2068 2090	20
mmu-miR-6238	NM_175271.4	150.00	-18.78	2 21	1071 1091	19
mmu-miR-6238	NM_028973.2	156.00	-15.15	2 20	3432 3454	20
mmu-miR-6238	NM_008524.2	140.00	-11.64	2 21	2024 2045	19
mmu-miR-6238	XM_006532410.3	143.00	-14.33	2 17	5905 5930	19
mmu-miR-6238	NM_001270475.1	140.00	-14.26	2 17	1487 1508	15
mmu-miR-6238	NM_001005423.2	151.00	-14.00	2 14	1182 1202	12
mmu-miR-6238	NM_001005423.2	148.00	-12.53	2 21	2063 2084	19
mmu-miR-6238	XM_006537659.2	143.00	-18.02	2 21	4468 4490	20
mmu-miR-6238	XM_006537659.2	141.00	-6.48	2 20	6878 6898	18
mmu-miR-6238	XM_006498901.3	147.00	-16.65	2 16	1311 1332	14
mmu-miR-6238	NM_008760.4	144.00	-19.14	2 17	1299 1320	15
mmu-miR-6238	XM_011239969.2	167.00	-16.55	2 20	6455 6476	18
mmu-miR-6238	XM_011239969.2	146.00	-16.06	2 16	3567 3589	15
mmu-miR-6351	XM_006520437.2	147.00	-29.63	2 21	10 34	21
mmu-miR-6351	NM_001081185.1	143.00	-25.90	2 22	7509 7534	23
mmu-miR-6351	NM_001081185.1	141.00	-22.62	2 18	101 123	16
mmu-miR-6351	NM_172862.3	159.00	-31.12	3 22	8015 8036	19
mmu-miR-6351	NM_172862.3	142.00	-17.21	2 19	7534 7556	17
mmu-miR-6351	NM_172862.3	141.00	-17.68	2 18	3865 3884	16
mmu-miR-6351	XM_006533539.3	146.00	-19.97	2 20	7214 7240	22
mmu-miR-6351	NM_001033221.3	147.00	-19.85	2 20	1632 1654	18
mmu-miR-6351	NM_008067.4	145.00	-22.85	2 18	3403 3425	16
mmu-miR-6351	XM_006530353.3	151.00	-18.74	2 21	15134 15159	22
mmu-miR-6351	XM_006530353.3	140.00	-27.58	2 22	11746 11771	23
mmu-miR-6351	XM_011248576.2	151.00	-32.93	3 20	2978 3000	17
mmu-miR-6351	NM_016697.3	148.00	-26.08	3 22	1210 1234	21
mmu-miR-6351	XM_011250978.2	145.00	-20.41	3 18	1287 1309	15
mmu-miR-6351	NM_175520.4	149.00	-19.12	2 22	994 1016	20
mmu-miR-6351	XM_006505602.3	140.00	-22.88	2 17	4574 4596	15
mmu-miR-6351	NM_010450.3	142.00	-22.67	3 21	789 813	20
mmu-miR-6351	NM_013558.2	144.00	-15.55	2 13	584 606	11
mmu-miR-6351	NM_013868.4	145.00	-22.95	2 22	2391 2414	22
mmu-miR-6351	XM_006516224.3	142.00	-18.33	3 20	746 770	19
mmu-miR-6351	NM_001302471.1	146.00	-18.89	2 22	513 536	22
mmu-miR-6351	NM_001302471.1	142.00	-23.90	2 18	898 918	16
mmu-miR-6351	NM_010608.2	148.00	-22.04	2 22	1275 1298	21
mmu-miR-6351	XM_006497892.3	144.00	-22.91	2 19	3424 3449	20
mmu-miR-6351	NM_029274.2	153.00	-32.76	2 19	7432 7457	20
mmu-miR-6351	NM_029274.2	143.00	-26.25	2 21	4600 4618	19
mmu-miR-6351	XM_017313062.1	147.00	-21.26	2 20	1747 1769	18
mmu-miR-6351	XM_006523061.3	140.00	-12.99	2 9	594 616	7
mmu-miR-6351	NM_001302765.1	140.00	-18.61	2 21	863 882	19
mmu-miR-6351	NM_144945.3	146.00	-24.13	2 21	66 87	19
mmu-miR-6351	XM_006519969.3	151.00	-20.11	2 20	2844 2866	18
mmu-miR-6351	NM_144799.2	143.00	-20.62	2 20	87 109	18
mmu-miR-6351	XM_006506340.3	159.00	-23.33	2 22	557 578	20
mmu-miR-6351	NM_028973.2	145.00	-15.90	2 22	1125 1147	20
mmu-miR-6351	NM_028838.2	156.00	-15.51	2 22	1855 1879	22
mmu-miR-6351	XM_006527321.2	140.00	-24.69	2 21	1223 1243	19
mmu-miR-6351	XM_006498872.3	141.00	-13.25	2 22	4121 4143	20
mmu-miR-6351	XM_011243266.1	147.00	-21.34	2 22	1056 1080	22
mmu-miR-6351	XM_011243266.1	146.00	-20.67	2 11	510 532	9
mmu-miR-6351	NM_177595.4	160.00	-24.40	2 21	678 700	19
mmu-miR-6351	NM_177595.4	144.00	-25.92	2 20	1276 1298	19

mmu-miR-6351	XM_006504613.3	140.00	-20.34	3 18	595 618	16
mmu-miR-6351	NM_001039545.2	147.00	-19.61	2 12	4436 4458	10
mmu-miR-6351	XM_017315841.1	142.00	-19.95	3 17	626 647	14
mmu-miR-6351	NM_173437.2	156.00	-24.09	2 19	3333 3354	17
mmu-miR-6351	NM_173437.2	146.00	-18.95	2 16	1749 1774	17
mmu-miR-6351	XM_006541297.3	147.00	-20.33	2 12	7028 7050	10
mmu-miR-6351	XM_006541297.3	140.00	-18.76	3 22	2989 3012	20
mmu-miR-6351	NM_011424.3	141.00	-17.63	2 20	5314 5332	18
mmu-miR-6351	XM_006498901.3	153.00	-15.06	2 19	883 906	18
mmu-miR-6351	NM_133500.2	146.00	-17.00	2 20	1463 1486	19
mmu-miR-6351	NM_133500.2	146.00	-24.93	2 22	1531 1554	22
mmu-miR-6351	NM_145226.2	153.00	-24.44	2 19	3320 3345	20
mmu-miR-6351	NM_145226.2	140.00	-11.64	2 9	1748 1770	7
mmu-miR-6351	NM_011854.2	143.00	-16.74	2 12	937 959	10
mmu-miR-6351	NM_001286743.1	141.00	-27.43	3 18	1288 1310	15
mmu-miR-6378	XM_006532962.2	144.00	-21.53	3 21	272 293	18
mmu-miR-6378	XM_006520437.2	151.00	-20.00	2 12	555 576	10
mmu-miR-6378	NM_001081185.1	144.00	-17.41	2 17	7562 7583	15
mmu-miR-6378	NM_172862.3	151.00	-28.30	2 16	2310 2331	14
mmu-miR-6378	NM_172862.3	148.00	-23.31	2 18	9176 9198	17
mmu-miR-6378	NM_172862.3	140.00	-17.10	2 9	8578 8599	7
mmu-miR-6378	NM_008067.4	146.00	-17.09	2 20	1695 1717	19
mmu-miR-6378	NM_008067.4	142.00	-23.63	3 20	2993 3016	19
mmu-miR-6378	XM_006514444.1	149.00	-15.46	2 20	1308 1328	18
mmu-miR-6378	XM_006530353.3	153.00	-26.01	2 19	11217 11239	18
mmu-miR-6378	XM_006530353.3	148.00	-21.83	2 19	11609 11629	17
mmu-miR-6378	XM_011248576.2	148.00	-23.07	2 21	835 856	19
mmu-miR-6378	XM_011250978.2	156.00	-22.91	2 19	887 907	17
mmu-miR-6378	XM_011250978.2	143.00	-17.62	2 20	700 721	18
mmu-miR-6378	XM_011250978.2	141.00	-18.80	2 10	637 658	8
mmu-miR-6378	XM_006515096.2	171.00	-32.07	2 21	1696 1718	20
mmu-miR-6378	XM_006515096.2	158.00	-22.77	2 21	7715 7735	19
mmu-miR-6378	XM_006515096.2	146.00	-20.92	2 20	914 938	21
mmu-miR-6378	NM_008185.3	158.00	-21.74	2 20	214 237	20
mmu-miR-6378	XM_006529759.1	150.00	-19.01	2 15	246 267	13
mmu-miR-6378	XM_006529759.1	145.00	-19.31	2 18	1675 1696	16
mmu-miR-6378	XM_006505602.3	162.00	-22.29	2 20	11575 11597	19
mmu-miR-6378	XM_006505602.3	157.00	-19.75	2 20	11824 11844	18
mmu-miR-6378	XM_006505602.3	147.00	-16.09	2 20	1961 1982	18
mmu-miR-6378	XM_006505602.3	146.00	-26.63	2 21	7106 7131	23
mmu-miR-6378	XM_006505602.3	142.00	-24.07	2 17	607 627	15
mmu-miR-6378	NM_001164086.1	150.00	-21.18	2 19	9829 9850	17
mmu-miR-6378	NM_001327998.1	143.00	-17.05	2 12	4231 4252	10
mmu-miR-6378	XM_006539957.3	143.00	-21.41	2 20	409 430	18
mmu-miR-6378	XM_006511311.2	142.00	-15.53	2 18	4349 4368	16
mmu-miR-6378	XM_006516224.3	143.00	-17.87	2 12	1101 1122	10
mmu-miR-6378	NM_001302471.1	146.00	-25.55	2 19	2868 2889	17
mmu-miR-6378	NM_001289875.1	140.00	-20.22	2 13	2322 2343	11
mmu-miR-6378	NM_010608.2	144.00	-21.54	2 14	1359 1381	13
mmu-miR-6378	NM_173427.2	147.00	-18.54	2 16	866 887	14
mmu-miR-6378	XM_017320093.1	140.00	-21.82	2 13	1184 1205	11
mmu-miR-6378	XM_006512296.3	145.00	-20.96	2 18	2619 2640	16
mmu-miR-6378	NM_029274.2	147.00	-17.64	2 12	630 651	10
mmu-miR-6378	XM_017316676.1	141.00	-22.58	3 19	4697 4719	17
mmu-miR-6378	XM_017316676.1	141.00	-13.61	2 10	19642 19663	8
mmu-miR-6378	XM_017313062.1	144.00	-15.89	2 13	4084 4105	11
mmu-miR-6378	NM_175271.4	140.00	-13.06	2 9	493 514	7
mmu-miR-6378	XM_006506340.3	140.00	-19.15	2 19	1863 1883	17
mmu-miR-6378	XM_006498872.3	140.00	-11.86	2 9	2665 2686	7
mmu-miR-6378	XM_006532410.3	140.00	-12.89	2 9	1869 1890	7
mmu-miR-6378	XM_006520023.2	141.00	-24.55	2 19	858 880	18
mmu-miR-6378	NM_001252563.1	152.00	-22.89	2 21	1322 1343	19
mmu-miR-6378	XM_006504940.3	141.00	-20.13	3 20	515 535	17
mmu-miR-6378	XM_006504940.3	140.00	-10.96	2 9	833 854	7
mmu-miR-6378	NM_001270475.1	151.00	-28.46	2 16	4335 4356	14

mmu-miR-6378	NM_001290512.1	148.00	-19.08	2 21	2773 2794	19
mmu-miR-6378	NM_001290512.1	144.00	-21.19	2 21	2961 2982	19
mmu-miR-6378	NM_177595.4	140.00	-24.66	3 19	42 62	16
mmu-miR-6378	NM_007641.5	151.00	-19.79	2 20	587 608	18
mmu-miR-6378	NM_007641.5	144.00	-18.74	2 18	168 190	17
mmu-miR-6378	NM_175260.2	142.00	-14.88	2 11	6686 6707	9
mmu-miR-6378	XM_017317978.1	146.00	-16.93	2 21	778 798	19
mmu-miR-6378	NM_173437.2	140.00	-16.72	2 17	10901 10922	15
mmu-miR-6378	XM_006541297.3	142.00	-23.05	2 20	6956 6979	20
mmu-miR-6378	XM_006541297.3	140.00	-13.30	2 9	4375 4396	7
mmu-miR-6378	NM_011424.3	143.00	-24.25	2 20	3791 3812	18
mmu-miR-6378	XM_006529456.3	149.00	-19.42	2 14	2776 2797	12
mmu-miR-6378	NM_001134300.2	141.00	-16.36	2 10	5345 5366	8
mmu-miR-6378	NM_001109985.1	143.00	-19.26	2 18	1626 1646	16
mmu-miR-6378	NM_001029877.3	140.00	-22.67	2 13	2432 2453	11
mmu-miR-6378	NM_001029877.3	140.00	-19.50	2 21	3109 3130	19
mmu-miR-6378	XM_006526747.3	140.00	-13.56	2 9	1974 1995	7
mmu-miR-6378	XM_006526747.3	140.00	-10.23	2 9	5208 5229	7
mmu-miR-6378	NM_001077403.1	140.00	-20.05	2 21	1865 1886	19
mmu-miR-6378	NM_198190.1	143.00	-14.43	2 20	1689 1710	18
mmu-miR-6378	NM_001282961.1	152.00	-17.93	2 13	1449 1470	11
mmu-miR-6378	NM_001282961.1	147.00	-28.04	2 18	418 443	20
mmu-miR-6378	NM_145210.2	147.00	-26.85	3 20	1101 1122	17
mmu-miR-6378	XM_006530294.3	150.00	-18.69	2 20	1315 1338	20
mmu-miR-6378	XM_011239969.2	140.00	-14.05	2 9	1784 1805	7
mmu-miR-3473e	XM_006509531.3	166.00	-26.88	2 20	865 887	20
mmu-miR-3473e	XM_006509531.3	152.00	-24.71	2 18	2655 2676	17
mmu-miR-3473e	XM_006509531.3	150.00	-20.86	2 20	1241 1263	20
mmu-miR-3473e	XM_006509531.3	148.00	-23.41	2 19	2272 2295	20
mmu-miR-3473e	XM_006509531.3	142.00	-26.54	2 19	3107 3127	17
mmu-miR-3473e	XM_006524407.3	149.00	-25.85	2 19	448 469	18
mmu-miR-3473e	XM_006524407.3	142.00	-23.49	2 20	354 375	19
mmu-miR-3473e	XM_006524407.3	141.00	-22.29	2 18	1312 1330	16
mmu-miR-3473e	NM_177743.5	163.00	-31.44	3 20	2689 2709	17
mmu-miR-3473e	NM_177743.5	155.00	-32.86	2 20	2480 2500	18
mmu-miR-3473e	XM_017319626.1	149.00	-23.40	2 19	2383 2404	18
mmu-miR-3473e	XM_017319626.1	146.00	-26.59	2 17	3653 3675	17
mmu-miR-3473e	XM_017319626.1	143.00	-25.22	2 16	4086 4106	14
mmu-miR-3473e	XM_006532962.2	145.00	-30.12	2 18	1091 1111	16
mmu-miR-3473e	NM_001160262.1	155.00	-29.56	2 16	3003 3023	14
mmu-miR-3473e	NM_001160262.1	150.00	-20.55	2 20	2700 2723	21
mmu-miR-3473e	NM_010181.2	161.00	-30.12	2 18	134 154	16
mmu-miR-3473e	NM_010181.2	152.00	-23.77	2 20	872 890	18
mmu-miR-3473e	NM_010181.2	149.00	-27.35	3 20	8156 8175	17
mmu-miR-3473e	NM_010181.2	143.00	-23.01	2 16	8521 8541	14
mmu-miR-3473e	XM_006527803.1	140.00	-20.86	2 20	218 236	18
mmu-miR-3473e	NM_001081185.1	151.00	-21.98	2 18	3743 3762	16
mmu-miR-3473e	NM_001081185.1	142.00	-19.64	2 17	2270 2289	15
mmu-miR-3473e	XM_006496949.3	164.00	-29.65	2 18	625 646	17
mmu-miR-3473e	XM_006496949.3	152.00	-21.35	2 14	1288 1309	13
mmu-miR-3473e	XM_006496949.3	145.00	-28.38	2 19	1684 1705	18
mmu-miR-3473e	NM_172862.3	153.00	-19.04	2 16	300 319	14
mmu-miR-3473e	NM_172862.3	151.00	-27.93	2 18	94 119	21
mmu-miR-3473e	NM_172862.3	149.00	-25.49	3 19	3581 3602	17
mmu-miR-3473e	NM_172862.3	145.00	-20.14	2 16	1326 1345	14
mmu-miR-3473e	XM_006533539.3	152.00	-23.12	2 17	4892 4912	15
mmu-miR-3473e	NM_001033221.3	145.00	-26.47	2 20	1216 1239	21
mmu-miR-3473e	NM_001033445.2	152.00	-26.99	2 18	3835 3858	19
mmu-miR-3473e	NM_001033445.2	150.00	-20.92	2 20	4530 4551	19
mmu-miR-3473e	NM_145741.2	147.00	-19.67	2 12	210 230	10
mmu-miR-3473e	NM_145741.2	144.00	-22.34	2 20	1093 1111	18
mmu-miR-3473e	NM_010279.3	147.00	-19.40	2 17	1718 1739	16
mmu-miR-3473e	NM_010279.3	144.00	-19.50	2 17	1043 1063	15
mmu-miR-3473e	NM_010279.3	140.00	-20.82	2 9	4401 4421	7
mmu-miR-3473e	XM_006510297.3	146.00	-17.39	2 19	559 579	17

mmu-miR-3473e	XM_006510297.3	142.00	-27.29	2 20	1363 1384	19
mmu-miR-3473e	XM_006530353.3	150.00	-24.83	2 20	6597 6614	18
mmu-miR-3473e	XM_006530353.3	143.00	-27.08	2 20	9898 9918	18
mmu-miR-3473e	XM_006530353.3	142.00	-27.90	2 16	9457 9478	15
mmu-miR-3473e	XM_006530353.3	142.00	-18.85	2 11	11285 11305	9
mmu-miR-3473e	XM_006530353.3	140.00	-20.21	2 13	2846 2866	11
mmu-miR-3473e	XM_006530353.3	140.00	-18.70	2 9	4638 4658	7
mmu-miR-3473e	NM_001024731.2	156.00	-26.29	2 17	2411 2431	15
mmu-miR-3473e	XM_011248576.2	158.00	-23.95	2 20	6520 6542	20
mmu-miR-3473e	XM_011248576.2	154.00	-27.40	2 19	4441 4461	17
mmu-miR-3473e	XM_011248576.2	147.00	-18.04	2 20	5467 5487	18
mmu-miR-3473e	XM_011248576.2	146.00	-23.95	2 16	13057 13080	17
mmu-miR-3473e	XM_011248576.2	145.00	-19.68	2 10	11580 11600	8
mmu-miR-3473e	XM_011248576.2	144.00	-20.52	2 19	1431 1450	17
mmu-miR-3473e	XM_011248576.2	141.00	-20.79	2 20	12712 12731	18
mmu-miR-3473e	XM_011248576.2	140.00	-16.25	2 9	4688 4708	7
mmu-miR-3473e	NM_001198955.1	143.00	-21.66	2 18	98 117	16
mmu-miR-3473e	NM_001198955.1	142.00	-21.63	2 20	816 837	19
mmu-miR-3473e	NM_001198955.1	141.00	-17.83	2 19	2230 2251	18
mmu-miR-3473e	NM_001198955.1	140.00	-20.23	2 18	2463 2485	18
mmu-miR-3473e	NM_016697.3	151.00	-27.28	2 18	680 699	16
mmu-miR-3473e	NM_016697.3	148.00	-17.59	2 18	1306 1327	17
mmu-miR-3473e	XM_006521554.3	159.00	-38.02	2 20	28 48	18
mmu-miR-3473e	XM_006515096.2	143.00	-22.10	2 20	7387 7404	18
mmu-miR-3473e	XM_006526069.2	153.00	-21.26	2 14	1671 1691	12
mmu-miR-3473e	XM_006526069.2	151.00	-21.55	3 20	2715 2735	17
mmu-miR-3473e	XM_006499444.3	141.00	-18.92	2 19	507 529	19
mmu-miR-3473e	XM_006496277.3	161.00	-26.91	2 18	1037 1057	16
mmu-miR-3473e	XM_006496277.3	142.00	-26.18	3 20	2379 2403	21
mmu-miR-3473e	XM_011246309.2	143.00	-21.41	2 20	497 517	18
mmu-miR-3473e	NM_175520.4	144.00	-23.92	2 20	947 967	19
mmu-miR-3473e	NM_001162950.1	159.00	-27.03	2 20	1976 1996	18
mmu-miR-3473e	NM_001162950.1	147.00	-22.27	2 19	5985 6006	19
mmu-miR-3473e	NM_001162950.1	140.00	-21.69	2 20	4224 4247	21
mmu-miR-3473e	XM_006505602.3	159.00	-26.68	2 20	5954 5972	18
mmu-miR-3473e	XM_006505602.3	156.00	-23.30	2 13	5764 5784	11
mmu-miR-3473e	XM_006505602.3	153.00	-23.56	2 20	3762 3781	18
mmu-miR-3473e	NM_001164086.1	183.00	-38.52	2 20	7487 7507	18
mmu-miR-3473e	NM_001164086.1	165.00	-27.08	2 20	4781 4800	18
mmu-miR-3473e	NM_001164086.1	152.00	-23.62	2 17	10287 10307	15
mmu-miR-3473e	NM_001164086.1	150.00	-21.82	2 16	9101 9122	15
mmu-miR-3473e	NM_001164086.1	142.00	-18.45	2 11	3823 3843	9
mmu-miR-3473e	NM_010450.3	166.00	-30.94	2 20	160 183	21
mmu-miR-3473e	NM_010450.3	157.00	-31.82	2 20	755 774	18
mmu-miR-3473e	NM_010450.3	147.00	-19.43	2 16	1024 1044	14
mmu-miR-3473e	NM_001327998.1	157.00	-29.01	2 19	3618 3640	19
mmu-miR-3473e	NM_001327998.1	149.00	-20.79	2 14	4476 4496	12
mmu-miR-3473e	NM_001327998.1	144.00	-22.96	2 20	94 114	19
mmu-miR-3473e	NM_013560.2	141.00	-19.86	2 20	357 376	18
mmu-miR-3473e	XM_006539957.3	154.00	-25.28	2 20	19 40	19
mmu-miR-3473e	XM_006539957.3	147.00	-26.34	2 16	730 750	14
mmu-miR-3473e	NM_013868.4	167.00	-29.34	2 20	946 966	18
mmu-miR-3473e	NM_013868.4	154.00	-23.88	2 18	337 357	17
mmu-miR-3473e	NM_013868.4	150.00	-24.99	2 20	1095 1112	18
mmu-miR-3473e	NM_013868.4	142.00	-20.95	2 19	2064 2084	17
mmu-miR-3473e	NM_030704.3	146.00	-23.60	2 20	594 611	18
mmu-miR-3473e	NM_030704.3	141.00	-16.93	2 18	822 842	16
mmu-miR-3473e	XM_006511311.2	153.00	-22.09	2 20	1653 1675	20
mmu-miR-3473e	XM_006511311.2	151.00	-20.96	2 16	1703 1723	14
mmu-miR-3473e	XM_006511311.2	147.00	-23.68	2 16	3714 3734	14
mmu-miR-3473e	XM_006511311.2	143.00	-25.20	2 12	721 741	10
mmu-miR-3473e	XM_006511311.2	142.00	-26.49	2 20	861 882	19
mmu-miR-3473e	XM_006511311.2	140.00	-19.78	2 17	878 898	15
mmu-miR-3473e	NM_023670.3	157.00	-20.19	2 14	1796 1816	12
mmu-miR-3473e	XM_006523711.3	149.00	-21.30	2 14	534 554	12

mmu-miR-3473e	XM_017316439.1	141.00	-18.03	3 20	1902 1921	17
mmu-miR-3473e	XM_017316439.1	140.00	-20.57	2 18	1428 1451	19
mmu-miR-3473e	XM_006496593.3	163.00	-31.67	2 18	450 472	18
mmu-miR-3473e	XM_006496593.3	146.00	-19.09	2 20	1258 1280	20
mmu-miR-3473e	XM_006516224.3	155.00	-24.83	2 16	7 27	14
mmu-miR-3473e	XM_006516224.3	149.00	-24.80	3 20	1003 1022	17
mmu-miR-3473e	XM_006516224.3	149.00	-23.56	2 18	3423 3443	16
mmu-miR-3473e	XM_006516224.3	145.00	-27.25	2 16	1430 1449	14
mmu-miR-3473e	XM_006516224.3	145.00	-25.11	3 19	2042 2064	18
mmu-miR-3473e	XM_006516224.3	142.00	-27.28	2 19	2488 2508	17
mmu-miR-3473e	XM_006528842.1	147.00	-28.22	2 19	2340 2358	17
mmu-miR-3473e	NM_001161541.1	156.00	-25.70	2 18	1169 1190	17
mmu-miR-3473e	NM_001302471.1	144.00	-18.35	2 17	3008 3028	15
mmu-miR-3473e	NM_001302471.1	141.00	-21.34	2 18	4649 4669	16
mmu-miR-3473e	NM_001302471.1	140.00	-28.91	2 19	52 71	17
mmu-miR-3473e	NM_001302471.1	140.00	-19.88	2 18	1424 1446	18
mmu-miR-3473e	NM_001289875.1	145.00	-24.35	2 10	872 892	8
mmu-miR-3473e	NM_146125.2	141.00	-25.40	3 20	991 1013	19
mmu-miR-3473e	NM_008423.2	143.00	-22.46	2 16	3134 3154	14
mmu-miR-3473e	NM_008423.2	140.00	-23.18	2 9	548 568	7
mmu-miR-3473e	NM_021342.1	140.00	-20.17	2 9	260 280	7
mmu-miR-3473e	XM_017315734.1	151.00	-19.41	2 14	1948 1967	12
mmu-miR-3473e	NM_010608.2	145.00	-20.99	2 10	2237 2257	8
mmu-miR-3473e	NM_010608.2	143.00	-27.39	2 20	1944 1965	19
mmu-miR-3473e	XM_006497892.3	150.00	-24.10	2 20	4163 4184	19
mmu-miR-3473e	XM_006497892.3	147.00	-20.70	2 17	5259 5280	16
mmu-miR-3473e	XM_006497892.3	146.00	-19.47	2 19	5751 5771	17
mmu-miR-3473e	XM_006497892.3	145.00	-19.06	2 18	336 356	16
mmu-miR-3473e	XM_011248919.2	156.00	-25.14	2 17	2298 2318	15
mmu-miR-3473e	XM_011248919.2	147.00	-26.47	2 20	6075 6095	18
mmu-miR-3473e	XM_011248919.2	147.00	-22.80	2 16	6163 6183	14
mmu-miR-3473e	XM_011248919.2	142.00	-22.83	2 16	2238 2259	15
mmu-miR-3473e	XM_011248919.2	141.00	-18.57	2 16	1089 1108	14
mmu-miR-3473e	NM_173427.2	150.00	-26.93	3 19	1818 1838	16
mmu-miR-3473e	NM_001081667.2	149.00	-22.47	2 18	776 794	16
mmu-miR-3473e	XM_006512296.3	151.00	-25.87	3 17	1173 1194	15
mmu-miR-3473e	NM_008940.3	142.00	-15.26	2 16	498 519	15
mmu-miR-3473e	NM_008940.3	141.00	-23.13	3 16	356 375	13
mmu-miR-3473e	NM_029274.2	144.00	-23.11	2 16	1372 1390	14
mmu-miR-3473e	NM_029274.2	141.00	-19.43	2 14	3905 3925	12
mmu-miR-3473e	XM_017316676.1	169.00	-35.73	2 20	18079 18103	22
mmu-miR-3473e	XM_017316676.1	158.00	-22.64	2 16	16955 16976	15
mmu-miR-3473e	XM_017316676.1	154.00	-27.60	3 19	15085 15105	16
mmu-miR-3473e	XM_017316676.1	152.00	-23.02	2 17	2990 3010	15
mmu-miR-3473e	XM_017316676.1	146.00	-21.19	2 20	10245 10266	19
mmu-miR-3473e	XM_017316676.1	144.00	-19.63	2 19	6291 6310	17
mmu-miR-3473e	XM_017315313.1	164.00	-27.58	2 20	342 360	18
mmu-miR-3473e	XM_017315313.1	153.00	-23.30	2 20	47 66	18
mmu-miR-3473e	XM_017313062.1	146.00	-35.69	2 20	1339 1361	20
mmu-miR-3473e	XM_006523061.3	155.00	-28.84	2 17	1618 1639	16
mmu-miR-3473e	NM_001302765.1	142.00	-21.25	2 20	1035 1057	20
mmu-miR-3473e	XM_006504999.1	146.00	-20.81	2 20	2736 2753	18
mmu-miR-3473e	NM_144945.3	150.00	-19.32	2 20	3560 3581	19
mmu-miR-3473e	XM_006506340.3	157.00	-25.56	2 20	1486 1510	22
mmu-miR-3473e	XM_006506340.3	152.00	-25.03	2 17	444 464	15
mmu-miR-3473e	XM_006506340.3	141.00	-18.74	2 10	249 269	8
mmu-miR-3473e	NM_028973.2	158.00	-30.32	2 20	3906 3930	22
mmu-miR-3473e	NM_028973.2	146.00	-25.14	2 18	4806 4824	16
mmu-miR-3473e	NM_028973.2	142.00	-18.02	2 20	493 516	21
mmu-miR-3473e	NM_028973.2	141.00	-21.11	2 20	1439 1462	21
mmu-miR-3473e	NM_028838.2	147.00	-22.72	3 20	820 840	17
mmu-miR-3473e	XM_006527321.2	151.00	-24.44	2 16	1157 1177	14
mmu-miR-3473e	XM_006506086.2	159.00	-23.75	2 20	2251 2269	18
mmu-miR-3473e	XM_006541232.3	163.00	-32.07	2 20	5328 5348	18
mmu-miR-3473e	XM_006541232.3	161.00	-27.86	2 19	758 780	19

mmu-miR-3473e	XM_006541232.3	153.00	-20.22	2 18	1292 1312	16
mmu-miR-3473e	XM_006541232.3	143.00	-18.84	2 20	1721 1741	18
mmu-miR-3473e	XM_006498872.3	164.00	-30.68	2 20	9041 9059	18
mmu-miR-3473e	XM_006498872.3	154.00	-25.64	2 19	5660 5678	17
mmu-miR-3473e	XM_006498872.3	153.00	-26.50	2 18	6222 6242	16
mmu-miR-3473e	XM_006498872.3	148.00	-20.91	2 19	3115 3134	17
mmu-miR-3473e	XM_006498872.3	143.00	-24.15	2 20	7608 7628	18
mmu-miR-3473e	XM_006498872.3	141.00	-24.73	3 20	6038 6062	21
mmu-miR-3473e	XM_006532410.3	159.00	-22.67	2 20	1581 1601	18
mmu-miR-3473e	XM_006532410.3	154.00	-31.61	2 19	1736 1756	17
mmu-miR-3473e	NM_001290273.1	147.00	-22.18	2 20	1259 1279	18
mmu-miR-3473e	NM_001290273.1	141.00	-23.64	2 17	154 172	15
mmu-miR-3473e	NM_001252563.1	159.00	-23.48	2 20	1389 1409	18
mmu-miR-3473e	NM_013593.3	153.00	-24.82	2 16	623 642	14
mmu-miR-3473e	NM_013593.3	151.00	-26.03	2 19	107 131	21
mmu-miR-3473e	NM_013593.3	144.00	-26.57	2 19	407 426	17
mmu-miR-3473e	XM_011243266.1	161.00	-30.03	2 20	1175 1194	18
mmu-miR-3473e	XM_011243266.1	160.00	-28.00	2 17	1072 1092	15
mmu-miR-3473e	XM_011243266.1	159.00	-25.60	2 16	2741 2761	14
mmu-miR-3473e	XM_011243266.1	143.00	-21.74	2 20	1321 1341	18
mmu-miR-3473e	XM_011243266.1	140.00	-18.21	2 9	4150 4170	7
mmu-miR-3473e	XM_006504940.3	141.00	-16.99	2 19	1005 1021	17
mmu-miR-3473e	NM_001270475.1	159.00	-26.53	2 16	3772 3792	14
mmu-miR-3473e	NM_001270475.1	153.00	-23.04	2 16	4679 4698	14
mmu-miR-3473e	NM_001270475.1	152.00	-24.61	2 20	4166 4184	18
mmu-miR-3473e	NM_001270475.1	143.00	-22.64	2 20	1693 1713	18
mmu-miR-3473e	NM_001270475.1	142.00	-23.34	2 15	8573 8593	13
mmu-miR-3473e	NM_001270475.1	141.00	-23.57	3 20	5847 5869	19
mmu-miR-3473e	NM_001270475.1	140.00	-22.79	2 18	799 820	17
mmu-miR-3473e	NM_177595.4	141.00	-22.97	2 14	1719 1739	12
mmu-miR-3473e	NM_001320077.1	179.00	-36.41	2 20	1886 1906	18
mmu-miR-3473e	NM_001320077.1	141.00	-20.59	2 18	2082 2100	16
mmu-miR-3473e	NM_001320077.1	140.00	-17.30	2 9	831 851	7
mmu-miR-3473e	NM_010809.2	140.00	-27.35	2 18	1076 1097	17
mmu-miR-3473e	XM_006504613.3	141.00	-22.65	2 20	727 746	18
mmu-miR-3473e	NM_008623.5	161.00	-28.13	2 18	228 248	16
mmu-miR-3473e	NM_008623.5	157.00	-28.71	2 19	174 195	18
mmu-miR-3473e	NM_029844.3	147.00	-20.68	2 16	66 86	14
mmu-miR-3473e	NM_001005423.2	143.00	-25.58	2 19	930 948	17
mmu-miR-3473e	NM_001099314.1	146.00	-19.98	2 19	18 38	17
mmu-miR-3473e	XM_006537659.2	151.00	-23.20	2 16	115 135	14
mmu-miR-3473e	XM_006537659.2	142.00	-21.07	2 16	1934 1955	15
mmu-miR-3473e	XM_006529753.2	158.00	-23.19	2 19	557 577	17
mmu-miR-3473e	NM_001039545.2	153.00	-28.61	2 20	946 965	18
mmu-miR-3473e	NM_001039545.2	142.00	-27.54	2 19	3607 3624	17
mmu-miR-3473e	XM_006532412.1	148.00	-21.56	2 19	930 949	17
mmu-miR-3473e	XM_006532412.1	141.00	-21.73	3 20	3800 3819	17
mmu-miR-3473e	XM_017315841.1	144.00	-19.63	2 19	975 994	17
mmu-miR-3473e	NM_010858.4	141.00	-21.08	2 18	197 217	16
mmu-miR-3473e	XM_006513859.3	153.00	-32.66	2 18	27 47	16
mmu-miR-3473e	XM_017320223.1	162.00	-24.85	2 17	1614 1633	15
mmu-miR-3473e	NM_173437.2	165.00	-26.78	2 20	2345 2364	18
mmu-miR-3473e	NM_173437.2	163.00	-30.30	2 20	9965 9985	18
mmu-miR-3473e	NM_173437.2	156.00	-22.11	2 18	3571 3592	17
mmu-miR-3473e	NM_173437.2	151.00	-26.50	2 18	774 793	16
mmu-miR-3473e	NM_173437.2	149.00	-27.19	2 14	2209 2229	12
mmu-miR-3473e	NM_173437.2	146.00	-19.12	2 18	862 880	16
mmu-miR-3473e	NM_173437.2	142.00	-26.03	3 17	6805 6824	14
mmu-miR-3473e	NM_173437.2	142.00	-17.09	2 16	11777 11798	15
mmu-miR-3473e	NM_173437.2	141.00	-21.32	3 20	645 664	17
mmu-miR-3473e	NM_173437.2	141.00	-20.16	3 16	2469 2488	13
mmu-miR-3473e	NM_173437.2	141.00	-27.16	2 18	4199 4219	16
mmu-miR-3473e	NM_173437.2	140.00	-32.43	2 19	4682 4705	20
mmu-miR-3473e	XM_006541297.3	170.00	-29.22	2 20	1205 1226	19
mmu-miR-3473e	XM_006541297.3	164.00	-33.09	2 18	7765 7786	17

mmu-miR-3473e	XM_006541297.3	156.00	-22.17	2 20	2408 2428	19
mmu-miR-3473e	XM_006541297.3	146.00	-24.57	2 20	1024 1045	19
mmu-miR-3473e	XM_006541297.3	141.00	-21.77	2 19	2929 2950	18
mmu-miR-3473e	NM_001242558.1	145.00	-18.69	2 10	3092 3112	8
mmu-miR-3473e	NM_011424.3	153.00	-24.98	2 16	488 507	14
mmu-miR-3473e	NM_011424.3	148.00	-21.79	2 17	526 546	15
mmu-miR-3473e	NM_011424.3	145.00	-22.10	2 10	6129 6149	8
mmu-miR-3473e	NM_011424.3	144.00	-30.17	2 19	6507 6530	20
mmu-miR-3473e	NM_011424.3	141.00	-20.47	3 18	4944 4964	15
mmu-miR-3473e	XM_006498901.3	165.00	-26.84	2 19	796 817	18
mmu-miR-3473e	XM_006510077.3	155.00	-28.61	2 20	218 238	18
mmu-miR-3473e	XM_006510077.3	145.00	-24.51	2 20	573 596	21
mmu-miR-3473e	NM_001134300.2	150.00	-21.26	2 16	968 991	17
mmu-miR-3473e	NM_001029877.3	162.00	-30.30	2 20	6395 6416	19
mmu-miR-3473e	NM_001029877.3	147.00	-24.53	2 16	5671 5691	14
mmu-miR-3473e	NM_001029877.3	143.00	-29.34	2 20	1859 1879	18
mmu-miR-3473e	NM_001029877.3	141.00	-19.87	2 20	5726 5745	18
mmu-miR-3473e	NM_023456.3	141.00	-23.20	2 18	281 301	16
mmu-miR-3473e	XM_006526747.3	153.00	-23.27	2 14	585 605	12
mmu-miR-3473e	NM_001077403.1	144.00	-23.01	2 18	5853 5874	17
mmu-miR-3473e	NM_001282961.1	144.00	-22.03	2 19	1115 1134	17
mmu-miR-3473e	NM_145210.2	145.00	-23.88	3 19	1201 1222	17
mmu-miR-3473e	XM_006530325.3	159.00	-24.87	2 20	1272 1292	18
mmu-miR-3473e	NM_145226.2	153.00	-23.06	2 14	2463 2483	12
mmu-miR-3473e	NM_001310636.1	140.00	-18.72	2 19	1863 1882	17
mmu-miR-3473e	NM_008760.4	148.00	-21.41	2 13	1865 1885	11
mmu-miR-3473e	NM_008760.4	140.00	-15.52	2 9	1162 1182	7
mmu-miR-7005-5p	NM_177743.5	141.00	-19.60	2 10	33 54	8
mmu-miR-7005-5p	XM_017319626.1	153.00	-22.10	2 16	2075 2095	14
mmu-miR-7005-5p	XM_006532962.2	166.00	-30.25	2 21	542 565	21
mmu-miR-7005-5p	XM_006532962.2	146.00	-27.00	2 20	2320 2343	20
mmu-miR-7005-5p	XM_006532962.2	140.00	-16.14	2 9	244 265	7
mmu-miR-7005-5p	XM_006532962.2	140.00	-24.17	3 13	1334 1355	10
mmu-miR-7005-5p	NM_001160262.1	169.00	-30.17	2 21	2937 2958	20
mmu-miR-7005-5p	NM_001160262.1	152.00	-27.97	3 21	1838 1859	18
mmu-miR-7005-5p	NM_001160262.1	151.00	-27.42	2 21	2998 3021	21
mmu-miR-7005-5p	NM_001160262.1	146.00	-22.50	3 19	3349 3370	16
mmu-miR-7005-5p	NM_010181.2	148.00	-19.24	2 13	8361 8382	11
mmu-miR-7005-5p	NM_145141.2	155.00	-21.57	2 19	944 963	17
mmu-miR-7005-5p	XM_006527803.1	150.00	-23.12	2 11	143 164	9
mmu-miR-7005-5p	XM_006527803.1	141.00	-18.87	2 10	974 995	8
mmu-miR-7005-5p	NM_001081185.1	152.00	-28.69	2 13	254 275	11
mmu-miR-7005-5p	NM_001081185.1	145.00	-23.65	2 18	3954 3975	16
mmu-miR-7005-5p	NM_001081185.1	141.00	-23.13	3 14	8178 8199	11
mmu-miR-7005-5p	XM_006496949.3	142.00	-19.08	2 11	1036 1057	9
mmu-miR-7005-5p	NM_172862.3	147.00	-20.50	2 18	5883 5903	16
mmu-miR-7005-5p	NM_172862.3	144.00	-30.85	2 21	3883 3904	19
mmu-miR-7005-5p	XM_006533539.3	163.00	-25.46	2 21	4946 4968	20
mmu-miR-7005-5p	XM_006533539.3	156.00	-19.74	2 21	7533 7554	19
mmu-miR-7005-5p	XM_006533539.3	149.00	-22.70	2 19	8280 8302	18
mmu-miR-7005-5p	XM_006533539.3	145.00	-17.45	2 10	5104 5125	8
mmu-miR-7005-5p	NM_001033445.2	153.00	-25.97	2 20	4358 4383	22
mmu-miR-7005-5p	NM_001033445.2	144.00	-22.46	3 18	4494 4516	16
mmu-miR-7005-5p	NM_001033445.2	143.00	-23.81	3 20	1603 1624	17
mmu-miR-7005-5p	NM_001033445.2	142.00	-18.47	2 11	2430 2451	9
mmu-miR-7005-5p	XM_006530353.3	163.00	-31.16	2 17	10584 10606	16
mmu-miR-7005-5p	XM_006530353.3	151.00	-26.63	2 21	15607 15628	21
mmu-miR-7005-5p	XM_006530353.3	150.00	-20.23	2 11	8882 8903	9
mmu-miR-7005-5p	XM_006530353.3	144.00	-20.24	2 13	9895 9916	11
mmu-miR-7005-5p	XM_006530353.3	144.00	-22.43	2 21	15115 15138	22
mmu-miR-7005-5p	XM_006530353.3	142.00	-19.44	2 21	1886 1906	19
mmu-miR-7005-5p	XM_006530353.3	140.00	-22.06	2 9	11081 11102	7
mmu-miR-7005-5p	XM_011248576.2	167.00	-30.44	2 21	2690 2713	21
mmu-miR-7005-5p	XM_011248576.2	166.00	-28.27	2 19	1384 1405	17
mmu-miR-7005-5p	XM_011248576.2	163.00	-25.90	2 16	2749 2770	14

mmu-miR-7005-5p	XM_011248576.2	156.00	-27.03	2 13	10360 10381	11
mmu-miR-7005-5p	XM_011248576.2	151.00	-31.82	2 16	2721 2742	14
mmu-miR-7005-5p	XM_011248576.2	151.00	-21.26	2 21	13044 13067	21
mmu-miR-7005-5p	XM_011248576.2	150.00	-24.66	2 16	7766 7788	15
mmu-miR-7005-5p	XM_011248576.2	149.00	-22.77	2 14	2375 2396	12
mmu-miR-7005-5p	XM_011248576.2	147.00	-24.27	3 21	12863 12885	19
mmu-miR-7005-5p	XM_011248576.2	145.00	-22.88	2 15	2311 2333	14
mmu-miR-7005-5p	XM_011248576.2	144.00	-21.89	2 21	7734 7755	19
mmu-miR-7005-5p	XM_011248576.2	144.00	-28.15	2 17	11224 11245	15
mmu-miR-7005-5p	XM_011248576.2	142.00	-23.01	3 16	3247 3269	14
mmu-miR-7005-5p	XM_011248576.2	141.00	-22.04	2 18	6723 6744	16
mmu-miR-7005-5p	XM_011248576.2	140.00	-27.06	2 14	752 774	13
mmu-miR-7005-5p	XM_011248576.2	140.00	-23.94	2 9	11278 11299	7
mmu-miR-7005-5p	NM_001198955.1	163.00	-27.61	2 21	2897 2919	20
mmu-miR-7005-5p	NM_001198955.1	140.00	-19.97	2 19	2339 2359	17
mmu-miR-7005-5p	XM_006532232.3	152.00	-21.24	2 18	1765 1787	17
mmu-miR-7005-5p	XM_006532232.3	148.00	-23.66	2 18	201 223	17
mmu-miR-7005-5p	XM_006532232.3	144.00	-21.20	3 17	1301 1322	14
mmu-miR-7005-5p	XM_006532232.3	143.00	-22.22	3 20	35 56	17
mmu-miR-7005-5p	XM_006532232.3	143.00	-27.42	2 16	485 506	14
mmu-miR-7005-5p	XM_011250978.2	150.00	-31.61	2 21	1383 1403	19
mmu-miR-7005-5p	XM_011250978.2	148.00	-22.84	2 13	354 375	11
mmu-miR-7005-5p	NM_010340.2	160.00	-35.63	2 17	2173 2194	15
mmu-miR-7005-5p	XM_006521554.3	148.00	-24.52	2 15	1262 1282	13
mmu-miR-7005-5p	XM_006521554.3	147.00	-26.39	2 21	254 276	20
mmu-miR-7005-5p	XM_006515096.2	141.00	-22.20	3 21	1318 1337	18
mmu-miR-7005-5p	XM_006526069.2	152.00	-24.96	2 17	3385 3406	15
mmu-miR-7005-5p	XM_006526069.2	147.00	-20.80	2 13	1642 1665	13
mmu-miR-7005-5p	XM_006526069.2	147.00	-26.01	2 21	2710 2733	21
mmu-miR-7005-5p	XM_006526069.2	144.00	-23.83	2 21	3689 3710	19
mmu-miR-7005-5p	XM_006499444.3	163.00	-31.97	2 17	660 683	17
mmu-miR-7005-5p	XM_006496277.3	151.00	-27.86	2 16	1155 1176	14
mmu-miR-7005-5p	XM_011246309.2	160.00	-24.01	2 17	242 263	15
mmu-miR-7005-5p	NM_175520.4	150.00	-26.09	2 19	2705 2723	17
mmu-miR-7005-5p	NM_175520.4	143.00	-26.29	2 16	2688 2709	14
mmu-miR-7005-5p	XM_006529759.1	149.00	-33.70	2 21	1308 1333	24
mmu-miR-7005-5p	XM_006529759.1	143.00	-22.98	2 21	112 134	20
mmu-miR-7005-5p	NM_001162950.1	158.00	-29.29	2 19	5313 5334	17
mmu-miR-7005-5p	NM_001162950.1	154.00	-30.76	2 21	5616 5636	19
mmu-miR-7005-5p	NM_001162950.1	143.00	-18.85	2 21	3250 3272	20
mmu-miR-7005-5p	NM_001162950.1	143.00	-17.01	2 12	4122 4143	10
mmu-miR-7005-5p	XM_006505602.3	168.00	-29.18	2 21	7326 7347	19
mmu-miR-7005-5p	XM_006505602.3	151.00	-25.25	2 21	13443 13469	24
mmu-miR-7005-5p	XM_006505602.3	148.00	-17.98	2 19	5427 5447	17
mmu-miR-7005-5p	XM_006505602.3	147.00	-29.81	3 21	13704 13726	19
mmu-miR-7005-5p	XM_006505602.3	143.00	-26.69	3 17	12676 12700	17
mmu-miR-7005-5p	NM_001164086.1	150.00	-25.86	2 21	2546 2566	19
mmu-miR-7005-5p	NM_001164086.1	142.00	-21.07	2 19	1268 1289	17
mmu-miR-7005-5p	NM_001164086.1	141.00	-24.97	3 19	3051 3073	17
mmu-miR-7005-5p	NM_010450.3	140.00	-20.77	2 21	160 181	19
mmu-miR-7005-5p	NM_001327998.1	150.00	-21.31	2 21	5281 5301	19
mmu-miR-7005-5p	NM_001327998.1	145.00	-17.07	2 10	2505 2526	8
mmu-miR-7005-5p	NM_001327998.1	142.00	-30.96	3 20	1794 1816	18
mmu-miR-7005-5p	NM_013558.2	153.00	-23.64	2 20	352 372	18
mmu-miR-7005-5p	NM_013560.2	144.00	-20.77	2 17	80 101	15
mmu-miR-7005-5p	NM_013560.2	140.00	-19.29	2 18	100 123	18
mmu-miR-7005-5p	NM_013868.4	151.00	-25.51	2 20	2061 2082	18
mmu-miR-7005-5p	NM_030704.3	171.00	-30.96	2 17	992 1014	16
mmu-miR-7005-5p	NM_030704.3	146.00	-25.09	2 21	870 896	24
mmu-miR-7005-5p	NM_030704.3	142.00	-25.30	3 21	1441 1461	18
mmu-miR-7005-5p	NM_133871.2	141.00	-28.02	2 19	1786 1808	18
mmu-miR-7005-5p	XM_006511311.2	162.00	-31.70	2 21	3145 3168	21
mmu-miR-7005-5p	XM_006511311.2	151.00	-21.91	2 12	3439 3460	10
mmu-miR-7005-5p	XM_006511311.2	144.00	-24.95	2 21	5466 5487	19
mmu-miR-7005-5p	XM_006511311.2	141.00	-17.09	2 10	1625 1646	8

mmu-miR-7005-5p	NM_023670.3	152.00	-23.49	2 16	224 243	14
mmu-miR-7005-5p	NM_001190325.1	157.00	-31.71	2 21	119 138	19
mmu-miR-7005-5p	XM_006496593.3	140.00	-29.16	2 19	2964 2984	17
mmu-miR-7005-5p	XM_006496593.3	140.00	-23.69	2 21	6283 6301	19
mmu-miR-7005-5p	XM_006516224.3	155.00	-31.98	2 21	3835 3853	19
mmu-miR-7005-5p	XM_006516224.3	150.00	-25.87	2 21	1893 1918	23
mmu-miR-7005-5p	XM_006516224.3	144.00	-30.46	3 21	2070 2089	18
mmu-miR-7005-5p	XM_006528842.1	147.00	-23.08	2 17	4977 4999	16
mmu-miR-7005-5p	XM_006528842.1	143.00	-18.12	2 12	5249 5270	10
mmu-miR-7005-5p	NM_001161541.1	158.00	-31.77	2 21	2553 2577	22
mmu-miR-7005-5p	NM_001161541.1	151.00	-24.76	2 16	2848 2869	14
mmu-miR-7005-5p	NM_001161541.1	144.00	-26.28	3 21	2634 2655	18
mmu-miR-7005-5p	NM_001161541.1	143.00	-26.01	3 18	2994 3014	15
mmu-miR-7005-5p	NM_001161541.1	141.00	-19.29	2 10	2861 2882	8
mmu-miR-7005-5p	NM_001302471.1	153.00	-20.99	2 20	1577 1597	18
mmu-miR-7005-5p	NM_001302471.1	142.00	-27.17	2 21	2603 2626	21
mmu-miR-7005-5p	NM_008423.2	150.00	-25.87	2 21	3132 3152	19
mmu-miR-7005-5p	NM_021342.1	144.00	-23.30	2 21	1354 1375	19
mmu-miR-7005-5p	NM_010608.2	169.00	-29.76	2 21	1931 1950	19
mmu-miR-7005-5p	NM_010608.2	156.00	-21.65	2 17	1513 1534	15
mmu-miR-7005-5p	NM_010608.2	146.00	-28.06	2 16	3695 3717	15
mmu-miR-7005-5p	NM_010608.2	145.00	-28.20	3 21	1151 1173	20
mmu-miR-7005-5p	NM_010608.2	144.00	-19.36	2 13	2052 2073	11
mmu-miR-7005-5p	NM_010608.2	143.00	-25.25	2 21	3155 3177	20
mmu-miR-7005-5p	NM_010608.2	141.00	-20.13	2 10	1477 1498	8
mmu-miR-7005-5p	XM_006497892.3	155.00	-24.47	2 20	4238 4259	18
mmu-miR-7005-5p	XM_006497892.3	154.00	-21.08	2 20	2738 2760	19
mmu-miR-7005-5p	XM_006497892.3	149.00	-31.01	2 20	1188 1213	22
mmu-miR-7005-5p	XM_006497892.3	146.00	-32.72	2 21	4582 4607	23
mmu-miR-7005-5p	XM_006497892.3	145.00	-20.27	2 10	4922 4943	8
mmu-miR-7005-5p	XM_006497892.3	141.00	-19.20	2 19	5140 5162	18
mmu-miR-7005-5p	XM_011248919.2	163.00	-26.64	2 17	2836 2858	16
mmu-miR-7005-5p	XM_011248919.2	155.00	-21.52	2 16	6383 6404	14
mmu-miR-7005-5p	XM_011248919.2	152.00	-28.76	2 13	2227 2248	11
mmu-miR-7005-5p	XM_011248919.2	147.00	-31.18	2 18	435 455	16
mmu-miR-7005-5p	XM_011248919.2	143.00	-23.03	2 16	2686 2707	14
mmu-miR-7005-5p	XM_011248919.2	140.00	-24.82	3 18	6057 6080	17
mmu-miR-7005-5p	XM_017316478.1	142.00	-22.67	3 17	3904 3924	14
mmu-miR-7005-5p	NM_173427.2	145.00	-23.45	2 10	4157 4178	8
mmu-miR-7005-5p	NM_173427.2	142.00	-22.20	2 11	1866 1887	9
mmu-miR-7005-5p	XM_017320093.1	146.00	-25.59	2 21	216 236	19
mmu-miR-7005-5p	NM_001081667.2	155.00	-22.43	2 21	816 838	20
mmu-miR-7005-5p	XM_006512296.3	147.00	-25.72	2 21	1170 1192	20
mmu-miR-7005-5p	XM_006512296.3	145.00	-27.48	3 21	1668 1690	20
mmu-miR-7005-5p	NM_008940.3	159.00	-24.69	2 21	398 421	21
mmu-miR-7005-5p	NM_008940.3	156.00	-29.50	2 19	192 212	17
mmu-miR-7005-5p	NM_008940.3	143.00	-35.50	2 16	133 154	14
mmu-miR-7005-5p	NM_008940.3	141.00	-16.95	2 10	72 93	8
mmu-miR-7005-5p	NM_029274.2	159.00	-34.76	2 21	5640 5663	21
mmu-miR-7005-5p	NM_029274.2	150.00	-29.29	2 16	1740 1762	15
mmu-miR-7005-5p	NM_029274.2	145.00	-24.30	2 16	7182 7202	14
mmu-miR-7005-5p	NM_029274.2	142.00	-23.14	3 19	5274 5295	16
mmu-miR-7005-5p	XM_017316676.1	159.00	-27.71	2 17	12808 12830	16
mmu-miR-7005-5p	XM_017316676.1	156.00	-31.20	2 17	3155 3176	15
mmu-miR-7005-5p	XM_017316676.1	152.00	-25.01	3 21	19211 19232	18
mmu-miR-7005-5p	XM_017316676.1	150.00	-20.95	2 11	5693 5714	9
mmu-miR-7005-5p	XM_017316676.1	148.00	-20.38	2 13	2458 2479	11
mmu-miR-7005-5p	XM_017316676.1	145.00	-33.05	2 14	2299 2320	12
mmu-miR-7005-5p	XM_017316676.1	143.00	-28.43	2 19	383 402	17
mmu-miR-7005-5p	XM_017316676.1	143.00	-28.32	2 21	11727 11749	20
mmu-miR-7005-5p	XM_017316676.1	143.00	-26.49	3 21	13371 13393	19
mmu-miR-7005-5p	XM_017316676.1	143.00	-21.27	2 21	18050 18073	21
mmu-miR-7005-5p	XM_017316676.1	140.00	-23.32	2 18	7390 7414	19
mmu-miR-7005-5p	XM_017316676.1	140.00	-20.57	2 9	9762 9783	7
mmu-miR-7005-5p	XM_017316676.1	140.00	-16.40	2 17	16953 16974	15

mmu-miR-7005-5p	NM_008479.2	148.00	-21.88	2 17	117 138	15
mmu-miR-7005-5p	XM_017313062.1	145.00	-16.11	2 10	1521 1542	8
mmu-miR-7005-5p	XM_017313062.1	140.00	-20.14	2 21	3672 3691	19
mmu-miR-7005-5p	XM_006523061.3	140.00	-15.10	2 9	345 366	7
mmu-miR-7005-5p	XM_006527221.3	144.00	-24.52	2 19	382 406	20
mmu-miR-7005-5p	NM_144945.3	149.00	-20.44	2 20	760 784	21
mmu-miR-7005-5p	NM_144945.3	142.00	-22.57	3 21	1383 1407	21
mmu-miR-7005-5p	XM_006519969.3	140.00	-14.33	2 9	2906 2927	7
mmu-miR-7005-5p	NM_053098.2	143.00	-20.89	3 21	1408 1430	19
mmu-miR-7005-5p	NM_175271.4	142.00	-26.10	2 21	1033 1053	19
mmu-miR-7005-5p	XM_006506340.3	147.00	-24.49	2 16	3880 3901	14
mmu-miR-7005-5p	XM_006506340.3	147.00	-29.77	2 17	5066 5088	16
mmu-miR-7005-5p	XM_006506340.3	141.00	-20.92	2 15	3782 3804	14
mmu-miR-7005-5p	NM_028973.2	148.00	-18.46	2 13	4902 4923	11
mmu-miR-7005-5p	NM_028973.2	146.00	-24.21	2 19	4068 4089	17
mmu-miR-7005-5p	NM_028973.2	142.00	-24.97	2 21	1070 1094	22
mmu-miR-7005-5p	XM_006527321.2	146.00	-23.80	2 20	135 157	19
mmu-miR-7005-5p	XM_006527321.2	142.00	-28.98	2 21	1150 1175	23
mmu-miR-7005-5p	XM_006527321.2	140.00	-19.31	3 17	497 518	14
mmu-miR-7005-5p	XM_006506086.2	158.00	-30.21	2 21	1523 1546	21
mmu-miR-7005-5p	XM_006506086.2	145.00	-31.41	2 18	1607 1628	16
mmu-miR-7005-5p	NM_001171187.1	147.00	-31.41	3 21	892 916	21
mmu-miR-7005-5p	XM_006541232.3	156.00	-37.18	2 21	2898 2919	19
mmu-miR-7005-5p	XM_006541232.3	149.00	-32.74	2 21	2747 2768	20
mmu-miR-7005-5p	XM_006541232.3	145.00	-26.01	2 21	2376 2399	22
mmu-miR-7005-5p	XM_006541232.3	140.00	-22.23	2 17	5536 5557	15
mmu-miR-7005-5p	XM_006498872.3	152.00	-22.31	2 20	3294 3320	23
mmu-miR-7005-5p	XM_006498872.3	147.00	-19.74	2 21	4262 4284	20
mmu-miR-7005-5p	XM_006498872.3	147.00	-21.46	3 20	10332 10353	17
mmu-miR-7005-5p	XM_006498872.3	142.00	-23.53	3 16	6046 6069	15
mmu-miR-7005-5p	XM_006498872.3	142.00	-20.92	2 21	6220 6240	19
mmu-miR-7005-5p	XM_006498872.3	140.00	-16.73	2 21	345 366	19
mmu-miR-7005-5p	XM_006498872.3	140.00	-20.57	2 9	1547 1568	7
mmu-miR-7005-5p	XM_006498872.3	140.00	-19.66	2 9	4173 4194	7
mmu-miR-7005-5p	XM_006498872.3	140.00	-23.98	3 13	6930 6951	10
mmu-miR-7005-5p	XM_006498872.3	140.00	-23.70	2 17	9831 9852	15
mmu-miR-7005-5p	XM_006532410.3	147.00	-29.34	2 21	5954 5976	20
mmu-miR-7005-5p	NM_001290273.1	144.00	-25.00	2 21	149 170	19
mmu-miR-7005-5p	NM_001290273.1	142.00	-29.36	2 21	298 322	22
mmu-miR-7005-5p	XM_011243266.1	158.00	-32.25	2 16	947 969	15
mmu-miR-7005-5p	XM_011243266.1	158.00	-28.72	2 19	3716 3737	17
mmu-miR-7005-5p	XM_011243266.1	157.00	-27.69	2 19	1993 2015	18
mmu-miR-7005-5p	XM_011243266.1	156.00	-27.75	2 21	3964 3985	19
mmu-miR-7005-5p	XM_011243266.1	150.00	-23.70	2 20	847 871	21
mmu-miR-7005-5p	XM_011243266.1	148.00	-23.59	2 18	1894 1916	17
mmu-miR-7005-5p	XM_011243266.1	148.00	-26.60	2 21	3853 3874	19
mmu-miR-7005-5p	XM_011243266.1	147.00	-23.79	2 12	1785 1806	10
mmu-miR-7005-5p	XM_011243266.1	147.00	-39.04	2 21	2192 2215	21
mmu-miR-7005-5p	XM_011243266.1	147.00	-26.82	2 12	2705 2726	10
mmu-miR-7005-5p	XM_011243266.1	145.00	-24.99	3 21	4094 4113	18
mmu-miR-7005-5p	XM_011243266.1	144.00	-25.52	2 21	2738 2759	19
mmu-miR-7005-5p	XM_011243266.1	143.00	-24.97	3 21	500 518	18
mmu-miR-7005-5p	XM_011243266.1	143.00	-31.21	2 21	3389 3411	20
mmu-miR-7005-5p	XM_011243266.1	142.00	-23.21	2 17	1070 1090	15
mmu-miR-7005-5p	XM_011243266.1	141.00	-26.12	3 21	929 948	18
mmu-miR-7005-5p	XM_006504940.3	151.00	-30.08	2 16	1162 1183	14
mmu-miR-7005-5p	NM_010825.3	154.00	-20.76	2 19	1437 1458	17
mmu-miR-7005-5p	NM_001270475.1	154.00	-27.01	2 21	3814 3834	19
mmu-miR-7005-5p	NM_001270475.1	148.00	-26.86	2 19	3766 3790	20
mmu-miR-7005-5p	NM_001270475.1	145.00	-19.67	2 10	6845 6866	8
mmu-miR-7005-5p	NM_001270475.1	143.00	-29.46	2 21	8731 8754	21
mmu-miR-7005-5p	XM_006504613.3	163.00	-32.64	2 20	455 474	18
mmu-miR-7005-5p	XM_006504613.3	141.00	-30.15	2 19	226 248	18
mmu-miR-7005-5p	NM_008623.5	143.00	-25.29	2 17	1166 1188	16
mmu-miR-7005-5p	NM_008623.5	140.00	-21.50	2 21	228 246	19

mmu-miR-7005-5p	NM_001005423.2	143.00	-27.58	2 19	973 992	17
mmu-miR-7005-5p	NM_001005423.2	142.00	-26.93	3 21	179 199	18
mmu-miR-7005-5p	NM_007641.5	148.00	-22.43	2 17	1592 1613	15
mmu-miR-7005-5p	NM_001099314.1	142.00	-23.07	2 21	35 55	19
mmu-miR-7005-5p	XM_006537659.2	145.00	-20.92	2 21	152 173	20
mmu-miR-7005-5p	XM_006529753.2	142.00	-15.79	2 11	300 321	9
mmu-miR-7005-5p	NM_001039545.2	142.00	-23.51	2 16	59 81	15
mmu-miR-7005-5p	NM_001039545.2	142.00	-15.67	2 11	2258 2279	9
mmu-miR-7005-5p	XM_006530186.3	142.00	-23.72	2 21	77 100	21
mmu-miR-7005-5p	XM_006530186.3	140.00	-22.12	3 18	793 815	16
mmu-miR-7005-5p	XM_006513859.3	150.00	-24.85	2 20	3522 3544	19
mmu-miR-7005-5p	XM_017320223.1	154.00	-26.15	2 21	185 208	21
mmu-miR-7005-5p	XM_017320223.1	143.00	-23.43	2 21	1238 1262	22
mmu-miR-7005-5p	XM_017320223.1	143.00	-22.24	2 14	4943 4963	12
mmu-miR-7005-5p	XM_017320223.1	141.00	-27.38	2 14	345 366	12
mmu-miR-7005-5p	XM_006501829.3	150.00	-18.68	2 11	468 489	9
mmu-miR-7005-5p	NM_173437.2	158.00	-25.23	2 21	11321 11341	19
mmu-miR-7005-5p	NM_173437.2	151.00	-23.52	2 21	2716 2738	20
mmu-miR-7005-5p	NM_173437.2	151.00	-31.13	2 21	8727 8749	20
mmu-miR-7005-5p	NM_173437.2	147.00	-33.07	2 21	4687 4709	20
mmu-miR-7005-5p	NM_173437.2	144.00	-24.82	2 13	9550 9571	11
mmu-miR-7005-5p	NM_173437.2	142.00	-22.97	2 21	3227 3250	21
mmu-miR-7005-5p	NM_173437.2	140.00	-15.78	2 9	2305 2326	7
mmu-miR-7005-5p	XM_006541297.3	156.00	-29.25	2 15	2365 2385	13
mmu-miR-7005-5p	XM_006541297.3	147.00	-20.63	2 12	3884 3905	10
mmu-miR-7005-5p	XM_006541297.3	142.00	-26.76	3 17	475 495	14
mmu-miR-7005-5p	XM_006541297.3	141.00	-26.70	3 21	6788 6809	19
mmu-miR-7005-5p	NM_001242558.1	142.00	-18.33	2 11	2197 2218	9
mmu-miR-7005-5p	NM_001242558.1	140.00	-18.53	2 9	898 919	7
mmu-miR-7005-5p	NM_011424.3	165.00	-30.45	2 20	2712 2732	18
mmu-miR-7005-5p	NM_011424.3	155.00	-25.91	2 21	2790 2812	20
mmu-miR-7005-5p	NM_011424.3	154.00	-25.90	2 21	2682 2702	19
mmu-miR-7005-5p	NM_011424.3	149.00	-27.29	2 19	5551 5573	18
mmu-miR-7005-5p	NM_011424.3	145.00	-24.19	3 19	3491 3513	17
mmu-miR-7005-5p	NM_011424.3	142.00	-17.91	2 11	3855 3876	9
mmu-miR-7005-5p	NM_011424.3	141.00	-18.01	2 10	6779 6800	8
mmu-miR-7005-5p	XM_006529456.3	143.00	-24.42	2 21	3329 3354	23
mmu-miR-7005-5p	XM_006498901.3	152.00	-26.73	2 13	1892 1913	11
mmu-miR-7005-5p	XM_006498901.3	146.00	-17.34	2 21	2912 2932	19
mmu-miR-7005-5p	XM_006498901.3	141.00	-20.00	3 18	113 134	15
mmu-miR-7005-5p	XM_006510077.3	149.00	-27.54	2 18	338 359	16
mmu-miR-7005-5p	XM_006510077.3	146.00	-27.14	2 15	854 875	13
mmu-miR-7005-5p	XM_006510077.3	144.00	-19.92	2 13	1387 1408	11
mmu-miR-7005-5p	NM_001134300.2	158.00	-26.71	2 21	696 716	19
mmu-miR-7005-5p	NM_001134300.2	151.00	-27.89	2 20	4715 4736	18
mmu-miR-7005-5p	NM_001134300.2	148.00	-27.19	3 21	4328 4349	18
mmu-miR-7005-5p	NM_001134300.2	140.00	-22.08	2 21	4555 4573	19
mmu-miR-7005-5p	NM_001109985.1	157.00	-29.13	2 14	2477 2498	12
mmu-miR-7005-5p	NM_001029877.3	165.00	-25.56	2 18	3847 3868	16
mmu-miR-7005-5p	NM_001029877.3	159.00	-26.76	2 19	296 315	17
mmu-miR-7005-5p	NM_001029877.3	152.00	-25.89	2 13	1856 1877	11
mmu-miR-7005-5p	NM_001029877.3	146.00	-19.67	2 21	92 112	19
mmu-miR-7005-5p	NM_001029877.3	146.00	-21.19	3 19	2995 3016	16
mmu-miR-7005-5p	NM_001029877.3	140.00	-27.75	2 21	3545 3566	19
mmu-miR-7005-5p	NM_001077403.1	158.00	-30.19	2 21	996 1019	21
mmu-miR-7005-5p	NM_001077403.1	141.00	-20.87	2 18	5167 5188	16
mmu-miR-7005-5p	NM_198190.1	161.00	-24.99	2 21	264 283	19
mmu-miR-7005-5p	NM_001282961.1	149.00	-21.87	2 20	7383 7403	18
mmu-miR-7005-5p	NM_001282961.1	144.00	-26.99	2 13	2945 2966	11
mmu-miR-7005-5p	NM_145210.2	155.00	-26.32	2 20	1199 1220	18
mmu-miR-7005-5p	NM_145210.2	142.00	-17.56	2 15	433 454	13
mmu-miR-7005-5p	XM_006530325.3	144.00	-25.49	3 18	3474 3496	16
mmu-miR-7005-5p	NM_145226.2	162.00	-26.74	2 19	3075 3096	17
mmu-miR-7005-5p	NM_145226.2	150.00	-22.03	2 21	1406 1430	22
mmu-miR-7005-5p	NM_145226.2	145.00	-23.04	2 10	3132 3153	8

mmu-miR-7005-5p	NM_145226.2	143.00	-20.31	2 13	1457 1479	12
mmu-miR-7005-5p	NM_145226.2	142.00	-21.62	2 17	1881 1901	15
mmu-miR-7005-5p	XM_006530294.3	156.00	-27.52	2 13	1237 1258	11
mmu-miR-7005-5p	NM_011854.2	142.00	-24.17	2 16	2290 2313	16
mmu-miR-7005-5p	NM_011854.2	140.00	-18.83	2 17	2782 2803	15
mmu-miR-7005-5p	XM_011239969.2	145.00	-24.08	2 21	1395 1414	19
mmu-miR-7005-5p	NM_001286743.1	156.00	-29.85	3 21	2552 2573	18
mmu-miR-7005-5p	NM_001286743.1	153.00	-24.66	2 16	3448 3468	14
mmu-miR-7005-5p	NM_001286743.1	151.00	-29.63	2 21	3883 3908	23
mmu-miR-7005-5p	NM_001286743.1	150.00	-18.67	2 20	3947 3969	19
mmu-miR-7005-5p	NM_001286743.1	145.00	-22.16	2 20	2130 2150	18
mmu-miR-7005-5p	NM_001286743.1	143.00	-28.09	2 16	184 205	14
mmu-miR-7005-5p	NM_001286743.1	143.00	-18.88	3 21	2763 2785	19
mmu-miR-7005-5p	XM_017322026.1	166.00	-32.89	2 15	483 504	13
mmu-miR-7009-5p	XM_006509531.3	148.00	-21.65	2 21	1545 1566	19
mmu-miR-7009-5p	XM_006509531.3	140.00	-16.19	2 9	753 774	7
mmu-miR-7009-5p	XM_006524407.3	144.00	-22.03	2 17	1041 1062	15
mmu-miR-7009-5p	NM_177743.5	165.00	-31.03	2 19	311 333	18
mmu-miR-7009-5p	NM_177743.5	141.00	-26.89	3 19	1445 1467	17
mmu-miR-7009-5p	XM_017319626.1	151.00	-26.03	2 21	166 188	20
mmu-miR-7009-5p	XM_017319626.1	144.00	-21.11	2 17	1121 1142	15
mmu-miR-7009-5p	XM_017319626.1	143.00	-27.56	3 21	3705 3729	21
mmu-miR-7009-5p	XM_017319626.1	140.00	-21.52	2 21	2004 2025	19
mmu-miR-7009-5p	XM_006532962.2	150.00	-26.70	2 19	1679 1700	17
mmu-miR-7009-5p	XM_006532962.2	143.00	-22.77	2 21	1326 1348	20
mmu-miR-7009-5p	XM_006532962.2	142.00	-26.55	2 21	1111 1136	23
mmu-miR-7009-5p	NM_001160262.1	149.00	-17.76	2 19	2423 2446	19
mmu-miR-7009-5p	NM_001160262.1	142.00	-15.45	2 11	3058 3079	9
mmu-miR-7009-5p	NM_001160262.1	141.00	-19.14	2 19	2018 2041	19
mmu-miR-7009-5p	NM_010181.2	152.00	-19.10	2 18	4697 4719	17
mmu-miR-7009-5p	NM_010181.2	147.00	-19.96	2 12	2075 2096	10
mmu-miR-7009-5p	NM_010181.2	146.00	-27.47	2 19	8854 8875	17
mmu-miR-7009-5p	NM_010181.2	140.00	-16.65	2 9	6273 6294	7
mmu-miR-7009-5p	XM_011244488.2	151.00	-22.44	2 19	234 253	17
mmu-miR-7009-5p	XM_011244488.2	150.00	-27.24	3 20	472 494	18
mmu-miR-7009-5p	NM_145141.2	155.00	-37.94	2 21	898 920	20
mmu-miR-7009-5p	NM_145141.2	152.00	-26.59	3 21	948 969	18
mmu-miR-7009-5p	NM_001081185.1	153.00	-23.19	2 19	4339 4361	18
mmu-miR-7009-5p	NM_001081185.1	151.00	-30.35	3 20	3476 3497	17
mmu-miR-7009-5p	NM_001081185.1	146.00	-27.20	2 20	921 944	20
mmu-miR-7009-5p	NM_001081185.1	141.00	-24.43	3 18	8323 8344	15
mmu-miR-7009-5p	XM_006496949.3	140.00	-20.10	2 21	1539 1560	19
mmu-miR-7009-5p	NM_172862.3	141.00	-19.70	2 18	1627 1648	16
mmu-miR-7009-5p	NM_001033221.3	162.00	-28.85	2 17	119 139	15
mmu-miR-7009-5p	NM_001033221.3	140.00	-20.40	2 9	210 231	7
mmu-miR-7009-5p	NM_001033445.2	146.00	-22.36	2 15	2171 2192	13
mmu-miR-7009-5p	NM_001033445.2	145.00	-19.95	2 10	4469 4490	8
mmu-miR-7009-5p	NM_001033445.2	140.00	-19.44	2 9	2615 2636	7
mmu-miR-7009-5p	XM_006514444.1	140.00	-27.81	3 21	1536 1557	18
mmu-miR-7009-5p	NM_145741.2	165.00	-31.27	2 19	767 790	19
mmu-miR-7009-5p	NM_010279.3	140.00	-14.07	2 9	4108 4129	7
mmu-miR-7009-5p	XM_006510297.3	147.00	-36.49	2 21	309 333	22
mmu-miR-7009-5p	XM_006530353.3	153.00	-25.28	2 15	7859 7882	15
mmu-miR-7009-5p	XM_006530353.3	153.00	-26.11	2 14	12451 12472	12
mmu-miR-7009-5p	XM_006530353.3	141.00	-23.75	3 21	8634 8653	18
mmu-miR-7009-5p	XM_006530353.3	141.00	-18.53	2 18	12846 12865	16
mmu-miR-7009-5p	XM_006530353.3	140.00	-18.08	2 9	6349 6370	7
mmu-miR-7009-5p	XM_011248576.2	143.00	-27.56	3 21	564 587	20
mmu-miR-7009-5p	XM_011248576.2	143.00	-26.35	2 17	7682 7704	16
mmu-miR-7009-5p	XM_011248576.2	142.00	-17.91	2 11	7848 7869	9
mmu-miR-7009-5p	XM_011248576.2	140.00	-27.14	2 15	3732 3752	13
mmu-miR-7009-5p	XM_011244235.2	155.00	-22.39	2 16	1305 1326	14
mmu-miR-7009-5p	NM_001198955.1	158.00	-32.24	2 19	720 741	17
mmu-miR-7009-5p	XM_011250978.2	140.00	-14.69	2 9	2896 2917	7
mmu-miR-7009-5p	NM_010340.2	140.00	-23.81	2 9	1215 1236	7

mmu-miR-7009-5p	XM_006521554.3	144.00	-20.67	2 21	200 221	19
mmu-miR-7009-5p	XM_006515096.2	150.00	-22.12	2 20	3186 3208	19
mmu-miR-7009-5p	XM_006515096.2	143.00	-20.95	2 20	6182 6203	18
mmu-miR-7009-5p	XM_006526069.2	168.00	-24.85	2 21	1650 1671	19
mmu-miR-7009-5p	XM_006526069.2	150.00	-26.41	2 21	3824 3847	21
mmu-miR-7009-5p	XM_006526069.2	148.00	-18.54	2 20	4687 4708	19
mmu-miR-7009-5p	XM_006526069.2	147.00	-18.28	2 12	4426 4447	10
mmu-miR-7009-5p	XM_006526069.2	143.00	-22.69	3 21	4477 4499	19
mmu-miR-7009-5p	NM_008185.3	141.00	-25.92	2 19	741 765	20
mmu-miR-7009-5p	XM_011246309.2	144.00	-27.83	2 21	199 220	19
mmu-miR-7009-5p	XM_011246309.2	143.00	-15.35	2 12	1389 1410	10
mmu-miR-7009-5p	NM_175520.4	143.00	-22.71	3 21	1160 1182	19
mmu-miR-7009-5p	NM_001162950.1	166.00	-29.08	2 21	5112 5132	19
mmu-miR-7009-5p	NM_001162950.1	160.00	-29.33	2 21	3019 3040	19
mmu-miR-7009-5p	NM_001162950.1	153.00	-31.81	2 21	2378 2397	19
mmu-miR-7009-5p	NM_001162950.1	143.00	-23.90	3 21	466 488	19
mmu-miR-7009-5p	NM_001162950.1	142.00	-20.00	3 19	4441 4462	16
mmu-miR-7009-5p	NM_001162950.1	140.00	-19.30	2 9	1342 1363	7
mmu-miR-7009-5p	XM_006505602.3	154.00	-21.47	2 19	9124 9145	17
mmu-miR-7009-5p	XM_006505602.3	150.00	-26.50	2 15	12672 12693	13
mmu-miR-7009-5p	XM_006505602.3	142.00	-25.40	2 20	1248 1271	20
mmu-miR-7009-5p	XM_006505602.3	142.00	-25.78	2 19	9858 9879	17
mmu-miR-7009-5p	NM_001164086.1	147.00	-24.98	2 20	197 218	20
mmu-miR-7009-5p	NM_001164086.1	147.00	-26.90	3 16	7060 7081	13
mmu-miR-7009-5p	NM_001164086.1	146.00	-28.33	3 20	2688 2710	18
mmu-miR-7009-5p	NM_001164086.1	145.00	-31.42	2 21	2282 2311	27
mmu-miR-7009-5p	NM_001164086.1	145.00	-20.36	2 10	2979 3000	8
mmu-miR-7009-5p	NM_001164086.1	141.00	-23.83	3 19	3409 3431	17
mmu-miR-7009-5p	NM_010450.3	147.00	-26.68	2 21	187 209	20
mmu-miR-7009-5p	NM_008277.2	142.00	-25.07	2 19	479 500	17
mmu-miR-7009-5p	NM_001327998.1	151.00	-22.51	2 21	3267 3289	20
mmu-miR-7009-5p	NM_001327998.1	148.00	-22.40	2 13	630 651	11
mmu-miR-7009-5p	NM_001327998.1	146.00	-23.47	2 20	2333 2355	19
mmu-miR-7009-5p	NM_013560.2	140.00	-19.88	2 9	583 604	7
mmu-miR-7009-5p	XM_006539957.3	148.00	-21.94	2 21	426 447	19
mmu-miR-7009-5p	XM_006539957.3	147.00	-23.93	2 21	797 819	20
mmu-miR-7009-5p	NM_030704.3	152.00	-18.92	2 21	1496 1517	19
mmu-miR-7009-5p	NM_030704.3	147.00	-26.28	2 21	183 205	20
mmu-miR-7009-5p	NM_133871.2	141.00	-23.29	2 14	2231 2252	12
mmu-miR-7009-5p	XM_006511311.2	160.00	-26.13	2 18	1019 1042	18
mmu-miR-7009-5p	XM_006511311.2	148.00	-20.46	2 21	5440 5458	19
mmu-miR-7009-5p	XM_006511311.2	142.00	-33.20	2 21	4997 5017	19
mmu-miR-7009-5p	XM_006511311.2	141.00	-27.63	2 19	2648 2670	18
mmu-miR-7009-5p	XM_017316439.1	143.00	-35.24	2 21	1326 1348	20
mmu-miR-7009-5p	XM_006496593.3	141.00	-27.74	2 19	1837 1859	18
mmu-miR-7009-5p	XM_006516224.3	150.00	-18.89	2 21	217 237	19
mmu-miR-7009-5p	XM_006528842.1	158.00	-28.75	2 21	1067 1087	19
mmu-miR-7009-5p	XM_006528842.1	155.00	-29.13	2 16	4361 4382	14
mmu-miR-7009-5p	XM_006528842.1	145.00	-27.64	2 14	5334 5355	12
mmu-miR-7009-5p	NM_001161541.1	155.00	-25.90	2 21	3349 3372	21
mmu-miR-7009-5p	NM_001161541.1	146.00	-23.71	2 21	2092 2112	19
mmu-miR-7009-5p	NM_001302471.1	140.00	-16.51	2 9	977 998	7
mmu-miR-7009-5p	NM_001289875.1	145.00	-23.11	2 10	3294 3315	8
mmu-miR-7009-5p	NM_008423.2	140.00	-32.35	2 21	3130 3151	19
mmu-miR-7009-5p	XM_017315734.1	143.00	-19.85	2 16	1863 1884	14
mmu-miR-7009-5p	NM_010608.2	144.00	-26.07	3 17	1707 1728	14
mmu-miR-7009-5p	XM_011248919.2	152.00	-23.39	2 14	1872 1895	14
mmu-miR-7009-5p	XM_011248919.2	152.00	-23.98	2 13	6253 6274	11
mmu-miR-7009-5p	XM_011248919.2	150.00	-22.34	2 19	4419 4440	17
mmu-miR-7009-5p	XM_011248919.2	148.00	-30.12	3 19	1496 1516	16
mmu-miR-7009-5p	XM_011248919.2	146.00	-26.30	3 19	3155 3176	16
mmu-miR-7009-5p	NM_031180.2	145.00	-28.79	2 21	3315 3334	19
mmu-miR-7009-5p	NM_173427.2	142.00	-20.91	2 15	3932 3953	13
mmu-miR-7009-5p	NM_173427.2	141.00	-24.56	2 14	3477 3498	12
mmu-miR-7009-5p	NM_173427.2	140.00	-24.94	2 21	2344 2365	19

mmu-miR-7009-5p	NM_173427.2	140.00	-21.93	2 21	2413 2434	19
mmu-miR-7009-5p	NM_173427.2	140.00	-23.86	2 17	2982 3003	15
mmu-miR-7009-5p	NM_173427.2	140.00	-23.47	2 21	4496 4517	19
mmu-miR-7009-5p	XM_006512296.3	152.00	-22.80	2 20	2079 2100	19
mmu-miR-7009-5p	NM_008940.3	143.00	-24.76	3 21	1162 1184	19
mmu-miR-7009-5p	NM_029274.2	159.00	-23.86	2 21	6454 6476	20
mmu-miR-7009-5p	NM_029274.2	148.00	-31.07	3 21	6693 6714	18
mmu-miR-7009-5p	NM_029274.2	144.00	-26.22	2 17	6371 6392	15
mmu-miR-7009-5p	NM_029274.2	140.00	-15.67	2 9	4462 4483	7
mmu-miR-7009-5p	NM_029274.2	140.00	-20.02	2 9	4660 4681	7
mmu-miR-7009-5p	XM_017316676.1	162.00	-39.58	2 21	3806 3830	22
mmu-miR-7009-5p	XM_017316676.1	162.00	-29.27	2 16	7406 7429	16
mmu-miR-7009-5p	XM_017316676.1	146.00	-30.08	2 21	3582 3605	21
mmu-miR-7009-5p	XM_017316676.1	145.00	-28.33	2 21	7840 7859	19
mmu-miR-7009-5p	XM_017316676.1	144.00	-31.49	3 21	7475 7496	18
mmu-miR-7009-5p	XM_017316676.1	142.00	-24.60	2 21	7495 7521	24
mmu-miR-7009-5p	XM_017316676.1	141.00	-26.28	3 19	3606 3628	17
mmu-miR-7009-5p	XM_017316676.1	141.00	-33.88	2 19	7804 7827	19
mmu-miR-7009-5p	XM_017316676.1	141.00	-23.96	2 14	10279 10300	12
mmu-miR-7009-5p	XM_017316676.1	140.00	-21.56	3 21	3250 3271	18
mmu-miR-7009-5p	XM_017316676.1	140.00	-24.57	2 21	7290 7311	19
mmu-miR-7009-5p	XM_017313062.1	146.00	-33.40	3 19	2767 2788	16
mmu-miR-7009-5p	XM_006511102.2	143.00	-22.36	2 18	1395 1418	18
mmu-miR-7009-5p	XM_006519969.3	154.00	-26.33	2 19	2075 2096	17
mmu-miR-7009-5p	NM_144799.2	140.00	-17.08	2 9	487 508	7
mmu-miR-7009-5p	NM_175271.4	141.00	-27.03	3 14	1555 1576	11
mmu-miR-7009-5p	NM_028973.2	141.00	-29.52	2 20	4909 4929	18
mmu-miR-7009-5p	NM_028973.2	140.00	-18.24	2 9	3624 3645	7
mmu-miR-7009-5p	XM_006527321.2	143.00	-22.30	2 21	34 56	20
mmu-miR-7009-5p	XM_006527321.2	141.00	-24.92	2 14	824 845	12
mmu-miR-7009-5p	NM_001171187.1	140.00	-20.93	2 21	2163 2183	20
mmu-miR-7009-5p	XM_006541232.3	159.00	-38.02	2 21	2896 2918	20
mmu-miR-7009-5p	XM_006541232.3	156.00	-20.83	2 21	96 117	19
mmu-miR-7009-5p	XM_006541232.3	150.00	-22.99	2 11	4316 4337	9
mmu-miR-7009-5p	XM_006541232.3	146.00	-29.36	2 21	2923 2948	23
mmu-miR-7009-5p	XM_006541232.3	145.00	-27.23	2 19	1338 1360	18
mmu-miR-7009-5p	XM_006541232.3	142.00	-31.86	2 21	2397 2422	23
mmu-miR-7009-5p	XM_006541232.3	140.00	-22.26	3 21	963 984	18
mmu-miR-7009-5p	XM_006541232.3	140.00	-18.76	2 21	3394 3415	19
mmu-miR-7009-5p	XM_006498872.3	151.00	-33.51	2 21	6238 6260	20
mmu-miR-7009-5p	XM_006498872.3	150.00	-21.58	2 11	2630 2651	9
mmu-miR-7009-5p	XM_006498872.3	140.00	-18.97	2 9	6923 6944	7
mmu-miR-7009-5p	XM_006532410.3	149.00	-28.80	2 14	2960 2981	12
mmu-miR-7009-5p	XM_006532410.3	147.00	-28.89	3 21	1017 1039	19
mmu-miR-7009-5p	XM_006532410.3	140.00	-19.46	2 21	4733 4754	19
mmu-miR-7009-5p	XM_006532410.3	140.00	-26.91	2 21	6116 6134	19
mmu-miR-7009-5p	XM_006520023.2	146.00	-26.76	2 19	948 969	17
mmu-miR-7009-5p	XM_006520023.2	144.00	-22.69	2 13	1567 1588	11
mmu-miR-7009-5p	NM_013593.3	153.00	-25.58	2 21	876 898	21
mmu-miR-7009-5p	XM_011243266.1	159.00	-36.55	2 21	680 702	20
mmu-miR-7009-5p	XM_011243266.1	148.00	-26.51	2 21	2326 2347	19
mmu-miR-7009-5p	XM_011243266.1	147.00	-30.25	2 20	1254 1275	18
mmu-miR-7009-5p	XM_011243266.1	147.00	-35.08	3 21	1284 1306	19
mmu-miR-7009-5p	XM_011243266.1	146.00	-26.02	3 15	2959 2980	12
mmu-miR-7009-5p	XM_011243266.1	143.00	-26.41	2 21	53 76	21
mmu-miR-7009-5p	XM_011243266.1	140.00	-21.88	3 17	1581 1602	14
mmu-miR-7009-5p	XM_011243266.1	140.00	-20.69	2 9	2624 2645	7
mmu-miR-7009-5p	NM_010825.3	159.00	-27.05	2 21	1443 1467	22
mmu-miR-7009-5p	NM_010825.3	147.00	-29.25	3 20	580 601	17
mmu-miR-7009-5p	NM_001270475.1	156.00	-22.80	2 21	7550 7571	19
mmu-miR-7009-5p	NM_001270475.1	153.00	-24.50	2 14	4514 4535	12
mmu-miR-7009-5p	NM_001270475.1	145.00	-18.99	2 10	5955 5976	8
mmu-miR-7009-5p	NM_001270475.1	140.00	-17.44	2 9	4215 4236	7
mmu-miR-7009-5p	NM_001290512.1	146.00	-25.74	3 21	2826 2846	18
mmu-miR-7009-5p	NM_001290512.1	142.00	-21.32	2 16	761 783	15

mmu-miR-7009-5p	NM_010809.2	145.00	-22.52	2 10	500 521	8
mmu-miR-7009-5p	XM_006504613.3	150.00	-24.37	2 11	634 655	9
mmu-miR-7009-5p	NM_008623.5	142.00	-26.56	2 19	1607 1628	17
mmu-miR-7009-5p	NM_205810.4	147.00	-29.30	2 21	2701 2723	20
mmu-miR-7009-5p	XM_006537659.2	144.00	-20.83	2 13	1760 1781	11
mmu-miR-7009-5p	XM_006529753.2	145.00	-22.35	2 10	1364 1385	8
mmu-miR-7009-5p	XM_006529753.2	145.00	-26.51	2 19	2382 2404	18
mmu-miR-7009-5p	XM_006529753.2	141.00	-27.02	2 19	1700 1723	19
mmu-miR-7009-5p	XM_006532412.1	144.00	-20.05	2 13	2141 2162	11
mmu-miR-7009-5p	XM_017315841.1	150.00	-25.42	2 19	243 264	17
mmu-miR-7009-5p	XM_017315841.1	147.00	-20.84	2 12	776 797	10
mmu-miR-7009-5p	NM_173437.2	150.00	-20.65	2 15	3166 3187	13
mmu-miR-7009-5p	NM_173437.2	149.00	-31.17	2 19	4685 4708	19
mmu-miR-7009-5p	NM_173437.2	149.00	-22.72	2 14	5623 5644	12
mmu-miR-7009-5p	NM_173437.2	148.00	-20.27	2 17	504 525	15
mmu-miR-7009-5p	NM_173437.2	148.00	-20.67	2 13	2122 2143	11
mmu-miR-7009-5p	NM_173437.2	148.00	-20.39	2 21	11874 11895	19
mmu-miR-7009-5p	NM_173437.2	148.00	-20.39	2 21	11971 11992	19
mmu-miR-7009-5p	XM_006541297.3	163.00	-31.78	2 21	9011 9033	20
mmu-miR-7009-5p	NM_001242558.1	145.00	-21.64	2 10	2176 2197	8
mmu-miR-7009-5p	NM_001242558.1	144.00	-20.12	2 21	795 816	19
mmu-miR-7009-5p	NM_011424.3	166.00	-28.24	2 21	5115 5135	19
mmu-miR-7009-5p	NM_011424.3	145.00	-23.29	2 10	1562 1583	8
mmu-miR-7009-5p	NM_011424.3	140.00	-15.62	2 9	2353 2374	7
mmu-miR-7009-5p	NM_011424.3	140.00	-26.58	2 17	6785 6806	15
mmu-miR-7009-5p	XM_006498901.3	142.00	-25.51	2 17	311 331	15
mmu-miR-7009-5p	XM_006510077.3	145.00	-25.43	2 21	1260 1279	19
mmu-miR-7009-5p	XM_006510077.3	142.00	-27.40	2 19	611 632	17
mmu-miR-7009-5p	NM_001134300.2	144.00	-27.33	2 21	1083 1104	19
mmu-miR-7009-5p	NM_001134300.2	144.00	-24.67	2 18	2025 2047	17
mmu-miR-7009-5p	NM_001134300.2	142.00	-28.15	2 19	1909 1930	17
mmu-miR-7009-5p	NM_001134300.2	141.00	-22.26	2 21	1718 1737	19
mmu-miR-7009-5p	NM_001029877.3	140.00	-22.58	2 19	8076 8096	17
mmu-miR-7009-5p	NM_001029877.3	140.00	-17.26	2 9	8126 8147	7
mmu-miR-7009-5p	XM_006526747.3	142.00	-19.84	2 15	4461 4482	13
mmu-miR-7009-5p	NM_001077403.1	145.00	-23.14	2 14	2492 2513	12
mmu-miR-7009-5p	NM_001077403.1	143.00	-24.43	2 21	5159 5181	20
mmu-miR-7009-5p	NM_001077403.1	140.00	-24.00	2 21	3461 3482	19
mmu-miR-7009-5p	NM_133500.2	140.00	-11.93	2 9	1 8	7
mmu-miR-7009-5p	XM_006530325.3	141.00	-22.20	2 19	2117 2140	19
mmu-miR-7009-5p	NM_145226.2	145.00	-28.48	2 19	1778 1801	19
mmu-miR-7009-5p	NM_145226.2	145.00	-25.75	2 19	2279 2302	19
mmu-miR-7009-5p	NM_145226.2	140.00	-21.70	2 17	4115 4136	15
mmu-miR-7009-5p	XM_006530294.3	161.00	-25.75	2 19	1129 1152	19
mmu-miR-7009-5p	NM_011854.2	161.00	-22.81	2 19	1646 1669	19
mmu-miR-7009-5p	NM_011854.2	144.00	-28.82	3 19	2602 2622	16
mmu-miR-7009-5p	NM_011854.2	142.00	-18.28	2 11	2756 2777	9
mmu-miR-7009-5p	NM_011010.2	150.00	-24.85	2 19	283 304	17
mmu-miR-7009-5p	NM_011010.2	149.00	-30.06	2 14	533 554	12
mmu-miR-7009-5p	XM_011239969.2	141.00	-28.94	2 21	29 48	19
mmu-miR-7009-5p	NM_001286743.1	159.00	-31.17	2 21	3593 3617	22
mmu-miR-7042-5p	XM_017319626.1	142.00	-18.53	2 20	485 506	19
mmu-miR-7042-5p	XM_006532962.2	149.00	-18.27	2 19	2572 2594	19
mmu-miR-7042-5p	NM_001160262.1	151.00	-20.10	2 16	3560 3580	14
mmu-miR-7042-5p	NM_001160262.1	145.00	-21.31	2 18	2108 2128	16
mmu-miR-7042-5p	NM_001160262.1	145.00	-18.35	2 18	2153 2173	16
mmu-miR-7042-5p	XM_006520437.2	146.00	-30.59	2 20	2015 2038	21
mmu-miR-7042-5p	NM_010181.2	153.00	-22.12	2 20	4010 4029	18
mmu-miR-7042-5p	NM_010181.2	151.00	-17.91	2 16	1135 1155	14
mmu-miR-7042-5p	XM_006527803.1	158.00	-24.43	2 20	1547 1568	19
mmu-miR-7042-5p	NM_001081185.1	149.00	-23.41	3 20	8657 8676	17
mmu-miR-7042-5p	NM_001081185.1	142.00	-19.39	2 12	7659 7681	12
mmu-miR-7042-5p	NM_172862.3	140.00	-21.67	2 13	11945 11965	11
mmu-miR-7042-5p	XM_006533539.3	165.00	-22.21	2 19	5408 5429	18
mmu-miR-7042-5p	XM_006533539.3	161.00	-21.28	2 18	4913 4933	16

mmu-miR-7042-5p	XM_006533539.3	144.00	-17.99	3 17	7872 7892	14
mmu-miR-7042-5p	NM_001033221.3	141.00	-15.52	2 19	1176 1197	18
mmu-miR-7042-5p	NM_001033445.2	162.00	-24.75	2 17	2691 2710	15
mmu-miR-7042-5p	XM_006510297.3	160.00	-22.65	2 18	2666 2687	17
mmu-miR-7042-5p	XM_006510297.3	156.00	-18.36	2 18	3620 3641	17
mmu-miR-7042-5p	XM_006510297.3	149.00	-20.29	2 19	3606 3627	18
mmu-miR-7042-5p	XM_006530353.3	160.00	-26.79	2 19	14601 14626	22
mmu-miR-7042-5p	XM_006530353.3	142.00	-15.87	2 11	8281 8301	9
mmu-miR-7042-5p	XM_011248576.2	141.00	-19.68	2 19	12317 12338	18
mmu-miR-7042-5p	XM_011244235.2	156.00	-16.85	2 17	1080 1100	15
mmu-miR-7042-5p	XM_011244235.2	141.00	-21.17	2 10	1044 1064	8
mmu-miR-7042-5p	NM_001198955.1	161.00	-21.57	2 18	1286 1306	16
mmu-miR-7042-5p	NM_001198955.1	141.00	-28.22	3 20	2105 2127	19
mmu-miR-7042-5p	NM_016697.3	147.00	-13.60	2 12	1056 1076	10
mmu-miR-7042-5p	NM_016697.3	141.00	-10.82	2 12	610 629	10
mmu-miR-7042-5p	XM_011250978.2	143.00	-14.36	2 16	2572 2592	14
mmu-miR-7042-5p	XM_011250978.2	140.00	-18.63	2 18	1817 1838	17
mmu-miR-7042-5p	XM_006515096.2	162.00	-24.11	2 20	6092 6113	19
mmu-miR-7042-5p	XM_006515096.2	140.00	-17.47	2 9	2482 2502	7
mmu-miR-7042-5p	XM_006515096.2	140.00	-13.28	2 9	5626 5646	7
mmu-miR-7042-5p	NM_175520.4	140.00	-19.02	2 18	2296 2318	18
mmu-miR-7042-5p	NM_001162950.1	156.00	-18.74	2 17	5645 5665	15
mmu-miR-7042-5p	NM_001162950.1	155.00	-23.02	2 12	1085 1105	10
mmu-miR-7042-5p	NM_001162950.1	152.00	-15.19	2 17	5548 5568	15
mmu-miR-7042-5p	NM_001162950.1	149.00	-19.07	2 18	3045 3065	16
mmu-miR-7042-5p	NM_001162950.1	145.00	-17.51	2 19	6650 6671	18
mmu-miR-7042-5p	NM_001162950.1	143.00	-21.45	3 20	6061 6081	17
mmu-miR-7042-5p	NM_001162950.1	142.00	-17.87	2 19	4304 4324	17
mmu-miR-7042-5p	NM_001162950.1	141.00	-18.54	2 18	2312 2332	16
mmu-miR-7042-5p	XM_006505602.3	149.00	-25.80	2 20	7976 7999	21
mmu-miR-7042-5p	XM_006505602.3	141.00	-12.16	2 10	9522 9542	8
mmu-miR-7042-5p	NM_001164086.1	160.00	-22.75	2 18	9391 9412	17
mmu-miR-7042-5p	NM_001164086.1	157.00	-24.68	2 18	9808 9828	16
mmu-miR-7042-5p	NM_001164086.1	153.00	-16.63	2 16	6764 6783	14
mmu-miR-7042-5p	NM_001164086.1	147.00	-21.38	2 18	10138 10163	21
mmu-miR-7042-5p	NM_001164086.1	145.00	-21.34	2 19	9530 9551	18
mmu-miR-7042-5p	NM_001164086.1	143.00	-21.72	3 20	7667 7687	17
mmu-miR-7042-5p	NM_001164086.1	140.00	-14.92	2 9	974 994	7
mmu-miR-7042-5p	NM_001164086.1	140.00	-19.79	2 17	10833 10853	15
mmu-miR-7042-5p	NM_013558.2	149.00	-18.41	2 14	1362 1382	12
mmu-miR-7042-5p	NM_013558.2	144.00	-14.61	2 15	2375 2394	13
mmu-miR-7042-5p	NM_013868.4	148.00	-19.53	2 19	2383 2402	17
mmu-miR-7042-5p	NM_030704.3	146.00	-20.25	2 13	329 348	11
mmu-miR-7042-5p	NM_023670.3	154.00	-20.47	2 20	2353 2374	19
mmu-miR-7042-5p	NM_023670.3	141.00	-16.71	2 14	2676 2696	12
mmu-miR-7042-5p	XM_017316439.1	142.00	-14.44	2 13	3182 3201	11
mmu-miR-7042-5p	XM_006496593.3	152.00	-19.22	2 13	5227 5247	11
mmu-miR-7042-5p	XM_006496593.3	141.00	-20.48	3 18	1038 1058	15
mmu-miR-7042-5p	XM_006496593.3	141.00	-14.26	2 10	3743 3763	8
mmu-miR-7042-5p	XM_006528842.1	143.00	-25.58	3 20	120 140	17
mmu-miR-7042-5p	NM_001302471.1	159.00	-19.16	2 20	4942 4962	18
mmu-miR-7042-5p	NM_001302471.1	150.00	-16.38	2 19	4628 4648	17
mmu-miR-7042-5p	NM_001302471.1	149.00	-17.91	2 19	366 387	18
mmu-miR-7042-5p	NM_001302471.1	143.00	-22.33	3 16	1841 1861	13
mmu-miR-7042-5p	XM_006518921.3	142.00	-15.51	2 16	1797 1818	15
mmu-miR-7042-5p	NM_008423.2	147.00	-14.65	2 12	929 949	10
mmu-miR-7042-5p	NM_008423.2	147.00	-18.04	2 13	2300 2322	13
mmu-miR-7042-5p	NM_008423.2	141.00	-15.07	2 10	3412 3432	8
mmu-miR-7042-5p	XM_017315734.1	149.00	-19.73	2 18	3176 3196	16
mmu-miR-7042-5p	XM_017315734.1	143.00	-16.92	2 20	4088 4108	18
mmu-miR-7042-5p	NM_010608.2	141.00	-22.97	2 18	2287 2307	16
mmu-miR-7042-5p	NM_010608.2	141.00	-20.88	2 10	2727 2747	8
mmu-miR-7042-5p	XM_006497892.3	148.00	-17.60	2 13	4527 4547	11
mmu-miR-7042-5p	XM_006497892.3	144.00	-15.86	2 13	2417 2437	11
mmu-miR-7042-5p	XM_011248919.2	152.00	-16.94	2 19	6612 6631	17

mmu-miR-7042-5p	XM_011248919.2	149.00	-19.31	2 18	5922 5942	16
mmu-miR-7042-5p	XM_017316676.1	153.00	-22.83	2 18	17575 17595	16
mmu-miR-7042-5p	XM_017316676.1	153.00	-19.10	2 18	17607 17627	16
mmu-miR-7042-5p	XM_017315313.1	145.00	-14.33	2 18	1885 1905	16
mmu-miR-7042-5p	NM_008479.2	165.00	-22.21	2 19	269 290	18
mmu-miR-7042-5p	NM_008479.2	149.00	-21.81	3 18	116 136	15
mmu-miR-7042-5p	XM_017313062.1	149.00	-14.19	2 16	231 250	14
mmu-miR-7042-5p	XM_006504999.1	150.00	-24.62	3 19	933 953	16
mmu-miR-7042-5p	NM_144945.3	141.00	-17.97	3 14	3472 3492	11
mmu-miR-7042-5p	NM_010714.3	143.00	-15.77	2 16	3934 3954	14
mmu-miR-7042-5p	NM_010714.3	141.00	-20.10	2 19	4381 4402	18
mmu-miR-7042-5p	XM_006519969.3	158.00	-20.09	2 19	3895 3915	17
mmu-miR-7042-5p	XM_006519969.3	146.00	-14.19	2 11	3290 3310	9
mmu-miR-7042-5p	XM_006519969.3	142.00	-10.08	2 11	521 541	9
mmu-miR-7042-5p	NM_144799.2	140.00	-21.35	2 17	143 163	15
mmu-miR-7042-5p	NM_175271.4	150.00	-18.40	2 20	1680 1702	20
mmu-miR-7042-5p	XM_006506340.3	163.00	-28.15	2 20	2546 2566	18
mmu-miR-7042-5p	XM_006506340.3	161.00	-21.06	2 16	2846 2865	14
mmu-miR-7042-5p	XM_006506340.3	154.00	-18.45	2 19	766 786	17
mmu-miR-7042-5p	NM_028973.2	157.00	-22.30	2 20	2306 2325	18
mmu-miR-7042-5p	NM_028973.2	145.00	-21.71	2 20	3056 3075	18
mmu-miR-7042-5p	NM_028973.2	140.00	-17.06	2 17	5078 5098	15
mmu-miR-7042-5p	XM_006506086.2	140.00	-16.96	2 17	6 26	15
mmu-miR-7042-5p	NM_008524.2	155.00	-19.17	2 18	69 88	16
mmu-miR-7042-5p	NM_001171187.1	142.00	-15.90	2 19	1606 1626	17
mmu-miR-7042-5p	XM_006541232.3	162.00	-26.21	2 20	1451 1473	20
mmu-miR-7042-5p	XM_006541232.3	150.00	-16.28	2 11	5322 5342	9
mmu-miR-7042-5p	XM_006541232.3	147.00	-20.51	2 20	1352 1372	18
mmu-miR-7042-5p	XM_006541232.3	142.00	-16.01	3 19	5567 5587	16
mmu-miR-7042-5p	XM_006541232.3	141.00	-25.54	3 20	2735 2760	22
mmu-miR-7042-5p	XM_006498872.3	144.00	-17.00	2 19	763 785	19
mmu-miR-7042-5p	NM_001290273.1	143.00	-19.77	2 20	1389 1409	18
mmu-miR-7042-5p	XM_006520023.2	154.00	-16.31	2 19	206 226	17
mmu-miR-7042-5p	XM_006520023.2	150.00	-22.20	2 20	2586 2607	19
mmu-miR-7042-5p	XM_006504940.3	143.00	-16.15	2 12	1226 1246	10
mmu-miR-7042-5p	NM_008623.5	148.00	-18.51	2 19	1330 1352	19
mmu-miR-7042-5p	NM_029844.3	153.00	-25.36	2 19	896 920	21
mmu-miR-7042-5p	NM_029844.3	143.00	-14.39	2 12	159 179	10
mmu-miR-7042-5p	NM_007641.5	154.00	-22.14	2 19	1533 1553	17
mmu-miR-7042-5p	NM_001099314.1	152.00	-18.76	2 17	287 307	15
mmu-miR-7042-5p	XM_006537659.2	151.00	-21.79	3 20	5476 5496	17
mmu-miR-7042-5p	XM_006529753.2	152.00	-21.39	2 13	104 124	11
mmu-miR-7042-5p	NM_175260.2	145.00	-14.11	2 19	6710 6727	17
mmu-miR-7042-5p	XM_006532412.1	140.00	-26.22	2 20	471 491	19
mmu-miR-7042-5p	NM_010858.4	140.00	-14.14	2 9	18 38	7
mmu-miR-7042-5p	XM_017320223.1	148.00	-15.03	2 15	549 568	13
mmu-miR-7042-5p	XM_017320223.1	144.00	-26.19	2 13	992 1012	11
mmu-miR-7042-5p	NM_173437.2	162.00	-22.99	2 20	9124 9145	19
mmu-miR-7042-5p	NM_173437.2	161.00	-29.89	2 20	3199 3218	18
mmu-miR-7042-5p	NM_173437.2	160.00	-20.94	2 17	9016 9036	15
mmu-miR-7042-5p	NM_173437.2	140.00	-12.05	2 9	8206 8226	7
mmu-miR-7042-5p	NM_173437.2	140.00	-18.88	2 9	11372 11392	7
mmu-miR-7042-5p	XM_006541297.3	161.00	-22.31	2 19	8965 8987	19
mmu-miR-7042-5p	XM_006541297.3	146.00	-17.39	2 20	5318 5339	19
mmu-miR-7042-5p	XM_006541297.3	145.00	-15.11	2 10	4107 4127	8
mmu-miR-7042-5p	XM_006541297.3	143.00	-14.77	2 16	8426 8446	14
mmu-miR-7042-5p	XM_006529456.3	141.00	-14.17	2 10	298 318	8
mmu-miR-7042-5p	XM_006510077.3	145.00	-15.73	2 10	932 952	8
mmu-miR-7042-5p	XM_006510077.3	141.00	-14.55	2 10	1312 1332	8
mmu-miR-7042-5p	NM_001134300.2	146.00	-18.53	2 15	5291 5311	13
mmu-miR-7042-5p	NM_001109985.1	161.00	-20.92	2 20	812 831	18
mmu-miR-7042-5p	NM_001109985.1	157.00	-20.82	2 14	2360 2380	12
mmu-miR-7042-5p	NM_001029877.3	162.00	-26.09	2 20	4842 4863	19
mmu-miR-7042-5p	NM_001029877.3	140.00	-13.89	2 9	6799 6819	7
mmu-miR-7042-5p	NM_001029877.3	140.00	-17.80	2 17	7719 7739	15

mmu-miR-7042-5p	XM_006526747.3	140.00	-15.09	2 9	1769 1789	7
mmu-miR-7042-5p	NM_001282961.1	156.00	-21.52	2 18	3274 3297	19
mmu-miR-7042-5p	NM_001282961.1	152.00	-21.11	2 18	8440 8464	20
mmu-miR-7042-5p	NM_001282961.1	141.00	-20.40	3 14	5836 5856	11
mmu-miR-7042-5p	NM_001282961.1	140.00	-19.20	2 18	3299 3320	17
mmu-miR-7042-5p	NM_001282961.1	140.00	-18.08	2 9	8621 8641	7
mmu-miR-7042-5p	XM_006530325.3	157.00	-22.53	2 19	2809 2830	18
mmu-miR-7042-5p	NM_145226.2	142.00	-16.82	2 19	2637 2657	17
mmu-miR-7042-5p	XM_006530294.3	141.00	-16.70	3 19	1789 1810	17
mmu-miR-7042-5p	NM_011854.2	149.00	-17.00	2 20	2682 2701	18
mmu-miR-7042-5p	NM_001310636.1	141.00	-23.92	2 18	1732 1752	16
mmu-miR-7042-5p	NM_011010.2	146.00	-19.66	2 15	1814 1834	13
mmu-miR-7042-5p	NM_011010.2	141.00	-16.02	2 20	2035 2054	18
mmu-miR-7042-5p	XM_011239969.2	147.00	-21.87	2 17	1798 1819	16
mmu-miR-7042-5p	XM_011239969.2	141.00	-18.36	2 18	6093 6113	16
mmu-miR-7042-5p	NM_001286743.1	156.00	-20.39	2 20	2399 2417	18
mmu-miR-7042-5p	NM_001286743.1	142.00	-24.11	3 20	1964 1986	19
mmu-miR-7042-5p	NM_001286743.1	140.00	-12.22	2 9	2184 2204	7
mmu-miR-7042-5p	XM_017322026.1	148.00	-21.17	2 20	584 602	18
mmu-miR-7042-5p	XM_017322026.1	142.00	-18.26	2 20	147 171	22
mmu-miR-7219-5p	XM_006527803.1	140.00	-19.27	2 9	1812 1834	7
mmu-miR-7219-5p	NM_172862.3	140.00	-20.43	2 21	1929 1951	19
mmu-miR-7219-5p	NM_010279.3	140.00	-19.55	3 20	3940 3960	17
mmu-miR-7219-5p	XM_006510297.3	156.00	-25.23	2 21	2876 2898	19
mmu-miR-7219-5p	XM_006510297.3	153.00	-16.48	2 14	2580 2602	12
mmu-miR-7219-5p	XM_006530353.3	159.00	-19.97	2 22	4025 4046	20
mmu-miR-7219-5p	XM_006530353.3	153.00	-29.46	2 22	3164 3186	20
mmu-miR-7219-5p	XM_006530353.3	153.00	-24.68	2 20	11761 11782	18
mmu-miR-7219-5p	XM_011248576.2	143.00	-26.13	3 20	315 337	17
mmu-miR-7219-5p	XM_011248576.2	142.00	-19.10	2 22	6920 6946	24
mmu-miR-7219-5p	XM_001481172.6	142.00	-16.49	3 21	90 111	18
mmu-miR-7219-5p	XM_006532232.3	147.00	-14.41	2 22	1083 1104	22
mmu-miR-7219-5p	XM_006521554.3	156.00	-18.66	2 21	335 357	19
mmu-miR-7219-5p	XM_006521554.3	147.00	-21.84	2 21	521 544	20
mmu-miR-7219-5p	XM_006529759.1	141.00	-22.06	2 20	1655 1680	21
mmu-miR-7219-5p	NM_001162950.1	146.00	-20.71	2 21	4704 4725	19
mmu-miR-7219-5p	NM_001162950.1	144.00	-17.31	2 21	1467 1489	19
mmu-miR-7219-5p	XM_006505602.3	145.00	-20.62	3 22	2030 2052	19
mmu-miR-7219-5p	XM_006505602.3	143.00	-21.39	2 18	12908 12929	16
mmu-miR-7219-5p	NM_178184.2	150.00	-21.11	2 22	427 447	20
mmu-miR-7219-5p	NM_001164086.1	153.00	-21.44	2 22	2693 2715	20
mmu-miR-7219-5p	NM_010450.3	147.00	-26.07	3 21	1652 1675	19
mmu-miR-7219-5p	NM_010450.3	144.00	-21.28	2 18	110 133	17
mmu-miR-7219-5p	NM_013868.4	162.00	-29.63	3 22	1988 2010	20
mmu-miR-7219-5p	NM_013868.4	153.00	-19.06	2 16	1788 1809	14
mmu-miR-7219-5p	XM_006511311.2	150.00	-22.69	3 20	3775 3799	19
mmu-miR-7219-5p	XM_006511311.2	143.00	-22.46	2 17	2644 2668	17
mmu-miR-7219-5p	NM_023670.3	158.00	-21.52	2 20	3301 3325	20
mmu-miR-7219-5p	NM_023670.3	156.00	-21.56	2 18	2680 2703	17
mmu-miR-7219-5p	NM_001190325.1	147.00	-21.66	2 22	827 848	20
mmu-miR-7219-5p	NM_009349.3	156.00	-21.19	2 22	858 882	22
mmu-miR-7219-5p	NM_001289875.1	159.00	-20.41	2 21	97 120	20
mmu-miR-7219-5p	NM_021342.1	152.00	-23.38	2 17	1499 1521	15
mmu-miR-7219-5p	XM_011248919.2	151.00	-18.49	2 22	4453 4474	20
mmu-miR-7219-5p	NM_031180.2	142.00	-18.90	3 21	2062 2083	18
mmu-miR-7219-5p	NM_173427.2	143.00	-19.26	2 13	1371 1394	12
mmu-miR-7219-5p	NM_029274.2	149.00	-18.19	2 22	6769 6788	20
mmu-miR-7219-5p	NM_029274.2	140.00	-14.37	2 21	1520 1542	19
mmu-miR-7219-5p	XM_017316676.1	156.00	-21.11	2 21	4223 4245	19
mmu-miR-7219-5p	XM_006523061.3	143.00	-20.79	3 22	3108 3129	19
mmu-miR-7219-5p	NM_010714.3	154.00	-15.93	2 19	1629 1651	17
mmu-miR-7219-5p	NM_010714.3	150.00	-27.50	2 19	2088 2110	17
mmu-miR-7219-5p	NM_010714.3	143.00	-21.20	2 22	3562 3583	20
mmu-miR-7219-5p	NM_175271.4	147.00	-12.79	2 13	2848 2871	12
mmu-miR-7219-5p	XM_006506340.3	153.00	-22.67	2 21	2460 2480	19

mmu-miR-7219-5p	NM_028973.2	140.00	-17.82	2 22	255 279	22
mmu-miR-7219-5p	XM_006506086.2	140.00	-19.80	2 21	67 89	19
mmu-miR-7219-5p	NM_001171187.1	152.00	-16.71	2 21	2427 2449	19
mmu-miR-7219-5p	NM_001171187.1	141.00	-15.95	2 22	87 109	20
mmu-miR-7219-5p	XM_006541232.3	160.00	-21.69	2 22	4393 4416	21
mmu-miR-7219-5p	XM_006541232.3	151.00	-26.12	2 22	14 35	20
mmu-miR-7219-5p	XM_006541232.3	145.00	-18.42	3 22	1034 1056	19
mmu-miR-7219-5p	XM_006541232.3	140.00	-18.64	2 21	219 241	19
mmu-miR-7219-5p	XM_006498872.3	152.00	-19.26	2 21	6729 6751	19
mmu-miR-7219-5p	XM_006498872.3	148.00	-17.39	2 20	1 20	20
mmu-miR-7219-5p	XM_006498872.3	144.00	-15.29	3 21	10386 10408	18
mmu-miR-7219-5p	NM_001290273.1	148.00	-14.29	2 21	881 903	19
mmu-miR-7219-5p	NM_001252563.1	141.00	-23.85	2 21	831 857	24
mmu-miR-7219-5p	XM_006504940.3	143.00	-20.02	2 17	1858 1881	16
mmu-miR-7219-5p	NM_001320077.1	143.00	-23.82	2 22	2951 2977	24
mmu-miR-7219-5p	NM_010809.2	140.00	-24.73	2 18	744 767	17
mmu-miR-7219-5p	XM_006504613.3	143.00	-18.66	2 20	89 111	18
mmu-miR-7219-5p	NM_205810.4	148.00	-13.78	2 22	3288 3311	21
mmu-miR-7219-5p	NM_001099314.1	148.00	-20.27	2 22	585 610	23
mmu-miR-7219-5p	NM_175260.2	141.00	-20.34	2 21	4404 4427	21
mmu-miR-7219-5p	NM_001039545.2	149.00	-21.69	2 21	3008 3028	19
mmu-miR-7219-5p	XM_006532412.1	149.00	-21.69	2 21	2980 3000	19
mmu-miR-7219-5p	XM_006532412.1	145.00	-15.89	2 20	3075 3099	20
mmu-miR-7219-5p	NM_010859.2	150.00	-19.37	2 21	713 734	19
mmu-miR-7219-5p	XM_017320223.1	143.00	-19.76	2 22	3585 3612	25
mmu-miR-7219-5p	XM_006501829.3	144.00	-21.87	2 18	768 792	18
mmu-miR-7219-5p	NM_173437.2	143.00	-23.26	2 21	8117 8140	20
mmu-miR-7219-5p	XM_006541297.3	146.00	-18.42	2 21	4214 4235	19
mmu-miR-7219-5p	XM_006541297.3	140.00	-23.38	3 22	1419 1442	20
mmu-miR-7219-5p	NM_011424.3	151.00	-18.13	2 20	6112 6134	18
mmu-miR-7219-5p	XM_006510077.3	144.00	-16.95	3 21	2341 2363	18
mmu-miR-7219-5p	XM_006510077.3	141.00	-19.69	2 21	2377 2397	19
mmu-miR-7219-5p	NM_001134300.2	141.00	-18.47	2 20	2734 2755	18
mmu-miR-7219-5p	NM_001029877.3	145.00	-18.50	2 18	3412 3434	16
mmu-miR-7219-5p	XM_006530325.3	149.00	-21.76	2 18	1357 1379	16
mmu-miR-7219-5p	NM_145226.2	140.00	-24.09	2 22	634 657	21
mmu-miR-7219-5p	NM_008760.4	142.00	-23.57	2 21	154 179	22
mmu-miR-7219-5p	NM_172907.3	145.00	-22.73	2 20	1216 1237	18
mmu-miR-7219-5p	XM_006516928.2	144.00	-19.69	2 22	316 339	21
mmu-miR-7219-5p	XM_011239969.2	140.00	-21.99	2 21	2018 2040	19
mmu-miR-7221-3p	XM_006524407.3	157.00	-28.69	2 19	1277 1301	21
mmu-miR-7221-3p	NM_177743.5	158.00	-24.50	2 20	1157 1179	20
mmu-miR-7221-3p	XM_017319626.1	146.00	-27.24	2 20	1333 1355	20
mmu-miR-7221-3p	XM_017319626.1	140.00	-17.97	3 18	110 132	17
mmu-miR-7221-3p	XM_006532962.2	146.00	-22.22	2 16	1288 1311	17
mmu-miR-7221-3p	XM_006532962.2	141.00	-21.93	2 19	2921 2942	18
mmu-miR-7221-3p	XM_006520437.2	153.00	-19.89	2 18	2183 2203	16
mmu-miR-7221-3p	NM_001081185.1	159.00	-27.92	2 20	7157 7174	18
mmu-miR-7221-3p	NM_001081185.1	142.00	-18.61	2 11	2783 2803	9
mmu-miR-7221-3p	NM_001081185.1	140.00	-23.91	3 20	4687 4711	21
mmu-miR-7221-3p	NM_172862.3	155.00	-33.88	2 20	3928 3948	18
mmu-miR-7221-3p	NM_172862.3	154.00	-21.42	2 15	9603 9623	13
mmu-miR-7221-3p	NM_172862.3	151.00	-33.24	2 20	4957 4977	18
mmu-miR-7221-3p	NM_172862.3	146.00	-23.48	2 20	313 334	19
mmu-miR-7221-3p	XM_006533539.3	151.00	-20.62	2 17	4497 4518	16
mmu-miR-7221-3p	XM_006533539.3	151.00	-26.21	2 17	7579 7602	18
mmu-miR-7221-3p	XM_006533539.3	148.00	-20.19	2 18	1761 1785	20
mmu-miR-7221-3p	NM_008067.4	165.00	-28.79	2 19	1182 1203	18
mmu-miR-7221-3p	NM_010279.3	145.00	-25.24	2 20	3473 3492	18
mmu-miR-7221-3p	NM_010279.3	140.00	-16.37	2 9	1316 1336	7
mmu-miR-7221-3p	XM_006510297.3	140.00	-22.97	2 17	949 969	15
mmu-miR-7221-3p	XM_006530353.3	154.00	-31.16	2 20	10875 10897	20
mmu-miR-7221-3p	XM_006530353.3	151.00	-28.87	3 20	11184 11204	17
mmu-miR-7221-3p	XM_006530353.3	149.00	-22.68	2 18	9477 9497	16
mmu-miR-7221-3p	XM_006530353.3	143.00	-13.77	2 20	4216 4236	18

mmu-miR-7221-3p	XM_006530353.3	143.00	-30.99	2 20	14686 14706	18
mmu-miR-7221-3p	XM_006530353.3	142.00	-18.69	2 16	6754 6776	16
mmu-miR-7221-3p	NM_001024731.2	158.00	-24.03	2 20	1176 1198	20
mmu-miR-7221-3p	NM_001024731.2	149.00	-20.03	2 18	338 358	16
mmu-miR-7221-3p	XM_011248576.2	157.00	-24.22	2 18	2228 2248	16
mmu-miR-7221-3p	XM_011248576.2	146.00	-31.14	2 20	6770 6791	19
mmu-miR-7221-3p	XM_011248576.2	143.00	-27.76	3 17	525 546	15
mmu-miR-7221-3p	XM_011244235.2	145.00	-18.73	2 14	839 859	12
mmu-miR-7221-3p	XM_006532232.3	155.00	-25.90	2 20	2352 2372	18
mmu-miR-7221-3p	NM_010340.2	141.00	-17.85	2 10	247 267	8
mmu-miR-7221-3p	XM_006515096.2	167.00	-27.26	2 18	7826 7845	16
mmu-miR-7221-3p	XM_006515096.2	150.00	-21.07	2 15	3206 3226	13
mmu-miR-7221-3p	XM_006515096.2	147.00	-24.59	2 20	4171 4191	18
mmu-miR-7221-3p	XM_006526069.2	152.00	-24.91	2 17	2578 2598	15
mmu-miR-7221-3p	XM_006526069.2	146.00	-17.42	2 11	5444 5464	9
mmu-miR-7221-3p	XM_006526069.2	141.00	-23.27	2 15	1595 1617	15
mmu-miR-7221-3p	NM_008185.3	155.00	-24.47	2 20	843 863	18
mmu-miR-7221-3p	NM_001162950.1	163.00	-31.02	2 17	2144 2167	18
mmu-miR-7221-3p	NM_001162950.1	145.00	-20.59	2 20	824 843	18
mmu-miR-7221-3p	NM_001162950.1	140.00	-22.54	2 20	3246 3267	20
mmu-miR-7221-3p	XM_006505602.3	163.00	-31.65	2 17	2926 2947	16
mmu-miR-7221-3p	XM_006505602.3	157.00	-23.90	2 19	2995 3016	18
mmu-miR-7221-3p	NM_001327998.1	168.00	-26.85	2 19	4483 4502	17
mmu-miR-7221-3p	NM_001327998.1	146.00	-20.79	3 19	3745 3765	16
mmu-miR-7221-3p	NM_013868.4	144.00	-22.97	2 18	1682 1704	18
mmu-miR-7221-3p	NM_013868.4	140.00	-18.58	2 9	1338 1358	7
mmu-miR-7221-3p	NM_030704.3	141.00	-26.72	2 19	1225 1246	18
mmu-miR-7221-3p	XM_006496593.3	143.00	-22.41	3 20	417 437	17
mmu-miR-7221-3p	XM_006496593.3	143.00	-26.91	3 20	7455 7475	17
mmu-miR-7221-3p	NM_009349.3	153.00	-22.08	2 20	900 919	18
mmu-miR-7221-3p	XM_006528842.1	148.00	-23.20	2 20	5978 5996	18
mmu-miR-7221-3p	NM_001161541.1	142.00	-22.95	3 19	439 459	16
mmu-miR-7221-3p	NM_001289875.1	151.00	-24.24	2 16	2462 2482	14
mmu-miR-7221-3p	NM_001289875.1	142.00	-20.80	2 16	1932 1955	17
mmu-miR-7221-3p	NM_010608.2	140.00	-26.14	2 18	829 850	17
mmu-miR-7221-3p	XM_006497892.3	157.00	-25.97	2 18	793 813	16
mmu-miR-7221-3p	NM_173427.2	151.00	-24.17	2 13	4647 4668	12
mmu-miR-7221-3p	NM_173427.2	140.00	-12.54	2 9	3004 3024	7
mmu-miR-7221-3p	NM_001081667.2	147.00	-30.79	2 20	114 135	19
mmu-miR-7221-3p	XM_006512296.3	157.00	-26.83	2 19	1994 2015	18
mmu-miR-7221-3p	XM_006512296.3	143.00	-23.93	2 16	1284 1304	14
mmu-miR-7221-3p	XM_006512296.3	142.00	-27.86	3 20	2560 2582	19
mmu-miR-7221-3p	NM_008940.3	148.00	-26.81	2 13	822 842	11
mmu-miR-7221-3p	NM_029274.2	155.00	-21.21	2 12	6977 6997	10
mmu-miR-7221-3p	XM_017316676.1	156.00	-27.52	2 19	14772 14794	19
mmu-miR-7221-3p	XM_017316676.1	151.00	-22.90	2 17	2486 2508	17
mmu-miR-7221-3p	XM_017316676.1	147.00	-26.88	2 20	7590 7610	18
mmu-miR-7221-3p	XM_017316676.1	143.00	-26.50	2 20	12970 12990	18
mmu-miR-7221-3p	XM_017316676.1	141.00	-22.06	3 18	900 920	15
mmu-miR-7221-3p	XM_017316676.1	141.00	-34.46	2 20	7385 7404	18
mmu-miR-7221-3p	NM_146063.1	141.00	-21.63	2 18	2023 2043	16
mmu-miR-7221-3p	XM_017315313.1	157.00	-22.38	2 18	1796 1816	16
mmu-miR-7221-3p	XM_006504999.1	161.00	-26.03	2 20	2977 2999	20
mmu-miR-7221-3p	XM_006519969.3	143.00	-20.24	2 14	1385 1404	12
mmu-miR-7221-3p	XM_006519969.3	140.00	-16.81	2 9	1870 1890	7
mmu-miR-7221-3p	XM_006506340.3	150.00	-28.36	2 20	3793 3819	24
mmu-miR-7221-3p	NM_008524.2	140.00	-23.24	3 13	174 194	10
mmu-miR-7221-3p	XM_006532410.3	174.00	-33.78	2 19	6039 6059	17
mmu-miR-7221-3p	NM_001290273.1	143.00	-25.57	3 20	94 114	17
mmu-miR-7221-3p	XM_006520023.2	145.00	-14.64	2 14	976 996	12
mmu-miR-7221-3p	XM_011243266.1	149.00	-26.59	2 20	2109 2128	18
mmu-miR-7221-3p	XM_006504940.3	143.00	-18.95	3 20	1063 1083	17
mmu-miR-7221-3p	NM_001290512.1	148.00	-22.36	2 15	1681 1700	13
mmu-miR-7221-3p	NM_001290512.1	148.00	-22.36	2 15	1806 1825	13
mmu-miR-7221-3p	NM_001290512.1	148.00	-22.36	2 15	1837 1856	13

mmu-miR-7221-3p	NM_001290512.1	148.00	-19.76	2 15	1899 1918	13
mmu-miR-7221-3p	NM_001290512.1	148.00	-22.36	2 15	1929 1948	13
mmu-miR-7221-3p	NM_001290512.1	148.00	-22.36	2 15	2053 2072	13
mmu-miR-7221-3p	NM_001290512.1	148.00	-22.36	2 15	2084 2103	13
mmu-miR-7221-3p	NM_001290512.1	148.00	-22.36	2 15	2146 2165	13
mmu-miR-7221-3p	NM_001290512.1	144.00	-20.35	2 15	2022 2041	13
mmu-miR-7221-3p	NM_001320077.1	142.00	-14.30	2 18	2952 2970	16
mmu-miR-7221-3p	NM_001320077.1	141.00	-19.61	3 20	2902 2921	17
mmu-miR-7221-3p	NM_008623.5	141.00	-30.19	2 20	1018 1037	18
mmu-miR-7221-3p	NM_029844.3	153.00	-25.59	2 19	220 241	18
mmu-miR-7221-3p	NM_029844.3	140.00	-18.53	2 9	435 455	7
mmu-miR-7221-3p	NM_007641.5	146.00	-21.62	3 20	626 647	18
mmu-miR-7221-3p	XM_006537659.2	143.00	-28.23	3 20	2471 2489	17
mmu-miR-7221-3p	NM_175260.2	148.00	-22.73	2 17	1411 1431	15
mmu-miR-7221-3p	XM_006532412.1	149.00	-20.82	2 18	29 49	16
mmu-miR-7221-3p	NM_010858.4	140.00	-17.45	2 9	569 589	7
mmu-miR-7221-3p	XM_006513859.3	147.00	-18.52	2 18	1706 1725	16
mmu-miR-7221-3p	XM_017320223.1	159.00	-21.80	2 16	2996 3016	14
mmu-miR-7221-3p	XM_017320223.1	140.00	-14.95	2 9	5754 5774	7
mmu-miR-7221-3p	XM_006501829.3	142.00	-27.60	2 19	415 435	17
mmu-miR-7221-3p	XM_017317978.1	153.00	-22.79	2 15	217 238	14
mmu-miR-7221-3p	NM_173437.2	142.00	-22.60	2 19	2022 2042	17
mmu-miR-7221-3p	NM_173437.2	142.00	-23.46	2 20	9742 9763	19
mmu-miR-7221-3p	XM_006541297.3	142.00	-24.15	2 20	8849 8870	19
mmu-miR-7221-3p	NM_011424.3	142.00	-25.96	2 17	5855 5878	18
mmu-miR-7221-3p	NM_001134300.2	152.00	-30.88	2 20	2966 2986	19
mmu-miR-7221-3p	NM_001134300.2	143.00	-22.44	3 20	4781 4801	17
mmu-miR-7221-3p	NM_001109985.1	144.00	-26.70	2 18	2273 2294	17
mmu-miR-7221-3p	NM_001109985.1	141.00	-18.64	3 20	2583 2602	17
mmu-miR-7221-3p	NM_001029877.3	158.00	-26.93	2 18	6078 6096	16
mmu-miR-7221-3p	XM_006526747.3	172.00	-28.47	2 17	3507 3527	15
mmu-miR-7221-3p	XM_006526747.3	165.00	-31.70	2 19	4872 4893	18
mmu-miR-7221-3p	NM_001077403.1	146.00	-27.53	3 20	2671 2692	18
mmu-miR-7221-3p	NM_198190.1	148.00	-20.51	2 14	1268 1289	13
mmu-miR-7221-3p	NM_133500.2	150.00	-31.00	2 15	484 504	13
mmu-miR-7221-3p	NM_145210.2	153.00	-21.64	2 14	742 762	12
mmu-miR-7221-3p	XM_006530294.3	148.00	-23.38	2 14	1703 1724	13
mmu-miR-7221-3p	NM_008760.4	157.00	-24.23	2 20	63 82	18
mmu-miR-7221-3p	NM_008760.4	153.00	-25.23	2 15	174 195	14
mmu-miR-7221-3p	XM_006516928.2	147.00	-27.22	3 20	2620 2640	17
mmu-miR-7221-3p	XM_006516928.2	140.00	-31.66	2 17	938 958	15
mmu-miR-7221-3p	NM_011010.2	146.00	-30.46	3 20	676 697	18
mmu-miR-7226-5p	XM_006509531.3	148.00	-21.65	2 24	1180 1205	23
mmu-miR-7226-5p	XM_006509531.3	148.00	-19.10	2 17	3359 3384	15
mmu-miR-7226-5p	XM_006524407.3	154.00	-24.36	2 22	1265 1288	20
mmu-miR-7226-5p	XM_006524407.3	149.00	-17.79	2 22	1329 1354	20
mmu-miR-7226-5p	XM_006524407.3	146.00	-19.07	2 22	656 679	20
mmu-miR-7226-5p	XM_006524407.3	140.00	-19.63	2 22	758 784	21
mmu-miR-7226-5p	XM_017319626.1	142.00	-19.37	2 19	3457 3482	17
mmu-miR-7226-5p	XM_017319626.1	140.00	-17.49	3 18	532 558	16
mmu-miR-7226-5p	XM_006532962.2	143.00	-19.60	3 25	1808 1835	24
mmu-miR-7226-5p	NM_178908.3	151.00	-26.66	2 25	1102 1129	25
mmu-miR-7226-5p	NM_178908.3	140.00	-19.04	2 24	1003 1031	26
mmu-miR-7226-5p	NM_001160262.1	151.00	-20.06	2 20	4623 4648	18
mmu-miR-7226-5p	XM_006520437.2	148.00	-26.01	2 25	2019 2040	24
mmu-miR-7226-5p	XM_006520437.2	147.00	-21.56	2 25	526 554	26
mmu-miR-7226-5p	XM_006520437.2	140.00	-24.43	3 25	2125 2148	22
mmu-miR-7226-5p	NM_010181.2	158.00	-21.82	2 25	4243 4267	23
mmu-miR-7226-5p	NM_010181.2	158.00	-23.18	2 23	10145 10168	21
mmu-miR-7226-5p	NM_010181.2	146.00	-21.89	2 25	410 439	27
mmu-miR-7226-5p	NM_010181.2	146.00	-18.22	2 15	1464 1489	13
mmu-miR-7226-5p	NM_010181.2	146.00	-26.55	2 21	3127 3154	21
mmu-miR-7226-5p	NM_010181.2	145.00	-21.76	2 25	4907 4932	24
mmu-miR-7226-5p	NM_010181.2	143.00	-21.45	2 16	3987 4012	14
mmu-miR-7226-5p	NM_010181.2	141.00	-21.47	2 23	3317 3346	26

mmu-miR-7226-5p	XM_011244488.2	144.00	-21.68	2 17	463 488	15
mmu-miR-7226-5p	XM_011244488.2	140.00	-22.54	2 25	619 644	23
mmu-miR-7226-5p	XM_006527803.1	142.00	-21.19	3 25	1492 1519	24
mmu-miR-7226-5p	XM_006527803.1	142.00	-28.47	2 15	1717 1742	13
mmu-miR-7226-5p	NM_001081185.1	158.00	-20.80	2 24	3709 3735	23
mmu-miR-7226-5p	NM_001081185.1	158.00	-21.06	2 24	7750 7776	23
mmu-miR-7226-5p	NM_001081185.1	153.00	-22.22	2 21	5825 5850	20
mmu-miR-7226-5p	NM_001081185.1	145.00	-25.11	2 22	2516 2542	21
mmu-miR-7226-5p	NM_001081185.1	145.00	-16.14	2 19	8433 8460	19
mmu-miR-7226-5p	NM_001081185.1	143.00	-21.90	2 16	4055 4080	14
mmu-miR-7226-5p	NM_001081185.1	141.00	-25.05	3 25	1681 1707	24
mmu-miR-7226-5p	NM_172862.3	165.00	-25.13	2 24	6789 6817	25
mmu-miR-7226-5p	NM_172862.3	161.00	-25.70	2 25	2962 2988	25
mmu-miR-7226-5p	NM_172862.3	149.00	-21.82	2 24	403 426	23
mmu-miR-7226-5p	NM_172862.3	142.00	-25.44	3 23	3738 3763	20
mmu-miR-7226-5p	NM_172862.3	140.00	-18.12	3 19	4483 4507	16
mmu-miR-7226-5p	XM_006533539.3	148.00	-20.33	2 23	3258 3282	21
mmu-miR-7226-5p	XM_006533539.3	144.00	-21.02	2 23	393 417	21
mmu-miR-7226-5p	XM_006533539.3	143.00	-22.94	2 25	7103 7124	23
mmu-miR-7226-5p	XM_006533539.3	142.00	-23.65	2 25	6168 6200	30
mmu-miR-7226-5p	NM_001033221.3	150.00	-24.56	2 25	243 271	26
mmu-miR-7226-5p	NM_008067.4	166.00	-24.89	2 25	322 350	26
mmu-miR-7226-5p	NM_008067.4	162.00	-27.15	3 25	1646 1670	22
mmu-miR-7226-5p	NM_001033445.2	141.00	-22.07	2 25	3360 3385	24
mmu-miR-7226-5p	XM_006514444.1	160.00	-23.66	2 25	2332 2358	25
mmu-miR-7226-5p	XM_006514444.1	150.00	-19.63	2 20	1817 1843	19
mmu-miR-7226-5p	XM_006514444.1	149.00	-25.41	3 25	916 939	22
mmu-miR-7226-5p	XM_006514444.1	143.00	-25.24	2 21	891 917	20
mmu-miR-7226-5p	NM_145741.2	147.00	-20.50	2 20	1589 1614	18
mmu-miR-7226-5p	NM_010279.3	154.00	-20.56	2 21	2393 2417	19
mmu-miR-7226-5p	NM_010279.3	147.00	-31.28	2 22	245 275	25
mmu-miR-7226-5p	NM_010279.3	146.00	-21.77	2 11	4292 4317	9
mmu-miR-7226-5p	XM_006510297.3	150.00	-27.39	2 25	3213 3241	26
mmu-miR-7226-5p	XM_006504899.3	146.00	-20.97	2 25	516 540	23
mmu-miR-7226-5p	XM_006530353.3	173.00	-29.90	2 24	11413 11437	22
mmu-miR-7226-5p	XM_006530353.3	160.00	-22.54	2 22	9714 9740	21
mmu-miR-7226-5p	XM_006530353.3	158.00	-22.04	2 23	13872 13897	21
mmu-miR-7226-5p	XM_006530353.3	146.00	-18.24	2 24	14187 14213	23
mmu-miR-7226-5p	XM_006530353.3	144.00	-24.14	2 25	7848 7873	23
mmu-miR-7226-5p	XM_006530353.3	142.00	-23.90	3 24	9360 9389	25
mmu-miR-7226-5p	XM_006530353.3	142.00	-30.93	2 25	10249 10273	23
mmu-miR-7226-5p	XM_011248576.2	155.00	-21.20	2 22	3553 3577	20
mmu-miR-7226-5p	XM_011248576.2	153.00	-21.01	2 25	12942 12970	26
mmu-miR-7226-5p	XM_011248576.2	152.00	-27.24	2 25	12290 12318	27
mmu-miR-7226-5p	XM_011248576.2	149.00	-27.89	2 21	11156 11179	19
mmu-miR-7226-5p	XM_011248576.2	148.00	-22.77	2 23	6217 6246	25
mmu-miR-7226-5p	XM_011248576.2	145.00	-19.85	3 22	5010 5035	19
mmu-miR-7226-5p	XM_011248576.2	140.00	-20.71	2 23	4804 4832	24
mmu-miR-7226-5p	XM_011244235.2	140.00	-20.48	2 23	901 928	23
mmu-miR-7226-5p	NM_001198955.1	163.00	-24.01	2 23	2878 2903	22
mmu-miR-7226-5p	NM_001198955.1	144.00	-21.42	2 25	2663 2688	23
mmu-miR-7226-5p	XM_006532232.3	150.00	-34.34	2 25	767 798	29
mmu-miR-7226-5p	XM_006532232.3	145.00	-15.78	2 10	1 16	8
mmu-miR-7226-5p	XM_017316736.1	152.00	-18.55	2 21	181 206	19
mmu-miR-7226-5p	NM_010340.2	153.00	-25.69	3 23	1665 1691	21
mmu-miR-7226-5p	XM_006521554.3	144.00	-19.33	2 25	307 332	23
mmu-miR-7226-5p	XM_006515096.2	146.00	-23.69	2 15	1299 1324	13
mmu-miR-7226-5p	XM_006515096.2	146.00	-23.87	2 21	6016 6043	21
mmu-miR-7226-5p	XM_006515096.2	145.00	-19.76	2 23	2166 2194	24
mmu-miR-7226-5p	XM_006515096.2	145.00	-20.85	2 23	4047 4069	21
mmu-miR-7226-5p	XM_006515096.2	144.00	-23.49	2 25	1326 1352	25
mmu-miR-7226-5p	XM_006515096.2	144.00	-28.86	2 25	6834 6860	24
mmu-miR-7226-5p	XM_006515096.2	142.00	-23.00	2 19	5603 5628	17
mmu-miR-7226-5p	XM_006526069.2	147.00	-21.69	3 20	5651 5676	17
mmu-miR-7226-5p	XM_006526069.2	145.00	-30.67	2 25	2361 2389	27

mmu-miR-7226-5p	NM_008185.3	144.00	-24.66	2 14	559 585	13
mmu-miR-7226-5p	NM_008185.3	141.00	-15.79	3 22	429 454	19
mmu-miR-7226-5p	XM_011246309.2	147.00	-24.95	2 25	1642 1668	24
mmu-miR-7226-5p	XM_011246309.2	143.00	-16.65	2 21	2555 2581	20
mmu-miR-7226-5p	NM_175520.4	157.00	-19.67	2 25	1904 1927	23
mmu-miR-7226-5p	NM_175520.4	143.00	-21.27	2 24	2685 2710	22
mmu-miR-7226-5p	XM_006529759.1	147.00	-21.99	2 25	1539 1567	26
mmu-miR-7226-5p	XM_006529759.1	140.00	-24.37	3 22	1772 1798	20
mmu-miR-7226-5p	NM_001162950.1	164.00	-30.82	2 22	2997 3027	25
mmu-miR-7226-5p	NM_001162950.1	159.00	-24.30	2 25	2825 2852	25
mmu-miR-7226-5p	NM_001162950.1	159.00	-25.33	2 23	5709 5734	22
mmu-miR-7226-5p	NM_001162950.1	158.00	-28.05	2 25	1150 1174	23
mmu-miR-7226-5p	NM_001162950.1	157.00	-25.02	2 24	3314 3341	24
mmu-miR-7226-5p	NM_001162950.1	149.00	-24.33	2 25	3681 3702	23
mmu-miR-7226-5p	NM_001162950.1	149.00	-23.34	2 23	3908 3937	25
mmu-miR-7226-5p	NM_001162950.1	148.00	-25.08	2 22	1222 1248	21
mmu-miR-7226-5p	NM_001162950.1	147.00	-15.99	2 25	5546 5572	24
mmu-miR-7226-5p	NM_001162950.1	144.00	-18.60	3 23	1707 1731	20
mmu-miR-7226-5p	NM_001162950.1	144.00	-18.83	2 25	2764 2789	23
mmu-miR-7226-5p	XM_006505602.3	157.00	-24.04	2 23	5120 5146	22
mmu-miR-7226-5p	XM_006505602.3	156.00	-20.80	2 24	12210 12233	22
mmu-miR-7226-5p	XM_006505602.3	155.00	-22.48	2 25	7815 7841	24
mmu-miR-7226-5p	XM_006505602.3	154.00	-19.72	2 19	8880 8905	17
mmu-miR-7226-5p	XM_006505602.3	153.00	-30.63	2 22	8268 8291	20
mmu-miR-7226-5p	XM_006505602.3	149.00	-23.08	2 22	533 558	20
mmu-miR-7226-5p	XM_006505602.3	148.00	-22.81	3 22	12120 12147	21
mmu-miR-7226-5p	XM_006505602.3	147.00	-19.17	3 21	11129 11156	20
mmu-miR-7226-5p	XM_006505602.3	143.00	-17.36	2 23	1459 1484	22
mmu-miR-7226-5p	XM_006505602.3	143.00	-18.16	2 25	10096 10122	24
mmu-miR-7226-5p	XM_006505602.3	140.00	-18.24	3 22	9111 9137	20
mmu-miR-7226-5p	NM_001164086.1	150.00	-25.29	2 22	9000 9023	20
mmu-miR-7226-5p	NM_001164086.1	149.00	-21.00	2 25	6457 6485	26
mmu-miR-7226-5p	NM_008277.2	152.00	-21.87	2 25	221 245	24
mmu-miR-7226-5p	NM_001327998.1	148.00	-23.06	2 23	3006 3034	24
mmu-miR-7226-5p	NM_001327998.1	142.00	-18.81	2 19	1292 1317	17
mmu-miR-7226-5p	NM_013558.2	147.00	-22.46	2 21	1892 1920	22
mmu-miR-7226-5p	NM_013558.2	140.00	-16.98	2 25	67 92	23
mmu-miR-7226-5p	NM_013560.2	143.00	-22.85	2 25	425 451	24
mmu-miR-7226-5p	NM_013868.4	155.00	-26.89	2 22	443 473	25
mmu-miR-7226-5p	NM_013868.4	143.00	-23.96	3 25	289 317	25
mmu-miR-7226-5p	NM_013868.4	140.00	-29.15	2 25	169 195	25
mmu-miR-7226-5p	NM_030704.3	153.00	-23.52	2 21	1094 1119	20
mmu-miR-7226-5p	NM_030704.3	151.00	-26.53	2 22	1549 1573	20
mmu-miR-7226-5p	NM_030704.3	150.00	-23.67	2 11	639 664	9
mmu-miR-7226-5p	NM_030704.3	148.00	-23.42	3 25	1327 1352	22
mmu-miR-7226-5p	NM_030704.3	144.00	-26.10	3 25	797 826	27
mmu-miR-7226-5p	XM_006511311.2	159.00	-25.72	2 23	2172 2195	21
mmu-miR-7226-5p	XM_006511311.2	157.00	-23.53	2 23	2436 2458	21
mmu-miR-7226-5p	XM_006511311.2	148.00	-18.44	2 21	1977 2002	19
mmu-miR-7226-5p	XM_006511311.2	148.00	-25.25	2 25	2020 2045	23
mmu-miR-7226-5p	XM_006511311.2	144.00	-21.40	2 21	2916 2941	19
mmu-miR-7226-5p	XM_006511311.2	143.00	-19.67	2 20	5626 5651	18
mmu-miR-7226-5p	NM_023670.3	172.00	-26.95	2 25	1559 1584	25
mmu-miR-7226-5p	XM_006523711.3	160.00	-28.54	2 25	1094 1119	23
mmu-miR-7226-5p	XM_006523711.3	149.00	-24.23	2 25	1180 1205	24
mmu-miR-7226-5p	XM_017316439.1	145.00	-19.76	2 22	2602 2627	20
mmu-miR-7226-5p	XM_017316439.1	144.00	-19.76	2 25	1065 1090	23
mmu-miR-7226-5p	XM_017316439.1	141.00	-19.00	3 25	34 62	25
mmu-miR-7226-5p	XM_006496593.3	155.00	-22.71	2 25	4228 4254	24
mmu-miR-7226-5p	XM_006496593.3	145.00	-25.72	3 25	1307 1332	23
mmu-miR-7226-5p	XM_006496593.3	144.00	-20.72	2 23	6208 6232	21
mmu-miR-7226-5p	XM_006496593.3	143.00	-16.42	2 24	2243 2268	22
mmu-miR-7226-5p	XM_006496593.3	140.00	-22.67	2 24	3941 3967	24
mmu-miR-7226-5p	XM_006516224.3	160.00	-24.17	2 25	3772 3795	23
mmu-miR-7226-5p	XM_006516224.3	152.00	-24.58	2 25	4046 4071	23

mmu-miR-7226-5p	XM_006516224.3	146.00	-15.74	2 21	3465 3489	19
mmu-miR-7226-5p	XM_006516224.3	145.00	-25.50	2 22	1736 1759	20
mmu-miR-7226-5p	NM_009349.3	169.00	-28.21	2 22	807 832	20
mmu-miR-7226-5p	NM_009349.3	141.00	-18.89	2 20	625 649	18
mmu-miR-7226-5p	XM_006528842.1	156.00	-24.66	2 25	1776 1801	23
mmu-miR-7226-5p	XM_006528842.1	154.00	-21.93	2 25	255 282	25
mmu-miR-7226-5p	XM_006528842.1	152.00	-22.33	2 25	3978 4003	23
mmu-miR-7226-5p	XM_006528842.1	148.00	-24.93	2 25	6050 6074	24
mmu-miR-7226-5p	XM_006528842.1	144.00	-20.68	3 24	3797 3820	21
mmu-miR-7226-5p	XM_006528842.1	144.00	-23.34	2 25	5142 5167	23
mmu-miR-7226-5p	XM_006528842.1	140.00	-27.97	2 25	3444 3469	23
mmu-miR-7226-5p	NM_001161541.1	141.00	-27.22	2 22	525 550	20
mmu-miR-7226-5p	NM_001302471.1	163.00	-23.65	2 25	571 595	24
mmu-miR-7226-5p	NM_001302471.1	153.00	-24.76	2 25	687 710	23
mmu-miR-7226-5p	NM_001302471.1	148.00	-26.17	2 25	1276 1301	23
mmu-miR-7226-5p	NM_001302471.1	146.00	-23.76	2 25	4271 4298	25
mmu-miR-7226-5p	NM_001302471.1	146.00	-27.43	2 25	4667 4691	23
mmu-miR-7226-5p	NM_001302471.1	142.00	-25.65	2 25	3050 3078	26
mmu-miR-7226-5p	NM_001302471.1	140.00	-16.00	2 25	944 969	23
mmu-miR-7226-5p	XM_006518921.3	146.00	-21.33	3 25	119 143	22
mmu-miR-7226-5p	NM_001289875.1	150.00	-26.51	2 25	1355 1386	29
mmu-miR-7226-5p	NM_001289875.1	146.00	-23.90	2 23	1856 1881	21
mmu-miR-7226-5p	NM_001289875.1	142.00	-20.04	3 25	2383 2407	22
mmu-miR-7226-5p	NM_146125.2	152.00	-25.56	2 25	1730 1757	26
mmu-miR-7226-5p	NM_008423.2	155.00	-24.76	2 21	2661 2690	23
mmu-miR-7226-5p	NM_008423.2	150.00	-31.71	3 25	566 590	22
mmu-miR-7226-5p	NM_008423.2	145.00	-22.30	2 21	2262 2285	19
mmu-miR-7226-5p	NM_008423.2	145.00	-21.99	3 23	3119 3146	22
mmu-miR-7226-5p	NM_008423.2	141.00	-22.78	3 20	2015 2039	17
mmu-miR-7226-5p	NM_021342.1	162.00	-23.05	2 25	2007 2034	25
mmu-miR-7226-5p	NM_021342.1	153.00	-21.31	2 24	1482 1506	22
mmu-miR-7226-5p	XM_017315734.1	163.00	-25.41	2 25	263 289	24
mmu-miR-7226-5p	XM_017315734.1	145.00	-22.73	2 25	3353 3381	27
mmu-miR-7226-5p	XM_017315734.1	140.00	-22.26	2 25	1803 1831	27
mmu-miR-7226-5p	NM_010608.2	152.00	-27.30	2 23	2870 2894	21
mmu-miR-7226-5p	NM_010608.2	145.00	-24.59	2 22	2284 2309	20
mmu-miR-7226-5p	NM_010608.2	144.00	-22.07	2 24	2745 2771	24
mmu-miR-7226-5p	XM_006497892.3	143.00	-19.52	3 24	5500 5525	21
mmu-miR-7226-5p	XM_011248919.2	159.00	-24.14	2 22	4606 4630	20
mmu-miR-7226-5p	XM_011248919.2	155.00	-27.83	2 22	3661 3691	25
mmu-miR-7226-5p	XM_017316478.1	155.00	-22.52	2 25	4222 4248	24
mmu-miR-7226-5p	NM_031180.2	152.00	-20.98	2 25	238 263	23
mmu-miR-7226-5p	NM_173427.2	157.00	-27.39	2 24	361 388	24
mmu-miR-7226-5p	NM_173427.2	146.00	-21.07	2 25	2954 2981	25
mmu-miR-7226-5p	NM_173427.2	145.00	-22.13	2 25	4067 4092	24
mmu-miR-7226-5p	NM_173427.2	145.00	-21.71	2 10	5137 5162	8
mmu-miR-7226-5p	NM_173427.2	143.00	-24.69	2 22	2569 2593	20
mmu-miR-7226-5p	NM_173427.2	140.00	-26.68	2 22	2231 2257	21
mmu-miR-7226-5p	NM_173427.2	140.00	-19.44	2 9	5528 5553	7
mmu-miR-7226-5p	NM_001081667.2	165.00	-25.79	2 22	1129 1154	20
mmu-miR-7226-5p	XM_006512296.3	142.00	-24.10	2 19	1132 1155	17
mmu-miR-7226-5p	NM_029274.2	181.00	-30.01	2 25	5224 5249	24
mmu-miR-7226-5p	NM_029274.2	162.00	-33.38	2 25	7562 7586	23
mmu-miR-7226-5p	NM_029274.2	160.00	-28.12	2 25	7420 7446	25
mmu-miR-7226-5p	NM_029274.2	154.00	-22.43	2 21	5459 5487	22
mmu-miR-7226-5p	NM_029274.2	154.00	-28.68	3 23	6089 6114	20
mmu-miR-7226-5p	NM_029274.2	151.00	-23.70	3 25	5655 5681	23
mmu-miR-7226-5p	NM_029274.2	151.00	-20.82	2 22	6477 6501	20
mmu-miR-7226-5p	NM_029274.2	150.00	-24.25	2 11	8307 8332	9
mmu-miR-7226-5p	NM_029274.2	142.00	-20.37	2 19	6836 6861	17
mmu-miR-7226-5p	NM_029274.2	140.00	-21.99	2 24	7736 7761	23
mmu-miR-7226-5p	XM_017316676.1	162.00	-25.13	3 25	606 630	22
mmu-miR-7226-5p	XM_017316676.1	155.00	-24.27	2 22	11145 11169	20
mmu-miR-7226-5p	XM_017316676.1	147.00	-23.26	2 25	14708 14730	23
mmu-miR-7226-5p	XM_017316676.1	146.00	-22.34	3 19	17269 17294	16

mmu-miR-7226-5p	XM_017316676.1	144.00	-22.07	2 18	3771 3798	18
mmu-miR-7226-5p	XM_017316676.1	143.00	-28.66	2 20	3485 3510	18
mmu-miR-7226-5p	XM_017316676.1	143.00	-25.91	2 20	6503 6528	18
mmu-miR-7226-5p	XM_017316676.1	143.00	-21.75	2 22	12845 12869	20
mmu-miR-7226-5p	XM_017316676.1	142.00	-22.35	3 21	3923 3947	18
mmu-miR-7226-5p	XM_017316676.1	140.00	-28.96	3 25	14660 14682	22
mmu-miR-7226-5p	NM_146063.1	147.00	-27.24	3 22	2029 2053	19
mmu-miR-7226-5p	NM_146063.1	143.00	-22.38	2 23	807 832	22
mmu-miR-7226-5p	NM_008479.2	142.00	-21.12	3 22	1968 1993	20
mmu-miR-7226-5p	NM_008479.2	141.00	-22.02	3 22	462 487	19
mmu-miR-7226-5p	XM_017313062.1	159.00	-27.64	2 25	1395 1422	25
mmu-miR-7226-5p	XM_017313062.1	145.00	-20.97	2 21	2444 2469	20
mmu-miR-7226-5p	XM_017313062.1	142.00	-16.97	3 23	944 969	20
mmu-miR-7226-5p	XM_006523061.3	144.00	-19.84	2 21	1729 1754	19
mmu-miR-7226-5p	XM_006511102.2	144.00	-20.65	3 23	604 632	23
mmu-miR-7226-5p	XM_006511102.2	142.00	-22.17	2 21	1289 1313	19
mmu-miR-7226-5p	XM_006511102.2	140.00	-19.08	2 17	302 327	15
mmu-miR-7226-5p	NM_001302765.1	141.00	-22.44	3 23	1064 1090	21
mmu-miR-7226-5p	XM_006504999.1	149.00	-21.14	2 22	1640 1665	20
mmu-miR-7226-5p	NM_144945.3	156.00	-20.71	2 19	802 830	20
mmu-miR-7226-5p	NM_144945.3	150.00	-26.71	2 24	5465 5492	24
mmu-miR-7226-5p	NM_144945.3	142.00	-26.73	3 25	368 392	22
mmu-miR-7226-5p	NM_144945.3	141.00	-16.54	2 19	4711 4738	19
mmu-miR-7226-5p	NM_144799.2	140.00	-18.87	2 21	201 226	19
mmu-miR-7226-5p	NM_053098.2	160.00	-25.03	2 25	1303 1328	23
mmu-miR-7226-5p	NM_175271.4	146.00	-26.18	2 25	513 537	23
mmu-miR-7226-5p	XM_006506340.3	143.00	-22.90	3 23	2043 2069	22
mmu-miR-7226-5p	XM_006506340.3	141.00	-25.11	2 25	565 590	24
mmu-miR-7226-5p	XM_006506340.3	141.00	-20.03	2 25	3279 3302	23
mmu-miR-7226-5p	NM_028973.2	179.00	-29.95	2 25	1483 1509	24
mmu-miR-7226-5p	NM_028973.2	150.00	-30.86	2 20	1209 1236	20
mmu-miR-7226-5p	NM_028973.2	146.00	-19.53	3 23	995 1020	20
mmu-miR-7226-5p	NM_028973.2	141.00	-19.47	3 23	907 935	23
mmu-miR-7226-5p	NM_028973.2	140.00	-23.49	3 25	2974 2999	22
mmu-miR-7226-5p	XM_006527321.2	154.00	-23.38	2 25	617 645	26
mmu-miR-7226-5p	XM_006527321.2	143.00	-17.48	2 19	1756 1779	17
mmu-miR-7226-5p	XM_006527321.2	142.00	-18.56	3 23	1722 1746	21
mmu-miR-7226-5p	XM_006527321.2	142.00	-24.22	3 25	2436 2465	26
mmu-miR-7226-5p	XM_006527321.2	141.00	-25.05	2 25	535 558	23
mmu-miR-7226-5p	XM_006527321.2	141.00	-15.78	2 20	872 896	18
mmu-miR-7226-5p	XM_006527321.2	140.00	-20.35	2 17	1316 1341	15
mmu-miR-7226-5p	XM_006506086.2	151.00	-23.51	2 25	2504 2530	24
mmu-miR-7226-5p	XM_006506086.2	149.00	-26.51	2 25	96 119	23
mmu-miR-7226-5p	XM_006506086.2	149.00	-28.02	2 25	1921 1944	23
mmu-miR-7226-5p	XM_006506086.2	141.00	-25.79	3 23	551 579	23
mmu-miR-7226-5p	NM_001171187.1	142.00	-24.82	3 25	614 634	22
mmu-miR-7226-5p	NM_001171187.1	141.00	-20.53	2 21	1803 1828	20
mmu-miR-7226-5p	XM_006541232.3	160.00	-31.34	2 22	2713 2739	21
mmu-miR-7226-5p	XM_006541232.3	152.00	-19.77	2 22	2218 2245	22
mmu-miR-7226-5p	XM_006541232.3	147.00	-19.78	3 25	5525 5551	23
mmu-miR-7226-5p	XM_006498872.3	164.00	-27.87	2 25	3425 3450	23
mmu-miR-7226-5p	XM_006498872.3	162.00	-26.92	2 25	859 883	23
mmu-miR-7226-5p	XM_006498872.3	155.00	-27.58	2 25	4048 4075	25
mmu-miR-7226-5p	XM_006498872.3	154.00	-27.29	2 25	5886 5915	27
mmu-miR-7226-5p	XM_006498872.3	153.00	-27.82	2 25	2942 2971	27
mmu-miR-7226-5p	XM_006498872.3	145.00	-26.13	2 22	10230 10255	20
mmu-miR-7226-5p	XM_006498872.3	143.00	-22.76	2 25	7238 7264	24
mmu-miR-7226-5p	XM_006498872.3	142.00	-22.66	2 25	9222 9245	24
mmu-miR-7226-5p	XM_006498872.3	140.00	-26.54	2 25	2169 2194	23
mmu-miR-7226-5p	XM_006498872.3	140.00	-22.11	2 23	4314 4341	23
mmu-miR-7226-5p	XM_006532410.3	162.00	-18.78	2 23	2396 2421	21
mmu-miR-7226-5p	XM_006532410.3	160.00	-25.77	2 25	3884 3909	23
mmu-miR-7226-5p	XM_006532410.3	150.00	-19.95	2 25	2923 2947	23
mmu-miR-7226-5p	XM_006532410.3	148.00	-17.70	2 21	5015 5040	19
mmu-miR-7226-5p	XM_006532410.3	143.00	-24.52	2 16	2611 2636	14

mmu-miR-7226-5p	XM_006532410.3	141.00	-26.06	2 25	6254 6284	28
mmu-miR-7226-5p	NM_001290273.1	143.00	-19.49	3 25	1373 1400	24
mmu-miR-7226-5p	NM_001290273.1	141.00	-20.45	2 15	1321 1347	14
mmu-miR-7226-5p	XM_006520023.2	148.00	-26.05	2 23	2307 2335	24
mmu-miR-7226-5p	XM_011243266.1	153.00	-24.67	2 22	567 592	20
mmu-miR-7226-5p	XM_011243266.1	148.00	-23.52	2 14	2759 2786	14
mmu-miR-7226-5p	XM_011243266.1	142.00	-29.41	2 25	2690 2714	23
mmu-miR-7226-5p	XM_006504940.3	154.00	-22.84	2 22	1483 1508	21
mmu-miR-7226-5p	NM_010825.3	149.00	-26.21	3 25	3746 3771	23
mmu-miR-7226-5p	NM_001270475.1	150.00	-25.69	2 25	3191 3218	25
mmu-miR-7226-5p	NM_001270475.1	150.00	-20.46	2 24	6383 6411	25
mmu-miR-7226-5p	NM_001270475.1	147.00	-25.51	2 25	7023 7050	25
mmu-miR-7226-5p	NM_001270475.1	147.00	-20.14	2 21	8864 8890	20
mmu-miR-7226-5p	NM_001270475.1	145.00	-25.84	2 25	5514 5539	24
mmu-miR-7226-5p	NM_001270475.1	143.00	-26.68	2 22	1368 1392	20
mmu-miR-7226-5p	NM_001270475.1	142.00	-22.35	2 25	7061 7088	25
mmu-miR-7226-5p	NM_001270475.1	142.00	-22.64	2 23	8136 8159	21
mmu-miR-7226-5p	NM_001290512.1	147.00	-23.22	2 25	74 100	24
mmu-miR-7226-5p	NM_001290512.1	143.00	-25.72	2 25	750 777	25
mmu-miR-7226-5p	NM_177595.4	144.00	-22.59	2 22	314 341	22
mmu-miR-7226-5p	NM_001320077.1	161.00	-23.83	2 25	992 1020	26
mmu-miR-7226-5p	NM_001320077.1	150.00	-17.51	2 24	552 579	24
mmu-miR-7226-5p	NM_010809.2	153.00	-21.09	2 23	729 756	23
mmu-miR-7226-5p	NM_010809.2	151.00	-24.20	3 24	1206 1231	21
mmu-miR-7226-5p	NM_010809.2	146.00	-23.90	2 22	905 930	21
mmu-miR-7226-5p	XM_006504613.3	146.00	-23.91	2 25	872 899	25
mmu-miR-7226-5p	NM_029844.3	144.00	-18.71	2 23	157 181	21
mmu-miR-7226-5p	NM_205810.4	140.00	-19.66	2 9	619 644	7
mmu-miR-7226-5p	NM_007641.5	143.00	-21.97	2 24	1220 1245	22
mmu-miR-7226-5p	NM_007641.5	140.00	-19.85	2 25	186 211	23
mmu-miR-7226-5p	NM_001099314.1	161.00	-23.71	2 21	515 540	20
mmu-miR-7226-5p	NM_001099314.1	157.00	-22.82	3 22	468 493	19
mmu-miR-7226-5p	NM_001099314.1	150.00	-23.07	3 25	341 365	22
mmu-miR-7226-5p	XM_006537659.2	159.00	-23.26	3 25	1141 1167	23
mmu-miR-7226-5p	XM_006537659.2	148.00	-25.28	2 22	5043 5069	21
mmu-miR-7226-5p	XM_006537659.2	142.00	-15.66	2 24	7152 7178	23
mmu-miR-7226-5p	NM_175260.2	152.00	-22.42	2 21	4740 4765	19
mmu-miR-7226-5p	NM_175260.2	143.00	-18.79	2 20	39 64	18
mmu-miR-7226-5p	NM_175260.2	142.00	-23.71	2 25	1138 1169	29
mmu-miR-7226-5p	NM_175260.2	140.00	-16.01	2 19	911 935	17
mmu-miR-7226-5p	NM_001039545.2	145.00	-23.84	2 22	4365 4390	20
mmu-miR-7226-5p	NM_001039545.2	142.00	-21.37	2 25	4550 4579	27
mmu-miR-7226-5p	XM_006532412.1	158.00	-22.83	2 21	4410 4434	19
mmu-miR-7226-5p	XM_006532412.1	147.00	-20.77	2 17	1830 1857	17
mmu-miR-7226-5p	XM_006532412.1	145.00	-18.70	2 22	4337 4362	20
mmu-miR-7226-5p	XM_017315841.1	152.00	-23.58	2 25	4946 4968	23
mmu-miR-7226-5p	XM_017315841.1	144.00	-18.56	2 21	2387 2412	19
mmu-miR-7226-5p	XM_017315841.1	144.00	-16.92	2 13	4793 4818	11
mmu-miR-7226-5p	XM_017315841.1	140.00	-15.39	2 9	3254 3279	7
mmu-miR-7226-5p	NM_010859.2	148.00	-26.34	2 25	790 815	23
mmu-miR-7226-5p	NM_010858.4	166.00	-28.12	2 25	820 848	26
mmu-miR-7226-5p	XM_006513859.3	141.00	-24.38	2 25	16 45	27
mmu-miR-7226-5p	XM_006513859.3	141.00	-21.49	2 19	2164 2190	18
mmu-miR-7226-5p	XM_017317978.1	140.00	-18.26	2 9	853 878	7
mmu-miR-7226-5p	NM_173437.2	166.00	-28.60	2 25	4165 4192	25
mmu-miR-7226-5p	NM_173437.2	156.00	-25.76	2 22	6670 6696	21
mmu-miR-7226-5p	NM_173437.2	156.00	-22.95	2 22	12644 12671	22
mmu-miR-7226-5p	NM_173437.2	153.00	-21.01	2 23	891 913	21
mmu-miR-7226-5p	NM_173437.2	152.00	-28.83	2 21	5411 5433	19
mmu-miR-7226-5p	NM_173437.2	151.00	-25.88	2 25	4131 4157	24
mmu-miR-7226-5p	NM_173437.2	150.00	-22.20	2 23	1418 1441	21
mmu-miR-7226-5p	NM_173437.2	142.00	-20.70	2 25	1156 1180	23
mmu-miR-7226-5p	NM_173437.2	142.00	-27.12	2 17	3112 3139	17
mmu-miR-7226-5p	NM_173437.2	142.00	-27.20	2 25	12076 12103	25
mmu-miR-7226-5p	NM_173437.2	140.00	-19.96	2 25	8295 8320	23

mmu-miR-7226-5p	NM_173437.2	140.00	-24.30	2 22	9235 9261	21
mmu-miR-7226-5p	XM_006541297.3	162.00	-26.08	2 25	3307 3331	23
mmu-miR-7226-5p	XM_006541297.3	151.00	-29.83	2 25	7081 7105	24
mmu-miR-7226-5p	XM_006541297.3	146.00	-24.46	2 25	5313 5341	26
mmu-miR-7226-5p	XM_006541297.3	145.00	-26.81	2 25	3818 3841	23
mmu-miR-7226-5p	XM_006541297.3	142.00	-21.08	2 25	7363 7387	23
mmu-miR-7226-5p	XM_006541297.3	141.00	-17.93	2 22	933 958	20
mmu-miR-7226-5p	NM_001242558.1	142.00	-18.26	2 24	783 810	24
mmu-miR-7226-5p	NM_011424.3	147.00	-24.61	2 25	8608 8634	24
mmu-miR-7226-5p	NM_011424.3	143.00	-21.06	2 25	1673 1700	25
mmu-miR-7226-5p	XM_006529456.3	149.00	-19.89	2 23	3131 3158	23
mmu-miR-7226-5p	XM_006498901.3	164.00	-32.09	2 25	2825 2852	26
mmu-miR-7226-5p	XM_006498901.3	150.00	-28.72	2 23	1002 1027	21
mmu-miR-7226-5p	XM_006510077.3	159.00	-22.97	2 21	1516 1542	20
mmu-miR-7226-5p	XM_006510077.3	152.00	-26.04	3 25	1004 1027	22
mmu-miR-7226-5p	XM_006510077.3	143.00	-28.71	3 25	1308 1334	23
mmu-miR-7226-5p	XM_006510077.3	141.00	-17.99	3 23	2076 2102	21
mmu-miR-7226-5p	NM_001134300.2	153.00	-25.58	2 25	3399 3428	27
mmu-miR-7226-5p	NM_001134300.2	141.00	-23.05	3 23	1601 1628	22
mmu-miR-7226-5p	NM_001109985.1	147.00	-21.28	2 20	2042 2068	20
mmu-miR-7226-5p	NM_001109985.1	143.00	-19.61	2 14	1127 1151	12
mmu-miR-7226-5p	NM_001029877.3	156.00	-24.30	2 23	3498 3522	21
mmu-miR-7226-5p	NM_001029877.3	153.00	-25.75	2 25	2340 2368	26
mmu-miR-7226-5p	NM_001029877.3	141.00	-21.95	2 24	7585 7612	24
mmu-miR-7226-5p	NM_001029877.3	140.00	-14.99	2 24	7190 7215	23
mmu-miR-7226-5p	XM_006526747.3	146.00	-22.75	3 25	3605 3629	22
mmu-miR-7226-5p	XM_006526747.3	140.00	-21.19	3 24	2608 2634	23
mmu-miR-7226-5p	NM_133500.2	146.00	-25.19	2 24	1655 1682	24
mmu-miR-7226-5p	NM_133500.2	141.00	-20.13	2 23	504 526	21
mmu-miR-7226-5p	NM_001282961.1	151.00	-24.44	2 20	6929 6954	18
mmu-miR-7226-5p	NM_001282961.1	150.00	-24.32	2 25	6821 6845	23
mmu-miR-7226-5p	NM_001282961.1	146.00	-18.88	3 21	6773 6800	20
mmu-miR-7226-5p	NM_145210.2	146.00	-25.19	3 25	75 104	26
mmu-miR-7226-5p	NM_145210.2	144.00	-21.96	2 25	1310 1332	23
mmu-miR-7226-5p	XM_006530325.3	164.00	-24.08	2 21	3086 3111	19
mmu-miR-7226-5p	XM_006530325.3	151.00	-25.64	2 18	3903 3927	16
mmu-miR-7226-5p	XM_006530325.3	148.00	-22.14	2 21	3798 3823	19
mmu-miR-7226-5p	XM_006530325.3	146.00	-21.76	3 25	2814 2838	22
mmu-miR-7226-5p	XM_006530325.3	145.00	-31.30	3 25	376 401	23
mmu-miR-7226-5p	XM_006530325.3	142.00	-25.56	3 25	2983 3013	27
mmu-miR-7226-5p	NM_145226.2	151.00	-24.30	2 22	4272 4300	23
mmu-miR-7226-5p	NM_145226.2	142.00	-16.84	2 11	4506 4531	9
mmu-miR-7226-5p	NM_001310636.1	153.00	-23.64	2 22	984 1009	20
mmu-miR-7226-5p	NM_001310636.1	149.00	-29.35	2 22	1562 1585	20
mmu-miR-7226-5p	NM_172907.3	171.00	-33.25	2 25	344 371	25
mmu-miR-7226-5p	NM_172907.3	154.00	-24.59	2 23	1889 1914	21
mmu-miR-7226-5p	NM_172907.3	152.00	-20.56	2 25	297 322	23
mmu-miR-7226-5p	NM_172907.3	145.00	-21.72	2 24	949 973	22
mmu-miR-7226-5p	NM_011010.2	164.00	-24.61	2 25	649 674	23
mmu-miR-7226-5p	XM_011239969.2	144.00	-24.08	3 25	18 43	22
mmu-miR-7226-5p	NM_001286743.1	151.00	-21.61	2 17	2231 2258	17
mmu-miR-7226-5p	NM_001286743.1	147.00	-25.97	2 22	3766 3790	20
mmu-miR-7226-5p	NM_001286743.1	146.00	-20.78	3 25	3333 3357	22
mmu-miR-7226-5p	NM_001286743.1	142.00	-19.06	2 21	2428 2452	19
mmu-miR-7226-5p	XM_017322026.1	149.00	-19.39	2 19	88 114	18
mmu-miR-3473f	NM_001160262.1	148.00	-10.75	2 15	4234 4252	13
mmu-miR-3473f	NM_001160262.1	143.00	-12.21	2 16	2336 2352	14
mmu-miR-3473f	NM_001160262.1	140.00	-8.79	2 9	1771 1790	7
mmu-miR-3473f	NM_010181.2	143.00	-16.24	3 16	393 412	13
mmu-miR-3473f	NM_010181.2	142.00	-18.24	2 19	141 160	17
mmu-miR-3473f	XM_006527803.1	145.00	-21.29	2 18	2052 2071	16
mmu-miR-3473f	NM_001081185.1	142.00	-9.50	2 14	2653 2670	12
mmu-miR-3473f	NM_172862.3	140.00	-10.89	2 19	10366 10384	17
mmu-miR-3473f	NM_010279.3	147.00	-16.24	2 19	2226 2245	18
mmu-miR-3473f	XM_006530353.3	142.00	-19.17	2 17	1475 1493	15

mmu-miR-3473f	XM_011244235.2	141.00	-10.65	2 18	1408 1427	16
mmu-miR-3473f	XM_006532232.3	164.00	-24.97	2 19	2026 2047	19
mmu-miR-3473f	XM_011250978.2	152.00	-18.82	2 19	1818 1836	17
mmu-miR-3473f	NM_010340.2	140.00	-13.72	2 9	2374 2393	7
mmu-miR-3473f	XM_006526069.2	156.00	-14.42	2 17	5073 5092	15
mmu-miR-3473f	XM_006526069.2	152.00	-14.95	2 18	1176 1198	19
mmu-miR-3473f	XM_006526069.2	147.00	-9.92	2 18	1778 1796	16
mmu-miR-3473f	XM_006526069.2	146.00	-14.36	2 15	4954 4973	13
mmu-miR-3473f	XM_006499444.3	145.00	-15.24	2 18	1718 1737	16
mmu-miR-3473f	NM_001162950.1	146.00	-12.83	2 19	4144 4163	17
mmu-miR-3473f	XM_006505602.3	144.00	-17.91	2 19	8741 8759	17
mmu-miR-3473f	NM_001164086.1	148.00	-12.63	2 13	6970 6989	11
mmu-miR-3473f	NM_001164086.1	144.00	-18.02	2 18	9647 9667	17
mmu-miR-3473f	NM_001164086.1	140.00	-10.64	2 17	10237 10256	15
mmu-miR-3473f	NM_013558.2	158.00	-11.28	2 19	1091 1110	17
mmu-miR-3473f	NM_013558.2	151.00	-15.58	2 14	611 629	12
mmu-miR-3473f	NM_001159424.2	141.00	-7.72	2 12	1180 1198	10
mmu-miR-3473f	XM_006528842.1	152.00	-24.95	2 19	3991 4014	21
mmu-miR-3473f	XM_006528842.1	149.00	-16.31	2 18	5906 5925	16
mmu-miR-3473f	NM_001289875.1	141.00	-13.03	2 18	3735 3754	16
mmu-miR-3473f	NM_001289875.1	141.00	-12.03	2 19	4094 4114	18
mmu-miR-3473f	XM_017315734.1	145.00	-15.22	3 19	4630 4650	17
mmu-miR-3473f	NM_010608.2	149.00	-18.42	3 19	700 720	17
mmu-miR-3473f	XM_011248919.2	149.00	-11.83	2 18	6655 6674	16
mmu-miR-3473f	NM_001081667.2	145.00	-14.34	2 10	1089 1108	8
mmu-miR-3473f	XM_017316676.1	140.00	-15.47	2 19	18049 18067	17
mmu-miR-3473f	XM_006523061.3	143.00	-10.01	2 16	2548 2567	14
mmu-miR-3473f	XM_006511102.2	153.00	-12.22	2 18	1159 1178	16
mmu-miR-3473f	XM_006504999.1	143.00	-12.51	2 18	1035 1053	16
mmu-miR-3473f	NM_144945.3	153.00	-18.57	3 18	2269 2288	15
mmu-miR-3473f	NM_175271.4	150.00	-20.31	2 19	43 62	17
mmu-miR-3473f	XM_006506340.3	150.00	-11.53	2 19	3543 3559	17
mmu-miR-3473f	NM_001171187.1	144.00	-7.46	2 13	670 689	11
mmu-miR-3473f	XM_006541232.3	141.00	-16.54	3 14	251 270	11
mmu-miR-3473f	XM_006541232.3	140.00	-13.20	2 17	5184 5203	15
mmu-miR-3473f	NM_205810.4	147.00	-12.90	2 19	3560 3582	20
mmu-miR-3473f	NM_205810.4	142.00	-19.18	2 19	2417 2436	17
mmu-miR-3473f	XM_017320223.1	145.00	-19.69	2 19	1888 1908	18
mmu-miR-3473f	XM_017320223.1	142.00	-10.11	2 12	4381 4401	11
mmu-miR-3473f	NM_173437.2	157.00	-14.02	2 19	6525 6546	19
mmu-miR-3473f	NM_173437.2	150.00	-17.98	3 19	9492 9511	16
mmu-miR-3473f	NM_173437.2	144.00	-8.58	2 17	6506 6525	15
mmu-miR-3473f	NM_173437.2	143.00	-7.64	2 15	10627 10644	13
mmu-miR-3473f	NM_173437.2	143.00	-15.69	2 18	12267 12285	16
mmu-miR-3473f	NM_001242558.1	141.00	-11.64	2 18	3442 3461	16
mmu-miR-3473f	XM_006498901.3	147.00	-8.04	2 12	964 983	10
mmu-miR-3473f	XM_006510077.3	142.00	-14.94	2 17	903 921	15
mmu-miR-3473f	NM_001134300.2	158.00	-18.11	2 19	5069 5088	17
mmu-miR-3473f	NM_001134300.2	143.00	-11.63	2 16	2519 2538	14
mmu-miR-3473f	NM_001029877.3	149.00	-19.98	2 19	6860 6880	18
mmu-miR-3473f	NM_001077403.1	166.00	-20.79	2 19	4582 4601	17
mmu-miR-3473f	NM_001282961.1	145.00	-14.17	3 18	3415 3434	15
mmu-miR-3473f	NM_001282961.1	142.00	-8.66	2 15	3617 3636	13
mmu-miR-3473f	NM_011854.2	147.00	-11.54	2 16	1209 1228	14
mmu-miR-3473f	NM_008760.4	144.00	-13.95	2 13	2375 2394	11
mmu-miR-3473f	NM_008760.4	140.00	-19.89	2 18	766 786	17
mmu-miR-3473f	NM_011010.2	145.00	-16.30	2 19	2047 2067	18
mmu-miR-3473f	XM_011239969.2	141.00	-14.72	3 19	3281 3301	17
mmu-miR-8094	NM_177743.5	145.00	-16.62	2 19	3489 3510	18
mmu-miR-8094	XM_017319626.1	162.00	-16.45	2 19	3112 3132	17
mmu-miR-8094	XM_017319626.1	141.00	-10.03	2 10	304 324	8
mmu-miR-8094	XM_006532962.2	158.00	-17.86	2 19	2655 2675	17
mmu-miR-8094	NM_001160262.1	142.00	-15.01	2 19	2967 2987	17
mmu-miR-8094	NM_010181.2	147.00	-17.15	2 16	9425 9445	14
mmu-miR-8094	NM_001081185.1	140.00	-18.74	3 19	6248 6267	16

mmu-miR-8094	NM_172862.3	150.00	-9.85	2 17	10878 10897	15
mmu-miR-8094	NM_172862.3	143.00	-19.00	2 18	481 503	18
mmu-miR-8094	NM_172862.3	140.00	-12.36	2 9	400 420	7
mmu-miR-8094	XM_006533539.3	156.00	-17.46	2 19	4556 4575	17
mmu-miR-8094	XM_006533539.3	141.00	-11.17	2 10	6145 6165	8
mmu-miR-8094	NM_001033221.3	152.00	-16.21	2 19	1531 1556	22
mmu-miR-8094	NM_145741.2	152.00	-16.47	2 17	1884 1904	15
mmu-miR-8094	XM_006510297.3	146.00	-11.43	2 16	749 770	15
mmu-miR-8094	XM_006530353.3	143.00	-22.10	3 20	2822 2842	17
mmu-miR-8094	XM_006530353.3	142.00	-11.56	2 11	11659 11679	9
mmu-miR-8094	XM_011248576.2	147.00	-14.37	2 12	6341 6361	10
mmu-miR-8094	XM_011248576.2	147.00	-16.99	2 12	10867 10887	10
mmu-miR-8094	XM_011238891.1	141.00	-18.86	2 20	112 131	18
mmu-miR-8094	NM_001198955.1	145.00	-17.03	2 16	1883 1902	14
mmu-miR-8094	XM_011250978.2	149.00	-17.56	2 19	1254 1275	18
mmu-miR-8094	XM_011250978.2	147.00	-15.50	2 16	4299 4319	14
mmu-miR-8094	XM_006521554.3	147.00	-13.10	2 12	2154 2174	10
mmu-miR-8094	XM_006515096.2	154.00	-14.06	2 20	4469 4490	19
mmu-miR-8094	XM_006515096.2	152.00	-13.60	2 13	3124 3144	11
mmu-miR-8094	XM_006515096.2	148.00	-13.17	2 13	3688 3708	11
mmu-miR-8094	XM_006515096.2	141.00	-12.01	2 11	5892 5913	10
mmu-miR-8094	XM_006515096.2	140.00	-11.63	2 9	1668 1688	7
mmu-miR-8094	XM_006526069.2	147.00	-11.00	2 16	1185 1205	14
mmu-miR-8094	NM_008204.2	154.00	-14.91	2 20	1078 1099	19
mmu-miR-8094	NM_001162950.1	145.00	-16.04	3 18	4437 4457	15
mmu-miR-8094	XM_006505602.3	155.00	-16.55	2 16	6030 6050	14
mmu-miR-8094	XM_006505602.3	140.00	-9.36	2 9	8911 8931	7
mmu-miR-8094	NM_001164086.1	150.00	-13.08	2 15	6302 6322	13
mmu-miR-8094	NM_001164086.1	140.00	-9.79	2 9	6757 6777	7
mmu-miR-8094	XM_006511311.2	150.00	-16.10	2 20	5373 5394	19
mmu-miR-8094	XM_006511311.2	141.00	-10.32	2 10	2788 2808	8
mmu-miR-8094	NM_001190325.1	143.00	-9.96	2 16	232 252	14
mmu-miR-8094	XM_017316439.1	143.00	-17.80	2 18	1219 1241	18
mmu-miR-8094	XM_006496593.3	149.00	-16.69	2 18	4679 4699	16
mmu-miR-8094	XM_006496593.3	146.00	-11.97	2 18	3049 3067	16
mmu-miR-8094	XM_006528842.1	150.00	-16.59	2 11	1909 1929	9
mmu-miR-8094	XM_006528842.1	145.00	-12.41	2 14	4540 4560	12
mmu-miR-8094	NM_001161541.1	145.00	-14.45	2 10	2531 2551	8
mmu-miR-8094	NM_001302471.1	148.00	-16.37	2 18	449 466	16
mmu-miR-8094	NM_001289875.1	140.00	-11.88	2 9	1151 1171	7
mmu-miR-8094	NM_001289875.1	140.00	-9.33	2 9	2123 2143	7
mmu-miR-8094	NM_008423.2	150.00	-15.47	2 19	3296 3316	17
mmu-miR-8094	XM_017315734.1	166.00	-17.53	2 20	75 96	19
mmu-miR-8094	XM_011248919.2	140.00	-9.36	2 9	3029 3049	7
mmu-miR-8094	XM_017316478.1	151.00	-13.16	2 19	1625 1643	17
mmu-miR-8094	XM_017316478.1	142.00	-13.53	2 18	4095 4113	16
mmu-miR-8094	XM_017316478.1	141.00	-19.96	2 19	5702 5723	18
mmu-miR-8094	NM_173427.2	142.00	-10.74	2 17	4270 4289	15
mmu-miR-8094	XM_017320093.1	141.00	-13.76	2 20	2285 2304	18
mmu-miR-8094	NM_001081667.2	152.00	-15.81	2 13	1833 1853	11
mmu-miR-8094	NM_016659.3	148.00	-22.76	3 18	1134 1155	16
mmu-miR-8094	NM_016659.3	146.00	-17.80	2 20	744 765	19
mmu-miR-8094	NM_029274.2	141.00	-11.72	2 16	7260 7279	14
mmu-miR-8094	NM_008479.2	141.00	-17.60	3 19	405 426	17
mmu-miR-8094	XM_017313062.1	141.00	-15.22	3 20	2857 2879	19
mmu-miR-8094	XM_006511102.2	142.00	-11.18	2 11	812 832	9
mmu-miR-8094	XM_006504999.1	142.00	-15.54	3 19	801 821	16
mmu-miR-8094	NM_144945.3	142.00	-12.21	2 17	5187 5206	15
mmu-miR-8094	NM_010714.3	141.00	-11.11	2 10	2981 3001	8
mmu-miR-8094	NM_010714.3	141.00	-18.35	3 19	4394 4415	17
mmu-miR-8094	XM_006519969.3	160.00	-16.13	2 18	3440 3461	17
mmu-miR-8094	XM_006519969.3	140.00	-13.93	3 17	2529 2549	14
mmu-miR-8094	NM_175271.4	149.00	-21.05	3 20	3806 3825	17
mmu-miR-8094	XM_006506340.3	148.00	-13.86	2 18	1333 1355	18
mmu-miR-8094	XM_006541232.3	146.00	-17.25	3 19	1073 1093	16

mmu-miR-8094	XM_006541232.3	146.00	-11.89	2	11	1321	1341	9
mmu-miR-8094	XM_006541232.3	143.00	-13.77	2	19	5796	5814	17
mmu-miR-8094	XM_006498872.3	150.00	-16.57	2	11	6435	6455	9
mmu-miR-8094	XM_006498872.3	142.00	-9.35	2	11	2474	2494	9
mmu-miR-8094	XM_006532410.3	161.00	-18.30	2	20	5553	5575	20
mmu-miR-8094	XM_006504940.3	140.00	-11.72	2	9	602	622	7
mmu-miR-8094	NM_010825.3	145.00	-18.75	2	20	2216	2235	18
mmu-miR-8094	NM_001320077.1	146.00	-18.78	2	20	1332	1353	19
mmu-miR-8094	NM_010809.2	161.00	-17.61	2	20	1417	1436	18
mmu-miR-8094	NM_010809.2	148.00	-15.00	2	19	367	386	17
mmu-miR-8094	XM_006537659.2	143.00	-18.97	3	18	426	445	15
mmu-miR-8094	XM_006529753.2	151.00	-16.37	2	16	1841	1861	14
mmu-miR-8094	NM_175260.2	150.00	-16.73	2	20	1337	1358	19
mmu-miR-8094	NM_001039545.2	141.00	-10.73	2	10	1956	1976	8
mmu-miR-8094	NM_010858.4	140.00	-9.70	2	9	266	286	7
mmu-miR-8094	XM_006513859.3	142.00	-9.55	2	11	676	696	9
mmu-miR-8094	XM_006501829.3	145.00	-13.01	2	10	110	130	8
mmu-miR-8094	NM_173437.2	145.00	-16.52	2	10	3688	3708	8
mmu-miR-8094	NM_173437.2	142.00	-19.29	2	19	2980	3000	17
mmu-miR-8094	NM_173437.2	141.00	-11.61	2	14	11133	11153	12
mmu-miR-8094	NM_173437.2	140.00	-12.82	2	9	2870	2890	7
mmu-miR-8094	XM_006541297.3	140.00	-15.46	3	19	5164	5183	16
mmu-miR-8094	NM_011424.3	146.00	-11.75	2	11	6974	6994	9
mmu-miR-8094	NM_001134300.2	141.00	-16.48	3	18	5755	5775	15
mmu-miR-8094	NM_001109985.1	152.00	-14.40	2	14	2740	2761	13
mmu-miR-8094	NM_181547.3	141.00	-14.22	3	19	179	200	17
mmu-miR-8094	NM_001282961.1	144.00	-10.05	2	13	1558	1578	11
mmu-miR-8094	NM_145226.2	141.00	-13.66	2	20	4650	4669	18
mmu-miR-8094	NM_145226.2	140.00	-10.80	2	9	4420	4440	7
mmu-miR-8094	XM_006530294.3	143.00	-19.64	3	20	780	800	17
mmu-miR-8094	XM_006530294.3	142.00	-11.42	2	17	1854	1873	15
mmu-miR-8094	NM_008760.4	150.00	-16.56	2	19	834	854	17
mmu-miR-8094	NM_008760.4	141.00	-11.76	2	10	324	344	8
mmu-miR-8094	NM_008760.4	140.00	-12.55	2	9	89	109	7
mmu-miR-8094	XM_006516928.2	154.00	-19.76	2	19	523	543	17
mmu-miR-8094	XM_006516928.2	145.00	-16.16	2	10	3353	3373	8
mmu-miR-8094	XM_006516928.2	143.00	-10.47	2	20	1388	1408	18
mmu-miR-8094	XM_011239969.2	149.00	-16.90	2	20	6415	6434	18
mmu-miR-8117	NM_177743.5	145.00	-21.63	2	20	3280	3299	18
mmu-miR-8117	XM_017319626.1	142.00	-24.81	3	20	3585	3606	18
mmu-miR-8117	NM_010181.2	140.00	-24.45	2	9	3651	3671	7
mmu-miR-8117	XM_006527803.1	146.00	-21.92	3	20	1807	1828	18
mmu-miR-8117	NM_172862.3	153.00	-26.14	2	20	6714	6739	23
mmu-miR-8117	NM_172862.3	140.00	-15.44	2	9	5763	5783	7
mmu-miR-8117	NM_010279.3	145.00	-15.97	2	10	4520	4540	8
mmu-miR-8117	XM_006510297.3	144.00	-14.34	2	14	2584	2605	13
mmu-miR-8117	XM_011248576.2	146.00	-29.55	2	20	9484	9506	20
mmu-miR-8117	XM_011248576.2	140.00	-13.63	2	9	11149	11169	7
mmu-miR-8117	XM_011246309.2	161.00	-25.14	2	20	1030	1054	22
mmu-miR-8117	NM_175520.4	141.00	-23.89	3	19	532	553	17
mmu-miR-8117	XM_006505602.3	140.00	-12.22	2	9	7381	7401	7
mmu-miR-8117	NM_001164086.1	149.00	-15.33	2	14	9714	9734	12
mmu-miR-8117	NM_001164086.1	147.00	-29.75	2	20	174	194	18
mmu-miR-8117	NM_133871.2	140.00	-20.52	2	9	713	733	7
mmu-miR-8117	XM_006511311.2	140.00	-17.10	2	9	2218	2238	7
mmu-miR-8117	NM_008423.2	150.00	-24.27	2	11	610	630	9
mmu-miR-8117	NM_021342.1	143.00	-19.26	2	18	1554	1573	16
mmu-miR-8117	XM_006497892.3	151.00	-26.26	2	16	3240	3260	14
mmu-miR-8117	XM_017316676.1	143.00	-26.22	2	16	10163	10183	14
mmu-miR-8117	NM_028973.2	146.00	-21.09	2	11	3249	3269	9
mmu-miR-8117	NM_001171187.1	155.00	-17.04	2	20	2455	2475	18
mmu-miR-8117	XM_006541232.3	140.00	-25.20	2	17	4491	4511	15
mmu-miR-8117	XM_006498872.3	145.00	-18.19	2	10	8964	8984	8
mmu-miR-8117	XM_006520023.2	140.00	-17.22	2	9	1261	1281	7
mmu-miR-8117	NM_001270475.1	142.00	-21.24	2	11	8586	8606	9

mmu-miR-8117	NM_001290512.1	140.00	-15.99	2 9	1246 1266	7
mmu-miR-8117	NM_010809.2	149.00	-24.36	2 18	1156 1176	16
mmu-miR-8117	NM_001039545.2	147.00	-17.39	2 17	5658 5679	16
mmu-miR-8117	XM_006513859.3	142.00	-14.73	2 19	932 952	17
mmu-miR-8117	NM_173437.2	166.00	-27.76	2 20	9037 9058	19
mmu-miR-8117	XM_006541297.3	140.00	-20.37	2 9	1827 1847	7
mmu-miR-8117	NM_011424.3	153.00	-19.42	2 18	5812 5832	16
mmu-miR-8117	XM_006526747.3	142.00	-18.39	2 20	3998 4019	19
mmu-miR-8117	NM_133500.2	143.00	-15.62	2 12	747 767	10
mmu-miR-206-3p	NM_172454.2	160.00	-19.18	2 21	2221 2242	19
mmu-miR-206-3p	NM_133167.3	146.00	-19.60	2 21	2426 2446	19
mmu-miR-206-3p	NM_013743.2	145.00	-20.18	2 20	831 851	18
mmu-miR-206-3p	XM_006509465.2	152.00	-13.87	2 13	632 653	11
mmu-miR-206-3p	NM_001290822.1	147.00	-17.60	2 19	1214 1233	17
mmu-miR-206-3p	NM_001164593.1	156.00	-18.90	2 21	2550 2571	19
mmu-miR-206-3p	XM_006538456.3	147.00	-17.49	2 21	5021 5044	21
mmu-miR-206-3p	NM_153104.3	144.00	-20.07	2 13	1785 1806	11
mmu-miR-206-3p	XM_017314870.1	152.00	-21.77	2 21	2923 2944	19
mmu-miR-206-3p	XM_017314870.1	140.00	-11.16	2 9	5893 5914	7
mmu-miR-206-3p	XM_017312497.1	148.00	-14.40	2 21	1241 1262	19
mmu-miR-206-3p	XM_006502235.3	140.00	-14.57	2 9	5208 5229	7
mmu-miR-206-3p	NM_001195084.1	147.00	-22.68	2 21	507 532	23
mmu-miR-206-3p	NM_148932.2	153.00	-20.44	2 20	2769 2789	18
mmu-miR-206-3p	NM_148932.2	141.00	-18.95	3 19	1671 1693	17
mmu-miR-206-3p	XM_006527008.2	150.00	-13.90	2 20	3620 3642	19
mmu-miR-206-3p	NM_011169.5	151.00	-17.12	2 21	9333 9355	20
mmu-miR-206-3p	NM_011169.5	150.00	-14.90	2 11	5085 5106	9
mmu-miR-206-3p	NM_011169.5	145.00	-12.31	2 10	3115 3136	8
mmu-miR-206-3p	NM_011169.5	143.00	-16.35	2 21	7143 7161	19
mmu-miR-206-3p	NM_011169.5	141.00	-16.18	2 21	5417 5438	20
mmu-miR-206-3p	NM_011169.5	140.00	-17.85	3 21	5143 5164	18
mmu-miR-206-3p	NM_175563.5	155.00	-20.47	2 21	3645 3667	20
mmu-miR-206-3p	XM_006505015.3	156.00	-17.40	2 21	5510 5531	19
mmu-miR-206-3p	NM_027455.3	149.00	-15.28	2 21	1115 1136	20
mmu-miR-206-3p	NM_027455.3	140.00	-16.97	2 9	1269 1290	7
mmu-miR-206-3p	NM_198024.2	142.00	-14.51	2 21	4074 4094	19
mmu-miR-206-3p	NM_031192.3	147.00	-20.17	2 20	1004 1025	18
mmu-miR-206-3p	XM_006529829.3	145.00	-11.55	2 10	1819 1840	8
mmu-miR-206-3p	XM_006529829.3	144.00	-21.96	3 17	1547 1568	14
mmu-miR-206-3p	XM_011240779.2	140.00	-12.46	2 9	4882 4903	7
mmu-miR-206-3p	XM_006507942.2	147.00	-20.04	2 21	1888 1911	21
mmu-miR-206-3p	XM_006507942.2	142.00	-15.70	2 20	1611 1633	19
mmu-miR-206-3p	NM_001195662.1	163.00	-25.54	2 21	925 947	20
mmu-miR-206-3p	NM_019732.2	140.00	-11.09	2 9	122 143	7
mmu-miR-206-3p	NM_018732.3	140.00	-10.08	2 9	463 484	7
mmu-miR-206-3p	NM_008458.2	140.00	-11.72	2 9	1271 1292	7
mmu-miR-206-3p	XM_011250732.2	140.00	-16.77	2 9	3306 3327	7
mmu-miR-206-3p	NM_177578.4	143.00	-16.31	3 18	845 865	15
mmu-miR-206-3p	XM_006496884.3	145.00	-17.20	2 10	1709 1730	8
mmu-miR-206-3p	NM_001310705.1	140.00	-14.98	2 9	1380 1401	7
mmu-miR-206-3p	NM_009199.2	161.00	-18.03	2 19	1075 1098	19
mmu-miR-206-3p	XM_006503080.3	144.00	-23.57	2 21	2773 2794	19
mmu-miR-206-3p	XM_006538687.2	140.00	-20.39	2 21	4464 4485	19
mmu-miR-206-3p	XM_006500109.3	144.00	-17.10	2 21	41 62	19
mmu-miR-206-3p	NM_009223.3	144.00	-15.34	2 21	2309 2330	19
mmu-miR-206-3p	XM_006534520.3	156.00	-16.60	2 21	3075 3096	19
mmu-miR-206-3p	NM_031183.2	141.00	-12.18	2 21	2000 2021	20
mmu-miR-206-3p	NM_178753.4	150.00	-23.02	2 20	3406 3428	19
mmu-miR-206-3p	NM_001204201.1	147.00	-21.86	2 21	817 840	21
mmu-miR-206-3p	NM_001304266.1	152.00	-23.30	2 21	8570 8591	19
mmu-miR-206-3p	XM_011245529.2	140.00	-13.71	2 17	3884 3905	15
mmu-miR-206-3p	NM_001080979.1	151.00	-20.84	2 21	336 358	20
mmu-miR-206-3p	XM_006537836.1	140.00	-15.12	2 9	819 840	7
mmu-miR-206-3p	XM_006515787.3	140.00	-9.59	2 9	1217 1238	7
mmu-miR-206-3p	XM_006538830.1	146.00	-15.50	2 21	1623 1647	22

mmu-miR-206-3p	NM_177371.3	152.00	-17.59	2 21	4163 4184	19
mmu-miR-206-3p	NM_177371.3	151.00	-18.91	2 21	529 553	22
mmu-miR-206-3p	NM_177371.3	149.00	-16.92	2 21	1266 1285	19
mmu-miR-206-3p	XM_006529382.3	140.00	-13.71	3 21	223 244	18
mmu-miR-206-3p	NM_146241.2	151.00	-15.11	2 18	3372 3396	19
mmu-miR-206-3p	NM_146241.2	146.00	-12.66	2 21	3953 3976	21
mmu-miR-206-3p	XM_011249157.2	154.00	-20.42	2 21	555 575	19
mmu-miR-206-3p	NM_001243916.1	144.00	-18.10	2 13	448 469	11
mmu-miR-206-3p	XM_006508002.3	155.00	-20.95	2 21	2705 2727	20
mmu-miR-206-3p	XM_006506152.3	142.00	-19.82	2 20	2353 2375	19
mmu-miR-206-3p	NM_001033149.3	163.00	-15.06	2 21	4506 4528	20
mmu-miR-206-3p	XM_006525099.3	143.00	-14.63	2 16	260 281	14
mmu-miR-206-3p	XM_006525028.2	146.00	-13.36	2 19	2573 2594	17
mmu-miR-206-3p	NM_001244031.1	158.00	-19.26	2 21	442 462	19
mmu-miR-206-3p	NM_177789.4	149.00	-17.68	2 18	696 717	16
mmu-miR-206-3p	NM_009518.2	150.00	-18.06	2 20	1561 1583	19
mmu-miR-206-3p	XM_011243586.2	142.00	-18.37	2 20	722 744	19
mmu-miR-206-3p	XM_006511007.3	146.00	-17.26	2 21	5791 5811	19
mmu-miR-206-3p	XM_006537508.2	140.00	-13.79	2 9	4260 4281	7
mmu-miR-206-3p	XM_006519134.3	155.00	-14.18	2 21	2620 2643	21
mmu-miR-206-3p	XM_006519134.3	148.00	-13.22	2 21	3162 3183	19
mmu-miR-214-3p	NM_172454.2	160.00	-22.66	2 21	197 219	21
mmu-miR-214-3p	NM_172454.2	150.00	-22.79	2 21	568 591	21
mmu-miR-214-3p	NM_028748.2	152.00	-19.90	2 18	133 155	17
mmu-miR-214-3p	NM_133167.3	151.00	-22.99	2 21	1778 1802	22
mmu-miR-214-3p	NM_133167.3	140.00	-13.18	2 9	2300 2321	7
mmu-miR-214-3p	NM_008804.4	149.00	-18.42	2 14	1210 1231	12
mmu-miR-214-3p	NM_013743.2	148.00	-19.73	2 21	1695 1716	19
mmu-miR-214-3p	XM_006509465.2	150.00	-20.85	2 11	1392 1413	9
mmu-miR-214-3p	NM_001290822.1	157.00	-27.79	2 20	99 119	18
mmu-miR-214-3p	NM_001164593.1	165.00	-28.31	2 19	553 575	18
mmu-miR-214-3p	NM_001164593.1	152.00	-22.13	2 21	218 238	20
mmu-miR-214-3p	NM_001164593.1	140.00	-13.62	2 9	491 512	7
mmu-miR-214-3p	XM_006538456.3	159.00	-20.44	2 16	2758 2779	14
mmu-miR-214-3p	XM_006538456.3	154.00	-23.85	2 21	1580 1606	24
mmu-miR-214-3p	XM_006538456.3	153.00	-21.93	2 19	2970 2992	18
mmu-miR-214-3p	XM_006538456.3	145.00	-21.98	3 19	3123 3145	17
mmu-miR-214-3p	XM_006538456.3	145.00	-16.94	2 18	4045 4063	16
mmu-miR-214-3p	XM_006538456.3	144.00	-18.98	2 21	5207 5228	19
mmu-miR-214-3p	XM_006538456.3	142.00	-28.97	2 21	4499 4520	20
mmu-miR-214-3p	XM_006537815.3	144.00	-15.36	2 21	1431 1452	19
mmu-miR-214-3p	XM_006537815.3	140.00	-14.76	2 9	1935 1956	7
mmu-miR-214-3p	XM_006537815.3	140.00	-13.62	2 9	3986 4007	7
mmu-miR-214-3p	XM_006537815.3	140.00	-18.29	2 9	6064 6085	7
mmu-miR-214-3p	NM_153104.3	140.00	-17.78	2 9	1657 1678	7
mmu-miR-214-3p	XM_017314870.1	170.00	-30.40	2 21	5113 5137	22
mmu-miR-214-3p	XM_017314870.1	164.00	-19.53	2 17	4172 4193	15
mmu-miR-214-3p	XM_017314870.1	156.00	-16.07	2 17	5734 5755	15
mmu-miR-214-3p	XM_017314870.1	150.00	-22.14	2 21	5755 5775	19
mmu-miR-214-3p	XM_017314870.1	146.00	-18.35	2 15	379 400	13
mmu-miR-214-3p	XM_017314870.1	146.00	-19.83	2 21	2545 2572	25
mmu-miR-214-3p	XM_017317946.1	150.00	-18.17	2 17	1092 1112	15
mmu-miR-214-3p	NM_018807.5	163.00	-25.85	2 21	2102 2124	20
mmu-miR-214-3p	NM_018807.5	154.00	-24.00	2 15	4902 4923	13
mmu-miR-214-3p	NM_008872.3	164.00	-20.33	2 21	1158 1179	19
mmu-miR-214-3p	XM_017312497.1	159.00	-21.27	2 20	841 862	18
mmu-miR-214-3p	XM_017312497.1	149.00	-15.94	2 14	1230 1251	12
mmu-miR-214-3p	XM_017312497.1	147.00	-18.93	2 21	2307 2329	20
mmu-miR-214-3p	XM_011250776.2	140.00	-15.28	2 9	207 228	7
mmu-miR-214-3p	NM_026385.4	140.00	-19.04	2 20	1697 1718	19
mmu-miR-214-3p	XM_006502235.3	155.00	-19.58	2 20	4646 4667	18
mmu-miR-214-3p	NM_001195084.1	151.00	-18.83	2 21	1117 1139	20
mmu-miR-214-3p	XM_006512076.3	167.00	-25.29	2 16	6784 6805	14
mmu-miR-214-3p	XM_006512076.3	165.00	-27.02	2 14	4073 4094	12
mmu-miR-214-3p	NM_001302257.1	145.00	-24.84	2 16	57 77	14

mmu-miR-214-3p	NM_001302257.1	140.00	-19.60	3 21	1744 1765	18
mmu-miR-214-3p	NM_001291068.1	153.00	-22.33	2 19	1177 1201	20
mmu-miR-214-3p	NM_001291068.1	150.00	-23.07	3 20	6625 6647	18
mmu-miR-214-3p	NM_148932.2	161.00	-29.38	2 21	1694 1717	22
mmu-miR-214-3p	NM_148932.2	147.00	-18.03	2 12	1033 1054	10
mmu-miR-214-3p	NM_148932.2	146.00	-15.91	2 15	1795 1816	13
mmu-miR-214-3p	NM_148932.2	143.00	-15.00	2 20	3199 3220	18
mmu-miR-214-3p	NM_011145.3	146.00	-16.85	2 19	417 438	17
mmu-miR-214-3p	NM_011145.3	146.00	-25.65	3 21	2780 2803	20
mmu-miR-214-3p	NM_011145.3	144.00	-21.81	2 19	1823 1843	17
mmu-miR-214-3p	XM_006525698.2	158.00	-23.12	2 20	7937 7960	20
mmu-miR-214-3p	XM_006525698.2	152.00	-25.89	2 21	554 575	19
mmu-miR-214-3p	XM_006525698.2	152.00	-28.11	2 21	7686 7707	19
mmu-miR-214-3p	XM_006525698.2	143.00	-20.45	2 21	7422 7444	20
mmu-miR-214-3p	XM_006527008.2	169.00	-32.44	2 21	2571 2594	22
mmu-miR-214-3p	XM_006527008.2	142.00	-17.91	2 16	817 839	15
mmu-miR-214-3p	XM_006527008.2	142.00	-19.86	2 11	3401 3422	9
mmu-miR-214-3p	XM_006527008.2	141.00	-15.81	2 12	1680 1700	10
mmu-miR-214-3p	XM_006527008.2	140.00	-19.98	2 18	1779 1802	18
mmu-miR-214-3p	NM_011169.5	145.00	-25.30	2 21	16 37	20
mmu-miR-214-3p	NM_011169.5	144.00	-20.75	2 21	1646 1667	19
mmu-miR-214-3p	NM_011169.5	140.00	-13.62	2 9	853 874	7
mmu-miR-214-3p	NM_175563.5	142.00	-19.66	2 21	1796 1819	21
mmu-miR-214-3p	NM_175022.2	159.00	-23.11	2 17	630 653	17
mmu-miR-214-3p	NM_175022.2	159.00	-23.87	2 19	6055 6076	18
mmu-miR-214-3p	NM_175022.2	158.00	-25.78	2 20	5127 5150	20
mmu-miR-214-3p	NM_175022.2	146.00	-16.11	2 19	3202 3223	17
mmu-miR-214-3p	XM_006524382.3	142.00	-22.36	2 19	651 672	17
mmu-miR-214-3p	XM_011240476.2	151.00	-14.89	2 21	1977 1999	20
mmu-miR-214-3p	XM_011240476.2	143.00	-20.67	3 21	2273 2295	19
mmu-miR-214-3p	XM_011240476.2	140.00	-22.11	2 21	1843 1862	19
mmu-miR-214-3p	XM_011240476.2	140.00	-17.44	2 9	2428 2449	7
mmu-miR-214-3p	NM_008966.3	151.00	-22.65	2 21	1063 1085	20
mmu-miR-214-3p	NM_016933.3	155.00	-22.23	2 21	166 188	20
mmu-miR-214-3p	NM_016933.3	147.00	-19.09	2 14	153 173	12
mmu-miR-214-3p	NM_016933.3	140.00	-16.90	2 9	539 560	7
mmu-miR-214-3p	XM_006505015.3	140.00	-14.71	2 9	4112 4133	7
mmu-miR-214-3p	NM_027455.3	147.00	-17.07	2 20	388 409	18
mmu-miR-214-3p	XM_006514153.2	154.00	-21.94	2 18	295 316	17
mmu-miR-214-3p	XM_006506624.2	140.00	-23.20	2 20	124 145	19
mmu-miR-214-3p	XM_017320735.1	150.00	-18.69	2 19	2332 2353	17
mmu-miR-214-3p	XM_017320735.1	149.00	-21.82	2 14	2994 3015	12
mmu-miR-214-3p	XM_017320735.1	146.00	-15.85	2 15	2428 2449	13
mmu-miR-214-3p	XM_017320735.1	142.00	-14.33	2 11	2554 2575	9
mmu-miR-214-3p	XM_017320735.1	140.00	-16.83	2 9	3259 3280	7
mmu-miR-214-3p	NM_016809.6	146.00	-22.89	2 21	1312 1336	22
mmu-miR-214-3p	NM_031192.3	140.00	-16.33	2 9	711 732	7
mmu-miR-214-3p	XM_006529829.3	150.00	-24.15	2 20	1849 1871	19
mmu-miR-214-3p	XM_006529829.3	142.00	-16.37	2 20	4714 4738	21
mmu-miR-214-3p	XM_006529829.3	141.00	-15.04	2 20	1330 1350	18
mmu-miR-214-3p	XM_006507942.2	145.00	-17.63	2 15	717 739	14
mmu-miR-214-3p	XM_006507942.2	140.00	-14.62	2 9	744 765	7
mmu-miR-214-3p	XM_006525075.3	149.00	-22.98	2 20	838 858	18
mmu-miR-214-3p	NM_019732.2	140.00	-18.24	2 9	2680 2701	7
mmu-miR-214-3p	NM_023893.4	145.00	-29.04	3 21	879 905	23
mmu-miR-214-3p	XM_006540138.3	154.00	-20.32	2 20	385 407	19
mmu-miR-214-3p	XM_006540138.3	141.00	-19.70	3 21	1250 1271	19
mmu-miR-214-3p	XM_006540134.3	148.00	-24.06	2 19	607 632	21
mmu-miR-214-3p	NM_009127.4	148.00	-15.71	2 18	513 535	17
mmu-miR-214-3p	NM_009127.4	143.00	-17.85	2 20	942 961	18
mmu-miR-214-3p	NM_018732.3	140.00	-13.18	2 9	7963 7984	7
mmu-miR-214-3p	NM_009135.2	156.00	-19.62	2 21	567 588	19
mmu-miR-214-3p	NM_009243.4	146.00	-26.55	2 20	1400 1422	19
mmu-miR-214-3p	NM_009244.4	146.00	-26.55	2 20	1395 1417	19
mmu-miR-214-3p	NM_009246.3	146.00	-26.38	2 20	1405 1427	19

mmu-miR-214-3p	NM_009246.3	142.00	-14.02	3 19	972 993	16
mmu-miR-214-3p	XM_011244046.2	153.00	-24.32	2 21	141 164	22
mmu-miR-214-3p	XM_006521796.1	140.00	-14.33	2 9	38 59	7
mmu-miR-214-3p	XM_017322211.1	157.00	-22.14	2 20	4628 4648	18
mmu-miR-214-3p	XM_006530238.3	150.00	-20.06	2 19	5248 5269	17
mmu-miR-214-3p	XM_006530238.3	143.00	-19.32	2 19	7099 7118	17
mmu-miR-214-3p	XM_006530238.3	142.00	-22.79	2 21	8564 8584	19
mmu-miR-214-3p	XM_017316715.1	167.00	-24.60	2 21	3071 3093	20
mmu-miR-214-3p	XM_017316715.1	160.00	-20.79	2 21	3757 3778	19
mmu-miR-214-3p	XM_017316715.1	158.00	-27.30	2 21	4160 4180	19
mmu-miR-214-3p	XM_017316715.1	151.00	-22.29	2 14	4147 4167	12
mmu-miR-214-3p	XM_017316715.1	145.00	-17.35	2 10	537 558	8
mmu-miR-214-3p	XM_017316715.1	144.00	-15.51	2 20	741 762	19
mmu-miR-214-3p	XM_017316715.1	140.00	-16.88	2 9	2362 2383	7
mmu-miR-214-3p	XM_017317034.1	157.00	-23.47	2 19	2962 2984	18
mmu-miR-214-3p	XM_017317034.1	155.00	-17.46	2 20	2132 2153	18
mmu-miR-214-3p	XM_017317034.1	145.00	-18.80	2 15	2773 2795	14
mmu-miR-214-3p	XM_017317034.1	145.00	-18.48	2 15	3478 3502	16
mmu-miR-214-3p	XM_017317034.1	144.00	-16.69	2 21	4021 4042	19
mmu-miR-214-3p	XM_011250732.2	140.00	-15.35	2 9	4028 4049	7
mmu-miR-214-3p	NM_177578.4	141.00	-14.95	3 20	1625 1645	17
mmu-miR-214-3p	XM_006518339.3	147.00	-17.60	2 20	439 460	18
mmu-miR-214-3p	XM_006496884.3	142.00	-19.88	2 19	2497 2518	17
mmu-miR-214-3p	NM_144539.5	144.00	-21.29	2 19	1157 1180	19
mmu-miR-214-3p	XM_017313038.1	150.00	-25.78	2 20	104 131	24
mmu-miR-214-3p	NM_009199.2	155.00	-29.00	3 21	3166 3188	19
mmu-miR-214-3p	NM_001033167.3	154.00	-18.26	2 15	2194 2215	13
mmu-miR-214-3p	NM_001033167.3	143.00	-23.44	2 21	4827 4850	21
mmu-miR-214-3p	NM_001033167.3	142.00	-18.13	3 19	1415 1436	16
mmu-miR-214-3p	NM_001007570.2	156.00	-22.45	2 19	1300 1325	21
mmu-miR-214-3p	NM_001007570.2	148.00	-22.69	3 19	2042 2062	16
mmu-miR-214-3p	NM_001007570.2	146.00	-18.68	2 17	178 201	17
mmu-miR-214-3p	NM_001007570.2	144.00	-16.75	2 19	1006 1026	17
mmu-miR-214-3p	NM_001033286.2	146.00	-15.97	2 16	2376 2398	15
mmu-miR-214-3p	NM_001033286.2	145.00	-15.94	2 10	1744 1765	8
mmu-miR-214-3p	NM_001290993.1	142.00	-18.40	2 11	265 286	9
mmu-miR-214-3p	XM_006503080.3	170.00	-30.96	2 21	4225 4245	19
mmu-miR-214-3p	XM_006503080.3	160.00	-23.62	2 21	1253 1274	19
mmu-miR-214-3p	NM_027052.3	147.00	-22.34	3 21	860 882	19
mmu-miR-214-3p	XM_006538687.2	154.00	-16.90	2 19	1484 1505	17
mmu-miR-214-3p	XM_006538687.2	151.00	-19.15	2 21	1390 1412	20
mmu-miR-214-3p	XM_006538687.2	149.00	-24.26	3 20	1 20	17
mmu-miR-214-3p	XM_006538687.2	147.00	-18.43	2 14	1662 1682	12
mmu-miR-214-3p	NM_025540.2	146.00	-23.85	3 20	46 69	19
mmu-miR-214-3p	NM_001146217.1	145.00	-15.85	2 10	1249 1270	8
mmu-miR-214-3p	NM_001146217.1	140.00	-16.15	2 9	1 19	7
mmu-miR-214-3p	XM_006500109.3	144.00	-15.00	2 21	2228 2249	19
mmu-miR-214-3p	NM_011430.3	141.00	-16.54	2 20	617 637	18
mmu-miR-214-3p	NM_175692.3	153.00	-26.29	2 19	5767 5790	19
mmu-miR-214-3p	NM_175692.3	148.00	-18.31	2 21	4999 5020	19
mmu-miR-214-3p	NM_009223.3	140.00	-16.00	2 9	16 37	7
mmu-miR-214-3p	XM_006534520.3	159.00	-23.65	2 21	763 785	20
mmu-miR-214-3p	XM_006534520.3	151.00	-22.44	2 21	1944 1967	21
mmu-miR-214-3p	XM_006534520.3	146.00	-18.85	2 19	812 833	17
mmu-miR-214-3p	XM_006534520.3	140.00	-16.78	2 9	1546 1567	7
mmu-miR-214-3p	NM_031183.2	150.00	-24.18	3 21	3190 3210	18
mmu-miR-214-3p	XM_006539074.3	159.00	-20.65	2 20	8843 8864	18
mmu-miR-214-3p	XM_006539074.3	152.00	-20.58	2 18	9031 9053	17
mmu-miR-214-3p	XM_006539074.3	149.00	-17.50	2 14	5289 5310	12
mmu-miR-214-3p	XM_006539074.3	147.00	-17.23	2 20	7587 7608	18
mmu-miR-214-3p	XM_006539074.3	146.00	-22.27	2 20	3629 3651	19
mmu-miR-214-3p	NM_019866.1	150.00	-22.61	2 20	539 562	20
mmu-miR-214-3p	NM_178753.4	145.00	-16.98	2 19	969 993	20
mmu-miR-214-3p	NM_001304266.1	153.00	-21.42	2 19	8081 8104	19
mmu-miR-214-3p	NM_001304266.1	152.00	-19.09	2 19	8117 8137	17

mmu-miR-214-3p	NM_001304266.1	151.00	-20.79	2 21	9318 9340	20
mmu-miR-214-3p	NM_001304266.1	143.00	-18.18	2 20	2119 2140	18
mmu-miR-214-3p	NM_001304266.1	142.00	-15.69	2 11	7456 7477	9
mmu-miR-214-3p	NM_001242411.1	148.00	-24.77	2 18	3967 3990	18
mmu-miR-214-3p	NM_001242411.1	145.00	-19.50	2 10	3214 3235	8
mmu-miR-214-3p	NM_001242411.1	143.00	-14.69	2 16	2346 2367	14
mmu-miR-214-3p	XM_006506145.1	166.00	-25.54	2 19	8922 8943	17
mmu-miR-214-3p	XM_006506145.1	152.00	-18.94	2 21	6219 6240	19
mmu-miR-214-3p	XM_006506145.1	145.00	-18.83	2 19	1661 1684	19
mmu-miR-214-3p	XM_006506145.1	143.00	-23.42	2 21	8281 8304	21
mmu-miR-214-3p	XM_011248193.2	151.00	-16.28	2 20	7026 7047	18
mmu-miR-214-3p	XM_011248193.2	148.00	-21.69	2 20	7271 7290	18
mmu-miR-214-3p	XM_011248193.2	146.00	-21.94	2 21	5137 5161	22
mmu-miR-214-3p	XM_011248193.2	143.00	-18.07	2 20	4218 4239	18
mmu-miR-214-3p	XM_011248193.2	142.00	-26.71	2 21	8133 8160	25
mmu-miR-214-3p	XM_011245529.2	162.00	-24.15	2 19	5360 5381	17
mmu-miR-214-3p	XM_011245529.2	149.00	-16.73	2 20	1002 1022	18
mmu-miR-214-3p	XM_011245529.2	146.00	-14.87	2 20	1356 1378	19
mmu-miR-214-3p	XM_011245529.2	143.00	-23.50	2 21	267 290	21
mmu-miR-214-3p	XM_011245529.2	141.00	-24.45	2 19	4064 4086	18
mmu-miR-214-3p	XM_011245529.2	140.00	-14.45	2 9	5517 5538	7
mmu-miR-214-3p	NM_011374.2	142.00	-13.64	2 11	2248 2269	9
mmu-miR-214-3p	NM_011374.2	140.00	-15.69	2 9	1261 1282	7
mmu-miR-214-3p	XM_017316056.1	144.00	-26.87	2 21	656 677	19
mmu-miR-214-3p	XM_017316056.1	141.00	-15.53	2 14	773 794	12
mmu-miR-214-3p	NM_009332.3	147.00	-20.56	2 20	2566 2587	18
mmu-miR-214-3p	NM_001080979.1	145.00	-25.97	2 20	2566 2591	22
mmu-miR-214-3p	NM_001080979.1	144.00	-16.18	2 19	2088 2108	17
mmu-miR-214-3p	NM_001080979.1	142.00	-32.30	4 21	2184 2204	17
mmu-miR-214-3p	NM_011581.3	165.00	-22.80	2 19	4778 4800	18
mmu-miR-214-3p	NM_011581.3	150.00	-24.21	2 21	390 415	23
mmu-miR-214-3p	NM_011581.3	145.00	-17.41	2 10	4096 4117	8
mmu-miR-214-3p	NM_009379.3	174.00	-32.11	2 21	562 585	21
mmu-miR-214-3p	NM_009381.3	150.00	-18.14	2 11	69 90	9
mmu-miR-214-3p	XM_006501237.3	140.00	-12.69	2 17	829 850	15
mmu-miR-214-3p	XM_006533479.2	161.00	-23.56	2 19	815 837	18
mmu-miR-214-3p	XM_006533452.3	162.00	-21.14	2 15	3618 3639	13
mmu-miR-214-3p	XM_006533452.3	161.00	-22.78	2 18	3412 3433	16
mmu-miR-214-3p	XM_006533452.3	140.00	-15.73	2 9	2784 2805	7
mmu-miR-214-3p	NM_144936.1	142.00	-14.42	2 11	528 549	9
mmu-miR-214-3p	XM_006521428.3	147.00	-21.17	2 20	1659 1677	18
mmu-miR-214-3p	XM_006538830.1	157.00	-19.18	2 19	473 495	18
mmu-miR-214-3p	XM_006538830.1	142.00	-18.47	2 21	256 276	19
mmu-miR-214-3p	XM_006538830.1	142.00	-21.68	2 19	1267 1288	17
mmu-miR-214-3p	NM_177371.3	167.00	-27.76	2 21	196 218	20
mmu-miR-214-3p	NM_177371.3	155.00	-18.05	2 21	1039 1061	20
mmu-miR-214-3p	XM_006539726.3	151.00	-25.00	2 21	839 861	20
mmu-miR-214-3p	XM_011248201.2	149.00	-20.24	2 14	685 706	12
mmu-miR-214-3p	XM_011248201.2	149.00	-21.44	2 20	3721 3741	18
mmu-miR-214-3p	XM_011248201.2	140.00	-16.55	2 9	3611 3632	7
mmu-miR-214-3p	NM_001039047.1	150.00	-23.61	3 20	1508 1530	18
mmu-miR-214-3p	XM_006508002.3	158.00	-26.32	2 21	515 539	22
mmu-miR-214-3p	XM_006508002.3	149.00	-27.43	2 18	800 821	16
mmu-miR-214-3p	XM_006508002.3	147.00	-21.06	2 12	772 793	10
mmu-miR-214-3p	XM_006508002.3	144.00	-17.37	2 21	2040 2058	19
mmu-miR-214-3p	XM_006508002.3	140.00	-19.56	2 9	562 583	7
mmu-miR-214-3p	XM_017321602.1	147.00	-27.79	2 21	112 135	21
mmu-miR-214-3p	XM_017321602.1	147.00	-21.41	2 20	770 791	18
mmu-miR-214-3p	XM_017321602.1	145.00	-21.82	2 19	439 461	18
mmu-miR-214-3p	XM_006506152.3	156.00	-23.13	2 19	1994 2014	17
mmu-miR-214-3p	XM_006506152.3	144.00	-17.09	2 21	3252 3270	19
mmu-miR-214-3p	XM_006506152.3	142.00	-21.75	2 19	2246 2267	17
mmu-miR-214-3p	XM_006506152.3	141.00	-13.10	2 14	634 655	12
mmu-miR-214-3p	NM_001033149.3	146.00	-22.75	3 20	542 565	19
mmu-miR-214-3p	NM_001033149.3	143.00	-21.89	2 21	916 938	20

mmu-miR-214-3p	NM_177709.3	154.00	-26.13	3 21	2109 2129	18
mmu-miR-214-3p	XM_006501309.3	152.00	-21.02	2 20	504 525	19
mmu-miR-214-3p	XM_006501309.3	140.00	-15.79	2 9	680 701	7
mmu-miR-214-3p	XM_011248394.1	140.00	-15.04	2 9	190 211	7
mmu-miR-214-3p	NM_009504.4	140.00	-14.48	2 9	4063 4084	7
mmu-miR-214-3p	NM_016982.2	149.00	-17.98	2 19	330 352	18
mmu-miR-214-3p	NM_016982.2	142.00	-23.46	3 21	149 169	18
mmu-miR-214-3p	XM_017316944.1	149.00	-17.98	2 19	670 692	18
mmu-miR-214-3p	XM_017316944.1	142.00	-23.46	3 21	402 422	18
mmu-miR-214-3p	NM_177789.4	168.00	-22.77	2 21	892 913	19
mmu-miR-214-3p	NM_009518.2	151.00	-22.62	2 21	961 983	20
mmu-miR-214-3p	XM_006533569.3	161.00	-27.79	2 19	766 789	19
mmu-miR-214-3p	XM_006499466.3	153.00	-20.92	2 20	2691 2711	18
mmu-miR-214-3p	XM_006499466.3	141.00	-22.40	2 20	1289 1313	21
mmu-miR-214-3p	XM_006530585.3	159.00	-17.51	2 20	4028 4049	18
mmu-miR-214-3p	XM_006530585.3	148.00	-19.78	3 21	10878 10899	18
mmu-miR-214-3p	XM_006530585.3	145.00	-19.41	2 10	5596 5617	8
mmu-miR-214-3p	XM_006530585.3	140.00	-13.10	2 9	4798 4819	7
mmu-miR-214-3p	NM_199029.2	159.00	-23.88	2 21	3367 3389	20
mmu-miR-214-3p	NM_199029.2	149.00	-25.85	3 21	2707 2726	18
mmu-miR-214-3p	XM_006511007.3	152.00	-24.00	2 21	3241 3262	19
mmu-miR-214-3p	XM_006511007.3	142.00	-14.41	2 11	4519 4540	9
mmu-miR-214-3p	XM_006537508.2	155.00	-27.95	2 21	6825 6849	22
mmu-miR-214-3p	XM_006537508.2	148.00	-18.10	2 20	1539 1560	19
mmu-miR-214-3p	XM_006537508.2	146.00	-17.23	2 16	397 420	16
mmu-miR-214-3p	XM_006519134.3	161.00	-24.01	2 18	2938 2959	16
mmu-miR-214-3p	XM_006519134.3	157.00	-25.68	2 16	1188 1211	16
mmu-miR-214-3p	XM_006519134.3	147.00	-20.14	2 19	1225 1244	17
mmu-miR-214-3p	XM_006519134.3	146.00	-22.44	3 20	5260 5282	18
mmu-miR-214-3p	XM_006519134.3	140.00	-17.27	2 9	1725 1746	7
mmu-miR-214-3p	XM_006519134.3	140.00	-17.09	2 9	5242 5263	7
mmu-miR-378a-5p	NM_172454.2	149.00	-23.46	2 21	2049 2075	24
mmu-miR-378a-5p	NM_008804.4	141.00	-24.29	2 20	706 731	22
mmu-miR-378a-5p	NM_013743.2	148.00	-20.54	2 20	99 118	18
mmu-miR-378a-5p	XM_006509465.2	143.00	-11.66	2 13	1645 1667	12
mmu-miR-378a-5p	XM_006538456.3	148.00	-28.83	2 21	733 751	19
mmu-miR-378a-5p	XM_006538456.3	145.00	-14.19	2 10	1703 1724	8
mmu-miR-378a-5p	XM_006538456.3	141.00	-12.42	2 14	1984 2005	12
mmu-miR-378a-5p	XM_006537815.3	164.00	-25.05	2 21	1922 1943	19
mmu-miR-378a-5p	NM_153104.3	143.00	-25.65	2 21	1124 1149	23
mmu-miR-378a-5p	XM_017312497.1	151.00	-22.52	2 21	3836 3858	20
mmu-miR-378a-5p	XM_006502235.3	140.00	-23.90	2 21	6111 6132	19
mmu-miR-378a-5p	NM_001195084.1	146.00	-15.41	2 15	206 227	13
mmu-miR-378a-5p	XM_006512076.3	151.00	-30.47	2 21	3271 3294	21
mmu-miR-378a-5p	XM_006512076.3	147.00	-24.59	3 21	8286 8309	20
mmu-miR-378a-5p	NM_175022.2	178.00	-36.63	2 21	5983 6003	19
mmu-miR-378a-5p	NM_175022.2	148.00	-20.03	2 21	3793 3814	19
mmu-miR-378a-5p	NM_175022.2	144.00	-24.36	2 18	5872 5896	19
mmu-miR-378a-5p	NM_001081224.2	147.00	-15.85	2 12	1990 2011	10
mmu-miR-378a-5p	XM_011240476.2	167.00	-37.59	2 21	2500 2523	21
mmu-miR-378a-5p	XM_006514153.2	152.00	-22.50	2 19	460 480	17
mmu-miR-378a-5p	XM_017320735.1	159.00	-20.20	2 20	1743 1764	18
mmu-miR-378a-5p	NM_011254.5	169.00	-31.42	2 18	2474 2495	16
mmu-miR-378a-5p	XM_006529829.3	140.00	-18.47	2 9	5333 5354	7
mmu-miR-378a-5p	XM_006507942.2	142.00	-24.69	2 19	852 873	17
mmu-miR-378a-5p	XM_006522506.3	142.00	-29.23	3 21	123 149	23
mmu-miR-378a-5p	XM_006525075.3	147.00	-16.90	2 16	621 642	14
mmu-miR-378a-5p	XM_006525075.3	146.00	-30.72	2 21	272 292	19
mmu-miR-378a-5p	NM_009127.4	154.00	-25.27	2 15	168 189	13
mmu-miR-378a-5p	NM_018732.3	160.00	-16.01	2 21	2157 2178	19
mmu-miR-378a-5p	NM_020052.2	151.00	-17.89	2 16	1516 1537	14
mmu-miR-378a-5p	NM_020052.2	145.00	-17.41	2 15	2013 2035	14
mmu-miR-378a-5p	NM_020052.2	140.00	-14.20	2 9	1616 1637	7
mmu-miR-378a-5p	XM_017322211.1	143.00	-22.54	2 19	5007 5028	18
mmu-miR-378a-5p	XM_017322211.1	140.00	-18.80	2 17	3933 3954	15

mmu-miR-378a-5p	XM_006530238.3	140.00	-19.60	2 9	2610 2631	7
mmu-miR-378a-5p	XM_006530238.3	140.00	-24.68	2 21	4420 4440	20
mmu-miR-378a-5p	XM_017316715.1	155.00	-16.69	2 20	3376 3397	18
mmu-miR-378a-5p	XM_017316715.1	144.00	-28.93	2 21	4447 4468	19
mmu-miR-378a-5p	XM_011250732.2	155.00	-29.80	2 20	3418 3436	18
mmu-miR-378a-5p	NM_177578.4	148.00	-24.32	2 21	1824 1844	20
mmu-miR-378a-5p	NM_144539.5	156.00	-22.31	3 21	765 786	18
mmu-miR-378a-5p	NM_009199.2	148.00	-22.05	3 21	2559 2580	18
mmu-miR-378a-5p	XM_006524777.3	152.00	-23.98	3 21	571 592	18
mmu-miR-378a-5p	NM_009223.3	140.00	-22.64	3 19	2612 2632	16
mmu-miR-378a-5p	XM_006534520.3	147.00	-19.17	2 12	145 166	10
mmu-miR-378a-5p	XM_006539074.3	148.00	-16.59	2 16	2193 2212	14
mmu-miR-378a-5p	XM_006539074.3	141.00	-23.52	2 21	7198 7217	19
mmu-miR-378a-5p	NM_001304266.1	143.00	-25.11	2 16	8957 8978	14
mmu-miR-378a-5p	NM_001304266.1	142.00	-13.85	2 21	5530 5553	21
mmu-miR-378a-5p	NM_001242411.1	148.00	-15.09	2 15	5422 5442	13
mmu-miR-378a-5p	XM_006506145.1	148.00	-21.34	2 21	1516 1537	19
mmu-miR-378a-5p	XM_006506145.1	146.00	-25.81	2 20	2403 2425	19
mmu-miR-378a-5p	XM_011248193.2	150.00	-17.09	2 11	5966 5987	9
mmu-miR-378a-5p	NM_011374.2	143.00	-23.92	2 21	7063 7086	21
mmu-miR-378a-5p	NM_011374.2	140.00	-15.45	2 9	5443 5464	7
mmu-miR-378a-5p	XM_006527452.3	153.00	-19.20	2 20	1382 1402	18
mmu-miR-378a-5p	XM_006527452.3	145.00	-16.39	2 18	632 653	16
mmu-miR-378a-5p	NM_011581.3	150.00	-21.74	2 20	5580 5604	21
mmu-miR-378a-5p	XM_006533452.3	154.00	-28.34	2 19	2943 2964	17
mmu-miR-378a-5p	XM_006515787.3	145.00	-14.11	2 10	986 1007	8
mmu-miR-378a-5p	NM_177371.3	155.00	-25.57	2 17	500 522	16
mmu-miR-378a-5p	NM_177371.3	142.00	-24.81	2 21	4998 5021	21
mmu-miR-378a-5p	XM_011248201.2	162.00	-29.19	2 19	318 339	17
mmu-miR-378a-5p	XM_011248201.2	151.00	-25.23	2 16	3898 3919	14
mmu-miR-378a-5p	NM_001243916.1	145.00	-25.61	2 21	478 500	21
mmu-miR-378a-5p	XM_006508002.3	151.00	-22.94	2 21	663 686	21
mmu-miR-378a-5p	NM_177709.3	152.00	-25.19	2 17	2567 2588	15
mmu-miR-378a-5p	XM_006525099.3	142.00	-21.56	2 15	3019 3040	13
mmu-miR-378a-5p	XM_011248394.1	157.00	-17.34	2 20	3313 3333	18
mmu-miR-378a-5p	NM_009504.4	143.00	-13.87	2 21	2320 2344	22
mmu-miR-378a-5p	NM_009504.4	140.00	-27.95	2 17	1147 1169	16
mmu-miR-378a-5p	XM_006525028.2	163.00	-31.66	2 21	940 962	20
mmu-miR-378a-5p	NM_011724.3	156.00	-20.09	2 21	4814 4833	19
mmu-miR-378a-5p	NM_011724.3	140.00	-26.34	3 20	508 530	19
mmu-miR-378a-5p	NM_199029.2	155.00	-24.65	2 17	350 372	16
mmu-miR-378a-5p	NM_199029.2	141.00	-25.22	3 21	2608 2633	22
mmu-miR-378a-5p	XM_006511007.3	153.00	-29.41	2 21	2680 2703	22
mmu-miR-378a-5p	XM_006511007.3	152.00	-27.15	2 21	3020 3041	19
mmu-miR-378a-5p	XM_006537508.2	155.00	-15.39	2 21	3791 3813	20
mmu-miR-378a-5p	XM_006519134.3	140.00	-24.09	3 21	1444 1465	18
mmu-miR-494-3p	NM_011044.2	142.00	-10.61	3 20	2503 2526	19
mmu-miR-494-3p	NM_013743.2	153.00	-11.29	2 21	3103 3124	20
mmu-miR-494-3p	XM_006509465.2	140.00	-6.21	2 9	1471 1492	7
mmu-miR-494-3p	XM_006538456.3	144.00	-15.61	2 19	454 474	17
mmu-miR-494-3p	XM_006537815.3	142.00	-19.98	2 16	4658 4680	15
mmu-miR-494-3p	NM_011082.3	140.00	-7.49	2 9	3073 3094	7
mmu-miR-494-3p	NM_018807.5	141.00	-15.81	2 19	494 519	21
mmu-miR-494-3p	XM_017312497.1	148.00	-16.21	2 21	2381 2402	19
mmu-miR-494-3p	NM_001195084.1	144.00	-16.46	2 13	1618 1639	11
mmu-miR-494-3p	NM_001291068.1	147.00	-9.61	2 16	2089 2110	14
mmu-miR-494-3p	XM_006525698.2	163.00	-14.54	2 18	8770 8790	16
mmu-miR-494-3p	NM_011169.5	143.00	-16.64	2 16	6261 6282	14
mmu-miR-494-3p	NM_008966.3	145.00	-6.82	2 19	2594 2618	20
mmu-miR-494-3p	NM_008966.3	140.00	-14.54	2 21	2563 2584	19
mmu-miR-494-3p	NM_027455.3	140.00	-9.48	3 18	1434 1456	16
mmu-miR-494-3p	NM_009019.2	140.00	-11.45	2 18	6532 6556	19
mmu-miR-494-3p	NM_009020.3	152.00	-10.39	2 21	817 838	19
mmu-miR-494-3p	NM_198024.2	157.00	-14.96	2 19	3420 3442	18
mmu-miR-494-3p	XM_011240779.2	142.00	-7.34	2 18	4583 4607	19

mmu-miR-494-3p	NM_001195662.1	146.00	-6.71	2 19	987 1008	17
mmu-miR-494-3p	NM_001195662.1	140.00	-12.25	2 9	1015 1036	7
mmu-miR-494-3p	XM_006522506.3	142.00	-15.81	2 15	471 492	13
mmu-miR-494-3p	NM_018732.3	151.00	-13.79	2 18	3957 3982	20
mmu-miR-494-3p	NM_009135.2	155.00	-16.29	2 18	1241 1261	16
mmu-miR-494-3p	NM_009135.2	143.00	-12.80	2 13	7238 7260	12
mmu-miR-494-3p	NM_009135.2	140.00	-7.25	2 9	5476 5497	7
mmu-miR-494-3p	NM_020052.2	159.00	-21.92	2 21	2917 2939	20
mmu-miR-494-3p	NM_008458.2	159.00	-19.08	2 16	1558 1579	14
mmu-miR-494-3p	XM_006515637.2	159.00	-19.08	2 16	1293 1314	14
mmu-miR-494-3p	XM_006530238.3	141.00	-7.56	2 19	8541 8563	18
mmu-miR-494-3p	XM_017316715.1	147.00	-9.31	2 12	4919 4940	10
mmu-miR-494-3p	XM_011250732.2	157.00	-18.03	2 20	8031 8051	18
mmu-miR-494-3p	NM_144539.5	153.00	-13.17	2 14	3123 3144	12
mmu-miR-494-3p	NM_009199.2	147.00	-13.39	2 12	1480 1501	10
mmu-miR-494-3p	NM_001033286.2	148.00	-16.18	2 21	4313 4334	19
mmu-miR-494-3p	NM_001033286.2	145.00	-9.72	2 10	2083 2104	8
mmu-miR-494-3p	NM_001290993.1	141.00	-8.50	2 12	2927 2947	10
mmu-miR-494-3p	XM_006503080.3	142.00	-8.76	2 19	4371 4392	17
mmu-miR-494-3p	NM_009223.3	143.00	-11.73	2 21	750 774	22
mmu-miR-494-3p	NM_031183.2	142.00	-12.51	2 20	2604 2626	19
mmu-miR-494-3p	XM_006539074.3	144.00	-15.33	3 21	9489 9510	18
mmu-miR-494-3p	NM_178753.4	144.00	-12.45	2 19	1582 1602	17
mmu-miR-494-3p	NM_001242411.1	144.00	-21.74	2 13	6046 6067	11
mmu-miR-494-3p	NM_001242411.1	143.00	-16.48	3 21	6866 6888	19
mmu-miR-494-3p	NM_011374.2	154.00	-11.62	2 20	7675 7697	19
mmu-miR-494-3p	NM_011374.2	146.00	-17.69	2 20	1924 1946	19
mmu-miR-494-3p	XM_006527452.3	144.00	-10.18	2 19	2162 2182	17
mmu-miR-494-3p	XM_006527452.3	140.00	-11.19	2 9	856 877	7
mmu-miR-494-3p	NM_153801.3	140.00	-8.90	2 9	1729 1750	7
mmu-miR-494-3p	XM_006537836.1	143.00	-10.77	2 20	1643 1664	18
mmu-miR-494-3p	XM_006533452.3	157.00	-16.50	2 18	2042 2063	16
mmu-miR-494-3p	XM_011249157.2	148.00	-11.99	2 17	3530 3549	15
mmu-miR-494-3p	XM_011249157.2	140.00	-6.85	2 9	2451 2472	7
mmu-miR-494-3p	NM_001243916.1	142.00	-12.71	2 21	1081 1107	24
mmu-miR-494-3p	XM_006501309.3	161.00	-17.25	2 14	1959 1980	12
mmu-miR-494-3p	XM_006501309.3	140.00	-6.32	2 9	3420 3441	7
mmu-miR-494-3p	NM_009504.4	149.00	-14.79	3 18	2951 2972	15
mmu-miR-494-3p	NM_009504.4	149.00	-14.60	2 20	3096 3122	23
mmu-miR-494-3p	XM_006525028.2	140.00	-11.77	2 9	2689 2710	7
mmu-miR-494-3p	NM_011724.3	140.00	-13.98	3 21	1710 1731	18
mmu-miR-494-3p	XM_017318541.1	146.00	-7.91	2 19	982 1003	17
mmu-miR-494-3p	XM_006530585.3	150.00	-13.48	2 19	13450 13471	17
mmu-miR-494-3p	XM_006530585.3	147.00	-10.12	2 12	11860 11881	10
mmu-miR-494-3p	NM_199029.2	148.00	-18.01	2 15	718 738	13
mmu-miR-494-3p	XM_011243586.2	144.00	-15.62	2 13	4226 4247	11
mmu-miR-494-3p	XM_006511007.3	143.00	-13.06	2 16	4877 4898	14
mmu-miR-494-3p	NM_001110508.1	140.00	-12.97	2 18	2233 2256	18
mmu-miR-494-3p	XM_006537508.2	148.00	-10.25	2 17	5565 5586	15
mmu-miR-494-3p	XM_006537508.2	142.00	-14.19	2 20	6517 6539	19
mmu-miR-494-3p	XM_006537508.2	140.00	-7.26	2 9	3669 3690	7
mmu-miR-135a-1-3p	NM_172454.2	140.00	-20.13	3 20	485 504	17
mmu-miR-135a-1-3p	NM_028748.2	140.00	-23.99	2 21	53 74	19
mmu-miR-135a-1-3p	NM_133167.3	147.00	-24.03	2 21	1235 1257	20
mmu-miR-135a-1-3p	NM_133167.3	143.00	-24.03	2 21	2923 2949	24
mmu-miR-135a-1-3p	NM_011044.2	149.00	-24.92	3 21	1799 1820	19
mmu-miR-135a-1-3p	NM_013743.2	140.00	-15.32	2 9	3279 3300	7
mmu-miR-135a-1-3p	XM_006509465.2	146.00	-20.91	2 21	1429 1452	21
mmu-miR-135a-1-3p	NM_001290822.1	161.00	-26.67	2 21	1115 1134	19
mmu-miR-135a-1-3p	NM_001290822.1	143.00	-25.04	3 21	1345 1367	19
mmu-miR-135a-1-3p	NM_001164593.1	145.00	-13.95	2 14	3442 3463	12
mmu-miR-135a-1-3p	XM_006537815.3	143.00	-20.01	2 16	4815 4836	14
mmu-miR-135a-1-3p	NM_153104.3	146.00	-21.71	2 20	1235 1261	23
mmu-miR-135a-1-3p	NM_011082.3	153.00	-23.82	2 21	1278 1297	19
mmu-miR-135a-1-3p	NM_011082.3	142.00	-19.70	2 21	2839 2859	19

mmu-miR-135a-1-3p	XM_017314870.1	144.00	-19.50	3 21	1428 1449	18
mmu-miR-135a-1-3p	XM_017314870.1	144.00	-17.36	2 19	4334 4360	22
mmu-miR-135a-1-3p	XM_017314870.1	141.00	-21.11	2 20	6886 6909	20
mmu-miR-135a-1-3p	NM_018807.5	143.00	-17.38	2 13	3871 3893	12
mmu-miR-135a-1-3p	XM_011250776.2	148.00	-18.41	2 19	1334 1354	17
mmu-miR-135a-1-3p	XM_006502235.3	142.00	-18.33	2 19	2108 2129	17
mmu-miR-135a-1-3p	NM_001291068.1	152.00	-17.90	2 21	280 301	19
mmu-miR-135a-1-3p	NM_148932.2	149.00	-18.85	2 21	4072 4091	19
mmu-miR-135a-1-3p	NM_011145.3	140.00	-17.14	2 21	1987 2008	19
mmu-miR-135a-1-3p	XM_006525698.2	150.00	-20.15	2 21	11128 11152	22
mmu-miR-135a-1-3p	XM_006527008.2	147.00	-24.50	3 21	3017 3039	19
mmu-miR-135a-1-3p	NM_011169.5	147.00	-29.08	2 21	970 992	20
mmu-miR-135a-1-3p	NM_175022.2	171.00	-24.92	2 21	2931 2953	20
mmu-miR-135a-1-3p	XM_006524382.3	145.00	-25.87	2 21	1092 1118	24
mmu-miR-135a-1-3p	XM_006505015.3	140.00	-20.19	2 20	3990 4012	20
mmu-miR-135a-1-3p	NM_027455.3	142.00	-15.59	2 11	688 709	9
mmu-miR-135a-1-3p	XM_006514153.2	141.00	-20.83	3 19	2932 2954	17
mmu-miR-135a-1-3p	NM_009019.2	140.00	-22.09	2 19	2401 2421	17
mmu-miR-135a-1-3p	NM_016809.6	142.00	-17.54	3 19	1483 1504	16
mmu-miR-135a-1-3p	NM_011254.5	147.00	-25.02	3 21	25 47	19
mmu-miR-135a-1-3p	NM_001204959.1	150.00	-19.36	2 20	193 215	19
mmu-miR-135a-1-3p	XM_006529829.3	145.00	-24.58	2 19	5278 5300	18
mmu-miR-135a-1-3p	XM_006507942.2	159.00	-25.98	2 20	2125 2146	18
mmu-miR-135a-1-3p	XM_006507942.2	143.00	-20.74	2 21	1143 1161	19
mmu-miR-135a-1-3p	XM_006507942.2	140.00	-15.62	2 9	1191 1212	7
mmu-miR-135a-1-3p	NM_019732.2	140.00	-18.94	3 19	1239 1259	16
mmu-miR-135a-1-3p	NM_009127.4	157.00	-23.18	2 21	3436 3455	19
mmu-miR-135a-1-3p	NM_009127.4	150.00	-27.35	2 20	1709 1731	19
mmu-miR-135a-1-3p	NM_009127.4	141.00	-17.31	2 21	686 705	19
mmu-miR-135a-1-3p	NM_018732.3	151.00	-20.46	2 21	2560 2582	20
mmu-miR-135a-1-3p	NM_018732.3	143.00	-29.87	4 21	5764 5786	18
mmu-miR-135a-1-3p	NM_018732.3	143.00	-23.89	2 21	6022 6044	20
mmu-miR-135a-1-3p	NM_009135.2	157.00	-19.36	2 15	359 381	14
mmu-miR-135a-1-3p	NM_020052.2	159.00	-28.34	2 17	2732 2755	17
mmu-miR-135a-1-3p	NM_020052.2	146.00	-20.29	2 21	1256 1276	19
mmu-miR-135a-1-3p	XM_011244046.2	142.00	-21.44	2 21	1609 1629	19
mmu-miR-135a-1-3p	XM_006521796.1	142.00	-18.92	3 21	1781 1801	18
mmu-miR-135a-1-3p	XM_017322211.1	140.00	-17.84	2 21	2385 2406	19
mmu-miR-135a-1-3p	XM_006530238.3	145.00	-24.32	2 20	6296 6319	20
mmu-miR-135a-1-3p	XM_006530238.3	145.00	-27.89	2 18	7340 7361	16
mmu-miR-135a-1-3p	XM_006530238.3	140.00	-23.46	3 21	2431 2452	18
mmu-miR-135a-1-3p	XM_011250732.2	148.00	-19.27	2 21	8008 8029	19
mmu-miR-135a-1-3p	XM_011250732.2	143.00	-25.15	3 21	3290 3313	20
mmu-miR-135a-1-3p	NM_144539.5	143.00	-13.59	2 18	1950 1970	16
mmu-miR-135a-1-3p	XM_017313038.1	140.00	-20.08	2 20	670 689	18
mmu-miR-135a-1-3p	NM_001310705.1	148.00	-20.84	2 21	1390 1411	19
mmu-miR-135a-1-3p	NM_001007570.2	156.00	-21.57	2 17	666 687	15
mmu-miR-135a-1-3p	XM_006538687.2	142.00	-20.32	2 21	2800 2824	22
mmu-miR-135a-1-3p	XM_006538687.2	142.00	-20.64	3 21	4521 4544	20
mmu-miR-135a-1-3p	NM_025540.2	140.00	-19.59	3 21	437 458	18
mmu-miR-135a-1-3p	XM_006534520.3	155.00	-21.06	2 21	902 924	20
mmu-miR-135a-1-3p	XM_006534520.3	141.00	-21.51	2 15	3084 3106	14
mmu-miR-135a-1-3p	XM_006539074.3	147.00	-17.04	2 12	2510 2531	10
mmu-miR-135a-1-3p	NM_019866.1	152.00	-20.41	2 21	175 196	19
mmu-miR-135a-1-3p	NM_178753.4	140.00	-15.77	2 13	2006 2027	11
mmu-miR-135a-1-3p	NM_001304266.1	160.00	-32.86	2 21	4955 4976	19
mmu-miR-135a-1-3p	NM_001304266.1	143.00	-31.11	2 20	6005 6026	18
mmu-miR-135a-1-3p	NM_001304266.1	140.00	-28.31	2 21	5477 5498	19
mmu-miR-135a-1-3p	NM_001242411.1	140.00	-18.46	2 21	1632 1653	19
mmu-miR-135a-1-3p	XM_011248193.2	160.00	-21.31	2 21	7594 7615	19
mmu-miR-135a-1-3p	XM_011245529.2	144.00	-21.97	2 21	4422 4443	19
mmu-miR-135a-1-3p	XM_011245529.2	140.00	-21.74	3 21	4138 4159	18
mmu-miR-135a-1-3p	NM_011374.2	148.00	-21.12	2 15	7541 7561	13
mmu-miR-135a-1-3p	NM_001080979.1	141.00	-27.43	2 21	2424 2450	24
mmu-miR-135a-1-3p	NM_009381.3	141.00	-21.14	2 19	195 219	20

mmu-miR-135a-1-3p	XM_006501237.3	148.00	-22.81	3 21	194 215	18
mmu-miR-135a-1-3p	XM_006515787.3	155.00	-28.41	2 21	538 561	21
mmu-miR-135a-1-3p	NM_178715.3	142.00	-16.90	2 15	2805 2826	13
mmu-miR-135a-1-3p	NM_177371.3	146.00	-23.66	3 21	4888 4908	18
mmu-miR-135a-1-3p	NM_177371.3	143.00	-27.71	2 21	702 724	20
mmu-miR-135a-1-3p	XM_006496905.1	146.00	-20.52	2 15	4553 4574	13
mmu-miR-135a-1-3p	NM_001039047.1	147.00	-23.51	2 21	794 813	20
mmu-miR-135a-1-3p	XM_006508002.3	146.00	-24.01	2 20	797 819	19
mmu-miR-135a-1-3p	XM_017321602.1	144.00	-19.89	2 17	1766 1787	15
mmu-miR-135a-1-3p	XM_006506152.3	141.00	-16.04	2 19	3525 3547	18
mmu-miR-135a-1-3p	NM_023716.2	140.00	-13.27	2 9	1725 1746	7
mmu-miR-135a-1-3p	XM_006525099.3	145.00	-18.43	2 20	3377 3397	18
mmu-miR-135a-1-3p	XM_006525099.3	142.00	-20.11	3 19	527 548	16
mmu-miR-135a-1-3p	XM_006499466.3	140.00	-16.37	3 21	55 76	18
mmu-miR-135a-1-3p	XM_006530585.3	142.00	-21.83	2 21	10052 10072	19
mmu-miR-135a-1-3p	NM_199029.2	151.00	-27.81	2 21	1325 1347	20
mmu-miR-135a-1-3p	XM_011243586.2	145.00	-19.64	2 19	4607 4630	19
mmu-miR-135a-1-3p	XM_006511007.3	140.00	-21.02	3 21	6732 6751	18
mmu-miR-135a-1-3p	NM_001110508.1	150.00	-29.19	2 21	160 183	21
mmu-miR-135a-1-3p	XM_006519134.3	143.00	-28.93	2 21	2115 2140	23
mmu-miR-135a-1-3p	XM_006519134.3	142.00	-12.44	2 19	2510 2531	17
mmu-miR-135a-1-3p	XM_006519134.3	142.00	-24.43	2 21	5133 5156	21
mmu-miR-135a-1-3p	XM_006519134.3	140.00	-23.26	3 21	5958 5976	18
mmu-miR-1892	NM_133167.3	149.00	-20.76	2 14	3249 3270	12
mmu-miR-1892	NM_133167.3	144.00	-28.77	2 21	2162 2183	19
mmu-miR-1892	NM_011044.2	157.00	-19.67	2 14	441 462	12
mmu-miR-1892	NM_008804.4	146.00	-22.10	2 19	603 624	17
mmu-miR-1892	XM_006509465.2	157.00	-24.51	2 21	1197 1218	20
mmu-miR-1892	NM_001290822.1	140.00	-17.79	2 21	1296 1317	19
mmu-miR-1892	XM_006538456.3	146.00	-25.39	2 20	5768 5792	21
mmu-miR-1892	XM_006538456.3	143.00	-25.39	2 21	2828 2850	20
mmu-miR-1892	XM_006538456.3	140.00	-25.08	2 21	2566 2586	20
mmu-miR-1892	XM_006537815.3	142.00	-25.52	2 20	1698 1720	19
mmu-miR-1892	NM_153104.3	141.00	-18.78	2 17	188 207	15
mmu-miR-1892	NM_011082.3	160.00	-38.50	2 21	2585 2606	19
mmu-miR-1892	XM_017317946.1	147.00	-27.48	2 21	503 527	22
mmu-miR-1892	NM_018807.5	148.00	-24.16	2 18	1952 1974	17
mmu-miR-1892	NM_008872.3	155.00	-31.18	3 21	1410 1432	19
mmu-miR-1892	XM_017312497.1	141.00	-23.03	3 21	4293 4315	20
mmu-miR-1892	NM_026385.4	141.00	-20.64	2 20	933 953	18
mmu-miR-1892	XM_006502235.3	153.00	-21.34	2 18	3200 3221	16
mmu-miR-1892	XM_006502235.3	142.00	-26.95	3 21	2709 2729	18
mmu-miR-1892	XM_006512076.3	158.00	-25.14	2 21	2494 2519	23
mmu-miR-1892	XM_006512076.3	148.00	-27.51	2 21	8094 8115	19
mmu-miR-1892	XM_006512076.3	142.00	-20.08	3 19	2521 2542	16
mmu-miR-1892	XM_006512076.3	140.00	-21.31	2 15	2176 2196	13
mmu-miR-1892	NM_001302257.1	140.00	-12.44	2 9	787 808	7
mmu-miR-1892	NM_001291068.1	156.00	-23.08	2 20	6050 6071	19
mmu-miR-1892	NM_001291068.1	153.00	-28.66	2 21	125 144	19
mmu-miR-1892	NM_001291068.1	145.00	-25.33	3 21	5266 5285	18
mmu-miR-1892	NM_001291068.1	143.00	-20.48	2 21	737 759	20
mmu-miR-1892	NM_001291068.1	142.00	-14.75	2 13	550 570	11
mmu-miR-1892	NM_148932.2	180.00	-34.94	2 21	2036 2057	19
mmu-miR-1892	NM_148932.2	151.00	-33.94	2 20	1282 1303	18
mmu-miR-1892	NM_148932.2	144.00	-28.23	2 21	1523 1544	19
mmu-miR-1892	NM_148932.2	143.00	-24.69	2 18	4818 4838	16
mmu-miR-1892	NM_148932.2	141.00	-20.18	2 21	1904 1923	19
mmu-miR-1892	NM_011145.3	163.00	-33.93	2 21	2589 2611	20
mmu-miR-1892	XM_006525698.2	146.00	-27.10	2 21	2168 2194	24
mmu-miR-1892	XM_006525698.2	145.00	-21.24	2 19	6081 6103	18
mmu-miR-1892	XM_006525698.2	144.00	-21.70	2 21	1906 1927	19
mmu-miR-1892	XM_006525698.2	143.00	-28.20	2 20	1970 1991	18
mmu-miR-1892	XM_006527008.2	143.00	-26.03	3 21	5047 5069	19
mmu-miR-1892	XM_006527008.2	141.00	-26.85	3 19	4392 4414	17
mmu-miR-1892	XM_006527008.2	140.00	-13.07	2 9	3918 3939	7

mmu-miR-1892	NM_011169.5	167.00	-32.10	2 21	1902 1926	22
mmu-miR-1892	NM_011169.5	152.00	-16.41	2 17	9812 9833	15
mmu-miR-1892	NM_011169.5	142.00	-20.44	2 21	7188 7208	19
mmu-miR-1892	XM_011239558.2	142.00	-15.74	2 15	1235 1256	13
mmu-miR-1892	XM_011239558.2	142.00	-27.19	2 19	4300 4321	17
mmu-miR-1892	XM_011239558.2	140.00	-27.36	2 21	2317 2338	19
mmu-miR-1892	NM_175563.5	163.00	-34.33	2 21	178 200	20
mmu-miR-1892	NM_175563.5	145.00	-14.35	2 10	219 240	8
mmu-miR-1892	NM_175022.2	167.00	-28.08	2 21	2424 2446	20
mmu-miR-1892	NM_175022.2	155.00	-31.95	2 21	4568 4590	20
mmu-miR-1892	NM_175022.2	144.00	-27.93	3 20	4349 4371	19
mmu-miR-1892	NM_175022.2	141.00	-29.08	2 21	675 699	22
mmu-miR-1892	NM_001081224.2	147.00	-27.70	3 21	788 811	20
mmu-miR-1892	NM_001081224.2	141.00	-19.91	2 14	1648 1669	12
mmu-miR-1892	XM_006505015.3	155.00	-21.51	2 17	4381 4403	16
mmu-miR-1892	XM_006505015.3	142.00	-24.55	2 20	1851 1873	19
mmu-miR-1892	NM_027455.3	147.00	-23.14	2 21	774 796	20
mmu-miR-1892	XM_006514153.2	163.00	-26.64	2 21	3856 3879	21
mmu-miR-1892	XM_006514153.2	151.00	-26.08	2 21	3907 3930	21
mmu-miR-1892	XM_006514153.2	144.00	-25.98	2 17	1435 1456	15
mmu-miR-1892	XM_006514153.2	143.00	-36.40	2 21	1766 1790	22
mmu-miR-1892	NM_009019.2	145.00	-15.26	2 10	4450 4471	8
mmu-miR-1892	NM_198024.2	141.00	-13.05	2 19	2846 2869	19
mmu-miR-1892	NM_016809.6	145.00	-16.34	2 10	1215 1236	8
mmu-miR-1892	NM_016809.6	143.00	-16.54	2 16	1362 1383	14
mmu-miR-1892	XM_011240779.2	152.00	-23.09	2 21	5581 5602	19
mmu-miR-1892	XM_011240779.2	150.00	-23.97	2 21	976 996	19
mmu-miR-1892	NM_001190258.1	142.00	-26.97	2 21	51 71	19
mmu-miR-1892	XM_006525075.3	140.00	-26.98	3 21	859 880	18
mmu-miR-1892	NM_019732.2	143.00	-18.93	3 20	2293 2314	17
mmu-miR-1892	NM_019732.2	142.00	-27.18	2 21	1917 1942	23
mmu-miR-1892	XM_006540134.3	144.00	-21.54	2 18	2556 2578	17
mmu-miR-1892	NM_009127.4	144.00	-25.20	2 21	2171 2192	19
mmu-miR-1892	NM_018732.3	155.00	-28.25	2 20	1966 1987	18
mmu-miR-1892	NM_009135.2	144.00	-22.51	2 21	5282 5303	19
mmu-miR-1892	NM_020052.2	147.00	-21.82	2 21	2511 2533	20
mmu-miR-1892	NM_020052.2	145.00	-12.48	2 10	2451 2472	8
mmu-miR-1892	NM_026535.2	144.00	-24.77	2 21	1491 1512	19
mmu-miR-1892	NM_026535.2	144.00	-22.49	2 21	3353 3374	19
mmu-miR-1892	NM_026535.2	140.00	-27.47	2 21	3045 3066	19
mmu-miR-1892	XM_011244046.2	144.00	-17.68	2 13	1345 1366	11
mmu-miR-1892	XM_006530238.3	142.00	-18.60	2 11	3094 3115	9
mmu-miR-1892	XM_006530238.3	142.00	-34.51	2 21	4225 4248	21
mmu-miR-1892	XM_006530238.3	141.00	-23.74	3 21	6834 6853	18
mmu-miR-1892	XM_006530238.3	141.00	-21.27	2 14	8286 8307	12
mmu-miR-1892	XM_006530238.3	140.00	-17.01	2 21	2082 2103	19
mmu-miR-1892	XM_006530238.3	140.00	-18.14	2 9	4174 4195	7
mmu-miR-1892	XM_017316715.1	167.00	-28.31	2 21	4187 4209	20
mmu-miR-1892	XM_017316715.1	166.00	-33.56	2 21	3921 3945	22
mmu-miR-1892	XM_017316715.1	153.00	-22.90	2 18	2174 2195	16
mmu-miR-1892	XM_011250732.2	159.00	-35.38	2 21	5275 5297	20
mmu-miR-1892	XM_011250732.2	148.00	-33.29	2 21	110 131	19
mmu-miR-1892	XM_011250732.2	147.00	-29.99	3 21	3285 3307	19
mmu-miR-1892	XM_011250732.2	145.00	-14.87	2 10	7274 7295	8
mmu-miR-1892	XM_011250732.2	144.00	-27.51	3 21	7723 7744	18
mmu-miR-1892	XM_011250732.2	140.00	-23.56	2 17	7079 7100	15
mmu-miR-1892	XM_006496884.3	153.00	-18.47	2 20	2479 2499	18
mmu-miR-1892	XM_006496884.3	151.00	-16.09	2 20	2074 2095	18
mmu-miR-1892	XM_006496884.3	140.00	-12.87	2 9	1699 1720	7
mmu-miR-1892	NM_144539.5	155.00	-18.99	2 21	1359 1381	20
mmu-miR-1892	NM_144539.5	147.00	-25.69	2 20	1061 1082	18
mmu-miR-1892	NM_144539.5	141.00	-21.83	2 18	87 108	16
mmu-miR-1892	NM_001033167.3	144.00	-25.40	2 19	3758 3781	19
mmu-miR-1892	NM_001033167.3	143.00	-23.45	2 21	4555 4577	20
mmu-miR-1892	NM_001033167.3	140.00	-17.01	2 21	4942 4963	19

mmu-miR-1892	NM_001007570.2	148.00	-28.35	2 18	2710 2732	17
mmu-miR-1892	NM_001033286.2	149.00	-23.68	2 21	2102 2121	19
mmu-miR-1892	NM_001033286.2	149.00	-21.31	2 14	4207 4228	12
mmu-miR-1892	NM_001290993.1	143.00	-27.03	2 21	844 867	21
mmu-miR-1892	NM_001290993.1	141.00	-18.74	2 18	2754 2775	16
mmu-miR-1892	NM_001290993.1	140.00	-19.67	2 19	5066 5086	17
mmu-miR-1892	XM_006503080.3	144.00	-18.67	2 13	3581 3602	11
mmu-miR-1892	NM_027052.3	146.00	-30.26	2 21	2108 2128	19
mmu-miR-1892	XM_006538687.2	164.00	-22.61	2 17	3428 3449	15
mmu-miR-1892	XM_006538687.2	152.00	-30.95	3 21	1278 1299	18
mmu-miR-1892	XM_006538687.2	147.00	-35.63	2 21	264 286	20
mmu-miR-1892	NM_001146217.1	152.00	-26.11	2 21	2077 2098	19
mmu-miR-1892	NM_001146217.1	144.00	-27.37	2 19	169 189	17
mmu-miR-1892	NM_175692.3	155.00	-20.47	2 16	5182 5203	14
mmu-miR-1892	NM_175692.3	140.00	-28.27	2 21	3907 3928	19
mmu-miR-1892	XM_006534520.3	159.00	-35.46	2 21	1699 1721	20
mmu-miR-1892	XM_006534520.3	144.00	-23.74	2 21	888 909	19
mmu-miR-1892	XM_006539074.3	157.00	-29.45	2 21	8658 8683	23
mmu-miR-1892	XM_006539074.3	155.00	-23.33	2 16	3611 3632	14
mmu-miR-1892	XM_006539074.3	152.00	-23.42	2 18	7987 8009	17
mmu-miR-1892	XM_006539074.3	147.00	-27.71	2 21	7923 7945	20
mmu-miR-1892	XM_006539074.3	144.00	-25.96	2 21	6880 6901	19
mmu-miR-1892	XM_006539074.3	144.00	-28.15	2 21	8528 8549	19
mmu-miR-1892	XM_006539074.3	143.00	-34.58	2 21	7137 7159	20
mmu-miR-1892	XM_006539074.3	142.00	-11.47	2 11	5684 5705	9
mmu-miR-1892	XM_006539074.3	142.00	-18.79	2 19	8750 8771	17
mmu-miR-1892	NM_019866.1	150.00	-20.77	2 21	1115 1135	19
mmu-miR-1892	NM_019866.1	143.00	-28.66	2 21	895 918	21
mmu-miR-1892	NM_178753.4	145.00	-22.67	2 21	3961 3980	19
mmu-miR-1892	NM_001304266.1	153.00	-23.61	2 18	8180 8201	16
mmu-miR-1892	NM_001304266.1	148.00	-23.78	2 17	1020 1041	15
mmu-miR-1892	NM_001304266.1	144.00	-22.35	2 17	8507 8528	15
mmu-miR-1892	NM_001304266.1	143.00	-26.76	3 21	9519 9541	19
mmu-miR-1892	NM_001304266.1	142.00	-24.09	3 21	7910 7930	18
mmu-miR-1892	NM_001304266.1	142.00	-21.11	2 16	8990 9012	15
mmu-miR-1892	NM_001304266.1	142.00	-18.32	2 19	10722 10743	17
mmu-miR-1892	NM_001304266.1	140.00	-32.79	2 21	3747 3768	19
mmu-miR-1892	NM_001304266.1	140.00	-25.53	3 21	5963 5984	18
mmu-miR-1892	NM_001304266.1	140.00	-19.88	3 21	9215 9236	18
mmu-miR-1892	NM_001242411.1	146.00	-27.95	2 18	4640 4659	16
mmu-miR-1892	NM_001242411.1	145.00	-15.15	2 10	2696 2717	8
mmu-miR-1892	NM_001242411.1	141.00	-20.25	2 19	5773 5795	18
mmu-miR-1892	NM_001242411.1	141.00	-23.09	2 21	6683 6702	19
mmu-miR-1892	XM_006506145.1	164.00	-25.73	2 21	8748 8769	19
mmu-miR-1892	XM_006506145.1	160.00	-26.26	2 20	6613 6632	18
mmu-miR-1892	XM_006506145.1	145.00	-25.92	3 19	6798 6820	17
mmu-miR-1892	XM_011248193.2	161.00	-28.06	2 18	6525 6546	16
mmu-miR-1892	XM_011248193.2	155.00	-29.88	2 21	5270 5292	20
mmu-miR-1892	XM_011248193.2	140.00	-20.88	2 15	4327 4347	13
mmu-miR-1892	XM_011245529.2	142.00	-26.96	2 21	1803 1827	22
mmu-miR-1892	XM_011245529.2	140.00	-10.53	2 9	4869 4890	7
mmu-miR-1892	NM_011374.2	161.00	-29.31	2 21	3018 3037	19
mmu-miR-1892	NM_011374.2	140.00	-20.14	2 21	8700 8721	19
mmu-miR-1892	XM_006527452.3	148.00	-17.96	2 21	1933 1954	19
mmu-miR-1892	NM_009332.3	180.00	-31.77	2 21	2091 2112	19
mmu-miR-1892	NM_009332.3	152.00	-26.50	2 21	708 729	19
mmu-miR-1892	NM_009332.3	147.00	-31.37	3 21	2453 2476	20
mmu-miR-1892	NM_001080979.1	141.00	-34.23	3 21	299 320	19
mmu-miR-1892	NM_011581.3	150.00	-17.73	2 20	3668 3691	20
mmu-miR-1892	NM_011581.3	142.00	-14.74	2 13	2537 2557	11
mmu-miR-1892	NM_011581.3	140.00	-22.17	2 18	1780 1802	17
mmu-miR-1892	NM_009379.3	150.00	-28.29	2 20	462 485	20
mmu-miR-1892	NM_009379.3	141.00	-27.95	2 20	1265 1285	18
mmu-miR-1892	XM_006501237.3	152.00	-24.57	2 21	195 216	19
mmu-miR-1892	XM_006533452.3	147.00	-33.59	2 21	460 485	23

mmu-miR-1892	XM_006533452.3	143.00	-20.77	2 20	485 506	18
mmu-miR-1892	XM_006533452.3	143.00	-25.04	2 21	4028 4050	20
mmu-miR-1892	XM_006533452.3	140.00	-14.16	2 9	5615 5636	7
mmu-miR-1892	XM_006521428.3	159.00	-23.45	2 16	1483 1504	14
mmu-miR-1892	NM_177371.3	147.00	-18.99	2 20	3858 3879	18
mmu-miR-1892	NM_177371.3	144.00	-24.74	2 21	4907 4928	19
mmu-miR-1892	NM_177371.3	142.00	-17.82	2 15	5032 5053	13
mmu-miR-1892	NM_177371.3	141.00	-13.26	2 14	2233 2254	12
mmu-miR-1892	XM_006496905.1	149.00	-23.27	2 18	3778 3799	16
mmu-miR-1892	XM_006496905.1	146.00	-32.81	2 21	5506 5529	21
mmu-miR-1892	XM_006496905.1	143.00	-13.72	2 12	2093 2114	10
mmu-miR-1892	XM_006496905.1	143.00	-13.72	2 12	2621 2642	10
mmu-miR-1892	XM_006496905.1	141.00	-13.38	2 18	3149 3170	16
mmu-miR-1892	XM_006496905.1	140.00	-15.51	2 9	1829 1850	7
mmu-miR-1892	XM_006539726.3	143.00	-24.13	2 21	188 210	20
mmu-miR-1892	XM_006539726.3	141.00	-13.67	2 12	152 172	10
mmu-miR-1892	XM_011248201.2	141.00	-25.18	2 19	600 622	18
mmu-miR-1892	XM_011248201.2	141.00	-25.69	3 18	3848 3869	15
mmu-miR-1892	NM_177409.3	142.00	-28.97	2 19	1100 1121	17
mmu-miR-1892	XM_011249157.2	148.00	-23.65	2 21	2366 2387	19
mmu-miR-1892	XM_011249157.2	142.00	-33.20	2 20	867 889	19
mmu-miR-1892	NM_001039047.1	147.00	-27.61	2 17	1112 1134	16
mmu-miR-1892	NM_001039047.1	143.00	-20.97	2 18	1517 1537	16
mmu-miR-1892	XM_017321602.1	141.00	-23.83	2 21	2033 2052	19
mmu-miR-1892	NM_001033149.3	144.00	-22.23	2 21	2926 2947	19
mmu-miR-1892	NM_001033149.3	142.00	-18.63	2 21	2778 2798	19
mmu-miR-1892	NM_001033149.3	142.00	-33.74	2 21	3459 3482	21
mmu-miR-1892	NM_177709.3	142.00	-14.76	2 11	564 585	9
mmu-miR-1892	NM_177709.3	141.00	-28.41	2 18	913 934	16
mmu-miR-1892	NM_177709.3	140.00	-13.49	2 9	2400 2421	7
mmu-miR-1892	XM_006538224.1	148.00	-22.00	2 21	2247 2268	19
mmu-miR-1892	XM_006538224.1	140.00	-12.66	2 9	2679 2700	7
mmu-miR-1892	XM_011248394.1	153.00	-28.00	3 18	726 747	15
mmu-miR-1892	XM_011248394.1	153.00	-18.78	2 20	3413 3433	18
mmu-miR-1892	NM_009504.4	148.00	-20.51	2 19	2848 2868	17
mmu-miR-1892	NM_009504.4	140.00	-19.70	2 21	1130 1151	19
mmu-miR-1892	XM_006525028.2	146.00	-18.96	2 16	481 504	16
mmu-miR-1892	XM_006525028.2	140.00	-11.42	2 9	697 718	7
mmu-miR-1892	NM_016982.2	153.00	-22.24	2 14	44 65	12
mmu-miR-1892	NM_177789.4	143.00	-21.05	2 21	1201 1223	20
mmu-miR-1892	NM_177789.4	140.00	-18.58	2 17	121 142	15
mmu-miR-1892	NM_009518.2	148.00	-31.24	2 21	1712 1733	19
mmu-miR-1892	NM_009518.2	142.00	-26.97	2 21	288 308	19
mmu-miR-1892	XM_006533569.3	152.00	-20.29	2 15	415 435	13
mmu-miR-1892	NM_011724.3	163.00	-27.95	2 21	1919 1943	22
mmu-miR-1892	NM_011724.3	148.00	-21.29	2 17	266 287	15
mmu-miR-1892	NM_011724.3	146.00	-30.74	2 21	236 262	24
mmu-miR-1892	NM_011724.3	146.00	-20.93	2 19	2176 2197	17
mmu-miR-1892	NM_011724.3	143.00	-16.59	2 16	4094 4112	14
mmu-miR-1892	NM_011724.3	142.00	-20.14	2 19	3536 3557	17
mmu-miR-1892	NM_011724.3	141.00	-17.47	2 11	3059 3081	10
mmu-miR-1892	NM_011724.3	140.00	-25.84	2 21	5776 5797	19
mmu-miR-1892	XM_006499466.3	158.00	-28.51	2 21	1592 1612	19
mmu-miR-1892	XM_006499466.3	142.00	-21.93	2 21	1155 1175	19
mmu-miR-1892	XM_006530585.3	160.00	-35.87	2 18	9870 9892	17
mmu-miR-1892	XM_006530585.3	158.00	-23.71	2 19	13418 13439	17
mmu-miR-1892	XM_006530585.3	150.00	-22.48	2 21	14914 14934	19
mmu-miR-1892	XM_006530585.3	149.00	-31.83	2 21	6809 6830	20
mmu-miR-1892	XM_006530585.3	148.00	-26.00	3 21	12396 12417	18
mmu-miR-1892	XM_006530585.3	144.00	-25.80	3 21	14241 14262	18
mmu-miR-1892	XM_006530585.3	143.00	-28.36	3 21	14204 14226	19
mmu-miR-1892	XM_006530585.3	141.00	-14.43	2 11	2019 2041	10
mmu-miR-1892	XM_006530585.3	140.00	-16.53	2 9	10411 10432	7
mmu-miR-1892	XM_017314347.1	142.00	-14.97	2 11	618 639	9
mmu-miR-1892	NM_199304.1	156.00	-30.73	2 21	585 606	19

mmu-miR-1892	NM_199029.2	150.00	-19.43	2 21	831 851	19
mmu-miR-1892	XM_011243586.2	164.00	-21.98	2 21	4388 4409	19
mmu-miR-1892	XM_011243586.2	152.00	-27.00	2 21	81 102	19
mmu-miR-1892	XM_006511007.3	152.00	-19.80	2 21	1188 1209	19
mmu-miR-1892	XM_006511007.3	141.00	-21.29	3 18	1585 1606	15
mmu-miR-1892	XM_006511007.3	141.00	-29.83	2 21	7519 7538	19
mmu-miR-1892	NM_001110508.1	151.00	-20.68	2 19	2148 2167	17
mmu-miR-1892	NM_001110508.1	147.00	-20.85	2 18	2607 2627	16
mmu-miR-1892	XM_006537508.2	142.00	-17.48	2 11	1690 1711	9
mmu-miR-1892	XM_006537508.2	142.00	-10.74	2 11	1802 1823	9
mmu-miR-1892	XM_006537508.2	141.00	-24.72	2 18	1052 1073	16
mmu-miR-1892	XM_006519134.3	152.00	-26.28	3 17	727 748	14
mmu-miR-1892	XM_006519134.3	149.00	-23.73	2 18	4926 4947	16
mmu-miR-1892	XM_006519134.3	148.00	-26.79	2 18	4199 4221	17
mmu-miR-5113	NM_172454.2	155.00	-25.01	2 22	115 136	20
mmu-miR-5113	NM_172454.2	144.00	-23.31	3 22	799 824	22
mmu-miR-5113	NM_028748.2	145.00	-23.45	2 18	884 906	16
mmu-miR-5113	NM_133167.3	147.00	-19.41	2 18	1379 1400	16
mmu-miR-5113	NM_133167.3	146.00	-23.87	3 17	2057 2078	14
mmu-miR-5113	NM_133167.3	140.00	-18.23	3 18	2068 2091	16
mmu-miR-5113	NM_011044.2	141.00	-14.95	2 10	1699 1721	8
mmu-miR-5113	NM_013743.2	152.00	-30.00	2 22	1562 1585	21
mmu-miR-5113	NM_013743.2	147.00	-21.98	2 22	996 1017	20
mmu-miR-5113	NM_013743.2	141.00	-21.52	3 18	2938 2960	15
mmu-miR-5113	XM_006538456.3	155.00	-25.96	2 16	5976 5998	14
mmu-miR-5113	XM_006538456.3	146.00	-22.17	2 20	339 362	19
mmu-miR-5113	XM_006538456.3	144.00	-18.26	2 22	262 286	22
mmu-miR-5113	XM_006538456.3	143.00	-17.39	2 19	6244 6266	18
mmu-miR-5113	XM_006538456.3	140.00	-24.96	2 19	2641 2662	17
mmu-miR-5113	XM_006537815.3	140.00	-21.07	2 13	5024 5046	11
mmu-miR-5113	NM_153104.3	152.00	-20.12	2 21	1402 1424	19
mmu-miR-5113	NM_153104.3	141.00	-18.23	3 22	1153 1175	19
mmu-miR-5113	NM_011082.3	140.00	-20.95	3 19	2838 2865	21
mmu-miR-5113	XM_017314870.1	168.00	-26.70	2 22	7057 7080	21
mmu-miR-5113	XM_017314870.1	146.00	-18.70	2 15	1355 1377	13
mmu-miR-5113	XM_017317946.1	144.00	-18.45	2 22	1371 1395	22
mmu-miR-5113	NM_018807.5	144.00	-20.19	2 17	2939 2961	15
mmu-miR-5113	NM_018807.5	140.00	-21.99	2 17	1181 1203	15
mmu-miR-5113	NM_008872.3	154.00	-25.81	2 22	1482 1508	24
mmu-miR-5113	NM_008872.3	147.00	-22.34	3 22	1842 1868	23
mmu-miR-5113	XM_011250776.2	154.00	-25.08	3 19	16 38	16
mmu-miR-5113	XM_011250776.2	145.00	-21.87	3 18	28 50	15
mmu-miR-5113	XM_011250776.2	143.00	-21.09	3 22	1954 1975	19
mmu-miR-5113	NM_026385.4	166.00	-23.19	2 19	281 303	17
mmu-miR-5113	XM_006502235.3	167.00	-31.69	2 22	4439 4460	20
mmu-miR-5113	XM_006502235.3	148.00	-22.49	2 18	3392 3415	17
mmu-miR-5113	XM_006502235.3	145.00	-24.58	2 22	2127 2149	20
mmu-miR-5113	XM_006502235.3	144.00	-24.45	2 13	1401 1423	11
mmu-miR-5113	XM_006502235.3	143.00	-19.97	2 16	3898 3920	14
mmu-miR-5113	XM_006502235.3	140.00	-17.81	2 9	1996 2018	7
mmu-miR-5113	XM_006512076.3	144.00	-18.88	2 22	5241 5264	21
mmu-miR-5113	XM_006512076.3	143.00	-19.41	2 16	7218 7240	14
mmu-miR-5113	XM_006512076.3	141.00	-18.08	2 19	8563 8586	18
mmu-miR-5113	NM_001302257.1	146.00	-21.02	2 15	165 187	13
mmu-miR-5113	NM_001302257.1	143.00	-19.24	2 21	442 466	21
mmu-miR-5113	NM_001302257.1	141.00	-16.06	2 18	1749 1771	16
mmu-miR-5113	NM_001291068.1	153.00	-21.44	2 19	218 242	19
mmu-miR-5113	NM_001291068.1	145.00	-15.95	2 10	2655 2677	8
mmu-miR-5113	NM_001291068.1	142.00	-18.95	2 20	729 752	19
mmu-miR-5113	NM_001291068.1	141.00	-16.19	3 18	6476 6498	15
mmu-miR-5113	NM_001291068.1	140.00	-20.05	3 13	135 157	10
mmu-miR-5113	NM_148932.2	177.00	-30.31	2 22	1122 1144	20
mmu-miR-5113	NM_148932.2	164.00	-24.31	2 22	4045 4068	21
mmu-miR-5113	NM_148932.2	153.00	-20.93	2 19	4099 4123	19
mmu-miR-5113	NM_148932.2	150.00	-20.77	2 19	3057 3079	17

mmu-miR-5113	NM_148932.2	141.00	-21.91	2 22	4181 4201	20
mmu-miR-5113	NM_148932.2	140.00	-14.06	2 9	1971 1993	7
mmu-miR-5113	NM_011145.3	144.00	-19.20	2 22	2703 2726	21
mmu-miR-5113	XM_006525698.2	156.00	-18.87	2 22	4581 4604	21
mmu-miR-5113	XM_006525698.2	155.00	-26.05	2 22	5568 5595	25
mmu-miR-5113	XM_006525698.2	154.00	-24.00	2 20	9517 9543	22
mmu-miR-5113	XM_006525698.2	153.00	-17.22	2 19	8507 8530	18
mmu-miR-5113	XM_006525698.2	147.00	-21.33	2 18	3215 3236	16
mmu-miR-5113	XM_006525698.2	144.00	-21.15	2 22	4146 4169	21
mmu-miR-5113	XM_006525698.2	143.00	-18.23	3 20	1901 1923	17
mmu-miR-5113	XM_006525698.2	143.00	-16.14	2 13	2405 2428	12
mmu-miR-5113	XM_006525698.2	140.00	-17.40	2 9	2228 2250	7
mmu-miR-5113	XM_006527008.2	157.00	-20.25	2 22	2169 2191	20
mmu-miR-5113	XM_006527008.2	148.00	-21.00	2 17	2553 2575	15
mmu-miR-5113	XM_006527008.2	146.00	-23.96	3 17	4469 4490	14
mmu-miR-5113	XM_006527008.2	144.00	-19.10	2 22	4423 4448	23
mmu-miR-5113	XM_006527008.2	140.00	-16.68	2 17	2688 2710	15
mmu-miR-5113	NM_011169.5	149.00	-22.49	3 19	494 517	17
mmu-miR-5113	NM_011169.5	144.00	-23.01	3 17	344 366	14
mmu-miR-5113	XM_011239558.2	146.00	-22.81	2 20	1976 2000	20
mmu-miR-5113	NM_175563.5	149.00	-22.91	3 18	1679 1701	15
mmu-miR-5113	NM_175563.5	149.00	-19.83	3 19	1706 1729	17
mmu-miR-5113	NM_175563.5	147.00	-22.86	2 22	1876 1903	25
mmu-miR-5113	NM_175563.5	146.00	-23.96	3 17	659 680	14
mmu-miR-5113	NM_175563.5	146.00	-13.29	2 19	2586 2608	17
mmu-miR-5113	NM_175022.2	160.00	-21.87	2 22	416 440	22
mmu-miR-5113	NM_175022.2	144.00	-19.94	3 21	304 326	18
mmu-miR-5113	NM_175022.2	144.00	-21.98	2 19	732 758	21
mmu-miR-5113	NM_175022.2	142.00	-17.98	2 19	3152 3174	17
mmu-miR-5113	XM_006524382.3	152.00	-20.21	2 21	579 601	19
mmu-miR-5113	XM_006524382.3	148.00	-23.87	2 22	1130 1153	21
mmu-miR-5113	XM_006524382.3	140.00	-23.65	2 22	200 223	21
mmu-miR-5113	XM_011240476.2	152.00	-29.05	3 22	44 70	23
mmu-miR-5113	NM_008966.3	156.00	-20.57	2 19	2051 2072	17
mmu-miR-5113	NM_008966.3	149.00	-20.25	2 18	2474 2496	16
mmu-miR-5113	XM_006505015.3	152.00	-23.31	3 18	186 209	16
mmu-miR-5113	NM_009019.2	158.00	-27.57	2 15	1737 1759	13
mmu-miR-5113	NM_009019.2	150.00	-20.48	2 19	4841 4863	17
mmu-miR-5113	NM_009019.2	144.00	-24.82	3 22	4251 4276	22
mmu-miR-5113	NM_198024.2	146.00	-20.38	2 20	3522 3545	19
mmu-miR-5113	XM_006506624.2	142.00	-20.03	2 17	14 35	15
mmu-miR-5113	XM_017320735.1	145.00	-18.87	2 19	3546 3569	18
mmu-miR-5113	NM_016809.6	162.00	-25.41	2 19	207 229	17
mmu-miR-5113	NM_016809.6	140.00	-18.22	3 17	40 62	14
mmu-miR-5113	NM_031192.3	147.00	-20.73	2 17	404 427	16
mmu-miR-5113	NM_001204959.1	143.00	-16.06	2 21	47 70	20
mmu-miR-5113	XM_006529829.3	151.00	-25.25	3 17	3498 3521	15
mmu-miR-5113	XM_006529829.3	141.00	-17.89	2 20	1124 1145	18
mmu-miR-5113	XM_006529829.3	141.00	-16.07	2 19	3565 3588	18
mmu-miR-5113	XM_011240779.2	165.00	-25.47	2 19	3497 3520	18
mmu-miR-5113	XM_011240779.2	143.00	-18.46	2 12	722 744	10
mmu-miR-5113	XM_011240779.2	141.00	-26.86	2 22	4347 4366	20
mmu-miR-5113	XM_006507942.2	155.00	-21.78	2 17	1000 1023	16
mmu-miR-5113	XM_006507942.2	147.00	-14.82	2 12	3843 3865	10
mmu-miR-5113	XM_006507942.2	145.00	-21.10	2 20	1632 1653	18
mmu-miR-5113	NM_001190258.1	148.00	-20.51	2 21	201 224	21
mmu-miR-5113	NM_019732.2	146.00	-18.52	2 11	712 734	9
mmu-miR-5113	NM_019732.2	140.00	-17.14	2 21	3639 3661	19
mmu-miR-5113	NM_023893.4	152.00	-19.17	2 13	198 220	11
mmu-miR-5113	XM_006540134.3	157.00	-24.59	3 18	1293 1315	15
mmu-miR-5113	XM_006540134.3	149.00	-22.23	3 19	1399 1422	17
mmu-miR-5113	XM_006540134.3	149.00	-24.15	3 18	1498 1520	15
mmu-miR-5113	XM_006540134.3	148.00	-21.44	3 18	1345 1369	17
mmu-miR-5113	XM_006540134.3	146.00	-26.57	2 21	676 697	19
mmu-miR-5113	XM_006540134.3	145.00	-19.03	3 18	1326 1348	15

mmu-miR-5113	XM_006540134.3	145.00	-24.32	3 18	1359 1381	15
mmu-miR-5113	XM_006540134.3	144.00	-20.29	3 17	1427 1449	14
mmu-miR-5113	XM_006540134.3	143.00	-20.33	3 17	1514 1537	15
mmu-miR-5113	XM_006540134.3	142.00	-18.80	3 17	1303 1324	14
mmu-miR-5113	XM_006540134.3	142.00	-18.80	3 17	1437 1458	14
mmu-miR-5113	NM_009127.4	164.00	-23.09	2 18	2160 2183	17
mmu-miR-5113	NM_009127.4	152.00	-25.02	2 18	3706 3729	17
mmu-miR-5113	NM_009127.4	145.00	-21.43	2 19	1743 1766	18
mmu-miR-5113	NM_009127.4	144.00	-23.39	3 22	3801 3825	21
mmu-miR-5113	NM_018732.3	155.00	-25.75	2 22	7014 7038	22
mmu-miR-5113	NM_018732.3	143.00	-18.29	2 20	4149 4171	18
mmu-miR-5113	NM_018732.3	141.00	-17.20	2 10	2210 2232	8
mmu-miR-5113	NM_009135.2	150.00	-21.31	3 21	3750 3771	18
mmu-miR-5113	NM_009135.2	148.00	-20.50	2 22	2248 2273	23
mmu-miR-5113	NM_009135.2	143.00	-22.58	3 20	4841 4863	17
mmu-miR-5113	NM_009135.2	142.00	-15.41	2 19	2118 2140	17
mmu-miR-5113	NM_008458.2	159.00	-23.98	2 22	146 171	23
mmu-miR-5113	XM_006515637.2	145.00	-19.96	3 22	1321 1343	19
mmu-miR-5113	XM_011244046.2	163.00	-24.24	2 22	146 171	23
mmu-miR-5113	XM_006521796.1	152.00	-22.21	2 21	2010 2032	19
mmu-miR-5113	XM_017322211.1	158.00	-22.76	2 19	4458 4480	17
mmu-miR-5113	XM_017322211.1	154.00	-25.34	2 19	4773 4795	17
mmu-miR-5113	XM_017322211.1	152.00	-23.85	2 22	2018 2041	21
mmu-miR-5113	XM_017322211.1	147.00	-21.35	3 19	4428 4450	17
mmu-miR-5113	XM_017322211.1	146.00	-21.63	3 17	4438 4459	14
mmu-miR-5113	XM_017322211.1	143.00	-21.60	2 19	5511 5533	18
mmu-miR-5113	XM_006530238.3	157.00	-22.87	2 22	3211 3230	20
mmu-miR-5113	XM_006530238.3	145.00	-24.19	3 18	7746 7768	15
mmu-miR-5113	XM_006530238.3	140.00	-28.02	2 18	7784 7808	18
mmu-miR-5113	XM_017317034.1	147.00	-15.12	2 21	1021 1044	20
mmu-miR-5113	XM_017317034.1	146.00	-21.38	2 17	2516 2537	15
mmu-miR-5113	XM_017317034.1	143.00	-20.52	2 21	2490 2514	21
mmu-miR-5113	XM_017317034.1	142.00	-16.33	3 19	3000 3022	16
mmu-miR-5113	XM_017316897.1	140.00	-25.91	2 22	2330 2354	22
mmu-miR-5113	XM_011250732.2	141.00	-18.78	3 22	7681 7703	19
mmu-miR-5113	XM_006496884.3	148.00	-24.31	2 22	2396 2421	23
mmu-miR-5113	XM_006496884.3	147.00	-23.30	2 22	1310 1331	20
mmu-miR-5113	XM_006496884.3	143.00	-22.14	2 21	561 586	22
mmu-miR-5113	XM_006496884.3	143.00	-15.74	3 20	2429 2451	17
mmu-miR-5113	XM_006496884.3	140.00	-18.39	2 13	2489 2511	11
mmu-miR-5113	NM_144539.5	160.00	-24.50	2 19	473 498	20
mmu-miR-5113	NM_144539.5	144.00	-18.58	2 19	2138 2159	17
mmu-miR-5113	NM_144539.5	144.00	-16.11	2 22	3126 3149	21
mmu-miR-5113	NM_001310705.1	146.00	-18.45	2 19	413 435	17
mmu-miR-5113	NM_009199.2	141.00	-15.45	2 18	2858 2880	16
mmu-miR-5113	NM_009199.2	140.00	-15.26	2 9	3148 3170	7
mmu-miR-5113	NM_001033167.3	155.00	-23.03	2 21	6057 6080	20
mmu-miR-5113	NM_001033167.3	145.00	-21.09	2 22	2049 2071	20
mmu-miR-5113	NM_001033167.3	142.00	-15.79	2 11	5758 5780	9
mmu-miR-5113	NM_001007570.2	142.00	-16.34	2 17	1296 1317	15
mmu-miR-5113	NM_001007570.2	141.00	-20.04	3 18	2138 2160	15
mmu-miR-5113	NM_001007570.2	141.00	-29.55	2 19	2216 2240	19
mmu-miR-5113	NM_001007570.2	140.00	-28.02	2 18	2198 2222	18
mmu-miR-5113	NM_001290993.1	155.00	-24.29	2 22	3555 3580	23
mmu-miR-5113	XM_006503080.3	161.00	-18.87	2 19	1734 1757	18
mmu-miR-5113	XM_006503080.3	157.00	-25.38	3 18	2129 2151	15
mmu-miR-5113	XM_006503080.3	151.00	-25.08	3 18	2029 2050	15
mmu-miR-5113	XM_006503080.3	151.00	-23.07	3 18	2080 2101	15
mmu-miR-5113	XM_006503080.3	146.00	-21.60	3 17	2139 2160	14
mmu-miR-5113	XM_006503080.3	141.00	-25.89	4 18	2091 2113	14
mmu-miR-5113	XM_006538687.2	149.00	-24.30	2 22	4020 4042	20
mmu-miR-5113	XM_006538687.2	143.00	-19.58	3 20	1995 2017	17
mmu-miR-5113	XM_006538687.2	142.00	-18.29	2 19	1291 1313	17
mmu-miR-5113	XM_006538687.2	142.00	-18.50	2 19	2247 2269	17
mmu-miR-5113	XM_006538687.2	141.00	-21.89	3 20	1477 1498	17

mmu-miR-5113	NM_025540.2	141.00	-16.52	3 22	52 74	19
mmu-miR-5113	NM_001146217.1	162.00	-23.72	2 21	2672 2696	21
mmu-miR-5113	NM_001146217.1	155.00	-20.12	2 17	2039 2062	16
mmu-miR-5113	NM_001146217.1	143.00	-18.92	2 21	2149 2172	20
mmu-miR-5113	XM_006524777.3	154.00	-22.49	2 20	1243 1266	19
mmu-miR-5113	XM_006524777.3	148.00	-16.27	2 18	1895 1920	19
mmu-miR-5113	XM_006500109.3	145.00	-18.45	2 10	729 751	8
mmu-miR-5113	NM_011430.3	148.00	-15.38	2 17	640 662	15
mmu-miR-5113	NM_175692.3	150.00	-22.35	2 21	5814 5835	19
mmu-miR-5113	NM_175692.3	149.00	-19.84	3 18	3045 3067	15
mmu-miR-5113	NM_175692.3	149.00	-19.66	3 18	3078 3100	15
mmu-miR-5113	NM_175692.3	145.00	-21.13	3 19	3170 3193	17
mmu-miR-5113	NM_175692.3	142.00	-18.53	3 17	3055 3076	14
mmu-miR-5113	NM_175692.3	142.00	-18.53	3 17	3064 3085	14
mmu-miR-5113	NM_175692.3	141.00	-18.68	2 22	2198 2220	20
mmu-miR-5113	NM_009223.3	164.00	-24.11	2 22	1298 1321	21
mmu-miR-5113	XM_006534520.3	146.00	-17.01	3 19	3214 3236	16
mmu-miR-5113	XM_006539074.3	150.00	-22.89	2 11	9464 9486	9
mmu-miR-5113	NM_178753.4	144.00	-24.13	2 19	113 134	17
mmu-miR-5113	NM_178753.4	141.00	-19.92	3 18	1694 1716	15
mmu-miR-5113	NM_001204201.1	145.00	-20.44	2 20	322 343	18
mmu-miR-5113	NM_001304266.1	164.00	-27.73	2 18	5460 5484	18
mmu-miR-5113	NM_001304266.1	149.00	-14.92	2 22	830 852	20
mmu-miR-5113	NM_001304266.1	143.00	-20.16	3 22	4852 4873	19
mmu-miR-5113	NM_001304266.1	143.00	-20.25	3 18	8106 8127	15
mmu-miR-5113	NM_001304266.1	141.00	-14.36	2 10	9909 9931	8
mmu-miR-5113	NM_001242411.1	141.00	-20.95	2 21	1 24	23
mmu-miR-5113	XM_006506145.1	164.00	-27.59	3 22	5717 5740	20
mmu-miR-5113	XM_006506145.1	148.00	-18.69	2 22	8895 8918	21
mmu-miR-5113	XM_006506145.1	147.00	-24.81	3 22	1209 1230	19
mmu-miR-5113	XM_006506145.1	146.00	-23.96	3 17	5761 5782	14
mmu-miR-5113	XM_006506145.1	145.00	-20.78	3 18	8102 8124	15
mmu-miR-5113	XM_006506145.1	141.00	-22.99	2 19	5680 5703	18
mmu-miR-5113	XM_006506145.1	140.00	-17.96	3 17	5464 5486	14
mmu-miR-5113	XM_011248193.2	152.00	-24.85	3 17	8112 8134	14
mmu-miR-5113	XM_011248193.2	146.00	-16.14	2 15	2202 2224	13
mmu-miR-5113	XM_011248193.2	143.00	-17.53	2 22	6809 6834	23
mmu-miR-5113	XM_011248193.2	142.00	-22.89	3 19	7662 7684	16
mmu-miR-5113	XM_011245529.2	149.00	-20.87	2 19	1169 1193	19
mmu-miR-5113	XM_011245529.2	145.00	-18.65	3 18	5206 5228	15
mmu-miR-5113	XM_011245529.2	141.00	-20.51	3 20	3222 3246	19
mmu-miR-5113	NM_011374.2	159.00	-29.09	2 22	5211 5236	23
mmu-miR-5113	NM_011374.2	148.00	-21.32	3 17	7528 7550	14
mmu-miR-5113	NM_011374.2	145.00	-17.20	2 22	6704 6726	20
mmu-miR-5113	NM_011374.2	145.00	-15.64	2 10	6976 6998	8
mmu-miR-5113	NM_011374.2	140.00	-17.09	2 9	782 804	7
mmu-miR-5113	NM_011374.2	140.00	-15.22	2 9	6547 6569	7
mmu-miR-5113	XM_006527452.3	148.00	-19.26	2 22	1679 1702	21
mmu-miR-5113	NM_032400.2	144.00	-21.85	2 22	175 198	21
mmu-miR-5113	XM_017316056.1	148.00	-23.64	2 21	2363 2385	19
mmu-miR-5113	NM_009332.3	153.00	-17.05	2 19	2380 2403	18
mmu-miR-5113	NM_009332.3	145.00	-25.21	3 19	2193 2216	17
mmu-miR-5113	NM_001080979.1	162.00	-27.43	2 19	2447 2469	17
mmu-miR-5113	NM_001080979.1	147.00	-18.83	2 21	348 373	22
mmu-miR-5113	NM_153801.3	140.00	-13.76	2 9	1891 1913	7
mmu-miR-5113	NM_011581.3	143.00	-13.85	2 12	2889 2911	10
mmu-miR-5113	NM_009381.3	141.00	-23.22	2 20	496 517	18
mmu-miR-5113	XM_006501237.3	149.00	-18.73	2 18	1991 2013	16
mmu-miR-5113	XM_006501237.3	147.00	-20.57	2 21	294 318	21
mmu-miR-5113	XM_006533479.2	179.00	-32.79	2 22	32 53	20
mmu-miR-5113	XM_006533452.3	152.00	-25.03	2 22	4408 4431	21
mmu-miR-5113	XM_006533452.3	148.00	-23.93	3 22	1541 1565	21
mmu-miR-5113	XM_006533452.3	145.00	-20.00	2 10	3491 3513	8
mmu-miR-5113	XM_006533452.3	142.00	-14.39	2 11	2421 2443	9
mmu-miR-5113	XM_006515787.3	151.00	-20.14	2 20	2600 2622	18

mmu-miR-5113	XM_006515787.3	145.00	-31.44	2 22	45 65	20
mmu-miR-5113	XM_006515787.3	141.00	-22.83	2 21	576 598	20
mmu-miR-5113	XM_006515787.3	140.00	-20.43	3 22	1001 1024	20
mmu-miR-5113	NM_178715.3	148.00	-17.88	2 22	2372 2395	21
mmu-miR-5113	NM_178715.3	144.00	-19.30	2 22	2969 2993	22
mmu-miR-5113	NM_178715.3	143.00	-19.41	2 20	157 179	18
mmu-miR-5113	NM_144936.1	156.00	-20.74	2 18	1490 1514	18
mmu-miR-5113	NM_177371.3	142.00	-16.67	2 19	1479 1501	17
mmu-miR-5113	XM_006496905.1	165.00	-28.44	2 20	54 75	18
mmu-miR-5113	XM_006529382.3	140.00	-25.63	4 22	91 114	19
mmu-miR-5113	XM_011248201.2	156.00	-24.78	2 22	2529 2552	21
mmu-miR-5113	XM_011248201.2	144.00	-15.69	2 21	1842 1862	19
mmu-miR-5113	NM_177409.3	140.00	-19.23	2 9	1234 1256	7
mmu-miR-5113	NM_146241.2	161.00	-21.30	2 20	2345 2366	18
mmu-miR-5113	NM_146241.2	149.00	-21.79	2 20	546 567	18
mmu-miR-5113	XM_011249157.2	152.00	-22.40	3 17	1607 1629	14
mmu-miR-5113	XM_011249157.2	152.00	-21.39	2 13	1720 1742	11
mmu-miR-5113	XM_011249157.2	149.00	-26.76	2 22	2947 2969	20
mmu-miR-5113	NM_001243916.1	155.00	-25.53	2 22	377 398	20
mmu-miR-5113	NM_001243916.1	145.00	-18.77	2 20	449 473	20
mmu-miR-5113	NM_001243916.1	145.00	-15.45	2 19	1504 1527	18
mmu-miR-5113	NM_001039047.1	140.00	-24.65	2 13	1009 1031	11
mmu-miR-5113	XM_006508002.3	157.00	-23.44	2 14	1554 1576	12
mmu-miR-5113	XM_006508002.3	141.00	-26.71	2 22	2915 2937	20
mmu-miR-5113	XM_017321602.1	141.00	-18.07	2 19	537 560	18
mmu-miR-5113	XM_006506152.3	149.00	-21.78	2 18	2098 2120	16
mmu-miR-5113	XM_006506152.3	146.00	-23.88	2 22	2655 2677	21
mmu-miR-5113	XM_006506152.3	140.00	-19.12	2 22	3122 3145	21
mmu-miR-5113	NM_023716.2	141.00	-21.65	3 18	1717 1739	15
mmu-miR-5113	NM_177709.3	158.00	-22.09	2 21	2269 2290	19
mmu-miR-5113	NM_011658.2	155.00	-21.84	2 22	235 256	20
mmu-miR-5113	NM_011658.2	146.00	-16.45	2 11	1431 1453	9
mmu-miR-5113	XM_006538224.1	161.00	-21.45	2 18	4068 4090	16
mmu-miR-5113	XM_006538224.1	156.00	-25.05	2 21	1638 1660	19
mmu-miR-5113	XM_006501309.3	142.00	-20.23	3 19	174 196	16
mmu-miR-5113	XM_006525099.3	151.00	-29.34	2 22	254 275	20
mmu-miR-5113	XM_006525099.3	143.00	-15.84	2 20	682 704	18
mmu-miR-5113	XM_011248394.1	151.00	-25.24	2 14	2178 2199	12
mmu-miR-5113	NM_009504.4	171.00	-30.87	2 21	1508 1531	20
mmu-miR-5113	NM_009504.4	161.00	-24.03	2 18	2798 2820	16
mmu-miR-5113	NM_009504.4	156.00	-25.11	2 21	1962 1982	19
mmu-miR-5113	NM_009504.4	153.00	-22.89	3 22	2301 2323	19
mmu-miR-5113	NM_009504.4	148.00	-21.34	2 21	1431 1453	19
mmu-miR-5113	XM_006525028.2	148.00	-22.45	3 22	130 153	20
mmu-miR-5113	NM_009514.4	170.00	-27.79	2 20	488 511	19
mmu-miR-5113	NM_177789.4	144.00	-26.08	3 22	1314 1337	20
mmu-miR-5113	XM_006541047.3	140.00	-17.36	2 9	1800 1822	7
mmu-miR-5113	NM_009518.2	145.00	-17.11	2 10	1404 1426	8
mmu-miR-5113	XM_006533569.3	142.00	-17.69	3 21	746 771	21
mmu-miR-5113	XM_006499466.3	162.00	-27.92	2 20	421 445	20
mmu-miR-5113	XM_006499466.3	158.00	-22.14	2 21	843 867	21
mmu-miR-5113	XM_006499466.3	156.00	-20.59	2 18	998 1021	17
mmu-miR-5113	XM_006530585.3	155.00	-20.99	2 22	4200 4221	20
mmu-miR-5113	XM_006530585.3	148.00	-27.38	3 22	5063 5087	21
mmu-miR-5113	XM_006530585.3	147.00	-20.37	3 18	11050 11071	15
mmu-miR-5113	XM_006530585.3	140.00	-17.14	3 21	12824 12846	18
mmu-miR-5113	XM_006530585.3	140.00	-21.04	2 22	13017 13040	21
mmu-miR-5113	NM_199304.1	140.00	-19.56	2 22	151 169	20
mmu-miR-5113	NM_199029.2	141.00	-21.49	3 18	2068 2090	15
mmu-miR-5113	XM_011243586.2	140.00	-18.60	2 9	5423 5445	7
mmu-miR-5113	XM_006511007.3	153.00	-19.97	2 22	1718 1740	20
mmu-miR-5113	XM_006511007.3	149.00	-23.78	2 22	4277 4296	20
mmu-miR-5113	XM_006511007.3	149.00	-18.05	2 19	4786 4810	19
mmu-miR-5113	XM_006537508.2	150.00	-21.34	2 11	1340 1362	9
mmu-miR-5113	XM_006519134.3	159.00	-27.23	2 22	5207 5228	20

mmu-miR-5113	XM_006519134.3	146.00	-23.80	2 17	5582 5603	15
mmu-miR-5113	XM_006519134.3	140.00	-18.78	2 13	4877 4899	11
mmu-miR-5128	NM_133167.3	141.00	-24.73	2 22	3510 3532	20
mmu-miR-5128	NM_001290822.1	141.00	-23.88	2 18	1113 1135	16
mmu-miR-5128	XM_017314870.1	145.00	-23.48	2 22	5551 5573	20
mmu-miR-5128	NM_018807.5	143.00	-23.21	3 22	2065 2086	19
mmu-miR-5128	XM_017312497.1	154.00	-25.04	2 22	4296 4316	20
mmu-miR-5128	XM_006502235.3	156.00	-35.04	2 21	1243 1265	19
mmu-miR-5128	XM_006502235.3	149.00	-15.85	2 20	27 48	18
mmu-miR-5128	NM_001291068.1	154.00	-22.26	2 21	5737 5758	19
mmu-miR-5128	XM_006527008.2	157.00	-21.61	2 20	583 604	18
mmu-miR-5128	XM_006527008.2	140.00	-23.66	2 20	2298 2320	19
mmu-miR-5128	NM_011169.5	153.00	-21.34	2 22	1919 1941	20
mmu-miR-5128	NM_011169.5	152.00	-19.18	2 13	965 987	11
mmu-miR-5128	NM_011169.5	141.00	-18.30	3 22	3087 3109	19
mmu-miR-5128	NM_175563.5	145.00	-24.07	2 19	3264 3289	20
mmu-miR-5128	NM_175563.5	144.00	-15.11	2 22	1399 1422	21
mmu-miR-5128	NM_175022.2	149.00	-23.36	2 22	317 339	20
mmu-miR-5128	NM_001081224.2	163.00	-22.35	2 22	791 812	20
mmu-miR-5128	NM_008966.3	147.00	-22.81	2 22	4241 4266	23
mmu-miR-5128	XM_006514153.2	148.00	-23.86	3 22	3751 3775	21
mmu-miR-5128	XM_006514153.2	147.00	-14.89	2 16	3523 3545	14
mmu-miR-5128	XM_006514153.2	145.00	-23.02	2 18	1975 1997	16
mmu-miR-5128	XM_006514153.2	140.00	-17.12	2 22	2087 2111	22
mmu-miR-5128	NM_009019.2	149.00	-13.79	2 14	2046 2068	12
mmu-miR-5128	XM_006506624.2	147.00	-19.05	2 22	469 493	22
mmu-miR-5128	XM_017320735.1	168.00	-23.89	2 22	365 388	21
mmu-miR-5128	XM_006507942.2	141.00	-19.52	2 22	2304 2326	20
mmu-miR-5128	NM_001190258.1	155.00	-25.09	2 12	99 121	10
mmu-miR-5128	NM_023893.4	147.00	-23.97	2 18	609 630	16
mmu-miR-5128	XM_006540138.3	140.00	-29.54	4 21	1014 1036	17
mmu-miR-5128	XM_006540134.3	147.00	-19.22	2 12	1221 1243	10
mmu-miR-5128	NM_018732.3	142.00	-19.81	2 22	5534 5554	20
mmu-miR-5128	XM_011244046.2	142.00	-26.29	2 22	1654 1681	25
mmu-miR-5128	XM_017322211.1	143.00	-20.11	2 22	6763 6787	22
mmu-miR-5128	XM_006530238.3	143.00	-25.34	3 22	812 833	19
mmu-miR-5128	XM_017316715.1	153.00	-18.26	2 15	557 580	14
mmu-miR-5128	XM_017316715.1	145.00	-14.02	2 10	3935 3957	8
mmu-miR-5128	XM_017317034.1	149.00	-21.37	2 22	2294 2316	20
mmu-miR-5128	XM_011250732.2	153.00	-28.94	2 22	4910 4932	20
mmu-miR-5128	XM_011250732.2	142.00	-20.20	3 22	3288 3308	19
mmu-miR-5128	NM_177578.4	161.00	-19.77	2 22	898 920	20
mmu-miR-5128	NM_001033167.3	150.00	-23.26	2 22	740 762	21
mmu-miR-5128	NM_001033167.3	146.00	-17.75	2 15	2833 2855	13
mmu-miR-5128	NM_001007570.2	147.00	-20.15	2 22	957 978	20
mmu-miR-5128	NM_027052.3	143.00	-20.45	3 21	1735 1758	19
mmu-miR-5128	NM_027052.3	140.00	-22.95	2 21	2107 2130	20
mmu-miR-5128	XM_017322365.1	143.00	-23.62	2 20	434 456	18
mmu-miR-5128	XM_006538687.2	140.00	-19.10	2 22	985 1008	21
mmu-miR-5128	NM_001146217.1	140.00	-29.59	2 22	271 294	21
mmu-miR-5128	NM_009223.3	148.00	-28.46	2 18	960 983	17
mmu-miR-5128	NM_009223.3	140.00	-22.34	2 22	151 174	21
mmu-miR-5128	XM_006534520.3	149.00	-24.34	2 22	2942 2964	20
mmu-miR-5128	XM_006534520.3	142.00	-20.71	3 21	2768 2789	18
mmu-miR-5128	NM_001304266.1	155.00	-21.30	2 16	9487 9509	14
mmu-miR-5128	NM_001304266.1	148.00	-26.52	2 22	3994 4017	21
mmu-miR-5128	NM_001304266.1	148.00	-14.33	2 14	8806 8829	13
mmu-miR-5128	NM_001304266.1	145.00	-20.56	2 22	5618 5640	20
mmu-miR-5128	NM_001304266.1	144.00	-28.35	2 22	8347 8370	21
mmu-miR-5128	NM_001304266.1	140.00	-22.85	3 19	657 681	18
mmu-miR-5128	XM_006506145.1	144.00	-21.79	3 22	6744 6768	21
mmu-miR-5128	XM_011248193.2	144.00	-22.58	3 21	2252 2274	18
mmu-miR-5128	XM_011245529.2	147.00	-17.41	2 12	2916 2938	10
mmu-miR-5128	XM_011245529.2	141.00	-14.55	2 21	2170 2190	19
mmu-miR-5128	XM_011245529.2	141.00	-20.29	2 22	4092 4114	20

mmu-miR-5128	NM_011374.2	140.00	-21.94	2 21	1423 1445	19
mmu-miR-5128	NM_009332.3	148.00	-35.56	2 22	2240 2263	21
mmu-miR-5128	NM_009332.3	147.00	-23.84	3 22	2456 2477	19
mmu-miR-5128	NM_001080979.1	142.00	-24.11	2 21	2607 2628	19
mmu-miR-5128	NM_153801.3	141.00	-19.14	2 22	1030 1052	20
mmu-miR-5128	NM_011581.3	148.00	-22.24	2 22	1846 1870	22
mmu-miR-5128	NM_009379.3	143.00	-19.23	3 21	807 831	20
mmu-miR-5128	XM_006501237.3	147.00	-12.77	2 20	872 894	18
mmu-miR-5128	XM_006533479.2	148.00	-16.46	2 14	188 211	13
mmu-miR-5128	NM_178715.3	143.00	-12.00	2 12	2161 2183	10
mmu-miR-5128	NM_178715.3	142.00	-23.11	2 22	1369 1392	22
mmu-miR-5128	NM_146241.2	142.00	-13.80	2 21	5699 5720	19
mmu-miR-5128	XM_011249157.2	149.00	-17.58	2 22	1892 1913	21
mmu-miR-5128	XM_006508002.3	149.00	-23.97	3 20	2890 2911	17
mmu-miR-5128	XM_006506152.3	144.00	-30.43	4 22	3362 3386	20
mmu-miR-5128	NM_177709.3	147.00	-23.47	2 19	3005 3027	18
mmu-miR-5128	XM_006538224.1	141.00	-23.32	2 18	4411 4433	16
mmu-miR-5128	XM_006525028.2	142.00	-16.74	2 22	1221 1241	20
mmu-miR-5128	NM_011704.3	143.00	-13.42	2 17	1103 1122	15
mmu-miR-5128	XM_006499466.3	153.00	-25.38	2 22	335 354	20
mmu-miR-5128	XM_006499466.3	141.00	-17.98	3 22	315 337	19
mmu-miR-5128	XM_006530585.3	148.00	-21.57	2 22	7804 7827	21
mmu-miR-5128	XM_006530585.3	146.00	-19.09	2 22	12363 12383	20
mmu-miR-5128	NM_199304.1	147.00	-15.16	2 21	502 525	20
mmu-miR-5128	NM_199304.1	142.00	-33.98	3 22	2503 2525	20
mmu-miR-5128	NM_199029.2	143.00	-30.00	2 22	796 821	23
mmu-miR-5128	NM_001110508.1	162.00	-27.39	2 22	252 274	21
mmu-miR-5128	XM_006519134.3	141.00	-31.42	2 22	2806 2828	20
mmu-miR-344i	NM_028748.2	154.00	-24.95	2 19	2674 2693	17
mmu-miR-344i	NM_133167.3	145.00	-20.88	2 18	1960 1979	16
mmu-miR-344i	NM_133167.3	140.00	-13.64	2 9	938 957	7
mmu-miR-344i	NM_008804.4	141.00	-20.87	3 19	1204 1225	18
mmu-miR-344i	NM_013743.2	140.00	-12.92	2 9	1589 1608	7
mmu-miR-344i	XM_006538456.3	146.00	-22.12	2 19	6224 6243	17
mmu-miR-344i	XM_006537815.3	164.00	-25.52	2 19	1575 1593	17
mmu-miR-344i	XM_006537815.3	145.00	-25.59	2 19	910 930	18
mmu-miR-344i	XM_006537815.3	140.00	-13.64	2 9	153 172	7
mmu-miR-344i	NM_011082.3	142.00	-29.28	2 19	559 578	17
mmu-miR-344i	XM_017314870.1	149.00	-16.26	2 18	7401 7420	16
mmu-miR-344i	XM_017314870.1	140.00	-18.71	2 9	1903 1922	7
mmu-miR-344i	NM_018807.5	140.00	-15.85	2 9	4567 4586	7
mmu-miR-344i	NM_008872.3	149.00	-18.47	2 19	1426 1448	20
mmu-miR-344i	XM_017312497.1	150.00	-21.26	2 16	2111 2131	15
mmu-miR-344i	XM_017312497.1	141.00	-23.18	3 19	1057 1077	17
mmu-miR-344i	XM_011250776.2	148.00	-19.62	2 17	386 405	15
mmu-miR-344i	NM_001195084.1	140.00	-13.90	2 9	279 298	7
mmu-miR-344i	XM_006512076.3	147.00	-13.76	2 16	3963 3982	14
mmu-miR-344i	XM_006512076.3	142.00	-23.78	2 16	2894 2915	16
mmu-miR-344i	NM_001302257.1	147.00	-26.69	2 16	26 45	14
mmu-miR-344i	NM_001291068.1	143.00	-22.05	2 18	1221 1242	18
mmu-miR-344i	NM_148932.2	149.00	-28.28	2 19	4826 4846	18
mmu-miR-344i	NM_148932.2	141.00	-24.43	2 19	200 221	19
mmu-miR-344i	XM_006525698.2	148.00	-20.89	2 18	8397 8418	18
mmu-miR-344i	XM_006525698.2	147.00	-20.81	2 16	5223 5242	14
mmu-miR-344i	XM_006525698.2	145.00	-22.87	2 18	2645 2664	16
mmu-miR-344i	XM_006527008.2	146.00	-15.69	2 19	1890 1909	17
mmu-miR-344i	XM_006527008.2	141.00	-21.03	3 18	115 134	15
mmu-miR-344i	NM_011169.5	155.00	-22.78	2 16	1117 1136	14
mmu-miR-344i	NM_011169.5	146.00	-28.08	2 19	10278 10297	17
mmu-miR-344i	NM_011169.5	141.00	-19.34	2 18	3958 3977	16
mmu-miR-344i	NM_175563.5	148.00	-17.90	2 18	1185 1205	17
mmu-miR-344i	NM_001081224.2	150.00	-14.93	2 19	673 692	17
mmu-miR-344i	NM_001081224.2	146.00	-16.57	2 19	360 379	17
mmu-miR-344i	XM_011240476.2	147.00	-14.37	2 16	1 19	14
mmu-miR-344i	XM_011240476.2	140.00	-17.49	2 9	2349 2368	7

mmu-miR-344i	NM_008966.3	148.00	-18.66	2 14	1740 1760	13
mmu-miR-344i	NM_008966.3	143.00	-23.53	2 19	2831 2850	18
mmu-miR-344i	XM_006505015.3	150.00	-22.86	3 19	3518 3537	16
mmu-miR-344i	XM_006505015.3	140.00	-15.31	2 9	2101 2120	7
mmu-miR-344i	XM_006505015.3	140.00	-16.99	2 9	2740 2759	7
mmu-miR-344i	XM_006514153.2	151.00	-24.82	2 16	2182 2201	14
mmu-miR-344i	NM_009019.2	140.00	-15.73	2 9	5378 5397	7
mmu-miR-344i	NM_009020.3	144.00	-21.39	2 19	1106 1131	23
mmu-miR-344i	NM_016809.6	140.00	-18.89	2 18	2179 2200	18
mmu-miR-344i	XM_006514077.1	141.00	-28.13	2 19	1213 1234	19
mmu-miR-344i	XM_006529829.3	146.00	-19.61	2 18	763 780	16
mmu-miR-344i	XM_011240779.2	141.00	-13.48	2 11	3119 3139	10
mmu-miR-344i	XM_006507942.2	145.00	-17.80	2 16	1549 1570	16
mmu-miR-344i	NM_001195662.1	140.00	-13.41	2 9	1521 1540	7
mmu-miR-344i	NM_019732.2	164.00	-31.23	2 19	2735 2753	17
mmu-miR-344i	NM_019732.2	140.00	-16.52	2 9	51 70	7
mmu-miR-344i	XM_006540138.3	141.00	-22.40	2 19	1268 1289	19
mmu-miR-344i	XM_006540134.3	149.00	-21.46	2 19	502 522	18
mmu-miR-344i	NM_009127.4	146.00	-27.18	2 19	1269 1288	17
mmu-miR-344i	NM_018732.3	150.00	-22.69	3 19	2251 2270	16
mmu-miR-344i	NM_018732.3	145.00	-14.45	2 18	716 735	16
mmu-miR-344i	NM_018732.3	141.00	-25.41	2 19	3448 3468	18
mmu-miR-344i	NM_018732.3	140.00	-16.34	2 9	5291 5310	7
mmu-miR-344i	NM_009135.2	158.00	-26.60	2 19	4548 4567	17
mmu-miR-344i	NM_020052.2	142.00	-20.34	2 17	2530 2548	15
mmu-miR-344i	NM_009246.3	141.00	-21.81	2 15	1395 1416	15
mmu-miR-344i	XM_006530238.3	146.00	-19.54	2 19	1791 1808	17
mmu-miR-344i	XM_017316715.1	159.00	-17.76	2 16	576 595	14
mmu-miR-344i	XM_017316715.1	145.00	-18.85	2 10	765 784	8
mmu-miR-344i	XM_017316715.1	144.00	-25.16	3 19	789 807	16
mmu-miR-344i	XM_006496884.3	144.00	-23.36	2 18	2346 2366	17
mmu-miR-344i	NM_001310705.1	141.00	-25.40	2 19	1783 1805	20
mmu-miR-344i	NM_001033167.3	149.00	-19.35	2 14	245 264	12
mmu-miR-344i	NM_001033167.3	142.00	-20.70	2 19	1928 1947	17
mmu-miR-344i	NM_001007570.2	155.00	-22.97	2 18	1129 1147	16
mmu-miR-344i	NM_001007570.2	153.00	-27.61	2 19	2033 2053	18
mmu-miR-344i	NM_001007570.2	145.00	-23.66	2 19	196 216	18
mmu-miR-344i	NM_001033286.2	141.00	-21.60	2 19	732 752	18
mmu-miR-344i	XM_006503080.3	149.00	-20.17	2 19	784 804	18
mmu-miR-344i	NM_027052.3	140.00	-18.70	3 18	263 284	17
mmu-miR-344i	XM_017322365.1	147.00	-26.61	2 16	484 503	14
mmu-miR-344i	XM_017322365.1	143.00	-20.87	2 16	538 557	14
mmu-miR-344i	XM_006538687.2	144.00	-23.63	2 19	298 316	17
mmu-miR-344i	XM_006500109.3	155.00	-21.85	2 17	523 543	16
mmu-miR-344i	NM_009223.3	142.00	-13.41	2 11	1761 1780	9
mmu-miR-344i	XM_006534520.3	142.00	-15.28	2 11	2112 2131	9
mmu-miR-344i	XM_006539074.3	149.00	-17.39	2 16	8306 8324	14
mmu-miR-344i	XM_006539074.3	141.00	-22.55	3 18	5789 5808	15
mmu-miR-344i	XM_006539074.3	140.00	-17.62	2 9	10222 10241	7
mmu-miR-344i	NM_019866.1	145.00	-25.49	2 19	126 146	18
mmu-miR-344i	NM_178753.4	140.00	-13.64	2 9	1947 1966	7
mmu-miR-344i	NM_001304266.1	152.00	-20.56	2 16	10499 10516	14
mmu-miR-344i	NM_001304266.1	150.00	-17.59	2 19	6723 6742	17
mmu-miR-344i	NM_001304266.1	142.00	-25.40	2 19	9819 9838	17
mmu-miR-344i	NM_001242411.1	143.00	-27.42	3 18	2790 2811	17
mmu-miR-344i	XM_011248193.2	150.00	-17.95	2 19	7867 7886	17
mmu-miR-344i	XM_011245529.2	145.00	-25.07	2 19	5162 5183	19
mmu-miR-344i	XM_011245529.2	140.00	-24.24	2 19	2847 2869	20
mmu-miR-344i	NM_011374.2	142.00	-14.86	2 11	3645 3664	9
mmu-miR-344i	NM_032400.2	152.00	-25.99	2 19	604 622	17
mmu-miR-344i	XM_017316056.1	149.00	-20.06	2 14	1093 1112	12
mmu-miR-344i	XM_017316056.1	145.00	-19.99	2 10	2451 2470	8
mmu-miR-344i	NM_009332.3	140.00	-14.17	2 9	2206 2225	7
mmu-miR-344i	NM_001080979.1	160.00	-25.87	2 18	2779 2800	18
mmu-miR-344i	NM_011581.3	141.00	-24.88	3 19	518 539	18

mmu-miR-344i	XM_006533479.2	141.00	-19.82	2 14	363 382	12
mmu-miR-344i	XM_006533452.3	140.00	-13.05	2 9	2600 2619	7
mmu-miR-344i	XM_006515787.3	145.00	-23.57	2 16	495 513	14
mmu-miR-344i	XM_006515787.3	141.00	-25.97	2 19	877 899	20
mmu-miR-344i	XM_006496905.1	144.00	-16.77	2 19	1988 2006	17
mmu-miR-344i	XM_011248201.2	142.00	-33.22	4 19	4228 4247	15
mmu-miR-344i	XM_011248201.2	140.00	-14.86	2 9	1473 1492	7
mmu-miR-344i	XM_011249157.2	148.00	-23.95	2 18	540 560	17
mmu-miR-344i	XM_006508002.3	154.00	-17.63	2 19	582 601	17
mmu-miR-344i	NM_001033149.3	141.00	-25.99	2 19	541 561	18
mmu-miR-344i	NM_001033149.3	140.00	-16.54	2 9	2898 2917	7
mmu-miR-344i	NM_009504.4	140.00	-19.86	2 9	2710 2729	7
mmu-miR-344i	NM_177789.4	141.00	-14.18	2 18	242 261	16
mmu-miR-344i	XM_006541047.3	145.00	-14.59	2 18	1565 1584	16
mmu-miR-344i	XM_006541047.3	144.00	-14.49	2 19	861 882	19
mmu-miR-344i	XM_006533569.3	156.00	-20.80	2 18	1039 1060	18
mmu-miR-344i	NM_011724.3	147.00	-20.06	2 19	1653 1673	19
mmu-miR-344i	XM_006499466.3	157.00	-29.11	2 19	1246 1266	18
mmu-miR-344i	XM_006499466.3	157.00	-24.08	2 16	1682 1703	16
mmu-miR-344i	XM_006530585.3	158.00	-20.89	2 19	3958 3977	17
mmu-miR-344i	XM_006530585.3	152.00	-22.87	2 19	8353 8371	17
mmu-miR-344i	XM_006530585.3	148.00	-25.99	2 19	4342 4363	19
mmu-miR-344i	XM_006511007.3	148.00	-23.69	2 19	6129 6152	21
mmu-miR-344i	NM_001110508.1	142.00	-31.42	3 19	1826 1845	16
mmu-miR-344i	NM_001110508.1	142.00	-20.20	3 19	2776 2795	16
mmu-miR-344i	XM_006537508.2	142.00	-25.81	2 16	3989 4010	16
mmu-miR-6238	NM_172454.2	149.00	-19.83	2 21	1158 1177	19
mmu-miR-6238	NM_001164593.1	155.00	-20.10	2 20	3322 3343	18
mmu-miR-6238	XM_011239558.2	142.00	-16.07	3 19	2932 2953	16
mmu-miR-6238	NM_009019.2	156.00	-12.69	2 21	5118 5139	19
mmu-miR-6238	NM_009019.2	147.00	-11.58	2 16	5179 5200	14
mmu-miR-6238	XM_006506624.2	148.00	-17.58	2 21	693 714	19
mmu-miR-6238	NM_009127.4	150.00	-14.05	2 21	3842 3866	22
mmu-miR-6238	NM_018732.3	140.00	-11.93	2 21	8839 8860	19
mmu-miR-6238	NM_009135.2	140.00	-17.12	2 21	3197 3218	19
mmu-miR-6238	NM_177578.4	147.00	-18.55	2 21	3371 3396	23
mmu-miR-6238	XM_006496884.3	143.00	-7.29	2 20	2718 2739	18
mmu-miR-6238	NM_001310705.1	145.00	-9.49	2 17	1441 1463	17
mmu-miR-6238	NM_001033167.3	142.00	-16.02	2 19	1759 1780	17
mmu-miR-6238	NM_001290993.1	164.00	-21.66	2 19	1518 1538	17
mmu-miR-6238	NM_175692.3	143.00	-12.39	2 21	3414 3437	21
mmu-miR-6238	NM_178753.4	143.00	-11.63	2 20	2344 2365	18
mmu-miR-6238	XM_006527452.3	148.00	-14.62	2 21	2176 2197	19
mmu-miR-6238	XM_006527452.3	141.00	-12.39	2 18	2312 2333	16
mmu-miR-6238	NM_153801.3	140.00	-13.67	2 21	1839 1857	19
mmu-miR-6238	XM_006501237.3	141.00	-13.82	2 20	1965 1985	18
mmu-miR-6238	XM_006515787.3	157.00	-13.22	2 14	1657 1678	12
mmu-miR-6238	XM_006521428.3	140.00	-16.45	2 17	297 318	15
mmu-miR-6238	NM_177371.3	148.00	-6.10	2 13	5714 5735	11
mmu-miR-6238	NM_146241.2	143.00	-19.15	2 21	5176 5198	20
mmu-miR-6238	NM_146241.2	141.00	-9.23	2 18	3130 3151	16
mmu-miR-6238	NM_001243916.1	147.00	-15.28	2 16	268 289	14
mmu-miR-6238	XM_017321602.1	156.00	-15.49	2 21	2228 2249	19
mmu-miR-6238	XM_006501309.3	144.00	-11.33	2 21	2209 2230	19
mmu-miR-6238	XM_011248394.1	147.00	-10.99	2 18	1769 1789	16
mmu-miR-6238	XM_006537508.2	140.00	-10.72	2 21	5353 5374	19
mmu-miR-6351	NM_028748.2	152.00	-19.75	2 22	2941 2965	22
mmu-miR-6351	NM_013743.2	146.00	-30.30	3 19	146 168	16
mmu-miR-6351	NM_011082.3	145.00	-19.00	2 19	357 381	19
mmu-miR-6351	XM_017314870.1	143.00	-24.57	2 22	2758 2779	20
mmu-miR-6351	XM_017317946.1	155.00	-27.08	2 20	835 857	18
mmu-miR-6351	NM_148932.2	143.00	-23.28	3 19	1460 1480	16
mmu-miR-6351	NM_011145.3	142.00	-18.03	2 19	677 699	17
mmu-miR-6351	XM_006525698.2	140.00	-32.00	2 21	2608 2630	19
mmu-miR-6351	XM_006527008.2	146.00	-24.77	2 19	27 49	17

mmu-miR-6351	NM_011169.5	151.00	-24.19	2 21	3634 3657	20
mmu-miR-6351	XM_011240476.2	149.00	-18.87	2 22	2754 2776	20
mmu-miR-6351	XM_006505757.3	140.00	-20.71	2 21	385 407	19
mmu-miR-6351	NM_016933.3	151.00	-32.22	2 21	582 608	23
mmu-miR-6351	XM_006514153.2	144.00	-18.26	3 21	2772 2794	18
mmu-miR-6351	NM_019732.2	157.00	-21.44	2 14	857 879	12
mmu-miR-6351	XM_006540134.3	142.00	-21.13	2 16	223 247	16
mmu-miR-6351	NM_009127.4	149.00	-15.86	2 22	603 625	20
mmu-miR-6351	NM_018732.3	150.00	-19.59	2 19	3985 4007	17
mmu-miR-6351	NM_018732.3	142.00	-11.28	2 11	6439 6461	9
mmu-miR-6351	NM_009135.2	140.00	-17.49	2 9	2199 2221	7
mmu-miR-6351	NM_020052.2	144.00	-21.86	2 22	2240 2264	22
mmu-miR-6351	XM_017322211.1	156.00	-19.21	2 22	2777 2800	21
mmu-miR-6351	XM_017322211.1	144.00	-32.71	2 21	5877 5899	19
mmu-miR-6351	XM_006530238.3	144.00	-23.49	3 22	1581 1604	20
mmu-miR-6351	XM_006530238.3	141.00	-19.26	2 19	2009 2032	18
mmu-miR-6351	XM_011250732.2	145.00	-17.50	2 18	4186 4208	16
mmu-miR-6351	XM_006518339.3	148.00	-20.50	2 20	745 765	18
mmu-miR-6351	XM_006518339.3	143.00	-13.03	2 20	2986 3008	18
mmu-miR-6351	NM_144539.5	147.00	-24.36	2 22	1137 1158	20
mmu-miR-6351	NM_001033286.2	153.00	-20.90	3 22	453 475	19
mmu-miR-6351	NM_027052.3	150.00	-25.62	3 15	361 383	12
mmu-miR-6351	XM_006538687.2	161.00	-27.77	2 19	3364 3390	21
mmu-miR-6351	NM_025540.2	148.00	-26.42	2 22	269 295	24
mmu-miR-6351	XM_006534520.3	144.00	-22.66	2 21	3270 3292	19
mmu-miR-6351	NM_178753.4	154.00	-23.28	2 20	1889 1916	23
mmu-miR-6351	NM_001304266.1	152.00	-21.75	2 18	3251 3274	17
mmu-miR-6351	XM_011248193.2	144.00	-24.65	2 19	3877 3903	21
mmu-miR-6351	XM_011245529.2	142.00	-18.32	2 20	1369 1393	20
mmu-miR-6351	XM_006527452.3	149.00	-18.45	2 14	1969 1991	12
mmu-miR-6351	NM_011581.3	144.00	-28.84	3 22	3226 3250	21
mmu-miR-6351	XM_006533452.3	146.00	-24.46	2 19	824 844	17
mmu-miR-6351	XM_006533452.3	140.00	-15.38	3 21	4929 4951	18
mmu-miR-6351	XM_006496905.1	142.00	-15.39	2 11	3982 4004	9
mmu-miR-6351	XM_006496905.1	140.00	-22.95	2 20	922 942	18
mmu-miR-6351	XM_011249157.2	146.00	-29.69	2 20	571 594	19
mmu-miR-6351	XM_006501309.3	159.00	-21.27	2 21	767 790	20
mmu-miR-6351	XM_006525099.3	145.00	-20.30	2 22	1815 1837	20
mmu-miR-6351	XM_011248394.1	147.00	-24.26	2 22	1556 1580	22
mmu-miR-6351	NM_009504.4	156.00	-24.47	2 21	233 255	19
mmu-miR-6351	NM_016982.2	143.00	-27.31	4 20	261 283	16
mmu-miR-6351	XM_017316944.1	143.00	-27.31	4 20	601 623	16
mmu-miR-6351	NM_009518.2	146.00	-23.59	2 19	444 466	17
mmu-miR-6351	XM_006530585.3	166.00	-27.95	2 22	3217 3241	23
mmu-miR-6351	XM_006530585.3	151.00	-18.92	2 20	3813 3835	18
mmu-miR-6351	NM_199304.1	142.00	-20.43	2 22	873 895	21
mmu-miR-6351	NM_001110508.1	142.00	-19.61	2 21	3182 3203	19
mmu-miR-6351	XM_006537508.2	148.00	-30.88	3 21	6340 6362	18
mmu-miR-6378	NM_133167.3	146.00	-24.35	2 15	3548 3569	13
mmu-miR-6378	XM_006509465.2	150.00	-22.64	2 16	773 797	17
mmu-miR-6378	XM_006538456.3	147.00	-20.38	2 12	3707 3728	10
mmu-miR-6378	XM_006538456.3	140.00	-24.69	2 19	4480 4500	17
mmu-miR-6378	NM_011082.3	148.00	-22.43	2 18	3622 3644	17
mmu-miR-6378	NM_011082.3	140.00	-13.32	2 9	713 734	7
mmu-miR-6378	NM_011082.3	140.00	-10.71	2 9	2705 2726	7
mmu-miR-6378	NM_018807.5	146.00	-17.90	2 20	2321 2343	19
mmu-miR-6378	XM_006512076.3	146.00	-25.63	3 19	1763 1784	16
mmu-miR-6378	XM_006512076.3	144.00	-18.03	2 17	219 240	15
mmu-miR-6378	XM_006512076.3	140.00	-21.84	2 13	4343 4364	11
mmu-miR-6378	XM_006525698.2	143.00	-15.10	2 16	9990 10011	14
mmu-miR-6378	XM_006525698.2	141.00	-20.82	2 15	7969 7991	14
mmu-miR-6378	NM_011169.5	150.00	-17.47	2 16	3337 3359	15
mmu-miR-6378	NM_011169.5	144.00	-23.01	2 19	7531 7551	17
mmu-miR-6378	NM_001081224.2	143.00	-19.48	2 21	722 744	20
mmu-miR-6378	XM_011240476.2	141.00	-19.48	2 18	1198 1219	16

mmu-miR-6378	NM_008966.3	140.00	-16.46	2 9	3239 3260	7
mmu-miR-6378	XM_006529829.3	158.00	-22.62	2 19	2618 2639	17
mmu-miR-6378	NM_019732.2	140.00	-26.56	3 21	3438 3459	18
mmu-miR-6378	NM_009127.4	142.00	-21.38	2 20	2165 2187	19
mmu-miR-6378	NM_009135.2	145.00	-24.02	2 14	128 149	12
mmu-miR-6378	NM_026535.2	142.00	-16.81	2 11	1767 1788	9
mmu-miR-6378	XM_006515637.2	146.00	-17.35	2 15	1077 1098	13
mmu-miR-6378	XM_006530238.3	148.00	-26.66	2 18	7635 7658	18
mmu-miR-6378	XM_011250732.2	141.00	-16.87	2 20	6626 6646	18
mmu-miR-6378	XM_006518339.3	147.00	-20.69	2 20	2639 2660	18
mmu-miR-6378	NM_009199.2	151.00	-20.14	2 17	1502 1524	16
mmu-miR-6378	NM_001033167.3	149.00	-25.62	2 20	1660 1683	20
mmu-miR-6378	XM_006503080.3	141.00	-20.14	2 10	120 141	8
mmu-miR-6378	XM_006538687.2	142.00	-21.69	2 15	1701 1722	13
mmu-miR-6378	NM_031183.2	166.00	-27.53	2 15	2882 2903	13
mmu-miR-6378	NM_031183.2	153.00	-18.55	2 14	3284 3305	12
mmu-miR-6378	NM_019866.1	156.00	-18.18	2 21	1545 1566	19
mmu-miR-6378	NM_001304266.1	144.00	-20.95	2 21	6997 7018	19
mmu-miR-6378	NM_001304266.1	143.00	-15.53	2 12	434 455	10
mmu-miR-6378	NM_001242411.1	151.00	-20.08	2 20	397 418	18
mmu-miR-6378	XM_006506145.1	152.00	-28.53	2 17	3144 3165	15
mmu-miR-6378	XM_006506145.1	147.00	-26.24	3 20	7546 7567	17
mmu-miR-6378	XM_006506145.1	144.00	-27.16	3 21	565 586	18
mmu-miR-6378	XM_006506145.1	142.00	-21.08	2 15	1220 1241	13
mmu-miR-6378	XM_006506145.1	140.00	-12.89	2 9	8255 8276	7
mmu-miR-6378	NM_011374.2	141.00	-13.77	2 10	2981 3002	8
mmu-miR-6378	XM_006527452.3	157.00	-27.56	3 18	1527 1548	15
mmu-miR-6378	NM_001080979.1	142.00	-18.01	2 21	1921 1941	19
mmu-miR-6378	NM_011581.3	141.00	-17.24	2 15	1234 1256	14
mmu-miR-6378	XM_006533452.3	147.00	-23.61	2 16	3028 3049	14
mmu-miR-6378	XM_006533452.3	143.00	-15.38	2 16	5783 5804	14
mmu-miR-6378	XM_006515787.3	156.00	-20.53	2 17	547 568	15
mmu-miR-6378	NM_178715.3	145.00	-21.21	3 18	1695 1716	15
mmu-miR-6378	XM_006496905.1	140.00	-12.89	2 9	4983 5004	7
mmu-miR-6378	XM_011249157.2	141.00	-26.41	3 19	1977 2003	21
mmu-miR-6378	XM_006538224.1	157.00	-22.84	2 20	4049 4072	20
mmu-miR-6378	XM_006538224.1	140.00	-20.36	2 17	1974 1995	15
mmu-miR-6378	XM_006501309.3	142.00	-15.99	2 11	1838 1859	9
mmu-miR-6378	XM_006525099.3	143.00	-19.77	2 16	2833 2854	14
mmu-miR-6378	XM_011248394.1	163.00	-24.24	2 20	1553 1574	18
mmu-miR-6378	XM_011248394.1	156.00	-26.05	2 21	3579 3600	19
mmu-miR-6378	XM_011248394.1	140.00	-17.71	2 9	1015 1036	7
mmu-miR-6378	XM_006530585.3	149.00	-19.56	2 14	3824 3845	12
mmu-miR-6378	XM_006530585.3	146.00	-20.08	2 15	10646 10667	13
mmu-miR-6378	XM_006530585.3	143.00	-28.71	2 21	658 680	20
mmu-miR-6378	NM_199304.1	146.00	-23.17	3 19	1711 1732	16
mmu-miR-6378	NM_199029.2	151.00	-27.62	2 20	3790 3811	18
mmu-miR-6378	NM_199029.2	146.00	-20.47	2 11	3473 3494	9
mmu-miR-6378	XM_006537508.2	151.00	-26.29	2 21	5732 5754	20
mmu-miR-6378	XM_006537508.2	149.00	-16.91	2 14	4291 4312	12
mmu-miR-6378	XM_006537508.2	144.00	-15.59	2 13	1504 1525	11
mmu-miR-3473e	NM_028748.2	151.00	-25.31	2 16	562 582	14
mmu-miR-3473e	NM_028748.2	145.00	-22.30	2 15	1098 1119	14
mmu-miR-3473e	NM_133167.3	167.00	-27.23	2 16	3538 3558	14
mmu-miR-3473e	NM_133167.3	150.00	-19.33	2 17	675 694	15
mmu-miR-3473e	NM_133167.3	150.00	-24.30	2 16	1484 1507	17
mmu-miR-3473e	NM_011044.2	143.00	-23.82	2 18	2072 2094	18
mmu-miR-3473e	NM_001290822.1	146.00	-21.36	2 20	1214 1237	21
mmu-miR-3473e	NM_001164593.1	141.00	-23.56	2 20	1192 1211	18
mmu-miR-3473e	NM_001164593.1	141.00	-23.43	3 20	1447 1469	19
mmu-miR-3473e	XM_006538456.3	150.00	-23.05	2 20	1385 1406	19
mmu-miR-3473e	XM_006538456.3	146.00	-24.58	2 20	1685 1708	21
mmu-miR-3473e	XM_006538456.3	141.00	-25.03	2 19	2410 2431	18
mmu-miR-3473e	XM_006537815.3	155.00	-25.03	2 16	2993 3013	14
mmu-miR-3473e	XM_006537815.3	151.00	-18.63	2 18	6351 6370	16

mmu-miR-3473e	XM_006537815.3	147.00	-25.14	2 19	3582 3607	22
mmu-miR-3473e	XM_006537815.3	146.00	-26.40	2 17	5073 5092	15
mmu-miR-3473e	XM_006537815.3	141.00	-21.91	2 18	5384 5404	16
mmu-miR-3473e	XM_017314870.1	157.00	-25.38	2 20	1152 1175	21
mmu-miR-3473e	XM_017314870.1	140.00	-16.99	2 9	3947 3967	7
mmu-miR-3473e	XM_017317946.1	151.00	-24.70	2 20	563 583	18
mmu-miR-3473e	XM_017317946.1	142.00	-20.20	2 20	80 96	18
mmu-miR-3473e	NM_018807.5	152.00	-25.79	2 18	3904 3926	18
mmu-miR-3473e	NM_008872.3	142.00	-15.56	2 15	1530 1550	13
mmu-miR-3473e	XM_017312497.1	155.00	-27.67	2 20	312 332	18
mmu-miR-3473e	XM_011250776.2	140.00	-29.68	2 19	920 939	17
mmu-miR-3473e	NM_026385.4	141.00	-21.09	3 19	684 705	17
mmu-miR-3473e	XM_006502235.3	146.00	-19.14	2 20	4212 4233	19
mmu-miR-3473e	XM_006502235.3	145.00	-19.45	2 18	1803 1823	16
mmu-miR-3473e	XM_006502235.3	141.00	-22.41	3 20	3955 3974	17
mmu-miR-3473e	NM_001195084.1	147.00	-23.70	2 17	202 223	16
mmu-miR-3473e	XM_006512076.3	153.00	-21.83	2 18	3066 3086	16
mmu-miR-3473e	XM_006512076.3	148.00	-31.28	2 17	2092 2112	15
mmu-miR-3473e	XM_006512076.3	146.00	-20.31	2 17	380 404	19
mmu-miR-3473e	XM_006512076.3	143.00	-18.38	2 14	1988 2007	12
mmu-miR-3473e	XM_006512076.3	140.00	-19.83	2 9	1747 1767	7
mmu-miR-3473e	NM_001291068.1	153.00	-22.02	2 16	269 288	14
mmu-miR-3473e	NM_001291068.1	153.00	-25.29	2 20	5813 5836	21
mmu-miR-3473e	NM_001291068.1	150.00	-25.49	2 20	4787 4809	20
mmu-miR-3473e	NM_001291068.1	145.00	-23.91	2 18	6094 6114	16
mmu-miR-3473e	NM_001291068.1	141.00	-27.39	2 20	5064 5086	20
mmu-miR-3473e	NM_148932.2	168.00	-26.81	2 18	2142 2163	17
mmu-miR-3473e	NM_148932.2	153.00	-25.13	2 20	4200 4223	21
mmu-miR-3473e	NM_148932.2	150.00	-24.19	2 11	1774 1794	9
mmu-miR-3473e	NM_148932.2	145.00	-25.15	2 20	615 634	18
mmu-miR-3473e	NM_148932.2	142.00	-23.80	2 16	4812 4833	15
mmu-miR-3473e	NM_011145.3	140.00	-18.84	2 9	872 892	7
mmu-miR-3473e	XM_006525698.2	147.00	-28.29	2 20	5216 5234	18
mmu-miR-3473e	XM_006525698.2	146.00	-24.13	2 18	1963 1981	16
mmu-miR-3473e	NM_026814.3	141.00	-25.01	2 18	680 700	16
mmu-miR-3473e	NM_001167908.1	140.00	-19.26	2 9	1359 1379	7
mmu-miR-3473e	XM_006527008.2	156.00	-26.19	2 17	3413 3433	15
mmu-miR-3473e	XM_006527008.2	149.00	-19.32	2 18	4305 4325	16
mmu-miR-3473e	XM_006527008.2	145.00	-21.56	2 19	3694 3715	18
mmu-miR-3473e	XM_006527008.2	142.00	-16.13	2 11	5028 5048	9
mmu-miR-3473e	XM_006527008.2	141.00	-21.04	2 18	3824 3844	16
mmu-miR-3473e	NM_011169.5	146.00	-21.01	3 19	612 632	16
mmu-miR-3473e	NM_011169.5	146.00	-24.97	2 20	6803 6825	20
mmu-miR-3473e	NM_175022.2	146.00	-21.35	2 16	4399 4420	15
mmu-miR-3473e	NM_175022.2	141.00	-24.84	2 20	1130 1149	18
mmu-miR-3473e	XM_011240476.2	140.00	-22.51	2 17	2268 2288	15
mmu-miR-3473e	NM_008966.3	183.00	-35.09	2 20	3553 3573	18
mmu-miR-3473e	XM_006505015.3	150.00	-26.27	2 20	2144 2166	20
mmu-miR-3473e	XM_006514153.2	155.00	-25.79	3 20	2389 2409	17
mmu-miR-3473e	XM_006514153.2	148.00	-24.73	2 17	1900 1920	15
mmu-miR-3473e	XM_006514153.2	146.00	-21.95	2 20	731 752	19
mmu-miR-3473e	NM_198024.2	165.00	-26.43	2 18	224 244	16
mmu-miR-3473e	NM_198024.2	142.00	-17.04	2 19	1796 1816	17
mmu-miR-3473e	XM_017320735.1	153.00	-24.66	2 19	784 808	21
mmu-miR-3473e	XM_017320735.1	145.00	-22.72	2 18	2507 2527	16
mmu-miR-3473e	XM_017320735.1	141.00	-16.81	2 10	3162 3182	8
mmu-miR-3473e	XM_017320735.1	140.00	-21.37	2 18	2863 2885	18
mmu-miR-3473e	NM_016809.6	159.00	-38.48	2 20	2489 2509	18
mmu-miR-3473e	NM_016809.6	153.00	-19.36	2 20	1358 1377	18
mmu-miR-3473e	XM_006529829.3	154.00	-25.24	2 20	361 383	20
mmu-miR-3473e	XM_011240779.2	140.00	-15.47	2 9	532 552	7
mmu-miR-3473e	NM_019732.2	162.00	-27.08	2 20	1757 1778	19
mmu-miR-3473e	NM_019732.2	159.00	-24.12	2 19	1839 1857	17
mmu-miR-3473e	NM_019732.2	155.00	-22.33	2 16	1942 1962	14
mmu-miR-3473e	NM_019732.2	151.00	-21.02	2 20	2436 2454	18

mmu-miR-3473e	NM_019732.2	143.00	-18.61	2 20	2533 2553	18
mmu-miR-3473e	NM_019732.2	143.00	-23.19	2 20	3151 3171	18
mmu-miR-3473e	XM_006540138.3	150.00	-21.36	2 20	603 625	20
mmu-miR-3473e	NM_009127.4	147.00	-19.84	2 17	4221 4242	16
mmu-miR-3473e	NM_018732.3	143.00	-26.63	2 20	9504 9524	18
mmu-miR-3473e	NM_026535.2	154.00	-22.87	2 20	2576 2599	21
mmu-miR-3473e	NM_026535.2	143.00	-20.69	2 17	2979 3000	16
mmu-miR-3473e	NM_009243.4	159.00	-23.35	2 17	1571 1592	16
mmu-miR-3473e	XM_006521796.1	145.00	-18.44	2 20	1087 1106	18
mmu-miR-3473e	XM_017322211.1	150.00	-26.37	2 20	3069 3090	19
mmu-miR-3473e	XM_017322211.1	150.00	-23.02	2 20	4038 4059	19
mmu-miR-3473e	XM_017322211.1	147.00	-20.54	2 16	5260 5280	14
mmu-miR-3473e	XM_017322211.1	142.00	-18.68	2 20	4845 4866	19
mmu-miR-3473e	XM_017322211.1	141.00	-19.21	3 18	1927 1947	15
mmu-miR-3473e	XM_017322211.1	141.00	-18.95	3 14	1994 2014	11
mmu-miR-3473e	XM_006530238.3	163.00	-27.25	2 19	1522 1540	17
mmu-miR-3473e	XM_006530238.3	158.00	-22.44	2 16	947 968	15
mmu-miR-3473e	XM_006530238.3	155.00	-25.93	2 16	978 998	14
mmu-miR-3473e	XM_006530238.3	148.00	-22.26	2 17	1382 1402	15
mmu-miR-3473e	XM_006530238.3	148.00	-29.25	2 20	5146 5166	19
mmu-miR-3473e	XM_006530238.3	143.00	-26.18	2 20	4753 4773	18
mmu-miR-3473e	XM_006530238.3	140.00	-20.33	3 19	3744 3763	16
mmu-miR-3473e	XM_017316715.1	155.00	-27.15	2 20	309 326	18
mmu-miR-3473e	XM_017316715.1	146.00	-19.55	2 16	2691 2712	15
mmu-miR-3473e	XM_017316897.1	161.00	-28.41	2 20	382 401	18
mmu-miR-3473e	XM_011250732.2	157.00	-20.22	2 20	5764 5783	18
mmu-miR-3473e	XM_011250732.2	154.00	-24.72	2 20	4748 4770	20
mmu-miR-3473e	XM_011250732.2	151.00	-24.83	2 20	4906 4926	18
mmu-miR-3473e	XM_011250732.2	151.00	-24.00	2 12	7667 7687	10
mmu-miR-3473e	XM_011250732.2	149.00	-20.53	2 19	6374 6395	18
mmu-miR-3473e	XM_006518339.3	146.00	-21.74	2 19	1710 1730	17
mmu-miR-3473e	XM_006496884.3	154.00	-25.59	2 19	1636 1656	17
mmu-miR-3473e	XM_006496884.3	147.00	-20.99	2 12	109 129	10
mmu-miR-3473e	XM_017313038.1	140.00	-24.22	2 20	349 372	22
mmu-miR-3473e	NM_009199.2	155.00	-25.08	2 17	2127 2148	16
mmu-miR-3473e	NM_009199.2	153.00	-21.85	2 18	2411 2431	16
mmu-miR-3473e	NM_001033167.3	158.00	-29.80	2 19	272 292	17
mmu-miR-3473e	NM_001033167.3	144.00	-20.17	2 19	4076 4101	22
mmu-miR-3473e	NM_001033167.3	140.00	-20.74	2 9	521 541	7
mmu-miR-3473e	NM_001033167.3	140.00	-25.15	2 20	4008 4026	18
mmu-miR-3473e	NM_001007570.2	140.00	-23.53	2 20	3049 3069	19
mmu-miR-3473e	XM_006503080.3	146.00	-25.10	2 20	4179 4200	19
mmu-miR-3473e	XM_017322365.1	142.00	-18.78	2 20	574 595	19
mmu-miR-3473e	XM_006538687.2	163.00	-33.38	2 20	203 221	18
mmu-miR-3473e	XM_006538687.2	159.00	-24.85	2 16	3736 3756	14
mmu-miR-3473e	XM_006538687.2	155.00	-21.27	2 20	365 385	18
mmu-miR-3473e	XM_006538687.2	144.00	-19.00	2 17	521 541	15
mmu-miR-3473e	NM_001146217.1	156.00	-32.51	2 20	109 132	21
mmu-miR-3473e	XM_006500109.3	145.00	-23.89	2 19	1512 1533	18
mmu-miR-3473e	NM_011430.3	143.00	-25.10	2 20	31 51	18
mmu-miR-3473e	NM_175692.3	191.00	-43.31	2 20	3902 3922	18
mmu-miR-3473e	NM_175692.3	183.00	-38.52	2 20	3390 3410	18
mmu-miR-3473e	NM_175692.3	145.00	-21.51	2 19	525 547	19
mmu-miR-3473e	NM_009223.3	149.00	-22.92	2 18	160 180	16
mmu-miR-3473e	NM_009223.3	142.00	-19.73	2 20	1269 1290	19
mmu-miR-3473e	XM_006539074.3	152.00	-25.48	2 16	7916 7934	14
mmu-miR-3473e	XM_006539074.3	149.00	-23.83	2 18	5862 5882	16
mmu-miR-3473e	XM_006539074.3	146.00	-27.56	2 19	1299 1319	17
mmu-miR-3473e	XM_006539074.3	141.00	-17.25	2 20	8035 8054	18
mmu-miR-3473e	XM_006539074.3	140.00	-15.44	2 9	8741 8761	7
mmu-miR-3473e	NM_001304266.1	159.00	-29.24	2 20	2004 2025	20
mmu-miR-3473e	NM_001304266.1	157.00	-26.70	2 18	5188 5208	16
mmu-miR-3473e	NM_001304266.1	152.00	-24.54	2 18	2440 2462	18
mmu-miR-3473e	NM_001304266.1	150.00	-22.02	2 16	4032 4054	16
mmu-miR-3473e	NM_001304266.1	143.00	-22.16	3 18	3472 3494	17

mmu-miR-3473e	NM_001242411.1	154.00	-20.62	2 16	7275 7296	15
mmu-miR-3473e	NM_001242411.1	140.00	-20.86	3 19	3128 3151	19
mmu-miR-3473e	XM_006506145.1	150.00	-23.61	2 19	168 188	17
mmu-miR-3473e	XM_006506145.1	143.00	-21.38	2 20	1936 1956	18
mmu-miR-3473e	XM_006506145.1	140.00	-29.34	2 18	2945 2966	17
mmu-miR-3473e	XM_011248193.2	144.00	-23.28	2 20	2308 2328	19
mmu-miR-3473e	XM_011245529.2	142.00	-21.37	2 18	4249 4267	16
mmu-miR-3473e	XM_017316056.1	150.00	-31.95	2 20	1122 1144	20
mmu-miR-3473e	NM_009332.3	155.00	-25.22	2 17	1664 1685	16
mmu-miR-3473e	NM_009332.3	145.00	-20.47	2 20	916 935	18
mmu-miR-3473e	NM_001080979.1	158.00	-22.78	2 19	830 850	17
mmu-miR-3473e	NM_001080979.1	150.00	-22.64	2 11	1355 1375	9
mmu-miR-3473e	NM_001080979.1	149.00	-24.27	2 19	2396 2417	18
mmu-miR-3473e	NM_001080979.1	148.00	-19.92	2 18	965 986	17
mmu-miR-3473e	NM_001080979.1	141.00	-29.77	2 20	245 264	18
mmu-miR-3473e	NM_001080979.1	141.00	-25.51	3 20	318 341	20
mmu-miR-3473e	NM_011581.3	143.00	-19.29	2 14	4093 4112	12
mmu-miR-3473e	NM_009379.3	153.00	-23.21	2 19	438 459	18
mmu-miR-3473e	XM_006533452.3	154.00	-27.31	2 20	1936 1959	21
mmu-miR-3473e	XM_006533452.3	149.00	-28.25	2 20	686 708	20
mmu-miR-3473e	XM_006515787.3	141.00	-25.16	2 19	873 894	18
mmu-miR-3473e	NM_144936.1	140.00	-21.84	2 13	1522 1542	11
mmu-miR-3473e	XM_006521428.3	163.00	-26.48	2 20	967 987	18
mmu-miR-3473e	XM_006538830.1	142.00	-20.83	2 16	1432 1453	15
mmu-miR-3473e	XM_006529382.3	163.00	-25.48	2 20	1020 1040	18
mmu-miR-3473e	XM_006529382.3	152.00	-25.58	2 20	959 977	18
mmu-miR-3473e	XM_011248201.2	147.00	-23.11	2 18	2928 2952	20
mmu-miR-3473e	XM_011248201.2	145.00	-26.83	2 18	510 530	16
mmu-miR-3473e	NM_177409.3	143.00	-23.63	2 16	136 156	14
mmu-miR-3473e	NM_001243916.1	147.00	-24.02	2 12	1486 1506	10
mmu-miR-3473e	XM_006508002.3	152.00	-23.10	2 14	2912 2933	13
mmu-miR-3473e	XM_006508002.3	145.00	-25.50	2 14	2426 2446	12
mmu-miR-3473e	XM_006506152.3	152.00	-25.47	2 18	3355 3376	17
mmu-miR-3473e	NM_001033149.3	140.00	-17.69	2 9	468 488	7
mmu-miR-3473e	NM_177709.3	141.00	-19.10	2 20	2800 2819	18
mmu-miR-3473e	NM_011658.2	155.00	-28.87	2 17	326 347	16
mmu-miR-3473e	XM_006538224.1	142.00	-22.41	3 16	1090 1111	14
mmu-miR-3473e	XM_006538224.1	141.00	-24.37	2 20	3592 3615	21
mmu-miR-3473e	XM_006538224.1	140.00	-20.95	3 20	3156 3174	17
mmu-miR-3473e	XM_006501309.3	151.00	-19.66	2 20	721 741	18
mmu-miR-3473e	NM_009504.4	158.00	-22.97	2 20	1327 1349	20
mmu-miR-3473e	XM_017316944.1	142.00	-22.14	3 20	228 250	19
mmu-miR-3473e	NM_009518.2	150.00	-25.40	2 19	506 526	17
mmu-miR-3473e	NM_009518.2	148.00	-20.67	2 14	460 481	13
mmu-miR-3473e	NM_009518.2	145.00	-27.34	2 20	1036 1058	20
mmu-miR-3473e	NM_011724.3	154.00	-26.37	2 16	2902 2923	15
mmu-miR-3473e	NM_011724.3	153.00	-20.89	2 18	4062 4082	16
mmu-miR-3473e	NM_011724.3	147.00	-24.87	2 20	1142 1162	18
mmu-miR-3473e	NM_011724.3	147.00	-21.44	2 18	4519 4538	16
mmu-miR-3473e	XM_006499466.3	157.00	-22.00	2 20	276 295	18
mmu-miR-3473e	XM_006499466.3	154.00	-26.11	2 20	2210 2231	19
mmu-miR-3473e	XM_006499466.3	153.00	-28.57	2 20	1989 2012	21
mmu-miR-3473e	XM_006530585.3	165.00	-30.46	2 20	14457 14476	18
mmu-miR-3473e	XM_006530585.3	156.00	-23.76	2 18	356 377	17
mmu-miR-3473e	XM_006530585.3	149.00	-26.70	2 20	2392 2411	18
mmu-miR-3473e	XM_006530585.3	146.00	-20.63	2 19	5470 5490	17
mmu-miR-3473e	XM_006530585.3	143.00	-18.65	2 18	3092 3111	16
mmu-miR-3473e	XM_017314347.1	141.00	-26.89	2 20	828 847	18
mmu-miR-3473e	NM_199304.1	153.00	-24.64	2 20	119 142	21
mmu-miR-3473e	NM_199304.1	141.00	-20.29	2 18	2056 2076	16
mmu-miR-3473e	NM_199029.2	150.00	-20.45	2 16	1183 1204	15
mmu-miR-3473e	NM_199029.2	149.00	-21.80	2 14	3633 3653	12
mmu-miR-3473e	XM_006511007.3	150.00	-27.07	2 11	7492 7512	9
mmu-miR-3473e	XM_006511007.3	148.00	-24.18	2 17	7144 7164	15
mmu-miR-3473e	XM_006511007.3	145.00	-19.50	2 20	2204 2227	21

mmu-miR-3473e	NM_001110508.1	150.00	-27.73	2 20	1227 1249	20
mmu-miR-3473e	NM_001110508.1	140.00	-22.78	2 9	2027 2047	7
mmu-miR-3473e	XM_006537508.2	141.00	-22.57	2 20	6915 6934	18
mmu-miR-3473e	XM_006519134.3	161.00	-24.49	2 18	1610 1630	16
mmu-miR-3473e	XM_006519134.3	158.00	-27.63	2 17	5062 5081	15
mmu-miR-3473e	XM_006519134.3	145.00	-22.51	2 10	606 626	8
mmu-miR-3473e	XM_006519134.3	141.00	-27.11	2 18	4291 4311	16
mmu-miR-3473e	XM_006519134.3	140.00	-26.87	2 18	4028 4052	20
mmu-miR-7005-5p	NM_172454.2	164.00	-27.69	2 19	2296 2316	17
mmu-miR-7005-5p	NM_172454.2	142.00	-24.05	3 21	131 155	21
mmu-miR-7005-5p	NM_028748.2	155.00	-32.18	2 21	803 825	20
mmu-miR-7005-5p	NM_028748.2	153.00	-25.59	2 14	1993 2014	12
mmu-miR-7005-5p	NM_028748.2	145.00	-22.72	2 21	1683 1702	19
mmu-miR-7005-5p	NM_028748.2	144.00	-16.12	2 13	2355 2376	11
mmu-miR-7005-5p	NM_133167.3	149.00	-22.57	2 18	1186 1207	16
mmu-miR-7005-5p	NM_133167.3	140.00	-22.76	3 13	1671 1692	10
mmu-miR-7005-5p	NM_011044.2	143.00	-24.20	2 21	976 998	20
mmu-miR-7005-5p	NM_011044.2	140.00	-22.04	3 13	440 461	10
mmu-miR-7005-5p	NM_008804.4	156.00	-22.59	2 18	601 623	17
mmu-miR-7005-5p	NM_013743.2	144.00	-25.37	2 13	161 182	11
mmu-miR-7005-5p	NM_001290822.1	149.00	-27.97	2 18	1346 1367	16
mmu-miR-7005-5p	NM_001164593.1	149.00	-22.36	2 20	3084 3104	18
mmu-miR-7005-5p	XM_006538456.3	155.00	-23.03	2 12	968 989	10
mmu-miR-7005-5p	XM_006538456.3	151.00	-36.56	2 21	4211 4235	22
mmu-miR-7005-5p	XM_006538456.3	145.00	-26.25	2 14	799 820	12
mmu-miR-7005-5p	XM_006537815.3	164.00	-27.76	2 21	4186 4207	19
mmu-miR-7005-5p	XM_006537815.3	159.00	-27.31	2 17	3195 3218	17
mmu-miR-7005-5p	XM_006537815.3	151.00	-27.49	2 21	1696 1719	21
mmu-miR-7005-5p	XM_006537815.3	151.00	-31.26	2 21	2495 2518	21
mmu-miR-7005-5p	NM_153104.3	147.00	-22.65	2 17	694 716	16
mmu-miR-7005-5p	NM_011082.3	155.00	-30.09	2 21	2465 2488	21
mmu-miR-7005-5p	NM_011082.3	151.00	-26.63	2 16	2584 2605	14
mmu-miR-7005-5p	NM_011082.3	145.00	-23.11	2 14	238 259	12
mmu-miR-7005-5p	XM_017314870.1	145.00	-17.72	2 21	3698 3717	19
mmu-miR-7005-5p	XM_017314870.1	143.00	-21.94	2 21	1642 1665	21
mmu-miR-7005-5p	XM_017314870.1	140.00	-19.89	2 13	3870 3891	11
mmu-miR-7005-5p	XM_017317946.1	159.00	-24.53	2 17	504 526	16
mmu-miR-7005-5p	NM_018807.5	143.00	-20.53	2 21	1180 1203	21
mmu-miR-7005-5p	XM_017312497.1	150.00	-26.92	2 11	1028 1049	9
mmu-miR-7005-5p	XM_017312497.1	148.00	-25.61	2 19	3983 4006	19
mmu-miR-7005-5p	NM_026385.4	140.00	-34.81	4 21	972 993	17
mmu-miR-7005-5p	XM_006502235.3	143.00	-19.22	2 21	1022 1044	20
mmu-miR-7005-5p	XM_006502235.3	143.00	-26.01	2 21	4195 4218	21
mmu-miR-7005-5p	XM_006502235.3	141.00	-24.07	2 19	2127 2149	18
mmu-miR-7005-5p	NM_001195084.1	145.00	-18.41	2 15	531 555	16
mmu-miR-7005-5p	XM_006512076.3	155.00	-24.33	2 20	2174 2195	18
mmu-miR-7005-5p	XM_006512076.3	150.00	-23.77	2 19	8031 8052	17
mmu-miR-7005-5p	XM_006512076.3	141.00	-17.40	2 11	7613 7635	10
mmu-miR-7005-5p	NM_001030305.2	159.00	-28.93	2 17	1049 1071	16
mmu-miR-7005-5p	NM_001302257.1	140.00	-22.49	2 21	174 195	19
mmu-miR-7005-5p	NM_148932.2	152.00	-23.16	2 17	1259 1280	15
mmu-miR-7005-5p	NM_148932.2	150.00	-20.55	2 11	1163 1184	9
mmu-miR-7005-5p	NM_148932.2	149.00	-22.19	2 19	5055 5077	18
mmu-miR-7005-5p	NM_148932.2	145.00	-20.75	2 18	4200 4221	16
mmu-miR-7005-5p	NM_148932.2	143.00	-22.60	3 21	3168 3190	19
mmu-miR-7005-5p	NM_148932.2	141.00	-20.60	2 10	1375 1396	8
mmu-miR-7005-5p	NM_148932.2	141.00	-20.91	2 18	2493 2514	16
mmu-miR-7005-5p	NM_011145.3	143.00	-19.96	2 21	1829 1852	21
mmu-miR-7005-5p	XM_006525698.2	158.00	-23.29	2 15	1610 1631	13
mmu-miR-7005-5p	XM_006525698.2	156.00	-30.45	2 20	4914 4935	19
mmu-miR-7005-5p	XM_006525698.2	151.00	-25.56	2 17	5719 5741	16
mmu-miR-7005-5p	XM_006525698.2	147.00	-23.71	2 21	668 690	20
mmu-miR-7005-5p	XM_006525698.2	147.00	-23.56	2 16	9071 9092	14
mmu-miR-7005-5p	XM_006525698.2	140.00	-24.96	3 19	7606 7626	16
mmu-miR-7005-5p	XM_006525698.2	140.00	-21.43	2 9	8993 9014	7

mmu-miR-7005-5p	XM_006527008.2	149.00	-22.54	2 18	757 778	16
mmu-miR-7005-5p	XM_006527008.2	146.00	-21.43	2 21	3233 3253	19
mmu-miR-7005-5p	XM_006527008.2	143.00	-20.91	2 21	3192 3214	20
mmu-miR-7005-5p	XM_006527008.2	141.00	-22.38	2 11	3624 3646	10
mmu-miR-7005-5p	NM_011169.5	152.00	-28.81	2 19	8902 8922	17
mmu-miR-7005-5p	NM_011169.5	147.00	-26.17	2 21	3783 3805	20
mmu-miR-7005-5p	NM_011169.5	146.00	-18.85	2 15	434 455	13
mmu-miR-7005-5p	NM_011169.5	141.00	-19.31	2 19	9573 9595	18
mmu-miR-7005-5p	NM_011169.5	140.00	-22.17	2 17	4936 4957	15
mmu-miR-7005-5p	NM_175563.5	160.00	-25.77	2 17	42 63	15
mmu-miR-7005-5p	NM_175563.5	144.00	-28.92	2 17	378 399	15
mmu-miR-7005-5p	NM_175022.2	167.00	-30.14	2 17	4566 4589	17
mmu-miR-7005-5p	NM_175022.2	158.00	-27.73	2 21	2369 2392	21
mmu-miR-7005-5p	NM_175022.2	145.00	-17.01	2 10	2059 2080	8
mmu-miR-7005-5p	NM_175022.2	144.00	-22.16	2 21	316 337	19
mmu-miR-7005-5p	NM_175022.2	143.00	-23.23	2 16	470 491	14
mmu-miR-7005-5p	XM_006524382.3	148.00	-23.61	2 18	42 64	17
mmu-miR-7005-5p	XM_006524382.3	146.00	-20.42	2 19	1229 1250	17
mmu-miR-7005-5p	XM_011240476.2	151.00	-25.47	2 16	2208 2229	14
mmu-miR-7005-5p	XM_011240476.2	143.00	-21.25	2 16	2435 2456	14
mmu-miR-7005-5p	NM_008966.3	140.00	-16.05	2 9	1120 1141	7
mmu-miR-7005-5p	NM_016933.3	146.00	-22.67	2 15	336 357	13
mmu-miR-7005-5p	NM_016933.3	146.00	-19.89	2 16	763 786	16
mmu-miR-7005-5p	XM_006505015.3	146.00	-23.60	2 21	2176 2196	19
mmu-miR-7005-5p	XM_006514153.2	162.00	-30.23	2 15	1768 1789	13
mmu-miR-7005-5p	XM_006514153.2	158.00	-26.18	2 16	3906 3929	16
mmu-miR-7005-5p	XM_006514153.2	146.00	-23.54	2 17	2814 2837	17
mmu-miR-7005-5p	XM_006514153.2	144.00	-18.75	2 20	3320 3339	18
mmu-miR-7005-5p	XM_006514153.2	143.00	-23.43	2 21	1922 1945	21
mmu-miR-7005-5p	NM_009019.2	150.00	-26.46	2 11	2192 2213	9
mmu-miR-7005-5p	NM_009020.3	150.00	-20.10	2 21	2744 2767	21
mmu-miR-7005-5p	XM_006506624.2	141.00	-27.57	2 19	498 520	18
mmu-miR-7005-5p	XM_017320735.1	154.00	-29.28	2 21	1628 1648	19
mmu-miR-7005-5p	NM_011254.5	153.00	-21.79	2 18	1986 2007	16
mmu-miR-7005-5p	NM_011254.5	149.00	-22.50	2 21	1377 1396	19
mmu-miR-7005-5p	XM_006514077.1	158.00	-30.80	2 20	1101 1124	20
mmu-miR-7005-5p	XM_006514077.1	141.00	-20.69	2 21	884 905	20
mmu-miR-7005-5p	XM_006514077.1	141.00	-25.95	2 15	1174 1197	15
mmu-miR-7005-5p	XM_006529829.3	152.00	-22.56	2 15	4482 4502	13
mmu-miR-7005-5p	XM_006529829.3	152.00	-26.53	2 21	5015 5036	19
mmu-miR-7005-5p	XM_006529829.3	140.00	-22.18	2 21	4806 4824	19
mmu-miR-7005-5p	XM_006529829.3	140.00	-17.23	2 9	4996 5017	7
mmu-miR-7005-5p	XM_011240779.2	160.00	-26.98	2 19	62 82	17
mmu-miR-7005-5p	XM_011240779.2	140.00	-23.67	2 19	4388 4411	19
mmu-miR-7005-5p	XM_006507942.2	155.00	-31.17	2 21	156 179	21
mmu-miR-7005-5p	XM_006507942.2	140.00	-19.60	2 17	429 450	15
mmu-miR-7005-5p	NM_001190258.1	144.00	-21.70	2 21	49 70	19
mmu-miR-7005-5p	XM_006525075.3	155.00	-24.56	2 21	741 763	20
mmu-miR-7005-5p	NM_019732.2	147.00	-24.43	2 16	1939 1960	14
mmu-miR-7005-5p	NM_023893.4	151.00	-19.62	2 16	744 765	14
mmu-miR-7005-5p	NM_023893.4	143.00	-21.81	3 17	601 623	15
mmu-miR-7005-5p	XM_006540138.3	140.00	-29.39	4 21	820 841	17
mmu-miR-7005-5p	XM_006540134.3	160.00	-29.22	2 21	1651 1672	19
mmu-miR-7005-5p	XM_006540134.3	159.00	-25.60	2 21	1629 1651	20
mmu-miR-7005-5p	XM_006540134.3	150.00	-22.37	2 21	2595 2618	21
mmu-miR-7005-5p	XM_006540134.3	140.00	-25.68	3 21	1672 1693	18
mmu-miR-7005-5p	NM_009127.4	153.00	-21.32	2 21	3724 3743	19
mmu-miR-7005-5p	NM_018732.3	147.00	-30.68	3 21	5589 5611	19
mmu-miR-7005-5p	NM_009135.2	144.00	-17.75	2 17	211 232	15
mmu-miR-7005-5p	NM_009135.2	140.00	-19.22	2 9	5994 6015	7
mmu-miR-7005-5p	NM_020052.2	164.00	-26.31	2 18	2873 2896	18
mmu-miR-7005-5p	NM_026535.2	152.00	-25.48	3 17	407 428	14
mmu-miR-7005-5p	NM_009243.4	160.00	-21.63	2 17	299 320	15
mmu-miR-7005-5p	NM_009243.4	155.00	-23.76	2 21	143 165	20
mmu-miR-7005-5p	NM_009243.4	149.00	-18.80	2 21	1007 1026	19

mmu-miR-7005-5p	NM_009243.4	144.00	-19.15	2 13	200 221	11
mmu-miR-7005-5p	NM_009244.4	160.00	-21.63	2 17	294 315	15
mmu-miR-7005-5p	NM_009244.4	158.00	-27.82	2 21	996 1021	23
mmu-miR-7005-5p	NM_009244.4	144.00	-19.15	2 13	195 216	11
mmu-miR-7005-5p	NM_009246.3	160.00	-21.63	2 17	304 325	15
mmu-miR-7005-5p	NM_009246.3	155.00	-23.76	2 21	148 170	20
mmu-miR-7005-5p	NM_009246.3	153.00	-24.49	2 20	1007 1031	21
mmu-miR-7005-5p	NM_009246.3	144.00	-19.15	2 13	205 226	11
mmu-miR-7005-5p	NM_008458.2	151.00	-26.53	2 21	156 179	21
mmu-miR-7005-5p	XM_006515637.2	140.00	-30.10	2 21	79 100	19
mmu-miR-7005-5p	XM_011244046.2	151.00	-28.26	2 21	156 179	21
mmu-miR-7005-5p	XM_011244046.2	140.00	-18.54	2 17	1989 2010	15
mmu-miR-7005-5p	XM_006521796.1	142.00	-30.94	2 21	1822 1842	19
mmu-miR-7005-5p	XM_017322211.1	150.00	-21.21	2 21	2690 2713	21
mmu-miR-7005-5p	XM_017322211.1	147.00	-19.44	2 21	2304 2326	20
mmu-miR-7005-5p	XM_017322211.1	146.00	-20.03	2 20	1811 1833	19
mmu-miR-7005-5p	XM_006530238.3	159.00	-29.16	2 20	6151 6172	18
mmu-miR-7005-5p	XM_006530238.3	153.00	-22.66	2 21	4635 4654	19
mmu-miR-7005-5p	XM_006530238.3	153.00	-31.57	2 20	6894 6921	24
mmu-miR-7005-5p	XM_006530238.3	152.00	-26.39	2 18	3193 3215	17
mmu-miR-7005-5p	XM_006530238.3	151.00	-22.68	2 16	4529 4550	14
mmu-miR-7005-5p	XM_006530238.3	151.00	-25.37	2 21	4823 4845	20
mmu-miR-7005-5p	XM_006530238.3	147.00	-25.58	2 21	6686 6713	25
mmu-miR-7005-5p	XM_006530238.3	143.00	-23.27	2 17	3613 3635	16
mmu-miR-7005-5p	XM_017316715.1	146.00	-24.46	2 21	4057 4077	19
mmu-miR-7005-5p	XM_017316715.1	146.00	-23.21	3 21	4188 4208	18
mmu-miR-7005-5p	XM_017316715.1	145.00	-26.38	3 20	1235 1255	17
mmu-miR-7005-5p	XM_017317034.1	147.00	-33.41	3 21	3394 3416	19
mmu-miR-7005-5p	XM_017317034.1	144.00	-20.64	2 18	3966 3989	18
mmu-miR-7005-5p	XM_017317034.1	140.00	-27.13	3 18	521 544	17
mmu-miR-7005-5p	XM_017317034.1	140.00	-24.90	2 19	3807 3830	19
mmu-miR-7005-5p	XM_017316897.1	145.00	-27.81	3 21	528 549	19
mmu-miR-7005-5p	XM_011250732.2	164.00	-28.22	2 19	6620 6640	17
mmu-miR-7005-5p	XM_011250732.2	159.00	-34.13	2 16	109 130	14
mmu-miR-7005-5p	XM_011250732.2	145.00	-29.60	3 18	5454 5475	15
mmu-miR-7005-5p	NM_177578.4	140.00	-14.49	2 9	3225 3246	7
mmu-miR-7005-5p	XM_006518339.3	140.00	-36.14	2 21	563 584	19
mmu-miR-7005-5p	XM_006496884.3	174.00	-32.87	2 20	2368 2390	19
mmu-miR-7005-5p	NM_144539.5	151.00	-23.37	2 16	1060 1081	14
mmu-miR-7005-5p	NM_144539.5	140.00	-15.73	2 17	1852 1873	15
mmu-miR-7005-5p	NM_009199.2	144.00	-32.39	2 21	2740 2761	19
mmu-miR-7005-5p	NM_009199.2	141.00	-19.82	2 21	2127 2146	19
mmu-miR-7005-5p	NM_001033167.3	164.00	-28.68	2 19	3332 3352	17
mmu-miR-7005-5p	NM_001033167.3	151.00	-29.26	2 21	98 120	20
mmu-miR-7005-5p	NM_001033167.3	142.00	-21.47	2 15	3464 3485	13
mmu-miR-7005-5p	NM_001007570.2	150.00	-22.86	2 16	2709 2731	15
mmu-miR-7005-5p	NM_001007570.2	148.00	-19.94	2 15	1527 1547	13
mmu-miR-7005-5p	NM_001007570.2	148.00	-26.47	3 21	2788 2809	18
mmu-miR-7005-5p	NM_001007570.2	143.00	-21.22	3 21	2958 2981	20
mmu-miR-7005-5p	NM_001033286.2	140.00	-20.06	2 18	3361 3383	17
mmu-miR-7005-5p	NM_001033286.2	140.00	-22.32	2 9	3481 3502	7
mmu-miR-7005-5p	NM_001033286.2	140.00	-25.56	2 17	4857 4878	15
mmu-miR-7005-5p	XM_006503080.3	143.00	-22.49	2 18	40 64	19
mmu-miR-7005-5p	XM_017322365.1	151.00	-33.30	3 21	432 454	19
mmu-miR-7005-5p	XM_017322365.1	150.00	-33.00	2 21	193 216	21
mmu-miR-7005-5p	XM_017322365.1	143.00	-23.67	3 20	1540 1561	17
mmu-miR-7005-5p	XM_006538687.2	169.00	-38.29	2 21	4586 4607	20
mmu-miR-7005-5p	XM_006538687.2	162.00	-33.30	2 19	3306 3327	17
mmu-miR-7005-5p	XM_006538687.2	160.00	-26.78	2 21	1265 1286	19
mmu-miR-7005-5p	XM_006538687.2	147.00	-26.61	3 16	3427 3448	13
mmu-miR-7005-5p	NM_001146217.1	148.00	-28.83	2 21	302 323	19
mmu-miR-7005-5p	NM_001146217.1	146.00	-27.25	3 19	167 188	16
mmu-miR-7005-5p	XM_006524777.3	145.00	-19.07	2 20	2639 2659	18
mmu-miR-7005-5p	XM_006524777.3	144.00	-34.74	2 21	1051 1072	19
mmu-miR-7005-5p	XM_006500109.3	141.00	-20.61	2 10	907 928	8

mmu-miR-7005-5p	NM_011430.3	143.00	-20.24	2 18	565 585	16
mmu-miR-7005-5p	NM_175692.3	146.00	-31.82	2 21	5600 5620	19
mmu-miR-7005-5p	NM_009223.3	148.00	-23.83	2 17	1494 1515	15
mmu-miR-7005-5p	XM_006534520.3	147.00	-28.45	3 21	1224 1248	21
mmu-miR-7005-5p	XM_006534520.3	145.00	-26.42	3 20	2864 2884	17
mmu-miR-7005-5p	NM_031183.2	143.00	-19.65	2 12	2238 2259	10
mmu-miR-7005-5p	NM_031183.2	142.00	-30.28	3 21	2866 2891	22
mmu-miR-7005-5p	XM_006539074.3	152.00	-29.74	2 18	7910 7932	17
mmu-miR-7005-5p	XM_006539074.3	150.00	-20.76	2 16	5698 5720	15
mmu-miR-7005-5p	XM_006539074.3	148.00	-31.80	2 21	9788 9809	19
mmu-miR-7005-5p	XM_006539074.3	146.00	-24.08	2 21	7671 7691	19
mmu-miR-7005-5p	XM_006539074.3	140.00	-24.02	2 9	7511 7532	7
mmu-miR-7005-5p	NM_019866.1	145.00	-23.33	2 19	883 906	19
mmu-miR-7005-5p	NM_001304266.1	156.00	-17.32	2 21	10702 10723	19
mmu-miR-7005-5p	NM_001304266.1	152.00	-26.45	2 18	9672 9694	17
mmu-miR-7005-5p	NM_001304266.1	148.00	-24.94	2 21	1644 1665	19
mmu-miR-7005-5p	NM_001304266.1	148.00	-28.22	2 21	6449 6470	19
mmu-miR-7005-5p	NM_001304266.1	148.00	-23.00	2 21	6509 6528	19
mmu-miR-7005-5p	NM_001304266.1	145.00	-28.05	2 19	9442 9464	18
mmu-miR-7005-5p	NM_001304266.1	144.00	-22.10	2 21	651 672	19
mmu-miR-7005-5p	NM_001304266.1	142.00	-25.87	3 21	5024 5048	21
mmu-miR-7005-5p	NM_001304266.1	141.00	-22.87	2 21	3470 3492	21
mmu-miR-7005-5p	NM_001304266.1	141.00	-40.00	2 21	3908 3927	19
mmu-miR-7005-5p	NM_001304266.1	140.00	-25.16	2 21	3922 3943	19
mmu-miR-7005-5p	NM_001304266.1	140.00	-23.66	2 21	5893 5914	21
mmu-miR-7005-5p	NM_001242411.1	144.00	-20.16	2 17	7316 7337	15
mmu-miR-7005-5p	XM_006506145.1	158.00	-29.07	2 19	3450 3471	17
mmu-miR-7005-5p	XM_006506145.1	149.00	-24.76	2 21	6052 6071	19
mmu-miR-7005-5p	XM_006506145.1	147.00	-29.08	3 21	6797 6819	19
mmu-miR-7005-5p	XM_006506145.1	146.00	-32.38	3 21	8808 8828	18
mmu-miR-7005-5p	XM_006506145.1	141.00	-16.93	2 16	2944 2964	14
mmu-miR-7005-5p	XM_006506145.1	141.00	-18.29	2 10	6729 6750	8
mmu-miR-7005-5p	XM_006506145.1	140.00	-19.53	2 17	4842 4863	15
mmu-miR-7005-5p	XM_011248193.2	166.00	-37.47	2 21	1852 1872	19
mmu-miR-7005-5p	XM_011248193.2	151.00	-21.61	2 14	4326 4346	12
mmu-miR-7005-5p	XM_011248193.2	142.00	-22.56	3 17	1741 1761	14
mmu-miR-7005-5p	XM_011245529.2	143.00	-22.24	2 13	3113 3135	12
mmu-miR-7005-5p	XM_011245529.2	140.00	-20.65	3 18	602 624	16
mmu-miR-7005-5p	NM_011374.2	143.00	-25.19	3 16	7392 7413	13
mmu-miR-7005-5p	NM_011374.2	140.00	-18.73	2 9	536 557	7
mmu-miR-7005-5p	NM_032400.2	140.00	-29.27	2 17	1117 1138	15
mmu-miR-7005-5p	XM_017316056.1	144.00	-22.61	2 21	2203 2224	19
mmu-miR-7005-5p	NM_009332.3	150.00	-21.55	2 15	707 728	13
mmu-miR-7005-5p	NM_009332.3	145.00	-19.34	2 10	1064 1085	8
mmu-miR-7005-5p	NM_009332.3	143.00	-24.46	2 19	586 605	17
mmu-miR-7005-5p	NM_009332.3	143.00	-24.18	3 16	2090 2111	13
mmu-miR-7005-5p	NM_001080979.1	167.00	-27.81	2 21	2604 2626	20
mmu-miR-7005-5p	NM_001080979.1	156.00	-25.39	2 15	299 319	13
mmu-miR-7005-5p	NM_009379.3	154.00	-26.46	2 16	1094 1116	15
mmu-miR-7005-5p	NM_009379.3	144.00	-21.70	2 21	1498 1519	19
mmu-miR-7005-5p	NM_009381.3	148.00	-22.54	2 20	133 152	18
mmu-miR-7005-5p	XM_006501237.3	140.00	-24.27	2 19	503 523	17
mmu-miR-7005-5p	XM_006537836.1	150.00	-20.51	2 17	1784 1804	15
mmu-miR-7005-5p	XM_006533452.3	163.00	-32.48	2 21	4100 4123	21
mmu-miR-7005-5p	XM_006533452.3	142.00	-24.27	2 21	1564 1588	22
mmu-miR-7005-5p	XM_006521428.3	145.00	-19.87	2 21	375 394	19
mmu-miR-7005-5p	NM_177371.3	142.00	-30.69	2 21	263 287	22
mmu-miR-7005-5p	XM_006496905.1	160.00	-29.46	2 21	836 857	19
mmu-miR-7005-5p	XM_006496905.1	143.00	-30.93	2 21	1006 1028	20
mmu-miR-7005-5p	NM_009393.2	144.00	-18.77	3 21	646 667	18
mmu-miR-7005-5p	XM_006529382.3	156.00	-27.72	3 21	118 139	18
mmu-miR-7005-5p	XM_006539726.3	140.00	-22.90	2 19	5 28	19
mmu-miR-7005-5p	XM_011248201.2	157.00	-25.11	2 15	472 495	15
mmu-miR-7005-5p	NM_177409.3	160.00	-29.41	2 18	1098 1120	17
mmu-miR-7005-5p	XM_011249157.2	153.00	-21.86	2 18	1829 1850	16

mmu-miR-7005-5p	XM_011249157.2	151.00	-25.13	2 21	2150 2175	23
mmu-miR-7005-5p	NM_001039047.1	156.00	-24.62	2 17	154 175	15
mmu-miR-7005-5p	NM_001039047.1	156.00	-26.82	2 18	1111 1133	17
mmu-miR-7005-5p	XM_006506152.3	142.00	-20.60	2 17	3240 3260	15
mmu-miR-7005-5p	NM_001033149.3	144.00	-27.10	2 17	1393 1414	15
mmu-miR-7005-5p	NM_001033149.3	143.00	-28.66	2 21	2242 2264	20
mmu-miR-7005-5p	NM_001033149.3	140.00	-18.62	2 17	1146 1167	15
mmu-miR-7005-5p	NM_023716.2	142.00	-22.12	2 20	1447 1469	19
mmu-miR-7005-5p	NM_177709.3	146.00	-31.85	2 21	1728 1748	19
mmu-miR-7005-5p	XM_006538224.1	144.00	-21.84	2 17	2327 2348	15
mmu-miR-7005-5p	XM_006538224.1	143.00	-29.72	2 18	2556 2576	16
mmu-miR-7005-5p	XM_006525099.3	149.00	-24.78	2 21	3209 3228	19
mmu-miR-7005-5p	XM_006525099.3	148.00	-28.94	2 21	1650 1671	19
mmu-miR-7005-5p	XM_006525099.3	140.00	-23.03	2 20	529 548	18
mmu-miR-7005-5p	XM_011248394.1	161.00	-29.04	2 16	2672 2692	14
mmu-miR-7005-5p	XM_011248394.1	147.00	-28.66	3 21	724 746	19
mmu-miR-7005-5p	NM_009504.4	156.00	-27.72	2 21	2124 2145	19
mmu-miR-7005-5p	NM_009504.4	148.00	-23.55	2 21	3105 3126	19
mmu-miR-7005-5p	NM_009504.4	145.00	-20.97	2 21	1131 1150	19
mmu-miR-7005-5p	NM_009504.4	141.00	-22.97	2 10	2162 2183	8
mmu-miR-7005-5p	NM_009504.4	141.00	-23.25	2 18	3794 3815	16
mmu-miR-7005-5p	NM_011704.3	141.00	-24.77	2 20	682 702	18
mmu-miR-7005-5p	NM_009514.4	148.00	-22.71	2 21	164 185	19
mmu-miR-7005-5p	XM_006541047.3	150.00	-30.33	2 21	258 278	19
mmu-miR-7005-5p	NM_009518.2	172.00	-33.00	2 21	1711 1732	19
mmu-miR-7005-5p	NM_011724.3	151.00	-33.53	2 21	3325 3347	20
mmu-miR-7005-5p	NM_011724.3	150.00	-23.41	2 21	3533 3556	21
mmu-miR-7005-5p	NM_011724.3	140.00	-24.39	2 21	2906 2927	19
mmu-miR-7005-5p	XM_006499466.3	144.00	-25.47	2 21	2767 2788	19
mmu-miR-7005-5p	XM_006530585.3	161.00	-27.44	2 21	13515 13534	19
mmu-miR-7005-5p	XM_006530585.3	156.00	-24.65	2 19	10383 10403	17
mmu-miR-7005-5p	XM_006530585.3	155.00	-24.88	2 21	1377 1400	21
mmu-miR-7005-5p	XM_006530585.3	155.00	-22.48	2 16	5511 5532	14
mmu-miR-7005-5p	XM_006530585.3	151.00	-21.55	2 16	6277 6298	14
mmu-miR-7005-5p	XM_006530585.3	150.00	-25.92	2 20	12305 12327	19
mmu-miR-7005-5p	XM_006530585.3	146.00	-23.81	3 20	10673 10695	18
mmu-miR-7005-5p	XM_006530585.3	145.00	-21.09	2 18	5689 5710	16
mmu-miR-7005-5p	XM_006530585.3	143.00	-26.36	2 16	6759 6780	14
mmu-miR-7005-5p	XM_006530585.3	142.00	-29.85	3 16	9869 9891	14
mmu-miR-7005-5p	XM_006530585.3	141.00	-28.26	3 19	5441 5466	20
mmu-miR-7005-5p	XM_006530585.3	140.00	-29.85	2 21	1278 1299	19
mmu-miR-7005-5p	XM_006530585.3	140.00	-24.97	2 21	10169 10190	19
mmu-miR-7005-5p	XM_006530585.3	140.00	-21.10	3 17	14198 14219	14
mmu-miR-7005-5p	NM_199304.1	141.00	-20.60	3 18	357 378	15
mmu-miR-7005-5p	NM_199029.2	147.00	-19.36	2 21	4055 4078	21
mmu-miR-7005-5p	NM_199029.2	144.00	-19.65	3 21	1475 1496	18
mmu-miR-7005-5p	NM_199029.2	140.00	-23.21	3 21	3403 3422	18
mmu-miR-7005-5p	XM_006511007.3	144.00	-19.29	2 21	3573 3594	19
mmu-miR-7005-5p	XM_006511007.3	143.00	-17.60	2 17	2763 2786	17
mmu-miR-7005-5p	XM_006511007.3	142.00	-24.15	2 16	6059 6081	15
mmu-miR-7005-5p	XM_006511007.3	141.00	-19.55	2 10	247 268	8
mmu-miR-7005-5p	XM_006511007.3	140.00	-19.86	3 17	6361 6382	14
mmu-miR-7005-5p	NM_001110508.1	149.00	-25.28	2 19	736 758	18
mmu-miR-7005-5p	NM_001110508.1	143.00	-33.57	2 21	708 730	20
mmu-miR-7005-5p	NM_001110508.1	140.00	-17.86	2 17	2873 2894	15
mmu-miR-7005-5p	XM_006537508.2	141.00	-17.03	2 10	484 505	8
mmu-miR-7005-5p	XM_006519134.3	175.00	-32.80	2 21	4924 4946	20
mmu-miR-7005-5p	XM_006519134.3	151.00	-25.17	2 21	4565 4587	20
mmu-miR-7005-5p	XM_006519134.3	147.00	-25.55	3 16	726 747	13
mmu-miR-7005-5p	XM_006519134.3	143.00	-30.38	3 21	3898 3920	19
mmu-miR-7009-5p	NM_133167.3	156.00	-23.78	2 18	2113 2135	17
mmu-miR-7009-5p	NM_133167.3	140.00	-21.07	2 9	265 286	7
mmu-miR-7009-5p	NM_008804.4	147.00	-27.43	2 18	228 251	18
mmu-miR-7009-5p	NM_008804.4	142.00	-24.89	2 15	1351 1372	13
mmu-miR-7009-5p	NM_008804.4	142.00	-25.06	2 15	1420 1441	13

mmu-miR-7009-5p	NM_013743.2	162.00	-25.87	2 15	921 942	13
mmu-miR-7009-5p	NM_001290822.1	153.00	-20.81	2 18	394 415	16
mmu-miR-7009-5p	NM_011082.3	145.00	-33.07	2 19	1758 1780	18
mmu-miR-7009-5p	XM_017314870.1	150.00	-18.45	2 20	2815 2838	20
mmu-miR-7009-5p	NM_018807.5	165.00	-29.54	2 21	2693 2712	19
mmu-miR-7009-5p	NM_018807.5	159.00	-26.80	2 16	543 564	14
mmu-miR-7009-5p	NM_018807.5	156.00	-29.38	2 19	3838 3862	20
mmu-miR-7009-5p	NM_008872.3	170.00	-34.70	2 19	1409 1430	17
mmu-miR-7009-5p	XM_017312497.1	152.00	-29.24	2 21	1919 1940	19
mmu-miR-7009-5p	XM_017312497.1	140.00	-20.37	2 21	3567 3588	19
mmu-miR-7009-5p	XM_011250776.2	152.00	-23.78	2 19	1816 1836	17
mmu-miR-7009-5p	XM_006502235.3	156.00	-30.67	2 17	3602 3623	15
mmu-miR-7009-5p	XM_006502235.3	140.00	-33.50	2 19	3693 3716	19
mmu-miR-7009-5p	NM_001195084.1	146.00	-30.00	2 20	552 575	20
mmu-miR-7009-5p	XM_006512076.3	162.00	-26.23	2 15	2180 2201	13
mmu-miR-7009-5p	XM_006512076.3	152.00	-23.54	2 21	5965 5986	19
mmu-miR-7009-5p	XM_006512076.3	145.00	-16.88	2 14	4151 4172	12
mmu-miR-7009-5p	XM_006512076.3	144.00	-18.47	2 21	527 548	19
mmu-miR-7009-5p	XM_006512076.3	144.00	-33.29	2 17	7540 7562	16
mmu-miR-7009-5p	XM_006512076.3	143.00	-25.22	2 19	2629 2648	17
mmu-miR-7009-5p	XM_006512076.3	141.00	-26.16	3 14	2687 2708	11
mmu-miR-7009-5p	XM_006512076.3	141.00	-21.94	2 14	7203 7224	12
mmu-miR-7009-5p	XM_006512076.3	140.00	-29.19	2 17	2897 2918	15
mmu-miR-7009-5p	NM_001302257.1	144.00	-20.35	2 21	1720 1741	19
mmu-miR-7009-5p	NM_001302257.1	140.00	-28.31	2 21	172 193	19
mmu-miR-7009-5p	NM_001291068.1	159.00	-29.70	2 21	6444 6467	21
mmu-miR-7009-5p	NM_001291068.1	149.00	-28.63	2 14	1504 1525	12
mmu-miR-7009-5p	NM_001291068.1	145.00	-22.32	2 10	5013 5034	8
mmu-miR-7009-5p	NM_148932.2	160.00	-31.81	2 19	2035 2055	17
mmu-miR-7009-5p	NM_148932.2	154.00	-26.03	2 19	3573 3594	17
mmu-miR-7009-5p	NM_148932.2	148.00	-25.63	2 21	4156 4177	19
mmu-miR-7009-5p	NM_148932.2	142.00	-21.41	2 15	1900 1921	13
mmu-miR-7009-5p	NM_011145.3	140.00	-24.07	2 17	2299 2320	15
mmu-miR-7009-5p	XM_006525698.2	160.00	-24.84	2 21	6788 6809	19
mmu-miR-7009-5p	XM_006525698.2	148.00	-26.35	2 21	5231 5252	19
mmu-miR-7009-5p	XM_006525698.2	143.00	-29.01	2 17	5667 5689	16
mmu-miR-7009-5p	XM_006525698.2	142.00	-23.47	2 16	7813 7836	16
mmu-miR-7009-5p	XM_006525698.2	140.00	-23.29	2 9	893 914	7
mmu-miR-7009-5p	XM_006525698.2	140.00	-27.86	2 18	1373 1396	18
mmu-miR-7009-5p	XM_006527008.2	154.00	-29.38	3 19	3043 3064	16
mmu-miR-7009-5p	XM_006527008.2	151.00	-26.50	2 21	1771 1793	20
mmu-miR-7009-5p	XM_006527008.2	149.00	-30.56	2 21	4885 4904	19
mmu-miR-7009-5p	NM_011169.5	146.00	-27.61	2 21	9654 9677	21
mmu-miR-7009-5p	XM_011239558.2	143.00	-25.78	2 21	2225 2242	19
mmu-miR-7009-5p	XM_011239558.2	142.00	-22.17	2 21	4487 4507	19
mmu-miR-7009-5p	NM_175022.2	142.00	-22.82	2 21	2938 2961	21
mmu-miR-7009-5p	NM_175022.2	142.00	-23.21	2 19	2980 3001	17
mmu-miR-7009-5p	NM_175022.2	140.00	-17.63	2 9	2885 2906	7
mmu-miR-7009-5p	XM_011240476.2	150.00	-28.78	3 20	1329 1352	19
mmu-miR-7009-5p	XM_006505015.3	147.00	-28.75	2 20	2346 2367	18
mmu-miR-7009-5p	XM_006514153.2	150.00	-28.51	2 16	2652 2676	17
mmu-miR-7009-5p	XM_006514153.2	142.00	-25.48	2 19	1575 1596	17
mmu-miR-7009-5p	XM_006506624.2	140.00	-16.68	2 9	434 455	7
mmu-miR-7009-5p	XM_017320735.1	156.00	-22.00	2 21	43 64	19
mmu-miR-7009-5p	XM_017320735.1	154.00	-28.96	2 21	1845 1865	19
mmu-miR-7009-5p	XM_017320735.1	150.00	-22.63	2 15	364 385	13
mmu-miR-7009-5p	NM_011254.5	140.00	-15.98	2 9	2586 2607	7
mmu-miR-7009-5p	XM_006514077.1	170.00	-33.29	2 15	1550 1571	13
mmu-miR-7009-5p	XM_006514077.1	143.00	-15.58	2 12	830 851	10
mmu-miR-7009-5p	NM_031192.3	143.00	-29.04	3 21	411 433	19
mmu-miR-7009-5p	XM_006529829.3	142.00	-23.84	2 20	2931 2954	20
mmu-miR-7009-5p	XM_006507942.2	150.00	-23.46	2 21	3675 3699	22
mmu-miR-7009-5p	XM_006507942.2	146.00	-29.30	2 21	692 717	23
mmu-miR-7009-5p	NM_001195662.1	148.00	-30.25	2 21	2347 2368	19
mmu-miR-7009-5p	NM_019732.2	162.00	-27.71	2 19	1276 1297	17

mmu-miR-7009-5p	NM_019732.2	152.00	-26.63	2 13	1173 1194	11
mmu-miR-7009-5p	XM_006540138.3	143.00	-29.84	2 20	149 170	18
mmu-miR-7009-5p	XM_006540138.3	143.00	-32.06	2 21	310 332	20
mmu-miR-7009-5p	NM_009127.4	144.00	-16.20	2 15	441 461	13
mmu-miR-7009-5p	NM_009127.4	141.00	-19.43	2 14	2121 2142	12
mmu-miR-7009-5p	NM_009135.2	150.00	-30.15	2 20	1437 1459	19
mmu-miR-7009-5p	NM_026535.2	160.00	-22.41	2 21	1453 1474	19
mmu-miR-7009-5p	NM_026535.2	147.00	-21.55	2 20	3351 3372	18
mmu-miR-7009-5p	NM_026535.2	140.00	-18.46	2 9	1321 1342	7
mmu-miR-7009-5p	XM_006515637.2	142.00	-22.50	3 15	481 502	12
mmu-miR-7009-5p	XM_006515637.2	141.00	-27.71	2 21	1119 1138	19
mmu-miR-7009-5p	XM_017322211.1	167.00	-33.62	2 19	5054 5073	17
mmu-miR-7009-5p	XM_006530238.3	154.00	-26.00	2 19	4162 4183	17
mmu-miR-7009-5p	XM_006530238.3	152.00	-24.24	2 21	986 1007	19
mmu-miR-7009-5p	XM_006530238.3	150.00	-31.05	2 21	3798 3821	21
mmu-miR-7009-5p	XM_006530238.3	146.00	-21.82	2 18	2043 2062	16
mmu-miR-7009-5p	XM_006530238.3	142.00	-14.95	2 11	6667 6688	9
mmu-miR-7009-5p	XM_006530238.3	142.00	-25.61	3 21	6705 6725	18
mmu-miR-7009-5p	XM_006530238.3	141.00	-25.07	2 14	1859 1880	12
mmu-miR-7009-5p	XM_006530238.3	140.00	-20.09	2 9	3092 3113	7
mmu-miR-7009-5p	XM_017316715.1	148.00	-26.78	2 21	4638 4659	19
mmu-miR-7009-5p	XM_017316715.1	144.00	-34.91	3 17	4364 4385	14
mmu-miR-7009-5p	XM_017316715.1	143.00	-20.04	2 12	556 577	10
mmu-miR-7009-5p	XM_017316715.1	142.00	-28.05	2 21	2806 2826	19
mmu-miR-7009-5p	XM_017316715.1	140.00	-27.40	2 21	4885 4906	19
mmu-miR-7009-5p	XM_017317034.1	146.00	-20.97	2 19	572 593	17
mmu-miR-7009-5p	XM_017316897.1	144.00	-26.24	2 21	1359 1380	19
mmu-miR-7009-5p	XM_017316897.1	142.00	-22.69	3 15	511 532	12
mmu-miR-7009-5p	XM_011250732.2	148.00	-29.31	2 17	6050 6071	15
mmu-miR-7009-5p	XM_011250732.2	146.00	-29.81	2 21	5857 5880	21
mmu-miR-7009-5p	XM_011250732.2	144.00	-20.55	2 21	3713 3731	19
mmu-miR-7009-5p	XM_011250732.2	143.00	-18.31	2 16	913 934	14
mmu-miR-7009-5p	XM_011250732.2	143.00	-21.72	2 20	1054 1075	18
mmu-miR-7009-5p	XM_011250732.2	143.00	-26.50	3 19	7519 7538	16
mmu-miR-7009-5p	XM_011250732.2	141.00	-18.72	2 20	5012 5032	18
mmu-miR-7009-5p	XM_011250732.2	140.00	-19.00	2 17	2578 2599	15
mmu-miR-7009-5p	XM_006496884.3	143.00	-20.84	2 21	2521 2543	20
mmu-miR-7009-5p	XM_006496884.3	142.00	-29.56	2 21	223 246	21
mmu-miR-7009-5p	NM_144539.5	143.00	-32.85	2 21	2617 2639	20
mmu-miR-7009-5p	NM_144539.5	142.00	-23.25	2 21	2103 2123	19
mmu-miR-7009-5p	NM_009199.2	152.00	-28.17	2 21	1641 1662	19
mmu-miR-7009-5p	NM_009199.2	146.00	-18.98	2 20	1617 1640	20
mmu-miR-7009-5p	NM_001033167.3	149.00	-24.76	2 21	2550 2569	19
mmu-miR-7009-5p	NM_001007570.2	144.00	-18.45	2 13	1641 1662	11
mmu-miR-7009-5p	NM_001033286.2	157.00	-22.13	2 18	1016 1037	16
mmu-miR-7009-5p	XM_006503080.3	150.00	-28.60	2 15	79 100	13
mmu-miR-7009-5p	NM_027052.3	147.00	-21.58	2 17	110 132	16
mmu-miR-7009-5p	XM_006538687.2	142.00	-26.06	3 21	2962 2982	18
mmu-miR-7009-5p	NM_001146217.1	148.00	-23.16	2 21	1331 1352	19
mmu-miR-7009-5p	NM_001146217.1	140.00	-17.25	2 9	2414 2435	7
mmu-miR-7009-5p	XM_006524777.3	156.00	-27.91	2 21	2215 2236	19
mmu-miR-7009-5p	XM_006524777.3	147.00	-22.75	2 12	1269 1290	10
mmu-miR-7009-5p	XM_006500109.3	145.00	-28.57	2 14	1687 1708	12
mmu-miR-7009-5p	XM_006500109.3	142.00	-29.50	2 19	109 130	17
mmu-miR-7009-5p	XM_006500109.3	142.00	-29.08	2 19	2242 2263	17
mmu-miR-7009-5p	NM_175692.3	161.00	-26.38	2 19	5179 5201	18
mmu-miR-7009-5p	NM_009223.3	147.00	-34.44	2 21	1306 1330	22
mmu-miR-7009-5p	XM_006534520.3	141.00	-24.93	2 15	3389 3412	15
mmu-miR-7009-5p	NM_031183.2	167.00	-31.82	2 21	2634 2657	21
mmu-miR-7009-5p	NM_031183.2	163.00	-28.34	2 21	2449 2471	20
mmu-miR-7009-5p	NM_031183.2	143.00	-24.32	2 21	501 523	20
mmu-miR-7009-5p	XM_006539074.3	157.00	-22.61	2 18	7745 7766	16
mmu-miR-7009-5p	XM_006539074.3	150.00	-23.45	2 17	7984 8007	17
mmu-miR-7009-5p	XM_006539074.3	148.00	-23.36	2 19	6416 6436	17
mmu-miR-7009-5p	XM_006539074.3	147.00	-25.31	3 21	968 990	19

mmu-miR-7009-5p	XM_006539074.3	145.00	-18.08	2 10	4245 4266	8
mmu-miR-7009-5p	XM_006539074.3	142.00	-24.80	2 19	5279 5300	17
mmu-miR-7009-5p	XM_006539074.3	141.00	-23.61	2 15	8093 8115	14
mmu-miR-7009-5p	XM_006539074.3	140.00	-19.61	2 9	4464 4485	7
mmu-miR-7009-5p	XM_006539074.3	140.00	-17.76	2 9	5682 5703	7
mmu-miR-7009-5p	XM_006539074.3	140.00	-18.06	2 9	6555 6576	7
mmu-miR-7009-5p	XM_006539074.3	140.00	-31.76	3 19	9244 9264	16
mmu-miR-7009-5p	NM_001204201.1	140.00	-25.37	2 19	944 964	17
mmu-miR-7009-5p	NM_001304266.1	158.00	-32.76	2 21	6449 6469	19
mmu-miR-7009-5p	NM_001304266.1	155.00	-30.73	2 21	5081 5106	23
mmu-miR-7009-5p	NM_001304266.1	155.00	-25.42	2 21	9517 9539	20
mmu-miR-7009-5p	NM_001304266.1	151.00	-28.71	2 16	4462 4483	14
mmu-miR-7009-5p	NM_001304266.1	146.00	-25.19	2 19	9503 9524	17
mmu-miR-7009-5p	NM_001304266.1	143.00	-24.71	3 21	4490 4512	19
mmu-miR-7009-5p	NM_001304266.1	141.00	-23.46	2 21	6053 6072	19
mmu-miR-7009-5p	NM_001304266.1	140.00	-24.89	2 19	9486 9506	17
mmu-miR-7009-5p	XM_006506145.1	146.00	-30.19	2 19	8806 8827	17
mmu-miR-7009-5p	XM_006506145.1	142.00	-18.06	2 11	1557 1578	9
mmu-miR-7009-5p	XM_011248193.2	142.00	-23.49	2 15	7149 7170	13
mmu-miR-7009-5p	XM_011248193.2	140.00	-19.14	2 9	2194 2215	7
mmu-miR-7009-5p	NM_011374.2	150.00	-27.05	2 15	326 347	13
mmu-miR-7009-5p	NM_011374.2	143.00	-28.03	2 21	4013 4037	22
mmu-miR-7009-5p	XM_006527452.3	141.00	-21.67	2 14	2031 2052	12
mmu-miR-7009-5p	XM_017316056.1	146.00	-20.51	2 15	407 428	13
mmu-miR-7009-5p	NM_009332.3	146.00	-26.87	2 21	72 92	19
mmu-miR-7009-5p	NM_009332.3	143.00	-24.93	2 21	2452 2474	20
mmu-miR-7009-5p	NM_009332.3	140.00	-23.92	2 19	1870 1890	17
mmu-miR-7009-5p	NM_011581.3	154.00	-24.08	2 21	3669 3689	19
mmu-miR-7009-5p	NM_011581.3	151.00	-31.83	2 18	3276 3299	18
mmu-miR-7009-5p	NM_011581.3	149.00	-25.04	2 19	2388 2414	22
mmu-miR-7009-5p	NM_011581.3	147.00	-21.45	2 20	3866 3887	18
mmu-miR-7009-5p	NM_011581.3	140.00	-29.44	2 21	521 542	19
mmu-miR-7009-5p	NM_009379.3	158.00	-30.55	2 21	463 483	19
mmu-miR-7009-5p	NM_009379.3	143.00	-21.90	2 21	806 828	20
mmu-miR-7009-5p	XM_006533452.3	141.00	-26.26	2 21	608 627	19
mmu-miR-7009-5p	XM_006515787.3	141.00	-24.68	2 20	3028 3048	18
mmu-miR-7009-5p	NM_001161746.1	143.00	-31.35	2 21	737 759	20
mmu-miR-7009-5p	XM_006538830.1	141.00	-22.75	2 14	1406 1427	12
mmu-miR-7009-5p	NM_177371.3	158.00	-23.79	2 15	4905 4926	13
mmu-miR-7009-5p	NM_177371.3	140.00	-15.59	2 9	3336 3357	7
mmu-miR-7009-5p	XM_006496905.1	146.00	-29.90	2 21	297 320	21
mmu-miR-7009-5p	XM_006496905.1	143.00	-23.26	2 17	4154 4176	16
mmu-miR-7009-5p	XM_011248201.2	144.00	-27.72	2 19	4174 4194	17
mmu-miR-7009-5p	XM_011248201.2	141.00	-29.74	2 21	3947 3973	24
mmu-miR-7009-5p	NM_177409.3	144.00	-28.63	2 13	1256 1277	11
mmu-miR-7009-5p	NM_177409.3	143.00	-32.15	3 21	1103 1126	20
mmu-miR-7009-5p	NM_146241.2	145.00	-25.86	2 20	1409 1433	21
mmu-miR-7009-5p	XM_011249157.2	149.00	-20.22	2 20	1581 1601	18
mmu-miR-7009-5p	XM_006508002.3	156.00	-26.88	2 19	1520 1540	17
mmu-miR-7009-5p	NM_023716.2	142.00	-20.54	2 18	200 219	16
mmu-miR-7009-5p	NM_177709.3	147.00	-25.59	2 21	2308 2330	20
mmu-miR-7009-5p	NM_011658.2	142.00	-28.11	2 20	1212 1234	19
mmu-miR-7009-5p	XM_006538224.1	147.00	-30.81	2 21	2361 2383	20
mmu-miR-7009-5p	XM_006538224.1	147.00	-23.75	2 21	2938 2962	22
mmu-miR-7009-5p	XM_006538224.1	143.00	-25.10	3 21	3143 3166	20
mmu-miR-7009-5p	XM_006538224.1	140.00	-30.10	3 21	3448 3469	18
mmu-miR-7009-5p	XM_006501309.3	153.00	-26.93	2 19	421 444	19
mmu-miR-7009-5p	NM_009504.4	154.00	-23.91	2 20	2335 2358	20
mmu-miR-7009-5p	NM_009504.4	148.00	-18.31	2 21	556 577	19
mmu-miR-7009-5p	NM_009504.4	143.00	-28.27	2 21	2844 2866	20
mmu-miR-7009-5p	NM_009504.4	140.00	-17.36	2 9	3214 3235	7
mmu-miR-7009-5p	XM_006525028.2	157.00	-25.53	2 14	917 938	12
mmu-miR-7009-5p	XM_006525028.2	149.00	-28.12	2 19	480 502	18
mmu-miR-7009-5p	XM_006541047.3	147.00	-28.46	2 21	1714 1736	20
mmu-miR-7009-5p	XM_006541047.3	143.00	-23.59	2 21	2226 2248	20

mmu-miR-7009-5p	XM_006533569.3	144.00	-25.80	2 13	1042 1063	11
mmu-miR-7009-5p	NM_011724.3	164.00	-28.20	2 17	5073 5094	15
mmu-miR-7009-5p	NM_011724.3	157.00	-23.63	2 14	5753 5774	12
mmu-miR-7009-5p	NM_011724.3	146.00	-32.87	2 21	997 1026	27
mmu-miR-7009-5p	NM_011724.3	142.00	-17.40	2 11	102 123	9
mmu-miR-7009-5p	NM_011724.3	142.00	-22.89	2 20	2012 2034	19
mmu-miR-7009-5p	XM_006499466.3	143.00	-19.35	2 21	2475 2497	20
mmu-miR-7009-5p	XM_006499466.3	140.00	-23.02	2 21	3060 3081	19
mmu-miR-7009-5p	XM_006530585.3	171.00	-32.62	2 21	9657 9679	20
mmu-miR-7009-5p	XM_006530585.3	162.00	-34.70	3 19	1276 1297	16
mmu-miR-7009-5p	XM_006530585.3	158.00	-26.25	2 15	4169 4190	13
mmu-miR-7009-5p	XM_006530585.3	158.00	-21.53	2 19	9995 10016	17
mmu-miR-7009-5p	XM_006530585.3	155.00	-29.99	2 17	11200 11222	16
mmu-miR-7009-5p	XM_006530585.3	151.00	-28.69	2 21	6216 6238	20
mmu-miR-7009-5p	XM_006530585.3	145.00	-27.59	2 21	5628 5647	19
mmu-miR-7009-5p	XM_006530585.3	140.00	-19.54	2 9	4553 4574	7
mmu-miR-7009-5p	XM_017314347.1	141.00	-19.06	2 19	615 637	18
mmu-miR-7009-5p	NM_199304.1	161.00	-27.96	2 14	84 105	12
mmu-miR-7009-5p	NM_199304.1	140.00	-23.17	2 21	2181 2202	19
mmu-miR-7009-5p	NM_199029.2	150.00	-23.12	2 15	2726 2747	13
mmu-miR-7009-5p	NM_199029.2	140.00	-13.71	2 9	3057 3078	7
mmu-miR-7009-5p	XM_011243586.2	140.00	-17.92	2 9	1440 1461	7
mmu-miR-7009-5p	XM_006511007.3	155.00	-23.68	2 16	3211 3232	14
mmu-miR-7009-5p	XM_006511007.3	151.00	-28.78	3 16	1725 1746	13
mmu-miR-7009-5p	XM_006511007.3	151.00	-22.34	2 20	4921 4942	18
mmu-miR-7009-5p	XM_006511007.3	142.00	-26.06	3 15	1653 1674	12
mmu-miR-7009-5p	XM_006511007.3	140.00	-23.28	2 9	4114 4135	7
mmu-miR-7009-5p	XM_006537508.2	162.00	-33.10	2 21	4634 4660	24
mmu-miR-7009-5p	XM_006537508.2	140.00	-16.85	2 9	1125 1146	7
mmu-miR-7009-5p	XM_006519134.3	159.00	-26.47	2 17	3376 3399	17
mmu-miR-7009-5p	XM_006519134.3	146.00	-28.47	2 21	2108 2128	19
mmu-miR-7009-5p	XM_006519134.3	140.00	-20.32	3 21	3810 3831	18
mmu-miR-7042-5p	NM_172454.2	159.00	-24.73	2 16	303 323	14
mmu-miR-7042-5p	NM_172454.2	145.00	-16.20	2 18	350 370	16
mmu-miR-7042-5p	NM_028748.2	151.00	-18.14	2 20	2409 2429	18
mmu-miR-7042-5p	NM_028748.2	141.00	-19.25	2 14	3390 3410	12
mmu-miR-7042-5p	NM_011044.2	140.00	-13.22	2 9	2337 2357	7
mmu-miR-7042-5p	NM_013743.2	149.00	-16.91	2 18	2957 2977	16
mmu-miR-7042-5p	XM_006538456.3	149.00	-21.17	2 20	528 550	20
mmu-miR-7042-5p	XM_006538456.3	146.00	-14.52	2 19	4372 4392	17
mmu-miR-7042-5p	XM_006538456.3	145.00	-14.12	2 10	3022 3042	8
mmu-miR-7042-5p	XM_006538456.3	143.00	-23.15	3 20	2047 2067	17
mmu-miR-7042-5p	XM_006538456.3	141.00	-23.71	2 18	350 370	16
mmu-miR-7042-5p	XM_006537815.3	152.00	-18.90	2 18	301 322	17
mmu-miR-7042-5p	XM_017314870.1	157.00	-20.63	2 18	4403 4423	16
mmu-miR-7042-5p	XM_017314870.1	145.00	-13.48	2 10	3582 3602	8
mmu-miR-7042-5p	XM_017314870.1	140.00	-11.52	2 9	1978 1998	7
mmu-miR-7042-5p	XM_017314870.1	140.00	-25.38	2 18	5223 5244	17
mmu-miR-7042-5p	NM_018807.5	145.00	-16.83	2 10	1292 1312	8
mmu-miR-7042-5p	NM_008872.3	145.00	-23.30	2 19	681 703	19
mmu-miR-7042-5p	XM_017312497.1	143.00	-20.69	3 17	850 871	15
mmu-miR-7042-5p	XM_017312497.1	141.00	-21.96	2 18	4184 4204	16
mmu-miR-7042-5p	XM_006502235.3	142.00	-17.50	2 18	5795 5815	17
mmu-miR-7042-5p	XM_006512076.3	168.00	-25.86	2 17	6793 6813	15
mmu-miR-7042-5p	XM_006512076.3	162.00	-27.76	2 20	8146 8167	19
mmu-miR-7042-5p	XM_006512076.3	149.00	-16.15	2 14	3764 3784	12
mmu-miR-7042-5p	NM_148932.2	144.00	-22.12	3 20	1863 1881	17
mmu-miR-7042-5p	NM_011145.3	159.00	-24.66	2 20	1799 1819	18
mmu-miR-7042-5p	NM_011145.3	144.00	-21.51	3 18	1827 1848	16
mmu-miR-7042-5p	XM_006525698.2	147.00	-20.52	2 13	7682 7703	12
mmu-miR-7042-5p	XM_006525698.2	143.00	-23.58	2 20	6683 6703	18
mmu-miR-7042-5p	XM_006527008.2	148.00	-26.80	3 18	3736 3757	16
mmu-miR-7042-5p	XM_006527008.2	141.00	-16.48	2 14	2993 3013	12
mmu-miR-7042-5p	NM_011169.5	150.00	-12.25	2 15	125 145	13
mmu-miR-7042-5p	NM_011169.5	145.00	-20.40	3 19	483 505	18

mmu-miR-7042-5p	NM_011169.5	143.00	-22.49	2 20	976 996	18
mmu-miR-7042-5p	NM_011169.5	141.00	-17.54	2 10	5467 5487	8
mmu-miR-7042-5p	XM_011239558.2	140.00	-21.64	3 20	4249 4272	20
mmu-miR-7042-5p	NM_175563.5	148.00	-19.25	2 13	873 893	11
mmu-miR-7042-5p	NM_175563.5	141.00	-23.92	2 18	3490 3510	16
mmu-miR-7042-5p	NM_175022.2	148.00	-17.74	2 17	3119 3139	15
mmu-miR-7042-5p	XM_011240476.2	154.00	-27.33	3 19	2434 2454	16
mmu-miR-7042-5p	XM_006505015.3	147.00	-18.28	2 18	4800 4824	20
mmu-miR-7042-5p	NM_027455.3	151.00	-19.31	2 19	417 435	17
mmu-miR-7042-5p	NM_027455.3	144.00	-17.20	2 18	897 919	18
mmu-miR-7042-5p	XM_006514153.2	157.00	-19.23	2 19	121 143	19
mmu-miR-7042-5p	XM_006514153.2	150.00	-30.52	2 20	1786 1807	19
mmu-miR-7042-5p	XM_006514153.2	142.00	-16.51	2 11	665 685	9
mmu-miR-7042-5p	XM_006514153.2	141.00	-22.44	3 20	3625 3644	17
mmu-miR-7042-5p	NM_009020.3	149.00	-19.00	2 19	1063 1085	19
mmu-miR-7042-5p	NM_009020.3	142.00	-19.08	2 20	817 839	20
mmu-miR-7042-5p	NM_198024.2	148.00	-19.09	2 18	43 64	17
mmu-miR-7042-5p	XM_006529829.3	155.00	-17.82	2 16	1865 1885	14
mmu-miR-7042-5p	XM_011240779.2	164.00	-22.02	2 18	3508 3529	17
mmu-miR-7042-5p	NM_001195662.1	146.00	-16.15	2 12	1296 1317	11
mmu-miR-7042-5p	XM_006525075.3	164.00	-24.70	2 17	104 124	15
mmu-miR-7042-5p	XM_006525075.3	145.00	-19.49	2 14	834 854	12
mmu-miR-7042-5p	NM_019732.2	148.00	-18.01	2 18	3075 3097	18
mmu-miR-7042-5p	XM_006540134.3	140.00	-23.06	2 18	878 899	17
mmu-miR-7042-5p	NM_009135.2	148.00	-16.32	2 15	4479 4498	13
mmu-miR-7042-5p	NM_009135.2	143.00	-20.38	2 16	5457 5477	14
mmu-miR-7042-5p	NM_026535.2	164.00	-23.80	2 19	439 462	20
mmu-miR-7042-5p	XM_006530238.3	161.00	-21.91	2 16	6242 6261	14
mmu-miR-7042-5p	XM_017316897.1	171.00	-27.55	2 20	1649 1669	18
mmu-miR-7042-5p	NM_177578.4	140.00	-12.25	2 9	3153 3173	7
mmu-miR-7042-5p	XM_006496884.3	153.00	-16.27	2 18	2221 2241	16
mmu-miR-7042-5p	XM_006496884.3	149.00	-15.42	2 19	94 116	19
mmu-miR-7042-5p	NM_001310705.1	154.00	-16.15	2 15	146 166	13
mmu-miR-7042-5p	NM_001310705.1	153.00	-19.32	2 20	493 512	18
mmu-miR-7042-5p	NM_009199.2	143.00	-20.02	2 17	3158 3179	16
mmu-miR-7042-5p	NM_001033167.3	143.00	-20.11	2 18	4022 4045	19
mmu-miR-7042-5p	NM_001007570.2	160.00	-22.64	2 20	2243 2263	19
mmu-miR-7042-5p	NM_001007570.2	158.00	-20.20	2 18	2207 2227	17
mmu-miR-7042-5p	NM_001007570.2	158.00	-22.39	2 18	2225 2245	17
mmu-miR-7042-5p	NM_001007570.2	158.00	-20.20	2 18	2263 2283	17
mmu-miR-7042-5p	NM_001033286.2	141.00	-12.37	2 10	3573 3593	8
mmu-miR-7042-5p	NM_001290993.1	144.00	-16.34	2 13	1756 1776	11
mmu-miR-7042-5p	XM_006503080.3	153.00	-23.13	2 18	1850 1870	16
mmu-miR-7042-5p	NM_027052.3	150.00	-22.20	2 20	2484 2505	19
mmu-miR-7042-5p	XM_017322365.1	153.00	-19.82	2 19	727 748	18
mmu-miR-7042-5p	XM_017322365.1	145.00	-15.86	2 19	581 602	18
mmu-miR-7042-5p	XM_006538687.2	157.00	-27.15	3 18	2793 2813	15
mmu-miR-7042-5p	XM_006538687.2	147.00	-20.84	3 20	3505 3525	17
mmu-miR-7042-5p	NM_175692.3	146.00	-14.63	2 11	912 932	9
mmu-miR-7042-5p	NM_175692.3	142.00	-15.26	2 11	519 539	9
mmu-miR-7042-5p	NM_175692.3	142.00	-24.91	2 20	4224 4245	19
mmu-miR-7042-5p	NM_031183.2	150.00	-17.54	2 11	2307 2327	9
mmu-miR-7042-5p	XM_006539074.3	175.00	-31.47	2 20	3639 3659	18
mmu-miR-7042-5p	XM_006539074.3	152.00	-24.02	2 19	4638 4657	17
mmu-miR-7042-5p	XM_006539074.3	140.00	-20.86	3 19	5461 5480	16
mmu-miR-7042-5p	XM_006539074.3	140.00	-23.07	2 17	9041 9061	15
mmu-miR-7042-5p	NM_019866.1	141.00	-12.67	2 18	1858 1878	16
mmu-miR-7042-5p	NM_178753.4	153.00	-19.49	2 18	3497 3517	16
mmu-miR-7042-5p	NM_001304266.1	163.00	-26.64	2 20	4474 4494	18
mmu-miR-7042-5p	NM_001304266.1	150.00	-22.30	2 11	3260 3280	9
mmu-miR-7042-5p	NM_001304266.1	142.00	-20.43	3 20	3875 3896	18
mmu-miR-7042-5p	NM_001304266.1	141.00	-19.49	3 19	5103 5124	17
mmu-miR-7042-5p	NM_001304266.1	141.00	-15.60	2 10	5859 5879	8
mmu-miR-7042-5p	NM_001242411.1	153.00	-17.26	2 14	6334 6354	12
mmu-miR-7042-5p	XM_006506145.1	153.00	-20.00	2 16	2127 2146	14

mmu-miR-7042-5p	XM_006506145.1	147.00	-20.17	3 20	5600 5620	17
mmu-miR-7042-5p	XM_006506145.1	145.00	-15.86	2 19	6569 6591	19
mmu-miR-7042-5p	XM_006506145.1	142.00	-14.88	2 11	1718 1738	9
mmu-miR-7042-5p	XM_006506145.1	142.00	-24.08	3 20	6832 6853	18
mmu-miR-7042-5p	XM_006506145.1	140.00	-16.63	3 17	4841 4861	14
mmu-miR-7042-5p	XM_011248193.2	153.00	-16.86	2 19	4831 4852	18
mmu-miR-7042-5p	XM_011245529.2	155.00	-20.71	2 19	1018 1039	19
mmu-miR-7042-5p	NM_011374.2	141.00	-30.71	2 20	793 812	18
mmu-miR-7042-5p	XM_017316056.1	145.00	-15.53	2 19	1168 1189	18
mmu-miR-7042-5p	NM_001080979.1	155.00	-22.45	2 20	2565 2585	18
mmu-miR-7042-5p	NM_153801.3	155.00	-15.89	2 20	1174 1194	18
mmu-miR-7042-5p	NM_153801.3	155.00	-19.16	2 12	1646 1666	10
mmu-miR-7042-5p	NM_011581.3	144.00	-19.35	2 20	3998 4016	18
mmu-miR-7042-5p	XM_006533452.3	143.00	-15.87	2 18	4853 4877	20
mmu-miR-7042-5p	XM_006533452.3	140.00	-25.03	2 20	2041 2064	21
mmu-miR-7042-5p	XM_006515787.3	140.00	-14.46	2 17	1770 1790	15
mmu-miR-7042-5p	NM_177371.3	145.00	-14.54	2 18	5229 5249	16
mmu-miR-7042-5p	XM_006496905.1	154.00	-17.12	2 19	5136 5155	18
mmu-miR-7042-5p	XM_006496905.1	153.00	-20.17	2 19	4840 4861	18
mmu-miR-7042-5p	NM_009393.2	142.00	-24.87	2 20	639 661	20
mmu-miR-7042-5p	XM_006529382.3	149.00	-19.30	2 19	516 537	18
mmu-miR-7042-5p	XM_006529382.3	145.00	-20.38	3 18	302 322	15
mmu-miR-7042-5p	NM_146241.2	150.00	-21.50	2 20	2779 2800	19
mmu-miR-7042-5p	NM_146241.2	145.00	-16.46	2 17	958 976	15
mmu-miR-7042-5p	XM_011249157.2	153.00	-20.53	2 18	2924 2944	16
mmu-miR-7042-5p	NM_001039047.1	164.00	-21.38	2 17	2084 2104	15
mmu-miR-7042-5p	XM_017321602.1	156.00	-16.36	2 18	1833 1854	17
mmu-miR-7042-5p	XM_006506152.3	144.00	-15.95	2 13	2990 3010	11
mmu-miR-7042-5p	NM_001033149.3	166.00	-22.93	2 20	4118 4139	19
mmu-miR-7042-5p	XM_006501309.3	145.00	-23.71	2 20	882 904	20
mmu-miR-7042-5p	XM_006525099.3	152.00	-20.37	2 13	447 467	11
mmu-miR-7042-5p	XM_006525099.3	142.00	-17.83	2 19	3268 3288	17
mmu-miR-7042-5p	XM_006525099.3	140.00	-21.14	3 19	2199 2218	16
mmu-miR-7042-5p	XM_006525099.3	140.00	-15.81	2 18	3406 3427	17
mmu-miR-7042-5p	XM_011248394.1	156.00	-20.86	2 20	2037 2057	19
mmu-miR-7042-5p	NM_009504.4	141.00	-24.79	3 19	1124 1146	18
mmu-miR-7042-5p	NM_009518.2	149.00	-17.08	2 18	1828 1848	16
mmu-miR-7042-5p	XM_006499466.3	159.00	-22.62	2 16	1834 1854	14
mmu-miR-7042-5p	XM_006499466.3	144.00	-23.34	3 18	1196 1219	18
mmu-miR-7042-5p	XM_006530585.3	149.00	-12.11	2 17	11486 11504	15
mmu-miR-7042-5p	XM_006530585.3	141.00	-17.22	2 14	1775 1795	12
mmu-miR-7042-5p	XM_011243586.2	145.00	-23.25	2 20	602 621	18
mmu-miR-7042-5p	XM_006511007.3	164.00	-24.94	2 18	5920 5941	17
mmu-miR-7042-5p	XM_006519134.3	152.00	-15.68	2 13	3851 3871	11
mmu-miR-7042-5p	XM_006519134.3	150.00	-17.41	2 19	3143 3163	17
mmu-miR-7042-5p	XM_006519134.3	144.00	-14.15	2 13	842 862	11
mmu-miR-7219-5p	NM_172454.2	144.00	-19.90	2 21	13 35	19
mmu-miR-7219-5p	NM_013743.2	141.00	-17.53	2 18	2225 2247	16
mmu-miR-7219-5p	XM_006509465.2	144.00	-15.80	2 19	683 704	17
mmu-miR-7219-5p	NM_001290822.1	151.00	-19.08	2 22	1525 1550	23
mmu-miR-7219-5p	NM_001164593.1	144.00	-24.03	2 22	1101 1125	22
mmu-miR-7219-5p	NM_001164593.1	140.00	-20.66	2 18	3646 3669	17
mmu-miR-7219-5p	XM_006538456.3	156.00	-19.28	2 18	2774 2798	18
mmu-miR-7219-5p	XM_006538456.3	142.00	-24.33	2 21	2651 2672	19
mmu-miR-7219-5p	XM_006537815.3	155.00	-24.50	2 22	1502 1528	24
mmu-miR-7219-5p	XM_006537815.3	152.00	-19.77	2 21	287 306	19
mmu-miR-7219-5p	NM_011082.3	140.00	-17.34	2 22	792 815	21
mmu-miR-7219-5p	XM_017314870.1	153.00	-20.59	2 20	797 822	21
mmu-miR-7219-5p	XM_017314870.1	144.00	-20.37	2 22	2171 2198	25
mmu-miR-7219-5p	NM_018807.5	144.00	-19.93	2 22	4507 4531	22
mmu-miR-7219-5p	NM_026385.4	153.00	-23.01	2 22	224 246	20
mmu-miR-7219-5p	NM_001195084.1	140.00	-24.05	3 22	142 165	20
mmu-miR-7219-5p	XM_006512076.3	146.00	-27.24	2 21	6108 6134	23
mmu-miR-7219-5p	NM_001302257.1	156.00	-16.07	2 21	976 998	19
mmu-miR-7219-5p	NM_148932.2	144.00	-14.71	2 21	3963 3985	19

mmu-miR-7219-5p	NM_148932.2	140.00	-23.60	2 21	1732 1754	19
mmu-miR-7219-5p	XM_006525698.2	147.00	-25.31	2 22	1149 1170	20
mmu-miR-7219-5p	XM_006525698.2	145.00	-23.00	2 19	8479 8503	19
mmu-miR-7219-5p	XM_006525698.2	141.00	-20.99	2 21	717 737	19
mmu-miR-7219-5p	XM_006525698.2	141.00	-18.05	3 21	733 757	21
mmu-miR-7219-5p	XM_006525698.2	141.00	-25.30	2 22	6703 6726	21
mmu-miR-7219-5p	NM_001167908.1	142.00	-20.35	2 19	465 487	17
mmu-miR-7219-5p	NM_175563.5	169.00	-23.24	2 22	2280 2302	20
mmu-miR-7219-5p	NM_175563.5	144.00	-18.17	2 22	261 286	23
mmu-miR-7219-5p	NM_016933.3	142.00	-18.34	2 22	747 769	21
mmu-miR-7219-5p	NM_009020.3	153.00	-24.20	2 20	2967 2988	18
mmu-miR-7219-5p	NM_198024.2	143.00	-20.02	2 22	2440 2465	23
mmu-miR-7219-5p	XM_006507942.2	158.00	-26.07	3 20	1824 1848	19
mmu-miR-7219-5p	NM_019732.2	144.00	-18.89	2 21	1469 1491	19
mmu-miR-7219-5p	NM_019732.2	141.00	-17.24	2 20	3294 3315	18
mmu-miR-7219-5p	NM_009127.4	161.00	-17.53	2 22	3337 3359	20
mmu-miR-7219-5p	NM_018732.3	156.00	-16.40	2 19	7989 8010	17
mmu-miR-7219-5p	XM_017322211.1	147.00	-18.53	2 16	164 184	14
mmu-miR-7219-5p	XM_017322211.1	141.00	-18.56	2 20	3349 3370	18
mmu-miR-7219-5p	XM_017316715.1	148.00	-24.23	2 21	127 149	19
mmu-miR-7219-5p	XM_017317034.1	145.00	-20.90	3 22	3819 3841	19
mmu-miR-7219-5p	XM_006518339.3	147.00	-14.27	2 21	1089 1112	20
mmu-miR-7219-5p	NM_144539.5	141.00	-25.19	2 18	305 327	16
mmu-miR-7219-5p	XM_017313038.1	156.00	-19.33	2 22	603 628	23
mmu-miR-7219-5p	XM_017313038.1	155.00	-19.45	2 22	505 526	20
mmu-miR-7219-5p	NM_001310705.1	142.00	-19.05	2 17	1443 1464	15
mmu-miR-7219-5p	NM_001007570.2	154.00	-22.26	2 21	1026 1052	23
mmu-miR-7219-5p	NM_027052.3	144.00	-13.44	3 21	2959 2981	18
mmu-miR-7219-5p	XM_006538687.2	157.00	-18.17	2 22	4269 4291	20
mmu-miR-7219-5p	NM_009223.3	141.00	-21.42	2 19	1547 1571	19
mmu-miR-7219-5p	XM_006539074.3	164.00	-25.18	2 18	10227 10250	17
mmu-miR-7219-5p	XM_006539074.3	155.00	-23.07	2 20	3695 3717	18
mmu-miR-7219-5p	XM_006539074.3	141.00	-24.03	2 22	5014 5036	20
mmu-miR-7219-5p	NM_001304266.1	142.00	-22.43	2 21	6061 6082	19
mmu-miR-7219-5p	NM_001242411.1	151.00	-14.87	2 21	839 862	20
mmu-miR-7219-5p	NM_001242411.1	144.00	-14.25	2 20	6458 6478	18
mmu-miR-7219-5p	XM_006506145.1	154.00	-19.23	2 20	8056 8080	20
mmu-miR-7219-5p	XM_011248193.2	142.00	-14.28	2 11	8089 8111	9
mmu-miR-7219-5p	XM_006527452.3	161.00	-22.13	2 16	1648 1669	14
mmu-miR-7219-5p	XM_017316056.1	142.00	-20.04	2 21	1518 1544	23
mmu-miR-7219-5p	NM_178715.3	152.00	-18.52	2 21	1168 1190	19
mmu-miR-7219-5p	NM_178715.3	148.00	-24.34	2 18	1567 1590	17
mmu-miR-7219-5p	NM_001161746.1	142.00	-17.94	2 21	704 725	19
mmu-miR-7219-5p	XM_006521428.3	152.00	-25.53	2 22	1611 1634	21
mmu-miR-7219-5p	XM_006521428.3	140.00	-15.96	2 9	1802 1824	7
mmu-miR-7219-5p	XM_011248201.2	153.00	-26.89	2 20	4205 4226	18
mmu-miR-7219-5p	NM_177409.3	141.00	-13.40	2 14	954 976	12
mmu-miR-7219-5p	NM_146241.2	150.00	-17.40	2 20	3129 3152	19
mmu-miR-7219-5p	NM_146241.2	143.00	-18.79	3 16	4056 4078	13
mmu-miR-7219-5p	XM_011249157.2	162.00	-24.01	2 22	2356 2376	20
mmu-miR-7219-5p	XM_011249157.2	140.00	-22.23	2 22	3222 3246	22
mmu-miR-7219-5p	NM_001039047.1	155.00	-30.91	2 21	969 993	21
mmu-miR-7219-5p	XM_017321602.1	143.00	-21.62	2 21	1527 1550	20
mmu-miR-7219-5p	XM_006506152.3	148.00	-23.24	2 18	1995 2018	17
mmu-miR-7219-5p	NM_001033149.3	143.00	-22.24	2 17	1899 1922	16
mmu-miR-7219-5p	NM_001033149.3	141.00	-20.82	2 22	4503 4525	20
mmu-miR-7219-5p	NM_023716.2	153.00	-25.18	2 22	633 655	20
mmu-miR-7219-5p	XM_006501309.3	141.00	-14.75	2 22	1721 1743	20
mmu-miR-7219-5p	XM_011248394.1	140.00	-17.71	2 21	1768 1790	19
mmu-miR-7219-5p	NM_009504.4	164.00	-20.35	2 21	3233 3255	19
mmu-miR-7219-5p	XM_006541047.3	140.00	-19.57	2 21	915 937	19
mmu-miR-7219-5p	XM_006530585.3	143.00	-19.61	2 21	5567 5591	21
mmu-miR-7219-5p	XM_017314347.1	140.00	-21.00	2 22	1446 1469	21
mmu-miR-7219-5p	XM_011243586.2	150.00	-19.55	2 19	3409 3431	17
mmu-miR-7219-5p	XM_006511007.3	143.00	-20.57	2 20	4428 4450	18

mmu-miR-7219-5p	XM_006537508.2	148.00	-30.26	2 22	2926 2949	21
mmu-miR-7219-5p	XM_006537508.2	147.00	-20.42	2 22	1369 1390	20
mmu-miR-7219-5p	XM_006519134.3	145.00	-12.79	2 14	6698 6720	12
mmu-miR-7221-3p	NM_028748.2	151.00	-18.92	2 20	940 960	18
mmu-miR-7221-3p	NM_133167.3	161.00	-21.00	2 19	1173 1194	18
mmu-miR-7221-3p	NM_133167.3	145.00	-22.36	3 18	2083 2103	15
mmu-miR-7221-3p	NM_133167.3	141.00	-27.29	2 19	29 53	21
mmu-miR-7221-3p	NM_011044.2	145.00	-27.92	3 19	2418 2439	17
mmu-miR-7221-3p	NM_001164593.1	152.00	-23.90	2 13	2489 2509	11
mmu-miR-7221-3p	XM_006538456.3	148.00	-26.88	2 18	3106 3128	18
mmu-miR-7221-3p	XM_006538456.3	140.00	-18.76	2 13	6345 6365	11
mmu-miR-7221-3p	NM_153104.3	150.00	-18.75	2 17	116 135	15
mmu-miR-7221-3p	NM_011082.3	151.00	-29.74	2 20	1146 1166	18
mmu-miR-7221-3p	XM_017314870.1	140.00	-18.75	2 9	647 667	7
mmu-miR-7221-3p	NM_018807.5	140.00	-26.91	2 20	975 995	19
mmu-miR-7221-3p	NM_008872.3	151.00	-21.29	2 16	1354 1374	14
mmu-miR-7221-3p	XM_017312497.1	147.00	-19.67	2 17	1444 1466	17
mmu-miR-7221-3p	XM_006502235.3	146.00	-18.61	2 11	1705 1725	9
mmu-miR-7221-3p	NM_001195084.1	153.00	-24.15	2 15	570 591	14
mmu-miR-7221-3p	XM_006512076.3	167.00	-29.91	2 18	2493 2512	16
mmu-miR-7221-3p	XM_006512076.3	154.00	-35.53	2 20	2506 2527	19
mmu-miR-7221-3p	XM_006512076.3	145.00	-18.14	2 10	8268 8288	8
mmu-miR-7221-3p	NM_001302257.1	148.00	-27.11	2 18	1552 1573	17
mmu-miR-7221-3p	NM_001291068.1	150.00	-16.65	2 20	1912 1934	20
mmu-miR-7221-3p	NM_001291068.1	148.00	-26.55	2 20	5935 5953	18
mmu-miR-7221-3p	NM_001291068.1	146.00	-19.36	2 20	5869 5890	19
mmu-miR-7221-3p	NM_001291068.1	145.00	-16.73	2 10	5835 5855	8
mmu-miR-7221-3p	NM_001291068.1	144.00	-25.07	3 17	3946 3966	14
mmu-miR-7221-3p	XM_006525698.2	145.00	-23.50	2 20	9015 9034	18
mmu-miR-7221-3p	XM_006525698.2	143.00	-16.43	2 13	8954 8975	12
mmu-miR-7221-3p	XM_006527008.2	146.00	-24.09	2 20	2114 2136	20
mmu-miR-7221-3p	NM_011169.5	148.00	-25.90	2 20	9667 9685	18
mmu-miR-7221-3p	XM_011239558.2	140.00	-12.53	2 9	3924 3944	7
mmu-miR-7221-3p	NM_175563.5	144.00	-16.76	2 13	403 423	11
mmu-miR-7221-3p	NM_175022.2	155.00	-26.19	2 20	3030 3049	19
mmu-miR-7221-3p	XM_011240476.2	164.00	-28.13	2 18	269 290	17
mmu-miR-7221-3p	XM_011240476.2	142.00	-32.07	2 20	3212 3234	20
mmu-miR-7221-3p	NM_009019.2	161.00	-29.17	2 20	677 700	21
mmu-miR-7221-3p	NM_009019.2	154.00	-22.70	2 19	1984 2004	17
mmu-miR-7221-3p	NM_009019.2	141.00	-23.67	3 20	4882 4901	17
mmu-miR-7221-3p	NM_009020.3	141.00	-18.07	2 20	3124 3143	18
mmu-miR-7221-3p	NM_009020.3	140.00	-15.32	2 9	945 965	7
mmu-miR-7221-3p	NM_016809.6	147.00	-23.10	2 18	1644 1663	16
mmu-miR-7221-3p	NM_016809.6	145.00	-24.84	2 18	161 181	16
mmu-miR-7221-3p	XM_006514077.1	145.00	-37.69	2 20	1107 1126	18
mmu-miR-7221-3p	XM_006529829.3	145.00	-25.66	3 20	5473 5492	17
mmu-miR-7221-3p	XM_011240779.2	148.00	-22.55	2 20	4684 4702	18
mmu-miR-7221-3p	NM_001195662.1	157.00	-17.87	2 18	3028 3047	16
mmu-miR-7221-3p	NM_019732.2	168.00	-30.11	2 17	2570 2590	15
mmu-miR-7221-3p	NM_023893.4	146.00	-14.08	2 20	423 444	19
mmu-miR-7221-3p	NM_009127.4	152.00	-19.44	2 17	3353 3373	15
mmu-miR-7221-3p	NM_018732.3	159.00	-31.42	2 20	6525 6545	18
mmu-miR-7221-3p	NM_026535.2	165.00	-28.22	2 18	3049 3069	16
mmu-miR-7221-3p	NM_026535.2	142.00	-21.45	2 16	3273 3294	15
mmu-miR-7221-3p	NM_009244.4	140.00	-19.98	2 20	1190 1211	20
mmu-miR-7221-3p	NM_009246.3	140.00	-19.98	2 20	1200 1221	20
mmu-miR-7221-3p	XM_006521796.1	140.00	-19.08	2 9	247 267	7
mmu-miR-7221-3p	XM_006530238.3	152.00	-23.42	2 20	538 556	18
mmu-miR-7221-3p	XM_017316715.1	147.00	-25.09	3 16	3249 3269	13
mmu-miR-7221-3p	XM_017316715.1	146.00	-16.35	2 17	4738 4757	15
mmu-miR-7221-3p	XM_011250732.2	149.00	-18.52	2 19	7258 7279	18
mmu-miR-7221-3p	XM_006518339.3	147.00	-28.90	2 16	1141 1161	14
mmu-miR-7221-3p	NM_001033167.3	147.00	-18.95	2 20	5314 5332	18
mmu-miR-7221-3p	NM_001033286.2	146.00	-23.31	2 11	2513 2533	9
mmu-miR-7221-3p	NM_001290993.1	151.00	-28.67	2 18	4147 4170	19

mmu-miR-7221-3p	XM_006503080.3	145.00	-31.78	2 20	3476 3495	18
mmu-miR-7221-3p	NM_027052.3	153.00	-22.13	2 18	2572 2592	16
mmu-miR-7221-3p	XM_006538687.2	155.00	-28.53	2 19	3166 3184	17
mmu-miR-7221-3p	NM_001146217.1	161.00	-27.98	2 16	1726 1745	14
mmu-miR-7221-3p	XM_006524777.3	141.00	-27.12	2 18	1909 1929	16
mmu-miR-7221-3p	XM_006534520.3	140.00	-27.16	2 17	1414 1434	15
mmu-miR-7221-3p	XM_006539074.3	143.00	-23.46	2 20	5655 5675	18
mmu-miR-7221-3p	NM_178753.4	156.00	-22.30	2 18	1710 1732	18
mmu-miR-7221-3p	NM_178753.4	140.00	-14.71	2 9	1664 1684	7
mmu-miR-7221-3p	NM_001304266.1	158.00	-23.23	2 15	4793 4813	13
mmu-miR-7221-3p	NM_001304266.1	152.00	-20.91	2 13	2184 2204	11
mmu-miR-7221-3p	NM_001304266.1	143.00	-18.94	2 13	9657 9680	14
mmu-miR-7221-3p	NM_001242411.1	147.00	-27.72	3 18	7475 7494	15
mmu-miR-7221-3p	NM_001242411.1	144.00	-24.09	2 18	5902 5924	18
mmu-miR-7221-3p	NM_001242411.1	141.00	-20.14	3 18	6535 6555	15
mmu-miR-7221-3p	NM_001242411.1	141.00	-25.11	3 20	7290 7309	17
mmu-miR-7221-3p	XM_006506145.1	163.00	-21.11	2 20	1883 1903	18
mmu-miR-7221-3p	XM_006506145.1	141.00	-21.53	2 15	1415 1438	16
mmu-miR-7221-3p	XM_011248193.2	163.00	-23.02	2 20	8026 8046	18
mmu-miR-7221-3p	XM_011248193.2	155.00	-28.70	2 18	1385 1404	16
mmu-miR-7221-3p	NM_011374.2	144.00	-22.91	2 18	2895 2917	18
mmu-miR-7221-3p	XM_017316056.1	143.00	-17.43	2 20	833 853	18
mmu-miR-7221-3p	NM_009332.3	147.00	-27.66	2 17	559 580	16
mmu-miR-7221-3p	NM_009332.3	146.00	-21.86	2 17	1373 1392	15
mmu-miR-7221-3p	NM_153801.3	158.00	-21.08	2 20	805 827	20
mmu-miR-7221-3p	NM_153801.3	145.00	-22.68	2 10	241 261	8
mmu-miR-7221-3p	NM_011581.3	153.00	-18.59	2 14	5391 5411	12
mmu-miR-7221-3p	NM_011581.3	142.00	-26.79	2 20	4537 4560	21
mmu-miR-7221-3p	XM_006533452.3	143.00	-21.29	3 20	1862 1882	17
mmu-miR-7221-3p	XM_006533452.3	143.00	-22.59	3 20	2072 2092	17
mmu-miR-7221-3p	XM_006496905.1	159.00	-28.46	2 20	416 436	18
mmu-miR-7221-3p	XM_006496905.1	143.00	-15.26	2 13	2243 2264	12
mmu-miR-7221-3p	XM_006496905.1	143.00	-15.26	2 13	2771 2792	12
mmu-miR-7221-3p	XM_006496905.1	140.00	-15.03	2 9	3036 3056	7
mmu-miR-7221-3p	XM_011248201.2	143.00	-27.74	3 16	155 175	13
mmu-miR-7221-3p	NM_001039047.1	140.00	-21.41	2 18	2103 2124	17
mmu-miR-7221-3p	XM_006506152.3	152.00	-21.19	2 20	3777 3797	19
mmu-miR-7221-3p	NM_011658.2	144.00	-26.72	2 13	605 625	11
mmu-miR-7221-3p	XM_006538224.1	140.00	-27.95	2 17	1511 1531	15
mmu-miR-7221-3p	XM_006525099.3	151.00	-21.92	2 20	2639 2659	18
mmu-miR-7221-3p	XM_006525028.2	167.00	-24.16	2 20	1023 1043	18
mmu-miR-7221-3p	XM_017316944.1	160.00	-23.23	2 13	202 222	11
mmu-miR-7221-3p	XM_006533569.3	148.00	-20.61	2 13	1 17	11
mmu-miR-7221-3p	NM_011724.3	141.00	-16.88	2 16	2037 2056	14
mmu-miR-7221-3p	NM_011724.3	140.00	-21.15	2 18	2990 3012	18
mmu-miR-7221-3p	XM_006499466.3	142.00	-30.35	2 20	281 302	19
mmu-miR-7221-3p	XM_006530585.3	149.00	-21.70	2 14	3269 3289	12
mmu-miR-7221-3p	XM_006530585.3	145.00	-27.01	2 20	1177 1196	18
mmu-miR-7221-3p	NM_199304.1	161.00	-27.98	2 18	243 263	16
mmu-miR-7221-3p	NM_199304.1	140.00	-15.12	2 9	1131 1151	7
mmu-miR-7221-3p	XM_011243586.2	145.00	-23.51	2 18	1816 1836	16
mmu-miR-7221-3p	XM_006511007.3	150.00	-22.40	2 20	2198 2222	22
mmu-miR-7221-3p	XM_006511007.3	144.00	-19.02	2 20	1638 1656	18
mmu-miR-7221-3p	XM_006511007.3	144.00	-21.18	2 20	3978 3998	19
mmu-miR-7221-3p	XM_006537508.2	147.00	-25.77	2 17	5811 5833	17
mmu-miR-7221-3p	XM_006519134.3	166.00	-33.43	2 20	2311 2333	20
mmu-miR-7226-5p	NM_172454.2	144.00	-23.59	2 22	457 483	21
mmu-miR-7226-5p	NM_172454.2	144.00	-20.13	3 22	941 967	20
mmu-miR-7226-5p	NM_028748.2	150.00	-19.01	2 15	103 128	13
mmu-miR-7226-5p	NM_028748.2	141.00	-22.92	3 25	599 622	22
mmu-miR-7226-5p	NM_028748.2	141.00	-17.14	2 25	2279 2302	23
mmu-miR-7226-5p	NM_133167.3	168.00	-24.47	2 25	1197 1222	23
mmu-miR-7226-5p	NM_133167.3	157.00	-30.29	2 15	1217 1243	14
mmu-miR-7226-5p	NM_133167.3	154.00	-23.26	2 25	577 601	23
mmu-miR-7226-5p	NM_133167.3	144.00	-29.76	2 25	2331 2354	23

mmu-miR-7226-5p	XM_006509465.2	144.00	-19.50	2 25	83 108	23
mmu-miR-7226-5p	XM_006538456.3	142.00	-23.60	2 23	1909 1931	21
mmu-miR-7226-5p	XM_006537815.3	148.00	-23.38	2 22	4141 4168	22
mmu-miR-7226-5p	XM_006537815.3	144.00	-23.42	2 23	444 468	21
mmu-miR-7226-5p	XM_006537815.3	144.00	-21.93	2 21	1755 1780	19
mmu-miR-7226-5p	XM_006537815.3	142.00	-25.29	2 22	5220 5245	21
mmu-miR-7226-5p	XM_006537815.3	140.00	-15.76	3 21	229 254	18
mmu-miR-7226-5p	NM_011082.3	149.00	-19.53	2 21	3584 3609	20
mmu-miR-7226-5p	NM_011082.3	143.00	-22.27	2 23	22 47	22
mmu-miR-7226-5p	NM_011082.3	140.00	-16.87	2 21	2717 2742	19
mmu-miR-7226-5p	XM_017314870.1	179.00	-33.07	2 25	5655 5681	24
mmu-miR-7226-5p	XM_017314870.1	156.00	-22.32	2 23	2339 2363	21
mmu-miR-7226-5p	XM_017314870.1	145.00	-22.71	2 25	7246 7269	23
mmu-miR-7226-5p	XM_017314870.1	142.00	-24.61	3 23	191 216	20
mmu-miR-7226-5p	XM_017314870.1	141.00	-19.36	2 18	6204 6227	16
mmu-miR-7226-5p	XM_017314870.1	140.00	-18.61	2 15	7266 7290	13
mmu-miR-7226-5p	XM_017317946.1	161.00	-27.52	2 24	13 37	22
mmu-miR-7226-5p	XM_017317946.1	149.00	-21.28	2 25	563 588	24
mmu-miR-7226-5p	NM_018807.5	159.00	-26.81	2 25	204 230	24
mmu-miR-7226-5p	NM_018807.5	154.00	-30.10	2 25	3520 3541	23
mmu-miR-7226-5p	NM_018807.5	150.00	-21.93	2 24	1114 1140	23
mmu-miR-7226-5p	NM_018807.5	144.00	-27.60	3 25	3389 3413	23
mmu-miR-7226-5p	NM_008872.3	143.00	-18.18	3 24	1400 1425	21
mmu-miR-7226-5p	XM_017312497.1	156.00	-25.72	3 22	2589 2615	20
mmu-miR-7226-5p	XM_017312497.1	146.00	-17.27	2 24	249 275	23
mmu-miR-7226-5p	XM_017312497.1	141.00	-22.34	2 22	4486 4511	20
mmu-miR-7226-5p	XM_011250776.2	155.00	-19.75	2 25	66 88	23
mmu-miR-7226-5p	NM_026385.4	145.00	-15.06	3 23	1541 1567	21
mmu-miR-7226-5p	NM_026385.4	140.00	-22.84	3 18	441 467	16
mmu-miR-7226-5p	XM_006502235.3	160.00	-20.81	2 25	39 64	23
mmu-miR-7226-5p	XM_006502235.3	159.00	-26.54	2 25	1869 1895	24
mmu-miR-7226-5p	XM_006502235.3	148.00	-22.47	3 22	451 478	21
mmu-miR-7226-5p	XM_006502235.3	147.00	-24.02	3 22	3664 3688	19
mmu-miR-7226-5p	XM_006502235.3	146.00	-25.07	2 25	2664 2691	25
mmu-miR-7226-5p	XM_006502235.3	141.00	-21.15	3 25	1373 1400	25
mmu-miR-7226-5p	XM_006512076.3	162.00	-24.37	2 23	5084 5109	21
mmu-miR-7226-5p	XM_006512076.3	159.00	-25.20	2 22	1661 1685	20
mmu-miR-7226-5p	XM_006512076.3	154.00	-26.75	2 23	5027 5052	21
mmu-miR-7226-5p	XM_006512076.3	153.00	-25.58	2 22	4379 4404	20
mmu-miR-7226-5p	XM_006512076.3	153.00	-20.64	2 20	5949 5973	18
mmu-miR-7226-5p	XM_006512076.3	145.00	-23.90	3 25	4304 4335	28
mmu-miR-7226-5p	NM_001302257.1	172.00	-34.86	2 25	1608 1633	23
mmu-miR-7226-5p	NM_001302257.1	146.00	-24.78	2 22	306 329	20
mmu-miR-7226-5p	NM_001291068.1	159.00	-27.19	2 20	4861 4886	18
mmu-miR-7226-5p	NM_001291068.1	150.00	-21.50	2 25	4017 4041	23
mmu-miR-7226-5p	NM_001291068.1	146.00	-22.48	2 25	2326 2347	23
mmu-miR-7226-5p	NM_148932.2	154.00	-21.71	2 21	5139 5166	21
mmu-miR-7226-5p	NM_148932.2	152.00	-25.35	2 25	1133 1156	23
mmu-miR-7226-5p	XM_006525698.2	166.00	-29.69	2 25	5791 5819	26
mmu-miR-7226-5p	XM_006525698.2	155.00	-24.85	2 22	4868 4892	20
mmu-miR-7226-5p	XM_006525698.2	152.00	-25.58	2 22	6204 6230	21
mmu-miR-7226-5p	XM_006525698.2	149.00	-25.89	3 25	5395 5424	26
mmu-miR-7226-5p	XM_006525698.2	147.00	-22.56	2 25	2636 2662	24
mmu-miR-7226-5p	XM_006525698.2	143.00	-19.42	2 22	5706 5733	22
mmu-miR-7226-5p	XM_006525698.2	143.00	-26.48	2 23	7003 7030	24
mmu-miR-7226-5p	XM_006525698.2	142.00	-19.84	3 24	8240 8268	24
mmu-miR-7226-5p	NM_026814.3	147.00	-25.81	2 25	671 698	25
mmu-miR-7226-5p	NM_001167908.1	141.00	-17.03	2 18	1306 1331	16
mmu-miR-7226-5p	XM_006527008.2	149.00	-22.26	3 25	3358 3387	26
mmu-miR-7226-5p	XM_006527008.2	148.00	-21.20	2 25	5011 5036	23
mmu-miR-7226-5p	XM_006527008.2	144.00	-20.02	2 25	1009 1034	23
mmu-miR-7226-5p	NM_011169.5	155.00	-21.79	2 25	580 608	26
mmu-miR-7226-5p	NM_011169.5	143.00	-20.94	2 25	10293 10319	24
mmu-miR-7226-5p	XM_011239558.2	153.00	-26.91	3 22	2176 2201	19
mmu-miR-7226-5p	NM_175022.2	144.00	-24.62	2 22	376 402	21

mmu-miR-7226-5p	NM_175022.2	140.00	-24.56	3 22	898 926	22
mmu-miR-7226-5p	NM_175022.2	140.00	-23.33	2 25	1768 1793	23
mmu-miR-7226-5p	NM_001081224.2	148.00	-20.33	3 21	1882 1907	18
mmu-miR-7226-5p	XM_011240476.2	161.00	-25.44	2 25	691 719	27
mmu-miR-7226-5p	XM_011240476.2	142.00	-20.82	3 19	2195 2220	16
mmu-miR-7226-5p	NM_008966.3	147.00	-23.04	2 22	1175 1206	26
mmu-miR-7226-5p	XM_006505015.3	147.00	-28.66	2 25	2009 2035	24
mmu-miR-7226-5p	XM_006505015.3	146.00	-20.11	2 21	4695 4719	19
mmu-miR-7226-5p	XM_006505015.3	143.00	-19.35	2 21	1817 1843	20
mmu-miR-7226-5p	XM_006505015.3	143.00	-17.81	2 24	5354 5382	26
mmu-miR-7226-5p	XM_006505015.3	140.00	-20.74	3 21	1116 1141	18
mmu-miR-7226-5p	XM_006514153.2	155.00	-31.18	2 25	3596 3625	27
mmu-miR-7226-5p	XM_006514153.2	152.00	-25.05	2 21	1361 1388	22
mmu-miR-7226-5p	XM_006514153.2	140.00	-15.77	3 25	3128 3153	22
mmu-miR-7226-5p	NM_009019.2	151.00	-26.14	2 25	5211 5235	24
mmu-miR-7226-5p	NM_009019.2	141.00	-23.83	2 25	3197 3223	25
mmu-miR-7226-5p	NM_009020.3	151.00	-29.29	3 25	194 222	25
mmu-miR-7226-5p	NM_009020.3	151.00	-24.75	2 25	3151 3177	24
mmu-miR-7226-5p	NM_009020.3	144.00	-20.72	2 21	1176 1201	19
mmu-miR-7226-5p	XM_017320735.1	141.00	-27.51	2 25	1867 1892	24
mmu-miR-7226-5p	XM_017320735.1	141.00	-17.33	2 24	2190 2214	22
mmu-miR-7226-5p	NM_016809.6	143.00	-15.84	2 17	450 476	16
mmu-miR-7226-5p	NM_016809.6	141.00	-16.41	2 23	1301 1328	23
mmu-miR-7226-5p	NM_011254.5	150.00	-19.39	2 19	651 676	17
mmu-miR-7226-5p	NM_011254.5	145.00	-22.91	2 25	1339 1359	23
mmu-miR-7226-5p	NM_031192.3	158.00	-31.31	3 25	1360 1388	25
mmu-miR-7226-5p	NM_001204959.1	147.00	-20.50	3 23	187 215	24
mmu-miR-7226-5p	XM_006529829.3	148.00	-19.81	2 22	1452 1478	21
mmu-miR-7226-5p	XM_006529829.3	147.00	-22.29	2 22	1119 1146	22
mmu-miR-7226-5p	XM_006529829.3	146.00	-25.45	2 25	2500 2529	27
mmu-miR-7226-5p	XM_006529829.3	142.00	-25.15	2 25	1838 1862	23
mmu-miR-7226-5p	XM_006529829.3	141.00	-17.80	3 22	753 778	19
mmu-miR-7226-5p	XM_011240779.2	149.00	-28.12	2 25	3421 3444	23
mmu-miR-7226-5p	XM_011240779.2	144.00	-26.25	2 22	2089 2116	22
mmu-miR-7226-5p	XM_011240779.2	144.00	-18.08	3 24	2440 2463	21
mmu-miR-7226-5p	XM_006507942.2	152.00	-27.39	3 25	2530 2552	22
mmu-miR-7226-5p	XM_006507942.2	143.00	-26.56	3 25	1446 1472	23
mmu-miR-7226-5p	NM_001195662.1	140.00	-21.16	2 24	931 956	23
mmu-miR-7226-5p	XM_006522506.3	144.00	-27.79	2 25	274 299	25
mmu-miR-7226-5p	XM_006525075.3	147.00	-27.53	2 25	718 747	27
mmu-miR-7226-5p	XM_006525075.3	140.00	-23.10	2 25	225 250	23
mmu-miR-7226-5p	NM_019732.2	148.00	-26.23	2 22	2145 2171	21
mmu-miR-7226-5p	NM_023893.4	140.00	-23.17	2 25	816 841	23
mmu-miR-7226-5p	XM_006540138.3	168.00	-25.82	2 22	880 906	21
mmu-miR-7226-5p	XM_006540138.3	140.00	-25.96	4 21	449 474	17
mmu-miR-7226-5p	XM_006540134.3	155.00	-23.35	2 25	2364 2390	24
mmu-miR-7226-5p	XM_006540134.3	151.00	-25.21	3 24	1178 1203	21
mmu-miR-7226-5p	XM_006540134.3	151.00	-24.89	2 25	2463 2492	27
mmu-miR-7226-5p	NM_009127.4	151.00	-25.45	2 21	1133 1160	21
mmu-miR-7226-5p	NM_009127.4	150.00	-31.69	2 25	1 25	23
mmu-miR-7226-5p	NM_018732.3	147.00	-20.77	2 22	5388 5415	22
mmu-miR-7226-5p	NM_018732.3	142.00	-18.62	2 25	6505 6529	23
mmu-miR-7226-5p	NM_018732.3	140.00	-20.83	2 25	1484 1509	23
mmu-miR-7226-5p	NM_018732.3	140.00	-17.32	2 9	1877 1902	7
mmu-miR-7226-5p	NM_009135.2	159.00	-24.59	2 25	4227 4249	23
mmu-miR-7226-5p	NM_009135.2	141.00	-18.35	2 18	724 749	16
mmu-miR-7226-5p	NM_026535.2	143.00	-20.90	3 22	3298 3322	19
mmu-miR-7226-5p	NM_009244.4	149.00	-23.83	2 25	1082 1111	28
mmu-miR-7226-5p	NM_008458.2	164.00	-27.65	2 25	1589 1614	23
mmu-miR-7226-5p	NM_008458.2	150.00	-27.64	2 25	352 374	23
mmu-miR-7226-5p	XM_006515637.2	160.00	-25.67	2 25	1324 1349	23
mmu-miR-7226-5p	XM_006515637.2	147.00	-24.74	2 25	78 100	23
mmu-miR-7226-5p	XM_011244046.2	150.00	-27.28	2 25	352 374	23
mmu-miR-7226-5p	XM_011244046.2	144.00	-23.10	3 25	1508 1532	23
mmu-miR-7226-5p	XM_011244046.2	143.00	-17.81	3 20	920 945	17

mmu-miR-7226-5p	XM_017322211.1	157.00	-24.65	2 22	1092 1117	20
mmu-miR-7226-5p	XM_017322211.1	157.00	-22.29	2 25	3270 3295	24
mmu-miR-7226-5p	XM_006530238.3	153.00	-27.09	3 25	4444 4467	22
mmu-miR-7226-5p	XM_006530238.3	143.00	-23.67	2 25	6357 6383	24
mmu-miR-7226-5p	XM_006530238.3	141.00	-24.69	2 24	4726 4754	25
mmu-miR-7226-5p	XM_006530238.3	140.00	-22.01	2 21	6784 6809	19
mmu-miR-7226-5p	XM_006530238.3	140.00	-18.79	2 9	8193 8218	7
mmu-miR-7226-5p	XM_017316715.1	145.00	-22.99	3 22	1942 1967	19
mmu-miR-7226-5p	XM_017316715.1	144.00	-23.31	2 23	334 358	21
mmu-miR-7226-5p	XM_017317034.1	153.00	-20.04	2 19	511 537	18
mmu-miR-7226-5p	XM_017317034.1	153.00	-29.12	2 23	827 853	22
mmu-miR-7226-5p	XM_017317034.1	149.00	-30.31	2 25	2513 2538	24
mmu-miR-7226-5p	XM_017317034.1	142.00	-26.59	2 25	3637 3665	26
mmu-miR-7226-5p	XM_017317034.1	141.00	-19.55	2 24	335 359	22
mmu-miR-7226-5p	XM_017317034.1	141.00	-25.10	2 18	4297 4322	16
mmu-miR-7226-5p	XM_017316897.1	145.00	-22.25	2 24	1643 1671	25
mmu-miR-7226-5p	XM_011250732.2	157.00	-22.79	2 22	1046 1069	20
mmu-miR-7226-5p	XM_011250732.2	155.00	-32.29	2 25	4190 4214	24
mmu-miR-7226-5p	XM_011250732.2	155.00	-30.43	2 25	6991 7017	24
mmu-miR-7226-5p	XM_011250732.2	151.00	-22.37	2 22	7043 7067	20
mmu-miR-7226-5p	XM_011250732.2	146.00	-27.13	2 25	6705 6735	28
mmu-miR-7226-5p	XM_011250732.2	142.00	-23.41	3 21	263 290	20
mmu-miR-7226-5p	XM_011250732.2	140.00	-22.66	2 21	5406 5431	19
mmu-miR-7226-5p	NM_177578.4	159.00	-22.33	2 25	2205 2231	24
mmu-miR-7226-5p	NM_177578.4	151.00	-14.88	2 24	1730 1755	22
mmu-miR-7226-5p	XM_006518339.3	164.00	-29.34	2 25	991 1016	23
mmu-miR-7226-5p	XM_006496884.3	149.00	-23.91	3 25	812 837	23
mmu-miR-7226-5p	XM_017313038.1	143.00	-18.58	2 18	195 222	18
mmu-miR-7226-5p	XM_017313038.1	143.00	-18.56	2 25	987 1015	26
mmu-miR-7226-5p	NM_001310705.1	144.00	-22.15	2 25	489 514	23
mmu-miR-7226-5p	NM_009199.2	158.00	-25.84	2 18	3101 3124	16
mmu-miR-7226-5p	NM_009199.2	146.00	-18.81	2 25	1802 1826	23
mmu-miR-7226-5p	NM_009199.2	142.00	-26.82	2 25	2138 2166	26
mmu-miR-7226-5p	NM_009199.2	140.00	-21.78	3 25	507 532	22
mmu-miR-7226-5p	NM_001033167.3	159.00	-26.76	2 20	4917 4942	18
mmu-miR-7226-5p	NM_001033167.3	156.00	-26.15	2 23	3830 3854	21
mmu-miR-7226-5p	NM_001033167.3	152.00	-28.27	2 25	4265 4287	23
mmu-miR-7226-5p	NM_001033167.3	151.00	-23.61	2 18	2816 2840	16
mmu-miR-7226-5p	NM_001033167.3	150.00	-26.14	3 22	5439 5465	21
mmu-miR-7226-5p	NM_001033167.3	145.00	-23.24	3 25	1440 1465	23
mmu-miR-7226-5p	NM_001033167.3	142.00	-18.64	2 11	5010 5035	9
mmu-miR-7226-5p	NM_001033167.3	141.00	-29.08	2 25	1862 1887	24
mmu-miR-7226-5p	NM_001007570.2	144.00	-20.15	2 21	1972 1997	19
mmu-miR-7226-5p	NM_001007570.2	140.00	-25.70	2 25	1365 1390	23
mmu-miR-7226-5p	NM_001033286.2	162.00	-26.87	2 25	4707 4734	25
mmu-miR-7226-5p	NM_001033286.2	154.00	-19.71	2 25	3979 4003	23
mmu-miR-7226-5p	NM_001290993.1	151.00	-25.73	2 24	1217 1242	24
mmu-miR-7226-5p	NM_001290993.1	140.00	-26.65	2 9	5 30	7
mmu-miR-7226-5p	XM_006503080.3	158.00	-20.72	2 25	3751 3778	25
mmu-miR-7226-5p	XM_006503080.3	153.00	-26.96	2 25	3679 3702	23
mmu-miR-7226-5p	XM_006503080.3	143.00	-26.87	2 25	1849 1872	24
mmu-miR-7226-5p	XM_006503080.3	142.00	-21.88	2 15	3098 3123	13
mmu-miR-7226-5p	XM_006503080.3	142.00	-16.53	2 23	4565 4590	21
mmu-miR-7226-5p	XM_006503080.3	141.00	-23.43	3 23	776 802	21
mmu-miR-7226-5p	XM_006503080.3	140.00	-20.58	2 20	741 767	20
mmu-miR-7226-5p	NM_027052.3	151.00	-19.75	3 25	842 869	24
mmu-miR-7226-5p	XM_017322365.1	151.00	-26.59	2 25	1234 1261	25
mmu-miR-7226-5p	XM_017322365.1	143.00	-23.60	2 22	1269 1293	20
mmu-miR-7226-5p	XM_006538687.2	156.00	-30.64	2 25	1254 1279	23
mmu-miR-7226-5p	XM_006538687.2	141.00	-23.93	2 24	2568 2589	22
mmu-miR-7226-5p	NM_001146217.1	163.00	-25.23	2 25	2973 2999	24
mmu-miR-7226-5p	NM_001146217.1	141.00	-22.68	2 18	3293 3318	16
mmu-miR-7226-5p	NM_009223.3	142.00	-20.75	3 22	2484 2507	19
mmu-miR-7226-5p	NM_009223.3	141.00	-27.30	2 25	289 314	24
mmu-miR-7226-5p	NM_031183.2	157.00	-22.86	2 22	2688 2713	20

mmu-miR-7226-5p	NM_031183.2	141.00	-20.07	2 24	852 879	24
mmu-miR-7226-5p	XM_006539074.3	148.00	-21.88	3 23	10008 10035	22
mmu-miR-7226-5p	XM_006539074.3	141.00	-23.64	2 23	7817 7843	22
mmu-miR-7226-5p	NM_178753.4	161.00	-29.39	2 25	819 844	24
mmu-miR-7226-5p	NM_178753.4	149.00	-20.37	3 24	1028 1052	21
mmu-miR-7226-5p	NM_001304266.1	167.00	-19.96	2 24	5784 5809	22
mmu-miR-7226-5p	NM_001304266.1	164.00	-28.24	2 25	4230 4255	23
mmu-miR-7226-5p	NM_001304266.1	158.00	-25.03	2 25	4870 4898	26
mmu-miR-7226-5p	NM_001304266.1	155.00	-26.16	2 23	8481 8509	25
mmu-miR-7226-5p	NM_001304266.1	150.00	-35.36	2 25	6072 6100	26
mmu-miR-7226-5p	NM_001304266.1	148.00	-23.18	2 21	5856 5881	19
mmu-miR-7226-5p	NM_001304266.1	145.00	-19.70	3 24	6716 6740	21
mmu-miR-7226-5p	NM_001304266.1	143.00	-22.59	2 24	6608 6633	22
mmu-miR-7226-5p	NM_001242411.1	168.00	-31.27	2 25	3863 3889	25
mmu-miR-7226-5p	NM_001242411.1	155.00	-22.42	2 25	4457 4484	25
mmu-miR-7226-5p	NM_001242411.1	146.00	-22.60	2 25	1437 1465	26
mmu-miR-7226-5p	NM_001242411.1	143.00	-21.92	2 23	3375 3396	21
mmu-miR-7226-5p	NM_001242411.1	140.00	-22.47	3 25	2340 2365	22
mmu-miR-7226-5p	XM_006506145.1	150.00	-22.15	3 25	7485 7513	25
mmu-miR-7226-5p	XM_006506145.1	149.00	-22.95	2 21	7390 7413	19
mmu-miR-7226-5p	XM_006506145.1	146.00	-20.49	2 19	3720 3743	17
mmu-miR-7226-5p	XM_006506145.1	145.00	-19.05	3 22	734 759	19
mmu-miR-7226-5p	XM_006506145.1	145.00	-21.15	2 24	7904 7928	22
mmu-miR-7226-5p	XM_006506145.1	143.00	-20.82	2 24	4747 4769	22
mmu-miR-7226-5p	XM_006506145.1	143.00	-18.06	2 20	7401 7426	18
mmu-miR-7226-5p	XM_006506145.1	143.00	-18.58	3 23	8601 8624	20
mmu-miR-7226-5p	XM_006506145.1	142.00	-22.01	3 24	8321 8343	21
mmu-miR-7226-5p	XM_011248193.2	160.00	-24.66	2 25	5620 5646	25
mmu-miR-7226-5p	XM_011248193.2	156.00	-29.19	2 25	6097 6119	23
mmu-miR-7226-5p	XM_011245529.2	154.00	-18.46	2 25	4538 4566	26
mmu-miR-7226-5p	XM_011245529.2	143.00	-22.55	2 24	3125 3150	22
mmu-miR-7226-5p	XM_011245529.2	142.00	-26.99	2 21	2081 2105	19
mmu-miR-7226-5p	XM_011245529.2	140.00	-27.19	2 18	605 631	17
mmu-miR-7226-5p	NM_011374.2	154.00	-32.28	2 25	3170 3199	27
mmu-miR-7226-5p	NM_011374.2	150.00	-20.63	2 24	7878 7905	24
mmu-miR-7226-5p	NM_011374.2	145.00	-19.15	3 24	801 825	21
mmu-miR-7226-5p	NM_011374.2	141.00	-20.89	2 15	7616 7642	14
mmu-miR-7226-5p	NM_011374.2	140.00	-21.42	3 25	3005 3029	23
mmu-miR-7226-5p	XM_006527452.3	141.00	-28.21	3 22	1853 1878	19
mmu-miR-7226-5p	NM_032400.2	145.00	-21.39	3 23	1546 1571	21
mmu-miR-7226-5p	XM_017316056.1	173.00	-27.83	2 25	1950 1975	24
mmu-miR-7226-5p	NM_009332.3	159.00	-24.37	2 25	269 296	25
mmu-miR-7226-5p	NM_009332.3	143.00	-19.64	2 25	1805 1832	25
mmu-miR-7226-5p	NM_001080979.1	147.00	-22.05	2 24	1095 1120	22
mmu-miR-7226-5p	NM_001080979.1	140.00	-22.43	2 17	601 626	15
mmu-miR-7226-5p	NM_153801.3	168.00	-33.43	2 25	1057 1082	23
mmu-miR-7226-5p	NM_153801.3	156.00	-21.01	2 24	196 221	23
mmu-miR-7226-5p	NM_153801.3	143.00	-18.39	2 24	1519 1544	22
mmu-miR-7226-5p	NM_011581.3	153.00	-25.64	2 14	181 206	12
mmu-miR-7226-5p	NM_011581.3	150.00	-23.23	2 23	291 316	21
mmu-miR-7226-5p	NM_011581.3	150.00	-25.35	3 21	2073 2097	18
mmu-miR-7226-5p	NM_011581.3	145.00	-23.91	2 23	785 809	22
mmu-miR-7226-5p	NM_011581.3	141.00	-19.32	2 23	2046 2073	23
mmu-miR-7226-5p	NM_011581.3	140.00	-18.16	2 25	1213 1236	23
mmu-miR-7226-5p	NM_009379.3	159.00	-28.46	2 25	1226 1248	23
mmu-miR-7226-5p	NM_009379.3	144.00	-19.80	2 23	1105 1129	21
mmu-miR-7226-5p	NM_009381.3	149.00	-25.84	2 25	955 986	29
mmu-miR-7226-5p	XM_006501237.3	145.00	-19.63	2 22	59 84	20
mmu-miR-7226-5p	XM_006501237.3	144.00	-22.66	2 22	130 157	22
mmu-miR-7226-5p	XM_006537836.1	153.00	-20.33	3 22	637 662	19
mmu-miR-7226-5p	XM_006537836.1	142.00	-15.47	3 24	956 978	21
mmu-miR-7226-5p	XM_006533452.3	159.00	-23.93	2 24	3166 3191	22
mmu-miR-7226-5p	XM_006533452.3	152.00	-23.72	2 15	874 901	15
mmu-miR-7226-5p	XM_006533452.3	151.00	-24.85	3 20	4550 4575	17
mmu-miR-7226-5p	XM_006533452.3	148.00	-27.07	2 25	4905 4930	23

mmu-miR-7226-5p	XM_006533452.3	147.00	-22.36	2 25	5161 5188	25
mmu-miR-7226-5p	XM_006533452.3	146.00	-19.93	2 24	3328 3354	23
mmu-miR-7226-5p	XM_006515787.3	145.00	-20.21	3 23	1216 1242	21
mmu-miR-7226-5p	XM_006515787.3	143.00	-28.09	2 25	172 199	25
mmu-miR-7226-5p	XM_006515787.3	143.00	-21.32	2 22	601 625	20
mmu-miR-7226-5p	XM_006515787.3	140.00	-19.15	3 23	1082 1110	23
mmu-miR-7226-5p	NM_178715.3	149.00	-21.63	2 19	1594 1620	18
mmu-miR-7226-5p	NM_178715.3	142.00	-24.18	2 24	2350 2376	23
mmu-miR-7226-5p	NM_178715.3	140.00	-17.63	2 23	985 1009	21
mmu-miR-7226-5p	NM_144936.1	152.00	-22.62	2 25	1011 1035	24
mmu-miR-7226-5p	NM_144936.1	152.00	-22.62	2 25	1047 1071	24
mmu-miR-7226-5p	NM_144936.1	152.00	-22.62	2 25	1083 1107	24
mmu-miR-7226-5p	NM_144936.1	142.00	-20.22	2 25	780 808	26
mmu-miR-7226-5p	NM_144936.1	140.00	-21.71	3 21	524 547	18
mmu-miR-7226-5p	NM_177371.3	159.00	-23.39	2 22	481 505	20
mmu-miR-7226-5p	NM_177371.3	150.00	-25.47	2 25	4868 4896	26
mmu-miR-7226-5p	XM_006496905.1	150.00	-21.42	2 23	3866 3891	21
mmu-miR-7226-5p	XM_006496905.1	147.00	-22.66	2 25	4598 4624	24
mmu-miR-7226-5p	XM_006496905.1	144.00	-26.92	3 25	5496 5521	22
mmu-miR-7226-5p	XM_011248201.2	150.00	-24.47	2 23	1882 1907	21
mmu-miR-7226-5p	XM_011248201.2	148.00	-24.31	2 22	948 974	21
mmu-miR-7226-5p	XM_011248201.2	143.00	-22.91	3 25	1633 1659	23
mmu-miR-7226-5p	NM_146241.2	154.00	-26.15	2 25	539 568	27
mmu-miR-7226-5p	NM_146241.2	144.00	-20.78	2 21	2311 2336	19
mmu-miR-7226-5p	XM_011249157.2	167.00	-27.68	2 25	2020 2046	24
mmu-miR-7226-5p	XM_011249157.2	164.00	-26.04	2 22	2196 2222	21
mmu-miR-7226-5p	XM_011249157.2	156.00	-22.97	2 25	1346 1373	26
mmu-miR-7226-5p	XM_011249157.2	155.00	-20.87	2 22	1370 1394	20
mmu-miR-7226-5p	XM_011249157.2	146.00	-20.59	3 19	3310 3335	16
mmu-miR-7226-5p	XM_011249157.2	142.00	-24.20	3 25	559 586	24
mmu-miR-7226-5p	XM_011249157.2	141.00	-16.29	3 19	1878 1904	17
mmu-miR-7226-5p	NM_001243916.1	154.00	-21.25	2 25	564 591	25
mmu-miR-7226-5p	NM_001243916.1	142.00	-19.10	2 24	1472 1494	22
mmu-miR-7226-5p	NM_001039047.1	147.00	-21.79	2 25	815 843	26
mmu-miR-7226-5p	XM_006508002.3	147.00	-21.31	3 21	793 819	19
mmu-miR-7226-5p	XM_006508002.3	141.00	-19.23	2 22	1200 1225	20
mmu-miR-7226-5p	XM_006506152.3	159.00	-24.93	2 25	1980 2007	25
mmu-miR-7226-5p	NM_001033149.3	155.00	-25.89	2 25	3720 3750	28
mmu-miR-7226-5p	NM_001033149.3	152.00	-21.58	3 25	2890 2915	22
mmu-miR-7226-5p	NM_001033149.3	145.00	-19.77	2 25	4507 4532	24
mmu-miR-7226-5p	NM_001033149.3	141.00	-18.52	3 24	3945 3972	23
mmu-miR-7226-5p	NM_177709.3	155.00	-26.63	2 25	1722 1748	24
mmu-miR-7226-5p	NM_177709.3	150.00	-19.44	2 21	25 49	19
mmu-miR-7226-5p	XM_006538224.1	145.00	-20.49	2 22	3704 3729	20
mmu-miR-7226-5p	XM_006538224.1	140.00	-22.58	3 23	4040 4067	22
mmu-miR-7226-5p	XM_006501309.3	145.00	-25.33	2 25	572 600	27
mmu-miR-7226-5p	XM_006501309.3	144.00	-22.69	2 23	2232 2259	23
mmu-miR-7226-5p	XM_006525099.3	146.00	-19.75	3 21	1367 1391	18
mmu-miR-7226-5p	XM_011248394.1	157.00	-21.18	2 25	2304 2329	24
mmu-miR-7226-5p	XM_011248394.1	152.00	-17.21	2 24	3168 3191	22
mmu-miR-7226-5p	XM_011248394.1	147.00	-23.45	2 25	2072 2098	24
mmu-miR-7226-5p	XM_011248394.1	141.00	-24.50	2 25	3605 3628	23
mmu-miR-7226-5p	NM_009504.4	158.00	-24.88	2 20	3650 3676	19
mmu-miR-7226-5p	NM_009504.4	153.00	-23.20	2 25	852 875	23
mmu-miR-7226-5p	XM_017316944.1	169.00	-26.95	2 25	255 278	23
mmu-miR-7226-5p	NM_009514.4	140.00	-22.29	2 25	36 61	23
mmu-miR-7226-5p	NM_177789.4	161.00	-33.37	2 25	1028 1053	24
mmu-miR-7226-5p	XM_006541047.3	142.00	-25.25	2 25	1264 1291	25
mmu-miR-7226-5p	NM_011724.3	150.00	-17.35	2 25	1209 1233	23
mmu-miR-7226-5p	NM_011724.3	147.00	-22.49	2 18	2379 2403	16
mmu-miR-7226-5p	NM_011724.3	143.00	-17.15	2 15	5572 5595	13
mmu-miR-7226-5p	XM_017318541.1	140.00	-16.97	3 24	820 847	24
mmu-miR-7226-5p	XM_006499466.3	145.00	-18.58	2 16	1857 1881	14
mmu-miR-7226-5p	XM_006499466.3	142.00	-29.42	2 25	2784 2812	26
mmu-miR-7226-5p	XM_006530585.3	150.00	-21.51	2 23	10067 10092	21

mmu-miR-7226-5p	XM_017314347.1	146.00	-21.39	3 23	532 557	20
mmu-miR-7226-5p	XM_017314347.1	140.00	-19.32	2 25	2 27	23
mmu-miR-7226-5p	NM_199304.1	150.00	-26.76	2 15	434 459	13
mmu-miR-7226-5p	NM_199029.2	149.00	-22.16	2 25	3522 3551	27
mmu-miR-7226-5p	NM_199029.2	141.00	-20.23	2 25	1914 1939	24
mmu-miR-7226-5p	NM_199029.2	140.00	-21.54	2 25	2667 2692	23
mmu-miR-7226-5p	XM_006511007.3	153.00	-25.31	3 24	7127 7151	21
mmu-miR-7226-5p	XM_006511007.3	150.00	-25.26	2 22	4442 4467	21
mmu-miR-7226-5p	XM_006511007.3	147.00	-22.62	2 25	7439 7466	25
mmu-miR-7226-5p	XM_006511007.3	145.00	-22.61	2 23	911 938	23
mmu-miR-7226-5p	XM_006511007.3	141.00	-27.92	2 22	7175 7200	20
mmu-miR-7226-5p	NM_001110508.1	141.00	-23.35	2 25	2710 2736	25
mmu-miR-7226-5p	NM_001110508.1	140.00	-20.97	4 25	3154 3179	21
mmu-miR-7226-5p	XM_006537508.2	163.00	-24.26	2 21	6326 6354	22
mmu-miR-7226-5p	XM_006537508.2	140.00	-17.46	3 25	3386 3411	22
mmu-miR-7226-5p	XM_006519134.3	145.00	-30.39	2 23	2255 2281	22
mmu-miR-7226-5p	XM_006519134.3	143.00	-24.12	2 25	5465 5487	23
mmu-miR-7226-5p	XM_006519134.3	140.00	-21.51	2 23	6171 6198	23
mmu-miR-3473f	NM_013743.2	149.00	-10.65	2 16	2529 2547	14
mmu-miR-3473f	NM_001290822.1	140.00	-17.01	3 18	1116 1136	16
mmu-miR-3473f	NM_001164593.1	158.00	-12.56	2 19	3508 3527	17
mmu-miR-3473f	NM_001164593.1	141.00	-6.38	2 15	3445 3465	14
mmu-miR-3473f	XM_006538456.3	140.00	-9.79	2 19	5558 5576	17
mmu-miR-3473f	XM_017314870.1	145.00	-15.15	2 19	7128 7149	19
mmu-miR-3473f	XM_017317946.1	142.00	-9.76	2 16	1050 1072	17
mmu-miR-3473f	XM_006502235.3	150.00	-15.16	2 17	3161 3179	15
mmu-miR-3473f	XM_006502235.3	141.00	-11.29	2 18	2272 2291	16
mmu-miR-3473f	NM_001302257.1	141.00	-6.91	2 18	883 902	16
mmu-miR-3473f	NM_148932.2	149.00	-15.20	2 19	4078 4093	17
mmu-miR-3473f	XM_006525698.2	146.00	-10.38	2 19	11135 11154	17
mmu-miR-3473f	XM_006525698.2	140.00	-9.86	2 17	11280 11299	15
mmu-miR-3473f	XM_006527008.2	143.00	-13.13	2 16	2449 2468	14
mmu-miR-3473f	NM_011169.5	148.00	-15.39	2 19	5683 5701	17
mmu-miR-3473f	NM_011169.5	146.00	-8.83	2 19	10241 10260	17
mmu-miR-3473f	XM_011239558.2	145.00	-11.66	2 10	2995 3014	8
mmu-miR-3473f	XM_011239558.2	142.00	-12.65	2 15	4609 4628	13
mmu-miR-3473f	XM_011239558.2	141.00	-13.26	2 14	3037 3056	12
mmu-miR-3473f	NM_175563.5	140.00	-6.56	2 9	3593 3612	7
mmu-miR-3473f	NM_175022.2	146.00	-8.38	2 19	6564 6583	17
mmu-miR-3473f	NM_175022.2	145.00	-13.68	2 19	6709 6730	19
mmu-miR-3473f	NM_008966.3	148.00	-8.50	2 18	1685 1705	17
mmu-miR-3473f	XM_011240779.2	147.00	-13.74	2 12	553 572	10
mmu-miR-3473f	XM_006507942.2	154.00	-20.11	2 16	1588 1608	15
mmu-miR-3473f	NM_001195662.1	148.00	-12.47	2 18	1531 1553	19
mmu-miR-3473f	XM_006540134.3	140.00	-11.43	2 18	2447 2467	17
mmu-miR-3473f	NM_018732.3	151.00	-11.20	2 16	3918 3937	14
mmu-miR-3473f	NM_018732.3	140.00	-13.54	2 18	6454 6475	18
mmu-miR-3473f	NM_026535.2	152.00	-14.61	2 14	3342 3362	13
mmu-miR-3473f	XM_006521796.1	149.00	-17.80	2 18	1600 1619	16
mmu-miR-3473f	XM_017322211.1	149.00	-14.96	2 19	2478 2498	18
mmu-miR-3473f	XM_017322211.1	147.00	-10.83	2 16	6425 6444	14
mmu-miR-3473f	XM_017316715.1	144.00	-10.01	2 17	4491 4510	15
mmu-miR-3473f	XM_006518339.3	153.00	-10.79	2 19	2619 2639	18
mmu-miR-3473f	NM_144539.5	147.00	-18.75	2 14	1150 1168	12
mmu-miR-3473f	NM_009199.2	141.00	-16.48	2 19	533 553	18
mmu-miR-3473f	NM_009199.2	141.00	-10.48	2 19	971 991	18
mmu-miR-3473f	NM_001007570.2	152.00	-14.49	2 18	1548 1568	17
mmu-miR-3473f	NM_001007570.2	148.00	-14.54	2 17	3063 3082	15
mmu-miR-3473f	NM_001033286.2	140.00	-14.24	2 19	2425 2443	17
mmu-miR-3473f	XM_006503080.3	146.00	-14.31	2 19	2147 2166	17
mmu-miR-3473f	XM_006503080.3	142.00	-18.01	2 19	2740 2759	17
mmu-miR-3473f	NM_027052.3	141.00	-16.70	2 18	715 734	16
mmu-miR-3473f	NM_001146217.1	143.00	-20.78	2 16	3294 3313	14
mmu-miR-3473f	NM_001304266.1	160.00	-12.87	2 19	7223 7241	17
mmu-miR-3473f	NM_001242411.1	145.00	-16.83	2 18	5666 5685	16

mmu-miR-3473f	NM_001242411.1	142.00	-21.19	2 18	4384 4401	16
mmu-miR-3473f	XM_011245529.2	144.00	-11.51	2 17	3040 3059	15
mmu-miR-3473f	XM_006527452.3	141.00	-16.19	2 19	1326 1346	18
mmu-miR-3473f	NM_032400.2	141.00	-16.15	2 19	1413 1435	20
mmu-miR-3473f	XM_006533452.3	145.00	-8.42	2 10	4407 4426	8
mmu-miR-3473f	XM_006496905.1	142.00	-13.88	2 19	3866 3885	17
mmu-miR-3473f	NM_177409.3	151.00	-12.05	2 16	241 260	14
mmu-miR-3473f	NM_146241.2	141.00	-15.30	2 15	949 970	15
mmu-miR-3473f	NM_001033149.3	145.00	-12.21	2 10	4582 4601	8
mmu-miR-3473f	NM_001033149.3	141.00	-15.90	2 19	4639 4659	18
mmu-miR-3473f	XM_011248394.1	150.00	-21.66	2 19	3421 3440	17
mmu-miR-3473f	XM_011248394.1	148.00	-16.17	2 18	2087 2107	17
mmu-miR-3473f	XM_011248394.1	140.00	-15.93	3 19	2389 2407	16
mmu-miR-3473f	XM_006499466.3	140.00	-7.00	2 9	1 11	7
mmu-miR-3473f	XM_006530585.3	149.00	-7.97	2 19	13871 13891	18
mmu-miR-3473f	NM_199029.2	144.00	-20.77	2 19	1328 1349	19
mmu-miR-3473f	XM_006511007.3	156.00	-21.14	2 18	3272 3293	18
mmu-miR-3473f	XM_006511007.3	142.00	-15.65	2 18	5144 5161	16
mmu-miR-3473f	XM_006511007.3	141.00	-14.82	2 19	5668 5688	18
mmu-miR-3473f	XM_006519134.3	164.00	-30.26	2 17	4039 4058	15
mmu-miR-8094	NM_172454.2	141.00	-13.58	2 19	366 387	18
mmu-miR-8094	NM_028748.2	145.00	-13.79	2 10	2879 2899	8
mmu-miR-8094	NM_013743.2	141.00	-10.29	2 10	566 586	8
mmu-miR-8094	XM_006509465.2	140.00	-11.43	2 9	789 809	7
mmu-miR-8094	NM_001290822.1	151.00	-17.62	2 13	1245 1266	12
mmu-miR-8094	NM_001290822.1	140.00	-15.21	2 19	1500 1519	17
mmu-miR-8094	XM_006538456.3	143.00	-9.86	3 20	1157 1177	17
mmu-miR-8094	XM_006537815.3	155.00	-14.46	2 20	6387 6407	18
mmu-miR-8094	NM_011082.3	145.00	-17.47	2 20	2426 2450	22
mmu-miR-8094	NM_011082.3	145.00	-12.13	2 18	2884 2904	16
mmu-miR-8094	NM_011082.3	140.00	-9.41	2 9	1085 1105	7
mmu-miR-8094	XM_017314870.1	145.00	-13.01	2 10	8084 8104	8
mmu-miR-8094	XM_017314870.1	141.00	-11.49	2 10	2849 2869	8
mmu-miR-8094	NM_018807.5	162.00	-16.82	2 20	4053 4074	19
mmu-miR-8094	NM_018807.5	151.00	-13.39	2 19	3422 3442	18
mmu-miR-8094	NM_018807.5	146.00	-14.93	2 15	2206 2226	13
mmu-miR-8094	NM_018807.5	145.00	-13.21	2 10	1974 1994	8
mmu-miR-8094	NM_018807.5	145.00	-13.43	2 18	2487 2507	16
mmu-miR-8094	NM_018807.5	145.00	-15.63	2 10	3509 3529	8
mmu-miR-8094	XM_017312497.1	151.00	-13.39	2 20	1199 1219	18
mmu-miR-8094	NM_026385.4	141.00	-10.41	2 19	821 842	18
mmu-miR-8094	XM_006502235.3	142.00	-9.32	2 11	2783 2803	9
mmu-miR-8094	NM_001195084.1	141.00	-15.15	2 20	284 303	18
mmu-miR-8094	XM_006512076.3	144.00	-22.82	3 19	1162 1184	18
mmu-miR-8094	NM_148932.2	155.00	-13.99	2 18	3368 3387	16
mmu-miR-8094	NM_148932.2	141.00	-10.03	2 17	2670 2688	15
mmu-miR-8094	NM_148932.2	141.00	-15.70	3 18	2740 2760	15
mmu-miR-8094	NM_011145.3	148.00	-16.30	2 19	1226 1248	19
mmu-miR-8094	XM_006525698.2	153.00	-19.40	2 14	6676 6696	12
mmu-miR-8094	NM_001167908.1	150.00	-17.00	2 18	1534 1552	16
mmu-miR-8094	NM_001167908.1	146.00	-20.75	3 20	1588 1611	20
mmu-miR-8094	XM_006527008.2	162.00	-20.92	2 18	3013 3033	17
mmu-miR-8094	NM_011169.5	141.00	-10.86	2 19	9963 9985	19
mmu-miR-8094	NM_175022.2	153.00	-21.16	2 20	6048 6067	18
mmu-miR-8094	NM_175022.2	145.00	-12.22	2 10	509 529	8
mmu-miR-8094	NM_001081224.2	154.00	-16.14	2 17	1861 1880	15
mmu-miR-8094	XM_006505015.3	150.00	-14.38	2 11	2489 2509	9
mmu-miR-8094	XM_006514153.2	140.00	-9.35	2 9	1451 1471	7
mmu-miR-8094	NM_009019.2	152.00	-16.86	2 14	3930 3952	14
mmu-miR-8094	NM_009019.2	149.00	-13.55	2 19	141 162	18
mmu-miR-8094	NM_009020.3	154.00	-16.57	2 18	107 125	16
mmu-miR-8094	XM_006514077.1	147.00	-15.61	2 12	184 204	10
mmu-miR-8094	XM_006514077.1	144.00	-10.36	2 13	421 441	11
mmu-miR-8094	NM_031192.3	155.00	-15.74	2 17	75 96	16
mmu-miR-8094	XM_006529829.3	160.00	-18.04	2 20	5124 5145	20

mmu-miR-8094	XM_011240779.2	142.00	-12.13	2 16	1283 1304	15
mmu-miR-8094	XM_006507942.2	141.00	-16.54	3 20	1436 1455	17
mmu-miR-8094	XM_006522506.3	151.00	-17.06	2 20	505 525	18
mmu-miR-8094	NM_019732.2	150.00	-15.15	2 17	2840 2863	18
mmu-miR-8094	NM_018732.3	151.00	-19.61	2 20	4164 4184	18
mmu-miR-8094	NM_018732.3	150.00	-20.73	2 20	3072 3095	21
mmu-miR-8094	NM_018732.3	140.00	-12.19	2 9	1677 1697	7
mmu-miR-8094	NM_018732.3	140.00	-11.48	2 9	8980 9000	7
mmu-miR-8094	NM_009135.2	155.00	-19.20	2 20	5921 5941	18
mmu-miR-8094	NM_009135.2	146.00	-14.81	2 19	3415 3435	17
mmu-miR-8094	XM_017322211.1	146.00	-15.71	2 16	1382 1404	16
mmu-miR-8094	XM_017317034.1	145.00	-13.01	2 10	3449 3469	8
mmu-miR-8094	NM_177578.4	152.00	-18.47	2 18	2620 2642	18
mmu-miR-8094	NM_177578.4	148.00	-12.68	2 20	1669 1690	20
mmu-miR-8094	NM_177578.4	142.00	-10.80	2 11	3020 3040	9
mmu-miR-8094	NM_009199.2	142.00	-9.57	2 11	3094 3114	9
mmu-miR-8094	NM_001033167.3	151.00	-15.68	2 18	4748 4770	18
mmu-miR-8094	NM_001033167.3	142.00	-15.47	3 19	3119 3139	16
mmu-miR-8094	NM_001007570.2	145.00	-15.30	2 10	1211 1231	8
mmu-miR-8094	NM_001033286.2	152.00	-14.69	2 13	4051 4071	11
mmu-miR-8094	NM_001033286.2	143.00	-20.81	2 20	1376 1396	18
mmu-miR-8094	NM_001033286.2	141.00	-13.60	3 18	5211 5231	15
mmu-miR-8094	NM_001033286.2	141.00	-12.56	2 10	5308 5328	8
mmu-miR-8094	XM_006503080.3	150.00	-14.58	2 11	3853 3873	9
mmu-miR-8094	XM_006503080.3	144.00	-17.47	2 19	4093 4112	17
mmu-miR-8094	XM_006503080.3	141.00	-20.20	3 20	3913 3932	17
mmu-miR-8094	NM_027052.3	142.00	-12.86	2 20	1413 1435	20
mmu-miR-8094	XM_017322365.1	163.00	-18.32	2 20	869 887	18
mmu-miR-8094	NM_001146217.1	157.00	-20.73	2 20	2869 2888	18
mmu-miR-8094	XM_006534520.3	158.00	-18.90	2 20	237 258	19
mmu-miR-8094	NM_031183.2	145.00	-14.01	2 10	1782 1802	8
mmu-miR-8094	NM_019866.1	147.00	-14.56	2 12	98 118	10
mmu-miR-8094	NM_001304266.1	155.00	-15.72	2 16	354 374	14
mmu-miR-8094	NM_001242411.1	153.00	-18.96	2 19	3814 3835	18
mmu-miR-8094	NM_001242411.1	148.00	-13.00	2 17	5388 5408	15
mmu-miR-8094	NM_001242411.1	146.00	-17.19	3 19	1643 1663	16
mmu-miR-8094	NM_001242411.1	140.00	-9.32	2 9	7642 7662	7
mmu-miR-8094	XM_006506145.1	156.00	-12.37	2 19	7573 7592	17
mmu-miR-8094	XM_011248193.2	146.00	-12.67	2 20	3375 3397	20
mmu-miR-8094	NM_011374.2	143.00	-10.25	2 20	4924 4944	18
mmu-miR-8094	NM_011581.3	148.00	-13.73	2 17	3404 3424	15
mmu-miR-8094	NM_009379.3	142.00	-14.44	2 20	993 1016	21
mmu-miR-8094	NM_009379.3	140.00	-10.81	2 9	355 375	7
mmu-miR-8094	XM_006501237.3	142.00	-15.05	3 15	985 1005	12
mmu-miR-8094	XM_006533479.2	146.00	-12.97	2 13	563 582	11
mmu-miR-8094	XM_006521428.3	151.00	-17.50	2 16	628 648	14
mmu-miR-8094	NM_177371.3	150.00	-19.48	2 20	1494 1516	20
mmu-miR-8094	XM_006529382.3	146.00	-19.78	3 19	531 551	16
mmu-miR-8094	XM_011249157.2	148.00	-12.62	2 17	3209 3229	15
mmu-miR-8094	XM_006506152.3	150.00	-15.91	2 16	3728 3750	16
mmu-miR-8094	XM_006525099.3	147.00	-13.85	2 20	1686 1705	19
mmu-miR-8094	NM_001244031.1	144.00	-12.79	2 20	518 544	24
mmu-miR-8094	NM_011704.3	148.00	-12.37	2 17	2118 2138	15
mmu-miR-8094	XM_017314347.1	142.00	-11.79	2 12	373 395	12
mmu-miR-8094	XM_017314347.1	140.00	-10.14	2 9	580 600	7
mmu-miR-8094	NM_199029.2	146.00	-20.82	3 19	3670 3690	16
mmu-miR-8094	XM_006537508.2	142.00	-12.51	2 15	3850 3870	13
mmu-miR-8094	XM_006537508.2	141.00	-12.12	2 10	6784 6804	8
mmu-miR-8117	NM_028748.2	140.00	-19.30	2 19	3346 3368	19
mmu-miR-8117	NM_013743.2	140.00	-12.33	2 9	1466 1486	7
mmu-miR-8117	NM_001290822.1	140.00	-12.54	2 9	584 604	7
mmu-miR-8117	XM_006538456.3	143.00	-28.89	2 20	661 681	18
mmu-miR-8117	XM_006537815.3	150.00	-19.88	2 16	160 181	15
mmu-miR-8117	XM_017317946.1	145.00	-26.10	2 20	2241 2265	22
mmu-miR-8117	NM_011169.5	150.00	-22.41	2 20	518 540	20

mmu-miR-8117	XM_011240476.2	161.00	-28.77	2 20	2607 2630	21
mmu-miR-8117	XM_006505015.3	141.00	-27.69	2 20	1349 1368	18
mmu-miR-8117	XM_017320735.1	145.00	-26.18	3 20	41 60	17
mmu-miR-8117	XM_011240779.2	150.00	-21.39	2 15	5075 5095	13
mmu-miR-8117	XM_006525075.3	151.00	-22.72	2 12	405 425	10
mmu-miR-8117	NM_009127.4	142.00	-19.94	3 20	324 345	18
mmu-miR-8117	NM_018732.3	151.00	-18.51	2 19	1354 1372	17
mmu-miR-8117	NM_018732.3	146.00	-20.09	2 15	4241 4261	13
mmu-miR-8117	XM_017316897.1	148.00	-20.36	2 17	363 383	15
mmu-miR-8117	XM_006518339.3	143.00	-19.24	2 20	2860 2880	18
mmu-miR-8117	NM_001033167.3	142.00	-23.43	2 20	3092 3113	19
mmu-miR-8117	NM_001290993.1	140.00	-14.61	2 9	4171 4191	7
mmu-miR-8117	NM_031183.2	147.00	-21.88	2 16	190 210	14
mmu-miR-8117	XM_006539074.3	148.00	-24.57	2 17	8772 8792	15
mmu-miR-8117	XM_006539074.3	142.00	-17.40	2 20	747 768	19
mmu-miR-8117	NM_019866.1	142.00	-23.37	2 20	2381 2402	19
mmu-miR-8117	NM_001304266.1	143.00	-18.98	2 19	1602 1620	17
mmu-miR-8117	XM_006506145.1	142.00	-20.10	2 16	1546 1567	15
mmu-miR-8117	XM_006506145.1	141.00	-25.96	2 19	2189 2211	19
mmu-miR-8117	NM_011374.2	152.00	-20.92	2 17	2409 2429	15
mmu-miR-8117	XM_017316056.1	140.00	-19.90	2 9	620 640	7
mmu-miR-8117	NM_011581.3	157.00	-28.91	2 20	5343 5365	20
mmu-miR-8117	NM_011581.3	143.00	-24.08	2 16	3628 3648	14
mmu-miR-8117	NM_009381.3	150.00	-16.33	2 17	516 535	15
mmu-miR-8117	XM_006529382.3	140.00	-13.80	2 9	446 466	7
mmu-miR-8117	NM_001033149.3	145.00	-20.56	2 10	311 331	8
mmu-miR-8117	XM_011248394.1	146.00	-19.22	2 17	2416 2438	17
mmu-miR-8117	NM_199304.1	154.00	-33.00	2 20	1951 1972	19
mmu-miR-8117	XM_011243586.2	153.00	-23.62	2 20	320 339	18
mmu-miR-8117	NM_001110508.1	141.00	-27.81	2 20	2579 2598	18
mmu-miR-8117	XM_006519134.3	143.00	-31.87	2 20	2067 2087	18

Table S8 MRE analysis of circRNA and miRNA

miRNA	circRNA	score	energy	miRNA_location	circRNA_location	MRE_length
mmu-miR-206-3p	circRNA_0020|Chr1:10315205_10321709_+	143.00	-19.03	2 21	234 256	20
mmu-miR-206-3p	circRNA_0961|Chr11:67192403_67254873_+	156.00	-21.85	2 21	27619 27640	19
mmu-miR-206-3p	circRNA_0961|Chr11:67192403_67254873_+	150.00	-28.53	2 21	41302 41327	23
mmu-miR-206-3p	circRNA_0961|Chr11:67192403_67254873_+	149.00	-13.94	2 15	19277 19299	14
mmu-miR-206-3p	circRNA_0961|Chr11:67192403_67254873_+	147.00	-14.77	2 16	43300 43321	14
mmu-miR-206-3p	circRNA_0961|Chr11:67192403_67254873_+	144.00	-14.86	2 21	8823 8844	19
mmu-miR-206-3p	circRNA_0961|Chr11:67192403_67254873_+	144.00	-11.43	2 21	19350 19371	19
mmu-miR-206-3p	circRNA_0961|Chr11:67192403_67254873_+	144.00	-16.01	2 18	39230 39254	19
mmu-miR-206-3p	circRNA_0961|Chr11:67192403_67254873_+	140.00	-21.55	2 20	19520 19539	18
mmu-miR-206-3p	circRNA_0961|Chr11:67192403_67254873_+	140.00	-19.01	2 21	21906 21927	19
mmu-miR-206-3p	circRNA_0961|Chr11:67192403_67254873_+	140.00	-12.85	2 9	42411 42432	7
mmu-miR-206-3p	circRNA_1042|Chr11:80385854_80403408_+	149.00	-18.41	2 20	1007 1027	18
mmu-miR-206-3p	circRNA_1042|Chr11:80385854_80403408_+	141.00	-12.57	2 14	4310 4331	12
mmu-miR-206-3p	circRNA_1980|Chr14:50951404_50963869_+	147.00	-18.13	2 21	5478 5504	24
mmu-miR-206-3p	circRNA_3643|Chr2:122441559_122486090_+	147.00	-17.77	2 21	8072 8094	20
mmu-miR-206-3p	circRNA_3643|Chr2:122441559_122486090_+	147.00	-19.88	2 17	23282 23306	18
mmu-miR-206-3p	circRNA_3643|Chr2:122441559_122486090_+	143.00	-20.97	2 20	20469 20490	18
mmu-miR-206-3p	circRNA_3643|Chr2:122441559_122486090_+	141.00	-16.78	3 19	16300 16324	19
mmu-miR-206-3p	circRNA_3643|Chr2:122441559_122486090_+	141.00	-19.73	2 20	19766 19786	18
mmu-miR-206-3p	circRNA_3643|Chr2:122441559_122486090_+	141.00	-11.46	2 21	27111 27130	19
mmu-miR-206-3p	circRNA_5074|Chr6:40685277_40747150_+	159.00	-24.66	2 21	943 961	19
mmu-miR-206-3p	circRNA_5074|Chr6:40685277_40747150_+	159.00	-24.66	2 21	3628 3646	19
mmu-miR-206-3p	circRNA_5074|Chr6:40685277_40747150_+	152.00	-14.47	2 18	4976 5000	19
mmu-miR-206-3p	circRNA_6390|Chr9:121727316_121731565_+	154.00	-16.87	2 21	116 136	19
mmu-miR-214-3p	circRNA_0020|Chr1:10315205_10321709_+	149.00	-18.82	2 19	95 118	19
mmu-miR-214-3p	circRNA_0020|Chr1:10315205_10321709_+	141.00	-19.56	3 20	2873 2893	17
mmu-miR-214-3p	circRNA_0961|Chr11:67192403_67254873_+	169.00	-27.47	2 20	25385 25408	20
mmu-miR-214-3p	circRNA_0961|Chr11:67192403_67254873_+	169.00	-27.79	2 20	49538 49561	20
mmu-miR-214-3p	circRNA_0961|Chr11:67192403_67254873_+	159.00	-26.21	2 20	41250 41271	18
mmu-miR-214-3p	circRNA_0961|Chr11:67192403_67254873_+	151.00	-18.41	2 18	43917 43937	16
mmu-miR-214-3p	circRNA_0961|Chr11:67192403_67254873_+	145.00	-22.43	3 19	35626 35649	18
mmu-miR-214-3p	circRNA_0961|Chr11:67192403_67254873_+	145.00	-23.36	2 19	41280 41302	18
mmu-miR-214-3p	circRNA_0961|Chr11:67192403_67254873_+	144.00	-18.40	2 15	11374 11394	13
mmu-miR-214-3p	circRNA_0961|Chr11:67192403_67254873_+	144.00	-14.62	2 13	35390 35411	11
mmu-miR-214-3p	circRNA_0961|Chr11:67192403_67254873_+	140.00	-14.45	2 9	1278 1299	7
mmu-miR-214-3p	circRNA_0961|Chr11:67192403_67254873_+	140.00	-15.47	2 9	12182 12203	7
mmu-miR-214-3p	circRNA_0961|Chr11:67192403_67254873_+	140.00	-14.45	2 9	28462 28483	7
mmu-miR-214-3p	circRNA_0961|Chr11:67192403_67254873_+	140.00	-16.73	2 9	46189 46210	7
mmu-miR-214-3p	circRNA_0962|Chr11:67194348_67220998_+	169.00	-27.47	2 20	9874 9897	20
mmu-miR-214-3p	circRNA_0962|Chr11:67194348_67220998_+	140.00	-15.47	2 9	6516 6537	7
mmu-miR-214-3p	circRNA_0962|Chr11:67194348_67220998_+	140.00	-14.45	2 9	10890 10911	7
mmu-miR-214-3p	circRNA_1632|Chr13:42055400_42055542_+	140.00	-19.48	3 20	37 58	18
mmu-miR-214-3p	circRNA_1980|Chr14:50951404_50963869_+	170.00	-30.58	2 19	1274 1295	17
mmu-miR-214-3p	circRNA_1980|Chr14:50951404_50963869_+	141.00	-18.47	2 20	3498 3518	18
mmu-miR-214-3p	circRNA_1980|Chr14:50951404_50963869_+	140.00	-16.42	2 9	2769 2790	7
mmu-miR-214-3p	circRNA_1980|Chr14:50951404_50963869_+	140.00	-13.62	2 9	4560 4581	7
mmu-miR-214-3p	circRNA_3643|Chr2:122441559_122486090_+	158.00	-21.88	2 20	27315 27338	20
mmu-miR-214-3p	circRNA_3643|Chr2:122441559_122486090_+	154.00	-25.63	2 21	26823 26843	19
mmu-miR-214-3p	circRNA_3643|Chr2:122441559_122486090_+	149.00	-20.64	2 19	19105 19128	19
mmu-miR-214-3p	circRNA_3643|Chr2:122441559_122486090_+	145.00	-20.71	2 19	4283 4306	19
mmu-miR-214-3p	circRNA_3643|Chr2:122441559_122486090_+	142.00	-17.99	3 15	574 595	12
mmu-miR-214-3p	circRNA_3643|Chr2:122441559_122486090_+	141.00	-17.73	2 21	20865 20886	20
mmu-miR-214-3p	circRNA_4487|Chr4:149156607_149161694_-	140.00	-17.37	2 9	228 249	7
mmu-miR-214-3p	circRNA_4987|Chr6:4529068_4531010_+	153.00	-22.50	2 21	73 92	19
mmu-miR-214-3p	circRNA_5074|Chr6:40685277_40747150_+	149.00	-14.48	2 14	1368 1389	12
mmu-miR-214-3p	circRNA_5074|Chr6:40685277_40747150_+	149.00	-14.48	2 14	4053 4074	12
mmu-miR-214-3p	circRNA_5074|Chr6:40685277_40747150_+	148.00	-17.98	3 21	2692 2713	18
mmu-miR-214-3p	circRNA_5311|Chr6:145147636_145149357_+	149.00	-20.56	2 19	211 233	18
mmu-miR-378a-5p	circRNA_0020|Chr1:10315205_10321709_+	153.00	-19.46	2 20	2774 2797	20
mmu-miR-378a-5p	circRNA_0961|Chr11:67192403_67254873_+	158.00	-21.24	2 21	25588 25608	19
mmu-miR-378a-5p	circRNA_0961|Chr11:67192403_67254873_+	155.00	-20.08	2 21	38043 38065	20
mmu-miR-378a-5p	circRNA_0961|Chr11:67192403_67254873_+	155.00	-18.88	2 20	44375 44396	18
mmu-miR-378a-5p	circRNA_0961|Chr11:67192403_67254873_+	154.00	-22.28	2 21	17106 17126	19
mmu-miR-378a-5p	circRNA_0961|Chr11:67192403_67254873_+	147.00	-18.01	2 15	24180 24199	13
mmu-miR-378a-5p	circRNA_0961|Chr11:67192403_67254873_+	147.00	-24.52	2 16	32537 32558	14
mmu-miR-378a-5p	circRNA_0961|Chr11:67192403_67254873_+	143.00	-18.49	2 18	7078 7098	16
mmu-miR-378a-5p	circRNA_0961|Chr11:67192403_67254873_+	142.00	-31.79	2 20	37808 37830	19
mmu-miR-378a-5p	circRNA_0962|Chr11:67194348_67220998_+	143.00	-18.49	2 18	5133 5153	16
mmu-miR-378a-5p	circRNA_1042|Chr11:80385854_80403408_+	151.00	-18.44	2 20	5649 5670	18
mmu-miR-378a-5p	circRNA_2951|Chr18:39120119_39150118_+	156.00	-26.10	2 17	307 328	15
mmu-miR-378a-5p	circRNA_3643|Chr2:122441559_122486090_+	157.00	-26.26	2 21	10413 10432	19
mmu-miR-378a-5p	circRNA_3643|Chr2:122441559_122486090_+	142.00	-17.04	2 21	1702 1722	19

mmu-miR-378a-5p	circRNA_3643|Chr2:122441559_122486090_+	142.00	-21.05	2 16	26604 26626	15
mmu-miR-378a-5p	circRNA_3643|Chr2:122441559_122486090_+	140.00	-12.03	2 9	10240 10261	7
mmu-miR-494-3p	circRNA_0961|Chr11:67192403_67254873_+	155.00	-12.61	2 18	18406 18429	18
mmu-miR-494-3p	circRNA_0961|Chr11:67192403_67254873_+	151.00	-14.69	2 20	20651 20672	18
mmu-miR-494-3p	circRNA_0961|Chr11:67192403_67254873_+	145.00	-7.85	2 10	40881 40902	8
mmu-miR-494-3p	circRNA_0961|Chr11:67192403_67254873_+	142.00	-10.89	2 19	7607 7628	17
mmu-miR-494-3p	circRNA_0961|Chr11:67192403_67254873_+	142.00	-11.81	2 19	12897 12918	17
mmu-miR-494-3p	circRNA_0961|Chr11:67192403_67254873_+	142.00	-13.40	2 17	33362 33382	15
mmu-miR-494-3p	circRNA_0961|Chr11:67192403_67254873_+	140.00	-6.32	2 9	40201 40222	7
mmu-miR-494-3p	circRNA_0962|Chr11:67194348_67220998_+	142.00	-10.89	2 19	5662 5683	17
mmu-miR-494-3p	circRNA_1042|Chr11:80385854_80403408_+	152.00	-23.78	2 21	7447 7468	19
mmu-miR-494-3p	circRNA_1042|Chr11:80385854_80403408_+	149.00	-21.60	2 21	2109 2128	19
mmu-miR-494-3p	circRNA_1042|Chr11:80385854_80403408_+	140.00	-9.18	2 21	2008 2029	19
mmu-miR-494-3p	circRNA_3643|Chr2:122441559_122486090_+	154.00	-14.76	2 21	7593 7618	23
mmu-miR-494-3p	circRNA_3643|Chr2:122441559_122486090_+	152.00	-13.10	2 21	25480 25501	19
mmu-miR-494-3p	circRNA_3643|Chr2:122441559_122486090_+	150.00	-9.44	2 20	26923 26946	20
mmu-miR-494-3p	circRNA_3832|Chr2:169883526_169886459_+	151.00	-13.34	2 16	2423 2444	14
mmu-miR-494-3p	circRNA_3832|Chr2:169883526_169886459_+	148.00	-17.15	2 15	255 275	13
mmu-miR-494-3p	circRNA_3832|Chr2:169883526_169886459_+	143.00	-18.85	2 17	778 802	18
mmu-miR-494-3p	circRNA_6390|Chr9:121727316_121731565_+	145.00	-13.29	2 10	1 19	8
mmu-miR-135a-1-	circRNA_0961|Chr11:67192403_67254873_+	167.00	-26.36	2 21	45166 45189	21
mmu-miR-135a-1-	circRNA_0961|Chr11:67192403_67254873_+	160.00	-26.00	2 18	24864 24887	18
mmu-miR-135a-1-	circRNA_0961|Chr11:67192403_67254873_+	154.00	-21.14	2 21	30322 30342	19
mmu-miR-135a-1-	circRNA_0961|Chr11:67192403_67254873_+	148.00	-17.08	2 20	22314 22333	18
mmu-miR-135a-1-	circRNA_0961|Chr11:67192403_67254873_+	147.00	-14.98	2 20	23978 23999	18
mmu-miR-135a-1-	circRNA_0961|Chr11:67192403_67254873_+	146.00	-21.80	2 21	41068 41088	19
mmu-miR-135a-1-	circRNA_0961|Chr11:67192403_67254873_+	144.00	-19.11	3 17	4668 4689	14
mmu-miR-135a-1-	circRNA_0961|Chr11:67192403_67254873_+	142.00	-17.13	2 19	7453 7474	17
mmu-miR-135a-1-	circRNA_0961|Chr11:67192403_67254873_+	141.00	-18.63	2 20	27443 27463	18
mmu-miR-135a-1-	circRNA_0961|Chr11:67192403_67254873_+	140.00	-14.82	2 9	12791 12812	7
mmu-miR-135a-1-	circRNA_0961|Chr11:67192403_67254873_+	140.00	-23.75	2 17	29864 29885	15
mmu-miR-135a-1-	circRNA_0962|Chr11:67194348_67220998_+	144.00	-19.11	3 17	2723 2744	14
mmu-miR-135a-1-	circRNA_0962|Chr11:67194348_67220998_+	142.00	-17.13	2 19	5508 5529	17
mmu-miR-135a-1-	circRNA_3643|Chr2:122441559_122486090_+	162.00	-23.22	2 19	10988 11009	17
mmu-miR-135a-1-	circRNA_3643|Chr2:122441559_122486090_+	151.00	-26.38	2 21	20686 20708	20
mmu-miR-135a-1-	circRNA_3643|Chr2:122441559_122486090_+	143.00	-21.74	2 20	20243 20264	18
mmu-miR-135a-1-	circRNA_5074|Chr6:40685277_40747150_+	144.00	-21.57	2 19	215 240	21
mmu-miR-1892	circRNA_0020|Chr1:10315205_10321709_+	159.00	-22.01	2 16	2541 2562	14
mmu-miR-1892	circRNA_0020|Chr1:10315205_10321709_+	149.00	-21.76	2 18	6039 6060	16
mmu-miR-1892	circRNA_0020|Chr1:10315205_10321709_+	143.00	-24.03	2 21	3284 3309	23
mmu-miR-1892	circRNA_0157|Chr1:66801049_66802168_-	140.00	-24.41	2 21	236 257	19
mmu-miR-1892	circRNA_0961|Chr11:67192403_67254873_+	157.00	-23.13	2 19	35398 35422	20
mmu-miR-1892	circRNA_0961|Chr11:67192403_67254873_+	157.00	-20.08	2 18	35663 35684	16
mmu-miR-1892	circRNA_0961|Chr11:67192403_67254873_+	152.00	-23.67	2 21	38159 38180	19
mmu-miR-1892	circRNA_0961|Chr11:67192403_67254873_+	151.00	-17.68	2 20	13755 13776	18
mmu-miR-1892	circRNA_0961|Chr11:67192403_67254873_+	150.00	-20.69	2 19	45402 45423	17
mmu-miR-1892	circRNA_0961|Chr11:67192403_67254873_+	148.00	-24.80	2 21	16835 16856	19
mmu-miR-1892	circRNA_0961|Chr11:67192403_67254873_+	148.00	-27.19	3 21	25877 25898	18
mmu-miR-1892	circRNA_0961|Chr11:67192403_67254873_+	147.00	-27.29	2 20	21999 22020	18
mmu-miR-1892	circRNA_0961|Chr11:67192403_67254873_+	144.00	-21.87	2 21	27175 27196	19
mmu-miR-1892	circRNA_0961|Chr11:67192403_67254873_+	144.00	-27.84	3 21	41497 41518	18
mmu-miR-1892	circRNA_0961|Chr11:67192403_67254873_+	140.00	-22.48	2 21	5131 5152	19
mmu-miR-1892	circRNA_0961|Chr11:67192403_67254873_+	140.00	-16.96	2 17	13874 13895	15
mmu-miR-1892	circRNA_0961|Chr11:67192403_67254873_+	140.00	-14.40	2 9	24333 24354	7
mmu-miR-1892	circRNA_0962|Chr11:67194348_67220998_+	140.00	-22.48	2 21	3186 3207	19
mmu-miR-1892	circRNA_0962|Chr11:67194348_67220998_+	140.00	-16.96	2 17	7000 7021	15
mmu-miR-1892	circRNA_1042|Chr11:80385854_80403408_+	155.00	-17.98	2 16	4251 4272	14
mmu-miR-1892	circRNA_1042|Chr11:80385854_80403408_+	144.00	-21.78	3 17	1666 1687	14
mmu-miR-1892	circRNA_1042|Chr11:80385854_80403408_+	140.00	-11.75	2 9	995 1016	7
mmu-miR-1892	circRNA_1980|Chr14:50951404_50963869_+	157.00	-21.29	2 18	5363 5384	16
mmu-miR-1892	circRNA_1980|Chr14:50951404_50963869_+	145.00	-17.22	2 10	4266 4287	8
mmu-miR-1892	circRNA_3607|Chr2:119057281_119064097_+	142.00	-21.18	2 21	111 131	19
mmu-miR-1892	circRNA_3643|Chr2:122441559_122486090_+	159.00	-32.77	3 21	8123 8145	19
mmu-miR-1892	circRNA_3643|Chr2:122441559_122486090_+	152.00	-27.09	2 21	578 599	19
mmu-miR-1892	circRNA_3643|Chr2:122441559_122486090_+	148.00	-20.08	2 21	12051 12072	19
mmu-miR-1892	circRNA_3643|Chr2:122441559_122486090_+	148.00	-22.07	2 19	13693 13713	17
mmu-miR-1892	circRNA_3643|Chr2:122441559_122486090_+	148.00	-23.43	3 21	22201 22222	18
mmu-miR-1892	circRNA_3643|Chr2:122441559_122486090_+	148.00	-24.36	2 21	24607 24628	19
mmu-miR-1892	circRNA_3643|Chr2:122441559_122486090_+	145.00	-22.77	3 18	12724 12745	15
mmu-miR-1892	circRNA_3643|Chr2:122441559_122486090_+	144.00	-25.64	2 21	16559 16580	19
mmu-miR-1892	circRNA_3643|Chr2:122441559_122486090_+	140.00	-13.33	2 9	10972 10993	7
mmu-miR-1892	circRNA_3643|Chr2:122441559_122486090_+	140.00	-28.12	3 21	20708 20729	18
mmu-miR-1892	circRNA_3832|Chr2:169883526_169886459_+	148.00	-18.75	2 13	2340 2361	11
mmu-miR-1892	circRNA_3832|Chr2:169883526_169886459_+	147.00	-18.14	2 21	1252 1274	20
mmu-miR-1892	circRNA_3832|Chr2:169883526_169886459_+	141.00	-14.84	2 16	283 303	14

mmu-miR-5113	circRNA_0020|Chr1:10315205_10321709_+	156.00	-20.64	2 22	3321 3344	21
mmu-miR-5113	circRNA_0020|Chr1:10315205_10321709_+	156.00	-21.72	2 13	5235 5257	11
mmu-miR-5113	circRNA_0020|Chr1:10315205_10321709_+	147.00	-26.22	2 22	6169 6193	22
mmu-miR-5113	circRNA_0611|Chr10:75274017_75279961_+	153.00	-24.35	3 18	180 202	15
mmu-miR-5113	circRNA_0961|Chr11:67192403_67254873_+	168.00	-21.46	2 21	31590 31612	19
mmu-miR-5113	circRNA_0961|Chr11:67192403_67254873_+	166.00	-23.75	2 19	39329 39351	17
mmu-miR-5113	circRNA_0961|Chr11:67192403_67254873_+	161.00	-25.67	2 18	38537 38559	16
mmu-miR-5113	circRNA_0961|Chr11:67192403_67254873_+	155.00	-21.08	2 21	39403 39426	20
mmu-miR-5113	circRNA_0961|Chr11:67192403_67254873_+	153.00	-18.18	2 22	9105 9125	20
mmu-miR-5113	circRNA_0961|Chr11:67192403_67254873_+	153.00	-21.86	3 18	34091 34113	15
mmu-miR-5113	circRNA_0961|Chr11:67192403_67254873_+	152.00	-23.68	2 19	35327 35348	17
mmu-miR-5113	circRNA_0961|Chr11:67192403_67254873_+	149.00	-21.56	3 18	34674 34696	15
mmu-miR-5113	circRNA_0961|Chr11:67192403_67254873_+	148.00	-17.47	2 19	6602 6623	17
mmu-miR-5113	circRNA_0961|Chr11:67192403_67254873_+	148.00	-22.57	3 17	34684 34706	14
mmu-miR-5113	circRNA_0961|Chr11:67192403_67254873_+	148.00	-22.57	3 17	34694 34716	14
mmu-miR-5113	circRNA_0961|Chr11:67192403_67254873_+	147.00	-24.92	3 20	4334 4356	17
mmu-miR-5113	circRNA_0961|Chr11:67192403_67254873_+	147.00	-25.10	2 21	12253 12276	20
mmu-miR-5113	circRNA_0961|Chr11:67192403_67254873_+	147.00	-20.14	2 16	13478 13500	14
mmu-miR-5113	circRNA_0961|Chr11:67192403_67254873_+	146.00	-25.66	2 15	12754 12776	13
mmu-miR-5113	circRNA_0961|Chr11:67192403_67254873_+	146.00	-20.17	2 20	44524 44547	19
mmu-miR-5113	circRNA_0961|Chr11:67192403_67254873_+	145.00	-26.42	4 22	12137 12159	18
mmu-miR-5113	circRNA_0961|Chr11:67192403_67254873_+	144.00	-20.18	2 18	16265 16288	17
mmu-miR-5113	circRNA_0961|Chr11:67192403_67254873_+	144.00	-21.12	3 17	34789 34811	14
mmu-miR-5113	circRNA_0961|Chr11:67192403_67254873_+	143.00	-28.84	2 22	4509 4534	23
mmu-miR-5113	circRNA_0961|Chr11:67192403_67254873_+	143.00	-21.58	3 22	6265 6286	19
mmu-miR-5113	circRNA_0961|Chr11:67192403_67254873_+	143.00	-22.75	2 12	10965 10987	10
mmu-miR-5113	circRNA_0961|Chr11:67192403_67254873_+	143.00	-18.54	2 16	30998 31020	14
mmu-miR-5113	circRNA_0961|Chr11:67192403_67254873_+	142.00	-22.06	3 17	34704 34728	16
mmu-miR-5113	circRNA_0961|Chr11:67192403_67254873_+	141.00	-16.71	2 19	38980 39004	19
mmu-miR-5113	circRNA_0961|Chr11:67192403_67254873_+	141.00	-22.48	2 20	40104 40125	18
mmu-miR-5113	circRNA_0961|Chr11:67192403_67254873_+	140.00	-18.18	2 17	16718 16740	15
mmu-miR-5113	circRNA_0961|Chr11:67192403_67254873_+	140.00	-15.28	2 9	31074 31096	7
mmu-miR-5113	circRNA_0961|Chr11:67192403_67254873_+	140.00	-18.55	3 18	41075 41098	16
mmu-miR-5113	circRNA_0961|Chr11:67192403_67254873_+	140.00	-18.31	3 21	41943 41963	18
mmu-miR-5113	circRNA_0962|Chr11:67194348_67220998_+	148.00	-17.47	2 19	4657 4678	17
mmu-miR-5113	circRNA_0962|Chr11:67194348_67220998_+	147.00	-24.92	3 20	2389 2411	17
mmu-miR-5113	circRNA_0962|Chr11:67194348_67220998_+	143.00	-28.84	2 22	2564 2589	23
mmu-miR-5113	circRNA_0962|Chr11:67194348_67220998_+	143.00	-21.58	3 22	4320 4341	19
mmu-miR-5113	circRNA_0962|Chr11:67194348_67220998_+	140.00	-18.18	2 17	7701 7723	15
mmu-miR-5113	circRNA_1042|Chr11:80385854_80403408_+	161.00	-24.67	2 22	4313 4335	20
mmu-miR-5113	circRNA_1042|Chr11:80385854_80403408_+	140.00	-19.44	2 22	6612 6636	22
mmu-miR-5113	circRNA_1980|Chr14:50951404_50963869_+	160.00	-24.09	2 21	1041 1063	19
mmu-miR-5113	circRNA_1980|Chr14:50951404_50963869_+	153.00	-19.80	2 15	401 424	14
mmu-miR-5113	circRNA_1980|Chr14:50951404_50963869_+	147.00	-17.67	2 21	893 916	20
mmu-miR-5113	circRNA_1980|Chr14:50951404_50963869_+	144.00	-18.17	2 22	3298 3322	22
mmu-miR-5113	circRNA_1980|Chr14:50951404_50963869_+	144.00	-17.92	2 21	3616 3638	19
mmu-miR-5113	circRNA_1980|Chr14:50951404_50963869_+	141.00	-18.91	2 20	5506 5527	18
mmu-miR-5113	circRNA_1980|Chr14:50951404_50963869_+	140.00	-13.85	2 9	2179 2201	7
mmu-miR-5113	circRNA_3003|Chr18:67545615_67587859_-	141.00	-22.91	3 22	101 123	19
mmu-miR-5113	circRNA_3643|Chr2:122441559_122486090_+	158.00	-21.16	2 15	21365 21387	13
mmu-miR-5113	circRNA_3643|Chr2:122441559_122486090_+	157.00	-22.60	2 19	10129 10152	18
mmu-miR-5113	circRNA_3643|Chr2:122441559_122486090_+	155.00	-19.45	2 22	15651 15676	23
mmu-miR-5113	circRNA_3643|Chr2:122441559_122486090_+	154.00	-19.33	2 19	26222 26244	17
mmu-miR-5113	circRNA_3643|Chr2:122441559_122486090_+	152.00	-16.79	2 13	486 508	11
mmu-miR-5113	circRNA_3643|Chr2:122441559_122486090_+	152.00	-26.04	2 22	15177 15200	21
mmu-miR-5113	circRNA_3643|Chr2:122441559_122486090_+	148.00	-21.36	2 22	17936 17961	23
mmu-miR-5113	circRNA_3643|Chr2:122441559_122486090_+	148.00	-19.62	3 19	23692 23713	16
mmu-miR-5113	circRNA_3643|Chr2:122441559_122486090_+	145.00	-18.10	2 22	1067 1089	20
mmu-miR-5113	circRNA_3643|Chr2:122441559_122486090_+	145.00	-23.20	2 20	14475 14496	18
mmu-miR-5113	circRNA_3643|Chr2:122441559_122486090_+	142.00	-17.80	2 11	8231 8253	9
mmu-miR-5113	circRNA_3643|Chr2:122441559_122486090_+	141.00	-15.96	3 22	564 586	19
mmu-miR-5113	circRNA_3643|Chr2:122441559_122486090_+	140.00	-28.02	2 18	9862 9886	18
mmu-miR-5113	circRNA_5074|Chr6:40685277_40747150_+	144.00	-18.84	2 17	948 970	15
mmu-miR-5113	circRNA_5074|Chr6:40685277_40747150_+	144.00	-18.84	2 17	3633 3655	15
mmu-miR-5113	circRNA_5074|Chr6:40685277_40747150_+	140.00	-24.36	2 21	2419 2441	19
mmu-miR-5128	circRNA_0020|Chr1:10315205_10321709_+	148.00	-20.49	2 22	2989 3013	22
mmu-miR-5128	circRNA_0020|Chr1:10315205_10321709_+	145.00	-22.27	2 22	3632 3654	20
mmu-miR-5128	circRNA_0020|Chr1:10315205_10321709_+	140.00	-22.50	2 22	860 883	21
mmu-miR-5128	circRNA_0020|Chr1:10315205_10321709_+	140.00	-14.29	2 9	3263 3285	7
mmu-miR-5128	circRNA_0961|Chr11:67192403_67254873_+	164.00	-23.57	2 22	39100 39125	23
mmu-miR-5128	circRNA_0961|Chr11:67192403_67254873_+	148.00	-20.31	2 22	41496 41519	21
mmu-miR-5128	circRNA_0961|Chr11:67192403_67254873_+	146.00	-16.83	2 21	36780 36797	19
mmu-miR-5128	circRNA_0961|Chr11:67192403_67254873_+	142.00	-14.15	3 19	39535 39557	16
mmu-miR-5128	circRNA_0961|Chr11:67192403_67254873_+	141.00	-15.09	2 22	33759 33781	20
mmu-miR-5128	circRNA_1980|Chr14:50951404_50963869_+	153.00	-15.37	2 14	5557 5579	12

mmu-miR-5128	circRNA_3643	Chr2:122441559_122486090_+	183.00	-34.18	2 22	5416 5437	20
mmu-miR-5128	circRNA_3643	Chr2:122441559_122486090_+	161.00	-27.25	3 22	2985 3007	19
mmu-miR-5128	circRNA_3643	Chr2:122441559_122486090_+	155.00	-15.62	2 20	12724 12746	18
mmu-miR-5128	circRNA_3643	Chr2:122441559_122486090_+	153.00	-19.93	2 20	26935 26956	18
mmu-miR-5128	circRNA_3643	Chr2:122441559_122486090_+	141.00	-15.46	3 22	21292 21314	19
mmu-miR-5128	circRNA_3643	Chr2:122441559_122486090_+	140.00	-18.70	2 22	23306 23330	22
mmu-miR-344i	circRNA_0961	Chr11:67192403_67254873_+	154.00	-21.35	2 19	25543 25562	17
mmu-miR-344i	circRNA_0961	Chr11:67192403_67254873_+	149.00	-21.33	2 18	33881 33900	16
mmu-miR-344i	circRNA_0961	Chr11:67192403_67254873_+	146.00	-21.95	3 19	20646 20665	16
mmu-miR-344i	circRNA_0961	Chr11:67192403_67254873_+	141.00	-18.13	2 18	16811 16830	16
mmu-miR-344i	circRNA_0961	Chr11:67192403_67254873_+	140.00	-14.66	2 9	44787 44806	7
mmu-miR-344i	circRNA_1042	Chr11:80385854_80403408_+	148.00	-21.16	2 18	2425 2445	17
mmu-miR-344i	circRNA_1980	Chr14:50951404_50963869_+	156.00	-22.65	2 19	4738 4756	17
mmu-miR-344i	circRNA_3832	Chr2:169883526_169886459_+	143.00	-21.55	2 16	1727 1746	14
mmu-miR-344i	circRNA_3832	Chr2:169883526_169886459_+	140.00	-16.69	2 9	948 967	7
mmu-miR-6238	circRNA_0961	Chr11:67192403_67254873_+	147.00	-9.48	2 20	4304 4325	18
mmu-miR-6238	circRNA_0961	Chr11:67192403_67254873_+	145.00	-12.19	2 14	41821 41842	12
mmu-miR-6238	circRNA_0961	Chr11:67192403_67254873_+	141.00	-11.44	2 21	44020 44041	20
mmu-miR-6238	circRNA_0962	Chr11:67194348_67220998_+	147.00	-9.48	2 20	2359 2380	18
mmu-miR-6238	circRNA_3425	Chr2:37624173_37627526_-	146.00	-15.71	2 20	482 505	20
mmu-miR-6238	circRNA_3643	Chr2:122441559_122486090_+	145.00	-19.23	2 14	4894 4915	12
mmu-miR-6238	circRNA_3643	Chr2:122441559_122486090_+	145.00	-14.62	2 18	7509 7530	16
mmu-miR-6238	circRNA_3643	Chr2:122441559_122486090_+	141.00	-18.47	2 20	16023 16046	20
mmu-miR-6238	circRNA_3643	Chr2:122441559_122486090_+	141.00	-19.89	2 20	19779 19799	18
mmu-miR-6238	circRNA_3643	Chr2:122441559_122486090_+	140.00	-7.49	2 9	15491 15512	7
mmu-miR-6238	circRNA_4728	Chr5:97027831_97045167_+	145.00	-11.49	2 19	413 436	19
mmu-miR-6238	circRNA_6109	Chr9:57056714_57057861_+	154.00	-21.75	3 20	150 172	18
mmu-miR-6351	circRNA_0961	Chr11:67192403_67254873_+	147.00	-19.61	2 12	121 143	10
mmu-miR-6351	circRNA_0961	Chr11:67192403_67254873_+	142.00	-20.11	2 22	35451 35471	20
mmu-miR-6351	circRNA_3643	Chr2:122441559_122486090_+	144.00	-16.08	2 22	1316 1340	22
mmu-miR-6351	circRNA_4055	Chr3:126796838_126798280_+	144.00	-20.89	2 15	110 131	13
mmu-miR-6351	circRNA_5074	Chr6:40685277_40747150_+	141.00	-18.08	2 22	2108 2130	20
mmu-miR-6351	circRNA_5074	Chr6:40685277_40747150_+	141.00	-18.08	2 22	4790 4812	20
mmu-miR-6378	circRNA_0020	Chr1:10315205_10321709_+	144.00	-29.29	2 21	2660 2682	20
mmu-miR-6378	circRNA_0376	Chr1:172173943_172187460_+	149.00	-17.01	2 20	275 299	21
mmu-miR-6378	circRNA_0961	Chr11:67192403_67254873_+	153.00	-20.28	2 14	5562 5583	12
mmu-miR-6378	circRNA_0961	Chr11:67192403_67254873_+	147.00	-22.00	2 16	35396 35417	14
mmu-miR-6378	circRNA_0961	Chr11:67192403_67254873_+	146.00	-24.77	2 19	29735 29756	17
mmu-miR-6378	circRNA_0961	Chr11:67192403_67254873_+	141.00	-17.38	2 10	1147 1168	8
mmu-miR-6378	circRNA_0961	Chr11:67192403_67254873_+	141.00	-22.21	2 20	40645 40665	18
mmu-miR-6378	circRNA_0962	Chr11:67194348_67220998_+	153.00	-20.28	2 14	3617 3638	12
mmu-miR-6378	circRNA_1042	Chr11:80385854_80403408_+	145.00	-14.15	2 16	4413 4433	14
mmu-miR-6378	circRNA_3643	Chr2:122441559_122486090_+	144.00	-16.45	3 21	6973 6994	18
mmu-miR-6378	circRNA_4728	Chr5:97027831_97045167_+	142.00	-19.11	2 11	223 244	9
mmu-miR-6378	circRNA_5811	Chr8:71992832_71998295_-	143.00	-21.51	2 20	188 209	18
mmu-miR-3473e	circRNA_0020	Chr1:10315205_10321709_+	141.00	-24.36	2 19	1822 1843	18
mmu-miR-3473e	circRNA_0537	Chr10:43393871_43395692_+	150.00	-20.42	2 17	176 195	15
mmu-miR-3473e	circRNA_0961	Chr11:67192403_67254873_+	152.00	-25.66	2 19	13115 13134	17
mmu-miR-3473e	circRNA_0961	Chr11:67192403_67254873_+	148.00	-25.43	2 19	46340 46359	17
mmu-miR-3473e	circRNA_0961	Chr11:67192403_67254873_+	147.00	-21.83	2 16	44904 44924	14
mmu-miR-3473e	circRNA_0961	Chr11:67192403_67254873_+	145.00	-24.55	2 16	15803 15822	14
mmu-miR-3473e	circRNA_0961	Chr11:67192403_67254873_+	142.00	-27.54	2 19	22640 22657	17
mmu-miR-3473e	circRNA_0961	Chr11:67192403_67254873_+	142.00	-27.54	2 19	48992 49009	17
mmu-miR-3473e	circRNA_0962	Chr11:67194348_67220998_+	152.00	-25.66	2 19	6667 6686	17
mmu-miR-3473e	circRNA_0962	Chr11:67194348_67220998_+	142.00	-27.54	2 19	9328 9345	17
mmu-miR-3473e	circRNA_1042	Chr11:80385854_80403408_+	140.00	-23.04	3 19	7001 7023	18
mmu-miR-3473e	circRNA_1980	Chr14:50951404_50963869_+	147.00	-19.30	2 18	1538 1557	16
mmu-miR-3473e	circRNA_1980	Chr14:50951404_50963869_+	141.00	-20.11	3 19	849 870	17
mmu-miR-3473e	circRNA_2313	Chr15:93452117_93465245_+	142.00	-20.60	2 19	60 80	17
mmu-miR-3473e	circRNA_3643	Chr2:122441559_122486090_+	159.00	-38.02	2 20	5412 5432	18
mmu-miR-3473e	circRNA_3643	Chr2:122441559_122486090_+	155.00	-26.73	3 20	19398 19418	17
mmu-miR-3473e	circRNA_3643	Chr2:122441559_122486090_+	154.00	-18.80	2 20	1926 1947	19
mmu-miR-3473e	circRNA_3643	Chr2:122441559_122486090_+	151.00	-32.21	2 20	17985 18005	18
mmu-miR-3473e	circRNA_3643	Chr2:122441559_122486090_+	150.00	-19.73	2 19	21562 21582	17
mmu-miR-3473e	circRNA_3643	Chr2:122441559_122486090_+	149.00	-23.85	3 18	3280 3300	15
mmu-miR-3473e	circRNA_3643	Chr2:122441559_122486090_+	145.00	-20.67	2 16	27496 27515	14
mmu-miR-3473e	circRNA_3643	Chr2:122441559_122486090_+	143.00	-34.11	2 20	11157 11177	18
mmu-miR-3473e	circRNA_3832	Chr2:169883526_169886459_+	151.00	-22.77	2 16	2126 2146	14
mmu-miR-3473e	circRNA_4635	Chr5:43758222_43773659_-	151.00	-21.49	2 12	1520 1540	10
mmu-miR-3473e	circRNA_4635	Chr5:43758222_43773659_-	145.00	-22.73	2 20	35 54	18
mmu-miR-3473e	circRNA_4926	Chr5:143080224_143081077_-	147.00	-20.91	2 20	185 205	18
mmu-miR-3473e	circRNA_5074	Chr6:40685277_40747150_+	147.00	-20.86	2 16	301 321	14
mmu-miR-3473e	circRNA_5074	Chr6:40685277_40747150_+	147.00	-20.86	2 16	2986 3006	14
mmu-miR-3473e	circRNA_5074	Chr6:40685277_40747150_+	146.00	-22.60	2 17	1 17	15
mmu-miR-3473e	circRNA_5074	Chr6:40685277_40747150_+	146.00	-18.17	2 19	2682 2702	17

mmu-miR-3473e	circRNA_5074|Chr6:40685277_40747150_+	141.00	-18.30	2 16	355 378	17
mmu-miR-3473e	circRNA_5074|Chr6:40685277_40747150_+	141.00	-18.30	2 16	3040 3063	17
mmu-miR-3473e	circRNA_6446|ChrX:42217416_42250037_+	141.00	-18.94	2 14	927 947	12
mmu-miR-7005-5p	circRNA_0020|Chr1:10315205_10321709_+	145.00	-27.78	3 21	2173 2192	18
mmu-miR-7005-5p	circRNA_0020|Chr1:10315205_10321709_+	140.00	-21.80	2 17	1028 1049	15
mmu-miR-7005-5p	circRNA_0020|Chr1:10315205_10321709_+	140.00	-32.61	2 17	5141 5162	15
mmu-miR-7005-5p	circRNA_0961|Chr11:67192403_67254873_+	169.00	-27.12	2 18	38213 38234	16
mmu-miR-7005-5p	circRNA_0961|Chr11:67192403_67254873_+	151.00	-26.14	2 17	34586 34609	17
mmu-miR-7005-5p	circRNA_0961|Chr11:67192403_67254873_+	148.00	-29.56	2 19	3969 3989	17
mmu-miR-7005-5p	circRNA_0961|Chr11:67192403_67254873_+	145.00	-17.33	2 10	2980 3001	8
mmu-miR-7005-5p	circRNA_0961|Chr11:67192403_67254873_+	145.00	-17.31	2 10	3836 3857	8
mmu-miR-7005-5p	circRNA_0961|Chr11:67192403_67254873_+	144.00	-20.62	2 21	21580 21601	19
mmu-miR-7005-5p	circRNA_0961|Chr11:67192403_67254873_+	142.00	-17.41	2 11	18214 18235	9
mmu-miR-7005-5p	circRNA_0961|Chr11:67192403_67254873_+	142.00	-22.29	2 20	27707 27730	20
mmu-miR-7005-5p	circRNA_0961|Chr11:67192403_67254873_+	141.00	-25.24	2 21	25095 25117	21
mmu-miR-7005-5p	circRNA_0961|Chr11:67192403_67254873_+	141.00	-25.11	2 21	36267 36286	19
mmu-miR-7005-5p	circRNA_0961|Chr11:67192403_67254873_+	140.00	-28.31	4 21	15791 15812	17
mmu-miR-7005-5p	circRNA_0961|Chr11:67192403_67254873_+	140.00	-15.45	2 9	47643 47664	7
mmu-miR-7005-5p	circRNA_0962|Chr11:67194348_67220998_+	148.00	-29.56	2 19	2024 2044	17
mmu-miR-7005-5p	circRNA_0962|Chr11:67194348_67220998_+	145.00	-17.33	2 10	1035 1056	8
mmu-miR-7005-5p	circRNA_0962|Chr11:67194348_67220998_+	145.00	-17.31	2 10	1891 1912	8
mmu-miR-7005-5p	circRNA_0962|Chr11:67194348_67220998_+	142.00	-17.41	2 11	7979 8000	9
mmu-miR-7005-5p	circRNA_0962|Chr11:67194348_67220998_+	141.00	-25.24	2 21	9754 9776	21
mmu-miR-7005-5p	circRNA_1042|Chr11:80385854_80403408_+	160.00	-25.20	2 21	806 827	19
mmu-miR-7005-5p	circRNA_1042|Chr11:80385854_80403408_+	159.00	-26.01	2 21	5204 5226	20
mmu-miR-7005-5p	circRNA_1042|Chr11:80385854_80403408_+	157.00	-24.78	2 21	7599 7618	19
mmu-miR-7005-5p	circRNA_1042|Chr11:80385854_80403408_+	148.00	-26.20	3 21	1665 1686	18
mmu-miR-7005-5p	circRNA_1399|Chr12:72783683_72786789_+	146.00	-20.36	2 15	652 673	13
mmu-miR-7005-5p	circRNA_1980|Chr14:50951404_50963869_+	147.00	-22.59	2 20	3155 3176	18
mmu-miR-7005-5p	circRNA_1980|Chr14:50951404_50963869_+	141.00	-16.65	2 18	847 868	16
mmu-miR-7005-5p	circRNA_3643|Chr2:122441559_122486090_+	144.00	-20.74	3 17	12723 12744	14
mmu-miR-7005-5p	circRNA_3643|Chr2:122441559_122486090_+	143.00	-21.99	2 17	1767 1789	16
mmu-miR-7005-5p	circRNA_3643|Chr2:122441559_122486090_+	140.00	-19.82	2 21	3308 3329	19
mmu-miR-7005-5p	circRNA_3832|Chr2:169883526_169886459_+	144.00	-18.81	2 17	984 1005	15
mmu-miR-7005-5p	circRNA_3954|Chr3:88346445_88349444_+	148.00	-18.47	2 21	66 87	19
mmu-miR-7005-5p	circRNA_5074|Chr6:40685277_40747150_+	151.00	-23.83	2 21	2297 2321	22
mmu-miR-7005-5p	circRNA_5639|Chr7:132771578_132779385_-	143.00	-20.74	2 21	7 29	20
mmu-miR-7009-5p	circRNA_0020|Chr1:10315205_10321709_+	140.00	-26.01	2 21	3102 3123	19
mmu-miR-7009-5p	circRNA_0157|Chr1:66801049_66802168_-	153.00	-25.29	2 15	17 39	14
mmu-miR-7009-5p	circRNA_0961|Chr11:67192403_67254873_+	153.00	-20.50	2 20	10483 10503	18
mmu-miR-7009-5p	circRNA_0961|Chr11:67192403_67254873_+	152.00	-20.03	2 17	15688 15709	15
mmu-miR-7009-5p	circRNA_0961|Chr11:67192403_67254873_+	151.00	-21.19	2 21	35660 35682	20
mmu-miR-7009-5p	circRNA_0961|Chr11:67192403_67254873_+	141.00	-22.60	2 14	33095 33116	12
mmu-miR-7009-5p	circRNA_0961|Chr11:67192403_67254873_+	140.00	-19.35	2 17	31843 31864	15
mmu-miR-7009-5p	circRNA_0961|Chr11:67192403_67254873_+	140.00	-22.72	2 17	34635 34656	15
mmu-miR-7009-5p	circRNA_1980|Chr14:50951404_50963869_+	141.00	-23.46	2 14	4024 4045	12
mmu-miR-7009-5p	circRNA_3643|Chr2:122441559_122486090_+	154.00	-29.30	2 21	11678 11698	19
mmu-miR-7009-5p	circRNA_3643|Chr2:122441559_122486090_+	148.00	-22.34	2 13	26514 26535	11
mmu-miR-7009-5p	circRNA_3643|Chr2:122441559_122486090_+	143.00	-31.24	3 21	15660 15682	19
mmu-miR-7009-5p	circRNA_3643|Chr2:122441559_122486090_+	142.00	-21.18	2 21	18241 18264	21
mmu-miR-7009-5p	circRNA_3832|Chr2:169883526_169886459_+	164.00	-32.85	2 21	949 970	19
mmu-miR-7009-5p	circRNA_3832|Chr2:169883526_169886459_+	152.00	-22.28	2 14	1422 1444	13
mmu-miR-7009-5p	circRNA_3832|Chr2:169883526_169886459_+	145.00	-20.64	2 21	1253 1272	19
mmu-miR-7009-5p	circRNA_5367|Chr7:28990988_28991325_+	141.00	-19.56	2 18	29 50	16
mmu-miR-7009-5p	circRNA_5639|Chr7:132771578_132779385_-	141.00	-19.95	2 19	52 74	18
mmu-miR-7042-5p	circRNA_0020|Chr1:10315205_10321709_+	144.00	-18.07	2 13	1819 1839	11
mmu-miR-7042-5p	circRNA_0913|Chr11:54005394_54014456_-	148.00	-16.43	2 18	58 79	17
mmu-miR-7042-5p	circRNA_0961|Chr11:67192403_67254873_+	161.00	-22.82	2 20	41073 41092	18
mmu-miR-7042-5p	circRNA_0961|Chr11:67192403_67254873_+	159.00	-24.55	2 18	32576 32598	18
mmu-miR-7042-5p	circRNA_0961|Chr11:67192403_67254873_+	154.00	-16.34	2 19	43948 43968	17
mmu-miR-7042-5p	circRNA_0961|Chr11:67192403_67254873_+	153.00	-18.71	2 16	14052 14071	14
mmu-miR-7042-5p	circRNA_0961|Chr11:67192403_67254873_+	152.00	-17.17	2 14	34042 34063	13
mmu-miR-7042-5p	circRNA_0961|Chr11:67192403_67254873_+	149.00	-21.77	2 20	14446 14465	18
mmu-miR-7042-5p	circRNA_0961|Chr11:67192403_67254873_+	148.00	-17.47	2 17	34894 34914	15
mmu-miR-7042-5p	circRNA_0961|Chr11:67192403_67254873_+	148.00	-17.92	2 19	41376 41395	17
mmu-miR-7042-5p	circRNA_0961|Chr11:67192403_67254873_+	147.00	-15.44	2 19	20336 20354	17
mmu-miR-7042-5p	circRNA_0961|Chr11:67192403_67254873_+	146.00	-16.84	2 13	41860 41879	11
mmu-miR-7042-5p	circRNA_0961|Chr11:67192403_67254873_+	145.00	-17.20	2 10	38061 38081	8
mmu-miR-7042-5p	circRNA_0961|Chr11:67192403_67254873_+	144.00	-15.07	2 13	31036 31056	11
mmu-miR-7042-5p	circRNA_0961|Chr11:67192403_67254873_+	144.00	-18.01	2 19	34079 34098	17
mmu-miR-7042-5p	circRNA_0961|Chr11:67192403_67254873_+	142.00	-18.11	2 16	38228 38249	15
mmu-miR-7042-5p	circRNA_0961|Chr11:67192403_67254873_+	142.00	-21.91	2 20	39931 39953	20
mmu-miR-7042-5p	circRNA_0961|Chr11:67192403_67254873_+	142.00	-18.13	2 19	41029 41049	17
mmu-miR-7042-5p	circRNA_0961|Chr11:67192403_67254873_+	140.00	-18.86	3 17	13485 13505	14
mmu-miR-7042-5p	circRNA_0961|Chr11:67192403_67254873_+	140.00	-11.22	2 9	27395 27415	7

mmu-miR-7042-5p	circRNA_0961|Chr11:67192403_67254873_+	140.00	-9.64	2 9	32684 32704	7
mmu-miR-7042-5p	circRNA_1042|Chr11:80385854_80403408_+	141.00	-21.34	2 20	7447 7469	20
mmu-miR-7042-5p	circRNA_1980|Chr14:50951404_50963869_+	141.00	-18.19	3 19	1720 1741	17
mmu-miR-7042-5p	circRNA_1980|Chr14:50951404_50963869_+	140.00	-13.34	2 17	1920 1940	15
mmu-miR-7042-5p	circRNA_3643|Chr2:122441559_122486090_+	163.00	-19.17	2 20	3274 3294	18
mmu-miR-7042-5p	circRNA_3643|Chr2:122441559_122486090_+	158.00	-28.32	2 20	16221 16242	19
mmu-miR-7042-5p	circRNA_3643|Chr2:122441559_122486090_+	158.00	-19.40	2 19	19593 19613	17
mmu-miR-7042-5p	circRNA_3643|Chr2:122441559_122486090_+	145.00	-19.19	2 10	25152 25172	8
mmu-miR-7042-5p	circRNA_3643|Chr2:122441559_122486090_+	144.00	-12.92	2 13	5444 5464	11
mmu-miR-7042-5p	circRNA_3643|Chr2:122441559_122486090_+	141.00	-23.92	2 18	15188 15208	16
mmu-miR-7042-5p	circRNA_3643|Chr2:122441559_122486090_+	141.00	-16.29	2 20	19562 19581	18
mmu-miR-7042-5p	circRNA_3643|Chr2:122441559_122486090_+	140.00	-16.93	2 19	16785 16804	17
mmu-miR-7042-5p	circRNA_3832|Chr2:169883526_169886459_+	157.00	-22.57	2 19	503 525	19
mmu-miR-7042-5p	circRNA_6140|Chr9:61935380_61937535_-	141.00	-13.94	2 10	434 454	8
mmu-miR-7219-5p	circRNA_0961|Chr11:67192403_67254873_+	170.00	-27.64	2 21	44196 44217	19
mmu-miR-7219-5p	circRNA_0961|Chr11:67192403_67254873_+	156.00	-18.67	2 15	21330 21351	13
mmu-miR-7219-5p	circRNA_0961|Chr11:67192403_67254873_+	154.00	-17.35	2 21	14449 14470	19
mmu-miR-7219-5p	circRNA_0961|Chr11:67192403_67254873_+	153.00	-26.18	2 21	48393 48413	19
mmu-miR-7219-5p	circRNA_0961|Chr11:67192403_67254873_+	149.00	-22.15	2 22	3133 3155	20
mmu-miR-7219-5p	circRNA_0961|Chr11:67192403_67254873_+	149.00	-21.69	2 21	20989 21009	19
mmu-miR-7219-5p	circRNA_0961|Chr11:67192403_67254873_+	144.00	-19.17	2 21	13024 13046	19
mmu-miR-7219-5p	circRNA_0961|Chr11:67192403_67254873_+	144.00	-12.02	2 22	25002 25025	21
mmu-miR-7219-5p	circRNA_0961|Chr11:67192403_67254873_+	144.00	-15.78	2 21	27959 27980	20
mmu-miR-7219-5p	circRNA_0961|Chr11:67192403_67254873_+	143.00	-16.04	2 22	8793 8814	20
mmu-miR-7219-5p	circRNA_0961|Chr11:67192403_67254873_+	142.00	-18.09	2 22	12325 12347	21
mmu-miR-7219-5p	circRNA_0961|Chr11:67192403_67254873_+	142.00	-15.96	2 11	32826 32848	9
mmu-miR-7219-5p	circRNA_0962|Chr11:67194348_67220998_+	149.00	-22.15	2 22	1188 1210	20
mmu-miR-7219-5p	circRNA_0962|Chr11:67194348_67220998_+	149.00	-21.69	2 21	8729 8749	19
mmu-miR-7219-5p	circRNA_1042|Chr11:80385854_80403408_+	144.00	-17.62	2 17	6645 6667	15
mmu-miR-7219-5p	circRNA_1042|Chr11:80385854_80403408_+	143.00	-20.50	2 20	901 923	18
mmu-miR-7219-5p	circRNA_1980|Chr14:50951404_50963869_+	155.00	-19.47	2 19	1822 1842	17
mmu-miR-7219-5p	circRNA_1980|Chr14:50951404_50963869_+	143.00	-16.53	2 22	2306 2327	20
mmu-miR-7219-5p	circRNA_3643|Chr2:122441559_122486090_+	157.00	-22.28	2 22	3779 3801	20
mmu-miR-7219-5p	circRNA_3643|Chr2:122441559_122486090_+	149.00	-16.57	2 22	16025 16047	20
mmu-miR-7219-5p	circRNA_3643|Chr2:122441559_122486090_+	145.00	-20.81	2 18	16089 16111	16
mmu-miR-7219-5p	circRNA_6446|ChrX:42217416_42250037_+	140.00	-15.74	3 13	1153 1175	10
mmu-miR-7221-3p	circRNA_0020|Chr1:10315205_10321709_+	151.00	-21.29	2 12	1690 1710	10
mmu-miR-7221-3p	circRNA_0961|Chr11:67192403_67254873_+	159.00	-25.94	2 16	727 747	14
mmu-miR-7221-3p	circRNA_0961|Chr11:67192403_67254873_+	159.00	-27.07	2 18	45263 45282	16
mmu-miR-7221-3p	circRNA_0961|Chr11:67192403_67254873_+	144.00	-14.82	2 13	37226 37246	11
mmu-miR-7221-3p	circRNA_0961|Chr11:67192403_67254873_+	143.00	-14.23	2 20	5960 5980	18
mmu-miR-7221-3p	circRNA_0961|Chr11:67192403_67254873_+	143.00	-15.75	2 13	6674 6695	12
mmu-miR-7221-3p	circRNA_0961|Chr11:67192403_67254873_+	142.00	-13.10	2 11	12062 12082	9
mmu-miR-7221-3p	circRNA_0961|Chr11:67192403_67254873_+	141.00	-26.33	2 20	8004 8023	18
mmu-miR-7221-3p	circRNA_0961|Chr11:67192403_67254873_+	141.00	-16.93	2 18	15197 15217	16
mmu-miR-7221-3p	circRNA_0962|Chr11:67194348_67220998_+	143.00	-14.23	2 20	4015 4035	18
mmu-miR-7221-3p	circRNA_0962|Chr11:67194348_67220998_+	143.00	-15.75	2 13	4729 4750	12
mmu-miR-7221-3p	circRNA_0962|Chr11:67194348_67220998_+	141.00	-22.56	2 20	5774 5793	18
mmu-miR-7221-3p	circRNA_1042|Chr11:80385854_80403408_+	148.00	-22.60	2 18	1019 1043	20
mmu-miR-7221-3p	circRNA_1980|Chr14:50951404_50963869_+	150.00	-21.03	2 15	1828 1848	13
mmu-miR-7221-3p	circRNA_1980|Chr14:50951404_50963869_+	141.00	-21.31	3 18	5372 5392	15
mmu-miR-7221-3p	circRNA_1980|Chr14:50951404_50963869_+	140.00	-12.78	2 9	1 15	7
mmu-miR-7221-3p	circRNA_3643|Chr2:122441559_122486090_+	153.00	-27.51	3 18	42 62	15
mmu-miR-7221-3p	circRNA_3643|Chr2:122441559_122486090_+	147.00	-25.67	3 20	7196 7216	17
mmu-miR-7221-3p	circRNA_3643|Chr2:122441559_122486090_+	144.00	-24.45	3 17	19002 19022	14
mmu-miR-7221-3p	circRNA_3643|Chr2:122441559_122486090_+	143.00	-20.56	2 20	17485 17505	18
mmu-miR-7221-3p	circRNA_3643|Chr2:122441559_122486090_+	140.00	-20.27	2 17	8529 8549	15
mmu-miR-7221-3p	circRNA_3643|Chr2:122441559_122486090_+	140.00	-19.55	3 18	12852 12874	17
mmu-miR-7221-3p	circRNA_5074|Chr6:40685277_40747150_+	156.00	-25.59	2 19	4950 4969	17
mmu-miR-7226-5p	circRNA_0020|Chr1:10315205_10321709_+	158.00	-21.71	2 24	5133 5162	26
mmu-miR-7226-5p	circRNA_0020|Chr1:10315205_10321709_+	142.00	-20.15	4 25	5409 5433	21
mmu-miR-7226-5p	circRNA_0020|Chr1:10315205_10321709_+	142.00	-15.12	3 23	5689 5714	20
mmu-miR-7226-5p	circRNA_0961|Chr11:67192403_67254873_+	161.00	-26.91	2 22	26825 26850	20
mmu-miR-7226-5p	circRNA_0961|Chr11:67192403_67254873_+	151.00	-22.23	2 21	3065 3092	21
mmu-miR-7226-5p	circRNA_0961|Chr11:67192403_67254873_+	151.00	-17.77	2 24	16587 16611	23
mmu-miR-7226-5p	circRNA_0961|Chr11:67192403_67254873_+	149.00	-18.66	2 15	47241 47267	14
mmu-miR-7226-5p	circRNA_0961|Chr11:67192403_67254873_+	148.00	-23.93	2 23	23636 23660	21
mmu-miR-7226-5p	circRNA_0961|Chr11:67192403_67254873_+	148.00	-25.44	2 21	40553 40578	19
mmu-miR-7226-5p	circRNA_0961|Chr11:67192403_67254873_+	147.00	-21.60	2 24	19371 19396	22
mmu-miR-7226-5p	circRNA_0961|Chr11:67192403_67254873_+	147.00	-24.71	3 25	41061 41088	24
mmu-miR-7226-5p	circRNA_0961|Chr11:67192403_67254873_+	146.00	-24.86	2 19	7296 7321	17
mmu-miR-7226-5p	circRNA_0961|Chr11:67192403_67254873_+	146.00	-24.20	3 25	32802 32830	25
mmu-miR-7226-5p	circRNA_0961|Chr11:67192403_67254873_+	145.00	-23.84	2 22	50 75	20
mmu-miR-7226-5p	circRNA_0961|Chr11:67192403_67254873_+	144.00	-19.80	3 25	12776 12799	22
mmu-miR-7226-5p	circRNA_0961|Chr11:67192403_67254873_+	144.00	-20.93	3 25	47702 47727	22

mmu-miR-7226-5p	circRNA_0961	Chr11:67192403_67254873_+	143.00	-20.83	2 21	14955 14981	20
mmu-miR-7226-5p	circRNA_0961	Chr11:67192403_67254873_+	143.00	-21.10	2 25	15965 15992	25
mmu-miR-7226-5p	circRNA_0961	Chr11:67192403_67254873_+	142.00	-21.37	2 25	315 344	27
mmu-miR-7226-5p	circRNA_0961	Chr11:67192403_67254873_+	141.00	-22.93	2 25	7873 7894	23
mmu-miR-7226-5p	circRNA_0961	Chr11:67192403_67254873_+	141.00	-23.85	3 22	39141 39166	19
mmu-miR-7226-5p	circRNA_0961	Chr11:67192403_67254873_+	140.00	-18.69	3 19	2714 2738	16
mmu-miR-7226-5p	circRNA_0961	Chr11:67192403_67254873_+	140.00	-14.71	3 21	18498 18523	18
mmu-miR-7226-5p	circRNA_0962	Chr11:67194348_67220998_+	161.00	-26.91	2 22	10086 10111	20
mmu-miR-7226-5p	circRNA_0962	Chr11:67194348_67220998_+	151.00	-22.23	2 21	1120 1147	21
mmu-miR-7226-5p	circRNA_0962	Chr11:67194348_67220998_+	151.00	-17.77	2 24	7570 7594	23
mmu-miR-7226-5p	circRNA_0962	Chr11:67194348_67220998_+	146.00	-24.86	2 19	5351 5376	17
mmu-miR-7226-5p	circRNA_0962	Chr11:67194348_67220998_+	140.00	-18.69	3 19	769 793	16
mmu-miR-7226-5p	circRNA_0962	Chr11:67194348_67220998_+	140.00	-14.71	3 21	8038 8063	18
mmu-miR-7226-5p	circRNA_1042	Chr11:80385854_80403408_+	143.00	-20.09	2 23	6529 6555	23
mmu-miR-7226-5p	circRNA_1042	Chr11:80385854_80403408_+	140.00	-19.11	3 22	1005 1031	20
mmu-miR-7226-5p	circRNA_1980	Chr14:50951404_50963869_+	141.00	-24.50	2 20	5604 5628	18
mmu-miR-7226-5p	circRNA_3003	Chr18:67545615_67587859_-	147.00	-21.68	2 21	24 52	22
mmu-miR-7226-5p	circRNA_3425	Chr2:37624173_37627526_-	140.00	-20.32	2 25	470 492	23
mmu-miR-7226-5p	circRNA_3643	Chr2:122441559_122486090_+	165.00	-25.42	2 25	7260 7285	24
mmu-miR-7226-5p	circRNA_3643	Chr2:122441559_122486090_+	157.00	-24.28	2 22	14436 14461	20
mmu-miR-7226-5p	circRNA_3643	Chr2:122441559_122486090_+	154.00	-22.82	2 25	16539 16570	29
mmu-miR-7226-5p	circRNA_3643	Chr2:122441559_122486090_+	152.00	-23.67	2 25	12733 12756	23
mmu-miR-7226-5p	circRNA_3643	Chr2:122441559_122486090_+	151.00	-21.35	2 25	10944 10966	23
mmu-miR-7226-5p	circRNA_3643	Chr2:122441559_122486090_+	151.00	-23.19	2 25	13000 13026	24
mmu-miR-7226-5p	circRNA_3643	Chr2:122441559_122486090_+	151.00	-18.33	2 20	26056 26081	18
mmu-miR-7226-5p	circRNA_3643	Chr2:122441559_122486090_+	148.00	-22.97	2 25	16337 16360	23
mmu-miR-7226-5p	circRNA_3643	Chr2:122441559_122486090_+	148.00	-29.93	2 25	21418 21441	23
mmu-miR-7226-5p	circRNA_3643	Chr2:122441559_122486090_+	147.00	-19.33	2 25	8294 8320	24
mmu-miR-7226-5p	circRNA_3643	Chr2:122441559_122486090_+	147.00	-22.50	3 23	18245 18271	22
mmu-miR-7226-5p	circRNA_3643	Chr2:122441559_122486090_+	146.00	-20.93	3 19	7519 7544	16
mmu-miR-7226-5p	circRNA_3643	Chr2:122441559_122486090_+	145.00	-18.39	2 23	18531 18557	22
mmu-miR-7226-5p	circRNA_3643	Chr2:122441559_122486090_+	143.00	-18.44	2 23	16243 16266	21
mmu-miR-7226-5p	circRNA_3643	Chr2:122441559_122486090_+	143.00	-21.11	3 21	20111 20137	19
mmu-miR-7226-5p	circRNA_3643	Chr2:122441559_122486090_+	143.00	-15.59	3 24	21636 21661	21
mmu-miR-7226-5p	circRNA_3643	Chr2:122441559_122486090_+	142.00	-20.81	2 25	164 188	23
mmu-miR-7226-5p	circRNA_3643	Chr2:122441559_122486090_+	141.00	-21.69	2 25	3916 3942	25
mmu-miR-7226-5p	circRNA_3643	Chr2:122441559_122486090_+	141.00	-14.05	2 24	26254 26278	22
mmu-miR-7226-5p	circRNA_3643	Chr2:122441559_122486090_+	140.00	-18.54	2 23	282 306	21
mmu-miR-7226-5p	circRNA_3643	Chr2:122441559_122486090_+	140.00	-18.96	2 19	1525 1549	17
mmu-miR-7226-5p	circRNA_3643	Chr2:122441559_122486090_+	140.00	-21.24	2 25	22734 22757	23
mmu-miR-7226-5p	circRNA_3832	Chr2:169883526_169886459_+	142.00	-25.59	2 22	1265 1288	20
mmu-miR-7226-5p	circRNA_3832	Chr2:169883526_169886459_+	140.00	-20.99	3 23	802 826	20
mmu-miR-7226-5p	circRNA_5074	Chr6:40685277_40747150_+	150.00	-24.82	2 25	282 311	27
mmu-miR-7226-5p	circRNA_5074	Chr6:40685277_40747150_+	150.00	-24.82	2 25	2967 2996	27
mmu-miR-7226-5p	circRNA_5074	Chr6:40685277_40747150_+	144.00	-25.90	2 19	5099 5123	17
mmu-miR-7226-5p	circRNA_5074	Chr6:40685277_40747150_+	142.00	-17.93	2 12	2812 2839	12
mmu-miR-7226-5p	circRNA_5639	Chr7:132771578_132779385_-	141.00	-21.37	3 22	222 247	19
mmu-miR-7226-5p	circRNA_5811	Chr8:71992832_71998295_-	156.00	-28.54	2 22	466 493	22
mmu-miR-3473f	circRNA_0020	Chr1:10315205_10321709_+	154.00	-10.25	2 19	3748 3767	17
mmu-miR-3473f	circRNA_0020	Chr1:10315205_10321709_+	147.00	-8.46	2 16	4611 4630	14
mmu-miR-3473f	circRNA_0020	Chr1:10315205_10321709_+	146.00	-11.53	2 19	865 884	17
mmu-miR-3473f	circRNA_0961	Chr11:67192403_67254873_+	162.00	-11.39	2 19	20288 20307	17
mmu-miR-3473f	circRNA_0961	Chr11:67192403_67254873_+	155.00	-14.39	2 18	514 532	16
mmu-miR-3473f	circRNA_0961	Chr11:67192403_67254873_+	152.00	-15.26	2 18	47372 47392	17
mmu-miR-3473f	circRNA_0961	Chr11:67192403_67254873_+	145.00	-14.17	2 14	4263 4282	12
mmu-miR-3473f	circRNA_0961	Chr11:67192403_67254873_+	145.00	-15.95	2 18	8049 8068	16
mmu-miR-3473f	circRNA_0961	Chr11:67192403_67254873_+	145.00	-11.13	2 18	25326 25345	16
mmu-miR-3473f	circRNA_0961	Chr11:67192403_67254873_+	141.00	-7.61	2 11	5682 5702	10
mmu-miR-3473f	circRNA_0961	Chr11:67192403_67254873_+	141.00	-13.67	2 17	14999 15016	15
mmu-miR-3473f	circRNA_0961	Chr11:67192403_67254873_+	140.00	-12.41	2 19	27456 27477	19
mmu-miR-3473f	circRNA_0961	Chr11:67192403_67254873_+	140.00	-14.31	3 17	35572 35591	14
mmu-miR-3473f	circRNA_0962	Chr11:67194348_67220998_+	145.00	-14.17	2 14	2318 2337	12
mmu-miR-3473f	circRNA_0962	Chr11:67194348_67220998_+	141.00	-7.61	2 11	3737 3757	10
mmu-miR-3473f	circRNA_1980	Chr14:50951404_50963869_+	143.00	-15.01	2 18	4216 4234	16
mmu-miR-3473f	circRNA_2888	Chr18:21010677_21020999_+	142.00	-9.69	2 14	254 271	12
mmu-miR-3473f	circRNA_3643	Chr2:122441559_122486090_+	149.00	-24.35	2 19	11164 11185	19
mmu-miR-3473f	circRNA_3643	Chr2:122441559_122486090_+	145.00	-10.58	2 10	23052 23071	8
mmu-miR-3473f	circRNA_3643	Chr2:122441559_122486090_+	142.00	-18.72	2 19	5419 5438	17
mmu-miR-3473f	circRNA_3643	Chr2:122441559_122486090_+	140.00	-12.34	2 18	3030 3051	18
mmu-miR-3473f	circRNA_5811	Chr8:71992832_71998295_-	151.00	-17.15	2 13	22 42	12
mmu-miR-3473f	circRNA_6412	ChrMT:13917_14141_-	148.00	-11.47	2 16	134 151	14
mmu-miR-8094	circRNA_0961	Chr11:67192403_67254873_+	151.00	-15.87	2 18	25363 25382	16
mmu-miR-8094	circRNA_0961	Chr11:67192403_67254873_+	150.00	-18.04	2 11	7810 7830	9
mmu-miR-8094	circRNA_0961	Chr11:67192403_67254873_+	144.00	-16.87	2 18	38332 38353	17
mmu-miR-8094	circRNA_0961	Chr11:67192403_67254873_+	143.00	-16.95	3 20	4427 4447	17

mmu-miR-8094	circRNA_0961|Chr11:67192403_67254873_+	142.00	-14.97	2 15	19805 19825	13
mmu-miR-8094	circRNA_0961|Chr11:67192403_67254873_+	141.00	-18.13	3 19	7883 7904	17
mmu-miR-8094	circRNA_0961|Chr11:67192403_67254873_+	140.00	-10.81	2 9	4216 4236	7
mmu-miR-8094	circRNA_0961|Chr11:67192403_67254873_+	140.00	-17.28	3 20	39417 39437	18
mmu-miR-8094	circRNA_0962|Chr11:67194348_67220998_+	151.00	-15.87	2 18	9852 9871	16
mmu-miR-8094	circRNA_0962|Chr11:67194348_67220998_+	143.00	-16.95	3 20	2482 2502	17
mmu-miR-8094	circRNA_0962|Chr11:67194348_67220998_+	140.00	-10.81	2 9	2271 2291	7
mmu-miR-8094	circRNA_1042|Chr11:80385854_80403408_+	142.00	-9.82	2 11	8172 8192	9
mmu-miR-8094	circRNA_1980|Chr14:50951404_50963869_+	150.00	-20.28	2 20	5176 5197	19
mmu-miR-8094	circRNA_1980|Chr14:50951404_50963869_+	142.00	-13.43	2 19	2399 2419	17
mmu-miR-8094	circRNA_3643|Chr2:122441559_122486090_+	159.00	-19.08	2 19	14566 14589	20
mmu-miR-8094	circRNA_3643|Chr2:122441559_122486090_+	151.00	-16.45	2 16	631 651	14
mmu-miR-8094	circRNA_3643|Chr2:122441559_122486090_+	150.00	-15.31	2 11	24307 24327	9
mmu-miR-8094	circRNA_3643|Chr2:122441559_122486090_+	146.00	-14.52	2 11	18578 18598	9
mmu-miR-8094	circRNA_3643|Chr2:122441559_122486090_+	142.00	-11.31	2 19	784 804	17
mmu-miR-8094	circRNA_3643|Chr2:122441559_122486090_+	142.00	-18.45	3 20	22032 22053	18
mmu-miR-8094	circRNA_3643|Chr2:122441559_122486090_+	140.00	-10.80	2 9	12453 12473	7
mmu-miR-8094	circRNA_4635|Chr5:43758222_43773659_-	143.00	-12.95	3 20	108 128	17
mmu-miR-8094	circRNA_5074|Chr6:40685277_40747150_+	154.00	-18.68	2 20	5114 5136	20
mmu-miR-8094	circRNA_6390|Chr9:121727316_121731565_+	142.00	-13.01	2 20	158 180	20
mmu-miR-8117	circRNA_0961|Chr11:67192403_67254873_+	147.00	-17.39	2 17	2464 2485	16
mmu-miR-8117	circRNA_0962|Chr11:67194348_67220998_+	147.00	-17.39	2 17	519 540	16
mmu-miR-8117	circRNA_1042|Chr11:80385854_80403408_+	158.00	-26.34	2 20	6717 6738	19
mmu-miR-8117	circRNA_3643|Chr2:122441559_122486090_+	155.00	-22.63	2 20	10610 10630	18
mmu-miR-8117	circRNA_4487|Chr4:149156607_149161694_-	141.00	-23.30	2 20	2 21	18
mmu-miR-8117	circRNA_5311|Chr6:145147636_145149357_+	149.00	-20.52	2 19	230 252	19
mmu-miR-8117	circRNA_6446|ChrX:42217416_42250037_+	146.00	-18.67	2 15	1037 1057	13

Table S9 circRNA-associated ceRNA networks involved in osteoporosis

mRNA	genesymbol	circRNA	CeRNA_Score	P value	Shared_miRNA_Nunber	Shared_miRNA
NM_173427.2	Klhdc7a	circRNA_0020|Chr1:10315205_10321709_+	0.8	1.68547E-13	5	mmu-miR-206-3p;mmu-miR-6378;mmu-miR-7226-5p;mmu-miR-7005-5p;mmu-miR-7009-5p
XM_006510077.3	Nnmt	circRNA_0020|Chr1:10315205_10321709_+	0.9	1.68547E-13	5	mmu-miR-206-3p;mmu-miR-7226-5p;mmu-miR-7005-5p;mmu-miR-7042-5p;mmu-miR-7009-5p
XM_006515980.3	A530016L24Rik	circRNA_0020|Chr1:10315205_10321709_+	0.7	5.89656E-13	5	mmu-miR-206-3p;mmu-miR-6378;mmu-miR-7005-5p;mmu-miR-7042-5p;mmu-miR-7009-5p
NM_011169.5	Prlr	circRNA_0020|Chr1:10315205_10321709_+	0.7	5.89656E-13	5	mmu-miR-206-3p;mmu-miR-6378;mmu-miR-7005-5p;mmu-miR-7042-5p;mmu-miR-7009-5p
XM_017312497.1	Plekhg4	circRNA_0020|Chr1:10315205_10321709_+	0.8	5.89656E-13	5	mmu-miR-206-3p;mmu-miR-7226-5p;mmu-miR-7005-5p;mmu-miR-7042-5p;mmu-miR-7009-5p
NM_015814.2	Dkk3	circRNA_0020|Chr1:10315205_10321709_+	0.5	1.57173E-12	5	mmu-miR-206-3p;mmu-miR-6378;mmu-miR-7226-5p;mmu-miR-7005-5p;mmu-miR-7009-5p
NM_009930.2	Col3a1	circRNA_0020|Chr1:10315205_10321709_+	0.9	7.0666E-12	5	mmu-miR-206-3p;mmu-miR-6378;mmu-miR-7226-5p;mmu-miR-7005-5p;mmu-miR-7009-5p
NM_028973.2	Lrrc15	circRNA_0020|Chr1:10315205_10321709_+	0.9	7.0666E-12	5	mmu-miR-206-3p;mmu-miR-7226-5p;mmu-miR-7005-5p;mmu-miR-7042-5p;mmu-miR-7009-5p
XM_011250176.1	Agrn	circRNA_3832|Chr2:169883526_169886459_+	0.66667	3.42283E-09	3	mmu-miR-344i;mmu-miR-1892;mmu-miR-3473e
NM_153178.4	Ago2	circRNA_3832|Chr2:169883526_169886459_+	1	3.42283E-09	3	mmu-miR-344i;mmu-miR-1892;mmu-miR-3473e
NM_001198955.1	Gm7694	circRNA_3832|Chr2:169883526_169886459_+	0.66667	3.42283E-09	3	mmu-miR-344i;mmu-miR-1892;mmu-miR-3473e
NM_001290469.1	Atp1a3	circRNA_3832|Chr2:169883526_169886459_+	0.5	3.42283E-09	3	mmu-miR-344i;mmu-miR-1892;mmu-miR-3473e
XM_006530325.3	Oas2	circRNA_3832|Chr2:169883526_169886459_+	0.83333	3.42283E-09	3	mmu-miR-344i;mmu-miR-1892;mmu-miR-3473e
NM_148932.2	Pom121	circRNA_3832|Chr2:169883526_169886459_+	1	3.42283E-09	3	mmu-miR-344i;mmu-miR-1892;mmu-miR-3473e
NM_019732.2	Runx3	circRNA_3832|Chr2:169883526_169886459_+	0.83333	8.55708E-09	3	mmu-miR-344i;mmu-miR-1892;mmu-miR-3473e
NM_001302471.1	Itga10	circRNA_3832|Chr2:169883526_169886459_+	0.83333	8.55708E-09	3	mmu-miR-344i;mmu-miR-1892;mmu-miR-3473e
XM_006515096.2	Greb1	circRNA_3832|Chr2:169883526_169886459_+	0.83333	8.55708E-09	3	mmu-miR-344i;mmu-miR-1892;mmu-miR-3473e

NM_001007570.2	Slc25a42	circRNA_3832|Chr2:169883526_169886459_+	0.66667	8.55708E-09	3	mmu-miR-344i;mmu-miR-1892;mmu-miR-3473e
NM_010181.2	Fbn2	circRNA_3832|Chr2:169883526_169886459_+	1	8.55708E-09	3	mmu-miR-344i;mmu-miR-1892;mmu-miR-3473e
XM_006532962.2	Fam222b	circRNA_3832|Chr2:169883526_169886459_+	1	8.55708E-09	3	mmu-miR-344i;mmu-miR-1892;mmu-miR-3473e
NM_178676.4	Entpd3	circRNA_3832|Chr2:169883526_169886459_+	0.66667	8.55708E-09	3	mmu-miR-344i;mmu-miR-1892;mmu-miR-3473e
XM_011248602.2	Cpt1a	circRNA_3832|Chr2:169883526_169886459_+	0.5	8.55708E-09	3	mmu-miR-344i;mmu-miR-1892;mmu-miR-3473e
XM_006503080.3	Slc35d1	circRNA_3832|Chr2:169883526_169886459_+	0.5	8.55708E-09	3	mmu-miR-344i;mmu-miR-1892;mmu-miR-3473e
XM_011245529.2	St3gal1	circRNA_3832|Chr2:169883526_169886459_+	0.83333	8.55708E-09	3	mmu-miR-344i;mmu-miR-1892;mmu-miR-3473e
XM_006530131.1	Cit	circRNA_3832|Chr2:169883526_169886459_+	0.83333	8.55708E-09	3	mmu-miR-344i;mmu-miR-1892;mmu-miR-3473e
XM_006537815.3	Phf24	circRNA_3832|Chr2:169883526_169886459_+	0.66667	8.55708E-09	3	mmu-miR-344i;mmu-miR-1892;mmu-miR-3473e
XM_006524328.3	Brpf3	circRNA_3832|Chr2:169883526_169886459_+	1	8.55708E-09	3	mmu-miR-344i;mmu-miR-1892;mmu-miR-3473e
XM_006511007.3	Zfp609	circRNA_3832|Chr2:169883526_169886459_+	0.83333	8.55708E-09	3	mmu-miR-344i;mmu-miR-1892;mmu-miR-3473e
XM_011248201.2	Tpcn1	circRNA_3832|Chr2:169883526_169886459_+	0.83333	8.55708E-09	3	mmu-miR-344i;mmu-miR-1892;mmu-miR-3473e
XM_006540566.1	Acan	circRNA_3832|Chr2:169883526_169886459_+	1	8.55708E-09	3	mmu-miR-344i;mmu-miR-1892;mmu-miR-3473e
NM_138955.3	Abcg4	circRNA_3832|Chr2:169883526_169886459_+	1	8.55708E-09	3	mmu-miR-344i;mmu-miR-1892;mmu-miR-3473e
XM_006529727.2	Atp2b4	circRNA_3832|Chr2:169883526_169886459_+	0.83333	8.55708E-09	3	mmu-miR-344i;mmu-miR-1892;mmu-miR-3473e
NM_175692.3	Snhg11	circRNA_0020|Chr1:10315205_10321709_+	0.3	1.71142E-08	3	mmu-miR-7009-5p;mmu-miR-7042-5p;mmu-miR-7005-5p
NM_001291145.1	Emilin3	circRNA_0020|Chr1:10315205_10321709_+	0.5	1.71142E-08	3	mmu-miR-7009-5p;mmu-miR-7226-5p;mmu-miR-7042-5p
NM_001033167.3	Slc22a23	circRNA_3832|Chr2:169883526_169886459_+	1	1.71142E-08	3	mmu-miR-344i;mmu-miR-1892;mmu-miR-3473e
NM_133167.3	Parvb	circRNA_3832|Chr2:169883526_169886459_+	0.83333	1.71142E-08	3	mmu-miR-344i;mmu-miR-1892;mmu-miR-3473e
NM_001134300.2	Nol4l	circRNA_3832|Chr2:169883526_169886459_+	0.83333	1.71142E-08	3	mmu-miR-344i;mmu-miR-1892;mmu-miR-3473e
XM_006508990.2	Ank1	circRNA_3832|Chr2:169883526_169886459_+	1	1.71142E-08	3	mmu-miR-344i;mmu-miR-1892;mmu-miR-3473e
XM_011248576.2	Gm22	circRNA_3832|Chr2:169883526_169886459_+	1	1.71142E-08	3	mmu-miR-344i;mmu-miR-1892;mmu-miR-3473e
XM_006530353.3	Gm15800	circRNA_3832|Chr2:169883526_169886459_+	1	1.71142E-08	3	mmu-miR-344i;mmu-miR-1892;mmu-miR-3473e
NM_001077403.1	Nrp2	circRNA_3832|Chr2:169883526_169886459_+	0.5	2.99498E-08	3	mmu-miR-344i;mmu-miR-1892;mmu-miR-3473e
XM_006520023.2	Matn2	circRNA_0020|Chr1:10315205_10321709_+	0.3	6.83761E-08	3	mmu-miR-7009-5p;mmu-miR-7226-5p;mmu-miR-7042-5p

XM_006520437.2	Fbln1	circRNA_0020|Chr1:10315205_10321709_+	0.5	6.83761E-08	3	mmu-miR-7226-5p;mmu-miR-6378;mmu-miR-7042-5p
NM_031180.2	Klb	circRNA_0020|Chr1:10315205_10321709_+	0.3	6.83761E-08	3	mmu-miR-206-3p;mmu-miR-7226-5p;mmu-miR-7009-5p
XM_006522398.3	Arl6	circRNA_0020|Chr1:10315205_10321709_+	0.4	6.83761E-08	3	mmu-miR-7226-5p;mmu-miR-6378;mmu-miR-7042-5p
NM_001161541.1	Islr2	circRNA_0020|Chr1:10315205_10321709_+	0.5	1.70739E-07	3	mmu-miR-7226-5p;mmu-miR-7009-5p;mmu-miR-7005-5p
XM_006510297.3	Glbl12	circRNA_0020|Chr1:10315205_10321709_+	0.3	1.70739E-07	3	mmu-miR-206-3p;mmu-miR-7042-5p;mmu-miR-7009-5p
NM_177578.4	Skint3	circRNA_0020|Chr1:10315205_10321709_+	0.4	1.70739E-07	3	mmu-miR-206-3p;mmu-miR-7226-5p;mmu-miR-7042-5p
NM_001310705.1	Slc16a4	circRNA_0020|Chr1:10315205_10321709_+	0.3	3.41076E-07	3	mmu-miR-206-3p;mmu-miR-7226-5p;mmu-miR-7042-5p
NM_011581.3	Thbs2	circRNA_0020|Chr1:10315205_10321709_+	0.5	3.41076E-07	3	mmu-miR-7226-5p;mmu-miR-7042-5p;mmu-miR-7009-5p
NM_009127.4	Scd1	circRNA_0020|Chr1:10315205_10321709_+	0.3	5.9618E-07	3	mmu-miR-7009-5p;mmu-miR-6378;mmu-miR-7005-5p

Table S10 GO annotations of the ceRNA network

Term_ID	Term_description	Fold Enrichment	GeneSymbols	P_value	Category
GO:0005201	extracellular matrix structural constituent	50.3004386	Col3a1;Fbln1;Acan;Fbn2	1.18E-06	Function
GO:0005604	basement membrane	23.7254902	Fbln1;Thbs2;Matn2;Acan;Agrn	2.19E-06	Component
GO:0005578	proteinaceous extracellular matrix	10.35987643	Fbln1;Emilin3;Thbs2;Matn2;Fbn2;Col3a1;Acan	4.70E-06	Component
GO:0031012	extracellular matrix	10.29367913	Fbln1;Emilin3;Thbs2;Matn2;Fbn2;Col3a1;Agrn	4.91E-06	Component
GO:0042383	sarcolemma	16.1499828	Ank1;Atp1a3;Agrn;Atp2b4	0.000111	Component
GO:0086036	regulation of cardiac muscle cell membrane potential	107.0294785	Atp1a3;Agrn	0.000151	Process
GO:0005509	calcium ion binding	5.669456215	Fbln1;Thbs2;Fbn2;Matn2;Acan;Agrn;Phf24	0.000212	Function
GO:0016020	membrane	1.763503869	Arl6;Atp2b4;Slc16a4;Slc25a42;A530016L24Rik;Nrp2;Klb;Oas2;Tpcn1;Ank1;Slc35d1;Slc22a23;Atp1a3;Cpt1a;Scd1;Parvb;Islr2;Skint3;Pom121;Cit;Prlr;Ago2;Agrn;St3gal1;Greb1;Klhdc7a;Lrrc15	0.000518	Component
GO:0005773	vacuole	41.84313725	Cit;Glb1l2	0.00104	Component
GO:0043395	heparan sulfate proteoglycan binding	41.55253623	Atp1a3;Agrn	0.001055	Function
GO:0016021	integral component of membrane	1.879020081	Atp2b4;Slc16a4;Slc25a42;A530016L24Rik;Nrp2;Klb;Tpcn1;Abcg4;Slc35d1;Slc22a23;Atp1a3;Cpt1a;Scd1;Islr2;Skint3;Pom121;Prlr;Agrn;St3gal1;Greb1;Klhdc7a;Lrrc15	0.001092	Component
GO:0004553	hydrolase activity, hydrolyzing O-glycosyl compounds	36.75801282	Glb1l2;Klb	0.00135	Function
GO:0001968	fibronectin binding	31.85694444	Fbln1;Lrrc15	0.001797	Function
GO:0007411	axon guidance	10.54670043	Runx3;Matn2;Nrp2	0.002901	Process
GO:0005576	extracellular region	2.728361054	Dkk3;Fbln1;Emilin3;Thbs2;Matn2;Fbn2;Col3a1;Acan;St3gal1;Glb1l2	0.003021	Component
GO:0006641	triglyceride metabolic process	23.49427576	Scd1;Cpt1a	0.003289	Process
GO:0030199	collagen fibril organization	23.49427576	Col3a1;Acan	0.003289	Process
GO:0007275	multicellular organism development	3.22316307	Islr2;Dkk3;Cit;Ago2;Zfp609;Agrn;Nrp2	0.005561	Process
GO:0046332	SMAD binding	17.06622024	Runx3;Col3a1	0.006145	Function
GO:0046872	metal ion binding	2.074851091	Scd1;Oas2;Arl6;Cit;Atp2b4;Prlr;Col3a1;Acan;Ago2;Nrp2;Zfp609;Atp1a3;Phf24	0.00712	Function
GO:0006006	glucose metabolic process	15.5365372	Cpt1a;Hectd4	0.007376	Process
GO:0097110	scaffold protein binding	14.48042929	Cit;Atp2b4	0.008447	Function
GO:0003725	double-stranded RNA binding	14.05453431	Oas2;Ago2	0.008947	Function
GO:0007229	integrin-mediated signaling pathway	13.76093294	Itga10;Col3a1	0.009322	Process
GO:0055085	transmembrane transport	4.816326531	Tpcn1;Slc16a4;Slc22a23;Slc25a42	0.009448	Process
GO:0006486	protein glycosylation	13.19541515	Oas2;St3gal1	0.010104	Process
GO:2000481	positive regulation of cAMP-dependent protein kinase activity	96.32653061	Atp2b4	0.010339	Process
GO:0072378	blood coagulation, fibrin clot formation	96.32653061	Fbln1	0.010339	Process
GO:0015867	ATP transport	96.32653061	Slc25a42	0.010339	Process
GO:0034435	cholesterol esterification	96.32653061	Scd1	0.010339	Process
GO:0015672	monovalent inorganic cation	96.32653061	Ank1	0.010339	Process
GO:0007091	metaphase/anaphase transition of mitotic cell cycle	96.32653061	Cit	0.010339	Process
GO:0008582	regulation of synaptic growth at neuromuscular junction	96.32653061	Agrn	0.010339	Process
GO:0070885	negative regulation of calcineurin-NFAT signaling cascade	96.32653061	Atp2b4	0.010339	Process
GO:0061551	trigeminal ganglion development	96.32653061	Nrp2	0.010339	Process
GO:0060414	aorta smooth muscle tissue morphogenesis	96.32653061	Col3a1	0.010339	Process
GO:0016717	oxidoreductase activity, acting on paired donors, with oxidation of a pair of donors resulting in the reduction of molecular oxygen to two molecules of water	95.57083333	Scd1	0.010421	Function
GO:0043262	adenosine-diphosphatase activity	95.57083333	Slc25a42	0.010421	Function
GO:0070051	fibrinogen binding	95.57083333	Fbln1	0.010421	Function
GO:0042030	ATPase inhibitor activity	95.57083333	Agrn	0.010421	Function

GO:0098639	collagen binding involved in cell-matrix adhesion	95.57083333	Itga10	0.010421	Function
GO:0007507	heart development	6.25496952	Acan;Col3a1;Nrp2	0.012249	Process
GO:0007160	cell-matrix adhesion	11.89216427	Itga10;Col3a1	0.012326	Process
GO:1990535	neuron projection maintenance	80.27210884	Atp1a3	0.012394	Process
GO:1903243	negative regulation of cardiac muscle hypertrophy in response to stress	80.27210884	Atp2b4	0.012394	Process
GO:0090080	positive regulation of MAPKKK cascade by fibroblast growth factor receptor signaling pathway	80.27210884	Klb	0.012394	Process
GO:0038084	vascular endothelial growth factor signaling pathway	80.27210884	Nrp2	0.012394	Process
GO:1902083	negative regulation of peptidyl-cysteine S-nitrosylation	80.27210884	Atp2b4	0.012394	Process
GO:0071559	response to transforming growth factor beta	80.27210884	Runx3	0.012394	Process
GO:0036376	sodium ion export across plasma membrane	80.27210884	Atp1a3	0.012394	Process
GO:0008093	cytoskeletal adaptor activity	79.64236111	Ank1	0.012492	Function
GO:0004768	stearoyl-CoA 9-desaturase activity	79.64236111	Scd1	0.012492	Function
GO:0004565	beta-galactosidase activity	79.64236111	Glb1l2	0.012492	Function
GO:0035374	chondroitin sulfate binding	79.64236111	Agrn	0.012492	Function
GO:0014731	spectrin-associated cytoskeleton	76.7124183	Ank1	0.012966	Component
GO:0044326	dendritic spine neck	76.7124183	Atp1a3	0.012966	Component
GO:0070776	MOZ/MORF histone acetyltransferase complex	76.7124183	Brpf3	0.012966	Component
GO:0005886	plasma membrane	1.803583502	Parvb;Islr2;Arl6;Cit;Atp2b4;Entpd3;Tpcn1;Slc16a4;Abcg4;Ank1;A530016L24Rik;Agrn;Nrp2;Atp1a3;Klb	0.013687	Component
GO:1902305	regulation of sodium ion transmembrane transport	68.80466472	Atp2b4	0.014446	Process
GO:1901166	neural crest cell migration involved in autonomic nervous system development	68.80466472	Nrp2	0.014446	Process
GO:0006999	nuclear pore organization	68.80466472	Pom121	0.014446	Process
GO:0061180	mammary gland epithelium development	68.80466472	Prlr	0.014446	Process
GO:0070542	response to fatty acid	68.80466472	Scd1	0.014446	Process
GO:0009143	nucleoside triphosphate catabolic process	68.80466472	Entpd3	0.014446	Process
GO:0035280	miRNA loading onto RISC involved in gene silencing by miRNA	68.80466472	Ago2	0.014446	Process
GO:2000647	negative regulation of stem cell proliferation	68.80466472	Fbln1	0.014446	Process
GO:0030548	acetylcholine receptor regulator activity	68.26488095	Agrn	0.014559	Function
GO:0005021	vascular endothelial growth factor-activated receptor activity	68.26488095	Nrp2	0.014559	Function
GO:0005347	ATP transmembrane transporter activity	68.26488095	Slc25a42	0.014559	Function
GO:0015217	ADP transmembrane transporter activity	68.26488095	Slc25a42	0.014559	Function
GO:0005577	fibrinogen complex	65.7535014	Fbln1	0.015111	Component
GO:0070588	calcium ion transmembrane transport	10.58533303	Tpcn1;Atp2b4	0.015376	Process
GO:0048846	axon extension involved in axon guidance	60.20408163	Nrp2	0.016492	Process
GO:0009437	carnitine metabolic process	60.20408163	Cpt1a	0.016492	Process
GO:0030346	protein phosphatase 2B binding	59.73177083	Atp2b4	0.016622	Function
GO:0005890	sodium:potassium-exchanging ATPase complex	57.53431373	Atp1a3	0.017252	Component
GO:0061549	sympathetic ganglion development	53.51473923	Nrp2	0.018535	Process
GO:0030166	proteoglycan biosynthetic process	53.51473923	Acan	0.018535	Process
GO:0030856	regulation of epithelial cell differentiation	53.51473923	Prlr	0.018535	Process
GO:0010873	positive regulation of cholesterol esterification	53.51473923	Scd1	0.018535	Process
GO:0030206	chondroitin sulfate biosynthetic process	53.51473923	Slc35d1	0.018535	Process
GO:0060346	bone trabecula formation	53.51473923	Fbn2	0.018535	Process
GO:0032000	positive regulation of fatty acid beta-oxidation	53.51473923	Cpt1a	0.018535	Process
GO:0030007	cellular potassium ion homeostasis	53.51473923	Atp1a3	0.018535	Process

GO:0071872	cellular response to epinephrine stimulus	53.51473923	Atp2b4	0.018535	Process
GO:0071340	skeletal muscle acetylcholine-gated channel clustering	53.51473923	Agrn	0.018535	Process
GO:0035197	siRNA binding	53.09490741	Ago2	0.018681	Function
GO:0000340	RNA 7-methylguanosine cap binding	53.09490741	Ago2	0.018681	Function
GO:0005178	integrin binding	9.101984127	Col3a1;Fbln1	0.020419	Function
GO:0021612	facial nerve structural organization	48.16326531	Nrp2	0.020574	Process
GO:0060736	prostate gland growth	48.16326531	Prlr	0.020574	Process
GO:0030031	cell projection assembly	48.16326531	Parvb	0.020574	Process
GO:0051001	negative regulation of nitric-oxide synthase activity	48.16326531	Atp2b4	0.020574	Process
GO:0010586	miRNA metabolic process	48.16326531	Ago2	0.020574	Process
GO:0008064	regulation of actin polymerization or depolymerization	48.16326531	Cit	0.020574	Process
GO:0048733	sebaceous gland development	48.16326531	Scd1	0.020574	Process
GO:0001730	2'-5'-oligoadenylate synthetase activity	47.78541667	Oas2	0.020735	Function
GO:0005391	sodium:potassium-exchanging ATPase activity	47.78541667	Atp1a3	0.020735	Function
GO:0002162	dystroglycan binding	47.78541667	Agrn	0.020735	Function
GO:0050774	negative regulation of dendrite morphogenesis	43.78478664	Cit	0.022608	Process
GO:0050777	negative regulation of immune response	43.78478664	Col3a1	0.022608	Process
GO:0097435	supramolecular fiber organization	43.78478664	Col3a1	0.022608	Process
GO:0060213	positive regulation of nuclear-transcribed mRNA poly(A) tail shortening	43.78478664	Ago2	0.022608	Process
GO:0036122	BMP binding	43.44128788	Agrn	0.022785	Function
GO:0017111	nucleoside-triphosphatase activity	43.44128788	Entpd3	0.022785	Function
GO:0017154	semaphorin receptor activity	43.44128788	Nrp2	0.022785	Function
GO:0044327	dendritic spine head	41.84313725	Atp1a3	0.023646	Component
GO:0070578	RISC-loading complex	41.84313725	Ago2	0.023646	Component
GO:0001527	microfibril	41.84313725	Fbn2	0.023646	Component
GO:0016442	RISC complex	41.84313725	Ago2	0.023646	Component
GO:0005845	mRNA cap binding complex	41.84313725	Ago2	0.023646	Component
GO:0060644	mammary gland epithelial cell differentiation	40.13605442	Prlr	0.024638	Process
GO:0008347	glial cell migration	40.13605442	Matn2	0.024638	Process
GO:0021675	nerve development	40.13605442	Nrp2	0.024638	Process
GO:0031054	pre-miRNA processing	40.13605442	Ago2	0.024638	Process
GO:0015718	monocarboxylic acid transport	40.13605442	Slc16a4	0.024638	Process
GO:0032228	regulation of synaptic transmission, GABAergic	40.13605442	Phf24	0.024638	Process
GO:0033089	positive regulation of T cell differentiation in thymus	40.13605442	Zfp609	0.024638	Process
GO:2001223	negative regulation of neuron migration	40.13605442	Col3a1	0.024638	Process
GO:0032609	interferon-gamma production	40.13605442	Runx3	0.024638	Process
GO:0016504	peptidase activator activity	39.82118056	Fbln1	0.024831	Function
GO:0048407	platelet-derived growth factor	39.82118056	Col3a1	0.024831	Function
GO:0017110	nucleoside-diphosphatase activity	39.82118056	Entpd3	0.024831	Function
GO:0031748	D1 dopamine receptor binding	39.82118056	Atp1a3	0.024831	Function
GO:0030165	PDZ domain binding	8.168447293	Cit;Atp2b4	0.02498	Function
GO:0043083	synaptic cleft	38.35620915	Agrn	0.025768	Component
GO:0048699	generation of neurons	37.04866562	Cit	0.026665	Process
GO:0045947	negative regulation of translational initiation	37.04866562	Ago2	0.026665	Process
GO:1900025	negative regulation of substrate adhesion-dependent cell spreading	37.04866562	Fbln1	0.026665	Process
GO:2000146	negative regulation of cell motility	37.04866562	Fbln1	0.026665	Process
GO:0006779	porphyrin-containing compound biosynthetic process	37.04866562	Ank1	0.026665	Process
GO:0008253	5'-nucleotidase activity	36.75801282	Entpd3	0.026873	Function
GO:0030018	Z disc	7.801262878	Ank1;Atp2b4	0.027217	Component
GO:0043231	intracellular membrane-bounded organelle	2.924234497	Scd1;Oas2;Atp2b4;Runx3;Cpt1a	0.027598	Component
GO:0001764	neuron migration	7.58476619	Matn2;Nrp2	0.028659	Process
GO:1900153	positive regulation of nuclear-transcribed mRNA catabolic process, deadenylation-dependent decay	34.40233236	Ago2	0.028687	Process

GO:0035235	ionotropic glutamate receptor signaling pathway	34.40233236	Atp1a3	0.028687	Process
GO:0031985	Golgi cisterna	32.8767507	Cit	0.029999	Component
GO:0005515	protein binding	1.596172582	Parvb;Islr2;Arl6;Cit;Fbln1;Prlr;Thbs2;Fbn2;Runx3;Acan;Ago2;Nrp2;Zfp609;Agrn;Klb;Phf24	0.030523	Function
GO:0048678	response to axon injury	32.10884354	Matn2	0.030704	Process
GO:0006883	cellular sodium ion homeostasis	32.10884354	Atp1a3	0.030704	Process
GO:0050872	white fat cell differentiation	32.10884354	Scd1	0.030704	Process
GO:0005388	calcium-transporting ATPase activity	31.85694444	Atp2b4	0.030944	Function
GO:0008028	monocarboxylic acid transmembrane transporter activity	31.85694444	Slc16a4	0.030944	Function
GO:0007009	plasma membrane organization	30.10204082	Agrn	0.032718	Process
GO:1903077	negative regulation of protein localization to plasma membrane	30.10204082	Lrrc15	0.032718	Process
GO:0055117	regulation of cardiac muscle contraction	30.10204082	Agrn	0.032718	Process
GO:0098656	anion transmembrane transport	30.10204082	Slc25a42	0.032718	Process
GO:0035196	production of miRNAs involved in gene silencing by miRNA	30.10204082	Ago2	0.032718	Process
GO:0045019	negative regulation of nitric oxide biosynthetic process	30.10204082	Atp2b4	0.032718	Process
GO:0006401	RNA catabolic process	30.10204082	Oas2	0.032718	Process
GO:0098655	cation transmembrane transport	30.10204082	Slc25a42	0.032718	Process
GO:0006405	RNA export from nucleus	30.10204082	Pom121	0.032718	Process
GO:0006811	ion transport	3.281994229	Slc22a23;Tpcn1;Atp1a3;Atp2b4	0.033262	Process
GO:0032039	integrator complex	28.76715686	Zfp609	0.034212	Component
GO:0002063	chondrocyte development	28.33133253	Acan	0.034728	Process
GO:0035278	miRNA mediated inhibition of translation	28.33133253	Ago2	0.034728	Process
GO:0006816	calcium ion transport	6.783558494	Tpcn1;Atp2b4	0.035164	Process
GO:0031091	platelet alpha granule	27.07497116	Thbs2	0.036312	Component
GO:0009314	response to radiation	26.75736961	Col3a1	0.036734	Process
GO:0006855	drug transmembrane transport	26.75736961	Slc25a42	0.036734	Process
GO:0006164	purine nucleotide biosynthetic	26.75736961	Oas2	0.036734	Process
GO:2001224	positive regulation of neuron migration	26.75736961	Zfp609	0.036734	Process
GO:0005487	nucleocytoplasmic transporter	26.5474537	Pom121	0.037019	Function
GO:0017056	structural constituent of nuclear pore	26.5474537	Pom121	0.037019	Function
GO:0008201	heparin binding	6.591091954	Thbs2;Nrp2	0.037043	Function
GO:0031175	neuron projection development	6.464867826	Runx3;Matn2	0.038378	Process
GO:0009311	oligosaccharide metabolic process	25.349087	St3gal1	0.038735	Process
GO:0010842	retina layer formation	25.349087	Arl6	0.038735	Process
GO:0001502	cartilage condensation	25.349087	Acan	0.038735	Process
GO:0042789	mRNA transcription from RNA polymerase II promoter	25.349087	Runx3	0.038735	Process
GO:0060749	mammary gland alveolus development	25.349087	Prlr	0.038735	Process
GO:0017127	cholesterol transporter activity	25.1502193	Abcg4	0.039036	Function
GO:0005605	basal lamina	24.2249742	Agrn	0.040498	Component
GO:0044130	negative regulation of growth of symbiont in host	24.08163265	Scd1	0.040733	Process
GO:0006471	protein ADP-ribosylation	24.08163265	Arl6	0.040733	Process
GO:0008373	sialyltransferase activity	23.89270833	St3gal1	0.041049	Function
GO:0050431	transforming growth factor beta binding	23.89270833	Agrn	0.041049	Function
GO:0006631	fatty acid metabolic process	6.13544781	Scd1;Cpt1a	0.042183	Process
GO:0050966	detection of mechanical stimulus involved in sensory perception of	22.93488824	Phf24	0.042726	Process
GO:0001676	long-chain fatty acid metabolic process	22.93488824	Cpt1a	0.042726	Process
GO:0004521	endoribonuclease activity	22.75496032	Ago2	0.043057	Function
GO:0030673	axolemma	21.9178338	Ank1	0.044666	Component
GO:0061512	protein localization to cilium	21.89239332	Arl6	0.044715	Process
GO:0008589	regulation of smoothened signaling pathway	21.89239332	Arl6	0.044715	Process
GO:0000070	mitotic sister chromatid segregation	21.89239332	Cit	0.044715	Process
GO:0050998	nitric-oxide synthase binding	21.72064394	Atp2b4	0.045062	Function
GO:1904646	cellular response to amyloid-beta	20.94055013	Atp1a3	0.046701	Process
GO:0007259	JAK-STAT cascade	20.94055013	Prlr	0.046701	Process
GO:0048821	erythrocyte development	20.94055013	Ank1	0.046701	Process
GO:0008610	lipid biosynthetic process	20.94055013	Scd1	0.046701	Process
GO:0005540	hyaluronic acid binding	20.77626812	Acan	0.047062	Function

GO:0008139	nuclear localization sequence binding	20.77626812	Pom121	0.047062	Function
GO:0010107	potassium ion import	20.06802721	Atp1a3	0.048682	Process
GO:0061098	positive regulation of protein tyrosine kinase activity	20.06802721	Agrn	0.048682	Process
GO:0031672	A band	20.01193521	Ank1	0.048816	Component
GO:0031307	integral component of mitochondrial outer membrane	20.01193521	Cpt1a	0.048816	Component
GO:0035198	miRNA binding	19.91059028	Ago2	0.049058	Function
